# DNA promoter hypermethylation of melanocyte lineage genes determines melanoma phenotype

**DOI:** 10.1172/jci.insight.156577

**Published:** 2022-10-10

**Authors:** Adriana Sanna, Bengt Phung, Shamik Mitra, Martin Lauss, Jiyeon Choi, Tongwu Zhang, Ching-Ni Njauw, Eugenia Cordero, Katja Harbst, Frida Rosengren, Rita Cabrita, Iva Johansson, Karolin Isaksson, Christian Ingvar, Ana Carneiro, Kevin Brown, Hensin Tsao, My Andersson, Kristian Pietras, Göran Jönsson

**Affiliations:** 1 Division of Oncology, Department of Clinical Sciences, Lund University, Lund, Sweden.; 2 Laboratory of Translational Genomics, Division of Cancer Epidemiology and Genetics, National Cancer Institute, NIH, Bethesda, Maryland, USA.; 3 Department of Dermatology, Harvard Medical School, Boston, Massachusetts, USA.; 4 Division of Translational Cancer Research, Department of Laboratory Medicine;; 5 Department of Clinical Pathology, Skåne University Hospital; and; 6 Division of Surgery, Department of Clinical Sciences, Lund University, Lund, Sweden.; 7 Department of Surgery, Kristianstad Hospital, Kristianstad, Sweden.; 8 Department of Oncology, Skåne University Hospital, and; 9 Department of Neurology, Epilepsy Center, Lund University, Lund, Sweden.

**Keywords:** Cell Biology, Oncology, Melanoma

## Abstract

Cellular stress contributes to the capacity of melanoma cells to undergo phenotype switching into highly migratory and drug-tolerant dedifferentiated states. Such dedifferentiated melanoma cell states are marked by loss of melanocyte-specific gene expression and increase of mesenchymal markers. Two crucial transcription factors, microphthalmia-associated transcription factor (MITF) and SRY-box transcription factor 10 (SOX10), important in melanoma development and progression, have been implicated in this process. In this study we describe that loss of MITF is associated with a distinct transcriptional program, *MITF* promoter hypermethylation, and poor patient survival in metastatic melanoma. From a comprehensive collection of melanoma cell lines, we observed that *MITF*-methylated cultures were subdivided in 2 distinct subtypes. Examining mRNA levels of neural crest–associated genes, we found that 1 subtype had lost the expression of several lineage genes, including *SOX10*. Intriguingly, SOX10 loss was associated with *SOX10* gene promoter hypermethylation and distinct phenotypic and metastatic properties. Depletion of SOX10 in MITF-methylated melanoma cells using CRISPR/Cas9 supported these findings. In conclusion, this study describes the significance of melanoma state and the underlying functional properties explaining the aggressiveness of such states.

## Introduction

Melanoma is a notoriously heterogeneous cancer comprising different molecular subtypes that are reflected in patient survival and therapeutic response (1, 2). The dominant cause of death in patients with advanced melanoma is tumor relapse following therapy resistance, which can result from melanoma cells that are able to survive immunotherapies and targeted therapies because of their capacity to dedifferentiate (3). This intrinsic property is retained from the early developing melanocytes originating from the pluripotent neural crest cell (NCC) population, which differentiate into a range of cell types that are critical for normal embryonic development (4, 5). In order to disseminate from the neural crest (NC), the transient ectodermal structure of the vertebrate embryo, expression of specific transcription factors is required for NCC specification into several lineages (6).

A crucial transcription factor expressed in migrating NCCs is SRY-box transcription factor 10 (SOX10), which is found to be expressed from the prospective cells of the NC in the dorsal part of the neural tube, named neural crest stem cells (NCSCs). SOX10 is one of the NCSC markers expressed in several adult-derived NCSC types, and its expression is needed to specify the nonmesenchymal NC derivatives, including glia, neurons, and melanocytes (7). Besides SOX10, the melanocyte lineage is defined by the downstream target microphthalmia-associated transcription factor (MITF) (5, 8, 9). Interestingly, the development from NCSCs to NCCs, and further progenitors of differentiated cell types, has been provocatively suggested to be dynamic and reversible, even over extended periods in the mouse embryo (10). However, this has never been shown in humans because of ethical limitations (6, 11). Moreover, such properties may be inherited to tumor cells derived from NC lineages and need to be further explored.

The aggressive, undifferentiated melanoma subtype is characterized by the loss of the melanocytic program mainly driven by the transcription factors MITF and SOX10 (1, 12). The MITF-low (MITF^lo^) melanoma subtype is often enriched in tumors resistant to targeted therapy (1, 13, 14). In accordance with this, MITF expression was recently shown to be reduced in approximately 50% of relapsed melanoma cases (15). Upstream of MITF, reduced SOX10 expression has been reported throughout melanoma development, from early to metastatic stages (16). Strikingly, similar to the MITF^lo^ phenotype, reduced expression of SOX10 in melanoma has been shown to confer resistance to MAPK pathway inhibition (17). With the major advances of single-cell RNA sequencing, SOX10-negative melanoma cells have recently been detected among therapy-resistant tumors (18, 19). Upon these discoveries, a potentially novel melanoma differentiation model has been established. This comprises 4 distinct states of melanoma differentiation: the undifferentiated, NC-like, transitory, and melanocytic stages (12, 18, 19). Subsequently, the classic MITF rheostat model has been updated in view of such lineage cell states together with a new proposed nomenclature (20); however, these novel melanoma phenotypes remain to be formally established. Moreover, although these findings undeniably indicate the importance of *SOX10* and *MITF* repression in tumorigenesis and therapy resistance (19), the mechanisms that sustain low levels of MITF and SOX10 in melanoma are not well understood, and the MITF^lo^ melanoma subtypes in view of SOX10 expression have not yet been characterized.

We have previously found anticorrelation between *MITF* promoter methylation and expression in melanoma tumors and cell lines, highlighting the epigenetic involvement in the regulation of *MITF* (21). This may represent one of several mechanisms of MITF regulation. Furthermore, methylation-mediated repression of *SOX10* has been reported in a variety of disorders (22–24); however, studies addressing the regulation of *SOX10* through methylation in melanoma have been limited and contradictory. Nevertheless, a recent screening of hypermethylated genes in melanoma identified *SOX10* to be methylated and consequently silenced in a subset of these tumors (25), thus demonstrating that SOX10 expression may be regulated by gene methylation in melanoma. In this study, we have explored how the melanocyte differentiation–specific transcription factors, MITF and SOX10, are epigenetically regulated and determine melanoma aggressiveness, cell phenotype, and therapy response.

## Results

### Tumor-intrinsic transcriptional landscape of melanoma.

**Figure 1:**
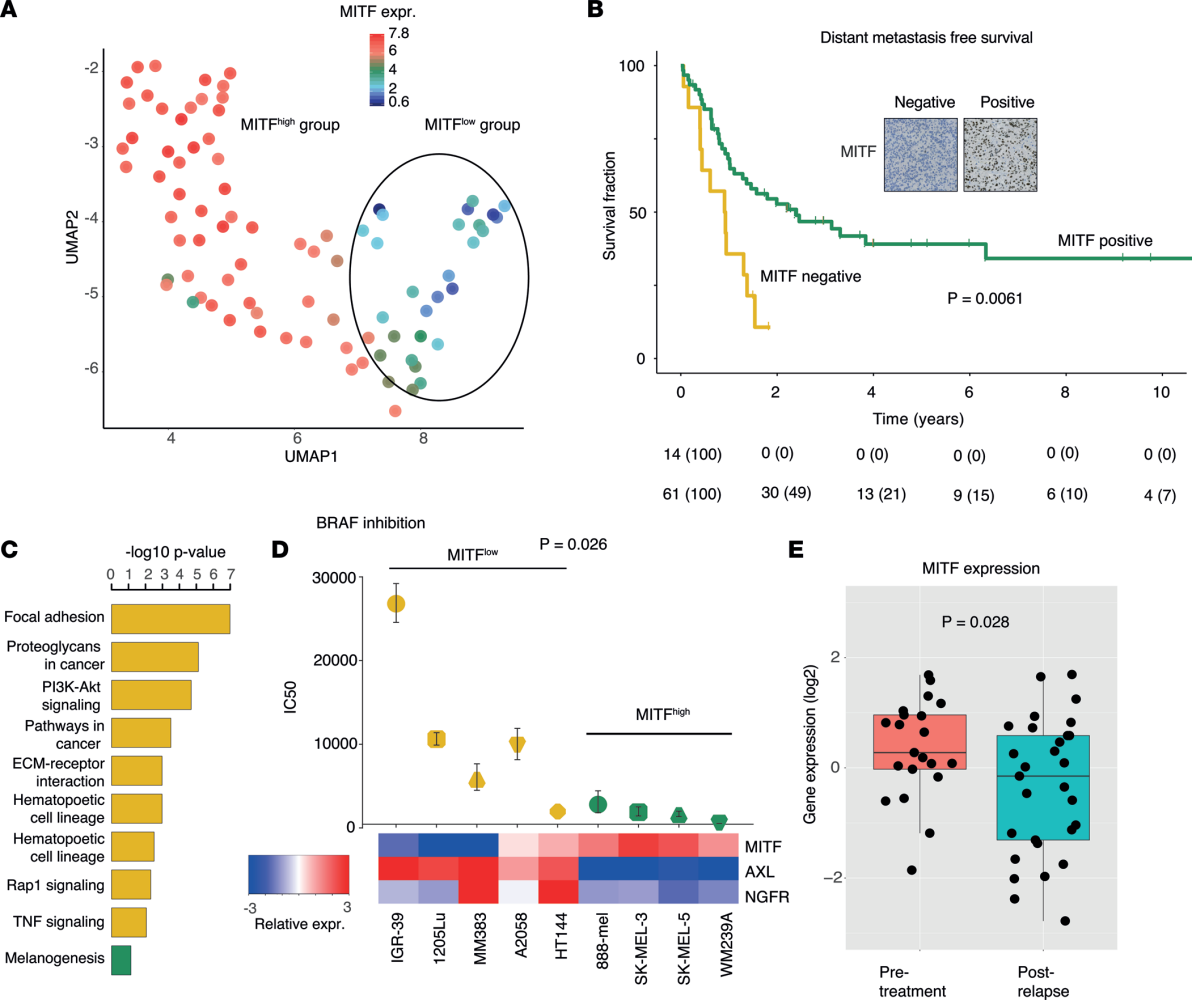
The role of MITF in melanoma. (**A**) UMAP analysis of the 1,500 genes with greatest variation across 86 melanoma cell lines driven by *MITF* gene expression levels. (**B**) MITF protein staining in a cohort of 75 lymph node metastases and association with distant metastasis–free survival using Kaplan-Meier and log-rank test. Original magnification, ×200. (**C**) Gene ontology analysis of genes discriminating MITF^lo^ and MITF^hi^ cell lines (FDR = 0). (**D**) Nine melanoma cell lines were tested for sensitivity to BRAF inhibition using vemurafenib. IC_50_ values are plotted with individual upper and lower limit indicated as whiskers. Heatmap of *MITF*, *NGFR*, and *AXL* gene expression for each cell line is included. (**E**) Box plot of *MITF* gene expression levels in pre- and postrelapse samples in a cohort of 21 patients (50 tumor samples) treated with BRAF inhibition, from National Center for Biotechnology Information Gene Expression Omnibus (GEO)GSE50509. *P* values were calculated using Mann-Whitney-Wilcoxon test.

The transcriptional landscape in melanoma has been extensively explored (1, 2, 26). To examine tumor-intrinsic transcriptional properties of melanoma, we analyzed 86 short-term melanoma cultures using RNA sequencing (RNA-Seq). Using uniform manifold approximation and projection (UMAP) analysis on 1,500 genes with largest expression variation across the melanoma cell lines, we found that *MITF* and other melanocyte lineage genes contributed significantly to the main separation of melanoma cell lines (Figure 1A). To investigate the clinical relevance of such findings, we analyzed MITF protein expression by immunostaining of a melanoma tissue microarray including 177 metastases and found 17% of these cases to be MITF negative. Importantly, treatment-naive patients with regional lymph node metastases lacking MITF protein had an inferior distant metastasis–free survival as compared with those expressing the MITF protein (Figure 1B, P = 0.0061). Our results validate at protein level that loss of MITF in melanoma cells specifically is a marker of aggressive disease, as previously shown only at transcriptional level (1, 2, 26). Next, we used the RNA-Seq data from the 86 melanoma cell lines to identify tumor cell–intrinsic transcriptional differences between the MITF^lo^ and MITF^hi^ groups. In total, 814 genes were significantly different (FDR = 0, fold change ≥ 2). Genes upregulated in the MITF^hi^ group were mostly melanocyte-specific genes while genes upregulated in the MITF^lo^ group, among other pathways, were enriched in focal adhesion and PI3K/Akt and MAPK signaling (Figure 1C). However, transcriptional heterogeneity in particular among the MITF^hi^ cell lines was apparent, supporting previous data (12). Confirming previous results (14), we also found cells with *MITF* downregulation to be more resistant to BRAF inhibition in vitro (Figure 1D, P = 0.026). Such cells expressed higher levels of *AXL* and *NGFR*, markers that have previously been associated with BRAF inhibition resistance (27, 28). To further reinforce the association between loss of MITF and resistance to MAPK inhibition, we used tumor data from 21 patients treated with BRAF inhibitors alone where transcriptomic data were obtained on available 50 pre- and postrelapse tumors (29). Indeed, *MITF* mRNA levels were significantly decreased in postrelapse as compared with pretreatment metastases (Figure 1E, P = 0.028).

Collectively, these results demonstrate that tumor-intrinsic transcriptional alterations in the melanocyte differentiation pathway correlate with melanoma aggressiveness and therapy response.

### Expression of melanocyte lineage genes is regulated by DNA promoter methylation.

**Figure 2:**
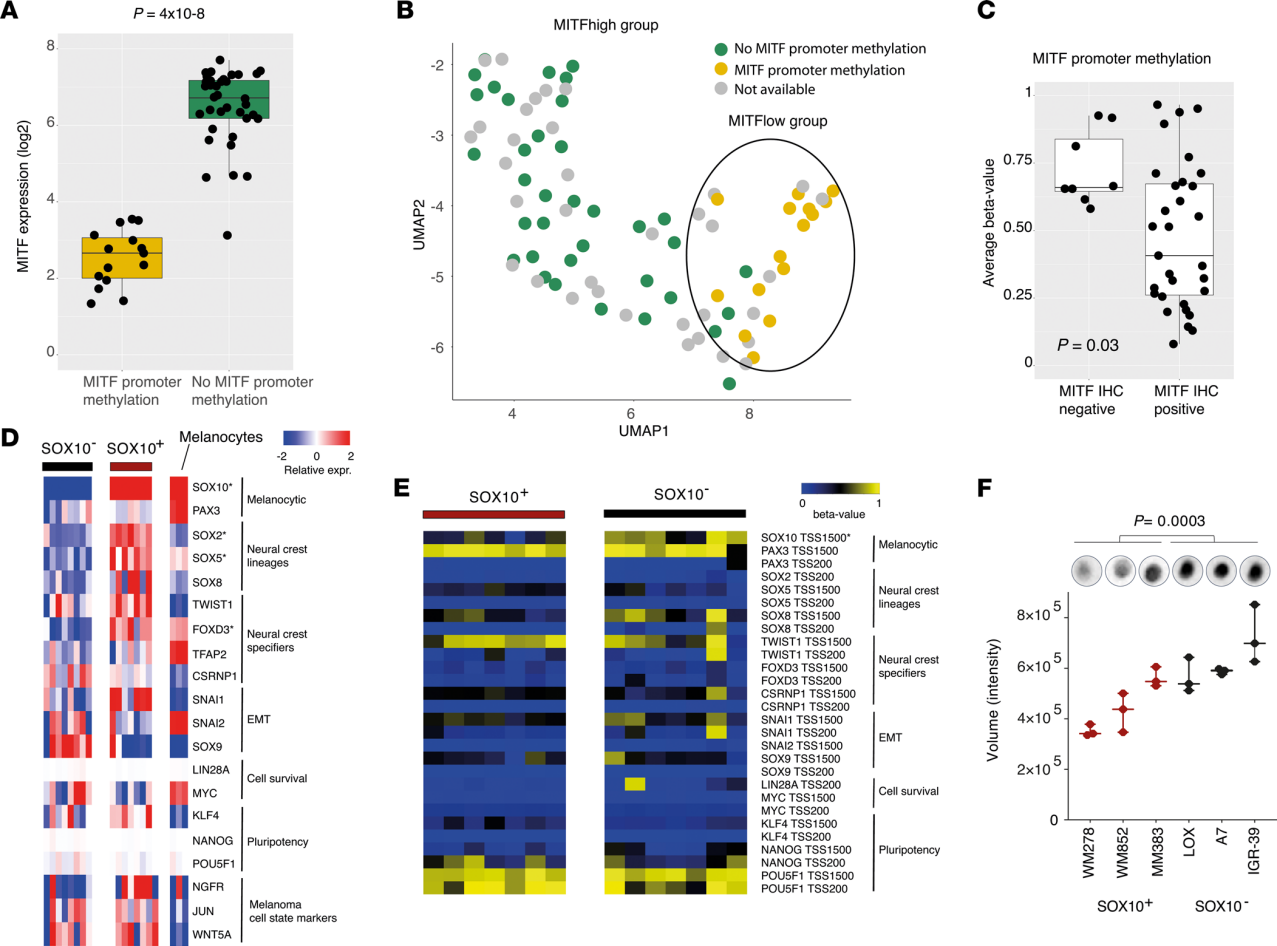
DNA methylation changes in melanocytic and NC genes. (**A**) Box plot of *MITF* gene expression levels in melanoma cell lines (*n* = 51) with and without *MITF* promoter hypermethylation. (**B**) Association between UMAP analysis of RNA-Seq data and *MITF* promoter hypermethylation in 86 melanoma cell lines. (**C**) Box plot between average β values of CpGs using GSE144487 in the MITF promoter and MITF immunostaining of 39 distant metastases. (**D**) Heatmap of gene expression levels of NC-associated genes in SOX10^–^ (*n* = 8) and SOX10^+^ (*n* = 7) melanoma cell lines and melanocytes (*n* = 3). * indicates genes significantly different between SOX10^–^ and SOX10^+^ groups (Mann-Whitney-Wilcoxon test, Bonferroni corrected). (**E**) Methylation β values from CpGs located in the promoters of NC genes in SOX10^–^ (*n* = 7) and SOX10^+^ (*n* = 7) melanoma cell lines. * indicates genes significantly different between SOX10^–^ and SOX10^+^ groups (Mann-Whitney-Wilcoxon test, Bonferroni corrected). (**F**) Bar plot with results from global methylation analysis between SOX10^–^ and SOX10^+^ melanoma cell lines using a dot blot assay. A representative membrane is showed on top of each bar for each cell line, indicating increased total 5-mC in the SOX10^–^ (black) compared with SOX10^+^ (red) cell lines. *P* value was calculated using 2-sided *t* test.

We have recently demonstrated that *MITF* gene expression is regulated by promoter methylation (21). Thus, we performed bisulfite Sanger sequencing of the *MITF* promoter to investigate the methylation pattern in 51 out of the 86 melanoma cell lines analyzed by RNA-Seq. Out of 51 cell lines, 15 harbored hypermethylation of the *MITF* promoter. As expected, there was a striking correlation between downregulation of the *MITF* transcript and *MITF* promoter hypermethylation (Figure 2A, P = 4 × 10^–8^). Moreover, melanoma cell lines with *MITF* promoter hypermethylation were heavily enriched in cases belonging to the MITF^lo^ group as shown in the UMAP analysis (Figure 2B). To confirm this, we used 39 distant melanoma metastases from a cohort of stage III/IV metastases we previously profiled using the EPIC DNA methylation arrays (30). Those metastases lacking expression of MITF by immunostaining had increased methylation β values of probes mapped to the *MITF* promoter analyzed by Illumina EPIC arrays (Figure 2C, P = 0.03). Next, we wanted to explore transcriptional heterogeneity within *MITF*-methylated cell lines. We selected the 15 melanoma cell lines that harbored *MITF* promoter hypermethylation and analyzed them separately by RNA-Seq and found 2 distinct groups by consensus clustering (Supplemental Figure 1; supplemental material available online with this article; https://doi.org/10.1172/jci.insight.156577DS1). As melanomas have an NC origin, we explored the differences between the 2 consensus groups of MITF-methylated melanomas using expression of genes involved in the NC. This included genes associated with NC self-renewal and specification, cell survival, signal mediators, epithelial-mesenchymal transition (EMT), stem cell maintenance, melanocytic, ecto-mesenchymal, glial, neural, and pluripotency (31). Intriguingly, expression of many of the NC-specific lineage genes including *SOX10*, *SOX2*, *SOX5*, and *FOXD3* (31) were significantly downregulated in 8 of the 15 melanoma cell lines belonging to group 1 (FDR < 0.05) while the only upregulated gene in these samples was *SOX9* although not reaching significance (Figure 2D). Overall, cells with decreased *SOX10* expression had loss of several genes expressed in normal melanocytes while those that retained *SOX10* had acquired expression of the NC genes as compared with melanocytic (Figure 2D). This finding largely corresponds to the groups previously identified by Tsoi et al. and Rambow et al. (12, 20). Genome-wide epigenetic characterization of the 15 melanoma cell lines with MITF promoter hypermethylation using the Illumina EPIC arrays displayed that lack of *SOX10* mRNA was associated with hypermethylation of the *SOX10* promoter. None of the other NC lineage gene promoters had different methylation patterns between the 2 groups (Figure 2E). Moreover, using dot blot assay with antibody against 5-methylcytosine (5-mC), we observed that global methylation was also increased in the SOX10^–^ melanoma cell lines (Figure 2F, P = 0.0003), suggesting a global effect on methylation patterns in cells that have lost the expression of *SOX10*.

In conclusion, melanoma cells lacking the melanocyte transcription factor MITF can be divided based on *SOX10* mRNA levels. Such grouping is associated with specific expression of NC-specific lineage genes and methylation pattern of the *SOX10* promoter.

### MITF-methylated melanomas have diverse functional phenotypes discriminated by SOX10 expression.

**Figure 3:**
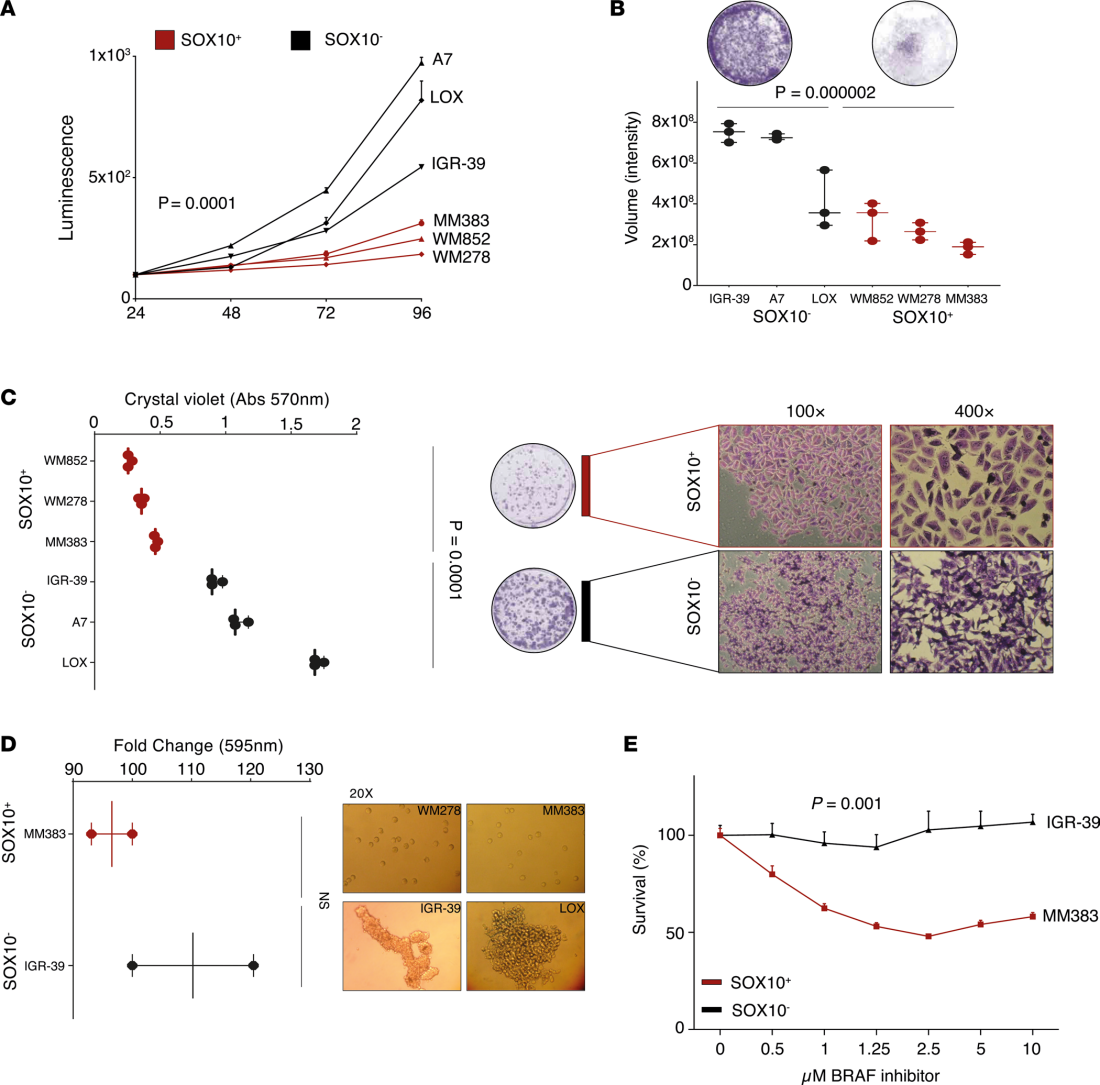
Phenotypic characterization of MITF-methylated SOX10^+^ and SOX10^–^ melanoma cell lines. (**A**) Cell proliferation assessed by cell total protein levels in 24–96 hours’ time course; the MITF-methylated SOX10^–^ group (black) shows significantly higher proliferation rate than the MITF-methylated SOX10^+^ cell lines (red). *P* values were calculated with ANOVA with Dunnett’s multiple-comparison test. (**B**) Cell migration through semipermeable membrane shows that MITF-methylated SOX10^–^ cells (black) have significantly higher migration capacities than the SOX10^+^ melanomas (red) at 72 hours’ time point. *P* value was calculated with 2-sided *t* test. (**C**) Colony forming assay in 2-week period shows significantly higher number and size of colonies in the MITF-methylated SOX10^–^ subgroup (black) than SOX10^+^ cells (red). As seen microscopically, SOX10^–^ colonies are sparse with loose cell-to-cell contact, while the SOX10^+^ group forms compact colonies. Measurements were performed at indicated absorbance (Abs). *P* value was calculated with 2-sided *t* test. (**D**) Cell anchorage-independent growth of MITF-methylated SOX10^–^ group (black) and SOX10^+^ group (red) does not show significant differences in cell viability at 48-hour time point. As seen microscopically, SOX10^–^ cells cluster in spheroid elongated structures, while the SOX10^+^ group remains spread in a single-cell suspension. *P* value was calculated with 2-sided *t* test. (**E**) Treatment of SOX10^+^ (red) and SOX10^–^ cells (black) with increasing concentrations of BRAF inhibitor shows complete resistance of the SOX10^–^ cells. *P* value was calculated with 1-way ANOVA with Dunnett’s multiple-comparison test.

Melanomas with low *MITF* expression have previously been associated with an aggressive phenotype in vitro and in vivo (1, 14, 26). However, the functional difference between distinct subsets of such melanomas has not been fully explored. To analyze this, we first assessed the proliferative capacity of each *MITF*-methylated melanoma subset in vitro. Using a total of 6 melanoma cell lines (Supplemental Figure 2), we found that the SOX10^–^ cell lines (IGR-39, LOX, A7) had an increase in proliferative capacity after 96 hours in culture as compared with the SOX10^+^ cell lines (MM383, WM278, WM852) (Figure 3A, P = 0.0001; Supplemental Figure 3, *P* < 0.001). Next, we used a Transwell migration assay to measure the migratory properties of these 6 melanoma cell lines. The 3 SOX10^–^ melanoma cell lines showed increased migratory potential as compared with the SOX10^+^ melanoma cell lines (Figure 3B, *P* = 0.000002). The 3 different SOX10^–^ melanoma cell lines also displayed increased colony formation capacity in a 2-week assay, and colonies appeared structurally and morphologically different at the microscopic level (Figure 3C, *P* = 0.0001). Specifically, melanoma cells from the different SOX10^+^ cell lines clustered in small and compact structures maintaining tight cell-to-cell contact, while melanoma cells from the SOX10^–^ cell lines formed considerably larger colonies in which the cells tended to segregate and distribute sparsely (Figure 3C). We further tested the ability of anchorage-independent growth of the melanoma cells. After 48 hours of cell growth in ultra-low-attachment plates, we did not observe any significant difference in cell viability between the SOX10^+^ and SOX10^–^ melanoma cell lines (Figure 3D). As both subgroups of the *MITF* promoter–methylated cells remained viable in the low-attachment environment, this suggests that *MITF* methylation is an independent factor for these cells to overcome anoikis. However, melanoma cells from the SOX10^–^ cell lines (IGR-39 and LOX) formed aggregates whereas the melanoma cells from the SOX10^+^ cell lines (MM383 and WM278) grew as single-cell suspensions, indicating an added property to mimic the structural character of microtumors (Figure 3D). Finally, we subjected SOX10^+^ (MM383) and SOX10^–^ (IGR-39) melanoma cell lines to increasing concentrations of BRAF inhibitor. While we verified that the *MITF*-methylated subgroups harbored high resistance to this treatment, we observed that the drug had virtually no effect on the survival of the SOX10^–^ melanoma cell line (Figure 3E, *P* = 0.001).

Conclusively, melanoma cells with concurrent *MITF* and *SOX10* promoter methylation are phenotypically different from the *MITF*-methylated SOX10^+^ cells.

### In vivo and ex vivo characterization of MITF-methylated melanoma cells.

**Figure 4:**
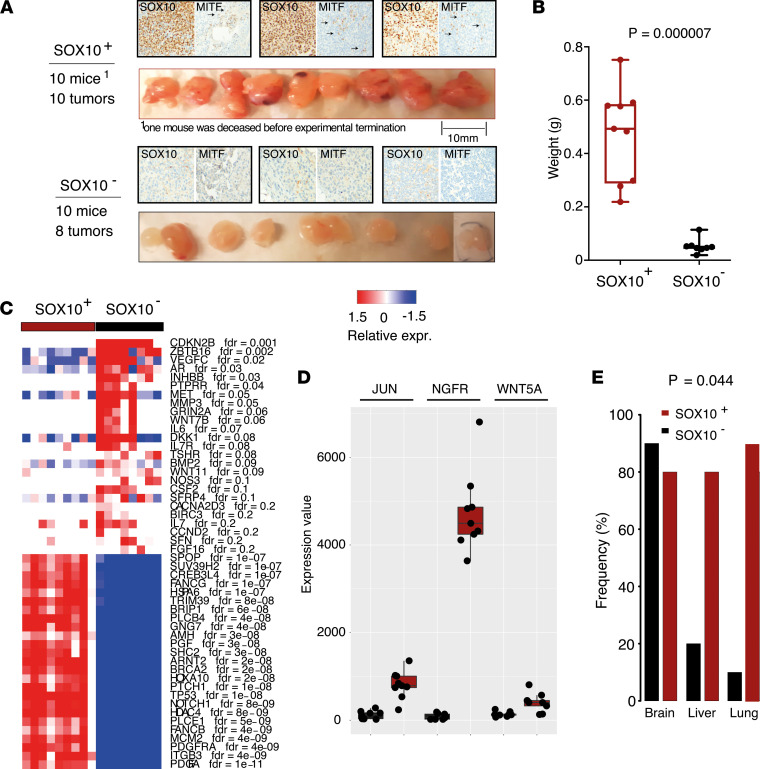
In vivo characterization of MITF-methylated melanomas. (**A**) Photos and staining of xenograft tumors from NSG mice injected with SOX10^+^ (MM383) or SOX10^–^ (IGR-39) melanoma cells. Tumors were analyzed for MITF and SOX10 protein expression using immunostaining. Arrows indicate MITF-positive melanoma cells. Original magnification, ×200. (**B**) Box plot showing weight differences between SOX10^+^ (red) and SOX10^–^ (black) cell line–derived tumors. *P* value was calculated using 2-sided *t* test. (**C**) Transcriptomic analysis using the NanoString PanCancer Pathways Panel describes significant differences between SOX10^+^ (red) and SOX10^–^ (black) derived xenograft tumors. (**D**) Box plot showing 3 selected genes (*JUN*, *NGFR*, *WNT5A*; FDR = 0) from the NanoString PanCancer Pathways Panel gene expression analysis. (**E**) Frequencies (%) of metastases detected in brain, liver, and lungs of the NSG mice injected with SOX10^+^ MM383 (red) and SOX10^–^ IGR-39 (black) melanoma cells. *P* value was calculated using χ^2^ test.

**Figure 5:**
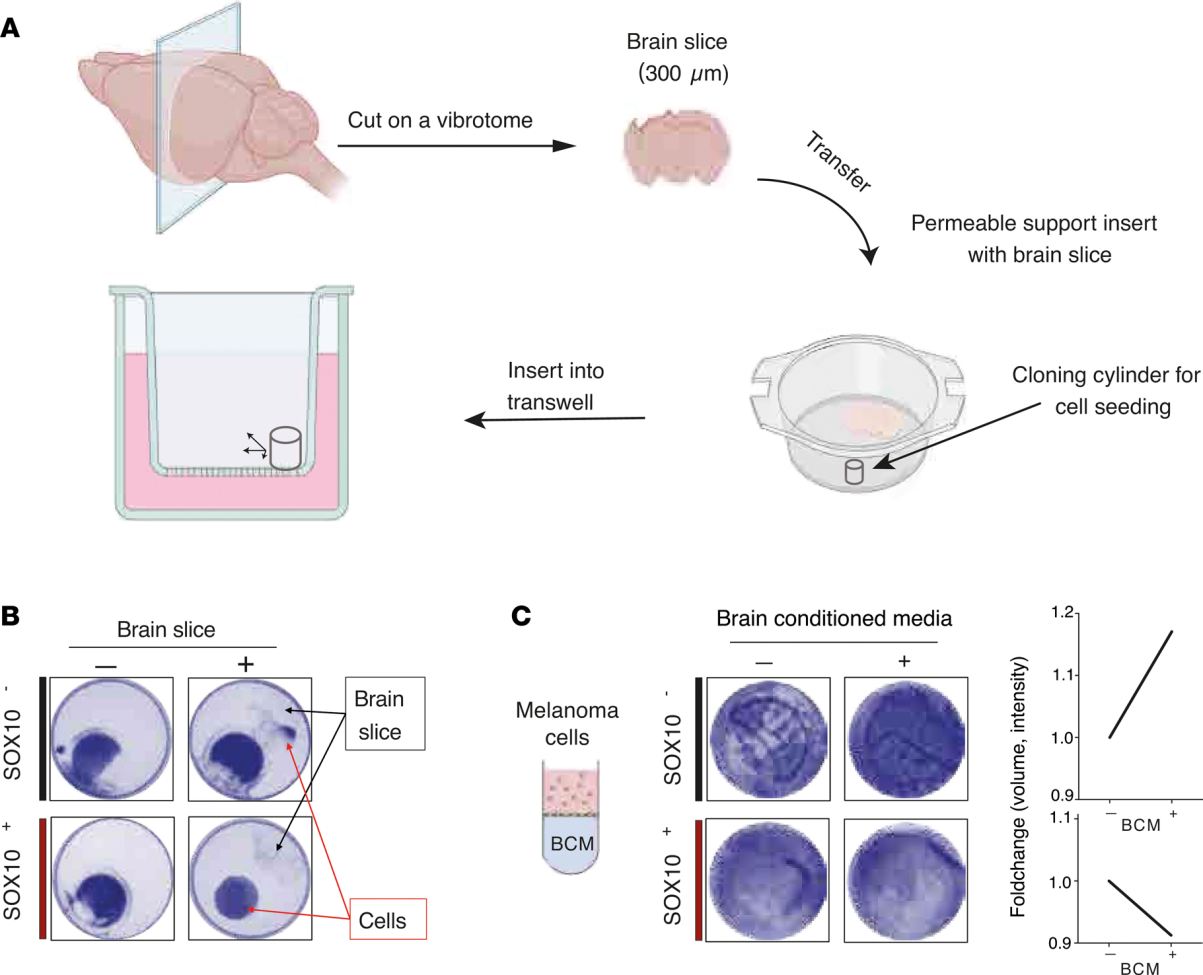
Ex vivo migration patterns in SOX10^–^ and SOX10^+^ melanoma cells. (**A**) Illustration of the workflow used in the ex vivo organotypic brain slice culture experiment. (**B**) Brain slice migration assays in SOX10^+^ MM383 (red) and SOX10^–^ IGR-39 (black) melanoma cell lines seeded onto membranes display SOX10^–^ cells moving toward the brain slice. (**C**) Transwell migration experiment of SOX10^+^ MM383 (red) and SOX10^–^ IGR-39 (black) melanoma cell lines using brain conditioned media shows increased migration of the SOX10^–^ IGR-39 cells.

Switching of melanoma phenotype or tumor microenvironmental adaptation of melanoma cells is a key feature of melanoma survival (32). To understand whether *MITF* promoter–methylated melanoma cells can undergo phenotype switching in NOD/SCID-γ (NSG) mice, we selected 1 cell line that expresses *SOX10* (MM383) and 1 that does not express *SOX10* (IGR-39). At experimental termination, in NSG mice transplanted with the SOX10^–^ IGR-39 melanoma cell line, only 8 out of the 10 mice developed tumors. In contrast, the SOX10^+^ MM383 melanoma cell line formed primary tumors in all 10 transplanted mice. Furthermore, primary tumors from NSG mice engrafted with SOX10^–^ IGR-39 melanoma cells were significantly smaller compared with primary tumors developed in NSG mice engrafted with SOX10^+^ MM383 melanoma cell line (Figure 4A). Strikingly, the primary tumors from NSG mice transplanted with SOX10^+^ MM383 melanoma cells showed variable shapes (mono-/bi-/multi-lobar), densities (solid/spongy/soft), and necrotic features, while the nonpigmented primary tumors from SOX10^–^ IGR-39 injected NSG mice were consistently characterized by confined margin, rounded shape, and clear color (Figure 4A). We further observed that the primary tumors from SOX10^–^ IGR-39 engrafted NSG mice remained negative for MITF and SOX10 protein expression, while we found scattered MITF^+^ melanoma cells in the primary tumors derived from SOX10^+^ MM383 engrafted NSG mice (Figure 4A). This supports the idea of cooperative behavior between melanoma cell states that leads to a faster tumor growth (33). In concordance with this, the final weight of tumors formed from SOX10^+^ MM383 was significantly higher than the SOX10^–^ IGR-39 tumors (Figure 4B, *P* = 0.00007). We then characterized the transcriptional changes in the developed primary tumors by NanoString nCounter PanCancer Pathways Panel and revealed a distinct separation between the SOX10^+^ and SOX10^–^ tumors, indicating that their difference in transcriptional profile persisted over the course of tumor growth in vivo. Furthermore, most genes included in the NanoString PanCancer Pathways Panel (*n* = 770) were differentially expressed between the 2 tumor groups (Figure 4C). Importantly, the molecular phenotype of the cells was sustained with increased expression of *NGFR*, *JUN*, and *WNT5A* in SOX10^+^MITF^–^ melanoma tumors (Figure 4D). Finally, we investigated the metastatic potential of the SOX10^+^ and SOX10^–^ cell lines by detection of human *GAPDH* (hGAPDH) by quantitative PCR (qPCR) analysis as previously described (34) in the liver, lung, and brain from the tumor-bearing mice. Interestingly, while mice injected with the SOX10^+^ melanoma cell line MM383 showed similar expression of human *GAPDH* in all organs tested, we detected increased presence of *hGAPDH* in the brains of mice injected with SOX10^–^ IGR-39 melanoma cells, compared with liver and lungs, suggesting a trend of preference in metastatic site for this subgroup (Figure 4E, P = 0.044). To pursue the observation of preferential brain metastasis in SOX10^–^ melanoma, we performed a melanoma cell migration experiment based on organotypic brain slice cultures. Briefly, we sectioned a mouse brain and cultured the slices on Transwell membranes directly following brain harvest. Melanoma cells were then seeded using a cloning cylinder to confine the initial growth on the opposite side in respect to the brain slice placed onto the same membrane. By removing the cloning cylinder from the now membrane-attached cells, we allowed them to migrate freely for 72 hours (Figure 5A). Supporting the in vivo results, the SOX10^–^ IGR-39 melanoma cells showed an increase in migration toward the brain slice, while the SOX10^+^ MM383 melanoma cells remained confined in their seeding position (Figure 5B). For further validation, conditioned media from the brain slice migration experiments was collected and used as a chemoattractant for melanoma cells in Transwell migration experiments (Figure 5C). Consistent with our previous findings, the SOX10^–^ IGR-39 cells showed a higher tendency to migrate toward the lower chamber containing the brain slice conditioned medium in comparison with the SOX10^+^ MM383 cells (Figure 5C). These data support that the SOX10^–^ melanoma cells preferentially migrated and metastasized to the brain.

Together, our findings further support the genetic and phenotypic distinction between the *MITF*-methylated subgroups. Primary tumors developed from the *MITF*-methylated SOX10^+^ and SOX10^–^ melanoma cells were molecularly different and had different preferential metastatic spread.

### Effects of CRISPR/Cas9-mediated SOX10 knockout in MITF-methylated melanoma cells.

**Figure 6:**
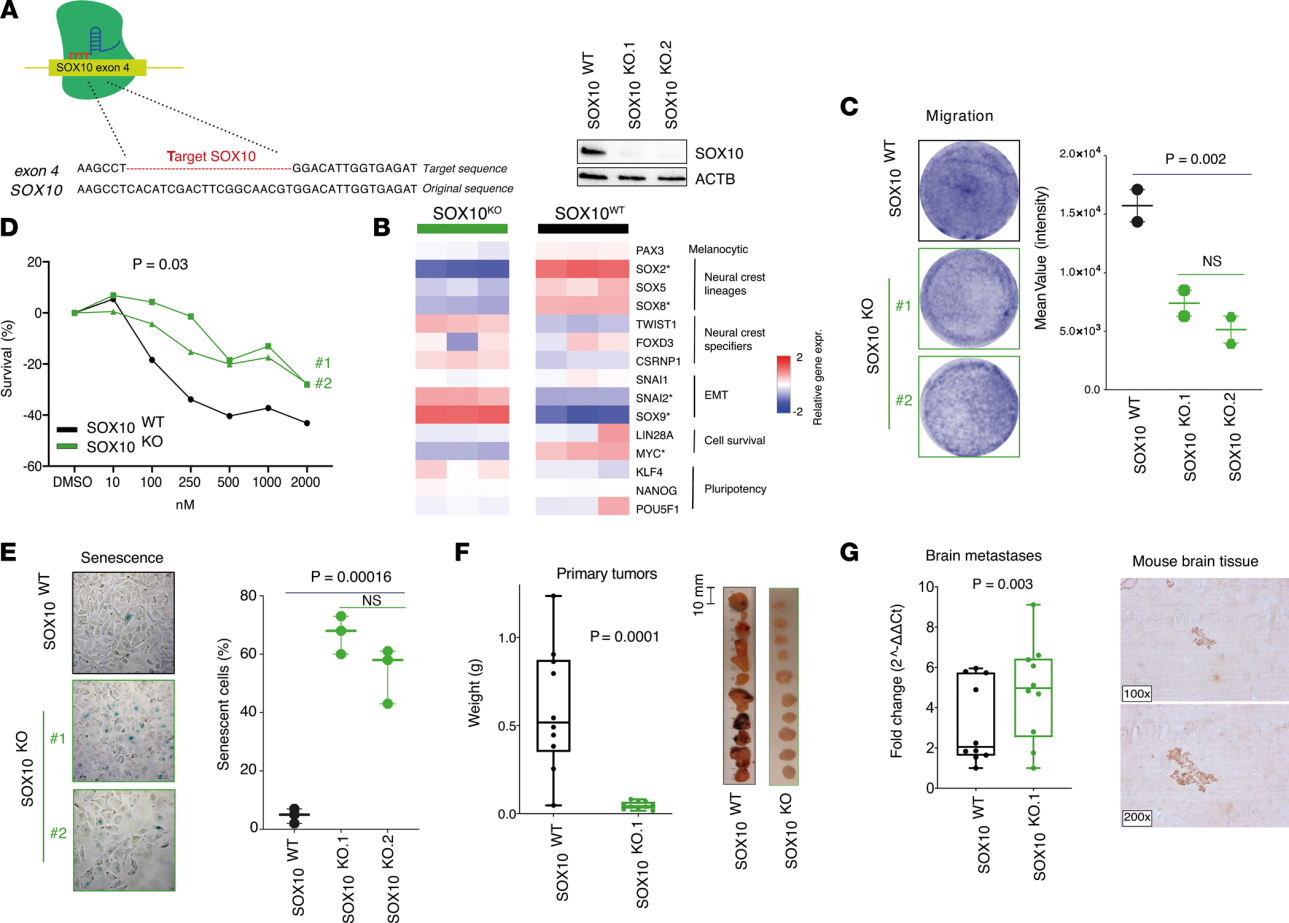
CRISPR/Cas9 editing of SOX10 in MITF-methylated melanomas. (**A**) Target sequence and confirmation of SOX10 KO by Sanger sequencing and Western blot in MM383 melanoma cells. (**B**) Transcriptomic analysis describes differences in the expression levels of NC-associated genes between SOX10^WT^ (black) and SOX10^KO^ (clone 1, green) cells. * indicates significantly different expression (FDR = 0). (**C**) Migration of SOX10^WT^ (black) and SOX10^KO^ (green) cells analyzed using a Transwell assay shows decreased migratory potential of the SOX10^KO^ clones. *P* value was calculated using Mann-Whitney-Wilcoxon test. (**D**) Treatment of SOX10^WT^ (black) and SOX10^KO^ (green) cells with BRAF inhibitors shows increased resistance of the SOX10^KO^ clones compared with wild-type cells. *P* value was calculated using 1-way ANOVA with Dunnett’s multiple-comparison test. (**E**) β-Galactosidase staining used to measure the fraction of senescent cells in SOX10^WT^ (black) and SOX10^KO^ (green) displays higher senescent cell count in the SOX10^KO^ clones. *P* value was calculated using Mann-Whitney-Wilcoxon test. Original magnification, ×400. (**F**) Box plot showing difference in weight between SOX10^WT^ (black) and SOX10^KO^ (green) derived primary tumors and tumor photos. *P* value were calculated using Mann-Whitney-Wilcoxon test. (**G**) qPCR analysis of human GAPDH in brain tissues from SOX10^WT^ (black) and SOX10^KO^ (green) melanoma-injected NSG mice. Representative immunostaining of SOX10^KO^ mouse brain tissue using human nuclear mitochondria antibody. *P* value was calculated using Mann-Whitney-Wilcoxon test.

In order to discern the role of SOX10 in determining the aggressive phenotype of *MITF*-methylated melanoma, we specifically targeted *SOX10* by CRISPR/Cas9 to generate a SOX10 knockout (SOX10^KO^) *MITF*-methylated melanoma cell line with *SOX10*-unmethylated promoter CpGs (Figure 6). Since both SOX10 and MITF have fundamental roles in developing melanocytes and in melanoma progression (35, 36), depletion of *SOX10* in an MITF^lo^ background resulted in high lethality for most of the melanoma cell cultures tested. We tested 3 *MITF*-methylated melanoma cultures, and stable SOX10^KO^ clones were generated exclusively in the MM383 cell line. We inquired the newly curated SynLethDB (37), containing CRISPR screen data analyses of KO gene combinations, and observed synthetic lethality of *SOX10^KO^* in cells with concurrent depletion of phosphatase and tensin homolog (*PTEN*). By matching mutational data from the cell lines tested, we indeed verified MM383 to be the only MITF-methylated cell line being *PTEN* wild-type. Indeed, hypermethylation of promoters has been shown to be dependent on high levels of reactive oxygen. Also, PTEN can be oxidatively inactivated in tumors with very high levels of reactive oxygen, thus preventing the need for mutation or deletion of PTEN (38–40). Therefore, 2 synthetic guide RNAs (gRNAs) were designed to target exon 4 of the *SOX10* gene in the MM383 cells (Figure 6A). These gRNAs were individually cotransfected with the Cas9 protein and GFP plasmids via electroporation. The GFP^+^ cells were then single-cell-sorted through FACS and the monoclones were expanded. Finally, colonies were screened by Sanger sequencing for *SOX10* insertions or deletions, and the absence of SOX10 protein expression was confirmed by Western blot (Figure 6A). In total we obtained 2 different SOX10^KO^ clones. As a control we used 1 clone that had been transfected with scrambled gRNA. Since SOX10 has been identified as a key transcriptional regulator of NCC development, we further assessed the expression of NC-associated genes by RNA-Seq (31) (Figure 6B). Seven genes were differentially expressed, including 3 SOX genes (*SOX9*, *SOX2*, and *SOX8*). In particular, *SOX9* was upregulated, suggesting that SOX10^KO^ cells are more similar to premigratory NCCs and thus are less lineage restricted (41). Indeed, using a Transwell migration assay, we found SOX10^KO^ cells to have an inferior migratory capacity (Figure 6C, *P* = 0.002). SOX10^KO^ cells were also more resistant to BRAF inhibition (Figure 6D, *P* = 0.03) and had a higher fraction of senescent cells as measured by β-galactosidase staining (Figure 6E, *P* = 0.000016) as compared with wild-type cells. Finally, we found that NSG mice injected with SOX10^KO^ cells formed significantly smaller primary tumors compared with those injected with SOX10 wild-type cells (Figure 6F, *P* = 0.0001). To understand whether cells preferentially formed brain metastasis due to SOX10 depletion, we further analyzed the mouse brains by measuring the presence of hGAPDH expression by qPCR. Strikingly, we detected the presence of melanoma cells in 80% of the brains in mice injected with the SOX10^KO^ clones, corresponding to 2-fold increase in comparison with the SOX10^WT^-injected group. This is concordant with the finding previously observed for the SOX10^–^-injected mice, reinforcing that SOX10 depletion in *MITF*-methylated cells enhances the development of brain tropism (Figure 6G, *P* = 0.003). Finally, we confirmed the presence of melanoma cell clusters in the mouse brain by immunostaining of human nuclear protein (Figure 6G), concordantly enriched in mice injected with SOX10^KO^ cells.

In conclusion, we generated SOX10^KO^ clones using CRISPR/Cas9 in an *MITF*-methylated melanoma background. Such clones had lost NC-specific transcriptional properties, rendering them less migratory in vitro but more prone to spread to the brain in vivo.

## Discussion

The NC is a transient population of cells giving rise to a wide array of cell types that can be categorized into craniofacial skeleton (mesenchymal cells), other mesenchymal cells, cells of the peripheral nervous system, endocrine cells, and melanocytes. Melanoma, originating from pigment-producing melanocytes, is a highly aggressive cancer. Two transcription factors crucial to the NC and melanocyte development have fundamental functional properties also in melanoma development and progression. In this study, we determine the functional and transcriptional phenotypes of melanoma cells with retained or loss of *MITF* and *SOX10* expression, respectively. Corroborating previous studies (12, 42), we observed that the transcriptional landscape across a large collection of melanoma cell lines was mainly driven by differences in expression levels of melanocytic genes. Moreover, validating previous reports (21), the subset of cell lines with decreased *MITF* gene expression was enriched with cases harboring *MITF* promoter methylation, suggesting that epigenetic mechanisms are crucial in the regulation of the melanoma cell differentiation state. In this study, we further demonstrate that *MITF* gene expression levels are also correlated to *MITF* promoter methylation status in tumors. While loss of MITF at transcriptional level has recently been described as a marker of resistance to BRAF/MEK targeted therapy (1, 14), we also demonstrate in an independent melanoma cohort by immunostaining that loss of MITF at protein level is an inferior prognostic factor in stage III metastatic melanoma.

The most updated model of phenotypic plasticity in melanoma includes 6 cell states, including 2 with low expression of MITF (20). The NCSC-like melanoma cells express the transcription factor *SOX10*, while the undifferentiated melanoma cells have concomitant loss of *MITF* and *SOX10*. In addition, Tsoi et al. found that melanoma cells expressing *MITF* and *SOX10* can revert to an undifferentiated subtype under therapeutic stress (12). In this study, we further establish that MITF^lo^ melanoma cells exist as either NCSC-like or as undifferentiated. Interestingly, when we analyzed the expression of NC-specific genes, we found that SOX10^–^ undifferentiated melanoma cell lines had downregulation of genes expressed in normal melanocytes, with the exception of acquired expression of *SOX9*. In comparison, the SOX10^+^MITF^–^ NCSC-like melanoma cell lines had acquired expression of other NC-derived lineage markers such as *SOX2* (expressed in glial lineages) (43), *SOX5*, and *SOX8* (expressed in neural progenitors). This suggests that the NCSC-like melanoma cells had acquired expression of NC lineage markers, while undifferentiated cells had lost most of their NC- and melanocyte-specific properties and regressed to a pre-NC-like state. In our study, we found MITF^lo^SOX10^–^ melanoma cell lines to be more proliferative, migratory, and resistant to BRAF inhibition, which was also supported in the study by Capparelli et al. (44). However, when injecting such cells in NSG mice, we observed that the undifferentiated SOX10^–^ melanoma cells developed significantly smaller tumors compared with SOX10^+^MITF^–^ melanoma cells. This discrepancy may be due to the fact that the MITF^lo^SOX10^–^ population depend on other melanoma subpopulation to grow in vivo (32). Importantly, MITF immunostaining of SOX10^+^ and SOX10^–^ primary tumors from NSG mice detected a subpopulation of MITF^+^ melanoma cells in primary tumors from NSG mice injected with SOX10^+^MITF^–^ melanoma cells, while such melanoma cells were not found in SOX10^–^ NSG primary tumors (Figure 4A). This suggests a cooperative behavior between MITF^+^ and MITF^–^ melanoma cells particularly occurring in vivo. Indeed, cooperativity between mesenchymal and melanocytic melanoma states was recently demonstrated to be associated with faster tumor growth (33). Moreover, analyses of SOX10^–^ and SOX10^+^MITF^–^ tumors developed in NSG mice at molecular level suggested a profound difference between the tumor types and reflected the tumor growth difference and sustained increased expression of *NGFR*, *JUN*, and *WNT5A* in the SOX10^+^MITF^–^ melanoma tumors. Such *NGFR*-high melanomas have previously described to be refractory to MAPK inhibition and anti–programmed cell death 1 therapy (27), and we hereby show that such melanoma cells develop rapidly growing melanomas with sustained dedifferentiation status in vivo and have scattered MITF^+^ melanoma cells in the tumor. Such tumors were larger, underlining a possible cooperation between MITF^–^ and MITF^+^ melanoma cells and the tumor microenvironment in vivo as described previously (45).

To further explore the differences observed between in vitro and in vivo findings, we used CRISPR/Cas9 to deplete SOX10 in an *MITF*-methylated melanoma cell background. Supporting the results from the melanoma cell lines, we found increased expression of *SOX9* and loss of expression of *SOX2*, *SOX5*, and *SOX8*, suggesting that SOX10^KO^ cells reverted to a pre-NC state. Indeed, *SOX2* expression characterizes the neural plate. However, it is also expressed by NCCs restricted to the glial lineage (43). Downregulation of *SNAI2* in SOX10^KO^ cells was also observed. Indeed, SNAI2 was previously described to downregulate E-cadherin in premigratory NCCs, reverting them into mesenchymal-like cells (46). In accordance with this, we found that SOX10^KO^ cells were less migratory and more senescent. Moreover, NSG mice injected with SOX10^KO^ cells developed smaller primary tumors, similarly to the SOX10^–^ cell lines, which is consistent to our in vitro findings. By contrast, the SOX10^KO^ cells were less migratory in vitro when using FBS as a chemoattractant. This finding could be partially explained by intrinsic differences in transcriptional programs between SOX10^–^ and SOX10^KO^, leading to separate biological entities relying on alternative pathways to compensate SOX10 absence rather than artificial depletion. However, SOX10^KO^ engineered melanoma cells displayed more aggressiveness in vivo as demonstrated by the presence of more melanoma cells in the mouse brain, compared with wild-type cells. This suggests an enhanced attraction specific to secreted brain factors in the SOX10^KO^ cells. In fact, using conditioned media from sliced brain ex vivo culture as a chemoattractant in a Transwell migration assay with spatial separation in coculturing the MITF^–^ melanoma cells together with ex vivo brain slice showed in unison that the SOX10^–^ cells migrated with an increased rate toward the conditioned media and brain tissue, respectively, compared with the SOX10^+^ melanoma cells. Importantly, up to 40% of patients with advanced melanoma disease present with brain metastases and a particularly poor prognosis. In the current study, we found *SOX10* expression to be regulated by DNA methylation, which is in line with a recent study where SOX10^–^ glioblastoma cells also harbored features that are more aggressive and a mesenchymal phenotype (47).

Our analyses of dedifferentiated melanomas support 2 distinct phenotypes with similarities in *MITF* promoter methylation but differences in *SOX10* mRNA levels and DNA methylation status. Moreover, expression of NC lineage genes was lost in SOX10^–^ melanoma cells, suggesting that such cells have lost several NC-specific properties. In contrast, SOX10^+^MITF^–^ melanoma cells had acquired expression of several NC lineage genes as compared with differentiated melanocytes. Such changes in dedifferentiated melanomas are also reflected in in vitro and in vivo assays, where SOX10^–^ melanomas have enhanced propensity to metastasize to the brain as compared with lung and liver in mice. Our findings further describe that dedifferentiated melanoma cells injected in vivo sustained their transcriptional phenotype. The existence of dedifferentiated melanoma cells in tumors may explain targeted therapy and immunotherapy resistance, and further investigation of combination drugs mediating cell differentiation and immunotherapy is warranted.

## Methods

### Cell lines

The melanoma cell lines used in this study were purchased from ATCC or provided by the lab of Hensin Tsao (Department of Dermatology, Harvard Medical School, Boston, Massachusetts, USA) and cultured according to ATCC guidelines. Specifically, MM383 and LOX were grown in RPMI-1640 containing l-glutamine (Nordic Biolabs) and IGR-39 in DMEM-high glucose (Nordic Biolabs). A7 were grown in Eagle’s Minimum Essential Medium (ATCC) added with 10 mM HEPES (Nordic Biolabs) and 0.5 mg/mL geneticin (Invitrogen). All abovementioned media were supplemented with 10% FBS (Gibco) and 1% penicillin–streptomycin (p/s) (Gibco). WM278 and WM852 were grown in 2% FBS Tumor Medium (Tu2%) containing a 4:1 mixture of MCDB 153 medium (Gibco) with 1.5 g/L sodium bicarbonate (MilliporeSigma) and Leibovitz’s L-15 medium (Gibco) with 2 mM l-glutamine (Gibco) supplemented with 0.005 mg/mL bovine insulin (Gibco), 1.68 mM CaCl_2_ (MilliporeSigma), and 2% FBS (Gibco). The mutational status of melanoma cell lines was determined by targeted DNA sequencing as described previously (48).

### Immunohistochemistry staining

For a representative view of the tumors, an average of 3 cores of 1 mm per tumor were mounted on tissue microarrays. For each tissue block, a 4 μm section was cut and dried at 60°C for 1 hour. The sections were deparaffinized and pretreated in the PT-Link (Dako) with Target Retrieval Solution buffer at pH 9. The following steps (except for the primary antibody staining) were performed in the Dako staining equipment (Autostainer plus) with Dako kit K8010 solutions: peroxidase block (5 minutes), EnVision HRP-conjugated polymers for 30 minutes, DAB substrate-chromogen solution twice for 5 minutes, and counterstaining with hematoxylin for 4 minutes. Between the steps, the sections were rinsed with washing buffer. Finally, the sections were dehydrated and mounted with Pertex mounting medium (Histolab). The primary antibodies used from Dako were MITF (clone C5, Biocare) in 1:100 dilution and SOX10 (clone BC34, Biocare) in 1:100 dilution. Cores were scanned and uploaded in PathXL (Philips) platform to be further analyzed.

Mouse brains from in vivo assays were processed in the same way and stained by anti-mitochondria antibody 113-1 (catalog ab92824, Abcam) to visualize human melanoma metastatic cells.

### Dot blot global methylation assay

In order to assess global DNA methylation in the MITF-methylated subgroups, anti–5-mC dot blot assay was used as reported previously (49). Briefly, samples were denatured at 99°C for 10 minutes, transferred on ice immediately, and neutralized in 0.1 vol of 6.6 M ice-cold ammonium. Then, DNA was spotted onto nitrocellulose membranes (1 ng in 5 μL per sample) and air-dried for 15 minutes. The membrane was UV–cross-linked (20 seconds, 1,200 J/cm^2^) and blocked in 5% blocking solution at room temperature (RT) for 2 hours. Finally, the membrane was incubated with the anti–5-mC monoclonal antibody for 2 hours (clone 7D21, 1:2,000 dilution) (Zymo Research), washed 3 times in PBS/Tween, and incubated with the secondary antibody anti-mouse for 1 hour (1:10,000 dilution; polyclonal, Thermo Fisher Scientifc, catalog 62-6520). Positive and negative dsDNA controls (100% and 0% methylated, respectively) were titrated (5, 10, 20, and 100 ng) in Tris-EDTA buffer pH 8 to 10 μL and used for reference.

### EPIC arrays, RNA-Seq, and bisulfite Sanger sequencing

DNA was extracted from patient samples as described previously (50), and bisulfite conversion was performed using EZ DNA methylation kit (Zymo) according to the manufacturer’s instructions, followed by Sanger sequencing using the same primers and protocols as described in Lauss et al. (21). Melanoma cell lines were analyzed using Illumina Infinium methylation EPIC BeadChip array. Methylation data for tumor samples have been deposited to GEO with accession number GSE144487. RNA-Seq data were processed as previously described using TopHat2 and Cufflinks v2.1.1. Isoform fragments per kilobase million were summed up to obtain gene-level expression (51). Data are deposited in GEO under accession number GSE211906 (Supplemental Table 1).

Preprocessing of DNA methylation data from patient samples and melanoma cell lines

Raw idat files were processed using R package ChAMP (52), and background correction was performed using ssNoob (53, 54) from package minfi (55). Furthermore, type I/II probes were normalized using BMIQ (56) and filtered for polymorphic and off-target probes (57). For patient samples, samples with a large number of failed probes (probe detection *P* > 0.01; sample cutoff > 4% of total probes) and probes that failed in more than 10% of the remaining samples were removed. Next, methylation β values of probes that failed in 10% or less samples were imputed using the impute.knn function in R and default settings. After preprocessing, the patient tumor methylation data set contained 788,174 probes and 196 samples. The processed methylation data for the Lund cohort is available in the GEO database, under accession number GSE144487. Preprocessing of DNA methylation profiles from 14 *MITF* promoter–methylated melanoma cell lines was done for the patient samples, except that any probe above the *P* value cutoff in 1 or more samples was removed, instead of imputing the methylation β value.

### Data sets

Microarray expression data using Illumina HT12 arrays, as previously described in Cirenajwis et al. (1) and Rizos et al. (29), were derived from GEO, GSE65904 and GSE50509. Mutation data from 1,697 cancer-associated genes were derived from Cirenajwis et al. (1). Methylation data for The Cancer Genome Atlas (TCGA) skin cutaneous melanoma melanoma samples (*n* = 475) from the TCGA data portal (https://tcga-data.nci.nih.gov/tcga/) consisting of level 3 β values from the Illumina HumanMethylation450 array, were processed as described previously (58). Briefly, for the Infinium I and II assays of each sample, the data were scaled to move the unmethylated β value peak to β value of 0 and the methylated peak to 1; values below 0 and above 1 were set to 0 and 1, respectively.

### Lysis and Western blot

Proteins from cell lines for Western blot assays were extracted using M-PER lysis buffer (Thermo Fisher Scientific) supplemented with Halt Protease and phosphatase inhibitor cocktail (Thermo Fisher Scientific) at 4°C for 10 minutes, and concentrations were determined by Bradford assay (Thermo Fisher Scientific). Prior to blotting, proteins were denatured in sample buffer (10% glycerol, 2% SDS, 62.5 mM Tris-HCL, pH 6.8) added with 10% β-mercaptoethanol and boiled for 5 minutes at 99°C. Western blots were performed as follows: samples were run in 4%–20% TGX stain-free gels (Bio-Rad) for 40 minutes at 180 V, and electrophoretic transfer was performed by Trans-Blot Turbo (Bio-Rad) onto PVDF membranes (Bio-Rad). Membranes were blocked in 5% Blotting-Grade Blocker for 1 hour at RT and washed 3 times in PBS-Tween (Medicago) prior to antibody staining. Antibodies used in this study are listed: anti-SOX10 1:2,000 dilution (Atlas Antibodies, catalog HPA068898), anti-MITF 1:2,000 dilution (Atlas Antibodies, catalog HPA003259), and β-actin 1:5,000 dilution (MilliporeSigma, catalog A5441).

### Cell proliferation and viability

#### Sulforhodamine B staining.

To quantify cell proteins in cultured cells, sulforhodamine B (SRB) was used as a fluorescent dye according to the manufacturer’s instructions (Thermo Fisher Scientific). Briefly, medium was removed, and plates were washed in PBS. Cells were fixed in ice-cold 17% trichloroacetic acid for 1 hour at 4°C, washed 5 times in distilled water, and air-dried before the addition of SRB (0.4% *w/v* in 1% acetic acid; MilliporeSigma). After staining for 20 minutes at RT, plates were washed 5 times with 1% acetic acid and allowed to air-dry. Last, 10 mM Tris base (unbuffered, pH > 9) was added to each well to dissolve the dye, and plates were read at 570 nm in a FLUOstar Omega (BMG LabTech) microplate reader.

#### CellTiter-Glo.

Cell viability was assessed using the CellTiter-Glo Luminescent Cell Viability Assay (Promega) according to the manufacturer’s protocol. Briefly, CellTiter-Glo (1:2 reagent to media ratio) was added directly to the cells in the cell culture media, and the plates were incubated for 10 minutes prior to luminescent detection in a FLUOstar Omega (BMG LabTech) microplate reader at 560 nm.

#### xCELLigence.

xCELLigence real-time cell analysis system (ACEA Biosciences) was used for label-free and real-time monitoring of cell viability according to the manufacturer’s instructions. Briefly, cells were seeded in microtiter plates containing interdigitated gold microelectrodes to noninvasively monitor the viability of cultured cells using electrical impedance as readout.

#### Senescence β-galactosidase staining.

The senescence β-galactosidase staining kit (Cell Signaling Technology) was used according to the manufacturer’s protocol.

### Migration assay

Melanoma cells were starved for 15 hours and then seeded in low-FBS-containing medium (0.1%) in the upper compartment of a 6.5 mm Transwell with 8.0 μm pore polycarbonate membrane insert into 24-well culture plates (MilliporeSigma Corning Transwell inserts, 5 × 10^4^ cells/well). To drive cell migration, the lower compartment was filled with complete media including FBS as chemoattractant, and plates were incubated at 37°C and 5% CO_2_. After 72 hours, cells that had not migrated to the lower chamber were scraped off with a cotton bud. The membranes were fixed in 100% methanol for 10 minutes at RT and cells stained with crystal violet (0.5%, MilliporeSigma) in 25% methanol for 10 minutes at RT. The excess was washed away in tap water. After air-drying, the stained Transwell membranes were cut out with a scalpel, mounted onto microscope slides, and analyzed using Image Lab software (Bio-Rad) to quantify the migrated cells. Experiments were done in triplicates and negative controls consisting of 0.1% FBS containing media in the lower chamber were included.

### Clonogenic assay

Melanoma cells were plated in triplicates into 6-well cell culture dishes (500 cells/well) and allowed to form colonies over 2 weeks. Colonies were then stained with crystal violet as described in the section above and counted under the microscope. Absorbance measurements were acquired in a FLUOstar Omega (BMG LabTech) microplate reader at 570 nm after dissolving the dye in 95% ethanol for 10 minutes in agitation.

### Anchorage-independent assay

Melanoma cells (1,000 cells/well) were seeded in triplicates into ultra-low-attachment surface 96-well cell culture dishes (Corning) and incubated at 37°C in 5% CO_2_ for 48 hours. Pictures were taken under the microscope (Olympus IMT-2), and viability was measured by CellTiter-Glo (Promega) as described above at 560 nm.

### Targeted inhibitor assays

Inhibitor compounds were purchased from SelleckChem: vemurafenib (PLX4032, RG7204). Drug assays were performed as follows: melanoma cells were seeded in appropriate medium into 96-well tissue culture plates (5 × 10^3^ cells/well). Cells were allowed to adhere at 37°C in 5% CO_2_ before being treated with the reported concentrations for 72 hours (vemurafenib). Cell viability was assessed by SRB staining as described above.

### In vivo assay

NSG mice (10 per group) were subcutaneously injected into the flank with the MM383 MITF-methylated SOX10^+^ or the IGR-39 MITF-methylated SOX10^–^ (3 × 10^6^ cells) and in a separate experiment with the MM383 SOX10^WT^ or CRISPR/Cas9-mediated SOX10^KO^ (5 × 10^6^ cells), in 1:1 ratio with Matrigel (Corning) and weighed at regular intervals. Dimensions of the primary tumors were measured by using a caliper and the volumes calculated with the ellipsoid formula (L × W × W × π/6). When the largest tumor volume reached 1 cm^3^, all mice were sacrificed at once. Tumors and organs of the mice were harvested and stored in RNA*later* (Thermo Fisher Scientific) and in paraffin blocks for further analyses.

### NanoString gene expression analysis

Core punch samples were taken from paraffin-embedded mouse melanoma primary tumors for RNA extraction and profiling. The NanoString nCounter PanCancer Pathway Panels assay, containing 770 cancer pathway genes, was performed according to the manufacturer’s instructions. The obtained count data were normalized using the NanoString nSolver software: background thresholding was applied to set the minimum value to 20 (default), and a scaling factor, derived from the geometric mean of the positive ERCC control probes, was applied to each sample. The data were log-transformed as log_2_[data] – log_2_[20], and positive and negative control probes were removed (Supplemental Table 2).

### Melanoma cells detection by qPCR

The organs of the xenograft models were harvested and stored in RNA*later* for RNA extraction, followed by qPCR to quantify the presence of human cells by human GAPDH, normalized by mouse GAPDH in the mouse tissue as done previously using a cutoff of the normalized GAPDH fold change set at 1.5 according to a previous publication (59). Total RNA was extracted using RLT buffer (QIAGEN) with 1% β-mercaptoethanol, then homogenized in the TissueLyser II sample disrupter (QIAGEN). The High Capacity cDNA Reverse Transcription kit (Life Technologies) was used to perform reverse transcription according to the manufacturer’s instructions. cDNA was amplified by qPCR using TaqMan Assays (Thermo Fisher Scientific) with human GAPDH probes (Hs99999905_m1, Thermo Fisher Scientific) to detect human melanoma cells, and mouse GAPDH probes (Mm03302249_g1, Thermo Fisher Scientific) were included for normalization. Each reaction was run in triplicates in an ABI QuantStudio 7 Flex System. The presence of human melanoma cells was evaluated by GAPDH detection using the cutoff of the normalized GAPDH fold change set at 1.5, according to a previous publication (59).

### Ex vivo assays

#### Preparation of cell lines.

SOX10^+^ melanoma cells MM383 and SOX10^–^ IGR-39 were grown in DMEM-F12 medium (Gibco) supplemented with 2% FBS (Thermo Fisher Scientific), 1% p/s (Gibco), 2 mM glutamine (Thermo Fisher Scientific), and 2% B-27 without vitamin A (Gibco) and seeded in membrane inserts (1.2 × 10^5^ cells/well). Cells were seeded in an 8 mm diameter Pyrex cloning cylinder (Corning) placed on a Transwell membrane. The cells were then allowed to adhere for 4 hours. A vibrating blade microtome–prepared (Leica VT1200 S) whole mouse brain slice was placed onto the Transwell membrane on the opposite side of the seeded cells (see animal and slice preparation description below) (Figure 5A). The cloning cylinder was then removed, and the cells were allowed to migrate freely for 96 hours. Experiments were performed in triplicates and negative controls consisted of either seeded cells only or a brain slice only.

#### Preparation of whole brain slices.

Animals were sacrificed by decapitation under isoflurane anesthesia, and the brain was quickly removed and transferred to 4°C sucrose-containing artificial cerebrospinal fluid, containing (in mM) sucrose 75, NaCl 67, NaHCO_3_ 26, glucose 25, KCl 2.5, NaH_2_PO_4_ 1.25, CaCl_2_ 0.5, and MgCl_2_ 7 and bubbled with carbogen (95% O_2_ and 5% CO_2_). Horizontal, whole brain slices (300 μm) were cut using a vibrating blade microtome, collected, and placed in a holding chamber with carbogen-bubbled washing solution containing Hanks’ balanced salt solution and (in mM) NaCl, 138; Na_2_HPO_4_, 0.33; CaCl_2_, 1.3; MgCl_2_, 0.5; MgSO_4_, 0.4; KCl, 5.4; KH_2_PO_4_, 0.4; d-glucose, 5.5; HEPES, 20. Drops of NaOH (10 mM) were added to adjust pH to 7.3, and osmolarity was adjusted to 305–315 mOsm. Slices were washed for 15 minutes in 6-well plates and transferred using a blunt glass pipette to culturing membrane inserts. The membranes were fixed and stained by crystal violet as described in the “Migration assay” section above.

### Migration assay in brain slice conditioned media

The migration assay in Transwells was performed as described in the “Migration assay” section above, except for media used. Cells were starved in the same media and media supplements as in the ex vivo assay for 15 hours, then seeded in the top chamber (5 × 10^5^ cells/well). The bottom chambers were filled with concentrated brain slice conditioned media in complete DMEM-F12. Experiments were done in triplicates and negative control with no-brain-sliced released factors was included. Cells were allowed to migrate for 48 hours prior to Transwell staining with crystal violet.

### CRISPR/Cas9-mediated SOX10 KO

A pair of gRNA oligonucleotides (Eurofins and GeneArt Precision gRNA synthesis kit, Thermo Fisher Scientific) was designed (Benchling) to target the coding sequence in exon 4 of *SOX10*. CRISPR/Cas9 scrambled gRNAs was used as control. Cas9 protein (TrueCut V2 Thermo Fisher Scientific, 1.25 mg per sample) was incubated with 120 ng of the upstream and downstream flanking gRNA in a 10 mL final volume of Cas9 reaction buffer (100 mM NaCl, 5 mM MgCl_2_, 20 mM HEPES, 0.1 mM EDTA, pH 6.5 at 25°C) for 10 minutes at RT. This 10 mL of CRISPR/Cas9 complex was then added to 10^5^ cells suspended in 20 mL of the SF Nucleofector Solution (Lonza). The cell solution was transferred to 16-well Nucleovette strips (Lonza) and nucleofected in the 4D Nucleofector System (Lonza) using the CM-137 Nucleofector program. Finally, electroporated cells were transferred to a 24-well plate with preheated cell culture media and incubated for 48 hours before monoclonal selection. CRISPR/Cas9-edited cells were verified with Sanger sequencing (Eurofins/GATC) and at protein level by Western blot.

### Statistics

Statistical analyses were performed in R and GraphPad Prism v9.1. Survival analyses were performed using Kaplan-Meier plots along with log-rank test using R package survival. Box-and-whisker graphs indicate the median and the 25th and 75th percentiles, with minimum and maximum values at the extremes of the whiskers. Associations between categorical variables were analyzed using the Fisher’s exact test, whereas such associations between numerical and categorical variables were explored using Mann-Whitney-Wilcoxon test, 2-tailed Student’s *t* test, and 2-way ANOVA. *P* < 0.05 was considered statistically significant. UMAP was used for dimensionality reduction.

### Study approval

This study was approved by the Regional Ethics Committee at Lund University (Dnr. 191/2007 and 101/2013). The sample cohort represents a population-based retrospective collection (*n* = 177) obtained at the Department of Surgery at Skåne University Hospital between 1997 and 2012. This study included 113 lymph node metastases, 35 subcutaneous metastases, 10 visceral metastases, and 15 primary tumors and 4 melanomas of unknown origin, as reported in detail in our previous study (60). We also used EPIC DNA methylation data that were generated in the Mitra et al. study (30). This cohort overlaps considerably with the Cabrita et al. (60) cohort. All patients were targeted therapy and immunotherapy naive, thus making the cohort suitable for prognostic studies. All patients gave written informed consent.

The in vivo studies were performed using male C57BL/6 mice (The Jackson Laboratory) 7–9 weeks of age, bred at the Lund facility, and were housed together with ad libitum access to food and water and fixed 12-hours-per-day light cycle, and the cages were enriched by a cardboard tunnel and nesting materials. All experiments were approved by the Malmö/Lund Ethical Committee for Experimental Animals, Lund, Sweden (Permit number M47-15 and 12548/19) and performed according to international guidelines for the use of research animals.

## Author contributions

AS was responsible for conducting experiments, acquiring data, analyzing data, and writing the manuscript. BP was responsible for conducting experiments, acquiring data, analyzing data, and writing the manuscript. SM was responsible for analyzing data. ML was responsible for analyzing data. JC was responsible for analyzing data. TZ was responsible for analyzing data. CN was responsible for acquiring data. EC was responsible for acquiring data. KH was responsible for analyzing data. FR was responsible for acquiring data. RC was responsible for acquiring data. IJ was responsible for analyzing data. KI was responsible for acquiring data. CI was responsible for acquiring data. AC was responsible for acquiring data. KB was responsible for analyzing data. HT was responsible for analyzing data. MA was responsible for analyzing and acquiring data. KP was responsible for designing research studies and analyzing data. GJ was responsible for designing research studies, analyzing data, and writing the manuscript.

## Supplementary Material

Supplemental data

## Version 1 08/30/2022

In-Press Preview

## Version 2 10/10/2022

Electronic publication
