# Supplementary material for: DNA promoter hypermethylation of melanocyte lineage genes determines melanoma phenotype
Source: JCI Insight. 2022 Oct 10;7(19):e156577. doi: 10.1172/jci.insight.156577 (PMC9675437; doi:10.1172/jci.insight.156577)
Supplement: Supplemental data [file jciinsight-7-156577-s089.pdf]

Supplementary Figures

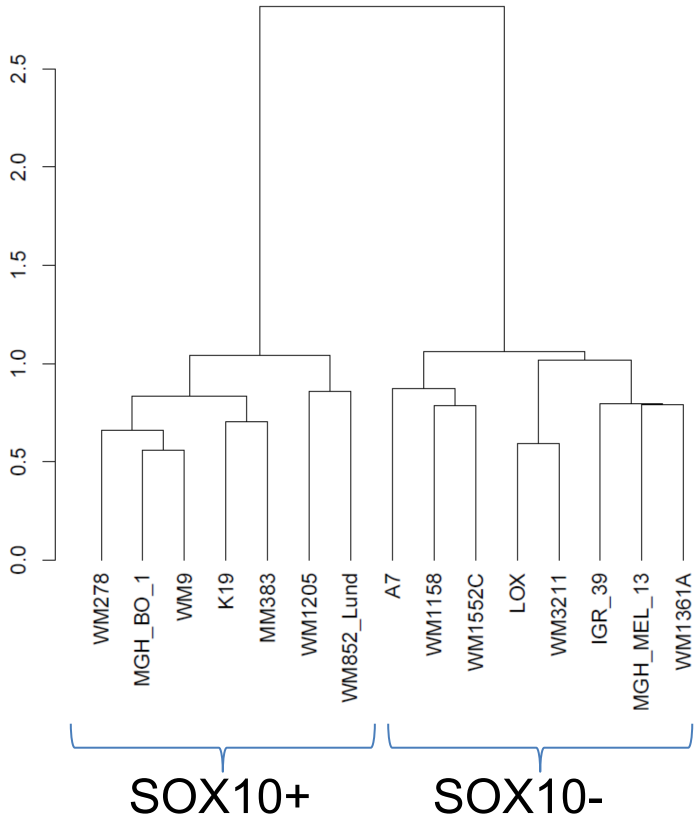

Supplementary Figure 1. Consensus clustering of 15 MITF methylated melanomas using 1,500 genes with largest variation across the cell lines.

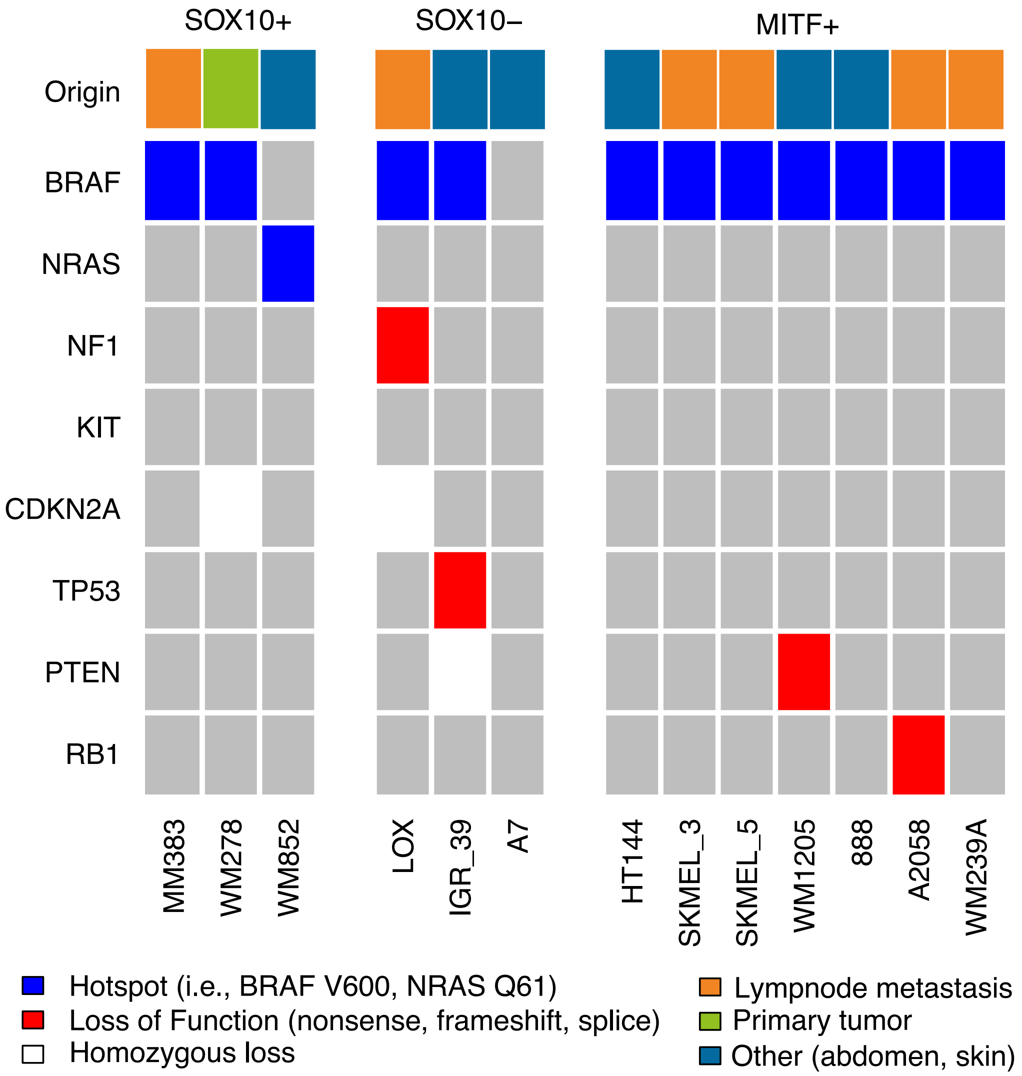

Supplementary Figure 2. Characteristics of included melanoma cell lines divided by MITF and SOX10 expression. Mutations in melanoma driver genes are described and determined as previously described [1].

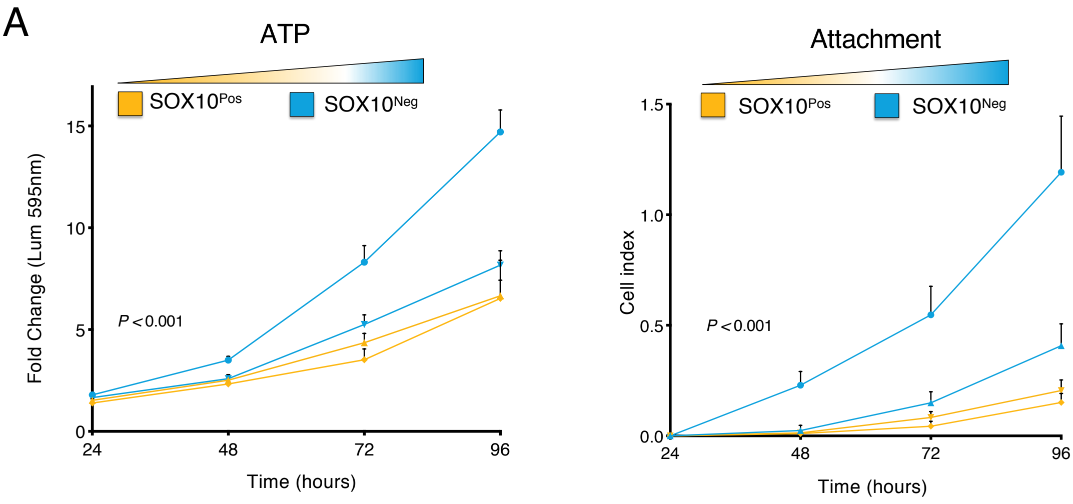

Supplementary Figure 3. Phenotypic characterization of MITF^{Met} SOX10^{+} (yellow) and SOX10^{-} melanoma cell lines (blue). **A**. Cell proliferation assessed by cell ATP content for 96 hours shows that the MITF^{Met}SOX10^{-} subgroup has significantly higher proliferation rate than the MITF^{Met}SOX10^{+} cell lines ($P < 0.0001$). **B**. Cell proliferation assessed by cell attachment to the surface of a culture plate for 96 hours the MITF^{Met}SOX10^{-} subgroup has significantly higher proliferation rate than the MITF^{Met}SOX10^{+} cell lines ($P < 0.001$).

References

1. Sanna, A., et al., *Tumor genetic heterogeneity analysis of chronic sun-damaged melanoma.* Pigment Cell Melanoma Res, 2020. **33**(3): p. 480-489.

Table S1. Normalized log2 transformed RNA sequencing data.

	1300	624	888	A2058	A375	C32	COLO783	
COLO858	DM13	DM6	G_361	HS944T	IGR_39	IPC_298	K19	K8
	LOX	MALME3M	MEL_H0	MEL_JUSO	MEW0	MGH_BA_1	MGH_BO_1	
MGH_CH_1	MGH_MC_1	MGH_MEL_13		MGH_QU_1	MGH_SW_1	MM455	NAE	
RPMI_7951		SK_MEL_2	SK_MEL_28		SK_MEL_39		SKMEL_31	
SKMEL_5	TC1308	TC2823_1	TC3018_1A		UACC_62	UACC903	WM1158	
WM1205	WM1341D	WM1361A	WM1552C	WM209	WM239A	WM278	WM3211	
WM853_2	WM9	WM902B	526	A7	ANBU	C918	CHL1_Lund	
	Gmel	HMCB	HT144	IGR_1	K4	M14	MEL202	
MEL205	MEL270	MGH_PO_1	MGH_ST_1	MM383	RVH421	Skmel_119		
SKMEL_3_Lund		Skmel_37	Skmel_63	SKMEL1	SKMEL30	TC2139	UACC257	
WM35	WM164	WM2067	WM373	WM608	WM852_Lund		WM983B	
HEM_adult		HEM_light		HEM_medium				
A1BG	1.9531	1.8568	0.9023	1.2109	1.886	1.6018	1.3693	
2.0496	1.5581	1.2109	2.135	1.2439	0.649	1.6665	2.142	
1.3771	0.2971	1.7621	1.4395	2.0841	2.2622	1.6954	2.2835	
0.9854	1.1217	1.4911	2.8783	1.9473	1.8648	1.5871	1.9143	
2.5219	2.0046	2.4605	1.7354	1.0161	1.3078	2.4027	2.4409	
1.2338	2.4136	1.4395	1.5768	1.2681	0.8642	1.3043	0.6236	
1.0361	2.1337	1.3455	1.5984	1.5455	1.1279	2.2717	1.6721	
1.8583	0	1.8094	1.9817	1.9754	2.1656	2.1808	1.1975	
1.3026	2.2876	0.6752	2.5657	2.3407	1.8133	2.0908	2.4926	
1.9678	2.1668	1.7022	0.7756	2.5506	2.0725	2.0517	1.6935	
2.5411	1.8637	1.7564	1.7807	2.7467	2.3315	2.1021	1.9995	
2.5028	1.8386							
A1CF	0	0	0	0.0214	0.0028	0	0.0315	
0.0055	0.0052	0	0.0015	0	0.0381	0.0232	0	
0.0022	0.0874	0.0177	0	0.0215	0.0255	9.00E-04	0.0013	0
	0.0025	0.0056	0.0118	0	0.0087	0.0094	0.005	
0.0015	0	0	0.0216	0.0048	0.0108	0	0.0051	
0.0102	0.0059	4.00E-04	0	0.0107	0.003	0	0	0
	0	0.0373	0	0.0284	0	0	0	0
	0	0.016	0.0102	0	0	0.0054	0.02	
0.0152	0	0.0078	0.0219	0.0064	0.0048	0.0322	0.0054	
0.0086	0.0224	0	0	0	0.0031	0	0.0029	0
	0	0.0055	0	0	0	0	0	
0.0201	0							
A2M	5.2226	4.1866	1.203	2.8043	1.041	7.5271	2.6967	

0.6448	4.8724	3.6434	5.0053	4.7575	0.0545	2.7738	7.7702	
5.3328	0.191	7.6149	1.5484	2.5834	1.0488	6.4962	6.9012	
0.39	7.4605	0	5.8914	4.3362	6.2992	5.6625	0.0155	
5.7532	6.3436	4.91	8.7392	5.1649	5.2774	8.8568	8.5174	
4.7553	5.4678	0.1464	3.8281	5.1516	0.8358	0.239	5.4748	
8.7651	1.956	0.1865	5.2233	5.7949	3.7648	1.0651	0.3672	
2.5747	1.0312	0.2576	7.7283	0.3236	4.4156	4.5912	6.0732	
4.6678	8.6418	0.2469	5.5653	5.2521	2.4927	1.1412	1.0746	
7.641	1.7408	6.6519	2.1396	7.3075	6.4517	6.0404	10.3687	
6.4345	6.76	4.4999	8.2123	4.777	4.9126	2.5133	8.3913	
8.2904	8.7392							
A2ML1	0.0334	0.0272	0.0434	0.0502	0.0064	0	0.0423	0
	0.0868	0.0303	0.0471	0	0.04	0.0449	0.0055	
0.3598	0.0292	0.178	0.1352	0.0267	0.0987	0.0161	0.0076	
0.0395	0	0.008	0.0154	0.0491	0.0125	0.0531	0	
0.0024	0.016	0.0347	0.0046	0.0167	0.0166	0.0357	0.0283	
0.0205	0.0284	0	0.0348	0.0388	0	0	0.0344	0
	0.0434	0.1206	0.0304	0.015	0.0669	0.0124	0.0227	
0.0689	0.0363	0.0939	0.03	0	0.0302	0.0166	0	0
	0.0543	0	0.0277	0.0347	0.0455	0	0.0366	
0.0062	0	0.0037	0.0076	0	0.0913	0.0237	0.0317	
0.0806	0.2857	0.0547	0.0439	0.0693	0.0238	0.1195	0.0358	
0.1141	0.0616							
A3GALT2	0.2968	0	0	0.3091	0.1172	0	0.0813	0
	0	0	0	0	0	0	0.1542	0
	0.4799	0	0.257	0	0	0.1147	0.0388	
0.0958	0.3513	0	0	0	0.0757	0	0	
0.0279	0	0	0.096	0	0.0551	0	0	0
	0.122	0.1895	0.1249	0	0	0	0	
0.1052	0.0659	0	0.0837	0.1891	0.1811	0	0	0
	0	0	0	0	0	0	0.0934	0
	0	0.2631	0	0.1185	0	0	0.1025	
0.1046	0	0.0722	0	0	0.1745	0	0.2055	0
	0	0	0	0.1103	0.1571	0	0	0
	0.2905							
A4GALT	2.1657	0.4357	0	0.1506	3.0265	0	0.2958	
1.6341	0.2643	1.725	1.2154	2.3878	1.3405	0.5012	0.5141	
0.0786	0	0.6617	0.1915	0.4972	1.1653	0.5528	0.3086	
0.5698	2.5234	0.3105	3.1432	0.1318	0.3516	0.8342	1.8858	
4.0661	0.3756	0.4442	0	0.7073	0.9427	0.4361	0.6305	
0.4932	0.2216	0.8407	1.6854	0.8911	0.652	0.5824	0	0
	0.6413	0	0.4345	1.7485	0.3943	0.424	1.3221	
0.2786	1.7674	3.4013	2.6214	2.7086	1.0486	0.3839	1.6808	
0.4756	0	1.9854	1.0045	0.436	0.0568	0.374	0.623	
0.2887	0.1742	1.3925	0.225	2.8714	0	0	0.0247	
0.3839	0	0.8472	0.3797	1.767	2.4369	0.2363	0.8662	
0.3291	0.2124							
A4GNT	0.05	0	0.0474	0	0	0	0.0102	0
	0.0586	0.0945	0	0.004	0.0326	0.0439	0	0
	0.1652	0	0.0606	0	0.1315	0	0.0743	

0.1165	0	0.013	0	0	0.0632	0.0208	0.0353	
0.0108	0	0.2849	0.0309	0.0513	0.0465	0.0327	0	
0.1265	0.0167	0.0391	0.0977	0.0555	0.0493	0.0194	0.0667	
0.0589	0.0181	0.2073	0.0428	0	0.0511	0.0512	0	0
	0	0	0	0	0	0	0	
0.3257	0	0.1201	0	0	0	0	0.0626	
0.0741	0	0.0125	0	0	0	0	0.0903	0
	0.0682	0.0271	0.0316	0.0708	0	0.0735	0	
0.0388	0.045							
AAAS	4.7989	5.1526	4.5029	4.1542	4.965	4.4212	3.6679	
4.3636	5.057	4.8516	4.5856	3.9496	3.7672	4.4344	4.013	
4.5348	5.009	4.4323	4.7428	4.7439	4.4921	5.115	4.4357	
4.784	4.5752	4.2119	4.1973	4.89	4.647	4.6081	5.0564	
4.5935	4.7377	4.6514	4.7416	4.2563	5.0919	4.5999	4.407	
4.6577	4.7678	4.8527	3.9014	5.0164	4.4304	4.4655	4.481	
4.4585	4.4623	5.3535	4.2173	4.266	4.567	4.4023	4.3791	
4.3788	4.7975	4.933	4.5057	4.7142	4.7441	4.5765	5.3721	
5.0525	4.5541	4.1929	4.5314	3.8921	4.3316	4.0837	4.5061	
4.5027	4.3927	4.5983	4.238	3.9455	4.4076	3.9297	4.6762	
4.8499	5.2607	4.2412	4.01	4.0301	4.6674	4.614	4.349	
4.7899	4.9323							
AACS	3.4548	3.6773	3.9378	2.995	4.1632	4.1705	3.8553	
3.0021	3.6822	3.5881	4.1042	2.7101	2.269	3.2367	3.3861	
3.1037	2.8087	3.9033	2.714	3.3455	3.373	2.9332	3.486	
3.8952	3.3959	2.7929	3.7666	3.1736	3.5572	3.7719	4.1374	
3.6323	3.2923	3.9799	3.5596	3.1879	2.7268	3.7846	2.6116	
3.5556	4.0943	3.9181	3.6851	3.7854	3.0464	3.438	3.5877	3.3
	2.9257	3.6389	3.5473	2.7903	3.8608	2.8483	3.9671	
3.6552	3.1539	2.7643	4.5014	2.8509	3.5381	4.1278	3.2129	
3.7641	3.7267	3.2234	3.313	3.4898	2.9913	3.7413	3.7945	
2.8946	3.648	3.3435	3.7776	5.066	3.7299	2.6965	2.7923	
3.6835	3.6537	4.1954	4.0283	3.0863	2.4744	3.7011	4.8166	
4.1654	4.5252							
AADAC	0	0	0	0	0.0289	0	0.0364	0
	0	0	0	0.1244	0.1605	0	0.1879	0
	0	0	0	0	0	0	0	0
	0	1.123	0	0	0	0	1.6339	
0.1411	0	0	0.0928	0.1587	0.0327	0	0	
0.0448	0	0.0221	0	0	0.1173	0.319	0	0
	0.4304	0	0	0	0	0	0.4442	0
	2.255	0	0	1.1847	0	0	0	0
	0	0.013	0	0	0	0	0.0118	0
	0	0.0067	0	0	0	0	0	0
	0	0	0	0	0.0835	0	0	0
	0							
AADACL2	0	0	0	0	0	0	0	0
	0	0	0	0	0	0	0	0
	0	0	0	0	0	0	0	0
	0	0	0	0	0	0	0	0
	0	0	0	0	0	0	0	0

	0	0	0	0	0	0	0	0
	0	0	0	0	0.0545	0	0	0
	0	0	0	0	0	0	0	0
	0	0	0	0	0	0	0	0
	0	0	0	0	0	0	0	0
	0	0	0	0	0	0	0	0
	0							
AADACL3	0	0	0	0	0	0	0	0
	0	0	0	0	0	0	0	0
	0	0	0	0	0	0	0	0
	0	0	0.0206	0	0	0	0	0
	0.021	0	0	0	0.004	0	0	0
	0	0	0	0	0	0	0	0
	0	0	0	0	0	0	0	0
	0	0	0	0	0	0	0	0
	0	0	0	0	0	0.025	0	0
	0	0	0	0	0	0	0	0
	0	0	0	0	0.0252	0	0	0
	0							
AADACL4	0	0	0	0	0	0	0	0
	0	0	0	0	0	0	0	0
	0	0	0	0	0	0	0	
0.0212	0	0	0	0	0	0	0	0
	0	0	0.0085	0	0	0	0	0
	0	0.0301	0	0	0	0	0	0
	0	0	0	0	0	0	0	0
	0	0	0	0	0	0	0	0
	0	0	0	0	0	0	0	0
	0	0	0	0	0	0.0329	0	0
	0	0	0	0	0	0	0	0
	0							
AADAT	2.6688	2.02	2.548	3.5322	3.5642	3.9523	1.4348	
2.4392	1.9221	2.4709	1.8232	2.2231	1.4456	3.2588	2.021	
2.573	2.3468	2.0406	2.496	2.8294	2.402	3.0116	1.6544	
4.2364	4.1386	2.1381	2.7202	2.4922	1.9285	1.4415	1.7758	
2.2039	2.2505	1.5944	2.3106	2.8543	2.0242	1.5779	1.9348	
2.63	2.4098	1.4221	2.4467	3.7813	1.5814	2.1458	3.1816	
1.9384	2.2054	2.6228	2.3598	3.1664	2.8101	2.5753	2.3548	
2.457	1.3315	1.2421	2.2547	2.0164	2.4613	2.6944	2.7126	
2.5517	2.857	1.9922	2.1463	3.5278	2.1003	3.2699	2.9298	
2.3642	3.8287	2.3908	4.1376	2.5197	2.6388	2.3282	1.3405	
2.6268	1.9141	2.0545	2.6843	2.3586	2.0563	2.7695	1.7839	
2.9188	2.3331							
AAED1	3.7097	3.3974	3.7031	3.3653	2.9428	4.1617	3.941	
2.6633	2.856	3.4042	3.7404	3.866	4.9357	3.8299	3.6494	
3.0026	4.2917	4.2211	3.5576	3.5681	2.9436	2.4012	3.0471	
3.5077	2.9036	3.0504	4.9037	3.8966	4.2981	3.0944	3.1419	
2.8988	3.6711	3.9255	3.4827	4.1111	3.3087	3.1232	3.5234	
3.7729	3.448	3.6002	3.6852	4.1738	3.8077	3.631	3.9847	
2.4251	3.7429	3.6743	3.9309	3.834	3.8706	2.3161	3.8447	

3.1667	4.433	3.6647	4.6001	3.6215	4.1652	3.3269	2.5894
4.2646	4.7812	3.1499	3.2676	4.3181	3.9236	3.8957	3.2234
3.469	3.202	2.8118	3.4301	3.0199	3.6315	2.9118	2.6565
3.713	3.1996	3.6788	4.1339	3.9687	3.2948	4.0206	4.2528
3.819	4.1909						
AAGAB	4.8971	5.1908	4.5024	4.6752	4.1111	4.6581	4.9506
4.2775	4.4264	4.071	4.2055	4.1538	4.4918	4.543	3.8014
4.7691	4.6261	5.0151	4.474	4.1686	4.2729	4.5049	4.5015
4.5069	4.7267	4.4813	3.7254	4.5707	4.3694	4.7565	4.8984
4.2374	3.8092	3.8244	4.2285	4.7571	4.8359	4.5918	4.8512
4.4099	4.4078	4.8336	4.1287	4.3836	5.1493	4.9601	5.0916
4.9595	4.0944	4.7904	4.5143	4.2735	4.3686	5.2507	5.0027
4.5202	4.1626	4.0675	4.3315	4.2599	4.6087	4.5743	4.5505
4.5392	3.9504	4.4814	4.1798	4.5419	5.3369	4.2747	4.158
3.9662	4.09	4.4755	4.5318	4.4394	4.5467	4.7414	4.1415
4.7399	4.0561	4.1538	4.4553	3.7665	5.1717	3.9217	4.163
4.1003	3.923						
AAK1	2.8465	2.3828	3.267	2.8444	2.8147	3.3331	3.1819
2.9084	3.9951	3.1877	3.0278	3.1382	3.3093	3.0815	3.3901
2.9849	3.1987	2.9695	3.0049	3.0671	3.4044	2.7295	2.7563
3.1948	3.0492	3.2887	2.9944	2.3679	2.7678	2.7989	2.4944
2.7034	3.3306	3.4292	3.4416	3.1041	3.5693	2.9924	2.8457
3.4041	2.6996	2.9645	3.8497	2.3541	3.4135	3.2312	3.3434
3.1815	2.5497	2.6204	3.1339	2.5012	2.9382	2.8327	2.8094
2.3897	2.8119	2.978	3.3958	2.9399	2.7286	3.2614	2.6358
2.9523	3.2743	3.4771	3.6881	3.6152	3.4464	2.8364	2.7473
3.0469	2.7599	3.3624	2.9679	3.5132	3.0674	3.8195	2.3394
2.5686	2.8098	2.9358	3.2468	3.4405	2.4577	2.5517	2.5745
2.2854	2.3659						
AAMDC	3.7879	3.8974	4.0637	3.1848	2.9915	3.4977	4.0867
2.6126	4.5256	3.7642	4.395	3.5864	4.2009	4.961	4.114
2.569	4.5031	3.4735	2.9275	4.2972	5.2382	3.452	3.8026
3.2543	4.06	4.2911	4.316	4.1645	4.3636	4.32	4.4828
2.6003	4.0162	4.5205	4.6693	3.7082	3.8596	3.6013	3.8423
3.606	4.1498	4.4421	4.4847	3.9636	3.351	4.7849	3.7998
3.3082	3.9688	4.9134	4.3877	3.1351	3.7364	3.9813	4.6085
3.8656	4.1303	4.3623	2.7618	3.607	3.2514	2.4338	2.4266
3.7121	4.0483	4.4459	4.184	5.1316	4.0239	3.652	4.8071
4.0325	4.1476	2.9334	3.508	3.7259	3.9053	4.1259	4.4678
4.4064	4.519	4.3347	5.9558	3.9994	4.9437	4.0194	5.6574
5.2823	5.5932						
AAMP	5.8465	5.8009	5.7527	4.8176	5.7856	6.3975	5.891
6.5234	5.1434	5.4758	5.7518	5.6717	5.5641	5.1269	6.0925
5.9484	5.8179	5.2435	5.1593	6.0466	5.9346	5.8802	6.0869
5.7972	5.3506	6.1612	4.5544	5.5278	5.7866	6.2906	5.561
6.1396	6.2628	5.6276	5.3744	5.1201	6.2215	6.2016	5.386
5.4002	5.912	5.7483	5.4891	4.8357	5.5084	5.3474	4.7349
5.8995	5.9719	5.1629	5.4357	5.0856	5.7077	6.3157	5.83
4.904	5.2207	6.3236	5.3447	6.0752	5.5722	5.9221	4.8699
5.2918	6.0681	5.2934	5.3464	5.6049	5.7565	5.3968	5.4568

5.4285	5.8621	5.491	5.4097	6.0752	6.0372	5.9029	5.6731	
5.8817	6.061	5.2841	5.2883	4.8352	6.0237	6.1907	5.2006	
5.4215	5.3305							
AANAT	0	0.0419	0	0.2378	0.0303	0.206	0	0
	0.139	0	0.2869	0.0628	0.0792	0	0.2275	
0.4184	0.6304	0.2054	0	0.3634	0.0976	0.1651	0.0309	0
	0.0211	0.0493	0	0	0	0	0.1783	
0.1891	0.1922	0	0.076	0	0.1468	0.1089	0	
0.1434	0.2403	0.0663	0.2235	0	0.0252	0.3844	0	
0.1304	0	0.3257	0.4095	0.0412	0	0	0.3139	
0.3359	0.0764	0	0.3326	0.0613	0	0.0546	0.1142	
0.4713	0.097	0.0209	0	0.026	0.0343	0.1294	0	
0.1317	0.0182	0.0636	0.106	0.016	0.3938	0.0163	0.1643	
0.2744	0.0571	0	0.0434	0.1875	0	0	0	0
	0							
AAR2	4.4851	4.768	4.596	4.0368	4.7965	3.9693	5.1136	
4.3021	4.2888	3.5484	4.3431	3.9566	4.3518	4.2415	4.3463	
4.836	4.4579	4.0592	3.9722	4.2511	3.7272	4.5309	4.5636	
4.1539	4.7877	4.859	3.2804	4.5108	4.7664	3.9262	3.5738	
4.5055	4.7959	4.1279	4.4172	3.6274	4.2354	4.8241	4.8382	
4.5245	4.052	4.0953	3.4178	4.577	4.8449	3.4578	4.1667	
4.4274	4.8123	4.903	4.3823	4.2788	4.437	4.2498	4.24	
4.8627	3.6466	4.4498	4.3669	4.5368	4.1334	4.4867	4.258	
4.185	3.7878	3.6572	3.6326	3.7162	4.2033	4.4762	4.7915	
4.2553	5.1809	4.1794	5.3753	4.0474	4.286	4.7985	3.834	
3.9414	4.092	4.6755	4.133	4.4018	4.2126	4.0641	4.2542	
4.2476	4.313							
AARD	0.0654	0.7247	0.295	0.0543	1.3598	0.4519	1.8216	
0.3591	0.2519	0.908	0.9438	0.2111	0.8624	0.9545	1.215	
0.0737	0.3522	0.2367	0.2519	1.1606	0.3987	0.8099	0.7409	
0.3261	1.6287	0.7754	0.1993	0.461	1.6554	0.313	1.1836	
2.0143	2.2568	1.1281	0.2696	0.5907	0.582	0.6389	0.8698	
0.8774	0.0565	0.0545	2.2292	1.3706	0.0456	0.1167	0.5023	
1.0413	0.3309	0.2321	0.2782	2.0732	0.8256	0.2495	0	
1.3893	1.0602	0.0299	0.9446	0.2357	2.1949	0.1121	0.0501	
0.5013	0.8403	0.3651	0	0.1977	0.8081	1.434	1.0018	
1.299	1.2273	0.8924	0.6587	0.2027	0.2073	1.0081	0.2257	
0.1539	0.8449	0.6285	0.2195	0.778	2.1276	0.5546	0.2251	
0.6187	0.2912							
AARS	6.6349	5.7488	5.8307	5.6656	6.0832	5.6559	5.6441	
5.7185	5.8268	7.2758	6.255	5.5183	7.4043	6.3038	5.545	
5.9038	6.8207	5.7405	5.4501	6.4925	6.0987	6.6525	5.3632	
5.3992	6.416	6.2575	5.266	5.6484	5.1162	5.5458	6.8847	
5.5058	5.5082	5.5998	6.294	5.7488	7.6554	7.2825	6.9216	
6.0262	6.5528	5.7309	6.2493	7.1985	6.2926	5.7131	5.6211	
6.7958	5.8997	6.0367	5.4495	5.8662	6.1966	6.7122	6.2788	
6.1342	8.2322	5.0295	6.4459	5.2318	6.1342	6.3492	6.0533	
5.0084	6.4569	6.0351	6.1358	5.7663	6.0146	5.7818	6.8155	
6.0121	5.244	5.4855	5.9626	6.4853	5.5051	6.0968	6.0279	
5.7498	6.2017	5.931	5.9685	6.3897	5.5462	6.3911	5.9027	

5.9673	5.906						
AARS2	2.4685	3.7504	3.4481	2.0332	2.8184	2.8824	2.3945
3.8015	2.842	3.0674	3.332	2.8371	1.8605	3.2098	2.6599
2.939	3.2452	3.1635	3.3013	2.9426	2.8198	3.4014	2.8783
2.3519	2.8259	3.308	2.2265	3.3506	3.181	2.7773	2.93
3.0728	3.2297	3.0572	3.0953	2.7275	3.4714	3.6547	3.5757
3.6671	2.5627	1.7507	2.5761	3.9994	2.2636	3.0365	2.1968
2.6552	2.9896	2.2472	2.6948	2.6285	2.9364	3.2006	2.4871
4.0111	2.0656	2.7198	2.8031	2.9051	3.6564	2.9577	3.8755
3.093	2.992	1.9031	3.6947	2.559	2.563	2.9758	3.0409
3.2336	2.8963	3.2397	2.8394	4.33	3.4405	3.3888	3.0615
3.1005	2.8965	3.0576	3.475	3.5072	3.2519	3.0331	2.4489
2.8812	2.4916						
AARSD1	4.4324	4.1725	3.4023	4.1845	4.0037	4.0476	2.5734
4.3221	3.7797	4.0283	4.0144	3.7476	4.1493	4.2249	3.696
3.871	4.6172	3.6593	3.934	4.4013	3.8972	3.5953	3.126
3.9975	4.0929	3.7797	4.2986	4.3234	3.8755	3.6761	3.2983
3.8337	3.7472	3.9476	4.0377	3.5053	4.1416	4.1858	3.989
4.2192	3.4887	3.3002	3.8809	4.274	3.324	3.9452	4.074
4.138	3.5841	3.8192	4.1881	3.8158	3.6913	4.029	3.5164
3.8684	3.6463	3.9076	4.2523	3.6814	4.2732	4.552	3.6257
3.4249	3.8256	3.4854	3.5148	4.0512	3.6004	4.4452	4.0034
3.7328	3.5408	4.11	4.4747	4.2816	3.6945	3.9897	3.778
3.7148	3.412	3.3183	3.5258	3.8478	4.6088	3.9065	3.4813
3.8052	3.7209						
AASDH	2.6282	2.062	1.7157	2.3849	2.5565	2.1521	2.1455
1.7921	2.2353	2.2413	1.9701	2.6167	1.4549	1.9803	1.5827
1.478	2.1945	2.7866	2.1319	1.925	1.9667	2.0695	1.3325
1.8334	1.5889	2.3599	2.1168	2.3724	1.569	2.1157	1.7068
2.0615	1.7944	1.9498	2.202	2.1819	2.5401	2.4139	2.3821
1.9316	2.5608	2.3301	2.1874	2.4315	2.3632	2.4951	2.5577
1.9646	2.2726	2.9368	2.1411	2.9	2.5955	2.4378	1.5575
1.979	2.8803	1.5854	1.8359	1.4445	2.1337	2.39	1.9522
1.9222	2.2997	2.1894	2.4355	2.0691	1.8205	1.8444	2.3132
1.9604	2.122	2.0052	2.1746	1.9042	1.8617	2.2567	1.7263
2.8158	2.3046	2.3552	1.4654	2.7647	1.3906	2.2359	2.4905
2.2427	2.3714						
AASDHPPT	4.6522	4.0153	4.3273	4.8523	4.7568	5.0734	4.8053
4.1594	4.7047	4.0135	4.2389	3.1455	4.764	4.7362	3.9857
4.9732	5.1217	4.2397	4.1785	4.82	3.9332	4.8348	4.615
3.6942	3.859	4.7356	3.3916	3.5217	5.1422	4.5227	4.7856
3.8254	4.7237	3.5019	2.9192	4.178	4.7334	4.3744	5.0351
5.4616	3.3388	4.3481	3.7242	4.6638	4.5954	4.9523	3.9988
4.8862	4.4331	5.0095	4.7568	4.5728	4.6843	5.4492	6.2873
4.4675	4.4498	4.1185	4.4193	3.9544	4.8442	4.3483	5.0265
4.488	4.5722	4.3195	4.2113	4.3877	3.8763	4.856	3.9588
4.2799	5.0656	4.4761	4.6405	4.5209	3.8212	3.7712	5.1203
4.3904	4.309	4.1578	3.0208	4.4161	4.2803	4.2725	4.2505
4.6928	4.4572						
AASS	2.472	3.4038	2.8462	2.0889	2.0823	2.6653	1.5007

2.3255	2.4039	2.722	3.0745	2.0877	1.0607	2.3555	2.2201	
3.2261	2.5495	3.1651	3.1821	3.2139	2.3122	2.3741	1.9163	
2.1687	1.3984	2.0268	1.9342	2.607	2.4016	2.9576	0	
2.3811	2.3386	2.2687	2.5527	2.4082	1.2444	2.7238	2.6874	
2.733	2.5579	1.8421	1.9151	2.4076	2.0574	2.6235	3.0239	
2.4082	2.7171	2.3161	2.2888	2.0023	2.119	1.3082	1.7047	
1.973	1.9526	3.0491	3.101	3.1914	2.2865	2.9491	2.3567	
2.8772	2.5348	2.1604	2.3196	1.7236	1.8666	1.993	2.0618	
2.116	3.6013	2.2667	2.9193	2.2281	2.3886	2.4283	2.8806	
2.4743	3.1014	2.4352	0.4049	2.4506	1.3954	1.8639	2.6301	
2.5362	3.0436							
AATF	5.3609	5.2646	4.9353	5.0392	5.4973	5.5983	4.8006	
4.6918	4.8645	5.1121	4.7477	4.7104	5.3781	5.3465	5.1765	
5.1224	5.9288	5.1921	5.7316	5.1667	4.1771	4.878	4.7847	
5.2928	4.9297	5.0057	5.3523	5.4211	5.64	5.1305	4.7568	
5.6428	5.6629	4.5665	5.0541	5.2412	5.0674	4.6557	4.4648	
5.7161	4.6901	4.5524	4.9414	4.7083	4.1265	5.5356	5.4422	
5.1981	5.1067	5.4674	5.1806	5.6258	4.4302	5.1688	4.9115	
5.643	3.978	5.2584	4.7777	5.4513	5.7092	5.2933	4.7564	
5.3634	4.8505	4.1133	4.475	5.0587	5.3063	5.7478	5.3747	
5.2473	5.4688	4.8013	5.0151	5.6305	5.294	5.0948	5.1303	
4.5309	4.2097	4.6806	5.6541	4.1493	5.8388	4.6635	4.3583	
4.2589	4.054							
AATK	3.5507	0.9277	0.8302	0.54	0.3332	0.207	0.208	
0.5325	1.187	0.7899	1.8496	0.5125	0.6971	0.6184	0.26	
1.5377	0.5934	2.2173	0.2014	1.0523	4.9263	1.2454	0.8934	
0.2726	1.0825	0.1863	1.6771	1.1777	0.673	0.667	0.7187	
2.1731	0.1826	0.7764	0.243	1.2049	3.1675	1.741	1.9931	
0.557	0.8479	0.365	0.3321	0.9568	1.3563	0	0.3813	
1.2512	0.2905	0.1953	1.1554	0.2692	1.999	1.1092	0.3826	
1.3847	0.1439	3.9946	1.0891	2.9482	1.1135	0.3519	3.0483	
0.0641	0.9918	0.0311	1.2049	0.4375	0.3719	2.5519	0.4315	
2.0388	1.655	1.6434	0.8427	0.9584	0.3588	0.7556	1.5811	
1.3619	0.9286	1.5734	0.2448	3.2477	0.1179	2.4587	1.3112	
1.6691	1.0892							
ABAT	0.9666	0.6642	0.6432	0.2698	2.0345	0.2691	1.2641	0
	1.3737	1.1218	1.2959	0.0021	0.534	0.148	1.2532	
0.5084	0.0457	0.5449	0.0077	0.0058	0.3636	0.5385	2.8755	
1.1023	1.9485	1.5895	0.1235	0.3803	1.2127	0.6754	2.2871	
0.6636	1.0114	2.2754	1.6921	0.5074	0.0487	1.98	1.3184	
0.9418	0.0689	0.5313	1.5005	0.0479	0.7446	0.0105	0	
0.5649	0.405	0.6975	0.2961	0.4386	0.3911	0.1628	0.6033	
0.1026	0.6211	1.4441	0.1568	1.8833	0.8264	0.5424	0.2193	
0.6538	0.4188	1.0035	1.7728	0.5648	0.3593	1.508	0.8252	
0.2105	0.4687	1.3309	1.4873	0.0784	0.265	2.1022	0.3098	
0.2411	0.6964	3.4853	0.0992	1.508	0.828	0.3398	2.0498	
1.6142	2.3254							
ABCA1	0.5017	0	0	0.3755	1.0217	2.0114	0	
3.7619	0.0092	1.4133	3.285	3.0873	4.9995	1.5523	4.2461	
1.104	4.0011	1.6696	0.3413	1.8125	1.5268	1.0046	1.4435	

0.4042	3.1367	3.2056	0.1491	0	1.9551	0.2385	0.8489	
0.2551	0.3836	3.2253	0	2.0986	2.341	0	4.1729	0
	0.692	2.5072	2.428	3.694	3.6382	2.2902	1.5098	
0.115	0.0262	4.5295	0.4418	3.1303	1.6944	2.1203	1.0476	0
	3.8819	0.7457	3.8241	0.6975	1.6851	0.4139	0.2573	
2.6516	0.5457	5.7196	0.2438	3.0971	0.229	1.2283	3.8157	
1.2011	0.2334	1.7528	0.3147	0.4756	0.717	0.5263	0.4101	
0.248	0.3206	3.9683	2.2798	3.7886	1.3625	1.3618	1.4772	
1.2008	0.7326							
ABCA10	0.2062	0.0632	0.0873	0.325	1.2601	0.3457	0.2472	
0.3895	0.215	0.2111	0.1207	0.1348	0.7602	0.7154	0.1107	
0.4413	0.0902	0.0613	0.2918	0.3045	0.3273	0.1685	0.3375	
0.2102	0.6139	1.3835	0.3079	0.3292	0.4793	0.1356	0.1812	
0.2542	0.2261	0.3406	0.5537	0.1266	0.3987	0.1681	0.2104	
0.688	0.172	0.1164	0.2467	0.3765	0.2486	0.8705	0.3243	
0.1106	0.6364	0.4651	0.2186	0.786	1.0258	0.3765	0.4043	
0.2127	0.2233	0.1813	0.1201	0.2485	0.477	0.0659	0.2458	
0.7261	0.3234	0.1602	0.0481	0.4203	0.4127	0.3295	0.3801	
0.3866	0.1385	0.0828	0.3848	0.1955	0.2076	0.2171	0.2104	
0.1022	0.1602	0.3128	0.277	0.8879	0.2697	1.0724	0.2687	
0.2109	0.286							
ABCA12	0.0236	0.0156	0.036	0.1186	0.1067	0.2235	0.376	
0.0608	0.0244	0.0163	0.0051	0.0399	0.0557	0.0355	0.0456	
0.1654	0.1154	0.038	0.0544	0.0733	0.0291	0	0.2123	
0.0688	0.0341	0.0798	0.0192	0.032	0.2969	0.0148	0.0286	
0.0111	0.3766	0	0.3452	0	0.0042	0.0164	0.0059	
0.1071	0.0146	0.0741	0.0235	0.0151	0.0215	1.1768	0.0836	
0.121	0.1136	0.0384	0.0128	0.0965	0.0262	0.0324	0.0069	
0.0419	0.0141	0.0083	0.0234	0.0041	0.012	0.0426	0.0181	
0.0144	0.0815	0.248	0.1285	0.042	0.0448	0.3144	0.2368	
0.0452	0.1629	0	0.0515	0.2157	0.0052	0.2866	0.2305	
0.0263	0	0.3089	0.1168	0.0368	0.0298	0	0.1402	
0.1324	0.0562							
ABCA13	0.1751	0.1964	0.3084	0.4728	0.4381	0.1311	0.8358	
0.2059	0.3523	0.201	0.1123	1.3074	0.0523	0.0789	0.4107	
0.2375	0.2394	0.8313	0.4954	0.3483	0.0982	0.0984	1.3627	
1.0156	0.0393	0.1099	0.1547	0.2564	0.6917	1.234	0.0301	
0.3727	0.1673	0.8774	2.2494	0.5373	0.1297	0.3334	0.2874	
1.0982	0.3717	0.0279	0.581	0.1622	0.1279	0.4285	1.4709	
0.3656	0.2106	0	1.3716	1.0133	1.3079	0.0336	0	
0.0396	0.4396	0.2007	1.2658	0.5428	0.4107	1.047	0.2225	
0.2899	0.2194	0.6982	0.0843	0.1679	0.3894	1.8055	0.0581	
0.0098	0.3506	1.2809	0.2949	0.2265	0.2031	0.6267	0.457	
0.5628	0.3724	0.1364	0.1763	0.6681	0.6082	0.9634	0.1958	
0.1039	0.2484							
ABCA2	3.2873	1.9424	2.9145	1.9654	3.468	2.9158	2.3229	
3.6774	1.0327	2.4352	3.987	2.5785	3.9376	1.512	1.1352	
2.4219	2.5252	1.6989	1.6678	3.3002	3.194	2.5738	2.6922	
2.1697	3.3276	1.4874	2.8754	2.5054	2.6977	2.4137	3.0817	
1.7586	2.2524	2.0204	2.5489	1.581	2.7073	4.19	3.6544	

1.7568	3.0359	2.7871	3.5172	2.8227	2.5217	1.4068	2.402	
2.839	3.9276	1.8689	2.8914	2.5031	1.9214	2.24	1.6632	
3.2176	2.7148	4.2437	2.4912	3.724	2.8252	1.4166	3.0706	
3.2291	3.0448	4.0517	2.7824	3.6783	2.7708	3.1492	3.1148	
2.3407	2.7246	3.3378	2.5135	3.6526	2.3943	3.6539	2.4031	
3.5641	2.081	3.3658	2.8058	4.3728	1.0904	2.2145	3.9821	
3.707	3.8701							
ABCA3	0.8962	0.3615	2.1519	0.48	0.9458	0.3136	1.5032	
2.6046	1.0334	0.23	0.1915	0.3994	1.7899	1.4414	2.3332	
2.0056	0.4361	2.0681	1.3214	4.0258	0.7319	0.1026	0.5557	
1.2692	0.6401	0.32	0.2268	1.0342	0.1307	0.1994	4.3876	
1.0441	0.2817	0.5437	0.2906	1.0647	2.2568	2.9852	2.5786	
0.1414	0.1559	2.1465	1.4391	1.9469	2.7375	0.0631	0.2043	
0.3325	0.228	0	1.0105	0.1073	0.2366	0.075	2.5588	
1.7291	1.1359	1.9798	0.3495	0.8171	0.0128	0.2158	2.0166	
0.773	2.8595	3.5853	3.0836	3.4268	1.1083	0.4695	0.5807	
0.2868	0.2991	0.2337	0.3843	0.1646	0.5829	2.097	1.606	
0.8643	0.1827	1.177	2.5907	0.2164	0.9322	0.3887	0.5076	
0.3744	0.1863							
ABCA4	0	0.1982	0.1074	0.0225	0	0	0.7522	0
	0	0.0064	0.0047	0.1	0.0054	0.0331	0.1532	
0.3208	0.0298	0	0.2428	0.0215	0.0253	0	0.0011	
0.1158	0.0143	0.0417	0.0152	0.325	0.0424	0.6714	0.0427	
0.2176	0.0709	0.0907	0.0184	0.0414	0.0327	0	0.0819	
0.0074	0.0476	0.0188	0	0	0.0084	0.112	0.145	
0.0715	0	0.1588	0	0	0.2281	0.1796	0.0151	
0.063	0	0	0	0	0.0109	0.0373	0.0125	
0.0246	0.0275	0.0497	0.028	0.4548	0.2325	0	0.0475	
0.0083	0.0337	0.017	0.0094	0.4555	0.6797	0	0.1027	
0.022	0.1607	0	0.0131	0	0.0255	0	0	0
	0.0257							
ABCA5	2.9554	1.2397	2.2939	1.62	0.8921	1.8119	2.6785	
1.3526	2.6639	2.6263	2.2638	2.0446	2.4885	3.9583	1.3303	
3.151	0	2.2556	2.4945	2.7545	3.3145	1.7477	1.5973	
2.4405	1.2322	2.9538	4.0241	3.2492	1.7523	2.4984	1.5747	
1.3019	3.1353	3.0529	2.1976	2.5389	2.2632	1.9765	1.5671	
2.702	2.4839	1.7785	1.2006	3.624	1.5846	2.1996	2.991	
2.7545	2.9964	0.8573	3.5576	3.1309	4.4341	2.4731	2.6268	
2.4046	1.229	0.6206	1.7911	0.6379	2.6522	1.3665	1.6143	
3.4242	2.0127	2.121	0	2.6377	2.5379	1.2123	2.6771	
2.6782	2.7979	1.6058	2.9413	3.5292	2.4262	1.9527	3.5529	
3.3999	3.3043	1.346	1.8627	3.4713	0.7766	3.7471	2.9089	
2.3235	2.7215							
ABCA6	0.0267	0	8.00E-04	0.0585	0.019	1.3754	2.1902	0
	0.0797	0	0	0.0149	0.9118	1.445	0.1517	
0.3896	0.1491	0	0	0.0284	0.0991	1.5864	1.0996	0
	4.0553	2.2948	2.7864	0.1057	1.9971	0	0	0
	0.3124	0.0445	1.4629	0	0	0	0	
0.7111	1.5062	0.0468	0	0.4686	0	0.3795	0	
0.5917	4.3322	0.0049	0.1691	2.198	1.2827	0.1995	0.1424	

0.2352	0.2268	0	0.518	0.149	0.7596	0	0.1151
0.6871	0.1245	0.2178	0.1765	0.1543	0.1182	4.003	0.2614
0.1689	0.2569	0.1715	0.1623	0.1808	0.1101	0.1177	0.18
0.2622	0	0.1924	0.4216	2.3899	2.3047	0.9189	0.8808
0.0635	0.044						
ABCA7	2.6574	1.1199	1.6903	1.0259	1.2875	2.0411	1.9039
1.6334	1.583	2.8099	1.2517	1.4954	1.2046	1.5436	1.0186
3.2896	2.2453	2.3721	1.2251	2.3159	2.0809	1.1606	2.3582
1.4534	0.9057	1.3503	0.3258	2.9031	1.5294	1.741	2.0915
2.0645	1.6996	2.1211	2.2294	1.7243	1.0284	2.217	2.806
1.4487	3.1257	3.6249	1.4294	1.6695	1.2014	1.6452	1.1855
1.8156	1.7903	0.8072	1.8461	1.6528	0.858	0.9886	1.8281
2.4083	2.8671	1.775	1.7774	1.1024	1.2612	0.8626	1.1805
2.2224	0.933	2.5324	2.6528	2.9304	2.2897	0.7797	1.7968
2.2011	1.1628	1.7634	1.5356	2.9099	2.0395	1.7708	2.0853
1.2866	1.6545	3.0138	2.3591	1.4929	1.8286	1.4468	2.2563
2.1096	2.6635						
ABCA8	1.9383	0.6338	0.1292	1.0988	0.2759	0.0679	0.0717
0.0825	0.0039	0.0226	0.2383	0.0104	0.048	0.0946	2.1582
0.0601	1.3431	0.1219	0	0.1626	0.6118	0.8807	0.9836
0.065	3.0009	2.1804	3.9335	0.1065	0.0961	0.0556	0.1552
0.1285	0.1155	0.1263	2.4296	1.1483	1.7127	3.4962	0.165
3.3575	2.3486	0.0644	0.0458	0.0072	0.0288	0.0572	1.2619
0.053	3.1037	0.075	0.1191	4.773	0.1252	0.5075	0.1551
1.8517	0.1588	1.9764	0.1993	0.8222	1.0825	0.1254	3.9745
0.0769	4.2241	0.2565	0.2399	0.0633	0.0604	0.2319	0.122
0.1717	1.2896	0.5808	0.0295	0.0652	0.2511	3.6264	0.1764
0.1774	0.0243	3.0715	0.3006	0.536	1.1714	0.0888	0.0661
0.737	0.2559						
ABCA9	0.0878	0.153	0	0.0449	0	0.5985	1.8764
0.0578	0.4273	0.0869	0	0.0051	0.0887	0.2626	0.0577
0.1248	0.1956	0.2547	0	0.1167	0.1053	0.7299	0.4877
0.0119	0.2325	0.9814	0.7219	0.0708	0.3311	0.0557	0
0.0345	0.12	0.4302	1.5291	0.2269	0.0214	1.052	1.1447
1.0235	0.2651	0.0978	0.0352	0.4167	0.0038	0.0368	0.0167
0.1995	3.2657	0.1866	0.0457	3.0929	3.2174	0.335	0
0.1023	0.0849	0.0478	0.0315	0.0734	0.3516	0.0721	0.8852
0.1571	0.8289	0.1295	0.114	0.354	0.0637	2.5929	0.0431
0.186	1.237	0.0601	0.0703	0.0615	0.0759	0.1518	0.1131
0.3079	0.0754	0.0553	0.1799	2.5859	0.2323	2.5975	0.3028
0.1111	0.0435						
ABCB1	1.566	1.3697	1.6818	0	1.7714	0.2071	0.7984
0.6652	0.0118	2.1172	2.125	0.2844	0	1.097	2.0626
0.0521	0.0453	0	0.1052	1.4135	0.3282	1.296	0.5088
0.1789	0.0809	0.0726	1.0984	3.136	0.9144	5.188	0.88
0.1238	0.0012	0	2.2597	1.7537	2.2265	1.421	1.2895
0.3852	0.007	0	1.0363	1.8866	1.7827	1.0831	1.3625
1.6513	0.5882	0.0245	0.0212	0.4596	0.1293	0.5708	1.0823
0.9342	0.5406	1.8291	2.7237	0.9942	1.7373	0.515	0.5477
1.0739	0.5281	0.1001	0.1194	0.8257	5.1995	0.592	0.4023

1.473	0.616	0.4344	0.4547	3.7238	1.0745	0.2454	0.2725	
0.8779	1.5276	0.3029	0.3975	0.7155	0.1966	0.2531	1.6296	
1.1608	0.8072							
ABCB10	4.1652	3.481	3.4915	4.3377	4.0633	3.4396	2.9379	
3.6881	3.0475	3.9632	2.6133	2.6505	2.247	3.6157	3.7066	
3.4102	2.5552	3.4997	3.8803	3.8654	3.9664	3.1674	3.8137	
4.1137	3.7925	2.2474	2.1244	3.4875	3.3234	3.8208	4.0075	
3.2907	3.4283	2.9462	3.8542	4.1466	3.1746	3.1376	3.6544	
4.0886	3.9472	2.7516	4.0645	3.6835	4.1511	3.4979	2.9749	
4.8564	2.7107	3.0247	3.7541	3.9125	3.3589	3.2399	3.9607	
3.9613	2.4885	4.149	4.4391	4.0947	3.2076	3.638	4.2328	
3.0595	4.7334	2.2287	4.5458	2.8885	3.0507	3.2012	2.8449	
3.3845	3.5578	2.7072	3.4124	3.2674	4.0367	3.4998	3.373	
2.6725	3.3415	2.5889	2.7744	3.4639	2.9527	3.3903	3.1657	
3.4494	3.481							
ABCB11	0	0	0	0	0	0	0	
0.0076	0	0	0	0	0	0	0	0
	0	0	0	0	0	0	0.0028	0
	0	0	0.0281	0	0	0	0	0
	0	0	0	0	0.0029	0	0	0
	0	0.0042	0	0.0623	0	0	0	0
	0.0246	0	0.0318	0.0294	0	0	0	0
	0	0.0659	0	0.0139	0	0	0	0
	0	0	0	0	0	0.0277	0	0
	0	2.00E-04	0	0	0	0.0117	0.0129	0
	0	0	0	0	0	0	0	0
	0							
ABCB4	1.7539	0.9906	1.0103	0.0886	0.6962	1.2058	0.0798	
0.2754	0.066	3.8845	0.7858	1.8344	0.127	1.2809	2.6146	
1.2236	0	1.8899	0.8726	0	0.2369	3.1179	2.4745	
0.2257	1.985	2.6644	2.7237	2.2528	2.9795	4.1121	0.0658	
1.1147	0	1.3696	4.5574	2.3439	2.3878	2.3448	4.2665	
0.154	0.5197	0.8351	1.0326	1.3059	0.1941	0.1483	2.7739	
2.1557	2.3746	0.2126	0.4681	2.6759	2.6046	0.7105	0	
0.2398	0	2.6075	2.6269	2.3677	2.4267	2.3591	0	
0.5469	2.3773	0	0.9491	2.0911	3.4297	2.3191	1.6845	
3.2247	2.6033	2.4989	3.3352	2.7749	1.0567	2.436	0.569	0
	0	1.7689	2.3683	2.8074	3.1096	2.3517	3.0288	
2.773	2.4525							
ABCB5	4.812	3.5189	1.3032	0.8094	0.0061	1.587	4.2092	
3.255	5.0093	4.3921	3.9004	3.3153	0.078	6.5928	0.7029	
5.4294	0.14	4.3658	2.2893	2.326	5.893	0.2637	0.9585	
0.7633	0.015	0.2026	0.762	1.8628	1.5716	0.9226	0.0905	
5.1288	4.9622	7.394	3.0177	4.3819	3.7412	6.4581	5.9008	
2.7859	6.0402	0	0.0506	1.1054	0.0164	0.0028	4.229	
2.0205	0.3295	0.0513	3.1182	0.6152	7.1854	4.8013	0.1369	
2.6743	0.1299	0.0972	2.0553	0.0814	3.403	5.9734	3.6312	
1.7481	5.9124	0.497	7.0243	1.6084	0.5131	0.1492	5.925	
5.1621	6.4722	0.1823	0.8921	0.742	4.6375	3.9264	6.1858	
3.9072	2.5024	0.4096	3.9183	6.4643	0.1401	6.9202	7.1308	

7.0949	7.4535						
ABCB6	4.1645	4.3318	3.3654	3.6481	3.3434	3.8929	4.442
3.7735	4.0409	3.5481	3.6008	3.4645	3.5841	3.451	2.5672
3.5853	2.4983	2.765	3.9381	4.1083	3.9676	4.1997	4.3817
4.3117	3.6313	2.4806	3.4931	4.4528	4.1012	4.5118	0.264
4.1336	3.9768	4.3996	3.6477	3.2301	3.3291	4.6814	3.6182
4.3031	3.9244	3.3935	4.1782	3.698	2.8495	2.9053	3.7171
4.9384	5.0186	2.3305	4.4241	3.8684	3.654	3.1875	1.1706
4.8812	1.0363	3.9832	3.9359	3.394	4.1378	3.4539	3.0981
3.9826	4.6304	3.4214	4.0649	2.9404	4.2622	4.088	3.7362
4.0599	4.1679	3.7988	3.3952	3.6593	4.019	4.205	3.6812
5.5824	3.98	2.9255	3.0874	3.4893	3.3173	3.7988	3.0671
2.7868	3.6718						
ABCB7	3.6558	4.0205	3.2397	3.6467	3.0795	3.0851	3.4925
2.2307	3.9907	2.8022	3.9241	3.9365	2.729	3.0892	2.9535
3.0484	3.8379	3.4457	3.2716	3.4876	3.6943	3.0836	2.9535
3.3228	2.6957	2.7409	4.857	2.6979	3.1194	4.1915	3.5258
3.4107	3.4151	3.6117	3.0451	3.6962	3.1893	3.6142	2.4337
3.498	3.0232	3.4128	2.8736	3.4661	3.5229	3.7005	3.791
3.2808	3.0903	4.8	3.3538	3.3995	3.0228	3.0675	4.2044
3.8877	4.0752	3.4629	3.1033	3.4418	2.4989	4.0679	3.8006
3.3974	2.2426	2.9884	3.43	2.9791	3.5583	2.6878	3.8595
2.6114	3.2318	3.6256	2.7546	3.1692	3.629	3.4598	3.742
3.0016	3.9308	3.366	3.4679	2.5386	2.6206	3.1477	2.9719
2.8453	3.1541						
ABCB8	3.8134	3.9538	3.7559	2.8439	3.4969	3.434	3.3411
4.1682	3.9318	4.287	3.0304	3.0039	3.6518	2.6384	3.5274
4.3392	3.2114	3.4052	3.8948	4.3555	4.1265	3.9406	4.3752
3.737	3.7053	2.8681	1.5554	3.1711	4.7042	3.0069	4.4494
4.2726	3.989	3.488	4.1789	3.2327	3.0789	4.5439	3.9821
2.7175	4.1594	3.06	3.3843	3.4539	2.5372	3.0117	2.6034
3.5881	4.2225	1.7618	3.2948	2.4983	3.5915	2.8686	3.5348
4.2465	1.8847	2.9701	4.2408	3.1672	3.704	4.2314	4.3298
3.7195	4.1464	3.1909	3.7626	3.4539	2.5164	3.6335	4.5423
4.401	4.625	3.6753	2.4061	3.9069	3.68	3.7596	3.5531
4.779	3.4461	3.1599	4.2823	3.6525	3.4977	4.8175	3.906
4.3555	4.3347						
ABCB9	2.0438	2.7276	2.4295	2.265	3.7696	2.6461	2.2732
3.068	2.2849	2.34	1.5613	1.462	2.9676	1.9845	1.9355
2.9805	2.0763	2.0935	2.2885	4.1842	2.3422	2.7272	3.3299
3.0623	1.9259	1.2925	1.2695	1.979	3.221	2.1214	1.91
3.2938	3.2993	2.9425	2.4915	1.6575	3.667	2.8999	2.1283
2.4197	2.687	2.2901	3.4115	5.1027	2.6418	1.5559	1.3825
2.5897	2.9396	2.2246	2.1478	1.7888	2.559	1.8892	2.0903
2.9564	1.4394	2.3164	3.0107	1.4221	3.39	3.8895	1.3817
2.3911	1.9969	2.15	2.1163	2.3841	2.0503	2.7721	2.4443
1.8427	2.3395	2.4675	2.7015	2.7824	2.6206	2.2515	1.3491
3.2728	2.1772	2.802	4.6287	2.1362	3.052	3.2489	2.0337
2.134	1.938						
ABCC1	3.995	4.1313	4.5587	3.1521	3.9714	3.5251	3.7509

4.2984	3.7387	3.6608	4.4149	3.3608	4.2416	2.733	3.8361	
3.6001	3.7888	3.9981	2.9143	4.6823	3.7433	4.1549	3.859	
4.267	3.7772	4.302	3.853	3.3368	2.8765	4.5047	4.3783	
2.8045	3.1182	3.7902	3.9013	3.2939	3.6698	3.8889	3.5456	
3.9834	3.6334	3.4202	3.7573	2.8854	4.7705	4.4955	3.8637	
3.7474	3.7429	3.5506	4.8963	2.7928	3.1875	3.6755	3.3735	
3.6914	4.6207	3.5809	4.2082	3.7893	3.1319	4.1167	3.9912	
3.894	3.6914	5.4699	4.1122	4.0745	4.091	3.4559	4.0666	
3.4976	3.7129	3.3546	3.1018	3.6058	3.2583	4.0678	3.3107	
4.4618	3.305	4.0314	4.0328	3.7977	2.9915	2.7031	3.5076	
3.3555	3.2013							
ABCC10	2.1379	2.433	2.9127	1.6895	1.999	2.306	1.9304	
2.5479	2.4771	3.1307	2.5479	2.8404	1.922	2.3949	2.6446	
2.1892	3.1606	2.7618	2.5796	2.4176	2.5705	2.1022	2.6342	
1.8342	2.1011	3.196	2.0707	3.0335	2.6346	1.8245	3.0474	
2.7356	2.5594	2.3509	2.6426	2.1946	2.6978	3.2936	2.6717	
1.9421	2.3205	2.1276	2.529	2.3387	2.2251	2.4217	2.0007	
1.7036	2.6007	1.272	2.5143	2.2615	2.4097	2.6244	2.7585	
2.4063	1.9824	2.3579	2.2775	2.027	2.2839	2.2414	2.3775	
2.178	2.9129	1.9442	3.6251	3.4148	3.0976	1.8199	1.9949	
2.5959	2.3483	2.4477	1.9151	2.5839	3.0043	2.7357	2.2603	
2.1287	1.8754	2.8103	3.5919	2.0415	2.0539	2.3939	2.974	
2.9314	3.0734							
ABCC11	0.3486	0.579	0.5637	0.0846	0.1339	0.1674	0.1867	
0.0716	0.3033	0.0734	0.1711	0.0558	0.0443	0.4206	0.1325	
0.3596	0.0381	0.2591	0.3432	0.2613	0.4649	0.1796	0.0129	
0.2973	0.1898	0.0281	0.1111	0.4087	0.1269	0.1355	0.2742	
0.0766	0.2509	0.3278	0.0988	0.0256	0.0953	0.7406	0.0802	
0.2181	0.1302	0.1467	0.0671	0.276	0.0215	0.0753	0.1435	
0.0583	0.0313	0.2916	0.2492	0.1417	0.0963	0.3799	0.2714	
0.2417	0.1158	0.0686	0.2766	0.0649	0.0726	0.1464	0.1021	
0.1399	0.9137	0.1249	0.484	0.0605	0.1279	0.2119	0.4816	
0.0253	0.1291	0.0634	0.366	0.1957	0.1642	0.3304	0.2755	
0.1608	0.2623	0.4065	0.186	0.3412	0	0	0.0603	
0.0669	0.2207							
ABCC12	0	0	0.0231	0	0	0	0	0
	0	0.0152	0	0.0378	0	0	0	0
	0	0	0	0	0	0	0	0
	0	0	0	0	0	0	0	
0.0013	0	0.0157	0	0	0.0022	0.0179	0	0
	0.0071	0	0	0	0	0	0	0
	0	0.0409	0	0	0	0	0	0
	0	0	0	0	0.0099	0	0	0
	0	6.00E-04	0.0253	0	0	0	0.0235	0
	0	1.00E-04	0	0	0	0.0299	0	0
	0	0	0	0	0.0226	0	0	0
	0							
ABCC2	3.9293	4.098	5.4923	0.4052	3.7977	5.6041	5.0445	
0.1529	3.4265	3.9657	2.9915	0.2361	4.5687	3.9219	0.7151	
5.3139	0.6236	3.5573	4.5976	2.2642	0.6186	2.2706	4.9367	

4.9216	3.1272	0	1.103	4.9587	3.2799	4.1963	0.1379	
2.4572	3.0182	0.7544	4.5427	3.8164	4.4553	2.2297	0.6717	
1.7329	2.8976	0.4076	0.2985	3.9156	0.0246	0.7021	5.1139	
3.5898	0.2773	1.9894	3.6323	2.6198	0.3443	0.4831	4.087	
4.0268	0.6435	0.2366	4.3165	0.215	1.2466	4.0661	4.9376	
5.1808	4.3357	0.2936	5.4514	2.1499	4.7791	0.2267	4.5921	
0.951	4.0862	3.4988	2.0567	4.3718	4.5682	5.1017	2.3189	
3.2465	1.3216	1.0235	4.7811	0.2626	0.4108	0.4204	4.4014	
3.5419	3.9953							
ABCC3	0.0228	0	0	2.3466	2.6537	0.0364	0.0864	
0.2457	0.0512	0.0765	0.0162	1.9731	5.106	0.0858	1.4941	
0.0091	0.5863	0	0	0.0256	0.4825	1.5059	2.9333	
0.0404	3.1903	0.4727	0.1943	0	1.3647	0	0.0144	
0.0788	0.041	0.0096	1.067	0.0187	1.0213	0.0456	0.0515	
0.0476	0.0782	4.7665	1.8827	0.0837	4.23	2.9559	0	
0.064	3.8649	0.303	1.0749	0.9828	0	0	0.1356	
0.0437	1.2476	0	0.0326	0.0703	0.5716	0.3109	0.0138	
0.0953	0.0378	5.5293	0	1.1274	0.013	4.1637	2.4064	
0.4378	0	0.1531	0.7719	0	0.0328	1.7963	0.0453	
0.1199	0.0241	2.7553	2.122	0.039	0.9854	0	1.1524	0
	0.2426							
ABCC4	3.8795	2.9512	3.6781	3.4799	3.2017	2.9296	3.1683	
2.8161	3.0977	3.4855	3.4573	2.7362	2.9907	3.9495	3.8659	
2.4519	4.0182	1.6187	3.8791	3.8489	3.5929	3.0397	4.0623	
3.7789	3.5827	2.7829	2.3535	2.0082	3.2759	2.7206	3.8288	
2.1444	3.5293	3.1946	2.4298	4.1118	2.2156	3.2526	2.6213	
3.6739	3.7739	4.1381	4.1355	3.1778	3.3116	2.765	3.9984	
2.9434	1.8042	4.2356	3.4319	3.3627	3.3458	2.5921	4.5108	
4.6225	3.0093	3.0475	3.3539	3.7895	2.8421	3.088	3.0978	
3.613	3.9391	2.1222	3.1709	3.6037	3.6423	3.6313	3.5203	
1.8262	3.3258	3.1098	2.7695	3.526	3.3651	3.9106	4.1687	
3.4441	3.0207	2.6635	2.8744	2.5856	1.808	2.9589	3.3243	
3.4198	3.0103							
ABCC5	3.0648	4.6459	4.8049	3.5808	3.1853	4.4008	4.6137	
3.9431	4.138	3.3635	4.2455	4.3299	3.2782	4.3009	4.6117	
4.1151	3.9297	4.7924	4.7373	3.2159	3.277	3.3495	3.5257	
4.7935	3.2811	3.145	4.0806	4.8939	3.7511	5.8701	3.6426	
4.0772	4.4635	4.1216	4.1282	3.4071	3.4118	4.4386	3.7529	
4.5877	4.8514	2.9394	3.2984	3.5637	3.813	3.3475	4.6237	
4.9422	3.0964	4.1305	3.7316	4.0265	4.0679	3.5403	3.5407	
3.916	3.0318	4.5885	4.1039	4.1821	3.7151	4.3423	3.6927	
4.5773	5.2424	3.995	5.0002	3.8366	5.3732	2.7582	3.1922	
4.0379	3.4072	4.3023	3.2913	4.8075	4.5792	3.6833	3.9709	
4.2245	3.6524	3.4437	4.3749	3.978	2.5111	3.7144	3.6373	
3.7374	4.4248							
ABCC6	0.0324	0.0149	0	0.4957	0.1839	0	0	
0.2057	0.169	0	0.3758	0.1611	0.2776	0.1533	0.1243	
0.0604	0.0275	0.2401	0	0	0	0.5323	0.5861	
0.0081	0.4607	0.007	1.3708	0.1608	0	0.2818	0.9169	
0.0687	0	0.0327	0.2129	0.1066	0.0502	0.4012	0.1949	

0.0836	0.1446	1.8081	0.9237	0.0929	0.3322	0.1055	0.5721	
0.0978	0.1917	1.7584	1.2756	0.5503	0.1951	0.1452	0.5738	0
	0.0377	1.0714	0.4905	0.773	0.3984	0.184	0	0
	0.0515	1.9268	0.0433	0.674	0.2273	0	0.2789	0
	0	0.4495	0	0.1814	0.2709	0.0147	0.0669	
0.1804	0.5125	0.2061	0.1545	1.0065	0.3811	0	0.1827	
0.1347	0.1702							
ABCC8	0	0	0.0983	0	9.00E-04	0	0.5774	
0.074	0.0156	0	0.0603	0.0442	0	0	0.0182	0
	0	0	0	0	0	0.0424	0.2012	
0.0174	0.4365	0.0055	0	0	0	0	0.5814	0
	0.0128	0	0	0.0931	0.0132	0.0282	0	0
	0	0	0.1602	0	0	0	0	0
	0	0	0.0828	0	0	0.0064	0.2683	0
	0	0	0.0146	0	0.1103	0.0063	0	0
	0	0.0033	0	0	0	0.6467	0	0
	0.0262	0.0038	0.0099	0	0	0	0.0724	0
	0.0083	0	0.3938	0	0.0235	0	0	0
	0							
ABCC9	0.0301	0.0081	0.0162	0.0368	0.0649	0.0396	0.0159	
0.0161	0.0435	0.0074	0.0465	0.0845	0.0396	0.1321	0.0336	
0.0184	2.1151	0.0297	0.0653	0.1133	0.0535	0.0314	0.0056	
0.0432	0	2.9153	0.0656	0.0702	0.0248	0.0063	6.00E-04	
0.0096	0.4689	0.0447	0.0571	0.0033	0.2459	0.0645	0.0187	
0.2664	0.0276	0.0167	0.0247	0.0341	0.5506	0.0262	0.058	
0.0109	0.0252	1.7198	0.0755	0.0549	0	0.0765	0.0979	
0.0832	3.0989	0.1148	0.1096	0.0673	0.1271	0.0337	0.0188	
0.0669	0.0291	0.3652	0.2205	0.0821	0.0205	0.0535	0.0792	
0.0648	0.0716	0.0577	0.0582	0.0746	0.0447	0.0467	0.1212	
0.063	0.0649	0.0388	0.0219	0.0435	0.032	0.0663	0.4669	
0.0313	0.0372							
ABCD1	5.6761	3.9271	4.8134	2.0025	3.2398	2.7431	2.8879	
2.6842	3.3598	4.3577	4.3234	3.4762	3.4762	2.2835	3.8828	
4.8512	1.6062	3.643	3.0437	5.0853	4.6182	3.4545	3.7784	
4.3986	2.7542	2.5805	1.2337	4.0077	2.7119	4.5747	2.6934	
5.5735	4.1666	4.8295	3.0179	3.64	4.6646	3.4751	3.3361	
1.6178	3.2768	3.4115	3.0078	3.0357	2.6155	2.6989	2.9594	
3.2997	3.6189	1.9967	2.1291	1.7355	2.0036	3.6947	3.0135	
3.3613	1.0328	3.1749	5.5701	2.4559	2.103	5.2792	3.6559	
4.49	2.8178	2.8342	4.0542	2.771	4.464	3.171	3.8908	
2.7161	5.0233	3.3065	3.3118	4.3226	4.7927	4.1335	3.6194	
2.3834	4.455	2.9273	3.4977	2.951	3.0711	2.9216	4.8512	
3.5972	3.342							
ABCD2	0.071	0.0119	0.1242	0.0817	0.0081	0.3027	0.0805	
0.0094	0.2636	0.1764	0.0496	0.0307	0.0451	0.1091	0	
0.0198	0.3366	0.038	0.047	0.1191	0.0451	0.0113	0.0414	0
	0.0101	0	0.0116	0.2815	0	0.0253	0.9629	
0.0084	0.0357	0.3723	0.0191	0.3658	0.0331	1.0277	0.0057	
0.077	0.1053	0.0748	0.0312	0.2023	0.12	0.0824	0.4258	
0.0982	0.0668	0.3169	0.2357	0.0153	0.2346	0	0.3886	

0.0454	0.0165	0.1393	0.009	0.0113	0	0	0.0036
0.0374	0.5494	0.173	0.4751	0	0.2913	0	0.1615
0.0036	0.2096	0.0684	0.3118	0.2507	0.1596	0.0505	0.1699
0.1893	0.0774	0.0639	0.266	0.1282	0.0228	0.3714	0.146
0.1565	0.3996						
ABCD3	4.2964	4.6785	4.787	5.1229	4.2787	4.8489	5.2459
3.5978	4.4322	4.3859	3.9698	4.7088	4.4244	4.8036	4.535
4.9173	3.7949	4.6192	4.8852	3.9754	4.6818	4.5304	4.728
4.729	4.5051	4.5799	3.5528	4.4219	3.892	3.8519	3.9761
4.5764	4.5836	4.5755	5.231	4.7368	4.6822	5.0287	4.4417
4.0435	4.249	4.9978	4.3309	4.5769	3.9053	5.0886	4.7738
3.4832	4.0399	5.9656	4.4464	4.9563	4.7054	4.0773	4.4869
4.6175	4.1458	5.1243	3.983	5.2751	3.9065	4.3436	4.0347
5.1791	4.297	5.0078	4.9682	4.607	4.1455	4.4085	4.1636
4.9483	4.3898	4.544	4.5402	5.1282	4.8524	4.6728	4.8702
4.1581	4.725	4.7841	3.881	3.5276	4.655	4.6911	4.894
4.8919	5.1148						
ABCD4	3.5637	3.5832	2.9791	2.9274	3.0443	3.1462	2.2523
3.147	2.9537	3.5908	2.7963	3.5249	2.9354	2.7167	2.9997
3.0244	3.5192	3.0859	3.1426	3.6697	3.3811	3.2988	3.819
2.9648	3.003	3.0618	3.5103	4.0485	3.5297	3.0187	4.2563
3.4148	2.9279	3.0044	2.7422	2.9492	3.3068	3.8505	3.0637
3.1066	4.631	3.6188	3.0257	3.4798	2.9717	3.0412	2.4171
2.893	3.6952	3.3121	2.7854	3.1872	2.7315	3.2895	2.8396
2.9671	3.4037	2.5737	2.3353	2.6468	3.2357	3.2706	3.6479
2.7681	3.672	3.4905	3.1305	3.1494	2.9932	3.3376	3.0419
2.8257	2.9601	3.121	4.2141	2.7607	3.1649	2.7786	3.5833
3.1872	4.2416	3.4447	2.6824	3.121	3.0948	2.9522	3.2431
3.5164	3.8837						
ABCE1	5.4138	5.3241	5.7168	5.8573	5.5985	5.4864	5.1595
5.1129	5.1392	5.222	4.8386	5.4442	4.6679	6.0681	4.9206
4.5182	5.4805	5.1379	5.463	5.2341	5.6654	5.2946	4.5984
5.4072	4.7333	5.8824	4.671	5.2341	4.9454	4.8949	4.6943
5.3241	5.4042	4.1757	5.0003	5.5857	5.1337	5.3006	5.4948
5.5985	5.1345	5.0914	5.0843	5.0882	5.1701	5.7736	5.421
4.6324	4.8077	6.2435	5.3557	5.7287	5.5017	5.8206	5.2261
5.1755	5.6086	5.1073	5.275	5.1948	5.1896	5.4979	5.3887
5.0485	5.3665	4.2012	5.1029	4.5876	5.2655	5.3085	5.432
5.3629	5.7993	5.1743	5.0283	4.6932	5.1978	4.6802	4.8215
4.8044	5.4737	5.171	3.9458	5.0884	4.9948	5.4224	4.6228
5.4922	4.7328						
ABCF1	4.7262	5.7843	5.1208	6.1608	5.3218	5.3429	4.7161
5.1809	5.0994	5.8056	4.5926	5.9088	4.4224	5.5023	4.7758
5.0936	5.215	5.438	5.3854	5.3297	5.5171	5.6369	5.406
5.1977	5.239	5.6988	4.7151	5.5061	5.5879	5.1865	4.9147
5.2962	5.0226	4.3454	5.2316	5.6928	6.0502	5.6849	5.0508
5.0981	5.1718	5.2996	4.9378	5.0311	4.6653	5.8349	4.609
4.9906	4.769	5.3876	5.0483	5.9603	5.1737	5.5252	5.4567
5.4705	4.4795	5.6857	4.6376	5.6058	5.322	5.794	5.2407
5.1088	5.7638	3.9054	6.1482	4.9704	5.2212	4.8302	5.0526

5.3301	5.1957	5.4528	5.4221	5.3301	5.6761	6.0678	5.2793	
5.6865	5.061	5.4022	5.3332	5.0884	5.3438	5.3134	4.6653	
4.7086	4.7971							
ABCF2	5.1675	5.3621	4.4175	5.3998	5.1059	6.2336	5.1627	
5.5733	5.3642	5.1771	5.3591	5.4232	5.0879	5.6956	5.3532	
6.0402	5.4042	5.7466	5.8907	5.4583	4.9286	5.6414	5.2516	
5.6893	5.6858	5.1826	4.1276	4.3254	6.0318	4.6533	5.3272	
5.2746	4.3549	3.7281	5.4702	5.3539	5.6995	4.9007	5.5733	
5.5577	5.184	4.7086	5.0256	5.2452	4.8283	5.4508	5.0321	
5.4013	5.6214	4.5025	5.0098	6.151	5.8726	5.1155	5.7809	
5.2801	3.6235	5.5546	5.7615	5.8262	5.3099	5.927	5.8288	
5.1919	5.2171	4.2646	4.5744	4.8912	4.208	5.5461	5.6733	
5.3644	5.8739	5.0779	4.8698	5.1004	5.1036	5.1342	4.9016	
5.239	4.9381	4.6989	5.5098	4.9592	5.0103	5.8134	4.2703	
4.6203	4.2014							
ABCF3	4.0344	4.3243	4.5952	3.9101	3.9341	4.4703	4.2444	
4.5742	4.274	4.1979	4.697	4.8243	4.1377	4.2677	5.0428	
4.3088	4.2723	4.2942	4.0557	4.4638	4.5348	4.414	4.2444	
4.9378	3.7216	4.2304	3.4973	3.7762	4.5795	4.1794	4.3773	
4.916	4.7584	4.8618	4.3673	3.8584	5.0663	4.9128	4.4372	
4.3138	4.4646	4.5925	4.2812	3.827	4.5047	4.0519	3.8993	
4.3758	3.4517	3.6665	4.5701	3.9501	4.1222	4.2643	4.5815	
4.6206	4.6176	4.3565	3.7336	4.6443	4.6509	4.3161	4.1226	
4.1081	4.0107	4.6646	4.4337	4.5599	4.1595	3.8002	3.7506	
4.2597	4.5254	4.5627	3.7463	4.3495	4.5112	4.5797	3.9331	
4.5391	3.7902	4.0788	4.5254	5.1824	4.4992	4.4056	4.3307	
4.2365	4.4425							
ABCG1	0.5094	0.1466	0.3268	0.3648	0.1791	0.0514	0.1759	
0.3876	1.2507	0.1282	0.8051	0.0557	2.3572	0	1.9536	
0.2072	0.1839	1.6776	0.0641	0.2918	0.0128	0.1504	0.5197	
0.1644	1.1114	0.4573	0	0.0272	2.3351	0.3271	1.6044	
0.0219	0	0.6628	0.042	0.4931	0.7055	1.7313	2.0415	
0.0381	0.0749	0.7445	0.1784	0.0431	1.8533	1.3433	0.1452	
0.0469	0.998	0.471	0	0.66	0.1328	0.2381	0.7816	
2.3744	2.8419	0.1264	0.1108	0.1867	0.287	0.2175	0.436	
0.1616	0.2385	1.0369	0.503	0.1404	0.1447	2.5645	0.0812	
0.3165	0.2426	0.1968	0.2748	0.1327	0.2507	0.4773	0.1729	
0.2071	0.2199	1.1291	0.4593	1.0908	0.4328	0.3735	0.5039	
0.1455	0.6115							
ABCG2	1.3519	0.4151	1.4342	1.5494	0.8621	0.4968	2.8577	
2.3809	1.1616	0.0614	0.6509	2.2447	1.8058	2.5529	0.4267	0
	0	0.7111	1.2898	1.5066	1.3477	0.7488	0.5475	
1.0725	1.0957	2.2547	0.2038	0.3275	0.9285	0.2885	0.3978	
1.138	1.06	1.5335	2.5635	0.6799	1.6105	0.9526	0.1947	
0.503	1.5737	1.8736	1.5684	0.6051	1.0814	2.9234	1.7206	
0.8796	2.0315	0.0573	2.6361	0.8276	1.4509	0.5151	1.6732	
0.6175	0.8438	0	0.6565	0.2294	1.7642	1.0291	0.6791	
1.5769	0.4242	0.3531	0.1572	2.1377	0.4774	0.6162	2.5948	
2.1136	1.4667	1.1141	0.5427	1.9496	1.4476	0.4706	0.6448	
0.1858	1.0472	3.2712	0.9419	0.6418	1.8941	1.1	0.5972	

1.0767	1.0096							
ABCG4	0.6557	0.0315	0.5508	0	0.1729	0	3.5121	
0.0624	0.2971	0.6801	0.4434	0.3978	0.3531	0.1945	0	
0.4888	0.4759	0.0015	0	0.3457	0	0.1344	0.1465	
0.0163	0.1135	0.0245	0.6573	0.2416	0.3119	0.1795	0.9655	0
	0.2382	0.3122	0	0.2255	0	0.1173	0.0343	
0.0718	0.3852	2.82	0.3398	0.3063	0.3852	0	0	
0.1674	0.1201	0.0064	0.4992	0.0047	0.0573	0.1245	3.655	
0.1816	0.1129	0.203	0.1177	0.1549	0.1044	0.128	0	
0.2141	0.1027	0.2455	0.763	0.4748	0.1801	0.1377	0.2503	
0.105	0.1558	2.0203	0.3952	0.1431	0.0945	0.0949	0	0
	0.1192	0.1716	0.1358	0.2335	0.1065	0.1254	0.8347	
0.3335	1.1301							
ABCG5	0.0862	1.0566	0.3788	0.7817	0.081	1.1051	0.6819	
0.1377	0.0691	0.1532	0.0348	0.1658	0.0416	0.1505	0.1094	
0.283	0.0832	1.7698	0.5976	0.6647	0.1882	0.0322	0.0808	
1.5698	0	0	0.0772	0.1744	0.2311	0.5617	0.1124	
1.8579	0.6033	0.2872	0.1513	0.3894	0.4372	0.4523	0.1252	
0.7306	0.3425	0.0285	0.0666	0.2352	0	0	0.8115	
0.3339	0.0502	0.1053	0.1498	0.1309	1.4289	0.4993	0.0328	
1.3737	0	0.0566	1.6301	0.0293	0.0425	1.681	0.8228	
0.582	0.6363	0	0.0194	0.026	0.3541	0.0588	0	
0.0866	0.4549	0.0515	0	0.5131	0.8474	0.2035	1.0544	
0.811	0.3325	0.0065	0.326	0.1929	0	1.1781	0.1345	
0.246	0.0812							
ABCG8	0	0	0.0344	0	0	0	0	0
	0	0.0161	0	0	0	0	0	0
	0	0	0	0.0692	0	0	0	0
	0.0067	0	0	0.0396	0	0	0	0
	0	0	0.0017	0	0	0	0	0
	0	0	0	0	0	0	0	0
	0	0	0	0	0.1118	0.0319	0	
0.0626	0	0	0	0	0	0	0	0
	0	0	0	0	0	0	0	0
	0	5.00E-04	0	0	0.0239	0	0	0
	0	0	0	0	0	0.0662	0	0
	0							
ABHD1	0.0927	0.5248	0	0.2278	0.2334	0.0722	0.1861	
0.023	0.3388	0.2821	0.4782	0.597	0.3317	0.3199	0.0918	
0.0586	0	0.2235	0	0.4403	0.064	0.0797	0.8275	
0.3121	0.2418	0.0619	1.0441	0.6001	0.4053	1.258	0.6276	
0.1358	0.0962	0.3712	0	0.3731	0.3854	0.3192	0.1488	
0.1542	0.7683	0.7122	0.2098	0.424	0.0616	0.4777	0.4095	
0.6924	0.3502	0	0.3095	0.3626	0	0.4335	0.1114	0
	0	0.2619	0.258	0.1025	0.1351	0.1984	0.2571	
0.1233	0	0.1207	0.2317	0.1006	0.9623	0.2318	0.2008	
0.2747	0.1674	0.3811	0.0805	0.2855	0.2261	0.1429	0.4295	
1.0323	0	0.4922	0.2195	0.3896	0.1841	0	0.189	
0.1503	0.169							
ABHD10	2.9718	4.2972	4.1721	4.0618	4.4634	3.8819	4.3335	

4.1987	4.4317	3.9847	4.8928	3.6743	2.6365	3.9046	3.8167	
4.5074	3.5787	4.5045	3.8942	3.6259	4.4579	3.5157	3.1023	
3.2899	2.9388	2.6915	3.3371	4.525	3.8554	4.7109	3.1444	
3.6187	3.9234	3.8855	3.5003	4.2109	3.1526	3.681	3.7185	
4.316	3.0231	3.6402	3.3967	4.0713	4.3472	3.903	4.6851	
3.8684	3.5953	4.022	4.54	3.7293	4.5631	3.8446	3.0366	
4.7926	3.8026	4.5226	3.4366	3.881	4.0121	4.2176	3.1039	
4.2915	3.2243	3.6803	4.0888	3.3147	4.295	3.8275	3.5736	
4.1805	5.1776	4.0823	3.006	3.9896	4.1227	3.7536	3.8523	
4.1153	4.1383	3.1902	2.9189	4.3604	3.5504	4.5125	4.0213	
4.3358	3.9314							
ABHD11	3.7128	2.5087	2.7017	2.8133	2.5646	2.8001	2.7175	
3.5832	3.4134	2.8531	2.4369	1.695	3.8549	2.3597	3.8484	
1.9448	2.7341	2.8851	3.3233	2.8446	3.0318	2.9974	3.9822	
3.3257	3.0969	3.0925	2.6205	2.2033	3.7715	2.1585	1.8323	
3.1531	2.5357	2.5534	3.9006	2.4585	2.7126	3.0652	3.5326	
2.7651	3.8684	2.5964	3.652	2.7763	2.5433	2.8673	2.1128	
2.8612	2.9659	1.622	2.7227	2.6509	1.6527	1.9866	3.1898	
2.6842	1.6276	3.1388	2.2428	3.7221	3.674	3.7128	3.4736	
2.6448	3.0374	2.7173	2.9703	2.8582	1.5223	3.683	3.0695	
2.8167	3.9721	1.4002	1.8496	2.4387	2.9185	3.3935	2.3471	
2.7404	2.4442	2.6211	3.0237	2.6913	3.865	2.6635	2.1128	
3.5272	2.9409							
ABHD12	4.9579	5.8267	5.2616	5.3757	5.061	5.6988	5.5983	
6.3827	5.8731	5.3833	5.7004	4.9006	5.9662	4.7117	5.7164	
5.8248	4.2801	5.7048	5.6206	5.1675	5.031	5.9821	6.4298	
4.9441	6.3038	5.4729	5.2726	5.2595	5.3049	4.3234	4.4965	
5.6443	6.1538	6.8742	6.082	5.0423	5.1583	6.189	6.1715	
4.982	5.7564	5.4858	5.8336	5.9662	5.9736	4.7522	5.4778	
5.0663	5.7679	4.3954	6.5896	4.7424	5.7164	5.2529	4.9943	
5.6292	3.4955	5.1929	6.1918	5.2322	4.7309	5.7026	5.576	
5.78	5.362	6.124	4.7714	5.2688	5.5285	4.7731	6.1252	
4.7986	6.2325	5.676	6.3602	4.9755	5.9363	6.2754	5.4401	
5.0065	5.726	5.8594	6.0069	6.1363	5.6995	5.825	5.8081	
5.189	5.5676							
ABHD12B	0.5879	0.2443	0	0.0814	0.0618	0.1871	0	
0.1632	0.0486	0.2876	0.8698	0.1343	0	0.2144	0.1092	
0.0955	0.0652	1.148	0.2811	0.0552	0.2149	0.0336	0.063	
0.2319	0.0379	0.0716	0.2649	0	0.1779	0.1667	0.0244	
0.0779	0.1076	0.0315	0.0063	0.4063	0	0.1182	0.0464	
0.0505	0.1279	0.0526	0.2924	0.241	0.1003	0.0842	0.0427	
0.1476	0.1606	0.0836	0.082	0.2489	0.5371	0.4589	0.3168	0
	0.1284	0	0.4896	0.1476	0.4245	0.1292	0.2749	0
	0	0.0104	0	0.0131	0.103	0.2086	0.1865	0
	0	0.0261	0.1426	0	0.022	0.6561	0.09	0
	0.3999	0.2185	0	0.0465	0.1005	0.6186	0.1806	0
	0.1035							
ABHD13	2.4028	2.4425	2.8233	3.3412	3.0575	2.8288	3.2958	
3.2159	2.9761	2.8821	2.4583	2.6951	2.456	2.8412	3.0757	
2.3229	2.7177	2.4248	2.6862	2.5682	3.245	2.7177	2.928	

2.4416	3.0539	2.0967	3.3475	3.0923	2.5077	2.7187	3.1125
1.8383	2.2966	2.1391	2.7502	2.9789	1.9624	3.0805	2.6882
2.3719	2.4054	2.3365	2.194	2.8337	2.9246	2.0592	2.9696
2.5812	3.1239	2.7151	2.9521	2.9419	2.8221	2.958	2.7607
3.2939	2.7563	2.322	2.4191	2.4176	2.2333	3.0649	3.1255
2.5161	2.2992	1.9973	2.2948	2.3551	3.4625	3.4141	2.9583
2.63	2.9701	2.326	2.8756	2.925	2.5282	2.2796	2.9907
2.3134	2.6792	2.7687	2.1878	2.9234	2.2953	2.4065	2.3216
2.6314	2.4569						
ABHD14A	2.8675	3.6523	2.8025	2.7353	3.5279	2.931	3.7492
3.1729	2.6354	2.0956	2.8886	3.5321	3.0907	1.5238	2.4752
3.6383	2.988	2.4258	2.2599	2.939	2.803	3.3863	4.9626
2.7375	3.0408	2.213	1.8035	3.7837	4.2006	3.6603	4.2538
4.5676	3.2888	2.763	3.583	2.2255	3.1154	4.1965	3.5249
2.0362	3.8727	3.7845	2.5647	3.0777	2.23	1.3483	3.2159
3.5154	3.4897	2.9433	3.1007	2.4949	1.7065	2.6091	3.2844
2.8623	2.241	2.8682	2.1551	2.3121	3.183	3.1717	4.6191
2.4334	2.6637	4.713	3.9204	2.8256	3.7785	2.6724	3.5109
2.2323	3.5647	3.2565	4.7351	2.4002	2.7042	3.7258	3.6218
3.2066	2.784	4.1608	3.105	2.2773	3.8261	2.0841	3.3643
3.3392	3.2774						
ABHD14B	5.5285	5.6172	4.1524	4.1737	5.4031	3.6424	4.4087
3.8257	4.2594	6.0294	5.6386	5.9796	4.8205	4.3019	3.9022
4.8707	5.2575	5.3132	4.235	5.2148	4.0797	5.0763	5.6876
5.4307	2.9146	3.5913	6.3842	5.7937	5.178	5.6356	4.0072
6.2048	3.8013	5.1749	5.3103	5.2659	6.4181	4.9401	5.4039
4.6729	5.8621	5.3521	4.5084	5.172	4.1288	3.7724	4.8939
5.5162	5.1867	5.6688	5.2506	5.0727	3.9624	4.9401	5.1496
3.9661	5.011	2.8302	4.7189	2.4419	4.9004	5.9693	4.7886
4.7374	5.1985	4.8098	5.9258	5.8469	4.8878	4.4533	4.4584
4.3997	4.6443	5.5884	5.6593	4.0135	5.6086	6.1688	4.8626
5.3202	4.7297	5.5677	5.2871	4.5929	4.2447	3.5929	5.7346
5.4358	5.38						
ABHD15	3.599	2.1014	2.2603	1.9953	2.6265	2.3557	2.4701
2.3249	2.3474	2.5057	3.2607	2.5201	2.1029	2.4309	2.1055
2.8844	2.152	3.5425	2.1076	2.8679	2.1524	1.8209	3.2643
2.5246	2.4404	3.2681	1.2742	3.0486	2.8607	2.6405	3.0713
2.4882	2.1298	2.9148	3.1978	2.8603	2.7661	3.3395	2.5302
2.8026	2.6009	3.3445	2.9221	1.7062	2.4129	2.2859	2.5784
3.4944	1.9673	1.7368	2.4982	2.6537	1.9324	2.6315	2.2327
3.0023	1.518	3.4635	2.0511	3.1226	2.8873	2.4148	2.3816
2.3827	1.933	3.3644	2.7591	2.9716	2.4956	3.2575	2.2534
2.3351	2.4696	2.268	2.6538	2.8327	1.8305	3.4024	2.4045
2.1283	1.9136	1.2836	2.4372	3.1576	2.4111	2.0315	2.7159
2.3714	2.5499						
ABHD16A	3.6333	3.8353	3.5817	3.6106	3.3087	4.2104	3.3356
3.6775	3.8552	4.2489	3.4257	3.8984	3.6146	3.7785	3.791
4.2754	3.8865	4.6166	3.8861	4.1267	3.195	4.1455	4.5823
3.449	4.7262	4.7174	2.9388	4.0796	4.5054	2.5969	3.6594
3.7287	3.6771	4.051	4.9503	3.5687	4.347	5.2598	3.9433

3.0925	3.9515	4.3078	3.2274	3.2342	3.2652	4.3577	3.0134	
3.2004	3.9272	3.2698	3.7305	3.8006	3.7529	4.1214	3.2269	
3.7915	3.0718	3.6584	3.5405	3.8631	3.7495	4.2569	4.6021	
3.5478	4.5974	3.0496	4.0117	3.7822	3.4461	3.8792	3.5991	
3.816	3.6607	3.2443	3.5816	4.0474	4.1699	5.0203	3.5653	
3.719	3.3166	4.2273	4.714	3.8706	4.9118	3.9853	4.1924	
3.9536	4.0482							
ABHD16B	1.4361	1.5769	1.7034	0.7986	1.3055	0.7829	0.9232	
1.9232	0.8008	0.7974	0.8057	0.647	1.1664	0.5994	0.8709	
0.5513	1.9924	1.2521	1.4789	1.5528	2.0964	1.1858	1.4435	
0.648	1.0115	0.9423	0.5626	0.9423	1.0233	0.7125	1.7586	
2.5465	1.0092	0.7273	1.081	0.6496	1.8305	1.6247	0.5744	0
	1.1759	1.2761	0.7585	1.0349	1.5264	1.0939	0.4871	
1.4964	1.6764	1.3668	0.8052	0.8039	1.4208	1.2703	1.723	
0.7074	1.4181	0.3026	1.2884	0.7912	1.4797	0.6752	0.9271	
1.0644	1.0748	1.8363	1.8408	1.2278	0.8854	1.5424	1.443	
1.4728	1.5246	1.5083	1.0825	2.0574	1.1462	0.9831	1.8629	
1.2062	1.7696	1.7884	1.7966	1.7433	2.0622	2.0864	1.4354	
1.4067	1.5352							
ABHD17A	3.8619	3.619	3.7537	1.8871	3.9516	3.0655	2.8783	
4.1565	3.2189	3.3513	3.6903	3.1484	3.2287	2.6624	4.1732	
3.4895	3.5347	2.5736	3.1335	3.9102	4.6096	4.1068	4.1171	
3.6037	2.5107	3.1873	3.9685	3.8084	3.4537	4.019	4.2033	
4.7724	3.8842	4.5752	3.5605	2.7121	3.7247	4.998	4.7795	
2.1389	4.959	3.8374	3.3241	3.371	2.5948	2.4523	2.531	
2.9147	4.3522	2.5522	2.9114	2.5862	2.5905	3.1078	4.5574	
4.692	3.2171	3.9301	3.0175	3.5305	4.515	3.3037	3.5884	
2.9711	4.1062	4.0371	5.3963	4.5489	3.6285	3.7292	4.5251	
3.9725	3.5291	4.2071	3.5357	4.0371	4.1341	3.9661	4.0635	
3.5305	3.7307	4.7575	4.2455	3.8965	4.8757	3.9381	4.0726	
4.8547	4.4631							
ABHD17B	3.0861	3.347	3.2352	4.0174	4.1748	4.2013	3.4451	
4.0463	3.175	3.2687	3.4099	3.6893	3.4964	3.1661	3.2655	
3.0957	2.856	3.5348	3.6968	3.3531	2.7961	2.5936	2.9633	
3.4769	3.2691	1.9725	4.6994	3.9773	2.9101	3.4479	3.2702	
3.5885	2.8515	3.5767	2.3575	3.6765	3.302	3.3765	4.3357	
3.6972	3.6803	3.2297	2.9797	4.0261	3.1915	3.2333	4.1213	
2.9697	2.5569	3.1565	2.9976	3.2918	3.9143	2.952	3.3639	
3.6277	3.2951	5.0022	4.0813	4.6775	3.3086	3.1368	3.6004	
3.5076	4.0237	3.1012	2.8775	3.3525	3.9001	4.0362	3.8355	
2.7339	3.3339	3.0234	3.3481	2.8516	2.8715	3.3831	2.7718	
3.7189	3.3854	3.3276	2.7863	3.9409	2.9063	3.701	3.5257	
3.896	3.7757							
ABHD17C	4.0639	3.9844	4.4138	6.4818	5.8917	4.4215	7.0831	
3.7998	3.1878	3.9316	4.0821	5.1756	3.3706	4.7779	4.525	
4.4286	2.5228	5.2411	4.2246	3.4211	3.7442	4.719	4.3165	
4.6347	4.9086	3.4215	4.3092	3.665	5.1764	4.1184	6.1671	
3.6945	4.026	3.2811	3.6253	4.1934	4.6978	3.2294	4.0501	
4.1583	3.9389	3.2387	3.1262	4.9118	4.9843	4.0024	4.0703	
4.6373	6.6071	4.5629	4.2332	5.8767	4.063	7.0033	4.1123	

4.0404	2.9917	3.4688	3.7754	3.7511	5.7606	3.1247	4.3146
5.4502	3.3653	3.3502	2.9098	3.8199	4.2258	4.7627	4.313
4.8344	4.9847	3.8673	3.7028	3.5841	5.1811	3.4155	3.6388
4.9137	3.5227	4.6	5.3554	4.4272	4.7535	3.3268	4.3402
3.9308	3.5478						
ABHD2	4.1735	4.2326	6.5405	3.6239	4.0924	6.0992	6.4687
4.2371	4.2071	5.1059	4.8521	3.8135	4.9397	4.6277	6.6777
7.0166	4.8157	6.7429	4.5809	6.4563	3.4996	5.3082	5.2797
4.5385	5.8729	4.2671	4.2156	5.2835	5.6701	6.1724	3.1685
3.0844	4.9535	3.9542	5.3628	4.9258	3.9519	3.6487	5.6094
4.5782	4.4617	4.5582	5.959	4.9198	5.2413	4.3464	5.7254
5.2367	5.655	3.7661	3.7044	4.9191	4.2426	3.8165	3.5221
3.6561	5.0175	3.2552	5.0225	3.7217	5.521	4.3218	4.0134
6.741	3.7576	4.7539	3.4803	7.0857	7.3739	4.9488	4.6845
5.3943	3.9581	4.6879	6.2852	7.2698	4.8266	5.2547	4.6975
4.0779	3.9313	4.6048	4.7069	4.0752	4.8164	4.0179	3.6588
4.3159	4.6816						
ABHD3	2.9065	2.9854	3.0209	4.3459	2.8859	4.1965	3.4378
2.4288	4.3063	3.8791	3.124	2.9632	2.5185	4.0409	3.8451
2.6802	2.1132	3.7248	4.1986	3.2664	2.8372	3.5852	3.4103
3.4676	3.7426	2.0308	3.0794	4.2426	3.9438	3.0526	4.1244
3.1096	3.7375	3.9367	3.672	4.166	3.1178	2.9467	3.2654
2.7064	3.0966	2.6295	3.4519	3.8502	3.5749	4.1356	3.8114
3.5571	4.0293	3.069	4.1558	3.7663	4.0579	2.4242	3.4907
3.6995	2.6242	3.3993	4.4734	2.9321	3.4561	2.7496	3.4861
4.4905	4.0018	2.6672	4.2142	2.4924	3.2929	2.6025	3.5953
4.0404	3.0392	3.2689	3.4125	3.4638	3.2653	2.496	3.6112
3.5188	3.2528	2.2301	3.0129	3.1917	3.6701	4.461	3.2852
3.5821	3.4612						
ABHD4	2.7323	3.4544	2.9688	3.6847	2.5826	3.7107	5.7611
3.3831	3.2767	3.0753	3.0093	4.1686	4.4496	3.1942	3.5821
4.2865	3.3827	3.8399	2.896	3.3842	3.366	3.3138	4.6865
2.5385	2.6907	4.0166	5.0887	3.9595	4.0209	4.0604	5.5569
3.097	3.7251	3.5935	4.7402	3.6783	4.013	5.0403	2.7906
3.1604	3.6783	4.3153	3.0576	3.1426	3.2279	3.6303	2.3622
4.4284	4.2266	4.5375	3.9673	4.0784	3.6232	3.3446	4.8921
3.4307	4.7921	3.146	2.7248	3.0881	3.3475	2.9225	2.6678
3.5948	3.3932	5.9305	3.6708	5.072	4.393	3.5899	3.1545
3.6567	3.8095	3.6377	4.2125	3.5475	3.1932	4.1413	3.6797
2.939	3.0953	3.5684	5.1802	3.7875	2.6624	3.1738	4.9755
3.3382	4.1736						
ABHD5	1.9634	2.2122	2.1861	2.5815	2.1153	3.0107	1.9151
1.6163	1.929	2.2162	1.8444	2.5913	3.2444	2.3723	1.8723
3.3734	2.0949	2.8723	2.8294	2.4608	2.4119	1.7424	2.1356
2.9794	1.8254	2.4617	1.6276	2.4839	2.6768	1.5873	1.9449
2.6859	2.4182	1.6308	2.4266	2.7074	2.313	2.0313	1.8025
1.956	1.6963	2.379	1.4043	2.2294	3.1244	1.3478	2.6575
2.185	1.24	3.198	1.4949	2.7262	1.4652	1.1459	1.808
1.5747	2.6067	1.9703	2.8507	2.2925	2.2936	2.2825	2.6374
2.722	1.9901	3.1677	2.3113	1.9926	2.6378	1.6973	2.4111

1.9908	1.9251	0.9131	3.2232	2.2945	2.3722	2.891	1.7601
2.0487	1.8779	1.8909	1.8292	1.6273	2.5859	1.5353	1.8717
1.8149	1.946						
ABHD6	3.7055	4.0023	4.0616	3.4846	3.3173	2.954	4.2589
3.1985	3.435	4.5122	4.6499	3.8181	3.178	3.3143	4.0161
4.1127	2.0756	4.1105	3.4936	4.1135	3.2275	2.7941	3.5234
4.3012	2.8422	2.159	2.2733	3.9198	3.7347	4.4322	3.0314
4.4354	3.0176	4.556	3.1403	4.23	3.4347	3.8728	3.2244
2.3934	3.7639	3.3052	2.8766	3.6846	3.3586	1.4892	3.9338
3.0547	3.5181	2.9874	3.6023	3.6478	5.8606	3.2267	2.5143
3.0201	2.161	3.364	5.0357	3.2447	3.7265	4.0629	3.1909
4.0399	2.3976	3.5593	4.2985	3.7912	4.275	2.2972	3.9526
3.929	3.6289	3.0094	3.9902	3.4972	3.7496	3.6223	2.7185
3.1784	2.9227	3.9111	3.2507	3.6867	3.1866	5.6334	4.8736
4.129	4.4854						
ABHD8	1.666	2.6968	3.0782	1.6078	3.388	1.5622	1.7504
1.9463	1.7871	1.6894	2.6823	2.4699	2.4524	1.2977	1.8371
2.6763	2.5414	1.1997	1.6084	3.0371	2.6063	2.82	1.1753
2.0335	1.8354	1.2586	2.6292	2.8023	1.4603	3.1562	3.9005
2.7236	2.2758	3.1864	1.8038	1.4799	2.775	2.6986	3.0893
1.219	3.1723	3.1211	1.6692	1.9116	1.4032	0.545	0.974
2.1035	3.1586	1.2028	1.4277	1.7126	1.412	2.51	3.8602
0.5271	2.2986	2.3146	3.1655	2.6973	1.5858	3.0995	2.2116
2.2088	0.6154	3.7219	2.6818	1.863	2.7202	1.9471	2.3972
2.581	1.4175	2.8256	1.4043	2.943	2.4072	1.8571	2.2548
2.5298	2.6605	2.9056	2.0924	2.227	3.4321	2.4247	2.0097
1.7504	1.9833						
ABI1	4.1037	3.1607	3.5595	3.9388	3.5554	3.7844	4.1144
3.088	4.594	4.8909	3.7176	4.0551	4.4913	4.9011	3.6303
3.7697	4.6091	4.2006	4.0709	3.6022	4.3272	3.1392	3.533
4.2305	3.6686	3.528	4.5839	3.9696	3.3626	2.9973	4.3243
4.903	4.4323	4.925	4.0197	4.7933	4.1849	3.7869	3.1955
3.8015	4.5586	4.0946	4.0946	4.5664	3.9172	3.3717	3.6826
3.5451	3.1175	4.8323	4.5634	3.3265	4.4401	3.6571	4.7889
3.8532	4.4118	3.1009	3.8946	3.3422	3.6597	3.5283	3.4454
4.5268	5.2489	4.7656	5.3943	4.5508	4.1983	3.677	4.58
3.8629	3.5446	3.6394	3.72	4.0415	4.4124	3.5437	4.2676
4.5519	4.1312	3.8651	4.6591	3.0257	3.5805	3.8983	5.1819
4.6514	4.9413						
ABI2	4.1869	3.952	3.9758	3.9862	3.5426	4.5603	3.8131
4.695	3.968	3.9786	4.2762	3.9886	4.2443	3.9689	4.5091
3.8173	4.3611	4.237	3.8946	3.4084	3.6508	3.913	4.3633
3.9886	5.2168	4.3602	4.7724	4.2783	3.8634	4.7167	4.6695
3.798	3.933	4.1146	4.2183	4.1703	3.7029	3.9657	3.9493
4.0459	3.9834	3.9029	5.0159	3.7215	4.9894	4.005	3.9117
4.78	4.7665	4.1294	4.5195	3.8135	4.9171	4.0061	4.6764
3.3341	3.6319	3.9952	4.0008	4.1894	3.7001	3.6817	4.024
3.7956	4.8878	3.9871	4.162	3.1971	4.0595	4.8772	4.7238
4.4298	4.1754	4.2976	4.3071	4.2518	3.7487	4.3384	4.151
3.801	4.736	3.9422	3.4803	4.6361	4.4428	4.2355	3.3759

3.4404	3.8143							
ABI3	0.1206	0.2065	0	0.2999	0.076	0	0	0
	0	0	0	0.0311	0.0409	0.1452	0	0
	0.2223	0	0	0	0	0.2324	0	0
	0.0359	0.2587	0	0.116	0	0	0.0407	
0.1062	0.0693	0.0646	0	0.0535	0.0323	0.0775	0.0495	0
	0	0	0.0959	0.0679	0	0	0	0
	0	0	0	0	0	0.0221	0.0725	
0.0394	1.8611	0	0.018	0	0.0135	0.0229	0	
0.0443	0	0	0.0251	0	0.0187	0	0	
0.0658	0	0.0459	0	0	0.0171	0.0119	0.0022	0
	0	0.0166	0.0211	0.0472	0.0637	0	0	0
	0							
ABI3BP	0.0924	0.1398	0.3371	0.3116	0.2013	0.1923	0.5181	
0.1892	0.1714	0.2294	0.2183	0.1411	2.3534	0.7563	0.178	
0.5181	2.2471	0.228	0.1625	0.1797	0.2548	0.2009	0.9466	
0.1512	0.1426	4.8154	2.6797	0.3687	0.1548	0.2033	0.0883	
0.2644	0.4107	0.5517	1.6447	0.1112	0.1952	0.1286	0.082	
0.4871	0.2324	0.4044	0.2009	0.5757	0.4443	1.1507	0.2616	
0.1199	3.2399	8.8467	0.519	0.8104	0.4289	0	0.092	
0.0901	2.7446	0.0524	0.1352	0.0126	2.1262	0	0	
0.2905	0.0052	6.4152	0.0895	0.0185	0.0228	0.4616	2.5432	
0.0076	0.3189	0.2082	2.0921	0	0.4368	1.4075	0.0308	
0.1858	0.0368	3.7255	0	0.6027	0.3131	0.554	0.0776	0
	0.1648							
ABL1	3.7863	3.4976	3.9038	3.5545	4.3547	4.2969	3.7974	
3.5514	3.8167	3.5074	4.7205	4.4324	5.456	3.65	3.7875	
3.2964	5.9422	3.63	2.8973	4.6739	4.7572	3.226	3.7795	
3.57	4.4239	4.9999	3.6492	4.2185	3.88	3.0928	4.2151	
3.8603	3.3937	4.4698	3.9662	3.2878	4.6015	5.3975	5.5878	
3.4435	4.7659	3.943	4.0081	4.29	4.3198	4.0399	3.4992	
3.4559	4.5361	4.497	3.9998	3.8936	4.1734	4.2634	4.4957	
3.6869	5.3998	4.2047	3.4287	4.6412	4.1149	3.8401	3.8177	
4.6205	5.3563	5.5164	3.0506	4.9848	4.4458	4.624	3.8162	
4.1622	3.4231	3.7878	4.1521	3.3357	3.7414	3.6474	3.6933	
4.8132	3.1304	4.3828	3.3979	5.4229	4.3026	4.3685	5.1228	
4.5191	4.666							
ABL2	4.465	3.2341	4.2418	3.7629	4.0178	4.5067	5.0088	
4.2954	3.6299	4.102	2.7068	4.05	4.4307	4.1118	4.9672	
4.2864	4.2396	4.4922	3.719	3.9803	4.7534	3.5374	3.5551	
3.7594	3.025	4.7723	3.955	3.4465	3.5413	2.7072	2.1577	
3.8455	3.1277	3.5246	4.4287	3.7914	5.2727	3.5078	4.3232	
3.345	4.009	2.3651	3.4273	4.6837	4.8267	3.3158	4.4754	
3.5949	2.6299	4.3897	3.6659	3.8079	3.8416	3.5322	2.9595	
3.0715	4.5507	2.353	4.0229	2.6786	3.6509	3.7608	3.5131	
3.8505	3.352	3.8573	4.1444	5.0098	4.3461	3.8889	3.8936	
3.8669	4.2348	2.8462	5.0538	3.4818	4.1653	4.8607	3.6201	
3.006	2.9965	4.4096	5.0566	4.5435	3.6675	3.7727	4.4149	
4.4196	4.317							
ABLIM1	3.1769	4.3896	3.8074	3.5453	2.2658	1.2469	3.4909	

2.605	3.2099	3.5085	3.164	3.6235	3.0802	2.6746	0.3697	
1.5015	0.5025	2.9047	2.5188	1.4381	2.7529	0.5776	1.4375	
1.1823	1.4673	1.0785	2.5043	5.5623	0.9942	3.0742	5.0748	
2.2245	1.8334	3.1671	1.3001	4.1802	2.09	2.7668	3.6584	
2.4779	1.0664	3.998	2.1726	1.6818	1.5318	1.4556	4.485	
1.5546	1.3036	0.8337	1.7168	1.3516	2.6878	2.4554	4.9103	
2.9069	2.968	6.3129	2.2008	4.4845	1.5603	1.5224	4.2903	
1.6685	3.9245	3.7011	1.4941	2.2916	2.2992	1.7688	1.3509	
1.3509	2.0089	2.902	0.6884	4.6688	3.18	1.5141	1.4215	
2.3814	2.0834	1.1585	4.1882	1.3861	1.2333	1.7339	1.4287	
0.8579	1.1373							
ABLIM2	0.3714	0.0062	1.5658	0.2012	0.0965	0	0.4774	0
	0.2554	0.4951	0.1777	0.0081	0.2679	0	0.0828	
0.4154	0.3203	0.3855	0	0.8234	0	0.0479	0.0225	
0.2136	0.1355	0.2254	0	0.6163	0	1.9352	0.6015	
0.4759	0	0.0189	0	0.1177	0.0844	0.6958	0.2877	0
	0.0569	0	0.6893	0	0.0719	0.2302	0.0501	
0.3282	0.1153	0.1131	0.0675	0.0251	0	0.1542	0.5129	
0.466	0.1676	1.9897	0.099	1.609	0.0331	0.1406	0.1428	0
	0.3403	0.0485	0.2811	0.1934	0.4289	0.1389	0.042	0
	0.1159	0.0624	0	2.009	0.1085	0.2328	0.0438	
0.1068	0	0	0.2392	0.054	0.0661	0	0.4094	
0.3284	0.3255							
ABLIM3	2.2261	0.8215	1.0534	1.0514	2.3055	4.5834	2.5643	
1.052	2.8832	1.5919	2.4553	3.4948	5.7732	3.9304	4.8415	
3.5647	2.3333	2.4515	2.0321	5.43	2.5666	2.3301	3.6211	
3.4363	0.7144	4.1291	1.5561	0.6304	4.5123	0.8221	1.063	
3.8888	4.2296	4.0156	5.0178	0.87	1.6789	2.4787	0.9626	
3.8058	1.9833	4.9881	4.0228	2.0513	3.1334	5.0185	0.2957	
1.546	3.8698	3.2189	2.9047	2.3745	2.1524	1.3099	0.3866	
0.7194	5.1999	0.6937	2.1896	0.5593	3.3304	3.99	1.2277	
4.2229	1.2283	4.0441	0	3.0809	1.0811	3.6745	4.3916	
4.2372	1.8026	4.1647	3.7392	2.072	2.6257	3.2683	3.4165	
1.8387	0.9423	3.2037	4.0113	2.306	4.0055	2.2334	4.0745	
2.1786	2.011							
ABO	0	0	0	0	0	0	0	0
	0	0	0	0	0	0	0	0
	0	0	0	0	0	0	0	0
	0	0	0	0	0	0	0	0
	0	0	0	0	0	0	0	0
	0	0.8001	0	0	0	0.0415	0	0
	0	0	0	0	0	0	0.0505	0
	0	0.303	0	0.4455	0	0	0	0
	0	0.106	0	0	0	0	0	0
	0	0	0	0	0	0	0	
0.08	0	0	0	0	0	0	0	0
	0							
ABR	4.6316	5.031	6.241	4.542	5.3167	4.8982	5.3105	
5.2804	4.1116	5.3846	4.8653	4.1853	4.6661	4.338	4.0287	
5.3606	4.5379	4.7282	4.0343	2.7587	5.4895	4.4039	4.2495	

4.4624	4.0395	4.6079	3.577	5.8272	5.0996	4.7683	4.0379	
2.2095	5.4041	5.8911	4.871	4.5375	3.9437	6.6077	5.3577	
4.4536	6.002	4.7403	5.0668	4.9996	4.4383	4.0736	5.2079	
3.8925	4.01	3.7928	5.2604	3.8012	4.8496	4.2705	3.6775	
4.466	3.8652	3.8798	4.9416	4.0986	4.656	3.8297	3.552	
6.9776	6.3705	5.0361	4.3814	6.1815	4.4003	5.4184	5.6481	
3.6464	4.9243	4.4096	5.3546	5.3096	5.1498	4.0751	5.3914	
4.686	5.0414	3.6325	4.3481	4.6823	4.3601	4.5486	6.0129	
5.9921	6.4608							
ABRA	0	0	0	0.0272	0	0.0253	0	0
	0.0093	0.0247	0	0	0.007	0	0.0137	0
	0	0	0	0	0	0	0	0
	0	0	0.0146	0	0.0135	0	0	0
	0.0166	0	0	0	0	0.2368	0	
0.0209	0.0104	0.0061	0.0237	0	0.0143	0.0128	0.066	
0.0185	0	0.0466	0	0	0	0.0067	0	0
	0	0	0.0802	0	0	0	0	0
	0.0267	0	0	0	0	0.1361	0.0113	0
	0	0	0	0	0.0197	0	0	0
	0	0	0	0	0	0.1002	0	0
	0							
ABRACL	5.7373	5.7071	6.5284	5.7254	5.2705	5.9527	6.1737	
5.4142	6.5593	5.7162	5.7817	5.232	4.9894	6.8209	5.6329	
5.5268	5.6857	6.2612	6.4845	4.6939	7.1315	5.5286	5.8823	
5.4118	3.8153	3.8074	5.2283	6.1118	5.8014	5.6083	5.8705	
5.9447	5.8941	5.5414	5.3197	6.3424	4.7911	4.484	4.0495	
5.2095	5.9014	5.2047	4.9857	5.1049	4.871	5.1918	6.3334	
6.184	5.6136	5.7071	6.3694	4.9169	6.1463	5.3521	6.2764	
5.8152	5.9662	5.78	6.5918	6.236	5.1584	6.3975	5.6086	
5.9909	4.7312	3.0786	5.4696	5.6475	6.6404	4.9921	5.9302	
6.3916	6.0342	4.5533	5.0686	6.0926	6.7596	4.5658	4.7361	
6.0076	6.1541	5.0796	6.4055	5.2908	5.5623	6.6875	6.433	
6.9703	6.3438							
ABT1	3.6039	4.0361	4.2489	4.0156	3.7691	3.138	2.6552	
4.4151	3.0353	3.718	3.2992	3.7849	2.5405	3.358	3.5357	
3.312	4.4572	3.8074	3.8635	4.0079	4.1667	4.867	3.3162	
3.9661	3.5208	3.9919	3.5724	3.7503	3.5434	3.4123	3.7703	
4.2385	3.4756	2.9155	2.9511	3.8133	4.4916	4.3004	3.99	
3.0579	3.19	3.1113	2.9152	3.2815	3.2558	4.3551	3.5255	
3.3876	3.2471	3.8153	3.5058	3.7309	3.7802	4.1703	4.31	
4.0175	3.7336	4.0623	3.1472	4.0558	3.589	4.3482	3.6604	
3.5305	3.8852	2.183	4.7196	3.3371	3.5258	3.671	3.5435	
3.6326	3.5576	4.1115	2.8892	3.7549	4.547	4.0029	3.2888	
3.2866	3.3101	3.5719	4.5529	3.1332	4.1561	3.8279	3.0641	
3.3865	3.1964							
ABTB1	1.587	1.9001	1.7436	0.9955	2.9219	1.7558	1.751	
1.7877	2.6647	2.3684	3.2009	1.1744	3.0438	2.1043	2.4518	
2.9146	2.7784	2.2362	1.6681	2.0031	2.7315	2.8147	2.7878	
1.8912	1.6527	1.5213	3.2577	2.5064	2.9672	2.6272	1.6466	
3.6156	3.3233	3.7663	2.1224	1.509	1.7763	3.4161	3.0297	

1.6834	3.3165	2.1574	3.0223	2.5133	1.619	2.1309	0.603
3.1233	4.4221	2.0791	2.3103	1.7235	2.2846	3.0583	0.9668
1.5017	3.2942	0.9154	2.0545	1.548	3.2515	2.1812	0.9388
2.2891	2.0494	3.7294	2.3696	3.0898	2.2481	2.5135	1.9993
3.9063	1.6309	3.4958	2.2201	2.9923	2.3555	2.5898	2.4947
3.6826	3.037	2.2649	2.9181	2.8728	3.2927	2.4852	3.0294
2.5474	3.7897						
ABTB2	1.7236	1.5907	1.1108	2.5952	2.9783	3.386	2.7314
3.5899	2.8131	1.8683	2.3009	3.0739	3.6182	2.6067	2.3469
3.5252	1.6136	2.1819	2.6051	3.0768	2.0502	3.5059	3.1696
3.1577	3.0662	2.4144	3.1329	1.6038	3.7962	1.4106	1.2767
3.3485	3.2911	1.8339	2.7542	1.689	2.0372	2.6354	3.2171
2.7888	2.9349	1.2415	3.4632	3.4458	1.7151	2.7669	1.988
3.5662	4.45	2.0837	3.1371	2.5996	3.7721	3.4376	1.8094
2.9141	3.0632	2.9453	3.2093	1.6316	2.2212	1.8014	3.569
2.3529	2.27	3.2228	2.5227	2.1752	1.2131	3.599	2.3928
3.2949	3.0844	3.9466	3.2299	2.5144	1.2072	1.8646	2.7317
3.476	2.125	2.6267	4.1077	2.9882	3.3521	3.2254	2.0772
2.2545	1.8485						
ACAA1	3.8503	4.638	4.7271	4.3022	4.1143	5.0659	4.3246
5.1706	4.3977	5.259	5.0178	4.415	4.3736	4.4586	4.265
5.5793	4.2106	4.3	4.9363	5.3913	5.1216	4.3886	4.7188
4.9653	3.9078	4.1996	3.7904	4.2323	5.1602	4.9266	4.5302
4.6447	4.4137	4.0863	4.8054	4.6301	4.3171	5.3613	5.353
4.3777	4.5856	4.7179	3.981	4.4815	4.9601	3.4618	4.2071
4.3073	4.9061	3.3591	4.164	3.8081	4.1221	4.3025	3.5568
3.8412	3.9817	4.5028	5.31	4.5329	4.7709	4.692	4.7416
4.5082	3.883	4.5078	5.0889	4.5283	5.1416	4.3706	4.9249
4.2041	5.2964	4.5783	5.2908	3.7763	4.2404	5.4377	3.9511
4.8099	4.2824	4.4772	4.4243	4.7788	4.7451	4.6566	4.9595
4.8263	4.9218						
ACAA2	4.3684	4.6895	3.8681	5.1249	3.5825	3.3085	4.9807
4.0231	6.1356	5.755	4.7061	4.8066	3.6156	4.5976	4.7743
4.8817	4.0277	4.8713	5.7287	4.2579	3.9811	4.5062	4.2222
4.6097	4.9443	4.5646	5.2411	0.5788	3.6429	5.6597	5.2747
4.555	4.69	5.5961	3.7043	5.5646	3.9675	4.3684	3.4359
4.6923	5.1834	5.4853	4.9197	5.6485	3.772	6.0329	2.5085
4.2968	3.9909	3.4864	4.3455	5.3328	4.4736	4.7674	5.1367
5.0199	1.2443	4.4986	4.5652	4.3154	3.8663	4.2195	3.3292
4.2062	5.281	5.1817	5.8233	5.247	2.5976	4.4642	4.9702
5.3016	5.5103	4.1727	4.9666	4.9532	1.8908	4.4813	5.3688
4.9491	5.5352	4.9379	4.1587	4.108	5.0192	4.6924	5.1518
5.601	5.5581						
ACACA	4.5262	3.8179	3.6654	4.023	3.2358	3.8967	2.3962
4.1259	4.4812	4.3023	3.8959	4.0514	3.2956	4.1532	3.7918
3.4884	4.2475	4.3129	4.0676	3.6106	3.9183	3.5577	3.4699
3.4598	4.1152	3.6782	3.9	4.1785	4.0942	3.8484	4.3738
3.9506	3.4854	3.5511	4.4935	4.0847	3.0276	3.9045	3.4903
4.9755	4.2148	4.3795	3.5625	3.5237	4.1876	4.054	4.7979
4.5061	2.4869	4.4283	4.4465	3.5408	3.8585	3.8759	3.7671

5.0421	3.9003	4.1036	4.2771	3.938	3.8568	4.2923	3.9983
4.2625	4.3958	3.03	2.6063	3.8464	4.3033	3.7357	4.5332
3.6583	3.9313	3.5863	3.9816	4.678	4.0906	4.3662	4.2146
3.752	3.4074	3.8556	3.3973	3.4496	3.475	3.6131	3.9464
4.5571	4.4612						
ACACB	1.6372	2.525	1.7234	0.8882	0.9046	1.6434	1.3246
1.0075	2.6825	1.9878	1.9184	0.33	0.4349	0.552	0.214
1.7457	0.4493	2.417	2.3854	1.703	1.6233	0.4873	0.7423
1.3632	0.8754	0.2964	0.9515	1.2632	0.7595	3.0134	1.857
0.9365	1.716	1.9621	1.4269	1.4147	0.9618	1.5309	0.4674
1.6076	2.0991	0.9053	0.8266	1.4868	0.9858	0.2119	1.1956
1.6962	0.7733	1.2121	1.2749	1.0168	1.4862	1.2979	2.0717
1.6202	0.5978	1.189	2.6302	1.1525	1.0124	2.2484	0.7229
1.6881	2.258	1.3893	2.1748	1.1121	2.0002	0.355	0.6966
1.2121	2.032	0.2742	1.0758	1.2144	1.6136	1.7327	2.4363
1.1451	1.8443	0.1245	0.6436	1.356	0.1855	1.7749	1.892
1.7218	2.6659						
ACAD10	3.352	2.7732	2.4708	2.8259	2.8525	2.0931	2.3387
2.5349	3.4037	3.4817	3.2845	2.0276	1.7937	2.8813	1.7926
1.7087	3.1041	2.993	2.853	2.4862	2.1528	2.6206	2.3947
2.8833	2.1893	2.1068	3.68	2.5485	2.7615	3.8124	2.6942
2.7485	2.4686	3.1695	2.8867	2.903	2.9034	4.1349	2.9376
2.9644	3.319	2.5061	2.3737	3.0894	2.3728	2.5436	3.4349
2.9816	2.0077	2.2894	2.9507	2.3873	2.9135	2.7716	2.3921
3.1353	2.7925	2.4115	2.8305	1.9639	3.1493	3.5465	2.0567
2.3764	3.6194	2.3115	3.8533	3.4132	2.4623	2.4518	2.401
2.3545	2.708	2.4103	1.9996	2.213	2.6679	3.1249	3.6076
3.0146	2.4565	2.6697	2.8084	3.0901	1.7549	2.5964	3.4716
3.0617	3.6051						
ACAD11	3.5084	2.5348	2.369	2.3147	2.2726	2.3545	2.0423
2.14	2.753	2.3383	3.6228	3.3721	3.2119	2.967	2.6122
2.0563	4.2021	2.6962	2.2081	2.2185	1.7143	1.7524	2.3141
1.3835	1.7727	3.1641	3.5044	2.4262	2.4455	3.1419	3.087
2.3123	2.3846	3.6667	2.2817	2.4671	2.6019	2.754	3.061
4.2402	2.9852	3.2275	2.875	3.0663	3.6252	2.4239	3.8809
2.2368	3.7899	3.5036	3.4712	2.8079	3.4444	3.8219	2.1364
2.3205	4.011	1.1429	1.9485	1.6736	2.6418	2.044	1.859
2.4993	2.7775	3.5632	3.0495	3.2797	2.5533	1.7354	3.1087
2.6941	2.0376	2.9562	2.1813	2.1927	2.8586	2.374	2.7943
2.7698	2.7474	3.5933	1.9707	2.9491	2.0935	3.0208	2.8556
2.4945	2.3795						
ACAD8	3.7488	3.4026	3.2618	3.436	3.3908	2.6964	3.9491
2.5214	3.9017	3.0667	3.1406	3.0059	3.5363	3.5352	2.7085
3.7695	3.6833	3.2746	3.0938	3.5396	2.9276	3.6254	3.7968
2.9352	3.2204	3.0719	3.6303	2.4012	3.7607	3.6516	3.8967
2.496	3.287	2.8881	3.3438	3.1188	2.5404	3.1221	3.2168
3.4975	3.4026	3.6785	3.1693	3.891	3.8509	3.3486	2.485
3.9627	3.6373	4.0293	2.9837	3.5281	3.1685	4.2895	2.855
3.4186	2.8655	3.2412	3.1186	2.9803	3.1636	3.8117	2.8927
3.5521	2.0594	3.4815	2.9779	3.5147	2.6547	3.2421	2.8446

2.8294	3.9613	3.296	3.4197	3.5259	2.6137	2.7361	3.1903	
3.2425	3.1413	3.5612	3.42	2.8375	3.6712	3.5285	3.5864	
3.9785	3.9781							
ACAD9	4.2504	4.3403	4.2715	3.7287	3.8315	3.3325	2.9736	
4.3065	4.4984	4.766	4.3235	3.7345	4.417	4.2437	4.3194	
3.071	4.4579	3.7614	4.7817	3.6966	3.8417	3.5282	3.4921	
3.3381	3.5167	3.3701	3.5454	3.681	4.1654	3.5607	3.8502	
3.8817	4.5273	4.5659	3.944	4.3847	2.8376	4.7103	4.1486	
4.6758	3.7737	3.6558	4.2522	4.0608	4.4814	3.8319	4.3279	
4.2601	3.6079	4.3318	4.4421	3.66	3.8541	4.0425	3.7235	
4.3752	3.9621	3.6363	3.239	3.5129	4.0989	4.3413	3.5559	
4.5963	3.7297	3.5376	4.0868	3.7803	4.0055	3.7414	4.0522	
4.7717	4.399	3.99	2.8349	3.4161	4.2594	3.71	4.2461	
4.9046	4.5696	3.0462	3.5948	4.3315	4.1793	4.1377	3.5875	
4.3012	4.151							
ACADL	0	0	0	0.1707	0.1797	0	0	
0.1003	0	0.3968	0.1256	0.0527	0.0745	0.3432	0.0523	0
	0	0	0.2155	0.0516	0	0.0898	0.0427	
0.055	0.1378	0	0.7094	0.3171	0	0.1886	0.6874	0
	0.0397	0	0.0705	0.3554	0.0527	0.0456	0.0919	
0.0309	0	0.3153	0	2.1201	0.4117	0.4912	0.4299	
0.4403	0.1574	0.0722	0	0.0688	0.0575	0	0.7991	
0.0192	0.188	0.0279	0.055	0.4607	0.3742	0.0255	0	
0.737	0.2807	0.9051	0	0.0093	0.6644	0.0137	1.0013	
0.8037	0.0831	0	0.3007	0.4244	0.0071	0	0.0097	
0.1822	0.0149	0.1836	0.0076	0.0977	0	0	0.3858	
0.6455	0.2384							
ACADM	5.5692	4.8001	5.1415	5.8186	4.3297	5.6292	5.5294	
4.2511	5.6192	5.4829	4.2613	5.4044	4.772	6.0149	4.0312	
4.94	4.6446	5.6048	6.0911	5.7983	5.4928	4.2779	4.1774	
5.0875	4.4636	4.4066	3.4214	5.1869	4.5952	5.8972	5.4044	
4.6231	5.6754	5.1208	4.099	5.912	4.568	4.7539	4.3796	
4.155	5.2961	5.4422	4.3774	5.8195	3.4047	4.8895	4.7464	
5.2242	4.4792	5.1402	5.2389	4.6106	5.8816	4.98	4.5675	
4.7103	4.3057	5.3126	5.5032	5.357	4.4032	5.6863	4.5456	
5.6035	4.3769	3.8269	5.0795	3.2975	4.7604	5.0893	5.6936	
5.4582	5.0323	4.5864	4.727	4.9324	5.7596	4.2407	5.4659	
5.6735	5.3187	4.637	3.8621	3.9783	4.7062	6.0092	5.0755	
5.6473	5.2307							
ACADS	2.9242	2.7502	2.6821	1.299	1.7131	2.1222	2.2001	
2.2432	2.4635	2.1232	2.4352	1.0078	1.4037	1.9421	1.2161	
2.7239	2.2857	2.1125	1.6977	2.1347	2.9529	1.5707	1.1636	
1.959	1.9079	0.7749	2.1744	3.0354	1.6191	3.2891	2.4758	
2.3848	2.0251	3.5369	1.917	1.461	2.3972	3.9449	2.3306	
1.6789	3.5651	2.6855	1.4317	3.3612	1.0179	0.9734	1.73	
1.8728	2.4189	1.2403	1.9936	1.4053	1.647	1.9584	2.1068	
2.5289	2.0756	2.3525	2.5208	1.7281	2.1591	2.6584	1.0066	
2.3881	2.8991	1.433	2.6689	2.8427	1.7861	1.2036	1.667	
1.4705	2.1829	0.9017	1.8942	4.1492	2.9734	1.6177	3.5117	
4.3432	2.4142	1.7543	2.1725	2.3738	1.4833	2.6996	3.2495	

3.9897	4.235							
ACADSB	2.8116	2.9043	2.6053	2.5268	1.9973	3.1554	2.3095	
2.5331	3.0453	2.8724	2.0187	2.3065	1.6167	2.8395	2.2034	
2.7972	1.6762	2.9118	2.6673	3.2451	2.8728	1.424	2.2193	
2.3079	1.2804	1.2214	2.5499	2.2787	1.711	2.1025	2.8546	
2.4914	2.3486	2.0951	1.8374	3.2988	2.1198	2.3889	1.9715	
2.4109	3.2608	1.9923	1.2207	1.6671	1.8186	1.149	2.5995	
2.7194	2.6113	2.0421	2.1046	1.7381	2.483	1.9835	3.3228	
1.7486	1.2284	2.281	2.9916	1.9529	1.8756	3.0215	2.6169	
2.6663	2.1207	2.0725	2.7037	2.7517	1.8807	1.9239	2.6495	
1.9812	2.1192	1.7469	2.1789	2.8598	2.7673	3.2815	1.9235	
1.928	2.0499	3.046	1.6037	2.2635	1.2979	2.2947	2.9661	
2.6804	2.9521							
ACADVL	6.6917	5.5329	4.9212	6.5364	7.6611	5.8493	7.0395	
6.5046	6.7945	5.7866	5.819	6.0373	6.1315	5.3965	5.5755	
6.5779	6.046	6.5347	5.1192	5.8896	5.8974	6.0423	7.4337	
5.9553	6.381	6.8269	6.9123	6.0875	6.628	6.271	7.7236	
4.9675	5.6193	6.4078	6.9804	5.8083	5.5385	6.9538	5.8095	
6.2575	7.1966	8.1649	6.755	6.6103	5.9924	6.7457	4.3068	
6.3059	6.0956	5.2909	6.5555	6.6699	6.1513	5.1334	6.5941	
4.7542	6.3041	4.9341	5.9328	4.5618	6.4008	5.7416	6.0659	
6.4705	5.6688	7.443	6.9332	6.8163	5.1247	6.599	6.5941	
6.6456	5.9378	7.5121	6.5595	5.9279	5.4257	6.6971	6.7072	
6.7056	6.2235	6.9708	6.1292	6.3551	6.0069	6.4422	5.8929	
6.0246	6.2488							
ACAN	0.1053	0	0.0719	0.0847	1.0155	0.6379	0.1206	
0.27	0.2982	0.2464	0.1717	0.1274	0.1012	0.8741	0.22	
0.5808	0.0662	0.0871	0.1185	0.1065	0.1114	5.5184	0.6669	
4.0727	4.4481	0.0407	2.4582	0	2.755	0	0.0878	
1.2957	2.3236	0.3406	0.6131	0.1562	0.0712	0	0.4215	
1.1451	0.0611	0.0373	4.9065	3.9017	0.0396	0	0.0966	
0.1342	1.2473	0.3879	0.1336	1.9996	0.1695	0.0812	0	
0.1238	0	2.4426	4.5531	1.057	1.4481	0.901	0.6803	
0.2115	0.3576	0	0.195	0	0	1.4208	1.7886	
1.9955	0.1558	2.921	5.6386	0	0.0191	0.3857	0	
0.3336	0	0.3723	0	1.7686	1.5116	0.1915	0	0
	0							
ACAP1	0	0.0842	0.0603	0.5206	0.1906	0.1876	0.0886	
0.0879	0.0835	0	0.0614	0.0507	0.1644	0	0	
0.3823	0.2937	0.2272	0	0.2764	0.2999	0.1473	0.0914	
0.0915	0.0585	0.1384	0.3428	0.0724	0.1473	0.1775	0.1917	
0.0593	0.0363	0.2757	0.1169	0	0.0595	0.3009	0.1281	0
	0.3519	0.1558	0.0587	0.1556	0.0794	0	0.0639	
0.0835	0.2089	0.2231	0.0971	0.119	0.1536	0.1322	0.2671	0
	1.7165	0.0602	0.0362	0.0171	0.0651	0.0385	0.0642	
0.1128	0.0387	0.3865	0.2899	0.1441	0.1128	0.2064	0.0737	
0.1412	0.0905	0.0434	0.2602	0.0816	0.1341	0.1156	0.0182	
0.2641	0.0564	0.0609	0.2119	0.2056	0.044	0.2676	0.2014	0
	0.1082							
ACAP2	3.9712	3.6686	3.8648	3.6709	2.883	3.9765	4.649	

3.9272	4.8323	4.1859	4.4983	4.7162	3.6382	4.7999	4.6123
3.9892	4.2081	4.6769	4.6731	3.2419	3.8719	3.7174	4.0139
4.4835	3.2518	3.3174	4.4161	3.8236	3.9397	4.1241	3.7513
4.1241	4.3223	4.663	3.9319	4.263	3.559	4.0491	3.921
4.3477	4.5173	4.0059	4.0487	4.0579	3.4933	4.1086	4.2943
4.3628	3.3187	4.2807	4.6825	3.9878	4.6592	4.8121	3.9617
3.8774	5.0708	3.6402	3.649	3.9755	4.2816	4.0769	3.8549
4.8377	3.9314	3.7837	4.9309	3.9333	4.4594	3.8443	3.3904
4.4196	4.0297	4.3804	3.4232	4.0393	4.033	4.0905	4.2679
4.0339	4.1366	3.2903	3.6796	4.4874	3.5592	4.0428	4.2143
3.8179	4.0423						
ACAP3	3.3676	3.6572	5.2207	2.7459	3.4546	3.5472	2.6438
3.948	3.0289	2.907	3.2813	2.2865	3.4833	2.4956	2.6914
3.6398	2.4861	2.5627	2.9456	3.5757	5.0412	3.1766	3.6716
3.8854	3.4735	3.5141	2.2649	3.5936	3.6583	3.579	3.791
4.1179	3.5398	4.4959	4.3701	2.2551	2.8454	4.1212	3.4036
2.8995	3.4599	3.6097	3.681	3.5639	2.9541	2.6802	2.595
3.1101	2.8736	2.0875	4.0223	2.3454	3.1421	3.3204	2.3423
3.5914	1.8397	3.6769	3.7964	3.9378	3.8542	2.8368	4.062
3.3346	4.058	3.1534	5.1314	4.4133	4.0599	3.3484	2.797
3.8589	3.3829	4.421	2.6559	4.4432	3.9857	3.46	2.9774
3.7114	2.829	3.037	3.6538	3.5381	3.2668	4.3086	3.4495
4.1704	4.2343						
ACAT1	5.6123	5.7772	5.3181	5.4774	4.9369	5.3644	5.0393
3.8988	5.9859	4.9901	5.0121	3.8428	4.6229	5.3738	4.4023
5.3384	4.8978	4.8147	4.9541	5.8408	4.3941	5.2102	5.401
4.3103	3.5407	3.736	5.0246	4.2189	5.441	5.9691	4.7802
3.9497	5.3238	5.2376	3.301	5.3652	3.7758	4.9593	5.0016
5.6249	4.5374	5.4988	4.8357	5.4546	4.7077	4.2816	5.1118
5.588	4.41	5.5115	5.5923	4.8583	5.3954	4.3845	5.0494
5.6642	4.3707	4.474	5.4244	4.3772	5.422	5.5048	4.8723
5.8954	5.7578	4.8206	4.8	5.4482	4.3121	4.2413	4.9087
5.2147	5.5515	4.1477	4.9768	5.6091	4.6623	4.3352	5.6453
5.5964	5.2453	5.9136	3.4898	4.4955	4.2265	5.5928	5.807
5.5066	5.9362						
ACAT2	3.2265	5.8887	6.1285	4.968	4.0611	5.8652	3.024
4.1146	4.2061	4.3645	4.3006	3.3883	4.3021	5.9849	5.7017
4.1954	4.5939	5.815	6.5913	0.4781	5.7561	4.3862	4.4276
3.6995	4.4549	3.7522	2.7731	3.8945	5.4041	4.7527	3.6265
4.484	5.276	3.5877	5.5656	5.0951	2.3656	0.4021	3.4599
4.5947	5.4311	4.983	4.4785	3.9089	6.0178	5.3894	7.0333
5.6348	2.8795	6.3881	6.4357	2.8014	6.5913	2.47	4.818
6.8049	7.0248	4.5279	5.8967	5.4715	4.2988	3.7259	4.959
5.1278	4.6512	1.8184	2.2833	2.6459	7.0688	3.7782	4.7787
4.896	5.7603	2.47	4.4165	6.7932	5.892	4.5374	3.9527
4.8394	5.0449	4.7601	5.0423	4.2182	4.7953	6.2874	7.0933
6.3256	6.2524						
ACBD3	4.2372	4.3405	4.0644	5.1021	4.9017	4.9493	5.6919
5.0581	4.3142	4.0438	4.6522	5.255	4.3383	4.4626	4.8868
5.6386	3.5288	5.7187	4.3603	4.741	4.7144	4.9422	4.9466

4.8241	4.7421	5.0465	6.2308	4.1429	4.7723	5.1211	4.6321
4.4194	4.5575	5.292	5.4401	4.5957	5.1665	4.5495	4.5146
4.8773	5.0184	5.0458	5.1391	4.4129	4.6223	4.7474	4.7176
5.1346	4.9174	4.2925	5.5234	5.0705	5.1472	4.0558	4.9227
4.6556	5.3534	4.2705	5.2542	4.4436	4.8849	4.5869	4.6636
4.9321	4.9656	4.9661	5.0025	5.2607	3.8492	4.8376	4.2501
4.9562	4.854	5.592	5.3016	4.5107	4.8348	5.8132	4.5253
3.9388	4.2294	4.8042	5.3043	5.7502	4.5227	4.7927	5.2058
4.7003	4.6864						
ACBD4	3.2325	1.9844	1.6984	1.2799	1.9209	1.4612	0.8779
1.7651	1.8063	1.5813	2.9443	2.6587	2.0587	1.2046	0.9548
2.1008	1.7468	2.0645	1.3721	1.9699	1.9883	1.0881	1.5617
1.4575	0.8693	0.7704	2.5762	2.3926	1.3073	1.9443	2.2184
2.6296	1.2586	3.2414	1.9164	1.2946	2.5647	2.5879	1.4617
1.1827	3.3416	2.5347	1.2276	1.7535	2.3345	0.6941	0.763
2.0077	2.6622	1.4642	1.8455	1.0545	1.0575	1.9513	1.7088
1.3818	2.0511	0.7428	1.7512	0.4729	1.5875	2.5687	1.5599
1.1249	1.4231	3.1778	2.9857	2.0678	1.2078	1.4945	1.8231
1.8454	1.5612	2.8139	2.035	2.2212	1.9546	1.6737	2.0089
2.2885	2.0642	2.6393	2.646	2.0678	0.3973	1.5832	2.4023
2.0971	2.5312						
ACBD5	3.9385	2.7025	3.8289	4.2098	3.6144	3.4162	3.5774
2.7237	3.1928	3.9998	3.4966	3.3117	3.5417	4.1369	2.6159
3.4329	3.3744	3.8501	3.5279	3.8106	4.1459	2.69	3.0647
3.9693	3.0195	2.9451	4.1165	4.0949	3.2133	3.4448	3.6193
3.921	3.2244	4.0178	3.5249	4.1146	3.3599	2.8013	2.4419
3.3073	3.696	3.7227	2.6922	3.7913	3.1491	3.2222	3.1171
2.7496	2.6774	3.6692	4.2147	3.6757	3.2709	2.4853	4.1461
3.5873	3.3021	2.9241	3.8855	2.7047	3.5554	3.7921	3.2028
4.0795	3.9544	3.8799	4.0384	4.3395	3.6939	2.6752	3.5332
3.5259	3.4942	3.0801	3.7481	4.0242	4.1078	3.4847	2.8259
3.3803	2.7389	3.7288	3.4393	2.9018	3.0651	3.2929	4.346
3.8426	4.2042						
ACBD6	5.3146	4.3242	4.5836	4.8771	4.6853	3.8563	4.5859
4.9079	4.9732	4.6151	4.7571	4.6442	4.559	4.6531	4.9472
4.4944	4.7689	5.3324	4.4902	4.776	5.0011	4.7093	5.0484
4.8733	5.1199	4.419	5.4608	3.9914	4.4511	5.0107	4.7024
4.4169	3.9005	4.4544	4.913	4.6332	5.1279	4.6837	5.1081
4.8339	5.7133	4.6758	4.9746	4.4396	4.7103	4.2198	5.4573
4.6102	4.5069	5.0554	5.0126	5.2282	4.7806	4.6764	5.0366
4.9747	5.0897	4.667	4.8019	4.6342	4.8183	4.3816	4.7795
4.207	4.7941	4.6508	4.599	4.6756	5.6347	4.7192	4.1472
4.6061	4.4792	3.9382	4.8079	4.1462	4.2189	5.5991	4.8615
4.6061	4.5793	4.7383	4.1131	5.3648	5.3205	4.8404	4.4934
4.7771	4.6705						
ACBD7	0.9242	1.2243	0.8764	1.1046	0.7016	0.6431	0.3349
0.2104	1.5196	0.3747	0.1493	0.5478	0.9371	0.8814	0.3146
0.756	0	0.5567	1.2281	1.137	0.3653	0.192	0.2675
0.78	0.4442	0.2542	0.6879	0.874	0.1743	0.569	1.6337
1.9969	1.5631	1.1237	0.2152	0.4761	0.1839	0.1754	0.1932

0.5621	0.847	0.4148	0.5239	0.441	0.5337	0.7232	0.5379	
0.553	0.6134	1.3643	0.9418	0.3695	0.8482	0.2843	1.1922	
0.6237	1.0616	0.5947	0.6428	0.4148	0.2316	1.5034	0.5876	
1.3582	0.454	0.1331	0.3468	0.1398	1.2996	0.1664	0.3745	
1.0231	0.2783	0.5458	0.4843	0.2043	0.8379	0.2347	0.7713	
0.8488	1.6588	0.4785	0.0897	0.5256	1.0808	1.1678	0.1553	
0.3835	0.4635							
ACCS	2.2665	2.2619	2.2072	1.2149	2.0168	2.1752	3.0902	
1.2654	2.5539	1.5552	3.112	1.7045	1.7122	1.5432	2.1144	
2.1483	2.5248	1.8227	3.0495	1.6828	2.0642	1.3431	2.4755	
2.2702	1.7641	1.78	1.5741	2.0642	1.8922	1.6624	2.6757	
2.8927	2.4589	2.3038	2.6559	1.5525	2.8914	2.2321	1.6402	
1.944	2.2219	2.8837	1.5907	3.8043	1.2358	1.2492	1.8076	
2.2432	3.2686	1.6269	3.2009	1.6457	2.7383	2.2692	2.9455	
1.5388	2.2686	0.9409	1.7451	0.4428	1.9075	1.5088	1.9934	
3.0371	2.0948	2.398	3.1903	2.0685	2.1417	1.6834	1.5872	
1.9719	1.4576	2.4798	2.8101	2.1185	2.0495	1.818	2.3586	
2.7463	2.2559	1.6987	2.4606	1.6279	0.7419	2.5707	2.1936	
2.4291	2.5093							
ACCSL	0.1989	0.4898	0.4174	0	0	0.0303	0.0259	0
	0.5286	0.5399	0.0566	0.0465	0	0.2529	0	
0.6902	0	0.3036	0.7113	0	0	0	0.0349	
0.2895	5.00E-04	0.0057	0	0.283	0.0106	0.0601	0	
0.2678	0.3478	0.33	0.1533	0.1284	0.0173	0.303	0.0059	0
	0.3138	0	0	0.2105	0	0	0.2419	0
	0.01	0	0.1797	0	0.084	0	0	
0.1085	0	0	0.1812	0	0.1076	0.0492	0.1217	
0.1714	0.1859	0	0	0.0559	0.2518	0	0.052	
0.1728	0.098	0.0406	0.131	0.3942	0.501	0.0586	0.3214	
0.4484	0.1937	0	0.0499	0.0623	0	0	0.7872	
0.3388	0.6096							
ACD	3.4488	3.3655	4.01	2.6779	4.1465	2.8857	1.5018	
3.1137	2.6885	2.9177	2.5041	2.4198	4.1601	2.9023	3.2968	
3.0506	3.3904	2.4898	2.7332	4.9814	4.0315	3.9721	3.1273	
3.6302	3.0586	3.3896	3.1868	3.2399	2.4417	3.0455	2.7972	
3.8507	3.2171	2.9139	3.1366	2.527	3.5669	2.8986	2.8699	
2.0694	3.3366	2.9208	3.5285	3.5765	2.9992	3.0396	2.4502	
2.9078	2.5925	2.7694	2.7586	2.5477	2.0683	2.8293	3.4353	
3.1949	3.5504	3.0974	3.2439	3.9624	3.8308	3.4732	2.9574	
3.6238	2.0161	2.2176	2.9048	3.0928	2.7281	3.9769	3.4647	
4.0009	3.3118	2.6977	2.434	3.6251	3.7641	2.8594	3.5098	
3.1533	3.2367	2.6395	2.8225	2.518	4.2773	3.2103	2.7283	
3.3683	3.2168							
ACE	0.2379	0.043	0.0296	0.4234	0.0395	0	0	0
	0.0024	0.2005	0.0532	0.1286	0.1192	0.3388	0.0577	
0.0174	0.1809	0.1738	0	0	0.1524	0.1883	0.0816	
0.0591	0.7028	0.1863	0.4651	0.037	0.9542	0.0773	0.4111	
0.0518	0.0369	0.0998	0.1376	0.1984	0.0676	0.0798	0.1815	
0.0716	0.1053	2.7546	0.3809	0.1567	0.5813	1.0992	0.3724	
0.1292	0.625	0	0.0741	2.0379	0.1721	0.1689	2.9924	

0.1982	0.1083	0.1298	0.2364	0.2467	0.1341	0.0929	0.0656	
0.2925	0.0551	0.9485	0.1429	0.1549	0.1897	0.6561	0.225	
0.0552	0.2508	0.1167	0.1013	0.0913	0.1206	0.0877	0.0983	
0.2162	0.0447	0.3039	0.1331	0.3278	0.2673	0.2143	0.4642	
0.6728	0.2037							
ACE2	0.0462	0.0403	0.0288	0	0.0394	0.1413	0.0379	
0.0344	0.0347	0.0207	0.0226	0	0	0.0393	0.0606	
0.3389	0.029	0.1017	0.0883	0.0413	0.0291	0.0049	0.0205	
0.035	0.0066	0.079	0.0234	0.0324	0.0477	0.0228	0.0091	
0.0359	0.0407	0.0216	0.2483	0.023	0	0.0462	0.0088	
0.0268	0	0.0044	0.0238	0.0259	0	0.0209	0.0233	0
	0.0456	0.0464	0	0.0163	0.0476	0.0379	0	
0.0897	0	0.012	0.0773	0.103	0.0291	0.074	0.1796	
0.0148	0	0.0127	0.2357	0	0	0.0306	0.0277	
0.054	0.0215	0.0137	0.0737	0	0.0284	0.0341	0.0682	
0.1579	0	0.0755	0.0507	0	0.0298	0	0.0225	0
	0							
ACER1	0	0	0	0	0	0	0	0
	0	0	0	0	0	0	0	0
	0	0	0	0	0.1223	0	0	0
	0	0	0	0	0	0	0	0
	0	0	0	0	0	0	0	0
	0	0	0	0	0	0	0	0
	0	0	0	0	0	0	0	0
	0	0	0	0	0	0	0	0
	0	0.1094	0	0	0.0322	0	0	0
	0	0	0	0	0	0	0	0
	0	0	0	0	0	0	0	0
	0							
ACER2	0.7972	0.3988	1.5272	0.4429	1.4581	0.3343	0.5726	
0.3727	0.6177	0.4678	0.5665	0.3667	0.1418	0.4928	0.3499	
1.327	2.2048	0.9428	0.3766	0.576	0.3509	0.1178	0.5997	
0.836	0.2567	0.4001	0.2556	1.1167	0.6437	0.3948	0.9023	
0.5386	0.2324	0.5665	0.5038	0.5954	0.4375	0.961	0.4718	
0.5731	0.7074	0.4566	0.458	0.4012	1.0773	0.8093	0.6525	
0.223	0.8728	0.5613	0.3943	1.1161	0.9735	0.1646	1.0781	
0.2339	1.208	0.1369	1.0096	0.2288	0.3608	1.0619	0.2293	
0.5784	0.9169	1.6472	0.8086	1.4777	0.3155	0.7374	0.5751	
0.5722	0.4706	0.3259	0.7681	0.6913	0.6758	1.0399	0.6099	
0.7078	0.5075	0.4956	1.2412	0.2378	0.3018	0.5985	0.7243	
0.479	0.7683							
ACER3	3.2204	3.286	3.9855	3.3991	2.8481	3.1909	3.3049	
2.9543	4.1693	3.4776	3.0945	3.8064	3.0134	3.4654	3.0096	
3.3651	3.1435	3.1104	3.273	3.2317	4.5472	4.0241	3.756	
2.8702	4.6858	2.9018	3.3205	3.203	3.7407	3.2273	3.0575	
2.1737	3.1935	2.733	3.5255	3.5117	2.8912	3.3563	3.4943	
3.1264	3.8862	4.0265	3.7473	2.6328	3.3844	4.0437	3.8341	
3.2988	4.3406	3.1854	3.1038	3.5796	3.6117	2.758	3.5563	
3.2978	4.2224	3.0263	3.8062	2.9897	2.4479	2.888	3.0625	
3.2274	3.1694	2.3289	3.0029	3.7336	3.4278	3.9229	4.0643	

2.9136	2.8685	3.5555	3.2413	3.7852	2.7477	3.4451	3.9995	
3.4343	3.9576	3.1954	4.2723	3.6153	3.5662	3.5601	3.3398	
3.3548	3.1415							
ACHE	0.1315	0.2664	0.1917	0	0.2916	2.2152	0.2212	
1.744	0.1113	0	0.3339	0.1468	0.9711	0.1892	0.4387	
0.6841	0.924	0.2888	0	1.1723	0.0884	0.1667	0.653	
0.1808	1.3342	0.6285	0.8035	0.8809	0.2785	0.1215	0.5456	
0.0632	0.1353	0.4805	1.8818	0.185	0.5044	1.7762	2.3553	
0.3759	0.0882	0	1.7047	0.2028	1.0283	0	0.2248	
0.2777	2.1801	0	1.0824	0	0.3035	0.0936	0.85	
0.1672	0	0.2009	0.014	0.2458	0.3607	0.2923	1.3814	
0.3661	2.5808	1.0956	3.2647	1.5834	0.0598	1.4202	1.4083	
0.3021	1.4433	0.0745	0.7618	0.2535	0.1875	0.3224	0.4757	
2.0435	0.2179	0.6215	0.0084	1.1133	0.5803	0.0959	0.9766	
0.9947	0.7392							
ACIN1	5.3833	5.4338	5.07	5.5059	5.2895	5.6184	5.5505	
5.3732	5.6677	5.5131	4.6809	5.1994	5.2528	5.3826	5.4213	
5.4142	5.3591	5.7557	5.4595	5.2583	5.3914	5.2077	5.2071	
5.2401	5.6063	5.6294	4.7311	5.5664	5.425	4.9686	6.2442	
5.0735	5.3683	4.9111	4.9924	5.2846	5.3724	5.2135	5.0236	
5.7601	5.013	5.1067	5.0316	5.2874	5.855	5.6117	5.1043	
5.3078	5.4293	5.2576	4.7486	5.166	5.5754	5.0889	5.9755	
5.461	5.2014	5.7229	4.8988	5.8496	5.2975	5.406	5.6646	
5.1426	5.5549	5.3601	5.4775	5.4036	5.2657	5.2625	5.0623	
5.4184	5.2464	5.0603	5.6479	4.9889	5.4431	4.7515	5.2866	
4.9755	5.5665	5.2014	5.2239	5.37	5.7812	5.2618	5.188	
5.2102	5.387							
ACKR2	0.3982	0.1212	0.2943	0.0479	0.0215	0.2222	0.0764	
0.0616	0.14	0.2634	0.1761	0.0484	0.0928	0.0899	0.0723	
0.3279	0.3621	0.1746	0.0674	0.0472	0.2265	0.0468	0.078	
0.1811	0.0928	0.1356	0.1696	0.1615	0.0786	0.1303	0.3049	
0.0968	0.0301	0.1551	0.2396	0.0545	0.3127	0.0483	0.0812	
0.0236	0.1126	0.092	0.0687	0.2295	0.1066	0.0712	0.1998	
0.2663	0.0443	0.1569	0.0756	0.1834	0.133	0.102	0.1707	
0.1974	0.2719	0.1067	0.1296	0.0848	0.0623	0.1287	0.1272	
0.0813	0.2009	0.2367	0.5609	0.0487	0.0987	0.2294	0.0461	
0.1914	0.0778	0.0347	0.1961	0.1661	0.1278	0.1546	0.0598	
0.1817	0.3116	0.149	0.221	0.1207	0.1127	0.0787	0.221	
0.2362	0.2003							
ACKR3	0.1832	0	0	0.1635	0.1895	0	0.1548	0
	0.1536	0	0.0921	0	0.6958	0	0.0973	0
	0	0.1511	0	0	0.158	0.2924	0.2975	
0.4115	0.5402	0	0.2367	0	0.591	0.174	0.1472	0
	0.1886	0.1223	0.4034	0	0.1267	0	0.3802	
0.1186	0	0.0794	0	0.1284	0.1827	0.1775	0	
0.2714	0.1597	0	0.117	0.1286	0	0.0336	0.0134	
0.0567	0.0181	2.6626	0.013	2.9424	0	0.0017	0	
0.0515	0.0967	2.9461	0.0025	0.0482	0	0.0335	0	
0.068	0	0.6679	0	0	0.0069	0.0686	0	
0.0034	0	0	0	0.0411	0.033	0.0137	1.6949	

0.427	0							
ACKR4	0.3156	0.1074	0.3242	0.2341	0.088	1.1709	1.6636	
0.1145	0.3017	0.3457	0.1207	0.2106	0.2114	0.5669	0.2164	
0.1362	0.7413	0.4345	0.3	0.2528	0.2119	0.2524	0.0994	
0.1862	0.034	0.5182	0.8112	0.2272	0.0767	0.081	0.0499	
0.2458	0.2528	0.2928	0.9855	0.1043	0.0231	0.1129	0.1202	
0.9217	0.4108	0	0.0897	0.3331	0.4485	0.0741	0.4537	
0.2066	1.4002	0.1794	0.2157	0.2001	0.1368	0.1613	0.1693	
0.2582	0.3383	0.1217	0.2659	0.0873	0.0501	0.1527	0.1117	
1.2314	0.1729	0.1847	0.4185	0.1107	0.1188	0	1.0874	
0.4388	0.1622	0.1049	0.0921	0.3606	0.3333	0.1573	0.1308	
0.323	0.1825	0.2469	0.1234	1.0125	0.1488	0.0764	0.4165	
0.1174	0.1474							
ACLY	6.6773	5.872	6.1985	6.206	5.9493	6.7671	5.0672	
6.1423	6.174	6.4248	6.4952	6.4077	6.0146	6.0264	6.3106	
6.0713	7.0409	6.049	6.1756	6.2473	6.5942	5.8654	5.8666	
5.685	6.291	6.622	5.4931	7.0153	6.3528	5.9481	6.4874	
6.6121	5.9694	6.1151	6.34	6.1901	6.6044	5.9944	5.9031	
6.7506	6.274	6.5138	6.1395	6.0542	5.8753	6.1502	7.512	
6.9117	5.4967	6.9473	7.1336	5.3924	6.7303	6.0196	6.0468	
7.1477	7.2655	6.695	6.3689	6.6407	6.5758	6.0297	5.9457	
6.3834	6.6474	5.8515	5.6384	6.8201	7.2118	6.3958	6.4511	
6.249	6.6103	6.3253	6.1915	7.1705	6.9123	6.3899	6.4948	
6.0647	5.3437	6.198	6.2172	5.9924	6.179	6.1642	7.4352	
6.6813	6.4097							
ACMSD	0	0.0258	0.0445	0.0378	0.0544	0.0357	0.0297	
0.0343	0.0345	0.0402	0.0497	0.0185	0.0119	0.1965	0.0088	
0.0103	0.0449	0.0331	0.1414	0	0	0	0.0052	0
	0.0078	0	0.0215	0.1419	0	0.042	0.1114	
8.00E-04	0.0524	0	0.0539	0	0	0	0.0313	
0.0997	0	0.0466	0.0884	0.0507	0.0262	0.0405	0	0
	0.0185	0	0	0.0445	0	0.0369	0.0356	0
	0.0388	0.0505	0.0559	0.0755	0	0	0.0255	
0.2169	0	0	0	0	0.0653	0	0	0
	0.0287	0	0	0	0.0352	0.0801	0.0568	
0.0482	0.0232	0	0	0	0	0.0971	0.0321	
0.0676	0							
ACO1	4.0721	3.6486	3.8421	3.8598	3.8454	3.3012	3.5611	
4.2591	4.4077	4.6985	4.3222	4.1181	4.7689	4.6198	3.8265	
4.3765	4.7183	4.1471	3.9781	3.0515	3.4516	3.5209	3.7353	
3.9529	3.886	4.2257	3.7536	1.7241	3.9137	3.6855	3.9987	
4.565	3.8361	3.4975	3.4744	4.5455	2.8604	3.5232	3.9692	
4.526	3.398	5.5858	5.737	4.1784	4.3802	5.7689	4.7141	
3.1339	4.2706	5.2048	3.5524	5.3295	4.0254	2.0635	4.9056	
3.1189	5.5553	4.1074	5.2486	4.1381	3.5313	4.2125	3.6125	
4.2148	3.9186	5.3276	2.9728	4.3334	3.4608	3.9952	4.2599	
3.8235	3.0832	4.5497	3.49	4.0949	2.9616	4.4117	4.2409	
4.146	4.8712	4.1815	3.0663	3.5666	4.1317	4.1344	5.1439	
4.2649	4.5775							
ACO2	6.1108	5.2521	5.6527	4.4271	4.0429	5.7233	5.0365	

6.1537	5.8127	5.5386	5.6746	5.0399	5.1423	4.8129	5.6431	
5.4993	4.2815	4.3749	5.3996	5.8841	5.3413	4.7233	4.6687	
5.8577	4.8609	4.3295	4.6173	5.4893	4.7659	6.7013	5.2553	
6.3076	5.1055	6.3781	5.3632	4.764	5.0879	5.2084	6.3961	
4.6549	6.1219	4.9054	4.382	5.4024	4.3295	4.3061	5.7392	
5.0601	4.5615	3.8762	4.828	3.8842	5.3763	4.245	5.1872	
5.2178	4.0731	5.0026	5.7859	5.3329	5.162	5.7756	5.5432	
5.7704	5.2775	4.6203	6.3117	5.5091	5.4861	4.2069	5.5491	
4.9052	5.5624	3.5979	5.1675	6.2855	5.368	6.0423	4.8587	
5.8117	5.582	4.4951	5.504	5.2704	4.3561	5.6323	5.9511	
5.8711	5.85							
ACOT1	2.55	0.1939	0.361	0.6294	0.7677	1.7918	1.0891	
0.4229	1.2412	1.0171	0.0681	1.7064	1.1108	0.4918	1.0476	
1.3252	1.9705	1.9517	1.2772	2.2806	2.113	0.8323	1.3881	
0.3772	1.5117	1.2635	0	2.3255	1.7021	1.256	3.2089	
1.4157	1.1533	1.6943	0.7397	1.3552	2.0679	2.692	0.6135	
1.2383	1.9853	2.4463	1.5742	1.5809	1.6364	1.3575	1.2441	
1.8622	1.7711	1.479	1.8688	1.8146	0.8783	1.1996	2.6938	
1.2221	1.4121	0.3589	0.9552	1.0139	0.9007	2.1171	0.1113	
0.1212	1.9805	1.904	2.0984	1.5903	0.8649	1.4259	1.6461	
1.3729	1.3496	0.6331	1.1533	0.7431	2.5517	1.3186	1.4734	
1.2919	0.4941	2.3661	0.6739	1.2345	0.9633	1.0596	2.0914	
2.0799	1.4395							
ACOT11	1.0287	0.1771	2.165	0.6771	0.2533	0.0644	0.0675	
0.3337	1.4237	2.1427	0.1254	0.1213	0.3636	0.2271	0.1497	
1.585	1.4237	1.0258	0.3154	0.189	0.8913	0.849	0.1816	
0.0707	0.1822	0.2287	0.1439	0.3262	1.3601	2.2025	1.1247	
0.8224	0.4139	0	0.6573	1.1368	0.4085	0.0564	0.5343	
0.1254	0.3259	0.3821	0.5277	0.2371	0.1773	0.3821	0.7022	
0.2356	0.1096	0.3619	0.1203	0.2094	0.1192	0.8108	1.162	
1.967	1.1006	0.0367	0.3549	0.0139	0.463	0.6465	0.142	
0.1608	0.0625	0.2869	1.1012	1.104	1.689	1.2856	0.0311	
0.0703	0.0442	0.2725	0.068	1.3706	0.5268	2.0703	1.3178	0
	1.0442	0.0903	0.0959	0	0.4248	0.2136	0.7839	
1.2018	1.0727							
ACOT12	1.763	2.3005	0	0	0.1106	0.0725	0	
0.091	0.0998	0	0.5664	0.0371	0.0178	0.1811	0.6018	
0.0557	0	0.4287	0	0	0.517	0.0703	0	0
	0.1024	0	0	0.218	0.0249	0	0.1134	
0.3011	0.1758	0.6151	0.0077	0.6097	0.0124	1.1791	0.7389	
0.0625	0.1052	0.0311	0	0	0.0168	0	0.2728	0
	0	0	0	0.0292	0.8574	0.3848	0.126	0
	0	0	0.1162	0	0	0.0405	0.8995	0
	0.4765	0.0493	2.1318	0.0324	0	0.1894	0.0099	0
	0.4376	0.1155	0	0.329	0.8756	0.0167	0	
0.0395	0.3681	0	0	0.4874	0.0602	0.6341	0.0368	
0.1224	0.1511							
ACOT13	2.8063	2.5566	2.9117	3.1212	1.7477	2.1022	2.5886	
3.1731	2.541	2.853	2.7601	3.1225	1.5572	2.6401	2.3499	
2.1869	3.0495	3.2243	3.0676	2.4804	2.4867	3.0314	3.0102	

3.7871	2.5044	3.4938	2.617	3.3632	2.2769	2.0439	3.3641	
2.355	2.303	2.9001	3.063	3.466	3.488	2.9448	2.7545	
2.7331	2.5211	2.9211	2.6181	2.6219	2.1207	3.4042	3.3636	
2.5062	2.0698	3.1019	2.6388	3.3636	3.0324	1.804	3.2066	
2.2569	3.0695	1.809	2.2167	2.1007	2.809	3.3149	2.3094	
3.0749	3.1322	2.1873	3.6078	3.2904	2.6121	2.4742	3.0547	
2.8088	2.4747	3.1928	2.0337	3.1873	2.6927	3.5534	2.5984	
3.3826	2.7098	3.0715	3.5773	3.2294	3.0729	3.4374	2.7337	
2.3747	2.4785							
ACOT2	3.2922	2.6805	3.2041	3.3816	1.9371	2.8105	2.5799	
0.6633	2.4139	2.3904	0.168	3.2074	3.7594	2.8235	1.8779	
3.8014	3.7931	3.2041	2.8322	3.5534	3.525	1.709	3.1756	0
	2.1186	2.3218	0	3.1895	2.9272	2.3355	4.2445	
2.6034	2.9845	2.5592	1.6294	3.0347	3.2249	3.8991	3.3399	
3.1813	4.0989	3.1281	2.3395	2.6885	2.8736	2.6185	2.7233	
3.1969	2.5454	3.6261	2.8289	3.4426	2.4697	2.2918	3.4373	
2.5668	2.7228	2.5201	2.0301	2.3801	3.3157	3.6881	0	0
	3.6666	3.8412	3.7775	3.6812	1.7778	2.9734	3.2853	
2.2102	2.7664	2.2713	3.6192	2.8808	3.3029	2.924	3.5662	
3.1233	3.1498	3.7983	1.7683	2.5438	3.1716	2.6571	3.9327	
3.7692	3.9865							
ACOT4	0.1649	0	0	0.441	0	0	0.0931	
0.4809	0.0299	1.2623	0.0367	1.8816	2.2331	1.8686	0.3502	0
	0.046	0.1835	0.0662	0.1092	0.0282	0.4716	0.1527	0
	0.7504	0.5651	0.1689	0	0.4928	0.3428	0.123	
0.7692	1.2084	0.7295	0.0472	0.7106	2.3126	0.0172	1.3227	
0.0886	0.7488	1.9928	0.107	2.3139	0.7338	0.9159	1.8719	0
	9.00E-04	0.9575	0.3572	0.9698	0.3981	0.1189	0	0
	0.8128	0	0.9433	0.1361	0	0.3131	0.3474	
0.1538	0	1.8532	0.1654	0.4228	0.1537	0.1765	0.1687	
0.7235	0	1.2685	0.8717	1.5262	1.7851	0	0.114	0
	0.1292	2.4642	1.6325	0.9458	0.3368	0.1991	1.3107	
1.7866	1.228							
ACOT6	0.1791	0	0	0	0	0	0	0
	0	0	0	0.1187	0	0.2236	0.0324	
0.0368	0	0.1391	0.2102	0	0.1881	0	0.0226	0
	0	0	0.0485	0	0.1271	0.0474	0.8729	
0.0753	0.0606	0.1655	0.0167	0.3617	0.068	0	0.0368	
0.042	0.0454	0	0	0.2527	0.0319	0.0469	0.1845	0
	0	0	0	0	0.1659	0	0.6716	0
	0	0	0.067	0	0	0	0.3253	
0.185	0	0.0911	0	0	0	0	0	0
	0	0.1862	0	0	0.1611	0	0	
0.2211	0	0.0445	0.0543	0.0662	0	0.2955	0	0
	0.0879							
ACOT7	6.4231	6.8592	7.0696	5.926	7.1577	6.5807	7.1033	
6.3172	6.5102	6.4096	6.3659	6.7231	7.3022	6.1465	6.4753	
7.1033	4.6182	5.8882	6.0958	7.048	7.1792	6.0452	6.989	
7.1623	5.5809	4.7854	4.8458	6.3096	6.266	6.1509	5.4888	
7.0301	8.0268	7.3591	6.9683	5.9016	6.0881	6.7927	6.6227	

5.5011	6.664	6.8361	7.0696	5.6799	6.6673	5.419	4.6603	
4.9611	7.085	4.568	6.7313	4.7804	7.0547	5.3633	5.712	
6.0416	5.8155	5.8812	7.6412	6.2433	6.0761	5.5462	5.9062	
7.5762	5.8399	4.1029	6.4824	7.0067	6.7738	6.406	6.7969	
7.5207	6.695	6.3521	5.8338	6.8318	7.385	5.2544	7.4849	
7.5616	6.9369	6.5268	6.7354	5.548	6.946	7.7212	6.3237	
6.9936	6.6937							
ACOT8	3.7576	4.0551	4.2302	2.5533	4.4304	2.8125	4.127	
4.5311	4.2731	3.813	4.6425	3.9039	4.3401	3.0518	3.2826	
4.0563	3.8886	3.2688	3.6015	4.0475	3.4268	5.0258	4.6725	
3.27	4.138	4.64	4.0588	3.4971	4.3592	4.388	4.2213	
4.1976	3.722	5.1123	4.0063	3.2967	3.5493	4.8429	4.725	
3.3955	4.3728	4.2926	3.6985	3.7748	3.8644	3.396	3.1753	
4.4362	4.9567	3.7666	4.1026	3.2087	3.7812	3.5718	3.9262	
4.2103	3.6463	2.8875	4.1744	2.5722	4.146	3.7762	3.3264	
3.9947	2.9025	4.069	3.7729	3.8079	4.0204	3.9199	5.0336	
4.8262	4.9461	4.9194	4.2342	3.2023	3.8651	4.2721	4.2791	
4.4953	4.0495	4.7783	4.0487	4.2306	4.5432	4.3827	4.0847	
4.1657	4.1745							
ACOT9	4.8638	4.8179	4.0337	4.4149	4.7148	4.5121	5.0563	
4.0934	5.4046	4.5858	4.0428	4.8871	5.1645	4.8533	4.7955	
4.9915	4.2337	4.5386	4.0441	4.0911	4.3484	3.8786	4.4891	
5.1061	4.0453	4.9339	4.7715	4.2739	4.5438	4.718	4.2915	
4.2132	4.6477	5.1005	4.6296	4.9481	4.5129	3.9511	4.0683	
4.9908	4.3191	4.2144	4.9784	5.2893	5.3345	4.6624	4.9351	
4.1773	4.581	4.6661	5.2257	4.6352	4.1605	4.3691	5.7537	
4.0903	5.4255	4.4313	3.9988	4.4007	4.2755	4.826	4.723	
5.2557	3.9764	4.3896	4.4922	5.0526	4.8489	4.6992	4.44	
4.0225	4.8126	4.2855	5.0884	4.2383	4.5928	4.7156	4.2965	
4.3147	4.4667	4.8048	4.6486	4.4773	4.9166	4.2302	5.1248	
5.0075	4.9168							
ACOX1	3.3105	3.1238	3.0896	3.2135	2.6839	3.0314	2.9097	
2.8516	3.0542	2.9332	2.9404	3.1151	2.3121	2.5582	2.8219	
3.1565	2.4908	3.3745	3.0033	4.1057	3.6809	2.0136	2.3849	
2.4856	2.724	3.099	2.9504	3.5872	2.7711	2.9207	2.4529	
2.7961	2.6623	3.1641	2.7957	3.2156	3.5819	3.3105	2.375	
3.2156	3.1183	3.6156	2.299	2.594	3.1014	3.1076	3.851	
2.7448	2.3397	2.693	3.2172	2.5054	3.2942	3.4384	3.3922	
2.4812	2.5919	2.934	2.8525	2.5507	2.4685	2.6795	3.536	
2.7042	3.5262	3.4448	3.4096	3.7923	3.0927	2.748	2.6229	
2.513	3.4289	2.5187	3.0838	3.7353	3.1168	3.0369	3.11	
2.2283	3.5571	2.7855	3.002	3.303	2.3508	3.0249	3.2635	
3.1912	3.1935							
ACOX2	1.1395	0.0593	0.0462	0.6861	2.2586	0.0446	0.6594	
0.6532	0.0588	1.4195	0.1496	0.4946	0.6173	0.3585	2.5364	
0.1881	1.4177	0.2039	0	0.0873	0.1365	0.5867	0.3793	
1.725	1.2322	0.2664	0.5599	0	0.5595	0.0305	0.019	
1.3965	0.226	0.0536	0.4668	0.699	1.0452	1.7669	0.0568	
1.4147	0.0418	1.7012	2.4632	0.5442	0.8611	0.2215	0.196	
0.035	0.1057	1.142	0.0672	2.0228	0.3125	0.1551	0.44	0

	1.13	0.1756	0.7175	0.0132	1.5008	0.2081	1.17	
0.2984	0.0505	1.8881	0.1526	0.0371	0.0596	1.0775	1.3985	
0.0612	0.1436	1.2722	0.4457	0.4476	0.2403	1.3127	0.0512	
0.3587	0.329	0.4647	0.1238	0.4505	2.0305	0.3976	0.2275	
0.1111	0.1245							
ACOX3	2.0384	2.1445	2.2926	2.3353	2.6187	2.899	1.0406	
2.5206	2.4211	2.3387	2.4449	2.3745	1.8237	2.1487	2.2724	
2.0318	3.9208	1.7485	2.0202	2.7546	2.5468	2.3214	2.2061	
2.4169	2.3269	2.7964	2.2354	2.4408	1.8515	2.4682	2.0966	
2.3064	2.3544	2.3457	2.3684	1.9157	2.14	2.7001	2.3129	
2.0202	2.1499	2.5107	2.3348	2.5184	1.8313	2.2549	1.754	
2.1884	1.7572	2.3171	1.6356	2.0472	2.2082	2.1716	2.4822	
1.8721	2.3144	1.9384	2.1958	2.2534	2.31	2.6228	1.7915	
2.1337	2.8011	1.9788	2.3865	2.7229	1.6035	2.3292	2.732	
2.1135	2.3095	2.2383	2.7543	3.5413	2.1667	2.9222	1.441	
2.737	1.985	2.6168	2.277	2.3506	2.2656	2.442	3.0542	
2.7421	2.8452							
ACOXL	0	0.0647	0	0	0	0	0	
0.0425	0.1675	0.0484	0.0032	0.019	0.0095	0	0.029	
0.0644	0	0.0404	0.0735	0	0	0.0654	0	
0.0847	0	0.0366	0	0.0578	0.0508	0.1075	0.0119	
0.1359	0.0643	0.0247	0.0283	0.011	0.0902	0.1097	0.0606	0
	0.0471	0.0471	0	0	0	0.1279	0	
0.1392	0.1742	0	0	0	0.1615	0.0377	0.0234	
0.0808	0.0425	0	0	0	0.0346	0.4584	0.0152	
0.0526	0	0.0222	0.0943	0.0271	0.0119	0.0419	0.0164	0
	0.041	0.0176	0.0408	0.0349	0.0348	0.1039	0.1037	0
	0.0642	0.0241	0.1011	0	0.029	0	0.106	
0.0829	0							
ACP1	6.7642	6.5392	6.4736	5.8073	5.6458	5.2561	5.1753	
6.7814	6.3806	6.0437	6.6015	5.1476	5.9678	5.8672	5.9123	
6.2286	6.2176	6.4166	5.6836	5.798	6.2441	5.5585	5.6387	
6.1577	5.997	6.0693	5.6609	6.4505	5.5473	6.574	6.2229	
6.0693	6.1386	5.5723	5.7496	6.2689	6.4753	5.6626	5.7641	
5.8705	5.5627	5.3994	5.5359	5.8484	5.6331	5.9262	6.415	
6.2069	5.5981	5.5043	6.2243	5.5261	6.399	6.5292	6.5123	
6.1592	5.497	6.3354	5.656	6.3206	5.7536	5.9531	5.84	
5.8696	6.7378	5.4612	6.6878	5.9178	7.0847	6.4045	5.5266	
5.6284	6.2025	5.5026	5.8715	5.5826	6.1778	5.8167	5.7787	
5.6495	5.9542	5.0994	5.8984	5.7389	5.4598	6.5223	5.4808	
5.7737	5.515							
ACP2	2.9428	4.295	3.3886	4.3205	3.9285	3.1266	4.5793	
3.5055	3.8307	3.271	2.8118	3.832	3.8037	3.5333	4.1276	
3.7058	3.6594	3.0518	4.032	3.7358	3.2977	4.0081	4.4656	
3.4097	5.1604	4.1594	2.5	3.2817	4.2048	2.8489	4.372	
3.6153	3.959	3.9345	4.1706	3.7154	4.4028	4.8588	3.993	
3.6852	4.0057	5.0318	3.6311	3.6953	3.6579	4.3567	3.1574	
3.8855	4.4509	3.1213	4.0787	4.767	3.9333	3.8578	4.4183	
3.7985	2.4404	4.0977	3.8367	3.9438	3.6466	4.0737	4.363	
3.5815	3.2784	5.7407	3.1547	3.755	2.7641	3.8803	3.9478	

3.3018	3.5127	4.187	3.8417	3.6186	3.486	4.2657	4.0596	
4.1275	3.5311	4.0166	4.2977	3.7027	3.3274	3.9219	3.8203	
3.702	3.9141							
ACP5	5.3856	6.1242	4.5689	3.5706	3.4426	5.2313	7.2234	
6.8863	7.0104	7.1578	5.2635	5.4679	2.1416	4.9337	0.3722	
6.851	0.319	5.3038	6.0911	1.672	4.3565	5.3915	6.6399	
5.6933	4.5412	0.8858	5.1983	5.8589	5.2348	5.55	0.2752	
6.0593	6.2973	6.8002	6.3595	5.6711	2.7211	6.8293	5.5141	
4.7753	7.8348	0.7211	0.4683	6.8777	0.3479	0.4607	3.7888	
7.4545	4.9172	0.7899	5.9201	2.492	3.83	6.1306	0	
6.7219	0.626	0.4532	8.3819	1.0745	5.4635	5.8269	6.3229	
6.4838	3.0348	0.334	6.1441	5.6547	5.5856	0.1528	3.7843	
5.0401	6.6379	6.0167	3.9346	6.3002	5.2716	6.0561	7.2489	
7.0773	4.5033	3.9281	5.9079	5.4522	2.212	3.4921	5.6144	
5.8792	5.8609							
ACP6	2.9875	2.1947	2.1549	4.1048	4.2041	3.9918	4.2032	
3.6366	2.8991	2.5601	3.2896	4.9855	2.8532	3.9171	3.6038	
4.7677	2.7788	3.2039	3.042	4.5935	3.1014	4.6534	3.5796	
4.1164	4.5445	2.2038	3.7836	2.2794	4.4765	3.6271	3.3301	
3.3586	3.4022	3.5354	4.1057	3.0165	4.5634	2.2499	4.4097	
4.721	4.6178	3.9997	3.8803	3.3338	2.5365	2.3103	3.2535	
3.5475	4.3375	2.5979	3.9799	5.047	3.2739	3.313	3.2068	
3.4077	3.8508	2.5704	3.3918	2.4274	2.9556	2.9191	4.1708	
3.3963	2.6597	3.2951	2.5182	3.1922	2.8045	4.0042	4.2697	
3.7159	3.8005	4.6614	5.8423	2.7124	3.473	4.4882	3.9469	
3.7174	1.7031	4.9122	2.8364	4.251	1.4516	3.5603	1.8541	
2.5387	2.0415							
ACPP	0	0	0.0488	1.6249	2.1318	0.4357	0.6195	
0.8888	0.0827	0	0	1.3748	0.5615	0.2386	0.0116	0
	0	0.0014	0.0045	0	0	3.1885	1.7669	
0.7038	1.5403	0	1.0565	0.1156	0.7504	0.1132	0	
0.3178	0.6574	0.5949	0.7419	0	0.0041	0.7148	0.0518	
3.1642	0.5165	2.2599	3.8217	0.1271	0.0184	1.2034	0.7845	
2.2089	0.209	0.3641	0.1452	1.4132	0.0165	0.1334	0	
0.2451	0.3356	0	0.1494	0	0.877	0	0.1718	
0.3391	0.1593	0.2539	0.1766	0.1266	1.5552	0.5944	0.869	
0.1738	0.6322	2.352	0	0.6116	0.3434	0.2204	2.0055	
1.9167	1.411	0.2712	0	0.9391	0.6007	0.1854	0	
0.2242	0.0931							
ACPT	0	0.107	0	0	0.1032	0	0	
0.1455	0.0727	0	0	0	0	0	0	
0.0599	0.1707	0	0	0	0	0	0	0
	0.0962	0.1929	0	0.1441	0	0.0957	0	
0.0222	0	0	0	0	0	0.0957	0	0
	0.1167	0	0.2206	0	0.0709	0	0	
0.1058	0	0	0	0	0	0	0.1261	
0.0946	0	0.0749	0.2199	0	0.0331	0.0248	0.1742	
0.1545	0	0.0318	0.0584	0	0.0198	0.1277	0.0299	
0.0718	0.039	6.00E-04	0.083	0	0	0	0	0
	0	0	0	0.0754	0	0.2084	0	

0.0959	0.1126							
ACR	0.4193	0	0	0	0	0.0771	0.0772	0
	0	0.1145	0	0	0.0936	0	0.034	0
	0	0.0777	0	0	0	0	0	
0.0708	0.0552	0.0514	0.1088	0.1119	0.1092	0.0891	0.0713	
0.0155	0	0.0971	0	0	0.0589	0	0	
0.273	0.266	0.0201	0.1608	0	0	0	0.0738	
0.0783	0	0	0.0842	0	0	0.0986	0.1642	
0.2396	0.1852	0.0974	0.0472	0	0	0	0	0
	0.2678	0.0296	0.2642	0.0959	0.1022	0	0.0733	0
	0.1294	0.0123	0.2092	0	0.0357	0	0.1483	
0.0755	0	0	0.0717	0.1953	0.1192	0	0.1965	
0.0842	0.6202							
ACRBP	0.0612	0.1455	0.0528	0	0.0945	0	0.0458	0
	0.13	0.2096	0.0919	0.0557	0.118	0	0.0662	0
	0.0582	0.4481	0	0.0606	0.0457	0.0456	0	
0.0488	0.1272	0.1773	0.4485	0.0592	0.0257	0.3776	0.0573	
0.0143	0.1024	0.0283	0.0685	0.0441	0.3492	0.0724	0.0974	
0.0285	0.2034	0.0621	0.0854	0.302	0.0424	0.06	0	
0.0321	0.0476	0.1762	0.1093	0.092	0	0.2762	0.1177	
0.2432	0.3303	0.0531	0.129	0.1073	0.0164	0.0785	0.5787	
0.1444	0.0868	0.3057	0.0417	0.1053	0.1124	0	0	
0.0988	0	0.0189	0.1233	0	0.0097	0.105	0.0105	0
	0.0909	0.1053	0	0.2323	0.0373	0	0.0724	
0.0781	0							
ACRC	1.0866	1.2424	0.601	0.4591	0.1232	0.2042	0.6532	
0.2025	0.4188	0.414	0.2444	0.2434	0.3905	0.3307	0.3529	
0.2707	0.9984	0.6706	0.7117	0.4143	0.4398	0.0984	0.2508	
0.8626	0.3651	0.7908	0.1658	0.3508	0.341	0.9958	0.0997	
0.1549	0.4527	0.5446	0.4779	0.12	0.4872	0.3728	0.418	
0.6205	0.6285	0.3446	0.1717	0.1952	0.3928	0.2954	0.4051	
0.5214	0.8016	1.2038	0.3563	0.9722	0.629	1.0526	0.0648	
1.0247	0.6027	0.3385	0.4892	0.3809	0.4292	0.2632	0.3326	
0.6968	0.3522	0.2548	0.4101	0.7366	0.4142	0.1847	0.5272	
0.3177	0.255	0.2893	0.3369	0.1693	0.6837	0.535	0.324	
0.3882	0.2439	1.01	1.3454	0.1981	0.1783	0.5	0.4978	
0.4046	0.5885							
ACRV1	0.703	0.2536	0.3689	0.5167	0.3544	0.0998	0.2944	
0.307	0.3287	0.0926	0.0713	0.1177	0.1094	0.3235	0.1304	
0.0674	0.8618	0.0365	0.2268	0.6694	0.1774	0.0935	0.0508	
0.1353	0.0494	0.4856	0.0299	0.4895	0.1088	0.051	0.3213	
0.2038	0.1364	0.0199	0.0613	0.1421	0.059	0.0346	0.0864	
0.3344	0.2204	0.2218	0.4828	0.4075	0.1881	0.0155	0.1053	
0.1584	0.4522	0.6224	0.1696	0.271	0.071	0.0895	0.3338	
0.348	0	0.8335	0.1974	0.8204	0.3445	0.0679	0.2403	
0.2969	0.1323	0	0.2972	0.1435	0.0702	0.319	0.0785	
0.0865	0.0883	0.0448	0.6274	0.27	0.4014	0.1127	0.0666	
0.1085	0.0777	0.156	0.074	0.1285	0.4421	0	0.0367	
0.3145	0.1442							
ACSBG1	0.745	2.1335	0.201	0.5954	0.0436	1.1671	0.2571	

0.4308	2.5719	3.1407	0.3462	0.0203	0	0.2252	1.5909	
0.3078	0.0588	1.9033	1.3792	0.4417	0.8977	1.2786	1.1373	
0.813	0.049	0.2031	0.183	0.5451	1.0781	2.1156	0.0033	
0.2551	1.3496	0.5559	2.6153	1.1464	0.2437	2.8406	2.6807	
0.2292	1.9406	0.1046	0.3211	0.238	0.4783	0.2192	1.2476	
2.2447	0.1934	0.2959	0.4801	0.0669	0.527	2.333	0.1463	
1.4096	0.1007	0.139	1.8872	0.141	0.8975	2.0436	0.7481	
0.8907	0.31	0.0837	0.738	0.1545	0.4481	0.1286	0.1734	
0.6033	0.5772	0.4802	0.444	0.8059	1.2171	0.9338	3.1366	
1.0529	0.6729	0.3349	0.3091	0.3516	0.4307	0.4837	0.207	
0.2459	0.6934							
ACSBG2	0.0958	0.0135	0.0708	0.0276	0.0155	0.0414	0.0144	
0.0061	0.052	0.0751	0.0907	0.0085	0.0047	0.0866	0.0229	
0.0149	0.0494	0.0214	0.0917	0	0.0257	0.0082	0.0014	0
	0.0434	0.0168	0.0142	0.0533	0.0564	0.1321	0.0057	
0.0685	0.0141	0.0671	0.0283	0.0067	0.0106	0	0.0271	
0.1189	0.0333	0.0177	0	0.0446	0.0047	0.0491	0.0218	
0.0157	0	0.1093	0.0102	0.0279	0.0966	0	0.1107	0
	0	0.0313	0	0.0545	0	0	0	
0.0697	0.0619	0.0539	0.048	0.0334	0.0559	0.0581	0.0175	
0.0447	0.0523	0.0053	0.0212	0.0288	0	0	0.0107	0
	0.0186	0	0	0.0721	0.0402	0	0	
0.042	0							
ACSF2	4.3155	3.9397	2.7149	2.5489	1.9408	1.877	3.1065	
1.3026	4.6539	1.8994	5.3189	3.3452	3.672	3.067	0.7712	
2.5814	2.263	3.3129	2.7964	3.7609	1.9764	1.9995	3.4488	
2.7384	0.9965	2.9211	4.038	3.4048	1.9482	3.9596	2.3319	
3.2095	2.9116	4.9941	1.1684	1.2902	2.723	2.9984	2.0921	
3.1381	3.8116	4.1252	2.9228	1.4801	1.8852	1.6005	2.3732	
4.4322	1.8381	3.7069	3.5765	0.6922	3.4553	3.6468	1.7347	
3.2338	3.5061	2.3533	2.7123	2.1212	1.4072	2.374	1.1415	
2.5257	1.7501	3.6991	3.2075	5.1094	3.0591	2.6294	2.9383	
3.5538	3.6119	4.4992	1.9468	1.753	2.6382	2.9255	2.6119	
3.3311	4.2531	0.8972	1.2812	3.6296	2.3159	3.6774	4.438	
3.2247	4.5376							
ACSF3	2.9036	3.6044	3.4857	2.1677	2.5909	2.1754	1.6058	
2.2497	2.083	3.2594	2.32	1.4247	1.6307	1.4696	2.2243	
1.5605	2.3943	2.5977	2.3733	2.946	2.5228	2.0765	1.4748	
2.1466	1.7625	1.964	2.11	2.8128	1.5205	3.1937	2.1721	
2.6254	1.781	1.8308	1.9965	2.5411	2.0751	3.1544	2.4299	
1.6824	2.754	1.6274	1.6641	1.6092	1.9433	1.8499	2.0554	
1.8617	1.5868	1.9195	2.1268	1.7466	2.1036	2.7158	2.9859	
3.1292	2.8249	1.7803	2.5581	1.6899	2.465	2.7427	2.4751	
1.9689	2.8409	1.7648	2.0335	2.4531	2.7912	2.7039	2.2888	
1.981	1.3721	1.6057	1.739	1.8567	2.5344	2.4984	2.8529	
2.2685	2.7329	1.8544	1.9239	2.5752	2.4092	2.219	2.4742	
2.5608	2.258							
ACSL1	4.9457	5.8259	7.4813	5.0153	3.6031	6.0456	6.535	
4.5935	5.2333	5.269	4.8035	6.0573	3.2676	5.9351	5.7629	
5.0476	1.8886	5.9829	5.2186	6.4728	4.4246	4.7226	4.3923	

5.1145	3.0319	4.2217	4.8113	7.4596	4.6703	6.0371	3.2357	
3.8416	5.1976	5.2044	4.5994	5.5379	3.124	6.5229	5.8002	
4.9848	5.8209	4.1782	4.8235	4.1454	3.7464	3.7319	7.9202	
5.269	3.4312	3.113	4.6694	4.6483	5.9141	5.1557	3.2969	
4.4149	4.4648	6.9879	5.4002	6.1685	3.9204	5.1671	4.5906	
5.9116	6.1814	4.1099	4.7125	7.139	7.3339	3.5452	5.5652	
5.0365	5.395	5.0482	5.6223	6.5499	5.3677	3.6556	3.8836	
5.3538	4.9806	4.4965	2.9001	6.3312	2.2963	5.213	6.2273	
5.6381	6.161							
ACSL3	7.9272	6.2015	6.1675	6.3936	5.9212	6.7564	6.2819	
6.4517	8.2241	7.7631	7.8945	6.5263	5.1256	5.8219	6.2881	
6.3495	5.6241	7.9633	6.8742	6.9666	7.3681	6.2245	6.3205	
6.0518	6.5548	5.2595	4.9028	6.4922	6.0127	6.801	5.3329	
5.6691	5.8196	6.6652	6.4793	7.773	6.0592	6.6194	6.8431	
6.5827	7.1521	6.058	6.6845	8.6383	6.1455	5.1074	7.4712	
7.0543	6.5693	6.2315	7.2683	7.1726	7.6519	7.3594	5.1514	
7.4379	6.3609	6.8062	7.8844	6.5852	6.1568	7.0252	6.2496	
6.4591	5.8422	4.694	5.7788	4.7248	7.021	6.9075	6.2713	
6.3875	7.096	6.6173	6.6625	6.4216	7.3348	7.8532	6.9309	
6.5583	8.1393	5.9683	6.8082	7.1723	5.3664	7.0166	7.773	
7.1408	6.6675							
ACSL4	4.6461	3.3522	4.4147	5.347	4.2141	4.6735	5.6546	
4.4155	4.6001	4.7272	4.616	6.2504	5.64	4.4636	5.0556	
4.8278	5.6629	5.9729	4.2384	4.6218	5.1867	4.7001	4.6194	
5.1736	3.9882	5.2978	5.3015	4.5916	4.8166	4.7799	4.0875	
5.3015	4.4885	4.9115	4.9068	5.1466	4.6772	5.7744	4.2895	
5.1472	4.8048	5.7764	4.7999	5.0506	6.1863	5.5062	5.2043	
4.5517	5.0629	6.765	5.0906	5.3485	4.8701	4.8808	4.5921	
4.71	6.281	4.7115	5.3598	5.0631	4.3621	3.9928	4.7566	
5.0414	4.0335	6.0885	4.4159	5.6372	5.626	4.7862	5.0168	
3.8938	4.8417	4.6896	4.0083	5.4554	5.4964	5.4053	4.656	
4.1248	4.6891	5.2071	4.8523	5.2405	4.4297	4.3705	5.1625	
4.7449	4.4411							
ACSL5	0.0542	2.4235	0.0498	0.665	0.2324	0	0.223	
0.0281	2.7621	0.632	0.0667	1.0338	3.0111	0.652	0.0422	
0.0842	0.9496	0.1102	1.0381	0	0.0506	1.6871	0.8352	
0.5482	0	1.7632	0.158	0.5604	7.00E-04	1.8277	0	
0.7342	0.2896	0.1289	2.471	0.1005	0.0538	2.9153	0.2257	
0.0316	0.9368	3.9143	0	0.0378	0.0451	3.6213	0.0766	
0.1413	2.2224	0.8951	0.2767	2.2366	0.8697	0.6099	0	
0.491	4.6686	0.1677	0.3901	0.15	0.3246	0.6344	0.2252	0
	0.1359	3.4663	0.3277	0.2639	1.8664	0	0.2305	
0.2281	0.5341	0.9776	0.1247	0.5255	0.6902	0.36	0.5021	
1.3643	1.0257	0.5845	0.5305	0.1086	0.2751	0.7976	0.1144	
0.296	0.4736							
ACSL6	0.4022	0.4517	0.5071	0.2762	0.3218	0.0556	1.2606	
0.2399	2.1739	0.7427	0.9722	0.5493	0.5498	0.2327	0.3645	
0.2223	0.008	0.1346	0.449	0.0361	0.0966	0.0509	0.6183	
0.2384	0.0031	0	0.1853	0.5869	0.5841	0.7218	0.398	
0.0766	0.3992	0.5042	1.3111	0.2821	0.2976	0.0614	0	

0.1299	0.3239	0	0.4353	0.3873	0.0572	0	0.4262	
1.0895	1.2502	0	1.0098	0.397	0.42	0.223	0.3906	
0.1272	0	0.3665	0.6493	0.2079	0.2477	0.2545	0.0902	
0.2798	0.8437	0.2855	0.1238	0.0683	0.8902	0.1484	0.072	
0.4224	0.1122	0.284	0.0928	1.2485	0.1076	0.1002	0.2517	
0.0958	0.4466	0.2415	2.3261	0.1694	0.1137	0.5367	0.2196	
0.0426	0.0997							
ACSM1	0	0	0	0	0	0	0.0187	
0.0229	0	0	0.032	0	0	0	0	0
	0	0	0	0	0	0	0	0
	0	0	0	0	0	0.0362	0	0
	0.0375	0	0	0	0	0	0	0
	0	0.007	0	0	0.0218	0	0	0
	0	0	0	0	0	0.0783	0	0
	0.0252	0.0183	0.0177	0	0	0	0	0
	0	0.0091	0.0349	0	0	0	0	
0.0123	0.0191	0	0	0	0.0049	0	0	0
	0	0.0527	0	0	0	0	0	0
	0							
ACSM2A	0	0.0292	0	0.027	0	0.0237	0	0
	0.0181	0.0593	0.0036	0.0089	0.0154	0	0.004	0
	0	0.0213	0.0602	0	0	0	0	
0.0449	0	0.0073	0	0.0845	0	0.0134	0	
0.0862	0	0	0.0029	0.0166	0.0044	0	0	0
	0	0	0	0	0	0.028	0	
0.0169	0.0473	0.0448	0	0.0145	0	0.0086	0	
0.0615	0	0	0.0224	0	0	0.0113	0.0159	0
	0	0	0	0.0136	0.0495	0.0551	0.0131	0
	0.0163	0.0324	0.0171	0.0248	0.0388	0	0	0
	0	0	0.043	0.0183	0	0	0.0204	0
	0							
ACSM2B	0	0	0	0	0	0	0	0
	0	0	0	0	0	0	0	0
	0	0	0	0	0	0	0	0
	0	0	0	0	0	0.0213	0	0
	0	0	0	0	0	0	0	0
	0	0	0	0	0	0	0	0
	0	0	0	0	0	0	0	0
	0	0	0	0	0	0	0	0
	0	0	0	0	0	0	0	0
	0	9.00E-04	0	0	0	0	0	0
	0	0	0	0	0	0	0	0
	0							
ACSM3	0.1855	0.1376	0.2627	0	0.5989	0.1685	0.7133	
0.0441	0.3333	0.0249	1.1746	1.3204	0.056	0.9582	0.0609	
0.1659	0.3517	0.0755	0.3397	0.5601	0.0493	0.1345	0.2888	
0.1894	0.0173	0.1531	2.0408	0.0544	0.2618	0.466	0.0238	
1.6662	1.5051	0.9197	0.1867	0.1763	0.5199	0.1813	0.0552	
1.2127	1.0034	1.3098	0.1831	0.7352	0.1271	0.221	0.1823	
0.3584	0.2841	1.5689	0.3124	1.0269	0.1746	0.2337	0.1752	0

	1.3243	1.3631	0.0623	1.0445	0.3251	0.4718	0	
0.2527	0.1089	0.2758	0.4217	0.2072	0.2359	0	0.583	
0.0497	1.4002	0.8029	0.1407	0.1797	0.0994	0.0768	0.402	
1.0297	0.8273	0.5017	0.053	0.2699	0	0.1418	0.2444	
0.5176	0.1223							
ACSM4	0	0	0	0	0.0364	0.0312	0.0621	0
	0	0	0.0036	0.0267	0.1836	0	0	
0.0072	0.9441	0	0	0.0369	0.0338	0.0015	0.2242	0
	0	0.0435	0.2371	0	0.0325	0	0	
0.0072	0	0.1144	0.086	0	0.036	0	0.0094	0
	0	0	0.029	0	0.1877	0.3828	0	0
	0.2249	0.2653	0.014	0.7487	0	0	0	0
	0.1674	0	0.0771	0	0.0972	0.1131	0	0
	0	0	0	0	0	0	0	
0.0664	0	0.089	0	0	0	0	0	0
	0	0	0.0555	0	0.203	0	0.0256	0
	0							
ACSM5	0	0	0	0	0	0	0.038	0
	0.1621	0.0119	0	0	0.6579	0	0	0
	0	0	0	0	0	0.3989	0.2034	0
	0.0201	0.0081	0	0	0.0679	0.0735	0	
0.553	0	0	0.6522	0	0.0786	0.0486	0	0
	0	0.4618	0	0	0	0.7195	0	0
	0	0	0.0711	0	0.0404	0	0	0
	0	0	0	0.0364	0.0274	0	0	
0.0462	0	0.1227	0	0	0.2264	0	0	0
	0	0.8572	0	0	0	0.0597	0.4401	0
	0.0607	0	0.1696	0.0683	0	0.1397	0.6227	
0.1609	0.1134							
ACSS1	4.5741	2.5243	0.6693	3.4752	2.4975	3.9685	3.8508	
4.7716	4.9639	3.7396	5.1851	3.0701	2.1862	3.2716	2.8345	
3.7367	0	4.3612	4.1831	3.1682	1.8904	4.5837	4.4561	
3.2526	3.7404	1.7121	2.6114	2.6073	3.9475	4.1632	0.6183	
2.9285	4.4293	4.7377	4.196	3.1547	2.0821	4.3981	4.3567	
4.1594	4.6546	1.2822	4.6533	4.9551	2.4491	1.0194	4.1021	
4.1504	4.2533	0.6433	4.8992	3.429	4.7819	4.0052	1.3864	
5.1602	1.5592	3.8446	5.0907	3.6482	3.9216	2.8923	3.4875	
4.7281	3.6603	0.2766	0.6461	1.5066	4.0126	4.3691	3.8658	
3.6896	5.4878	4.401	4.1602	2.7851	3.3035	1.8914	3.8789	
4.3159	2.8127	3.146	2.9427	5.2266	3.0787	4.3708	3.501	
3.8816	3.9006							
ACSS2	4.0368	3.6113	4.5133	3.0581	2.9071	4.1337	4.3644	
3.7228	4.179	4.265	4.1916	4.2404	3.5099	3.7324	3.5427	
4.2375	3.0178	4.4879	3.698	3.7544	3.3519	3.4925	3.9362	
4.0288	5.1805	4.0336	4.3337	3.6811	3.911	4.2912	3.1881	
3.1336	4.1685	3.9962	3.7683	3.9595	3.0764	3.8061	3.7557	
5.111	4.8552	4.6397	4.0957	4.8709	5.152	3.8162	7.0759	
4.5679	4.6166	2.9671	5.9174	3.8636	5.9975	3.0672	3.9852	
5.5166	5.4491	3.1804	4.8799	3.447	4.3308	2.7846	3.7541	
3.8628	3.6802	3.2539	3.6526	3.8297	5.9277	4.3445	5.0975	

3.0718	4.4352	3.1916	4.3496	5.7474	5.3464	4.6405	3.8828	
4.7073	3.7857	4.8048	4.667	4.1384	4.133	4.7795	6.6922	
4.1822	4.6015							
ACSS3	0.1829	1.5002	0.0255	2.7689	1.5149	1.2367	0.0236	
0.0847	2.6002	4.8795	0	0	0	3.0945	0	0
	2.7976	0.0433	0.0304	0	0	3.7942	3.4973	
2.6911	3.961	0.1581	0.0484	0	2.6093	0.6992	0	
4.0429	0.0534	0.0277	3.5416	3.1204	5.2078	0.014	1.2142	
0.0427	0.5549	2.5768	0	0.0341	0.5016	0	1.4047	
5.2525	1.4763	0.1275	0	2.9943	0.052	5.0224	0.105	
0.1646	2.6827	2.6085	3.1281	2.0929	0.0694	4.4028	1.3768	
3.6592	0.1114	3.3218	0.0807	0	0.0859	0.0881	3.4491	
0.4907	0.0997	3.574	0	2.8588	0	0	0.116	
0.0869	0.0957	0.0784	0.1103	2.0486	2.5448	0	0.3443	
0.4431	0.1014							
ACTA1	0.0899	0.0592	0.1279	0	0.063	0	0.1635	0
	0.1224	0	0.1078	0.0705	0.0379	0	0.0392	
0.072	0.0849	0.0883	0.1452	0.2491	0.0654	0.0828	0.0468	
0.2307	0.058	0	0.1794	0.0878	0	0	0.0612	
0.0712	0.0986	0	0.024	0.0502	0.1025	0.1331	0.0922	
0.4177	0.1953	0	0	0	1.9177	0	0	
0.0995	0.1271	0.1447	0.0525	0	0.1088	0.2913	0	
0.234	0.0135	0	0.0215	0.8304	0.1737	0.1696	0.1085	0
	0	0.0263	0.1449	0.0047	0	0.2328	0	
0.0611	0.077	0.0227	0.0344	0.1591	0.5072	0.002	0.0934	
0.252	0	0	0.2267	0.1203	0.1893	0	0	
0.0631	0.1541							
ACTA2	3.8798	0.2949	3.4873	2.9648	3.4797	2.8292	2.4682	
3.44	4.3365	2.5498	3.6701	2.23	1.5507	1.0993	2.0334	
2.7911	3.9795	3.2771	2.7911	4.088	0.4797	2.0636	4.8641	
3.0179	3.2639	4.7754	3.6049	1.9883	4.3129	2.0524	0.3867	
0.0323	1.0049	3.0468	4.6794	0.88	0.675	0.112	0.4536	
3.8348	4.8959	2.487	4.4832	1.4462	3.5575	3.7195	2.385	
2.8547	4.6081	0.2836	2.1402	3.9195	1.0723	0.6454	1.2139	
1.0544	5.0548	0.499	1.1896	0.6235	3.489	2.0646	2.0143	
0.9864	3.0352	5.0234	4.1274	4.4732	1.6838	4.038	4.1509	
1.7026	1.4029	2.0236	3.0678	3.1169	1.449	4.802	4.0894	
2.8952	1.5257	4.4812	4.0126	3.7527	1.5925	1.4458	3.9613	
3.7776	2.0729							
ACTB	10.7045	10.3261	11.5008	12.4048	10.624	11.2498	10.624	
11.4155	11.5008	11.2498	10.7045	10.3988	11.6006	11.6006	11.9778	
11.3212	10.221	10.4785	11.4155	11.4155	11.6006	11.6006	11.5008	
11.5008	11.7612	11.1801	8.7098	10.8325	11.7612	10.1367	11.4155	
10.8707	11.2498	10.9683	11.6006	11.1171	10.5722	10.4785	11.0615	
10.5722	11.7612	11.2498	11.6006	10.9683	11.0615	11.2498	11.6006	
11.012	10.8707	10.105	10.9225	10.1716	10.7619	10.124	11.1355	
11.4192	11.0426	11.2442	11.0426	12.1202	10.7862	11.6551	10.9399	
12.1202	10.7176	10.6031	10.1502	11.4192	11.1355	11.6551	11.6551	
12.1202	11.0426	11.1355	12.1202	11.0426	10.2076	11.6551	10.304	
10.9399	10.657	10.7862	10.5056	11.6551	12.1202	12.1202	11.3212	

11.6006	11.5008							
ACTBL2	0	0.0388	0.0565	0.2755	1.9649	0.4157	0.1988	
0.0611	0.4726	0.192	0.7344	0.088	0.3266	0.152	0.0537	
0.1859	0	0	0.1015	0.0533	0	0.0587	0.3539	
0.1538	0.2988	0.4122	0.0872	0	0.3977	0.1412	0	
0.0256	0.542	0.0556	0.2128	0.0565	0.0368	0	0.0743	
1.5998	0	0.0666	0.254	0.1878	0.0679	0.5011	0.0459	
0.0925	0.5025	0.0777	0.0333	0.2315	0.2719	0.0429	0	
0.6939	0.5307	0.1924	0.1861	0.7299	0.3748	0.4417	0.4405	
0.8208	0	2.1047	0	0.0422	0.0446	0.2693	0.1378	
0.2216	0.0661	0.0583	0.1266	0.1328	0	0.1073	0.0208	
0.0145	0.0185	0.5303	0.0944	0.0968	0.149	0.2119	0	
0.0513	0							
ACTC1	0	0	0	0	0	0	0.0061	
0.0077	0	0	0.0041	0.0037	0	0	0	0
	0.0464	0	0	0	0.0429	0.0223	0	
0.0088	0	0.0239	0	0	0	0	0	
0.0041	0.0147	0	0	0	0	0	0	
0.0184	0.0098	0.0325	0	0	0.266	0	0	0
	0	0	0.011	0	0	0	0	0
	0.0224	0.1328	0.0172	0	0	0.0046	0.0091	0
	0	0.0807	0	0	0	0.0488	0.0066	0
	0.0161	0	0	0	0	0	5.00E-04	0
	0	0	0.025	0	0.0294	0	0	0
	0							
ACTG1	10.4887	10.6871	11.1332	9.81	9.81	11.2968	10.101	
9.6347	10.9384	11.1332	11.1332	10.6485	10.7746	10.9384	10.9384	
11.4061	10.5176	10.7746	11.1332	9.579	12.5526	9.743	9.6869	
10.6871	9.6347	10.7303	9.4892	12.0577	9.4892	10.32	9.81	
10.1618	11.2968	10.9958	10.7303	11.6205	9.7747	10.8877	9.9643	
10.185	10.9384	10.6485	10.6871	9.7601	10.1618	9.4892	11.0602	
10.7303	9.5614	10.5514	9.9022	9.2164	9.2164	10.2259	11.0649	
10.2416	10.0537	10.2088	11.2251	10.6628	10.3127	6.4705	10.4179	
11.0649	10.8331	10.4426	10.6628	11.149	10.4909	10.3127	11.149	
11.0649	10.5954	10.0388	11.4576	11.3142	11.3142	10.5656	10.5656	
10.2088	11.149	10.0677	10.6287	11.3142	10.6287	9.4629	11.2139	
11.4061	11.4061							
ACTG2	3.1939	1.8493	0.1837	4.9323	0	1.0849	0.2681	
0.0363	0.8736	2.9815	0.2411	0.9928	1.2533	0.8795	0.4328	
0.0935	0.1295	4.8796	1.8087	0.5657	0.8443	0.2476	0.0208	
0.1277	0.0216	5.8702	0	0.2855	0.2525	1.0331	0.1078	
0.0618	0.2975	0.0135	0	3.2479	0.8788	0.0494	0.1193	0
	2.272	0.0205	0.1856	1.0365	4.5728	0.156	2.0129	
0.5579	0	0.4007	0.5515	0.1899	0.4885	0.2582	0.3898	
0.6138	0.3348	0.2861	1.4844	0.4761	0.5955	7.8367	0.6559	
0.5675	0.4363	0.5435	0.4332	0.2683	0.2278	0.2508	0.3342	
0.6366	0.1641	0.47	0.1529	0.3849	0.6067	2.9699	1.0245	
0.4323	0.8137	5.5443	0.2601	0.1926	3.2341	1.1068	1.6959	
0.5035	0.5296							
ACTL10	0.5374	0	0.6459	0	1.0857	0.3766	0.15	

0.0932	0.3534	0.7383	0.0696	0.343	0.3826	0.5563	0.0461	
0.4827	1.0149	0.4923	0.5388	0.2133	0.4317	0.1737	0.2671	
0.2372	0.2957	0.6985	0.603	0.098	0.2131	0.0668	0.0424	
0.3344	0.2627	0.1778	0.0846	0.3047	1.0018	0.2079	0.3315	
1.0262	0.6318	0.3301	0	0.8594	0.167	1.124	0.2614	0
	0.1449	1.2511	0.1015	1.0793	1.1776	1.3621	0.221	
0.3023	0.4536	0.2714	0.1646	0.2749	0.0036	0.3939	0.9244	
0.1276	0.151	0.5255	0.1469	0.5143	0.6956	0.3296	0.3212	
0.7298	1.0804	0.3712	0.2221	0.6169	0.9789	0.0217	0.1914	
0.0512	0.3383	0.5932	0.6114	0.1145	0.2865	1.1316	0.3562	
0.7539	0.6786							
ACTL6A	5.0954	5.8286	6.0053	5.7441	5.0225	5.3687	5.2145	
5.3986	6.0653	5.8438	5.1214	5.2975	5.8076	6.3313	6.5756	
5.077	5.7166	5.659	6.3493	5.062	5.4722	5.9415	5.1423	
6.3545	5.2249	4.6071	5.2645	5.4985	5.6876	5.8548	4.6089	
6.0131	5.821	5.6557	4.7572	6.0391	4.8462	4.9284	5.5567	
6.0653	5.4464	4.9329	6.1451	5.337	6.144	6.3704	6.233	
5.8695	4.0918	6.3752	5.7411	6.3265	5.9841	5.6645	5.3915	
6.0906	6.4696	6.2268	4.9954	6.6187	5.9224	5.8185	5.5061	
6.027	5.4123	4.1976	5.1741	4.7347	5.8654	5.2955	4.9594	
6.2512	5.0865	5.6792	4.8385	5.1075	5.7425	4.8705	5.7211	
5.8344	5.7717	4.6808	3.9603	5.7848	5.9953	5.9383	4.6275	
5.3908	5.0126							
ACTL6B	0	0.0646	0	0	0	0	0	0
	0	0	0	0	0.1057	0	0	
0.0762	0	0	0	0	0	0	0	0
	0	0	0	0	0.0879	0	0.125	0
	0	0	0	0	0	0	0	0
	0	0	0.0828	0	0	0	0	0
	0	0	0	0	0	0	0.1665	
0.1141	0	0	0	0	0	0	0.0169	
0.0307	0	0	0	0.0614	0.0115	0	0.0163	
0.0176	0.1251	0	0.0154	0.0426	0	0	0	0
	0	0	0.0905	0	0	0	0	
0.128	0							
ACTL7A	0	0.038	0	0	0.0424	0	0	0
	0	0	0	0	0	0	0	
0.0198	0	0	0	0	0	0	0	0
	0	0.0221	0	0	0	0	0	0
	0	0	0	0	0	0	0	0
	0	0.0077	0	0	0	0	0	0
	0	0	0	0	0	0	0	0
	0	0	0	0	0	0	0	0
	0	0	0	0	0	0	0	0
	0	0	0	0	0	0	0	0
	0	0	0	0	0	0	0	0
	0							
ACTL7B	0	0	0	0	0	0	0	0
	0	0	0	0	0	0	0	0
	0.2163	0	0	0	0	0	0	0

	0	0	0	0	0	0	0	0
	0	0	0	0	0	0	0	0
	0	0	0	0	0	0	0	0
	0	0	0	0	0	0	0	0
	0	0	0	0	0	0	0	0
	0	0	0	0.0183	0	0	0	0
	0	0.0039	0	0	0	0	0	0
	0	0.0719	0.0946	0.0581	0	0	0	0
	0							
ACTL8	0	0.1739	2.1651	3.6914	1.5042	0.0904	0.0466	
0.0859	0.1643	0.3209	0	0.2091	0.1579	0.507	0	
0.1089	0	0.0848	0.1372	0.6585	0	0.4791	0.1164	
1.5598	0.067	0.0454	0.2051	0	0.1594	0	0	
3.28	0.2828	0.1541	0.0499	0.3877	0.5334	0.4312	4.5368	
1.8351	0.5197	1.8513	0.2673	0.1974	0.0447	0.2824	0.0853	
0.072	0	0.7446	0.172	0.4937	0	0.0969	0.1716	
0.4751	0.0204	4.0722	0	5.1028	0.3785	0.3776	1.1016	
0.1318	2.1148	0.0105	2.7116	0.3674	0.0058	0	0.0358	
0.0122	0.4775	0.4055	0.0103	0	0.2517	0.4847	0.3426	
0.7334	1.1865	0.0043	0	0.017	0	0	0	
0.1243	0							
ACTL9	0	0	0	0	0	0	0	0
	0	0	0	0	0	0	0	0
	0	0	0	0	0	0	0	0
	0	0	0	0	0	0	0	0
	0	0	0	0	0	0	0	0
	0	0	0	0	0	0	0	0
	0	0	0	0	0	0	0	0
	0	0	0	0	0	0	0	0
	0	0	0	0.0257	0	0	0	0
	0	0	0	0	0	0	0	0
	0	0	0	0	0	0	0	0
	0							
ACTN1	7.4954	6.1019	6.1445	6.1457	6.7524	7.3291	6.5601	
5.8796	6.2961	7.1725	5.9931	6.9891	8.0073	6.3113	7.2818	
7.3234	7.8761	6.7135	7.0497	7.2127	6.8446	6.5009	7.4332	
6.9191	6.7524	8.101	4.4704	6.1943	7.691	4.9952	6.4994	
7.2313	6.9445	5.9873	6.3193	6.2663	7.3265	6.1165	6.5503	
6.527	6.3969	7.0002	7.2453	7.7365	7.8619	6.8466	5.9165	
7.6238	7.4004	7.5318	6.2783	6.6579	5.9726	6.6444	5.9842	
6.179	7.7799	5.6739	6.9904	6.5924	7.2077	7.4437	6.33	
6.6928	5.3375	7.2955	6.7725	7.7204	5.9268	7.4234	6.9468	
7.1187	5.7352	6.7385	8.0721	6.777	6.5013	6.4763	6.9904	
6.0594	7.6475	7.4114	7.1221	5.9255	7.0195	6.1106	6.4642	
6.3633	6.0618							
ACTN2	0.0425	0.9413	0.4716	0	0.2831	0.0855	0.806	
0.3466	0.4242	0.3177	0.4738	0.1465	0.3503	0.6748	0.2306	
0.3585	0.0393	0	0.0721	0.4981	0.4896	0.1888	0.4683	
0.5942	1.4738	0.6793	1.4638	0.2019	1.3507	0.1045	0.2476	
0.0867	0.8518	0.6632	0.2692	0.2662	0.5476	0.0736	0.8429	

1.639	0.032	0.0125	0.1777	0.5394	0.1242	0.0236	0.2875	
0.5088	1.6354	0.0586	0.622	0.4079	1.2324	0.0199	0.3911	
0.4696	0	0.1973	0	0.0773	0.9575	0.2071	0.5427	
1.1884	0.413	0.1759	0.7943	0.152	0	0.5001	1.3235	
0.9512	0.5717	0.8576	0.9489	0.2591	1.1575	0.131	0.242	
0.6474	0.5456	0.1817	0.1211	1.4704	0.0484	1.1496	0.2103	
0.4144	0.1781							
ACTN3	0.0337	0.0166	0.0512	0	0.002	0	0	
0.01	0.0103	0	0.0571	0.2089	0	0	0.0186	
0.0193	0	0	0	0.0596	0	0	0.0024	
0.0313	0.0042	0.0084	0.0846	0.0345	0.0408	0	0.1113	0
	0	0.0334	2.00E-04	0	0	0	0.0338	0
	0.1627	0.0236	0.0274	0.0161	0	0	0.0297	
0.1222	0.0236	0.1989	0	0	0.0428	0	0.1299	0
	0.0288	0	0	0.0265	0	0.0351	0.0169	
0.0344	0	0.0052	0	0.0058	0	0	0.0161	
0.0173	0	0.1284	0	0	0.0839	0	0	0
	0.041	0	0.0341	0	0	0	0	0
	0.0492							
ACTN4	6.9035	6.0466	6.6332	7.0447	7.9236	7.2639	6.636	
7.2568	6.6315	6.4076	7.0763	7.2164	8.0956	6.6488	7.5611	
7.259	7.3704	6.6933	7.1683	7.4351	6.9522	7.3315	8.0192	
6.7498	7.6127	7.621	6.3857	6.025	7.617	6.2021	5.9944	
6.9804	7.3484	6.6315	7.821	5.6969	7.3704	5.6978	7.5513	
7.3595	6.4527	7.6018	8.1171	7.5349	8.1563	7.1662	5.5903	
6.405	7.3148	6.6284	6.7095	5.9963	6.5315	7.6442	5.235	
6.904	8.1509	7.0999	6.5804	7.1234	7.3032	6.6749	7.7088	
6.9468	6.2208	7.2638	6.3738	6.8974	7.8708	7.7264	7.106	
7.2852	6.1597	8.1509	6.8756	7.6138	6.4838	6.8417	6.8583	
6.3051	6.904	7.2556	8.1685	6.7772	8.2668	6.5878	6.3857	
6.0223	6.2597							
ACTR10	5.3753	4.5202	4.5953	4.5694	3.9722	4.6188	4.6602	
4.3715	4.6345	5.1323	4.377	4.7932	4.4776	5.2214	4.8934	
4.9893	4.6555	5.2844	5.0453	4.5025	4.7812	4.0896	5.1009	
4.2692	4.1496	5.1844	3.4273	4.861	4.4439	4.5826	5.4914	
4.7023	4.8603	4.6948	4.3896	5.3482	4.993	4.9628	4.7279	
5.0723	4.2741	4.8141	4.2147	4.7123	4.7012	4.9696	4.4953	
4.2824	4.4293	5.5441	4.3161	4.3668	4.6155	4.6908	4.8114	
4.0811	5.0815	4.2628	4.7205	4.1778	4.5767	5.0258	4.5357	
4.2316	4.1403	5.5967	5.2935	5.6498	4.3481	4.2962	4.4909	
4.5628	4.5956	4.4893	4.7671	4.9016	4.8181	4.1953	4.941	
4.0746	4.5521	4.653	5.0088	3.7704	4.6146	4.6455	5.3029	
5.0638	5.1584							
ACTR1A	5.0754	5.8158	5.484	5.2289	5.7315	5.9294	4.7583	
5.4143	5.6545	5.6094	4.8295	5.7879	5.6075	5.7661	5.3099	
5.3726	5.5184	5.1383	4.6538	6.1459	5.9226	4.7283	5.0119	
5.1658	5.4517	5.2772	4.9997	5.9078	5.1	4.8332	6.5912	
5.7096	5.2552	5.7096	5.1945	5.9174	5.8069	5.2805	5.1626	
4.6157	5.5429	4.7937	5.8548	4.9472	5.222	5.7643	4.838	
4.9339	4.9272	5.7296	5.202	4.5307	5.4984	4.5112	6.4498	

4.9597	5.5919	5.8224	4.9302	5.8268	4.8974	6.0165	5.4521
5.6816	5.4888	5.7078	5.7414	6.2927	4.8365	5.3276	5.3904
5.2418	4.9223	4.9471	5.0696	5.4626	5.8124	5.2913	4.4523
5.0611	5.0877	5.4715	5.6806	4.7513	5.5074	5.8676	6.2032
6.1291	6.4102						
ACTR1B	4.4118	4.1509	3.664	3.834	4.6809	4.1633	4.0175
4.4697	4.2106	3.7327	4.1527	4.2275	4.0486	4.2068	2.9876
4.9653	4.5699	3.88	3.5363	4.1693	3.9949	3.8557	4.7011
4.5128	4.1469	3.9545	4.2821	4.2301	4.722	4.3224	4.3406
4.536	4.6165	5.5144	3.7478	3.2937	4.1378	4.767	3.869
3.8721	4.9302	5.1683	5.2441	5.3269	4.2284	4.1717	3.705
4.775	5.1708	3.8808	4.171	3.9897	4.5154	3.8521	3.8565
4.7605	4.5766	3.7108	4.4131	3.3473	4.1916	4.5425	4.0968
4.5425	3.9042	4.6383	4.1325	4.5998	4.0795	4.7025	5.0276
4.5097	3.4339	4.2863	4.5174	3.3344	3.734	3.4587	4.6948
5.0558	4.3362	4.6721	4.5771	4.1526	4.127	4.7312	4.2782
4.5036	4.555						
ACTR2	7.0942	6.4553	6.7792	7.4589	7.2712	7.0198	6.833
6.5437	8.016	7.6408	6.8746	7.5348	7.6408	7.4655	7.1915
6.6751	8.079	7.426	7.1292	6.5402	7.0294	6.6929	7.1837
6.9773	7.0566	6.6492	7.0039	7.2827	7.1791	7.1631	7.2196
6.633	7.3169	7.0782	6.9683	7.5934	7.4007	6.9556	6.4673
7.362	6.9326	7.7394	8.2658	7.3051	7.5411	7.2852	7.362
7.362	7.2599	7.3876	7.166	7.2437	7.2658	7.3917	7.3842
7.1105	7.9071	7.3562	6.7398	7.3842	7.1303	7.0911	6.8647
7.3004	7.74	6.6612	7.8139	7.6383	7.8139	7.4733	7.1789
7.3491	7.2217	7.4147	7.1045	6.7278	6.8595	7.1566	7.1251
7.6122	7.1303	7.52	6.8771	7.0145	6.4713	7.0516	7.1791
7.2467	7.1816						
ACTR3	5.9082	5.6607	5.6375	5.8312	4.9937	6.1881	6.1907
5.8403	5.9245	6.6549	5.4978	5.8993	6.622	6.2618	6.0771
5.5852	5.9863	5.9024	6.2089	5.3711	5.7934	5.48	5.7991
6.0984	5.3603	6.0073	5.3328	5.434	6.0426	5.5088	5.159
5.761	6.881	6.2825	6.0085	6.4766	5.6868	5.6649	5.3741
5.7289	5.1492	6.0724	6.7131	5.8208	6.0198	6.2932	5.7578
6.0256	5.8914	6.4931	5.7749	5.5366	5.8179	5.583	5.6391
5.5375	6.443	5.542	5.6363	5.9475	5.957	6.268	4.8367
6.4601	6.1087	5.3848	6.1714	6.8747	6.3728	5.9869	5.8273
6.3712	5.9645	6.2542	5.7231	5.6762	5.6479	5.6934	5.3965
5.5942	5.8969	5.8706	6.1939	5.1902	6.5194	5.906	6.667
6.1329	6.635						
ACTR3B	2.0476	2.5688	1.6944	2.1444	2.3452	2.2511	1.5318
1.7185	1.3336	1.5418	1.3972	1.1675	0.8823	2.1065	3.0109
1.2627	1.4916	2.2275	2.6783	2.8771	0.7524	1.2774	1.3515
1.8799	1.8617	0.4919	0.2731	1.4213	2.2549	2.5617	2.854
1.3682	1.7149	0.8116	0.3135	2.4801	2.359	1.0832	2.1587
1.6409	1.7489	1.3028	1.9364	1.8454	1.3664	2.4244	2.7669
1.7643	1.2592	1.4325	1.1208	2.1828	1.8602	1.8559	2.9607
2.6961	0.8654	1.4913	2.848	2.0407	1.6281	2.6337	2.866
1.1265	2.6666	0.5567	0.9342	1.283	0.7614	1.8301	1.7619

1.5294	2.1062	1.4283	0.9689	0.9934	1.9174	1.1713	1.3677	
1.9953	0.8654	1.5294	1.1109	1.0908	1.2594	1.3516	0.4437	
0.7785	0.8177							
ACTR3C	1.6599	1.8486	1.2316	1.5929	1.4444	1.4569	1.8225	
1.8209	1.1704	2.8047	1.9965	0.8663	0.2025	0.2928	1.215	
1.458	1.3649	1.4787	1.4576	1.959	1.9703	2.5834	1.6138	
1.5284	2.2483	1.7263	2.3572	1.1964	1.277	1.6988	1.8246	
1.9767	1.4569	0.1066	1.4932	2.5135	1.4473	1.3935	1.0018	
1.5272	1.0754	2.2059	1.3557	1.4355	1.1684	1.8387	1.5681	
0.2948	1.9783	1.4366	1.2316	2.2494	1.7283	1.5499	1.3334	
2.1034	0.8443	1.7408	2.3982	1.9628	1.4886	2.3091	2.4142	
1.1804	1.5464	1.1305	1.3878	0.7348	0.8987	2.2232	1.3445	
0.7237	1.8048	0.4384	0.7856	1.0989	1.8895	1.0345	1.4443	
1.4967	1.4612	1.7013	2.2201	1.6481	1.8997	1.9665	1.1735	
1.0193	0.8282							
ACTR5	2.7771	2.8425	2.7202	1.6878	2.4788	2.1337	2.9168	
2.2737	2.3714	2.8262	2.3998	2.003	2.4738	2.6676	2.453	
2.3685	2.621	2.0803	2.752	2.1738	2.3131	1.7856	2.0041	
2.6763	2.2155	2.456	1.8781	2.1922	2.5328	1.9056	3.0906	
2.7594	3.263	1.8792	1.5262	2.9831	2.0454	2.3664	1.9504	
2.4616	1.8228	1.1663	1.492	2.3351	2.3337	2.2433	2.8808	
2.5256	2.2391	3.2134	2.1165	2.6363	2.8607	2.7219	3.2028	
2.9563	1.9209	2.8391	2.7996	2.9727	2.526	2.7646	2.0712	
2.6083	1.9181	1.1908	1.6073	1.7564	2.7504	2.1412	2.5913	
2.3819	3.2471	1.931	2.4289	2.4564	2.6782	2.197	1.8176	
2.4146	2.3589	1.6953	2.0784	1.4537	2.4067	2.5158	1.6307	
1.5957	1.7136							
ACTR6	3.8795	3.7795	3.2999	4.179	4.3628	3.8014	4.6447	
3.2832	3.9624	4.4162	3.7494	3.749	3.0658	3.5559	3.0715	4.1
	3.5137	4.228	3.9607	3.378	3.6406	4.174	3.9292	
3.143	3.6685	3.2114	4.2346	3.2774	3.708	4.1407	4.2125	
3.4977	3.6566	3.155	3.2091	4.7008	3.6589	3.604	3.4527	
4.0569	4.2065	4.107	3.0691	3.1907	3.7736	3.976	3.1085	
3.5084	3.686	4.0942	3.3323	4.2587	4.1174	3.9368	4.8207	
3.8971	3.5898	3.8098	4.1111	3.5631	3.9383	4.1683	3.3655	
3.8627	3.7542	3.6545	3.5263	3.5438	3.194	2.9074	3.1768	
3.5034	3.9031	3.8993	3.6271	3.085	3.367	3.2871	2.7458	
3.8507	3.7056	2.9189	3.4045	3.4444	3.3936	4.192	3.7757	
3.5869	3.7753							
ACTR8	2.7717	2.9301	3.1847	3.0853	3.3461	3.376	3.5497	
2.8372	3.0391	3.2822	3.1529	3.4096	2.8114	3.0853	2.8744	
3.569	2.542	3.8034	3.2973	3.2207	3.0303	3.0121	3.3573	
3.1128	3.2854	3.4869	3.1439	3.082	3.2369	2.8067	3.1492	
3.0937	3.1215	2.5609	2.9354	3.559	3.2838	3.4355	3.1898	
3.2981	2.5213	2.9723	3.1456	3.3315	3.8851	2.7468	4.1422	
2.9157	2.9823	3.8275	2.826	3.2988	3.3412	2.9752	3.4259	
2.8161	3.2729	3.0424	3.4895	2.8613	3.1626	2.7738	3.4219	
2.5164	3.4427	3.2287	3.3546	3.4559	3.6184	3.0731	3.1578	
2.704	3.5649	2.687	3.6711	3.271	3.1118	3.2269	2.3621	
2.6932	2.6263	2.9151	3.3463	3.5016	3.5756	3.0083	3.5566	

3.3631	3.4421							
ACTRT1	0	0	0	0	0	0	0	0
	0	0	0	0	0	0	0	0
	0	0	0	0	0	0	0	0
	0	0	0	0	0	0	0	0
	0	0	0	0	0	0	0	0
	0	0	0	0	0	0	0	0
	0	0	0	0	0	0	0	0
	0	0	0	0	0	0	0	0
	0	0	0	0	0	0	0	0
	0	0	0	0	0	0	0	0
	0	0	0	0	0	0	0	0
	0							
ACTRT2	0	0	0	0	0	0	0	0
	0	0	0	0	0	0	0	0
	0	0	0	0	0	0	0	0
	0	0	0	0	0	0	0	0
	0	0	0	0	0	0	0	0
	0	0	0	0	0	0	0	0
	0	0	0	0	0	0	0	0
	0	0	0	0	0	0	0	0
	0	0	0	0	0	0	0	0
	0	0	0	0	0	0	0	0
	0	0	0	0	0	0	0	0
	0							
ACTRT3	0	0	1.2633	1.6143	0.8182	0.6334	0	
1.1696	0.0109	0.3255	0.2704	1.0752	0.9813	0.2846	0.7461	
0.3711	2.1783	0.5726	0.0109	0.7996	1.7831	0.496	0.6474	
0.002	0.5466	1.2108	0.0948	0.8522	0.7085	0.1176	2.1625	0
	1.3557	0.1985	0.1366	0.8414	0.569	0	0	
1.2673	0.2572	1.4307	0.5526	0.5362	0.8558	1.1139	1.1703	
0.5929	0	1.2322	1.128	0.1575	1.061	0.126	2.4272	
0.5487	2.2664	0.1493	0.3327	0.1795	0.3077	0.1077	0.4819	
0.7787	0.8118	1.2544	1.3723	1.7465	0.2543	0.9081	0.7315	
0.8618	0.178	0.3095	0.3119	1.0935	0.1958	0.1161	0.1941	
0.1366	0.1402	1.2105	0.677	0.9234	0.5101	0.9551	0.9581	
1.1722	0.9121							
ACVR1	3.7942	2.8786	3.7291	4.2965	3.0029	4.9743	4.8585	
4.8123	3.9003	4.1145	3.3932	4.0186	4.1635	4.7397	4.464	
5.2897	4.3449	4.6097	4.154	3.7859	4.1515	4.2706	4.4182	
4.8361	4.4398	5.373	4.6683	3.491	4.673	3.3211	2.5015	
3.853	4.3824	4.3584	4.9666	3.9565	3.6504	3.3802	4.4351	
4.7706	3.7154	4.3182	5.0295	4.5757	4.1652	4.5741	3.9688	
4.2166	4.5761	3.9643	4.2135	4.4126	4.1553	3.6261	2.795	
3.0023	3.8733	3.3807	4.373	3.8015	4.4443	3.9887	3.5885	
4.7513	3.2153	4.8578	4.2986	4.7238	4.6813	5.1843	5.2262	
4.6484	3.6901	3.7507	4.9284	4.4848	4.6842	4.0915	3.8618	
4.1402	3.6148	4.4578	4.963	4.8768	4.4812	3.7918	3.874	
3.1532	3.2107							
ACVR1B	2.8995	2.9563	2.827	2.9856	2.8169	2.8802	2.7117	

2.454	3.2305	2.8379	2.635	2.581	2.3556	2.6572	2.2388	
3.0608	3.167	2.7308	2.8784	2.4978	2.457	3.0069	2.5479	
2.669	2.9508	2.6355	3.1212	2.7746	2.5031	2.777	3.3995	
2.42	2.8588	2.5241	2.3091	2.7644	3.1302	3.3132	2.8081	
2.7661	2.4477	2.3852	1.5954	2.8387	2.7434	2.3707	3.1346	
3.3282	2.3101	3.8469	2.8807	2.8352	3.1079	3.0155	2.7123	
2.4811	3.002	3.0594	2.3056	3.4211	2.7662	2.2964	3.2203	
2.6056	2.5085	3.3322	2.6874	2.9741	2.7014	2.7644	2.7681	
2.2657	2.9269	2.358	3.0515	2.553	2.618	2.4181	3.0725	
3.2901	3.4286	2.2891	2.7302	2.7106	2.5705	2.8041	2.7153	
2.7337	2.6202							
ACVR1C	1.0471	0.0068	0.7411	0.5898	0.1718	0.7622	0.8772	
0.9643	0.7726	0.7782	0.6421	0.5327	0.0918	1.2694	0.034	
1.4914	0.2701	0.7733	1.0408	0.5658	0.9716	0.0792	0.0616	
1.2523	0.0991	0.5397	0.7863	0.6903	0	1.3443	0.4908	
0.7838	1.0105	1.7681	0.1889	0.2479	0.0028	0.4426	0.0211	
1.2425	1.1292	0.2251	0.1363	0.8509	0.0355	0.7296	0.3	
1.0326	0.6015	0.1189	0.0879	0.4432	0.9171	0.093	0.1718	0
	0.1602	0.1482	1.6441	0.1997	0.7291	0.6881	0.1846	
0.9016	0.4896	0.4121	2.0052	0.42	1.3688	0.2282	1.2362	
0.8222	0.4548	0.1759	0.3044	1.1973	0.9674	0.4108	1.0211	
1.3917	0.9628	0.2867	0.9709	0.4403	0.1159	0.6886	1.0213	
0.8425	1.1409							
ACVR2A	1.2736	1.7197	1.6556	1.9902	1.3197	1.1227	1.64	
1.9229	1.157	1.1672	1.0423	1.7641	1.8427	1.486	1.4574	
0.9894	1.7886	1.2938	1.4126	1.1718	1.7292	1.2802	1.2469	
1.5999	1.2469	1.5568	2.1286	1.7516	1.1258	1.8045	0.8209	
1.7073	0.9982	1.1876	1.1448	1.1813	1.1794	1.8829	1.8602	
1.4762	1.2681	1.815	0.8892	1.4372	1.5883	1.598	1.631	
1.5964	1.4848	2.2041	1.1118	1.7582	1.4704	1.9059	1.3409	
1.7186	1.6967	1.7386	0.9962	1.7136	1.2106	1.1452	1.3395	
1.2818	1.7989	1.8775	2.1435	1.1377	1.9853	1.4816	1.1122	
1.2737	1.8923	1.3279	1.5732	1.4312	1.5414	1.9282	1.6342	
1.4905	1.4532	1.264	1.17	0.7686	1.1193	1.3588	1.1021	
1.3681	1.187							
ACVR2B	1.4354	1.3694	1.9161	1.4427	1.2962	0.9377	1.0456	
2.1575	0.759	2.3598	2.0067	1.8297	0.7214	1.5477	0.5559	
2.5072	2.2332	2.5643	1.9825	1.3631	1.4427	1.1337	0.7181	
1.2726	0.7163	0.295	1.983	1.3725	0.9521	2.2278	2.2057	
1.5706	0.7577	0.7813	0.4471	2.0705	1.561	1.3663	1.9569	
0.9587	1.2216	0.5022	0.5346	1.6757	1.2167	0.6138	1.7595	
1.8884	1.0603	1.7162	1.0153	1.2001	1.4781	2.1141	1.5079	
1.0694	1.1919	1.4132	2.6159	1.3366	1.1207	2.7126	1.6242	
1.3465	1.5481	0.3806	2.6874	0.754	2.5116	0.9931	0.6091	
1.2209	1.4045	1.6946	1.4388	1.0155	1.3266	1.8844	1.0651	
1.5396	1.4506	0.9532	1.4039	1.042	0.9073	1.3254	1.249	
1.3546	1.5682							
ACVRL1	0.0386	0	0	0.3397	0.1322	0.0294	0.1161	
0.0403	1.072	0.1447	0	0.1046	0.0162	0.1137	0.0148	0
	0	0.0809	0	0.0326	0.052	0.318	0	

0.0141	0.1717	0	0.0389	0	0.1846	0.0144	0.0179	
0.3336	0	0.0919	0.7635	0.0059	0.019	0.5863	0.0044	
0.0955	0.3006	1.2799	0.0516	0.0987	0.0322	0.0715	0.0278	0
	0.0065	0.0604	0.0444	0.138	0.0442	0.1035	0.0374	0
	1.5922	0.0708	0.021	1.1897	0.0469	0.0468	0.1394	
0.2983	0.0232	0	0.827	0.0128	0.0709	0.0378	0.0374	
0.0225	0.059	0.1407	0.0162	0.0182	0.0467	0	0.6302	
0.2128	0.2995	0.0919	0.0236	0	0.4193	0	1.0129	
0.5483	0.8255							
ACY1	5.6941	4.5063	3.6744	3.2345	5.2563	3.216	3.564	
4.5436	4.0796	5.96	5.3216	4.871	3.5363	3.8111	2.7661	
3.5176	5.1362	3.869	4.146	3.7789	4.1093	4.001	5.1576	
3.9788	3.3999	3.1027	3.5111	4.5935	4.7578	5.2115	2.9296	
5.2824	3.8558	4.6656	3.925	5.0037	5.8825	5.9919	5.2031	
3.7819	6.7317	5.9811	4.0563	4.791	2.6965	2.5332	4.0284	
4.7302	4.8156	3.9576	3.8913	4.4832	3.4508	3.6821	2.7188	
3.7264	4.2853	3.3436	4.2212	2.8632	5.4819	5.0671	5.0063	
3.8765	5.8206	4.1245	6.0617	3.7256	4.0215	3.4447	4.3458	
3.3522	5.6704	4.5004	4.4472	2.9794	4.0023	4.3685	4.7255	
5.8522	5.2663	5.1894	3.3655	3.9922	4.1942	4.0641	4.3532	
5.0695	4.9093							
ACY3	0	0	0	0	0.0189	0.4944	0.1573	
0.0999	0	0	0	0.0819	0.1415	0	0	0
	0.4434	0	0.1814	0	0	0	0.0193	0
	0.1539	0.0402	0	0	0.4279	0	0	0
	0	0.1276	0.197	0	0.1047	0.0653	0	0
	0.3048	0.6682	0	0.1802	0	0	0.1938	
0.1315	0.124	0	0	0.1231	0	0	0	0
	0.3986	0	0.0574	0	0.1378	0	0	0
	0	0.574	0	0	0	0.0803	0.4362	0
	0	0.0269	0	0	0.1348	0	0.0815	0
	0	0.0283	0.4581	0.0848	0	0	0.0695	0
	0							
ACYP1	4.1927	4.2717	3.5291	4.3971	2.9355	3.1649	3.3727	
4.2282	4.0482	4.6809	3.9086	4.2936	4.4746	3.8267	3.808	
3.1871	3.0131	4.1187	4.3405	3.4909	5.0711	3.525	3.1212	
4.2079	3.4953	3.1186	4.1335	4.6173	4.044	4.5201	5.3288	
3.8568	4.8061	4.653	3.6809	4.6772	3.3543	2.713	3.0565	
4.1668	3.7218	4.0742	4.0986	4.1377	4.5642	3.836	3.7048	
3.4777	2.7175	4.7844	3.247	3.5197	3.8275	2.7216	3.7921	
3.8075	3.53	2.9571	4.048	2.8594	3.9918	4.4679	4.161	
3.5728	2.8651	3.5009	3.9539	3.6793	3.3744	3.1573	2.8739	
4.6114	3.8777	4.1167	2.8586	4.1427	4.8507	3.5124	4.1995	
4.3007	4.3421	2.4377	3.4832	3.7151	3.7432	4.2255	4.1494	
3.974	3.8987							
ACYP2	1.8211	1.7036	1.871	1.7314	1.8387	3.3759	2.3076	
1.9675	2.2624	1.6493	1.7371	2.157	2.2756	1.472	2.0799	
2.0381	1.9457	1.659	2.2418	2.6903	1.8589	1.2054	3.2834	
2.0441	1.9064	2.4262	2.6484	1.5775	3.2031	2.7936	1.6342	
2.582	2.6256	2.0457	3.3484	1.6372	2.8961	1.9993	1.1951	

2.0982	1.4204	3.1381	3.1287	0.9257	3.2159	3.1781	2.072	
1.4708	1.6437	3.1397	1.8496	2.1766	1.2428	2.341	1.5476	
2.6077	3.1148	1.6141	1.2799	1.6231	1.8304	2.5574	2.1738	
3.1014	2.0506	3.0343	1.9558	2.8325	2.5955	2.0497	1.3332	
1.8588	1.8274	2.4376	2.2067	2.3457	2.2067	3.0265	2.5537	
1.2162	1.3579	2.2585	3.7503	1.7211	2.1596	1.1726	2.8362	
2.1221	2.1366							
ADA	3.5172	3.096	3.8459	2.9104	3.6862	2.5972	3.6595	
3.9018	4.887	3.6351	3.9322	4.3293	3.9787	2.8645	2.7543	
3.4634	4.5159	1.7072	2.6637	3.3808	1.8475	4.499	3.5969	
3.6244	3.552	4.3013	5.1086	2.7904	3.3114	3.5504	4.4826	
3.2697	2.7632	3.085	3.0091	3.3977	3.0333	3.9261	3.3525	
4.4906	4.1218	3.2277	2.6831	4.2251	2.3252	1.7891	3.7938	
3.6793	3.2581	3.5161	3.2442	3.075	2.4256	3.5356	5.0039	
3.8471	1.3304	2.6291	2.6503	2.7466	3.8622	3.0245	2.4883	
3.209	3.3573	3.2394	4.3146	2.58	3.1431	2.1945	4.7968	
4.0177	4.5011	3.6683	3.8901	2.756	2.7099	4.1822	3.1583	
4.6512	3.7077	5.8869	2.5254	3.3151	2.7114	2.9161	1.3657	
1.877	1.9519							
ADAD1	0	0	0	0	0	0	0	0
	0	0	0	0	0	0	0	0
	0	0	0	0	0	0	0	0
	0	0	0	0	0	0	0	
0.1098	0	0	0	0	0	0	0	0
	0	0	0	0	0	0	0	0
	0	0	0	0	0	0	0	0
	0	0	0	0	0	0	0	0
	0	0	0.0712	0	0	0	0	0
	0	0.0021	0	0	0.0134	0	0	0
	0	0	0	0	0	0	0	0
	0							
ADAD2	0	0	0	0.1344	0	0	0	0
	0	0	0	0	0	0.1344	0	
0.0349	0	0.1002	0	0	0.0689	0	0	0
	0	0	0	0	0	0	0	
0.0076	0	0	0	0	0	0	0	0
	0	0	0	0	0	0	0	0
	0	0	0	0	0	0	0	
0.1233	0	0.0503	0	0	0	0	0	0
	0	0.0035	0	0	0	0	0	0
	0	0	0	0	0.1454	0	0.0107	0
	0	0	0	0	0	0.0757	0	0
	0							
ADAL	2.6633	3.5766	1.9651	2.0792	2.3255	3.3965	3.2438	
2.6809	3.19	2.6041	2.7986	2.0046	2.349	2.674	1.9355	
3.653	2.3261	2.8541	3.1478	2.5441	2.2146	2.0481	2.891	
3.2356	2.1846	2.6671	2.8767	3.2036	2.6384	3.4651	1.9998	
2.6194	2.2246	2.6751	3.1905	3.0361	2.7131	1.8118	2.5643	
2.5493	2.7001	2.2964	1.8027	2.9881	2.5504	2.6076	2.7048	
2.8392	2.3365	2.5914	2.5934	2.417	2.4005	3.2888	2.7992	

3.2016	0.5792	2.1414	2.6208	2.1115	2.5694	2.4829	2.1465
2.8906	2.0974	2.0022	2.6954	1.8333	2.6744	1.9931	2.2101
2.6393	2.9457	2.6413	3.2895	3.1453	2.2584	3.1866	2.6369
2.5942	2.7503	2.2658	2.4796	2.9356	2.8677	2.3745	2.1648
2.2492	2.1856						
ADAM10	4.943	5.7313	5.3229	5.0272	5.4567	4.9729	5.679
5.0659	6.9122	6.2146	5.9896	5.0117	4.4033	5.0336	5.101
5.8579	4.9482	5.8209	5.7419	5.5487	5.8754	6.3066	6.482
5.463	5.6156	4.7394	5.5931	4.9992	6.4343	5.8419	4.0834
5.205	5.2575	7.0037	5.8849	5.7865	5.2505	6.2442	6.23
5.7737	6.1339	5.1987	5.8671	5.4132	5.7894	5.5112	5.6772
5.9323	5.4142	5.2328	6.0634	5.9211	6.2993	5.6648	3.8649
5.4177	5.4472	5.0957	5.8138	5.2086	6.7437	5.9679	5.5605
5.6717	5.0204	5.8501	5.779	4.7924	5.4617	5.2027	4.987
6.1503	5.0946	6.0957	5.7982	5.4429	5.8747	6.0824	6.1613
6.5368	5.6393	6.3358	5.4399	7.0796	5.2414	5.9059	6.2357
5.9376	6.6128						
ADAM11	3.2044	3.0553	2.4689	0.742	0.8346	0.846	0.4148
1.726	0.8817	0.6794	2.4849	0.3896	0.8454	0.7408	2.0074
1.9372	0.8645	1.4026	0.7584	1.1125	1.935	0.7879	0.5454
0.448	0.9886	0.2546	1.9705	1.7195	1.02	1.2041	1.2425
0.5602	0.6702	0.732	0.4072	0.7541	1.0046	2.4647	1.2667
0.7746	1.6683	0.104	0.4224	0.763	1.2102	0.6555	1.3207
1.0129	1.2464	0.3288	1.0889	0.3357	0.7493	1.127	1.2061
2.1564	0.0826	1.2149	0.9033	0.6855	2.0485	0.5828	1.3438
1.0912	2.1817	0.1971	0.6683	0.8295	0.6739	0.7573	1.4499
1.0674	2.2589	0.5045	0.9518	0.5307	1.6102	1.2948	0.938
2.2389	1.1826	0.2353	0.2993	1.879	0.3544	0.8884	0.3726
0.9202	0.8163						
ADAM12	0.5483	0.5017	0.1792	0.729	0.7858	0.9208	0.6471
2.2899	0.6165	0.1751	0.6306	0.6145	3.4699	0.5987	3.8416
0.2969	0.89	0.8269	0.1352	0.6338	0.5673	1.0284	0.2108
0.1599	6.6971	1.1712	1.2963	0.3597	1.6027	0.1785	0.1378
0.2794	0.7418	0.2919	0.3482	0.1759	0.3828	0.5682	1.262
3.6215	0.9043	2.5665	5.1757	0.7059	1.8327	1.7798	0.5721
1.764	2.276	1.1263	0.3678	2.941	1.0117	0.2773	3.2431
0.0515	0.9252	0.0134	0.0923	0.043	3.4582	0.3324	3.6739
0.415	2.5258	3.5988	0.2105	0.559	0.2719	2.7655	2.2931
0.8532	0.7422	1.3051	0.2278	0.2815	0.5671	0.4523	0.2358
0.0968	0.1776	4.0257	1.3541	3.8775	2.8385	0.3935	1.2453
0.8463	1.9562						
ADAM15	6.0936	4.3884	5.3947	4.9154	5.6302	6.1838	5.5873
4.3786	4.7406	4.8025	5.414	4.4901	4.6535	3.5069	5.3018
5.4758	3.9106	3.6057	4.5558	5.8436	5.6188	5.561	5.618
4.8343	4.3386	4.7342	4.2638	4.0349	5.5729	5.3721	5.4767
4.815	5.4432	5.9068	4.6895	3.8134	5.4933	5.6155	4.9422
5.2866	6.1903	4.8759	5.3388	4.6374	4.2688	5.492	5.087
4.6326	4.1981	3.8305	4.2112	5.0421	4.1111	4.1877	4.4948
4.0755	5.6116	5.1191	4.4001	5.2115	5.098	4.7721	5.988
4.5261	5.494	6.7403	5.2042	4.8781	4.3026	5.6676	5.0167

4.4523	5.455	3.5818	5.1757	4.0949	4.9395	5.9976	5.4734	
5.1272	3.9822	5.4804	5.1151	5.6152	4.4379	4.7367	5.3353	
5.5092	5.3618							
ADAM17	3.6109	4.1868	3.9523	4.676	4.337	3.4589	3.9402	
3.3951	4.7044	4.2669	4.0267	4.1861	4.5911	3.8987	3.5567	
2.7973	5.4026	3.8368	4.1262	3.374	3.7882	5.0043	4.7068	
4.369	3.9608	3.9954	4.4607	3.6779	4.5571	4.4934	3.5646	
3.8748	4.2439	4.1096	2.9109	4.0982	4.5617	3.9671	3.7594	
4.5466	4.4906	4.4055	4.2134	3.889	4.7543	4.4435	4.5709	
4.6685	4.2166	4.3188	4.9827	4.8716	4.3593	4.4032	4.1216	
3.8288	4.8621	4.5413	3.2645	4.3788	4.3174	4.1013	3.7512	
3.8327	4.9901	4.483	4.5897	3.8157	5.1094	4.0525	3.5083	
4.1963	3.8731	3.7624	3.9551	3.1815	4.1852	4.4372	3.5073	
4.4958	4.0378	5.1901	3.8114	4.0859	3.5353	3.7552	3.8849	
3.0101	3.4316							
ADAM18	0	0	0	0	0.0511	0	0	0
	0	0	0	0	0	0	0	0
	0	0	0	0	0	0.112	0.0207	0
	0	0	0	0	0	0	0	0
	0	0	0	0.0651	0	0	0	0
	0	0.0138	0	0	0	0	0	0
	0	0	0	0	0	0	0	0
	0.0311	0	0	0	0	0	0	0
	0	0	0	0	0	0	0	0
	0	0.0486	0	0	0	0	0	0
	0.0122	0	0	0	0	0	0	0
	0							
ADAM19	0.6487	0.5676	1.8197	3.4975	6.0352	3.5817	3.8563	
3.0011	1.4681	0.3573	0.9844	4.0926	3.4242	0.2209	2.8642	
2.7735	6.0505	1.3656	1.3858	4.9089	0.8227	4.0022	3.757	
2.1756	3.8789	4.6445	0.8221	0.9664	4.1139	0.9608	1.6221	
4.093	3.356	0.2	4.462	0.4905	0.8111	0.4173	1.25	
2.1972	3.3473	4.0438	5.0785	1.9982	2.6984	1.9204	0.6433	
1.6887	2.3102	1.8769	0.8057	1.9221	2.0428	0	1.5117	0
	5.2334	0	1.737	0	4.4283	1.2952	0.1015	
2.6547	0.3937	2.2977	1.4383	3.1089	1.477	3.2503	4.8206	
3.2541	1.2319	4.7229	4.5375	2.2372	0.7802	3.5007	2.1606	
2.2342	1.0845	4.5034	3.9434	5.2646	2.6113	1.8252	0.7408	
0.2238	0.2473							
ADAM2	0	0	0	0	0	0	0	0
	0	0	0	0	0	0	0	0
	0	0	0	0	0	0	0	0
	0	0	0	0	0	0	0	0
	0	0	0	0	0	0	0	0
	0	0	0	0	0	0	0.0299	0
	0	0	0	0	0	0	0	0
	0	0	0	0	0	0	0	0
	0	0	0	0	0	0	0	0
	0	0	0	0	0	0	0	0
	0	0.0251	0	0	0	0	0	0

	0							
ADAM20	0	0.0288	0	0.0386	0.0018	0.0392	0.0135	
0.0165	0	0.0611	0.0022	0.0156	0	0	0.0114	
0.0148	0.0489	0	0.0388	0	0	0	0.0078	0
	0.0031	0.0155	0.0801	0.0304	0	0	0.0184	0
	0	0	0.0032	0.0347	0.0021	0.0198	0.0055	
0.0521	0	3.00E-04	0.0365	0.0134	0.0047	0.0345	0	
0.0168	0.01	0.1296	0.0103	0.0829	0.0312	0.0123	0	
0.0653	0.0358	0.0319	0	0	0	0.0156	0	0
	0	0.0047	0	0.0053	0.06	0	0.0169	0
	0.0261	0.0039	0.0437	0.0306	0	0	0	0
	0.0189	0.013	0.0395	0.1025	0	0	0	
0.0592	0.0295							
ADAM21	0.5088	0.074	0.6228	0.2507	0.1014	0	1.088	
0.0247	0.0257	0.1229	0.4828	0.2216	0.0104	0.1366	0.0091	
0.0217	0.1891	0.0453	0.1548	0.0815	0.0888	0.1375	0.1442	
1.006	0.079	0.2755	0.0572	0.2533	0.0294	0.5152	1.1624	
0.2846	0.4093	0.9773	0.026	0.1028	0.1357	0.1137	0.0235	
0.1266	0.7444	0.5073	0.3822	0.0731	0.0305	0.1099	0.045	
0.1989	0.0317	0.4647	0.4312	0.5152	0.2801	0.2319	0.3518	
0.2413	0.2992	0.1702	0.0157	0.1795	0.2983	0.0292	0	
0.1516	0.1907	0.3459	0.7461	0.2261	0.1147	0.1049	0.051	
0.1188	0.0395	0.8552	0.1612	0.1669	0.448	0.8944	0.0806	
0.1072	0	0.843	0.2162	0.2097	0.2603	0.0432	0.2213	
0.4144	0.4309							
ADAM22	1.9695	0.9952	1.4999	0.9625	1.9991	1.6299	1.6713	
2.1577	0.408	0.8797	0.7918	1.5788	0.254	2.1466	2.1294	
1.5254	2.3317	0.1993	0.5285	1.9607	0.5685	2.4452	2.476	
2.3253	2.1649	0.0846	2.364	1.8763	0.3203	1.1451	2.2774	
1.2614	0.7475	0.2628	2.1654	1.3776	0.9046	1.0303	0.6266	
1.8538	0.0913	1.2791	1.2959	2.1379	1.3796	0.8166	1.1706	
2.279	1.8763	2.1961	1.3789	2.5797	1.1712	0.3976	1.8552	
1.0576	0.615	2.8512	0.2666	3.0119	1.6628	0.2121	1.2785	
1.9828	1.2511	0.3986	1.3785	0.4968	1.2772	0.6742	1.7396	
2.001	1.9422	1.0795	2.8048	1.5582	1.325	3.1715	1.5334	
2.0134	1.9593	1.3425	1.1056	0.8343	0.5762	0.9487	0.5786	
0.8039	0.6892							
ADAM23	4.783	3.2422	3.086	3.2163	3.2068	4.8338	3.8293	
1.7819	3.8686	3.2304	3.033	3.3789	0.5893	4.7167	3.6832	
4.0869	2.452	0	3.8881	4.2557	4.4561	2.013	3.7662	
5.5232	2.6193	0.5403	2.1452	3.0593	4.0497	3.2103	2.9431	
0.2332	4.5559	5.3912	5.1051	3.1015	3.2268	4.7715	2.3049	
4.8351	3.7991	0	3.1732	2.6661	0.2135	0.2132	3.6355	
3.7317	0.5773	0.5893	4.0244	0.1043	4.1149	2.1506	2.6844	
2.9466	2.4982	5.714	0.1665	2.9155	3.6154	0.4561	4.9434	
0.7428	5.1291	4.3822	6.5211	2.7931	3.2891	2.3466	2.1733	
1.3465	3.4012	4.1374	3.563	2.8736	2.6683	2.6086	3.8933	
3.8336	3.4587	1.7241	4.2051	3.8186	0.2425	3.9072	3.9046	
2.8153	2.0294							
ADAM28	0.0464	0.0493	0	0	0.0076	0	0.1449	0

	0	0	0.0571	0.0553	0.1707	0	0.0122	0
	0.0801	0.0492	0	0.0337	0	0.008	0.052	0
	0.0361	0	0.0796	0.0441	0	0	0.0342	0
	0.0479	0.0196	0.0484	0	0	0	0.0138	
0.0277	0.0656	0.1742	0.0489	0	0.1385	0.1275	0.0535	0
	0.1537	0.3313	0.0212	0.077	0	0	0	
0.0594	0.1632	0.0652	0	0.0511	0.0598	0.1252	0.02	0
	0.0313	0.054	0.0516	0.1278	0.0572	0.09	0.1588	
0.1301	0	0.0137	0.0166	0	0	0.0171	0	0
	0	0.0331	0.0133	0.0454	0.0539	0	0.0879	0
	0							
ADAM29	0	0	0	0	0	0.0306	0.0071	0
	0	0	0.004	0	0	0	0	0
	0	0	0	0	0	0	0	0
	0	0	0	0	0	0	0.0974	
0.0011	0	0	0.0226	0	0.0037	0	0	0
	0.0121	0.0015	0	0	0.0071	0.0154	0	0
	0	0	0	0	0	0	0	0
	0.0398	0.0956	0	0.0145	0	0	0	0
	0	0	0	0	0	0	0	0
	0.0123	0	0	0	0	0	0	0
	0	0	0	0	0	0	0.0267	0
	0							
ADAM30	0	0	0	0	0	0	0.011	0
	0	0	0	0	0	0	0	0
	0	0	0	0	0	0	0	0
	0	0	0	0	0	0	0	0
	0	0	0.0021	0	0	0	0	0
	0	0	0	0	0	0	0	0
	0	0	0	0	0	0	0	0
	0	0	0.0224	0	0	0	0	0
	0	8.00E-04	0	0	0	0	0	0
	0	0	0	0	0	0	0	0
	0	0	0	0	0	0	0	0
	0							
ADAM32	0.0693	0.0433	0.0365	0.1414	0.0655	0.0524	0.0632	
0.0917	0.0125	0	0.1083	0.075	0.3019	0	0.074	
0.0402	0.5068	0.071	0.039	0.3084	0.0383	0.0156	0.092	
0.1373	0.2803	0.1434	0.1383	0	0.0464	0.0318	0.152	
0.0066	0.0828	0.1271	0.117	0.0231	1.5382	0.1445	0.1569	
0.1769	0.13	0.1528	0.1875	0.0634	0.1905	0.0698	0.0606	
0.0693	0.1631	0.3648	0.0805	0.0694	0.0323	0.1184	0.0375	
0.083	0	0	0.1039	0.3568	0.0705	0.0555	0.3796	0
	0.3908	0.1861	0.1314	0.2338	0	0.484	0.013	
0.069	0.0186	0.0837	0.2283	0.0814	0.0329	0.2331	0	
0.0879	0.0184	0.337	0.0159	0	0.2392	0.1046	0.1737	
0.4022	0.1561							
ADAM33	0	0.0078	0.1786	0	9.00E-04	0	0.0505	
0.074	0.0039	0.0053	0.1173	0.0787	0.1275	0.1406	0.0731	0
	0	0.0986	0.3042	0.2312	0.1337	0.2461	0.0248	

0.0304	0.0178	0.4464	0.1373	0.1849	0	0.063	0	
0.0668	0.0083	0	0.0214	0.0487	8.00E-04	0	0.083	0
	0.0522	0.1413	0.0867	0.0083	0.031	1.5711	0.1325	0
	0.0447	0	0	0	0.0226	0.0754	0.0175	
0.2588	0.0542	0	0.013	0.1541	0.0106	0	0.2245	
0.1466	0.1471	0.7511	0.0899	0	0	0.1867	0.0419	
0.0407	0.1126	0.2298	0.2349	0.011	0	0	0	
0.1914	0.0926	0.1281	0.0786	0.0168	0.0281	0	0.9545	
0.0938	0.5917							
ADAM7	0	0	0	0	0	0	0	0
	0	0	0.0021	0	0	0	0	0
	0	0	0	0	0	0	0	0
	0	0	0	0	0	0	0	0
	0	0	0	0	0	0	0	0
	0	0	0.0175	0	0	0	0	0
	0	0	0.0152	0	0	0	0	0
	0	0	0	0	0	0	0	0
	0	0	0	0	0	0	0	0
	0	0	0	0	0	0	0	0
	0	0	0	0	0	0	0	0
	0							
ADAM8	1.1332	0.4579	0.7555	0.4756	0.534	0.0896	0.4896	
0.1614	0.2297	1.1344	0.1072	2.3381	0.4172	0.0799	0.0729	
0.2832	0.4804	0.579	0.0893	0.6074	0.5124	0.1073	0.1937	
0.0479	0.051	0.6458	0.0673	0.4774	0	0	0.2802	
0.5258	0.0216	0.1062	0.0431	0.2746	0.1987	0.0262	0	
0.1534	1.0857	4.9782	0.1646	0.1378	2.8203	0.237	0	
0.2724	0.2467	0.7666	0.5213	0.0941	0.2984	0.3466	0.8437	
0.3131	5.1309	0.2203	0.1361	0.2853	0.3228	0.5281	0.4379	
0.2765	0.3483	0.253	0.9136	0.2328	0.3265	0.4105	0.1122	
0.0825	0.1703	0.1437	0.1291	0.0658	0.7449	0.256	0.0867	
0.044	0.2404	1.6056	0.1624	0.1186	0.5598	0.2798	0.3409	
0.2231	0.2567							
ADAM9	6.4087	4.6157	4.2532	5.7886	5.1273	5.3221	5.2203	
5.7087	5.996	3.2356	4.5647	5.4345	6.3168	5.8027	5.0953	
5.438	6.4395	6.2188	5.7019	5.6575	5.869	5.8038	6.1039	
6.2227	6.7477	7.0394	4.9622	4.5186	5.5092	5.1408	6.2342	
5.2163	5.1824	6.2462	6.6791	5.0736	5.1918	5.425	5.3412	
6.5216	6.1099	6.7683	6.577	4.7891	7.3492	7.2676	5.6017	
5.4548	5.7412	6.7558	6.2019	6.4745	5.7063	4.5928	6.2136	
4.3464	6.0677	5.7367	5.8366	6.5092	5.3598	5.925	6.8414	
5.273	6.3443	6.1392	6.792	6.0876	4.3319	6.8522	6.7558	
4.7333	5.2696	5.7044	6.8846	5.6213	5.035	6.7856	4.6378	
5.3415	4.8161	6.4994	4.8314	6.307	6.286	5.59	6.1948	
5.5467	5.609							
ADAMDEC1	0	0	0	0.0516	0.0219	0	0	0
	0	0.0155	0	0.005	0	0	0	0
	0	0.0283	0	0.0347	0	0.0034	0.0053	0
	0.028	0.0114	0	0	0.0169	0	0	
0.0022	0	0.0156	0.0022	0	0.0053	0	0	0

	0	0	0	0	0	0	0.0275	0
	0.0159	0.0565	0.0146	0	0.0421	0.0208	0.0229	0
	0	0	0.0165	0	0	0.0098	0	0
	0	0.028	0	0.0215	0.0418	0	0	0
	0	0.014	0	0.0226	0.0041	0	0	
0.0638	0.0125	0.0343	0	0	0	0	0	
0.061	0							
ADAMTS1	2.0789	1.6647	1.0726	2.4274	2.3706	2.4129	5.0037	
4.4625	3.5629	2.0899	3.1386	2.8583	3.4907	4.5821	4.6858	
1.9984	6.1741	1.6861	3.6635	2.9629	2.9669	5.5881	3.6217	
2.0316	3.3592	6.8293	5.4418	1.7747	4.297	1.6517	0.3238	
2.8003	1.8357	3.1238	3.6305	1.9551	5.0154	2.7646	2.3234	
4.5384	2.5597	1.5902	4.3296	2.8923	2.0537	2.6135	3.9606	
3.8194	4.7436	6.2076	3.8546	5.3466	4.697	3.9446	0.1605	
2.5631	7.0085	2.4137	3.8282	1.9184	4.0503	3.2157	2.839	
3.5664	2.9457	4.8182	2.7253	0.9052	1.5067	4.515	3.5027	
3.8508	4.4417	1.9809	2.4548	1.9342	2.4859	1.9711	1.5737	
2.6548	1.7284	4.515	3.1108	5.7808	2.578	3.8712	1.6587	
1.4155	1.1803							
ADAMTS10	0.1098	0.0646	0.0958	0.1423	0.0743	0	0	
0.0452	0.1066	0	0	0.0126	0.1957	0	0.0355	0
	1.369	0	0	0	0	0.0712	0.0877	0
	0.0145	1.3699	0.0389	0	0	0	0.5465	
0.0077	0	0	0.0347	0.0811	0	1.7031	0.1953	
0.1274	0	0.0633	0	0	0.1599	0.1765	0	0
	0.0363	0.5539	0.0607	0	0	1.3294	0.3785	0
	0	0	0.025	0.0482	0.0071	0.0263	0.0116	
0.0159	1.2377	0.025	1.5707	0	0.0079	0	0.0051	0
	0.3523	0.0679	0	0	0.0021	0	0.0102	
0.0333	0.0543	0.0739	0	0.1102	1.5789	0	0.165	
0.3801	0.7811							
ADAMTS12	0.0114	0.0032	0.0978	0.0704	3.1114	0.1549	0	
0.1409	0.252	0	0.8888	2.5164	0	0.0232	0.0611	
0.4174	1.8785	0.0575	0.8311	0.0836	0.2269	0.6121	0.9642	0
	1.1096	5.3804	0.6715	0.059	0.2274	0	0.0128	0
	0.0032	0	0.6439	0	0.1553	0.163	0	
0.009	0.7383	5.1446	4.0529	0.0494	0.1281	0	0	
0.787	0.39	3.6777	0.1085	0	0.0143	0.0512	0.0578	
0.1911	3.9007	0.3152	0.7218	0	0	0.4302	0	
0.0617	0	4.2321	0	0.1966	0.219	0	0.0541	
0.042	0.0932	3.4394	0.3227	0.0462	0.091	0	0.0959	0
	2.2797	1.3104	0.0858	0.0489	0.1801	0.1609	0.6647	
0.3868	0.238							
ADAMTS13	1.9304	0.3797	0.7052	0.1403	0.8335	0.2612	0.8315	
1.3615	0.5347	0.8165	2.2221	0.3573	1.1632	0.8389	0.908	
0.4338	0.53	1.1186	0.0939	0.5913	0.7457	0.2412	0.594	
0.6518	0.9374	0.8206	1.1283	1.2714	0.3943	0.8455	3.3108	
1.0557	0.4978	0.8147	0.7852	0.5695	1.7351	0.8284	2.043	
1.4397	1.6781	0.7977	1.4343	1.9887	0.2981	0.7669	0.8068	
1.0374	0.6091	1.2633	0.8259	0.2783	0.5291	1.5123	1.4554	

0.5004	0.9702	0.1796	0.3629	0	0.9639	0.3296	0.4368	
0.8899	2.1681	2.4784	1.5228	0.826	1.8342	1.1436	0.6543	
0.2561	0.8568	0.2982	0.6366	0.1573	0.6107	0.6771	0.6471	
0.9387	1.2312	0.9633	0.2794	2.0744	0.5058	0.761	0.8792	
0.7923	1.384							
ADAMTS14	0.1206	0.0436	0.2495	0	0	0	0.0256	
0.0802	1.0248	0.216	1.0874	0.205	0.5393	0	0.1352	
0.3954	0.0427	0.0533	0.178	0	1.1452	0.0995	0.0141	
0.0326	0.2765	1.5248	0.2798	0.1071	0.1002	0.0383	0.201	
0.0399	0.0277	0.0347	0.0639	0.1291	0.0192	0.0739	0.4315	
0.0269	0.0323	0.0893	0.4672	0.9155	1.2036	0.577	0.3819	
0.0308	0	0.0929	0.4536	0.2347	0.055	0.8803	0.0551	
0.109	0.0263	0.1993	0	1.0327	0.1778	0	0.0097	
0.5985	0.0726	1.6776	0.0191	1.4348	0	0.0281	0.1969	
0.6471	0.0667	0.7792	0.1891	0	0.0426	1.1709	0.0534	
0.0097	0.2638	0.3414	0	0.4118	0.3577	0	0.2929	
0.182	1.2527							
ADAMTS15	0	0.0958	0.0735	1.148	1.3782	0.1217	0.0363	
0.049	0.2383	0.0199	0.0108	0.3512	0.7036	0.0524	2.5567	0
	0	0	0.0678	0.1084	0.3822	2.2569	1.0734	
1.5389	0.9338	0.6881	0.5114	0	1.9138	0.0133	0.6448	
0.0621	0.1241	0.0396	1.3682	0.0148	0.231	0.2778	0.4792	
0.4297	0	1.1489	1.0084	0	0.458	0.6101	0.0123	
0.8466	0.3238	0.042	0.3228	0.893	0	0.0634	0.0616	
0.1579	0.0354	2.6184	0.0366	3.0941	0.6466	0.3183	0.1281	
0.0398	0.148	0.1332	0.0559	0.0328	0.0602	1.3095	0.0432	
0.2153	0.0335	1.4432	1.4695	0.0272	0.0342	1.5919	0.0565	
0.046	0.0446	1.0454	0.0334	0.4808	1.2244	0.1724	0	
0.0225	0.0192							
ADAMTS16	0.1208	0.389	0.0377	0.533	0.6218	0.1371	0	
0.0442	2.3752	0.1467	0.2472	0.2572	0.4261	0.1009	0.2323	
0.4797	1.6538	0.4619	0.1628	0.093	0.5029	0.7254	0.155	
1.047	0.1096	0.0156	0.042	0	1.2431	0.1352	0.0209	
0.1613	0.2567	1.2016	0.049	0.1004	0.0292	0.0583	1.8528	
0.0571	0.0885	0.9001	2.2042	0.1258	0.0168	0.6905	0.1073	
0.0698	2.3717	0.4855	0.0689	0.4538	0.5934	0.017	0	0
	1.821	0.0388	0.8925	0.0038	1.0636	1.5604	0.8737	
0.1968	0.0463	0.0378	0.1335	0	0.2175	0.4735	0.1761	
0.7396	0.0691	0.7816	0.6056	0.0149	0.2325	2.6603	0.6652	
0.0907	0	0.5581	0.3137	0.4665	0.0503	0.6955	0.0376	
0.0404	0.1832							
ADAMTS17	0.3363	1.0077	1.5803	0.4842	0.3097	0.4456	0.9619	
0.7387	1.4579	1.5408	0.5687	0.223	0.2266	0.488	0.6906	
1.1766	0.1707	1.3064	1.3707	1.0083	0.2836	0.4422	0.3708	
0.4939	0.6953	0.1399	0.3689	1.1409	1.2658	0.915	0.3208	
1.2208	3.7875	0.3016	1.129	0.3055	0.5619	0.3232	1.5141	
1.9533	0.2485	0.1096	0.4842	0.9102	0.3324	0.319	0.7278	
0.2828	1.4055	0	0.4497	0.3684	0.6497	1.4951	0	
0.9312	0.2099	2.1011	0.9605	1.7532	1.4832	1.2049	0.8098	
1.2953	0.1968	0	0.5168	0.3805	1.6154	0.4777	0.5017	

0.7144	0.0485	0.2145	1.0607	1.515	0.6291	1.122	0.7806	
1.4632	0.6147	1.1015	0.2521	1.0225	0.407	0.5864	0.405	
0.3832	0.3055							
ADAMTS18	0.1695	0.1325	0	0.0308	0	0	0.0164	
0.0146	0.0915	0.2012	0.0138	0.0135	0.0136	0.0487	0.0269	
0.043	2.6522	0	0	0.0306	0	0	0	
0.0553	0.0164	0.0145	0.0207	0	0	0.0276	0.014	0
	0	0	0	0.0891	0	0	0.2882	
0.0321	0.0163	0.0148	0.0272	0	0.0136	0.0183	0	0
	0.0697	0.7957	0	0	0	0.024	0	
0.1781	1.1524	1.0958	0	0.3671	0	0.8721	0.0601	0
	0	0	0	0	0	0	0.0068	
0.0011	0.0493	0	0.0072	0	0	0	0	0
	0.1707	0	0	0	0.0019	0	0	
0.0393	0							
ADAMTS19	0	0	0	0	0.0113	0	0.085	
0.0367	0.0072	0	0.0069	0.0033	0	0.0322	0	0
	0.0553	0	0.0343	0	0	0	0	0
	0.008	0	0	0	0	0.0114	0	
0.106	0.0131	0.0098	0	0	0	0	0	
0.1165	0	0.0046	0	0.0126	0.2482	1.1413	0.0256	0
	0.0614	0.0381	0	0	0	0	0	0
	0.0224	0.1195	0.0136	0.1171	0	0.0205	0.0533	0
	0	0	0	0.0541	0	0.0807	0	
0.0691	0	0	0.0265	0.3622	0	0	0.0094	
0.4139	0	0	0.0039	0.0169	0	0	0.0178	0
	0.0515							
ADAMTS2	0.0916	0.1548	3.6781	0	0.0958	0	0	0
	0.9543	0.1591	0	0.0532	0.0385	0.1495	0.1397	
0.0629	0.059	0.2548	0.305	0.8096	0.2665	0.2365	0.2848	
0.6633	0.4953	0.866	0.0862	0.117	1.542	0.3245	1.9827	
0.1877	0.0853	0.1201	0.5919	0.1396	0.2363	0.1292	0.0647	
0.4931	0.067	0.0871	0.8384	0.0966	0.3146	0.6447	0	
0.6112	0.439	0.1206	0.1549	0.1468	0	0	0.2941	
0.6126	1.3595	2.9089	0.2504	0.0664	0.0278	0.3058	0.4364	
0.0168	0.556	0.045	0.0668	0.0727	0	2.7628	0.0089	
0.3284	0.3049	1.5087	0	0.0043	0.2644	0.2433	0.0089	
0.0956	0.2032	0.4393	0	0.2924	0.0997	0	2.0287	
4.2921	5.9388							
ADAMTS20	0.0127	0.7409	0	0.0322	0.691	0.0136	0.2588	0
	0.2209	1.071	0.3921	0	0	0.0245	0	
0.0039	1.3728	0	0.4102	0	0	0.3158	0.0921	
0.0171	0.1009	0.0172	0.4799	0	0.0725	0.0909	0.32	
0.4212	0.0515	0	0.2988	0.2722	0.4191	0	0.1088	
0.1414	0.2503	0.3552	0.01	0.0554	0.0259	0.1178	0.3408	
0.0047	0.0437	0	0.0127	0.0403	0.3906	0.1156	0.1349	
0.1139	0	0.529	1.5585	0.187	0.201	0.5488	0.0958	
0.0658	0	0.0257	0	0.0617	0.3375	0.1496	0	
0.1627	0	0.1263	0.032	0	0.0602	0.0463	0.0617	0
	0	0.2586	0.072	0	0	0.4283	0	0

	0.0215							
ADAMTS3	0	0.1557	2.00E-04	2.43	1.4429	1.3295	0.5733	
0.9866	0.1594	1.1423	0.5388	0.927	0	2.2766	0.8543	
0.2593	1.1167	0.0516	0.1777	0.9566	0.9265	2.1167	0.3538	
0.5286	1.1691	0.1982	1.5264	0.67	1.2327	0.8288	0	
0.0812	1.1758	1.9615	2.1019	1.1263	0.6799	1.1515	1.3544	
0.1338	0.3615	1.6884	1.6928	0.8828	0.3806	2.7379	0.6828	
1.3645	1.3069	0.7393	0.0278	2.3437	1.5611	0.2987	0.0897	
0.082	0.6498	0.1016	1.4635	0.2533	1.7676	0.9555	0.6111	
2.0124	0.4088	0.3408	0.6468	0.1936	0.1512	1.6755	2.8915	
1.891	1.2162	0.0472	2.2717	1.8564	0.4701	0.3723	0.4696	
2.0066	0.8286	0.8247	0.3333	0.9815	2.1296	1.2443	0.6072	
1.2569	0.4646							
ADAMTS4	0.4858	0.0952	0.0369	3.0189	0.1349	5.1737	0.1351	
0.1603	3.0847	1.0608	0.0067	0.8406	0	0.0878	1.3269	
4.2605	0.0413	1.7224	0.0357	0.1544	0.0278	4.7774	3.2573	
1.2718	3.296	2.4285	0.0357	0.0623	4.1031	0.1539	0.0335	
0.0113	0.031	4.2123	1.4824	0.1961	0.0897	0.0696	0.3646	
0.2115	3.4365	1.3182	0.0099	0.0486	0.2877	0.046	0.1348	
0.1713	0.0038	0	0.1145	2.1566	0.4714	0.2013	0.0979	
1.8499	0.082	0.0669	0.1008	0.0779	0.267	1.8235	0.152	
0.1114	0.2043	0.0474	1.2853	3.2686	0.1382	2.7422	0.114	
5.1611	0.079	0.4343	3.557	0.1683	0.2796	4.7682	0.1979	
0.9386	0.144	3.4335	0.1086	0.2284	3.3165	0.287	0.5987	
1.024	1.5254							
ADAMTS5	0.0346	0.0126	0.0312	0.0395	0.0049	0.0206	0.0037	
0.008	0.0366	0	0.0359	0.0072	0.6297	0.2972	0.064	
0.1216	0.7621	0.0169	0	0.02	1.2113	0.3075	0.1209	
0.0061	0.0881	2.4401	0.0113	0	1.4763	0.0104	0	
4.00E-04	0.0577	0.0118	0.1172	0	1.333	0.0154	0.0038	0
	0	0.0487	0.0188	0.1396	0.076	0.8733	0.046	
0.0447	0.265	0.9837	0.0906	0.5561	0.268	0.5792	0.0192	
0.0628	0.1474	0.06	0.7191	0.5858	0.2008	1.1911	0.0088	
0.0396	0.0423	2.314	0.0264	0.3201	0.0623	0.0648	0.066	
0.0381	0.0764	0.0287	0.054	0.1065	0.0037	0.0243	0.0048	
0.0535	0.0134	0.4031	0.0361	0.1804	0.1089	0.1889	0.1025	
0.0757	0.0554							
ADAMTS6	0.1473	0.034	0.1367	0.6783	0.331	0.2991	0.3221	
0.4475	0.1797	0.152	0.2184	0.4994	2.5498	0.1866	0.2788	
0.1083	4.403	0.0413	0.2219	0.6356	0.3425	0.0893	0.1725	
0.2807	0.2889	2.2123	0.8226	0.6026	0.0889	0.0335	0.38	
0.4332	0.0351	0.3023	0.4616	0.3395	0.407	0.3116	0.0938	
0.203	0.1958	0.4748	0.7922	0.7801	3.3842	0.4272	0.1281	
0.4305	1.4897	2.7953	0.0387	0.2094	0.3128	0.1106	0.4789	
0.0832	3.574	0.0569	0	0.112	0.2787	0.188	0.2484	
0.1265	0.2353	2.2398	0.982	0.15	0.1439	0.9322	0.6938	
0.1266	0.5593	0.6026	0.5418	0.3347	0	0.3031	0.1446	
0.4236	0.1366	1.4568	0.0425	0.3881	0.5144	0.2855	0.3743	
0.1101	0.1557							
ADAMTS7	1.1147	2.0294	1.8995	0.4165	1.6899	0.3736	0.1725	

1.3322	0.925	1.3265	0.8221	0.3817	2.73	0	2.3739	
0.6693	0.0725	1.5416	0.5465	0.8914	0.2457	1.3443	1.3655	
0.3652	2.0882	1.2422	1.1362	2.0126	1.7255	2.5081	0.7275	
0.2678	0.2275	0.2969	0.744	0.3215	0.3836	0.5782	1.4366	
0.2543	2.3611	2.6488	0.9901	1.4234	1.404	0.2833	0.8829	
0.6431	2.0436	1.0675	0.9871	1.1097	0.4473	1.7744	0.4912	0
	1.5679	1.5116	0.0934	1.6977	1.0717	0.3728	0.8323	
0.798	1.7921	2.6178	2.5482	2.0505	1.7423	1.2742	1.0142	
0.7123	2.9077	0.9449	2.3559	0.6737	0.3815	1.2832	0.714	
0.0566	0.5313	2.5282	0.1757	1.5613	2.3084	0.6737	0.7218	
1.8891	1.8015							
ADAMTS8	0	0	0	0	0.0083	0	0	0
	0.0229	0	0	0	0	0	1.0308	0
	0	0	0	0.0974	0	0.011	0.0229	
0.0188	0	0	0.0921	0	0.2604	0	0.0396	
0.0061	0	0	0	0	0	0	0.1252	
0.0301	0	0	0	0	0.0186	0	0	0
	0	0	0	0	0	0.0055	0.0422	0
	0	0.8939	0	0.1352	0	7.00E-04	0.0273	0
	0.079	0	0	0	0	0.0287	0	
0.016	0.0147	0.0405	0	0.0141	0	0	0	
0.0199	0.0092	0.3668	0	0.0091	0	0	0.0406	0
	0.0514							
ADAMTS9	0.0404	0.0321	0.4636	0.048	2.0689	1.9383	1.7763	
2.0787	1.318	1.3001	0.9422	1.5383	0.2619	1.3099	4.7478	
1.7423	1.3506	0	0.7946	2.2866	1.1143	4.0404	2.454	
1.8782	5.1809	0.2951	1.8317	0.0402	2.3579	0.189	0	
1.8017	2.7749	1.0027	3.5361	0.5355	2.7351	0.7131	3.7147	
3.2833	0.3021	0.0206	3.6058	2.5063	0.3043	1.1023	0.6917	
0.6764	4.3445	0.6256	1.6256	4.3457	2.0287	0.0188	0	
0.0911	2.167	4.0944	0.4501	4.104	2.6218	0.3158	1.9347	
1.7779	0.8238	0.9582	2.7429	0.125	0.0705	3.3778	0.0232	
1.8073	0.9079	3.5848	2.8897	0.0155	0.0346	1.4163	0.9528	
1.1774	1.8516	1.2052	2.2365	5.1157	2.3724	1.6931	0.6467	
0.7678	0.6911							
ADAMTSL1	0	0	0.0735	0.0372	0.8696	0.0649	0.0592	
0.049	0.0357	0	0	1.8985	0.03	0	0.0558	
0.0573	1.1516	0.0621	0.0966	0	0.1139	0.0769	0.2506	0
	0.0162	4.37	0.0285	0.0517	0.3525	0	0.021	
0.3714	0.0808	0.0432	0.2227	0	0.0494	0.0331	0.0218	
0.1202	0.0705	1.8392	0	0.326	0.0789	0.0219	0	
0.0733	0.0721	0.4276	0.0956	0.0409	0	0	0.0031	0
	4.4268	0.2114	0.0228	0.4515	0	0	0.0059	0
	0.0058	0.8969	0.1782	0.0069	0	0	1.3815	
0.0326	0	1.2924	0.0049	0	0.0055	0.6288	0	
0.0331	0.0297	0.823	0	0.0527	0.0133	0	1.0865	
0.0371	0							
ADAMTSL2	0.5061	0.0913	0.0387	0	0	0	0.0164	
0.1809	0.0349	0.0592	0.0393	0	0.0717	0	0	
0.0293	0	0.0377	0.0689	0.0751	0.1574	0.0585	0.0107	

0.0452	0.0131	0.0611	0	0.0418	0.0536	0.0289	0.039	
0.0636	0.0437	0.0454	0.013	0.0367	0.0207	0	0.1639	0
	0.0326	0.0146	0.0384	0.0877	0.0718	0.0491	0.0731	
0.0455	0	0.1174	0.0236	0.0301	0.0555	0.0968	0.0274	
0.033	0.0207	0.094	0.1591	0.0702	0	0.0847	0.0845	
0.033	0.0056	0	0.0906	0	0.0368	0.1733	0	
0.0491	0.0192	0	0.0741	0.1654	0.0556	0.1207	0	
0.0166	0.0095	0	0.0459	0.1437	0.0164	0.122	0.032	
0.0317	0.061							
ADAMTSL3	0.1025	0.1014	0.1792	0.1095	0.0757	0.0707	0.3344	
0.0039	0.4593	0.1071	0.0588	0.1036	0.0092	0.4332	0.1615	
0.0729	0.3168	0.0398	0.0634	0.4896	0.1061	0.1163	0	
0.1342	0.1404	0.0093	0.1357	0.244	0.0805	0.0898	0.7284	
0.0921	0.2147	0.1316	0.0664	0.0236	0.0177	0.1902	0.0417	
0.7624	0	0.0364	0.0612	0.1281	0.2067	0.076	0.2272	
0.0867	0.0325	0.048	0.0877	0.1471	0.031	0.0803	0.1143	
0.0427	0.8235	0.0745	0.0676	0.0773	0.8809	0.042	0.1743	
0.0436	0.233	0.3795	0.0881	0.0885	0.0829	0.0484	0.0782	
0.0803	0.1915	0.0396	0.0772	0.0675	0.117	0.1695	0.1275	
0.0397	0.3357	0.4739	0.0357	0.079	0.0827	0.0661	0.0915	
0.0931	0.0957							
ADAMTSL4	1.8069	0.0315	0.0522	0.5368	1.5646	0.4053	0.6542	
1.5161	0.5138	0.6092	0.4462	1.9945	0.1594	0.2726	0.7279	
0.3658	1.5767	0.6419	0.1673	0.1119	0.3509	0.9758	1.064	
0.2279	2.1098	1.2985	3.6515	0.1935	1.1461	0.3678	3.3696	
0.1805	0.2798	0.584	0.7075	0.8138	0.7827	1.4142	1.3107	
1.0802	1.3521	3.1066	1.3432	2.5354	1.6335	1.6764	1.6413	
0.2691	0.8421	2.9738	0.8072	1.7697	0.5555	0.264	2.6916	0
	1.7329	0.4995	0.4239	0.7844	1.8608	0.5046	0.8455	
0.6488	1.5532	1.7509	1.8906	0.4268	0.3182	1.3165	1.1075	
0.7421	0.4656	1.5109	1.2928	0.0424	0.3496	2.4828	0.3303	
0.08	0.2295	3.3331	0.3884	1.3339	1.1688	0.5563	1.0499	
0.5579	0.9073							
ADAMTSL5	2.2908	2.2731	1.4286	0.6474	1.0689	1.4814	0.9357	
1.4989	1.8457	2.3337	2.2333	1.539	2.0486	1.4589	0.8878	
1.0605	2.2301	1.8828	1.4909	1.2228	1.7417	1.1146	0.968	
1.8158	0.3407	2.0869	1.5603	1.2957	1.4531	1.5396	1.9696	
1.2429	1.7307	2.0549	1.8491	2.1668	1.5513	2.2589	1.2415	
1.8987	1.9415	2.6693	2.1159	0.8245	1.4424	2.3393	1.2075	
1.1407	0.6278	0.2261	1.3177	0.0614	0.8329	1.3169	2.6943	
1.8898	1.5857	2.1881	1.7953	2.0255	1.3105	1.7797	1.1319	
1.2417	1.6459	1.9957	0.8201	1.8755	1.125	0.9737	1.9474	
1.4123	1.536	1.3874	0.3994	1.0752	1.7448	1.0228	1.6071	
1.9474	2.6771	1.4269	1.8655	0.3771	0.3207	1.2901	1.7675	
1.5354	1.5769							
ADAP1	3.6594	0.3162	2.2489	0.4982	0.5676	0.2387	0.8342	
2.3032	0.8749	0.6703	0.2081	0.4876	1.2828	0.0965	0.26	
0.1624	1.0947	0.8943	0.2223	0.1689	0.094	1.4845	0.5695	
0.1494	2.289	0.88	1.8563	0.512	2.0072	0.2874	2.5601	
0.4888	0.2194	0.274	0.7547	0.277	1.3007	2.6064	2.2264	

1.4988	2.0078	0.2195	2.3392	0.225	1.7253	1.7046	1.5334	
1.3122	0.6059	0.2181	1.922	0.1352	0.8837	0.7376	1.8832	
1.586	0.5244	0.7144	0.7347	0.317	1.7278	0.7164	1.91	0
	1.4508	1.2824	1.712	2.8202	3.0124	0.244	0.0888	
0.5951	1.6118	1.6217	0.1624	0.1821	0.0872	1.558	1.7726	
3.3119	0.2569	0.4348	0.5264	3.3197	0.7134	1.6004	1.588	
1.5327	0.8342							
ADAP2	0.5841	0.1844	0.1038	0.0922	0	0.0441	0.0533	
0.2589	0.0402	0.1422	0.0482	0.3694	0.3085	0.1072	0.1398	
0.0746	0.0523	0.1577	0	0.0202	0	0	0	
0.0681	0.0022	0.0816	0.1221	0.2405	0.047	0.0388	1.7069	
0.0353	0.0266	0.0928	0.0837	0.9466	0.25	0.0708	0.0095	
0.0522	0.0575	0.2061	0.2208	0.0679	1.0562	0.3356	0.0622	
0.0279	0.0139	0.2735	0.0153	0.024	0.0294	0.034	1.8837	
0.105	0.0689	0.1863	0.0795	0.0793	0.0587	0.0514	0.1223	0
	0.2603	0.1024	0.223	0.0783	0.347	0.1143	0.0537	0
	0.1175	0.0766	0.039	0.1314	0.0716	0.1365	0.0699	0
	0.0372	0.0703	0.0511	0.1393	0.0742	0.1274	0.0337	
0.0139	0.0213							
ADAR	5.7073	5.4673	5.6739	6.0532	5.9486	5.5543	7.0937	
5.6095	5.5394	5.6913	6.2247	5.908	5.896	5.2933	6.0759	
6.0282	5.5436	6.3404	5.4741	5.2376	5.2737	5.4129	5.3794	
5.5596	5.9705	7.0439	5.3988	6.4194	5.4567	6.0933	6.8173	
5.765	4.836	5.5258	5.1914	5.9791	7.0734	5.8656	5.6748	
5.8916	5.8079	5.896	6.0665	5.4052	7.1051	6.8014	5.5936	
6.3774	5.8993	5.1997	5.9756	6.0837	5.6875	5.9972	6.4784	
5.5664	5.5775	5.6324	5.8263	6.0209	5.739	5.0782	5.82	
5.5079	6.4033	7.2305	5.4746	6.2351	6.3587	5.5501	5.2194	
5.4828	5.688	5.5713	5.9305	5.1139	6.5838	6.5052	5.8341	
5.2013	5.6045	5.3997	5.339	6.3048	5.1662	5.1508	5.6904	
5.6808	5.3489							
ADARB1	3.6932	2.0602	2.7626	2.737	2.1963	2.1676	2.7388	
2.8456	3.2375	2.2283	2.297	2.9002	1.2371	2.3121	4.3932	
3.1706	2.7863	2.6577	3.055	2.2876	1.7836	2.9885	3.5216	
3.0946	4.3474	2.8437	2.7119	2.9201	2.8388	3.6403	2.4981	
2.7477	2.3721	2.9011	3.9372	2.3074	1.4705	2.4918	4.7812	
1.7514	2.5489	1.4453	3.9739	3.1808	1.2723	2.0835	3.3522	
1.4531	4.8802	2.3747	2.5821	3.7544	2.6139	2.5174	2.4518	
3.3075	2.5763	1.8686	3.1529	2.332	3.0126	1.9763	3.474	
2.557	3.2863	2.2188	2.1088	2.1407	3.9024	4.3811	2.6908	
2.4203	2.0055	3.0576	4.8726	4.4112	2.1523	3.5024	2.4502	
2.2726	2.2929	4.4356	2.5293	4.0055	3.243	2.1564	4.1797	
3.2043	3.1636							
ADARB2	0.0703	0.0336	0.0449	0.0466	0.0185	0.0418	0.0177	
0.0224	0.0596	0.0474	0.1234	0.0087	0	0	0.0104	
0.01	0.0452	0.0515	0.0705	0.0836	0.043	0	0.0082	0
	0	0.0145	0.0262	0.3335	0	0.1782	0.0308	
0.0087	0.0257	0	0.0061	0.0297	0.0083	0	0.0325	
0.0206	0.0168	0.007	0.0386	0.0505	0.0918	0.0212	0.0518	
0.0296	0.017	0.0753	0.0469	0	0	0.0042	0	

0.2457	0.007	0.838	0.0056	1.0454	0	0	0.0174	0
	0.0262	0	0	0	0.0309	0	0	0
	0	0	0.0034	0.0052	6.00E-04	0.0046	0.0012	0
	0	0	0	0.0068	0.0119	0	0.0607	
0.0656	0.2849							
ADAT1	3.1748	2.5326	2.3839	3.4503	3.5771	4.1627	2.3418	
2.8476	3.055	2.907	2.7845	2.8352	2.3487	3.7334	2.5138	
2.7829	3.0241	3.1883	3.1171	4.2092	2.6452	2.8965	3.4081	
3.2844	3.1425	3.0698	1.5843	3.0217	2.8349	2.4789	2.3408	
3.1659	2.3493	2.5755	3.067	2.7496	2.9217	2.7686	2.5813	
3.1296	3.1711	2.9711	2.6123	4.1877	3.0594	3.2526	3.1752	
3.1838	2.4985	2.9595	2.4506	3.9663	2.8143	2.4436	2.7363	
3.1602	3.1706	1.8604	3.0169	2.2675	3.3798	2.9182	2.6477	
3.4896	2.6006	2.1535	2.7835	3.1101	2.5966	3.4353	3.4228	
3.4746	2.7522	3.0031	3.589	3.5051	2.7247	3.8702	3.3853	
2.809	2.4991	3.1263	2.4339	3.0961	2.539	2.536	2.9255	
3.2898	2.9953							
ADAT2	1.3966	1.1224	1.2682	1.471	0.9201	1.0321	0.6443	
1.1273	0.9015	0.8069	0.7639	0.9445	0.6418	1.7531	1.0194	
0.743	1.8588	1.0542	1.5158	1.214	1.4039	0.9613	0.6705	
1.2297	0.4926	0.8822	1.3585	1.0832	1.0486	0.8876	0.8186	
0.9155	0.8927	0.6936	0.7521	1.0092	0.7	0.6877	0.7814	
1.6539	1.0692	0.6222	0.7924	1.0692	0.9275	1.1845	1.4491	
0.8758	1.1359	2.0187	1.1705	1.3982	1.4165	1.2789	1.6456	
1.4067	1.034	1.1591	1.1703	1.2965	0.9987	1.4825	1.3507	
1.2274	1.1236	0.2626	1.3886	0.3843	1.1223	0.8164	0.8712	
1.3869	1.258	0.7988	0.873	0.5786	1.073	0.9115	0.6086	
1.0507	1.1265	1.196	0.826	0.7635	0.7021	1.3436	0.9069	
1.401	1.1803							
ADAT3	0.9181	1.3036	1.3524	0.6934	0.8813	1.1869	0.5278	
1.2946	0	0.7066	0.616	0.6676	1.0108	0	0.8972	
0.6379	0.8233	0	0.8127	1.5589	2.3442	0.9141	0.6562	
1.6146	0.6374	0	0.5859	1.1031	0.9691	0.6399	1.3675	
1.7833	2.0064	0.9227	0.5252	0	1.0185	1.8958	1.2897	0.6
	1.6904	0.6347	0.8756	1.0849	0.605	0.5595	0.8267	
0.5411	1.1938	0	0.5222	0.9603	0	0.6717	2.0474	
1.6976	0.6058	2.1504	1.1412	1.7492	1.5776	1.2124	1.915	
1.1317	1.3088	0.7381	2.2929	1.286	1.5339	1.156	1.3576	
1.2992	1.2679	0.7753	0.3529	0.7578	1.4981	0.257	1.244	
0.8511	1.5851	1.46	0.8894	1.4099	2.1971	1.4912	0.6467	
1.6985	1.6173							
ADCK1	3.128	2.0056	1.5601	1.692	1.4287	1.7064	1.5845	
2.3611	1.9923	2.2866	1.4381	2.2373	1.1183	1.8287	1.8816	
1.505	2.148	2.7334	2.2152	1.6685	2.2441	1.4226	2.3071	
1.854	1.1387	2.0431	0.8703	2.5773	2.0545	1.8682	2.1825	
2.8275	1.903	2.3797	2.0637	1.8816	1.5069	2.7837	1.6466	
1.8967	2.8334	1.9373	1.3723	2.1581	2.0939	1.5237	2.3044	
1.8226	1.4518	2.3957	2.0824	2.4563	1.7548	2.3929	2.6906	
2.6272	2.0537	1.6696	1.633	2.0819	2.1443	2.7831	2.871	
1.7905	2.0914	1.7416	2.2277	2.326	1.3482	1.5811	2.372	

1.6902	2.2769	1.7849	2.0601	1.2902	1.6844	2.2029	2.9649	
2.3379	2.2986	1.2987	2.4029	1.631	1.2055	1.9177	3.0251	
2.6837	2.8553							
ADCK2	4.2558	3.9361	4.3385	3.1886	3.4288	4.8157	2.9956	
4.4359	3.3704	3.947	4.1642	3.3782	3.6246	3.0455	3.0821	
5.2454	2.975	4.3326	3.3643	4.8862	3.6605	3.889	3.7776	
4.1219	3.0381	2.8492	3.3416	3.384	4.0349	4.0827	3.3284	
3.3754	2.6547	4.0902	3.9568	3.5043	4.5002	3.3782	3.475	
3.1654	3.8405	2.6595	3.5848	3.6477	3.7389	2.4557	3.1422	
3.9031	4.1952	1.1833	3.4114	3.471	2.8979	3.4899	3.8935	
3.8635	1.5758	4.3018	3.7725	4.3113	3.6686	4.4015	2.93	
3.8065	2.9072	3.1303	3.5982	3.4248	3.1976	3.4767	3.3569	
3.8756	4.0314	2.8993	3.3302	4.8602	3.2508	3.9396	3.083	
4.4564	3.1891	2.5529	4.03	3.4098	3.3487	3.3977	3.2389	
3.2521	3.1572							
ADCK5	2.0324	1.8676	2.4177	2.1988	1.9297	2.2544	0.9525	
1.9009	1.3008	2.3405	1.5883	0.8729	1.9519	1.0569	2.4538	
1.7865	2.08	1.5086	1.6939	2.4132	2.5005	1.5706	1.7215	
1.8593	2.1979	1.0092	0.8804	2.238	2.0922	0.6682	1.5015	
2.2348	1.9789	1.8408	1.801	1.3495	1.5079	3.3547	1.8676	
0.9359	2.7524	1.6834	1.8598	1.7239	1.1095	1.1822	1.6719	
1.6615	2.4911	1.2929	1.7092	1.4167	1.7166	2.1849	1.3434	
2.3795	2.1161	2.5713	2.4991	2.3957	1.6048	1.2053	2.0412	
1.7564	3.0012	1.1546	2.2588	2.5562	1.3254	1.8114	2.4959	
1.6315	1.4941	1.1869	1.6032	1.5647	1.9748	1.1004	2.7459	
2.6173	2.0283	2.1438	1.5337	1.6975	2.3243	2.2861	1.7636	
2.3446	2.0845							
ADCY1	3.5211	1.5416	3.75	3.2086	0.5829	2.4275	3.2612	
4.2941	4.5072	4.8272	6.2149	4.7851	3.1896	4.1218	1.8352	
4.5173	0.0348	2.7685	4.0584	4.1999	3.8347	2.23	3.118	
4.3777	1.6001	1.2992	4.9553	3.0838	2.896	3.2053	3.1649	
4.6708	2.0569	4.8674	2.2111	3.8355	2.2594	1.1818	5.4058	
3.9107	5.2404	2.0225	3.8887	3.26	1.6858	3.0434	3.9112	
3.7844	0.5019	0.057	3.7557	3.2555	4.9065	5.7721	1.3531	
3.9336	0.0608	0.2103	3.3971	1.2868	4.1127	4.0975	3.2261	
3.0404	1.9014	2.4297	1.5046	1.9014	3.1525	2.3286	3.3894	
4.4847	4.5573	4.0116	4.0863	3.6477	2.7326	5.2366	2.9939	
3.3161	3.7298	2.4797	5.6073	4.9158	1.9231	4.174	0.8659	
1.9811	2.1589							
ADCY10	0.0448	0.0353	0	0.0976	0	0.0339	0.0565	
0.023	0.0332	0.0386	0.0057	0	0.0115	0	0.069	
0.037	0.0205	0.0815	0	0.023	0.0529	0.0261	0	
0.0491	0	0	0.0559	0	0	0.0276	0.1923	
0.0109	0	0.0082	8.00E-04	0	0.0605	0.0177	0.1075	
0.1961	0.0165	0.0122	0.0196	0.059	0.034	0.1145	0	
0.0793	0.0244	0.0851	0.0385	0.1174	0.0461	0	0.0385	0
	0.0444	0.0375	0.1201	0	0	0.0223	0.013	0
	0	0.0399	0.2259	0.0294	0.0214	0.061	0.0282	
0.0106	0.1182	0.0095	0.0145	0.041	0.0283	0.0163	0.0161	0
	0.0119	0.0216	0.0115	0.0194	0.0416	0.0715	0.0302	0

	0							
ADCY2	4.1506	3.1031	1.0051	0	0	0.8674	1.9187	0
	1.9232	3.1551	4.4143	0.1062	0.0543	0.1197	0.0728	
3.4256	0.0587	2.9272	1.9712	0.0579	1.0756	0.0469	0.0464	
0.0805	0.0344	0.0369	0.0556	2.4414	0.0432	5.7798	0	
0.6531	1.66	0.1095	0.0579	2.7429	0	0.0731	1.0692	0
	0.0717	0.0328	0.0576	1.6347	0.0384	0	3.418	
1.9842	0	0	0.0418	0.0567	2.162	0	0	
2.2557	0	3.4828	2.8368	5.156	0	1.0504	0	
1.1994	0	0	2.6978	3.3847	4.0177	0	0.0027	
0.664	0.5036	0.0249	1.0989	2.8693	2.3025	0.2779	1.2047	
0.8851	3.3276	0	0	0	0	1.3657	3.0947	
1.6248	1.7149							
ADCY3	4.5903	3.7614	4.5042	3.5248	4.432	3.5733	2.9818	
4.0965	4.1504	4.5829	4.444	3.6329	4.2438	3.6407	4.3287	
4.2112	4.5539	3.4625	3.8821	4.5131	4.5763	4.6416	4.5647	
4.7748	3.9945	3.0702	3.1328	3.5913	4.649	3.6045	3.1999	
3.8103	4.6939	4.1679	3.2882	3.7933	4.078	2.7751	3.2493	
4.3781	3.8653	2.9498	4.8632	3.7969	4.2166	3.4896	3.6069	
4.3688	4.7708	3.1225	4.1365	3.8123	2.9706	4.0485	3.5298	
4.3619	3.1278	4.3574	3.5936	4.7078	4.6891	4.0256	3.883	
4.3112	4.1211	2.0226	4.6474	4.0939	4.3569	4.6819	3.9791	
4.3797	3.5999	3.0367	4.2476	3.658	3.9992	3.7862	4.1808	
4.2927	4.1355	3.0898	2.7328	3.6036	3.7784	3.4576	4.0226	
4.3417	4.054							
ADCY4	0	0	0	0.0699	0.0026	0	0	
0.0112	0.0111	0.0142	0	0.004	0.0199	0	0	0
	0	0.0564	0	0.0336	0	0.0134	0.0276	0
	0.0751	0.0759	0.0226	0	0.1442	0	0.0573	
2.00E-04	0	0	6.00E-04	0	0.0693	0	0	0
	0.0108	0.2422	0.0521	0.0772	0	0	0	
0.0409	0.0626	0	0.0314	0.0201	0	0	0	0
	0	0	0.0488	0	0.0211	0.056	0	0
	0.0519	0	0.0364	0.0038	0.0228	0	0	0
	0	0.0471	0.0579	0	0.0419	0	0	
0.0696	0.0329	0.257	0.0418	0.075	0.1055	0	0.1469	
0.1899	0.1311							
ADCY5	0.229	0.1324	1.3096	0.0866	0.0228	0.1284	0.2093	
0.0357	0.1579	0.1487	0.0952	0.0372	0.0751	0.0811	0.1511	
0.158	0.0896	0.0477	0.1673	0.0383	0.0883	0.0823	0.077	
0.0798	0.2543	0.0957	0.2136	0.2207	0.0647	0.0747	1.9256	
0.0261	0.0888	0.407	0.169	0.4455	0.0546	0.527	0.8702	0
	0.0292	0.0159	1.4872	0	0.4609	0.0256	0.1496	
0.199	0.0705	0.1563	0.3164	0	0.0459	0.1447	0.7008	
0.561	0.018	1.3208	0	0.4434	0.019	0.0208	0.005	
0.0947	0.0793	0.1728	0.5532	0.1951	0.3302	0	0.9661	
0.2038	0.052	0	1.0421	0.1002	0.0243	0.2091	0	
0.0849	0.404	0.0612	0.0645	0.5987	0.0757	0.0486	0.4293	
0.491	0.6075							
ADCY6	4.032	4.4885	3.9147	2.9168	3.8433	3.4802	2.8721	

2.7271	3.2331	3.2797	4.6543	2.8988	2.6791	2.6234	4.2863	
4.1755	2.4696	4.1958	2.509	3.885	3.4447	3.8522	3.643	
2.5474	3.8004	3.9542	4.4382	3.6066	3.5699	2.749	3.6822	
2.6346	2.9174	3.2522	4.7333	3.0157	3.7254	3.4729	3.6233	
2.7426	3.3494	3.9948	2.1564	3.2001	3.4468	2.9766	3.5916	
2.6663	3.5301	3.295	3.225	3.2135	3.2826	3.1103	3.1801	
2.6349	2.9903	3.3742	4.0484	3.1618	3.1043	3.0255	4.1308	
2.9492	3.6733	4.2749	4.493	3.6777	3.0758	2.859	3.5756	
3.6205	3.814	2.6468	3.6223	3.6782	3.8211	3.1984	2.9719	
3.5101	2.7593	4.0011	3.5963	4.6625	2.5975	2.8862	4.0584	
3.6395	4.3239							
ADCY7	2.4119	1.969	1.9162	2.6605	2.9874	1.984	0.2232	
1.9495	0.9667	1.689	1.6473	1.0643	3.0547	1.005	0.7945	
0.3816	3.6445	1.7562	1.243	0.928	2.1868	2.7001	2.0958	
2.383	2.3665	2.6368	1.662	1.9011	2.9267	1.5558	0.5999	
1.8811	0.2827	0.6367	2.9479	1.2724	2.4502	1.1325	0.7373	
2.2192	1.4844	2.9643	3.7098	1.575	3.7192	3.9796	2.1094	
1.709	0.2181	1.2404	1.9066	2.5454	0.7542	2.0367	1.354	
1.6002	5.0432	1.5156	2.1717	1.6792	2.1562	1.7464	1.2555	
1.2048	3.6316	0.8816	0.5727	1.3209	2.9107	2.8278	1.9578	
0.6206	0.9865	0.9914	0.4918	1.4013	2.2608	1.0162	1.731	
0.7925	1.5775	2.1251	1.1718	2.1953	2.5435	0.8481	1.3029	
1.0753	0.8351							
ADCY8	0	0	0	0.4311	0.1271	0	0.1237	
0.1264	0	0	0.4132	0.2091	0.2254	0	0.1339	0
	0.7731	0	0.1975	0.1641	0	0.1455	0.2173	0
	0.8938	0	0.2254	0	0.1561	0.1594	0.1235	
0.1173	0	0	0.1136	0.1705	0.1171	0	0.1394	
0.2453	0	0.1136	0.1582	0	0.1235	0.1881	0	0
	0.1777	0	0.1322	0.2528	0	3.6295	0	
3.3225	0.0018	0.3329	0	0.8217	0	0	0	0
	0	0.9257	0	0.004	0	0.1135	0	0
	0.6376	0.0232	0	0	0	0	0	0
	0	0	0	0	0	0	0	0
	0							
ADCY9	3.4002	2.3356	2.997	1.965	2.194	3.1255	2.2547	
3.0548	3.3744	2.6096	2.8489	2.1246	2.883	2.1322	2.2027	
3.3638	1.709	3.2424	2.4272	3.1665	2.251	2.7528	2.4783	
3.4099	2.8636	3.1693	3.8078	3.6799	3.5784	3.0326	3.3654	
2.1405	2.6063	3.596	4.3274	2.117	1.0833	3.4728	2.5213	
2.6555	3.2389	1.8052	3.1067	2.835	2.8401	2.5896	2.9194	
2.7073	4.7404	2.0406	2.8794	3.1006	1.8987	2.9514	2.2904	
3.2385	1.634	2.5112	2.7322	2.5764	2.9614	2.2372	3.1662	
2.6893	3.7196	2.9005	4.3869	3.9468	3.509	2.6708	3.6142	
2.6296	2.5211	2.1597	4.5631	4.3246	2.6458	4.2453	2.8513	
2.5638	2.3072	3.3919	3.3295	4.1715	2.1616	1.8831	4.4761	
4.1534	4.194							
ADCYAP1	0	0.0204	0	0.3531	0.0129	0.5673	0.0683	0
	0	0	0	0	0.5565	0	0.0047	
0.4744	0.2141	0	0	0	0	0.012	0.1535	0

	0	0	0	0	0	0.7717	0	0
	0	0	0.0873	0	0	0	0	
0.0532	0	0.0169	0	0.4869	0	0.0312	0.0247	
0.0717	0	0.055	0	0	0.0663	0.7367	0	
0.1884	0.0259	0	0	0.0213	0	0	0	0
	0.0265	0.0118	0	0.0135	0.4455	0	0.0139	0
	0.7651	0.0155	0	0.022	0.0078	0.0531	0.0375	0
	0.0148	0	0.0614	0.0208	0	0	0	0
	0							
ADCYAP1R1		0	0	0.0264	0.028	0.1224	0.0456	
0.0041	0	0	0.0089	0.0021	0.0188	0.0076	0.0309	
0.0189	0	0.0394	0	0	0	0	0.1953	
0.0155	0.0154	0.0581	0.1805	0.014	0.0413	0	0	
0.7989	0.0683	0.0136	0.0158	0.0819	0.0829	0.0018	0.0201	
0.0176	0.0335	0	0.0032	0.9658	0	0.0141	0.0457	
0.0658	0	0.0077	0	0.0089	0.014	0	0	
0.0551	0.0283	0.863	0	0.0153	0.0101	0.0677	0.0194	
0.0092	0.0137	0.0356	0	0.0256	0.0038	0.0168	0	
0.0148	0.016	0.8535	0.0048	0.007	0.0134	0.0024	0.0261	0
	0.018	0	0	0	0.0385	0	0.0371	
0.0203	0	0						
ADD1	5.6376	4.9332	5.4401	5.2353	5.1133	5.5531	4.5004	
5.5724	5.2898	5.6109	5.0709	5.1298	4.9351	5.5149	4.9526	
5.0722	5.9703	4.9018	5.2607	5.2713	5.7258	5.2231	5.0246	
4.7674	5.6089	6.2796	4.803	6.128	4.6309	5.2692	5.1386	
5.1978	5.0031	5.3154	5.7674	5.3384	5.4722	5.5691	5.4819	
5.1164	4.546	5.8132	5.5991	5.4836	5.2946	5.6814	4.8442	
5.1431	4.9398	5.4869	5.8272	4.7471	5.4526	5.3155	5.1333	
5.4804	5.4294	5.4352	5.1635	5.4424	5.0081	5.5109	5.4697	
4.8008	5.3591	5.6102	5.0263	6.4318	5.4745	5.4634	5.5166	
5.1825	5.7252	5.1284	6.0378	6.9482	5.3599	5.6111	4.8108	
5.4532	5.2688	5.6084	5.2479	5.7978	5.3983	5.2419	6.4453	
6.4259	6.5602							
ADD2	0.1047	3.6094	3.6116	0.2743	2.9063	0.0765	0.9294	
0.7232	0.9097	0.0613	2.536	1.8127	2.6348	0	0.2599	
0.5534	0.0814	0	0.1356	2.0452	0.2924	1.0616	0.5111	
1.555	1.6522	0.0668	4.208	1.5439	0.2184	0.9152	3.013	
0.0582	0.0812	0	2.0959	1.7831	2.6291	2.5508	1.3082	
1.3972	0.0706	0.104	3.922	0	0.2324	0.1075	0.7318	
0.7323	0.0594	0.1004	0.4213	0.0797	0.1344	0.717	2.6507	
2.0962	3.5457	3.7115	0	3.0482	0.9413	0.1874	1.8698	0
	2.693	0.124	2.0063	3.0778	2.4852	0.1194	0	0
	2.0318	0.1209	0.0168	0	0	1.6115	1.8475	
0.5994	1.0516	0	0.1002	1.022	0.2707	0	0	
0.6019	1.1344							
ADD3	4.7424	5.6285	5.0534	4.8896	5.0304	5.1073	4.7997	
4.0252	5.5202	5.2118	4.0615	5.3538	3.3929	5.3939	4.8196	
4.073	3.0557	5.5008	4.3412	3.8571	4.452	4.5719	4.9099	
1.1222	3.9161	3.8459	4.7646	6.3471	5.7028	5.2679	4.6449	
3.9557	4.8801	4.4016	4.6415	5.2739	3.1768	4.6677	4.7034	

3.7953	5.3455	4.163	4.9069	4.5772	3.6662	5.7218	5.5386	
4.4245	5.4011	4.6221	1.986	5.6111	5.088	5.1911	4.779	
5.1364	2.9282	4.2676	4.6886	3.9473	4.2858	4.6908	3.2392	
5.026	3.7251	4.419	6.1309	3.4505	5.4451	3.4956	4.7732	
4.9103	3.6918	5.1544	4.5364	4.8366	4.6928	4.9583	4.8361	
5.1014	5.9611	5.5356	2.6898	5.5356	3.5173	5.1684	5.6066	
5.463	5.7719							
ADGB	0.035	0	0.0302	0	0	0	0	0
	0	0	0	0	0	0.0323	0	0
	0	0	0.0477	0	0	0	0.0027	0
	0	0.0054	0	0	0	0.0343	0.004	
0.0398	0	0	0	0.0061	0	0.0352	0	0
	0	0	0	0	0	0	0.0231	
0.0175	0	0.0513	0	0	0	0	0	0
	0.0108	0	0	0	0	0.0096	0.0057	
0.012	0	0.0018	0	0.0029	0.0041	0	0.0046	
0.0057	0.0084	0.0014	0	0	0.0052	0	0	0
	0	0	0	0	0.1372	0	0	0
	0.0421							
ADH1A	0	0	0	0	0	0	0	0
	0	0	0	0	0	0	0	0
	0	0	0	0	0	0	0	0
	0	0	0	0	0	0	0.0991	0
	0	0.1456	0	0	0	0	0	0
	0	0	0	0	0	0.0357	0	0
	0	0	0	0	0	0	0	0
	0	0	0	0	0	0	0	0
	0.1421	0	0	0	0	0	0	0
	0	0	0	0	0	0	0	0
	0	0.0324	0	0	0	0.0808	0	0
	0							
ADH1B	0	0.2135	0	0	0	0	0.0172	0
	0	0	0.0273	0	0	0.0469	0.1128	0
	0	0.0958	0	0.0486	0	0	0.1406	0
	0.0821	0	0.1407	0	0.0439	0.0471	0	
0.0089	0.062	0.0755	0.0177	0	0.0321	0	0.0367	
0.0565	0.0489	0.0504	0	0	0	0.227	0.0392	0
	0	0.1219	0.0261	0.0294	0	0.0019	0.0859	
0.0385	0.2012	0.0726	0.0584	0.0333	0.0251	0.0432	0	
0.0484	0.1235	0.0526	0.4634	0.0165	0.0048	0.0304	0.0235	
0.092	0.0317	0.0073	0.1088	0.0162	0.0392	0.0104	0.016	
0.0857	0.0605	0	0.0061	0.0299	0.0338	0.0197	0.3707	0
	0							
ADH1C	0	0	0	0.1418	0	0	0.0212	
0.2866	0	0	0	0.0122	0	0.0627	0.0179	0
	0	0	0.2313	0	0	0	0.0106	
0.0547	0	0.0789	0	0	0.0599	0	0	
0.0048	0	0	0.2072	0	0.0152	0.0455	0.2679	
0.0305	0	0.0074	0.0513	0.0393	0	0.1658	0	
0.2206	0	0.19	0	0	0.1256	0	0	0

	0	0	0	0.0412	0.0548	0	0	0
	0	0.0116	0	0	0	0.2587	0.0633	
0.2681	0	0.023	0.0322	0	0	0	0	
0.1297	0	0.3026	0	0	0	0.1876	0	0
	0							
ADH4	0	0	0	0	0.0159	0.0297	0	0
	0	0.0157	0.0057	0	0.0107	0.0315	0	
0.0095	0.4944	0.0268	0.0446	0.033	0.0376	0	0.0057	
0.0192	0.0073	0	0	0	0	0	0.0133	0
	0	0.0164	0.0105	0	0.0064	0	0	
0.0138	0	0.0027	0	0	0	0	0.0442	0
	0	0	0	0	0	0	0	
0.1146	0.0705	0	0	0.1104	0	0	0	0
	0	0	0	0	0	0	0	0
	0.1017	0	0	0.0591	0.0157	0	0.0208	0
	0	0	0	0.0492	0	0	0	0
	0							
ADH5	5.3773	5.4719	5.6213	5.8255	5.4871	5.559	6.3532	
5.2249	4.4876	5.3222	5.4515	6.109	5.4901	5.7883	5.0491	
4.7143	5.0083	5.1232	4.8744	5.1912	5.7606	5.5383	5.0986	
5.0803	5.677	5.7289	5.2496	6.003	4.757	4.9339	5.5289	
5.5925	5.7414	5.3499	5.5058	5.5723	5.4417	6.0253	5.8669	
5.2377	5.8183	6.0767	5.6808	5.796	5.4344	4.9414	5.28	
5.3922	5.155	6.4907	5.8807	5.8403	5.5801	5.3733	5.526	
5.2881	6.1677	5.3943	4.8892	5.5747	5.4595	5.2156	5.8383	
6.0542	5.9071	5.5886	5.5371	5.8569	5.0127	5.2358	5.6694	
5.4403	5.7323	5.4205	5.9243	4.9332	5.4633	5.6819	4.8869	
5.4811	5.7713	6.0302	3.893	5.4649	5.3473	5.6659	6.1117	
5.911	5.9936							
ADH6	0	0	0.0574	0.0447	0.0081	0	0.0165	
0.0479	0.0215	0.0583	0.0326	0.0246	0.0187	0.0901	0.0889	0
	0	0	0	0	0.1127	0	0.0073	
0.2857	0.0123	0.0903	0.0512	0.1125	0	0.0279	0.0459	
0.1457	0.1738	0	0.0924	0.0386	0	0	0.0188	
0.0242	0.0231	0.0049	0.2173	0.0685	0	0.1068	0	
0.0388	0.0569	0.0846	0.0263	0.2625	0.1845	0	0.0554	0
	0	0	0	0.1004	0	0	0.1542	0
	0.2752	0.0417	0.1371	0.0164	0.0717	0	0	0
	0	0	0.0363	0.1044	0.0275	0.1133	0.0257	0
	0	0.1639	0	0	0	0.0821	0.044	
0.048	0.0548							
ADH7	0	0.0337	0	0	0	0	0	0
	0	0	0	0.0559	0	0	0	0
	0.061	0	0	0	0	0	0	0
	0	0	0	0	0	0.0283	0	0
	0	0	0.0035	0	0	0	0	0
	0	0	0	0	0	0	0	0
	0	0	0	0	0	0	0	0
	0	0	0	0	0	0	0	0
	0	0	0.0663	0	0	0	0	0

	0	0	0	0	0	0	0	0
	0	0	0	0.0729	0	0.0596	0	0
	0							
ADHFE1	1.2179	1.1819	0.3555	0.8941	0.2095	0.7484	0.5386	
0.4917	0.8681	0.3744	1.3183	0.6964	0.1859	0.8328	0.6363	
2.692	0.4246	1.6507	0.6182	0.4551	0.622	0.6542	0.3065	
0.4928	0.3182	0.2162	0.918	1.3145	0.4383	0.9538	1.8035	
0.2007	2.1423	3.2635	0.8546	1.8466	0.7028	1.8776	1.1596	
0.3208	1.1193	0.875	0.4385	0.9725	0.4487	0.4374	0.5647	
0.5301	0.6558	1.1696	1.0248	0.3858	1.1335	0.3989	1.4052	
0.4967	1.6011	0.0903	0.4884	0.0915	0.5492	0.4053	1.8782	
0.5502	1.3054	1.5867	1.4059	0.4738	0.5949	0.2085	0.9518	
0.512	0.5767	0.4109	0.9319	0.7191	0.7505	1.2277	1.358	
1.1838	1.7742	0.906	0.2582	0.2306	0.1367	1.0183	1.1878	
0.9227	1.2481							
ADI1	5.1486	6.0649	5.4415	5.8683	5.6888	5.545	4.8658	
5.245	5.1279	5.7067	5.525	4.6995	4.1788	5.2899	6.507	
6.2814	4.7746	5.7819	5.8535	5.8625	6.0112	5.3966	6.2548	
6.0112	5.0151	5.8488	5.1285	5.4508	6.1416	5.2717	5.2732	
5.7431	6.4627	4.9775	5.3064	5.6342	4.6272	6.3281	5.6428	
5.9928	4.5754	5.8135	4.3558	4.4305	6.1319	5.3337	5.955	
5.3142	5.4702	5.5568	4.7625	6.0166	6.1234	4.5835	5.8313	
6.0077	4.9531	6.8863	5.2467	6.1324	6.0443	5.3562	5.6784	
6.4722	6.8554	5.2431	4.9436	6.0762	5.7207	4.9722	5.8431	
5.0857	6.5647	3.8117	6.1534	4.9181	5.4735	6.3037	5.755	
5.2355	5.5685	5.7286	5.7013	5.9827	5.2371	5.9498	6.8792	
6.5132	6.8731							
ADIG	0	0	0	0	0.1839	0	0	0
	0	0	0	0	0	0	0	0
	0	0	0	0	0	0.2032	0.0346	0
	0.0462	0	0.0916	0	0	0	0	
0.1012	0	0	0	0	0	0	0.1221	0
	0	0	0	0	0	0	0	0
	0	0	0	0.0828	0	0	0	0
	0	0	0.092	0	0	0	0	0
	0	0	0.2404	0	0	0	0	0
	0	0.0507	0	0	0	0	0	0
	0	0	0	0	0.0942	0	0	0
	0							
ADIPOQ	0.0743	0.0422	0.092	0.0392	0.0452	0.0222	0.0187	
0.0241	0.0341	0.0289	0.0378	0.01	0.0015	0.0421	0.0772	
1.00E-04	0.0265	0.1003	0.042	0.13	0.026	0	0.0507	
0.0552	0.0238	0.0213	0.1022	0.0296	0.0179	0.0307	0.0246	0
	0.026	0.0176	0.0099	0.0114	0	0.0187	0	
0.0949	0.0165	0.0042	0.0341	0.0233	0.0075	0.0312	0.0754	0
	0.0312	0.0462	0.0068	0.0466	0.0502	0.0084	0.074	
0.0362	0.06	0.0405	0.0157	0.0256	0	0.0089	0.0318	
0.0344	0.0544	0	0.1244	0.0388	0.0252	0.0324	0.0478	
0.0114	0.0243	0.006	0.0288	0.0274	0.0356	0.0433	0.0457	
0.1846	0	0	0.0354	0.0244	0.0304	0	0.0492	0

	0						
ADIPOR1	6.5582	6.8791	7.0439	7.106	6.9269	7.7634	6.8439
6.2235	6.6831	6.826	6.9764	6.7948	5.5575	6.5788	6.0809
7.0206	5.6878	7.0839	6.6117	6.2645	6.698	6.6877	7.0897
7.0388	6.3634	6.4907	7.0668	6.4238	6.3343	7.4343	7.015
6.4677	6.0094	6.9666	6.726	6.8598	6.9387	6.4829	6.9456
6.7151	7.0288	6.1336	6.573	6.2542	6.6424	6.178	6.5228
6.788	5.6643	5.697	6.6747	6.9842	6.9989	6.0038	6.8748
6.7997	5.9844	6.4659	6.869	6.4646	6.5944	6.5896	6.4646
5.9377	6.233	6.4615	7.0843	6.4659	7.1773	6.3365	6.0661
6.4867	6.9516	6.5461	6.9492	6.5988	6.8056	7.5234	6.7738
6.3449	6.4378	6.1116	7.9685	6.9871	6.1798	6.4753	7.0288
6.6702	6.6443						
ADIPOR2	5.3171	5.7134	5.4982	5.4386	5.0768	5.6385	5.0094
5.5654	5.8002	5.5722	5.0321	5.2085	4.9808	5.4781	5.1351
5.5722	5.6936	6.2834	5.6881	5.5722	5.0479	5.8833	5.1063
5.0321	4.9394	4.8564	4.8822	5.5277	4.766	5.2055	6.1652
5.4468	5.4047	5.0281	6.1753	5.6479	4.9769	5.6073	5.9757
5.576	5.8174	5.9327	5.1093	6.2318	6.9068	5.4856	6.4699
6.2664	4.245	6.0496	6.6562	5.6509	5.9973	4.7189	5.1352
5.2008	5.9105	4.7625	6.0056	4.9685	5.3883	6.8001	5.8363
5.8434	5.8097	4.8759	5.1821	5.2097	5.7908	4.7604	5.7008
5.1702	5.5208	5.6929	5.1976	7.1099	5.9989	5.3322	5.6667
5.8957	5.9737	5.7323	5.522	5.7774	4.2971	5.5891	6.23
5.5163	6.0562						
ADIRF	1.2009	0.0282	0	0.0843	1.2681	1.626	0.5972
0.8417	0	0.6172	0.3904	0.9452	2.1857	0.9676	3.8657
0.4608	1.548	0.0751	0	2.1468	1.2114	0.7671	0.6473
0.6152	0.8512	0.9732	0.2759	0.2224	0.3638	0.091	0.0123
0.5865	0.7561	0.802	0.9846	0	1.4868	0.2302	1.1248
0.7021	0.8166	1.0158	1.1198	0.7092	1.5568	1.0393	0.4873
1.2929	0.9094	1.3375	0.6117	0.4507	0.5681	0.2993	0.3667
0.3202	4.763	0.2203	0.9438	0.7661	1.1475	0.3681	0.4058
1.0739	0.872	1.2741	0.4704	0.7969	0.5384	1.1274	0.2622
0.8452	0.5093	0.5869	0.2543	1.7005	0.7705	0.8673	0.6859
0.7863	0.2902	1.1651	1.07	0.7507	0.7154	0.77	1.1234
1.0201	0.8417						
ADK	4.9549	3.9401	4.306	4.4304	4.53	3.597	3.5994
4.3004	3.859	4.0367	3.956	4.8116	5.0395	4.5007	4.2839
3.1831	5.7162	3.9617	3.4496	4.426	4.5075	2.9684	4.1564
4.0689	3.6328	4.0717	4.4479	3.4549	3.7524	3.5897	3.4363
3.8961	3.9084	4.2834	3.6643	4.4636	3.9806	2.9503	3.1251
3.9519	4.1007	5.3976	4.5518	4.1308	4.8578	5.5298	4.1672
3.4791	3.5674	6.2309	4.2979	4.2944	3.9111	3.3608	5.5232
4.5732	5.5612	4.0215	4.0309	3.9194	4.4451	3.9976	4.1349
4.519	3.9692	4.4401	4.1747	3.9583	3.2347	4.3162	4.1761
3.9421	3.3543	4.1325	3.911	2.7724	4.2392	3.8873	3.3487
3.9392	3.3531	5.2077	3.4202	3.3689	4.6614	4.1186	4.4613
4.0689	4.0567						
ADM	3.071	4.5432	4.0411	1.6195	3.8546	3.261	0

4.6134	5.6322	1.9393	5.6024	0	3.6241	2.4681	0.2314	
0.3237	5.1304	1.7312	5.2553	5.1264	1.9638	0	5.3253	
3.731	2.6951	7.3099	5.0694	0.1793	2.303	4.5273	1.8933	0
	2.0804	4.7504	1.7619	3.7425	3.7616	0	0.3982	
2.9321	2.9925	4.0671	0.2127	3.6987	4.3001	0.2811	4.1209	
4.9004	6.5068	6.8864	3.6596	2.5828	0	0.4922	4.0908	
2.4509	6.4307	0.7931	5.8013	1.2532	4.2889	0.7182	4.6416	
4.3087	1.1722	7.8525	0.663	0.5008	0.3931	2.2352	5.4271	
0.6623	0.8635	5.3735	3.6995	5.6159	1.0545	1.4696	1.1836	
1.6383	3.8357	5.1155	5.3741	0.5641	2.3123	0.9163	2.6887	
1.8989	2.1114							
ADM2	0.3456	0.0773	0.9451	0.6962	0.4929	0.2244	0.5948	
1.7997	0.3747	2.5042	1.0642	0.2933	3.2199	0.2455	0.8939	
0.3648	0.0522	0.4107	0.2415	0.1048	0.122	1.7171	0.1322	
0.974	0.8873	0.9322	0.0405	1.2483	0.1593	0.1107	1.487	
0.3152	1.0213	1.3468	0.8849	0.3164	3.4648	1.3329	5.1494	
0.1118	1.5117	0.2037	0.1265	0.4691	0.2797	0.5502	0.7224	
1.5513	0.6962	1.0791	0.7997	0.1031	0.6901	3.7929	1.634	
0.0116	3.7231	2.0245	1.8782	1.807	1.054	1.7927	0.7817	
0.4677	0.0062	0.4834	0.758	1.2432	0.8918	0.1715	0.1837	
0.7596	0.2278	0.2689	0.2691	1.1764	0.3194	0.0995	0.1082	
0.0169	0.0461	0.3444	0.1753	0.7854	0.3869	1.3084	0.6708	
0.4174	0.1234							
ADM5	1.0815	1.9035	0.9817	0.7445	1.5675	1.1342	0.4717	
1.3012	0.6113	0.5031	1.4266	0.4933	0.3184	0.2685	0.7303	
0.4803	1.7235	0.1216	0.4854	1.9316	0.7411	0.6222	0.2628	
1.3377	0.5241	0.6453	0.757	1.1636	0.6478	0.6786	0.6282	
2.0371	0.6453	0.5921	0.2563	0.2229	0.128	0.402	0.9544	
1.0483	1.3108	0.6282	0.9169	0.839	0.6809	0.5736	0.4219	
0.5906	0.5327	0.2986	0.9582	0.3651	0.8349	0.7734	1.3582	
1.0529	0.3916	1.1597	0.9259	1.0644	2.1341	1.1873	1.3281	
0.8614	0.7716	0.0553	0.4084	0.0879	1.0977	0.723	0.273	
0.8019	0.7966	0.2076	0.6408	0.6108	0.9984	0.4466	0.1909	
0.8955	1.2925	1.0682	0.2209	0.2488	1.4529	0.382	0.3458	
0.5812	0.4278							
ADNP	4.7654	4.9063	5.0769	4.6464	4.9892	5.6151	5.1666	
5.0405	4.8883	5.1759	4.894	4.5693	5.0265	5.2921	4.678	
5.0898	5.0586	5.1671	4.991	4.0092	4.7204	5.2403	4.6886	
4.5454	5.0212	4.788	4.4039	4.582	4.5473	4.5741	4.9256	
4.7107	4.925	4.541	4.3029	5.0555	4.356	4.6469	5.1517	
5.1505	4.1747	4.4635	4.5268	5.3852	5.6858	4.5278	5.2192	
5.3493	5.0386	5.1354	4.6641	4.9407	5.4292	5.087	5	
4.6225	4.0509	4.7403	4.8788	4.7926	4.9767	4.4354	4.6684	
5.405	4.6469	4.3654	4.8593	4.6658	5.2361	4.5107	5.1545	
5.2706	5.249	4.8505	5.7679	4.8956	4.5761	5.0968	4.7408	
4.6554	5.2186	5.0035	4.208	4.9732	4.6014	4.9699	4.3941	
4.4027	4.6376							
ADNP2	2.9148	3.1895	3.518	3.6976	3.1798	3.2669	3.2316	
2.914	3.7609	3.2136	3.738	3.4351	2.7784	3.1103	2.7748	
2.5513	3.6575	2.9543	3.5474	2.6937	3.3158	2.8072	2.7683	

3.1725	3.401	2.9968	3.4084	3.6075	2.5117	3.2915	3.4294	
3.6452	2.9936	3.2397	2.0445	3.093	3.0452	3.4137	4.0496	
3.0716	3.2352	3.0022	3.5328	3.8011	3.5421	3.4924	3.5606	
3.1426	2.9557	0.2399	2.9443	3.0153	3.6075	2.8409	3.9697	
3.1683	2.6014	3.9189	3.0503	3.6448	2.9006	2.5938	3.5579	
2.3773	3.9891	2.9374	4.2768	2.848	2.6104	3.0391	2.8564	
3.1172	3.3016	3.6456	2.7352	2.4166	2.8911	2.4149	3.1452	
2.8202	3.7395	3.0764	2.4544	2.7958	2.9894	3.9502	2.9639	
3.0473	2.9278							
ADO	4.3199	4.7267	4.9034	4.5623	4.0221	3.2463	4.4256	
3.3033	4.4398	4.1797	4.3536	3.9591	3.9031	4.2101	3.4611	
3.8367	3.2985	4.4579	4.3839	4.0046	4.4555	3.5409	3.7978	
4.3802	3.9759	3.2963	4.397	4.8572	3.7859	3.9816	2.8229	
4.904	3.5844	3.592	3.6026	4.2035	4.087	3.4657	3.8209	
4.0843	4.646	4.0376	4.2547	3.9736	3.5169	3.7506	4.6223	
3.5117	3.6501	3.9877	3.9288	3.9977	4.4279	4.6136	3.6037	
4.8453	3.6167	3.6025	3.0854	3.595	3.8923	3.5577	4.7427	
3.5814	3.7679	4.4356	4.1699	4.3596	4.3142	4.3266	4.2488	
3.5364	3.5037	4.0629	3.79	3.6743	4.5374	4.1297	3.791	
4.2882	4.3212	4.1742	3.1866	3.8909	3.9204	3.9501	4.014	
4.3291	4.2802							
ADORA1	0	0.104	1.7455	1.776	2.0848	2.7224	1.0923	
0.6077	0.8142	0.3055	0.1004	0.358	1.6149	0.3325	0.0368	
2.2912	0.1982	0.0435	0	0.6496	0.2917	1.0247	1.2843	
0.6464	2.1381	0.0759	0.0698	1.2429	1.6329	0.3889	1.2473	
0.0361	0.6879	0.0448	0.399	0.3112	0.2995	1.0098	0.2165	
0.3132	0.1475	1.7946	1.5854	1.4588	1.3282	0.1503	0.2097	
0.5304	0.1681	0.3928	0.2165	1.8552	0	0.1285	1.0727	
0.9661	1.1942	0.0561	0.1005	0.0469	0.6375	0.1303	0.7505	
1.6241	0.5583	0.951	0.0553	2.5288	0.9221	0.4469	1.1754	
0.1711	0.0665	0.044	2.3625	0.4212	0.6112	0.9246	0.1845	
0.2236	0.2597	0.6921	1.1048	0.2305	3.279	0	0.0655	
0.1012	0.2274							
ADORA2A	0.5852	0.4197	0.7564	0.0499	0.2133	0.0477	0.5958	
2.4982	0.0317	0.0336	0.3247	2.1663	1.5963	0	0.4715	
0.8325	0.0485	0.0437	0.0651	0.6521	0.6476	0.5344	0.3874	
0.4777	0.3643	0.9554	0.6186	0.1739	0.0361	0.3064	1.2363	
0.4179	0.2157	0.5852	0.2427	0.1746	0.0275	1.092	0.1198	
0.0343	0.7949	0.3384	0.4702	0.0373	0.2247	0.3848	0.0452	
0.0406	0.6379	0.9009	0.3629	0.0377	0.0578	0.3816	1.5183	
0.082	0.4412	0.0508	0.124	0	0.0513	0.2378	0.3772	0
	0.5347	2.1387	0	0.6116	0.8444	0.1368	0.501	
0.5758	0.1681	0.5578	0.4992	0.1471	0.038	0.4501	0.3573	
0.0158	0	1.1285	2.6337	0.3342	0.5235	0.3	0.1834	
0.1808	0.0548							
ADORA2B	2.6534	1.3343	1.8368	2.8023	3.9107	1.3227	0.1636	
1.2722	2.2296	3.809	1.6662	2.2995	5.2698	2.5205	3.1589	
1.8336	1.4876	4.0615	3.203	2.8631	2.9331	2.5309	1.4725	
2.4295	4.145	3.7656	2.0272	0.0921	4.1676	0.6358	3.9384	
1.5191	3.4507	1.1179	2.0288	4.077	2.1571	0.6765	2.6946	

5.2729	1.5892	4.2813	4.2539	2.7314	4.4718	4.2336	2.6978	
0.2207	3.9541	2.7331	2.5508	3.3568	2.7153	0.8061	4.0014	
1.3273	2.8867	1.8393	3.2715	1.7364	1.1679	4.4257	3.6276	
2.5194	1.8515	4.6252	1.112	1.7726	0.5148	3.7973	3.1321	
2.1168	1.6635	2.9443	0.7604	2.4635	2.2594	0.4984	1.7073	
0.3202	2.0689	4.5571	2.2567	2.9857	4.8143	2.7605	2.2076	
1.8684	1.5513							
ADORA3	0.1978	1.0118	0	0.1071	0.0077	0	0.157	0
	0.0936	0.4014	0.1672	0.026	0	0.2017	0.1491	
0.6301	0	0.7363	1.002	0	0.461	0.1631	0.1282	
0.0136	1.7389	0.021	0.041	0	0.0916	0.1325	0	
0.0795	0.0652	0.103	1.4567	0.499	0.0432	0	0.0212	0
	0.5032	0	0.05	0.0181	0.0488	0	0.1556	
0.4407	0.3127	0.2109	0.2364	0.1391	0.5086	0.2165	0	0
	0	0	0.6164	0	0	0.1898	0.1884	0
	0.0463	0.0174	0.0905	0	0.2112	0	0.1041	
0.5176	0.279	0.596	0	0.1108	0.3785	0.0561	0.0575	
0.0973	0.2535	0.026	0	0.7428	0.3071	0.43	0.2531	
0.0539	0.1164							
ADPGK	4.5372	5.0751	5.0826	4.7516	4.8067	4.7417	5.8917	
5.2802	4.9324	5.0388	4.4825	4.3385	4.2819	4.4332	4.5481	
5.2732	4.5806	5.4834	4.9624	4.4253	4.8165	5.6565	5.3811	
5.1405	4.963	4.7303	4.1469	4.3684	5.2625	3.7782	4.473	
4.512	4.9209	4.7593	5.4493	5.026	4.5409	4.5066	4.4927	
5.0452	4.2271	4.5333	4.9053	4.9934	5.6322	5.1011	5.4336	
4.7725	4.5301	4.4459	4.9098	4.9707	4.4871	4.9469	4.4407	
4.6799	4.3377	4.4296	4.9287	4.742	5.0569	4.9984	4.8605	
5.6662	4.1335	5.0075	4.4448	4.1781	5.2006	4.5819	4.2516	
4.6577	4.6571	4.9177	5.0666	4.2713	4.8052	5.0231	4.2913	
5.3691	4.0771	3.9171	4.8691	4.9098	4.6924	4.2826	4.279	
4.3811	4.2781							
ADPRH	1.0253	0.0025	0	2.0789	1.8119	1.4122	2.2287	
1.4017	2.0333	1.9188	0.4237	2.6872	1.7524	2.8726	1.283	
0.0173	0	2.4045	2.5872	0.7403	0.1829	2.0003	2.5648	
1.8586	1.7782	1.3762	1.7016	0.0201	3.39	0.4766	0.1314	
0.5816	3.3946	2.7513	2.2097	1.8417	3.0062	0.8178	1.3835	
2.8706	1.5354	1.6804	2.7743	1.7473	0.5153	0.0647	2.6339	
2.3196	1.3078	0.0375	2.2146	2.4211	2.7651	1.4501	0.0908	
1.9154	0.384	0.108	1.8439	0.2033	1.5081	2.9857	0.768	
0.186	0.3198	2.2432	0.0785	0.3089	2.2389	1.852	2.4801	
2.1728	2.0572	2.5957	0.3337	2.5644	2.5747	0.7708	2.9045	
2.0072	1.3912	1.8663	3.1798	1.6557	0.9944	2.3351	2.3698	
2.293	1.4333							
ADPRHL1	2.7533	1.0209	1.1148	0.7628	1.1871	0.221	0.5576	
0.5487	1.359	2.0949	0.137	0.1011	0.3735	0.336	0.6478	
0.0431	0.1172	1.4416	0.4149	0.5646	1.2817	0.1579	0.2649	
0.6165	1.0324	0.3099	0.0043	0.6421	1.3232	0.3147	1.4774	
0.0927	0.2495	1.9518	0.1262	0.9466	0.2649	0.9351	2.2284	
0.3932	3.4043	2.4709	0.3246	4.00E-04	0.4254	0.4461	0.2387	
1.0689	0.3301	0.1816	0.2336	0.1868	0	0.1422	4.9253	

1.1318	1.7189	0.6944	0.4484	0.7037	0.1072	0.3754	1.0862	
0.3239	2.2074	0.3342	1.3952	0.8083	0.9847	0.3528	0.2102	
0.2973	1.0037	0.229	0.4404	0.2553	0.935	1.2406	0.484	
0.1988	0.182	0.411	0.6059	1.6168	0.4163	0.4171	0.2468	
0.2722	0.5337							
ADPRHL2	3.5307	4.9685	4.0087	3.6015	4.7625	3.9363	4.6737	
3.704	4.3791	3.9196	3.9259	4.2579	4.0536	3.6919	3.6375	
4.097	3.8773	2.9607	3.9579	4.3107	4.3756	3.9909	3.6148	
4.7693	3.1766	4.4891	2.3583	4.242	3.845	4.6607	4.6329	
4.9347	4.5805	3.9282	3.8953	3.4475	4.0639	4.3483	3.3687	
3.106	4.2485	4.5222	3.1171	3.6889	4.1318	3.6587	3.0478	
3.9892	3.4414	4.0598	4.0234	3.7188	3.7636	3.6956	4.2028	
3.8258	3.9026	3.9905	3.7672	3.9488	4.0175	4.9787	3.1771	
4.2639	3.3452	4.7876	3.729	4.1651	4.279	3.321	3.4377	
3.7725	3.7771	3.599	3.3644	3.7228	5.0414	3.5217	3.9997	
4.483	4.1902	4.2413	4.3984	2.4338	4.1893	4.3943	3.8162	
3.5513	3.5699							
ADPRM	2.2408	3.1147	2.1893	3.1059	2.7452	1.9907	3.7471	
1.5532	1.9072	2.0086	2.2397	2.4454	2.5782	1.9211	1.7796	
2.6538	2.9644	2.6647	1.9023	1.8987	1.9656	2.1743	2.1902	
2.4158	1.6638	3.8737	4.8115	2.6393	2.2632	2.4802	2.0489	
1.7316	2.3687	2.1567	1.2413	2.4076	2.0648	2.2055	2.5195	
2.4006	3.1332	2.7561	1.765	2.348	2.2349	2.7553	2.713	
1.9734	1.9667	2.8595	3.1005	2.9833	2.7742	2.9927	2.5539	
2.1103	3.2553	1.0474	1.9045	1.5272	2.803	2.9936	3.3839	
3.0745	2.1606	3.266	1.8895	2.6167	2.4027	2.7284	2.4106	
2.7536	2.0695	2.08	2.3095	2.1174	2.2984	2.1199	2.6964	
2.7864	2.5442	3.1576	3.5241	1.6685	1.214	2.4787	2.9712	
2.4049	2.9241							
ADRA1A	0.1247	0	0	0.148	0.0127	0	0.142	
0.1953	0.1785	0.0359	0.0482	0.0563	0.0538	0.1853	0.0262	
0.0554	0.1657	0	0.1175	0.2313	0.1874	0.0691	0.033	
0.104	0	0.1557	0.0494	0.191	0.0662	0	0.0826	
0.0417	0	0.0847	0.0024	0	0	0	0.1098	
0.1147	0.0293	0	0.0408	0.0878	0.0882	0.0431	0	
0.1391	0	0.1225	0.0363	0.1224	0.0879	0.0667	0.1327	0
	0	0.0503	0.0567	0.1307	0.0686	0.0313	0.0378	0
	0.0609	0.0124	0.074	0.0664	0.028	0.3139	0.0425	
0.0801	0.0943	0.0295	0.0663	0.0426	0.0582	0.0388	0.0233	0
	0	0.0263	0.0692	0.0468	0.0688	0.1133	0.0941	0
	0.1094							
ADRA1B	0	0.6135	0.0711	0.0936	1.015	0.781	0.198	
0.289	0.2939	0.4797	0.0235	1.7191	1.8634	0.9117	3.9719	
1.5261	0.8655	0.0742	0.2485	2.4012	0.7145	0.5213	0.6183	
1.7615	1.5401	0.2835	0.059	0	1.792	0.0495	0.2655	
0.8236	0.5803	0.432	1.1253	0.3006	1.4099	0.0509	3.5031	0
	0.263	0.177	3.7423	0.2816	0.9086	0.3451	0.076	
0.9804	0.2001	0	0.1571	0.8026	0.2426	0.685	0.3851	
0.7391	1.448	3.9877	0.3297	3.4617	0.915	0.4271	0.0687	
0.6886	0.0384	1.2965	0	0.1396	0	2.218	0.4581	

1.3641	0.1137	1.3292	2.6447	0.0249	0.1031	2.114	0.3008	0
	0.0513	1.6896	0.5263	0.1813	0.7309	0.2447	1.111	
0.5056	0.4142							
ADRA1D	0	0	0	0	0.0408	0	0.2111	
0.0781	0.0807	0	0	0.8982	4.1347	0	1.7359	
0.0341	0.0894	0	0	0	0	0.1361	0.1192	0
	0.1195	1.3221	0	0	0.2602	0	1.1463	
0.0308	0	0	0.3446	0	0	0	0.1376	0
	0.038	0.1665	0.6465	0	0.0736	0.4804	0	0
	0.0377	0	0.1617	0	0	0.1257	0.019	
0.1244	1.0494	0	0.0236	0	0.0671	0	0.1032	0
	0	0	0	0.1061	0	0	0	
0.0333	0.0463	0.0061	0.067	0	0	0.2624	0.0186	0
	0.1516	0.0658	0.9685	0	0.3585	0	0	0
	0							
ADRA2A	0	0.1632	0	0.6895	0	1.4858	0.0209	
2.0105	0.1075	0.0261	0.4024	0	0	0.1423	2.4145	
0.7905	0.053	0.1188	0.2825	0.1368	0.0785	1.3404	0.1612	
0.0189	2.5061	0.0401	3.2747	0.5815	1.9155	0.7147	0.3149	
0.7841	1.1562	0.1585	2.6465	0.0424	0.0135	1.416	0.1084	0
	0.3071	0.9009	0.4403	0.2724	0.0188	0.0798	0.0775	0
	2.0798	0	0.0494	0.1427	0.0612	0.2137	0.0554	0
	0.2099	0	0.7968	0.0299	0.9158	0.0706	0.5896	
0.3392	3.5067	0.4937	0.4865	2.3271	0.2956	0.4118	0.3185	
0.3571	0.0525	0.063	2.7932	0.3789	0.3789	1.0695	1.0523	
0.1836	0.369	0.8744	0.0956	0.3413	0.1798	0.1258	0.0391	
0.2303	0.0485							
ADRA2B	0	0	0	0	0	0.1042	0	0
	0	0	0.0238	0.0331	0.0131	0.317	0.0269	
0.1168	0	0	0	0.0427	0	0	0	0
	0.0987	0	0.0523	0.045	0.0559	0	0.4108	
0.0094	0.3257	0.1064	0.0051	0.0646	0	0	0	0
	0.079	0	0	0.2339	0.0142	0.0243	0	
0.0271	0.3755	0	0.021	0	0.1573	0.0028	0.1875	
0.0836	0.0134	0.2847	0	0.0116	0.0126	0.0017	0.0048	
0.1718	0	0	0.1694	0	0	0.0374	0.1181	
0.3201	0.0331	0	0.0062	0.0091	0.0118	0	0.3059	
0.16	0	7.00E-04	0.0958	0	0	0	0	
0.0328	0							
ADRA2C	0.3953	2.8249	4.2158	0	0	0.4668	0.7282	
0.1076	0.54	0.1179	1.5552	0	0.475	0	0.749	
0.3025	0.337	0.1769	0	2.9367	2.1103	0.1828	0.0722	
0.1234	1.1999	1.126	0.151	3.0188	0	2.8015	3.0293	
0.0582	1.2878	0	0.1556	0.5594	0.0848	0.1965	3.9709	
1.2858	0	0	0	1.7373	0	0	2.7675	
1.3781	0	1.2934	0.7269	0	0.3382	1.0435	0.7288	
1.6519	0.0601	0	0	0	1.3503	0.5407	3.2039	0
	2.29	0.2836	3.8943	4.7789	0.8259	0.1229	0	0
	2.2106	0	1.1726	0	0.0024	1.7855	2.2535	
1.6284	0.4138	0	2.0193	0.7155	0.0536	0.4605	0.1311	

1.2413	0.1433							
ADRB1	0	0.1214	0.4788	0.1651	0.2871	0.0625	0.1344	
0.4427	0.1369	0	0.4386	0.4868	0.189	0.1799	0.1442	0
	0.071	0	0.1994	0.1701	0.1106	0.4026	0.1448	
0.1641	0.6493	0.0564	0.9474	0.1635	0.1815	0.1174	0.4556	0
	0.1138	0.0725	0.1061	0.5573	0	0.1822	0.0578	
0.2409	0.0624	0.0495	0.4859	0	0.075	0.0431	0.419	
0.5664	0.2938	0.6571	0.3068	2.0499	0.2722	1.2709	1.3049	
0.4761	0.5675	0.4329	5.00E-04	0.107	0.2779	0.2923	0.4957	
0.0074	0.1109	0.0806	0.014	0.006	0.0839	0.1808	0.5003	
0.2449	0.2978	0.6381	0	0.1158	0	0	0.1047	0
	0.5591	6.00E-04	0	0.2396	0.1827	0.1932	0	0
	0							
ADRB2	0	0.8034	1.9894	0.3118	1.4368	2.2176	0.6423	
0.6255	0.2004	0.0689	1.5206	1.7591	3.0426	0.0808	3.4878	
0.4855	1.4773	0.3696	0.2501	3.4811	2.112	0.4939	0.6772	
0.6906	0.7156	1.8206	1.2271	0.7347	1.081	0.2278	0	
0.1005	0.3581	0.1799	1.3754	0.1186	1.2279	1.7109	0.2006	
0.3805	0.9682	0.6721	0.7564	1.1362	1.1682	1.8265	2.0018	
0.2754	2.4618	1.9926	0.5617	0.7551	0.2675	0.5657	0	
0.8389	3.515	2.6112	0.2628	2.6592	1.5653	1.7213	1.2032	
0.3439	2.4875	1.5922	3.332	1.7711	0.0727	2.2321	1.1483	
1.4258	0.4359	0.2844	0.5972	0.0877	1.4096	0.2163	0.1724	
0.5292	0.1612	1.6761	2.1855	0.1529	1.7449	0.2895	1.3037	
1.83	1.5249							
ADRB3	0.0612	0	0	0	0	0	0	0
	0	0	0	0.0101	0	0	0	0
	0.1184	0	0.2726	0	0	0	0	0
	0	0	0	0.2492	0	0.9394	0.0181	
0.1801	0	0.1556	0	0	0	0	0	0
	0	0	0	0	0	0	0.0442	0
	0.0207	0	0	0	0	0.0314	0	0
	0	0	0	0	0	0	0.0793	
0.0694	0.0673	0	0	0.1209	0.2243	0	0	0
	0	0	0	0	0.0566	0	0	0
	0.2283	0	0	0	0	0	0	0
	0							
ADRM1	6.7307	7.1764	7.0773	5.2729	7.0693	6.0464	5.8455	
7.3608	6.1894	6.5242	6.0547	5.8735	6.7048	5.5747	6.3291	
6.795	5.9645	5.5314	6.2384	6.3492	7.4419	7.1119	6.6067	
6.9537	6.5986	6.6563	5.796	5.8609	6.3831	5.872	6.7078	
7.244	6.9075	5.6972	6.1343	6.2147	7.1198	6.3763	6.3697	
5.5722	6.1753	6.4924	6.072	5.8027	5.8004	5.4327	5.4224	
6.4372	6.623	5.3485	5.9525	5.7926	5.7378	6.1319	6.6386	
6.7483	5.4933	6.0392	6.6302	5.9766	7.7108	6.7037	6.2181	
6.9508	5.6896	5.8022	5.9236	5.9941	6.7767	6.4679	6.6706	
7.2671	7.3857	6.841	7.1238	7.6425	6.8801	6.2025	5.5763	
6.4291	5.9836	6.279	7.0661	6.0189	7.0142	6.6918	5.3353	
6.1343	5.8947							
ADSL	6.2714	6.6587	6.2549	6.2841	6.0424	5.924	5.5511	

6.3562	6.5806	6.5077	6.0533	6.5044	6.256	6.9048	6.1825	
5.5217	5.6595	5.3704	7.1629	5.7872	5.6892	6.458	5.8898	
7.3961	6.2729	5.4093	5.9738	6.3343	6.4702	6.3699	5.4085	
5.52	6.5176	5.568	5.5612	6.5581	6.0233	4.913	6.6859	
6.5349	6.1265	5.7313	5.305	7.0784	5.699	6.0224	6.9875	
6.1401	5.843	6.1641	5.7228	6.3086	6.8912	6.1152	6.1712	
6.2322	5.4709	6.1447	6.587	6.0768	6.5256	6.9204	6.6146	
6.2126	5.9133	4.7269	6.4436	4.8664	5.9339	5.5662	6.2547	
5.8554	6.0264	6.6986	6.3736	5.6987	6.0218	5.7387	6.3414	
5.9935	6.9246	6.4185	4.8514	5.3975	6.4423	6.7502	5.2251	
5.4266	5.4981							
ADSS	4.8241	4.7649	4.4153	5.7223	5.6345	5.4737	5.1508	
3.8008	5.4615	4.8751	4.0167	5.2721	4.814	6.0752	5.3131	5.7
	4.4412	5.9181	4.7439	4.9703	5.5682	5.0307	5.3407	
5.8307	5.1706	4.8853	4.9958	5.115	5.5793	5.5666	4.3948	
5.6434	5.2651	4.7508	4.9334	5.1535	5.4522	4.2866	5.7145	
5.7537	5.1608	4.7449	5.0134	5.4299	5.1416	4.9644	4.8185	
5.7923	5.7865	5.1595	5.2439	5.9034	5.6867	4.7857	4.7716	
5.2006	5.0737	4.7363	6.2855	4.7846	5.5084	4.9908	5.3159	
5.4239	5.2467	4.2488	4.9555	4.433	4.6134	5.5075	5.6186	
5.6186	4.8271	5.4163	6.0201	4.5898	5.4507	5.9319	5.6362	
4.693	4.5485	4.9747	4.7215	5.9041	5.4624	5.5364	4.5404	
4.4651	4.4836							
ADSSL1	4.0965	0.9206	0.4876	1.6176	0.8591	1.94	0.9062	
3.2615	1.2698	1.611	2.2852	0.8635	1.2322	1.3733	1.4119	
1.1861	0.6916	1.3791	1.3173	0.6098	1.5283	0.9311	2.129	
0.9961	0.9999	0.5083	1.2348	1.0498	2.6081	1.7078	1.6768	
0.8561	0.5452	2.3682	1.2439	1.0298	2.4218	3.2752	2.7597	
1.7423	1.7276	2.5425	0.892	2.7124	1.6423	0.9594	1.2514	
0.8384	0.6984	1.2615	0.6624	1.0749	1.0385	1.1504	0.7871	
1.439	1.8252	1.5791	1.852	1.0641	0.872	0.9308	2.2305	
0.3579	2.556	1.4635	3.7463	0.6596	0.3503	1.1432	1.7776	
1.4677	2.6576	2.0404	3.0796	0.14	2.0558	1.3156	1.9025	
2.1547	0.9244	0.498	1.0628	2.7574	0.7263	1.4875	2.3974	
1.6768	2.8034							
ADTRP	0.122	0.1903	0.0669	0.129	0.1731	0.0752	0.0741	
0.0123	0.1477	0.0446	0.0701	0.7309	0.6859	0.4634	0.008	
0.0784	6.6445	0.4708	0.2409	0.1993	0.2626	0.0683	0.0641	
0.4235	0.0145	0.3779	0.3051	0	0.1296	0.022	0.0356	
0.129	0.1045	0.3284	0.1687	0.0868	0.9476	0.1599	0.0964	
0.1358	0.2167	0.2735	0.0488	0.2341	0.0807	1.0853	0.206	
0.0275	0.3972	3.7217	0.4502	0.2366	0.3864	0.097	0.0606	0
	4.9856	0.035	0.0532	0.1099	0.0668	0.0478	0.0269	
0.1161	0	4.5132	0.0533	0.173	0	0.1279	0.4478	
0.1691	0.0611	0.0278	0.12	0.1002	0.2092	0.1396	0.1801	
0.509	0.6693	0.0365	0.2189	0.0301	0.085	0.3994	0.6165	
0.277	0.2489							
AEBP1	5.4068	4.5839	0.6192	5.2219	4.1919	4.7979	0.2155	
5.0039	3.1261	5.1592	1.8734	4.5619	0.1951	5.1862	0.5262	
5.6801	0.3013	6.0079	5.2322	0.5904	0.989	5.716	3.2867	

3.3421	6.0382	5.2665	4.502	5.6728	5.2365	4.5695	0.3828
3.6202	0.7019	1.0864	7.2135	2.7638	3.1705	7.6298	6.791
0.2373	5.5308	3.1913	4.8532	5.3192	0.8747	2.5651	5.095
5.4956	6.5895	3.9761	4.6717	2.262	6.0314	6.4061	0.1019
0.4632	3.1828	4.5289	5.1185	4.1493	3.0886	2.9775	2.6917
1.9996	6.4061	0	5.87	3.5646	4.5823	6.1664	2.9674
4.5671	5.6194	5.5589	6.6083	4.7229	5.0074	6.0427	5.5314
4.94	6.5174	5.4991	2.9907	6.4101	4.907	6.4286	4.6267
4.9553	5.1189						
AEBP2	2.8558	3.8672	0	3.4075	3.4674	3.0281	2.5891
3.4386	3.1252	3.0167	3.0247	2.6647	3.4693	3.0968	3.2357
2.888	4.2518	3.3198	4.0799	2.9595	2.4655	3.3524	2.9859
2.6521	3.016	1.9897	3.6452	4.0015	2.6426	4.1877	3.1799
3.541	2.8512	2.5658	3.7695	3.1766	2.8977	3.3159	3.3659
3.3594	3.0961	2.9261	3.3431	3.2623	3.7994	3.5606	3.9142
3.5937	3.2558	4.44	4.0709	3.1065	3.785	3.051	2.647
3.0802	3.8522	5.8067	3.3142	3.9951	3.066	3.2244	3.6092
2.8866	2.9411	3.6266	2.6724	2.5482	3.3585	2.5956	3.1141
2.719	3.2389	2.9883	2.736	2.9275	3.2376	2.3402	3.3707
3.0802	4.0609	2.8164	3.1208	3.0599	2.1069	3.6913	2.6977
2.6827	2.7843						
AEN	5.1594	4.5966	5.9485	3.7213	5.2831	5.2869	4.9098
5.7792	3.8552	5.285	5.2692	3.4782	3.7916	3.6421	4.183
5.8927	5.2487	5.2602	5.1303	4.9299	4.3814	4.2693	4.5488
5.0704	5.1197	4.9061	4.7508	4.6525	4.8716	5.2642	3.2866
4.4009	4.7479	2.509	4.5817	4.9672	4.1348	4.3527	4.9525
4.696	3.8429	3.4692	4.6849	4.8661	5.1809	4.1184	5.091
4.628	4.9034	4.3727	3.4098	4.0507	3.6284	4.6152	4.1439
4.713	4.6397	3.9588	5.0532	3.9442	5.3641	5.2717	4.6477
4.2473	4.2449	5.0511	5.2925	5.0637	5.8908	4.641	4.4783
4.0893	5.4488	3.1225	5.3949	4.8465	4.7956	4.6775	4.5718
5.0688	3.283	4.2753	6.5334	3.7512	4.7175	3.6265	4.0103
4.7421	4.0823						
AES	6.7155	6.5397	7.6512	5.1012	6.8513	6.6517	6.1287
6.2616	6.6117	6.2556	7.1429	6.0608	7.3396	5.9238	6.6796
6.6517	7.013	5.7949	6.1571	7.2702	7.1252	6.7004	6.5024
5.7122	5.721	6.8242	6.9202	7.4223	6.1505	7.134	7.2122
5.7084	5.7695	7.2412	6.5482	5.6639	6.4653	7.1896	7.1998
5.5047	6.4176	6.9617	6.6375	6.6149	7.2268	6.1505	5.7403
6.8602	7.4551	5.7361	6.369	5.5149	5.9393	6.7018	6.7463
6.9191	6.9046	7.6177	5.2758	7.1464	6.3623	5.803	5.7801
6.834	6.658	6.847	6.5869	7.3689	7.5551	6.8934	7.0009
6.9105	6.7933	5.6924	6.4057	7.5026	6.7115	5.947	6.953
6.9856	6.9561	6.834	6.5094	6.9623	7.9798	6.2628	6.9559
7.1175	7.197						
AFAP1	3.1498	0.1078	1.6514	4.0719	4.9403	3.6984	2.9976
4.0281	3.8741	2.7831	1.2779	5.2957	4.8005	3.0158	3.3734
1.519	3.9229	1.6693	1.5881	2.3371	1.5241	4.1669	4.4491
3.391	4.4875	6.5483	4.2402	2.4795	3.0722	3.0639	1.3679
2.3134	1.2681	2.1525	4.8758	2.625	4.0923	4.1005	4.0119

3.2005	2.2875	5.1715	5.6952	4.0106	4.3868	3.8729	2.3932	
3.9554	5.5871	3.748	2.8802	4.7202	2.9601	2.8562	3.9037	
1.9702	4.4271	3.4705	0.8772	4.0465	3.701	1.2325	5.2402	
2.4241	3.3818	3.0784	2.8549	3.519	1.941	5.6246	3.1027	
2.8318	2.0075	5.2448	4.4711	4.6793	1.5291	4.3771	1.184	
5.0428	1.6284	5.3731	3.4412	5.0326	4.6216	2.6909	2.4508	
2.3956	2.4143							
AFAP1L1	3.4957	2.4824	3.4941	2.7692	2.7909	3.7908	2.5358	
2.607	3.8836	3.0441	3.3243	2.6417	2.0739	3.5712	3.6013	
2.9228	2.2685	2.2955	3.7614	3.9107	2.9352	3.5922	3.0655	
3.8655	3.5745	2.6634	3.5488	2.612	4.2719	2.6713	1.3756	
3.8147	3.7343	3.6785	4.0332	2.6376	1.0597	2.8829	2.5504	
4.2145	2.5491	1.0855	3.3121	3.8588	0.6516	1.205	3.7004	
3.4652	3.6579	2.7352	3.4361	2.8526	3.4728	3.972	0.9315	
3.1001	3.3431	3.3537	2.8085	3.3631	3.8247	3.7812	3.5137	
3.4889	2.4662	0.5131	2.4111	2.1649	3.0296	4.5057	3.7323	
3.8903	4.0191	3.3747	3.3463	2.4579	3.0275	3.6979	3.8464	
3.2613	3.3467	3.3908	2.5021	3.0745	3.7611	3.0874	3.09	
3.1801	2.891							
AFAP1L2	2.5025	0.2167	0.5212	1.7743	2.5251	1.4744	0	
1.3155	1.5676	0.3249	0.5262	3.1617	3.9509	1.2611	2.7658	
2.8012	3.00E-04	2.7358	0	0.0923	0.0888	3.162	3.4538	
2.0229	1.4255	0	1.8289	0.8939	3.8134	2.0131	1.317	
1.3582	0.4741	0.0709	1.2838	1.2226	2.406	0.5585	2.0131	
4.2251	1.0459	2.4904	3.5647	0.3534	1.2655	2.6477	1.4061	
4.9597	0.1193	0.2253	1.0872	1.6696	3.1245	3.686	1.2433	0
	1.5223	2.4521	0.5253	0.7589	1.281	0.5266	2.6067	
0.495	1.6388	0.5352	0.8665	0.4956	3.7797	4.906	0.344	
0.4259	0.5375	4.1112	0.7017	1.445	1.0642	1.8352	0.7082	
0.3013	0.5222	3.082	1.6746	4.0225	0.5954	3.422	0.8578	
1.6372	0.5148							
AFF1	3.178	3.1695	3.7123	5.0078	2.8794	3.7123	5.9471	
2.683	3.6481	3.8024	4.2306	3.7584	2.6455	4.3255	3.4673	
3.6079	4.3508	4.3589	4.6224	4.5536	3.0725	2.6642	2.6576	
3.3358	1.9207	5.5503	3.6047	5.198	2.0685	4.5438	3.5354	
4.4283	4.3611	4.835	3.283	3.7559	3.9377	4.0401	2.8757	
2.7228	3.9707	5.3935	3.3292	3.6629	2.7379	4.3915	3.9258	
3.2855	2.2952	4.9813	4.0032	3.37	4.3169	3.0616	4.2536	
2.8419	6.3604	4.6868	3.835	3.6281	2.2915	4.177	2.6296	
4.4271	2.8322	4.6921	3.3244	3.769	4.2413	3.6577	4.2328	
3.4731	3.841	3.3809	3.0266	4.9332	4.0991	3.3635	3.7809	
2.4471	3.7596	3.9308	4.6535	2.8936	1.3197	3.2224	4.8378	
3.2222	3.5098							
AFF2	0.0543	0.0399	0.795	0.0559	0.13	0.1008	0.286	
0.3875	0.0809	0.0968	1.087	0.0961	0.0366	0.0522	0.931	
1.4001	0.0508	0.0477	0.176	1.5349	0.0734	0.8459	0.9867	
1.303	0.5995	0.0843	1.159	0.0612	0.585	0.059	0.045	
0.0612	0.236	0.1231	0.6873	0.0461	0.2195	0.3434	0.4149	
0.0625	0.1129	0.6042	1.145	0.2388	0.0341	1.6511	0.1518	
0.1943	0	0.0794	0.039	0.9105	0.1714	0	0.102	

0.4525	0	0.9437	0.46	0.0901	0.4381	0	0.5345	
0.3256	0.3062	0	0.0129	1.1071	0	0.3235	0.718	
1.0369	0.1091	0	0.9707	1.3062	0	0.501	0.4625	
0.403	0.2186	0.3442	0.0637	0.4409	0.2438	0.0289	0.1479	
0.1073	0.1692							
AFF3	3.355	2.2741	2.1399	1.7545	0.6752	0.7595	0.9739	
1.806	2.4763	3.1543	3.0424	1.1613	2.7458	1.7938	1.3953	
1.8822	0.3854	3.0284	2.8495	1.7927	2.034	0.151	0.864	
1.6186	1.3425	3.0624	1.7078	2.3832	0.7022	2.5827	0.9546	
1.6112	0.9884	2.156	1.0388	2.1717	2.8384	2.4378	2.1656	
0.9964	0.7559	1.2038	2.6392	1.3449	1.2503	0.1796	1.6527	
0.9608	2.1946	2.6423	2.5202	0.4101	2.4979	2.8247	0.4097	
2.1771	1.7691	0.8824	2.6634	1.3556	1.3723	2.2667	1.2202	
1.9929	1.5879	2.4331	0.6901	1.6918	3.0915	2.4596	1.0206	
1.904	0.9195	0.3757	1.0308	2.9028	2.4722	2.4986	1.6002	
2.6093	2.6526	0.5109	1.7507	1.8766	1.6731	2.0854	1.994	
1.1652	2.0262							
AFF4	4.2604	3.6452	4.8109	4.5466	4.5914	5.0669	5.5197	
4.6802	5.1188	4.92	5.0333	4.5796	5.1749	4.7354	5.2055	
4.8881	4.6826	4.4262	4.9046	4.8515	3.8756	4.2971	4.322	
4.593	4.8556	5.2712	3.8379	4.8457	4.3462	4.1612	4.5641	
5.1571	4.4987	4.8693	5.2099	4.8951	3.763	5.139	3.9801	
5.1325	4.3709	4.4924	4.47	3.9666	4.9285	4.5068	4.5946	
4.8295	4.5983	4.5028	5.0126	4.6371	4.9053	4.5468	3.8604	
3.9321	4.8135	4.4383	4.7582	4.3939	4.0804	4.8915	4.5813	
4.7402	4.5934	4.8991	5.0586	5.32	4.7241	4.5622	4.5222	
4.7484	4.6914	4.6121	4.1362	5.1291	4.9172	4.7502	4.8504	
3.9168	4.5559	4.7001	5.6462	5.1581	3.5191	4.1213	5.0627	
4.8142	4.9645							
AFG3L2	4.8472	5.5985	5.2935	5.303	4.6476	5.5694	5.3965	
4.9402	6.491	5.5652	6.2643	5.0381	4.1823	6.4673	5.1441	
4.5239	5.0818	5.084	5.9522	5.7294	5.7249	5.1502	4.8008	
5.5448	4.6071	4.3688	4.666	5.4912	4.362	6.201	4.7587	
5.4513	5.0928	4.591	4.1239	5.6771	5.6548	6.0059	5.5532	
5.4707	5.7506	4.7293	3.5754	5.7506	5.2702	5.0951	5.5925	
5.9699	4.8317	5.0308	5.6937	5.0417	5.6947	5.3706	5.1796	
5.4318	4.787	4.9254	6.2905	4.9431	5.1485	5.9558	5.5706	
5.9451	5.97	4.2943	5.6956	4.6829	5.7131	4.5075	5.3821	
5.427	5.2765	5.0756	4.549	5.1672	5.4225	5.0195	5.8856	
6.2387	5.1333	4.795	4.4807	4.9475	3.9082	5.6763	5.2935	
5.4194	5.4325							
AFM	0	0	0	0.0453	0	0	0	0
	0	0.0302	0	0	0	0	0	0
	0	0	0	0	0	0	0.0636	0
	0	0	0	0	0	0	0	
0.022	0	0	0	0	0	0	0	0
	0	0	0	0	0	0	0	0
	0	0	0	0	0	0	0	0
	0	0	0.0288	0	0	0	0	0
	0	0.0034	0	0	0	0	0	0

	0	0.0117	0	0	0	0	0	0
	0	0	0	0	0	0	0	0
	0							
AFMID	3.7399	3.7505	3.2366	3.7	3.1297	3.4738	2.5333	
3.2999	3.3151	3.004	4.6984	3.3316	3.1551	3.9165	3.2594	
3.6908	4.1768	2.748	3.2313	4.5491	4.0883	3.3755	2.33	
3.6527	2.3756	3.3633	3.5965	4.1505	2.8971	4.2727	3.4079	
3.6607	3.4807	3.274	2.8794	3.0574	4.1289	4.1871	3.3718	
3.6323	2.8455	2.5627	2.8266	3.3854	3.5366	2.3377	4.3308	
4.1871	2.6167	3.829	4.0049	2.4738	3.3359	3.8502	4.4331	
3.8726	3.1633	3.6721	2.3003	3.2586	3.0262	3.1723	4.1574	
3.2253	4.4372	3.365	4.2825	3.2114	4.0106	2.3291	3.2151	
3.0946	4.088	2.8523	3.4371	3.363	3.0423	2.9007	4.0691	
2.846	4.0993	2.5112	2.4372	3.5089	3.0847	2.9702	2.6371	
3.1362	2.7732							
AFP	0	0.0359	0	0	0.1219	0	0.0728	
0.0194	0	0	0	0	0.359	0	0.4478	0
	0.8963	0	0	0	0.0605	0.0512	0.1423	
0.1009	0	0.7472	0	0	0.0246	0	0	0
	0	0	0.6709	0	0.0101	0	0	0
	0	0.053	0.0455	0	0	0.1186	0	0
	0	0	0	0	0	0	0.0263	0
	0.2117	0	0.028	0	0.027	0	0	0
	0	0.3521	0	0.0271	0	0	0.0636	0
	0	0	0	0	0	0.1976	0	0
	0	0.0815	0	0	0.1604	0	0.0464	0
	0							
AFTPH	3.1067	3.6515	3.3262	3.3987	3.5447	3.5003	4.0862	
3.0577	4.0012	3.4204	3.4886	3.8314	2.8647	3.706	3.8688	
3.9929	3.8384	3.9235	3.6247	3.0668	3.2178	3.5227	3.3613	
3.829	3.1836	3.5649	4.3258	3.5344	3.4232	3.9254	2.8799	
3.4197	3.7537	3.5634	3.1809	3.5834	3.7454	3.5003	2.9945	
3.294	3.3465	3.4691	3.4468	3.2464	3.6156	3.4237	3.7175	
3.8751	4.3917	3.5443	3.3915	4.1137	3.8827	3.3348	2.4506	
3.1606	3.3872	2.9715	3.3629	2.9999	3.3527	3.4552	3.1063	
3.6344	3.1723	3.8355	3.8045	3.7711	3.837	3.2525	3.541	
3.5304	3.0086	3.8751	3.8149	3.6905	3.5049	3.3405	3.6283	
3.8149	3.647	3.4704	4.2018	3.4727	2.5659	3.4661	3.1446	
2.7665	3.0153							
AGA	4.033	3.8747	3.6251	4.5504	3.9763	3.8114	4.7532	
2.2087	3.2851	3.0548	2.9138	3.896	3.5894	3.5898	2.8968	
3.3228	1.962	4.7052	3.3653	4.0518	3.3758	4.2281	3.9015	
3.1494	3.4157	3.7465	3.9672	3.9693	2.9287	1.9835	3.3029	
4.7682	4.1476	4.7233	3.8412	4.2557	3.7999	3.9257	3.7797	
3.2939	4.0979	3.5451	3.1464	4.0031	3.4124	3.3268	4.2053	
3.0878	2.9938	3.01	4.3249	4.5168	2.7183	3.0011	3.5814	
3.1433	2.7997	3.1249	3.8293	2.8672	3.6812	4.2153	4.1553	
3.2675	3.378	3.8508	3.0894	2.7833	3.0652	3.7318	5.1281	
3.5859	3.6612	4.2161	4.8405	3.5313	3.5217	4.3506	4.1688	
3.6831	4.0009	3.5029	3.1745	3.9782	2.1189	3.0182	3.9187	

3.5265	3.07						
AGAP1	3.2045	2.6746	3.6065	2.7152	2.5615	2.2341	2.8423
3.8146	3.1304	3.2735	3.1469	3.5369	2.4799	3.0313	3.637
2.6671	2.9661	2.1005	3.3046	3.0588	2.934	3.0259	3.2814
3.8105	3.2381	2.5765	3.3459	2.0332	2.9533	2.2011	2.9902
2.9187	3.3015	2.9488	2.7075	2.3879	2.7052	2.6442	2.8016
3.259	2.3748	2.1665	3.6969	3.7621	2.7056	2.6726	2.6721
3.6679	3.6383	2.2383	2.7698	2.6217	2.7481	3.1057	2.6644
2.7519	2.7967	4.8049	2.9808	4.323	2.3969	2.4354	2.447
2.6925	3.1307	2.2803	3.0258	3.2307	3.4179	2.7878	3.1057
2.4025	3.0615	3.1925	3.0251	3.4508	2.8034	3.398	2.9663
2.9928	3.4929	3.3189	3.5175	3.0094	2.2642	2.4094	2.9105
3.076	2.2165						
AGAP2	0.247	0.4047	0.1207	0.137	0.9891	0.0618	0.05
0.1018	0.083	0.7107	0.6825	0.3609	0.1296	0.3257	0.5891
0.2145	0.806	0.1372	0.0766	0.5801	0.4211	0.39	0.0879
0.0881	1.6989	0.6168	1.7465	0.3724	0.0526	0.5236	1.4725
0.7293	0.0116	0.1207	0.0189	0.1396	0.1667	0.2682	0.3599
0.0107	0.1848	1.8371	0.8118	0.3548	0.9212	0.0112	0.1939
0.2673	0.2482	0.1879	0.1009	0.0289	0.2077	0.2011	0.4931
0.1165	1.7798	0.6235	0.4969	0.1918	1.0036	0.0306	0.5113
0.1548	0.0573	0.0613	1.2272	0.6203	0.062	0.1531	0.4562
0.0301	0.0427	0.8689	0.2045	1.6806	0.0996	0.0181	0.1948
0.4711	1.0831	0.2075	0.4211	0.2303	0.219	0.0625	0.0464
0.0623	0.1177						
AGAP3	4.942	6.0936	5.3904	4.4535	5.0954	6.0743	4.9839
5.242	5.3806	5.8348	6.4111	4.9388	4.8274	4.6845	4.7755
6.6011	4.2845	6.279	5.4551	6.261	5.4702	6.2112	5.4526
5.8652	5.5805	4.091	4.9756	4.8386	5.7877	6.0305	4.591
6.0294	5.2518	5.1819	5.5258	4.7002	4.9694	5.4734	5.9969
4.5027	6.1067	4.4535	4.7527	5.924	4.0688	3.8617	4.3915
6.2829	6.5639	3.2193	5.6133	4.6456	6.3166	5.0166	4.2538
6.1672	3.0935	6.1397	6.6238	6.1689	5.1557	6.5478	5.978
5.2056	4.9111	4.7765	4.7435	5.2048	5.5474	5.505	5.5137
5.9615	5.6968	5.1584	4.7167	6.1316	5.0847	5.7721	5.6599
6.0831	5.7016	4.4534	6.1146	5.399	5.4395	6.8426	4.9439
5.2275	5.5055						
AGAP5	0.7824	1.1325	0.7714	0.8647	0.3056	0.6098	0.1927
0.4874	0.4088	0.4844	0.2577	0.2047	0.4142	0.8574	0.5962
0.3536	0.8616	0.7139	0.4871	0.3162	0.6829	0.247	0.2059
0.446	0.2792	0.5176	0.2966	1.0028	0.2387	0.2504	0.4114
0.4193	0.2245	0.4604	0.3104	0.4237	0.5846	0.2849	0.3061
0.3372	0.5749	0.2849	0.3477	0.6636	0.4975	0.6839	0.3329
0.3928	0.3917	1.3145	0.438	0.3113	0.4168	0.8409	0.7309
1.0759	0.6158	0.4105	0.7039	0.3814	0.2547	0.4517	0.3614
1.0364	0.658	0.479	0.4855	0.5198	0.1998	0.4082	0.2452
0.5981	0.2081	0.1344	0.5374	0.3329	0.8717	0.2044	0.332
0.5594	0.5183	0.6508	0.1441	0.3893	0.3267	0.624	0.4284
0.6954	0.5037						
AGAP6	2.0523	2.1108	2.013	2.0137	1.3868	1.1153	1.208

1.1959	1.807	1.7077	1.1355	0.7463	1.1394	1.8758	1.2869	
1.0355	1.841	1.8276	1.7102	1.564	2.179	1.2421	0.9188	
1.8028	1.5555	1.3248	1.547	1.6423	1.015	1.1536	0.7302	
1.7186	0.809	0.7779	1.5934	1.3413	1.9039	1.0678	1.0553	
1.7498	1.5444	1.2979	1.1823	1.6524	1.3874	1.0433	1.4227	
1.0037	1.2645	1.9958	1.3503	1.2617	1.5034	1.8043	1.7582	
2.4121	1.309	0.7444	1.3269	1.0498	1.0492	1.3064	2.0211	
1.7476	1.5667	1.3956	1.6701	1.0013	1.6236	1.0121	0.8941	
1.3886	0.9605	1.0462	1.5072	1.3654	1.6546	1.188	0.9488	
1.8475	1.6673	2.046	1.1165	0.8923	1.2497	1.5349	1.0097	
1.4376	1.4215							
AGAP9	0.9378	1.3887	1.2522	0.657	0.8923	0.8788	0.6959	
0.7886	1.4305	1.3515	0.5561	0.3623	0.8741	1.4425	0.9758	
0.7753	1.504	1.2733	1.254	1.1968	1.209	0.5082	0.6031	
0.5327	0.3179	1.3987	0.6907	0.9172	0.7026	0.3623	0.3945	
0.8805	0.4589	0.5625	1.1685	0.6344	0.877	0.564	0.68	
1.0397	0.3933	0.7208	0.7102	1.1253	1.1938	0.9718	0.6684	
0.4035	0.3686	1.4617	0.7341	1.0707	0.7256	1.4069	0.9149	
0.6396	1.018	0.6647	1.1592	0.7456	0.5951	0.5816	0.8877	
1.1935	0.8815	1.0387	1.1928	0.8628	1.2305	0.867	0.3257	
0.6146	0.5268	0.4667	0.7259	0.9218	0.9118	0.7468	0.4649	
1.1969	0.5662	1.9719	0.2269	0.4869	0.5821	0.8976	0.6829	
0.9184	0.9976							
AGBL1	0.0799	0.088	0	0	0	0	0.1701	0
	0.046	0	0.0039	0	0.0338	0	0	
0.0107	0	0.0332	0	0.0229	0	0.0021	0	0
	0	0.0091	0	0.0261	0.0258	0.0643	0	0
	0.0119	0	6.00E-04	0	0	0	0.0217	0
	0.0115	0.0078	0	0	0	0	0	
0.0518	0	0	0	0	0	0.0115	0	0
	0	0	0.0278	0	0.0342	0.0308	0	0
	0.0361	0	0	0.0329	0.0136	0	0	0
	0	0	0	0	0.0186	0	0.063	0
	0	0	0.015	0	0	0	0.0291	0
	0.0674							
AGBL2	0.4382	0.5599	0	0.1646	0.25	0.3748	0.4939	
0.375	0.6224	0.1472	0.5542	0.0998	0.2742	0.3662	0.0742	
0.2261	0.9033	0.2285	0.5534	0	0.1381	0.0273	0.1142	
0.1054	0.0842	0.2887	0.3092	0.8548	0.2212	0.1441	0.8889	
0.3053	0.2045	0.248	0.2084	0.198	0.7971	0	0.0396	
0.2005	0.2638	0.4318	0.0968	0.593	0.4573	0.627	0.4451	
0.5233	0.355	1.2095	0.2311	0.0998	0.3301	0.3884	0.927	0
	0.0669	0.1242	0.4323	0.0967	0.3565	0.1803	0.1289	
0.4535	0.2058	0.7601	0.9456	0.3188	0.2926	0.3558	0.2642	
0.1897	0.1285	0.1811	0.2935	0.5638	0.3735	0	0.1779	
0.45	0.2495	0.4626	0.2182	0.3235	0.2101	0.3014	0.2047	
0.1953	0.1411							
AGBL3	0.2055	0.2547	0.3775	0.1979	0.0421	0.6618	0.1886	
0.3871	0.0773	0.0399	0.0884	0.0569	0.149	0.3295	0.0769	
0.2019	0.2127	0.2016	0.653	0.1782	0.3546	0.036	0.0773	

0.3385	0.1924	0.0732	0.1174	0.3353	0.1005	0.0681	0.3002	
0.3007	0.3809	0.1857	0.398	0.1187	0.1857	0.2588	0.0893	
0.0951	0.1295	0.0842	0.0685	0.2538	0.1241	0.2073	0.2872	
0.1332	0.4288	0.3871	0.1228	0.2113	0.3935	0.1	0.1441	
0.2571	0.0415	0.2571	0.2513	0.3058	0.103	0.1584	0.2664	
0.2155	0.5096	0.0885	0.4987	0.057	9.00E-04	0.1939	0.1835	
0.3089	0.1904	0.101	0.0954	0.6558	0.3541	0.0666	0.119	
0.3324	0.2033	0.1219	0.1928	0.3273	0.3417	0.4743	0.0954	
0.1236	0.0572							
AGBL4	0.0436	0.0924	0.035	0	0.0222	0	0.035	
0.0886	0.0765	0.0318	0.0169	0.0597	0.0247	0.0572	0.019	
0.1406	0	0.0537	0.0663	0.035	0	0.0146	0	
0.0325	0.0437	0.0175	0.0646	0.064	0.0349	0.0311	0.6759	
0.0431	0.0443	0.0338	0.0242	0.0178	0	0.0657	0.1085	
0.0585	0.0485	0.0359	0.0306	0.0757	0.023	0.0547	0	
0.099	0.048	0.0534	0.0186	0.2666	0.0819	0.0732	0.1384	
0.2221	0.0026	0.3214	0	0.0548	0.0048	0.0072	0.392	0
	0.0628	0	0.1066	0.0454	0.0745	0.0266	0.1158	
0.0184	0	0.0707	0.0216	0.2467	0.0258	0	0.0452	
0.0456	0.1018	0.0422	0.0434	0.0174	0.0307	0.023	0.0285	0
	0.0785							
AGBL5	4.2914	4.1076	4.455	3.5792	3.7562	4.025	2.016	
4.5879	4.3508	4.4898	4.9649	3.8407	3.5644	3.4533	3.6924	
3.5958	5.1364	4.1413	3.9497	4.111	3.4406	4.2315	4.4733	
3.9191	4.0414	3.7346	5.0201	4.5772	3.4725	4.8828	4.9616	
3.7642	3.7535	4.1758	4.1929	4.2058	4.1434	3.862	3.5273	
4.2862	4.1175	4.3967	4.4884	3.8445	4.2772	4.4555	4.1344	
4.4141	4.7611	4.5632	4.5912	3.0802	3.8017	3.7961	5.0454	
3.8659	3.4573	4.1183	2.5921	4.1206	4.0386	4.5013	3.8548	
3.6616	4.7539	3.6578	4.7452	3.782	4.27	3.8147	3.8895	
4.3549	5.1704	3.8581	4.3832	4.1513	4.5443	4.7965	3.7852	
4.5239	4.4115	3.9534	4.1697	4.0272	3.8216	3.274	3.7316	
3.4928	3.8677							
AGER	0.9811	1.5452	1.1543	0.9798	0.5306	1.5899	0.4805	
1.1345	1.3254	1.5389	1.321	0.5574	0.4197	0.9798	1.0133	
1.0486	1.6568	0.9097	1.3204	0.9866	1.2009	1.4816	0.5322	
0.9159	1.4387	0.5823	0.9558	1.5864	0.7287	0.6652	0.9704	
0.484	0.4977	0.5995	0.9077	0.8824	0.9097	0.9572	0.8312	
0.5723	1.288	0.8299	0.4585	1.3632	0.9032	1.2668	0.5833	
0.537	1.0564	1.3777	0.9839	0.5056	0.6321	1.0667	1.4916	
1.0236	1.0847	0.8485	1.0632	1.2796	1.3465	1.3996	0.6625	
1.4513	0.9852	0.6498	1.348	0.2773	0.946	1.5454	0.658	
1.3274	0.547	0.942	1.6493	0.5352	1.5459	0.7059	0.4002	
1.2766	1.2444	1.4945	0.2017	0.6222	1.0247	0.9927	0.6513	
0.8162	1.091							
AGFG1	4.2306	3.5924	3.8838	3.7455	3.2374	4.5836	3.5445	
4.0895	4.0038	3.4811	4.0074	3.8028	3.6701	4.102	4.681	
3.6262	3.5241	4.1006	3.9916	3.667	4.7496	3.8907	3.8989	
4.0143	3.6791	2.8051	3.2615	4.2954	3.8819	4.2634	3.3665	
4.4412	3.9358	3.4333	3.7831	3.9039	3.6984	3.8859	3.5472	

4.2383	3.4003	3.402	4.4968	3.7688	3.7757	3.5855	4.5333
3.8062	4.0789	3.8036	4.0815	4.3941	4.0387	4.0324	4.2814
3.4514	3.7264	4.7342	4.263	4.8957	3.7528	3.5091	3.3264
3.5855	4.4342	3.4583	3.6466	3.564	4.193	3.7234	3.7143
3.9736	3.984	4.6977	3.8059	3.4914	3.9498	3.8923	3.7455
3.2135	3.9147	4.0804	4.2814	3.9359	3.2631	3.8614	3.6515
3.8198	3.6705						
AGFG2	1.6875	2.5763	1.9006	1.7649	2.0973	3.313	1.7592
2.8758	2.2273	1.8428	1.7814	1.2125	1.6433	2.0226	1.6986
2.154	1.3897	1.9931	2.5278	2.371	2.2501	2.5158	2.8954
3.0238	2.8533	3.0754	1.5921	1.8499	2.698	1.5557	1.4771
2.4349	2.2536	2.2316	3.0804	1.8994	1.4396	2.4791	2.1234
1.63	2.059	1.4047	1.911	2.2542	1.3593	2.1498	1.6447
2.2908	2.4762	1.1679	2.7968	1.8563	1.635	1.5651	2.0414
2.2019	1.164	1.5438	2.4605	1.6383	2.4879	1.9503	2.8254
2.3688	1.5714	1.3154	2.5363	0.6992	1.283	2.2158	2.3884
2.6876	2.5239	1.4159	1.9335	2.366	2.433	1.9223	1.9211
2.82	2.0562	1.6713	1.4129	2.3253	2.8892	1.8131	1.5124
2.1005	1.9316						
AGGF1	3.2197	3.4118	3.7314	4.1206	3.8903	3.4665	3.8573
3.4804	3.2981	3.4186	3.924	4.1137	3.9803	3.9496	3.6054
3.5763	3.8622	3.3757	4.078	3.9569	3.5681	3.8569	3.8428
3.9478	3.9427	3.3326	2.4225	3.8616	3.7918	3.6958	3.3963
4.0351	3.6461	3.3039	3.9175	3.5983	3.8092	3.8765	3.2289
3.8122	3.7502	4.107	3.7942	3.8385	3.4245	4.039	3.805
3.7753	3.6243	2.4529	3.6536	4.4901	3.6277	3.7622	3.073
3.4928	3.4579	3.4452	3.7788	3.3007	4.1422	4.1324	3.4542
2.9828	3.5752	3.2274	4.2986	3.8003	3.7107	3.6345	3.521
3.9454	3.6708	3.7138	3.487	3.5096	3.3772	3.793	4.0839
4.2776	3.6521	3.6425	3.8206	3.5129	4.1012	3.5051	3.6117
3.2879	3.5602						
AGK	4.2317	4.3703	3.7414	4.2431	3.7766	5.0483	4.2817
5.3159	4.5656	4.2584	4.1873	4.431	3.3007	4.4955	3.9808
5.5512	3.621	4.9551	4.7896	5.0839	3.5177	4.4717	4.2281
4.4581	4.3777	4.0435	3.5448	2.9575	4.9844	3.9748	3.4257
3.8795	3.9498	4.0871	4.8655	4.2909	4.0667	4.031	4.5523
4.8803	4.2431	3.0419	4.2229	4.5692	4.2049	4.3517	4.1353
4.2895	4.5115	3.4233	3.9069	4.3882	4.8314	3.8662	3.971
3.909	2.8383	5.3777	4.6057	4.6882	4.1859	4.8252	4.8025
4.3421	3.7081	2.7403	3.4741	3.3159	3.0201	4.5229	4.1384
4.4126	4.5992	5.2495	3.8309	3.9888	3.6941	4.2535	4.047
4.3962	4.1479	3.7701	4.0335	4.5082	4.1222	4.43	3.4686
3.5544	3.3636						
AGL	2.6249	2.5216	2.6355	2.5065	2.7738	3.0525	2.943
1.9218	2.9557	2.7431	2.6167	2.9997	2.4059	3.4199	2.4059
3.0536	2.4196	2.5474	3.0433	2.749	2.82	2.1792	1.764
2.3019	1.5009	3.1101	1.7479	3.2117	1.4295	2.3396	3.678
3.0525	2.7402	2.9385	3.1989	2.9906	1.8822	2.1861	1.893
2.3351	2.3558	3.1412	1.8344	2.647	2.0816	2.313	2.632
2.4977	2.253	3.5015	2.485	2.4265	2.8227	1.9674	3.2955

2.7758	2.6288	3.0539	2.4328	2.9091	1.8914	2.6644	1.7409
3.3685	2.685	2.4328	3.118	2.911	2.7177	2.2492	2.0314
2.5055	2.5871	2.2236	2.6103	2.9907	2.7914	2.4147	3.0857
1.9866	3.2071	2.8298	2.5229	1.4631	1.686	2.5361	2.814
2.4623	2.7962						
AGMAT	0.1267	0.4861	1.4099	0.8876	0.0674	0.1605	0.0995
0.3272	0.2579	0.4293	0.4905	0.8787	0.7487	0.7251	0.1595
0.1277	0.9281	0.4813	0.2978	0.1309	0.5418	0.8369	0.5087
0.1856	0.2617	0.2477	0.1228	0.2042	1.0337	0.2822	0.0898
0.5042	0.2589	0.221	0.1612	0.1017	0.934	0.0446	0.8235
0.2531	0.2689	1.0383	0.264	0.2441	0.5469	1.1985	1.8769
0.2345	0.1486	2.2373	0.1232	1.5612	0.6763	1.5985	0.2338
0.169	0.689	0.2216	0.5852	0.5194	0.1328	0.7856	0.1891
0.0956	0.2912	0.2193	0.2533	0.5025	0.7885	0.1186	0.4088
0.2348	0	0.3933	0.2332	0.6312	1.6881	0.1064	0.1817
0.2205	0.1714	0.5271	0.9377	0.3084	0.4443	0.646	0.2083
0.8551	0.3976						
AGMO	0.1158	0	0	1.0137	3.8526	3.7178	0.2273
0.8717	0.3039	0	0.0192	0	0.0051	0.0905	0.014
1.6681	1.8293	0.0489	0	2.543	0.295	3.4629	3.1278
0.1782	4.1447	0.0193	0.4081	0	3.438	0	0
0.5735	1.2198	0.4145	4.3066	0.0794	0.1731	0.0078	0.1293
5.5888	3.5785	0.1072	0	1.9481	0.0105	0	0.0226
2.1038	4.9917	0.1455	1.0596	1.8685	0.6908	0.124	0.1214
0.2925	0.1786	0.8355	0.1106	0.8945	3.3771	0	1.7514
0.1375	0.0939	0.0618	0.2482	0	0.1429	4.8366	1.7031
0.1782	0.4285	0.3991	0.5701	0.1217	0	1.1401	1.5052
2.6916	1.1891	0.344	0.1687	5.9364	2.7854	0.5833	0.0945
0.1337	0.1006						
AGO1	2.5568	3.4861	3.5653	3.288	3.5392	3.2631	3.2067
2.7823	3.7713	3.3926	2.7883	3.5689	2.3612	3.6895	2.655
3.7426	3.1311	3.0921	3.5519	3.0517	3.0374	3.047	2.565
3.1728	3.0105	3.0536	3.0039	2.7443	3.3035	3.31	3.6672
2.6396	3.3104	3.2266	3.2654	3.6274	3.002	2.7374	2.5272
3.6264	2.5897	3.148	2.7227	2.9287	3.4949	3.0528	3.3344
4.0305	2.8884	2.5923	3.0894	3.3464	3.666	3.1649	3.3862
2.8561	2.5762	3.5199	3.3719	3.2776	3.0235	3.4366	3.1595
3.8572	3.1374	2.6444	3.0595	2.9725	3.8359	2.8008	2.5791
3.3521	3.1981	3.3505	3.8439	3.6622	3.4121	3.19	3.5839
3.4071	3.4728	3.3394	2.7511	2.8004	2.3607	3.076	2.8924
2.674	2.8221						
AGO2	3.0196	2.7586	4.3628	3.897	3.7133	3.5775	2.2311
3.1122	2.2809	3.7996	2.9708	3.1667	3.0356	3.0334	3.9539
2.2278	4.1143	2.518	3.1751	3.5161	3.6441	3.658	2.164
3.7823	3.3989	2.0238	3.7776	2.3051	3.0204	1.983	3.096
2.9436	3.5008	3.1846	2.8455	3.343	3.4183	3.2343	3.0839
4.1386	2.8639	2.6494	4.0083	3.2241	3.538	3.6267	4.1338
3.4109	2.5626	3.3558	2.9064	3.1513	2.8514	3.3877	3.3734
3.7778	3.6447	4.4831	2.8813	4.1155	3.0788	2.3475	3.3058
3.2881	4.7181	1.9496	3.2152	2.8628	3.0048	3.2528	3.4101

2.8432	2.8771	3.0526	2.3692	3.2245	3.5862	2.8281	3.6173
3.8765	3.6052	3.5937	4.586	3.2525	2.3398	2.3738	2.6228
2.5367	2.7369						
AGO3	2.0003	2.4626	2.4789	2.3953	2.7205	2.3516	2.7559
2.0543	2.7094	2.4076	1.7662	2.6697	2.1317	2.0084	1.7596
1.7029	2.5906	1.749	2.2762	1.947	2.2888	2.514	2.0598
2.3057	2.5305	2.2157	3.1122	1.7198	2.2506	2.3781	2.7035
1.6911	2.6568	2.3597	1.9741	2.364	2.1539	2.3776	1.5898
3.0092	2.0945	3.1043	1.8276	1.954	2.7418	2.6448	2.1835
3.012	2.0138	2.8876	1.6554	2.8798	2.0384	2.9082	2.7161
2.4033	2.6153	2.3262	2.4022	2.4467	1.9128	1.8373	2.0784
3.1751	2.1185	2.1167	2.1877	2.167	2.2617	1.5407	1.5748
1.9404	1.8789	2.2974	2.1217	2.1463	2.5847	1.6889	2.4645
2.0864	2.8333	2.239	2.532	1.3169	1.4385	1.7048	1.3981
0.9491	1.2336						
AGO4	2.2199	2.5311	2.5656	1.6997	1.4715	1.7803	2.4796
1.8663	3.0568	3.2626	2.2649	2.1913	1.8524	2.5482	1.9401
2.069	2.5605	2.4503	2.4097	2.0486	2.357	1.744	1.1962
2.1193	1.7025	1.8121	2.1861	2.4038	1.5734	2.9201	2.8877
1.574	2.1698	2.5945	2.0034	3.0582	1.9727	1.8324	1.7412
2.1797	1.9365	1.5338	1.6511	2.3271	2.0074	1.9286	2.1384
2.0937	2.1979	2.0928	2.5515	1.5035	2.5494	2.5579	2.0424
2.2163	2.3272	2.1961	2.4495	2.195	1.7348	2.5707	1.8661
2.6634	2.1938	1.8001	2.1127	2.2677	2.8575	1.3792	1.7694
2.3869	2.297	1.5387	1.8213	2.1042	2.9121	1.7194	2.2117
2.3812	2.6489	1.7321	2.1702	1.1767	1.3676	2.1502	2.8026
2.3943	2.9676						
AGPAT1	6.1613	5.9103	6.0882	6.0751	5.9146	6.3856	4.9282
5.881	6.2597	6.4387	6.0029	6.8451	4.8387	6.6053	6.5699
6.9436	5.798	6.3365	6.0192	6.5569	5.9937	6.5091	7.0087
5.7905	6.1438	6.4304	6.0989	7.1774	6.85	5.8161	6.1476
6.0814	5.5629	6.0922	7.1268	6.1533	7.3016	6.5259	6.6176
5.3274	6.6502	6.0922	6.2006	6.3806	5.7696	6.3922	5.0166
5.6773	6.6155	5.708	6.1613	6.1414	5.5977	6.2474	6.053
5.9731	5.087	7.2451	5.3894	6.6054	6.4388	6.3128	6.2436
6.1538	6.3453	4.9605	7.3719	6.2496	5.9473	6.3165	6.3874
6.6808	5.7468	6.7199	6.1371	5.9019	6.3756	7.0808	6.1063
6.0519	5.6171	7.2166	6.2987	6.6735	6.5633	5.9242	6.5881
6.5915	6.3444						
AGPAT2	4.8289	4.7758	5.8009	2.9361	4.5169	4.012	3.0694
4.4208	3.475	3.8508	5.3628	3.5987	5.0587	2.9891	4.3981
3.9163	4.3286	3.242	3.9094	4.897	5.2085	4.554	4.0499
3.7931	4.2674	3.4871	3.4977	5.1995	4.2359	4.0669	3.759
4.81	4.253	4.4992	3.6998	3.2129	4.3787	5.6295	5.7066
2.7376	5.1593	4.3376	4.3926	4.3208	3.4589	3.3848	4.0017
3.599	5.1506	3.4388	4.069	3.0635	3.599	4.4555	4.0582
5.1959	5.3932	6.1178	4.3519	5.0098	4.5546	3.8756	3.6482
4.342	4.8282	3.817	3.8407	5.3114	4.8675	5.718	4.3014
4.3399	4.7103	3.3234	2.9781	5.8427	4.6622	3.3687	4.6078
4.6616	4.6329	3.5792	4.5412	5.0298	4.0164	4.4978	5.2299

5.6317	5.6729						
AGPAT3	4.0434	3.8954	4.3242	4.0905	3.9279	4.1601	4.5253
3.7048	4.5884	3.3772	3.6365	3.6783	3.9691	3.9006	3.9082
4.1717	2.8021	3.6068	3.8756	4.0049	3.3527	4.4642	4.0066
4.2221	4.2173	2.9423	3.0816	4.4733	3.9359	3.7442	3.7623
3.909	3.1522	4.3013	4.4291	3.3347	4.3417	4.0813	4.1423
3.4483	4.6181	4.4055	4.0602	3.5381	3.9805	2.9222	3.6384
3.9082	3.6717	3.6693	4.2255	3.7126	4.1372	3.5726	3.9263
4.4411	4.2056	4.3893	3.8266	3.7269	3.6356	3.5967	3.7026
3.9651	4.2039	4.4903	3.343	4.8589	4.7711	4.0492	3.7518
3.1331	3.9858	3.1939	4.2324	4.512	4.0707	4.8136	4.3833
3.9997	4.0366	4.6184	3.5182	4.7465	2.9898	4.012	4.9799
4.5558	4.6791						
AGPAT4	1.0772	0.3308	1.0023	1.21	0.8812	1.7613	0.99
0.8949	1.325	0.7748	1.9004	2.431	1.4133	0.6948	1.9652
0.2354	3.3519	0.208	1.4807	2.2397	1.7705	1.5141	1.4592
1.2806	1.0287	1.7402	1.8685	0.0294	0.626	1.3053	2.1012
0.8617	1.0174	0.7518	1.5522	0.9431	0.2383	0.4384	1.749
0.945	0.8688	2.4214	2.5351	0.6345	1.5405	1.6303	1.0445
1.1878	0.5835	1.8808	1.4967	1.1318	0.238	1.4904	2.2846
1.5421	2.989	1.6977	1.5445	1.4126	1.4364	1.0105	1.6682
0.9674	1.6533	1.697	0.1728	0.73	0.9377	1.3867	0.4745
1.329	2.5669	1.1842	0.5381	0.8634	0.4011	1.2336	0.3489
0.8329	1.7465	0.806	0.4777	1.2461	1.5591	0.1682	2.0433
1.4974	1.756						
AGPAT5	4.1565	3.4527	3.3679	4.2772	3.5899	3.5852	5.3255
4.2421	3.4313	3.6015	2.2244	3.2059	3.1165	3.9168	3.4597
2.9402	5.0368	3.8387	4.2408	3.0888	3.9145	4.4062	4.2182
4.3949	4.9821	2.9879	3.2132	3.3559	4.6521	3.1489	4.2514
4.1768	4.0046	3.5147	4.036	3.728	4.2439	2.9381	3.143
4.4934	2.8677	2.1984	4.4363	4.0194	4.5568	4.6703	4.5865
3.5171	4.1486	5.1463	4.3431	3.9691	4.5253	3.5746	3.5357
3.9649	2.8285	5.2225	4.0816	5.5882	3.7425	3.9927	4.8139
3.1322	5.339	2.4539	4.0645	3.2947	2.7313	4.1934	4.0062
3.6106	4.4023	3.4045	4.4452	4.4315	3.6674	3.305	3.5509
3.8517	3.4304	4.1806	3.9149	3.6184	3.5376	4.3202	3.3483
3.9926	3.6299						
AGPS	4.371	4.4781	4.4128	5.0392	4.8327	4.1629	3.8867
4.2222	4.3293	4.5006	4.0679	4.6019	4.6782	4.5456	4.663
4.3758	4.2956	4.3068	4.5309	4.192	4.589	3.5602	4.2326
4.3059	3.9494	3.0763	3.334	5.0418	4.152	4.8327	3.7516
3.9871	4.4206	3.3727	3.3747	4.7328	4.3388	3.9652	4.3995
4.3276	3.7825	3.9917	3.6763	4.1621	4.2791	3.8259	4.601
4.6097	3.7645	4.1317	3.8093	3.9526	4.6127	4.4347	4.1797
4.2442	4.3625	4.1163	4.9911	4.2628	4.0508	4.8649	3.7779
3.7528	4.191	3.8938	3.8877	3.1705	4.7069	4.2726	4.0296
4.2759	5.0197	4.8689	4.0768	4.0933	4.294	3.8938	4.7684
4.0504	4.4085	4.1113	3.4915	3.395	3.8428	4.3218	3.9747
4.2813	4.2289						
AGR2	0.2422	1.4866	0.3165	0.5585	0.242	0.2904	0.1344

0.2874	0	0	0.1745	0.2577	0.2061	0.4399	0.2552	0
	0.1252	0.63	0.2049	0.1119	0.2413	0.0862	1.6732	
0.1205	0.6314	0.0728	0.1186	0.1157	0.1678	0.255	0.1245	0
	0.2606	0.3559	0.2362	0.0689	0.1746	0.3533	0.3352	
0.5443	0	0.1843	0.144	0.3338	0	0.7302	0.1925	0
	0.1506	0.6733	0.3652	0.1762	0	0.523	0.2995	
0.1414	1.0095	0.3149	0.9687	0.2067	0.2419	0.3629	0.1774	
0.6875	0.1738	0.0012	0.2511	0.1545	0.7705	0.1474	0.1125	
0.4804	0.0175	0	0.4449	0.0894	0.3454	0	0.2553	
0.6512	0.3925	0.9869	0.0793	0.0107	0	0.8411	1.1129	
0.1068	0							
AGR3	0	0.4194	0	0	0	0.2066	0	
0.1714	0	0.1375	0.2232	0.1067	0	0.1034	0.1141	0
	0.1593	0	0	0	0	0	0.0876	0
	0.2711	0	0	0	0	0	0	0
	0.3658	0.0885	0	0	0.4557	0.1139	0.3081	
0.1799	0	0.1429	0.1043	0	0	0.4299	0.0973	
0.1022	0	0.4083	0.1837	0	0.2088	0.2961	0.0519	
0.1347	0.1925	0.3498	0.4136	0.1906	0.0841	0.1235	0	
0.4044	0.1906	0	0.1039	0.0876	0.2772	0.0871	0.1449	
0.2273	0	0	0.2574	0.0652	0.2199	0	0.1974	
0.0988	0.1147	0.1554	0.2059	0	0	0.2494	0.1859	0
	0							
AGRN	3.8938	3.3866	5.6362	3.1513	5.3269	3.6527	5.2838	
4.7704	4.1033	2.7939	5.2794	3.312	4.596	1.9021	4.1855	
4.3058	4.5855	3.3238	3.0046	4.3429	4.19	3.3211	4.7256	
3.3231	3.665	4.9109	3.054	3.8997	3.9757	3.4691	4.3127	
4.1649	2.788	6.0034	4.0109	2.0342	2.9741	5.6543	5.3534	
3.2937	5.879	5.5911	5.3145	4.094	5.6027	4.3295	2.3452	
3.7405	5.8557	2.7478	4.645	2.5968	3.5621	4.9976	3.5015	
4.0428	5.742	4.7404	5.2715	4.841	4.2503	1.6061	5.0323	
3.3603	3.8283	7.3411	5.0974	4.813	3.1234	4.2086	3.9392	
3.5174	4.5947	5.5116	3.0195	2.9147	5.6818	4.277	4.5442	
4.6894	3.9859	5.5051	3.7747	3.2297	4.9949	4.5034	2.9817	
3.7964	3.8678							
AGRP	0.1791	0	0	0	0	0	0	0
	0.0839	0	0.0616	0	0	0	0	0
	0	0	0	0	0	0	0	0
	0.0577	0	0	0	0	0	0	
0.1177	0	0.2101	0	0	0	0.2034	0	0
	0	0.168	0	0.1925	0	0	0	
0.1158	0.0749	0	0	0	0	0	0.1088	0
	0.1128	0	0	0	0	0	0.0854	
0.1336	0	0	0	0.1024	0.0649	0	0	0
	0.105	0	0	0.0876	0.068	0	0	0
	0	0	0	0.1862	0	0	0	0
	0							
AGT	0.9706	0.2411	0	0.2722	0	0.1427	1.2182	
0.0997	0.097	0.4601	0.134	0.2448	0.145	0	0.1625	
0.1694	0	1.2882	0.1372	0.1055	0	1.6824	0.3274	

0.339	0.2281	0.1991	0.6507	0	1.255	0.803	0.1417	
0.384	0	0.1698	3.9836	0.0948	1.484	3.0599	2.7493	0
	1.1158	0.6069	0	0	0.1892	0.3278	0.4695	
1.7195	0.0709	0.2215	0.755	2.2477	1.0663	1.2203	0	
1.6165	0	0.0408	2.3453	0.0412	0.4097	0	0.0467	
0.003	1.9008	0.3118	1.0871	1.6477	0.8206	0	0	
0.0744	0	4.031	0.7717	0.9609	0.8019	2.4282	0.0473	
0.0988	0.9273	0	2.913	0.0053	0.1353	0.6255	0.123	0
	0.1032							
AGTPBP1	3.1088	2.0729	2.5083	2.3919	2.4011	2.5607	2.4503	
2.9617	2.0545	3.1666	3.3842	2.9189	2.3252	3.2244	2.6972	
2.6701	3.2781	2.7476	3.362	2.7598	2.2794	1.6367	1.9713	
2.4776	1.6491	1.7278	3.9343	2.6658	1.7844	2.6139	3.0117	
2.8264	2.6645	3.419	1.7539	3.2489	3.1438	2.8812	3.696	
2.2389	2.7619	1.8856	2.2669	3.3482	2.3863	2.1132	3.0414	
1.9203	2.4036	3.5338	3.2233	2.6435	3.202	2.9174	2.2956	
2.8087	3.9405	2.846	3.6916	3.7524	2.3673	2.0658	1.5692	
3.3273	3.1612	1.8397	2.3215	2.6743	2.6	3.1979	3.2264	
3.0311	2.4567	2.8883	2.4438	2.4885	2.9142	2.2982	2.2486	
2.0813	2.6862	2.6359	3.1509	2.7064	2.1197	3.0657	3.0501	
2.7979	3.0718							
AGTR1	0.0866	0	0.0453	0.3107	0.8845	0	0.029	
0.0512	0.0871	0	0.2558	0.1512	0	0.6125	0.0266	0
	1.6172	0	0	0.0387	0.0426	0.5122	0.0066	
0.0122	0.0092	3.3875	0.1538	0	0.2134	0.1108	0.3136	0
	0	0	0.5364	0	0.1425	0.0331	0.0904	
0.2248	0	0.1747	0.1054	0	0.155	1.8827	0.076	
0.0298	0.0203	2.1856	0.667	0.06	0	0.0579	0.0704	
0.49	0.6903	0.9284	0.367	0.3056	0.158	0	0	0
	0	0.1153	0	0.13	0.0456	0.0623	0.5461	
0.0246	0.0291	8.00E-04	0.1421	1.213	0.0582	0	0.0365	
0.0496	0.0555	0	0	0.1219	0.3989	0.28	0.1092	0
	0.1086							
AGTR2	0	0	0	0	0	0	0	0
	0	0	0.0314	0	0	0	0	0
	0	0	0	0.1008	0.0337	0	0	0
	0	0	0	0	0	0	0	0
	0	0	0	0.0072	0.0293	0.0236	0	0
	0	0.001	0	0	0	0.0252	0	0
	0	0	0	0	0	0	0	0
	0	0	0	0	0	0	0	0
	0	0	0	0	0	0	0	0
	0	0	0	0	0	0	0	0
	0	0	0	0	0	0	0	0
	0							
AGTRAP	4.5061	4.7861	4.8193	3.6678	5.0067	3.2474	5.2617	
3.89	4.6275	4.0507	4.8235	4.6895	5.8562	3.5036	4.5413	
4.7399	4.5178	3.7305	3.1856	4.7492	5.149	4.4299	5.156	
4.5331	3.7602	4.9881	3.2545	5.5553	4.2262	4.8805	5.7596	
5.8752	5.6216	5.6888	5.0821	3.317	4.9994	5.8204	3.9961	

3.1349	5.1264	4.8078	4.0018	4.4338	3.7281	3.516	3.2373	
4.6997	5.7116	3.7125	4.652	3.6539	3.8868	4.0498	5.3204	
4.1117	5.2279	4.0718	4.8172	3.771	4.1047	3.7613	3.1354	
4.2393	4.9728	5.7188	5.0067	5.1995	4.8997	4.0866	5.1831	
4.3279	5.186	4.3597	3.1984	5.5728	4.6515	3.8859	5.3832	
4.7685	5.0549	4.1358	4.9728	4.1196	4.8602	4.8481	5.1021	
4.6395	5.1822							
AGXT	0.2302	0	0	0	0	0	0	0
	0	0.6597	0	0	0	0	0	0
	0	0	0	0	0	0	0	0
	0	0	0	0	0	0	0	0
	0	0.0684	0	0.0545	0.0356	0	0	0
	0	0.1333	0	0.0718	0	0	0	0
	0	0	0	0	0	0	0	0
	0	0	0.0622	0.0472	0	0	0	0
	0	0	0	0	0	0	0.0393	0
	0.1619	0	0	0.0848	0	0	0.0444	0
	0.0413	0	0.5193	0	0	0	0	0
	0							
AGXT2	0	0.1679	0	0	0	0	0	0
	0	0	0.0528	0.0706	0	0	0	0
	0	0	0	0.3484	0	0	0.0165	0
	0	0.1725	0	0	0	0.1688	0	
0.0074	0	0	0	0	0	0	0.0325	0
	0	0	0	0	0	0.0912	0.0963	
0.0454	0.1701	0.3415	0	0.0482	0	0	0	
0.1409	1.4275	0	0	0	0	0	0	0
	0	0	0	0	0	0	0.0319	0
	0	0.0046	0	0	0	0	0.0085	0
	0	0	0	0	0	0.0395	0	0
	0							
AHCTF1	3.536	3.7129	3.7301	4.6471	4.5486	3.8037	3.7625	
2.7243	3.477	3.239	2.6473	3.784	3.2885	3.8397	3.8508	
3.9533	3.5086	4.2608	3.5142	3.1832	3.874	4.0368	3.9668	
3.9279	4.0802	3.7408	3.3149	3.2805	3.6962	4.167	4.0172	
3.571	3.4452	2.866	3.4159	3.9455	4.0483	3.2141	3.8701	
4.4055	3.5838	3	4.0402	3.7796	3.9914	3.9279	3.8779	
4.1972	4.1092	3.9714	3.6836	4.5296	4.1139	3.2871	4.2984	
4.1024	3.2541	4.0645	4.142	4.2378	3.719	3.8806	3.8886	
3.6241	4.1254	2.5122	3.8296	2.9952	3.0943	3.8453	3.8164	
3.7847	3.6595	3.9768	3.9065	2.9257	3.7999	3.8524	3.7531	
3.0276	3.379	3.4859	2.6094	3.7073	3.6011	4.2187	3.0011	
3.2325	2.7413							
AHCY	7.9603	7.3779	7.854	6.1754	6.9768	6.5386	6.5977	
7.5495	7.0119	7.5193	7.1008	6.6608	6.5977	7.0483	7.0119	
6.5794	7.2866	6.0536	6.7467	7.1104	6.8053	6.7134	6.7904	
6.9431	6.1364	6.4628	7.3909	6.6521	7.118	7.3809	6.3876	
7.7278	6.7904	6.6269	5.8314	7.0598	7.2637	6.5515	7.3723	
7.7184	7.2723	5.7315	5.8137	7.1478	7.5428	5.9805	7.786	
7.3348	6.9961	7.0649	6.6161	6.303	7.2952	7.4888	7.159	

7.3477	5.8338	7.1712	6.9532	7.308	6.9038	7.4758	7.3414
6.9507	7.6657	5.5338	7.2618	6.6969	6.4125	6.2683	7.7225
6.8833	7.8936	6.3709	6.8908	7.3656	7.153	6.6992	6.8749
7.3688	7.3656	7.1418	6.3947	6.7521	6.1403	7.0039	6.0817
7.0554	6.7349						
AHCYL1	7.8593	7.128	5.7301	5.9804	5.4534	5.3352	5.6928
4.3976	5.7782	7.0929	7.7045	6.0843	5.4646	6.8549	5.0637
5.3825	5.4439	5.3301	6.004	5.9746	6.5493	5.5465	4.9333
4.9004	5.2699	5.4043	5.6032	5.4309	4.7904	6.0089	5.0827
6.3089	6.0231	6.4431	5.8591	6.5042	6.1521	5.5022	5.931
6.0537	5.9574	6.061	5.9163	5.2796	5.0274	6.5139	5.5247
5.8462	5.3366	5.4889	5.6582	5.3599	5.7223	5.97	5.796
6.2296	6.2684	6.2082	5.8093	6.2876	5.0669	6.8753	4.6564
6.2659	5.4607	5.5201	6.1952	5.0783	4.7369	5.2458	5.1256
6.2251	6.2397	5.5501	5.3002	5.2339	6.6935	4.7095	5.9085
5.0522	5.9751	4.9628	5.1204	5.1436	5.3009	5.6776	6.2798
5.5087	6.1205						
AHCYL2	3.3939	3.0674	3.0264	2.8565	2.4114	7.3899	3.6261
3.9891	3.1808	3.6449	3.9372	3.6456	2.7031	3.5416	3.4003
5.2602	2.358	5.0646	3.9802	3.0541	2.4499	4.0222	3.8727
4.1085	2.903	3.223	4.1995	2.7716	4.1819	3.5775	3.432
3.0322	3.5906	3.1905	4.0001	3.7206	2.6035	3.539	3.8346
3.2596	3.5702	2.9079	2.492	3.8385	2.823	3.2252	3.2556
2.4889	3.2267	2.5442	3.2606	3.4273	3.0888	2.6281	3.6428
3.2247	1.5613	2.4368	5.1442	2.6188	3.0661	3.7473	3.7944
4.1173	3.0724	3.2876	3.6975	3.8317	2.9518	2.6176	4.6274
2.4978	3.6621	3.4817	3.3329	4.277	2.9375	4.394	3.466
3.5922	2.7685	2.7534	4.7298	3.0608	2.2756	2.4485	3.9117
3.0073	3.443						
AHDC1	1.5561	2.037	2.0601	1.8482	2.709	1.9688	1.2364
1.679	1.8761	1.4184	2.5258	1.4152	2.2365	1.5819	1.6226
1.916	2.0169	2.1412	2.1301	1.8707	1.7813	2.0771	2.6816
1.6986	1.923	2.1273	1.7291	1.9534	2.0347	1.7371	1.4967
2.0925	2.4495	2.8738	1.7453	1.1029	1.2986	2.4318	1.9027
1.585	2.4106	2.3278	1.8561	1.6607	2.6109	2.2932	1.2986
1.6502	2.0874	1.9111	1.2777	1.619	2.2768	2.1916	1.5128
1.6686	1.5946	2.0615	2.3007	2.044	2.0722	2.1397	1.5846
2.1036	1.9683	3.419	2.2427	2.3424	2.0972	2.4253	2.1112
2.1163	1.8675	2.8172	1.6681	1.7342	1.9125	1.7374	2.121
2.4535	2.6406	2.5309	1.6795	2.0016	2.9162	2.2541	2.1787
2.6694	2.2429						
AHI1	2.4667	2.3676	1.9929	3.2428	1.9472	2.432	2.7838
2.8534	2.3056	1.9701	2.2998	2.2711	2.0462	2.6836	2.4667
2.3755	2.4483	3.4552	3.076	2.4244	2.4513	2.4377	1.9796
2.1425	2.918	2.8936	3.0501	1.8215	2.6294	2.356	5.3541
2.4607	2.1563	2.389	2.9721	2.7536	2.0507	1.7769	2.209
2.6236	2.3821	2.7704	2.3033	1.6657	2.4289	2.8916	2.7745
1.9939	2.8669	2.2	2.5569	2.7367	2.8609	2.5502	3.5247
2.6647	2.5138	1.5146	2.4322	1.9693	1.853	2.345	1.9991
2.9387	1.6642	1.9262	1.8293	2.0083	2.8599	2.4544	2.7865

3.5123	2.0129	1.8684	1.8373	2.1304	2.4066	1.985	2.0628
1.9369	2.8285	1.8237	3.4634	3.1752	2.582	2.7474	2.1567
2.1453	2.5126						
AHNAK	4.4755	1.1827	1.9444	3.8761	4.8207	6.4436	4.7196
3.0535	5.3635	4.2183	4.1796	3.8119	5.5683	5.8163	4.5283
5.3892	6.1404	4.1718	3.7635	4.5547	3.6972	4.3877	5.1384
4.208	5.8579	5.146	4.0719	2.7647	5.7841	1.5866	1.4752
2.9549	5.0713	5.3905	5.8744	3.5981	3.3307	1.7536	3.422
5.4059	3.0513	5.179	5.6788	5.6466	5.8035	5.1845	3.0052
3.2671	4.9695	5.0139	5.1037	4.2195	4.2897	5.4883	2.1791
3.3208	7.1954	2.7405	4.837	3.2235	4.2004	3.4074	2.96
6.4159	3.2404	5.662	2.8804	5.8782	3.4548	4.6495	6.1508
4.8495	3.8443	4.0416	5.3106	4.4731	4.5357	4.7399	3.3896
4.7657	1.7435	6.1272	5.8702	4.7137	4.2779	3.7221	6.5149
5.2303	5.5289						
AHNAK2	3.2878	0.2947	0.1862	0.4386	1.3934	0.4091	0.2374
0.8003	0.3836	3.3734	2.2407	2.208	3.668	2.6838	1.8732
3.9876	3.5321	4.306	0.5672	1.4031	2.921	0.2523	0.9526
1.5439	1.1033	2.6685	1.0429	1.5098	2.0377	0.6549	1.3226
2.9926	0.5474	3.3713	0.1788	3.0277	1.686	0.6865	1.7431
2.1759	0.4643	4.4042	3.7923	1.903	4.9699	1.2088	1.2462
0.7021	0.3865	2.3283	0.4041	0.051	2.0373	0.7667	1.2966
0.8047	5.81	1.2996	1.3409	0.6685	1.0989	0.4255	0.7343
0.4289	2.0667	5.2846	3.8721	4.3142	0.7652	1.3913	2.4116
0.7783	0.463	1.5806	2.8189	0.2518	1.2037	2.0868	0.8013
0.5394	1.3116	1.9738	2.8138	0.7257	2.6375	1.6514	5.629
0.8156	0.9708						
AHR	3.3411	1.9762	3.5296	5.1582	4.4637	4.9383	6.1633
4.2865	3.4369	3.1737	3.1135	4.0467	6.0586	4.4443	6.0107
4.4883	2.4702	3.6839	3.3158	4.3162	3.5304	5.5118	5.4048
4.0892	6.3762	5.4293	5.7153	2.6057	5.6037	3.4295	0.154
3.6308	3.946	4.5455	5.1537	3.2822	4.8681	4.1745	5.3475
5.3316	4.8295	5.3772	3.8347	6.3217	4.2371	6.4364	4.5882
3.89	6.4955	2.5843	5.1882	6.8291	4.679	4.2721	1.3968
3.5932	2.4056	2.2922	3.586	3.4875	5.3251	2.9456	3.8738
4.9881	1.6998	7.1311	3.5505	6.0498	3.0451	4.8834	5.4496
4.6168	4.2122	5.5661	4.7025	3.3221	3.19	5.4133	4.2553
4.4969	3.5258	5.6201	5.1904	5.1237	5.0993	4.616	3.6496
3.369	2.7161						
AHRR	4.0049	1.5486	2.896	2.4883	2.2293	2.2363	1.7663
1.7726	1.5206	2.1933	2.5964	1.8093	3.5561	3.0035	3.8221
1.4661	1.5503	2.9923	2.0185	3.1046	3.7644	3.3681	3.456
1.7709	3.3275	4.0334	3.6474	1.3691	3.459	0.234	0.3292
0.2824	1.7882	1.89	2.6952	1.9914	0.6439	1.6767	3.0684
2.3001	2.7646	2.9004	3.5702	3.5263	3.9899	3.987	1.4875
1.4509	5.1049	3.0847	3.2763	3.7064	2.0019	4.0923	2.1571
3.9175	0.3486	1.0478	3.0123	1.4842	2.6107	1.8619	1.9795
2.9586	2.7574	1.655	2.8795	4.7612	2.3937	3.7897	1.8951
3.8687	3.4131	2.5397	2.5313	2.3727	2.6583	1.7575	0.9391
1.9274	0.2246	4.258	2.8169	3.5477	4.4172	2.2913	4.226

3.8033	3.9451							
AHSA1	6.2975	6.5122	6.1415	7.2635	6.5474	6.3143	5.8551	
6.2281	5.3403	6.0535	5.5736	7.0446	5.9343	6.2673	6.4542	
6.2294	6.2987	6.6773	6.5887	6.6135	7.0331	6.8329	6.9399	
6.7583	5.685	5.8999	6.7305	6.2774	6.7227	6.5753	5.5536	
7.2665	6.1097	5.5814	5.5342	6.3776	6.5687	6.233	5.5484	
6.7087	6.6257	6.6257	5.9285	5.4012	7.156	6.8781	6.4621	
5.6736	5.9151	6.9822	5.3781	7.191	6.2895	5.7241	7.0549	
5.7365	6.042	7.2221	6.0089	6.9342	6.9888	7.1135	6.6142	
5.9038	7.1777	5.6708	6.2416	6.1181	6.1036	5.8243	6.3426	
5.6661	5.812	6.8401	6.947	6.2208	6.2176	6.3842	5.974	
6.1899	5.9578	6.4579	6.3925	6.1838	6.193	6.2665	5.8442	
6.1007	5.685							
AHSA2	3.4537	2.7263	2.0104	2.7102	2.3854	2.8747	2.6794	
2.8332	3.7478	3.3324	2.9724	2.4821	2.9281	2.7048	2.7386	
2.7467	3.6522	3.1299	2.4682	2.1392	3.2767	2.4773	3.1198	
2.2251	3.3931	2.2323	3.9238	2.5165	2.7129	2.7783	2.7839	
2.4377	2.2442	2.7271	3.6071	2.9427	3.1364	2.2097	1.6583	
2.921	3.0389	3.3517	2.7766	3.2805	2.97	2.5307	2.831	
3.0359	4.288	2.4386	2.6425	2.6503	2.5034	2.644	2.758	
2.8204	3.0271	1.8315	2.7498	2.2517	3.1191	2.3942	2.1624	
3.287	2.5463	3.3606	3.3563	2.4196	2.9766	2.5651	2.4778	
3.1966	2.9983	2.7776	3.586	2.6902	1.9221	2.5845	2.8565	
3.5793	3.4973	2.9854	3.4318	2.3769	2.0518	2.8582	2.2362	
2.5377	2.803							
AHSG	0	0	0.133	0	0.0311	0	0	0
	0	0.0605	0	0.0369	0	0	0	
0.0227	0.0766	0	0.1065	0	0	0	0.0318	0
	0	0	0	0	0	0	0.0788	0
	0	0	0	0.029	0.0161	0	0	0
	0	0.0211	0	0.0421	0	0.0659	0.06	
0.0972	0	0	0.0592	0	0	0	0.0883	
0.0632	0	0	0	0	0	0	0	0
	0	0	0.1277	0.0687	0	0	0	0
	0	0	0.0209	0.0451	0.0563	0	0	
0.1581	0	0	0	0	0	0	0.0524	0
	0.0648							
AHSP	0	0	0	0	0	0	0	0
	0.0813	0	0	0	0	0	0	0
	0	0	0	0	0	0	0	0
	0	0	0	0	0	0	0	0
	0	0	0	0	0	0	0	0
	0.0742	0	0	0	0	0	0	0
	0	0	0	0.1771	0	0.132	0	0
	0	0	0	0	0	0	0	0
	0	0	0	0	0	0	0	0
	0	0	0	0	0	0	0	0
	0	0	0	0	0	0	0	0
	0							
AICDA	0.0689	0.0433	0.0696	0	0.0311	0.0543	0.0481	

0.0109	0.0416	0.0314	0.0865	0.035	0.07	0.031	0.0184	
0.0554	0.0378	0	0.0458	0.1293	0.0371	0.0029	0.0048	
0.0374	0.0058	0.0535	0.0478	0.0684	0.0481	0.0472	0.0287	
0.0207	0	0.1484	0.0197	0.0231	0.0196	0	0	0
	0.0326	0	0	0.0405	0.0195	0.0148	0.0769	
0.0232	0.0154	0.0544	0.0148	0.0726	0.1142	0.0115	0	
0.1137	0.0372	0.0584	0.048	0	0.0222	0	0.0212	0
	0	0.0156	0.0485	0	0.0608	0.0878	0.0421	
0.0675	0.0804	0.0221	0.0631	0.0943	0.0706	0.0232	0.0107	0
	0.0187	0.033	0.0397	0.0752	0	0.0494	0.051	
0.0594	0.0667							
AIDA	5.2803	5.1656	5.1935	5.3823	5.4457	5.2899	6.1657	
5.2399	5.1901	4.8087	4.8412	5.4068	5.494	6.1331	5.6811	
5.8086	5.8911	6.0971	5.4963	4.9197	5.1409	5.3977	5.8706	
5.2603	5.9606	5.9093	6.1128	5.5938	5.4699	5.7088	5.6296	
5.4948	5.3853	5.802	5.7324	5.4144	5.562	4.5015	5.649	
5.6764	5.6196	5.3049	6.7521	5.4855	5.6694	6.282	5.3422	
6.2416	6.5183	5.8173	6.0479	6.0575	6.1053	5.1761	5.8102	
5.8868	5.9178	5.4759	6.2375	5.7307	5.3383	4.9205	5.5439	
5.5605	5.817	5.5355	4.952	5.923	4.8438	6.0865	5.6595	
6.235	5.318	5.7267	5.5558	5.1964	5.7366	5.5863	5.5837	
4.7067	5.3491	5.0262	5.5077	5.664	6.3785	6.1367	6.0223	
5.8528	5.6508							
AIF1	0	0.1543	0	0	0	0	0.0743	0
	0	0	0.0799	0	0	0.1946	0.0668	0
	0	0.3316	0	0.2445	0	0	0.1926	0
	0	0	0.1293	0	0	0.1166	0	
0.1289	0	0.1409	0	0	0.0597	0	0.2202	
0.1255	0.0807	0	0	0	0	0	0	0
	0	0	0.1183	0	0	0.0413	0.1226	
0.251	0.1549	0.0776	0.1569	0	0.1292	0.2651	0	0
	0	0.0254	0	0.133	0	0	0.1739	0
	0	0.0694	0.0602	0	0.08	0.1017	0.0468	0
	0.2096	0.1368	0	0	0	0	0.3078	0
	0							
AIF1L	4.6064	3.2592	2.0921	4.878	3.6582	2.0837	0.6626	
3.5423	3.4585	5.3498	3.2415	4.0622	0.4733	2.2678	3.5682	
0.9135	0.2019	4.1017	1.6508	1.9099	2.3596	3.9119	2.7007	
3.133	2.8229	0.7342	5.3803	4.5992	2.9092	3.2986	3.7052	
2.2272	0.5177	0.6326	2.1437	4.6641	2.8158	4.8014	4.9541	
3.6316	4.4773	0.0845	1.3209	4.5775	0.9643	0.9906	4.0968	
1.152	1.9649	0.2105	3.416	2.9419	4.5977	2.7198	2.7121	
4.6241	0.1753	5.2934	2.7992	4.767	3.6757	3.7348	3.221	
1.5663	4.8354	0.2522	0.006	0.9372	3.3241	4.1016	2.7041	
1.935	3.8018	3.1589	2.549	0.0868	2.9299	5.0709	2.5917	
2.8203	4.4173	3.8164	0.4421	4.2218	3.5094	3.991	3.484	
4.2867	4.8162							
AIFM1	5.6366	5.1734	5.5443	4.9955	4.9275	5.3296	5.818	
4.6913	5.8719	5.7076	5.4675	5.2908	4.7018	4.5944	5.2292	
5.5888	4.5829	5.2671	5.0865	5.6321	4.9168	4.8577	4.8229	

5.7152	3.8264	4.2665	4.418	4.9655	4.9168	5.5857	5.1616	
5.265	5.6522	5.6726	4.9547	5.7415	4.8229	5.4767	4.7462	
5.884	5.1355	4.7802	3.9463	4.6445	4.8566	4.6122	6.0124	
5.0309	3.7827	6.0529	4.9542	4.7437	4.4808	4.9365	5.5362	
5.4131	4.2948	5.7085	5.2479	5.6152	4.5776	4.9483	5.9244	
5.7545	5.1783	4.2128	5.4557	5.4604	5.3169	4.0717	5.2727	
4.7396	5.4457	4.6787	4.8194	5.3919	6.1178	5.6499	5.2859	
4.9587	4.9789	4.9175	4.8276	5.2831	3.9452	4.6875	5.1881	
5.3769	5.4311							
AIFM2	4.2301	4.0888	4.5815	2.9745	3.2664	3.3611	3.8108	
3.8621	3.9614	3.413	2.5008	3.172	3.5652	3.3582	2.6936	
3.5266	0.4243	2.5892	3.0626	4.3833	4.5683	2.9181	3.2817	
3.9625	2.9181	1.4738	2.8182	3.9155	2.5861	3.0372	3.4851	
3.5407	2.876	3.2589	3.4606	3.1532	4.4342	2.5887	3.3218	
1.8273	3.6694	3.4011	2.6143	2.9085	2.4598	1.1365	2.8952	
3.9665	2.9597	2.5298	3.5472	2.0605	2.3086	2.3684	4.6559	
3.8626	2.3342	4.099	3.3972	4.0187	3.1956	2.9696	4.171	
4.3326	2.5969	3.299	2.4168	4.4712	3.316	3.3834	1.8275	
3.0021	3.1748	2.2939	2.479	4.5003	4.0122	3.2446	2.3669	
4.4482	3.9741	2.4812	3.0653	2.682	2.4663	2.7012	3.5373	
3.6709	3.663							
AIFM3	1.1302	0.9158	0.6732	0.2648	0.1185	0	0.3701	
0.1412	1.0851	0.3135	0.5127	0.1818	0.194	0.1331	0.0526	
0.003	0	0.6068	0	0.0412	0.0672	0.069	0.1877	0
	0.116	0.0059	0.2289	1.5463	0.1832	0.8317	0.4467	0
	0.258	0.6297	0	0.0545	0.6654	0.0773	0.4669	0
	1.046	0.2809	0.2402	0.6042	0.092	0.1375	0.3281	
0.7337	0.0538	0.2446	0.3905	0.16	0.2412	0.418	0.4496	
0.0808	0.7871	0.8464	0.4052	0.2251	0.0806	0.0587	0.2414	0
	0.3374	0.2451	1.7214	0.5981	0.9275	0.188	0.1909	
0.2778	0	0.0953	0.0912	0.1786	0	0	0.8271	
0.0645	0.7679	0.1278	0.1936	0.1481	0.0661	0.137	0.4885	
0.6726	0.7858							
AIG1	2.693	0.2872	1.9317	4.1364	3.055	0.8233	3.1766	
2.7268	2.17	1.8081	2.7429	3.603	3.6201	0.6014	4.1761	
1.6907	4.5457	0.6487	1.4101	3.8113	2.381	2.2153	3.7369	
0.9579	1.9369	3.1682	3.9508	1.8667	2.6596	3.0955	5.229	
3.3312	1.545	1.8022	3.5246	2.6688	2.2194	3.7131	2.3043	
3.1981	3.4636	5.4534	3.5599	2.5506	4.5516	5.1326	2.2373	
2.7326	3.8645	2.7039	3.384	3.5822	2.4148	1.5935	4.8421	
1.7934	5.1255	4.344	1.8101	4.336	2.1401	1.2096	1.2073	
1.5896	1.7272	4.6717	1.4976	2.3186	2.622	2.5136	2.3143	
3.1349	2.9118	2.7726	2.5524	1.913	1.5177	4.3448	3.1606	
1.9542	2.1682	3.5032	4.3759	2.6227	3.719	2.0937	3.8162	
3.3644	3.2275							
AIM1	0.2408	0.3843	0.0901	2.3312	3.8801	4.5173	0.1298	
0.4858	0.4988	0.2086	0.3024	2.3856	2.3377	2.3187	0.7693	
0.6726	3.1833	1.4979	1.7296	0.0592	0.2869	2.2994	4.5966	
2.3289	2.1512	1.7041	0.4157	0.245	5.1862	0.3535	0.1813	
2.5357	3.6974	0.8767	3.1212	1.3276	0.2935	0	0.0793	

5.1081	0.5597	4.6926	4.5197	0.2479	0.2662	4.8405	0.481	
2.5546	2.9205	0.5031	0.3567	0.8021	0.3375	0.2301	0.652	
0.1393	1.2477	6.8292	3.1479	6.4286	2.1763	3.1085	0.3327	
3.1564	0.1801	1.4176	0.2579	0.1502	1.0739	0.2907	3.8395	
2.4037	0.3207	3.8625	0.3366	3.3108	0.7487	2.7239	0.2786	
0.3925	0.3174	0.3267	0.4439	0.2343	1.9997	0.3622	0.8795	0
	0.2317							
AIM1L	0.0306	0.0706	0.1325	0.0563	0	0	0.035	
0.0266	0.0256	0.1875	0.0787	0	0.0792	0.0384	0.0174	
0.0176	0	0	0	0.0457	0.0227	0.028	0	
0.0059	0	0.0043	0.0306	0.048	0.0185	0.0989	0.2214	0
	0.0373	0.0195	0	0.0581	0.0381	0.0822	0.1472	0
	0.0342	0.0736	0.0227	0	0	0.0377	0.0239	
0.0412	0	0	0.0539	0	0	0.0613	0.1983	0
	0.0597	0.0218	0.0359	0	0	0.0118	0.0096	
0.062	0.0186	0.0047	0.046	0.0474	0.0624	0	0.009	
0.033	0.0492	0.0074	0	0.027	0.0292	0	0.0087	0
	0.0611	0.0219	0.0202	0	0	0	0	0
	0							
AIM2	0.4344	0.5925	0	0.0558	2.5803	3.6949	0.2565	0
	0	0	0.0707	4.2333	0.479	2.1195	0	
2.1001	3.5835	0.0216	0.1794	0.5792	0.3461	4.023	3.1734	
1.6937	3.7057	1.0108	2.9982	0	3.8206	0	0	
1.8646	2.584	0.9656	0.2932	0.3048	2.5316	0	0.0461	0
	0.0408	4.444	3.324	0.3986	0.0406	4.0781	0	0
	3.0562	0	1.8786	4.3161	0.7567	0.3094	0	0
	0.2397	0.3027	1.9123	0	4.4445	1.8073	0	
0.5841	0	0.3208	0	0	0	1.1028	0.5882	
1.6748	0.645	4.6328	0.6224	0	0.9269	1.2803	0.2038	
0.3068	0	0.3464	4.8559	0.4782	4.8979	1.3663	0	0
	0.0435							
AIMP1	4.5886	4.283	4.1991	4.7048	4.506	4.3963	4.8758	
4.6311	4.0466	4.3022	4.136	4.5128	4.3256	4.7619	4.2602	
3.8132	4.2401	4.422	4.7248	4.4203	4.1405	4.512	3.9349	
4.7427	4.2144	4.8514	4.6403	4.2518	4.1557	4.0075	3.8118	
4.5313	4.2574	3.6804	4.583	4.54	4.8826	4.3334	4.3932	
4.4192	4.033	4.3396	4.4584	4.5317	4.4287	4.1738	4.7179	
4.3784	4.0705	4.7506	4.7421	4.846	4.7832	4.9825	4.3393	
4.3704	4.474	3.8935	4.485	4.4001	4.6433	4.8102	4.5477	
4.0584	4.6071	4.233	4.2214	4.3643	3.7288	4.446	4.1378	
4.5693	4.3957	4.3752	4.1647	4.1303	4.2245	4.2951	3.9345	
4.084	4.4399	4.4033	4.1653	4.2722	4.2813	4.7722	4.0254	
4.3617	4.0435							
AIMP2	4.5853	4.0785	5.0074	4.6305	4.6312	3.2851	4.1264	
4.6404	4.2638	4.0006	3.9678	3.4316	4.0117	4.6943	4.4648	
3.5457	3.7738	3.214	4.2935	5.3458	4.4385	4.5877	4.7446	
4.9429	5.4091	3.5044	2.6809	4.3192	4.9242	3.5859	4.7153	
4.7985	4.3517	3.1885	4.4435	4.1622	5.6646	4.4943	4.7335	
4.7682	4.5581	3.4376	4.2017	4.6074	3.7424	4.511	4.6041	
4.3834	4.1587	3.4471	4.6453	4.1501	4.5512	4.29	5.3399	

5.2309	3.4166	4.8387	4.4848	4.8589	4.8037	4.4712	4.7233	
4.5098	5.2003	3.2874	3.9592	3.6101	4.3969	5.0933	4.4954	
4.6866	5.2708	4.0431	3.7198	3.491	4.2883	4.1682	4.4131	
4.3963	4.543	3.5904	3.9223	3.9889	4.2824	4.7442	3.7921	
4.2238	3.8888							
AIP	4.6855	4.2574	5.2441	3.2748	5.313	4.4539	4.1399	
5.4622	4.1453	4.5131	4.5367	4.1754	4.0527	3.7145	4.0763	
4.7055	4.2262	3.3391	4.3077	4.6598	5.0939	5.552	5.065	
4.5984	5.0639	4.0245	4.3243	4.1264	4.5766	3.8887	4.8381	
4.3689	4.8964	4.9578	4.1314	3.5651	4.7232	5.4339	4.6638	
3.138	5.142	4.5206	4.372	4.552	3.8676	4.0285	3.5265	
5.1292	5.5277	3.9716	4.0371	3.6961	3.8916	5.1615	4.3489	
5.4718	4.4823	5.1018	3.7898	4.8476	4.5511	4.093	4.6558	
5.1441	4.4708	4.4904	4.8684	5.0145	3.4676	4.0053	5.3232	
4.7943	4.5107	4.3978	5.2189	5.1082	6.1573	4.1845	4.59	
5.1378	4.1812	4.7703	4.7826	4.2321	5.1035	4.8842	5.1309	
5.2581	5.2104							
AIPL1	0.0862	0.0505	0.0368	0.0416	0.0109	0	0.0697	
0.0706	0.0501	0.0363	0.0168	0.0135	0.0612	0.126	0.0622	
0.0248	0.0855	0.0661	0	0.0393	0.1035	0	0.0365	
0.0671	0.0181	0.0448	0.0998	0.082	0.0556	0	0.042	0
	0.0199	0.0144	0.0739	0.0813	0.0172	0.0516	0.0078	
0.0625	0.0111	0.0303	0.0621	0.0181	0.0613	0.0163	0	
0.0505	0.0103	0.0689	0.0375	0.0204	0	0.0129	0.0568	
0.207	0	0.04	0.022	0.0213	0.043	0.0571	0.0584	
0.0674	0.0792	0.039	0.0813	0.015	0.0496	0	0.0154	
0.0482	0.0209	0.0094	0.0571	0.0204	0.0335	0.02	0.0107	0
	0.0156	0.0126	0	0.08	0.0303	0.0704	0.0275	
0.0306	0.0392							
AIRE	0	0.0498	0	0	0	0	0	0
	0	0.038	0	0	0.0557	0	0.0195	
0.0262	0	0.0682	0	0.0791	0.0585	0.0144	0.0139	
0.0444	0	0	0.1319	0.0746	0	0	0.0277	0
	0	0	0.01	0	0	0	0	0
	0.0308	0.0121	0.0629	0.0898	0	0.0462	0	
0.0512	0.0315	0	0	0	0	0.0261	0	0
	0	0.0407	0.0991	0	0.0184	0	0.0294	0
	0	0.0049	0	0.0103	0	0.0745	0.0311	0
	0.0401	0	0.0559	0.0328	0.0506	0	0	0
	0	0	0	0	0	0	0	0
	0.1129							
AJAP1	0	0.1035	0	0	2.5483	0	0	
0.1082	0.1932	0	0	2.8006	4.0691	0	1.1394	0
	5.335	0.0526	0	0	0.0531	0.0653	0.168	
0.0749	0.04	0.0333	0.7748	0.33	0.0519	2.534	0.0847	
0.0216	0	0	0.029	0.1315	0.2157	0.0491	0.8256	
0.0382	0	0.3309	0	0.0606	4.6791	0	0.0518	
0.0899	0.2599	0	0.0882	0.043	0	0	0	0
	4.1067	2.844	0	1.9554	0.6054	0	0.0802	
0.0456	0.0807	0	0.3578	0.7181	0.4334	0	0	

0.0709	0.0606	0	0	0	0	1.893	0
0.0076	0	0.5412	0	0	0.276	0	0.4102
0.5348	0.3272						
AJUBA	2.657	1.9698	1.8988	3.5037	3.2074	3.3849	2.811
2.8585	1.8246	2.2372	1.6685	3.3165	5.9449	1.4642	3.6997
3.0035	3.5376	2.6362	2.1098	2.6095	2.3142	1.8593	3.3781
2.0656	2.3165	5.0285	1.7132	2.32	2.2085	1.7748	1.7056
2.0549	2.135	1.5123	2.0564	2.4228	2.3861	2.8187	3.0704
3.0339	2.0345	2.9204	2.0136	1.9383	4.9057	4.2478	1.7157
2.5193	3.8452	4.671	1.4541	2.7602	2.0635	2.3729	4.044
1.7042	5.008	2.1682	1.0567	3.3432	3.4093	2.1577	2.9698
1.5245	2.438	4.4042	2.6184	3.5547	1.1482	3.4927	2.4498
2.5559	2.0483	1.5827	3.4738	1.8046	1.5752	1.9764	1.7331
1.5486	1.8582	4.3895	2.0466	1.5641	4.3502	1.8623	0.929
0.8867	1.2767						
AK1	4.1878	3.532	4.3286	2.8773	4.3341	3.7569	2.857
4.3605	3.0029	2.9443	3.7508	3.4806	4.6673	2.5006	3.6684
3.9968	3.481	2.6107	2.5533	4.8909	4.6032	3.1294	3.6233
3.5456	3.9921	3.3107	3.1482	4.0236	3.8802	3.5142	3.9645
3.2602	3.5525	3.6375	3.4416	2.8064	3.8166	4.0793	4.0749
2.2275	3.7491	4.8773	3.5213	3.5345	2.7221	3.0926	3.3874
3.1744	4.342	3.6287	3.7524	3.6372	3.0512	2.4711	3.8998
3.4387	4.4805	4.3607	3.354	3.8805	3.9009	3.5257	3.5619
3.7092	4.5661	4.7757	2.5868	4.847	4.1801	5.0641	3.9458
3.072	4.7527	3.172	4.0115	3.7285	3.3636	3.89	3.3167
3.7181	3.2224	4.4598	3.8361	4.6249	4.0413	4.005	4.5515
4.8115	4.5432						
AK2	6.4583	6.4663	6.5916	5.9937	6.1403	5.9322	5.6902
5.5966	6.3474	6.1877	6.4262	6.2176	6.0156	6.6117	5.0369
5.642	5.9297	5.574	6.9645	5.961	6.1632	5.4975	4.8801
6.3812	5.0602	5.4347	5.3331	5.9808	5.8571	6.9519	6.1346
5.8972	6.6372	5.7214	5.7225	6.4328	6.3252	5.6994	6.1246
6.388	5.9662	5.6155	5.159	5.9599	5.6024	5.8559	6.0273
6.3293	4.8186	6.5894	5.9544	5.638	6.4071	6.6188	5.6591
6.1796	5.3103	5.9711	5.9711	5.8861	5.9438	6.264	5.4349
6.7617	5.5371	5.329	6.009	4.7826	6.2191	5.2155	5.4039
6.4558	6.2732	6.7042	5.3166	5.3774	6.6079	5.2942	6.3278
6.3958	6.3542	5.5836	5.3223	5.1583	5.2463	6.0607	5.3536
5.5825	5.6115						
AK3	5.0053	4.2228	4.6936	4.589	3.4692	2.3876	3.1972
3.5415	4.4185	4.2748	4.4579	4.3798	3.8003	3.2101	4.0569
3.8208	4.2723	4.8098	4.2769	3.977	4.2195	3.2347	4.1028
2.7774	4.4171	2.5604	5.858	4.4171	4.2191	5.7399	4.3223
2.892	3.4356	4.1096	3.5609	4.3103	3.1976	4.1291	3.4084
4.543	4.3227	4.93	4.7733	3.2978	5.2506	3.4787	4.3564
3.8324	4.3261	2.6569	4.6648	4.1991	4.0759	3.6045	3.9498
2.9074	4.8094	4.8077	4.274	4.5379	3.8635	5.4516	3.4177
3.2881	3.1156	5.4461	3.1319	5.1357	4.5137	3.8983	4.7842
3.423	3.7206	4.0828	4.2082	3.4492	3.0434	4.4246	4.518
4.6834	3.6425	5.2084	4.9125	3.4847	3.586	3.8691	5.1104

4.9352	5.1543						
AK4	2.7341	4.029	2.2228	3.2388	3.9148	4.12	3.5828
1.8254	3.5112	1.0764	4.0935	2.8646	3.4873	4.5532	1.6699
3.0209	2.6127	0.9957	3.2347	4.1282	3.5988	3.0434	3.3435
2.0323	2.0803	4.0472	2.7885	2.1207	2.4203	4.7773	4.6937
2.1257	4.0445	3.8593	0.9109	3.0724	2.2982	3.0843	2.9721
4.8798	2.0982	3.8974	2.8585	3.9972	3.579	4.7137	3.3697
4.2536	2.6375	3.7565	1.0468	3.7447	4.105	1.8367	3.4914
2.5559	3.6755	2.036	2.3542	1.2294	3.7573	3.5976	2.0011
3.6686	2.376	4.1632	0.9015	3.1755	2.8625	2.3008	3.6161
4.5455	3.5187	3.6592	3.3632	2.0533	3.5316	1.1347	4.8579
5.1067	3.9687	4.6874	3.8797	1.8948	2.182	3.2846	3.8855
3.0427	2.3217						
AK5	0.0526	0.1891	1.2386	0.7653	0.213	2.577	4.1265
0.7749	0.7766	0.0988	1.4017	0.3277	4.1261	2.6959	2.0599
0.5291	4.5357	0.2363	1.5197	0.6566	1.0537	0.1393	1.0956
0.6358	1.57	4.3057	0.1333	0.0558	2.3674	0.7653	0.3267
0.1061	4.8967	2.7665	1.9893	0.1414	0.2177	0.4566	1.0287
0.3237	0.8467	2.2208	3.7975	1.2706	2.1339	3.7013	0.3584
0.1664	4.2559	5.8956	2.651	0.2384	1.0794	0.2958	0.0935
0.7957	1.8416	2.0044	0.2596	2.1761	1.6683	0.4661	0.2818
4.9731	0.0992	1.9159	0	6.9125	0.2107	2.2171	2.8882
2.9759	1.2216	0.1226	1.9272	4.1274	2.01	0.5645	0.6829
0.662	0.2314	3.023	3.5278	0.4354	2.9427	0.9022	4.3382
3.6542	2.8535						
AK6	4.7708	4.5839	4.6817	5.1816	4.8713	4.762	4.5598
4.9547	4.1731	3.9885	4.2237	4.7439	5.2382	5.3079	5.3143
4.5233	4.959	4.4113	5.8227	5.3746	4.5993	4.9531	4.5409
5.3849	5.0331	4.7746	4.2122	4.8014	4.588	4.1805	4.5256
4.7037	5.2624	4.3057	5.5317	4.9705	4.0769	4.3256	3.8373
4.7347	3.9797	4.8162	4.9481	4.4324	4.3996	4.7918	4.3369
4.8051	4.5471	5.6128	5.1464	4.951	4.3607	5.292	4.3907
5.0236	4.5714	4.2382	5.0532	4.24	4.6978	4.8855	5.2193
4.9665	4.3407	4.5714	4.8614	4.9253	4.5212	4.6279	4.1414
5.0785	4.7554	4.5549	3.9939	5.6883	5.0023	4.4009	4.8272
4.6999	4.5152	4.2971	5.5694	4.7275	5.3611	4.7476	4.6256
4.6465	4.5169						
AK7	0.6443	0.5253	0.3747	0.1769	0.2104	0.1256	0.7927
0.4694	0.3318	0.5604	1.0297	1.2153	0.2855	0.8916	0.2838
0.0778	0.3986	0.4269	0.5008	0.1964	0.192	0.1701	0.1716
0.0807	0.0495	0.1412	0.6165	0.5278	0.2745	1.3658	2.2243
0.1732	0.3768	0.8831	0.0869	1.2474	0.3671	0.9021	0.5566
0.5274	1.8704	0.5553	0.0138	0.3747	0.0816	0.1465	0.2673
0.2411	0.2734	0.1691	0.2588	0.8141	0.2371	0.2695	1.4727
1.3629	1.7163	0.5816	1.2853	0.415	0.5575	0.2153	0.1548
0.6443	0.5265	0.3269	1.308	0.1625	0.0462	0.3228	0.5309
0.091	0.1236	0.4159	0.5088	0.0432	0.2766	0.5195	0.1938
0.8819	0.423	0.1808	0.2235	0.3224	0.2183	0.5357	1.492
1.069	1.4246						
AK8	0.4457	0.0956	0.0858	0.0977	0.0787	0.023	0.0143

1.0664	0.057	0.0308	0.3212	0	1.0744	0.1397	0.5565	0
	0.3611	0	0	0.107	0	0.0192	0.0349	0
	0.0644	0.338	0.0492	0.8802	0.1188	0.3295	0.6599	
0.0511	0.0405	0.3375	0.0266	0.0193	0.5796	0.3899	0.1524	
0.4737	0.6363	0.0982	0.1633	0.2069	0.0133	0.1251	0.0213	
0.2205	0.109	0.9456	0.1432	0.0433	0.1986	0.1705	0.4086	0
	0.2982	2.6001	0	1.8377	0	0.057	0.059	
0.1264	0.2605	0.8445	0.2844	0.115	0.1696	0.5252	0.1416	
0.0812	0.1028	0.05	0.0668	0.0639	0.0508	0.2546	0.1136	
0.2103	0.1333	0.101	0.0556	0.2336	0.1512	0.1111	0.5693	
0.2553	0.5067							
AK9	1.259	1.6936	1.2351	0.8552	0.4735	1.2014	1.3244	
0.7469	1.647	1.6643	1.8908	0.4148	0.7288	1.7893	1.2762	
0.7863	0.9618	1.3309	1.6815	1.8413	1.2621	0.2231	0.6958	
0.8246	0.5318	1.4871	0.4328	1.3364	0.4539	1.6276	1.0823	
1.522	0.8352	1.867	1.0725	1.5525	0.7085	0.8059	0.3971	
0.4727	0.8752	1.0757	0.68	1.1002	0.7549	0.9269	1.5191	
1.5138	0.5369	1.6913	1.3465	0.4612	1.3962	1.6955	0.9689	
1.3331	1.5452	0.8571	1.3568	1.0586	0.3784	0.9389	0.3093	
1.3318	0.8222	0.8663	1.2286	0.7041	1.5737	0.9548	0.8902	
2.5844	1.5411	1.4504	0.7328	1.1552	1.5571	1.1426	0.9382	
1.0996	1.3593	0.8302	2.1125	0.819	0.3455	1.1091	2.1094	
1.5248	2.0694							
AKAP1	4.6831	4.1994	4.3865	4.2469	4.0675	3.8952	3.1631	
3.7181	3.6661	3.8016	4.2398	3.8844	3.5286	4.078	3.3634	
3.7712	4.024	3.7058	4.0045	4.6415	4.4248	3.5592	3.4747	
4.5652	3.1204	3.4905	3.9774	4.9196	3.6959	4.3708	3.2625	
4.501	3.4882	3.3541	3.3288	3.9704	5.0064	3.7169	3.8246	
3.6977	3.9366	3.6428	2.9173	4.0813	4.2613	2.5806	4.7667	
4.334	3.2676	3.7428	4.049	3.5933	3.4791	4.144	3.6118	
4.3058	3.6056	3.2956	3.9611	3.5197	3.9682	3.9999	3.7048	
4.1971	4.8919	2.5498	4.7307	3.7646	4.2673	3.4308	3.7399	
3.8012	3.9232	3.2604	3.903	4.762	4.0147	3.819	3.8578	
4.4338	4.3297	3.1467	3.5256	4.3248	3.276	3.3245	3.1102	
3.6336	3.1283							
AKAP10	2.978	3.4529	3.1574	3.8884	3.4214	3.281	4.8088	
3.2994	3.379	3.5665	2.7145	3.3442	3.6975	4.0326	3.1064	
3.6241	3.9031	4.0403	3.2165	3.1796	3.2481	3.5805	3.3615	
3.3874	2.7725	3.9643	3.5414	3.205	3.5768	3.265	3.8504	
3.8978	3.4305	2.52	3.436	4.0131	2.5634	3.6496	3.2064	
4.0824	2.9401	3.7079	2.7686	3.745	3.2457	3.894	3.8365	
3.6421	2.5871	3.6265	3.7716	3.8606	3.4342	2.9893	3.6818	
3.1254	3.7378	3.0447	3.171	2.9824	3.8318	3.9136	3.1704	
3.304	3.0087	3.8443	3.222	3.6381	3.018	3.6185	3.5204	
3.5753	3.6292	3.5076	3.6218	3.613	3.3169	3.5484	3.5173	
3.9664	4.0166	3.5918	3.0817	0.8539	3.0491	3.306	3.6126	
3.3088	3.4097							
AKAP11	3.3857	2.2461	3.6976	3.6184	3.7197	3.8068	3.7783	
3.0035	4.1063	3.4533	3.2008	3.3602	3.4608	4.1668	3.5329	
3.0339	3.1276	3.3756	3.3599	3.2705	3.6999	3.3011	3.5639	

2.917	3.5023	3.5107	3.5467	3.0103	3.0792	3.0435	2.9965	
2.6939	3.2083	3.3722	3.4125	3.4587	2.8365	3.1057	2.368	
3.2184	3.1	3.6963	3.8726	4.3316	3.5995	2.9947	3.8361	
3.5258	3.4608	3.0016	3.1113	4.1216	2.6967	3.1739	2.7941	
3.2313	3.627	2.6502	3.4869	2.8056	3.4146	2.8022	3.5283	
3.7546	2.7639	3.0113	2.597	3.8474	2.8705	4.1674	3.9489	
3.7118	3.7088	3.2968	3.793	3.7949	2.7767	2.8624	3.1319	
3.7914	3.158	3.6479	3.219	3.902	2.424	2.7447	3.6839	
3.5691	3.2722							
AKAP12	4.1133	3.4889	3.7513	2.9318	4.9315	4.3581	3.9697	
4.66	5.6014	4.3527	3.7438	4.9354	4.0314	5.0787	3.281	
4.5733	6.9344	4.3946	5.4202	4.2298	3.5877	3.8535	5.0111	
4.2616	3.525	4.8605	7.122	4.0793	4.2671	5.405	4.276	
4.1027	4.3736	5.9319	5.8346	4.2418	2.8296	4.0678	6.4039	
4.3563	5.2323	3.1164	5.5226	5.5786	6.3817	2.0615	3.5477	
3.7489	6.0047	4.1652	5.1395	5.1762	5.3554	3.9941	4.7712	
1.5957	5.028	3.6839	4.2055	3.2742	4.8331	2.528	4.8814	
4.5976	3.9107	2.7312	4.1732	5.9906	4.6867	3.6131	4.1151	
5.9072	4.2468	5.1258	6.6114	6.1686	3.7828	5.548	3.5305	
4.3216	4.0064	5.2605	4.9236	6.2106	5.2469	5.0998	5.1144	
3.885	5.0696							
AKAP13	2.1187	2.9092	2.948	2.7703	2.6601	2.7843	3.5686	
2.8011	2.8015	3.1891	2.7363	2.7659	2.6523	2.4329	2.7429	
2.9933	2.9886	3.9418	3.3886	2.1903	2.3609	2.1819	2.4496	
2.9068	2.5715	2.5197	2.9796	3.1679	2.6618	3.6907	3.0239	
2.7438	2.1171	2.4284	2.6155	3.2068	2.6331	3.3138	2.1903	
3.1935	2.3294	2.9661	2.8299	3.1188	4.3797	3.0709	3.5077	
2.6005	2.5465	3.8922	2.569	2.5358	3.0649	3.0493	3.1775	
2.7762	3.3657	2.4584	2.8882	2.7243	2.2666	2.9301	2.8024	
3.412	4.2945	2.6056	2.7845	3.5482	4.6582	2.1565	2.5271	
1.9715	2.8244	2.5881	3.0251	3.0057	3.1246	3.146	2.5454	
2.7373	2.3713	2.99	2.839	2.6156	2.9701	2.2485	4.3746	
3.0749	3.9998							
AKAP14	0	0	0	0	0	0	0	
0.0922	0.4559	0	0	0.0269	0.0568	0	0	0
	0.1104	0	0	0	0	0.1397	0	0
	0.0647	0	0	0	0	0	0	
0.1193	0.1249	0	0.0623	0	0.0742	0	0	0
	0	0	0	0	0.0788	0.0686	0	
0.2286	0	0	0	0.1087	0.1039	0	0.13	0
	0.1788	0	0	0	0	0	0	
0.3898	0	0	0.1401	0	0	0	0.0899	
0.084	0	0.0124	0	0	0	0.0703	0.1417	0
	0.1118	0	0	0	0	0.1172	0	0
	0							
AKAP17A	4.2189	3.7806	3.9715	3.6056	3.4861	3.5543	3.8166	
3.1279	3.8587	2.7172	3.1044	3.8269	4.0498	2.496	3.8599	
4.0821	2.409	4.0406	3.0563	3.7452	2.9777	4.5258	3.6233	
4.129	3.3018	3.1636	5.3803	3.9976	3.598	3.9653	4.0862	
3.7877	3.2759	3.6407	4.3444	3.0697	3.7163	3.0858	3.5615	

3.2549	4.1351	3.753	3.5186	3.772	4.0668	2.5051	3.7826	
2.9105	3.7514	2.6057	4.3553	3.3186	3.5962	3.17	4.4145	
3.5543	2.958	3.3576	3.8291	3.9134	4.164	4.8434	4.3758	
3.1115	3.7545	3.95	3.3994	3.8617	4.0068	3.9542	2.8426	
3.6253	3.0271	3.6959	3.4524	4.4085	4.3517	4.2265	2.4782	
3.4981	2.8919	3.8819	3.92	4.1681	2.9587	3.6175	3.3844	
3.6031	3.8513							
AKAP2	4.0318	3.0955	4.5529	3.7604	3.9664	4.1852	3.3797	
3.6001	3.0951	3.3657	3.8514	4.491	5.9762	3.3502	4.4493	
3.2598	5.79	3.6497	3.6851	5.2923	4.7429	3.0994	3.5531	
4.1364	3.9166	5.006	3.6381	0.4135	3.4856	3.5947	0.0621	
3.3535	3.8272	4.8923	4.0981	3.7777	3.4757	4.2324	3.8016	
3.8742	4.1501	4.3433	3.8626	2.5602	5.5914	4.7186	3.9409	
3.4194	4.1688	5.8435	4.4122	4.5386	3.8008	4.1218	0.6233	
2.9404	6.8295	2.621	4.4386	2.8462	4.1033	3.7075	3.7611	
4.4377	4.6089	6.1367	3.6955	4.064	3.7763	4.4972	2.5584	
3.4187	2.4709	4.0444	3.7003	3.9115	3.2648	3.3383	3.4441	
3.1006	3.6859	4.4326	3.5445	4.7843	4.2205	3.6992	4.501	
4.1122	4.2846							
AKAP3	0.0455	0.0537	0.3224	0.0552	0	0.462	0.3208	
0.2939	0.1332	0.0419	0.1895	0.0282	0.5039	0.8635	0.0797	
0.3783	0.339	0.2695	0.1455	0.1401	0.3112	0.1016	0.0204	
0.119	0.0639	0.3142	0	0.5172	0.0399	0.1349	0.8848	
0.2029	0.5339	0.1009	0.5301	0.1422	0.445	0.0217	0.073	
0.4488	0.0818	0.0628	0.5548	0.2425	0.0612	0.3439	0.174	
0.3133	0.1494	0.4677	0.3051	0.2264	0.0499	0.1533	0.2255	
0.5802	0.1625	0.2108	0.1206	0.1423	0.0312	0.1457	0	
0.1166	0.4293	0.2116	0.0532	0.787	0.2662	0.1596	0.3162	
0.105	0.2402	0.1175	0.3139	0.4604	0.4689	0.0062	0.4038	
0.2017	0.1463	0.0093	0.2566	0.1815	0.0398	0	0.1753	
0.1132	0.4493							
AKAP4	0	0	0	0	0.0099	0	0	
0.013	0	0	0	0	0	0	0	0
	0	0	0	0	0	0	0	
0.0207	0	0	0	0	0	0	0	0
	0	0	0	0	0	0	0	0
	0	0	0	0	0	0	0	0
	0	0	0	0	0	0	0	
0.041	0	0	0	0.0179	0	0	0	0
	0	0	0	0.0023	0	0	0.0109	0
	0	0	0	0	0	0	0	0
	0	0	0	0	0	0.0347	0	0
	0							
AKAP5	0.3452	0.6185	0.6363	1.2674	0.2306	0.2129	0.1364	
0.3351	0.2908	0.357	0.1574	0.3887	0.2164	0.5751	0.1718	
0.079	0.3507	0.4812	0.7527	0.2266	0.1998	0.147	0.2696	
0.1882	0.3196	0.1812	0.2419	0.922	0.1395	0.2561	2.4024	
0.2412	0.3217	0.2632	0.0721	1.3348	0.1663	0.4224	0.0492	
0.3446	0.3343	0.9043	0.2783	0.625	1.0346	0.9564	0.1841	
0.312	0.0953	1.2195	0.2344	0.3111	0.2833	0.342	2.1676	

0.2799	0.1199	0.4599	0.4941	0.5575	0.216	0.3442	0.3096
0.2252	0.2772	0.1324	1.4771	0.093	0.3372	0.0864	0.2974
0.2321	0.2449	0.2705	0.5091	0.6536	0.6693	0.2441	0.2472
0.1742	0.6523	0.4705	0.1255	0.1105	0.5692	0.2728	0.0481
0.154	0.1453						
AKAP6	1.8703	0.1922	0.4782	0.8294	0.2115	2.4863	1.0543
1.2245	0.5718	0.7531	0.3691	3.7848	0.0953	2.2061	2.9321
0.7651	0.5674	0.8205	1.3864	0.9817	1.6354	3.1079	2.8094
1.156	0.131	0.3483	1.1926	0.6887	4.9946	0.5803	0.5852
1.0069	1.2189	1.858	3.9396	0.6542	2.7394	4.4846	1.7336
5.0626	0.8748	3.6315	0.1663	0.743	0.0087	1.8648	1.8264
1.2682	0.3659	0.8391	1.2462	0.8558	0.9771	0.531	0.3777
0.3267	0.7549	0.3052	0.6823	0.7421	1.2864	1.3497	4.8424
0.2432	1.1972	0.8463	1.4755	4.0376	0.863	0.3499	2.4907
0.6444	1.7102	1.3927	0.9348	0.9196	1.584	4.0864	1.8524
3.105	0.8357	1.584	2.4747	4.435	0.4313	0.9149	2.1948
1.1931	1.6986						
AKAP7	2.602	2.115	1.9734	2.0879	1.0828	1.9647	1.5523
1.8469	2.6048	1.7926	1.4722	2.0885	0.9396	2.3811	2.4546
1.5226	1.6433	2.3911	2.4276	1.8066	2.6539	1.4417	1.9671
1.8227	1.8704	1.3336	2.0012	1.5287	2.2453	2.2098	1.4927
2.058	1.7287	1.6988	2.1876	2.1615	1.5195	1.5975	2.241
1.7802	1.8501	2.6261	1.5256	1.4562	1.5802	1.6879	1.825
1.3442	1.2638	1.8967	2.0252	1.7402	1.4636	2.5529	2.2723
1.7819	1.6116	1.578	1.4599	1.6384	1.8288	2.4009	1.6666
1.8784	1.3498	0.9293	1.5695	0.77	1.9406	1.9488	1.666
2.1969	2.1841	1.2724	1.9841	1.1552	2.2463	2.1513	1.6489
2.3847	1.8834	1.5988	1.6019	2.2896	1.593	2.2196	2.1525
2.0962	2.2484						
AKAP8	3.1505	3.5938	3.6554	3.6562	3.6584	3.4082	3.4265
3.871	2.9965	3.5262	2.9382	3.0261	3.4138	4.0181	3.7713
2.8838	3.2398	2.8917	3.5663	3.2161	3.6849	2.9126	2.8682
2.603	3.2311	3.4291	3.2753	3.3079	3.2105	3.2676	3.4486
3.5376	3.2651	2.7806	2.8623	3.2893	3.4585	3.2617	3.6173
3.3565	2.9447	2.8363	3.1004	3.3892	3.3969	3.0783	3.256
2.9692	3.1544	3.5893	3.0935	3.0637	3.2206	3.8194	3.7358
3.9273	3.5355	3.5661	3.2247	3.743	3.4691	3.2751	3.0701
3.3365	3.2965	3.0038	3.4233	3.322	3.9017	3.4703	3.2491
3.5253	3.3842	2.8152	2.8406	3.1204	3.7194	2.9742	3.0808
2.9705	3.0852	3.4558	3.1532	2.8214	3.9471	3.1689	2.727
3.1808	3.1231						
AKAP8L	4.5495	4.4526	4.4294	4.2925	4.2086	4.0686	4.5073
4.3159	4.3486	4.6052	5.155	4.1585	4.6977	4.4792	4.5882
3.8053	4.2539	4.2985	4.1857	4.2079	4.6769	4.0036	4.2942
3.167	4.2481	5.1544	4.3731	4.2724	4.0404	4.9616	4.8362
4.1557	4.1626	4.8072	4.3546	4.2764	4.7275	4.2567	4.7743
4.6625	4.5284	4.6741	4.3537	3.7002	4.6692	4.1838	4.3163
4.2243	4.3605	4.2104	4.3619	3.8963	4.1216	4.8441	4.7252
4.4754	4.6482	4.7067	3.6156	4.6192	4.4711	4.1424	4.4049
3.6761	4.1712	4.9195	4.8573	4.4811	4.8385	3.8967	4.1203

4.2929	4.3524	4.2846	3.5464	4.7096	4.4134	3.8879	4.2664	
3.9327	4.2431	4.3993	4.746	4.5252	4.8981	4.1337	4.4697	
4.2104	4.5371							
AKAP9	2.0706	3.0092	2.1827	3.3287	2.918	4.743	3.3668	
3.9635	3.4511	2.7625	3.4307	2.476	2.776	3.6334	2.8197	
3.7405	2.5437	4.5771	3.5923	2.3424	2.9449	2.7505	3.2151	
3.2845	2.8766	3.3032	3.4184	3.0141	3.262	2.9675	2.7564	
3.9663	3.4056	3.0033	3.9772	2.9421	2.6433	3.9454	3.6693	
2.9389	3.1366	2.3351	2.5526	3.6922	3.0301	3.242	2.7247	
3.0319	4.2155	2.4305	2.991	3.1369	3.2244	2.2938	2.8849	
3.4226	1.9906	2.6598	4.0555	2.7839	2.9114	3.2216	3.6542	
3.1527	2.8386	3.5579	2.9389	3.392	3.4744	3.1283	3.3127	
2.9409	2.9294	2.3635	3.0602	3.1794	2.5247	3.7808	3.7071	
3.3684	3.0802	2.945	1.577	4.0141	3.3337	3.2174	2.6891	
2.509	2.6377							
AKIP1	4.2119	4.1569	3.8474	4.2809	3.3512	4.0768	3.5724	
4.64	3.9375	4.1408	4.6538	3.4676	4.6416	4.2368	4.4165	
3.8805	4.3764	3.6207	5.0861	3.7182	3.8395	4.0098	3.3314	
4.2993	4.5766	4.4648	3.6376	4.2229	4.0285	3.1495	3.6674	
3.735	4.6029	4.23	4.3434	4.2567	4.7716	4.0508	4.0434	
4.416	4.0668	4.2868	4.7397	4.2936	4.0408	5.0134	3.8074	
4.0876	3.993	4.6801	4.0548	3.8983	4.0127	3.5453	4.6693	
3.8061	4.64	3.5816	4.1104	3.5953	3.6593	3.9081	4.2298	
4.2442	4.2001	4.3563	4.3005	4.6981	3.969	4.2511	3.9193	
3.912	4.2711	4.2523	3.7146	4.2875	3.7973	3.4747	4.2649	
4.3766	3.6958	4.3874	4.6742	3.2118	4.5095	4.2658	4.1561	
4.1244	4.0552							
AKIRIN1	5.1813	5.484	5.4312	5.2873	5.467	5.1862	6.1242	
5.6133	5.9925	5.623	5.3091	5.5462	4.5839	5.6889	5.0674	
5.6908	5.2926	5.122	5.7644	4.878	5.0303	5.0148	4.8147	
5.6044	5.4125	5.7204	4.7995	6.1912	4.8033	6.4198	6.3438	
5.7581	5.7309	5.3891	5.6708	5.9172	5.6689	5.6326	4.9446	
5.7204	5.1583	5.2422	5.0631	4.9978	5.4212	5.3441	5.5097	
6.1493	5.36	5.3664	5.4042	5.229	5.9549	6.0733	5.772	
5.2457	4.9432	5.6767	5.302	5.3914	4.8398	5.3784	4.7943	
5.3702	5.4378	5.7037	5.7113	5.0927	6.6245	4.857	5.0011	
5.2349	5.6028	5.528	4.5111	5.2206	6.1264	4.8844	5.3104	
5.1814	5.5587	5.1855	5.4928	4.9487	4.9487	5.5786	4.9022	
5.0896	4.7021							
AKIRIN2	4.3447	4.0003	3.9489	5.1	4.3245	4.133	5.3721	
4.1885	4.0841	3.8562	3.718	4.3245	4.1561	4.6004	4.3278	
4.0756	4.5779	4.1885	5.3168	4.2955	4.2981	5.2839	5.7245	
4.2615	5.2586	4.2741	4.5941	3.4641	4.8023	4.1538	4.6432	
4.4485	4.077	4.0124	4.5237	4.3884	4.1564	3.876	4.8052	
4.4853	3.9564	4.7036	5.6281	4.3707	4.1059	5.2586	4.7021	
3.6549	4.5317	4.5663	4.8791	4.9582	4.5993	5.2184	4.9289	
4.5246	5.1386	4.6959	3.6846	4.7271	3.9646	4.2308	4.6482	
4.0408	4.8386	4.1391	3.3774	3.8797	4.6451	4.953	3.647	
4.5791	3.9307	5.3648	4.681	3.5543	4.1823	4.4097	3.7392	
4.3366	4.1711	4.1819	5.6086	3.9549	4.8208	4.3358	4.1435	

3.9202	3.6122							
AKNA	2.6721	1.1606	1.2614	1.4125	0.8335	0.903	0.787	
0.8638	0.8329	2.7649	0.7793	0.453	2.2413	0.7527	0.6402	
1.1188	2.1252	1.5821	0.4764	1.1416	1.1631	0.7751	0.3971	
0.8015	1.7212	1.1742	0.5917	0.9143	0.9129	0.356	1.1148	
0.8597	0.4831	1.7025	1.1774	0.5735	3.5549	2.9072	3.7442	
0.2302	1.0599	0.8235	0.3795	0.7426	0.5966	1.0719	1.8522	
0.5903	0.5552	1.306	1.6494	0.3189	0.9974	3.9348	1.2302	
1.42	2.8384	2.6781	1.2991	2.9195	0.7484	1.1496	0.5486	
1.4059	1.344	1.2764	0.8593	2.2874	1.1831	0.8466	0.5472	
1.0137	0.3832	0.4338	0.2426	1.453	0.976	0.4964	0.5526	
0.8452	1.5046	0.328	0.5757	0.9628	1.3724	1.2353	1.905	
1.5996	1.9306							
AKNAD1	0.0333	0.1148	0	0.1634	0.1677	0.1138	0.0731	
0.1958	0.0392	0.0278	0.0607	0.1699	0.3184	0.1406	0.1477	0
	0.6643	0.115	0.1681	0.7201	0.0229	0.0558	0.0312	
0.1746	0.1321	0.5289	0.009	0.0302	0.0957	0	0	
0.2449	0.0915	0.0424	0.2814	0.0475	0.1769	0.0059	0	
0.1846	0.1258	0.2369	0.4116	0.1708	0.1352	0.2286	0.1252	
0.1218	0.5089	0.6939	0.0381	0.8158	0.3303	0.0985	0	
0.1833	0.2643	0.0601	0.4039	0.1262	0.062	0.1498	0.0366	
0.1696	0.0746	0.0298	0.1011	0.0327	0.0342	0.1784	0.0593	
0.21	0.0505	0.0651	0.1394	0.0801	0.0608	0.0507	0.1031	
0.0906	0.0635	0.2296	0.0466	0.0807	0.54	0.1749	0.0364	
0.0397	0.0169							
AKR1A1	4.3416	5.7447	4.8135	4.7431	4.4867	4.7831	5.6295	
4.9258	5.9124	4.9887	5.2919	6.3566	4.3359	5.1707	4.8988	
6.4649	5.5574	5.3622	7.9888	4.7149	5.1246	3.4175	3.6513	
5.0034	4.1905	5.1014	5.0743	6.0875	4.3295	5.3217	5.7385	
7.2671	5.502	6.291	5.211	4.7783	4.9092	5.2603	4.6488	
4.7693	4.7854	5.3956	4.6298	4.9378	5.0237	4.7562	4.9654	
7.7787	4.3919	5.114	4.9966	4.6028	6.6702	4.6133	5.0477	
4.7116	5.4709	4.6072	5.0042	4.693	4.6283	5.1804	4.1557	
5.2801	4.5266	5.6546	4.7329	6.0508	6.2617	4.4314	4.1467	
4.3953	3.7122	4.6308	4.4092	5.357	5.4998	4.1588	7.0055	
5.2886	8.2668	4.4811	5.9926	4.3518	4.954	6.2741	5.0944	
4.9611	5.0073							
AKR1B1	6.6813	6.638	6.4466	6.9997	7.5128	7.6491	8.3864	
7.759	6.9253	7.2278	6.0948	8.1834	8.0645	6.3796	7.0378	
8.6868	6.243	5.5204	6.9364	7.4888	6.0619	7.5544	7.6887	
7.4074	7.6336	8.4585	6.1788	5.9865	6.9457	6.9479	5.6415	
6.7141	5.9379	6.6776	7.4984	6.7856	7.1464	5.845	5.4345	
6.3998	5.9414	10.4556	8.0704	6.4735	6.1355	7.4632	6.2542	
6.585	7.851	4.4852	6.833	7.2109	6.4909	5.7177	6.7909	
6.5139	6.1938	7.6131	6.3407	7.1849	6.7839	7.333	6.6493	
7.2469	5.6119	10.4207	5.3768	7.2536	5.884	6.7726	8.2402	
7.2209	6.851	6.4815	6.7592	7.4424	6.2417	6.7682	6.3377	
6.5619	6.5985	8.2473	6.0313	6.142	7.3395	6.6754	6.5186	
6.7899	6.9508							
AKR1B10	0.0972	0.0409	0.057	1.0123	0.0693	1.4856	1.9076	

0.7301	0.0309	0	0.0384	0.9336	0.1373	0.2372	0.4773	
0.344	0.1022	0.0502	0.5709	1.577	0	0.4251	3.3603	
2.056	0.6058	0.1887	0.0858	0.0622	0.3716	0	0	
0.1804	0.1627	0.2026	2.7571	0	0.22	0.1683	0	
0.3115	0.0329	0.9794	0.2457	0.2822	0.0776	0.709	0.1387	
0.1651	3.75	0.2013	0.6916	0.1578	0.065	0	0.2308	
0.432	0.0652	0.4515	0.1603	0.7187	0.7705	0.2815	0.0295	
0.7572	0	3.2062	0	0.5823	0	0.7187	1.4669	
0.0838	0.0799	0	0.1741	0.0038	0	1.635	0.0359	
0.0975	0	0.5453	1.2806	0.1692	0.7026	0.102	0.1476	
0.0806	0.0895							
AKR1B15	0.0389	0	0	0.0877	0	0.0318	0	
0.063	0.0613	0.0537	0.0575	0.1706	0.0978	0.063	0.0268	
0.0422	0.1167	0.1554	0	0.0359	0	0.0694	0.3261	
0.0445	0.021	0.1239	0.0394	0.0409	0	0.037	0.0117	
0.0779	0	0	0.0818	0	0.0635	0.0529	0.0115	
0.233	0	0.4348	0.1599	0.0436	0	0.0208	0.0509	
0.0457	0.3498	0.0571	0.1611	0	0.0428	0.0862	0	0
	0.0474	0.2274	0.1117	0.1405	0.1646	0.0973	0.1036	
0.1008	0	0.0418	0.0647	0.0248	0.0186	0.1252	0.0676	
0.0707	0.0333	0.033	0.0254	0	0.0094	0.0875	0.0404	0
	0.0644	0.273	0.0217	0	0.0628	0	0.028	0
	0.038							
AKR1C1	0	0	0.101	0.1235	0.5604	0.1983	1.555	0
	0.0483	0.15	0.2173	2.6038	2.1368	0.3531	0.1815	
0.1506	0.4167	0.4167	0.0586	0.394	0	1.5292	2.6662	
0.3592	3.1367	1.9925	4.8603	0	0.1493	0.3034	0.1255	
0.7454	1.154	1.4102	1.1806	0.0639	0.8781	0.2006	0.0641	
0.6501	0.1706	6.6905	0.0994	0.413	0.0872	2.6129	0	
0.2471	3.7249	1.0123	0.6814	4.8568	0.0462	0	0.7559	0
	5.3706	0	0.0867	0	3.1909	0.952	0.2916	
0.6804	0	3.9121	0	0.2897	0	0.4387	0.9476	
0.1205	0.1922	0.4269	0.2319	0	0.3821	3.5507	0.196	
0.1068	0	1.2352	0.5325	0.0556	2.5922	0.2853	1.1897	
0.3494	0.2919							
AKR1C2	0	0.0133	0	0.0334	2.4275	0.0462	0.9768	0
	0.0011	0.0046	0.0723	0.8921	0.2106	0.2924	0.0447	
0.0771	0.1463	0.1256	0.0108	0.2861	0	0.4488	2.1686	
0.3995	2.9843	1.0239	4.9359	0	0.1567	0.0375	0	
0.1853	1.4904	1.6785	0.3183	0	0.5604	0.1727	0	
0.8231	0.1956	2.067	0.0079	0.0555	0	0.7629	0	
0.0866	0.6076	0.737	0.8444	2.9445	0	0.1754	0.2447	0
	3.2804	0	0.1554	0	0.9884	0.3278	0.1148	
0.2798	0.1212	0.9455	0.1549	0.1323	0.074	0	1.2138	
0.0777	0.0974	0.1231	0.2231	0	0.0805	1.8725	0.1396	
0.096	0	0.7848	0.1994	0.1659	0.7636	0	1.1734	
0.3148	0.0375							
AKR1C3	0	0	0.0552	0.8117	3.1642	3.268	3.8032	0
	0	0	0.2501	4.7342	1.9258	0.5209	1.335	
1.5768	2.1847	0.1421	0.0338	1.7743	0.0026	1.8303	5.1741	

3.1311	3.0055	2.2367	5.1945	0	2.6436	0.0162	0.5262	
1.2866	0.4047	2.7708	3.5487	0.0337	0.4709	1.4352	0	
1.8635	0.2342	6.4007	1.2064	1.7232	0.0348	4.3129	0	
0.207	3.2451	0.9474	1.6416	3.9768	0	0.1325	1.5579	0
	5.3921	0	0	0	1.6539	0.7676	0.8082	
0.5424	0	5.7287	0.1777	0.2928	0.1689	0.3419	3.1567	
0.8289	1.3656	1.4057	2.8874	0.1539	0.3965	4.3894	1.0116	
0.8337	0.2992	2.4709	1.2075	0.8514	2.6209	0.3603	0.8477	
0.0118	0							
AKR1C4	0	0	0	0	0.0847	0	0	0
	0	0	0	0.011	0	0	0	0
	0	0	0	0	0	0	0	0
	0	0	0	0	0	0	0	0
	0	0	0	0	0.0363	0	0	0
	0	0.0066	0	0	0	0	0	0
	0	0	0	0.0834	0	0	0	0
	0.0729	0	0	0	0	0	0	0
	0	0	0	0	0	0	0	0
	0	0.0064	0	0	0	0	0	0
	0	0	0.0387	0	0	0	0	0
	0							
AKR1D1	0	0.0474	0.0409	0	0.1106	0.0317	0.0067	
0.0101	0.0108	0.0145	0.0289	0.003	0	0.0545	0.0159	
0.0536	0.2158	0.0768	0.0511	0.1356	0.0743	0.0012	0	
0.0286	0.0045	0.0404	0	0.0769	0	0.0169	0.046	
0.2692	0.0468	0.1343	0.0316	0.0088	0.0519	0.0299	0.0251	0
	0.0343	0.0361	0.0748	0.0192	0.0061	0.1532	0.0272	
0.0481	0	0.0993	0.0325	0.0544	0.0437	0.0082	0.041	0
	0.0254	0.0407	0.05	0.038	0.043	0.2133	0.0498	
0.1247	0	0.0561	0.0619	0.0334	0.0263	0	0.0124	0
	0.1398	0.0229	0.0149	0.3182	0.1051	0	0.0078	
0.0294	0.0485	0.0228	0	0.0356	0	0.035	0	0
	0							
AKR1E2	0	0	1.6866	1.0052	0	1.395	1.062	
0.3248	0.6633	1.6859	0.9875	1.8255	2.3394	1.9433	1.0052	0
	1.6639	0.8394	0.9535	1.5362	0	1.6168	1.9081	
0.2652	0.2038	0.9619	1.7208	0	1.777	1.3498	0.3597	
0.6518	1.9518	2.082	2.082	1.1776	1.1265	0.4388	1.1892	
2.1294	0.9952	1.7023	1.3081	2.6503	0.4765	0	1.9801	
1.249	0	3.2785	2.088	0	1.3559	0	0.829	0
	0	0	0	0	2.2779	0.2659	1.6874	
2.0684	0	0.2579	0	1.0367	0.3997	0.6481	2.4312	
2.149	0	1.6764	0.3341	0	2.3621	0.4847	1.2857	
0.5627	0.5282	1.1034	2.3239	0	1.3956	2.0569	0.7275	
0.8692	0.4971							
AKR7A2	4.7134	4.9711	4.4394	3.3817	4.2364	3.204	3.6157	
3.8655	5.1891	4.727	4.4346	3.5265	3.5438	3.8773	4.2079	
3.6731	3.531	4.3042	3.9971	4.1769	4.922	4.2635	4.9932	
4.5646	3.9258	3.3846	3.7468	4.2234	4.6416	4.3741	4.7018	
4.4706	5.6462	4.6758	4.6649	4.1874	3.7936	4.411	4.1928	

4.1525	4.643	3.9118	4.0251	3.8399	4.023	3.7522	3.7438
4.5614	4.0016	3.3012	4.2203	3.8341	4.1191	3.8746	4.2312
4.393	4.2385	4.3896	4.0463	4.3523	4.4136	5.5894	4.3836
4.4063	3.6826	3.5374	4.053	3.936	4.8242	4.1662	3.7052
4.6367	4.3226	4.1873	3.4957	4.2285	4.7614	3.7006	4.8601
5.0763	5.0656	4.1418	3.6481	3.3752	4.3375	4.7309	4.2604
4.6237	4.6426						
AKR7A3	1.6765	0.3507	0.1124	0	0.0978	0.2131	0.1708
0.3984	0.171	1.5	0.5936	0.1278	0.1294	1.4022	0.3761
0.1854	0.3316	0.5125	0.3225	0.1335	0.9757	0.3037	0.0759
0.5166	0.1679	0.2641	0.2854	0.2729	0.118	0.4716	0.4208
0.2926	1.0397	0.4316	0.047	0.5316	0.2422	0.3272	0.436
0.33	0.716	0.0914	0.2194	0.4467	0.2801	0.2029	0.8669
0.3592	0.208	0.529	0.5196	0.5602	0.2313	0.4153	0.2541
0.1904	0.4244	0.5741	0.2861	0.3769	0.1101	0.6966	0.4273
0.7732	0.395	0.0511	0.7349	0.1872	1.2646	0.195	0.1982
0.4375	0.0444	0.0634	0.1169	0.0208	1.1165	0.0775	0.3756
0.7928	0.6866	0.4533	0.5303	0.083	0.2557	0.6231	0.1473
0.4192	0.2591						
AKR7L	0.3689	0.2115	0.2019	0.1187	0.1217	0.0697	0.118
0.0824	0.1052	0.1067	0.0717	0.0162	0.0264	0.1382	0.1258
0.0494	0.0448	0.0928	0.1037	0.1707	0.0699	0.0493	0.0284
0.1136	0.02	0.0622	0.0436	0.1145	0.0371	0.1786	0.1078
0.0427	0.024	0.07	0.0073	0.03	0.1362	0.0576	0
0.2117	0.1186	0.0745	0.0621	0.0224	0.1131	0.0382	0.0315
0.164	0.1248	0.538	0.1021	0.0446	0	0.0318	0.1769
0.1514	0.0314	0.0738	0.0259	0.2268	0.1252	0.1593	0.0217
0.1473	0.1289	0.029	0.0445	0.0193	0.1961	0.0511	0.0405
0.0653	0.0268	0.0126	0	0.0276	0.5363	0.0437	0.1165
0.1146	0.0614	0.0294	0.0195	0.1454	0.1594	0.0514	0
0.0881	0						
AKT1	6.7938	5.3935	6.0313	5.0066	5.3553	6.5029	6.0087
6.6782	5.4002	5.9779	5.9789	5.5857	6.0164	5.2077	6.136
6.2367	5.6192	5.7607	5.699	5.5707	7.1005	6.2817	6.7845
5.8274	6.0951	5.7007	5.0269	6.7663	6.3206	5.6873	5.9109
6.0852	5.7439	6.1948	5.5543	5.0879	6.0227	5.9857	6.2869
5.3592	6.6093	5.7456	6.1018	6.5125	6.1383	5.8439	5.0388
5.9439	6.6954	4.7818	5.2968	5.4853	5.2011	5.8106	5.2887
6.3525	5.4823	5.2375	5.8285	5.4136	6.1761	5.7881	6.195
6.0681	5.9003	5.5341	6.6894	6.6641	5.8657	6.6041	6.5938
6.251	6.6066	5.6264	7.2577	4.3621	6.0065	5.96	6.422
6.2627	6.2714	6.3525	5.2132	6.2201	6.1312	6.0331	6.408
7.065	6.7474						
AKT1S1	4.3633	5.7838	5.3817	5.3521	6.1756	5.809	5.2537
5.7821	4.9895	4.6559	5.4358	5.3863	5.2951	5.0567	5.8026
5.1843	5.053	3.8987	4.7901	5.8134	5.5507	5.7927	5.9224
5.4196	5.3615	6.3443	5.4638	5.0418	5.7637	5.0486	5.2286
5.6669	5.8407	5.1202	5.392	4.6604	5.3646	5.575	5.3634
5.4559	5.6669	5.8108	5.2438	4.451	5.0937	5.4118	4.0636
5.2018	4.8449	4.1339	4.8731	4.905	4.7021	5.8234	5.5504

4.9978	5.3118	4.8501	5.1648	5.3989	6.3607	5.8942	5.4686
5.5174	4.9834	5.322	3.8699	5.6042	5.3779	4.8085	5.8796
6.0207	5.1707	5.767	4.7492	5.7684	4.7226	5.098	5.2559
5.4646	4.8809	5.9037	5.4699	5.1581	5.4666	5.0452	5.068
4.9549	4.9911						
AKT2	4.8387	5.2265	5.1172	4.0129	5.0443	4.4648	4.0707
4.628	4.3537	4.6484	5.3521	4.7116	4.5568	3.61	4.6133
4.4921	4.8184	4.2433	4.3296	4.4822	4.8357	4.7462	4.9814
4.3138	4.3419	5.0606	4.3047	4.9108	4.7434	5.0212	5.4514
4.7487	5.0126	5.0649	4.4286	3.7387	4.424	5.3158	4.9292
4.4676	5.2082	4.7678	4.3774	4.0047	4.8412	3.7842	3.5083
4.5306	4.5754	4.4464	4.2716	4.0125	4.213	4.768	4.1621
5.0436	4.8044	4.363	4.688	4.384	4.7874	5.0554	4.9347
4.8386	4.546	4.6375	4.0703	4.5097	5.4802	4.2025	5.0313
4.7897	4.4539	4.8861	3.8154	4.9918	4.501	4.0226	4.9106
4.5717	4.9198	5.0304	4.0896	4.0624	4.4611	4.927	5.1351
5.1126	5.3266						
AKT3	4.5472	3.5084	4.1643	4.6589	5.1433	3.697	5.3725
3.3485	4.671	4.0623	4.1154	5.0351	4.2675	4.4413	4.8653
4.8786	4.8174	4.9914	3.424	4.0031	4.3607	4.563	4.6746
4.4157	4.8963	3.456	6.43	4.454	3.9699	4.9472	3.1319
4.4918	3.6423	4.706	4.6999	3.993	5.1982	5.4153	5.7798
4.822	5.1618	3.9371	5.14	4.4385	4.4135	3.4996	4.1442
5.7476	5.913	4.8073	4.6388	4.8467	4.9292	4.7024	1.2779
4.2865	5.3157	4.3606	5.0297	4.3512	4.6239	4.2973	4.9926
4.533	4.6062	4.7024	4.6712	3.9482	4.6243	4.4018	4.8092
5.0431	4.1585	5.3185	5.4479	4.4798	4.6833	5.5887	5.0993
3.8473	4.2665	4.5171	4.2732	5.7693	4.29	4.4722	4.6076
4.2005	4.7468						
AKTIP	2.6554	3.2597	2.5802	3.2189	3.033	3.0226	2.4174
3.1369	3.3452	3.4387	3.083	3.2985	3.8187	3.9596	3.3172
3.1783	3.5004	3.0174	3.1912	3.3472	3.2675	2.8362	3.0711
3.246	2.5693	3.3878	3.734	3.6413	2.1137	3.7533	3.3136
3.5621	2.1847	3.3978	3.3285	3.4177	3.0148	2.4242	3.0185
2.3785	3.11	3.0853	2.9037	2.1599	3.8033	3.1762	2.9057
3.1844	2.5471	3.7852	3.0759	3.3124	2.9688	2.9299	3.6229
1.9455	3.4611	2.2887	3.5862	2.5439	2.8669	3.1854	2.8706
3.019	2.2131	3.642	2.9581	3.7299	3.463	2.6448	3.2336
3.4195	2.8643	2.7049	3.5983	4.0113	2.9624	3.3604	3.0283
2.5697	2.751	3.1766	3.1672	2.6892	3.1882	3.1828	2.995
2.5416	2.8474						
ALAD	2.472	2.8054	2.5064	2.7398	3.1957	3.4086	1.9488
3.1497	2.3326	2.7988	3.6469	3.0398	3.2774	3.0939	3.2815
3.0918	3.4456	3.2188	2.6836	4.4037	3.3692	2.5649	2.9683
2.937	3.0637	2.5612	3.0343	2.8742	3.4305	2.2329	2.6489
3.5853	3.0049	3.8535	3.2275	2.6545	2.6607	4.0815	3.8933
3.3016	3.7765	3.4641	3.0683	3.2883	1.8922	2.5625	2.1849
2.4245	3.1918	2.5649	3.3635	2.9283	3.4641	1.8587	2.5069
3.0439	3.6845	2.6306	3.3916	2.4561	3.8427	2.759	1.9707
3.5573	3.2044	3.6771	3.309	3.5555	3.1975	4.1999	3.239

2.97	3.7758	3.6409	3.5861	2.487	3.0338	4.04	2.6814	
3.2978	3.0361	3.4583	2.8299	4.2141	2.3703	3.6013	4.6975	
4.2449	4.7092							
ALAS1	5.0064	5.6616	4.8379	4.7757	5.0715	4.9438	5.1965	
5.2802	5.4579	5.8251	5.6955	5.1978	4.9235	4.7422	4.3573	
6.2009	5.0623	5.2268	5.4688	5.3685	4.6927	4.7192	4.9258	
5.2508	4.6058	4.5245	4.5652	5.0481	4.8033	5.3333	5.4143	
4.6867	4.8295	5.0179	4.8779	5.8241	5.0076	4.2303	5.067	
4.7628	4.7454	5.0895	4.3628	5.1903	4.0474	4.1058	5.771	
5.0755	4.6698	5.0119	5.1538	4.8017	5.2946	4.0705	5.8411	
4.3382	4.8978	4.3691	5.6613	4.8627	5.0168	5.0563	4.9585	
4.9308	4.4371	5.3752	5.383	5.6294	5.4969	4.5261	4.8142	
4.7524	5.4716	5.2623	4.9183	5.3553	5.14	4.9761	4.2225	
4.9451	4.8983	4.4773	4.7815	4.6869	4.8344	5.0474	5.6504	
5.4042	5.0398							
ALAS2	0	0	0	0	0	0	0.0333	0
	0.0153	0.0195	0	0	0.0118	0	0	
0.0304	0.0444	0.0363	0	0	0	0	0.0054	0
	0	0.0329	0	0	0	0.0624	0	
0.0013	0	0	0	0.0157	0	0	0	0
	0.0173	0	0.0349	0.0511	0.0124	0	0	0
	0	0.1298	0	0	0	0	0	0
	0	0	0	0	0	0	0	0
	0	0	0	0	0.0614	0	0	0
	0	0	0	0	0	0	0.0557	0
	0.0345	0	0	0	0	0	0	0
	0							
ALB	0.0373	0.4035	0.0891	0	0	0	0.0138	0
	0.055	0	0	0	0.0553	0	0.0117	
0.014	0.0378	0	0	0	0.0458	0	0	0
	0.0282	0.0343	0.0231	0	0	0	0.0123	
0.0018	0	0	0.0035	0	0.0107	0	0.0379	
0.3615	0	0.0029	0.0356	0.022	0.0338	0	0	0
	0.0226	0.0647	0	0.1173	0	0.0183	0	0
	0	0	0	0.0356	0.0712	0	0	0
	0	0.0743	0	0	0.1974	0.1392	0	0
	0	0	0.055	0.1504	0.0134	0	0	
0.071	0.0296	0	0	0	0	0.078	0	0
	0							
ALCAM	3.8511	0.7532	2.7038	3.6172	3.413	2.9463	1.3209	
2.1115	3.091	2.5386	2.7891	4.8364	5.4379	3.3318	5.7365	
3.6853	7.2661	3.1476	0.8022	4.8527	4.2932	3.9695	4.9269	
1.5575	1.8001	5.7345	4.5755	2.8327	5.2507	3.1946	1.7255	
5.6666	3.1192	4.196	6.1236	3.0763	4.9249	0	2.8838	
4.0012	3.9504	3.8185	2.5457	2.0751	5.4264	4.8186	3.7995	
4.5387	3.8036	5.2687	2.1048	1.3946	2.7572	3.1242	3.8762	
1.8896	6.1891	2.2832	3.8647	3.0087	4.8143	2.1237	3.6005	
3.6708	3.4477	7.1206	2.6418	1.8439	3.624	3.4803	4.6937	
3.8187	1.5926	3.5576	1.4427	4.7038	3.9206	3.0809	3.5631	
0.493	0.4379	5.9183	4.7712	5.2456	4.7765	3.5668	3.8768	

2.9237	4.08							
ALDH16A1	2.2669	3.1331	2.9805	1.9484	3.2305	2.0262	1.8532	
2.4007	2.5771	1.882	1.9266	1.723	1.672	2.7847	2.5639	
1.9624	2.3053	2.4569	2.2199	2.3685	3.0022	2.7603	2.4394	
2.4832	1.8058	1.8645	1.8494	2.6045	2.4523	1.6809	1.4355	
3.3598	2.62	2.3895	1.5613	1.6511	2.5964	3.4059	2.4137	
1.785	2.6743	1.9314	1.9345	2.3207	2.4342	2.2565	1.8197	
1.8393	2.6067	2.0801	2.2772	2.666	2.6727	3.2283	2.2254	
2.6924	1.933	2.5138	1.7244	2.2724	3.6274	3.0189	2.976	
2.9834	2.7562	1.9646	2.2595	2.8459	2.5887	2.2241	2.4163	
2.9193	2.1502	2.4195	1.647	1.902	3.0028	2.03	2.6906	
2.4781	3.1565	2.3958	2.4854	2.4947	3.6258	3.5367	2.2361	
3.4096	3.4448							
ALDH18A1	4.9894	5.0523	4.723	5.0478	5.4724	5.6585	4.269	
5.8978	4.8469	5.0911	5.3124	5.4223	4.5723	5.2555	4.876	
5.0831	4.2636	5.0323	4.6207	5.7028	5.0016	5.0412	4.7141	
5.2504	4.6092	4.5048	5.4816	4.6344	4.657	4.8867	4.1001	
4.8504	4.4639	5.2434	5.1663	4.8373	5.2396	4.528	4.916	
5.1572	4.4712	3.8206	4.9326	5.1579	5.0567	5.0374	5.2562	
4.6217	4.6753	4.9034	5.2685	4.8119	5.0831	4.9254	5.35	
4.7352	5.1464	4.9928	4.4557	5.0454	4.6462	4.8296	5.4477	
5.9429	4.9654	4.6978	4.8417	4.397	4.5308	5.2914	4.8301	
5.0114	3.9972	4.8371	4.5312	3.5963	4.8945	5.1218	4.4486	
5.5626	4.6216	5.4499	4.2934	5.2028	4.3347	4.8644	3.7586	
4.5121	4.1809							
ALDH1A1	0.898	2.7852	0.8956	2.1572	0.2429	7.0267	1.5872	
3.0924	4.9591	0.5978	0.7989	4.8422	0.0851	3.2057	0.9576	
3.9717	0.1579	3.2528	1.8535	1.1846	1.5158	3.5214	2.7209	
5.0783	0.178	0.6085	3.3378	1.1417	2.2482	2.2282	9.8544	
3.5307	5.2566	5.7337	6.0178	6.2726	1.6585	6.4547	2.092	
3.4972	6.5445	2.8195	0.3455	3.0499	0.4991	0.0925	2.4131	
2.9211	1.0689	0.3039	2.0243	4.4676	3.4412	2.3256	4.9677	
1.4592	0.3333	0	6.2378	0	2.9597	2.3804	0.217	
3.6204	0.7026	0.6297	2.1952	0	1.939	1.3487	7.3569	
2.1794	3.5804	1.7454	3.4841	0.9411	1.7072	5.5789	3.7135	
2.2334	1.393	1.712	0.252	6.555	4.1768	4.1003	0.8968	
1.3105	0.4999							
ALDH1A2	0.1503	0.6255	0.1626	0	0.0094	0.024	0.2571	0
	0.4945	0.0322	0.1678	0.1715	0.038	0	0.6591	
0.0618	0.0293	0.0506	0.0572	0.027	0.1719	0.0177	0.8942	0
	0.1211	0	0.0154	0.0316	0.0137	1.6601	0.0678	
0.0652	0.1776	1.5002	0.0855	0.0256	0.0491	2.582	0.5597	
0.0521	0.1486	0.0065	0.2241	0	0.0073	0.1855	0.0226	
0.1167	0.0372	0	0.2541	0.2505	0.0669	0.3627	0.0188	
1.5309	0.1616	0.0174	0.0129	0	0.0055	0	0.0325	
0.0242	0.1803	0.1178	0.112	0.0137	0.1067	0.0481	0.002	
0.495	0.1197	0.0354	0.3778	0.1287	0.5951	0.8623	0.3075	0
	0.1323	0	0.0638	0.3231	0.2438	0.0448	0.1177	
0.1301	0.4385							
ALDH1A3	5.0525	0	0.8174	3.998	6.9428	4.2691	4.4269	

2.2501	2.8637	4.3114	0.4731	5.0896	2.7311	4.4828	6.794	
2.3876	4.8299	6.046	2.7926	4.5592	1.6067	5.3542	5.8599	
5.1563	8.1706	5.5204	3.5076	5.1171	6.6683	3.5482	1.1126	
2.4795	5.1907	1.4491	7.2162	3.0564	5.5698	0.8455	9.0231	
0.9523	5.3909	6.5019	7.0928	4.9701	7.6151	2.0972	3.6444	
8.4434	8.868	0.623	3.7713	5.2575	5.4281	1.6312	2.1995	
0.5743	4.9909	0.6349	4.9763	0.5931	6.6219	1.1852	4.4481	
5.5585	1.3337	1.7791	0.5346	4.4418	6.5115	5.4259	5.6976	
5.7555	2.163	6.4304	8.7569	9.0275	1.2041	4.3297	5.1781	
4.735	2.1444	7.259	7.6854	6.4673	6.5184	4.6475	1.2536	
3.024	2.3815							
ALDH1B1	5.268	4.5047	4.0946	3.171	3.0921	3.9977	2.9646	
5.3682	4.6614	4.7562	4.4709	4.1116	3.171	4.1505	3.2375	
4.496	3.6451	3.5591	4.2025	4.34	3.3547	1.924	2.9842	
4.6311	2.736	2.2486	2.0049	2.3991	3.3038	3.5311	2.888	
4.3818	3.1949	4.1893	1.0782	4.3548	2.6351	4.254	3.99	
3.9836	5.2943	3.0109	3.653	5.284	3.7835	2.1331	4.2919	
3.8193	1.6911	3.2218	4.2883	2.3813	4.2619	3.6162	3.7229	
3.7618	3.5871	3.5649	5.2317	3.3011	4.3245	4.6671	5.57	
2.4744	3.9945	4.388	3.2064	2.3841	2.5818	2.8432	4.2032	
2.6164	3.2737	3.0376	2.7405	3.1898	3.614	3.9991	5.8131	
5.4192	4.7463	3.3862	3.2085	2.9531	2.2457	4.5572	2.4406	
3.2405	3.3756							
ALDH1L1	0	0.1064	0.0624	0	0.012	0.2987	0.1912	
0.1079	0.4311	0.7822	0.1956	0.8653	0.0036	0.327	0.0029	
0.0453	1.5566	0.6642	0.3793	2.1105	0.0375	0.0994	0.1813	
0.0048	0.2068	0.0548	0.7633	0.0345	0.0614	0.1469	0	
2.0717	0.0335	0.0927	0.0668	0.1049	0	0.0106	1.243	
0.0132	0.2076	0.0826	0.0297	0.2417	0.0667	0.0206	0.027	
0.1378	0.1609	0.0568	0.0517	0.0993	0.0525	0.0401	0.0394	0
	0.157	0.0391	0.0512	0.036	0.2484	0.2726	0.067	
0.2188	0.0748	0.1671	0.069	0.0231	0.1022	0.0629	0.0592	
0.232	0.1061	2.0348	0.6401	0.9403	0.0576	0.1922	0.0622	
0.2599	0.1288	0.2167	1.1613	0.2221	0.1811	0.0682	0.0236	
0.0986	0.2482							
ALDH1L2	1.6925	0.0217	2.1967	0.013	1.5063	0.1219	0.028	
0.0315	2.1038	3.637	0.6741	0.8622	0.7211	2.8967	0.1092	
2.3762	1.1889	0.702	0.2924	1.0926	0.0256	0.8343	0.6301	0
	0.877	2.3243	0.4214	0.1792	0.5771	1.1475	3.6671	
0.1921	2.5749	3.4884	0.4476	2.1887	3.7002	0.0342	3.2136	
2.5891	2.7184	1.4904	3.0759	1.1641	0.1925	0.1417	2.6437	
0.1413	0.0773	2.0977	3.3816	0.2703	2.3186	3.9007	3.9568	
2.5775	0.1685	2.4513	2.0349	1.2781	1.6709	0.0818	0.1911	
0.8092	2.2109	0.2725	1.1921	4.6073	0.2241	0.146	2.5653	
0.5953	1.3913	0.9914	0.5985	0.1102	1.1715	0.0979	1.649	
0.1865	0.3903	1.0337	2.1219	0.2339	0.4738	2.2849	4.5306	
3.5479	3.7999							
ALDH2	3.9329	5.4463	0.1786	0.1336	3.5475	0.0892	0.1551	0
	6.0982	4.8105	0.5187	0.1544	0.1008	0.3257	0.1899	
0.1029	0.1499	5.6123	0	0.2412	0	0.0877	0.2592	

0.1255	4.3874	0.58	5.5083	0.7757	0.0903	0.5953	4.9681	
0.0725	0.1338	5.5307	0.1736	4.1423	0.1591	0.213	5.1899	
0.0688	0.8498	0.2065	0.3398	0	0.234	0.5748	0.0869	
0.2973	0.3947	0.4317	0.1159	2.0026	0.1918	5.4559	5.5965	
0.2184	2.8339	4.4803	1.9942	4.2169	0	0.1057	3.494	
0.134	0.6834	4.825	2.1335	6.0827	0.0094	0.0246	1.7965	
0.0651	0.8595	0	0	0.0114	0.3197	0	4.5592	
0.3263	0.1207	0.008	0.0808	2.3439	0.0011	0.2707	1.6504	
2.7742	4.538							
ALDH3A1	0.0483	0.1982	0.2341	0	0	0.0358	0	
0.0325	0.2567	0.1118	0.095	0.1052	0.2168	0.0637	0	
0.1536	0	0.0969	0.1904	0.2105	0.1825	0	0.9808	
0.0663	0.099	0.0891	0.1146	0.6633	0.0499	0.0228	0	
0.216	0.0535	0.1743	0	0.0323	0.2028	0.0183	0.0517	
0.0111	0.2485	0.1284	0.1676	0.3307	0.026	0.5864	0.0921	0
	0.0601	0	0.2599	0.0155	0.1303	0.0472	0.0874	
0.1713	0.0611	0.321	0.0433	0.5169	0.04	0.0309	0.1013	
0.0861	0.1784	1.5358	0.1241	0.0425	0.0585	0.1014	0.0337	
0.0919	0.1167	0.0503	0.0565	0.07	0.0271	0.0549	0.055	
0.0526	0.0533	0.3123	0.1343	0	0.0493	0.1087	0.1029	
0.0664	0.0423							
ALDH3A2	3.1807	3.3204	3.2766	4.1587	4.184	4.7925	5.4035	
3.0535	4.7678	3.3141	2.9741	3.4667	4.0722	3.8509	4.1774	
4.7925	3.4992	4.0523	3.3056	3.5238	3.7603	3.5416	5.4338	
4.1759	5.1688	2.9057	4.9975	3.3199	4.1378	3.4055	5.5168	
5.1614	3.4928	2.9826	4.9928	4.2527	2.6303	3.6456	3.3384	
4.5701	3.5148	4.7129	4.7353	4.778	4.2587	4.0513	3.6039	
4.1029	5.6789	3.1763	5.2025	4.6087	3.6865	2.9619	5.2245	
3.5701	4.2739	3.8228	3.1729	3.8062	4.0515	4.2526	5.4508	
3.9203	3.502	5.7639	3.19	3.2633	3.1038	4.9199	4.3436	
4.1115	3.5941	4.6916	4.6008	3.2833	3.5278	5.0576	3.763	
3.8373	4.9534	4.9888	3.447	0.769	4.0383	3.8626	3.6565	
3.6092	3.9531							
ALDH3B1	2.8845	0.8368	1.511	1.7614	2.5069	1.6835	1.1276	
1.8809	1.4868	1.789	2.011	1.7865	2.0386	2.1104	0.8502	
0.8304	2.3114	1.4621	1.2862	2.2075	2.9305	1.7574	3.0584	
1.0018	1.2188	1.4324	1.4089	0.7856	2.5245	0.4843	0.1494	
0.9976	0.471	2.2261	2.3571	0.8966	2.9075	2.8271	3.3723	
1.5023	2.5802	3.4336	2.3039	2.0828	2.1264	2.9897	1.3669	
0.9958	1.36	2.3357	1.9032	1.0404	1.0492	0.3843	2.0834	
1.5905	3.1801	1.6924	1.7727	1.6075	2.4258	1.2227	1.4963	
0.8734	2.7904	3.7877	2.2207	2.3028	0.9342	3.4995	2.0598	
1.5566	1.6559	0.9762	1.6844	0.3098	2.1656	2.0688	1.0684	
1.2313	0.5147	2.7546	1.8903	2.082	2.8192	1.6034	3.1642	
2.4195	2.8818							
ALDH3B2	1.127	0.1822	0.0905	0	0	0.3381	0.0716	
0.0473	2.1554	0.2413	0.611	0.772	0.0223	0.138	0.0661	
0.5964	0	0.1592	1.2557	1.2753	0.0468	0.0166	0.085	
0.1242	0.0636	0.0266	0.1341	0.8394	0	0	0	
1.6189	0.1041	0.3442	0.0374	0.0497	0.1304	0	0.9371	

0.0982	0.0668	0	0	3.8129	0	0	0.1954
0.0643	0	0	0.0524	0	0	0.4395	0.066
0.259	0.1057	0.0679	1.7911	0.0601	0.2458	0.0512	0.0349
0.1411	0.012	0.2187	0	0.0097	0.0896	0	0.1352
3.0047	0.7261	0	0.0943	0.0392	0.2966	0.11	0
0.0557	0.1228	0.7625	1.8611	0.0956	0	0	0.0667
0.045	0.1392						
ALDH4A1	3.0811	3.7598	3.3609	1.896	2.4359	3.2136	2.2929
2.8802	3.3629	3.0729	2.9194	2.2748	1.6954	1.7719	2.416
4.0329	1.5256	3.6058	2.5885	2.7058	3.017	2.6417	3.5126
2.2256	2.3858	2.1512	1.5108	2.8119	2.9585	2.7424	4.1184
2.9838	2.7314	2.0551	3.4144	2.4531	3.3796	4.2555	4.5462
2.3049	3.3613	1.853	1.729	2.3072	2.4816	1.4801	2.2833
4.0367	2.0442	2.1314	3.0612	1.1075	2.5592	1.9477	2.9709
2.7741	1.5386	2.8218	2.8676	2.5135	2.6519	4.7043	2.1594
3.0985	2.0432	1.0533	3.7085	2.5321	2.9192	1.7897	2.3393
2.585	2.8131	3.6096	2.7517	4.1572	3.1743	3.1723	3.7619
2.3464	3.7269	3.5782	2.6688	2.5667	3.7077	2.4276	4.0667
4.0713	4.2622						
ALDH5A1	0.1712	0.5834	2.4794	1.5131	1.7261	0.6542	0.3285
1.9339	2.1016	1.9285	1.8888	1.9689	1.4649	1.766	0.542
1.3575	0.0013	3.3886	2.253	0.2101	0.9317	1.5198	1.5711
0.5675	0.9577	0.6206	2.9119	2.9287	0.8324	1.8797	2.5856
2.0085	0.6517	1.4287	0.5415	2.1132	4.0564	2.7933	1.5773
2.0468	1.396	2.4142	0.9245	2.5238	1.527	2.095	2.5294
1.8198	0.1671	0.5927	1.4982	1.7542	1.7287	1.367	2.7578
0.0924	1.5758	1.8788	0.4607	1.9269	1.2277	2.2812	1.6113
0.87	2.9388	0.8609	0.1799	2.5245	2.9156	1.1487	1.5327
1.2899	0.4425	0.1695	1.3338	2.6712	2.4105	0.1717	2.5313
1.7068	2.1474	1.2954	2.5043	2.0277	0.6439	1.5772	0.5895
1.1692	0.8311						
ALDH6A1	3.1307	2.3467	1.6864	2.3331	1.4382	1.2301	1.5645
1.3044	2.0671	2.7177	3.1834	2.3024	1.8932	1.9138	1.6232
1.776	1.7661	3.5349	1.8822	2.2856	1.6472	2.0635	2.7734
1.0223	1.2785	2.3391	3.6302	2.5269	2.2966	2.9241	4.3738
2.1201	1.6849	2.7065	1.8917	2.3934	2.5344	3.388	2.8836
3.0932	3.6395	2.8874	1.8093	2.5352	2.7233	3.3175	2.5361
2.2516	2.2394	3.3953	2.4048	2.7537	2.7697	1.5193	2.5401
1.199	1.9166	2.1504	1.7591	2.0089	2.003	2.9342	1.8713
1.3603	2.6802	3.6641	2.4625	2.243	1.4378	1.8361	2.1885
1.7784	2.6821	2.0913	2.9568	1.5089	1.7975	3.2999	2.96
2.2506	4.563	2.8159	1.4095	2.9988	2.0078	2.6371	3.5959
3.071	3.4188						
ALDH7A1	3.6536	4.0984	3.7791	3.978	3.8787	4.5385	2.9938
3.0136	3.832	3.8388	3.8916	4.2312	3.5522	3.7957	3.5444
4.176	3.6707	3.6383	4.1977	4.9184	3.9597	3.4172	4.1752
3.925	3.1683	3.1109	4.3853	4.0866	3.8953	3.8463	3.9746
3.9813	4.0384	4.3594	3.1355	3.8758	3.5412	0	1.8355
4.6052	4.0892	4.4499	2.9428	3.4415	4.1348	0	3.7062
4.2454	3.853	3.9058	3.7055	3.6818	3.8649	3.4417	3.117

3.3741	3.6238	2.3341	3.8187	2.0578	4.405	3.8546	4.3676	
3.8566	1.9259	3.841	3.8558	3.765	3.1682	3.0994	4.1673	
4.0697	3.6561	4.1175	4.613	4.0887	4.0039	4.4258	3.3621	
0.4904	4.6061	4.4842	2.28	3.0466	4.6019	4.0515	3.9751	
4.3121	4.2527							
ALDH8A1	0.3091	0.0231	0.0374	0.062	0.0056	0.0627	0.2255	
0.0301	0.0465	0.0548	0.007	0.2103	0.1205	0.041	0.0728	0
	0.2675	0	0.092	0	0.0683	0.0183	0.0552	
0.0868	0.0343	0.1675	0.1115	0.042	0	0.039	0	
0.0024	0.0226	0.0618	0.0554	0.0292	0	0.0933	0.1127	
0.0947	0.2338	0.1341	0.032	0.0218	0.0102	0.0184	0.0543	0
	0.0357	0	0.0364	0.1076	0	0.0103	0.1127	
0.0618	0.1996	0.0552	0	0.0247	0.0675	0.0396	0	
0.0348	0.062	0.0678	0	0.2074	0.1671	0	0.0149	
0.0467	0.0228	0.1782	0.0993	0	0.0263	0.2319	0	0
	0.0974	0.0091	0.1707	0.1006	0.0353	0	0.1771	
0.0803	0.0911							
ALDH9A1	5.3904	6.295	4.9503	5.5725	5.2428	5.2275	4.9393	
4.6308	5.8325	6.0489	5.3881	4.7468	4.4934	4.7626	4.5982	
5.7859	4.3456	6.0579	5.861	3.655	5.1219	4.2807	4.7564	
5.3867	5.3867	4.8592	5.4947	5.9876	4.7115	5.6311	4.5624	
5.5268	4.817	5.6049	4.3724	5.8725	5.2372	6.0652	4.8286	
5.4711	5.9117	4.8514	4.9728	5.9106	5.0202	5.7431	5.5999	
4.811	3.6243	5.5929	4.6186	5.3888	5.9356	4.226	4.9161	
5.8384	5.6652	4.7933	5.5279	4.743	4.8712	4.8554	5.5756	
5.2723	5.6748	5.6421	5.3692	3.8732	6.3488	4.7729	5.324	
4.9934	4.7955	4.0684	5.9022	5.0491	5.6218	5.6764	6.235	
5.8743	5.8743	4.291	4.218	4.7648	4.7724	5.5239	5.6066	
5.7929	5.7078							
ALDOA	9.1861	9.5508	10.0687	8.4714	9.4984	9.8976	9.7108	
10.31	10.0843	8.7906	9.5647	8.4091	9.6529	8.2553	9.6221	
9.3687	9.0799	7.8367	8.958	10.356	9.869	10.1513	10.1513	
10.2893	9.0917	10.0061	8.7818	9.2343	9.4718	9.5647	9.7239	
9.5119	9.7679	10.1329	10.2008	8.0381	9.443	9.6381	9.3568	
8.5428	9.8572	9.3687	8.958	8.6768	8.2301	9.2712	8.5489	
9.7811	9.1154	7.7402	10.4204	8.2982	8.0424	8.3287	9.2306	
8.5878	8.3679	8.8835	10.2874	9.3072	9.5316	9.6098	9.8108	
9.6098	8.4516	10.0274	9.961	9.3286	9.5517	9.1203	9.8332	
9.7866	10.2129	9.2154	8.4789	10.1901	9.3673	9.4303	9.7257	
10.6506	9.4896	8.732	10.3792	9.3286	8.934	8.4351	9.7811	
9.594	9.594							
ALDOB	0	0.0216	0.04	0	0.094	0.0343	0.0075	0
	0.0303	0.0356	0.0033	0	0.0446	0	0.0569	0
	0	0.031	0	0	0	0.0012	0	0
	0.1277	0.078	0.0595	0	0	0	0.0102	0
	0.0481	0.0634	0.0351	0	0.0199	0	0.0277	
0.0334	0	0	0.0582	0	0.0382	0.0158	0.0296	
0.0517	0	0.0643	0	0	0.0471	0.0104	0.0436	
0.0819	0	0	0	0.021	0.0286	0	0.1039	0
	0	0.0047	0	0.0253	0	0	0	0

	0.0388	0	0	0.0221	0	0	0	0
	0	0.024	0.029	0.038	0.0925	0.1467	0.0298	
0.0659	0.043							
ALDOC	4.8798	4.6064	4.38	2.2692	4.7397	4.2609	0	
4.7601	5.5734	5.0369	2.7348	4.0914	0	4.2561	2.9508	
4.1755	3.7736	3.0118	3.9784	5.0567	4.3446	4.9851	2.5986	
4.0256	3.1457	1.0964	4.9944	1.056	3.7298	5.8721	4.0349	0
	0	3.699	0.279	3.9001	3.0247	4.2034	4.9159	
4.9706	6.2111	5.1356	0.0328	5.1106	2.466	0.2375	4.8601	
4.3236	3.2085	0.0573	2.6415	4.9491	3.9567	3.1457	2.6079	
5.2523	0.7727	2.4934	3.1269	1.3615	5.263	1.6127	4.037	
2.3426	0.8526	4.0685	4.6333	3.3059	0.8601	2.0939	1.5441	
5.2903	5.368	0.8568	4.8828	0.8548	1.1437	4.0331	5.2002	
6.1348	5.795	6.4107	3.9553	3.7167	1.0665	4.132	5.2769	
3.6652	3.978							
ALG1	2.914	3.8042	2.514	2.9262	3.0112	3.0743	3.0845	
3.9982	3.027	2.6851	2.4243	2.3016	3.012	2.8777	3.3336	
3.3718	3.2938	3.4522	3.3369	3.3772	3.4691	2.9334	3.1616	
3.7055	2.7918	3.2433	1.5106	3.1964	3.4934	2.2531	2.6229	
3.4855	3.7337	2.4723	3.6731	2.5777	3.3183	4.0637	2.7714	
2.8866	2.9262	2.6161	2.8297	2.9664	2.95	2.9314	3.3746	
3.3648	2.846	2.9637	3.2391	3.0568	2.744	3.0015	2.4525	
3.3143	2.3744	3.5421	2.9189	3.457	3.3918	2.7769	3.5123	
3.4658	3.4595	2.7609	3.1424	3.0161	3.4265	3.5863	3.5899	
2.7047	3.8714	2.353	3.1938	3.4781	2.8635	3.4913	3.4142	
2.209	2.6971	3.1908	3.3635	2.453	2.9664	2.6837	3.2066	
3.399	3.1145							
ALG10	1.3398	2.2474	1.9712	1.8357	1.9634	1.5509	1.233	
1.1368	1.6481	1.6973	1.5103	1.271	1.7853	1.8878	1.9907	
1.799	2.1045	1.9624	2.251	1.6292	1.3022	1.585	1.357	
1.8619	1.5455	0.9325	1.8485	1.8151	1.1605	1.1919	1.5479	
1.9458	2.6447	1.6245	1.7876	1.7925	1.5343	0.9257	0.9155	
1.9697	1.1005	0.9148	1.2376	1.9454	2.1862	2.6399	2.2581	
2.146	1.22	3.074	2.2747	2.0657	2.036	1.8017	1.73	
1.2446	2.5958	2.1675	1.6372	1.6435	1.5005	2.0462	2.0179	
2.1397	1.8366	0.8955	1.8495	0.8273	1.8218	1.3804	1.5293	
1.7848	2.0449	1.4683	1.4992	1.1566	1.9838	1.0977	1.8321	
1.8744	2.3733	0.8011	1.3707	0.9661	0.9286	2.0838	1.1789	
1.7046	1.5153							
ALG10B	1.5495	1.5179	1.435	1.6157	1.5864	1.5825	1.2521	
1.4107	1.8866	2.8325	1.7186	1.1162	0.8812	1.6164	1.2807	
1.5704	1.8597	2.0521	2.012	1.4641	1.7764	2.1434	1.0719	
1.4959	1.3759	0.7763	2.115	2.193	1.1452	1.1125	2.7954	
1.4646	2.1497	1.3211	1.399	2.3721	1.6076	1.0946	1.0788	
1.8769	1.0842	0.6624	0.8425	1.9952	1.7812	2.2654	1.7136	
1.9635	1.2892	2.3646	1.8677	1.8576	2.018	2.1355	1.7286	
1.3366	1.8127	1.8293	1.5493	1.5351	1.3449	1.9706	1.9734	
2.0896	1.9351	0.711	2.3149	1.1149	1.8367	1.1822	1.4484	
1.3032	2.2829	1.8133	1.6385	0.7019	1.7293	1.4467	1.9734	
1.9683	1.8951	1.2748	0.8814	1.2685	0.9578	2.2975	1.3028	

1.4378	1.2727						
ALG11	3.0649	2.3318	3.0464	3.3292	2.5751	2.8686	3.9136
3.2676	2.9594	2.6761	2.364	2.3957	2.7527	2.7741	3.441
2.8659	2.4304	3.419	2.9981	2.9995	2.6059	2.8273	2.8264
2.7069	3.2611	3.2428	1.1246	3.0657	2.5888	2.2865	2.3557
2.0013	2.5136	2.4379	3.3027	2.8878	2.9202	3.2797	2.6673
2.4171	2.4582	2.3466	3.2994	2.2965	2.9916	2.4853	3.2947
3.0511	3.0479	2.9378	3.2983	2.8508	2.6643	3.2626	2.4659
3.4349	2.758	2.786	2.4517	3.008	2.4769	2.3651	3.097
2.682	2.6082	2.9903	2.2585	2.7265	2.7447	3.9968	2.6068
3.0469	2.829	1.9229	3.3075	2.7657	2.5244	2.835	3.041
2.9291	2.6197	2.7107	2.7415	3.5898	2.3424	2.226	3.0987
2.885	2.9604						
ALG12	3.7461	3.852	3.3356	2.5651	2.8633	3.6681	2.8738
4.178	3.6641	3.5403	3.9934	2.8153	3.3125	2.6622	3.9951
3.4901	2.7202	2.5506	2.8738	4.1182	3.7394	4.1299	3.6933
3.8574	3.624	3.0723	2.9049	3.9058	3.5533	2.6262	4.141
2.6855	3.7981	4.2445	3.9828	3.1113	3.2168	3.4168	4.0854
2.934	3.6782	3.0766	3.4431	3.4075	3.0562	2.7878	3.6108
3.5609	4.1756	2.8556	3.7384	2.5642	3.3078	4.0073	3.028
3.7571	3.1374	3.5462	4.1543	3.5899	3.8791	4.1255	3.8838
3.8175	2.7092	3.9032	4.4167	3.1667	3.1602	2.6988	4.2018
3.9205	3.7331	2.3301	3.9419	3.5249	3.3148	3.8895	3.368
3.6103	3.2754	3.8035	2.9131	3.7639	3.5365	3.9881	3.4632
3.5727	3.7335						
ALG13	3.8192	2.7229	2.9699	3.004	3.0873	3.0151	3.5302
3.3425	3.5448	3.3776	2.482	3.4764	2.8194	2.9048	3.6553
3.2685	3.3882	3.2301	2.8093	3.6624	3.2365	3.0058	3.8767
3.8258	2.7637	2.5166	3.6021	2.715	3.3612	2.5179	2.8198
3.7568	3.3117	2.8905	4.0927	3.0918	3.2657	3.0788	3.032
4.3411	3.0254	3.0451	2.6715	3.2991	3.2449	3.3886	2.9521
3.4943	3.2682	4.2386	2.8787	3.6808	2.8737	3.495	3.0157
3.2314	3.0754	2.7495	3.0992	2.6768	3.0184	2.734	4.0019
3.3226	3.017	2.4617	3.4319	2.2211	3.1296	3.1788	3.7247
2.8591	3.3813	2.8888	3.396	3.6603	2.6336	4.163	3.4038
2.7983	3.0718	2.8144	3.3306	3.2182	2.2695	2.9299	2.7644
2.5512	2.7724						
ALG14	4.1881	4.3542	3.1584	3.5563	2.8468	3.0083	3.5421
3.5952	3.6932	4.0899	3.6591	2.6978	2.4891	3.6654	3.1639
2.435	3.2116	3.6008	4.5992	3.1275	3.6897	3.5008	3.2638
3.0455	3.1415	3.4	2.0921	3.464	3.4907	2.8111	2.8843
3.4888	3.6913	3.655	3.851	3.9054	3.392	3.8719	4.0576
3.2722	3.0573	3.3131	2.8027	3.2604	2.5274	3.4159	3.6327
2.8413	3.581	4.6539	3.6087	3.6725	3.9788	3.6418	3.0238
3.895	2.8403	4.7068	3.2054	4.0744	3.2331	4.1316	3.1623
4.0261	3.139	3.0278	3.4023	3.1949	2.9164	3.4501	2.8258
3.8926	3.5838	3.401	2.4915	2.5266	4.0573	3.4256	3.6092
3.5775	3.525	2.53	3.3367	2.5029	3.7925	3.8458	4.0547
3.9805	3.8936						
ALG1L	0.2884	0.3503	0.328	0.2033	1.1859	0.1177	0.3822

0.6984	1.0259	0.3591	0.9059	0.2224	0.6222	0.2701	0.2019
0.3815	1.6193	0.6865	0.6563	0.9877	0.2237	0.5411	0.3084
0.691	0.1136	0.0389	0.1933	0.3167	0.4665	0.4006	0.5854
0.5035	0.9812	0.4625	0.0214	0.1816	0.404	1.117	0.4925
0.2598	0.5186	0.237	0.4593	0.2654	0.5525	0.3513	0.3361
0.5782	0.5653	0.7702	0.67	0.6462	0.3576	0.5277	0.2972
0.7692	0.2258	0.4108	0.3467	0.2494	0.1066	0.7513	0.1655
1.2233	0.2576	0.1699	0.4941	0.1085	0.0744	0.1589	0.5071
0.8808	0.4075	0.4988	0.2503	0.2837	1.0204	0.3904	0.5443
0.9158	0.4027	0.1476	0.0874	0.437	0.5334	0.4309	0.4139
0.3989	0.2584						
ALG1L2	0.6716	0.1511	0.096	0.4149	0.9384	0.2038	0.555
0.2793	0.3172	0.6596	0.1971	0.1078	0.0749	0.4488	0.3541
0.6259	0	0.9391	0.4227	0.3693	0.5211	0.0219	0.1697
0.7334	0.0614	0.2849	0.9657	0.2799	0.2852	0.2582	0.1445
0.5927	0.3162	0.4219	0.5118	0	0.2962	0.5927	0.5098
0.4594	0.5025	0.1692	0.0798	0.3787	0.1276	0.1197	0.0896
0.4567	0.1796	0.6596	0.7905	0.5192	0.1134	0.2006	0.2296
0.1466	0.3221	0	0.6508	0.0756	0.174	0.1746	0.7502
1.1217	0.2913	0.3165	0.4992	0.1334	0.1317	0.4911	0.4139
0.324	0.1308	0.2296	0.2227	0.2368	0.4162	0.8402	0.8787
0.7711	0.222	0.1436	0.4644	0.5349	0.2485	0.1334	0.3842
0.1431	0.3534						
ALG2	4.0287	3.5115	3.6485	3.8718	3.6376	3.5015	4.1694
4.3609	3.3624	3.7188	4.2387	4.0027	4.5905	3.718	4.2122
3.3043	4.0719	3.6454	3.2968	4.2751	2.933	3.0498	3.0695
3.6581	3.9077	4.4084	3.3038	4.0676	3.4209	3.3543	3.7188
3.5767	3.4996	3.8891	3.4886	3.9061	4.6488	4.1933	4.576
3.9918	3.3204	4.1068	3.6002	3.4617	4.1686	4.602	4.3006
2.2853	3.8737	3.6643	4.3334	3.6977	4.4191	3.8223	3.654
3.533	4.6452	3.7401	4.5395	3.6687	3.7083	3.2618	3.41
3.4817	4.1743	4.5677	2.9482	4.1496	3.9953	4.7598	3.6625
3.1651	3.7204	3.1735	3.3682	3.1504	3.8505	3.8334	2.9719
3.2678	3.49	3.8609	4.1935	4.6675	3.27	4.0821	4.1501
3.9593	3.7506						
ALG3	4.6256	5.501	5.8963	5.3371	5.5487	5.0128	5.941
5.9003	5.507	5.0696	6.1925	6.5252	5.183	5.7182	6.4077
5.7576	5.6044	4.9638	5.7585	6.0919	5.3145	6.321	5.9439
6.4803	4.8654	4.951	6.3417	5.4405	5.851	5.6337	5.1105
6.6062	6.2485	5.8054	5.5471	5.2292	5.763	6.2107	5.7818
5.4027	5.928	5.1189	5.2552	5.2445	5.4711	4.9357	5.8126
6.1044	4.9952	5.9157	6.0023	5.794	5.4059	5.5429	5.5211
6.3715	5.4664	5.6121	4.6306	5.6462	5.7614	6.0764	5.8599
6.1848	5.1672	5.6396	6.4561	4.9277	5.8966	4.9048	5.5986
6.1447	5.8191	6.0874	4.7175	5.369	6.0167	5.8705	5.7583
5.8671	5.2596	5.5172	6.208	6.5453	5.2197	5.1045	5.0385
5.3062	5.1085						
ALG5	5.0923	5.3604	4.8487	5.9473	4.8546	4.1952	4.7203
4.9336	4.4834	4.7555	3.7966	4.0826	4.1508	4.9917	4.7655
3.2275	4.8372	4.3336	5.4247	5.0549	4.4825	4.9906	4.293

4.1682	5.1624	4.2565	4.0316	5.2628	4.216	4.2603	4.0096
3.7796	4.5775	3.6774	4.7311	5.1655	4.7018	5.4075	4.8632
4.3273	4.4417	4.3803	4.8525	4.7922	4.1407	4.0463	5.94
4.4141	4.7225	5.1239	5.3626	5.4717	4.6577	4.8227	3.7728
5.9032	4.1582	4.3434	4.4895	4.5954	4.7832	4.4767	5.6968
4.408	4.5364	4.2117	4.931	4.2421	4.1009	5.6243	5.2974
4.7987	5.3956	4.0691	4.4335	4.2493	4.3961	4.0619	5.6443
5.3267	4.934	4.8865	4.4245	4.2421	4.4687	4.4098	5.157
5.1114	4.9042						
ALG6	2.5449	2.7162	2.3553	3.1564	2.8439	2.7332	3.3463
2.5928	3.5915	3.454	2.3844	3.0256	2.0381	3.5592	3.177
2.3834	2.895	3.1292	4.0629	2.1518	2.5413	3.8522	2.3694
2.2517	2.5014	1.905	2.4845	3.9471	3.8333	3.2582	3.0677
2.7209	4.2437	3.1385	3.3723	3.2947	3.1972	2.9541	3.0914
4.5164	3.03	2.2741	2.7503	3.4628	2.4252	3.0929	3.7107
3.6018	2.6592	3.4058	3.3983	3.6447	3.7324	3.0545	2.7325
3.1291	2.4116	3.2947	3.4066	3.2363	2.7028	3.3278	3.4352
3.9752	3.1025	2.3369	4.4425	2.1561	2.9741	2.537	3.1409
3.4992	3.5132	3.489	2.8585	2.4	3.4666	3.1516	3.9207
3.8672	3.7775	2.8876	2.0322	1.9801	1.8832	3.5349	3.4687
3.652	3.5489						
ALG8	5.3006	6.0103	5.7963	5.6644	5.5666	4.6781	5.5222
4.1118	5.6181	5.2615	4.9006	5.0536	4.1142	5.2806	4.7951
5.1187	5.7329	4.826	5.2783	5.8949	7.0991	6.2758	5.6841
5.2296	5.765	4.7167	4.0327	4.718	6.2048	5.6093	5.1209
4.6565	5.3068	5.6386	5.8345	5.4992	5.7272	5.5458	5.1293
5.4851	5.5939	5.1908	5.2159	5.0774	4.4976	5.7482	6.8418
6.2702	4.757	5.4921	5.1995	5.6625	5.5866	4.8286	5.1154
5.6328	4.3631	6.0578	5.0945	5.6493	4.9248	5.7287	5.5927
5.0549	5.6077	4.969	5.8207	4.8226	4.5283	4.8176	6.6991
5.005	6.0314	5.0489	4.5624	5.4999	4.5486	5.1373	6.2992
5.8362	5.978	4.5209	6.3079	4.5456	4.6353	5.14	5.1341
5.6874	5.3741						
ALG9	3.7258	2.6988	3.1686	3.9245	2.7622	3.0075	3.1658
2.4612	3.8921	3.1153	2.7705	2.0905	3.3076	3.6675	3.0343
3.4154	3.6397	3.0997	3.5925	4.016	2.983	3.7133	3.2416
2.9051	2.7613	1.9728	3.3541	2.7236	3.6259	2.774	2.8868
2.2396	3.251	3.1157	2.1765	3.4216	2.4652	3.0367	3.0989
3.6442	2.4599	2.7684	3.4177	3.5409	3.2577	3.4098	3.1741
3.2794	2.8043	3.7672	3.2331	3.6495	2.2933	3.2879	2.6475
3.8236	3.2649	3.2376	3.1127	3.1564	2.971	3.5765	3.8407
3.5431	2.7315	3.5054	3.3249	3.3836	2.8913	3.1191	2.7472
3.7495	3.739	2.5688	3.6	4.1548	2.8858	2.7664	3.7307
3.5399	2.8479	3.1956	2.6084	2.5209	2.4128	1.9308	4.0559
3.7812	3.8533						
ALK	0.173	0.4769	0.3115	0.3006	0.2148	1.3894	0.2452
0.1964	2.0097	0.9371	0.0983	0.3231	0.1848	2.1258	1.3614
1.3357	0.1374	1.671	1.5527	0.8024	0.2569	0.1227	0.3393
0.543	0.6917	0.0601	0.1422	0.6747	2.9972	0.6041	0
0.7724	1.396	0.9891	0.1228	0.5073	0.2581	1.1028	2.084

0.4272	2.5708	0.0908	1.3929	0.3773	0.0926	0.0651	0.987	
1.7891	0.3882	0	0.3029	0.1775	2.2565	0.3278	0	
0.4487	0	0.0215	1.0168	1.5481	0.8623	1.4326	0.0665	0
	1.2948	0.15	0.3939	0.1473	0.4223	0.5341	1.8768	
1.0638	2.7015	0.2505	0.6786	0.1295	0.8334	0.4435	2.2193	
1.1735	1.0023	0.6341	3.2355	0.5506	0.2448	0.9899	0.1788	
0.0786	0.5621							
ALKBH1	2.542	3.037	2.558	3.1813	2.3943	2.3667	2.207	
2.9095	1.786	1.8971	2.0369	2.9999	2.4097	2.635	2.207	
2.6618	2.8108	2.8115	2.8091	2.2832	2.7543	2.4183	2.9134	
2.6678	2.1753	2.8619	2.3977	2.5175	2.6992	2.7294	2.1371	
2.8663	2.5025	1.8726	1.6833	2.8719	3.0404	2.8883	2.4179	
2.7314	2.4083	2.4891	1.9592	2.7294	2.7335	2.9291	2.7654	
2.3285	1.9995	3.7703	1.8992	3.1533	2.454	2.4892	2.9661	
2.0887	2.2383	2.8062	2.3353	2.9985	2.8799	3.3433	2.9059	
2.0446	2.5178	2.4184	2.6423	2.6335	1.7457	2.4279	2.3424	
2.2383	2.6187	3.4302	2.7881	2.8531	2.7977	1.8252	2.5748	
2.284	2.6963	2.3983	2.7871	1.9593	2.3331	2.3116	2.5576	
2.5335	2.5087							
ALKBH2	4.3755	4.5948	3.8226	3.5498	4.5186	3.7761	4.1392	
4.315	2.9986	3.4567	4.2045	3.6839	2.8786	3.7508	2.6394	
4.0316	4.9149	3.5844	3.7941	4.4372	4.3988	4.3536	3.6456	
4.887	3.6522	3.3416	4.6142	3.9755	3.0662	3.5329	3.9017	
4.4288	4.4622	3.4123	2.6562	3.5944	4.7527	3.5209	4.0327	
3.7457	3.9845	3.0996	3.1959	4.213	2.28	2.4068	2.8128	
4.3377	3.3475	4.3199	2.8811	4.2004	3.6515	4.3881	4.2242	
4.9115	2.6034	4.108	3.7844	3.7046	4.3017	4.7197	3.7121	
4.3724	4.6869	3.1504	4.6186	3.3653	3.4531	2.9456	3.8621	
3.8005	3.7659	3.9987	4.0099	3.2063	4.2453	3.2411	4.2003	
4.499	3.7596	3.9246	3.8407	2.9485	3.4509	3.7626	2.7437	
3.284	3.0511							
ALKBH3	3.4239	4.3757	3.9277	3.5257	3.3905	3.4166	4.0276	
3.8503	3.8561	3.6021	4.0016	2.9167	3.0941	4.028	3.67	
4.0161	3.9812	3.2687	4.7982	3.6253	3.6379	3.6391	3.2623	
3.4644	3.8522	3.6776	2.3489	3.3831	3.7092	3.5996	3.2781	
3.4202	4.3312	3.1912	3.2014	3.6609	4.3409	3.857	3.7566	
3.4887	3.2569	3.1776	3.7946	3.7546	2.9475	3.5561	3.9821	
3.7302	3.8955	4.0637	3.9513	3.0766	3.4872	4.1805	3.3977	
4.2253	2.9818	3.369	3.7835	3.0548	3.2172	3.8717	3.1138	
3.6634	3.2253	3.1382	3.5239	3.5562	3.0087	3.3428	3.5215	
3.3467	3.766	4.0104	3.256	4.2696	3.4358	3.1722	3.3646	
4.2919	3.344	3.0761	4.1843	3.0917	3.3846	3.592	3.6428	
3.3532	3.3038							
ALKBH4	2.2207	3.1763	2.3204	2.0953	2.6353	3.2281	1.2884	
3.2756	2.0648	1.8282	2.5329	1.5898	1.8199	1.971	2.2761	
2.3841	2.3375	2.6865	2.1678	2.3899	2.5448	3.2731	2.5763	
2.588	2.7229	2.4263	1.7289	2.3386	2.7672	2.5895	2.4549	
2.4345	2.3472	1.4765	2.3998	1.7989	2.6006	2.9024	2.8149	
1.7121	2.7026	2.3019	2.1533	2.3933	1.7811	2.1093	1.3469	
2.5891	2.3904	1.9051	2.2955	2.3365	1.6322	2.4056	2.9162	

2.5864	1.2737	2.9946	2.4701	3.0289	2.8078	2.7085	2.7735
2.2758	2.369	1.9776	2.1866	1.9644	1.5456	2.6509	2.4832
2.5741	3.2305	1.9195	2.4556	2.1659	2.3121	2.4275	2.1059
2.7085	2.4244	2.4614	0.6609	2.6792	2.7681	1.9756	1.9271
2.2276	2.0154						
ALKBH5	5.0187	5.205	5.4321	5.6473	5.0411	5.6248	5.4945
5.1084	4.7497	5.0019	4.9684	4.5555	5.13	4.0214	4.6377
5.8732	4.9218	5.5506	4.5444	5.5761	5.5427	5.055	5.1156
6.0782	4.3262	5.9514	5.0527	5.4932	5.4548	5.1755	6.5615
4.2285	4.9407	5.0249	5.2208	5.1417	4.398	5.4858	5.1091
4.931	4.9711	4.7915	4.8634	4.59	4.7155	5.3056	4.7598
4.9786	4.8115	4.8005	5.2166	4.5939	5.0933	5.0437	6.1824
5.2612	6.0058	4.6782	4.6699	4.744	5.0773	5.7371	4.7287
5.2791	4.8372	6.0404	4.831	5.2225	4.7416	5.1992	5.4094
5.614	4.8336	4.8649	5.7679	4.9402	4.8428	5.5016	5.208
5.7965	5.6183	4.8442	4.9262	4.9727	4.5121	5.1735	5.5536
4.9874	5.4073						
ALKBH6	3.4667	3.2013	3.6734	1.9797	3.7468	3.8247	3.8404
3.6044	3.2545	3.4717	3.7339	3.4165	3.913	4.0396	3.6943
3.9383	3.3026	3.0927	3.4572	3.6593	3.8136	3.1484	4.2077
2.9396	3.2937	4.0536	3.4683	3.78	3.1313	2.7561	4.9873
3.4303	4.5821	4.5999	3.3725	3.4615	4.2002	3.1885	3.4688
3.119	3.9026	3.9826	4.0227	2.8569	4.0199	4.0274	2.7561
3.1405	3.5638	3.719	3.5352	3.0112	3.3945	3.2514	4.5577
3.6456	2.5469	3.4562	3.3106	3.4164	3.801	3.3603	3.2418
4.0368	2.9985	4.1034	2.8527	3.6235	3.908	3.5377	4.1153
4.1726	3.5084	4.1072	2.8648	4.2961	3.8425	3.1548	3.2241
3.8071	3.3786	3.4899	4.0476	3.5137	4.6562	4.1246	3.5392
3.6226	3.6712						
ALKBH7	3.3242	2.9219	3.6964	1.6316	2.6144	2.586	2.4988
3.5626	2.6357	2.6864	3.1837	1.7747	3.0693	1.6437	2.8571
2.9179	3.0815	1.896	1.8955	3.455	3.2569	2.7723	2.9702
2.5769	1.8767	2.4363	3.2192	3.1076	2.4943	3.2976	3.0677
3.5428	3.3231	3.5676	2.9405	2.043	3.0519	4.3453	3.7635
1.8564	3.8462	1.9858	2.308	2.5899	2	2.6372	2.364
2.1813	3.4503	2.2862	2.3015	1.8479	2.6452	3.3145	4.3648
3.7722	2.686	3.6352	2.6282	2.9553	3.6996	3.398	2.5929
2.487	3.4231	3.3969	3.654	2.3383	2.7567	2.9	3.4092
2.9764	3.5689	1.8254	1.684	3.8471	3.1332	2.6363	3.1365
3.1714	3.2963	2.4856	3.2103	3.2627	3.7047	3.5125	3.1928
3.8398	3.6855						
ALKBH8	1.9658	2.0123	2.2201	2.4666	3.5921	2.515	2.6852
1.6624	1.983	1.4528	1.2365	1.3694	2.3964	2.6106	1.7808
2.672	3.069	1.4361	2.0782	2.6524	2.1273	2.4792	1.8739
2.2685	1.8807	2.1089	1.3048	1.7288	2.6304	1.4496	2.0138
1.5073	2.6052	1.2485	1.0001	1.9709	1.8032	1.7172	1.77
2.8452	1.1735	1.7144	2.8079	2.4649	1.806	1.7619	2.0957
2.5126	1.8958	3.0403	1.5634	2.6914	1.9478	2.6876	2.055
2.8023	2.0964	1.8026	1.6908	1.8807	2.6602	2.2084	2.362
2.5632	2.0774	1.5629	2.1578	2.4947	1.4431	2.7468	1.5703

2.1429	2.5154	1.7467	3.0108	2.9725	1.3448	1.3724	1.8066	
1.1308	1.6059	1.9298	1.3448	1.9359	2.4633	1.9538	1.7852	
1.8592	1.7608							
ALLC	0	0	0	0	0	0.0549	0	
0.0522	0.3616	0	0.0568	0.0632	0.0711	0	0.1154	
0.66	0	0	0	0	0	0	0.0304	0
	0	0.0722	0	0	0.0587	0	0	
0.0068	0	0	0	0	0	0	0.0423	0
	0	0	0	0	0.0468	0	0.0503	
0.0421	0	0.0953	0.1163	0	0	0.016	0	0
	0	0	0	0	0	0	0.0258	0
	0.2635	0	0	0	0.0174	0	0	
0.023	0.322	0	0.044	0.0334	0	0	0.0619	0
	0	0	0.4655	0.166	0	0.0654	0	0
	0							
ALMS1	2.1964	1.992	2.0853	1.6898	2.102	2.0226	2.1231	
1.3377	2.9186	2.2445	1.5469	1.4589	1.6598	1.9925	2.0739	
1.515	2.6493	2.3053	2.094	1.4577	1.7756	1.6258	1.3796	
2.0145	1.4636	1.9423	1.5807	1.8708	1.4328	1.3958	2.1418	
2.0092	1.6909	1.4907	1.6127	2.4813	2.1192	1.3399	1.4654	
2.486	1.5807	1.0796	2.4926	2.2492	1.9692	2.4337	2.477	
2.0261	1.4913	2.7185	2.2859	2.4166	2.4673	2.3795	2.2343	
2.6757	1.5956	1.7427	2.0461	2.133	1.243	1.7291	1.9843	
2.5006	2.1116	1.3566	2.0385	1.6807	2.4474	1.4459	1.3352	
1.9827	1.6513	1.4615	1.6926	1.54	2.1808	1.9933	1.7827	
1.692	2.1306	1.2149	1.3345	1.042	1.4242	2.2276	1.4182	
1.5939	1.5417							
ALOX12	0.3035	0.6269	0.6297	0.4065	0.0613	1.0576	0.4046	
0.2174	0.51	0.6765	0.1828	0.1739	0.302	0.4693	0.2333	
0.4451	0.8047	0.4382	0.578	0.6475	0.5972	0.0797	0.1584	
0.4876	0.1222	0.2589	0.4566	0.3816	0.3739	0.3434	0.2834	
0.2534	0.3724	0.116	0.5753	0.2205	0.1134	0.3448	0.3082	
0.5509	0.2517	0.2591	0.0936	0.4344	0.2094	0.111	0.4863	
0.5697	0.3295	0.4113	0.2886	0.3293	0.1547	0.3207	0.238	
0.5328	0.3185	0.1917	0.0429	0.3449	0.1717	0.3237	0.309	
0.6627	0.7115	0.2826	0.9414	0.1748	0.1378	0.4812	0.2938	
0.3771	0.26	0.202	0.4349	0.5803	0.5923	0.5546	0.3294	
0.5011	1.0042	0.3342	0.2233	0.7018	0.1469	0.4387	0.1197	
0.3057	0.6388							
ALOX12B	0	0.0351	0.094	0	0.0742	0	0.0759	0
	0.0223	0.0262	0.0519	0	0.0153	0	0.0151	
0.0391	0	0.1452	0	0.1122	0.0441	0	0.0075	0
	0	0	0.1169	0.0605	0	0.1423	0.4223	0
	0	0.0282	0	0.091	0.0321	0.0993	0.0441	
0.0691	0	0.0189	0.0478	0.0664	0.0185	0.0644	0	
0.069	0.0196	0	0.0264	0	0	0.0708	0.3804	0
	0.13	0	0	0	0	0	0.1199	
0.0269	0	0.0328	0.1537	0.0378	0.0935	0.0423	0.0636	
0.0544	0.0189	0.0015	0.11	0	0.07	0	0.0598	0
	0	0	0	0.0189	0	0	0	0

	0							
ALOX15	0.0727	0	0	0	0	0	0	
0.044	0.0419	0	0.0047	0.0036	0	0	0.0376	0
	0	0	0	0.0339	0.0294	0	0.0024	
0.0719	0.0414	0.1214	1.9121	0.1243	0.014	0	0.2386	0
	0.0184	0.0149	0.03	0.0268	0.0171	0.0427	0.1054	
0.0575	0	0	0.0276	0	0.1732	0	0	0
	0	0.0579	0.0321	0	0	0	0.099	0
	0.1003	0	0	0.0459	0.0714	0.0261	0.033	0
	0	0.1238	0	0.032	0	0	0	
0.0334	0	0.1961	0.0346	0.0414	0.0457	0	0.4982	0
	0.117	0	0.1291	0.0443	0	0	0.0233	0
	0							
ALOX15B	0.1079	0.1685	0	0	0.1111	0	0.1231	
0.0262	0.0681	0.1016	0.0355	0.0446	0.1403	0	0.1377	
0.1534	0	0.1861	0	0.188	0	0.0632	0.0933	
0.0857	0.0475	0	0.148	0	0.0499	0.0917	0.0206	
0.0882	0.0629	0.0312	0	0.0471	0.068	0.0408	0.0238	
1.0298	0.1998	0.0226	0.0481	0	0	0	0	
0.0372	0.0481	0	0	0.0764	0.114	0.2008	0.0881	0
	0.0338	0.0119	0.0558	0.0101	0.3456	0.0742	0.0374	
0.0176	0	0.0098	0.7905	0	0.079	0	0.0203	
0.134	0.0525	0	0.2059	0.045	0.1186	0.3027	0	
0.1551	0.04	0.275	0	0.0481	0	0.0419	0.0724	0
	0							
ALOX5	0	0.0975	0.0466	0.0644	0	0	0	
0.0482	0.0233	0	0	0.0101	0.0825	0	0.0153	
0.042	0.1038	0.0518	0	0.0598	0	0	0	0
	0.0334	0	0.0377	0	0.0263	0	0.0563	0
	0.0343	0	0	0	0.0478	0	0.0197	
0.0329	0	0.0161	0	0	0.0453	0	0.0537	0
	0	0	0.0274	0	0	0.0084	0	0
	0.0907	0	0.094	0	0	0.0276	0	0
	0.0655	0.0421	0.0314	0.0218	0	0.1391	0.0254	
0.0128	0	0.0201	0	0.0161	0.0429	0	0.0061	0
	0.051	0.0641	0	0	0	0	0	
0.0408	0							
ALOX5AP	0.1489	0.2898	0.1426	1.2979	0.0465	0.2318	0.4261	
0.0737	0	0.0819	0.0556	0.2799	2.0633	0.245	0.1164	0
	0	0.4989	0	0.2788	0.3902	0.1417	1.2334	
0.1872	0.4769	1.4776	0	0.2357	0.075	0	0	
0.0414	0.0944	0.0866	0.2225	0	0.1507	0	0.0685	
0.0813	0	0.1178	0.1077	0.0969	0.1091	0.3741	0	
0.6958	0.7476	1.3174	0.4557	0	0	0.0969	0.0499	0
	1.287	0	0.1845	0	0.0581	0.9645	0.0339	0
	0	0.2356	0.0831	0.5179	0.1604	0	0.6929	
0.3501	0	0.7637	0.2178	0	0.2899	0.3065	0.1293	
0.1851	0.0367	0.6742	0.0777	0	0.3151	0	0	0
	0							
ALOXE3	0	0	0.0762	0	0.1699	0	0.1893	

0.0269	0.026	0.0135	0.0905	0.0514	0.2507	0	0.0173	
0.0602	0.1397	0.0685	0	0	0.0563	0	0.0487	0
	0.0138	0.4274	0.0836	0.2065	0.0529	0.1535	0.514	
0.0147	0.0722	0.0682	0.0232	0.0316	0.0935	0.0458	0.029	
0.0462	0	0.1477	0.1704	0	0.4645	0.0435	0.0366	0
	0.0093	0	0.0374	0.0439	0.056	0.011	0.2591	0
	0.749	0	0	0.0178	0	0.0109	0.0414	
0.1174	0.0227	0.062	0.0836	0.3376	0.017	0.1691	0.1371	0
	0.0932	0.0075	0	0.0252	0	0.027	0.0087	
0.0484	0.0608	0.0266	0.2483	0.0184	0	0	0.1399	
0.0299	0.1259							
ALPI	0	0	0	0.1044	0.0059	0	0	0
	0	0	0	0	0	0	0	0
	0	0	0	0	0	0	0	0
	0	0	0	0	0	0	0	0
	0	0	0	0	0.0081	0	0	0
	0	0	0	0	0	0	0	0
	0	0	0	0	0	0	0	0
	0	0	0.0813	0	0	0.0095	0	0
	0	0	0	0	0	0	0	0
	0	0	0	0	0.0054	0	0	0
	0	0	0	0	0	0	0	0
	0							
ALPK1	1.6657	1.1419	1.3984	1.0148	1.7528	1.987	2.4766	
1.4627	1.5951	1.2912	1.4573	1.8402	1.6371	1.7068	1.1934	
1.5993	2.4524	1.8067	0.8282	1.7405	2.0185	1.7053	1.4326	
1.2511	1.948	2.1976	2.5264	1.2486	1.1459	1.1063	0.1445	
1.8339	1.8678	2.6557	2.2127	0.7292	2.5318	1.7724	1.3062	
1.9298	2.0103	2.0572	0.5616	1.9368	1.4154	1.7502	1.7739	
1.7372	2.5217	2.3933	2.0155	2.3613	2.0745	1.6026	1.229	
1.7604	2.5506	1.9481	1.3856	2.1988	1.8975	1.2581	1.251	
1.7137	0.7873	2.5626	2.0467	1.319	0.5461	1.86	1.9639	
1.627	1.8069	1.2855	2.0688	2.2724	1.4539	1.8724	1.5443	
1.9938	1.6018	1.4576	0.9579	2.2762	1.2179	1.8396	2.239	
2.1655	2.1358							
ALPK2	0	0	0	0.7551	0.3987	2.2519	0.1207	
0.09	0.0218	0.0235	0.0178	0.0625	3.265	0	0.3125	
0.0209	3.3364	0	0	0.0355	0	0.4089	0.2518	
0.035	0.7882	3.6372	0.1021	0	0.0891	0.0264	0.0199	
0.0272	0.0351	0.0315	0.362	0.0214	0.218	0	0.0936	
0.1726	0.0411	2.1389	1.1177	0	0.0356	2.2652	0.0386	
0.0276	0.2018	3.3396	0.0393	0.0748	0	0.0903	0.2155	0
	0.0123	0.5256	0	1.7726	0.1069	0.086	0.0229	
0.1762	0	0.1768	0	0.8144	0	0.0919	2.2034	0
	0	0.1741	0.8173	0	0	1.3715	0	0
	0	3.4813	0	0	0.719	0	0.0322	0
	0.0623							
ALPK3	0.0919	0.0047	0.0088	0.3874	0.084	0	0.1523	0
	0.2693	0.5655	0.0974	0.0649	0.1206	0	0.2307	
0.0449	0.1234	0.1781	0.1032	0.0326	0.0938	0.8168	0.3456	

0.2083	0.0594	0.3597	0	0.0399	0.3503	0.0096	0.016	
0.0462	0.012	0.164	0.1576	0.0977	0.0336	0.6936	0.0478	
0.5674	0.5101	0.4029	0.0036	0.0149	0.1448	0.0223	0	0
	0.1167	0.1505	0	0.1196	0.164	0.0998	0.0332	
0.0832	0.0547	0.039	0.0315	0	0.1213	0.0736	0.052	
0.0601	0.0362	0.0743	0.0668	0.0692	0.0802	0.0622	2.626	
0.0403	0.0504	0.0733	0.0679	0.0319	0.0649	0.0781	0.1555	
0.7318	0.0468	0.0564	0.4248	0.2142	0.1675	0.0678	0.0619	0
	0.0034							
ALPL	0.18	0.1391	0.2592	0	0.1674	0	0.0293	
0.2056	0.0931	0.0819	0.4764	0.0991	0.0769	0.0774	0.822	
0.1315	0	0.1187	0	0	0.0772	0.591	0.0546	0
	0.5275	0.7321	0.0466	0.1349	0.1281	0.7927	2.8025	
0.0152	0.0589	0.0562	0.0491	0.8346	0.5929	0.1807	0.1713	
0.3662	0.0307	0.0364	0.418	0	0.164	0.0893	0.664	0
	0.0839	0	0.0788	0.0432	0.0731	0.1361	1.3737	
0.0337	0.107	0.0199	0	0.1381	0	0	0.0527	0
	1.0697	0.0871	0.4921	0.2023	0.4785	0.1667	0.839	0
	0.0218	0.2001	0	0	0.0675	0.0423	0.2009	0
	0.6052	0.0805	0.1541	1.3189	0.2266	0.1714	0	
0.0739	0							
ALPP	0	0	0.0478	0	0.4405	0	0	
0.0176	0	0.0222	0	0	0.0348	0	0.01	0
	0.1153	0	0	0	0	0.0458	0	0
	0.0279	0.1144	0	0.1094	0	0.0307	0.0534	0
	0.0291	0.024	0	0	0.0032	0.034	0	0
	0.0168	0.0431	0.0889	0	0.0136	0.029	0.0552	0
	0	0	0	0	0.137	0	0.0508	
0.0397	0	0	0	0	0	0.0098	0.0124	0
	0	0	0	0	0.0786	0	0	
0.0123	0	0.0061	0	0.0898	0.0072	0.0158	0	0
	0	0	0.0112	0	0	0.0442	0.038	
0.0391	0							
ALPPL2	0	0	0	0	0	0	0	0
	0	0	0	0	0	0	0	0
	0	0	0	0	0	0	0	0
	0	0	0	0	0	0	0	
0.003	0	0	0	0	0	0	0	0
	0	0	0	0	0	0.0428	0	0
	0	0	0	0	0	0	0.0373	0
	0	0	0	0	0	0	0	0
	0	0	0	0.0016	0	0	0	0
	0	0.01	0	0	0	0	0	0
	0	0	0	0	0.0279	0	0	0
	0							
ALS2	3.6417	3.7512	3.5001	3.2319	2.5974	4.5985	4.3599	
4.0868	4.0168	3.8306	3.5159	3.5687	3.4576	3.563	3.6544	
3.769	3.526	3.9906	3.6602	3.087	3.3736	3.1294	3.4955	
3.2748	3.826	3.9574	3.6567	4.3013	3.1508	4.1246	3.6491	
3.0813	3.5139	3.6384	3.4338	3.8757	3.5122	3.5066	3.362	

3.5438	3.3582	3.9545	3.6652	3.4267	3.6506	4.1158	3.7091	
4.0152	3.5846	3.5816	3.5203	3.3746	4.057	3.3443	3.6998	
3.0886	3.4541	2.9731	3.9741	2.8405	2.9352	3.3162	3.027	
3.4375	3.7767	3.4098	3.6745	3.4382	4.3219	3.5213	4.2634	
3.5317	3.777	3.6197	3.4933	4.0808	3.4875	3.509	3.1244	
3.5837	3.7968	3.594	3.8581	3.4201	3.4402	3.8107	3.4444	
3.1088	3.272							
ALS2CL	1.3504	0	1.0028	0.0833	0.5597	0	0	
0.0607	0.0402	0.8823	0.6423	0.6026	0.8937	0.0084	0.2373	
1.0092	1.3194	0	0.0263	0.4448	0.514	0.1017	0.2913	0
	0.0359	1.2107	0.0182	0.4402	0.2713	0.9441	0.8737	
0.136	0.0257	0	0	0.1421	1.0972	0.5312	0.0352	0
	0.764	1.3925	0.7715	0.4377	1.444	0.6268	0.3454	
0.032	0.1943	0.3657	0.0147	0.3722	0.0993	0.371	0.53	
0.4427	1.9279	0.1109	0.1041	0.1218	0.1358	0.1573	0.1314	
0.1016	0.7368	0.8093	0.8784	2.4333	0.2343	0.256	0.1579	
0.1307	0.1498	0.1778	0.172	0.155	0.1388	0.0989	0.0869	
0.1501	0.1041	1.7283	0.5642	0.3379	0.3415	0.315	0.5669	0.3
	0.3165							
ALS2CR11	0	0.8716	0	1.0181	1.4189	0.3378	0.0906	
0.5723	0.2013	0.5303	0.0656	2.0677	1.518	2.0971	1.0265	
0.3272	0	0.1167	0.0153	1.8671	0.9327	0.4226	0.6638	
1.6709	0.1662	0.6255	0.6985	0	0.5347	0.0906	0	
1.0916	2.082	2.2202	0.4062	0.5374	0.8248	0	0.2473	
2.2169	1.7203	2.1443	0	1.2022	0.5629	1.5627	0.4392	
1.3107	0	1.156	1.3107	1.1012	0.9406	0.9163	0.1424	
0.2004	0	0	1.4861	0	0.5943	1.3128	0.5034	
0.1784	0.2016	0.1437	0.1645	1.222	0.1541	0.2935	0	
0.3105	0.1597	1.0046	1.6103	0.7706	0.1606	1.5405	0.8287	
0.15	0	1.5121	1.4548	2.291	0.1682	1.222	0.6787	
0.5033	0.4239							
ALS2CR12	0.6222	0.5049	0.4249	0.5085	0	0.7211	0.5085	
0.0891	0.235	0.5953	0.1325	0.0768	0.1771	0.7899	0.1975	
0.3822	0.6278	0.3746	0.6698	0.0494	0.9437	0	0.0524	
0.4701	0.4822	0.1301	0.0775	0.3589	0.0803	0.5146	0.1442	
0.0827	0.6278	0.7222	0.1587	0.2	0.3137	0.796	0.2986	
0.5632	0.7363	0.091	0.2314	0.5419	0.1352	0.2887	0.4405	
0.4007	0.3445	0.3191	0.4179	0.1332	0.4459	0.611	0.1833	
0.789	0.0689	0.0626	0.2399	0.1314	0.2022	0.6587	0.2159	
0.7906	1.0835	0.1806	0.1865	0.2285	0.2514	0.2791	0.712	
0.4202	0.6052	0.3116	0.1372	0.394	0.8244	0.0764	0.5673	
0.4361	0.3634	0.1472	1.2057	0.1187	0.1273	0.5936	0.7174	
0.5425	1.216							
ALX1	3.8268	3.5874	2.2407	3.9323	3.0916	3.3635	3.8356	
3.0329	2.7762	3.6581	2.4642	2.499	1.9147	4.3737	2.1072	
3.3394	2.4967	3.5711	3.5198	2.9874	4.6393	3.4211	2.6562	
2.12	2.7214	1.6461	4.621	3.9358	3.0149	3.0819	2.0917	
3.3988	3.2431	2.8415	3.382	3.7439	3.7423	4.4718	2.834	
5.2312	3.8839	2.8533	2.9006	2.7059	3.519	4.7687	3.8332	
4.0204	3.1891	2.9176	3.2379	3.3279	4.4304	3.5454	2.1235	

4.2992	1.3469	4.618	4.0601	3.336	3.7957	3.5875	4.2726	
3.7889	4.3472	1.2025	3.2424	2.8201	3.0355	3.3384	3.383	
2.4643	4.2341	2.4602	3.2576	3.7503	3.8357	3.047	3.2733	
4.0763	2.7556	3.2679	3.2861	3.405	3.8044	4.454	4.7158	
3.8239	2.1722							
ALX3	1.8059	1.4297	1.0935	0.6152	0.9009	1.3505	1.0224	
0.4499	0.9988	1.3739	1.4269	0.8285	0.574	1.8101	0.8869	
1.5909	1.6297	1.0255	0.774	1.0029	1.3196	1.094	0.5086	
1.1939	1.0567	1.6339	0.8507	0.8882	0.2694	0.3548	0.8167	
1.2947	1.3512	1.6703	0.5846	0.4432	1.0543	1.5683	1.2814	
0.8096	1.0928	1.2519	0.6957	0.6418	0.3891	1.0217	0.205	
0.9958	0.9884	0.977	0.6413	0.41	1.1861	1.7099	1.1865	
1.1701	1.0868	0.527	0.464	0.8011	0.479	1.0169	0.6142	
1.1472	1.245	0.7025	1.3941	0.6118	0.562	0.6962	0.5075	
0.9278	0.6212	0.4061	0.5991	0.6973	1.3705	0.5635	1.1277	
1.0643	1.511	0.3821	0.8729	0.285	1.1673	1.094	1.4906	
1.0602	1.0804							
ALX4	0	0	0.0122	0	0.1462	0.0539	0.0283	
0.0472	0.2508	0.0067	0.297	0.0455	1.1824	0	1.2968	
0.0449	0.0134	0	0.0974	0	0.011	0.0148	0.3913	
0.0314	0.9446	0.0034	0.0039	0.0301	0.0235	0.5352	0.9162	0
	0.0946	0	0.2904	0.1016	0.1829	0.213	0.0347	
0.5045	0	0.3237	0.7142	0.002	0.3033	0.8994	0.4129	
0.0254	0	0	0.2669	0	0	0.0387	0.0681	
1.26	0.0798	0	0	0	0.1384	0.1162	0.2496	0
	0.1075	0.0461	0.0827	0.3165	0.5176	0.3773	0.0416	
0.0521	0.1565	0.0436	0.0442	0	0.0358	0.0472	0.1072	0
	0.0826	0.0547	0.3342	0.0896	0.0795	0	0	0
	0							
ALYREF	7.0576	6.6384	7.1504	4.8412	6.7226	5.9624	4.6527	
6.6909	5.1314	6.1346	5.6641	5.601	6.8594	5.7199	6.5019	
5.7819	6.6925	5.4021	6.0734	6.9423	6.3068	6.3884	5.6626	
6.541	6.2634	5.8199	4.9643	6.7074	6.0725	5.9837	6.6205	
6.8994	6.1207	5.6201	5.7585	6.0953	7.3804	5.7056	5.9972	
5.5027	5.2286	5.6836	6.3933	6.2034	6.3201	5.9595	6.3584	
6.2292	5.416	6.2904	6.1217	5.0565	5.2405	5.9096	7.292	
6.6286	5.6382	7.2187	6.3643	7.0932	6.516	6.2949	6.7285	
6.0776	7.1172	5.0132	6.3811	5.1186	6.3233	6.7763	6.0761	
6.5893	6.8106	5.5397	6.395	5.9237	6.514	5.4494	5.1888	
5.9057	6.6394	5.3259	4.9325	6.3369	7.292	6.5421	5.0305	
6.3201	5.6954							
AMACR	2.4278	4.0399	3.7029	3.5284	5.2323	3.654	2.625	
2.1587	2.2465	2.0026	3.1635	2.1536	2.65	2.595	3.1918	
3.8034	2.8723	2.5153	3.1952	2.2976	2.5888	3.3658	3.3048	
3.2864	3.8991	2.8964	3.4948	5.2113	2.911	5.3628	3.5654	
2.4329	2.3105	1.8625	3.1137	3.116	2.3877	5.2286	3.1814	
4.7287	2.9717	2.5855	3.5516	2.4206	2.2872	2.5787	2.3596	
3.1414	2.3356	2.1812	1.854	3.5	1.9798	2.4414	3.4837	
2.816	2.4475	1.6776	3.8156	1.4135	4.1928	2.8486	3.818	
2.4964	2.4144	2.6752	2.2295	2.9024	4.0003	3.8889	2.635	

1.9126 2.7425 3.9215 3.8653 3.6476 3.3803 4.9533 3.025
3.5838 3.0616 1.839 2.5115 3.3881 2.9623 1.5693 2.5036
2.4651 2.0816
AMBN 0 0 0 0 0 0.0885 0.0429 0
0 0.1399 0.0349 0 0 0 0 0
0 0 0.2166 0 0.2341 0.0291 0
0.1463 0 0 0.3539 0.1092 0 0 0
0.0226 0.0698 0 0 0.0463 0.0335 0 0 0
0.1331 0 0 0 0 0 0.2444 0
0 0 0.0557 0 0.18 0.1122 0.0322
0.0725 0 0 0 0 0.0289 0 0 0
0 0.0037 0.0819 0 0 0 0.0616
0.007 2.6951 0 0.0058 0 0.0127 0 0.0259
0.0222 0.006 0.0015 0 0.0271 0 0 0
0.1432 0.1593
AMBP 0 0.0457 0 0 0 0 0.0321
0.0344 0 0 0 0.0395 0.1768 0 0 0
0 0.0624 0 0 0.0598 0.0448 0.7125
0.1133 0.1822 0 0 0 0.0641 0 0.031
0.1312 0 0 0.0353 0.0336 0.0465 0.0848 0 0
0.0674 0.1906 0 0 0.0584 0.5032 0
0.0906 0.0682 0 0 0 0 0.0185 0.1963
0.1137 0.2543 0.0449 0 0.2061 0.0503 0 0 0
0.0534 0.1459 0 0.0047 0 0.0784 0 0
0 0.032 0.1077 0 0.0525 0.4646 0.0165 0
0.0349 0 0 0 0 0 0.0879 0
0
AMBRA1 2.8091 4.0547 3.6961 3.0815 3.2358 3.556 3.5265
3.3595 3.1298 2.8186 3.6336 2.8395 2.3158 2.993 3.3124
3.4372 3.5893 2.3812 3.1161 3.1766 3.0389 3.4895 2.7265
3.4536 3.8382 3.223 3.0386 3.7016 3.2876 3.4347 3.248
3.2997 3.6211 3.2135 2.9638 2.8972 3.3592 4.5007 3.2993
3.1956 3.2215 3.3977 3.5242 3.2876 2.9039 2.8615 3.6861
3.6807 3.3542 3.0129 3.4362 2.9583 3.3055 3.0645 3.4466
3.9682 3.0751 3.3796 3.6515 3.1656 2.7696 3.3118 3.5049
3.5451 3.7886 3.2604 3.2559 3.7255 3.2126 3.0011 3.3561
2.7986 3.4207 3.5284 2.8187 4.2123 3.4829 2.6759 3.5588
3.828 2.8703 3.1172 3.8311 3.0858 2.639 3.0186 4.0744
3.4668 3.8386
AMD1 5.3171 5.2732 6.1517 6.023 5.7063 6.5424 6.6457
5.5623 6.0048 5.6195 5.2723 5.627 4.5742 6.0466 5.5551
5.3342 5.627 4.9836 5.5508 5.645 5.7467 5.9699 6.2522
5.9418 6.261 5.8232 7.6979 5.0613 6.3686 5.1119 5.0641
6.023 5.947 4.7763 5.3314 5.5579 3.7819 4.7619 5.5701
6.1027 6.1559 5.6258 5.405 5.5623 5.3155 5.3163 5.6101
5.512 6.8204 5.5459 6.5727 6.1976 6.1139 5.0412 5.9784
7.1448 4.8538 5.6061 5.4802 5.5984 5.8765 5.7003 5.3119
5.991 4.7191 4.2069 4.7931 4.2839 6.8206 5.9752 5.9416
6.1788 5.1935 5.8341 6.0118 5.0369 5.6035 5.517 4.916
5.7713 5.4683 6.6981 5.4226 5.99 5.208 5.3723 5.3879

6.4085	5.6349							
AMDHD1	1.0211	0.1697	2.136	1.1844	0.1492	0.0967	0.5157	
0.3745	0.181	0.0933	0.3905	0.7268	0.3014	0.555	0.443	
0.4526	0.84	0.2641	1.044	0.8073	1.2916	0.0484	0.2274	0
	0.2829	0.1873	0.2614	2.5233	0.3727	0.7358	0.744	
0.227	0.1349	0.29	0.0575	0.247	1.4597	0.4166	0.1193	
0.0825	0.155	0.9334	0.5915	0.8817	0.0556	0.3718	1.7785	
0.9771	0.1669	0.7489	0.6259	0.9638	0.7618	0.1891	0.5707	
1.0415	1.2747	0.545	0	0.7645	0.0618	0	0	
0.578	0.7798	0.4021	0.7005	0.4352	2.2376	0.9554	0.1306	
0.1874	0.5219	0.1435	0.1201	0.8036	1.5608	0.4223	0.5931	
0.2698	1.151	0.1038	1.0842	0.1753	0.2077	1.166	0.1942	
0.4777	0.2829							
AMDHD2	3.9872	4.3286	5.0195	1.4868	2.7885	1.9697	2.2603	
3.9245	2.8304	2.6936	3.0842	1.4047	2.7415	2.1588	2.7903	
2.9679	3.3712	2.4198	2.9172	2.4919	4.1272	2.226	2.2439	
2.9762	2.1928	1.6196	1.1351	3.5407	2.235	2.4405	2.7899	
4.4106	3.6922	3.5969	3.3486	1.9667	3.0229	6.0293	2.6223	
1.2763	3.2714	2.3949	1.8489	3.0661	1.2617	1.3242	2.3233	
3.3077	3.0203	1.5777	2.5609	1.1768	2.0965	2.5974	1.0996	
3.7402	1.8769	2.8291	3.7843	2.2263	3.25	3.6796	2.4965	
2.7418	3.9644	3.1841	5.929	4.2976	3.838	1.8436	3.6623	
3.3496	3.9151	2.5641	2.1456	4.2074	4.1866	2.5041	4.2021	
4.3852	4.6195	2.0661	4.2126	2.8576	1.7509	3.767	4.128	
5.0549	5.0831							
AMELX	0	0	0	0	0	0.1765	0	0
	0	0	0	0	0	0	0	0
	0	0	0	0	0	0	0	0
	0	0	0	0	0	0	0	0
	0	0	0	0	0	0	0	0
	0	0	0	0	0	0	0	0
	0	0	0	0.1068	0	0	0	0
	0	0	0	0	0	0	0	0
	0	0	0	0	0	0	0	0
	0.1644	0.0467	0	0	0	0	0	0
	0	0	0	0	0	0	0	0
	0							
AMELY	0	0	0	0	0	0	0	0
	0	0	0	0	0	0	0	0
	0	0	0	0	0	0	0	0
	0	0	0	0	0	0	0	
0.0253	0	0	0	0	0.0577	0	0	0
	0	0	0	0	0	0	0	0
	0	0	0	0	0	0	0	0
	0	0	0	0	0	0	0	0
	0	0	0	0	0	0	0	0
	0	0	0	0	0	0	0	0
	0	0	0	0	0	0	0	0
	0							
AMER1	1.2809	0.7312	1.3721	0.9759	1.3957	1.1632	1.707	

1.068	1.093	0.9277	0.8205	1.0336	0.9868	1.0407	1.0381	
1.0606	1.7626	1.0581	0.7945	1.2474	1.1671	0.9753	0.9803	
1.2531	0.7306	0.789	1.2204	0.9423	0.8297	0.8518	0.6328	
1.5925	1.2034	1.0204	0.482	0.7244	0.9823	0.8188	0.4849	
2.0296	0.9118	0.641	0.7819	1.0779	1.9724	1.1691	1.4181	
1.2441	1.3817	2.379	1.331	1.0984	1.1447	1.1803	1.6505	
1.9347	0.7483	1.3543	0.9497	1.3179	0.6503	1.1008	1.6592	
1.6955	1.4597	0.5501	1.2903	0.4274	0.9144	1.0139	1.2347	
0.8209	1.0747	0.6316	0.5479	0.7645	1.3819	1.3601	1.0741	
0.9413	0.9986	0.8309	0.7451	1.4437	0.4199	0.8655	0.4792	
0.8656	0.7722							
AMER2	0.0952	0.0749	0.0889	0.0927	0.046	0.1015	0.0498	
0.0528	0.0656	0.0645	0.1778	0.0463	0.0486	0.0943	0.047	
0.053	0	0.0749	0.1198	0.0855	0.1013	0	0	
0.0626	0.1198	0.0581	0.0756	0.1648	0.0582	0.1472	0.0582	
0.0433	0	0.0561	0.0442	0.1128	0.0633	0.0719	0.0561	
0.0677	0	0.0435	0	0.0819	0.053	0.0568	0.0763	0
	0.0577	0.1305	0.0549	0.0703	0.0917	0	0.0117	
0.0137	0	2.4146	0	2.2221	0	0	0.0126	
7.00E-04	0.0214	0	0	0	0	0	0	0
	0	0	0	0.007	7.00E-04	0	0	
0.0217	0	0	0	0.0034	0	0	0.0763	
0.1666	0.2564							
AMER3	0.0547	0.0189	0.0215	0.0243	0.0177	0.042	0.0073	
0.0183	0.0172	0.0289	0.01	0.0074	0.0127	0.0265	0.0055	
0.0188	0.0221	0.04	0.0712	0.0325	0.0278	0.0179	0.0022	
0.0521	0.0097	0.0161	0.0486	0.0627	0.0207	0.0342	0.0029	0
	0.0282	0	0.0015	0.0243	0.0027	0	0.0038	
0.0331	0.0246	0.0066	0.0385	0.0352	0.02	0.0151	0	
0.0396	0.0172	0.0397	0.0132	0.0372	0.0572	0.0377	0.0339	
0.0844	0.017	0.0173	0.0214	0.0199	0.0324	0	0.0305	
0.0264	0.0267	0	0.0267	0.0044	0.0074	0.0446	0.0121	
0.0094	0.0192	0.0075	0.028	0.0268	0.0246	0	0.0192	
0.047	0.0085	0	0.0121	0.0276	0	0	0	
0.0367	0.0252							
AMFR	4.8542	4.6863	4.8711	4.5544	4.5851	5.0104	4.6678	
4.7578	4.5154	4.6996	4.5586	4.0452	4.7837	4.9978	5.114	
4.5946	4.552	3.8833	4.3223	5.8857	4.7311	5.4329	4.4716	
4.298	4.7189	5.2741	5.6562	5.0196	3.4962	4.9432	4.5917	
5.0227	4.3608	4.843	4.9192	4.3755	4.8302	4.9286	4.1344	
4.8291	5.0744	4.9915	5.0174	3.6443	5.5842	4.9258	4.8407	
4.8923	4.2117	4.2545	4.4311	4.2075	4.7094	4.3622	4.9541	
4.8712	5.2837	4.2898	4.6972	4.4452	4.4262	4.8092	4.8593	
4.5946	4.2417	5.2697	4.5475	5.4063	4.9304	4.9779	5.2449	
4.6993	3.6372	3.7739	4.539	5.6276	4.4407	4.8847	5.2918	
4.86	4.2814	4.6381	4.8587	4.8569	4.6038	4.1684	5.3607	
4.7381	4.9196							
AMH	0	0.6465	0.829	0.3262	1.3364	1.0009	0.2799	
0.5515	0.6172	0.1903	0	0.1493	1.133	0	0.2553	
0.7179	0	0	0.4237	1.6668	0.3097	0.3339	0.2131	

2.1457	0.1562	0.1751	0	0.5769	0.1788	0	1.2191	
1.4469	0.1961	0	0.6061	0	0.3505	0.3543	1.4219	
0.239	1.4988	0.4536	0.7161	0.282	0	0	0	
0.2147	0.1621	0.4006	1.0196	0	0	0	1.549	
1.7207	0.514	1.7585	0	1.7847	1.0295	0.2305	0.7161	
1.2006	0.909	0.3177	0.4359	0.3811	1.2244	1.5779	0.0955	0
	0.5913	0	0.7129	0.4333	0.6887	0.5941	0	
0.3865	0.0483	0	0.6411	0.9393	0.329	0.0824	0	
0.2733	0							
AMHR2	0	0.3198	0.0963	0	0	0	0	
0.1587	0.2397	0	0.0175	0.049	0	0	0	0
	0	0.18	0.1636	0	0	0	0	
0.0427	0	0	0	0.7286	0	0.2086	0	
0.2344	0	0	0	0	0	1.0807	0.2186	0
	0	0	0	0	0.0802	0	0	0
	0	0.5852	0.1623	0	0	0.1168	0	0
	0.0316	0.032	0	0.1224	0	0.0209	1.8027	0
	0	0.0178	0	0.0107	0	0	0	0
	0	0.0252	0	0.0272	0.0764	0.1421	0.0914	0
	0.4925	0.0176	0	0	0	0.0652	0	0
	0							
AMIGO1	0.8017	0.6632	1.6906	1.56	1.6531	0.5099	0.6434	
1.0834	0.8567	1.7485	0.5915	1.5734	0.4574	1.5717	0.88	
0.2043	1.15	0.4495	0.5354	0.1278	1.1799	1.1482	0.9993	
1.2302	0.7766	0.8131	1.7813	1.1027	0.9532	0.1333	0.9043	
1.6207	1.3856	0.558	0.4881	1.5803	1.6601	1.7291	1.864	
0.5834	0.6049	1.8337	0.2448	1.104	0.3532	0.7997	0.4866	
0.8176	2.1629	0.6414	0.3485	2.345	1.2453	2.142	0.9691	
1.6338	0	1.9394	0.8416	1.191	0.8977	1.3959	0.8543	
1.83	1.3006	1.5407	0.4352	0.7952	0.334	1.5236	0.855	
1.1069	1.6324	1.4671	0.4907	0.8597	1.317	0.8121	0.2564	
0.3004	0.553	1.5823	1.0345	0.5096	1.205	0.7895	0.3514	
1.1329	1.1183							
AMIGO2	0	1.7966	1.8201	4.0268	4.954	0.0916	0.7081	
2.799	0.2045	2.4526	0.4623	2.951	4.0516	0.0487	1.4603	
1.6946	3.1463	0.0864	1.7868	1.5931	0.7356	1.5122	2.3771	
2.9084	2.6861	3.3001	1.934	3.1641	1.0672	1.3014	6.1164	
0.7837	0.1346	0.0582	0.486	2.8424	0.1019	0.5575	2.643	
0.367	0.2365	3.6276	0.287	2.1822	2.0783	6.179	2.4139	
0.4128	3.8096	3.8809	0.3993	3.2463	0.171	0.0599	2.8069	
1.7257	3.2843	0.4761	2.9223	0.5689	1.1368	3.8673	2.009	
0.9897	0	5.1548	0.0486	1.359	3.7536	3.0843	0.0568	
1.5728	0.658	2.0567	1.9926	0.1479	0.0522	2.3592	0.0988	
0.2166	1.0857	3.1213	0	1.2272	4.0009	0.068	0.0428	
0.2284	0.3889							
AMIGO3	1.1669	1.6474	2.0469	1.0555	1.8713	1.7376	1.8923	
1.941	0.4327	0.7617	0.576	0.5282	1.1839	0.4762	1.2879	
1.6159	0.9443	0.5833	0.585	1.9967	1.5988	0.9669	1.4133	
1.9956	0.8954	1.0067	0.3936	1.5697	1.3043	0.5274	1.9087	
1.4954	1.1398	0.8258	1.6454	0.4803	0.7546	1.4967	1.2795	

0.2596	1.1781	1.5724	0.8989	0.768	0.8832	0.3811	0.6239	
1.6589	0.9471	0.9285	1.4066	0.7345	0.5134	0.9177	1.8843	
1.3849	1.0797	2.3802	1.4303	2.3315	1.0862	1.2324	1.2514	
1.7282	0.6686	1.5954	1.1999	1.0414	1.4636	1.5786	0.9106	
0.9039	1.0688	0.5588	1.4597	0.579	1.4158	1.3282	0.536	
0.6508	0.7604	1.6821	0.4628	0.7143	2.5362	0.7881	0.6443	
0.7982	0.8071							
AMMECR1	2.3836	1.9446	2.8338	3.5317	2.5635	3.2123	2.5454	
3.1995	3.5678	2.7371	1.9311	2.896	4.0122	2.8429	1.9007	
2.1427	2.93	2.6466	2.6406	2.5811	3.1625	3.2309	3.0116	
4.0925	3.1271	3.0136	1.1553	2.3362	3.368	1.5508	3.3719	
3.3838	3.1745	2.5388	3.6084	3.0755	3.3083	1.8917	2.4205	
3.9623	2.1013	2.8016	3.521	3.6623	3.8234	2.7622	3.1276	
2.7793	3.0459	4.4461	3.2604	3.6589	2.8286	3.1971	3.5327	
3.7379	2.9193	3.783	2.5759	4.01	2.9105	2.0362	4.0271	
3.8446	2.5478	1.8923	2.6564	1.5338	2.5968	3.5362	3.0939	
2.2898	4.0128	2.5988	1.9544	2.132	2.6586	3.411	2.5353	
2.5141	2.6805	3.2122	3.217	3.7591	2.8698	2.7362	2.8816	
3.0486	2.7076							
AMMECR1L	4.2469	3.361	4.188	4.1791	3.4027	3.9015	4.4196	
3.7384	3.5733	4.6851	4.5428	4.3013	3.4766	4.8402	4.0954	
4.0627	3.5869	4.2469	3.5741	3.631	4.1711	3.352	3.5917	
3.7411	3.8201	3.8452	4.0543	4.1483	3.684	4.0279	2.7889	
3.4869	3.9254	3.636	3.3144	4.2033	4.4689	3.9634	3.5649	
3.8141	3.8452	3.0143	3.8091	4.0232	3.6415	3.5932	3.8274	
4.0414	3.8748	3.7944	3.5001	3.5377	4.7245	4.7179	3.3798	
3.9569	3.4367	3.6019	4.428	3.5773	3.7954	4.102	3.1926	
4.2085	4.3309	3.4084	4.041	3.7304	4.5549	3.7284	3.8044	
4.0385	4.8388	3.0758	3.7881	3.3014	4.074	3.9822	3.9392	
3.497	3.7498	3.8542	3.8546	3.8924	3.1769	4.1548	4.7196	
4.3348	4.6202							
AMN	0	0	0	0	0.0832	0.2057	0.3366	0
	0	0.2849	0.0701	0	0	0	0	0
	0.2049	0	0	0	0	0	0.1742	
0.1991	0.0637	0	0	0.2581	0	0	0.1508	
0.2028	0	0	0	0	0.0932	0.1112	0	0
	0.1953	0.0399	0	0	0	0	0	0
	0	0	0	0	0	0.172	0.0648	0
	0.0885	0	0	0.0581	0.0784	0.0195	0.0805	0
	0	0.0068	1.1465	0.0157	0.0772	0.0543	0	0
	0.0654	0.0245	0.1382	0	0.0994	0.0275	0.0562	0
	0.0248	0.0134	0.0291	0	0.2326	0.0988	0	0
	0.1969							
AMN1	2.3837	1.5792	1.4382	2.2148	2.122	2.1454	0.4238	
2.6384	2.6132	2.0389	2.6933	3.3427	2.2077	2.1009	1.7976	
1.7401	2.9337	2.3577	2.2806	1.7466	1.1595	3.2309	2.2067	
1.9281	2.249	2.3008	3.4307	2.4834	1.8078	3.1628	2.715	
2.0181	1.1367	2.3929	3.3914	2.1032	1.8379	0.5241	2.4156	
2.3669	1.9309	2.4039	3.5993	2.0332	4.1126	2.6591	2.1314	
3.0187	1.7929	3.0417	3.1495	2.6426	2.6678	1.6332	3.0093	

1.7115	1.7956	1.8576	1.5756	1.6876	1.6629	3.2635	2.749	
2.2127	1.5221	2.8607	2.9944	1.5797	1.8008	1.444	2.4885	
2.2212	2.0173	2.3631	2.4982	2.0159	1.5951	2.2833	2.7008	
2.7441	2.6982	3.0961	3.1104	3.3682	1.9306	2.4789	2.5985	
1.8861	2.0683							
AMOT	2.2292	0.8711	1.1546	3.0755	2.89	4.6287	2.5251	
3.4143	3.8387	2.7329	3.0969	2.6949	2.226	1.9873	2.4886	
4.3499	0.0226	2.3762	1.5558	2.4223	2.5858	2.4301	2.746	
4.8337	3.1025	1.0064	3.7864	1.7307	2.2632	3.6142	1.7048	
3.5996	3.5299	3.5734	4.0659	2.4362	1.2281	3.8099	2.7665	
4.3803	2.8244	1.0847	2.7424	4.3737	1.8784	2.0228	1.8583	
2.8951	2.0807	2.2276	1.7455	2.0997	2.578	1.7733	1.4304	
1.9304	0.0914	4.2334	2.0539	3.6714	3.0874	0.7415	4.8656	
3.3817	2.2653	1.2266	3.2143	1.0159	2.452	3.5091	2.9663	
2.7486	1.6586	2.9087	3.1586	2.9332	2.6607	4.7413	4.1493	
3.199	2.8521	2.303	3.4144	2.1896	2.5327	1.9771	1.6764	1.8
	2.2326							
AMOTL1	2.9163	2.7102	3.9621	4.2884	5.2422	3.8762	4.5547	
3.1935	2.6393	2.0731	3.5537	3.7122	3.5094	4.1746	4.4542	
4.5952	3.2981	3.2215	3.2075	4.4066	3.1809	4.9167	4.5135	
4.3433	5.3481	4.0414	3.564	2.0975	5.0209	2.8099	2.4214	
3.2265	4.0822	3.3274	4.6431	2.7426	4.5323	3.3468	4.0579	
4.2566	3.3247	4.1118	4.6436	5.1139	4.4326	3.4141	4.2472	
4.1393	4.9478	3.5422	3.2036	4.815	3.6473	3.1589	2.9184	
3.4124	3.4252	4.3173	3.3028	3.9951	4.0011	3.6803	4.1322	
4.3638	3.1891	3.9019	2.3197	4.546	4.1429	4.9955	5.6714	
4.2524	3.692	4.271	4.9597	4.1955	2.8061	3.5105	4.0486	
2.7483	2.7769	3.8631	6.1405	3.9844	4.0256	3.1304	3.0463	
3.588	3.2985							
AMOTL2	2.7078	2.2045	2.1251	3.0807	5.006	4.2755	2.3049	
4.0553	3.5374	3.3334	1.0888	3.7341	5.5522	2.8335	4.4726	
2.6061	5.6692	2.3798	3.1829	2.8777	2.0643	3.4532	3.1019	
2.5258	2.9342	5.4208	3.5582	1.9075	2.5746	0.7764	0.9707	
4.4622	3.4615	1.6073	3.5479	1.947	3.2863	3.1737	4.0586	
3.2483	2.7455	3.1457	3.982	3.3796	5.4176	4.75	2.4025	
2.627	5.1271	5.7784	3.5601	2.7568	2.9655	3.0276	4.8879	
3.1264	5.9652	3.9978	0.849	4.6857	3.5407	2.6984	3.4489	
3.7776	3.462	5.2962	3.1783	3.9273	1.0552	4.1968	2.5765	
3.2217	2.9482	2.7908	3.6088	1.1407	3.1156	3.6512	1.8177	
2.725	1.6476	4.323	2.9691	4.027	4.6422	3.1983	3.5401	
2.6625	2.6256							
AMPD1	0	0	0	0	0	0	0.0438	0
	0	0.0795	0.0328	0	0	0	0.0067	0
	0	0	0.0581	0	0	0	0	
0.1146	0	0	0	0	0	0.0197	0.0109	0
	0.0229	0.0845	0	0.0113	0	0	0	
0.0354	0	0	0.0326	0	0	0	0	0
	0.0158	0	0	0	0.0491	0	0	
0.0799	0	0	0	0.0196	0	0	0	0
	0	0	0	0.0036	0.0104	0	0	0

	0	0	0	0.0207	0	0	0	0
	0.1571	0	0	0	0	0	0	0
	0							
AMPD2	3.3609	4.0113	4.2024	3.5211	4.6963	4.5897	4.5295	
4.8977	3.3726	3.4435	5.2542	4.6297	3.6857	4.4915	4.3464	
4.4933	4.974	3.3598	3.2757	4.7956	3.9566	4.6498	4.2762	
4.1759	4.21	5.2113	4.0366	3.5679	3.7498	3.4175	4.3381	
5.9132	4.6182	5.5989	4.8098	3.0013	4.4344	4.5244	3.8879	
4.4483	4.6233	4.9542	4.5634	3.5808	3.6446	3.8373	3.2183	
4.39	4.8813	3.5798	4.2921	3.7109	4.1589	4.4259	5.0643	
4.2249	4.2108	4.0636	3.7082	4.6809	4.0105	3.7544	3.3847	
4.7435	3.875	4.9714	4.0352	5.0974	3.1043	4.0434	4.3011	
4.3766	3.8997	4.5607	3.5998	4.0152	4.1634	3.8216	3.6997	
3.3638	3.8586	4.5051	3.8991	4.0998	4.5991	4.3902	3.6849	
3.7239	3.8341							
AMPD3	0.1581	0.122	0.1267	0.4467	0.4531	0.212	0.5515	
0.2413	0.8818	0.2243	0.3481	2.3878	0.1845	0.381	0.3624	
0.4531	2.6558	0.0374	0.2991	0.4685	0.201	1.2393	0.5368	
0.2203	0.2954	4.677	0.0505	0.168	0.2261	0.0463	1.3706	
0.2921	0.1648	0.362	0.3016	0.1849	0.2661	0.0344	0.0906	
0.6294	0.173	2.8276	2.4911	0.2093	0.6266	2.7233	0.1887	
0.2123	0.5202	2.1269	0.4432	1.362	0.186	0.0765	0.1307	
0.1841	2.5786	0.0691	0.3448	0.0943	0.0602	0.5603	0.2598	
0.2608	0	3.59	0.5699	0.4749	0.0941	0.0961	1.1586	
0.3519	0.1579	0.6805	0.1515	0.2826	0.3626	0.2643	1.0517	
2.1284	0.3375	0.3731	1.0076	0.2267	0.9851	0.1488	0.4319	
0.51	0.5421							
AMPH	0.327	0.0709	0.0654	0.0672	0.1212	3.2747	3.8895	
0.0573	0.5159	0.9185	1.925	0.0453	3.1472	2.594	0.1785	
2.8827	0	0.1249	2.3173	5.4665	0.9377	0.2821	3.0145	
3.4338	1.9418	3.8531	0.0933	0.1037	0.1811	0.0502	1.1064	
0.8736	3.443	0.0477	3.2036	0.6892	2.243	0.1075	0.0648	
1.759	0.0599	0	3.7361	2.6596	1.2625	0.5888	0.1365	
0.1853	0.0616	0.088	1.1144	0.0515	0.5191	0	0	
0.1195	0	4.9158	3.5248	2.8579	2.0119	3.7455	0.0774	
1.6088	0.284	2.672	0	1.1887	0	0.3965	0.0823	
2.6024	2.6632	0	2.8595	1.631	1.4029	0.3262	0.2831	
0.229	0.6366	0	3.4655	0.0714	0.0143	0.2831	1.273	
1.7926	0.6015							
AMT	1.4374	0.9017	0.2483	0.5994	0.2581	1.0671	0.6537	
0.7905	0.7101	2.402	1.2919	1.0206	1.2738	0.8476	0.9417	
0.929	0.9745	2.1134	0.5475	0.5017	1.1916	0.6531	0.4609	
1.2325	0.26	1.0987	2.5032	1.554	0.4659	2.2715	0.5554	
0.8648	0.3199	0.3954	0.8777	1.5785	1.6199	0.7251	0.9907	
0.6895	1.4099	0.5811	0.5951	1.2375	1.5591	0.8249	0.4528	
0.8963	1.4129	1.0237	0.9327	0.3661	0.8903	0.9911	0.3483	
0.2935	1.257	0.1991	0.5258	0.2038	1.0642	0.8279	0.7443	
1.3798	1.0054	1.1426	3.0291	1.181	1.0454	0.4666	0.8423	
1.174	0.5519	0.4955	1.7206	1.1649	1.0701	0.8249	0.7906	
1.0839	0.6834	1.5585	0.6639	1.0576	0.7695	1.0562	1.336	

1.5514	1.5757							
AMTN	0	0	0	0	0.0155	0	0	0
	0	0	0	0	2.9947	0	0	0
	0	0	0	0	0	0.0209	0	0
	0	0.4682	0.1805	0.1064	0	0	0	0
	0.0636	0	0	0	0	0	0	0
	0	0	0	0	0	0	0.0787	0
	0	3.6171	0	0	0	0	0	0
	0	0	0	0	0	0.0441	0	0
	0	5.1513	0	0	0	0	0	0
	0	0	0	0	0	0	0	0
	0	0	0	0	0	0	0	0
	0							
AMY1A	0	0	0	0	0	0	0	0
	0	0	0	0	0	0	0	0
	0	0	0	0	0	0	0	0
	0	0	0.0093	0	0	0	0	0
	0	0	0	0	0	0	0	0
	0	0	0	0	0	0	0	0
	0	0	0	0	0	0	0	0
	0	0	0	0	0	0	0	0
	0	0	0	0	0	0	0	0
	0	0	0	0	0	0	0	0
	0	0	0	0	0	0	0	0
	0							
AMY1B	0	0	0	0	0	0	0	0
	0	0	0	0	0	0	0	0
	0	0	0	0	0	0	0	0
	0	0	0	0	0	0	0	0
	0	0	0	0	0	0	0	0
	0	0	0	0	0	0	0	0
	0	0	0	0	0	0	0	0
	0	0	0	0	0	0	0	0
	0	0	0	0	0	0	0	0
	0	0	0	0	0	0	0	0
	0	0	0	0	0	0	0	0
	0							
AMY1C	0	0	0	0	0	0	0	0
	0	0	0	0	0	0	0	0
	0	0	0	0	0	0	0	0
	0	0	0.0281	0	0	0	0	0
	0	0	0	0	0	0	0	0
	0	0	0	0	0	0	0	0
	0	0	0	0	0	0	0	0
	0	0	0	0	0	0	0	0
	0	0	0	0	0	0	0	0
	0	0	0	0	0	0	0	0
	0	0	0	0	0.0294	0	0	0
	0							
AMY2A	0	0	0	0	0	0	0	0

	0	0	0	0.0304	0	0	0	0
	0	0	0	0	0	0	0	0
	0	0	0	0	0	0	0	
0.0645	0	0	0	0	0	0	0	0
	0	0	0	0	0	0	0	0
	0	0	0	0	0	0	0	0
	0	0.0285	0	0	0	0	0	0
	0	0.0182	0	0.0058	0	0	0	0
	0.0239	0	0	0	0.043	0	0	0
	0	0	0	0	0	0.0713	0	0
	0							
AMY2B	0.7113	0.4456	0.5709	0.7348	0.3528	0.6822	0.9694	
0.6208	0.5186	0.6455	0.942	0.7805	1.0129	1.1606	0.7102	
0.3403	1.5611	0.7817	0.9962	0.8144	1.0952	0.2544	0.3256	
0.3849	0.3679	1.3069	1.4449	1.0745	0.259	0.1559	0.5214	
1.4065	0.6779	0.8167	0.916	0.4302	0.403	0.6444	0.5792	
0.6365	0.4042	0.7078	0.2381	1.1339	0.8772	0.5017	0.332	
0.2908	1.1676	2.2073	0.8995	1.0386	1.0279	0.4831	0.4043	
0.8379	0.9185	0.3857	0.5817	0.5863	0.2844	0.716	0.3561	
0.9924	0.4408	1.8125	0.9194	1.0598	0.9134	0.3973	0.3381	
1.3355	0.594	0.6086	0.3245	0.6096	0.7643	0.2837	0.594	
0.6715	0.9761	0.4088	1.401	0.228	0.3666	1.2022	0.5049	
0.7922	0.8006							
AMZ1	0.8095	0.0518	0.3119	0.1405	0.0581	0	0.0464	
0.0361	0.1292	0.0149	0.5088	0.1937	0.9543	0.0658	0.2995	
0.0182	0.1689	0.2493	0.0814	0.0313	0.2749	0.0639	0.0319	
0.0594	0.0713	1.1456	0.0206	0.0346	0.0162	0.1058	0.0408	
0.0152	0.0754	0.0152	0.0069	0.035	0.0985	0.0232	0.0599	
0.0168	0.099	0.2503	0.0464	0.0175	0.3263	0.4917	0.2006	
0.0685	0.0917	0.4754	0.0648	0	0.1564	0.0112	0.0645	
0.1302	0.2789	0.3359	0.1355	0.0702	0.0041	0.0684	0.0019	
0.106	0.2299	0.0505	0.1123	0.0358	0.0647	0	0.0478	
0.0797	0.149	0.0296	0.1009	0.0756	0.1776	0.007	0	
0.0234	0	0.6558	0.2681	0.0099	0.0892	0.1938	0.1466	
0.2446	0.1116							
AMZ2	6.3712	5.5166	5.2899	4.7903	4.9838	5.6178	5.6649	
5.4307	5.7121	6.0387	6.1263	4.8631	5.6579	5.8973	4.6842	
6.6847	5.7992	5.7891	5.0649	5.5265	6.7164	4.6255	5.6543	
5.6431	5.1793	5.9096	5.7259	7.02	5.1139	6.2465	4.8906	
5.3188	5.6259	5.9501	5.7379	6.1126	6.3302	4.6867	4.8045	
6.2431	5.8135	4.9479	5.7246	5.4779	6.2178	5.9677	6.6708	
6.1859	5.5113	6.2711	5.5538	5.2275	5.9501	5.3916	6.18	
5.4891	5.6769	4.9621	5.7737	5.1175	5.8245	5.0132	5.1118	
5.2148	6.1915	5.7266	6.7018	5.6222	5.6939	5.4802	5.843	
5.6621	2.3789	5.0865	5.939	6.9047	5.5063	5.4686	5.7135	
6.3181	5.6802	5.4092	5.6762	6.1554	4.9231	6.0167	5.5233	
5.3663	5.1303							
ANAPC1	3.5589	4.1133	3.5493	3.9685	2.9595	3.1393	3.4181	
4.1525	3.9506	4.3511	3.6991	3.5057	3.2842	3.902	3.3204	
3.023	3.5309	3.9591	4.2758	3.1973	3.4512	3.1123	3.1656	

3.742	3.1504	3.1064	2.6963	3.4709	3.6769	4.098	3.117
3.3523	3.6351	2.9633	2.9549	4.3342	3.5903	2.8218	3.4645
4.3017	2.9668	3.0839	3.4927	3.4262	3.6468	3.4974	4.3503
3.4185	2.975	3.6649	3.4791	3.1703	3.8922	3.7231	3.6065
3.665	3.2827	4.0191	3.8186	3.9423	3.3854	4.1264	3.2857
3.4265	3.8757	2.5564	3.4811	2.7144	4.1478	3.7569	3.0399
3.4712	4.0564	3.5359	2.866	3.4644	3.6045	3.2137	3.5076
3.2329	3.9517	3.0415	2.8691	2.7867	2.2022	3.385	2.6435
3.0967	2.7994						
ANAPC10	3.1399	3.1002	2.9966	3.0802	3.2753	2.7727	3.101
2.8569	2.6342	2.6347	2.4404	2.9091	2.9014	3.1349	2.5301
2.3551	2.907	3.0901	3.2115	2.4278	2.7455	3.0764	2.265
2.8614	2.665	3.519	3.35	2.9021	1.9923	2.5212	2.7821
3.1819	2.6632	2.1617	3.1795	2.9499	2.4787	3.1745	2.8367
2.4592	2.543	2.8443	2.9772	2.4588	2.9492	3.7162	2.8306
2.6385	2.6431	3.9498	3.1726	3.1802	2.9318	3.5636	2.809
3.231	3.6478	2.8153	2.8307	2.6855	2.7196	3.2356	2.9243
2.6628	2.5394	2.6951	2.9431	2.922	2.7136	3.114	2.1291
2.3231	2.6136	2.4683	2.807	2.9083	3.0458	3.0086	2.2852
2.6724	2.8524	3.0671	2.2962	2.7303	2.7162	3.1222	3.0806
2.9633	2.691						
ANAPC11	6.2743	5.231	6.0151	3.3449	5.3926	4.7109	4.3416
5.3468	4.3918	4.8766	5.5006	4.7268	6.1334	3.7367	5.4748
5.3782	5.7473	4.0956	4.1123	5.0308	4.5863	5.486	5.3814
5.0581	5.0639	5.3068	4.5747	6.1245	4.8248	5.1855	5.5977
5.7688	5.1836	5.8372	5.2057	4.2866	6.4506	5.9931	5.6848
3.7905	5.7053	5.0435	4.9635	4.5765	5.2233	4.4306	4.3494
5.2036	5.8718	4.4817	5.2569	3.9662	3.8542	5.3415	6.5544
5.1573	5.0819	4.4624	5.284	4.2932	5.3848	5.2793	6.2815
4.7521	6.3652	5.3306	6.6148	5.7708	5.7747	5.4227	5.4561
5.2038	5.2573	5.0474	5.5203	6.2362	5.3705	5.3071	4.6636
4.4282	5.8918	4.3199	5.4826	6.3678	5.8608	5.3884	5.4099
6.1138	5.6942						
ANAPC13	5.0075	4.8198	4.5621	5.2605	5.3902	5.6939	5.3789
5.6692	5.0234	4.697	5.2056	5.1979	5.6167	5.7942	5.4089
4.7859	6.1664	5.054	5.4735	4.8447	4.3311	5.1686	4.9212
4.9056	4.7985	5.8515	6.3001	4.8797	5.3415	4.6985	4.8792
5.1105	5.4595	5.4694	5.2627	4.7014	5.0247	5.0043	5.3178
6.6863	4.9192	5.4884	5.485	5.5973	6.3714	5.7582	5.6873
5.2192	5.6299	6.5151	5.7571	5.9079	4.9886	6.1068	5.5795
5.9474	6.2531	5.4917	4.588	5.7274	5.3852	4.9963	4.5579
5.8485	4.9275	5.7105	4.6395	5.7752	4.9891	5.0119	5.1108
4.8062	5.5309	5.1797	5.4566	4.9521	4.7771	5.4493	4.9324
5.0046	4.3586	5.1779	4.6528	5.7001	5.7902	5.213	5.4933
5.7046	5.1287						
ANAPC15	3.7736	5.0416	3.2865	3.5933	4.4101	4.3828	2.7872
3.6301	3.9084	4.4896	3.4091	3.3039	4.1521	4.0526	3.4211
3.4846	3.7563	2.9157	4.2002	4.1154	5.5715	4.2557	4.2494
4.5319	3.7174	3.758	3.408	3.6477	4.3289	3.7423	4.3921
3.0516	3.8953	3.6599	4.0109	4.3837	2.9339	3.5535	4.0873

3.8619	3.5626	3.4296	3.9787	3.3642	3.8484	4.4118	4.0637
3.8862	3.1358	4.8777	3.3807	3.3463	3.6788	3.3769	4.4101
4.7975	3.7619	4.0301	4.4869	3.7641	4.0184	4.1165	4.3936
4.1088	4.3027	3.1602	3.9855	4.329	2.9908	3.7159	4.1803
3.88	4.0849	3.5689	4.1565	3.796	5.5188	3.7723	3.7006
4.2558	5.4516	4.0146	4.4937	3.4138	4.1048	3.6731	4.1474
4.2791	4.4394						
ANAPC16	4.2548	4.6696	3.9386	4.6778	3.6232	4.306	3.7676
4.2016	3.8879	4.4556	3.4509	3.9058	4.4038	4.3088	3.4353
3.3978	3.7939	4.4923	3.7391	3.8823	3.3582	3.7459	3.993
3.7789	4.0032	3.565	3.9564	4.3635	2.8741	4.1726	3.6081
4.0269	3.4223	3.532	3.9155	4.1217	4.6628	3.6909	4.4316
3.217	3.9967	4.2122	4.0652	3.6448	3.8251	3.8174	4.3205
4.1271	4.1031	4.273	4.0387	3.8684	4.2583	4.5495	4.5768
4.4319	3.953	3.3245	3.7262	3.2911	3.243	3.6496	4.3022
4.0788	4.139	3.6668	4.1632	4.0848	3.7345	4.0493	3.7434
3.5678	3.0172	3.5281	3.4443	3.9219	3.5488	4.0192	3.8698
4.621	4.08	3.7478	3.6359	3.8481	4.0952	4.1263	4.4498
4.0706	4.0412						
ANAPC2	3.2613	3.0305	3.5859	2.5708	3.6492	3.3481	2.9799
4.2257	2.8722	2.99	3.7768	2.8869	3.8802	2.6804	3.3048
2.9783	3.3523	2.7391	3.2241	3.958	3.7825	2.6747	3.0247
3.2731	3.2899	3.2245	1.9419	3.3929	3.2142	2.7516	3.2861
3.691	2.7766	3.0574	3.3155	2.4588	3.7581	3.8693	4.0489
3.0095	3.5031	3.2896	2.8241	3.0593	2.7267	2.6596	2.5243
2.7476	3.4907	2.6149	2.8528	2.8413	2.6549	2.9618	3.6165
3.5377	3.9795	3.5079	3.1918	3.5447	3.6815	2.4552	3.1158
3.2292	3.3327	3.8739	2.6608	3.2187	3.6759	4.0789	3.1394
3.0553	3.1915	3.3453	2.9502	2.4519	3.1017	3.1676	2.8732
2.6586	3.0445	2.8108	3.1274	4.0826	3.1142	3.3345	3.007
3.1614	3.2413						
ANAPC4	3.2332	2.9771	2.9359	3.6723	3.5803	3.2404	2.538
2.8833	3.3542	3.3806	2.5714	3.2176	2.4111	4.1778	2.7179
3.4694	2.9447	3.6979	3.3294	2.8154	3.3765	3.2692	3.0028
3.3866	3.4234	3.5796	3.3224	3.1478	2.5524	2.8262	3.3988
3.0039	3.1028	3.1123	3.0016	3.5307	3.7068	2.7692	2.7614
3.6013	2.1722	3.6198	3.0226	3.6413	3.1543	3.5249	3.4623
2.9888	2.4897	4.285	3.5858	3.7763	3.4686	3.5054	3.1923
3.0901	3.1264	2.8855	3.1642	2.8933	3.1552	3.2931	3.0985
2.982	3.0646	2.9952	3.082	4.0801	2.7421	3.0755	3.0277
3.27	3.0097	3.1896	3.5583	4.5316	3.0917	2.9081	2.4907
3.3659	3.289	3.3097	2.9975	2.0537	3.7156	3.635	3.3028
3.4433	3.4267						
ANAPC5	5.5947	5.7016	6.3033	5.8938	6.1748	5.6014	5.8642
6.2054	6.1076	6.1362	6.0179	5.831	4.7329	6.8305	5.4655
6.2555	5.8866	6.4014	6.5756	5.9228	6.1428	5.744	5.8011
6.0046	5.598	5.2517	5.0812	6.1748	6.0141	6.1362	5.4516
6.1865	6.0083	6.3201	6.0072	6.2083	6.1125	6.0743	5.876
6.1282	5.6699	5.6345	5.3792	6.7106	5.5516	5.7043	5.816
6.1257	5.7933	6.1375	5.9093	5.5811	6.3107	5.8166	5.7013

6.5286	5.2971	6.1337	5.7914	5.643	5.7751	6.1843	5.2418
6.2336	6.2131	5.3969	5.911	6.0034	5.7951	4.9935	6.1903
5.7673	5.4267	6.113	5.6582	6.1086	6.0905	5.849	5.3834
6.2749	6.0235	5.7845	5.4063	5.4792	5.5626	6.4811	5.6092
6.139	5.8495						
ANAPC7	4.5882	4.5722	4.8626	5.1342	4.6499	4.2305	4.5035
4.7941	4.6879	4.7256	4.678	4.1588	3.7478	5.2618	4.2053
4.0598	5.2618	5.3134	5.5906	4.5447	4.5166	3.5083	3.6249
5.0494	4.4591	4.2038	3.7411	4.84	4.2688	4.6494	4.5016
4.9015	4.8307	3.7389	4.2476	5.1763	4.8669	4.6361	4.2562
5.0863	4.2702	4.3487	4.137	4.4471	4.3522	4.9469	4.9888
4.8139	4.0314	5.6187	4.3531	4.5332	5.1824	4.5302	4.6623
5.125	3.9772	4.8047	4.7624	4.2917	4.8137	5.0939	4.7957
4.5232	5.241	4.183	4.6488	4.4951	4.5878	3.8946	4.1238
4.3749	4.2943	4.3843	3.8063	3.5483	5.1335	3.9336	5.6231
4.7516	5.0351	4.3142	4.3593	4.0185	4.6052	4.9818	4.1193
4.3317	4.1365						
ANG	1.5688	1.2478	1.0328	1.2258	1.3465	2.8622	2.1898
2.1751	2.2325	2.0453	2.8691	2.596	1.8652	1.5922	1.9035
3.2585	1.7004	1.7177	1.3418	2.5819	2.1332	1.3503	2.4706
1.9656	1.1385	1.3694	3.3125	3.2615	0.8384	2.7098	5.7899
2.5845	2.1238	3.4008	1.7717	2.677	1.7289	1.5254	1.6191
2.4719	2.4445	2.2785	1.964	2.6637	1.1352	1.1466	2.0044
2.575	3.0335	1.2388	2.4128	2.4225	2.0216	0.2586	2.234
1.544	1.8808	1.4483	0.7412	1.2373	1.8867	2.7124	1.1407
2.5669	1.7769	3.071	3.6675	2.9053	1.1291	2.2267	2.213
2.5573	1.3267	2.1251	3.4424	1.8353	2.4306	2.1664	3.0422
3.27	2.5121	1.9996	3.0766	0.9956	1.4233	2.3013	3.0525
2.3217	2.7886						
ANGEL1	3.5788	2.7741	2.8744	2.496	1.9755	2.7857	2.6233
3.0664	3.1957	2.714	2.9625	3.5245	2.4107	2.6912	2.7622
3.1364	2.3801	3.9449	2.7957	2.8756	2.9163	3.1651	3.9011
2.2901	2.2582	3.1714	3.4443	3.2452	3.6901	3.7446	4.9351
2.8053	2.9503	3.0483	3.2236	3.2055	2.6944	3.5946	2.3661
3.5722	4.127	2.9163	2.3739	2.6986	3.3226	2.1174	3.4391
3.2314	2.8835	2.8434	2.4539	3.6218	2.8601	2.628	3.3381
3.063	3.0575	2.7249	2.0482	2.4336	2.9618	3.2906	3.354
2.0378	2.643	2.7368	3.2735	2.9369	2.988	2.2185	3.1371
2.6644	3.2492	3.1953	3.3225	4.2596	2.2214	2.9587	3.9255
3.1561	2.8243	2.2813	3.2143	3.9305	2.0482	2.2994	2.7119
2.4418	2.5521						
ANGEL2	3.2563	3.0049	3.1822	3.9963	3.7418	2.9087	3.799
3.1034	2.6038	3.5481	1.8503	2.8304	3.3039	2.9267	3.3306
3.1921	3.0747	3.8573	2.9631	2.8507	3.0597	3.1423	3.6543
3.3043	3.46	3.4258	3.2756	3.0624	3.3622	3.1494	3.5353
3.1753	2.9668	2.5404	3.0858	3.2682	3.789	2.4749	3.8557
3.278	3.307	2.9358	2.8013	3.4941	4.0126	3.296	3.4063
3.2301	2.4338	3.8839	3.3379	3.7716	3.415	3.5659	3.4864
3.3703	3.1615	4.0705	3.1815	3.8994	3.5621	3.0324	3.2669
3.2457	3.5397	2.8932	2.9428	3.323	2.7006	3.4164	2.6739

3.2665	3.0161	2.0931	3.7207	3.0972	3.3095	3.6948	2.9279	
2.5124	2.8221	3.0024	2.593	3.793	3.203	3.2614	2.8838	
3.0253	3.1059							
ANGPT1	0.1462	0.0595	0	3.9132	2.3364	2.0252	0.851	
2.2829	0.2274	0.5658	1.1729	2.6024	1.8516	0.8035	3.7311	
1.1278	3.1241	1.2017	0.0828	0.4991	0.1814	3.4815	2.9979	
2.2939	5.154	2.4313	0.8698	1.6611	3.6368	0.0154	2.526	
0.201	3.5584	1.9305	2.9121	1.6854	2.3969	1.6962	0.692	
4.364	2.8756	3.8303	1.6562	1.0649	1.341	5.0108	2.7961	
1.9885	3.2829	0.138	0.0327	1.9081	0.2011	0.6057	2.4987	0
	0.9981	1.8125	3.633	0	4.2183	2.9249	2.5416	
2.1025	0.029	1.4421	0.1002	3.6923	0.631	2.7139	2.6822	
0.2769	0.3363	2.7321	2.6494	1.6941	0.1001	2.3321	0.8429	
2.1487	0.0163	2.1347	3.463	1.1233	2.4046	0.5183	0.3718	0
	0.1093							
ANGPT2	0.0451	0.0488	0.0652	0.0396	0	0.0238	0.3927	
2.4955	0.1269	0.1545	0.0037	2.3012	0.0744	0.1704	0.0187	
0.0116	0.3603	0.2157	0.1092	0.0206	0.1071	0.2697	8.00E-04	
0.1036	0.4589	0.4897	0.2089	0.0652	0.4475	0	0.0072	
0.0488	0.987	1.2762	2.3419	0.0125	0	0.0922	0.0193	
0.6872	0.0598	0.0067	0.0524	0.0019	0.1298	5.00E-04	0.1066	
0.0498	0.0812	0.1638	2.1494	0.0309	0.6447	0.192	0.1511	
0.2267	0.1455	0.1521	0.1564	0.4162	0.1303	0.2014	0.1133	
0.2699	0.1761	0.1116	0.2376	0.1417	0.0978	0.5757	0.5729	
0.1414	0.3115	0.1355	0.1448	3.8811	0.1069	0.0872	0.1665	
0.1483	0.2346	0.1518	0.1292	0.1852	0.2777	0.6046	0.195	
0.8879	0.425							
ANGPT4	0	0	0.0459	0	0.0217	0	0	0
	0	0	0	0	0.0577	0	0	
0.0163	0.2161	0	0	0	0	0.011	0.1056	0
	0	0	0	0	0	0.0333	0	0
	0.0612	0	0	0	0	0	0	0
	0	0.0208	0.0469	0	0	0.0321	0	
0.101	0.0658	0	0	0	0	0.0212	0	0
	0.1625	0	0	0	0	0.0203	0	0
	0	0	0	0.0065	0	0	0	0
	0	0	0	0.0323	0	0.0317	0.0804	0
	0	0	0.0454	0	0	0	0	0
	0							
ANGPTL1	0	1.0924	0	1.5123	0.5964	0.1407	0.5237	
0.483	1.0972	0.2035	0.0774	0.5732	0.0571	0.1393	0.0783	
0.302	0.0703	0.1034	0.6201	0.0659	0.2235	0.3918	0.716	
0.5214	0.2122	0.6356	0.7286	0.1247	0.0283	0.2178	0	
0.0178	0.1393	0.1915	0.9046	0.1135	0.0352	1.2083	0.1574	
2.0733	0.3152	2.0552	0.1869	1.0298	0.1488	0.0355	1.1587	
0.8015	0.0985	0	0.5989	0.2437	0.1605	0.7082	0.0908	
0.6419	0.0571	0	0.005	0	0.6639	0.8659	1.7044	
0.2486	0	0	0.079	0	1.0904	1.0964	0.1974	
0.0367	1.1788	0.0832	0.4127	0.0193	0.7333	1.2308	0.4178	
0.8873	0.6431	0.083	0.02	0.5559	0.0304	0.047	0.0295	0

	0.071							
ANGPTL2	3.9166	5.4329	3.0839	0.0919	2.5746	0.317	3.1923	0
	5.026	1.7136	5.0297	0	4.4233	1.1144	0.196	0
	0.0668	4.2568	3.2928	0	6.0309	1.935	3.7703	0
	4.1604	2.8851	0.556	0	3.246	4.3925	0.0721	
3.2339	0	3.1601	0	1.8428	0.048	0.0662	1.1379	
3.7125	1.7348	4.2613	3.2171	0	1.4791	3.1002	2.2889	
2.5517	4.4928	1.996	0	3.3891	0	2.6611	0.2115	
2.1776	2.6395	2.3299	4.04	1.0488	0.1815	4.4197	0.1715	
0.2447	2.9173	4.5693	0.3276	3.6493	1.5793	3.7158	2.2858	
6.4064	4.5192	1.3841	0.1418	0.3204	0.3752	0.1329	3.6749	
1.4216	4.4961	1.1686	0.8864	2.0692	3.5459	0.2106	5.1308	
4.525	5.1707							
ANGPTL3	0	0	0.0274	0.0214	0.0144	0.0333	0.0189	
0.0052	0.0517	0.1232	0.0069	0.0054	0.0268	0.0229	0.0443	0
	0.0268	0.073	0.0803	0.0388	0.0276	0	0	0
	0.0021	0.0141	0.0365	0	0.0288	0.0275	0.0049	0
	0.0593	0.0084	0	0.0049	0.0014	0.0204	0	
0.0293	0.008	0	0.0449	0.1069	0.0161	0.0086	0.0268	0
	0	0	0	0.0202	0.0437	0.0194	0	0
	0	0.0259	0.0809	0	0	0	0	
0.1151	0	0	0.0427	0.0247	0.0145	0	0	
0.0192	0.0211	0	0	0.0302	0	0	0	0
	0.0363	0.0118	0	0	0	0.0764	0.0203	
0.0251	0							
ANGPTL4	0.3457	0.2672	1.0374	0.4573	4.0751	1.2998	0.5373	
0.3424	0.232	0.2009	0.8759	1.445	6.72	0.6509	5.089	
4.0992	1.6318	0.4225	0.6749	2.4403	0.4378	4.2278	2.0124	
1.9313	3.1535	4.3963	3.1002	0.0745	2.3478	1.2838	4.1511	
3.1579	0.8557	0.7962	1.5842	0.3794	1.8551	0.1411	1.0444	
1.0018	0.2378	1.828	0.2135	2.3332	1.7609	3.1859	0.4501	
0.4971	3.0415	1.3574	1.55	1.3162	0.7556	0.709	2.2447	
0.796	1.9092	0.2968	1.3433	1.1077	1.2419	1.253	0	
0.3275	0.1835	5.3057	0.3757	3.9083	0.2234	2.1444	4.4102	
4.4312	1.4641	3.2569	4.0564	0.4525	0.8506	0.1624	0.2838	
0.0993	0.3477	4.131	5.7812	1.0954	2.9921	0.4662	1.0577	
0.2183	0.2646							
ANGPTL5	0	0	0	0	0.0125	0	0.0387	0
	0	0	0	0	0.0335	0	0.0794	0
	0	0	0	0	0	0.0382	0	0
	0	0.0082	0.0786	0	0	0	0.0689	
0.0081	0	0	0.0011	0.0169	0.0037	0.0234	0	
0.171	0	0.0013	0	0	0	0.0121	0	
0.0383	0.0135	0	0	0.0518	0	0	0	0
	0.0515	0	0	0	0.0929	0	0	0
	0	0.0583	0	0	0	0	0.0206	0
	0	0.0021	0.0264	0	0	0	0	0
	0	0	0.0224	0.0663	0	0.0575	0	0
	0							
ANGPTL6	2.218	2.0097	1.9634	0.7974	1.8159	2.3468	3.4233	

2.1649	2.3981	2.5579	3.3437	2.0556	2.0194	2.281	3.1999	
2.5448	1.9297	2.4171	2.2261	2.1481	2.5489	2.0568	2.3695	
2.3558	1.1444	3.7936	1.914	1.9371	2.1545	2.5102	2.2651	
1.9844	2.7387	3.6687	2.8161	1.9898	2.4525	2.4646	2.4029	
2.3301	2.6511	2.3563	2.6135	2.4162	3.2393	1.7459	1.2159	
2.7462	2.7574	2.092	2.2454	1.7493	2.7514	3.0084	2.0361	
2.9729	1.0973	1.2517	2.62	1.151	2.0404	2.0471	0.8574	
2.3424	1.9839	3.4552	2.3786	2.439	1.735	1.7134	2.7658	
2.4584	2.2236	2.1773	1.9197	1.4039	2.8235	1.2734	2.6313	
2.3505	2.1357	1.7661	3.4221	2.38	2.1941	2.4381	2.6661	
1.9921	2.1916							
ANGPTL7	0.1686	0	0	0.046	0	0.6781	0.018	
0.0377	0.8197	0.0975	0	1.3703	0.0177	0.8683	0.5307	
0.0491	0.0462	0.0398	0.0627	0	0.0697	0.1215	0.0325	
0.0838	0.062	0.0196	0.3734	0.0488	0.2909	0	0	
1.1786	1.3811	0	0.5223	0	0.0655	0.0343	0.1495	
2.3096	0.1134	0.1071	0.0394	0.2633	0.1114	0	0.0624	
0.0555	0.0416	0.0689	0.1746	0.0294	0.5439	0.4848	0	
0.0901	0.0124	0.0345	0.0539	0.0322	0.0433	0.4287	0.0451	
0.3974	0.2764	0.2104	0.1374	0.0155	0.0346	0	0.2154	
0.0533	0.0316	2.1104	0.0015	0	0.047	1.2906	0.944	
0.6082	0.247	0.3777	0.0375	0.4488	0	0.2785	0.1177	
0.1274	0.1202							
ANHX	0	0	0	0	0	0	0	0
	0	0	0	0	0	0	0	0
	0	0	0	0	0	0	0	0
	0	0	0	0	0	0	0	0
	0	0	0	0	0	0	0	0
	0	0	0	0	0	0	0	0
	0	0	0	0	0	0	0	0
	0	0	0	0	0	0	0	0
	0	0	0	0	0	0	0	0
	0	0	0.0137	0	0	0	0	0
	0	0	0	0	0	0	0	0
	0							
ANK1	0	0.0522	0.0565	0.1357	0.0915	0.055	0.0681	
0.1684	0.0288	0.0327	0.0515	0.0609	0.9717	0	0.0582	
0.1435	0.11	0.0885	0	0	0	0.0279	0.1041	
0.026	0.3037	0.8091	0.0692	0	0.1831	0	0.2936	
0.0279	0.0677	0.0332	0.0371	0.0304	0	0	0.0941	
0.0664	0.0199	0.662	0.997	0.0677	0.9933	0.1585	0	0
	0.4248	0.1534	0.0978	0.0669	0.0611	0	0.0706	
0.6976	0.2619	0.0067	0	2.00E-04	0.0413	0.0102	0.1047	
0.0106	0.0744	1.4164	0.0376	0.7559	0.3929	0.0821	0.8321	
6.00E-04	0	0	0.3508	0.2256	0	0.0421	0	0
	0	0.4174	0.0191	0.0654	0.251	0	0.0472	
0.0604	0.0462							
ANK2	4.5392	2.8156	2.4126	2.7445	0.9483	3.7623	2.5231	
2.5782	4.2721	3.5225	2.0257	2.9343	2.3207	2.7424	1.4236	
3.2554	3.3277	3.5966	2.5547	2.727	2.2187	2.1737	1.6595	

3.7313	1.3988	1.8867	1.96	2.5358	1.4309	2.7614	2.5892	
3.1519	3.1597	2.8681	3.5673	2.556	2.7585	3.9495	4.0225	
2.9678	3.0376	2.6765	2.0801	2.0582	1.8043	1.6509	4.66	
3.506	1.0278	2.6559	3.688	1.9391	3.2489	3.422	2.4712	
2.775	3.7671	1.6036	2.8973	1.891	2.1038	3.7379	4.8995	
2.5942	3.7493	2.8748	2.9227	4.7792	2.6446	1.0219	3.1483	
1.3157	1.9521	3.9243	4.209	2.7821	1.9769	4.7863	2.723	
2.4671	3.2855	2.4402	2.464	3.0245	1.5119	2.7659	4.4028	
3.3167	2.7988							
ANK3	0	1.89	0.3068	2.2751	2.7765	1.2461	2.9762	
1.3786	1.4601	1.6775	1.9008	1.5428	0.1384	1.6995	3.4106	
1.2461	2.7237	0.0744	1.7813	0.9142	1.3631	2.6805	2.7966	
3.5178	1.1927	0.2131	0.95	0.0819	3.5627	1.1345	0	0
	1.7261	0	2.3114	1.9172	0.084	0.2363	2.1707	
5.8531	0.3827	2.5966	0.2563	1.7311	0	1.3607	1.4187	
1.0966	3.6447	0.7547	1.2251	1.7734	0.2673	1.3513	0.2137	
2.5568	2.8038	4.9508	2.7999	4.4846	2.7173	2.7183	1.5683	
0.4712	0.3152	0.9886	0.537	0.3356	0.2991	1.2919	0.351	
0.593	1.7629	2.2327	1.336	0.5107	0.4843	2.0429	0.2059	
0.9835	0.9004	0.3053	0.3404	2.9663	1.6092	0.3651	0.7845	
0.4881	0.5807							
ANKAR	0.2204	0.3725	0.3944	0.559	0.2739	0.2514	0.5519	
0.7545	0.4735	0.5043	0.3582	0.2466	0.5544	0.6545	0.179	
0.3173	1.2526	0.3567	0.5327	0.7933	0.4434	0.3821	0.2661	
0.2602	0.4107	0.6493	0.7129	0.4213	0.303	0.264	0.5395	
0.3582	0.3667	0.5788	0.5754	0.2862	0.5707	0.2678	0.1211	
0.7368	0.5187	0.5935	0.6223	0.5385	0.3243	0.5749	0.577	
0.7329	0.7247	0.7362	0.4567	0.5493	0.5845	0.6468	0.8761	
0.6044	0.337	0.3325	0.689	0.3153	0.2902	0.4993	0.3919	
0.2861	0.8697	0.4461	0.1897	0.4171	0.2829	0.458	0.4243	
0.5234	0.7436	0.1683	0.1586	0.7561	0.8825	0.2747	0.3333	
0.5876	0.6818	0.2635	0.623	0.4768	0.5353	0.4551	0.3382	
0.3982	0.4791							
ANKDD1A	1.2125	1.7457	1.3053	0.5505	0.6249	0.7358	0.6639	
2.3461	1.5059	1.8575	2.1137	1.2867	2.0323	1.685	1.1689	
1.0051	1.0012	1.4897	1.6717	1.1552	1.4872	1.2778	1.2362	
1.4069	1.0753	0.4945	2.17	1.304	1.1011	2.3052	1.8065	
1.7862	1.2549	1.7199	1.1159	1.2994	0.6961	2.0182	1.4056	
1.0025	2.5278	1.3914	0.8026	1.2974	0.603	0.5404	0.7427	
0.8171	1.7248	1.0972	1.4279	1.3001	1.6275	1.5306	2.1463	
1.2972	1.9162	0.5129	1.4498	0.5564	0.7263	0.858	1.1722	
1.9262	1.0457	0.5482	1.0669	1.4631	2.1529	0.7187	0.9033	
0.8725	1.2822	1.1455	1.1413	2.145	1.3628	1.1093	2.4179	
1.5246	1.4154	0.7055	1.4466	1.5706	1.1687	1.645	1.1777	
1.6822	1.9127							
ANKDD1B	0.2186	0	0.0519	0.0978	0.2408	0.6875	0.2746	0
	0	0.0171	0.0116	0.011	0.1121	0.2076	0.3235	
0.2126	0.3463	0.0404	0.2406	0.2812	0.0491	0.1985	0.2109	
0.2091	0.2421	0.0186	0.1177	0	0.6766	0.0268	0.0202	
0.0349	0.2205	0.0374	0.5428	0.0189	0.0338	0.0685	0.1026	

0.4695	0.1253	0.0639	0.0406	0.2393	0.0221	0.1404	0.1576	
0.0699	0.3625	0.136	0.2179	0.5228	0.3533	0.2639	0	
0.4352	0	0.4908	0.1261	0.5435	0.1681	0.0509	0.0851	
0.1144	0.1044	0.0656	0.6459	0.0925	0	0.1831	0.2479	
0.2285	0.2782	0	0.2078	0.0313	0.0874	0.1904	0.0121	
0.3311	0.0214	0.1237	0.1204	0.4217	0	0.278	0.2175	
0.119	0.2451							
ANKEF1	0.0283	0.8523	0.8698	1.0998	1.233	0.2544	0.1569	
1.5493	1.6232	0.8782	0.6636	0.8189	2.8673	0.1056	1.162	
0.6636	1.7319	0.0174	1.001	1.1693	1.0657	0.9792	1.7869	
1.8402	1.0102	2.4976	0.3734	0.0473	1.5798	1.9322	0.0036	
0.9604	1.7337	0.0442	1.2238	0.4992	0.0631	0.6061	0.4401	
1.8301	1.6026	2.7895	0.0198	1.4547	1.0403	2.671	1.7546	
0.3111	2.4372	0.931	0.7906	1.7618	0.1868	1.3941	1.2515	0
	1.668	1.0978	0.9637	0.8273	0.1332	0.7763	0.2021	
1.7554	0.221	2.0056	0.2405	1.8045	1.5402	2.7802	1.1265	
0.6438	2.5394	1.2384	1.8845	0.5579	0.023	2.4146	1.0966	
0.102	0	1.2285	0.3576	1.5164	1.3318	0.1381	0.475	
0.5026	0.843							
ANKFN1	1.0334	0	0	0.9084	0.4713	0.083	0.2941	0
	0.1385	0.1352	0.2366	0.0615	2.1596	1.3491	0	
0.0903	1.0015	0.0363	0	0	0	0.5815	0.5461	
0.4925	0	0.2873	0	0.0084	0.1846	0	0	
0.1575	0.1963	0.2322	0.5782	0.0167	1.6447	0.0468	0.1227	
0.1445	0.1285	0.0903	1.0075	0	0.4642	0.4159	0	
0.1116	0.1779	2.586	0.2344	0	0	0.901	0	0
	0	0.1382	0.5647	0.6815	0.4198	0.2233	0	
0.1459	0	0.1704	0	0.2823	0.1449	0	0.8744	
0.2035	0	0.2048	0	0	0	0	0.7182	0
	0.1578	0	0	0.1661	0	0	0.075	0
	0							
ANKFY1	3.7293	4.0258	3.534	4.1798	3.5413	3.9211	4.1891	
3.4809	4.2337	4.0193	2.9094	3.232	3.339	3.3219	3.896	
4.3216	3.3758	4.1167	3.4385	3.4102	4.5992	3.0727	3.5825	
3.4118	3.0465	4.4564	3.153	3.854	3.8543	3.4506	4.7769	
2.4726	3.7385	3.2006	4.2337	3.8489	2.9656	4.0075	3.2181	
4.0357	3.6924	3.9615	3.3061	3.8304	3.9698	3.8748	3.3904	
3.5982	2.9346	3.3305	4.0445	3.4532	3.6208	3.4331	4.1467	
3.0888	3.1078	3.344	3.7	3.6527	3.3864	4.1284	3.2363	
4.6504	3.7916	4.3582	3.6279	4.6353	3.0243	4.0417	4.0378	
3.6543	3.9814	3.9372	4.3354	3.2957	3.6884	4.259	3.8857	
3.8365	3.788	3.659	3.0069	3.6735	3.0192	3.4068	4.3771	
4.2714	4.2325							
ANKH	3.7151	2.8629	3.0066	4.236	4.4021	2.8017	2.4323	
3.7541	2.3842	2.3757	3.4904	4.4482	2.961	2.0674	4.8877	
2.9694	3.1023	2.7256	3.0005	3.7044	2.8464	4.5867	4.867	
3.3648	5.3904	2.4328	5.3634	0.6723	5.2464	4.7207	4.5316	
2.0119	2.3952	0.677	5.3357	2.7867	2.3274	3.5506	6.2242	
3.5516	2.9114	5.9369	5.2188	3.1691	2.5977	2.9106	2.4935	
2.9216	4.6753	1.8666	2.632	4.8068	2.3241	2.4969	2.4669	

2.5536	3.129	2.8521	3.1609	3.0539	4.9323	3.2747	3.6525	
2.293	2.128	1.687	2.6365	3.1069	2.476	4.9644	2.9617	
4.1577	2.4979	4.6017	4.705	6.614	2.0065	5.5639	2.654	
3.4984	2.2717	5.4376	3.6425	5.5252	4.5868	1.9935	2.0868	
1.8101	1.9134							
ANKHD1	4.4317	4.3997	4.2784	4.3535	4.3614	4.5888	4.9198	
4.7937	5.6592	5.1069	4.3183	4.7831	5.3234	5.1139	5.5525	
4.6768	4.8662	4.021	5.7666	4.9637	4.6035	4.5985	4.3927	
4.9171	4.7861	4.28	5.4477	4.5893	4.742	4.8148	4.8829	
4.8078	4.4618	4.8287	4.7238	5.2374	3.9878	4.8545	4.2107	
5.0048	4.489	4.2746	4.0685	4.2247	4.9999	4.7567	5.2108	
5.024	4.7305	4.6012	4.9266	4.8804	4.9488	5.2394	4.2331	
4.6156	4.804	4.1219	4.8244	4.2482	4.3317	4.6357	5.0679	
4.8122	4.6994	4.4701	4.7397	4.3994	4.9059	4.4184	4.1893	
4.9509	4.7796	4.8981	3.8785	5.0867	5.4422	4.8074	4.9308	
4.832	5.1365	4.6404	5.3031	3.9315	3.5874	4.1497	4.098	
4.3038	4.6073							
ANKIB1	3.3787	3.6139	2.9172	4.4426	4.1095	4.8638	5.0066	
4.0297	4.603	3.8701	4.9638	3.7532	4.1855	4.5124	3.8533	
4.1073	3.826	4.9897	4.5006	3.8508	3.4648	4.5142	4.835	
4.5549	4.8756	4.4955	3.612	3.8268	4.8663	4.3781	4.0661	
4.2675	3.8794	4.9287	5.0844	4.1113	3.9192	3.4736	3.8062	
4.3093	4.4098	4.4033	4.5585	4.5392	4.56	4.3522	3.6528	
4.1561	5.2876	3.7132	4.657	4.4882	3.8845	3.5311	3.9635	
3.5311	3.3612	3.5722	4.8891	3.9948	4.3109	4.3246	4.0977	
4.4144	4.2509	4.9803	4.1455	3.9631	3.3075	4.4662	4.8342	
4.3138	4.3824	3.9352	4.6357	4.1109	3.4223	4.7294	3.768	
4.2242	4.0404	4.0947	2.1391	4.6548	4.2993	3.7788	3.8238	
3.3468	3.5174							
ANKK1	0	0	0	0	0	0	0.1243	0
	0	0	0	0.0056	0.0676	0	0	
0.0873	0	0	0	0	0	0	0.0237	0
	0	0	0	0.0427	0.0515	0.0229	0.0266	
4.00E-04	0	0	0	0	0	0	0	
0.047	0.1176	0.0908	0.1344	0	0.2036	0.0672	0	0
	0.0756	0.0675	0	0.3447	0	0	0	0
	0	0.0291	0	0	0	0	0	
0.0355	0	0.2913	0	0.0339	0	0	0.0175	
0.0182	0	0.0067	0	0	0.0275	0	0.0088	
0.0465	0	0.1177	0	0.0527	0.1036	0	0.0277	0
	0							
ANKLE1	1.2643	1.1617	0.181	0.3683	1.5866	0.3751	0.1491	
0.4108	0.0565	0.5653	0.3822	0.9012	0.377	1.0944	0.2892	
0.0804	1.6181	0.9218	0.6427	0.0719	0.4052	0.9584	0	
0.3132	0.1511	0.4069	0.3559	0.2761	1.0419	1.0681	1.0233	
1.0561	0.5205	0.1173	0	0.6841	1.1944	0.0068	0.7224	
0.2368	0.5659	0.5163	0.8081	0.7567	0.6721	1.3922	1.5778	
0.0624	0.3904	0.1389	0.1369	0.9467	0.2086	2.1099	0.8966	
0.4562	0.2275	1.5164	0.3567	1.1066	0.1999	0.421	0.1882	
1.1814	0.6674	0.1831	0.3769	0.1962	1.5842	0.9455	0.7194	

1.0602	0.201	0.5534	0.5337	0.4606	0.3586	0.1793	0.1388
0.286	0.3016	0.2839	0.1639	0.1691	0.9829	0.2389	0.1375
0.3177	0.3974						
ANKLE2	5.0119	4.1164	4.8785	4.7364	4.1785	3.8948	5.0266
5.3279	4.7125	4.4781	4.1456	3.5788	4.1405	5.0051	3.9748
3.8452	5.0159	5.0278	5.1762	4.6053	4.7584	4.2092	3.9726
4.5349	4.7648	4.75	4.2607	4.2985	4.1312	4.1123	3.9295
4.159	4.4993	4.1199	4.0964	4.4757	4.7777	3.7587	3.8546
4.2544	3.6766	3.997	4.2376	4.5958	4.6898	4.3687	4.4099
4.3825	4.6553	4.9077	4.1936	4.0134	4.9605	4.2155	3.9945
4.2068	4.3309	4.7243	4.5982	4.2255	4.5029	4.9693	4.258
4.6004	4.4477	4.9977	4.1162	4.7342	4.99	4.1591	4.3415
4.4376	4.4911	3.5849	4.096	3.6776	4.3258	3.392	3.2305
4.2329	4.2708	4.1641	4.949	4.1781	4.2512	4.9633	4.081
4.4842	4.2603						
ANKMY1	1.5633	1.0065	2.0635	1.384	1.5061	1.0908	0.8705
1.5861	1.7577	0.7169	1.5652	0.6101	0.611	1.2528	1.4071
0.8129	2.0162	1.739	1.1832	1.6795	1.2232	1.8003	1.3989
1.8946	1.7781	1.4732	0.9546	1.5571	1.1001	0.6404	0.6399
2.1006	1.2084	1.4241	1.539	0.6567	1.9496	2.031	2.0282
0.9438	1.7455	1.0889	1.7325	1.1964	1.3073	1.466	1.4641
0.7735	2.3137	0.9018	1.6724	1.2271	1.5174	1.57	1.3291
1.684	1.4488	1.4906	1.2951	1.3922	1.7434	1.5234	2.1599
1.4695	2.6106	0.6803	1.166	1.4458	0.914	2.0075	0.7084
1.9149	1.9293	0.7743	0.8529	1.7022	1.9194	1.322	1.5302
1.7239	1.7273	0.9678	1.1933	1.264	1.4018	1.2223	1.2869
1.4331	1.3142						
ANKMY2	3.127	3.1574	3.2696	4.0864	2.5267	2.6591	4.0284
5.0992	3.4829	2.3939	3.7473	2.9025	3.4778	3.6555	2.8099
2.7849	3.4463	3.3415	3.9232	3.3071	3.4057	3.2058	3.3954
3.6636	4.142	3.8882	3.3683	3.7591	3.2173	4.113	5.226
3.4275	3.1832	3.7893	3.5126	3.9009	3.5682	2.9379	2.8036
3.6916	3.5845	4.3471	3.8615	3.4535	3.1962	4.3688	4.0838
3.8635	3.4735	3.1198	3.7079	3.215	4.3372	3.2682	4.2885
3.2846	3.3562	3.8041	3.5504	3.7416	2.5088	3.4895	2.9321
3.0929	3.1908	3.7325	2.5229	3.192	3.4415	2.9161	2.9864
3.5979	4.095	4.3019	3.0894	3.7279	3.0065	2.9783	3.8289
3.7539	3.8262	2.3469	3.8585	2.4144	3.4271	4.3912	2.5871
2.8184	2.8471						
ANKRA2	2.239	2.1615	2.0035	2.2799	3.1751	2.0688	2.394
2.367	1.8565	2.216	2.9012	3.1422	3.4556	2.6385	2.5213
2.5994	3.2003	2.6734	2.5822	2.6998	2.2023	2.4116	2.5511
2.1318	1.8987	3.3918	2.9715	2.4864	2.6931	2.8768	2.8346
2.4285	2.5583	2.4296	3.0117	2.6829	3.1742	3.0467	2.1504
2.9398	2.5587	2.7754	2.167	2.2256	2.7479	3.3246	1.8504
2.2187	3.2163	2.9123	2.145	2.7631	2.3991	2.489	1.9606
1.7307	3.1752	1.822	2.2013	1.8283	2.8451	3.0745	2.457
2.131	2.7837	3.8473	2.9006	3.0745	2.5357	2.6621	2.1177
2.3941	2.8736	2.2162	2.084	2.6576	2.2965	2.6099	2.4881
2.6242	2.2362	2.1826	3.1858	2.7081	3.1238	2.57	3.1022

2.7291	2.8921							
ANKRD1	0.3903	0.3392	0.5354	1.5144	3.2942	0.2106	0.8573	
3.2044	0.1397	0.146	0	2.051	1.0943	0.1747	1.9103	
0.1365	1.1901	0	0.2059	1.9394	0.1861	0.1829	2.1238	
0.2915	0.584	3.5168	0.2761	0.1924	0.4286	0.1484	0	
5.7691	0.2382	0	0.6203	0	0.7681	0	0.6952	
0.1608	0.1228	0.8763	0	0.1572	1.4276	3.3441	0.1383	
0.2373	0.3724	5.0464	0.2473	0.1278	0	0	0.3824	
0.0802	2.5638	0	0	3.9579	1.7815	2.8689	3.0162	
0.0143	0	4.5857	0.106	3.4294	0	0.3995	0	
0.0389	0.007	1.3932	1.7022	0	0.0342	1.144	0.007	0
	0	0.4308	1.5476	0	0.9007	0	0	
0.145	0.1525							
ANKRD10	4.3322	3.9845	4.1958	5.1802	4.6446	4.2455	5.0497	
4.7204	4.0137	3.7483	3.8576	4.1455	3.8454	4.5193	4.381	
2.9811	3.5469	3.8647	4.5447	5.3582	4.7412	3.8964	3.7887	
3.906	4.9944	3.5894	6.4204	4.1769	3.5068	4.6821	4.8865	
3.9005	2.9997	3.6567	4.5681	3.9316	3.6437	3.9139	4.4612	
3.6583	4.3486	3.8924	4.1735	4.2767	3.8013	2.6978	4.3313	
3.5262	5.1131	4.1484	4.81	4.2788	3.6976	4.7823	3.5769	
5.8758	3.8344	3.6191	3.2693	4.1852	3.5182	4.5794	4.6457	
4.1805	3.4491	3.1167	3.358	3.9846	5.4908	4.9455	3.5284	
4.4256	4.2827	4.4238	4.5328	3.222	4.0889	4.053	4.5627	
3.987	4.1286	4.2763	5.2996	4.5221	4.0792	3.6099	4.2219	
4.0827	4.3376							
ANKRD11	4.8602	4.4069	5.0126	4.5161	4.94	4.4107	4.0681	
4.9767	3.7976	5.2182	4.3959	3.6271	4.9852	3.5681	4.3127	
3.7571	5.6572	4.1294	4.0254	4.6136	4.6826	4.4896	4.0246	
4.6026	5.1487	4.9323	4.5414	4.2977	3.5366	4.0294	4.2999	
4.6983	3.8525	3.6757	4.8259	4.0279	4.6614	5.6465	5.401	
4.1811	4.847	4.1602	4.5682	3.9529	5.2099	4.6149	4.4624	
3.7312	4.8023	3.7893	4.4086	4.2238	4.0375	5.9059	4.3444	
4.8977	5.7536	3.9959	4.9357	4.2574	4.3902	4.725	4.9143	
4.3336	4.6935	4.6436	4.6474	5.3608	5.1142	4.712	4.823	
4.7307	3.7039	3.8838	4.514	4.8747	3.8897	4.7007	4.2522	
3.9769	4.5515	4.3521	4.4751	5.0052	4.5189	3.8913	4.4545	
4.7481	4.8124							
ANKRD12	1.9859	1.8346	3.0347	2.6899	1.7823	3.1322	2.9707	
2.1285	3.125	2.146	2.7836	2.1977	2.2442	3.3446	2.6253	
1.9302	2.7605	2.5962	2.8164	2.2348	2.5712	2.5944	2.7141	
2.2442	2.0192	2.6053	2.4742	2.8387	2.1548	1.8544	2.731	
1.9256	2.3608	1.6035	2.0244	2.508	2.508	2.6037	1.6768	
2.4452	2.4398	2.1086	1.4862	2.085	2.9542	3.5418	2.279	
3.0665	2.067	3.1396	2.6271	2.6032	3.0019	2.3161	2.2734	
2.3979	2.6135	2.2969	3.2153	2.7747	1.7707	2.5763	2.0327	
2.7958	2.6402	2.6729	3.5885	3.1248	3.4298	1.6977	2.468	
3.2964	2.6679	3.2006	2.5836	2.7296	2.3418	2.3938	2.3498	
2.5894	2.0204	2.4237	3.0279	2.4178	1.5018	2.6082	3.3077	
2.7884	2.885							
ANKRD13A	3.8216	2.9046	3.8898	3.7069	3.5576	3.6474	4.0546	

3.6864	3.9322	4.5959	3.7411	3.1136	4.3258	4.1727	3.6443
3.6821	4.4983	4.3342	4.2491	3.8809	3.8055	3.6227	3.2137
3.667	3.4996	5.627	4.2151	3.7717	3.4492	4.4361	3.1462
3.2117	3.9937	3.3905	3.5706	4.237	3.5065	4.1466	4.0034
3.7588	4.1569	4.29	4.5434	3.978	4.4412	3.8314	3.8338
3.9565	3.8055	4.8434	4.0463	3.7566	4.3835	3.5066	3.588
4.2793	4.26	3.5527	3.9231	2.9508	3.8804	3.5162	2.7484
4.0434	4.3981	4.4652	3.914	3.1883	3.9999	3.5607	4.2319
3.8106	3.2404	3.4735	3.8708	3.1078	3.99	3.7806	4.6892
3.8923	3.8606	4.4161	4.0171	3.768	4.1293	4.1527	4.2393
3.6678	3.9502						
ANKRD13B	2.4621	2.2877	2.324	2.0203	3.1861	2.5589	1.8703
2.0757	1.5792	1.7058	3.6767	2.2033	2.074	1.6738	2.5005
2.6605	2.9647	1.928	1.5283	3.0459	1.8797	2.6128	2.498
2.1551	2.7805	2.0757	3.8366	2.4412	2.3108	2.6048	1.7085
2.8391	1.9822	1.4562	2.0747	1.4338	2.2873	2.0142	2.566
2.8445	2.6932	1.6272	1.7466	1.9172	2.2314	1.6585	2.0337
2.7613	1.7727	2.3565	2.4149	1.5461	1.8857	2.1475	2.2841
2.6682	0.8119	2.9746	2.3256	3.1366	2.9285	1.9714	2.6139
2.4493	3.5735	2.5788	2.2865	2.9867	2.3726	2.4324	2.2388
2.5009	2.6432	2.1203	3.0696	2.9177	2.1145	2.8452	2.0479
2.0188	1.7065	2.3501	2.8112	3.6181	1.9976	1.9684	1.8558
2.0904	2.2567						
ANKRD13C	2.5899	2.8	3.1161	2.8565	3.1161	3.4405	3.9945
2.9525	2.7381	3.3317	2.4801	3.0201	2.9931	3.5941	3.1099
2.9428	2.8974	2.7972	3.4435	2.6646	3.0791	2.8494	2.7804
3.0522	2.7148	3.3381	3.5913	3.0599	3.1895	2.9843	2.9531
2.9036	3.7406	3.2855	2.5184	3.1379	2.6778	3.2875	2.4889
2.5304	2.7945	2.9816	2.8112	3.0548	2.9368	2.8143	3.0022
3.4665	3.003	3.6778	3.2936	3.1171	3.452	3.3497	3.2464
3.073	3.0985	3.491	2.5523	3.6087	2.7014	2.8068	3.0028
3.4419	2.8142	3.0069	3.1271	3.3989	3.5984	2.8948	2.7664
3.3083	3.2616	3.142	3.147	3.1563	3.155	2.8399	3.1213
2.5027	3.1058	3.3614	2.848	1.7158	3.3748	3.208	3.1243
3.0684	3.1989						
ANKRD13D	3.3593	2.9149	4.4842	2.2509	3.6148	3.3292	3.208
4.1381	2.5327	3.6427	3.6306	2.5128	2.8149	2.5251	3.5636
3.5454	3.4588	2.4624	2.7249	3.7957	3.8554	3.7598	2.7907
3.1712	3.9658	2.3466	2.8269	3.2529	3.2653	2.7999	3.0634
2.7372	2.5319	3.5179	3.2983	2.3659	3.69	4.0824	3.6034
1.7529	3.9227	3.2971	3.3575	2.7283	2.4097	2.4606	2.8348
3.1323	4.4942	1.8537	3.1637	1.9428	2.226	3.9909	3.5703
4.0671	2.6562	3.1683	4.1531	2.6335	2.809	2.9893	3.1438
2.9181	2.8858	3.6827	2.9629	4.2397	4.5045	3.1813	3.3192
3.0806	3.0384	2.3559	2.8821	3.8154	5.1425	2.7375	2.6251
3.5259	2.2239	3.5916	4.1924	2.8773	3.2045	3.0455	3.5861
3.4942	4.0581						
ANKRD16	2.1728	1.5743	1.7562	2.0786	2.0906	1.262	1.1094
1.6777	1.6554	2.4092	1.8922	1.2073	1.7264	2.2245	2.1852
1.473	2.2336	2.1591	1.7116	2.4826	2.0003	1.9468	1.5709

2.3809	2.1163	0.8636	1.5361	1.8565	1.4919	1.7161	1.9415	
2.562	1.6392	2.3428	1.2875	2.105	2.0531	1.5632	2.4028	
2.0515	2.4983	1.2228	1.4736	1.9024	1.4156	1.321	2.3613	
1.6025	1.2701	1.7487	2.2953	1.2863	2.0033	2.0161	1.9866	
2.488	1.2317	1.2974	2.101	1.6508	2.0231	2.156	2.4104	
2.39	2.115	0.9581	1.5392	1.7258	1.4543	2.0872	1.9459	
1.8415	1.7849	0.9977	1.327	1.933	2.346	2.059	1.0762	
2.1387	1.8278	1.852	2.0853	1.7431	1.4095	1.7999	2.0227	
1.9372	1.9559							
ANKRD17	3.9028	4.0132	4.4437	4.8893	4.3855	4.248	4.9633	
3.3693	4.1253	3.997	4.424	4.7369	3.4866	4.5141	3.5865	
4.0144	3.7463	4.8478	4.2734	4.0609	4.4695	4.4321	3.8794	
4.0014	4.1308	4.7995	3.8576	4.6105	3.6174	4.502	3.4719	
4.0883	4.1656	3.6146	4.2813	4.2489	4.0698	4.4486	4.2108	
4.2696	4.195	4.4028	4.0445	4.3355	4.394	4.4978	4.5177	
4.895	3.4064	4.5363	3.7331	5.0135	4.6884	4.2444	3.7936	
4.0215	4.2663	4.3725	4.1262	4.4044	3.695	4.1155	4.2762	
4.4727	4.7598	4.1747	4.1981	4.1926	4.851	3.7939	4.3565	
4.0032	4.2878	4.1835	4.3864	4.38	4.4092	4.4895	4.0951	
3.619	4.3872	4.6095	3.4827	4.5936	3.0845	3.7928	4.3958	
4.1488	4.2568							
ANKRD18A	0.039	0.1615	1.2556	0.8048	1.6394	0.2366	0.1561	
1.1083	1.3601	0.014	0.7793	0.2092	0.5835	0.0449	1.2189	
1.2664	0.134	0.0158	1.2682	1.1134	0.0452	0.0641	0.0933	
1.0628	1.4183	0.394	1.2682	0.0737	0.7075	0.0462	1.6265	
0.0558	0.5506	0.1357	0.5066	0.3789	2.00E-04	0.2742	0.7131	
1.7044	0.1099	0.0217	1.2316	1.0832	1.2394	0.9218	0.2736	
1.1784	0.7706	0.6316	0.896	0.1657	0.2545	0.1028	2.301	
0.0939	1.8539	0.5391	0.2115	0.778	0.2153	0.7036	0.7995	
0.3658	0.8354	0.3835	0.6554	0.0843	0.306	0.8943	0.0611	
1.3281	0.4985	1.1245	1.4257	0.2769	0.0675	0.4414	1.3155	
1.2093	0.0158	0.4569	0.0845	0.2756	0.8695	0.3047	0.2152	
0.149	0							
ANKRD18B	1.4234	0	0.7628	0.6999	1.4377	0.0136	0.1499	
1.2565	0.1669	1.5189	0.6844	0.6162	0.3456	1.5189	0.7186	
0.4711	0.2305	0.9213	1.5356	0.9592	0.3097	0.8131	0.552	
1.4385	0.3563	0.2005	1.1537	0.2891	1.118	0.2746	1.0871	
0.4329	0.7111	0.5623	0.0457	1.4412	0.5831	0.2516	0.398	
1.3264	0.1817	0.3572	0.6139	0.7915	1.5414	1.3174	2.0289	
0.5645	1.1118	2.605	0	0.9947	0.1531	0.6884	2.3131	
0.7159	1.5601	2.3144	1.6036	1.6285	0.8103	1.3781	1.551	
0.4909	0.513	0.461	0.2918	0.6256	0.3938	0.7922	0.9865	
0.9909	0.2412	1.0944	0.3583	0.2415	0.2347	0.3649	1.3497	
0.1167	0.0979	0.2427	0.1536	0.4835	0	0.4163	0.233	
0.1625	0.1627							
ANKRD2	0.1447	0	0	0	0.074	0	0.0708	
0.184	0	0.217	0.0522	0	0.06	0	0.0606	0
	0	0	0	0.2903	0.1137	0	0.039	0
	0.0884	0	0	0	0.0722	0	0	
0.3616	0	0	0.1128	0.0697	0.1636	0	0	0

	0.0686	0	0	0	0	0	0	0
	0.1695	0	0	0	0.1679	0.0557	0.8077	0
	0	0	0.0274	0	0.0251	0	1.2314	
0.0663	0	0.0659	0	0.1498	0	0.1598	0.0177	
0.0497	0	0.0201	0.0161	0	0.0894	0.019	0	
0.0442	0	0.2233	0.0381	0	0.0709	0.1797	0.6572	
0.1669	0.4841							
ANKRD20A1		0.0315	0	0.0918	0.0602	0.0061	0.0247	
0.0063	0.1372	0.1293	0.0445	0.0261	0.0059	0.0132	0.0307	
0.0128	0.0434	0.4418	0.1583	0.0496	0.0314	0.0243	0.0265	0
	0.0096	0.1291	0.0386	0.2372	0.0686	0.0118	0.0185	
0.0874	0.1149	0.0162	0.0187	0.0034	0.041	0.0082	0.0574	
0.038	0.1286	0.0091	0.0042	0.0706	0.1724	0.0348	0.0184	
0.087	0.0191	0.0242	0	0.0314	0.0153	0.0925	0	
0.1465	0.0271	0.0509	0	0.0456	0.0062	0.019	7.00E-04	
0.0386	0.2607	0.0114	0	0.0763	0.0109	0.0034	0.0235	
0.0644	0.0166	0.1177	0.3427	0.0707	0.1317	0.0024	0.06	
0.0388	0.0499	0.12	0.1235	0.0188	0.1484	0.0135	0.035	
0.0197	0.0264	0.0451						
ANKRD20A2		0	0.0368	0	0.0246	0.0049	0	
0.0039	0.0057	0.013	0.0094	0	0	0.0046	0.0264	
0.0024	0	0.0285	0	0.04	0	0	0	
0.0011	0	0	0	0.0493	0.0326	0.0114	0.0105	
0.0052	0	0.012	0	0	0	0	0	
0.0042	0.0084	0.0178	0	0.0206	0.0208	0.0039	0	0
	0.0149	0.0068	0	0	0	0.0322	0	
0.0122	0	0	0	0	0	0	0	0
	0.063	0	0	0.0185	0	0	0	
0.0087	0.007	0	0.0656	0.0117	0.0082	0	0.0093	
0.0031	0.0261	0.0232	0.0102	0	0.019	0	0	
0.0193	0.023	0						
ANKRD20A3		0.0274	0.0102	0.0203	0.033	0.0036	0.0186	
0.0037	0.0105	0.0116	0.0153	0.0021	0.0033	0.0052	0.0463	
0.0128	0.0354	0.0383	0.0157	0.0532	0.0273	0.1833	0.0016	
0.0054	0.0186	0.0027	0.005	0.0423	0.0761	0.0371	0.0102	
0.0071	0.0014	0.0166	0.0152	0.0015	0.0053	0.002	0.0154	
0.0359	0.0169	0.0066	0.0011	0.0278	0.0106	0.0045	0.0076	
0.0175	0.0507	0.1106	0	0.0273	0.0486	0.0369	0.0025	
0.0167	0.0343	0.0158	0	0.0102	0.0109	0.0119	0.0046	
0.0473	0.0149	0.0155	0	0.0233	2.00E-04	0.0043	0.0302	
0.0137	0.0375	0.0256	0.0246	0.1448	0.0511	9.00E-04	0.0107	
0.0025	0.0674	0.0103	0.0115	0.0045	0.0119	0.0838	0.043	
0.0218	0.0271	0.04						
ANKRD20A4		0.0348	0.1285	0.1239	0.061	0.0299	0.0952	
0.1063	0.242	0.3955	0.3615	0.1661	0.0216	0.0652	0.0876	
0.1311	0.2536	0.2004	0.1476	0.2179	0.0304	0.1272	0.1111	
0.0796	0.1885	0.1232	0.0012	0.5786	0.0684	0.2123	0.1834	
0.1468	0.631	0.122	0.3017	0.1452	0.0916	0.0174	0.1663	
0.0619	0.9008	0.0906	0.0594	0.1704	0.1605	0.1575	0.073	
0.3575	0.1207	0.2329	0	0.1311	0.1768	0.3938	0.3361	

0.1711	0.2167	0.0313	0.072	0.3488	0.0567	0.1228	0.0292	
0.0502	0.1755	0.0308	0.0423	0.194	0.1236	0.1454	0	
0.1693	0.0686	0.1941	0.8378	0.2205	0	0.3184	0.2376	
0.0912	0.42	0.1346	0.179	0.0541	0.1245	0.0347	0.2542	
0.1373	0.1104	0						
ANKRD22	0.0608	0	0	0.1638	0.0396	0	0.0395	
0.4392	0.0136	0	0.0033	0.1231	0.0058	0.0315	0.0152	
0.0105	0.0417	0.0387	0	0.0229	0.0248	0.0024	0.017	0
	0.8446	0.0773	0.0121	0.0267	0	0	0.0064	
0.0018	0	0.0097	0.0477	0.0113	1.1527	0	0.0061	
0.0092	0.0088	0.0171	0	0.0217	0.0152	0.0575	0.0182	
0.0155	0.0189	0.1124	0.0583	0.1828	0	0.0243	0	0
	0.0529	0	0.0133	0.0159	0.0637	0.0025	0.0509	
0.0523	0.0228	0.0631	0.0365	0.0231	0.004	0	0.1963	
0.0078	0.0137	0.0389	0	0.1928	0	0.0301	0.0111	
0.032	0.0454	0.0977	0.0209	0	0.0267	0	0	0
	0.0282							
ANKRD23	1.5725	2.9168	2.4226	0.5968	0.8148	0.6117	1.0117	
1.1779	0.7214	1.2369	1.6848	0.2726	0.4904	1.0918	1.0552	
0.9504	0.9251	2.3028	1.0345	1.2541	1.1238	0.8013	0.433	
1.5672	0.7116	0.7455	0.902	2.0336	0.5721	2.5317	0.514	
0.876	0.9245	0.9983	1.1238	0.7812	0.7086	0.9753	0.4738	
0.905	1.2547	0.424	0.7855	0.7311	0.3496	0.7829	0.9796	
0.8303	0.8647	0.5561	0.7161	0.5707	0.9966	0.9238	0.5839	
1.4791	0.4557	1.3354	1.6466	1.0691	0.452	1.463	0.6453	
1.1224	0.8646	0.8849	1.1926	1.5723	2.6518	0.5938	0.6796	
0.998	0.9296	0.4641	0.6362	3.2877	1.114	0.6308	0.7425	
0.7955	1.0055	0.8028	0.8633	0.9687	0.6396	1.0998	1.0729	
2.2113	2.2011							
ANKRD24	0.2342	0.0879	0.0799	0	0.3061	0.1033	0.3003	
0.0318	0.0961	0.0171	0.4719	0	0.2342	0	0.046	
0.3125	0.6797	0.3091	0.1231	0.3574	0.0355	0.0404	0.0814	
0.2939	0	0.2794	0.3856	0.2999	0.115	0.1609	0.8538	
0.3334	0.0759	0.6624	0.0952	0.095	0.3034	0.4309	0.3445	
0.5175	0.5408	0.1725	0.156	0.1015	0.0285	0.0955	0	
0.3256	0.0501	0.3307	0.1282	0	0.1746	0.3301	0.2329	
0.2031	0.3028	0.0842	0.1501	0	0.1884	0.0517	0.0352	
0.1987	0.3257	1.3726	0.7432	0.2058	0.0681	0.0892	0.1027	
0.084	0.1364	0.2569	0.1526	0.0473	0.1406	0.2124	0.181	
0.1958	0.2305	0.0728	0.2219	0.2167	0.0559	0.2851	0.0585	
0.0602	0.2602							
ANKRD26	1.9656	1.5391	2.1383	1.9843	1.3578	1.1935	1.1435	
1.1921	2.1479	2.2606	1.2785	1.3308	2.0599	2.2717	1.244	
1.3442	2.0639	2.1673	2.057	2.0576	2.1313	1.2912	1.2827	
2.0086	1.4335	1.3251	1.6161	2.3251	1.5158	0.9894	1.8503	
1.5684	1.6972	1.7113	1.3404	2.2866	2.1349	1.3751	1.1531	
1.5025	1.7637	1.2757	1.4103	2.0144	1.7551	1.8589	2.2257	
1.5247	1.3764	2.715	2.4515	1.3483	2.0153	1.8718	2.5202	
2.3549	1.9166	0.9458	2.0531	1.0524	1.465	1.5403	1.6379	
2.1088	2.2796	1.4938	2.1612	1.9826	2.356	1.594	1.916	

1.8379	1.465	1.2302	1.1612	1.66	2.0593	1.7844	1.1816	
1.6118	1.6663	1.6365	1.9802	1.4448	1.1297	1.9148	1.9226	
1.7807	1.9385							
ANKRD27	3.7782	3.4845	4.4793	3.2838	3.2752	3.4328	3.81	
3.5839	3.2207	3.5532	5.1939	3.3741	4.1126	4.5006	3.1531	
3.1055	4.6529	3.4028	4.4624	3.5044	3.5122	3.1217	2.7355	
2.9986	3.4743	3.4609	3.6369	5.0399	2.658	5.5342	3.4713	
3.4762	4.0651	4.4798	2.8263	3.8994	2.8689	2.6039	4.0314	
3.7599	3.1026	2.8566	3.5967	3.296	4.0692	3.6726	4.6544	
3.6504	3.1474	4.544	3.992	2.9182	4.4793	3.7528	3.5443	
4.5129	4.055	3.2833	4.8549	3.1716	3.8442	4.2763	4.0082	
4.4214	3.7005	2.4986	3.3051	2.6505	4.8804	3.256	3.6509	
4.1015	3.588	3.7205	2.6338	3.9325	3.6009	2.2617	3.7067	
3.1295	4.371	3.0567	3.1775	2.3382	3.2001	4.2564	3.5617	
2.9859	3.2749							
ANKRD28	5.7825	3.9661	4.4378	5.2856	4.6949	5.2115	5.898	
3.6825	5.4323	5.8263	5.6684	6.332	5.262	6.5724	5.1366	
6.2863	4.2795	6.1866	5.1551	4.7838	5.0329	4.4878	5.4974	
4.3827	3.361	4.3703	3.7154	4.362	4.941	4.4723	3.4267	
5.3584	4.5754	4.7936	4.5843	4.8938	5.6911	3.413	5.0202	
4.7337	6.2388	3.6633	3.8392	5.2995	5.7926	4.7332	5.6734	
4.4672	4.51	5.5888	5.0156	3.602	4.8672	6.1073	2.9056	
4.3146	5.1495	2.8715	5.2509	3.4041	4.8811	3.8494	4.3464	
4.9131	3.7648	4.736	5.6248	5.489	6.0192	4.7413	4.9539	
4.5429	4.7664	5.514	5.452	4.7266	4.6257	5.7606	4.3473	
4.0003	5.452	5.387	4.6739	5.0332	4.5795	4.8758	5.6075	
4.8994	4.8218							
ANKRD29	0.6014	0.2918	0.3288	1.0941	0.7797	1.9289	1.8163	
0.3991	0	0.0623	0.8105	2.7754	1.1639	0.2602	1.0563	
0.3505	0.6167	0	0.0537	1.2327	0	0	0.2764	
0.8902	1.6986	2.9943	1.2671	0.4595	0.3674	0	1.735	
0.0025	1.6933	1.3453	0.0803	0.2258	1.3359	0	0.2206	
0.9419	0.5324	2.8912	0.7339	0.3318	5.0592	2.5882	0.0303	
0.0568	0.9381	0.0638	1.8723	0	0.2496	0.0168	1.1797	0
	2.4898	3.073	0.1952	2.9711	1.0613	0.0674	0.3161	
0.6428	0.0887	0.8108	0.3438	1.9996	0.2247	0.1462	3.519	
0.3341	0.1034	0.2027	0.549	0.2852	0.0298	0.2017	0.4517	
0.7792	0.4864	1.9923	0.5044	0.4946	0.9318	0.0875	0.2921	
0.0392	0.0461							
ANKRD30A	0	0.5651	0	0	0.4111	0.0209	0	0
	0.0109	0	0.0129	0.0045	0.0028	0.0194	0.0035	0
	0.8078	0.0135	0.0294	0	0	0.0013	0.0147	
0.0786	0.0189	0.015	0.0607	0	0	0	0	
4.00E-04	0.0563	0.0206	5.00E-04	0	0.0011	0.0268	0	
0.0064	0	0.1142	0.0153	0	0	0.024	0	0
	0	0	0.0059	0.0092	0.0282	0.0052	0.0251	0
	0.1063	0.0384	0.0715	0.0574	0.068	0.0525	0.0248	
0.0457	0.0294	0	0.0668	0.0011	0.0083	0.0777	0.0173	
0.0251	0	0.1624	0.014	0	0.0064	0.0285	0	
0.0392	0.0369	0	0.0101	0	0.0371	0.0695	0.0193	0

	0							
ANKRD30B	2.1748	1.9995	2.8613	1.1324	0.1421	2.4889	2.2415	
0.1098	1.5175	2.1229	1.9854	0.8086	0.0399	2.9033	0.6897	
1.0016	0.1268	2.4092	1.8362	0.047	2.0856	0.1181	0.4115	
0.2078	0.0206	1.7052	0.0689	3.6726	0.2162	0.0676	0.093	
2.5989	2.4846	1.8026	1.2489	1.6205	3.9322	2.464	0.7102	
0.287	1.2524	0.1036	0	0.1169	0.6192	1.253	2.5725	
0.9296	1.1033	2.3981	0.7859	0.0866	1.1082	0.8708	0.3266	
1.5014	0.0234	0.0828	1.4364	1.5785	0.3968	0.7681	1.8874	
1.3187	3.4248	0.849	1.3739	3.8011	0.4479	0.0252	1.4622	
1.3791	1.7029	0.7131	0.0256	2.3538	2.0804	0.6865	2.4704	
1.2334	0.6042	0.1864	2.958	1.0322	0.0385	1.381	1.7289	
0.4553	0.9366							
ANKRD30BL		0	0	0	0	0.0101	0	0
	0	0	0.0278	0	0	0	0.0558	0
	0	0	0	0	0	0	0.0095	0
	0	0.057	0	0.0319	0	0	0.0311	
0.0231	0.0206	0.0376	0	0	0	0	0.0445	
0.0357	0	0	0.0222	0	0	0	0	0
	0	0	0	0	0.0494	0	0.0321	0
	0	0	0	0	0	0.064	0	
0.0216	0	0.0808	0	0	0	0.0498	0	0
	0	0	0	0	0	0	0.0236	0
	0	0	0	0	0	0	0	0
	0	0						
ANKRD31	0.2556	0	0.3423	0.0754	0.0824	0.1048	0.1289	
0.211	0.0505	0.0629	0.1081	0.0123	0.3855	0.0481	0.0679	
0.0844	0.4965	0.0302	0.0873	0.0985	0.0339	0.0024	0.0405	
0.3298	0.0338	0.0679	0	0.149	0.0452	0.041	0.1742	
0.1897	0.0878	0.1784	0.01	0.0435	0.227	0.0453	0.0465	
0.1984	0.0241	0.0264	0.0881	0.1245	0.0097	0.0813	0.5602	
0.1288	0.0149	0.3464	0.0831	0.1144	0.0641	0.1913	0.0137	
0.0573	0.0361	0.0554	0.0134	0.0256	0.1013	0	0.0467	0
	0.2319	0.2176	0	0.1684	0.1511	0	0.2129	
0.1246	0.329	0.007	0.1862	0.0751	0.1283	0.084	0.3735	
0.4566	0.144	0.0228	0.0297	0.2165	0	0.0534	0.0638	
0.1172	0.0837							
ANKRD33	0	0	0	0	0	0	0	0
	0	0	0	0	0	0	0	0
	0	0	0	0	0	0	0	0
	0	0	0	0	0	0	0.0214	0
	0	0	0	0	0	0	0	0
	0	0	0	0	0	0	0	0
	0	0	0	0	0	0	0	0
	0.0486	0	0	0	0	0	0	0
	0	0	0	0	0	0	0	0
	0	0	0	0	0	0	0	0
	0	0	0	0	0	0	0.0686	0
	0							
ANKRD33B	1.3551	1.7211	1.6881	0.7646	1.3274	0.1266	0.7479	

0.987	1.5669	0.9533	2.9691	0.2301	0.8914	1.4706	0.2149	
1.3902	1.2976	2.1429	2.941	0.1902	0.4867	0.4933	0.9031	
0.9813	0.4948	0.5106	0.2768	1.6766	0.3452	3.1528	1.596	
0.5069	0.2403	0.7277	0.4588	1.0127	0.1369	0.6795	0.1791	
0.6501	0.6696	0.6948	1.6636	1.8792	1.2014	1.3937	1.0855	
0.6367	0.6339	1.1395	1.4322	0.3088	1.7264	0.7341	1.8619	
1.1413	2.9749	1.5817	3.3521	0.9735	0.5511	0.4424	0.6013	
0.5776	0.7034	0.9778	0.5138	0.1261	1.3484	0.865	0.2598	
0.5732	1.062	1.2662	0.1843	2.6253	0.5527	0.7274	0.6717	
0.6642	1.5889	0.3452	0.1993	0.3177	0.8851	1.7117	0.0787	
0.1499	0.0436							
ANKRD34A	0.8201	0.8016	0.7983	1.6378	2.0763	1.5462	0.4934	
0.786	0.7516	0.7687	1.8655	1.928	0.5655	1.2747	0.5434	
1.5295	1.8525	1.494	0.6218	1.1527	0.937	1.1256	1.4226	
1.2433	1.3004	1.0452	1.0749	1.3845	1.2255	1.9285	1.7844	
0.8185	0.9386	1.4877	0.5759	1.0411	1.3826	0.9275	0.7331	
1.3545	1.8857	1.5481	1.9757	0.7347	1.0493	0.8822	0.9307	
1.4917	0.6797	0.9761	1.1118	1.568	1.1996	0.8404	1.4918	
0.6732	0.6724	1.0925	1.7025	0.9633	1.5673	1.0365	1.213	
1.8104	1.0565	1.8754	2.0052	1.2041	0.7962	0.9506	1.2086	
1.3704	1.0313	1.6896	1.7033	1.3032	1.2202	0.773	0.8076	
1.4261	1.3417	1.6157	1.316	0.716	0.9351	1.0661	1.035	
0.8539	0.9609							
ANKRD34B	0	0	0	0.1478	0.0149	0.0335	0.1401	
0.0676	0.0076	0	0.0367	0.1833	0.3661	0.064	0	
0.0236	0	0	0.0636	0.1487	0	0	0.0144	
0.0558	0.0038	0.1709	0.0809	0.1176	0.0112	0.0115	1.9804	0
	0.0144	0.2939	0.0035	0.423	0.0613	0.0485	0.0232	
0.3102	0	0.0421	0	0.0253	0.0915	0.0188	0.1909	0
	0	0.0579	0.2533	0.0125	0	0	1.1444	0
	0	0.0241	0	0.1918	0.1822	0.0227	0.0386	0
	0.1549	0	0.0429	0.0495	0.1091	0.0498	0	
0.0123	0.0178	0.0414	0	0.2238	0.0894	0.2613	0.082	0
	0.0329	0.0402	0.2415	0.1177	0.048	0.0482	0	
0.0236	0							
ANKRD34C	0	0.0213	0	0	0	0	0	0
	0.1071	0	0.0049	0	0	0	0	
0.0148	0	0.0281	0.1104	0	0.026	0.0029	0	
0.0081	0	0	0	0	0	0.012	0	
0.061	0.0139	0	0.0014	0	0	0	0.0057	
0.0098	0	0.0018	0	0	0	0	0.0569	
0.0165	0	0.0449	0	0	0.0328	0	0	0
	0	0	0	0	0	0.0034	0.0289	0
	0	0	0	0.0045	0.0149	0	0	0
	0	0.1159	0.0067	0.0751	0.0149	0	0.0174	0
	0.0918	0	0.0166	0	0	0.0267	0	0
	0							
ANKRD35	0	0	0	0	0	0	0	0
	0	0.0133	0	0	0	0	0.0076	0
	0.033	0	0	0	0	0	0	0

	0.0164	0.0548	0.0402	0	0.0155	0	0.0193	0
	0	0.0274	0	0.0104	0	0	0.0083	0
	0	0	0.047	0	0	0.0551	0	0
	0.0355	0	0	0	0	0	0	0
	0.0254	0	0	0	0.0525	0.0074	0.0111	0
	0	0	0	0	0	0	0.0932	
0.0123	0	0	0.0332	0.0187	0	0.0181	6.00E-04	
0.0449	0.0947	0	0	0.0441	0	0	0.3022	
0.025	0.0469							
ANKRD36	1.6338	2.3801	2.3865	1.3886	1.4095	1.8973	2.002	
1.8404	1.6435	2.4917	1.9014	1.3123	1.2865	2.104	1.8279	
1.4737	2.0581	2.9354	1.7282	1.6851	1.9627	0.9788	0.7292	
2.4403	1.492	1.1427	3.3684	2.0101	1.5418	2.5978	1.1398	
1.2929	1.9036	1.7402	1.5772	1.813	1.1823	0.5899	0.7776	
2.5261	1.735	0.9553	1.6391	2.1703	2.2277	1.7592	2.1599	
1.3341	1.8745	1.8776	1.5988	1.3252	1.7682	1.3957	1.8082	
1.6725	1.6801	1.1109	2.4463	1.2442	1.3151	1.0773	1.8482	
1.913	1.7636	1.2086	1.9546	2.3349	3.7078	1.4834	1.6256	
1.6189	1.3371	1.1546	1.5455	2.2762	1.9217	1.0414	1.4697	
1.8356	2.3538	1.6948	1.5419	1.0732	1.761	1.5169	1.4932	
1.9501	1.7537							
ANKRD36B	1.3742	0.9991	1.5572	0.6377	0.7185	1.7158	1.0811	
1.4258	1.3845	1.5667	0.7342	0.688	0.8677	1.836	1.2148	
1.2833	2.0711	1.1755	1.2655	1.5519	1.4954	0.7967	0.5296	
1.9401	0.9583	1.0992	1.6834	1.0576	1.0102	0.6605	0.9367	
1.06	1.9014	1.3484	0.8722	1.1421	0.8007	0.7179	0.5475	
1.6973	1.0425	0.7102	1.2084	2.3307	1.6693	1.4375	1.1628	
1.3929	1.1291	1.1273	1.0986	1.023	1.4863	0.9029	1.0636	
1.2225	1.9094	1.2175	1.2196	1.1889	1.0439	0.3535	1.1281	
1.9758	1.0494	0.5226	1.6041	0.8738	1.9594	1.2601	1.6197	
1.7005	0.9076	0.8718	0.9094	0.8346	1.5182	1.058	1.448	
1.4868	1.5114	0.9967	0.7859	0.6666	0.7942	0.8976	0.7552	
1.3415	1.0479							
ANKRD36C	0.3023	0.148	0.3074	0.1512	0.1211	0.5342	0.1344	
0.1637	0.6126	0.8049	0.2335	0.0988	0.2025	0.4826	0.1367	
0.1386	0.8732	0.5619	0.3413	0.0196	0.2976	0.1074	0.0227	
0.3926	0.2145	0.152	0.5087	0.2558	0.0741	0.2091	0.2	
0.1568	0.2081	0.3178	0.1726	0.1815	0.0904	0.0548	0.05	
0.6817	0.1905	0.0946	0.2111	0.6449	0.5062	0.1813	0.1869	
0.2154	0.2364	0.4779	0.141	0.21	0.3809	0.3791	0.1814	
0.2564	0.4036	0.1201	0.5308	0.2058	0.0103	0.0333	0.0913	
0.5484	0.2791	0.0889	0.4596	0.1331	0.4156	0.0364	0.2658	
0.1835	0.2062	0.102	0.2301	0.2802	0.2151	0.0494	0.2305	
0.4211	0.5155	0.2059	0.3326	0.1673	0.1062	0.5351	0.1663	
0.2636	0.364							
ANKRD37	1.5649	1.9617	0.8091	1.8602	2.5335	1.1715	0.9403	
1.202	0.6904	1.2184	2.1151	1.1407	0.7562	1.6952	0.5466	
0.6004	1.3809	1.127	0.5816	2.0295	1.3059	0.7966	1.196	
1.5084	0.2223	1.2149	1.0628	1.6163	0.7632	1.7962	1.065	
1.4021	1.5414	1.1821	0.5309	1.3937	1.0734	1.3979	0.8559	

0.4385	1.6728	0.4808	0.9481	0.2481	0.176	1.2452	1.3998
1.3482	0.7691	0.6296	0.4466	1.0522	1.1878	2.1651	0.5741
0.9033	0.7619	0.3839	1.0876	0.6106	1.3831	2.1979	0.9787
1.1533	1.0148	1.5328	1.1006	1.9293	0.7476	0.938	1.3341
1.9491	1.9809	1.0411	0.5681	0.6219	1.6142	0.234	1.2022
3.2612	1.3557	1.7158	3.0466	0.459	0.7691	0.9729	3.9684
2.1961	1.3992						
ANKRD39	4.6774	5.6016	5.8849	2.7312	3.3957	3.2775	2.8318
4.6378	3.269	4.3683	3.8483	3.4004	2.8955	3.1575	3.3539
4.1737	3.1489	4.749	3.7307	4.3481	4.6518	3.613	3.7532
4.747	3.0655	2.2502	3.5978	4.1191	3.546	5.1499	3.6276
3.7204	4.2252	3.9916	3.4808	3.9302	3.8228	4.3874	3.6028
2.925	3.9397	2.2827	3.6884	2.8293	2.5147	2.8508	2.6991
3.3885	3.6106	1.9003	3.2155	2.4531	2.7527	3.2028	4.4262
4.1035	1.6096	4.6522	4.3094	4.249	3.2102	5.1401	3.8633
3.211	3.8885	2.1543	4.1589	3.8172	5.1778	3.0674	3.5802
3.7869	4.7506	3.535	3.1269	6.6407	4.3373	3.9182	3.4588
4.4556	3.6997	3.201	3.5187	3.0518	4.3842	3.8737	4.7097
5.8448	5.5612						
ANKRD40	4.9526	4.4461	4.999	5.0669	4.5065	4.3543	4.7802
4.5456	4.2648	4.4263	4.0783	4.2916	4.5313	5.2244	4.091
4.618	4.6087	5.0681	4.6087	4.8319	4.6887	4.3888	4.2701
4.4966	4.6882	4.3929	4.0601	5.5825	5.0555	4.091	3.3452
5.1018	4.2624	3.6233	4.92	4.353	5.0597	4.5718	4.1646
4.4908	3.9696	3.9989	4.0465	4.4536	5.3698	4.3676	5.4792
5.0003	4.7399	4.5316	5.0735	4.4756	4.2592	4.777	4.1464
5.0574	4.2435	4.4891	4.1784	4.6812	4.7953	4.8371	3.9795
4.4056	4.2505	3.9261	5.0076	4.5753	5.0112	4.6835	4.3826
4.8353	5.0721	3.6458	5.22	4.988	4.3985	4.5551	4.6803
4.3943	4.9676	4.5968	4.1596	5.2011	4.4436	4.2089	4.3888
4.4929	4.7421						
ANKRD42	1.5249	1.9832	1.7546	1.7909	2.8789	2.2673	3.5257
1.6832	1.5894	1.8536	1.148	2.1489	2.166	1.5927	1.8184
2.4472	2.5409	1.3478	1.496	2.2776	1.2405	2.2513	2.9759
1.6677	1.9541	2.7751	2.89	1.4702	2.6612	1.8046	1.9482
1.6398	2.1763	1.7559	2.1447	1.8898	2.5754	2.2519	1.231
2.4641	2.7423	3.2239	1.224	1.4357	1.8507	2.9691	2.3822
1.6274	2.7135	2.0848	1.5204	2.6301	1.7377	2.2433	2.3828
1.9862	2.3697	0.8846	1.9336	1.15	2.0504	1.8215	1.4066
2.0613	1.5201	3.0512	1.7467	3.0339	1.6954	1.9094	3.2096
1.558	2.3219	1.977	2.6396	2.274	1.4972	2.4427	1.7467
1.5253	1.0854	2.503	3.3866	1.5627	1.8269	1.4461	1.9197
1.6548	1.6318						
ANKRD44	2.2753	2.076	2.0781	2.0447	1.1991	4.2925	2.229
2.7939	4.0389	4.3482	3.328	2.4251	1.6507	3.5889	2.0864
3.3952	1.3952	3.8564	4.1718	2.0965	2.8087	1.8055	2.1579
3.8085	1.9151	0.8985	3.2207	2.8389	3.2108	3.4931	0.7129
2.8538	4.3974	4.0484	2.5898	3.3967	1.1828	2.2564	2.3328
3.586	3.0262	1.9605	2.6259	3.2506	1.4761	1.3702	3.7684
3.1272	1.4667	1.4583	1.9748	1.7668	3.592	2.9824	0.6857

3.3289	1.0251	1.5445	3.5838	1.859	2.6714	2.4463	2.4552	
3.8065	3.3499	1.1185	3.8009	1.8553	4.1322	2.5261	2.9432	
3.3462	3.2457	2.6002	2.3709	3.3285	2.9611	2.5277	3.8927	
3.19	4.6433	1.3085	2.7999	2.737	1.2519	3.0232	3.5668	
2.864	3.5887							
ANKRD45	0.2588	0.0847	0.1605	0.7147	0.637	0.1389	0.1646	
0.8194	0.238	0.1049	0.2883	0.4549	0.0174	0.9889	1.2048	
0.0059	0.2121	0.0714	0.1749	0.0628	0.0284	0.7306	0.0786	
0.1418	2.3037	0.3075	1.0481	0.0312	1.7057	0.7722	0.5498	
0.5787	0.1209	0.1999	1.8802	0.2332	0.2156	0.2168	0.4335	
2.5884	1.583	0.3877	0.1237	0.3512	0.1212	0.2744	0.2756	
0.2022	0.2477	0.7031	0.8008	0.2904	0.3168	0.1664	1.5179	0
	0.2642	0.1195	0.5728	0.0392	0.0899	0.0795	1.6501	
0.0976	0.2304	1.2854	0.4532	0.6051	0.1128	1.6578	0.5128	
0.0831	0.0957	0.6767	0.0975	0.9421	0.1153	1.1847	0.303	
0.2174	0.0465	0.2751	0.345	2.5192	0.4214	0.2481	0.1448	
0.0244	0.1127							
ANKRD46	1.9963	3.0216	3.4576	3.5714	2.7682	2.8855	2.529	
2.6374	2.618	3.5073	2.868	2.3168	1.7926	2.7238	2.8497	
1.6399	2.2957	3.5032	3.8747	2.4705	2.1239	2.4737	2.6145	
2.5646	2.9852	2.0856	2.4713	3.0034	2.4614	3.8949	3.2344	
2.6317	3.2348	2.6407	2.8397	3.6384	2.8868	3.4228	2.0861	
3.4008	2.2496	2.8698	2.4922	2.1056	2.7889	3.2918	3.8583	
2.6347	2.1996	3.436	2.0564	2.0361	2.9572	3.7058	2.593	
3.1964	2.4269	3.8012	3.3367	3.3903	2.2897	3.1725	2.8186	
1.8894	3.6155	1.8285	2.8553	1.9698	2.7155	2.5253	2.5294	
2.1878	2.8573	2.8673	1.902	2.7477	2.7737	2.2822	3.7671	
3.5858	2.6698	2.1697	2.3489	2.1468	2.5227	2.6199	2.0634	
2.381	2.3235							
ANKRD49	2.2758	2.2304	2.775	2.8975	2.4808	1.9092	3.2056	
1.8381	3.0761	2.0443	2.3024	1.8094	2.7454	2.5144	2.169	
2.2342	2.7308	2.1973	2.3499	2.4693	2.1695	2.7294	2.236	
2.0015	2.9233	2.4498	1.8498	2.2215	2.7444	2.2062	2.5177	
1.6901	2.6793	2.268	2.2905	2.4237	2.5731	2.765	2.106	
2.4421	2.1235	1.7655	1.7967	2.3845	2.4871	3.0074	3.2884	
3.0727	2.1449	2.8334	2.4709	2.3003	2.454	2.8262	2.7501	
2.7489	2.1599	1.896	2.2011	1.9162	1.949	2.2215	2.7032	
2.539	2.3574	2.4916	2.526	2.4018	2.106	1.6321	3.2244	
2.5621	2.6935	2.1217	2.238	2.6283	1.9956	2.1904	2.7005	
2.3036	2.3758	1.8919	3.89	1.8744	2.1881	2.5881	2.5716	
2.6728	2.6271							
ANKRD50	2.8286	1.6908	2.7981	3.8922	4.1181	4.0049	2.5084	
3.4853	2.7988	3.0981	1.8498	2.966	1.9721	3.2148	2.9816	
2.9475	3.5347	3.1597	2.4562	2.8234	3.1053	3.2908	2.3104	
2.6313	2.3988	2.7652	3.4781	3.3424	2.9173	1.9774	2.6816	
3.2016	2.9547	3.35	3.7299	2.9479	2.4201	3.377	3.2403	
3.4012	3.1866	2.6634	2.0802	3.1825	2.4474	2.395	3.2073	
3.3328	2.2177	2.7241	3.2457	3.1484	2.8351	2.2584	2.7038	
2.2186	3.7963	2.3546	2.9364	2.4135	2.839	2.8346	3.5681	
2.8653	3.3344	2.5513	3.6787	3.385	2.6353	2.5462	3.6028	

2.7802	2.781	2.6353	3.5489	2.7857	2.7968	3.2668	2.8098	
2.839	2.4161	3.9764	2.2853	3.5634	2.3841	2.6425	3.2111	
3.0254	2.9638							
ANKRD52	4.0559	4.0892	4.9472	4.1829	5.0078	4.1147	4.687	
3.394	4.527	4.8307	4.296	3.6084	3.7635	4.1325	3.8484	
4.4685	3.7338	3.9708	4.0945	4.3107	4.5144	3.4453	3.9347	
4.1999	4.2171	3.9836	2.8918	4.6289	4.0811	4.0085	3.6456	
4.2238	4.0755	3.151	4.3027	4.3954	3.8427	4.7194	4.0356	
4.7158	3.8708	4.2568	3.9318	3.8967	4.225	3.3881	3.9853	
4.4524	3.8617	3.6922	3.6193	3.9589	4.0883	3.4348	4.1281	
3.8354	3.7116	4.5089	4.8584	4.3407	4.2697	4.7805	4.7958	
4.3106	4.1599	4.2116	3.9852	3.7684	5.026	4.099	4.2923	
3.732	4.0696	3.5466	4.195	3.7555	4.3961	4.5352	3.8574	
3.8563	3.9242	4.0535	3.5808	4.6666	3.2161	3.6675	4.1512	
4.2978	4.4587							
ANKRD53	0.0778	0.046	0.2637	0	0.013	0.0595	0	
0.1559	0	0.3942	0	0	0.0492	0	0	
0.1163	0.0716	0.5569	0	0	0.1713	0.0172	0.0456	
0.143	0.2415	0.1654	0.0849	0.07	0.3052	0.1272	0.0219	
0.0208	0.489	0.0358	0.0967	0.1259	0.0643	0	0.1618	0
	0.0984	0.1081	0.2454	0.1866	0	0.1374	0	
0.0456	0.2011	0.1024	0.091	0.3514	0.3977	0.1129	0	0
	0	0.0494	0.2172	0.1232	0.0914	0.0335	0.1052	
0.0307	0.1745	0.1393	0.2017	0.5705	0.0833	0.2501	0	
0.0415	0	0.1324	0.3456	0.5159	0.1929	0.019	0	0
	0.0686	0	0.2471	0.3357	0.2664	0.5629	0.4473	
0.5005	0.6239							
ANKRD54	4.1674	4.1563	4.249	3.4344	4.0145	4.7903	3.5395	
4.7983	3.5487	4.7226	4.1488	3.5246	3.855	4.0239	4.0024	
4.4957	2.2671	2.7395	4.1208	4.8535	4.2855	3.359	3.641	
5.443	3.9583	3.4213	3.5982	4.216	4.1163	4.6541	3.5496	
3.3823	4.2804	4.7601	4.4511	4.094	3.7625	3.5406	4.8562	
5.2388	3.5068	3.5679	3.1978	4.2604	2.8309	2.8569	3.7721	
4.351	3.7415	2.7413	3.2116	2.9318	4.0145	4.4475	3.2048	
4.4931	2.386	6.2487	4.4731	5.9592	3.9601	3.9142	4.0186	
4.2446	2.9025	3.3021	4.073	3.3055	4.091	3.7063	4.6689	
4.0315	4.1641	4.1698	4.2183	4.1609	3.827	4.3035	3.3474	
4.3957	4.4091	3.9701	3.4332	3.904	3.7309	4.2201	3.2909	
4.2029	3.4387							
ANKRD55	0	0.0244	0.0906	0.0765	0.1949	0.1157	0.0207	
0.0247	0.0835	0.0735	0	0.0436	1.5412	0.0803	0.0917	
0.0595	0.039	0	0	0	0.122	0.0295	0.0426	
0.1576	0.0756	0.0857	0.1298	0.1286	0	0	0.015	
0.0303	0	0.1304	0.1037	0	0.1042	0.0688	0.1428	0
	0.0266	0.0372	0	0.0337	0.1408	0.7588	0.0416	0
	0.1679	0.0568	0.0878	0.1051	0.0933	0.4527	0.2001	0
	0.0053	0.0195	0.1018	0.0643	0	0.0438	0.1382	0
	0.0729	0	0.1972	0	0	0.4929	0	
0.0912	0.0113	0	0.1025	0	0.1689	0	0.249	
0.1122	0	0.0013	0.1304	0	0.1304	0.1924	0.0416	0

	0							
ANKRD6	2.1922	2.2366	1.8413	1.3027	0.2617	1.3931	1.7989	
1.2449	2.5388	2.5896	2.4468	1.3708	0.6913	2.0203	1.184	
1.957	0.074	2.3471	3.4483	2.0333	1.3285	0.6208	1.1797	
1.9009	1.1233	0.1515	2.1143	2.2079	1.4876	2.574	2.1444	
1.7644	1.9454	2.2588	1.4951	2.4082	0.8505	1.7256	0.6771	
1.7479	2.0208	0.4781	1.1853	1.9494	1.6509	0.1702	1.8044	
2.0452	0.9163	0.1332	2.1772	1.3596	2.1404	1.9523	1.9041	
1.8702	0.8297	3.776	2.2219	3.2171	1.1001	0.7142	1.1499	
1.3915	0.9047	1.158	1.1946	1.3622	3.2244	1.3175	1.177	
1.2118	1.639	1.1611	1.1529	2.2081	2.3141	1.1318	1.9071	
2.1583	2.0447	0.4896	2.7186	1.2198	0.8222	1.5355	1.6461	
1.4139	1.9866							
ANKRD61	0.688	0	0.7306	0.4073	0.2628	0.5362	0.3034	
0.583	0.585	0.365	0.357	0.1418	0.3508	0.5249	0.4941	
0.2003	0.5641	0.6715	0.4766	0.5334	0.5486	0.4888	0.4669	
0.4198	0.8266	0.3631	0.9761	0.4314	0.5528	0.2019	0.5321	
0.4084	0.2867	0.1927	0.6759	0.2475	0.4009	0.1744	0.2531	
0.5556	0.3187	0.175	0.6769	0.7705	0.4682	0.5468	0.7888	
0.5203	0.6658	0.2982	0.461	1.0435	0.5532	0.4972	0.5465	
0.859	0.524	0.3622	0.642	0.6001	0.146	0.4522	0.8189	
0.4162	0.525	0.2125	0.6513	0.1383	0.3505	0.5568	0.1744	
0.5745	0.4912	0.2034	0.2559	0.1926	0.1449	0.4015	0.3036	
0.6193	0.3822	0.4739	0.2366	0.4322	0.2738	0.6694	0.2639	
0.3757	0.1983							
ANKRD62	0.0276	0.0132	0	0.0432	0	0.0283	0	
0.0102	0.0235	0.0094	0.004	0.0053	0.0091	0.036	0.0049	0
	0.0239	0.0894	0	0.0311	0.0597	0.0259	0.0055	
0.0145	0.0065	0.0103	0.0585	0.0624	0.0219	0.0119	0.0975	
0.0048	0.0136	0	0.003	0.0408	0.0038	0.0192	0	
0.0101	0.0432	0.0657	0	0.0134	0.0055	0.0106	0	0
	0.015	0	0.0145	0.0748	0	0.0094	0.0836	
0.0322	0.0415	0.0832	0.064	0.0295	0.0325	0.0177	0.0339	
0.0135	0.0319	0	0	0	0.0161	0.0286	0.0174	
0.0039	0.0676	0.0419	0	0.0212	0.0014	0.014	0	0
	0.0192	0.013	0.0178	0.0101	0	0.0284	0	0
	0.0289							
ANKRD63	0	0	0	0	0.3295	0	0	0
	0	0	0	0	0	0	0	0
	0	0	0	0	0	0	0	0
	0	0	0	0	0	0	0.1187	
0.0657	0	0	0	0	0	0	0	0
	0	0	0	0	0	0	0	0
	0	0	0	0	0	0.0747	0.195	0
	0.1434	0.1261	0	0	0	0	0.1247	0
	0.1249	0	0	0	0.096	0	0	0
	0	0	0	0	0	0	0	0
	0	0.024	0.0361	0	0	0	0	0
	0							
ANKRD65	0.0681	0.1707	0.1105	0	0.0144	0	0	

0.0296	0.0284	0.0939	0	0	0.0241	0	0	
0.0233	0.0679	0.0548	0.0954	0	0	0	0.0306	0
	0.0409	0	0	0.1138	0.1111	0.0382	0.0827	0
	0.0389	0.0371	0	0.029	0	0	0.0744	0
	0.0275	0	0	0.0375	0	0	0	0
	0	0	0.0325	0	0	0.1987	0.1601	0
	0.1086	0	0.1143	0.0409	0.0417	0	0	0
	0.0569	0.2007	0.0748	0.0382	0	0	0	
0.0878	0.1016	0.0112	0.0262	0.037	0.0565	0	0.0886	
0.0778	0.0303	0.118	0.016	0.04	0.0612	0	0.1742	
0.3671	0.4844							
ANKRD66	0	0	0	0	0	0	0	0
	0	0	0	0	0	0	0	0
	0	0	0	0	0	0	0	0
	0	0	0	0	0	0	0	0
	0	0	0	0	0	0	0	0
	0	0	0	0	0	0	0	0
	0	0	0	0	0	0	0	0
	0	0	0	0	0	0	0	0
	0	0	0	0	0	0	0	0
	0	0	0	0	0	0	0	0
	0	0	0	0	0	0	0	0
	0							
ANKRD7	0.8087	0.8775	0	0.7758	0.0236	1.8379	0.9705	
1.779	1.1932	0.3727	1.6078	0.4633	0.6283	2.1651	0.3871	
2.464	1.242	1.9647	2.0023	0.9391	0	0.7583	0.9684	
1.2199	1.2111	1.1983	1.0069	0	0.8498	1.1784	0.9214	
0.948	1.5448	0.7869	0.5275	1.029	1.3083	0.0173	0.3199	
1.5594	0.8319	0.5868	0.3852	2.2966	0.0538	1.555	1.0646	
1.0919	1.3471	1.1938	1.3923	1.4168	0.4972	1.4835	1.3195	
1.4842	0.4944	0.6717	1.7691	0.7828	0.4362	1.0106	0.6562	
0.3618	0.9986	0.2808	2.0924	0.1112	0	0.4869	1.9661	
1.5905	1.181	0.67	0.6369	1.4334	0.3343	0.1551	0.4969	
1.4376	0.5343	0.5814	0.5222	1.2852	0.5011	1.0241	0.2134	
0.6187	0.4351							
ANKRD9	3.6807	3.8339	3.1943	2.536	1.6847	1.9616	1.5613	
2.466	1.6811	3.6593	3.6833	3.2179	2.5657	2.605	2.3042	
2.5643	2.3692	4.333	3.0923	2.8643	3.3948	2.4153	1.4357	
2.3206	1.5785	1.7815	3.335	4.1056	1.7684	4.3829	2.7884	
2.5036	1.9542	3.5179	2.2694	3.3004	2.3285	3.1334	3.1248	
1.9199	2.8152	2.2519	2.106	2.0582	2.7906	2.6241	2.9491	
2.4655	2.5726	2.5514	2.6675	1.8186	2.8516	3.9003	2.1357	
4.0021	2.6701	2.6977	3.9759	2.3053	2.9333	4.0426	2.7337	
1.9189	3.4187	3.1667	3.8918	3.7391	2.6801	1.8725	3.0704	
2.5923	3.8895	1.4118	1.9874	2.3915	3.7391	2.7557	2.5774	
3.541	2.9183	2.1366	3.2448	2.9825	3.6831	3.7764	3.5349	
4.1389	4.4147							
ANKS1A	3.8164	4.1031	3.4351	3.9243	2.3676	5.5966	3.873	
3.2672	4.2409	4.5395	3.9201	4.4068	1.9228	5.0602	5.09	
5.428	3.4096	5.2955	3.976	3.4915	3.2768	3.9447	4.0873	

3.7424	4.0426	3.7915	3.0529	4.5703	5.1303	3.9501	3.665	
5.7272	5.0196	4.0697	4.8844	4.0669	6.4669	4.9399	3.4682	
5.9276	4.9958	3.6729	2.6138	3.3744	3.348	4.0446	3.443	
7.0356	3.0704	3.4208	3.6206	4.1123	5.1712	5.4333	3.3115	
4.1851	3.2851	4.2799	4.6464	3.2829	4.6318	3.7775	3.5303	
4.7295	4.9685	2.9068	4.5027	3.1976	4.6048	3.2752	4.692	
4.7917	3.9749	4.835	3.4523	5.5937	4.2578	4.6514	6.3324	
4.9499	3.755	3.0849	7.0653	5.4793	2.8583	4.6817	3.6662	
3.9504	3.9621							
ANKS1B	3.2841	1.256	1.5467	1.1124	0.7078	0.9045	1.1957	
3.0407	2.7976	2.6496	2.5708	0.3217	0.3302	3.2004	0.5024	
1.6569	1.3624	1.1745	2.0156	1.9588	1.7958	0.7177	0.3909	
1.6784	1.6833	0.6433	0.6135	2.6226	0.4993	1.0449	1.4607	
2.173	1.0845	1.0055	1.1301	2.122	1.2094	0.8353	1.2274	
2.3673	0.5799	0.41	1.6704	2.4701	0.9785	1.9186	1.9894	
1.0865	0.5226	1.6585	0.8066	0.4993	3.1962	0.9783	1.6164	
2.5658	0.9651	5.4858	1.3693	4.5703	1.7353	1.5357	1.3257	
1.6241	1.8235	0.7416	1.6651	0.8839	1.3347	1.1628	1.6359	
1.1318	1.866	0.5279	1.4495	0.9549	1.2056	1.2359	0.6645	
1.4511	1.0081	0.6244	1.3333	1.7578	1.3118	2.5249	1.0857	
1.7489	1.3217							
ANKS3	1.6675	2.6447	1.7121	1.7883	2.2808	1.8962	1.7209	
2.5371	1.506	1.2031	1.7365	1.329	2.085	1.1187	2.1892	
1.6844	2.0421	1.8253	1.4881	2.0673	2.354	2.8042	2.2162	
2.3189	2.0262	2.5299	2.2701	1.8323	1.9639	1.2712	1.9174	
2.2447	2.1493	1.7791	2.1682	0.9481	1.7906	2.3563	1.6749	
1.4991	2.2138	1.6438	1.0761	1.6742	1.5738	1.2527	1.0701	
1.831	2.6378	1.9997	1.5261	1.7685	1.236	2.1665	1.5599	
1.6686	1.43	2.0005	1.6207	1.9118	2.4062	2.4725	1.9654	
1.8687	1.1613	2.3631	1.7559	2.2904	1.6231	2.0228	1.6923	
2.1998	1.8261	1.5635	2.5255	1.8579	1.6807	1.8555	1.52	
1.4833	1.5238	3.0003	2.1443	1.5238	2.4855	1.8709	1.3795	
1.8705	1.6599							
ANKS4B	0	0	0	0	0.0024	0.0258	0.0279	0
	0.02	0.0505	0	0.0029	0.0398	0.0308	0.0045	
0.0049	0	0.0408	0.0677	0.0288	0.0481	0.0086	0.0024	
0.0364	0	0.0179	0.0286	0.0329	0.0131	0.0271	0.0068	0
	0	0.0423	6.00E-04	0	0.0116	0	0.0172	0
	0.0093	0	0.0926	0.0468	0.0146	0.0129	0.0233	
0.0344	0	0.0495	0.0377	0.0154	0.0573	0	0.0247	
0.0505	0	0	0.0413	0.0205	0	0.0103	0.014	
0.0282	0.0267	0	0.0664	0.003	0	0	0	
0.0363	0	0	0.0147	0.0439	0	0.0371	0	
0.0349	0.0124	0	0.0116	0.0753	0.0297	0.0468	0.0216	
0.0251	0.0313							
ANKS6	2.4179	2.7522	2.7977	2.8271	2.497	1.2727	1.9933	
2.1833	1.3991	1.9412	2.9186	2.5939	2.9802	2.0873	2.2939	
1.7547	2.8631	2.1655	1.8964	2.972	1.8279	2.1304	1.8244	
2.6648	2.3832	2.2934	2.731	1.9755	2.1202	2.1567	2.7646	
2.7333	1.527	1.4744	1.2289	2.0656	2.9043	2.6405	3.009	

1.9607	2.5944	1.668	2.5359	2.4017	2.2223	1.9962	2.8322	
1.6033	2.4075	1.1166	2.3147	2.3691	2.686	2.7313	2.9791	
2.6255	3.5029	2.6909	1.3313	3.0831	2.0639	1.4273	2.0282	
2.284	2.873	2.1372	1.9703	2.1169	2.39	3.002	1.9501	
1.7812	2.1602	1.9065	1.8493	1.605	2.1106	2.363	1.8444	
2.1005	2.3006	2.2731	2.5273	3.2794	1.3398	2.4252	2.4144	
2.3478	2.3632							
ANKUB1	0.1247	1.1453	0.0716	0.032	0.0386	0	0.0361	
0.0147	0.0805	0.0343	0.0392	0	0.0423	0	0.0095	
0.0282	0.08	0.114	0.2439	0.1557	0	0.0183	0	
0.0556	0	0.0144	0.02	0.4245	0.032	1.1465	0.0155	
0.0254	0.0667	0.037	0.0334	0.0566	0.0501	0.031	0	
0.0155	0.0528	0.0131	0	0.0447	0	0	0.2533	
0.0271	0	0	0	0.0201	0	0	0.0329	
0.7445	0	0.0326	0	0.0276	0.169	0.1922	0.0593	0
	0	0.0435	0.0442	0	0.4155	0.0643	0.0116	0
	0.3333	0	0	0.3799	0.0288	0.0215	0.102	
0.0371	0.0146	0	0.0853	0	0.1491	0.1005	0.0517	0
	0							
ANKZF1	3.5951	3.4595	2.8114	3.2274	3.3658	3.6579	3.1047	
3.5911	3.667	4.3029	4.1701	2.9005	3.7102	3.181	2.5582	
2.6095	3.3029	3.5379	2.9005	2.9914	3.47	3.8365	3.8879	
3.4533	3.1751	3.7857	4.7442	3.1171	3.4746	5.0287	4.8957	
2.9234	3.4382	3.646	2.8902	3.7354	3.4413	3.315	3.6334	
3.5244	3.242	3.0324	3.4054	3.6256	2.6684	3.449	3.3553	
3.3082	4.1223	2.5217	3.1956	3.5191	3.714	3.8531	3.8266	
2.9708	3.3499	3.1126	3.0792	2.7605	3.4278	3.4986	2.8513	
2.9402	3.3968	3.6865	3.5214	3.0043	3.2864	3.1081	2.9854	
4.0863	3.329	4.0664	2.6937	3.0189	3.0532	2.9545	3.3883	
5.3606	3.6257	3.4874	3.8696	2.5922	3.2331	3.4042	2.8926	
2.8272	3.2342							
ANLN	5.662	3.981	4.9317	6.2658	5.4018	5.4999	3.7579	
5.4077	6.2909	5.2433	3.8908	5.2819	6.3266	5.5643	6.3472	
4.6821	4.9694	5.7015	6.1585	5.9968	5.8392	5.2222	5.9254	
6.1384	6.111	5.4831	3.7639	4.2405	6.9099	4.5178	5.3937	
5.6698	6.0692	4.7506	6.2429	6.0165	5.1223	2.3597	4.4329	
6.0269	4.1526	4.9971	7.1104	6.219	6.3999	7.7928	5.5899	
5.006	5.662	6.1344	5.2561	5.9566	6.2084	3.1221	5.9822	
5.4995	5.6644	5.7129	6.0102	6.0436	5.8559	5.3995	5.4617	
6.0057	5.4143	2.6212	5.5584	2.6607	4.9602	6.9255	4.5396	
6.417	6.193	5.8029	5.8426	4.5594	4.9522	5.1438	5.1609	
5.2438	5.6402	4.4362	3.9651	5.1086	6.4184	6.7507	4.8452	
5.6265	5.1534							
ANO1	0.3556	0.1722	0.1305	0.1564	3.8997	0.1917	0.0939	
0.0648	0.0632	0.2894	0.4098	0.1515	1.3125	0.0803	2.4508	
1.6244	0.0764	1.3841	0	0.2453	0.1379	0.5118	0.2729	
0.0549	1.5943	0.4176	0.2421	0.1529	2.5535	0.213	0.4879	
0.1414	0.1733	0.1596	0.2796	0.4223	0.1669	0.1142	1.7286	
0.0722	0.8512	0.0492	2.2035	0.7248	2.4219	0.0924	0.3886	
0.3655	0.2796	0	0.1569	0.1053	0.1829	0.4127	0.0528	0

	0.4656	0.0428	0.7064	0	2.2737	0.3056	0.2396	
1.4138	0.3458	0	0.2285	0.272	0.095	1.3763	0.0184	
1.5311	0.0215	0.1684	0.2864	0.1574	0.1545	0	0.0914	
0.224	0.2309	0.4645	0.3511	2.2282	2.58	0.0418	0.1769	0
	0							
ANO10	3.6273	3.5085	2.5104	3.5491	3.3753	4.0967	4.0882	
3.9101	3.6414	4.4955	4.0281	4.7529	4.6822	3.7585	4.4935	
4.4286	4.4511	4.1667	3.6813	4.0104	3.2635	3.8694	4.4955	
4.5243	3.9508	4.1768	3.7253	3.4683	3.9508	3.2943	4.3983	
4.1549	3.792	4.5855	4.6988	4.3418	3.8217	4.3353	2.9231	
3.9072	3.9713	4.5071	5.3648	3.742	4.3557	3.1514	4.0599	
3.6446	4.1857	5.2259	4.3093	4.4355	3.6351	2.6008	3.5004	
3.6149	4.8122	3.3927	3.6411	3.8053	3.7182	3.3705	2.7843	
4.0843	3.4705	5.2104	4.3883	4.6957	4.0627	3.2124	4.727	
3.7388	3.7885	3.8341	4.57	4.0513	3.5575	4.69	3.6415	
3.8672	3.7877	4.6398	4.2043	3.651	4.2182	3.4485	4.9814	
4.4447	4.5038							
ANO2	0	0.0213	0.0561	0	0	0.1198	0.0109	0
	0.3724	0.4098	0.0596	0.0137	0.0201	0	0.0769	
3.3628	0	0	0.142	0.0588	0.0338	0.111	0.1854	
0.0487	0.0159	0.0224	0.0224	0.04	0.3578	0.034	0.0647	
0.0188	0.3143	0.0171	2.0446	0.3221	0.0141	0.0568	0.0334	
0.0179	0	0.0086	0.1008	0	0	0.0603	0.0496	
0.0715	0.027	0	0	0	0	0	0	
3.4266	0	0	0	0.0078	0.0721	0.2477	0.0428	
0.04	0	0.0031	0	0.0123	0.1484	0	0	
0.1517	0	0.0263	0.1359	0.4837	0.0296	0.056	0.4661	0
	0.1094	7.00E-04	0.0459	0	0	0	0.0278	0
	0							
ANO3	0.0398	0.0045	0	0.2758	0.5115	0.2479	1.5265	
0.0114	0.013	0	0.0038	0	0	0.0411	0.3677	
0.0119	0	0	0.0259	0.1587	0.2552	0.8723	0	
0.6163	0	0	0.0175	0	0.1592	0.0584	0.2645	
0.7322	0.0042	0.0357	0.0096	0.0887	0.0288	1.5173	0.6741	
0.1306	0.5367	1.0952	0	0.2337	0	0.9308	0.004	0
	2.4481	0.0218	0	0.0022	0.1277	0.054	0.3645	0
	0	0	0.919	0.1138	0.0732	0.0775	0.1219	
0.1162	0	0.0785	0	0.0439	1.0342	0.2033	0.377	
0.054	0.1814	1.1155	0.1915	0.0835	0	0	0.0914	
0.2649	0	0.7424	0	0.4622	0	0.2813	0.0537	0
	0							
ANO4	0.0593	0.108	0	1.0958	0.2451	1.2648	4.345	
0.635	4.9397	3.8589	3.744	0.1391	0.297	2.6716	1.9781	
0.051	0.2226	0.2364	3.3909	0.0815	0.0843	0.1715	1.1323	
3.2255	1.9831	1.3628	3.628	1.9994	4.7011	3.2091	1.0768	
2.3795	4.7412	4.1821	1.9	2.6895	0.6976	0.9004	2.0602	
2.6063	3.3267	0.5918	3.3306	0.4327	0.7386	0	2.7537	
4.5763	0.1374	0.0576	2.0819	0.6902	4.1554	4.3813	0.592	
0.0443	0.2016	0.5772	1.0428	0.3409	3.5971	0	3.4934	
3.5334	0.5669	0.0326	1.6063	1.8196	3.101	0.1082	1.4127	

3.1985	0.2802	3.4509	2.6024	3.3444	2.6886	0.0218	3.51	0
	4.4764	1.1325	2.3613	2.5122	2.2594	3.3048	2.8478	
2.4575	3.5896							
ANO5	0	2.2046	2.0015	2.081	0.1442	2.1427	2.7603	
2.8733	1.4322	1.5129	1.7795	1.2343	0	0.6038	0	0
	0	0	0.5888	0	0.129	0.5392	0.2189	
0.5882	0.1217	0	0.788	1.9008	1.291	2.4396	0	
0.48	0.116	2.6667	2.5572	0.8766	1.6819	0	0.5751	
0.3796	1.9992	0.6902	0.2652	1.1474	1.6047	0	1.5831	
2.548	0	0.0278	1.7163	0.8109	0	1.8682	1.0587	
2.6384	0	1.8006	0.1561	1.8591	0	0.2778	0.7723	
0.2547	3.2847	1.9394	3.4309	0.3919	1.9434	0.1227	2.252	
0.4675	2.8659	0.9496	0.4071	2.4705	0.0894	0.5005	2.0136	0
	0.265	0.8272	1.0734	0.5488	0.1887	0.1156	2.2597	
2.2025	2.5068							
ANO6	4.8895	3.2151	3.848	4.241	3.9953	4.9429	4.3507	
3.9917	4.8793	5.0162	4.208	3.7572	4.3234	4.6402	4.2751	
4.5758	5.0522	5.1925	4.6682	4.2798	4.2208	4.5541	4.4502	
4.2522	4.6122	4.872	4.7837	3.8968	4.6165	3.454	6.0112	
4.0002	4.0824	4.6555	5.1705	5.092	4.156	3.6185	3.8231	
4.9168	3.8518	5.0176	4.1134	4.7171	5.3662	5.141	4.6089	
3.4109	4.9689	5.4477	4.3897	4.2878	4.5332	3.9259	4.961	
3.6121	4.7536	4.9803	4.1765	4.881	4.482	4.1415	5.1453	
4.9458	3.9903	4.4185	4.5685	5.3496	4.3612	4.5952	4.7522	
4.4373	4.0199	4.0791	5.2814	2.4292	3.965	4.4574	4.2459	
3.8922	3.659	5.0143	5.1079	4.9509	3.9154	4.2321	4.7475	
4.3743	4.2972							
ANO7	0.5312	1.0135	0.6724	0.5129	0.3318	0.1973	0.5946	
1.0749	0.8459	0.6616	0.8371	0.7719	0.184	0.693	0.3774	
0.4611	0.9846	0.7478	0.7995	0.6123	1.2439	0.4332	0.6541	
0.7968	0.5043	0.3193	0.8976	0.923	0.6362	1.0595	0.6889	
0.9799	0.5316	0.6297	0.1015	0.7672	0.6347	0.7695	0.3375	
1.0446	0.6436	0.4256	0.627	0.5685	1.0649	0.5718	1.3351	
0.2466	0.6249	0.5861	0.6078	0.8045	1.1561	0.5731	0.942	
0.8424	0.6105	0.9178	0.714	0.689	0.6174	0.6046	0.7417	
0.6213	1.0575	0.1551	0.7101	0.294	1.2551	0.6835	0.7794	
0.6856	0.4953	0.4038	0.263	0.8038	0.7988	0.1876	0.5079	
1.1672	1.2528	0.8169	0.3146	0.6115	0.6776	0.8645	0.3579	
0.4105	0.6782							
ANO8	1.9388	2.1058	1.9796	1.4994	2.3741	1.2495	2.5018	
2.3577	1.6098	2.1989	2.046	2.0179	2.5014	2.2025	2.079	
2.3954	1.6153	2.2034	2.1893	2.9889	2.4666	1.5025	1.5087	
2.3273	1.5419	1.5413	2.0346	2.1803	1.4859	1.9337	2.5768	
2.4409	1.2632	2.3202	1.1878	1.6109	1.7457	2.1544	2.3959	
0.9502	2.1294	2.1304	1.6564	1.835	1.4981	1.415	0.8736	
1.6705	2.3542	1.0362	1.7307	1.0521	1.6128	2.0175	2.2577	
2.375	1.7411	1.9882	3.5545	2.4967	1.4572	1.7119	2.1843	
1.6123	1.5557	2.3233	2.1335	2.316	2.0248	0.8727	1.7915	
1.8257	1.088	1.763	1.852	2.526	1.9182	1.7114	1.7307	
1.4848	1.6719	1.9215	2.2981	1.8932	2.7207	1.9064	1.8468	

1.7307	2.1436							
ANO9	0.4376	0.0231	0.0496	0	0.0483	0	0.0508	
0.0229	0.0225	0.2561	0	0.0175	0.0764	0	0	0
	0	0	0	0	0	0	0	0
	0	0.0622	0.0394	0.2231	0.024	0	0.063	0
	0	0.0294	2.00E-04	0	0.0042	0	0.0205	0
	0.1897	0.048	0	0.0313	0	0	0	
0.0356	0	0.0972	0.0265	0	0	0.0239	0	
0.1124	0.0732	0.0377	0.4253	0	0.0205	0.0143	0	0
	0.1217	0.04	0	0.0094	0.014	0	0	0
	0	0.039	0.0498	0	0	0	0	
0.0444	0.0274	0	0	0	0	0.0933	0	
0.1897	0.1497							
ANP32A	5.6689	7.1802	6.1649	6.1273	6.274	5.6512	5.3952	
5.526	6.0984	6.3657	5.8594	5.4021	5.1195	5.8866	4.9432	
5.6478	6.3067	5.8806	6.113	5.4487	5.7112	5.9355	5.6445	
6.2601	6.013	5.0364	5.1557	6.6362	5.6242	6.2024	6.7437	
5.2244	4.9087	5.1405	4.8832	6.3584	5.7256	6.1584	6.3067	
5.8604	5.4349	5.6537	5.7122	5.9566	6.2679	6.1724	6.237	
6.4017	5.0113	6.9113	5.5933	5.2263	5.6951	6.4875	6.5056	
6.1148	5.7123	6.3388	5.3052	6.2607	6.2498	6.381	6.6257	
5.7938	6.5928	5.0575	6.0989	5.2527	6.4624	5.4885	5.2679	
5.5508	5.8744	5.8524	5.9409	5.2449	6.4141	5.6602	6.1682	
6.411	6.4315	5.3149	4.7932	5.0607	6.2961	5.7806	5.0449	
5.6191	5.7514							
ANP32B	6.2978	5.9888	6.1153	6.8642	6.3634	6.3374	4.8244	
6.2669	5.0395	6.8797	5.9314	6.4707	6.9263	6.4694	5.9252	
4.9776	7.4519	6.1075	6.0275	6.1811	5.0904	6.1762	5.3275	
5.8254	6.6207	5.2511	7.2877	6.0359	6.0833	5.5884	6.5199	
6.0596	5.6202	5.4585	4.5818	6.6671	7.0104	5.9859	7.2588	
6.3319	5.0015	5.9092	6.5875	6.8138	6.3593	6.7594	6.3306	
5.9442	5.8514	7.2694	5.6235	6.4957	6.3872	7.124	7.0179	
6.9882	7.3127	7.4575	6.7656	7.5148	6.701	5.892	6.2854	
6.4788	7.4657	5.491	5.8357	5.5133	6.1123	7.038	5.6399	
6.4291	5.7634	6.0212	6.0987	4.719	5.9927	5.4211	5.6046	
5.9265	6.7254	6.0158	5.2503	6.3691	6.5398	6.5437	6.411	
6.3136	6.5957							
ANP32D	0	0.1706	0	0.1261	0	0	0	0
	0	0	0	0	0	0	0.0867	0
	0	0	0	0	0	0	0	0
	0	0	0	0.1909	0	0	0.0832	0
	0	0	0	0.0693	0	0.1571	0	0
	0	0	0	0	0	0	0	0
	0	0	0	0	0	0	0	0
	0	0	0	0	0	0	0	0
	0	0	0	0	0	0	0	0
	0	0	0	0	0	0	0	0
	0	0	0	0	0	0	0	0
	0							
ANP32E	4.37	5.2433	5.1344	6.1423	5.9971	5.299	4.5548	

5.5399	5.1724	5.6703	5.1674	5.6921	4.4027	5.3484	5.4785	
5.1711	4.7899	5.5612	5.6695	4.8901	5.3415	5.3075	4.6883	
5.2997	6.0451	4.5351	5.1023	5.719	4.9461	5.8306	6.8324	
4.6721	4.9624	4.9577	4.9265	6.2258	4.9109	4.2009	4.3457	
5.5222	5.1246	4.9532	6.0516	5.0444	6.0246	6.8897	5.679	
5.6206	3.3626	6.1878	5.7146	5.9721	5.744	4.4905	6.682	
5.1466	5.2781	6.0079	5.5473	6.2253	5.5406	4.8525	6.1761	
5.5877	6.1446	4.5588	5.4406	3.4649	5.5113	5.3966	4.8045	
5.7321	5.2252	5.0683	5.9617	4.7993	5.7204	5.4057	5.2811	
5.2434	5.4117	4.3707	3.7451	5.395	5.7486	5.9175	3.9403	
5.0649	4.8513							
ANPEP	0.002	0	0	5.5539	5.8313	0.3526	1.8629	
0.0024	0.2506	0.0132	0.2125	4.6817	2.7244	0.1844	0.3677	0
	5.0429	0	0	0	0.0258	0.2434	0	0
	1.6983	0.3296	1.6252	0	4.0223	0.1392	0.0202	
0.2066	0.0059	0.1156	0.0384	0.0502	0.1102	0	0.5438	0
	0.6268	7.8256	3.057	0.8392	1.0799	7.1993	0.5036	
1.2362	0.5992	6.2038	1.338	0.149	0.0135	0.901	0.1616	
0.1979	8.6856	0.158	0.2836	0.2018	3.6199	0.2156	1.6115	
0.2627	0.2943	0.3312	0.1546	4.4901	0.6274	5.0234	0.2985	
0.2125	0.4483	0.3619	0.4572	0.2638	0.3747	0.1385	0.1698	
0.3264	0.3164	4.3754	0.1693	0.1986	5.8777	0.2025	4.5612	
1.3715	1.151							
ANTXR1	4.4239	3.9803	3.2285	5.8052	4.1592	4.9728	4.6409	
2.2994	7.4541	3.6578	5.4041	5.615	5.7007	5.1927	7.2668	
4.8693	5.9649	3.9771	3.821	3.947	4.3629	4.3812	5.4959	
5.4228	4.8253	5.4901	5.0487	4.2961	5.5294	4.3744	3.7607	
4.9072	5.1596	5.1975	5.3667	4.3546	4.0245	4.5497	3.6039	
6.5245	4.3716	5.3597	5.0387	4.5982	5.0243	5.0944	4.4583	
3.5488	6.2772	5.3499	6.2423	4.6738	5.0305	5.3141	5.8305	
4.2838	4.6612	5.6597	3.43	5.9287	5.3898	4.1453	5.414	
4.3442	4.2684	6.5227	3.7085	5.4756	4.5468	5.6334	5.6155	
5.5802	4.5222	4.9667	5.9929	2.1954	4.0597	6.6405	4.598	
4.5465	4.5555	6.0494	3.4551	6.4332	3.6362	4.0886	5.525	
4.4253	5.3574							
ANTXR2	1.3499	0.1411	1.1445	3.5531	2.1617	3.5439	2.9704	
1.9072	1.8102	1.2796	1.4558	3.6898	3.5749	2.2208	2.3372	
1.9514	6.0389	0.0274	1.5533	3.4658	1.7701	2.9306	2.5767	
0.803	4.6662	5.3554	2.6362	0.625	1.9999	1.4218	0.0573	
0.7963	2.903	3.5188	4.0745	1.2841	3.0818	3.1011	2.2777	
2.9026	2.3288	4.2247	4.074	2.7355	3.4735	6.3547	1.3422	
2.0828	4.4843	3.2889	3.3028	4.054	2.5083	1.2253	0.1412	
0.9008	6.2991	2.5987	2.1964	3.1164	3.3133	1.8238	1.226	
1.278	1.6864	5.2774	0.202	3.9023	0.695	4.5134	3.3644	
2.0478	2.7241	1.4244	2.7079	3.0877	2.5715	3.0066	1.4762	
1.6166	1.7873	3.2576	2.1351	2.6027	3.9135	2.5561	3.5521	
2.1623	2.023							
ANTXRL	0.3821	0.2692	0	0	0.0045	0	0	0
	0.0139	0.4504	0	0	0.0103	0	0	
0.0095	0	0.1233	0	0	0	0.0188	0.1066	0

	0	0	0	0	0.0172	0	0	
9.00E-04	0	0.0186	0.0013	0.0711	0	0.0736	0.0619	
0.019	0.2698	0.0418	0	0	0	0	0	
0.0249	0	0	0	0	0	0	0	
0.4239	0.0334	0	0.0312	0	0	0.0443	0.2098	0
	0	0	0	0.0054	0	0	0	
0.0218	0	0.0035	0.0452	0.0291	0	0.0541	0.0737	
0.1499	0	0	0	0	0	0	0	
0.0323	0							
ANXA1	4.6838	1.2013	1.6171	7.2009	8.7559	8.4223	7.9593	
5.5459	5.6373	3.94	0.893	7.4005	9.304	8.2228	7.9977	
6.6484	8.8962	5.4281	1.9407	6.942	6.3671	7.7787	7.545	
7.642	7.5248	7.9282	7.8076	0.2863	7.3363	0.1196	7.7787	
5.6977	7.7479	6.715	7.7479	5.8391	8.1567	6.5756	9.6237	
8.8129	5.8864	9.7166	6.1649	6.4023	8.1316	8.7997	5.7002	
5.5459	7.4345	7.8385	6.8715	8.5319	5.4886	4.7805	5.9285	
0.6249	9.3003	8.9252	5.0055	9.3743	7.8735	5.8199	7.1854	
8.1176	4.8871	8.8606	4.8075	7.4724	2.2351	9.3743	7.9274	
6.7785	4.3632	7.8142	7.8483	0.6955	5.7875	7.6485	5.8148	
5.453	2.0986	8.2172	8.1512	7.7781	8.639	6.4272	7.1052	
5.5038	3.7888							
ANXA10	0	0	0	2.5723	0.0208	0	0.9172	0
	0	0.0343	0	1.4084	0.1863	0	0	0
	0.4159	0	0	0	0.2037	0.1429	0.7771	
0.0538	0.0029	2.1475	0	0	0	0	0	
0.0349	0	0	0.165	0	0.1224	0	0	
0.0155	0	0	0	0.0374	0.7294	2.415	0	0
	0.4703	2.3099	0.0672	0.1338	0	0.0604	1.162	0
	0.1307	0	0.0979	0.1104	0	0.1435	0.2915	
0.0905	0	0.2471	0	0.2335	0	0	0.5288	0
	0	0.0822	0	0	0	0	0	0
	0	0.1285	0.0979	0	0.1537	0	0.0919	0
	0							
ANXA11	5.918	5.7018	5.8848	5.2186	5.7806	5.6078	4.2653	
4.9039	5.1974	5.8858	5.4581	5.3976	5.6934	5.4469	4.7142	
4.968	6.4189	5.5249	4.9427	6.0598	5.5592	4.6983	4.7535	
5.1052	5.1418	5.3511	5.1896	5.5662	5.163	5.0389	4.8904	
5.3949	5.539	5.6375	5.2425	5.3905	5.5029	5.4348	5.6326	
4.4001	6.0726	7.4719	6.1426	5.7449	5.9933	5.9944	5.3709	
5.0908	5.0469	5.0221	5.6212	4.2617	5.2518	5.0204	6.3615	
5.6793	6.3426	5.2971	6.1096	5.4237	5.3516	4.5184	5.4033	
6.2332	5.8736	4.9788	5.1037	5.8292	5.1191	5.0762	5.8311	
5.4561	4.3553	4.7333	5.2542	6.0688	5.5877	5.0251	4.6277	
6.3027	5.7728	6.0754	5.3751	4.9476	6.0112	5.2479	6.3126	
5.9026	6.2037							
ANXA13	0.1909	0.7919	0	0.1977	0.053	0.3689	0.2484	
0.1005	0.1554	0.2598	0.0762	0.2794	0.1117	0.3104	0.2653	
0.0541	0.0647	0.2109	0.4448	0	0.1504	0.0217	0.1102	
0.4527	0.1461	0.0536	0	0.5849	0.1319	0.7545	0	
0.4136	0.1384	0.2634	0.4362	0.6023	0.2808	0.3275	0.3588	

0.4309	0.0591	0	0.5239	0.188	0.0507	0.0663	0.4205	0
	0.0631	0	0.2733	0	0.4373	0.2043	0	
0.0633	0	0.3344	0.3716	0.5789	0.0805	0.6037	0.194	
0.0273	0.0218	0	0.4542	0.075	0.2807	0.5885	0.2067	
0.1356	0.4883	0.1898	0.1189	0.0589	0.3782	0.4127	0.3775	
0.6087	0.2226	0.0696	0.0953	0.2246	0	0.2113	0.1142	
0.0913	0.1665							
ANXA2	9.2311	9.7489	8.7917	9.1906	8.5928	10.1379	9.7646	
8.8499	10.2953	8.4577	8.3765	8.2684	9.7646	9.2051	9.9954	
9.2171	8.8755	9.4444	9.4585	8.4011	9.7309	9.4585	9.8722	
10.0117	10.0465	10.4171	7.692	8.6432	10.346	8.7586	7.5281	
8.4949	8.7917	8.9393	10.8015	8.815	9.4006	7.9646	8.3394	
9.3713	7.6747	10.6226	10.8466	9.3248	10.7542	10.3681	9.0291	
7.7718	8.2754	9.4444	9.3713	9.2577	8.1535	8.4956	7.9426	
7.9016	11.2121	9.0179	8.3848	9.6428	10.0207	9.9651	9.6428	
9.5471	7.7901	9.4563	7.7943	10.5154	8.9278	10.1632	9.0045	
9.298	9.3384	9.5299	10.6669	7.9479	9.082	9.726	8.8773	
8.7006	7.8866	9.4685	9.7907	8.6396	10.5154	9.4685	10.2725	
9.4006	9.2171							
ANXA2R	1.2185	1.0372	1.0249	0.7936	1.2796	0.7722	0.1915	
0.3152	0.5547	0.5693	0.7016	0.8461	0.8133	0.2362	0.3691	
0.6464	1.8658	0.6444	0.989	0.7297	1.5036	0.1505	0.4714	
0.5847	0.7082	0.775	1.3478	1.3287	0.2298	1.0625	2.2283	
0.7027	0.4491	0.7284	0.4456	0.8913	0.2701	0.4916	0.4616	
0.2917	0.4592	0.6152	0.9597	1.0495	0.3584	0.5626	0.2732	
0.8192	1.0913	0.8427	0.3685	0.4498	0.5163	0.4274	1.1352	
1.2346	0.9256	0.3273	1.1535	0.4522	0.7885	0.3825	0.9632	
0.7087	0.7095	0.0024	0	1.5756	0.4913	0.9966	0.6723	
0.953	0.2595	0.3108	0.4834	0.5131	0.7425	0.4178	1.5217	
0.6723	0.9587	0.5003	0.5832	0.4823	1.4325	0.8085	0.2931	0
	0							
ANXA3	0.2632	0.0593	0	0.0412	0.037	0	0	
1.6973	4.4426	2.177	0.7156	3.1356	4.3353	2.2058	0.2063	
2.9836	0.0487	4.8124	0	0.1372	0.7048	1.7802	0.7156	0
	0.1594	1.6319	1.8801	0	0.2657	2.2205	0.1622	
0.0326	2.5359	0.6197	0.0631	2.648	0.9795	0.0369	0.0151	
0.0201	0.9232	5.8752	2.1775	1.5228	4.6606	2.4167	3.233	0
	0	0	2.6398	0	3.1553	0.1018	0.2701	0
	6.4526	0.1042	3.8197	0	0.1664	0	0.6931	
1.6933	0.0462	1.4306	0.0638	0.0361	2.1878	0.0692	3.5682	
1.5646	0.0726	1.9441	0	3.1897	1.664	1.2836	0.6386	
0.0606	0.0341	3.9163	1.9561	0.1725	2.8742	3.165	0.3417	
0.191	0.0849							
ANXA4	5.3917	2.7947	4.1913	3.8818	4.2729	5.0211	4.4698	
3.9549	5.5037	5.3394	5.1016	4.3261	4.6699	4.9495	4.7119	
4.4087	5.0305	5.4035	4.4649	4.7213	4.6548	3.3137	5.1493	
4.612	4.1156	4.2863	4.9267	4.9334	5.0753	4.4394	3.6307	
3.8421	4.694	5.4859	4.7242	4.836	3.7523	4.831	3.8527	
4.5949	5.3415	5.5784	5.0555	4.8547	4.5019	4.8899	4.4786	
3.8861	4.7753	4.0093	4.546	4.1005	4.097	4.273	3.9083	

4.1167	4.9769	3.763	4.6203	4.243	4.8827	4.19	3.9815	
5.7794	5.3354	5.8589	5.318	6.2571	4.5595	4.3891	5.148	
4.965	4.3564	3.854	4.9532	4.4446	4.9613	4.7414	5.4389	
5.237	4.3316	5.1745	5.3563	3.6115	4.2761	4.1159	6.2443	
5.7716	5.7384							
ANXA5	9.0259	8.8831	9.0744	9.1845	7.8012	10.029	9.9607	
8.8831	10.029	9.7829	8.6971	8.5914	8.5386	10.0653	8.9068	
9.6072	7.3986	9.3991	9.8313	8.8947	9.9263	8.6298	7.8597	
9.6214	7.8477	9.6501	8.4013	9.9607	7.97	8.9068	7.899	
9.6214	9.6072	9.534	9.9607	9.7046	8.4926	9.862	8.9397	
8.9528	9.2095	8.2614	8.8831	9.2751	8.4013	8.0939	9.0744	
9.6072	8.5914	8.7794	9.3864	8.5116	9.2639	8.853	7.3025	
9.6309	8.7718	7.8907	8.479	8.535	8.7597	9.0012	8.8382	
9.413	9.0572	9.0012	9.5824	9.8136	8.748	8.8002	10.0745	
9.4986	9.5824	8.3344	9.9738	7.8717	9.531	8.9848	9.1845	
9.1845	9.7052	8.582	8.4993	9.4807	8.4322	9.4461	9.7368	
9.5606	9.6214							
ANXA6	6.0649	5.4375	6.1859	5.4885	4.8614	4.1358	5.9253	
4.989	6.9174	6.3947	5.6786	5.4354	6.9388	6.2539	5.7936	
6.5473	6.9593	5.0455	6.132	6.3693	6.2033	4.387	5.207	
5.9224	5.2509	5.6155	5.2307	6.5963	6.1625	5.9522	5.7612	
3.2815	6.4547	7.3715	6.3406	5.7046	4.4568	5.9461	3.6173	
5.5123	6.3284	6.915	6.8305	5.7718	6.5928	4.835	5.9674	
6.1709	5.4239	5.2986	7.0384	4.0935	6.5058	5.3152	6.0155	
4.9307	7.8592	5.4451	5.128	4.9517	5.8043	6.6362	4.235	
7.1384	6.6796	6.2717	5.355	7.0435	6.6256	6.6574	5.0106	
5.8008	6.7742	4.7361	6.2912	5.6922	6.5941	5.6934	4.9911	
4.9955	5.7428	6.5981	5.9607	4.9903	5.5595	6.5298	7.4459	
7.4814	7.4272							
ANXA7	5.2393	5.6016	5.0579	5.6177	5.114	5.7202	5.2526	
5.6521	5.8937	6.0651	5.0339	5.9434	6.039	6.3176	5.0208	
4.9175	5.5002	5.77	5.3035	5.2593	5.6044	4.9915	5.4585	
5.3426	4.9516	5.1559	5.4534	5.4809	4.8094	4.6978	4.9733	
5.4867	4.971	5.4298	5.2484	6.2718	5.7473	5.1112	5.0098	
5.1625	5.2727	6.2879	6.0079	5.491	5.7112	6.5326	5.2677	
5.3028	5.1511	6.2999	5.446	5.1632	5.5064	5.3205	6.355	
5.3167	6.6789	5.5066	5.3283	5.2385	5.0491	5.0558	5.4304	
5.9471	5.0737	6.1009	5.0655	5.4926	4.8487	5.1192	5.2554	
5.1499	4.8561	5.2454	4.8914	5.3746	5.6518	5.0518	4.7077	
5.7249	5.6853	5.5863	5.0443	4.866	5.1892	5.5599	5.2734	
4.9629	5.1422							
ANXA8	0	0	0	0	0.1134	0	0	0
	0	0	0	0.2342	0.0478	0	0	
0.0497	0	0	0	0	0	0	0.0779	0
	0	0	0	0	0	0	0	
0.0699	0	0	0	0	0.0277	0	0	0
	0	0.1064	0	0	0	0.61	0	0
	0.0447	0.8884	0	0	0	0	0.3866	0
	0	0	0	0	0	0	0	0
	0	0	0	0	0	0	0.0946	

0.0175	0.0122	0	0	0	0	0	0.0014	0
	0	0.0727	0	0	0	0	0	0
	0							
ANXA8L1	0	0	0	0	0.0174	0.0703	0.0263	0
	0	0	0	0.0206	0.0753	0	0	
0.03	0	0	0	0	0	0	0.066	0
	0.0208	0.0688	0	0	0	0.0533	0.011	0
	0	0	0.0062	0	0	0.0617	0.0353	0
	0	0.0124	0	0	0.1018	0.0903	0	0
	0.0376	0	0.0451	0.0588	0	0	0.541	0
	0	0.018	0	0	0.0098	0.0086	0	0
	0	0	0.0291	0	0	0	0	
0.0114	0.0149	0	0	0	0.0039	0	0	0
	0	0.0505	0	0.0154	0.01	0	0	0
	0							
ANXA9	0.6246	1.695	0.8026	1.6344	1.4869	1.1457	0.7039	
1.2793	1.0037	0.9792	1.2868	0.9602	0.3036	1.5142	0.827	
1.6812	0.7888	1.1759	0.7253	1.2298	0.8482	1.0348	1.0484	
1.1894	0.5072	0.5705	2.988	1.2729	0.6493	2.7328	0.723	
0.8099	0.6348	1.0834	1.5988	1.3104	0.5792	1.0275	1.0592	
1.1531	1.1844	1.9047	0.5526	0.9115	0.4793	1.0197	0.949	
1.2607	0.4287	1.1026	1.3824	0.8756	1.2234	0.5819	1.0582	
0.8796	0.6955	0.5315	1.7775	0.5411	0.4706	0.4589	1.2049	
1.2049	0.7046	0.6153	0.9251	1.0672	2.4381	0.8524	0.8626	
1.0728	0.5795	0.8118	0.5748	1.4796	1.1364	2.0456	1.3273	
1.5514	1.7935	0.6462	0.8363	1.1215	0.9738	1.1388	1.0155	
0.555	0.8229							
AOAH	0	0.0498	0	0	0.0051	0	0	0
	0.0031	0	0	0.0152	0.0144	0	0	
0.0508	0	0.04	0	0.0238	0	0	0.1208	0
	0	0.0023	0.0727	0	0.0348	0	0	0
	0.2359	0.0067	0.038	0.0023	0	0	0.0018	0
	0	1.963	0.0187	0	0	1.513	0	0
	0	0.1182	0.0058	0.2842	0.0571	0.1401	0	0
	0.0874	0	0.0771	0.1215	0.067	0.0555	0	
0.0936	0	0.0547	0	0.0411	0.0566	0	0	
0.1139	0	0.0603	0.066	0	0.0742	0	0.0812	
0.1076	0.0635	0.08	0.158	0	0	0	0	
0.0195	0							
AOC1	0.0628	0.0163	0.1115	0.0343	0	0.0441	0	
0.0144	0.1406	0.0388	0.0461	0.0074	0.0188	0.0984	0.0327	
0.0096	0.0216	0.0133	0.1607	0	0.0328	0.0708	0.0644	
0.0139	0	0.0032	0.057	0.0587	0.0489	0.0047	0	
0.1389	0.0289	0.0095	0.0332	0.003	0	0.0744	0.0226	
0.0774	0	0	0.0407	0.065	1.00E-04	0.0861	0.0257	
0.1129	0.0176	0.0626	0.0494	0.058	0.0446	0.0331	0.0182	
0.0417	0.0478	0.0227	0.3215	0	0.0202	0.019	0.0362	
0.0263	0.0202	0.0088	0.0318	0.0092	0.0292	0.0305	0.015	
0.054	0.0955	0.0119	0.0207	0.0348	0.0351	0.0193	0.0693	
0.1487	0.0223	0.0146	0.0178	0.057	0.0228	0.0399	0	

0.0854	0.1149						
AOC2	1.0821	1.1051	0.5524	1.1217	0.5552	1.4211	1.4649
0.5979	0.7184	0.5313	2.9487	1.0458	0.8571	0.9756	1.0903
1.3158	0.9686	0.4962	0.9618	0.7612	1.0708	0.4358	0.7337
1.7815	0.2778	2.343	1.285	2.3107	0.2073	0.8103	0.1886
2.1171	0.9418	0.7129	0.4298	0.6668	1.5763	0.4619	0.7396
1.4244	1.4744	0.6873	0.294	0.5473	0.2843	0.464	0.1152
0.4183	0.3194	0.7217	0.2906	0.5979	0.4235	0.8674	0.2296
0.8825	1.8235	0.6506	0.389	0.5659	1.153	0.8558	0.1196
0.9994	0.5648	1.2239	1.704	1.0494	1.5758	0.7246	0.337
0.5781	0.6791	0.5542	0.9849	0.645	2.0556	0.3422	0.5008
0.35	1.5406	0.5581	0.0759	0.0588	1.2206	0.0795	0.4063
0.3288	0.4205						
AOC3	0.8131	0.4397	0.1712	0.2562	0.1284	0.8222	0.3525
0.0747	0.2783	0.299	1.565	0.22	0.3347	0.4685	0.356
0.5084	0.4184	0.1685	0.5282	0.2751	0.5869	0.1045	0.0988
0.9409	0.1388	0.4366	0.2295	1.0982	0.0174	0.1958	0.0771
0.6784	0.5359	0.3795	0.0433	0.1787	0.4361	0.2983	0.1753
0.7235	1.1169	0.205	0.0344	0.3472	0.0977	0.1863	0.1996
0.3337	0.0331	0.3082	0.0743	0.2169	0.1695	0.2442	0.2005
0.619	0.8062	0.2448	0.1936	0.2535	0.2937	0.3904	0.035
0.3977	0.1134	0.2679	1.0923	0.1852	0.4335	0.1145	0.1013
0.1993	0.1484	0.1906	0.3327	0.1369	1.2847	0.1295	0.1232
0.5006	0.7623	0.0929	0.0828	0.0368	0.2181	0.1198	0.1832
0.063	0.0404						
AOX1	0.0824	0.2078	0.0154	0.482	0.3213	0.1392	1.3834
0.0453	0.051	0.0919	0.3454	1.5375	3.2855	0.253	0.5987
0.3353	6.5628	0.0648	0.5311	0.7175	0.1647	0.1516	0.5722
0.4205	0.4332	4.1985	0.1834	0	0.3323	0.0348	0
0.4501	0.4598	0.2927	0.6861	0.0588	0.9967	0	0.1029
0.5987	0.0116	1.5517	2.2519	0.0211	3.2305	2.2335	0.0712
0.1693	3.0303	3.918	0.4007	0.2921	0.2176	0.103	0.1743
0.1976	6.0174	0.1084	0.4126	0.17	0.3179	0.4031	0.1128
0.1276	0	3.4903	0	0.2756	0.1291	0.3996	2.6872
0.3327	0.2335	0.2587	0.2343	0.1096	0.22	0.6849	0.1104
0.187	0.0996	1.8711	0.2232	0.2532	2.2194	0.2536	1.3293
0.0837	0.0861						
AP1AR	3.528	3.7475	4.164	4.3042	3.6963	4.2311	5.1116
3.8426	3.079	3.9728	3.6416	4.2752	3.3771	3.9406	3.2329
3.7583	3.8474	4.1977	3.8859	3.7632	4.1966	3.4936	3.4254
3.6785	3.1621	3.636	3.0358	4.6367	3.154	3.576	3.7989
4.3829	3.7935	3.6069	3.2394	4.1905	3.8528	3.9548	3.9903
3.1778	4.211	3.6815	3.2394	3.4823	3.3783	3.2525	4.1367
3.9575	3.5656	3.9842	4.0857	4.1355	4.2945	4.4414	3.3846
3.8734	4.3776	3.8201	4.167	4.2231	3.801	4.0431	3.4864
3.8584	3.9792	3.8992	4.1848	4.1274	3.5323	3.1321	4.077
3.7463	4.2298	3.1006	3.9307	4.1566	3.9943	3.7088	3.0751
3.5494	3.9662	3.9066	2.7841	3.7463	3.4715	4.1695	4.1379
4.2062	4.1653						
AP1B1	5.0752	5.4071	5.2956	4.3455	5.0569	4.5761	3.8443

5.6606	4.78	5.4755	5.2494	4.8853	5.2007	4.5684	5.0672
5.1272	3.9538	3.7328	4.842	5.2401	5.0163	5.0398	5.0867
6.0694	4.3591	4.7107	3.7392	5.5239	4.9898	5.907	4.4658
3.9453	5.0631	5.6521	5.1861	4.6817	4.6603	5.6385	5.6936
4.2511	5.2572	5.6096	4.8472	4.2515	4.4189	4.4085	4.3015
4.7544	4.2971	3.4693	4.2602	4.2386	4.8083	4.2634	4.3643
4.6065	4.8866	4.5959	5.1843	4.456	5.2408	5.2732	5.1858
4.5772	4.475	4.5194	5.6073	5.0877	4.8413	4.4892	5.6407
4.7612	5.0871	2.0679	5.2258	5.6653	4.9111	5.3364	5.1454
5.257	5.6847	5.6207	4.6251	5.0177	4.9245	5.2947	4.8098
5.1877	5.0697						
AP1G1	3.9551	3.9394	4.1601	4.4969	4.423	4.7197	3.9863
4.2032	4.6948	4.2631	4.2237	4.0229	4.0676	5.1978	4.2946
4.5466	4.1471	4.5989	3.9427	5.0055	4.6394	3.9383	3.9534
4.4369	4.3611	4.7827	3.7877	4.4828	3.6164	3.8885	5.0168
4.0685	3.7131	3.7923	4.7244	4.6071	4.4635	4.734	3.5005
4.5367	3.987	4.6444	4.2395	4.8277	5.1187	4.6	4.3528
4.5003	3.3479	4.5524	4.0869	4.6077	4.351	3.7059	5.0699
3.6596	5.0218	4.0224	4.5595	4.388	4.1349	4.2678	4.5108
4.2647	4.6574	3.9373	4.1224	4.6944	4.5428	4.7085	4.5881
4.4791	3.8142	4.1573	4.4438	4.8008	4.0383	4.6831	4.6615
3.7089	3.9185	4.1349	3.9063	4.5815	3.7274	3.7536	4.554
4.2631	4.4795						
AP1G2	3.0901	2.7497	2.6104	2.5205	1.8865	3.3752	3.8681
2.5764	3.45	3.4682	1.8019	3.219	2.0285	2.382	2.0207
3.1655	1.9287	3.5013	2.7137	2.7935	3.5698	1.5937	3.5507
2.6203	1.5975	2.4066	2.8234	4.1715	2.7964	2.7099	4.4524
3.3823	2.7473	2.4868	1.4047	3.1159	3.0763	2.2478	1.6779
2.7506	3.2494	3.8985	2.3174	3.6688	3.3274	2.3134	1.8583
3.1592	2.9581	0.4585	2.2053	3.094	2.6026	0.5465	2.7284
0.5159	3.6326	2.4899	2.2245	2.0773	3.0307	2.6148	2.8037
3.1651	1.8596	2.7502	3.6093	3.5208	3.2042	2.7246	3.13
2.9389	1.7313	2.4354	3.5245	3.6086	2.5576	1.4167	3.8441
2.4679	3.3896	2.7437	2.8219	2.4873	1.6695	2.7843	2.7961
2.845	2.8545						
AP1M1	4.8633	5.4908	5.2957	3.6533	5.8212	4.7933	4.1334
4.9417	4.7033	4.8496	5.0953	5.0347	5.4734	5.2696	5.1089
4.968	4.854	3.9068	4.3576	5.16	5.2639	5.2612	5.1367
5.1856	4.6284	5.5511	4.488	4.7527	5.3066	5.3285	4.7552
5.5921	5.4586	5.3189	4.7168	4.4625	4.9463	4.5356	5.1154
4.3846	5.2957	5.0558	4.9081	4.4585	4.7947	4.5598	3.9032
4.6158	4.7223	3.9652	4.391	4.5943	3.9809	5.1313	4.9578
4.7458	5.2777	4.7485	4.2791	4.9328	5.5868	5.7068	4.9426
4.9733	4.0151	4.8632	4.384	5.6209	4.7443	4.756	5.2619
5.289	4.4089	5.204	4.8552	5.0449	5.1711	4.5079	4.9242
4.3122	4.4621	4.8475	4.9388	4.2021	5.9021	4.8625	5.0453
5.2334	4.9974						
AP1M2	0.0608	0.4457	0	0.208	1.015	0.1131	0.0223
0.4878	0	0.6914	0.0141	0.3483	0	0.0718	0.073
0.0191	0.7796	0.1518	0	0	0.102	0.7242	0.1222

0.0341	0	0.8144	0.3521	0	0	0	0.0462	
0.0805	0.1772	0.1032	0.1432	0.9062	0.0586	0	0.1083	
0.0334	0.1437	3.0451	2.4151	0	0.0724	1.7141	0.238	0
	0.0258	0.8151	0.0608	0.4409	0	0.0683	0.4017	0
	3.5547	0.2326	0.1794	0.067	0.2556	0.6959	0.1403	
0.2983	0.1427	0.1101	0.1523	0.0826	0.0881	0	0.2514	
0.0612	0.0344	0.3302	0.0494	0.0337	0.0928	0.0872	0.2309	
0.2569	0.242	0.4181	0.0746	0	0.0487	0.3364	0	0
	0							
AP1S1	5.8327	6.3462	5.475	5.9759	6.8797	7.9788	6.4583	
7.4838	6.2076	5.7222	6.8866	5.4641	6.1571	6.0264	6.3027	
7.1694	6.089	7.1902	6.3661	5.7148	5.3287	6.9739	6.8059	
7.3283	6.8775	6.2287	6.1715	5.8347	6.7543	6.2018	5.3037	
6.36	6.0967	6.1019	6.5842	5.6862	6.4671	6.124	6.4753	
6.4048	6.6964	6.3535	6.2706	6.6289	5.8636	5.8069	5.4631	
6.9367	6.5897	5.3597	6.6309	6.2617	5.3708	5.3839	6.1505	
6.1352	5.2479	6.5441	6.7876	6.2563	6.1588	6.2341	6.6899	
6.6781	6.5892	6.0835	6.21	7.3034	5.7392	6.8487	7.264	
6.7095	6.7489	5.9248	6.3884	6.2331	5.9268	6.6738	6.1331	
6.8923	5.9299	6.169	4.0202	6.9725	6.7937	5.7179	6.0411	
6.3692	6.0014							
AP1S2	7.195	7.1321	7.1882	6.6156	5.8647	7.3201	8.1404	
7.0649	8.7834	8.2635	6.5751	7.5534	5.0093	7.6337	8.7435	
7.8936	3.6034	7.8298	8.1927	7.6198	7.3076	6.5274	6.228	
7.8139	6.1063	4.2958	7.4288	7.1848	7.2744	7.7083	4.7957	
7.3822	7.858	7.5932	8.6591	7.7589	6.6441	7.345	8.029	
7.4365	7.3964	3.9368	6.0646	7.2936	5.5235	4.9436	6.7527	
8.1836	6.9361	5.3822	7.5151	5.9513	6.6563	7.3534	4.1284	
7.058	4.2085	4.377	7.0203	5.1486	6.1064	7.4745	8.044	
7.3274	7.7716	5.1175	7.6754	6.5037	7.7344	6.4852	7.6377	
7.363	7.0144	6.9191	7.6625	6.8673	7.0223	8.2624	8.5728	
7.7344	7.7679	6.2811	7.587	7.5721	6.2129	6.5606	8.0807	
7.2976	7.4327							
AP1S3	2.7487	1.1288	3.566	3.0057	1.8572	2.7813	2.3283	
3.5969	3.5379	3.4425	2.7337	3.0543	1.5633	3.932	2.7155	
3.5764	2.3637	3.7575	4.3669	3.2692	3.514	1.1118	1.8176	
3.6918	0.977	0.9441	1.0767	2.5289	3.1574	3.0674	2.9003	
2.9148	3.829	1.8137	1.9277	4.1928	2.0545	1.1006	1.4113	
3.2834	1.8008	2.3754	2.3439	2.1943	3.1269	2.4391	3.0156	
2.8032	2.3624	2.3793	2.4012	3.102	3.0993	1.7106	3.9001	
0.0666	1.1739	4.5313	4.5897	3.4248	2.5171	4.0483	1.812	
2.8037	2.8395	2.9497	1.8446	2.8157	4.3272	2.3267	2.6159	
2.6126	3.6691	2.2432	2.4149	3.9641	3.3923	2.4743	2.9633	
3.0451	2.9652	3.1266	3.1719	2.4244	1.4186	2.9606	2.015	
2.1609	1.6966							
AP2A1	4.5928	4.4101	4.9328	3.9083	4.9183	4.6076	4.3047	
4.5074	4.5721	4.3918	5.1508	4.4771	5.323	4.4877	5.1798	
4.4641	4.8699	3.8214	4.2678	4.9183	4.9883	4.4889	5.1363	
4.3098	4.7961	5.1561	4.7659	4.5367	4.8148	4.2402	4.2556	
5.0501	5.1274	5.6557	4.6851	4.0556	4.6793	5.0918	4.9016	

4.3448	5.0088	5.2099	4.5768	4.3555	4.7436	4.4961	3.4064
3.7016	4.7874	4.0582	4.4924	4.1364	4.2875	4.6778	4.6694
4.0553	4.6626	4.0133	4.9537	4.7393	5.0252	4.5556	4.7533
4.5417	4.5843	5.2209	4.2481	6.3377	4.768	4.6139	5.1613
4.908	4.353	4.9902	4.2032	5.0744	4.4618	4.4723	4.4351
4.6288	4.3247	4.8701	4.9222	4.8038	5.4951	4.8648	5.2731
5.282	5.3321						
AP2A2	4.4413	3.9773	3.8539	3.6812	3.6171	4.5556	3.3318
3.9259	3.7534	4.1463	3.9106	3.1942	4.0876	3.8842	4.169
3.9279	4.0368	3.0974	4.1899	4.0173	3.7878	4.2748	3.816
3.9431	5.0741	4.5824	3.6171	3.7791	3.6702	3.4927	4.6059
3.8066	4.2025	3.9796	4.2014	3.5687	4.0373	4.5367	4.6021
3.6412	3.7822	4.4951	5.1431	3.8917	4.4521	3.7551	3.6168
3.4594	5.1781	3.8869	4.1824	3.3571	4.0275	4.7399	4.4916
4.406	4.4625	4.3005	3.6593	4.0167	3.7141	3.8482	4.1783
4.6328	4.1389	4.526	3.7915	4.4498	3.2645	3.6418	4.5989
3.6878	4.5014	4.4301	3.9894	3.5407	3.4287	3.5161	4.0551
3.9243	3.4994	4.0951	4.8431	4.1574	4.0243	3.946	4.318
4.1541	4.3658						
AP2B1	6.546	5.7035	5.1543	4.6538	5.6277	5.7265	4.8169
5.1115	5.9943	6.2539	6.2227	5.9413	6.0112	5.7725	5.4806
5.8887	6.4877	5.9757	5.2737	5.294	4.6803	5.0446	5.5513
4.7613	5.745	6.3096	6.7206	7.1352	5.3825	6.0736	6.0388
5.6914	5.0638	5.9546	5.9558	5.8864	5.3704	6.3726	5.5061
5.7116	5.3489	5.9657	6.0667	5.6853	5.7862	6.5883	5.4578
5.8082	5.3592	6.4698	6.084	5.1955	5.3439	5.5825	5.5975
5.5595	5.1664	5.2457	5.6386	5.2859	5.6776	5.2294	5.3781
5.5926	6.127	5.7342	4.8804	6.946	6.5731	5.7099	6.046
5.6954	5.9892	5.3818	6.3133	6.4448	5.9627	6.423	6.1044
4.8006	5.0437	5.9176	6.018	4.8766	5.7037	4.7961	6.2952
6.1708	6.4917						
AP2M1	6.8079	7.2715	7.9754	7.0032	7.32	7.6043	7.2915
7.5809	7.7133	7.0838	7.2884	7.7425	8.031	7.5369	8.9204
7.7051	8.049	7.4656	7.1744	7.6162	7.4063	7.1744	7.4845
7.9543	7.4553	6.8414	7.4656	7.1277	7.4553	7.3293	7.1052
7.3523	8.0146	8.2839	7.4845	6.8293	7.0859	7.7089	7.2414
7.2339	7.8502	8.2332	8.4735	6.6479	8.4735	7.7851	6.5836
7.5445	6.6746	6.9489	7.4845	6.9302	6.8039	6.705	7.596
7.7023	8.6233	6.8737	6.6533	7.3511	7.4057	7.4327	7.4134
7.8401	7.5496	8.1355	7.596	7.7361	7.2252	7.0446	6.9909
7.8306	7.0564	7.9194	7.166	7.461	8.0374	7.5496	6.9742
7.2014	6.933	7.0879	7.6428	7.7998	7.8925	7.1031	7.5369
7.4063	7.7002						
AP2S1	6.3361	7.7752	7.0365	7.3958	8.0268	6.8585	7.2158
6.9007	7.2903	6.6978	6.2092	7.6151	7.5878	6.8386	7.2498
6.9896	7.644	6.8096	6.9742	7.0227	6.9852	7.528	8.2961
7.2498	7.5076	6.9583	7.5751	6.8516	7.3715	7.8926	6.6912
7.9289	7.8643	7.1335	6.684	6.6068	7.0537	7.3958	6.9427
7.8551	8.3739	7.6564	7.2184	6.5685	7.833	7.3689	6.0404
6.8647	6.8386	6.9788	6.5671	7.7035	7.0489	7.0955	6.7534

7.5851	7.4409	6.5315	7.0326	6.7358	7.8611	7.3605	7.1475
7.1413	6.8088	6.8911	5.9072	6.8176	7.6517	7.0984	7.3943
7.2431	7.218	8.3129	6.6033	7.7602	7.1211	6.9575	7.4922
7.4792	7.6203	7.4332	6.4854	7.3681	8.0608	7.5298	6.9427
7.2268	6.9319						
AP3B1	4.2544	3.8311	4.0951	4.4829	4.2608	4.2715	4.5487
4.4499	4.0624	4.5859	4.603	4.7194	4.9686	4.8315	4.6255
4.723	5.1628	4.0375	4.909	4.4924	4.378	3.9421	4.2959
4.3316	4.5626	5.0609	3.899	4.249	4.4264	3.9089	4.7178
3.2462	4.5212	4.4357	4.7749	4.809	4.7009	4.4967	4.2271
4.7854	4.209	4.7183	4.3856	4.7344	4.6135	4.8742	4.5622
3.9001	3.8837	5.5335	4.6562	4.4157	4.2811	4.3788	4.0977
3.7395	5.049	4.2128	4.2534	4.4401	4.7517	4.2025	4.2819
4.5302	3.9775	4.699	4.0086	4.9848	4.6259	4.8971	4.5861
4.5597	4.2277	4.6336	4.2379	4.0714	4.2827	4.2098	5.1049
4.7393	4.4362	4.281	4.651	3.9353	5.0427	4.0459	4.6681
4.5331	4.5058						
AP3B2	0	0.7271	1.28	0.2484	2.5946	0.3854	2.5147
1.2997	0.2013	1.7322	0.5776	0.1738	1.5663	1.0599	1.4746
1.4793	0.3002	1.0158	0.2252	0.3908	0.9582	3.1399	1.5306
1.3927	2.3359	0	0.3617	1.7045	2.7942	1.2815	1.0017
0.8796	1.3704	0.8816	1.1233	1.3814	1.9336	0.236	0.8418
0.6963	0	0.9755	4.3256	0.7511	0.4893	0.1151	0.4515
0.1372	0.0943	0	0.6533	0.7338	0.8677	0.9726	0.4675
1.6599	0	1.6193	0.1588	1.6638	1.2458	0.7758	0.6061
2.5699	0.3696	0.9638	0.3874	1.3558	0.5787	2.0401	0.8772
2.6196	1.6846	1.8671	1.8853	0.0948	1.5309	0.4816	1.0382
0.806	0	0.3099	4.2826	0.0933	2.7382	0.3154	0.183
0.9315	0.4354						
AP3D1	5.8958	5.7344	6.6657	4.5265	5.0047	5.3053	5.0575
5.9032	5.3961	6.0354	6.2062	4.8163	5.6391	5.1858	5.7156
5.4053	5.519	5.0472	5.6752	5.7373	5.8351	5.4601	5.3143
5.6041	4.3079	5.5922	5.0857	6.0332	5.351	6.0545	6.0908
6.0275	5.6033	5.9756	5.4613	5.6197	5.8301	6.3044	6.2204
5.5325	5.7293	5.3148	5.3143	5.219	5.0899	5.0398	5.4118
5.3246	5.7139	4.2965	5.169	4.5716	5.2677	5.0826	5.8069
6.0843	5.3197	5.9837	5.7372	5.6555	5.6966	4.7304	4.9081
5.5585	6.1104	5.3859	5.9322	6.3808	6.1289	5.0216	5.9091
6.1319	5.6036	5.2551	4.7327	6.2313	6.4111	5.2314	5.8959
5.4767	5.6555	5.3932	6.0666	5.599	5.3027	5.48	5.5634
5.5346	5.6278						
AP3M1	4.5088	4.5388	4.7355	4.4818	3.4914	4.4884	3.8341
4.6506	4.2884	4.7561	4.1272	4.0698	4.2632	4.974	3.95
3.9464	4.3751	4.3552	4.5074	4.2203	4.3273	3.5748	3.4816
4.2146	4.0404	4.0629	3.685	4.6192	3.414	3.7114	3.6616
4.1163	3.9184	3.9445	3.9527	4.7915	4.6843	3.8947	4.0399
3.7001	4.1268	4.087	3.937	3.742	3.9201	4.7075	4.4258
4.1806	2.9436	4.3365	4.2349	3.6555	4.4039	4.7903	5.18
4.4261	4.4247	4.2856	4.2076	4.3933	3.5426	3.8228	4.3701
4.3384	4.5045	3.6894	3.9372	4.3952	4.0405	4.0456	3.8707

4.0156	3.7165	3.8068	3.4449	4.358	4.642	4.2081	3.7132
4.2453	4.2368	4.0499	3.5881	3.5706	3.443	4.2332	4.4109
4.5042	4.3808						
AP3M2	4.7906	3.6375	3.4404	3.1418	2.2345	4.055	3.9228
3.6609	3.5413	3.5308	3.8139	2.7549	3.97	4.7805	2.465
4.1678	3.742	4.8103	3.8555	3.4123	3.1078	3.7538	3.5195
3.7678	3.3571	3.1274	2.9632	3.2249	3.6906	4.1769	3.2311
3.67	4.6899	4.5453	4.4603	3.5187	3.4104	3.6576	4.66
4.4707	4.2758	3.0619	3.3639	3.7036	4.1606	3.8705	4.4834
3.8163	3.451	3.8567	4.1342	3.2939	4.4081	3.4595	2.9902
3.8406	2.1103	2.9005	3.5581	3.2984	2.5018	3.8423	4.4876
3.9392	4.3162	3.3087	3.6712	4.5826	3.0286	2.8649	3.8115
3.8568	4.2675	3.766	3.5394	4.1635	3.0545	3.848	3.1653
4.2946	4.089	2.9647	2.9481	4.126	3.2339	4.5126	4.1858
4.2738	4.2869						
AP3S1	5.2998	5.6937	5.7171	5.4879	6.5301	5.6099	5.648
5.6625	5.6983	5.848	6.5233	5.8593	5.9856	6.0648	5.1855
5.6456	6.0412	5.0473	6.1723	5.3668	5.7459	5.2268	5.5176
5.6947	5.4062	5.7028	6.085	5.8723	5.4507	6.6886	5.9284
6.5099	5.8914	6.3583	5.7543	6.1084	4.9708	5.1407	5.1975
5.3937	5.9441	5.85	5.6719	5.6695	5.0735	6.1292	5.4706
5.2235	5.7239	6.408	5.8967	5.5132	5.6194	5.4443	5.3606
5.2897	5.862	5.4264	5.7678	5.5284	5.7953	5.3272	5.3554
5.664	5.3257	6.0194	5.8836	6.353	5.4131	5.5225	6.0636
6.1579	5.4187	5.5203	5.2215	6.2054	5.9263	5.0328	5.8596
6.3253	6.1006	6.0317	6.1529	5.4618	6.2788	5.7532	6.1828
5.8161	5.7853						
AP3S2	3.0702	3.4663	3.0907	2.8811	2.939	2.792	3.2932
3.666	3.2618	3.687	4.0867	2.5451	3.138	2.6309	2.6703
3.2887	2.9105	3.846	3.0618	3.1239	2.6643	3.3176	3.1181
2.895	2.9063	3.2161	3.2984	3.2957	2.4352	3.6161	2.7559
3.2059	3.2106	3.0685	3.3733	3.5401	2.782	3.1582	3.6358
2.603	2.5323	3.567	3.3354	2.2951	3.5842	3.1037	3.047
3.1369	2.3385	4.444	3.133	2.8452	3.0054	3.0219	3.1149
3.1414	2.7964	3.2454	2.7365	2.8923	2.3562	3.2146	3.1961
3.275	2.8234	3.5403	2.2028	2.9925	3.3037	2.4031	2.1404
2.4483	3.0874	2.9675	3.2347	2.2373	3.0541	3.0493	2.8388
3.2944	2.6431	2.4071	3.5138	2.2892	2.8459	2.3265	3.4074
2.6846	3.0011						
AP4B1	2.71	2.9014	2.3835	2.4199	3.2078	2.9402	3.1882
2.169	2.7863	2.7508	2.5099	2.583	2.6763	3.7968	2.4322
3.0311	2.7551	2.4668	3.3339	2.5462	2.8108	2.0695	2.2013
3.0901	2.0754	3.1815	2.157	2.4025	2.3557	1.3607	2.3107
3.402	3.3834	3.3499	2.903	2.8265	2.8282	2.2323	2.2474
3.1529	2.1107	2.2212	2.6841	3.2117	2.0058	3.1723	2.7219
2.9346	2.7018	3.6566	2.6233	3.1282	2.9189	2.3897	2.8548
2.6828	2.9711	2.9109	2.379	2.4287	2.587	2.1533	1.7738
3.6767	2.1633	2.5197	2.7856	2.6935	2.1314	2.2668	2.103
3.0397	2.9258	2.4962	2.5075	2.3453	2.6503	2.235	2.3387
1.9601	2.6763	2.2443	2.5506	1.9096	3.4857	3.2418	2.442

2.7867	2.6186						
AP4E1	2.2229	2.8382	2.4944	2.9213	2.7555	2.9564	3.0593
2.4419	2.8092	3.2046	2.4481	2.0658	2.9424	2.3353	2.1703
3.0932	2.3117	3.2172	2.9348	3.08	3.2833	2.8524	2.5749
3.2119	2.6048	2.5713	2.3814	2.4824	2.8371	2.4574	3.2498
2.5391	2.1755	1.9644	2.8453	3.2274	2.5823	2.1876	2.4304
2.488	2.3678	2.2946	2.2303	2.9589	2.587	3.199	2.7824
2.8596	2.429	3.2515	2.8811	2.5637	2.6199	3.044	3.5111
2.9295	2.4834	2.7948	2.8067	2.6859	2.8404	2.7296	3.0823
3.3279	2.3134	2.9295	2.2982	2.4214	2.99	2.4797	2.3181
2.3891	2.9377	2.2279	2.9168	2.8616	2.719	2.9275	1.9266
1.9582	2.4976	2.9239	2.4151	2.6855	2.3208	2.5792	2.8449
2.6062	2.4541						
AP4M1	3.0051	3.9465	2.5643	3.5044	3.3334	3.633	2.7686
3.2178	2.7018	2.8235	2.6886	2.1088	3.1627	2.9392	3.4789
2.9447	3.1258	3.4072	3.2247	2.257	2.7464	3.7724	3.078
3.0367	4.2696	3.8639	2.6951	2.58	3.3361	2.7299	3.3471
3.102	3.0078	2.3024	3.4859	3.0541	3.3392	2.8985	3.3031
2.5821	3.9575	3.6046	2.8658	3.4795	2.9212	3.1868	2.8337
3.0861	2.933	2.8152	2.7443	2.9188	2.2969	2.5264	3.2975
3.0358	2.3438	3.3331	2.6528	3.6428	3.1877	2.8987	3.6486
3.6825	3.2116	2.6644	3.572	3.1284	2.7454	3.3709	3.5352
3.3226	3.3229	2.1003	2.8462	2.9487	2.808	3.1572	2.7014
3.2491	3.006	2.4924	1.3989	2.8712	4.2365	2.5147	2.8735
2.782	3.2923						
AP4S1	1.5524	1.5281	1.0793	2.1024	1.0623	1.2639	1.9224
1.4486	1.0549	0.8647	0.4655	1.1179	1.213	1.3994	1.6285
1.1106	1.7281	1.9253	1.9698	0.9176	1.1174	0.5989	1.7536
0.8998	0.9793	1.3873	0.4269	1.649	1.3099	0.4187	1.4885
1.3696	1.4567	0.6199	0.8067	1.0234	1.2078	1.7292	1.1948
1.592	0.8339	0.6868	1.3978	1.4113	2.1543	2.1282	1.5676
1.2632	1.1208	2.6174	1.0356	1.6098	1.4702	1.6118	1.1259
1.0217	0.7968	0.8174	0.7896	0.9461	1.1625	1.4819	1.6071
0.652	1.1956	1.5091	1.0376	1.0361	1.0667	1.0108	1.3463
1.2786	0.8277	0.605	1.6031	1.0201	1.5958	0.7365	1.8075
1.4834	1.9985	1.0505	0.8357	0.6183	1.6871	1.608	1.0014
1.1491	1.1055						
AP5B1	3.0461	2.7324	3.5528	1.9604	2.9673	2.5167	1.991
3.0449	2.3762	2.6915	2.8763	1.4658	1.8113	2.8145	3.3128
3.3566	2.2214	2.4133	2.0968	2.6832	3.6149	2.7142	2.1063
2.695	3.066	2.1698	1.2659	3.1249	2.6353	2.4784	2.0168
0.8388	2.0035	2.3472	1.8527	2.5072	2.67	3.707	2.3521
1.978	2.5528	2.0096	1.8521	1.8715	2.35	2.094	2.5195
2.5888	2.9429	2.07	2.3979	1.4779	2.3486	2.5631	2.8053
3.2277	1.8073	3.0441	2.7606	2.4423	2.3745	2.0743	3.6016
2.3491	2.9197	2.117	2.3102	2.845	2.2658	2.2951	2.0863
2.4423	2.3	0.9529	2.275	3.0118	3.547	2.3304	2.5793
2.6277	1.8909	2.1382	4.7008	2.1619	2.0693	2.2084	3.5649
3.2908	3.5444						
AP5M1	3.7083	3.3585	3.8	4.056	2.9706	4.4843	4.2337

3.9633	3.8709	3.619	3.1668	3.9199	3.6994	4.0148	3.9859
3.7932	3.7219	4.4488	3.9949	4.4437	4.3438	3.4626	4.5954
3.646	3.7736	3.808	2.6089	4.2115	3.9957	3.8709	4.4727
4.0144	3.8734	3.5371	3.4234	4.2051	3.2553	4.0178	3.6575
4.0386	3.944	4.2342	3.618	3.6155	4.2678	4.1283	3.72
3.8845	3.9898	4.2841	3.3762	4.3455	3.692	3.2986	4.0268
3.8807	3.5959	3.6627	4.3747	3.7873	3.7109	3.8952	3.8053
3.6474	3.6683	4.5169	4.1903	4.2051	3.5727	3.2688	3.8439
3.7222	4.0098	3.6641	3.9412	4.407	4.1421	4.1833	4.2295
3.2361	3.4071	4.0718	3.4984	3.5387	3.2483	3.6818	4.3463
4.312	4.3666						
AP5S1	2.566	3.7857	3.2753	2.2598	3.1521	2.5238	2.7885
4.1004	3.486	2.806	2.8035	2.4931	3.2839	2.2383	3.5526
2.7614	3.119	2.5638	3.0523	3.2742	3.3124	2.9422	3.3571
2.3913	3.3264	3.4087	0.7771	3.0417	3.0534	2.5308	2.9487
3.7537	4.1549	3.0865	3.0809	2.2218	2.8758	4.0286	3.3895
2.6609	2.2913	2.63	2.4511	3.2767	3.1122	2.5419	2.6645
2.8433	3.1536	2.9395	3.118	2.482	3.0854	3.3257	3.608
4.0201	2.3735	3.3345	3.3655	2.5979	2.3937	3.403	3.1452
2.6018	2.4306	1.9777	1.8622	2.8119	3.4298	2.8243	2.7951
3.388	4.0197	2.8374	3.5429	2.2798	3.2287	3.3439	2.8014
2.5622	3.1438	2.9145	3.3742	3.4561	3.1886	3.674	3.1597
3.2353	3.1449						
AP5Z1	3.2838	2.6304	3.8563	1.284	1.9536	2.0513	1.9542
3.6555	2.2189	2.221	2.4523	0.8302	2.6408	2.2103	2.7126
2.7034	2.4889	1.7347	2.3066	2.6456	2.96	2.221	2.9425
2.6912	3.033	2.9304	0.932	2.1966	2.8683	1.2956	2.9713
3.5332	2.4857	2.2076	3.5856	1.5649	3.5245	3.9958	3.0379
1.5801	2.9332	1.4735	1.721	2.8606	1.4699	1.8563	2.0074
2.1756	3.6895	1.3244	2.7219	1.3962	2.4822	3.0324	2.3947
3.5436	1.9711	2.5083	2.3655	2.6351	2.5429	2.7056	2.5847
1.9383	2.6686	2.8538	2.8933	3.3824	2.2246	2.9817	2.302
3.0014	4.7302	1.4251	1.9499	2.745	2.3064	2.6461	2.131
1.8941	2.0233	1.9858	4.9721	2.3896	2.7458	3.512	2.7135
3.3022	3.018						
APAF1	2.3377	1.7311	2.0748	2.3054	2.4015	2.0395	2.6937
2.8292	2.8102	2.414	2.5251	1.8161	1.2021	2.7854	1.5287
2.3562	2.4378	2.8648	3.0786	2.3015	2.3287	2.0003	2.0478
2.4282	2.8754	2.2753	1.9773	3.0247	1.9078	1.9261	3.3681
1.6615	2.5364	2.5104	2.5837	3.0494	1.8506	1.5359	1.6144
3.0105	1.6567	1.9386	3.0235	2.5601	2.0502	2.9334	2.9078
2.6692	1.6902	2.9713	2.055	1.8386	3.0278	1.9109	2.69
2.8278	2.4703	3.4746	2.6884	3.2988	2.2948	2.4883	1.9966
2.5995	2.9509	1.86	2.631	1.8814	2.6692	2.4703	2.638
2.3418	3.0646	2.2668	1.8677	1.4191	2.2068	1.5722	1.7349
2.3875	2.4598	1.4243	1.8284	2.0032	2.4897	2.9216	2.2435
2.8578	2.6214						
APBA1	0.7424	0.062	0.4588	0.4032	0.1148	0.7586	0.6805
1.7201	1.1489	1.3128	1.3849	0.3176	0.9607	1.8322	0.3264
1.7627	1.7651	0.1372	1.2062	0.445	0.4851	0.1494	0.3842

0.9022	1.2538	0.7134	1.8009	0.8719	0.7145	0.1927	0.4662	
0.1673	1.2938	1.8844	0.2668	0.9809	0.1702	0.8095	1.1243	
1.3122	0	0	1.9685	1.7266	0.4847	0.0565	0.699	
0.8447	1.8049	0.11	0.4948	0.788	1.457	0.2035	0	
1.3832	1.3169	0.3905	0.8911	0.7475	0.9911	0.0241	0.2321	
1.1642	0.5392	0.9206	2.243	1.0614	0.9598	1.106	1.9851	
0.7576	0.2419	0.9546	2.1863	0.971	0.7862	1.1006	0.3666	
0.6779	1.0707	0.7195	1.2651	2.6697	0.984	0.9716	1.1128	
0.7048	0.9601							
APBA2	3.9193	5.1594	4.5854	2.2849	3.0139	2.7029	2.7848	
0.1997	2.5971	0.3496	3.202	1.5671	3.347	0.2863	3.3219	
1.0962	0.0639	1.4	1.2124	0.3236	1.1369	2.2937	4.0133	
2.9753	3.6798	1.0962	1.8432	2.48	3.0441	4.0464	2.7967	
0.0057	1.7984	0.0164	2.3026	1.1992	3.2025	2.6769	3.7027	
2.7489	1.5129	2.2544	1.9537	2.7767	3.7367	4.2232	3.1638	
0.9076	4.8247	0.1437	2.2033	4.1679	0.0516	4.1585	2.869	
3.6847	0.2135	0.2033	2.9086	0.1075	2.594	3.0849	3.4227	
2.6769	3.9415	2.7469	0.4785	4.3615	4.291	2.7454	0.0573	
0.6833	3.6632	0.3396	3.9804	3.691	0.4152	4.3871	2.4433	
3.6118	1.4471	3.638	3.3093	3.5615	2.5515	0.0924	2.9631	
3.9876	3.3443							
APBA3	2.3293	2.132	3.081	1.5047	2.6454	1.5242	2.1164	
2.2736	1.8737	1.6004	2.2585	1.5322	2.4646	1.3184	2.5327	
2.1405	2.3246	1.2677	1.7672	2.813	2.4602	2.6172	2.3373	
2.2477	1.6189	2.7494	1.747	2.5541	2.1678	1.8394	2.4673	
2.7773	2.508	2.2847	2.0611	0.8672	2.8731	2.3507	2.6744	
1.5554	2.1214	1.5958	2.3891	1.9277	1.6533	2.1229	0.8918	
1.9758	3.3006	1.3304	1.7567	1.5498	1.5849	2.294	2.5687	
3.0524	2.982	2.3826	1.2797	2.1093	2.525	2.0242	1.5719	
1.7233	1.8655	2.8948	2.3731	2.3933	2.1181	1.9466	2.0955	
2.6832	1.9673	2.6216	1.5973	2.2325	2.5026	1.913	2.3195	
2.111	2.0334	2.1056	3.1532	2.4824	3.1537	2.5687	2.1286	
2.2228	2.1957							
APBB1	5.0804	4.7682	4.6617	3.341	3.5201	1.4847	3.834	
5.0473	5.5789	4.4315	5.5798	3.9446	4.6096	4.1406	3.6061	
5.7951	0.2606	3.2828	3.2862	4.3092	5.0373	4.1337	4.3997	
4.8167	4.5776	5.2909	4.4334	5.0169	4.3861	4.9838	4.3915	
4.5658	4.811	6.0723	2.8459	4.2116	4.923	4.8225	4.923	
3.7194	4.1827	4.5841	5.3103	4.8605	3.0367	0.6786	4.3902	
4.6081	4.7619	4.7687	5.0855	3.7214	4.2937	3.8572	4.5491	
3.8015	0.2028	4.1037	5.0999	3.6828	4.0409	2.2027	4.114	
4.6045	4.4242	5.2268	5.3755	5.292	4.3728	4.693	4.7443	
4.8725	4.1171	5.4038	5.1858	5.4122	4.0139	5.0434	4.6721	
5.3654	4.6942	4.7751	5.27	4.5219	4.7951	4.3829	5.5366	
5.5536	5.9262							
APBB1IP	0	0.0507	0	0	0.0154	0.0496	0	0
	0	0.0332	0.0164	2.0021	0.3436	0	0.0178	
0.0217	4.9096	0	0	0.1321	0	0.2522	0.0997	0
	0.0191	0.0673	0.0454	0	0.6878	0	0.0579	
0.0679	0.3664	0	0.1928	0.0675	0.0279	0	0.0202	0

	1.2424	3.4146	0	0.0292	0.2716	0	0.0643	
0.0595	0.0666	0	0.1926	0.2079	0	0.0065	0	0
	0.0197	0	0	0.0102	0	0.1049	0	
2.1046	0.0517	0	0.0198	1.1221	0	0.0157	1.0473	
0.0747	0	0.0063	0	0	0	0.0308	0	
0.0967	5.00E-04	1.375	0	0	0.0277	0	0.6652	0
	0							
APBB2	2.9187	1.4668	2.3275	2.7545	2.1259	3.0689	2.3236	
2.2782	2.4745	2.5887	2.1871	3.301	3.9661	0.3041	2.9727	
2.7442	5.2466	1.8095	2.3574	3.5053	3.1037	1.4048	2.7863	
3.2298	1.9462	5.334	2.0453	0.6962	1.4716	1.6318	2.751	
2.3534	2.2534	2.1997	3.0103	2.3539	2.7976	2.5413	2.6524	
2.6158	2.8859	4.293	2.9272	2.9059	2.5271	2.1459	2.5008	
1.5378	2.3775	2.8725	1.5556	2.4025	2.3222	2.9447	2.2552	
2.1629	5.1064	2.4098	1.9922	2.6695	1.9241	1.8099	2.4271	
1.087	2.2228	4.3227	3.2597	3.1634	2.8603	1.9571	1.2998	
0.87	1.2101	2.3549	3.096	4.5282	1.8354	3.4428	1.9231	
2.1883	2.3071	3.6857	2.9278	2.6385	2.5706	1.9571	3.3659	
2.953	3.2807							
APC	2.6613	2.4543	3.3007	2.9183	3.1281	3.5454	3.0908	
2.9499	2.9428	2.7856	2.6697	2.2367	3.0245	3.5961	2.8416	
2.1538	2.9171	2.0378	3.1524	3.412	3.3148	2.0926	2.7653	
3.0534	2.7029	2.9651	1.8908	1.6892	2.8034	1.7621	2.3427	
2.7814	3.3221	3.219	3.2549	3.3072	2.4886	3.0893	2.0603	
3.5017	2.4065	2.387	2.553	3.2978	2.6707	2.4938	1.9906	
2.8532	2.3778	3.2409	2.7551	2.8197	2.9452	2.6624	1.736	
2.8825	2.7889	2.7706	3.0345	2.663	2.9183	2.5947	3.6641	
2.9725	3.3181	2.7556	3.4025	2.9277	1.4381	2.5693	3.635	
2.3447	2.8573	2.5524	2.469	3.2388	2.6079	3.2272	2.9281	
2.1531	2.78	3.1032	3.0283	3.1769	2.7568	2.621	3.4846	
3.0303	3.5589							
APC2	0.7099	1.3233	0.7151	0.7234	0.3325	0.9664	0.7615	
1.0077	0.4127	0.2068	0.4517	0.1963	0.5599	0.5015	0.3147	
0.8898	1.3923	0.4979	0.4227	1.0636	0.3694	0.4997	0.728	
0.4482	0.2808	0.3431	0.2512	1.0921	0.4626	0.4729	1.5491	
0.9824	0.6612	0.338	0.8469	0.1863	0.4803	2.0835	1.5809	
0.4459	0.8216	0.5311	0.7675	0.3015	0.2677	0.8732	0.203	
0.7639	0.3464	0.1937	0.6571	0.185	0.2729	0.8023	1.1145	
1.0892	0.3122	1.8606	0.1846	1.3606	0.7836	0.2465	1.1294	
0.5141	1.2337	0.3763	1.0909	1.2326	0.3949	0.4668	1.0539	
0.3003	0.9689	0.138	0.3359	1.0204	0.6573	0.9684	1.1704	
1.1737	1.1549	0.1753	0.4612	0.1464	0.3763	0.3251	0.4337	
0.4077	0.3733							
APCDD1	0	5.0288	6.1204	0.4556	0	1.9752	6.4495	
0.225	1.9649	2.0576	0.9597	0.1035	1.4422	0.5313	1.6672	
3.115	0	4.3378	2.0528	0.1373	0	0.8394	0.6296	
1.0292	0.2765	2.6354	0	7.8969	0.3992	7.87	0.4103	
1.8051	1.2423	1.0829	0.8777	0.6338	0.2676	0.1607	2.0061	
0.5637	0	0	0.7798	0.5097	0.4488	1.0095	6.0697	
3.3118	1.3151	0	0.4074	0.3117	0.8435	0.8215	0	

1.4076	0.0311	0.6581	3.4116	1.3336	0.8438	3.5502	1.3863	
0.9173	0.6387	0.0104	0	6.9789	8.1639	0.2574	0.0376	
2.1762	1.38	1.7351	1.4413	6.1241	2.9471	0.5072	1.7892	
1.8559	4.3203	0.3011	0	0.9808	0.5435	0.5994	0.8982	
2.4082	3.1819							
APCDD1L	0	0	0	0.1349	0	0	0	0
	0	0	0	0.4635	0	0	0	0
	1.5977	0	0.1481	0	0.0129	0.5503	0	0
	0.0014	0.4929	0	0	0	0	0.1631	
0.37	0	0.5409	0.0533	0	0	0	0	0
	0.0394	4.1247	0.0559	0	0.0571	0.9237	0	
0.1122	0.0379	2.4143	1.7702	1.9761	0	0.0476	0	0
	4.0621	0	0	0	0.0545	0.347	0	
0.0829	0	3.5125	0	0	0	0.0756	0.5988	
0.0655	0	0.2689	0	0	0.0432	0.0534	0	0
	0.0482	0	0	0.0549	0.0658	0	1.2525	0
	0							
APCS	0	0	0	0	0	0	0	0
	0	0	0	0	0	0	0	0
	0	0	0	0	0	0	0	0
	0	0	0	0	0	0	0	
0.0474	0	0	0	0	0	0	0	0
	0.0453	0	0	0	0	0	0	0
	0	0	0	0	0	0	0	0
	0	0	0	0	0	0.0545	0	0
	0	0	0	0	0	0	0	0
	0	0	0	0	0	0	0	0
	0	0	0	0	0	0	0	0
	0							
APEH	5.7291	5.8305	5.9621	4.718	5.4605	5.3673	5.1681	
5.7686	5.6612	6.4433	6.2213	5.998	5.4032	5.1895	4.1556	
6.207	6.1411	5.2802	5.2062	5.9077	5.8082	5.7162	5.8028	
5.7584	5.056	0.9696	4.8909	5.8387	6.0866	5.1146	4.7484	
6.4639	5.4133	5.471	5.4235	5.7008	5.5701	6.1925	5.9569	
5.2172	6.2816	5.9579	5.4197	5.7079	4.5975	4.2043	5.707	
5.9119	5.7936	4.9057	5.007	5.0666	5.0378	4.6752	4.6866	
5.5278	4.3984	4.7417	5.8587	4.3054	5.991	5.3454	6.1843	
5.3168	5.7996	4.555	6.4723	5.7272	5.5785	5.3011	5.8634	
5.2326	6.0931	5.5956	6.3989	5.9856	5.6614	6.0093	5.15	
6.4135	5.7388	5.8238	4.992	4.9236	3.4146	5.3379	5.477	
6.0241	5.9016							
APELA	0	0	0	0	0	0	0	0
	0	0	0.0284	0.0143	0.0101	0	0.0066	0
	0.0395	0	0.0448	0	0	0.003	0	
0.0186	0.0062	0.0246	0	0	0	0	0	
0.0016	0	0	0	0	0	0	0	0
	0.0156	0	0	0	0.0071	0.0317	0	0
	0	0	0	0.0345	0	0	0	0
	0	0	0.0206	0.0786	0	0	0	
0.0341	0	0	0	0	0.0137	0	0	0

	0	0.0017	0	0	0	0	0	0
	0.0167	0	0	0	0	0.0442	0.0732	0
	0.07							
APEX1	7.1822	6.7481	6.4339	6.7913	6.2951	6.3691	6.157	
6.9102	6.5473	6.3095	6.3545	6.7003	6.5746	6.6113	6.9017	
5.8622	7.3602	7.0919	6.681	6.4578	6.6523	5.6471	6.8163	
6.6963	6.6661	6.5443	7.0151	6.8544	6.7503	6.2887	8.4925	
6.8691	6.8975	5.764	5.6751	6.8733	6.8088	6.6042	6.7715	
6.9492	6.8068	6.197	6.2318	6.9742	6.8669	6.5504	6.4955	
6.9694	7.2477	6.9866	5.8952	6.9188	6.6372	6.7363	7.043	
6.4603	6.5984	7.3599	6.2565	7.298	7.1192	6.8491	7.5166	
6.3142	7.1965	6.4355	7.3085	6.8232	5.9177	6.3636	6.5578	
6.3059	6.3391	6.6396	7.0159	6.0493	6.5302	6.0403	7.0935	
6.5053	6.9473	6.3844	6.2767	6.3636	6.7035	6.5885	6.4297	
6.7246	6.601							
APEX2	4.6948	4.8989	4.1783	3.785	4.8806	3.7969	4.3344	
3.9397	4.1139	4.1443	4.1421	4.5482	4.6094	3.3046	4.1585	
4.5323	3.6982	3.8192	3.3084	4.1421	4.4165	4.3229	4.363	
4.7953	3.6491	4.197	3.3785	3.9139	4.355	3.4527	4.4099	
4.7636	4.4688	4.2794	3.9423	4.0332	4.6556	3.7485	4.0045	
3.551	3.9454	3.7715	3.828	4.4283	4.5965	3.9527	3.7708	
4.3971	3.0297	3.9639	3.7099	4.3216	3.2437	3.7151	5.0888	
4.4335	3.9617	4.3254	3.0362	4.2865	3.6207	4.3665	4.0104	
4.811	3.8123	3.4694	4.4074	3.7254	3.8201	3.7118	4.5156	
3.6882	3.1533	4.3836	4.0243	4.1517	4.519	4.254	4.0614	
3.6195	4.0409	3.8163	3.8892	3.5405	4.094	3.5422	3.5758	
4.1497	3.6865							
APH1A	6.9055	6.8711	6.5462	6.9964	6.6006	5.651	6.351	
6.2029	6.5405	6.7671	7.5007	6.7842	5.9344	5.9117	6.0261	
5.7135	6.0008	6.9567	6.3543	6.0333	6.3621	6.4785	6.1018	
6.5917	6.3591	6.4022	6.5557	6.6585	6.3884	7.4427	6.8166	
6.309	5.8159	6.6734	6.309	6.828	6.4284	6.5367	6.5333	
5.8609	7.2003	6.4037	6.3654	6.0651	6.4637	6.7821	6.4335	
6.2714	5.6451	5.8842	7.1395	6.7378	6.6734	6.8945	6.498	
6.5407	5.9375	6.853	6.8945	6.9832	6.3647	6.2872	6.5268	
5.9507	6.7027	6.3236	6.6362	6.1727	6.9174	6.2642	5.6958	
6.2453	6.3482	5.9168	6.1386	5.4105	6.8265	6.9721	6.0161	
6.6479	6.5105	6.0692	5.652	6.859	6.1509	6.5407	6.2559	
6.2617	6.4621							
APH1B	2.3429	1.3266	2.728	1.504	0.9805	2.3462	1.2387	
2.5676	2.5072	3.2981	3.2402	1.9228	1.9533	2.9233	1.6491	
2.9465	2.8299	3.4789	2.8633	2.0987	3.0336	1.4857	1.6893	
2.2449	2.1738	1.8075	1.3663	2.2022	1.9946	2.2763	2.1956	
1.7142	2.0952	3.1841	2.2775	3.0776	2.3187	1.8719	1.5052	
1.7442	3.399	2.6801	2.2712	2.6776	2.5579	3.1385	1.8811	
2.5013	2.0977	2.7774	3.1064	1.7772	2.6826	2.7238	2.7324	
1.6077	3.2089	0.4615	3.0039	0	2.2834	2.6035	1.3524	
2.3642	0.7895	3.6402	2.5009	3.3237	2.6724	1.8663	2.8639	
2.108	3.4964	2.5354	2.8848	2.9195	0.3851	1.0833	1.5282	
2.731	1.6821	2.6728	3.0854	2.1417	1.8409	2.6429	5.1635	

5.0831	5.0774						
API5	5.4584	5.8117	5.8928	5.5423	5.5713	5.6973	5.4742
5.3898	5.77	5.5438	5.8493	4.8296	5.2731	5.677	5.9633
5.4814	5.5931	5.3272	6.0267	5.2067	6.0267	5.4569	5.1479
5.4694	5.7205	5.4363	4.209	5.662	5.3663	5.3947	5.2287
5.2966	5.0866	4.801	5.2611	5.8608	5.6127	5.2461	5.2604
5.4291	4.8231	4.828	5.9774	5.6265	5.5376	5.3969	5.8737
5.7027	5.5992	5.9774	5.8512	5.4283	5.6873	5.6754	6.152
5.5666	5.6172	5.7018	5.7671	5.6091	4.8806	5.6927	5.4131
5.9379	5.4534	4.9683	4.9939	5.6249	5.3768	5.1701	5.4566
5.4432	5.7872	5.5866	5.4872	5.9494	5.7035	4.7342	5.802
5.7181	5.2868	4.8591	5.0295	5.191	4.8149	5.4996	5.3932
5.6982	5.4229						
APIP	4.2667	4.9268	4.3323	5.0486	4.7621	4.7264	4.7284
4.3414	5.263	4.3027	5.0599	4.4988	4.2068	4.7561	4.2362
3.8422	4.7833	4.1849	5.0106	4.6735	4.3027	4.8892	4.7973
5.0397	5.2181	3.7728	5.4833	4.1975	5.1415	4.2684	3.7358
4.1983	4.6109	3.6328	4.1165	4.4723	4.555	3.8	4.2294
5.2222	4.2515	4.0426	4.736	4.5795	4.4533	4.9204	4.7711
5.3037	5.1214	5.4131	5.6431	4.7827	4.681	4.6994	4.6994
5.0228	4.3547	4.2918	5.4062	4.3126	4.2018	4.663	4.4643
5.1546	3.9784	3.9931	3.4541	3.8105	4.5979	4.5896	4.7909
4.7135	4.6135	5.0834	3.8794	4.683	4.2549	3.7387	4.9456
5.3724	4.4046	4.5806	4.6952	4.5069	4.3727	5.4315	4.0748
4.2278	4.2515						
APITD1	2.9685	3.9454	3.2732	4.1928	3.7775	2.7766	3.5281
3.2658	3.7508	2.9902	3.1862	3.3831	3.0137	3.9884	3.3639
2.9144	2.6186	3.6666	4.0004	3.8296	3.9221	3.2643	3.8006
4.112	3.4804	2.7349	1.6484	3.783	3.6666	3.7067	3.5492
3.4963	4.5998	3.0023	3.0126	3.7921	3.1831	2.8784	2.7855
3.8414	3.1759	2.5912	3.4411	3.7651	3.5357	4.2949	4.0896
3.612	2.6395	3.9581	3.066	4.2172	3.6285	2.7011	3.6791
3.1464	2.3132	3.2752	3.4738	3.5869	3.278	3.1187	2.6888
4.0911	3.4856	2.775	3.5568	2.6985	3.7699	3.5146	3.3135
3.8756	3.5167	3.1274	3.081	3.3082	4.0196	2.9198	3.5315
3.6799	4.5626	2.7764	3.0277	2.9387	4.079	4.0961	3.0039
3.323	3.1124						
APLF	1.55	0.1462	1.0007	1.2519	0.5317	0.7958	1.711
1.108	1.8257	1.2361	1.3462	1.182	2.2074	1.7349	1.1808
1.3638	1.9279	1.8958	1.2701	1.3771	1.4339	0.9766	1.3025
1.2167	0.8577	1.2736	0.9803	1.4689	0.927	1.0771	0.7215
1.3504	1.1302	1.3717	1.3638	1.1726	1.2318	1.0101	0.8946
1.4669	1.2979	1.1502	1.8953	0.9759	0.7569	1.4638	1.3764
1.0038	1.6074	1.0227	1.4418	1.4352	1.8306	1.7712	1.2649
1.2936	1.5695	1.2426	1.1413	1.3061	0.4145	0.9035	0.6923
1.3005	1.3131	1.3156	1.7603	1.0578	1.4555	1.3702	1.0205
1.5478	1.2112	0.7725	1.409	1.27	1.4934	1.5658	1.3494
1.0542	1.8123	0.2287	0.848	1.4803	0.8838	1.9315	1.3418
1.241	1.1113						
APLN	0	0.0728	0	0.354	0.1052	0.584	0.1989

0.5544	0.1162	0.051	0.0427	0.1483	0.8595	0.1101	0.1029	
0.2202	0.1269	0.028	0	0	0.4036	0.9532	1.0148	
0.9661	0.4445	1.2937	0.0856	0.1064	1.5446	0.3424	0.2802	
0.4285	0	0.1519	0.1122	0.081	0.1142	0.3779	0.1714	
0.1739	0	0.0271	0.4927	0.4352	1.5459	0.8907	0.1135	
0.0464	2.7772	0.9681	0.0681	0.5004	0.1487	0.1043	0.2723	
0.4253	2.3128	0.1683	0.0711	0	0.2361	0.0205	0.6412	
0.0367	0.8999	0.1841	0.2858	0.1752	0.1095	1.7898	0.1812	
0.1335	0.1876	0.542	0.5849	0.0457	0.0295	0.2126	0.2568	
0.1352	0.0593	0.8315	0.0796	0.1339	2.0738	0.2902	0.159	
0.4784	0.3614							
APLNR	0.0581	0.0495	0	0	0.0114	0.0274	0.049	0
	0.0245	0	0.0066	0	0.0186	0.0798	1.5338	0
	0	0	0	0.1219	0	0.1179	0.0055	0
	0	0	0.0187	0	0.0771	0	0	
0.0038	0.0621	0	0.0076	0.0244	0.0233	0	0.0443	
0.0411	0	0.009	0	0.033	0	0.0158	0	0
	0.0238	0	0.0141	0.018	0.0374	0.0146	0.0481	
0.2454	0.175	0.0405	0.1143	0	0	0.0693	0.0514	
0.0203	0.1236	0	0.1592	0	0.0345	0.0917	0.1213	
0.0232	0	0	0.0232	0.0368	0.1678	0	0	
0.0798	0.1904	0	0.0892	0	0	0	0	0
	0							
APLP1	2.5422	0.7513	3.1732	1.4407	2.4626	4.1945	2.2064	
3.1942	2.0149	2.9597	3.0345	2.0053	5.5182	2.0648	3.4147	
4.4053	1.8748	1.8577	1.6726	5.1385	0.7876	2.2519	5.3531	
2.0786	3.9266	4.6602	1.4612	1.0725	3.7416	2.9421	7.4138	
1.8317	2.7096	2.0403	3.688	3.5017	2.2184	1.475	2.6635	
2.7763	0.3533	4.2667	4.6975	0.4507	1.4775	1.5226	1.1997	
2.3384	4.3305	1.4838	1.7649	2.4198	0.867	0.7662	5.1955	
3.6001	0.4414	4.0205	1.5976	2.7154	3.7298	1.9482	1.7284	
2.3692	1.0785	6.6547	3.2857	2.1866	1.2782	4.0905	4.5269	
3.0803	5.2	4.1323	3.3972	1.8122	0.3357	3.0495	1.0679	
3.1937	1.0647	2.3237	2.3892	2.0036	4.025	1.1406	2.0425	
2.1218	1.2087							
APLP2	6.6655	5.2919	6.1603	6.2175	5.8976	7.9248	6.4643	
6.014	6.3459	6.4643	6.2603	6.4012	5.812	6.3074	6.8916	
6.3277	8.5435	5.6567	5.4286	6.5658	5.8718	6.7512	6.8352	
7.4668	7.497	6.3516	8.302	5.3225	5.8747	5.1726	7.5564	
4.9316	5.1726	6.6617	6.0194	6.4595	5.321	7.2723	6.2908	
6.6386	6.472	6.2313	7.684	7.7469	6.4504	6.08	5.6829	
4.936	8.0563	5.6166	7.0691	8.5231	5.893	6.5186	7.1127	
6.9839	8.8263	6.4505	5.9078	6.6614	6.8104	4.8727	7.0439	
6.424	6.124	9.0511	6.0082	7.6021	5.3134	7.1284	6.3523	
6.0721	6.6706	5.9224	8.0569	6.4199	5.8073	6.3538	5.963	
6.5771	5.589	7.6642	5.5947	6.7436	7.2146	5.7012	7.1374	
6.6369	7.2317							
APMAP	5.741	5.8108	5.1525	6.3663	6.5425	6.7409	6.0342	
6.5302	6.7667	5.586	6.6048	6.8053	6.3295	5.5721	6.7624	
6.4472	5.7591	6.261	6.1288	6.1811	6.0367	7.2551	7.8724	

6.3976	6.8483	6.9597	5.7929	5.2988	6.7026	5.6895	5.5128	
6.465	7.4665	7.2372	6.6241	5.7619	5.5728	6.3549	6.0286	
6.8397	6.4133	6.3295	6.7156	7.4496	7.0391	6.1669	6.1173	
5.3609	5.9625	5.3723	6.7558	7.4633	6.4502	6.3303	5.9726	
5.8435	5.8931	6.389	6.4631	6.6066	6.1947	6.5119	5.8548	
6.6012	4.9101	6.4455	5.0272	6.0957	5.7934	6.3806	7.1951	
5.9185	6.9327	7.0569	8.0628	4.8595	6.2131	7.4107	5.956	
6.097	5.7786	7.2152	5.9825	7.0714	6.4569	6.2143	5.4769	
5.3777	5.2965							
APOA1	0	0.0998	0.3394	0.1677	0	0	0	
0.139	0.0751	0	0	0	0	0	0.0662	0
	0	0.1436	0	0	0.1198	0	0	0
	0.0506	0.0688	0	0	0.0765	0	0.1413	0
	0	0.0913	0.0404	0	0	0	0.1198	0
	0.0657	0.0678	0	0.0954	0	0	0	0
	0	0	0	0	0	0.0992	0.0792	0
	0	0.0733	0.1704	0.0726	0	0	0.059	0
	0.2972	0	0	0	0.0464	0	0.061	
0.0644	0	0.0142	0.0524	0.0632	0	0	0	
0.1316	0	0.0421	0	0	0	0	0.1006	0
	0							
APOA2	0	0	0	0	0.0605	0.44	0	
0.1058	0	0	0.1599	0	0.0888	0.223	0	
0.6764	0	0.3557	0.2804	0.2463	0.1954	0	0	
0.9632	0	0	0	0	0	0	0.2809	0
	0	0.1407	0	0.3444	0	0	0	
0.137	0.2447	0.0994	0	0.2723	0	0	0	
0.1445	0	0	0	0	0	0.1049	0.5369	0
	0	0	0.3765	0	0	0	0.1545	0
	0	0	0	0.0819	0	0	0	0
	0.1664	0.1202	0	0	0.5347	0	0.464	0
	0.3005	0	0.464	0	0	0	0	0
	0							
APOA4	0	0	0	0	0	0	0.0555	0
	0	0	0	0	0	0	0	0
	0	0	0	0	0	0.0323	0	0
	0	0	0	0	0	0	0	0
	0	0	0	0	0	0	0	0
	0	0	0	0	0	0	0	0
	0	0	0	0	0	0	0	0
	0	0	0	0	0	0	0	0
	0	0	0	0	0	0	0	0
	0	0	0	0	0	0	0	0
	0	0	0	0	0	0	0	0
	0							
APOA5	0	0	0	0	0.0102	0	0	0
	0	0	0.0152	0	0	0	0	0
	0	0	0	0	0	0	0	0
	0	0.0222	0	0	0	0	0.0566	
0.0212	0	0	0	0	0.0134	0.0471	0	0

	0.0309	0	0	0	0	0	0	0
	0	0.1328	0	0	0	0	0	0
	0	0	0.0411	0	0	0	0	0
	0	0.0429	0.1034	0	0	0	0	0
	0	0	0	0	0	0	0	0
	0.0254	0	0	0	0	0	0	0
	0							
APOB	0	0.0088	0	0	0	0	0	0
	0	0	0	0	0.003	0	0	0
	0	0	0	0	0	0	0	0
	0	0	0	0	0	0	0	
3.00E-04	0	0.0064	0	0	0.0014	0	0	0
	0.0054	7.00E-04	0	0	0	0.0079	0	0
	0	0	0	0	0	0.0045	0.0192	0
	0	0	0	0	0	0	0	0
	0	0.0011	0	0	0	0	0	0
	0	0	0	0	0	0	0	0
	0	0	0.0076	0	0	0	0.0167	
0.0392	0							
APOBEC1	0	0	0	0	0	0	0	0
	0	0	0	0	0	0	0	0
	0	0	0	0	0	0	0	0
	0	0	0	0	0	0	0	0
	0	0	0	0	0	0	0	0
	0	0	0	0	0	0	0	0
	0	0	0	0	0	0	0.0529	0
	0	0	0	0	0	0	0	0
	0	0	0	0	0	0	0	0
	0	0	0	0	0	0	0	0
	0	0	0	0	0	0	0	0
	0							
APOBEC2	0.1423	0.1678	0.0813	0.079	0.0359	0.4626	0.148	
0.085	0.2203	0.5074	0.077	0.0593	0.0153	0.1349	0.051	
0.0869	0.0507	0.1146	0.0652	0.145	0.0858	0.0763	0.062	
0.1047	0.0292	0	0.054	0.2392	0.0849	0.0507	0	
0.0991	0.0584	0.054	0.0657	0.0848	0.1008	0.0961	0.0178	
0.2573	0.0756	0.051	0.102	0.5083	0.1465	0.1697	0	
0.0566	0.0689	0.1407	0.1583	0.1397	0.3938	0.279	0	
0.1087	0.1809	0.1019	0.1485	0	0.0669	0.0353	0.0423	
0.1142	0.2499	0	0.2331	0.0434	0.2419	0.0918	0.1479	0
	0.0489	0.0248	0.0984	0.1486	0	0	0.2164	0
	0.1315	0.0602	0	0	0.152	0.3101	0.0667	
0.0411	0.0478							
APOBEC3A	0.0257	0.0506	0	0.0219	0	0	0.0314	0
	0.0691	0.0398	0.0228	0	0.086	0.1577	0	
0.03	0.0791	0.066	0	0.3097	0.0631	0	0.0797	
0.0661	0	0.0643	0.1758	0.0783	0.0722	0	0	
0.0886	0	0.0446	0.0387	0.0342	0	0.0962	0.1163	0
	0	0.0344	0	0.0938	0.029	0	0	0
	0.0703	0	0.0406	0.0872	0.0866	0.0504	0.0865	0

	0.0994	0.1841	0.2204	0	0.0457	0	0.0697	0
	0.0959	0	0	0.008	0.0213	0	0.096	
0.078	0	0.0483	0.058	0.0784	0	0.0336	0.0485	0
	0.0735	0.0179	0.0249	0.0363	0.1358	0.0815	0	
0.0602	0							
APOBEC3B	4.2609	2.2594	5.0349	3.8187	3.857	4.1176	3.2639	
4.5671	5.2488	4.2431	1.9388	4.9254	5.4072	4.5071	5.4506	
3.4251	2.9698	2.8941	3.8646	5.3048	4.4909	3.8336	3.596	
4.7702	4.5814	2.5358	0.0345	2.9209	4.9309	2.932	0.0761	
4.2664	4.9596	4.774	3.2522	4.2035	3.3621	1.8411	2.1708	
4.007	3.2583	3.662	4.2501	5.4575	3.7334	5.1401	4.1244	
4.6206	3.3936	4.0039	2.6723	3.8125	3.9588	2.0769	1.2283	
2.8067	3.7762	5.7067	2.352	5.551	4.6522	3.8933	3.9914	
4.8992	3.9559	1.8058	4.8403	2.0428	3.4705	4.9634	4.441	
4.4984	4.7011	0.5387	3.9176	3.343	4.3999	3.2513	3.5878	
4.7006	5.3681	3.3079	2.1788	2.8385	5.6181	4.8142	2.5634	
4.3526	3.9255							
APOBEC3C	5.3793	2.5008	5.4502	5.8536	5.6323	5.5087	4.7855	
4.5201	6.3488	5.5642	5.3001	5.7441	4.9462	5.2588	5.6767	
6.3005	5.0436	4.4034	5.4816	5.5316	5.3325	4.8201	5.6821	
5.7486	5.1201	5.224	4.8167	0.2329	5.9734	4.4368	3.0759	
3.9966	5.3927	5.3918	5.9666	4.7654	4.8471	4.9998	2.7166	
5.7717	5.5087	7.0164	5.5273	6.3351	4.956	5.2303	4.8527	
5.3237	5.2492	4.5029	4.3672	4.7543	4.9575	2.4394	3.2877	
4.0734	5.6595	2.3893	4.4257	2.4525	5.6703	5.652	5.1853	
5.1967	4.9668	3.131	1.6317	5.9528	5.1196	5.3	6.5405	
4.9443	5.2663	5.3895	5.6167	5.9358	4.7342	6.0008	5.8524	
5.7808	5.5819	7.0122	5.0702	4.9443	5.1922	5.3797	6.0387	
6.2495	5.8014							
APOBEC3D	1.8192	0.3264	1.3068	1.8377	1.707	1.1583	1.6872	
0.0825	1.1677	1.1299	0.3359	1.8154	0.0557	0.579	0.2923	
1.8405	1.0502	0.6502	0.2661	1.1701	0.7589	0.7896	0.535	
1.3588	0.4411	0.7542	0.8253	0.1118	0.3014	0.2021	1.4062	
0.8133	0.6179	2.2038	1.6039	0.919	1.7813	1.1225	0.5366	
1.5756	1.6274	3.5807	1.2813	2.1385	0.1905	0.2378	0.7129	
0.4121	0.96	1.8701	0.8662	0.7751	1.0874	0.4946	1.9822	
0.2477	1.2233	1.1205	0.6829	0.8523	0.9726	0.607	0.425	
0.2143	0.3378	0.2138	2.5318	1.289	0.3565	1.0888	1.0434	
0.3639	0.8037	1.3595	1.3556	1.0272	0.952	1.2624	0.3994	
2.7915	0.3793	2.5447	0.3332	1.0569	0.509	1.4436	0.9657	
0.8877	0.9783							
APOBEC3F	1.4994	0.7326	1.4386	2.6581	1.8098	1.1469	1.9878	
0.9075	1.9419	0.8312	1.9152	1.8891	2.1194	0.4055	1.6481	
1.4892	0.1601	1.1289	1.5829	1.5383	1.2589	1.3421	1.5958	
1.6892	1.4545	1.8885	1.1072	1.6167	1.6009	1.6764	1.565	
1.3838	1.3632	2.4313	2.0562	0.8728	1.6314	1.5878	0.3789	
1.484	1.8044	3.7131	0.9598	1.4067	0.8514	2.2902	0.3073	
1.6709	1.3582	1.7863	1.3171	0.9929	1.2194	0.3539	1.5823	
0.4678	1.9982	1.6268	1.1981	1.5067	0.5932	1.2355	0.8855	
1.3063	1.1308	2.4378	0.9429	1.8963	1.4332	1.4718	2.3443	

1.2216	1.2244	1.4981	0.7293	1.105	1.339	1.4103	1.8443	
1.774	2.0736	2.9539	1.1467	1.3588	1.2216	1.2868	0.956	
0.9764	0.9119							
APOBEC3G	1.5243	0.0744	2.0798	4.5306	4.5666	2.1891	4.054	
0.0991	3.8523	2.0878	0.6373	4.9583	0.3813	0.0813	2.546	
3.92	0.2564	2.1832	1.2513	0.0485	0.9205	1.8548	4.152	
0.4644	2.9845	3.0052	3.0529	0.024	3.442	0.2118	1.6896	
2.282	3.4967	4.2092	3.3694	2.2404	1.4299	2.1151	0.192	
1.2139	2.8135	6.3114	0.1348	1.6623	1.8424	4.2826	0.015	
3.5492	2.7228	2.1352	1.4324	2.5979	1.838	0.1256	0.9224	0
	2.7817	0.6155	2.5414	1.1888	3.309	2.8916	0.104	
0.2205	0.3548	0.1298	0.2447	3.255	1.8917	2.8492	4.0198	
0.2027	2.5227	4.173	2.8406	2.7334	1.2336	1.3551	3.3123	
2.4317	2.185	5.3561	2.6734	2.4105	1.2315	2.1982	1.5844	
1.4876	0.7657							
APOBEC3H	1.5519	0.1325	0.3889	0.8327	1.0462	0.2689	0.3341	
0.4052	1.4953	1.4631	1.7144	0.1893	0.1735	0	0.3428	
0.4513	0.2208	1.1502	0.7331	0.3067	0	0.0935	0.4835	
0.188	0.0593	0.0359	0.1706	0.3382	0.9837	1.2389	0.1053	
0.1777	0.3792	0.1708	0.3664	0.1683	0.1973	0.4123	0.0731	
0.8298	0.5953	0.5394	0.232	0.8959	0.109	2.0022	0.2222	
1.1653	0.2356	0	0.194	0.6034	0	0.0786	0	0
	0.7382	0.0729	0.2692	0	0.887	1.4522	0.223	0
	0.4683	0.5752	1.6011	0.6096	0.588	0.5122	0.3254	
0.1813	0.6331	0.0845	0.254	0.2545	0.1888	0.6754	1.6017	
1.7697	0.73	1.1244	0.1415	0.1925	0	0.1961	0.9035	
1.0248	0.2057							
APOBEC4	0	0	0	0.0246	0.0173	0.0227	0	
0.0074	0.0192	0.1432	0	0	0.0059	0	0.004	0
	0	0.0544	0.0574	0.0258	0	0	0.0262	0
	0	0	0.2605	0	0	0	0	
1.00E-04	0	0.0106	0.0044	0	0	0	0	
0.0194	0.0104	0	0.0215	0.0303	0	0	0.0348	0
	0.0271	0	0.0106	0.0132	0	0.0377	0	0
	0	0	0	0	0	0	0	0
	0	0.0066	0	0	0.1108	0	0	0
	0.0352	0.0057	0	0	0	0	0.0139	0
	0	0	0.0247	0	0	0	0	0
	0							
APOBR	0.0306	0.0597	0.0611	0.2037	0.0076	0.0456	0	
0.0474	0.0302	0.0234	0.0048	0.0042	0.0342	0.0656	0.0059	
0.0319	0.0471	0.043	0.0805	0.1499	0.0516	0.0151	0.0133	
0.0217	0.005	0.0382	0	0.0987	0	0	0.0917	
0.0279	0.0671	0.0368	0.0022	0.0098	0.0094	0.0327	0.0076	0
	0	0.0236	0	0.0148	0.0271	0.0281	0.0267	
0.03	0.0173	0	0.0571	0	0.0358	0.0633	0.0765	
0.0485	0.0803	0.0167	0.0585	0.0151	0	0.0047	0.007	
0.0951	0.043	0	0	0.0059	0.0165	0	0.0614	0
	0.0145	0	0.0244	0.0123	0.1446	0.0495	0.0143	0
	0.0249	0.064	0	0.0313	0.0746	0.0502	0.0603	

0.0621	0.0559							
APOC1	8.8627	5.8625	1.248	3.8943	2.6993	1.9008	1.3457	
5.0026	1.7865	7.883	1.9736	4.6589	0.93	3.8753	1.2829	
0.8775	2.545	7.2776	3.929	0.4505	0.807	5.0978	2.9635	
2.2637	2.3079	0.9667	3.2057	2.0173	1.7988	5.3607	6.3219	
2.7131	2.3868	10.3123	0	7.9239	1.6331	6.4696	9.7214	
2.4357	4.5697	3.6101	1.4039	2.4186	1.4746	1.9053	5.0783	
7.0428	2.5847	2.609	5.5858	8.4017	7.5511	6.8566	4.6264	
0.5051	0	2.3963	5.6757	2.398	2.7051	1.5821	4.0304	
0.3295	4.5507	0.2293	0.4239	4.6873	5.9044	0.8613	2.9024	
5.4472	2.0218	7.769	4.7144	10.2557	4.3374	5.0605	7.9417	
4.4228	7.7927	4.4589	4.0393	1.9682	1.69	6.6612	3.3245	
5.2327	4.0656							
APOC2	4.0368	0.8734	0.1938	0	0	0.0143	0	0
	0	2.8094	0.021	0.2925	0.0349	0	0.1731	0
	0.5244	0.9253	0.0291	0	0.1705	0.2178	0.0465	
0.0023	0.0285	0.0484	0.0068	0.5641	0.0443	0.6968	0	
0.0155	0.006	2.1801	0.0389	3.1586	0.2917	0.0112	2.554	
0.0036	0.1349	0	0	0.0063	0	0	0.2676	
0.2202	0.0806	0.0315	0.6114	0.0063	0.5531	0.0872	0.0822	
0.0429	0.0779	0.2975	3.3269	0.1337	0.0442	0.0132	0.0649	
0.0251	0.0883	0	0	0.011	3.3422	0	0.1098	
0.8676	0	0.0797	0.1033	0.4114	0.1476	0	2.2845	
0.1208	0.1317	0.0866	0	0.13	0	1.3441	0	
1.1557	0.0203							
APOC3	0	0	0	0	0	0	0	0
	0	0	0	0	0	0	0	0
	0	0	0	0	0	0	0.071	0
	0	0	0	0	0	0	0	0
	0	0	0	0	0	0	0	0
	0	0	0	0	0	0	0	0
	0	0	0	0	0	0	0	0
	0	0	0	0	0	0	0	0
	0	0	0	0	0	0	0	0
	0	0	0	0	0	0	0	0
	0	0.0651	0	0	0	0	0	0
	0							
APOD	3.1275	3.6772	1.1109	4.2985	0.6361	3.118	3.3691	
1.8278	6.3223	5.6998	6.5856	7.707	1.0599	6.349	5.3094	
4.5205	0	10.7161	5.6449	1.2451	1.3775	6.3462	6.6132	
4.6447	6.3306	0.5708	5.4655	2.7302	5.2337	5.4537	0.1339	
9.2847	5.9837	4.0739	3.9793	6.2803	5.1235	2.091	5.518	
6.0826	3.9458	0.1566	7.3958	5.7123	1.829	1.1135	1.8669	
6.5642	4.9115	1.283	3.1064	7.2554	8.2235	8.5005	0.2857	
6.1983	1.2756	4.3635	5.6312	2.404	4.2504	3.5383	6.8573	
3.6512	0.9631	0.5964	1.0212	0.2281	5.7282	5.3062	4.9126	
4.4637	2.6821	8.293	1.0336	5.1823	1.8514	4.4026	7.0869	
7.4044	9.3688	5.4957	4.0962	7.6365	4.2127	7.0841	7.0204	
4.3258	4.4016							
APOE	8.3094	7.7254	8.0869	4.1426	1.7581	4.7859	0.7378	

5.2967	7.1694	7.9436	7.2236	6.9418	1.4709	3.9392	5.4294	
7.1526	1.6398	8.6565	4.7218	2.2449	4.2533	5.7982	5.7062	
1.7132	4.6427	1.4071	5.9174	8.764	3.749	7.9324	9.9302	
5.3313	5.8921	10.6922	1.2085	6.9685	3.5802	9.7627	11.0935	
0.8555	7.915	5.0059	0.7878	4.3101	1.8758	0.963	6.0231	
8.0493	7.8335	1.955	7.89	7.9182	7.3785	8.7371	5.8669	
6.9618	0.167	0	8.7196	0.7415	5.1465	5.7458	5.6193	
3.0542	7.5483	1.9954	6.8969	7.228	9.7323	0	4.8674	
8.7371	6.7083	8.0337	6.7083	9.8121	8.3264	5.5983	8.5766	
6.7699	8.4102	7.3762	5.6658	6.6873	3.2343	8.6591	3.8891	
7.3785	6.4851							
APOF	0	0	0	0.0493	0	0.0874	0	0
	0.0216	0.0255	0	0	0.0182	0	0.0148	0
	0	0	0	0.054	0	0	0.0407	0
	0.0141	0.0423	0.0308	0	0.0735	0	0.0205	0
	0	0.0278	0	0.0195	0	0	0	0
	0	0.0198	0.043	0.0322	0	0	0.0429	0
	0	0	0	0	0	0.0152	0.0682	0
	0.0386	0.0342	0.0618	0	0.0195	0.0199	0	0
	0	0	0.0517	0.0055	0	0.0575	0	0
	0	0.001	0.0217	0	0	0	0.0138	0
	0.0234	0	0.0199	0	0	0.061	0	0
	0.0537							
APOH	0	0	0	0	0	0	0	0
	0	0	0	0.0168	0	0	0	0
	0	0	0	0	0	0	0	0
	0.0224	0	0	0	0	0	0	0
	0	0	0	0	0	0	0	0
	0	0.0124	0	0	0	0.1099	0	0
	0	0	0	0	0	0	0	
0.0919	0	0	0	0	0	0	0	0
	0	0	0	0	0	0	0.0312	0
	0	0	0	0	0	0	0	0
	0	0	0	0	0	0	0	0
	0							
APOL1	0.3608	0.4202	0.2749	0.6892	1.0782	3.4359	5.6889	
0.5184	3.9827	1.3427	0.2627	4.8849	4.4676	1.2541	2.0135	
3.6845	0.7676	1.7775	1.5246	0.8719	0.1847	3.9471	5.3022	
1.2356	0.3512	4.6815	3.0903	0.295	1.5479	0.1611	0.4859	
2.8747	3.5168	2.2006	4.2172	0.9337	1.1702	1.1405	0.1989	
0.7666	1.8646	5.4124	3.5361	0.3813	2.2562	4.5187	0.3032	
0.9022	2.7685	0.5033	3.7313	3.8736	1.8559	0.3347	0.4295	
0.0991	3.6569	0.1699	1.083	0.1308	3.4361	0.5092	0.3874	
1.9887	0	3.6688	0.0079	0.9354	0.1243	2.5329	3.2184	
1.753	3.0182	4.3872	0.7612	1.2063	1.2466	3.5532	1.4741	
1.2011	0.6682	3.3865	3.5709	2.9811	2.3804	2.0286	0.4052	
0.1404	0.1869							
APOL2	2.6753	3.4593	3.3942	2.6328	2.6944	4.574	5.076	
4.6789	4.104	2.5493	4.9297	3.8417	4.9585	3.4991	4.3155	
4.906	2.6601	2.8155	3.0137	3.7092	3.5467	2.9185	4.0365	

4.8595	1.8913	5.1986	2.393	3.9363	3.0716	3.7871	3.4328	
2.6177	4.2575	5.0804	4.2009	3.3732	3.9346	3.6416	3.1795	
3.8555	3.1115	4.814	3.4287	2.5821	4.1294	3.8385	3.0464	
3.8946	3.2582	1.9943	3.8136	3.4732	3.831	2.1483	3.3213	
1.7175	3.3022	2.6654	4.1362	2.458	3.7424	3.4833	3.4024	
4.5964	2.1627	5.8313	2.7058	4.1418	3.6975	2.7103	4.2415	
3.575	3.7974	2.9791	3.8175	4.537	3.7219	3.8897	3.4871	
3.2359	4.3132	3.7837	4.2851	3.7244	4.0516	4.1021	3.8284	
3.1657	3.5129							
APOL3	0	0.0327	0	0.6491	0.7235	0.7124	2.41	
0.1253	0.7255	0	0.0084	2.7513	3.8002	0.0902	1.086	
0.0699	0.2001	0.1514	0.1165	0	0	1.195	1.9762	
0.3266	0.097	2.8558	1.3556	0.0289	0.0493	0	0.0294	
0.5916	0.4986	0.8721	1.9335	0.0032	0.411	0.2621	0	
0.1343	0.5003	3.5942	1.5469	0.0104	0.6128	2.0411	0	
0.1547	0.8652	0.386	1.2225	1.432	0.3412	0.1111	0.1994	0
	2.5117	0	0.141	0.1285	0.5353	0.1029	0.0945	
0.3058	0.0632	3.0116	0.0805	0.0283	0.0854	1.7099	1.183	
0.3273	0.3559	0.6659	0.3259	0.0568	0.1767	0.5266	0.2053	
0.4309	0.2065	0.678	0.0978	1.4434	1.6393	0.5865	0.2413	
0.0827	0.1067							
APOL4	0.1078	0.0178	0.0485	0.0731	0.0539	0.1146	0.0406	
0.0309	0.0205	0.2348	0.0206	0.0312	0.0178	0.1196	0.1001	
0.0642	0.1196	0.0702	0.1085	0.2183	0.0836	0.0341	0.0458	
0.0965	0.022	0.0557	0.0617	0.0328	0.0244	0	0.0298	0
	0.0177	0.0261	0.0124	0.1353	0.013	0.2884	0.0287	
0.1943	0.0467	0.0196	0.0255	0.0903	0.0091	0.0372	0.0386	
0.0701	0.0443	0.0989	0.0538	0.0569	0.0351	0.0096	0.0827	
0.0432	0.0137	0.0117	0.0714	0.032	0.1127	0	0.0226	
0.0423	0	0.0406	0.0614	0.0969	0.0124	0.0392	0.0181	
0.0732	0.0284	0.0334	0.1244	0.0659	0.0752	0.0467	0.1587	
0.132	0.1807	0.117	0.0698	0.0089	0.0604	0.155	0.0368	0
	0.0783							
APOL5	0	0	0	0	0	0	0	0
	0	0	0	0	0	0	0	0
	0	0	0	0	0	0	0	0
	0	0	0	0	0	0	0	0
	0	0	0	0	0	0	0	
0.0868	0	0.006	0	0	0.0168	0	0	0
	0	0	0	0	0	0	0	0
	0	0	0	0	0	0	0	0
	0	0	0	0.0181	0	0	0	0
	0	0	0	0	0.0317	0	0	0
	0	0	0	0	0	0	0	0
	0.0487							
APOL6	2.064	0.7633	2.0201	2.4693	2.0118	3.5513	4.466	
0.4193	2.511	2.7534	1.1071	2.9082	4.9039	2.242	3.6155	
3.0063	1.9152	1.7831	1.5391	1.7535	0.6758	2.7326	2.9935	
2.001	1.0886	3.8603	0.5093	1.8793	1.6287	0.0544	1.8814	
1.3963	2.4031	2.9961	3.7387	1.5586	3.3368	2.4902	1.7007	

1.2728	2.3609	3.3871	3.1714	2.2181	3.2211	3.3955	1.2814	
2.9105	2.4332	1.8444	3.2912	2.3806	2.1621	2.335	1.23	1.4
	4.1825	0.7973	1.6408	1.2784	2.0196	1.4945	1.7291	
2.9996	0.0243	4.9596	0.1305	2.7207	1.5473	1.6322	3.2766	
2.6417	2.4913	1.3636	2.9931	2.2106	2.675	1.848	2.1896	
1.9683	2.2247	2.9125	2.9563	3.1703	2.2236	2.6009	2.424	
1.9709	1.4896							
APOLD1	4.3472	5.4511	6.991	0.7044	0.5381	1.3749	5.7493	
4.5772	4.2264	3.999	4.8049	0.0487	2.4737	6.1108	0.9934	
3.1595	1.2637	5.0572	5.6277	5.2134	3.9833	1.6258	2.447	
0.5189	0.246	0.9529	0.3661	4.6055	4.3676	6.6022	1.895	
2.7426	5.3345	4.0539	3.7248	5.1961	4.1833	1.4928	2.8778	
5.1991	2.5632	0.6242	1.1655	0.1431	1.2544	2.6444	6.9459	
3.7876	1.4671	2.4198	5.9667	1.7792	4.2863	4.8797	1.0212	
5.7329	1.9458	0.7233	4.667	0.7055	0.9932	6.8434	5.1333	
2.6574	0.7924	1.7302	1.6996	1.1579	5.2746	1.5805	0.9023	
4.6173	7.3375	3.5939	0.6895	4.1944	5.0974	1.6783	5.4684	
4.891	5.252	0.6709	2.4162	0.9389	1.2816	3.8109	7.036	
6.1298	4.7964							
APOM	1.5142	1.6915	1.5857	1.3241	1.357	1.3	1.152	
1.7676	1.366	1.5597	0.8085	1.2893	1.2448	2.1663	1.7559	
1.3704	1.5205	1.6448	2.2891	2.1236	1.3776	1.673	1.2103	
1.5511	1.7398	1.0035	1.9118	1.4159	1.7821	0.9242	0.7231	
1.6252	1.2467	1.3992	1.694	1.2084	1.8893	1.4723	1.6352	
1.0199	1.6246	1.2943	1.045	0.767	1.2009	2.058	1.4596	
1.2474	1.0296	1.8016	1.5926	1.3969	1.2009	1.8725	1.5427	
1.702	1.3227	1.3774	0.9001	1.3363	1.3314	1.6361	1.5066	
1.7438	1.5517	0.7186	1.9181	0.7865	1.0285	1.5059	1.0267	
1.7749	1.0362	1.3543	1.0799	1.2236	2.25	1.3888	1.2809	
1.6045	1.2737	0.9042	1.7102	0.8277	1.6434	1.355	1.1966	
1.1859	1.2311							
APOO	4.7218	5.4709	4.7748	4.8563	4.7942	4.8233	6.256	
4.3646	5.9106	4.9952	4.7965	5.0287	3.7377	5.06	5.0972	
4.8807	3.1755	5.071	5.4429	4.7532	4.0841	4.3181	4.6248	
5.5563	4.082	3.574	4.5526	4.4102	4.0133	6.03	4.2242	
4.3688	4.741	5.2575	4.6925	5.2893	3.5223	4.3363	4.9257	
5.3969	4.8031	3.613	3.9855	4.4374	4.2294	4.6694	4.8413	
4.7069	3.4168	3.9068	4.9417	4.6673	4.6493	3.7538	5.5889	
4.5594	3.1986	4.145	4.8561	4.2357	3.8678	5.4756	5.7185	
5.249	4.1825	3.6208	4.5384	3.5962	4.834	4.2575	4.2742	
3.934	5.1002	4.7542	4.1933	5.5955	4.5554	5.302	4.5993	
5.1612	4.8592	3.5398	4.6485	4.3291	4.0451	4.8536	4.8269	
4.7482	4.6545							
APOOL	2.3007	2.5594	2.0635	2.1277	1.9116	2.5982	2.7667	
2.4464	3.219	2.6529	2.9132	2.8611	2.3314	2.1282	2.2278	
2.902	3.0519	2.554	2.383	2.6727	2.0033	1.886	1.9633	
2.5743	1.7526	2.166	2.9731	2.085	2.0769	2.7746	2.8777	
2.6456	2.191	2.9626	3.1045	2.8933	2.662	2.2287	1.632	
2.4555	1.7705	2.0892	2.1751	2.3612	3.2063	2.4303	2.769	
2.5306	2.1875	3.2644	2.4269	2.6402	2.3001	2.6877	3.2631	

2.6843	2.5273	2.3852	2.3478	2.3046	1.5892	2.1018	2.3995
2.5397	2.5086	2.0159	2.6135	2.4945	2.9218	2.044	2.7561
1.9474	2.6749	1.7819	2.3321	2.5946	2.6961	3.3803	2.4794
2.1033	2.1171	2.0413	2.858	2.6435	1.711	2.2035	2.2966
2.0145	2.0248						
APOPT1	4.5062	3.9067	2.8643	3.9146	3.0143	3.9758	4.4992
4.0688	3.3417	3.6506	4.0323	3.9736	4.4091	3.7909	4.2023
3.8657	4.0957	3.7893	4.4215	2.7334	4.0751	3.71	3.6874
3.3677	3.815	4.1718	4.2911	3.7666	3.7694	3.8123	3.5533
3.9146	3.8657	3.3655	2.9661	3.9377	3.7583	3.5903	3.5493
3.7574	3.9871	3.5493	3.3808	3.2119	3.8653	4.2639	3.7997
3.3761	3.5224	4.3645	3.1255	4.0352	3.3998	3.6899	3.6046
3.823	3.9268	3.843	3.4415	3.9486	3.7775	4.0998	3.8949
3.1092	4.0438	3.7301	4.1249	4.9485	3.1654	3.363	3.9593
3.6147	3.677	3.4354	3.957	2.7129	3.8775	3.4308	4.136
3.8564	3.4576	3.6127	4.2882	3.1985	4.0952	4.1079	4.1136
3.9507	3.7377						
APP	7.5647	6.3478	7.2303	8.0062	7.1998	8.0235	9.1884
7.1998	8.79	7.6627	8.0946	7.494	6.8873	8.6422	8.2731
9.0791	8.2511	7.9605	7.7998	7.7876	7.7178	8.4129	8.2436
8.1846	8.9796	8.6614	8.9433	4.2044	7.9907	7.9662	7.9856
4.4811	8.1897	8.125	8.1846	7.5477	7.4249	7.8161	7.9809
8.5276	8.1846	8.5022	8.5833	8.6145	8.2022	7.8643	8.013
8.2436	7.9077	7.8044	8.3225	8.5553	8.308	7.3894	8.1323
7.7831	8.2348	7.3303	7.4802	7.1874	7.7228	6.5748	6.7063
8.1535	7.9316	9.5856	6.3401	8.5395	8.5499	8.379	7.75
7.1874	7.8395	8.1167	9.0468	8.2035	6.6606	9.1188	8.408
8.3638	7.9633	9.0341	7.1516	9.1489	7.8902	7.8395	7.5078
7.3653	7.5078						
APPBP2	3.4978	3.1267	3.0427	3.5419	2.8081	3.4602	3.3359
3.11	3.561	2.9001	3.3792	3.1147	3.5628	3.2414	3.0603
3.5535	3.3333	3.7514	3.1077	4.412	4.0851	2.9599	3.2157
3.0599	3.3515	4.1508	1.5658	4.5225	3.5225	3.5979	3.6822
3.4629	2.7349	3.3784	3.6369	3.4733	3.873	3.9751	3.085
3.4646	4.3461	3.6952	3.1946	2.5358	4.2543	3.5533	3.8441
3.6676	3.0256	3.9323	3.9506	3.1515	5.2237	4.0244	3.2346
3.1235	3.4043	3.3025	2.8843	2.9886	3.463	2.7914	2.9043
3.1345	4.6627	4.0503	3.737	3.7112	3.5149	3.4525	3.1638
3.3449	3.553	2.7914	3.531	3.0852	2.9079	3.591	3.6082
3.1967	3.4491	3.2842	3.442	4.3597	2.9903	4.9933	3.572
3.3279	3.6069						
APPL1	4.0592	3.5366	4.191	3.9677	3.9276	4.0461	3.5526
3.6406	3.7536	4.7806	3.8112	4.2775	3.563	3.9871	3.9862
4.0505	4.3436	4.2238	4.1159	3.7897	4.1542	3.6706	3.6636
4.4734	3.9326	3.3428	3.7103	3.9453	3.791	3.8779	4.2884
3.9797	3.6244	3.3234	3.6849	4.8101	3.7722	3.4904	3.7477
3.9013	3.4039	4.043	3.8652	3.9092	4.6064	2.6246	4.4441
4.0067	3.3086	4.2441	3.8037	4.049	5.5137	3.6863	4.4004
3.6898	4.1925	3.4505	4.1829	3.6023	3.9681	4.1459	4.1066
4.397	4.4045	3.1531	4.1904	3.6206	4.5745	3.6906	3.7679

3.8609	3.699	3.5974	4.1099	4.2334	3.9493	4.0666	3.5643
3.6523	3.6313	3.8924	3.391	3.3809	3.7613	5.3396	3.6713
3.8766	3.882						
APPL2	3.8773	3.5147	3.5217	3.8749	3.9506	3.7024	3.7032
3.1342	4.0396	3.814	3.4884	3.5848	3.108	3.8717	2.9991
3.4211	4.4265	4.4571	4.4948	3.1433	3.4135	3.9059	3.6675
3.3042	4.4274	3.7343	4.2739	3.8705	3.8532	4.54	3.5901
3.7393	3.405	3.3897	3.7692	3.8729	3.173	4.373	3.4308
4.1294	4.4833	3.6268	4.6791	3.4712	3.5344	4.8272	3.5488
4.2734	3.612	5.2946	3.6675	4.1399	4.4202	3.723	3.3741
3.3232	3.5022	3.0391	3.9168	2.7404	4.1065	4.3399	3.5855
3.3753	4.2324	4.3777	3.8872	3.3745	3.507	3.5605	3.7539
3.6398	4.0881	3.4139	4.3676	2.8495	3.2953	4.0172	5.023
4.1767	3.7507	3.898	3.1108	4.2681	3.5111	4.1827	3.1535
3.0908	3.8121						
APRT	6.37	6.0479	6.2049	4.8175	6.8121	5.6716	4.8792
6.7887	5.5069	5.8003	6.3003	5.2442	5.6476	4.6685	5.6024
5.013	6.6287	4.3284	4.7287	6.6732	6.6185	5.6967	6.1115
6.5117	5.8572	5.6967	5.3302	6.0149	5.722	5.556	6.3238
7.3957	5.7229	5.8914	5.6188	5.2164	5.7508	7.1882	6.2585
5.708	6.738	6.3109	6.0191	4.786	5.4458	5.1142	5.4694
6.4432	5.5458	4.3776	5.3244	5.3152	5.0216	5.2838	6.861
6.0335	6.1119	6.4909	5.7903	6.2265	6.8891	5.7578	7.1196
5.3255	6.5446	4.3578	6.0365	6.2027	5.265	6.5751	6.0002
5.9146	5.3093	4.9418	4.5766	6.3537	5.3224	6.0975	6.4132
6.3421	5.4697	5.815	5.4665	6.2368	5.5015	5.5601	6.1293
6.4834	6.2674						
APTX	4.0292	4.5342	3.7012	4.2793	4.1848	3.1823	3.8487
4.4089	3.8233	4.4021	4.024	3.7966	3.562	4.605	4.5516
4.0648	4.5778	4.214	4.1178	4.1883	2.9802	3.7987	3.6775
3.9486	3.6608	3.4872	3.8776	4.2358	3.937	3.8666	3.7918
4.1739	3.5327	3.3369	3.6991	4.6924	3.3044	3.9863	4.5998
4.2951	4.0083	4.0916	4.425	3.9496	4.2718	4.8272	4.6155
3.3797	3.7253	4.7106	4.0783	4.0304	3.6616	3.7466	4.6401
3.6288	4.3615	3.8795	4.9548	4.0687	3.8494	5.6005	4.0894
3.8999	4.1963	4.2874	3.7701	4.477	3.5936	3.8414	3.9053
3.8969	4.1708	4.5608	3.613	3.5713	3.947	4.0452	4.1768
4.0026	4.0433	4.2426	3.1805	3.7353	3.9493	3.786	4.2527
4.1712	4.0014						
AQP1	0.1399	0.2431	0.1631	0.3948	0.4879	0.3267	0.2173
0.1444	0.1423	0.0424	0.2183	0.2881	0.5861	0.4931	1.9683
0.3543	0.5294	0.2714	0.055	0.4545	0.111	0.8705	0.4746
0.1004	1.2453	1.313	0.8443	0.0982	3.5296	0.1943	0.1478
0.6348	0.1821	0.2859	1.4401	0.0558	0.3082	0.2046	0.8197
0.3205	0.5964	0.4675	0.5326	0.1547	0.5921	1.9432	0.1156
0.2624	0.3185	0.0891	0.0959	0.5789	0.107	0.207	0.1357
0.2058	0.6408	0.0781	0.1871	0.082	1.9613	0.1665	0.0867
0.426	0.4629	0.9265	0.6556	0.1323	0.1698	0.1511	0.2205
1.6644	0.0534	0.206	0.5469	0.0205	0.3257	0.969	0.2335
0.3867	0.018	0.449	0.0855	1.1819	0.7474	0.1123	0.7128

0.1038	0.3138							
AQP10	0	0	0	0	0	0.1217	0	
0.0291	0	0	0	0	0.0211	0	0.0196	
0.0203	0.0716	0	0	0	0	0	0	
0.0571	0	0	0	0	0.0307	0.0387	0	
0.0191	0	0.036	0	0	0	0.0445	0	0
	0	0	0	0	0	0	0	0
	0	0	0	0	0	0.0209	0	0
	0	0	0	0	0	0	0	0
	0	0.0057	0	0	0	0	0	
0.0256	0	0	0	0	0.0145	0	0	0
	0	0	0.1161	0	0	0.0779	0	0
	0							
AQP11	0.819	1.4015	1.2186	0.9987	0.7134	1.5431	1.3342	
0.6055	1.1066	0.7736	0.4086	1.044	0.4031	1.0964	0.6304	
1.6081	1.4055	1.2239	1.1182	0.7152	1.6968	0.435	1.0411	
1.4844	0.4859	0.4257	0.2411	0.7945	0.9865	0.7087	1.2338	
0.8016	0.8582	1.0321	0.8538	0.8335	0.6228	0.837	0.2503	
0.9894	0.7557	1.5367	0.6591	0.7606	0.6366	0.8137	1.0632	
0.7379	1.0604	1.0765	1.0008	0.9479	1.2549	0.5546	1.2627	
1.1114	0.6168	0.5855	0.8145	0.7103	0.4936	0.905	0.6367	
0.8063	0.5625	2.0975	1.0562	1.1897	0.47	0.5869	1.0467	
0.6754	1.0667	0.2848	1.1229	1.2336	0.6462	1.186	0.8733	
1.091	1.417	1.0629	2.0183	0.6893	0.5091	1.0102	0.988	
0.4932	0.9207							
AQP12A	0	0	0	0	0	0	0	0
	0	0	0	0	0	0	0	0
	0	0	0	0	0	0	0	0
	0	0	0	0	0	0	0	0
	0	0	0	0	0	0	0	0
	0	0	0	0	0	0	0	0
	0	0	0	0	0	0	0	0
	0	0	0	0	0	0	0	0
	0	0	0	0	0	0	0	0
	0	0	0	0	0	0	0	0
	0	0	0	0	0	0	0	0
	0							
AQP12B	0	0	0.1372	0	0	0	0	0
	0	0.0236	0	0	0	0	0	0
	0	0	0	0	0	0	0	0
	0	0	0	0	0	0	0	0
	0	0	0	0	0	0	0	0
	0	0	0	0	0	0	0	0
	0	0	0	0	0	0	0	0
	0	0	0	0.1198	0	0	0	0
	0	0	0	0	0	0	0	0
	0	0	0	0	0	0	0	0
	0	0	0	0	0	0	0	0
	0							
AQP2	0	0.2876	0.1196	0	0	0	0.0613	0

	0.062	0	0.0014	9.00E-04	0	0	0	
0.0021	0	0	0	0.0422	0.0694	0	0	0
	0	0	0	0.1134	0	0.0439	0	
3.00E-04	0	0.0083	0	0.0055	7.00E-04	0	0.0044	
0.009	0.0447	0	0	0	0	0	0	0
	0	0	0	0	0.0299	0	0	0
	0	0	0	0	0	0	0	0
	0	0	0	0.0809	0	0	0.0215	0
	0	0	0	0	0	0	0	0
	0.0223	0	0	0	0	0	0.0165	0
	0							
AQP3	0.2429	0.0644	0.4086	0.3396	2.9447	0.1381	0	
0.3825	0	0.0539	0.1607	0.246	0.8953	0.5363	0.6887	
0.0928	0.0922	0	0	1.6306	0.1875	1.5036	0.9112	
0.1323	0.8684	0.349	3.3481	0.0887	1.8842	0.1146	0.9004	
0.1313	0.0933	0.2022	0.7122	0.4547	0.1613	0.106	1.6955	
0.1854	0.0989	1.5838	0.9483	0.3287	0.5744	1.4791	0.2526	
0.153	0.076	0.5505	0.1891	1.6051	0.1519	0.5055	0.3368	
0.1288	0	2.963	0.6335	1.7248	1.4017	0.5753	0.1025	
0.4057	0.1732	0.3387	0.0217	0.1117	0.1382	1.419	0	
0.3736	0	0.5881	1.6985	0.2157	0.0462	0.0609	0.1776	
0.2034	0.0349	1.0924	0.7584	1.7776	0.3815	0.3778	0.1312	
0.2718	0.122							
AQP4	0	0.0375	0	0.021	0.0045	0	0.0233	
0.0056	0.1011	0	0.0967	0	0	0	0.0036	0
	0	0	0.0599	0	0	0	0.0085	0
	0.0105	0.0094	0.1487	0.1718	0	1.3409	0	0
	0	0.0369	0.0071	0	0.0029	0.5722	0	0
	0	0	0	0.0343	0	0.0091	0.1347	
0.0141	0.0231	0.0361	0	0	0	0	0	0
	0	0	0	0	0	0	0.0191	0
	0.0195	0	0	0	0.43	0	0	
0.0066	0	0	0.0186	0.0134	0	0.0508	0.2209	
0.8231	0.2714	0	0	0	0.0326	0	0.0169	0
	0.0354							
AQP5	0.1003	0.6734	0.3431	0.1877	0	0	0.0456	
0.0897	0.1776	0.0494	0.0338	0.0746	0.1	0	0	
0.0733	0	0	0	0	0.1352	0.029	0	0
	0	0	0	0.0908	0.046	0.1047	0.0355	
0.0544	0	0	0	0	0.4711	0	0.1486	0
	0.0759	0.2238	0.0783	0.1983	0	0	0	0
	0	0	0	0	0	0.1568	0	0
	0.0356	0.1221	0	0.0358	0.0112	0.0213	0.2303	
0.0551	0	0.0016	0	0.0868	0.0164	0.0797	0.1466	0
	0	0.047	0	0.1051	0.1852	0.0825	0	0
	0.1242	0.4939	0.0234	0	0.3165	0	0	0
	0.0745							
AQP6	0.4148	1.5171	0.4687	0.0793	0.1808	0.1061	0.1608	
0.0563	0.6172	0.2857	0.1633	0.0892	0.0155	0.052	0.3163	
0.3121	0.0462	0.2309	0.4121	0.4393	0.5354	0.0136	0.0521	

0.1379	0.027	0.0151	0.1829	0.2737	0.12	0.3808	0.1606	
0.2091	0.2515	0.2121	0.1112	0.1011	0.19	0.0799	0.109	
0.2754	0.107	0.1235	0.0378	0.2042	0.1259	0.0682	0.258	
0.0986	0	0	0.0601	0.0497	0.1829	0	0.0454	
0.4249	0.0636	0.2071	0.2233	0.0925	0.1106	0	0.2134	
0.0323	0.0296	0.0042	0.146	0.3268	0.1022	0.4155	0.4227	
0.0472	0.0563	0.0262	0.197	0.6246	0.322	0.0812	0.1862	
0.1663	0.3778	0.0848	0.0327	0.112	0.0349	0.4709	0.1006	
0.0609	0.1605							
AQP7	0.109	0.3629	0	0	0.0962	0.0955	0.1945	
0.046	0	0.1508	0	0.0589	0	0	0	0
	0.2425	0.1481	0.1552	0.213	0	0	0	0
	0.0497	0	0	0	0	0	0	
0.0189	0	0	0.1281	0	0.1029	0.3118	0	0
	0.0349	0.0598	0.0627	0	0	0.0642	0.1676	0
	0.051	0	0	0	0.1531	0.1348	0.0845	0
	0.1591	0	0.088	0	0	0.0506	0.0356	0
	0.217	0	0.1251	0.0079	0	0.2143	0.0954	0
	0.0789	0	0.2264	0.0587	0	0.0614	0	0
	0	0.123	0.0465	0.0632	0	0.1697	0.1012	
0.0736	0							
AQP8	0	0	0	0	0	0	0	0
	0	0.0796	0.0507	0	0	0	0	
0.0571	0	0	0	0	0	0	0.0318	0
	0	0.0661	0	0	0	0	0.0635	0
	0.1804	0.0943	0.0269	0	0	0	0	0
	0	0	0	0	0	0	0	0
	0	0	0.0803	0	0	0	0	0
	0	0.0333	0.0369	0	0	0.0226	0	0
	0	0.0083	0	0	0	0	0	0
	0	0	0	0.0277	0	0	0.4929	0
	0	0	0.021	0	0	0	0	0
	0							
AQP9	0.0389	0.1594	0.0348	0	0	0.0262	0	
0.0049	0.1848	0	0.0472	0.0837	0	0	0	0
	0	0.0233	0	0.0266	0.0664	0	0.0302	0
	0.0124	0	0.0305	0.0367	0	0.175	0	0
	0.0156	0.2285	6.00E-04	0.0595	0	0.5016	0.0841	0
	0.0083	0	0	0	0.001	0.0101	0.096	
0.0558	0.0512	0	0	0.0131	0.0836	0.0591	0	
0.0479	0	0.5925	0	0.0376	0	0.0162	0	0
	0	0.0245	0	0	0.0163	0	0.0172	
0.0203	0	0.0089	0	0	0.0637	0.0381	0.0382	0
	0.0187	0	0.0394	0	0	0.0429	0.0792	0
	0.1053							
AQR	3.3966	4.2488	3.0872	4.1878	3.8674	3.4406	3.3513	
3.3491	3.7385	3.3008	3.5843	3.407	3.5545	3.5027	2.7001	
3.5931	3.7241	4.1202	3.7752	2.866	3.4303	3.6785	3.5726	
3.0656	3.8565	3.0217	2.6008	3.31	3.9041	3.4345	3.6898	
3.216	3.0644	2.7506	3.6277	3.51	3.6731	2.9575	3.5511	

3.806 2.1896 3.7736 3.1447 3.6443 4.497 3.2455 3.9672
3.3183 3.2174 3.9637 3.3603 3.4936 3.3969 4.2468 3.8417
3.6674 3.9602 3.5578 3.5201 3.7643 3.2761 3.3581 3.7255
3.5101 3.3802 2.9611 3.6163 3.1838 3.6288 3.4045 2.9639
3.053 3.5776 3.4437 3.6943 3.2189 2.6572 3.6635 3.5838
3.2461 3.7487 3.5118 3.301 2.6492 3.2294 2.8019 3.2005 3.4
3.278
AR 0.0735 0.1145 0.0594 0.0613 0.5164 0.3943 0.5743
0.5332 2.233 0.0594 1.6499 0.8272 1.5206 0.1389 0.103
0.6228 0.0638 0.0374 0.2754 0.1914 0.0538 0.1799 1.4375
1.3479 1.0764 0.0368 1.5113 0 0.1177 0.1095 0.0561
0.1134 0.8149 2.2277 1.419 0.0821 0.2748 0.8069 0.1111
1.3853 0.2091 2.3058 0.4584 0.0461 2.1927 2.6133 0.3249
1.7317 0.1504 2.1194 0.6927 0 0.0577 0.1018 0.1167
0.0445 0.1839 0.0617 0.1314 0.103 0.3046 0.2313 0.1883
1.4167 0.1336 0.0363 0.3988 0.2079 0.1714 1.3594 1.8442
0.8142 0.987 0 0.4472 0 0.1945 1.5313 1.9769
1.4485 1.9261 1.2197 1.7133 0.3685 0.7832 0.1977 0.9034
0.1985 0.3648
ARAF 4.7127 4.6719 4.1307 3.7662 4.9701 3.81 4.2828
4.2291 4.5765 4.093 3.7154 4.6127 4.4388 3.2988 4.2121
4.0859 3.5716 3.8533 3.2809 4.2874 4.9077 4.6337 4.615
4.7448 3.7056 4.4083 4.771 3.7101 4.6375 4.0336 4.8172
4.566 4.3754 4.8891 4.6902 3.7621 4.3729 4.83 3.9267
3.75 4.0297 4.2526 4.0509 4.0775 4.7007 4.2891 3.5373
4.1125 3.9332 3.9623 4.4162 4.1498 3.5996 3.8779 5.4705
4.1694 4.1846 4.5525 3.4422 4.8115 4.1944 4.9752 4.7764
4.6147 3.7962 4.724 4.0429 4.2189 3.9248 3.9101 4.6585
3.8171 4.3465 4.4656 4.5309 4.1121 4.0343 4.9297 4.1145
3.5971 4.0813 4.4076 4.6493 4.9859 4.313 4.0239 4.4456
4.5312 4.591
ARAP1 4.0699 5.0433 4.8047 2.9382 3.9892 3.3309 3.5487
3.1436 4.0459 4.1087 4.7041 3.0717 2.775 3.2289 2.5656
3.8292 4.4622 2.6818 3.0509 3.9322 5.5456 4.0144 3.6576
3.4206 4.2221 3.1178 2.4695 4.4583 3.5189 4.7636 3.3188
2.8885 3.4658 4.0383 3.8269 3.1591 4.0109 5.1201 4.0821
2.7191 4.7376 4.4023 3.284 3.7234 3.1826 2.9649 3.3854
4.3225 4.3188 2.9794 3.5906 2.6174 3.1115 3.59 3.5133
3.7398 4.7316 4.9885 4.4522 4.0089 2.9094 3.3466 4.0218
2.8381 3.9195 3.5472 4.202 3.8272 3.1721 3.6215 3.7356
2.9785 4.2256 3.3603 3.745 4.4946 5.164 3.4376 3.9789
4.0588 5.8689 3.4138 5.1367 3.6426 2.6778 3.4181 4.1662
4.2825 4.6748
ARAP2 1.3405 1.0487 1.6653 1.8883 1.2629 1.1468 2.4703
0.783 1.1916 1.0748 0.6632 1.261 0 1.1051 0.8197
1.8919 1.1929 1.7801 1.5347 1.9139 2.122 1.327 0.8607
1.4626 0.222 1.1511 1.9747 1.8563 0.3636 1.2296 1.0702
2.0978 1.2807 0.8392 1.8309 1.3688 2.4896 1.0385 0.5668
1.2059 1.4814 1.768 0.0055 1.4718 1.1811 1.1474 1.4044
1.5347 1.1474 0 0.8862 1.9014 1.6144 1.5559 1.3514

1.3188	1.8301	0.5306	1.4249	0.4903	0.6682	1.773	1.0669	
1.4125	0.2583	1.2763	0.2492	0.4673	1.5877	1.5139	0.7979	
1.4757	0.6514	1.039	0.6369	3.2252	1.3281	1.6768	0.9406	
1.4408	1.6417	1.0213	2.3649	1.3721	0.5018	1.4043	0.7978	
0.5175	0.5933							
ARAP3	2.9102	2.7614	2.6338	2.9469	3.7901	3.9105	2.9603	
2.296	2.0246	3.0966	3.123	3.3288	4.2401	2.844	3.1477	
4.6241	2.7793	3.4267	0.6314	3.5299	3.8564	3.819	3.4845	
3.5181	3.7936	3.5617	3.1742	0	3.9315	2.4127	0	
2.9469	2.0037	3.8272	3.8431	2.4911	2.7723	2.8163	3.3795	
0.5995	3.0724	3.497	3.3503	0.0188	2.3727	2.4589	1.9852	
3.8999	3.969	2.9398	3.0162	3.5659	4.2351	2.8281	0.111	
2.4831	4.1064	3.5892	2.7501	3.4897	3.6428	3.3772	0.2484	
3.3016	1.7001	2.3069	2.3717	3.2398	2.9089	4.199	1.9601	
4.5977	2.0579	3.1387	3.1574	3.5169	2.8185	2.73	3.1518	
3.3345	3.1779	3.1027	2.8126	4.4465	3.769	3.8516	3.2245	
3.1897	2.98							
ARC	0.6632	0	0.058	0.1434	0.4348	0.4638	0.1427	
0.4836	0.0844	0.4569	0.5394	0.623	0.1955	0.0995	0.6871	
0.266	2.6656	0.1625	0.291	0.8715	0.186	1.494	0.083	
0.0405	0.5145	0.1907	1.8837	0.0669	0.2291	0	0.0763	
0.1449	0.1308	1.1354	0.0272	0.2026	0.8836	0.6643	2.1462	
0.0475	0.55	0.1951	1.8449	0	0.4451	0.8075	0.6294	
0.2775	0.1053	0.1089	0.2389	0.0472	0.1987	0.477	0.1243	
0.1656	1.4143	0.1973	0.0879	0.062	0.0578	0.081	1.1477	
0.1121	1.7021	0.0634	0.0468	0.5103	0	1.0172	0.0517	
0.4366	0.0059	0.4044	0.1473	0.1098	0.6052	0.2348	0.381	
0.5606	0.3728	0.0312	2.6245	0.1816	0.6493	0.1882	0.1945	0
	0							
ARCN1	5.6646	5.2141	5.61	6.1588	6.4934	6.4862	6.5804	
5.5446	6.324	4.9558	5.726	6.1421	5.9073	6.1588	5.2655	
6.321	6.0454	5.516	5.5142	5.8853	5.0088	6.527	5.9754	
5.6475	5.3215	6.2111	5.4464	4.8277	6.4262	5.5843	6.1131	
4.9291	5.93	6.0718	6.5148	5.5968	5.5039	5.5719	5.6746	
6.4055	5.4882	6.5565	6.2265	6.1463	5.9629	5.5324	5.5816	
6.1224	6.0407	6.3621	6.2666	6.4227	5.0375	6.0305	6.1232	
5.866	6.6679	5.9084	5.7713	5.9034	5.6491	5.3395	5.299	
5.8251	5.0344	6.3502	5.7657	6.5017	5.3175	6.3677	5.3579	
6.0012	6.4601	6.3394	6.285	6.1164	5.201	5.4533	6.1242	
5.2404	5.4582	6.0702	5.5516	6.0326	5.1334	4.5794	5.7785	
5.7106	5.6859							
AREG	0.0382	0.0124	0.2462	1.332	3.8206	0	2.3166	
2.2792	0	0	0.2395	4.8885	2.9229	0.0671	0.576	
0.399	0.4464	0.0239	0	1.9204	0.4238	2.4537	0.6049	0
	1.3616	1.0746	0.153	0.041	0.3611	0	0.0676	
0.0391	0.0543	0	0.2988	0.0967	2.1055	0.0616	0.5435	
0.0311	0	3.0617	0.0612	0.1545	0.8623	3.7394	0.2449	
0.3396	0.751	0.6954	1.0352	2.5895	0.0866	0	0.1057	
0.1549	1.8824	1.1046	0.1801	0.789	1.5826	0.7596	0.2711	
0.4991	0.1077	0.5267	0.1318	0.517	0.0862	2.1934	0.127	

0.3769	0.5354	0.2054	0.1895	0.2028	0.1501	0.3167	0.1909	
0.257	0.4231	0.8094	0.4439	2.9431	0.6189	0	1.8768	0
	0.1244							
AREL1	3.8957	3.6114	3.7769	4.3557	3.7464	4.2782	3.8628	
4.0105	3.6434	4.1012	3.2707	4.9066	3.2531	4.1874	3.7698	
4.217	4.0535	4.537	3.8624	3.7023	3.8844	4.1741	4.7024	
3.9787	3.8889	3.8472	2.995	3.882	4.3661	3.8043	5.0835	
4.3451	3.3169	3.3099	3.1341	4.3134	3.7483	4.0763	3.7343	
4.2964	4.2906	4.1349	3.7323	3.9742	4.8318	4.0422	3.9129	
3.643	3.9204	4.6415	3.5794	5.0737	4.0925	3.5075	4.0001	
3.6803	4.101	3.8068	3.6648	3.6937	4.0728	4.372	4.1386	
3.1258	3.7027	4.4695	4.2774	4.2863	3.4519	3.7159	4.1137	
3.4486	3.9171	4.4763	5.0091	3.9993	3.6937	4.3003	3.9594	
3.4366	3.6154	4.6855	3.8014	4.2703	3.0629	3.8418	4.1976	
4.2832	4.3382							
ARF1	8.2354	7.2184	7.7683	7.5049	8.148	7.4179	7.5352	
8.2474	6.7909	7.1741	7.352	7.4355	7.3148	7.1484	7.9414	
8.2474	7.5049	7.8365	6.869	7.9244	7.827	7.9988	7.8097	
8.0765	8.0352	7.8138	8.1593	7.4091	7.3747	7.8706	7.8365	
7.5781	7.0518	8.1297	8.3067	7.013	9.1183	8.2046	8.7291	
7.2261	7.8783	7.4268	8.3807	7.5781	7.5479	7.2208	6.7981	
8.1136	8.0816	7.0241	8.0408	7.182	7.4781	7.5522	8.2167	
8.5149	8.0388	8.3198	8.1995	8.4598	7.7831	7.4923	7.7658	
7.882	7.9703	7.4408	7.7772	7.9932	6.9141	8.1779	7.1557	
7.9633	7.8585	8.1276	7.9124	7.3479	8.0723	8.4013	7.4666	
6.222	7.2297	7.1267	7.8648	8.7231	8.1109	8.0122	8.0096	
8.2294	8.0861							
ARF3	5.7795	6.2093	5.9926	6.3707	6.3318	6.3187	5.8935	
6.1288	6.3229	6.1587	5.6357	6.229	5.3143	6.2214	6.1639	
6.0932	6.872	6.5302	6.2226	6.0732	5.7175	5.9371	6.104	
5.0551	6.0375	5.7424	6.1754	6.3982	5.7509	6.1959	6.8925	
6.2463	5.7902	5.784	6.0101	6.3361	5.9751	6.8055	5.8519	
6.4264	6.2158	6.3723	5.7795	5.6558	6.5121	6.4149	6.4676	
5.5832	5.7092	6.7125	5.9751	6.1526	6.9785	5.762	6.5215	
5.5241	6.2742	6.2063	6.0935	5.8823	6.2487	5.9755	6.5458	
5.8575	6.0734	6.2683	6.011	6.6866	5.9908	6.3717	6.5964	
5.4395	6.1305	6.2245	5.5465	6.2942	5.8992	6.0602	6.3534	
6.4334	6.2358	6.0294	6.0719	6.2942	5.1546	6.494	6.3618	
6.0721	6.1274							
ARF4	6.4164	7.0707	7.2663	7.7249	7.682	7.8217	8.4821	
8.3977	7.1838	7.5113	8.0345	8.1505	7.6285	7.5511	8.0415	
8.3303	7.3542	7.8994	7.6931	7.1804	7.4385	7.4837	7.6572	
7.8156	7.1441	8.4071	7.1193	7.1441	7.6716	7.1043	7.1412	
8.103	7.3251	7.312	8.5441	7.7249	7.827	6.8397	7.2419	
7.5065	7.0505	8.1814	7.6973	7.3359	8.3788	7.0736	8.0162	
7.8516	7.6716	7.9649	8.072	7.6931	9.367	7.2105	7.3234	
6.9306	7.8737	7.0877	7.2085	7.1093	7.5379	7.3465	7.3311	
7.5693	6.7966	8.2516	7.4836	8.004	8.0204	7.4306	7.2382	
7.1257	7.6794	7.884	7.5892	6.9257	7.2045	7.7761	6.6975	
6.4266	6.4827	7.5779	7.5164	7.5435	8.098	9.1337	7.2285	

7.1441	7.0302						
ARF5	5.9776	6.6128	6.2568	5.7435	6.1179	6.577	5.4356
6.9367	6.2202	6.452	6.6323	5.4496	5.9867	5.5658	6.2043
7.1219	5.6141	6.8858	5.7695	6.4602	5.8218	6.4212	6.5526
5.8218	7.0243	5.722	5.9386	6.3312	6.6037	6.6094	7.4227
6.2923	5.7656	5.5916	6.2937	6.1795	6.3519	6.0869	6.6751
5.2208	6.3769	5.8118	6.8264	6.3814	5.942	5.711	4.494
6.413	7.0366	4.8696	5.7238	5.722	4.8132	5.6226	6.7265
6.316	4.9971	6.5164	6.7084	6.9025	6.1764	6.1548	6.9274
6.5449	6.0722	5.6938	6.2172	6.1637	5.8064	6.9074	6.4857
6.3781	6.5519	5.7913	6.0175	6.5502	6.0187	6.182	5.6407
6.84	6.5115	5.7924	3.6954	6.5231	6.8735	5.5072	6.5426
6.2215	6.3924						
ARF6	5.7732	4.8205	5.3397	4.9517	4.6351	5.4025	4.4738
4.9585	4.4371	5.2911	4.7858	5.3272	5.3756	5.241	5.4396
6.1977	5.3397	6.1154	5.4696	5.5378	5.2194	4.8365	5.3095
4.4699	5.2639	5.855	5.0823	6.281	5.3298	4.8962	4.3453
5.2425	5.4696	5.2113	4.2378	4.9873	4.9654	4.7766	4.9271
4.8639	4.7258	5.5575	5.5204	5.4486	5.7996	5.851	4.8882
4.7167	5.3806	5.9764	4.392	5.65	5.049	5.1145	4.6098
5.4109	5.8848	5.4657	5.078	5.4557	5.4901	5.1867	5.0131
4.994	4.6991	5.062	6.3594	6.4024	5.2586	5.2508	5.1345
5.3165	4.2693	4.981	5.9111	6.0154	5.0495	4.8394	5.2671
4.6052	5.4533	4.7356	5.5174	4.8819	5.4446	5.0126	5.6713
5.6967	5.6303						
ARFGAP1	5.1651	5.6605	5.6524	3.0517	4.8069	4.0732	4.943
5.9857	4.273	4.9964	5.8766	4.0507	4.3672	4.0063	4.5301
5.0435	4.538	4.6153	4.4413	4.625	5.5524	5.353	4.6551
4.2174	4.6977	5.6291	4.2098	4.1978	4.1401	4.7658	5.7423
5.0553	4.6822	5.0501	4.5275	4.2908	5.1817	5.0778	4.8094
3.557	4.2425	4.037	4.5573	4.5549	4.3614	3.1624	3.718
4.3935	5.6524	3.6437	4.7442	3.3478	4.7579	4.9094	5.1058
4.5031	4.4478	2.8857	6.1686	2.8265	5.5299	4.5129	4.2247
4.7055	3.6331	5.0247	4.6262	4.7223	4.9336	4.5286	5.4626
3.9369	5.415	5.1578	4.9799	5.3732	4.9174	4.6087	4.3415
4.5509	5.1136	5.3663	4.9863	5.2853	4.7612	5.123	4.7356
4.9679	4.9634						
ARFGAP2	3.8561	4.8335	4.285	3.8596	4.587	4.4391	4.3911
4.4989	4.3058	3.9204	4.1432	3.7198	4.1131	3.1398	4.5145
4.1841	4.8412	3.4468	4.538	4.4323	4.235	4.8092	4.2499
4.2959	4.9108	4.3434	3.8301	3.9208	4.7434	3.4594	4.5875
4.389	4.2235	3.7441	4.5071	3.7332	5.0415	5.021	4.3968
4.2838	4.2374	4.6354	4.0685	4.4263	4.4219	4.5881	4.1018
4.5043	4.7467	4.3711	4.646	4.1143	3.9192	4.7576	5.2991
4.5034	4.1969	4.3925	4.5771	4.2243	4.107	4.2713	4.5863
4.1698	4.1761	4.3772	3.6031	4.5636	3.564	4.2559	3.985
3.4669	4.5927	4.4328	4.4524	5.2854	4.2509	4.0736	4.3448
4.864	4.017	4.5269	4.2736	4.0799	4.342	4.1918	4.6521
4.515	4.646						
ARFGAP3	4.5476	4.4586	4.2221	4.3435	3.5582	4.7957	4.9882

5.1775	4.8606	4.2182	5.1914	5.6742	4.3685	4.9246	5.114
4.9672	3.252	4.7638	4.7009	4.2508	4.2598	4.3465	4.0565
4.4951	4.5637	4.9539	4.8884	5.035	4.2659	5.5335	4.5407
3.16	4.9955	5.9192	5.0282	4.8871	4.6195	3.8301	3.6355
4.7371	3.629	4.3092	4.7649	5.0444	4.4018	4.7444	5.2348
3.9774	4.5143	3.6695	4.833	4.2841	4.9689	3.939	4.0055
4.2013	4.5387	3.8513	5.5454	3.8665	3.988	4.7817	3.7809
4.4717	4.1235	4.7656	5.1402	4.7505	4.5646	3.6817	4.795
4.9274	3.7891	3.5708	4.8479	5.1158	4.1967	4.1208	4.3406
5.4937	5.3863	4.8	4.147	4.6885	4.6694	4.8095	4.6949
4.1495	4.507						
ARFGEF1	3.4757	3.0176	3.3113	4.0449	3.3718	3.9721	3.7601
3.4647	3.2846	3.9132	4.0699	3.6738	3.6754	3.6759	3.4798
3.0267	4.4829	4.0233	3.9191	3.0187	3.6922	3.416	3.5669
3.2512	3.5497	3.2529	4.0084	3.4508	3.4118	3.4851	3.4167
3.6123	3.6055	4.1757	3.7561	4.2075	3.571	4.4675	3.2459
3.7421	2.1696	3.5423	4.3423	3.4259	3.9227	4.306	4.0524
3.2471	3.104	4.0312	3.371	3.5916	4.0088	3.0353	3.1842
3.0795	4.382	4.3313	3.8981	4.0442	3.6887	3.4842	3.6185
2.8056	4.6503	3.6203	3.6001	4.2501	2.9826	3.4922	3.7108
2.9689	3.6784	3.5997	3.5375	4.032	3.3785	3.5835	4.0174
3.2634	3.955	3.2718	3.5705	3.4063	3.6033	3.5414	3.9347
3.4254	3.5443						
ARFGEF2	4.4076	4.381	4.7865	3.772	3.9357	5.0389	5.5268
4.5277	5.366	5.3104	4.4718	3.9771	4.1973	4.4564	4.0096
4.8415	4.0613	4.7038	4.4802	3.5622	4.5738	4.0202	4.6767
4.4004	4.2083	4.5961	3.7579	3.9002	4.3771	4.0413	4.2714
4.3147	5.5303	4.4854	4.4195	5.0432	4.2219	3.5043	3.8587
4.6088	3.7349	3.6502	3.4988	4.8868	4.7313	3.7905	4.8729
4.8272	4.7086	4.5359	4.6121	4.2506	5.1652	4.2866	4.2911
4.232	3.5297	3.892	5.2612	3.7342	4.282	4.5733	4.3603
4.1389	3.5959	3.7359	4.6389	3.802	4.9864	3.7842	4.8499
4.6598	5.2326	4.5296	4.9318	5.0706	4.1376	4.652	4.2306
4.2885	4.486	4.4668	3.9174	4.3571	3.2053	4.5505	4.316
4.0019	4.1879						
ARFIP1	3.7053	2.996	3.3753	4.4886	3.9377	4.2525	3.7431
4.0736	3.792	3.4007	3.4153	4.25	3.2081	4.1176	3.7511
3.6628	3.4275	4.0046	3.6093	3.8684	3.7599	4.1486	3.5895
4.1453	3.7232	4.2686	4.1861	3.8274	3.7634	2.8159	4.0299
4.2211	3.6119	3.9141	4.1681	3.7507	3.2155	4.2686	3.8274
3.7443	4.2922	4.5691	4.4012	3.2123	3.567	4.3943	3.857
3.6262	3.3261	4.1945	2.8789	4.9425	4.2496	4.45	3.4757
2.6944	4.5261	3.8856	3.5184	3.6682	3.8719	3.7356	3.7833
3.5341	3.4403	4.2786	3.7389	4.0443	3.4321	4.1678	3.9457
3.8956	3.7129	4.2435	4.4148	3.9372	3.3596	4.395	3.6354
3.6317	3.6984	4.345	3.4565	4.3243	3.8064	3.972	4.2175
4.0782	3.9245						
ARFIP2	3.3785	4.6856	4.1152	4.0788	4.4444	3.2486	3.5772
5.0818	3.0002	3.453	4.7044	4.6285	3.6914	3.0131	4.4269
2.9084	3.9299	2.5675	3.5289	4.2151	4.3129	4.3584	4.2423

4.5172	4.0772	4.4741	4.6717	3.7573	4.0687	4.2338	4.0871	
4.6813	4.201	3.9861	3.3251	3.2416	4.2663	4.0368	3.8551	
3.5115	4.4881	4.952	4.2122	3.589	3.8105	3.0436	3.7132	
4.269	4.1105	3.8477	4.8018	3.5468	3.5683	3.4878	4.1367	
3.3205	3.6737	3.9324	4.309	3.7538	3.9229	4.5767	3.6827	
3.7974	3.6254	4.1389	3.0404	4.0687	3.7446	3.8211	4.1258	
3.5231	4.146	5.545	3.3648	3.4503	3.839	3.5623	3.5952	
4.0953	3.4014	4.8031	4.9125	3.2968	3.1245	3.9039	3.4778	
3.6079	3.503							
ARFRP1	3.4969	3.5951	3.4883	2.8324	3.7252	2.6811	2.4954	
3.5635	2.7606	2.8886	2.7897	2.4076	4.6172	2.534	3.248	
2.8565	3.9251	2.4768	2.8832	3.2947	3.5093	4.1793	3.3512	
2.9681	3.538	3.8328	3.024	2.1836	3.1259	2.2148	3.3782	
3.419	3.3272	2.4794	3.3501	2.8003	3.4313	3.9015	4.1766	
2.7637	2.7506	2.8603	3.8954	2.7779	4.0482	2.9996	2.5499	
3.1422	4.7322	2.4406	2.8662	2.5514	2.9444	3.6754	3.4344	
3.63	2.9682	2.1859	3.6337	2.0711	4.5257	3.0512	3.2039	
3.086	2.0635	3.5228	2.5861	3.0768	2.8475	3.4787	4.0572	
2.8681	3.9396	3.5017	3.6274	3.3393	2.7342	3.6956	2.7941	
3.3633	2.7782	3.9955	3.0697	3.4838	3.8844	3.9341	2.9806	
3.2802	2.9852							
ARG1	0	0.0387	0	0	0	0.0503	0.0509	
0.0729	0.193	0.0618	0	0.0113	0.0126	0.0678	0.0156	0
	0	0.0456	0.0715	0	0.0594	0	0	0
	0	0	0.022	0.047	0.0542	0	0.0239	
0.0135	0.1141	0	0.0139	0	0.008	0	0.0128	
0.0824	0.0401	0	0	0.0529	0.0816	0.0291	0.1141	0
	0.1188	0	0.0283	0.1118	0.0808	0	0	0
	0	0	0	0	0	0	0	0
	0	0.0388	0	0	0.035	0.107	0	
0.0471	0	0.0154	0.0765	0.1418	0	0.114	0	0
	0.0571	0	0.037	0.0626	0	0.0841	0.0859	
0.0383	0							
ARG2	2.8553	3.8149	2.3898	4.4306	3.1565	2.4826	3.4168	
3.7467	3.2315	2.963	2.4122	4.0746	1.2942	2.9515	2.3136	
3.5736	2.3753	3.5518	3.2521	2.8	2.6986	3.6616	3.3992	
3.8367	3.0702	3.4242	3.4161	3.0211	3.4446	3.863	4.0337	
3.4717	3.1036	2.9757	1.907	3.4079	2.2682	3.1377	2.8646	
3.0204	3.479	3.5256	2.8904	2.8159	2.7479	2.2449	2.7376	
4.0755	2.1167	3.5317	2.2254	4.0858	3.1729	2.0257	3.0374	
1.6303	2.5752	2.7153	3.5039	2.612	2.4436	3.8415	2.1834	
2.4727	2.3369	1.8335	3.7686	2.8534	3.4032	2.0659	3.7769	
2.6678	2.6984	3.7086	3.5541	3.723	3.0003	2.7841	4.1745	
2.6194	3.8215	3.194	2.794	3.0003	3.1889	3.3935	2.5412	
2.3325	2.6989							
ARGFX	0.076	0.0317	0.1035	0.0583	0.0422	0.0652	0.0341	
0.0482	0.0687	0.0546	0.0144	0	0.0307	0.0512	0.0541	
0.0293	0.1164	0.0844	0.0741	0.0605	0.1136	0.0289	0.0163	
0.0837	0.0286	0.0438	0.0241	0.0541	0.036	0.0615	0.016	
0.0107	0.0524	0.0516	0.0105	0.0337	0.0245	0.06	0.0157	

0.061	0.0556	0.0114	0.0181	0.05	0.0366	0.0455	0.0454
0.054	0.0607	0.1084	0.0297	0.0161	0.0667	0.0321	0.0245
0.0975	0.0865	0	0.0273	0.0482	0.0664	0.0378	0.0261
0.0187	0.0836	0.0053	0.0794	0.0082	0.0423	0.1103	0.0213
0.0678	0.0562	0.0107	0.0893	0.1313	0.0527	0.0601	0.0283
0.0714	0.0492	0.0547	0.014	0.0458	0	0.0834	0.0383
0.0582	0.1614						
ARGLU1	5.7594	4.9479	5.3013	6.2727	5.5906	5.3773	5.3072
5.9034	5.1115	5.511	5.1663	5.2488	5.4169	5.4825	5.3821
4.6203	5.4261	5.2973	5.3318	4.9486	5.7371	4.9404	5.3045
5.0787	6.1965	5.0514	6.792	5.6346	4.758	5.3695	6.7545
4.5721	4.4852	4.7543	5.6692	5.5012	4.6496	4.9225	4.9892
4.5578	5.4221	5.6762	5.3992	6.0989	5.0836	4.273	5.4995
4.6435	5.9908	5.3848	5.6227	5.5118	5.4114	5.7306	5.1609
6.3837	5.4046	5.117	4.5082	5.1525	5.0889	5.062	6.105
5.5866	5.1758	5.039	4.9908	4.8196	6.2904	6.0075	5.1531
6.0368	4.7994	5.3121	5.6208	5.1209	4.3297	4.4943	5.5794
4.476	5.5754	5.6087	5.1393	5.2331	4.9301	5.046	4.8217
5.5445	5.4748						
ARHGAP1	4.3877	5.0446	4.5103	4.2226	5.0597	4.3908	4.3353
4.3025	4.3164	3.8866	4.9971	3.4602	3.9118	4.0901	4.8136
4.6072	4.7383	3.6105	3.7263	4.1114	4.4037	5.0312	4.5693
4.6835	4.8312	5.1364	3.7384	4.8591	5.2765	4.3199	4.0658
4.9219	4.5048	4.5323	4.8628	3.4521	4.4606	5.7074	4.0794
3.5089	4.5274	4.8525	5.1476	4.6484	3.9737	4.2838	4.0176
3.8055	4.858	4.1079	4.6595	3.8681	3.9332	4.0463	4.3
4.3824	4.9718	4.014	4.5304	3.8664	4.41	4.4611	4.264
4.8079	5.446	5.5725	3.968	5.8228	4.0491	4.6271	4.2619
4.1933	4.6267	4.7366	5.1367	5.2929	4.4384	4.4791	3.7935
4.6107	3.7867	4.7291	4.0897	3.7087	4.5551	4.0974	5.1726
5.1257	5.4606						
ARHGAP10	2.7078	2.6707	2.6629	2.5809	2.209	1.9113	1.5674
1.8826	1.9745	2.358	2.2719	2.3212	2.7507	2.698	2.0228
1.1422	3.0803	2.3648	2.4121	2.63	3.1538	2.0942	1.3651
2.7218	1.7362	4.1268	1.5787	2.4467	1.5982	1.7093	2.0506
2.2039	2.0372	2.0376	2.29	2.5011	1.7214	2.0076	1.9059
2.0816	2.4458	3.1084	2.959	2.2297	3.1225	3.0362	2.6175
1.3554	1.7069	3.7253	2.359	2.6678	2.2268	2.6304	2.5207
2.1823	3.3028	2.3614	2.3791	2.1696	2.475	2.2552	2.5651
1.9756	2.3434	3.4473	1.318	3.0806	1.6539	2.9588	2.5448
2.3328	2.5338	0.9167	2.639	2.049	2.5789	1.9833	1.8098
1.7835	2.2768	2.5139	1.9696	1.4542	3.4818	2.0792	3.2665
2.6587	2.5472						
ARHGAP11A		3.6004	4.7361	4.4984	4.4538	4.5828	4.1061
3.4201	3.9276	4.785	4.5153	2.8245	3.8164	4.0854	4.7017
4.1948	4.1529	4.3354	4.3677	4.7997	4.4172	4.4868	4.6752
3.4846	3.946	4.3359	2.5221	3.0008	4.3903	4.4415	3.8908
4.5469	3.7475	4.8177	3.4989	3.5139	4.7704	3.4645	1.4609
3.5966	4.3444	1.9307	2.9395	4.497	4.4907	5.1229	4.9497
4.9371	4.4564	3.1512	4.8639	3.8973	3.9174	3.8734	3.3759

4.8034	4.7096	4.0291	4.2168	4.4246	4.3611	3.9259	4.083
4.4318	4.7767	4.4483	2.1381	3.6636	1.9988	4.0647	4.1014
3.5055	4.2624	3.9112	3.7423	4.8528	4.0088	3.7446	3.8606
3.98	4.3675	4.592	3.072	2.1922	2.7416	4.7412	4.4402
3.2029	4.2892	4.0629					
ARHGAP11B		1.8811	2.1937	2.3728	2.2534	1.8864	2.1071
1.9369	1.9116	2.4529	2.0632	0.9156	1.2237	1.7719	2.7014
2.0797	1.9111	2.3699	2.5808	2.4481	2.2105	2.8162	1.9487
1.219	1.8094	2.9028	0.5727	1.9659	2.0626	2.0114	1.4971
1.9898	1.5565	2.0679	1.8167	2.3732	1.9959	1.8811	0.5347
1.7581	2.1827	0.8535	1.119	2.3906	2.3948	3.1454	2.6035
2.4847	2.1353	2.1645	1.7763	1.8094	1.6369	1.7674	1.6019
2.1204	2.8051	2.1048	1.4882	2.0752	1.925	0.9864	1.9171
2.3478	2.8503	2.2715	0.9658	1.8917	0.5606	2.5381	1.6306
1.3895	2.3174	2.0805	2.0567	2.1059	1.7525	1.646	1.3238
2.1731	2.1996	2.5302	1.136	1.2471	1.4184	1.871	1.9677
1.4268	1.7616	1.6799					
ARHGAP12	4.0465	1.7682	4.0158	4.1009	4.6724	3.3556	4.2971
3.1089	3.5701	4.1311	3.1999	3.7032	4.0841	4.2136	3.6827
3.5179	3.8221	3.494	2.8655	3.8577	4.2678	3.1521	3.636
3.3481	3.9615	3.0375	4.3062	4.3343	3.4361	3.0627	3.2725
3.4394	3.0508	3.7699	3.3597	4.0851	3.8189	2.9108	3.8971
3.9714	3.7125	3.971	4.0859	4.1296	3.9858	2.7851	4.1658
3.3107	4.113	4.2577	4.3669	4.5809	3.889	3.6163	3.4043
3.5254	3.9088	2.448	3.083	2.7264	4.1592	3.1797	3.8296
4.1772	3.3355	3.8915	3.1277	4.2862	3.9495	4.2197	4.2816
3.6184	3.1209	4.0127	3.9196	3.466	3.9398	3.8927	2.8864
3.3602	3.038	4.6279	4.0039	3.8959	3.8463	3.8403	4.2836
3.5855	3.531						
ARHGAP15	0.8032	0.1898	0.4891	1.5555	0.0425	2.7808	0.0759
1.6804	1.0249	0.6658	0.3099	0.696	0	1.6147	2.1678
0.5309	0	0.2776	0.973	1.9538	0.2098	1.8798	0.677
1.1494	0.171	0	1.8413	0.4766	0.4209	0.3154	0
0.9158	0.8195	1.8441	3.8616	0.6973	2.9172	0.1805	0.6527
1.0275	1.7698	0	0.2928	1.3809	0.0152	0.21	1.5105
0.6816	0.4354	0	1.4597	0.9646	0.7091	0.5473	0
0.8923	0	0.5551	0.48	0.4285	0.781	0.6997	1.5962
1.6304	0.6015	0.1339	0.5586	0.0957	0.2816	0.8179	0.5938
0.6241	0.9722	0.8329	0.6663	0.1434	1.2779	4.2283	0.9439
0.9491	0.4812	1.696	0.6648	3.6433	0.5632	0.9202	0.6955
0.4393	0.4164						
ARHGAP17	3.7275	4.9924	4.6742	4.1529	4.0007	4.1964	3.7207
4.5359	4.2413	4.0689	4.2473	3.8299	4.8252	4.3359	4.4969
4.0265	4.066	4.3087	4.6285	4.2166	4.2315	4.0265	3.8235
4.6596	4.3229	4.1529	4.1299	4.5871	4.4824	4.441	4.4815
4.553	4.2482	4.5218	4.3954	4.3405	3.8686	3.7763	3.3701
4.2055	3.9804	4.6983	3.9932	3.5276	4.6181	3.9536	4.6181
3.6175	3.7614	4.7745	3.7825	3.4569	3.7872	3.5306	3.6503
3.9238	4.2602	5.1249	4.4721	5.3346	3.7791	4.5139	4.1942
4.4446	4.6001	4.6166	3.9306	4.3929	4.4974	4.1316	4.1

3.9124	4.0244	3.9171	4.3451	4.0822	3.9499	4.2077	4.1353
4.1071	4.2209	4.0484	3.8415	3.8526	4.6817	3.5868	3.8848
3.9845	4.5334						
ARHGAP18	3.0735	3.2313	2.6938	4.8084	3.5935	5.1798	3.3051
4.0503	3.9348	3.0844	2.3716	4.0114	4.513	5.2523	3.6088
3.7955	5.5002	3.6242	4.7136	3.7897	4.0147	3.6916	4.3854
4.503	1.4105	4.0308	2.7369	2.8034	4.6509	3.0105	1.4995
4.6973	3.7889	3.3697	4.4422	3.2613	3.9567	3.3344	3.4903
3.9777	4.6015	4.3902	2.5969	3.1618	5.3275	5.1084	4.0695
3.7802	4.1071	5.5674	4.4413	3.7527	3.6938	3.4455	2.6757
3.1341	4.37	5.142	3.6831	5.5157	3.4608	3.8723	4.0547
3.9658	0.2044	3.1801	3.3532	2.3695	4.2184	4.0811	4.2902
3.9267	4.0526	4.2738	3.7917	3.086	4.1143	3.7462	3.9313
4.7278	3.7587	4.9559	4.1558	4.3148	3.456	4.056	3.4783
3.9709	2.5466						
ARHGAP19	2.8023	3.6231	2.3633	3.016	3.0401	2.2012	1.5014
2.7762	2.7878	3.1614	1.9351	2.7972	1.7099	2.6607	2.1678
2.5317	3.2235	3.2334	2.6934	2.1074	3.6185	1.9833	1.7725
2.6555	2.7595	1.1813	2.2092	2.0353	2.3715	2.2507	3.5673
3.0803	2.1892	1.8668	1.9141	3.5438	3.1714	1.8786	3.8461
2.5284	1.6046	0.6384	2.9937	1.9622	2.6412	2.8969	2.9782
2.8074	0.7324	2.9857	2.2462	1.8044	3.6106	2.0853	4.1229
2.5568	2.3467	2.8583	2.7332	3.0011	2.3557	2.1038	4.0148
2.8953	2.988	1.1723	2.7431	1.2939	2.123	4.0056	1.8035
2.5927	2.2214	2.6378	1.6197	2.3434	2.68	1.7818	1.5244
2.308	2.9143	2.1148	1.9053	3.6687	2.4145	3.3187	2.1343
2.6161	2.3255						
ARHGAP20	0.0581	0.8599	0.735	0.9304	0.0287	1.1858	1.5736
0.407	1.4039	0.0876	0.3412	0.0806	0.8277	0.0414	0.7084
0.8893	0.0407	0.3721	0.768	0.0379	0.065	0.8246	0.5466
0.5845	0.8557	0.2302	0.4541	0.4829	0.4133	2.0136	0
0.3523	0.4807	0.4309	0.978	0.1152	0.0875	0.6384	0.7222
2.1366	0.1751	0.2929	1.1347	0.9441	0.965	1.8716	0.3696
0.3977	0.4085	0.1027	0.3089	0.3757	0.747	0.0428	0
0.3889	0.434	0.4236	1.3087	0.1251	1.3171	0.2331	0.8348
0.0394	0.2079	0.63	0.8486	1.9044	0.2718	0.3341	0.5882
0.2817	1.1115	0.4147	0.3237	1.0562	0.0189	0.2093	1.2554
0.8468	0.6853	0.0086	1.7432	1.0267	0.1993	0.373	0.132
0.3882	0.1557						
ARHGAP21	4.9801	3.0955	4.5168	4.4361	3.3944	3.422	4.1539
3.4332	4.6791	5.4428	4.9302	3.7368	4.5022	4.3173	3.6088
3.5868	4.5003	4.4245	4.0188	3.9733	4.4489	3.5006	3.9853
3.8068	4.2349	3.7824	3.6156	3.8947	3.5101	3.1324	3.0435
4.0401	3.7016	4.5759	3.7382	4.7166	4.3418	2.7133	4.8923
3.8247	4.479	3.9587	4.3556	5.1283	3.6686	3.0363	4.0161
3.6092	4.0074	4.3951	5.2618	3.9536	4.7582	3.4062	3.607
5.9329	4.74	3.501	4.5657	3.5516	3.9031	3.9383	3.7897
4.2662	3.1256	4.6604	4.5325	3.8949	4.7873	3.8936	4.521
4.0619	3.3297	3.4752	4.0882	4.1583	4.3427	4.4466	3.0769
3.5658	3.9943	4.454	4.2697	3.8228	3.091	3.8804	4.6139

4.1343	4.4404							
ARHGAP22	0	0	1.2079	1.3317	0.5448	0.319	0.3664	
0.1077	0.2678	0	1.5186	2.1692	3.0218	3.1682	1.0263	
0.6352	3.4227	0	0.2083	2.1699	0.0117	0.1935	0.4656	
1.6703	0.0104	3.6396	0.3193	0	0.561	0.3808	0.3981	
0.1085	1.1374	1.2513	0.3943	0.0759	1.975	0.5173	0.3292	
1.6799	0.9543	1.4379	3.5901	0	2.1542	1.6743	0	
0.6366	1.5038	3.1475	1.2474	0.6801	0.0418	0.5539	0.7955	
0.1427	4.8634	0	0.1726	0.1326	0.5491	0.1717	1.0316	
0.5667	2.0041	4.0749	1.7537	2.424	0.6848	1.2576	2.0119	
0.8188	0.5951	0.1262	0.1219	0.1027	0.1593	0.2335	0.1601	0
	0.2314	1.3831	2.1717	0.4155	2.5426	0.1258	1.0185	
0.7154	0.1322							
ARHGAP23	3.9127	2.1101	1.225	1.9055	2.8521	1.2258	0	
1.502	3.01	1.9653	1.5675	1.016	3.4642	0.3852	1.8108	
2.6448	4.4512	2.4573	0.7505	1.9489	1.9764	2.942	3.8974	0
	2.6067	5.4029	2.889	0.1607	3.7046	1.3234	0.9259	
1.45	0.0172	1.8349	4.2557	1.3361	3.3138	1.2482	2.5926	
2.8816	2.0866	3.2883	2.7655	0.1172	3.8217	2.2405	1.1691	
2.3595	0.9297	2.8576	1.98	1.5026	2.446	1.8726	1.0592	
1.7564	4.8106	3.5751	1.146	3.2726	3.0343	1.3601	1.1139	
0.7547	2.1748	3.2772	1.9026	2.5616	2.6699	4.7584	0.1698	
3.3015	0.2344	2.8844	2.8798	2.6184	0.1466	2.1366	0.3728	
0.1716	0.7401	2.5074	3.7301	3.0042	4.1525	2.6176	2.553	
2.2497	2.7516							
ARHGAP24	2.4808	1.0268	0.1776	1.0622	1.436	2.2098	2.8094	
0.8576	3.3251	2.5008	2.6825	2.0924	2.2047	3.5394	1.0096	
0.3682	3.2545	2.0404	1.8786	0.2959	2.2605	1.634	1.5017	
0.694	1.2083	3.9761	2.9735	0.36	2.2519	0.8601	0	
1.5723	2.36	2.7076	1.7047	1.3589	1.2338	0.9621	1.0443	
1.2852	3.4235	3.6174	2.2018	1.4601	0.9653	0.5975	2.2191	
1.8043	2.0566	2.2729	2.0592	1.8471	2.3367	3.1399	0.274	
2.913	3.7438	0.3936	0.9196	0.9137	2.1908	0.8254	0.5413	
3.4423	0.496	2.4314	1.0955	2.3719	1.1293	1.4513	3.4572	
2.2494	0.6134	1.4493	2.1563	0.9412	1.3151	1.081	1.7069	
2.0026	1.7262	3.839	1.8139	3.3745	2.0358	2.0203	4.1698	
2.518	3.2242							
ARHGAP25	0.319	0.0644	0.0083	0.179	0.4409	1.213	1.5361	
0.5238	2.9173	0.0429	0.0312	1.2366	0.8356	0.0175	0.3068	
0.1251	0	0.2359	0.8004	0.2205	0.5581	0.4023	0.04	
0.0775	0.1763	0.5813	0.392	0	1.7705	0.1962	0.3117	
0.2512	2.4031	1.5895	1.12	0.0778	0.7131	0.439	0.3941	
2.2563	1.575	2.6468	0.005	0.2345	0	0.1523	0.0443	
2.4047	0.1538	0.0929	0.1136	0.3684	0	0.182	0.1649	
0.1074	0.5476	0.1005	0.4658	0.113	0.2699	0.2844	1.6714	
0.6156	0	0.0674	0.1568	0.3018	0.1216	0.2087	0.1402	
0.2424	0.0897	1.3704	0.1287	0.3508	0.1683	0.2152	0.2287	
0.1522	3.8691	1.2413	0.9048	0.7338	0.1393	0	0.0179	0
	0.0755							
ARHGAP26	1.1827	1.4924	1.4582	3.1272	1.5328	1.262	1.632	

0.6317	2.1821	1.1529	1.4114	2.6469	2.848	0.7442	2.614
1.7434	1.0912	1.5394	1.973	1.8672	1.4841	1.6509	1.5459
1.6675	1.83	0.7356	1.98	2.6986	1.4552	2.7375	0.3242
2.4252	0.686	1.5826	1.2809	1.9773	0.6461	2.1639	2.3048
1.0374	1.7011	2.7649	2.8085	1.1359	1.1646	1.3469	1.8712
0.9772	3.0798	1.216	1.0374	1.2872	2.5394	1.7836	0.4509
1.1332	2.5962	0.6751	1.4273	0.8278	0.6852	1.9206	1.64
1.4736	0.6221	1.0925	1.3345	0.6135	1.4902	0.9993	1.1035
1.652	1.5165	2.4664	2.3587	1.3946	1.8247	3.9734	1.5492
1.6609	2.2419	2.4798	1.0376	2.387	1.2778	1.9098	1.0519
0.6743	1.172						
ARHGAP27	1.7281	1.6113	2.6898	1.1	2.0984	0.7951	1.1191
1.1526	1.9314	1.3585	1.186	0.4881	1.3299	0.1981	0.2553
2.6274	1.7312	1.2204	1.1258	2.1285	1.5784	0.3155	1.6163
0.8311	0.1754	0.6585	0.8014	2.035	0.8371	2.8338	3.3014
1.7827	0.9987	1.2709	1.0871	1.5333	1.3428	1.3454	0.3947
1.2562	1.3749	2.7409	0.3868	1.0405	2.0674	0.8661	1.2068
0.8727	0.5125	0.8059	1.2163	0.161	1.0443	0.933	2.8061
0.9588	2.4205	2.5182	2.4321	2.4092	1.1765	2.0695	0.5471
0.719	0.5013	1.4046	0.8808	1.5679	1.7172	0.3682	0.7964
0.5317	0.5547	1.0251	0.329	2.6588	0.9149	0.643	1.6003
1.2873	1.7986	0.2983	2.6428	0.1751	0.2261	1.2332	0.3235
0.4446	0.5508						
ARHGAP28	0.3946	1.7488	0.9183	1.3092	0.2725	1.4996	1.5745
1.6271	1.6812	1.3514	2.055	1.3371	0.3504	1.9049	0.4662
0.9847	0.0585	0.6076	2.2415	0.6198	1.3074	1.2655	0.8305
0.6713	0.9731	0.3382	0.2923	0.6795	0.5663	1.2346	2.4601
2.503	1.8713	0.7685	0.5325	1.4861	0.1027	1.2366	0.6046
1.2606	1.9579	1.7772	0.1899	1.0171	0.2111	2.4353	1.3684
1.4157	0.9693	0.0911	1.323	2.055	1.6456	0.6007	1.6701
1.1373	0.0904	0.1496	1.494	0.0071	0.2577	2.6212	1.1268
1.8902	0.5728	0.6358	2.5819	0.5445	1.0543	0.3776	1.4296
0.8864	1.8138	1.6229	1.092	1.0965	0.4585	1.28	1.3526
2.0579	1.319	0.0334	0.543	0.5377	0.8539	1.6765	0.0691
0.4365	0.4212						
ARHGAP29	0.899	3.3468	3.7856	2.2629	2.9698	1.1873	4.0675
1.3898	1.6231	1.9725	1.0135	4.1801	5.7519	2.6538	1.7705
2.9294	0.3178	0.1667	3.2177	0.9917	1.703	0.8733	2.7179
3.4733	0.9666	6.1009	1.6406	4.2275	0.6791	3.0104	0.8879
2.4893	2.3592	2.9004	1.6334	2.2347	0.9364	1.2868	0.6362
1.3308	1.3816	4.9882	3.6216	3.3731	4.2161	2.5762	1.7816
0.3801	3.8152	5.4047	1.9932	3.0338	3.8352	2.831	1.5852
2.5299	7.8796	1.0944	0.7906	0.6962	1.8367	1.713	1.1938
0.6129	0.4916	3.474	1.1337	3.6674	3.7972	1.9986	2.3688
1.4691	2.0014	1.6734	2.2149	1.1737	2.7411	1.1141	2.9414
1.7603	4.8476	4.26	0.8591	0.5286	3.4936	2.5579	1.2121
0.4844	1.5716						
ARHGAP30	0.9305	0.1486	0.2245	0.4586	0.4465	0.3583	0.0545
0.0425	0.1909	0.2517	0.0631	0.3544	0.0044	0	0.0305
0.0305	0.0535	0.5349	0.2	0.2316	0.5009	0.0227	0.0379

0.0877	0.1014	0.0755	0.2524	0.0658	0.0309	0.0244	0.7466	
0.9544	0.1606	0.1074	0.0083	0.1132	0.4916	0.1066	0.1423	
0.5299	1.2797	0.1981	0.3342	0	0.1172	0.5694	0.0214	
0.3092	0.0616	0.1124	0.1915	0.3441	0.1557	0.2461	0.2194	
0.094	2.3786	0.0571	0.2583	0.1049	0.1474	0.5101	0.1367	
0.0709	0.2493	0.1645	0.3088	0.1003	0.0946	0.1102	0.109	
0.1827	0.0743	0.1609	0.076	0.1331	0.2714	0.1389	0.128	0
	0.1366	0.1654	0.1618	0.2175	0.0716	0.2648	0.0564	
0.1033	0							
ARHGAP31	2.0557	2.5946	1.8041	2.2541	2.5573	3.4264	3.2215	
1.2291	4.3736	2.6969	3.4508	2.2721	2.1597	4.0058	1.7038	
3.7263	2.4948	2.9605	3.5283	2.6408	2.6155	3.5763	2.6391	
3.1176	2.9112	2.2788	0.9591	2.5207	2.885	2.8907	0.3417	
3.8727	3.8497	4.21	3.0104	2.6611	1.7256	2.5181	2.9148	
3.5152	3.0704	1.9061	3.5271	2.6326	1.9913	2.4874	2.31	
4.065	3.2033	1.4968	3.2664	3.7991	4.5074	3.393	1.1433	
3.6688	2.6086	1.0229	2.1461	1.3155	2.6603	2.5032	2.9868	
3.9865	1.5321	1.6546	2.9269	2.4413	4.2496	3.3769	2.5638	
3.5696	2.2403	3.1739	2.4541	3.2988	2.9128	3.4688	3.9915	
3.2519	3.9334	1.7577	2.4952	4.4667	2.4341	3.2745	3.5584	
2.9954	3.3402							
ARHGAP32	2.1114	2.4495	2.4993	2.5819	2.126	1.9309	3.6983	
2.0895	3.631	2.2709	1.98	2.3719	1.7186	2.4246	1.5353	
2.9567	2.859	2.8491	2.4284	2.4552	2.3976	3.0016	2.428	
2.228	2.5233	1.6553	2.9175	2.2533	3.0119	3.3578	2.2299	
1.779	2.422	2.4256	2.7653	1.978	1.8905	2.1191	2.8546	
2.9578	2.7841	0.5718	2.4781	2.4372	3.3267	1.6399	2.822	
3.7558	2.0661	2.2598	2.8728	2.3783	2.5719	2.5981	1.9726	
2.8723	1.8349	2.1582	2.2565	2.2083	1.8155	2.2152	2.5194	
3.0218	1.7586	1.6661	2.1817	2.3536	3.6528	2.5819	1.9257	
2.4587	2.1223	2.0375	2.6532	3.6177	2.0512	1.9623	3.4603	
2.3098	2.2754	2.0471	3.2559	3.0696	1.3588	1.7644	2.2752	
2.1698	2.3093							
ARHGAP33	1.7191	0.4714	1.506	0.8008	1.0942	1.0706	0.4332	
1.3808	1.1216	1.1471	0.9814	0.3521	2.1808	1.5122	2.0051	
0.942	1.5448	0.7666	1.0199	1.8391	2.1984	1.068	1.4005	
1.2061	1.5327	1.1159	0.8687	0.9769	1.1392	0.571	3.3111	
0.9965	1.355	1.5116	1.7895	0.8897	0.8873	1.2912	1.3795	
0.9623	1.0836	0.5047	1.7093	0.9579	1.5378	1.2628	0.3688	
0.8296	1.4649	1.1556	0.911	0.6975	0.8756	0.5607	2.0247	
1.7433	0.5839	2.2143	1.3215	2.307	1.8289	1.0817	1.9148	
1.28	1.3227	0.8761	1.2248	0.2661	0.7777	1.3767	1.0925	
1.7754	1.0122	1.1842	1.521	1.2281	0.8463	0.7647	0.6554	
1.1577	1.481	0.4932	0.4763	1.1683	2.3093	1.644	1.123	
1.6984	2.0611							
ARHGAP35	3.5017	3.7178	4.0582	3.8274	4.1586	3.7712	4.1912	
3.5487	4.0299	3.6155	3.2271	3.8701	4.7902	3.3964	4.0464	
3.5499	4.4681	3.8613	3.9079	3.3568	3.6669	4.42	4.6934	
3.4633	5.1898	4.5855	3.6632	3.6514	4.5846	3.915	4.0263	
3.9343	4.1034	3.8724	4.0907	3.4681	3.4812	4.1764	4.1595	

4.3571	4.0366	3.8827	4.2697	2.7735	5.4898	4.6274	3.7567	
4.096	4.9761	4.0347	4.1949	4.823	3.852	4.6737	3.3621	
4.4755	3.5142	3.374	3.5839	3.8988	3.9197	3.5653	4.2431	
3.935	3.5962	4.8784	3.048	4.3709	4.5149	4.1833	4.0833	
4.2844	2.9599	4.5564	3.8391	4.3983	3.3286	3.5732	3.7734	
4.0876	3.7994	4.1162	4.2475	4.7223	4.2561	3.3329	4.1201	
3.7555	3.9715							
ARHGAP36	0	0	0	0	0	0	0.0065	0
	0	0	0	0	0	0	0	0
	0	0	0	0	0	0	0	0
	0	0	0.0323	0	0	0	1.2418	0
	0	0	0	0.0084	0	0	0	0
	0	0	0	0	0	0	0	0
	0	0	0	0	0	0	0	0
	0	0	0	0	0	0.0319	0	0
	0	0.0255	0	0	0	0	0	0
	0.0489	0	0	0	0.0716	0	0	0
	0	0	0	0	0	0	0.0439	0
	0							
ARHGAP39	1.9209	1.6568	1.8631	1.3038	1.3077	1.5904	0.9038	
1.5439	0.9127	1.295	1.1109	0.4849	1.1139	0.7586	1.9979	
1.1209	1.916	0.9901	1.369	2.1518	2.0246	0.7446	0.8857	
0.6471	1.1386	1.2271	0.4465	1.5482	0.7928	1.3428	2.4586	
2.466	2.1931	1.2014	0.7175	1.4007	1.2363	3.0236	1.3167	
0.8952	1.5119	0.7913	0.7803	0.6838	0.8481	1.4363	1.5596	
0.8462	0.8462	1.936	0.9891	0.7208	1.0379	2.1638	2.6018	
1.8449	0.7274	1.9928	1.5844	1.7957	0.7073	1.8819	2.076	
1.0591	2.9459	0.6714	1.637	1.5076	1.1052	1.3745	1.7743	
0.4325	1.4634	0.923	1.7952	1.0379	1.8571	1.5963	1.6022	
1.6731	1.7041	1.4221	1.1955	0.7502	1.8261	0.9023	1.1451	
1.3214	1.331							
ARHGAP4	4.1122	0.2363	0.4258	0.1222	2.7624	0.7896	0.1922	
3.3194	0.1847	2.7532	0.3564	1.9818	0.335	0.3434	0.1299	
0.9472	0.1091	2.1761	0.7405	0.5103	1.2457	0.1625	0.4093	
0.3805	1.5826	0.1306	1.4031	0.5395	0.1249	0.3784	3.1589	
0.8191	0.205	4.662	0.1593	1.8129	0.8605	0.299	2.3996	
0.0782	0.8987	0.5829	0.1821	0.5615	0.1658	0.0982	0.1987	
3.4221	0.4019	0.5543	0.1148	2.4167	0.8685	0.7439	0.4482	
0.7621	0.3393	0	1.0819	0	0.0411	0.3431	1.1512	
0.8164	0.3389	0.7271	0.6036	0.8358	0.4644	0.316	0.3036	
0.3363	0.1921	0.4724	2.7752	0.1445	0.7227	4.0256	0.7905	
0.7573	2.824	0.2228	0.8921	0.6627	3.877	2.756	0.6368	
1.0287	0.771							
ARHGAP40	0	0	0	0	0	0	0	
0.0336	0	0.8663	0	0	0.1157	0.3173	0	
0.1276	0.1181	0	0	0	0	0.2093	0.2738	0
	0.0562	0.3231	0.0894	0.0271	0.0946	0.0712	0	
0.8138	0	0.155	0	0.2056	0.1177	0	0	
0.138	0	0.467	0	0.0269	0	0.0973	0.0183	
0.1573	0	0	0.019	0.3404	0.3823	0.499	0	0

	1.4295	0.0503	0.0517	0	0.0283	0.0259	0.0284	0
	0.0387	0	0	0	0.0975	0	0.0895	
0.0286	0.0328	0.2889	0.0291	0.0872	0.0332	0	0	0
	0	0	0.0391	0	0.0884	0.1147	0	
0.0127	0							
ARHGAP42	2.8272	2.2909	1.846	2.6303	0.7609	1.7571	2.3725	
2.4847	4.4462	3.557	2.0711	2.8493	0.7461	3.0985	1.47	
2.9825	1.9947	2.5694	2.5461	1.9984	1.9996	2.351	2.0044	
2.9922	1.3451	2.3842	1.6348	2.1232	2.6842	3.0753	1.8666	
1.701	2.8134	4.1719	1.5899	2.7612	2.1337	3.1898	2.4176	
3.6969	3.8648	1.2567	0.7649	3.5429	1.1223	2.1507	2.7523	
3.7249	1.7552	1.8327	2.8778	2.0229	3.219	2.8118	2.2564	
2.3037	1.2162	1.6417	2.8817	1.7546	1.8613	2.0808	2.6022	
2.6518	3.5583	2.3502	3.5211	1.5946	2.305	1.6335	4.137	
1.8498	3.0542	1.9059	2.414	3.1866	1.7857	2.7055	3.89	
2.8989	2.5493	2.3795	5.9937	2.7369	1.1791	2.3742	2.7121	
3.2316	3.463							
ARHGAP44	0.162	0.2775	1.4761	0.3023	0.4017	0.4649	0.3557	
0.1222	0.1268	0.4549	0.177	0.0566	0.0603	0.2723	0.1155	
1.3984	0.0646	0.3011	0.1418	0.263	0.2795	0.2944	0.151	
0.2803	1.2827	0.2012	0.2761	0	0.1788	0.0658	2.3838	
0.0702	0.2504	0.082	0.0289	0.6691	0.0622	0.1253	0.0807	
0.9944	0.0995	0.1781	1.1097	0.1434	0.7917	0.1192	0.1272	
0.1738	0.0472	0.1271	0.2458	0.7911	0.1805	0.1863	2.1857	
0.2418	0.0342	0.2209	0.0303	0.0262	0.7469	0.5259	0.2953	
0.7196	0.2041	0.1319	0.1148	0.3053	0.1208	0.0183	0.1499	
0.146	1.2911	0.0216	0.9235	0.0591	0.2776	0.1649	0.2007	
0.5147	0.3318	0.1354	0.2976	0.2126	0	0.1244	0.1109	
0.2669	0.1149							
ARHGAP5	3.8806	3.6021	3.8773	4.8071	3.6875	4.7359	5.7848	
5.5799	4.0995	3.6464	3.6266	4.8106	3.0966	4.5259	5.0369	
4.6432	3.5592	5.0228	4.4057	3.517	4.0934	4.3388	5.0277	
4.0695	4.8487	3.7142	5.301	4.3113	4.8382	3.6585	4.1085	
4.2966	3.9398	4.0392	4.2511	3.7114	3.8943	4.2018	4.5186	
5.4585	3.8468	3.4072	4.1881	4.3772	4.428	3.5834	4.2704	
4.8527	4.3388	3.8974	4.3718	5.1088	3.99	4.211	2.9879	
3.0029	3.9632	3.9983	4.2317	3.6606	4.9194	3.7798	4.9365	
3.4666	3.7023	4.623	4.57	3.903	4.192	4.4563	4.9457	
4.2991	4.01	4.8558	5.0177	4.0231	3.903	4.4898	4.9095	
3.9395	5.1103	4.471	4.6221	5.2183	3.3551	3.854	4.4338	
4.1664	4.1738							
ARHGAP6	0	0.0732	0	0.0577	0.09	3.7309	2.989	
0.0225	0.9582	0	1.0364	0.1576	0.0254	0	1.2403	
0.0499	0	0	0	0	0	1.2274	2.062	
2.6299	2.7547	0.4415	0.8252	0	1.8622	0.0284	0	0
	0.7927	0.1457	2.4339	0.031	0	0.0356	1.0289	
4.2204	0.1723	0	1.1235	1.5385	0.1422	0	0	
0.4882	0.3881	0.0852	0.1494	1.6116	0.4953	0	0.0058	
0.0151	0.6793	0.2573	0.4152	0.2137	2.1348	0.0803	1.1561	0
	0	0.5239	0	0	0	3.353	2.3539	

0.904	0.9335	0.8398	1.1756	0	0	0.7192	0.8701	
1.8177	0.6643	0.2277	0	4.1166	0.4474	0.2606	0.4188	
0.4091	0.6646							
ARHGAP8	0.4836	1.4722	0.1154	0.0464	0.0218	0.1339	0.3983	0
	0.3991	0.771	1.5156	0.0114	0.0947	0.1079	0.0121	
0.0891	0.2943	0.4022	0.1785	0.1017	0.4801	0.0243	0.0393	
0.045	0.0771	0.0329	0.0189	0.0734	0.0171	3.4983	1.7287	
0.1136	0.1887	0.1632	0.0811	0.9442	0.1401	0.1933	0.0494	
0.0255	0.171	0.0104	0.3227	0.0277	0.0814	0.147	0.354	
0.0208	0.0217	0	0.0785	0.0188	0.0684	0.0966	2.9665	
0.529	0.2386	0.3357	0.0257	0.6356	0.1336	0.5139	0.0301	
0.0878	0.2859	0.0856	0.3873	0	0.3015	0.1398	0.075	
0.0066	0.1368	0	0.052	0.6212	0.7979	0.362	0.2063	
0.0426	0.1054	0.0291	0.0332	0	0	0.0882	0.0593	
0.0992	0.2398							
ARHGAP9	0.4208	0.4897	0.3536	0.2734	0.8853	0.1341	0.2	
0.0354	0.2174	0.2431	0.0898	0	0.0595	0	0	
0.0667	0.6646	0.0477	0.0878	0	0.1233	0.3035	0	
0.1232	0.0727	0.1158	0.0541	0.1357	0.0301	0.031	0.0722	
0.1279	0.1918	0.027	0.2618	0.0754	0.3282	0.6821	0.2991	
0.0968	0.081	0.0634	0.0657	0.2533	0.0165	0.1102	0.1174	
0.2661	0.0385	0.8296	0.0529	0.083	0.0802	0.6888	0.0856	
0.3702	0.698	0.7212	0.0495	0.7887	0.1321	0.0521	0.0905	
0.0564	0.1007	0.1211	0.4583	0.1924	0.1291	0.1213	0.1127	0
	0.0773	0.0502	0.0709	0.1282	0.1097	0.0592	0.5537	
0.0884	0.2163	0.0831	0.1984	0	0	0.0884	0.2344	
0.2914	0.4636							
ARHGDIA	8.0974	7.2911	8.2035	5.2977	7.2678	6.7834	6.0911	
7.4699	6.4867	6.8436	7.1587	6.4443	7.3304	7.2383	6.9921	
7.212	7.4516	6.0079	6.4758	7.4988	7.5114	6.6673	6.6445	
7.5509	6.6981	6.7978	6.4212	8.193	7.0529	6.4923	5.9207	
7.7925	6.9363	7.1666	6.8717	6.727	8.1305	7.8755	7.1456	
6.2086	7.4469	6.7877	7.0547	7.0215	6.9105	6.1871	6.4243	
6.7605	7.1844	6.0496	7.093	5.616	5.7956	6.835	7.516	
7.5961	6.7412	7.319	6.9346	6.8865	7.2832	7.5085	8.251	
6.8865	7.4207	6.4806	7.8433	7.5015	7.8165	7.3622	7.7704	
7.4093	7.9304	5.9316	7.001	8.096	7.8633	6.7158	6.916	
6.2523	7.3821	6.3378	7.5458	8.3447	7.5597	6.3162	7.5622	
8.0107	7.9097							
ARHGDIB	0.6033	1.4059	0.207	5.2026	1.0559	1.2995	0.9799	
2.1921	3.357	0.332	0.0703	3.282	0.5186	0.4902	1.7223	
0.1853	4.9093	0.7993	0.4677	0.3811	0.1463	4.1159	3.0609	
1.9104	0.8958	3.8673	1.65	0.5647	1.9298	2.3346	0.0848	
1.6313	0.9983	2.0161	6.9501	0.2195	0.4459	2.9203	1.5267	
5.1333	2.1512	3.321	0.1992	2.0417	3.158	4.3159	1.501	
1.6841	0.5086	5.41	2.4778	1.5856	1.2013	1.7358	0.3817	
1.3576	8.1276	0.3201	1.0221	0.6611	2.052	4.5024	1.3764	
0.731	0.8492	0.5266	1.0221	0.2682	3.1893	1.4106	2.2839	
0.7838	1.7104	1.0315	1.4978	1.0187	0.778	2.4245	5.0118	
3.4962	2.0826	3.7684	0.4727	2.1261	1.3652	1.6522	0.6916	

0.9452	0.5948							
ARHGDIG	0.0411	0	0.5829	0.1827	0.3269	0	0	
0.0197	0	0	0.5297	0.0672	1.1728	0	0.3434	0
	0	0	0	0	0	0.0166	0	
0.0217	0.876	0	0.6335	0.0408	0	0.1741	1.8879	
0.0688	0.3958	0	0	0.3553	0.0169	0.4613	1.2638	
0.8044	0	0.0451	0.1351	0	1.9421	0	0.5041	0
	0.0217	0	0.138	0	0	0	1.2426	
1.1984	0	0	0	0	0.7947	0.0557	1.0166	
0.0438	1.995	0.1063	0	1.0409	0.5087	0	0.1376	
0.0511	0.5013	0.0359	0	0	0	0.2195	0	0
	0	0.0619	0	0.5027	0.1063	0	0	
0.0815	0							
ARHGEF1	4.5653	4.8405	4.5193	3.443	5.3537	4.0735	3.081	
3.8759	3.8599	3.4843	5.3477	3.8466	3.8814	3.3446	4.4543	
3.6032	4.7332	3.2768	3.6294	4.5696	4.3471	4.6767	4.7826	
4.122	4.4739	4.6604	3.7789	4.8308	4.4153	4.9674	4.6668	
4.7997	4.3885	4.4639	4.135	2.6771	5.1222	4.3051	4.3736	
2.9694	5.0912	4.4162	4.2017	4.3609	3.8391	3.5414	2.8983	
3.9771	4.7068	3.7171	4.0702	3.4124	3.4734	5.0032	3.655	
4.603	4.8954	4.2769	3.7629	4.435	5.2368	4.4888	4.8054	
4.5439	3.9848	3.7072	2.1593	4.119	4.4098	5.3589	4.0374	
4.9127	4.2306	4.4529	3.8782	4.467	4.0474	3.1835	4.1859	
3.668	4.1195	4.3469	4.1242	4.4394	5.6741	4.7721	3.9387	
4.561	4.4014							
ARHGEF10	3.9408	2.2679	2.8377	3.026	2.3097	2.8877	2.784	
4.2863	3.4651	3.2212	2.9418	2.8377	4.1287	3.5238	0.1855	
2.837	4.0139	3.0696	3.0446	2.4969	3.1299	3.2722	3.3144	
2.9646	3.7552	3.3257	3.0696	3.0187	3.9894	2.7126	3.8237	
2.8948	2.7405	3.8217	4.4791	2.8289	3.3167	3.1325	4.0963	
3.7438	2.0763	2.0112	3.3311	3.8904	4.3886	2.8591	3.9027	
2.699	3.7399	3.8465	3.9716	3.0852	3.8282	3.4422	2.9944	
2.8266	2.9691	3.3054	3.734	3.9184	2.8954	2.5262	4.0493	
2.6071	4.7322	2.1351	3.7251	3.0636	2.0928	4.3643	3.8273	
3.0505	2.9951	3.1231	3.8185	1.8576	2.6203	3.7072	2.4137	
3.0457	3.7691	3.1854	2.7212	4.5283	3.6658	3.8153	2.3949	
2.2789	2.6753							
ARHGEF10L		1.6666	1.7894	2.3338	1.2083	1.197	1.3965	
1.6784	1.4823	1.877	1.5185	2.3956	2.0384	2.5815	1.4194	
2.0536	1.8525	0.0398	1.2099	0.8053	1.9222	2.2636	1.9859	
2.0727	2.1825	1.6007	0.6791	1.5228	1.9216	1.378	2.1734	
1.3844	1.451	1.1915	3.4075	2.913	0.7339	2.8203	3.2935	
2.6652	1.0531	2.3442	1.722	1.691	2.0695	1.3437	0.9599	
0.7183	1.7135	2.1198	0.3719	2.279	1.1249	1.9196	2.3688	
1.4818	1.849	2.1221	4.507	1.0521	3.9952	1.3175	2.2218	
1.3126	1.0126	1.8698	3.6826	1.9749	2.4163	1.8674	1.6211	
0.846	0.7145	1.6344	1.6272	1.7423	1.8627	1.9908	1.9617	
2.0265	2.296	1.544	1.2382	1.9697	1.7818	2.273	1.8346	
2.8153	2.8301	3.2103						
ARHGEF11	4.1699	3.6639	4.0795	3.8072	4.4977	5.3319	5.0496	

4.7978	4.5073	5.2606	4.4146	4.6997	3.1371	5.1577	4.4104	
5.8284	2.8856	4.7017	4.4237	4.3409	4.2824	5.2804	4.1618	
4.8369	4.322	3.9372	3.8106	4.0218	4.8053	4.0822	4.1174	
5.8872	4.0561	4.563	4.2902	4.1769	4.9114	3.6396	3.9403	
4.3794	4.0574	2.7502	3.6539	4.96	3.4123	3.4727	3.7458	
5.1023	4.8904	2.3376	4.1532	4.8184	4.6601	4.7262	3.9559	
4.1744	3.1203	3.7558	4.8209	4.0789	4.2398	4.055	4.0671	
4.2195	3.8264	4.0039	3.9687	3.6941	5.3703	4.5892	3.8648	
4.9023	4.4412	4.9952	5.3827	3.492	3.909	5.2417	3.9815	
3.4959	3.5489	3.623	4.4486	5.6956	3.5971	4.7749	3.8428	
3.8021	3.5817							
ARHGEF12	4.1155	3.6594	3.9193	4.3779	4.2256	4.304	4.9211	
3.2743	5.0411	4.0977	3.311	3.966	4.1084	4.291	4.2194	
4.6543	4.6755	4.4013	4.0441	4.111	3.4399	4.3677	4.3992	
4.0923	3.5567	4.5168	3.8964	3.8763	4.7879	3.9956	4.3487	
3.2828	3.7259	3.8197	4.9722	3.9533	3.6647	4.3804	3.9183	
4.7241	4.41	4.4114	4.3565	4.7072	4.9518	3.6771	3.5276	
4.7626	4.406	4.4674	4.224	4.7856	3.4028	4.4843	3.8616	
4.3981	4.9772	3.9477	4.0127	4.1864	3.5875	3.722	3.9011	
4.331	3.5466	4.9766	4.6692	4.9223	4.1932	3.9583	3.8248	
3.9025	4.2516	3.9597	5.0237	4.8788	3.2271	4.3666	4.5447	
3.6485	3.7166	4.832	3.8935	4.806	3.5038	3.0058	5.1338	
4.7032	5.2417							
ARHGEF15	0.0824	0.0267	0	0	0.0143	0	0	0
	0	0	0.0222	0	0	0.0868	0.0386	0
	0.1134	0	0	0	0.0825	0.106	0	
0.0609	0.0107	0.0162	0	0.0751	0	0	0.0128	
0.0081	0.0464	0.0216	0.0094	0.0307	0.0191	0.0325	0	
0.0241	0	0.0219	0.1024	0	0.014	0	0.0667	0
	0	0	0.0538	0.081	0	0.0091	0.0214	
0.1388	0.0308	0.0083	0.0735	0	0.0137	0	0.0025	
0.0392	0	0.0092	0	0	0.0058	0.0371	0.1442	
0.0238	0.0161	0.0753	0.0267	0.034	0.0292	0.0344	0.0727	
0.0688	0.0831	0.0057	0.0612	0.0554	0.0691	0	0	0
	0							
ARHGEF16	0.6674	0	1.7063	0.2136	0.1798	0.3542	0.3462	
1.2881	0.4867	0.1653	0.0919	0.2159	0.6529	0.3715	0.2691	
2.5698	0	0.8768	0	1.1068	1.6827	0.9222	0.1328	
0.2716	0.2285	0.0658	0.2108	1.1669	0.3321	1.3382	2.9419	
0.3569	0.0838	0.1233	0.176	0.2893	1.1301	2.0913	0.8913	0
	0.0699	0.0332	0.4147	0.056	1.1798	0.7923	0.3243	
0.2663	0	0	0.3854	0	0.4732	0.8282	1.8282	
2.7117	0	2.1966	0.377	1.9043	0.021	0.0281	1.3106	
0.2504	1.3142	0.0018	0	0.1835	0.2423	0.9603	0	
0.0022	0.4463	0.2061	0.0704	0.0475	0.3709	0.5535	1.7729	
0.2392	0.4708	0.0827	0.0983	1.2848	0.1683	0.745	0	
0.2551	0.0806							
ARHGEF17	2.0404	3.0877	1.4987	1.7596	1.4397	2.2423	1.5207	
1.7136	3.0207	1.4172	2.7988	1.3599	2.1928	1.8799	1.5379	
2.4104	3.0837	1.5425	1.1154	2.138	3.6921	2.5136	2.9721	

1.7288	2.3806	2.9501	0.719	0.8587	1.5741	1.446	2.0472
1.4186	1.5676	2.8253	2.2079	0.8971	2.6379	2.4858	2.71
1.9906	2.8368	2.3437	2.9879	1.7893	2.6334	2.2575	0.6261
2.9098	3.0021	2.0985	0.8703	0.7791	2.1057	2.3702	2.3634
1.8699	2.7738	2.2982	1.7552	2.419	0.5422	1.7608	1.0284
2.1143	1.7308	0.6952	2.2098	2.2939	0.6273	2.5261	1.2173
1.8133	0.8859	1.8421	2.3509	0.3426	3.2569	2.2824	2.1936
1.8121	3.2253	2.846	3.5138	2.0536	2.3387	2.2838	2.5115
2.5657	3.1469						
ARHGEF18	3.2428	3.1379	3.4771	2.4131	3.0419	3.1454	3.0602
2.9607	3.1439	3.2432	3.4497	2.4084	3.9882	3.0232	3.2394
2.8577	3.9104	2.571	2.9199	3.1568	3.1516	2.8328	3.05
3.2166	2.8264	4.1887	2.3503	3.3357	2.8982	2.7709	3.5184
3.4394	3.4956	3.6915	2.7581	2.5331	3.4767	3.457	3.4934
2.6796	3.706	3.5161	2.7301	2.5697	2.8511	2.9798	2.4458
2.4898	3.1169	4.0549	2.6547	2.3376	2.6618	3.8664	3.9097
3.3777	3.9587	3.3904	3.0349	3.3173	2.9712	3.4243	1.97
2.9529	2.8995	2.9742	3.0037	3.8396	3.2938	3.046	3.3249
3.5695	2.8712	3.0586	2.643	3.1921	3.7243	2.537	3.4306
2.7759	3.0213	2.792	3.2881	2.2904	3.3955	3.0537	3.3697
3.3669	3.4301						
ARHGEF19	1.0448	0.7196	1.4008	1.3132	3.0037	0.6864	0.6719
2.2131	1.3884	1.3474	1.6683	1.632	1.7398	2.0325	0.4684
1.3707	1.3589	0.574	0.5082	1.695	1.777	2.5624	0.0376
0.8725	1.5034	2.126	0.2061	1.2836	1.9637	1.0681	2.2547
2.3094	2.4375	1.4877	2.1562	1.1304	1.968	1.1141	1.3132
0.9989	1.7265	1.9407	1.7439	1.8601	1.1054	1.2413	0.5464
2.7073	2.1604	1.3295	2.1306	1.1832	1.1547	1.8119	1.7954
0.3127	1.4221	2.0778	1.4678	2.3449	1.72	2.1623	0.9584
1.9345	0.1876	1.1803	1.3136	0.7679	0.9952	2.877	0.4277
1.5253	1.4422	0.5665	2.0895	1.7121	1.2048	1.4237	1.1713
1.9825	1.8917	1.4792	0.4794	1.5328	2.6464	1.2026	1.818
1.3112	1.127						
ARHGEF2	5.9852	4.1843	4.5021	5.3389	5.5568	5.428	5.7711
3.9061	4.9201	6.0842	5.389	5.1785	5.7034	5.0481	4.7584
6.1719	6.1512	5.4201	5.3804	5.5065	4.9441	4.7614	5.4804
5.4388	6.6758	6.4843	4.6185	4.0857	5.2257	3.6165	3.3365
4.3681	5.3124	5.6149	4.5412	4.5249	7.8832	6.3047	6.6955
5.2338	6.9863	5.2583	5.7562	5.171	4.6434	6.3753	5.5506
6.2226	4.9224	4.711	5.1872	5.7255	5.7192	8.0808	3.5258
4.787	5.5013	4.0488	5.3129	4.8585	5.885	5.2643	5.6255
5.205	4.1322	4.9613	4.6545	5.5607	6.2767	5.3754	4.3429
6.2781	5.0357	5.5866	5.4415	5.7158	5.1167	4.619	6.1011
5.816	5.6333	4.8507	6.2919	5.704	5.6981	5.1119	5.0423
5.2805	4.3932						
ARHGEF25	2.2629	2.2389	2.588	2.4057	2.8017	3.0866	3.0667
2.4813	2.4378	2.2434	3.3364	2.4634	2.0992	2.1885	5.7485
2.5362	3.0081	2.6412	1.3435	2.5548	1.5876	2.8371	3.145
1.4929	3.3252	6.2131	2.2555	2.4277	2.2849	2.7981	3.1181
0.3693	2.0102	2.5601	2.4947	1.7457	2.6503	2.6203	2.3705

2.2529	3.0048	3.9451	3.1285	2.6069	3.182	2.091	2.4265	
2.8808	3.7444	3.0374	2.5857	2.9077	2.2054	2.4038	3.2229	
1.2473	1.2696	2.5597	1.8458	2.319	2.9428	1.3197	2.6663	
2.6246	2.5532	3.0134	3.8348	3.6186	2.4286	2.5193	3.3351	
2.7307	2.522	2.9765	3.3175	5.0295	1.2852	2.1449	2.2876	
3.6719	2.217	3.8013	1.7211	2.9995	2.1019	2.2857	2.0261	
1.936	2.1046							
ARHGEF26	2.0483	2.1649	2.152	2.6563	1.7572	2.2212	1.6509	
1.9916	2.3869	2.2005	2.0869	1.5246	1.2583	1.779	3.7623	
1.7677	0.9778	0	2.1167	2.1064	2.4376	2.6732	1.996	
2.352	1.3016	1.0947	2.5536	2.0229	3.6417	2.352	1.732	
1.3838	2.343	2.3612	2.0106	3.3782	1.716	1.9321	2.181	
2.4248	1.7652	0.4258	1.2916	2.5776	3.0641	1.4283	2.4731	
2.6169	2.1796	2.1016	2.3381	2.7812	2.9711	2.3234	2.4712	
2.8445	0.5426	3.1341	1.6935	3.5438	2.6773	1.9662	0.7995	
2.6152	1.9237	1.3162	2.2796	0.4521	1.9551	1.5704	1.8315	
2.7884	3.0117	2.5984	2.1526	1.205	1.7479	1.9058	1.4605	
2.4225	2.0675	2.3858	0.9424	2.5912	1.8527	2.7798	1.8133	
1.767	1.6902							
ARHGEF28	1.5295	0.0423	0.7562	1.8733	2.2385	1.8638	1.1389	
0.9552	0.5802	1.2319	0.8201	1.8789	4.0243	2.0848	1.7473	
1.5999	2.6886	1.0089	0.7231	1.5882	1.2359	0.7339	1.5587	
2.5469	1.7558	4.5201	1.4438	0.8912	1.8576	0.128	1.5081	
1.3807	1.0359	0.9644	1.701	1.5301	1.7108	0.0612	1.4232	
1.3414	1.4553	2.6076	2.6962	2.0371	4.2131	2.3632	1.7462	
0.5612	3.962	4.1028	1.5507	2.2399	1.5888	1.2118	2.1932	
0.6366	4.2506	0.4494	1.1503	0.5438	2.0849	0.6155	1.3468	
1.6083	0.4812	3.1959	0.2099	1.3046	1.2565	2.7213	2.2091	
1.4399	0.5515	2.688	2.076	1.6407	1.0571	1.0381	0.9567	
0.7696	0.8202	2.605	2.7649	0.9699	3.8575	1.6612	1.0201	
1.0249	1.295							
ARHGEF3	1.8812	0.9525	2.3222	1.8279	1.6733	3.9297	2.5953	
2.6092	3.2307	3.4086	3.245	3.6465	2.4675	2.4972	3.0043	
3.6514	2.0479	3.1195	2.9111	2.6148	1.9271	1.7683	3.9297	
3.3231	2.6579	1.1815	3.3662	1.7568	4.0199	2.9472	2.0566	
1.6461	2.9323	3.0141	2.9583	3.3009	1.4162	3.2738	3.6542	
2.6259	4.4533	2.989	1.8095	4.4373	2.3397	1.5121	2.7818	
2.747	1.6296	1.0396	2.1595	4.0795	3.5775	2.4051	2.5076	
1.2463	2.4542	1.9462	3.1097	1.6844	2.8019	1.9113	0.8863	
2.9956	1.7123	3.6944	2.9642	3.5076	3.3288	2.2325	2.8181	
2.2702	2.2166	2.2058	4.6892	5.4364	2.3004	3.3661	2.3453	
2.3352	1.1519	3.0141	2.8483	2.2259	2.7491	3.0643	2.413	
2.0097	2.2476							
ARHGEF33	0.1076	0	0	0.0471	8.00E-04	0.028	0.0214	0
	0.039	0.0068	0.0332	0.1849	0.0088	0.1263	0.0187	
0.0021	0.0505	0.0253	0.0318	0	0.0328	0.0023	0.0184	0
	0.0016	0.0382	0.1016	0.0235	0.0145	0.0465	0.2345	
0.0394	0.0106	0.06	6.00E-04	0.0965	9.00E-04	0.0248	0.8405	
0.0121	0.1297	0.1144	0.0164	0.028	0.0497	0	0.0735	
0.0322	0.0062	0.227	0.0139	0.0336	0	0.0787	0	

0.142	0	0	0.1453	0.0254	0	0	0.1796	
0.0873	0.0338	0.1012	0.247	0.0178	0.0782	0.1709	0.0488	0
	0	0.1149	0.0504	0.0238	0.0138	0.0371	0.0163	
0.0711	0	0.0103	0.0284	0.0406	0	0.0616	0.1165	
0.0891	0.1091							
ARHGEF35	0	0.3107	0.0658	0	0	1.6848	0.0391	
0.5396	1.0768	1.3924	0	0.0865	0	0.5594	0.2687	0
	0	0.0608	0.3408	0.0677	0.315	1.4909	0.0347	
0.0414	0.036	0.0415	0.1237	0	0.0642	0.1613	0	
0.1568	2.1667	1.6558	2.5915	0.9115	0.4966	1.7546	0.1679	
0.1744	0.5241	1.3084	0	0	0.0823	0	0.3792	
1.9928	0.0827	0	0.5199	1.7795	1.0489	0.7903	0	
0.3217	0.5485	0	0.2338	0	0.064	0.1961	0	
1.6859	0	0.4689	1.2294	0	0.9837	0	0	
1.272	0	1.8487	0.1994	1.6234	1.1538	3.137	1.2382	0
	0.1034	0.6288	1.2242	0.1233	0.0343	0.8956	0.7141	
0.1756	0.8287							
ARHGEF37	0.3544	0.0536	0.6423	0.8956	1.03	0.5715	0.5173	
0.1868	1.036	0.7078	0.4347	1.0207	0.6982	0.4702	1.2639	
0.4651	0.0994	0.1828	0.3366	0.1422	0.4949	0.8159	0.5779	
1.5555	0.9824	0.3056	1.8139	0.4839	1.313	0.1621	0.2272	
0.0324	0.4061	1.4258	0.7866	0.6098	0.3647	2.0635	0.1719	
0.4211	0.4945	1.5033	0.1859	1.3058	0.1851	0.079	0.1609	
0.1696	2.1975	0.0481	0.4536	1.4586	0.1849	0.1636	0.4933	
0.7874	0.9995	0.719	0.4646	0.5265	0.2806	0.2842	0.0848	
0.2188	0.5133	1.5019	0.0541	1.788	0.3224	1.6128	0.9529	
0.3841	0.2493	0.5884	0.2537	0.5806	0.1078	0.8755	0.3282	
2.2029	0.1226	1.93	0.3442	1.1873	0.9124	0.2013	2.2155	
0.1206	0.4053							
ARHGEF38	0	0.0121	0.0462	0.0233	0.0031	0.0215	0.0038	
0.0053	0.0094	0.0121	0.0023	0.0023	0.0125	0.0298	0.005	
0.0057	0.0235	0.0233	0.0441	0	0	0.0135	0.0215	
0.0189	0.0322	0.0134	0.0156	0.0275	0	0	0	
0.0556	0.0669	0.009	0.0441	0.0231	0.0021	0.0244	0.0049	
0.0089	0.0318	0.0015	0.0198	0	0.0141	0.0092	0.0188	0
	0	0.0624	0	0.0211	0	0.0057	0.0126	
0.0417	0	0	0.0097	0.0358	0.0091	0.0036	0.0123	
0.0722	0.021	7.00E-04	0.032	0.001	0.0391	0.0278	0.0166	
0.0333	0.0097	3.00E-04	0.0192	0.0196	0.0552	0.0086	0.0193	0
	0.0053	0.0069	0	0	0.0299	0.0348	0.0333	
0.0223	0.0285							
ARHGEF39	3.6865	2.9096	2.7093	2.6092	2.5098	1.7125	1.4593	
2.5838	3.2858	4.3445	2.2763	2.4788	2.5783	3.3695	2.5352	
2.1373	2.5629	2.6743	2.7257	2.6619	2.5219	1.7083	1.9077	
2.0389	2.4487	0.8686	1.7765	2.9151	2.715	1.8518	3.1354	
3.0526	3.8264	2.6158	1.924	3.933	2.1621	2.133	2.7684	
3.0706	2.1829	1.6467	3.5295	3.4544	2.8759	1.959	2.8735	
3.2423	2.1902	2.8043	2.2248	2.5852	2.5275	1.4837	3.2208	
2.8049	1.9761	2.6358	3.6886	2.7455	2.7358	2.8594	2.7451	
2.7354	2.8734	1.2995	2.7175	1.5181	1.9205	2.5462	2.3992	

3.1301	2.6724	3.3887	1.3155	1.399	2.817	1.8447	3.0471	
3.2954	3.0984	2.0114	1.2609	1.3311	2.3895	3.2319	1.9867	
2.8735	2.5521							
ARHGEF4	0.1171	0.9565	2.8062	1.4252	2.8878	0.1363	0.9727	
2.3046	0.3594	0.8024	3.5275	1.7124	1.1023	1.7156	0.4368	
1.4886	0.1222	2.6019	1.2439	0.4361	1.1538	0.4565	0.3161	
0.1818	1.1467	0.7661	0.7753	1.8744	1.5758	4.4699	2.0733	
0.4186	0.9005	0.126	0.0498	1.4379	0.0629	0.1285	2.1254	
0.2393	0.1046	1.541	0.3954	0.157	0.992	1.2208	1.1326	
0.5663	0.2445	0	0.5948	0.5674	2.9977	1.4004	1.4618	
1.6933	1.3396	3.1409	0.1577	3.3304	0.1261	0.6505	0.3721	
1.3344	2.2546	1.3038	0.2562	1.4704	2.5495	0.254	0.0341	
0.7322	0.4393	1.5937	0.1709	2.5225	0.7951	0.2069	0.0093	
0.0697	0.1515	1.2668	0.3106	2.3787	0.7552	2.6089	0.254	
1.1804	0.3017							
ARHGEF40	3.1066	3.4194	2.6903	3.0634	3.393	2.8786	3.5145	
4.2418	4.1258	2.6435	4.6709	2.9793	3.3687	2.9781	3.6523	
4.9765	2.5018	5.9019	3.2934	2.4813	2.7543	3.9154	4.3898	
2.4143	4.1407	3.5834	2.5347	3.5876	3.6363	3.2155	3.386	
4.4047	2.4655	3.9408	3.2478	3.1519	2.8488	3.4423	3.6229	
3.6963	4.0542	3.7575	4.3637	3.5598	2.9981	3.053	2.139	
4.0701	4.4371	2.8243	3.2896	3.4039	4.3969	3.5522	2.5047	
3.5108	3.0125	3.3519	3.9862	3.1555	3.8942	2.486	4.951	
2.9755	2.6743	4.4073	2.3469	3.854	3.5364	4.8082	3.3452	
3.9945	2.7852	3.8003	3.8177	2.5163	3.3697	3.6176	3.7695	
3.0348	4.5053	2.5195	2.654	4.7764	3.5885	4.6416	3.8458	
4.2611	3.7901							
ARHGEF5	0.054	0.0777	0.592	1.2151	0.0755	0.5107	0.0448	
0.5895	1.8802	1.9702	0.2617	0.0594	0	1.0475	0.8079	
0.0832	0	0.0693	0	0.0776	1.0134	2.5571	0.3306	0
	0.0578	0.1655	0.3568	0	1.5577	0.847	0	
0.0897	1.8125	1.1097	4.1656	1.638	0.6555	3.3591	0.0845	
0.0375	1.3625	1.6883	0	0.9544	0.3528	0.155	0.2452	
1.5161	0.4517	0	1.1426	2.1705	1.2201	0.7725	0	
0.787	0.7593	0	1.7867	0.1614	0.0718	0.3333	0	
1.3263	0	1.0427	1.388	0	1.4619	0	0	
1.4787	0	1.8352	0.4713	1.972	0.9511	2.9876	1.2931	0
	0.9291	0.8401	2.0734	0.0826	0	1.1725	0.9134	
1.0555	1.2022							
ARHGEF6	2.6968	0	0.8411	0.9061	1.9516	1.2817	1.9129	
2.2219	2.1935	1.817	1.3793	2.2734	1.3087	1.3771	3.3244	
1.5038	2.0477	0.0865	0.6246	2.1072	0.3044	2.8935	2.9191	
3.0701	2.767	1.3532	3.2979	0.094	3.352	0.3778	1.2763	
1.4376	0.0475	2.7487	4.2941	1.134	1.96	1.1157	3.2779	
3.1777	1.6169	2.0561	2.6045	3.0388	0.3037	2.645	2.8681	
0.2065	0.9306	2.696	1.5999	2.5134	2.1383	0.224	0.9531	
1.9783	2.581	0.2012	1.0037	0.2544	2.4721	0.3872	1.2835	
2.5039	1.344	2.6347	0.434	2.276	1.7758	2.2821	2.9244	
1.7435	1.6843	2.9092	3.2963	2.0818	0.8621	2.8607	1.8973	
1.6361	0.2217	3.4344	2.4593	3.2758	1.1107	2.1503	2.2137	

1.4721	1.7751						
ARHGEF7	4.3799	4.1676	4.4352	4.1502	4.1832	4.0865	3.9636
4.0685	4.1312	4.206	3.7143	3.5439	3.6553	4.5026	4.2172
3.3128	3.1735	3.9243	4.5784	3.4617	4.4342	3.9949	3.9159
4.0654	4.7073	3.7939	3.8381	3.8932	3.7742	3.6977	4.8243
3.0536	3.1196	3.3394	3.8624	3.9729	3.7939	4.421	4.4501
3.21	4.1801	3.2598	3.8116	4.2295	3.596	2.6184	4.0835
2.8356	3.6506	3.7604	3.6429	3.8985	3.9269	3.9753	4.1698
5.1694	3.7415	4.1353	3.6543	4.06	3.2194	4.1624	4.4457
3.6709	4.0742	3.0868	3.3525	4.0023	5.5021	4.7888	3.9974
3.8489	4.3147	3.5697	3.8258	3.7213	3.6828	3.6906	3.9725
3.3646	3.8157	3.7454	3.8901	4.2578	3.2757	3.594	4.2494
3.8522	3.8539						
ARHGEF9	2.5641	0.0563	1.9527	1.9016	2.6278	3.1564	1.6466
2.0879	2.3972	2.1297	1.9674	0.9398	1.9649	2.4411	2.1265
2.3463	2.3046	2.4149	1.8359	2.4446	2.1739	2.1251	2.7402
2.8103	2.5672	2.0655	2.4004	1.5704	2.4122	0.9203	2.639
2.5306	1.9297	2.6578	2.1621	1.9581	1.6344	1.8429	1.9073
2.5013	1.9492	3.1494	2.6112	2.7536	3.0611	0.0053	2.8134
2.0829	2.1261	4.0827	1.8653	4.2648	0.1017	2.6842	2.7352
2.1932	0.0773	2.2886	1.4087	2.2378	2.0454	1.1401	2.584
3.2238	1.8928	2.2898	2.6439	2.1319	2.0449	2.8974	2.6225
1.7859	2.2448	3.1918	2.6472	2.0106	1.5838	3.6164	1.5339
1.22	1.8666	2.883	1.8392	2.5419	1.6552	0.0814	2.9273
2.2867	2.2462						
ARID1A	4.0151	4.0329	4.4759	4.1885	4.6059	4.3423	3.8673
3.7631	4.6034	4.5858	3.9047	4.1838	3.9929	4.186	3.8975
3.7413	4.307	3.941	4.179	3.4973	4.1991	4.2234	4.0114
3.9479	4.3048	3.7328	3.2673	4.4124	3.893	4.3482	5.6237
3.9244	3.6668	3.7231	3.8837	4.1855	4.1509	4.3889	4.3162
4.302	4.1241	3.5974	3.9031	4.7339	4.5627	4.1577	3.8808
4.6298	4.066	3.4041	3.7021	4.3846	4.0522	4.4088	4.6208
4.2264	4.4542	4.5567	4.0838	4.9005	3.9941	4.1371	4.236
4.4865	4.3485	3.9106	4.6555	3.8071	4.9123	3.7962	3.0693
4.2264	3.5463	4.7559	4.0929	3.7839	4.4556	4.0696	4.2991
4.107	4.5366	4.1437	3.3538	4.1115	4.2976	3.8884	3.8295
3.8584	4.0072						
ARID1B	3.2373	3.4679	3.5184	2.8789	2.8466	3.1513	2.6527
3.2056	3.1146	3.602	3.0703	2.7618	2.3562	3.1631	3.1902
2.6711	3.7671	3.2722	3.8876	3.4508	3.5886	3.4105	3.1765
3.4525	2.3033	3.2452	3.137	3.144	3.2581	3.7293	2.9946
3.6432	3.1142	3.1708	3.2742	2.9854	2.4174	3.5203	3.6813
3.0381	3.8889	3.0198	2.5874	2.5482	3.2007	2.9859	1.8188
3.4706	3.0933	3.1252	3.3769	2.666	3.1365	3.8542	3.5337
3.8657	3.2824	3.3932	3.1429	3.3707	2.8708	2.9726	3.6771
3.0951	3.5602	2.5538	3.3164	1.0682	3.9887	3.1978	3.2587
3.5714	3.5691	2.6922	3.2263	3.0958	3.1277	3.3753	3.243
2.6342	3.6671	3.3272	3.1369	3.5477	2.7503	3.023	4.0447
3.7748	4.2481						
ARID2	2.7926	2.3574	2.6723	2.8849	2.6819	2.8176	2.2429

2.1961	2.8324	3.2824	1.9802	2.212	1.6835	2.5715	2.1613	
1.9234	2.6744	3.3548	2.9843	2.2522	2.8396	2.6116	2.1504	
2.404	2.7727	1.7767	2.8094	2.8514	2.4442	2.1991	4.8995	
2.3012	2.4312	1.8188	2.3149	3.4833	2.7161	2.6822	2.5758	
3.3609	2.0426	2.224	1.5447	2.9817	3.2165	3.2134	3.3082	
2.2707	2.6188	3.4205	2.1122	3.0073	2.8836	2.5152	3.9817	
1.9413	2.7578	3.0746	2.9674	3.1066	2.6367	2.8607	3.5739	
2.9067	2.7297	1.8732	3.0049	2.2823	3.0471	2.5711	2.488	
2.2678	2.582	1.5604	2.7161	2.3232	2.6063	2.6058	3.0956	
2.7498	2.8624	2.6285	1.4068	2.724	2.0796	2.5701	2.4275	
2.5753	2.8671							
ARID3A	1.4962	1.6548	1.8787	1.0709	1.5817	1.6396	1.4067	
3.0495	1.273	1.6462	2.7476	1.1131	1.7727	1.9129	0.9336	
1.9251	2.5596	1.3535	1.6562	1.5955	1.8928	1.8577	1.3296	
1.6488	1.1091	2.7635	1.9705	2.5288	1.0262	2.5002	3.3666	
1.1589	1.5475	1.3444	0.9316	1.9072	1.3489	1.7234	1.8089	
1.6818	1.4158	1.1131	1.979	1.7748	1.3509	3.088	2.3058	
1.5615	1.7582	1.4539	0.9165	1.5828	1.798	1.0904	2.9864	
2.3415	1.6657	2.4976	1.3057	1.8345	2.0653	2.1284	1.1608	
1.5485	1.9872	1.6537	3.2421	2.609	2.6078	1.1509	2.0301	
2.0582	1.8169	2.0297	1.1682	2.4574	1.9991	1.0118	1.5198	
1.7414	1.9909	1.0735	2.5766	1.0397	2.2511	1.3724	1.5524	
1.7472	1.6087							
ARID3B	2.0945	2.9677	3.0458	1.1399	1.3582	1.256	1.1085	
2.2349	1.4112	1.8877	2.2362	1.1572	1.0212	1.0756	0.4741	
2.0276	1.4073	1.839	2.8319	1.5469	1.7526	1.008	0.4623	
1.1985	1.1059	0.8537	0.8596	2.1788	0.4682	2.8181	3.4583	
2.3365	1.4291	0.6038	0.8853	2.3745	1.5497	1.263	1.1257	
0.3185	0.7747	0.5121	0.4371	1.2205	1.0929	1.1024	2.1039	
1.3731	0.9948	1.7157	1.1346	0.7473	1.0138	2.181	2.8911	
1.7361	1.3722	1.4927	3.0223	1.5542	1.2044	3.3641	1.523	
1.1999	0.594	0.7949	1.1622	1.0038	2.6415	0.5	0.8003	
1.1882	1.8757	1.8897	0.7462	1.0565	2.8409	1.2215	1.1963	
1.4383	1.6718	0.4293	3.3423	0.3796	1.07	1.231	0.5837	
0.5192	0.6993							
ARID3C	0	0	0	0	0.0286	0	0	0
	0	0.069	0.0814	0	0	0	0	0
	0	0	0	0	0.0912	0.0394	0	
0.0716	0	0	0	0	0.062	0	0.0805	
0.1827	0	0	0.0264	0.0601	0	0.0762	0.1453	0
	0	0	0.1785	0	0	0	0	
0.0839	0.0538	0.3636	0	0	0.1437	0	0.1435	0
	0.096	0.3483	0	0.0897	0	0	0.0321	
0.0583	0.1103	0.102	0.0701	0.097	0.0227	0	0.0343	0
	0	0	0	0.0348	0.0215	0.0385	0.0541	0
	0	0.0212	0.0689	0.0867	0.0638	0	0	
0.1438	0							
ARID4A	1.7482	1.787	1.934	2.365	1.5786	2.4855	1.9167	
1.9569	1.5567	1.8049	1.4451	2.0605	2.1271	2.4169	2.0635	
2.1949	2.1331	2.8398	2.3952	1.8899	2.2979	1.7123	2.5126	

1.515	2.5226	1.8153	1.7382	2.0999	2.1808	1.4043	2.5532
1.5424	1.9733	1.6405	1.6473	2.1231	1.8984	2.109	1.5446
2.3636	1.9965	1.7583	1.6184	2.66	2.5726	2.4328	1.8549
2.1593	2.4796	2.9441	1.686	2.0886	2.5719	2.0388	1.8684
1.6779	2.4103	1.4489	2.1167	1.663	2.2187	2.0461	1.9738
1.9574	2.6818	2.494	2.8301	1.9832	1.8438	1.6144	2.1602
2.2718	2.5381	2.4503	2.4519	1.9876	2.2296	1.7061	2.3675
1.9008	1.8375	2.6485	2.2744	1.9489	1.8136	2.3874	2.3818
2.292	2.562						
ARID4B	2.5172	2.57	2.6527	3.368	3.3795	3.0114	3.3643
2.8251	2.1877	1.9648	1.979	2.875	3.0943	2.875	3.3195
3.2257	2.5788	3.6273	2.5167	2.5467	2.5521	2.8839	3.3345
2.809	3.7204	3.5957	3.2779	2.615	2.9819	3.2204	3.0114
2.016	2.6371	2.7104	3.055	2.6107	3.646	3.115	3.7828
2.8898	3.1573	3.0435	3.3072	2.4256	3.4782	3.3748	2.4194
3.868	3.801	3.0222	2.9765	3.3413	3.3276	2.8438	3.2145
2.7138	3.3141	3.2266	3.2913	3.4647	2.8902	2.4534	3.4188
2.5694	3.127	3.1927	3.4268	3.3374	2.4884	2.9385	2.5502
3.0369	2.7537	3.4148	3.5967	2.6	2.9886	3.759	3.0002
1.6599	2.5589	3.2685	2.2011	3.4562	3.3746	3.1784	2.9465
2.8314	2.928						
ARID5A	1.909	2.261	2.1018	1.0879	1.386	1.8126	1.8634
2.3019	1.0534	1.715	1.6364	1.4273	2.1166	1.0109	2.187
1.5613	2.3177	1.3436	1.4698	2.9451	2.137	1.9352	1.8313
1.7189	1.6543	2.6663	2.3477	2.1328	1.5471	2.2993	1.0473
2.8749	1.8033	1.9321	1.2522	0.8798	3.1449	3.2532	2.5516
1.0579	2.4733	1.0132	2.2261	1.9825	1.4698	1.2049	1.2143
1.9868	2.183	0.9241	2.4539	1.0075	1.5986	1.8988	1.7931
2.3958	1.4697	3.9107	2.0634	3.8658	1.635	1.3965	1.9272
1.4981	2.6054	1.8946	2.6932	2.3684	1.0831	2.419	1.6498
2.252	2.2909	1.7524	1.7738	0.718	1.8198	2.0483	1.6544
2.0749	1.9881	1.6001	3.6441	1.4322	3.2428	1.8649	3.5614
3.1488	3.4684						
ARID5B	1.5806	2.74	1.8032	2.5676	1.779	3.3125	3.57
2.4146	1.6766	2.8446	1.8651	3.3125	3.2836	3.3347	3.2732
2.7405	1.683	2.5855	2.466	1.6889	2.4141	1.9758	1.9741
2.6819	3.1379	3.5714	3.3879	2.3575	1.9089	2.3765	0
1.4064	2.3268	2.9419	2.4497	2.0652	2.2961	4.0313	3.1139
3.2903	3.9974	3.1502	2.7284	2.4082	2.6436	2.606	2.9392
2.3858	2.0773	2.1614	2.0568	2.0532	2.6654	2.7365	0.2753
3.1364	2.5108	0.7523	2.0466	1.0917	2.288	1.5573	3.655
2.9101	3.9847	4.3894	4.3412	2.4627	2.3003	2.4839	3.1409
2.3717	3.0393	1.9831	2.4087	2.9218	2.8009	2.2372	3.0542
3.3099	2.0504	3.087	3.7925	2.2322	3.1308	2.0328	4.3411
3.2944	4.5969						
ARIH1	4.5472	4.7064	4.5136	4.0845	3.8761	4.3342	4.9691
4.5457	4.3978	4.9518	4.9269	3.8261	4.0661	4.1855	3.7379
4.6895	4.1015	5.3974	4.5092	4.0656	4.4496	4.2607	4.1616
4.2701	4.0462	4.0052	4.4264	4.4164	4.1161	4.626	3.8257
3.8462	3.9946	3.9066	4.4895	4.7383	4.3776	4.2859	4.6161

4.5005	3.756	4.1581	4.0387	4.2863	5.1985	4.5131	4.8648	
4.4841	4.1936	4.5553	4.3791	3.8061	4.5195	5.0207	4.3677	
4.3562	4.4881	4.078	4.1968	4.0489	4.0931	4.4305	4.6606	
4.8743	3.8892	4.5094	4.5482	4.697	5.571	3.9779	3.7785	
3.7903	4.0558	4.0543	4.1806	4.6975	4.7071	4.3722	4.4579	
4.6829	4.3587	4.0517	4.7743	4.129	3.8849	4.037	4.5782	
4.1729	4.3441							
ARIH2	4.6835	4.9714	4.8919	4.9141	5.079	4.94	4.8308	
5.5519	4.1625	5.3483	5.0399	5.4113	4.8113	4.7581	4.0233	
5.2538	5.2474	5.0355	5.006	4.9231	4.6748	4.8743	4.8443	
5.4506	4.6132	4.5292	4.2687	4.7763	5.1144	5.2587	4.5556	
5.495	4.3475	3.6818	4.6651	5.2764	4.9418	4.8977	5.3654	
4.9226	4.7714	4.6544	4.4567	4.5199	5.3778	4.0279	5.3115	
5.3795	4.9541	4.3729	4.6784	5.1302	4.5806	4.8232	4.8721	
4.4461	4.3289	4.6938	4.9585	4.6025	4.9114	5.132	5.3613	
4.6095	4.974	4.3483	5.5026	4.3307	5.2738	4.135	4.6215	
4.357	5.1647	5.3879	5.059	4.5747	4.6086	5.3256	3.889	
4.4117	4.482	4.9633	4.4682	4.5724	4.5705	4.3913	4.1379	
4.3761	4.4256							
ARIH2OS	0.7772	0.7215	0.9467	0.7273	0.9714	0.6447	0.4438	
0.4243	0.3206	0.9462	0.9177	0.8869	0.9645	0.5932	0.5885	
0.4964	1.2206	0.416	0.3182	1.0386	0.4172	0.6167	0.4446	
0.696	0.3559	0.5062	1.7272	0.9115	0.4388	0.8461	1.0908	
1.2978	0.6467	0.5852	0.5166	1.0297	0.9364	1.7761	1.6535	
0.8702	1.0702	1.0197	0.6033	0.3292	0.916	0.4066	0.972	
1.1976	0.6493	1.4444	0.544	0.6482	0.9986	1.001	0.7223	
0.7276	1.0168	0.2298	0.3826	0.2856	0.5377	0.7487	0.8771	
0.9296	0.9808	1.6361	1.2632	1.0237	1.0834	0.3907	0.5317	
0.5192	0.7676	0.4311	1.0192	0.1845	0.8572	1.0819	0.7282	
1.1366	0.4615	0.4931	0.7338	0.7548	0.8287	0.7235	0.5287	
0.5345	0.7723							
ARL1	4.8682	4.3784	4.824	4.6694	4.5161	4.6331	5.9209	
5.2848	5.229	4.9307	5.0423	4.1966	3.4084	4.7632	4.5116	
5.1948	4.4366	5.8742	5.5285	4.5397	4.8647	4.2646	4.4241	
4.3065	4.4509	5.1558	4.4266	4.8015	4.2666	4.7422	4.9076	
4.7138	4.9291	5.1022	5.2091	5.1382	4.6444	4.8673	3.6355	
4.9335	4.218	4.7816	4.9072	4.5828	4.7353	4.7776	4.8358	
4.7667	5.0223	5.2475	5.0948	4.5493	5.2329	4.5512	4.8311	
5.1923	5.2712	4.7817	5.0311	4.2245	4.2128	5.175	4.3468	
4.6441	5.2479	5.6203	4.9397	5.1943	4.7822	4.2995	4.4898	
4.5837	4.7602	4.3642	4.553	4.3674	4.8768	4.2892	4.2302	
4.8768	4.8272	4.4986	5.4319	4.9065	4.2311	5.1307	4.8388	
5.0044	4.9426							
ARL10	0.6217	0	0.0536	1.3512	0.4316	1.7356	0	
2.0236	0.0693	0.9283	0.9678	1.6339	0.9928	1.9804	1.938	0
	2.5661	1.57	0.0895	0	0.602	0.492	0.8913	
0.0244	1.9712	1.7735	0.8372	0.0563	1.6452	1.5443	0.0858	
1.2639	0	0.3944	0.0806	0.8588	0.9789	0.1318	1.2913	0
	1.6712	1.2743	1.8836	1.4734	1.8057	2.8058	0.5846	
0.8342	0.1977	2.4119	1.5232	2.5545	0.0666	1.2484	0.1818	

0.1808	0	1.0346	1.5661	0.7721	0	0.7872	1.5134	
0.5872	3.128	0.6429	0.8729	2.4024	1.8402	1.7898	1.0545	
1.7793	0.0832	1.3718	2.1893	1.8446	1.3894	0.1168	0.31	0
	0.0606	2.6492	1.6989	1.9206	1.4413	0.1315	1.7839	
1.9034	2.1061							
ARL11	0.0435	0	0.0241	0.0247	0.0022	0.022	0.0116	
0.006	0.0338	0.1259	0.0026	0	0.0126	0	0.0038	0
	0.0251	0.0324	0	0	0	0.0054	0.0022	
0.0503	0.0088	0	0.0219	0.0451	0	0	0.0056	
0.0123	0	0.0544	0.0069	0.0455	0	0	0.0056	0
	0	0.0084	0	0	0.0106	0.0106	0.0313	
0.0411	0	0	0.0095	0.0129	0.03	0	0	
0.05	0.0675	0	0.033	0	0.0364	0.0072	0	0
	0.0427	0	0	0.0016	0	0	0	
0.011	0.0165	0.0292	0	0.0377	0.0256	0.0154	0.0038	
0.0564	0	0	0	0.0187	0	0	0	
0.036	0.0281							
ARL13A	0.0508	0	0	0	0	0	0.0284	
0.0244	0.0621	0	0.0281	0.0056	0	0.0724	0.0653	0
	0.1055	0	0.1551	0	0.0797	0	0	
0.0155	0.0289	0.043	0.2024	0	0	0	0.0136	
0.0353	0	0.0303	0	0.0612	0.0337	0.0576	0	
0.0195	0.0462	0.0672	0	0.0506	0.0619	0	0.0802	0
	0.0832	0.1094	0	0.2327	0.0516	0.1403	0	
0.133	0	0	0	0.1362	0	0	0	0
	0	0	0	0	0.0915	0.1025	0	0
	0	0	0.1007	0	0.0348	0.0796	0.0353	
0.0719	0	0	0.0704	0	0	0	0.125	0
	0							
ARL13B	2.5453	2.6324	2.7311	2.7326	3.1145	2.8793	3.0686	
2.8742	3.0853	2.2412	2.8395	3.1273	3.4356	3.3264	2.9382	
2.5098	2.9607	2.98	2.6092	2.6107	3.822	2.7777	2.7709	
2.1761	2.7347	1.6272	1.4873	2.4059	3.4446	2.1431	2.5025	
2.5009	2.2698	1.9548	2.5953	3.0886	2.3824	2.0556	2.9708	
2.8751	2.093	2.4569	2.6066	2.38	3.2707	3.4266	2.7373	
3.027	3.1066	3.3227	3.2033	2.9015	3.0027	2.7276	3.3848	
4.0335	2.5411	2.3125	2.4495	2.7388	3.0717	2.6984	1.7429	
3.321	2.5913	2.3926	4.3113	1.8944	2.836	3.0425	2.7279	
3.396	2.9531	2.6435	2.4895	2.1953	2.7984	2.4477	2.2693	
2.8519	2.8283	2.343	2.0456	3.3342	3.088	3.3439	2.286	
2.3873	2.3212							
ARL14	0	0	0	0.2715	0.1134	0	0.1101	
0.0478	0	0	0	1.9516	0.0914	0	0.0413	
0.0238	0.8268	0	0	0.0581	0	0.0694	0.0158	0
	0.0196	0.4856	0.0316	0	0	0	0	
0.5529	0	0.0324	0.0476	0	0	0	0	0
	0	0.0925	0	0	0.0409	1.498	0	
0.0758	0.0345	0.3684	0	0.1466	0	0	0	0
	1.7789	0	0.0666	0	0	0.0242	0	0
	0	0.2603	0	0	0	0	0.4191	0

	0	0.1261	0	0	0	0.0711	0	
0.0589	0.0708	0.1933	0.3748	0	0.212	0	0	0
	0							
ARL14EP	3.3426	3.6391	3.4993	3.5027	3.0119	3.3799	3.8221	
3.5158	3.4413	2.9234	3.6682	3.0823	3.4905	3.2616	3.9325	
3.4205	3.7067	2.9582	3.7967	3.3661	3.3406	3.9377	3.2839	
3.3918	3.6304	3.8834	3.6575	3.885	3.3587	3.2108	2.5854	
3.1573	3.3166	2.6173	3.1168	3.1156	3.5195	3.6469	3.5561	
3.6593	3.3787	3.3921	3.6614	3.4484	3.1522	3.2597	3.6318	
3.7312	3.9925	3.7774	3.45	3.9108	3.5849	3.843	3.2496	
2.9804	3.5147	3.4248	3.5767	3.6281	3.3745	3.4321	3.892	
3.2241	3.2443	3.9264	3.1486	3.5717	3.6478	3.1264	3.2553	
3.1548	3.5406	4.0316	3.6654	3.8352	3.2765	3.1894	3.5908	
3.7548	2.8704	3.5833	3.2604	3.2409	3.0921	3.2861	3.3073	
3.6171	3.2176							
ARL14EPL	0	0	0	0.423	1.8801	0.2286	0.931	
0.2816	0	0	0	0.4928	0.1535	0	0	
0.2692	0.707	0	0	0	0.1328	0	0	0
	0.2218	1.3107	0	0	0	0	0	
0.1947	0	0	0.033	0	0.4164	0	0.1556	0
	0	1.0528	0	0	0.343	0.3608	0	0
	0.3655	0	0.13	0.0686	0	0	0.2463	0
	1.2654	0	0	0.1352	0.2748	0	0	0
	0	0.0588	0	0	0	0.479	0	0
	0	0.0882	0	0	0	0	0	0
	0	0	0.6069	0	0.9107	0	0	0
	0							
ARL15	2.1917	1.3048	1.4683	3.0434	3.1002	3.3293	2.7125	
2.2662	3.2106	2.3308	2.49	3.3363	2.8058	3.4594	2.7522	
3.4482	3.144	1.3826	3.0487	2.7552	2.6703	2.7198	3.4538	
3.1788	2.8964	2.8828	3.082	3.1262	2.8811	2.5914	3.181	
2.8554	4.0218	2.7198	3.2349	2.7989	2.4101	3.272	1.8708	
3.5854	3.2825	2.381	3.0502	2.8184	2.8452	2.467	2.6522	
2.569	3.6161	3.0454	2.4444	2.304	3.0347	2.2616	2.4442	
2.5173	2.8168	2.5083	2.1976	2.8065	3.0639	2.228	2.5539	
3.2416	2.2119	3.3197	2.8747	2.7482	3.1267	3.999	3.1573	
2.7213	2.0207	3.6782	3.1197	2.3494	2.2765	3.4839	3.5332	
3.5382	2.2534	3.3849	2.2212	2.6346	3.5616	2.7623	3.0556	
2.655	2.9272							
ARL16	4.0843	4.1228	4.297	3.3885	3.9329	3.701	4.0426	
3.8962	3.5555	3.643	4.2163	3.2129	3.8203	4.4452	3.9635	
3.7062	3.9639	3.6184	3.7662	3.9398	3.3452	4.0664	3.7306	
4.4131	4.4929	4.3212	4.7871	3.9234	3.4316	3.3595	3.8883	
4.4899	3.9956	3.6163	3.8986	4.0965	4.8242	3.7086	3.7942	
2.9782	3.3983	4.0718	4.117	3.2727	3.731	3.5492	3.1261	
3.8357	3.884	4.9458	3.4964	3.9076	3.7346	3.8654	4.3968	
4.1841	3.7308	4.1736	3.7172	3.8466	3.8147	2.7324	4.0817	
3.8009	4.371	3.349	4.2249	4.2117	3.7752	4.1552	4.2909	
4.1024	4.5447	3.5142	3.6496	4.4596	4.1603	3.691	3.1389	
3.4791	3.7289	3.8309	3.9894	4.4246	4.693	3.9529	3.0945	

4.0213	4.0871						
ARL17A	1.3196	1.8561	1.3119	1.1323	0.791	1.3019	0.9948
0.6311	1.3609	0.6663	1.1503	0.4698	1.0537	1.2555	1.0949
0.6143	1.2942	1.086	0.9333	1.2648	0.5815	1.0085	0.1469
0.8353	0.8456	0.9028	0.4204	1.1552	0.6725	1.631	0.6988
0.8253	0.8999	0.6725	1.3031	0.4989	1.3161	0.9756	1.3667
1.8824	1.1474	1.0524	0.5536	1.1341	1.1616	1.4666	0.829
1.049	0.6979	1.791	0.9973	1.4596	1.0237	1.8043	0.6998
0.711	0.512	0.6607	0.9021	0.8032	1.2907	1.2203	0.706
0.9889	1.069	0.5679	1.0051	0.8695	0.6055	1.3524	0.8236
0.5108	0.334	0.562	1.158	0.5507	0.7613	0.8725	1.0564
1.2869	1.1172	0.6453	0.7538	1.6532	0.7765	1.1212	0.8234
0.4472	0.5536						
ARL17B	1.0439	0.9597	1.2095	1.2035	0.436	1.3349	0.8893
0.7373	1.0413	0.955	0.7042	0.5162	1.3211	1.1518	1.0738
0.8434	1.2768	0.8632	0.9132	1.5995	0.975	0.622	0.338
1.259	0.6775	1.2354	1.529	1.3324	1.0822	1.0912	0.9518
0.7851	0.8411	0.9396	1.7179	0.524	1.0509	1.084	0.5133
1.4067	1.1329	0.8632	0.6739	0.6891	1.4284	1.5731	1.0485
0.7851	0.6652	1.8452	1.1028	1.1449	0.8411	1.3832	0.8078
0.9278	0.6815	0.7375	0.5817	1.0422	0.7664	1.0675	0.759
1.3666	0.9208	0.6215	0.5894	0.7526	1.6184	1.0347	0.6565
0.995	0.7721	0.4819	1.413	1.6614	0.5426	1.0176	0.8912
1.1939	0.982	0.4777	1.1453	0.9498	0.5827	0.8524	0.7226
0.5573	0.6351						
ARL2	5.9963	5.5139	5.6906	5.0435	6.296	5.8392	4.8546
6.5476	5.9249	5.5666	6.228	5.5478	5.1009	5.4251	6.1968
5.3338	6.2897	5.5767	5.0424	5.8179	5.9771	6.6048	6.0248
5.4973	5.609	4.6479	6.0857	5.4175	6.4361	5.4215	6.3293
5.2938	5.4811	5.7325	5.6275	5.0078	6.1029	6.1399	6.2219
5.2146	6.1695	5.5101	5.5095	6.0984	6.2775	5.5806	4.8026
5.9963	6.8716	6.0568	6.2331	5.2759	5.3641	6.1675	6.6543
6.3714	5.6247	6.3901	4.9489	5.589	5.8675	5.5738	6.0626
6.0765	5.4128	5.6366	6.1435	6.7728	4.9339	5.3627	6.2669
5.5761	5.427	5.5851	5.9542	6.3483	5.8213	5.505	6.2432
5.9121	4.9063	6.6424	6.1951	6.0459	6.0011	5.9084	6.7504
6.4937	6.6237						
ARL2BP	5.6641	5.1	5.3042	5.2967	4.6707	5.4296	5.6447
6.0604	5.3801	4.8671	4.5213	4.7711	5.9477	5.9182	5.3232
4.9206	4.2021	5.2737	4.87	6.5491	5.897	5.1	4.3704
5.1962	4.7753	5.2274	5.1444	5.4016	4.5102	4.9169	5.6429
5.0336	4.5478	5.3064	5.7724	5.0932	5.2648	4.7699	4.7689
5.5232	4.7776	5.4436	4.5778	5.2195	6.3131	5.6238	5.7255
4.9491	4.7764	4.4918	5.4535	4.8656	4.843	5.308	6.0864
4.6064	5.7368	5.0514	5.5025	5.2793	5.245	5.6867	5.3539
5.6146	4.8136	4.726	3.9907	5.8339	5.3805	5.5412	5.3272
4.8105	4.7604	3.8054	4.6863	5.5613	5.12	5.3459	5.5359
4.712	4.6292	5.1055	4.88	5.6644	5.0075	4.8895	5.0017
5.2415	4.792						
ARL3	2.1518	3.4118	2.5449	3.1657	3.2762	2.7216	2.1965

2.6428	2.8478	2.3393	2.3968	3.0091	3.4533	2.3398	2.6975
2.1093	2.9123	2.3225	2.3616	2.9613	2.5062	2.1792	2.464
2.4472	2.9184	2.5698	3.311	3.0216	1.6353	2.554	4.5979
2.6179	1.9788	2.7094	2.1017	3.4043	2.322	1.6054	2.0472
2.0089	2.3154	1.986	3.3385	1.9595	2.5601	3.5418	2.6209
2.3019	2.1917	3.9019	2.6681	2.5151	2.6512	1.9516	4.1782
2.1371	2.251	2.4306	2.2649	2.7852	1.9691	3.0946	2.5881
2.6347	2.439	3.3602	2.8161	3.1632	2.2024	2.9231	2.7951
2.5446	1.7029	1.7929	2.2074	2.7937	2.5276	2.6015	1.6094
2.208	2.4985	2.8986	2.6401	2.3348	3.6551	2.9301	2.9138
2.7409	2.8241						
ARL4A	3.3943	2.5227	3.6565	3.3038	3.2256	2.4421	2.6391
4.2972	3.0771	1.9677	2.8972	3.1811	4.4212	3.4641	3.8722
2.7567	2.9881	3.172	2.9952	4.5376	3.7505	3.3058	3.4113
2.0224	4.3019	3.699	2.611	3.8709	4.0638	2.4811	4.0183
4.2422	2.5125	3.4432	4.015	2.9138	3.6699	2.1114	3.7806
2.5283	3.8297	1.9837	2.7298	2.6901	2.9715	3.7626	4.0881
3.1105	3.0479	2.7749	4.2737	3.6146	5.1225	2.6831	3.0448
2.2597	3.2004	2.9375	3.2868	3.0908	3.378	3.7602	2.956
2.4572	3.1067	2.6204	4.132	1.9414	2.156	3.9703	3.129
2.8018	2.9524	2.9753	2.3747	2.3921	2.0278	3.446	2.9414
2.4113	2.3514	3.7232	4.1155	3.4007	3.9775	4.6876	2.9243
2.9403	3.2557						
ARL4C	1.8578	2.8209	2.3713	2.6584	3.6715	2.8578	5.1979
3.8548	2.6893	2.0157	3.6966	3.8257	5.2213	2.3559	3.9871
3.9617	5.385	4.6812	2.827	3.6816	2.3814	3.0269	3.5935
2.746	4.2456	5.9669	5.5023	1.2509	4.0146	3.8241	0.0444
3.0947	2.7709	3.2776	4.4135	2.2392	1.0109	2.1021	2.8543
2.8186	1.8259	5.8918	6.3231	3.8886	4.6739	5.0708	1.4566
2.539	4.8444	4.4969	3.4045	2.556	2.3947	1.8862	0.334
2.5336	6.3275	3.9508	4.0178	5.2087	3.5404	3.642	1.6418
2.9195	2.8153	6.1132	0.956	5.994	2.2871	4.0828	5.8177
3.8195	2.6663	2.7674	5.1556	2.4266	2.1017	4.637	2.0726
2.6102	2.512	6.302	3.5867	3.8732	3.8662	2.2098	5.9187
5.0193	5.8547						
ARL4D	3.5554	2.5904	2.2533	2.4002	3.4297	1.7527	1.8028
1.2375	1.897	2.1133	3.1346	2.6694	4.2272	2.476	2.2494
3.017	1.99	2.3027	1.3589	2.697	1.6968	2.7429	2.3669
1.7794	2.8116	2.6892	4.0579	3.1757	3.1874	2.4025	3.3359
1.4108	2.5212	3.1939	2.0685	2.7918	3.1242	1.9967	2.2616
1.1342	1.8547	3.6352	3.6163	3.4063	1.332	3.4038	2.4976
1.9983	1.5217	3.74	2.5528	2.8726	2.0947	2.3711	2.57
2.064	3.7571	2.14	2.2766	2.3297	3.551	2.7406	0.9557
2.1275	1.9256	4.573	2.5939	2.6473	2.8163	2.4269	2.4494
2.9747	2.1101	3.5519	2.8453	2.6522	3.1685	2.646	1.1077
1.6873	1.7139	2.0809	2.2307	1.6015	3.6224	2.4691	2.4123
1.909	1.7776						
ARL5A	4.1907	4.0964	4.7425	4.8853	3.9754	4.7979	4.5282
5.3918	4.545	4.668	4.6834	4.6713	3.6207	4.7291	4.8875
4.4272	4.403	4.8106	5.3954	4.7053	5.0157	4.1887	4.6698

4.9166	4.4979	3.3017	4.4335	4.4236	4.2951	4.2726	4.2838	
4.61	4.3437	4.7253	4.3664	4.9013	4.1098	3.8781	3.5719	
4.9861	4.3218	4.2118	4.6788	4.4687	4.3284	4.2292	4.6519	
5.5464	4.6297	4.8446	4.5658	4.5898	4.7817	4.5226	4.6887	
4.3835	4.0346	4.9474	5.4351	4.9734	4.0586	4.6726	4.0907	
4.2657	5.514	4.1248	5.0319	4.3276	5.0233	4.5339	4.741	
4.8376	4.4317	4.3942	4.2441	4.9385	4.7297	4.5211	4.9843	
4.6176	4.5922	4.2404	3.9126	4.3412	3.6904	4.6408	4.3781	
4.6213	4.4339							
ARL5B	2.5678	1.7851	3.766	3.5664	3.3607	2.3633	3.9245	
2.9949	3.2132	3.5499	2.8229	3.6342	3.1185	3.2354	2.706	
2.7736	2.9579	3.113	3.1386	3.0662	3.4443	2.8346	2.3594	
2.9454	3.3184	1.8419	4.0146	3.4394	2.4954	2.4521	4.0524	
3.3159	2.4288	3.3542	2.6	3.7435	3.1522	2.0395	2.96	
2.5333	3.0289	3.0654	2.3451	3.5873	3.2536	3.1145	3.1842	
3.2651	2.6785	3.231	4.435	2.6366	2.6012	3.198	4.4058	
3.1199	2.934	3.3163	3.3219	3.0949	2.3903	2.7928	3.2805	
3.1462	3.064	3.0896	3.5171	2.9178	3.6877	2.8806	2.3967	
3.0669	2.4358	3.0803	2.5364	2.7712	3.5142	3.0305	2.788	
2.7999	3.0346	3.0213	4.5769	2.9813	2.2377	2.0349	2.5928	
2.9308	2.742							
ARL5C	0	0.1071	0.147	0.2402	0.0574	0.0704	0.031	0
	0.0348	0.1198	0.0777	0.0597	0.0288	0	0.0266	
0.0292	0.0874	0.1981	0	0.0782	0	0.0198	0.0585	
0.0446	0.0929	0.0316	0.1899	0.1801	0	0.0459	0.0806	
0.0178	0	0.0912	0.0628	0.0334	0.0538	0	0.0463	
0.044	0	0.0341	0.1165	0.0491	0.0286	0.0436	0	
0.0978	0	0	0.0405	0.0477	0	0.0261	0.3013	0
	0.1054	0.0459	0.0448	0.0896	0	0.033	0	
0.0564	0	0.0757	0.1434	0.0145	0.0282	0.0771	0.0334	
0.1776	0.0442	0.0243	0.0334	0.0872	0.0234	0.0402	0	
0.0732	0	0.025	0.0668	0.0849	0.1478	0.0858	0	
0.0629	0.2161							
ARL6	1.8398	2.0916	2.5404	2.3961	3.1408	2.5333	2.7741	
2.7882	2.2223	1.352	2.5691	2.4934	2.2742	2.3456	2.9431	
2.7231	2.3855	2.3693	2.1265	2.9973	2.4094	2.4773	2.0413	
2.0955	1.9847	2.3214	1.8825	2.398	2.2534	1.8986	2.5352	
2.9602	2.1126	1.7198	1.7484	2.2002	2.2944	1.9412	1.9098	
1.948	2.0388	2.4634	1.9787	1.3205	2.89	3.4317	2.6331	
2.3725	2.1645	2.4243	2.7703	2.8297	2.637	2.4435	3.2269	
1.9973	1.9548	1.8815	1.8211	1.8604	2.0751	2.3489	1.4502	
2.7478	1.8409	2.3035	2.7308	2.3275	2.308	2.2024	1.889	
2.9292	2.627	2.4134	2.1595	2.905	2.6533	2.4402	1.8707	2.4
	1.8562	2.16	2.3431	2.5672	2.3558	2.5661	2.34	
2.1259	2.2434							
ARL6IP1	6.6338	6.9965	6.7288	7.4509	6.4254	7.4699	6.7288	
6.9451	7.4886	6.8581	6.133	6.3716	6.9868	6.8747	7.4341	
5.8751	6.101	8.4767	7.6223	6.5092	6.7461	7.1265	6.6338	
7.3767	7.0241	5.8231	6.8151	7.458	7.4101	6.7854	7.3416	
6.7481	7.3839	7.5278	7.7747	7.0624	6.4374	5.6824	6.1368	

7.1441	6.168	6.4463	7.0296	6.7224	7.2038	7.1472	6.8016
6.9087	6.865	7.0187	6.7741	6.0863	6.5692	5.8131	6.7608
6.5124	7.0197	7.7506	6.8785	7.854	6.5096	6.3053	7.1358
7.6785	7.162	6.3312	6.1711	5.9647	7.5143	7.787	6.498
7.116	7.0219	6.2804	7.4791	6.7623	6.8765	6.6945	7.0219
7.0796	7.2071	6.1638	5.7441	6.5155	7.0152	6.3115	7.0478
7.6005	7.3176						
ARL6IP4	5.5946	5.8409	5.7578	4.7876	5.844	4.6832	5.1916
6.0663	4.9742	5.0983	5.4051	4.6433	6.0098	5.0543	5.1362
5.0246	5.7935	5.0154	5.4089	6.1134	5.8008	5.9597	5.3937
5.5502	5.6315	4.9484	5.7505	5.6729	5.2945	5.7806	5.218
5.9133	5.7233	5.6551	2.7204	4.5609	5.555	6.1509	5.7447
4.7323	6.3222	5.5556	5.3958	5.726	4.7694	5.0219	4.4163
5.4122	6.0959	5.0094	5.1778	4.6518	5.0548	5.5361	5.8485
7.0824	5.4301	5.6384	4.9092	5.0612	5.7515	5.7129	5.8891
4.8954	5.6302	5.2391	5.7076	5.4726	5.3434	5.6955	4.9759
5.1602	5.647	5.1858	5.055	6.5628	6.1016	5.4693	4.4808
5.9677	5.4067	5.9148	5.8991	5.8108	6.0379	5.7547	5.7776
6.059	5.957						
ARL6IP5	4.996	5.0019	5.6416	6.1637	5.9525	6.5739	6.2322
5.7906	5.8896	5.9525	6.2748	6.8001	5.3244	7.1026	6.2835
6.8494	5.7916	6.7718	6.3716	5.8591	6.3096	5.5552	5.9059
7.0539	5.1434	5.1195	5.9977	5.6818	5.6438	5.9856	4.8938
6.1993	7.5562	9.0353	6.058	6.2146	5.6062	6.1596	5.3738
6.6488	6.308	7.0692	6.7524	6.7566	6.7874	5.631	6.2914
5.9219	6.5012	5.0891	8.1697	6.136	7.9797	5.0378	5.2951
4.6364	6.256	4.6048	5.8024	4.3028	6.1556	5.3533	5.7718
6.9239	5.8634	6.731	7.0006	7.0166	6.1684	5.8191	6.522
6.7903	6.1322	6.3164	7.0048	5.9861	5.5298	6.2415	7.5382
7.4725	7.0048	6.6475	6.0263	5.6686	5.9942	7.7351	6.622
6.1637	6.2366						
ARL6IP6	3.5477	3.5302	3.4595	4.3618	3.2974	4.0493	3.6056
4.6245	3.784	3.362	3.8093	3.6852	3.9788	4.5108	3.6258
3.0834	3.9874	4.2798	4.33	3.6663	4.3948	3.6337	3.7432
4.2268	4.2807	3.0302	2.4975	3.9678	3.7679	3.9856	4.7071
3.7448	3.8833	3.7804	3.5441	3.9988	3.5239	1.5625	2.6958
4.0788	3.1504	3.3347	4.9375	4.712	4.9448	4.2186	4.2722
5.2934	4.0241	4.7884	4.5069	4.3743	4.5821	3.3612	3.9609
4.196	3.8592	4.9404	3.8643	4.9251	4.2792	3.4088	3.0369
3.8702	3.7493	3.4104	4.3624	2.0793	4.4308	4.4726	3.5948
4.2753	3.8877	3.3981	3.2829	2.7101	3.9013	2.5174	3.9308
3.7806	4.0444	3.031	3.3592	3.472	5.0282	5.0915	3.091
3.5004	3.2854						
ARL8A	4.9003	4.1698	4.7895	3.8023	4.0702	5.7858	4.61
4.5242	3.9677	4.3029	4.7317	4.0157	4.1236	3.7549	4.7433
5.0241	2.8861	4.8987	3.5546	4.596	4.6703	4.2195	4.6552
4.3293	4.6986	4.3088	3.9003	4.7264	4.1459	4.3353	4.4539
3.6396	3.5422	5.0929	4.5635	4.0556	5.1827	4.9263	5.6401
2.8491	4.9423	3.8163	4.0442	3.5934	4.4669	3.2446	4.036
4.0828	4.4076	2.8237	4.535	3.7322	4.0824	4.2914	4.3147

4.1987	4.4116	4.819	4.3364	4.9345	4.0264	3.7832	4.3745	
4.0826	4.7645	4.9856	4.9744	5.3485	5.2681	4.3013	3.6518	
4.383	4.3918	2.8993	5.0967	4.5528	4.7658	5.1815	3.8618	
3.7667	3.5045	4.3884	6.2024	4.9906	4.7722	4.048	4.8815	
4.7278	4.8885							
ARL8B	5.3438	5.5005	5.885	5.3643	4.889	5.6416	6.2062	
5.3172	5.3438	5.7285	5.9468	5.6043	5.4432	5.9668	6.2302	
7.271	5.8704	6.394	5.7993	5.1052	5.6155	4.9258	5.1052	
5.2583	5.2201	4.9691	4.8306	5.8438	5.5159	5.5375	5.8827	
6.1636	5.5169	5.0531	5.8674	6.0576	5.4546	4.9431	5.6069	
5.0779	5.3547	5.0967	5.0634	5.6781	6.1272	4.517	6.4657	
5.5778	5.4585	5.6069	5.478	5.1561	5.6846	5.3318	5.148	
5.2876	5.7068	4.9234	5.879	5.2074	5.4719	5.3984	4.7237	
5.6846	5.4849	5.88	6.1484	6.6258	6.4081	4.6561	5.4871	
6.235	5.8931	5.2004	6.2769	5.5715	5.5105	5.7886	4.8303	
4.9318	5.6752	5.2418	5.9774	5.2042	4.7872	5.5182	6.2272	
5.917	5.8469							
ARL9	1.0146	0	0	0.2025	0.1444	0.1676	0.0739	
0.3015	0.0791	0.8665	0.5843	0.0593	0.0234	1.7628	0.0728	0
	1.0134	1.1244	2.2934	0	0.4592	0.0151	0	
0.0122	0.1589	1.1707	0.4079	0.3086	0.0316	0.0413	0.2244	
0.0018	1.2315	0.1698	0	0.5874	0.2657	0	0.1536	
1.3225	0.3086	0.8334	0.1245	0.29	0.0991	0.5883	1.5978	
0.4976	0.0339	0.4627	0.4921	0.1363	0.1691	0.3058	0	
1.6051	0.152	0.3316	1.1916	0.1359	0	0.1982	0	
0.2576	1.9385	0.0788	0	1.0107	0.7524	0.491	0	
0.1281	1.1523	0.232	0	0.3939	0.5147	0	0.0979	
0.1894	0.6697	0.1483	0.1748	0.2065	0.4577	0	0.9208	
1.0912	0.6476							
ARMC1	4.0446	3.8973	4.5302	4.7357	4.5224	4.5549	4.1908	
4.473	4.1191	4.4373	4.5854	4.4603	4.4988	4.4879	4.8213	
3.6179	4.685	4.5569	4.4933	4.5269	4.5326	4.5103	4.152	
4.079	4.6531	3.9344	3.9926	4.4547	4.4397	4.0446	4.5518	
4.3309	4.7119	4.6605	4.6087	5.0023	4.5355	4.4988	3.5114	
4.6757	2.9417	4.1601	5.1223	4.2383	4.8094	5.6788	4.7772	
4.1453	3.6807	4.8161	4.4494	4.4121	4.6004	3.8359	4.6208	
4.0211	4.707	4.6802	4.3471	4.4473	4.4978	4.353	4.918	
3.7422	5.5306	3.7237	4.2485	4.572	3.62	4.2903	4.2366	
4.1337	4.4881	4.0889	4.0287	4.8039	4.4543	4.1227	4.9207	
4.3668	4.7271	4.2038	3.8899	4.2431	4.3213	4.6011	4.0466	
4.5067	4.0741							
ARMC10	4.9007	5.0777	3.8908	5.3735	4.6316	5.1239	5.1618	
5.1232	5.4881	4.9294	5.4698	4.4019	4.4157	4.9013	4.7675	
4.4936	4.7484	5.9568	5.7656	4.2779	4.4537	5.3627	5.3777	
5.2215	5.2572	3.9751	5.8042	4.406	5.4167	4.9147	3.9726	
5.3046	4.5761	4.1846	5.0629	4.9831	4.3935	4.4237	5.1094	
5.2915	5.3096	4.5341	4.6238	4.9837	4.7855	5.3686	4.6406	
5.173	4.9694	4.1525	4.8889	5.4658	4.3543	5.1336	4.8439	
4.9723	3.8485	4.7361	5.2936	5.2715	4.6417	4.9306	5.0008	
5.2025	5.1721	5.0176	3.9288	4.5113	4.2977	4.7119	5.2827	

4.9116	5.1988	4.8867	4.2132	4.5571	4.1903	5.0199	4.9621	
5.7106	5.2701	4.2955	3.128	4.4835	4.5953	4.2358	4.3663	
4.142	3.9327							
ARMC12	0.3072	0.1173	0.3306	0	0.2154	0.0853	0.0429	0
	0.0455	0.0509	0.5431	0.1468	0.0372	0	0.0799	
0.2144	0.4437	0.4694	0	0.5014	0	0.2372	0.2513	
0.0761	0.1929	0.2144	1.5954	0.5065	0.183	0.4992	0.5984	
0.1365	0.1845	0.3609	0.0738	0.1311	1.2129	0.1138	0.5431	
0.3551	0.2008	0.2599	0.3176	0.1094	0.1063	0.1513	0.0814	0
	0.3338	0.3617	0.3521	0.2223	0.2443	0.6977	0.8342	0
	0.1879	0.0501	0.1634	0.0456	0	0.0877	0.1406	
0.0653	0.5401	0.5418	0.9663	0.1	0.2955	0.7891	0.2049	
0.2987	0.0441	0.1082	0.2112	0.4247	0.1086	0.0884	0.1492	
0.6099	0	0.1714	0.3043	0.3736	0.1237	0.2201	0.2892	
0.2091	0.4095							
ARMC2	0.6844	0.8658	0.8013	0.6442	0.4347	0.6168	0.2928	
0.5966	0.9426	0.8076	0.3861	0.3704	0.5078	0.8729	0.5582	
0.5208	1.1009	0.6629	0.8687	0.7411	0.776	0.712	0.6031	
0.7542	0.4918	0.6494	0.653	0.5996	0.483	0.5158	1.4386	
0.4904	0.733	0.6453	0.5755	0.9898	0.2604	0.651	0.344	
0.7003	0.6706	0.3843	0.418	0.6337	0.6432	0.734	0.9057	
0.8142	0.5921	1.3052	0.7576	0.7475	0.8154	0.4537	1.2338	
0.8272	0.461	0.4741	0.4524	0.6246	0.5675	0.9292	0.3218	
0.767	0.5878	0.289	0.9045	0.2317	0.6068	0.5902	0.6826	
1.1809	0.7099	0.6488	0.6229	0.7015	0.8262	0.7269	0.5417	
0.8686	0.8445	0.6192	0.5494	0.902	0.347	0.7052	0.4456	
0.5422	0.6494							
ARMC3	0.261	0	0	0	0	0.0306	0.0263	
0.1691	0.1066	0.2639	0.0295	0	0	0.0285	0	0
	0	0.0392	0.0614	0	0	0	0	0
	0.0906	0	0.0185	0.0857	0	0.2187	1.3602	
0.0246	0.0201	0.1975	0.0016	0.3754	0.0052	0	0	
0.0131	0	0	0	0.0476	0	0	0.0961	0
	0	0.0942	0.0236	0.0189	0.1177	0.5196	1.061	0
	0	0	0.421	0.0195	0.0157	0	0.0259	0
	0.0719	0.0603	0.029	0.0015	0.0386	0	0.0135	0
	0.1126	0	0	0.1846	0.0231	0.0129	0.0581	
0.0257	0	0	0	0	0	0	0.0284	
0.0322	0.0603							
ARMC4	0	0.4856	1.04	0	0.2639	0.1358	0.0267	
0.0346	0.0563	0	0.0684	0.3333	2.9753	0	0.0699	0
	1.9573	0	0	0	0.2201	0.0548	0.4089	0
	0	0.0556	0.0388	0.0472	0.0476	0	1.5771	
0.0112	0	0.0244	0.2085	0.4612	0.023	0	0	
0.023	0	1.0172	0	0	2.9916	0.5473	0.0407	0
	0.1738	1.0001	0.0397	0.0589	0	0	1.8486	0
	2.9443	0.5064	0	0.8666	0	0	0.5963	
0.0101	0	0.9972	0	0	0	0.0999	0	0
	0.1252	0	0	0.2017	0	0	0	0
	0	0	0	0.0689	0.7347	0	0.0353	

0.0482	0.0464						
ARMC5	1.5329	2.7199	2.719	1.3673	2.6712	2.888	1.4837
2.6445	1.8137	1.5774	1.6474	1.2569	1.5855	1.5507	1.9286
2.3018	2.2291	1.3854	1.7918	2.3662	2.2532	2.1175	1.5868
2.3972	1.7032	1.7729	0.9096	2.2219	2.0613	1.1326	2.1568
2.9673	1.9654	1.657	1.9576	0.9182	1.9335	2.5978	1.5769
1.442	2.0582	1.6624	1.2197	1.671	1.4848	1.6524	0.7759
2.1012	1.5488	1.3431	1.8025	0.6935	1.5501	2.5977	2.2785
2.0876	1.5798	2.1176	1.4792	1.9819	2.3146	1.8868	2.7001
2.2245	1.1817	1.8251	1.5723	1.888	3.2429	1.8909	1.9328
1.9323	2.1731	1.0322	2.2436	2.0714	2.0223	1.6544	1.7739
1.7923	1.4329	1.583	2.6601	1.3278	2.7471	1.4675	1.9606
1.9236	1.7791						
ARMC6	3.9355	4.5129	4.9278	3.0665	5.0534	3.4496	2.907
4.6043	2.9145	3.1814	3.3641	2.9784	4.1441	3.8065	4.0291
3.5418	3.9719	2.6284	3.5796	4.4924	4.4097	3.7259	3.2256
4.5117	3.552	3.9839	1.5243	3.7836	3.9231	2.7075	3.0086
5.1028	4.7361	3.5857	3.4304	2.8101	3.9413	4.1767	4.3006
3.0696	3.3115	2.9049	3.1494	3.2256	2.8299	2.876	2.6922
3.434	3.5296	2.8318	3.0046	2.7228	2.9849	4.9672	3.7003
5.0865	3.0986	4.2362	3.3813	4.2074	4.296	3.9975	4.2368
3.9986	4.3702	2.1755	3.0845	4.1073	4.1779	3.16	3.8139
4.0335	3.5326	2.7213	2.7573	3.6421	4.5038	2.7222	3.6638
3.1397	3.6451	2.7129	3.7474	2.9906	4.5177	3.87	3.0524
3.7903	3.3328						
ARMC7	2.6609	3.1111	3.1429	2.5533	2.9601	2.505	2.2318
2.3518	1.8086	1.9826	2.3897	2.1158	2.1625	2.0726	2.4409
2.9551	2.3662	2.0954	2.0348	3.8475	3.6543	2.349	2.6363
2.4796	2.2902	2.7032	1.103	3.4768	2.3716	1.7651	2.694
2.6012	2.4522	2.0987	2.4898	2.255	3.1379	2.8065	2.4089
1.7012	1.9888	3.5147	1.5596	1.9161	2.6751	2.158	2.1268
2.7419	2.0283	2.5412	2.3598	1.7817	2.0895	2.7525	3.528
2.5624	1.9346	3.3844	2.4738	3.2784	2.1905	2.479	2.3479
2.2957	2.5536	1.8836	2.9529	2.9238	2.5282	2.3959	1.881
2.2867	2.8581	1.3974	2.6285	3.3394	2.8462	2.0856	2.1624
1.7352	2.2363	2.3518	2.1105	2.6316	2.9099	2.5287	2.3644
2.153	2.2366						
ARMC8	2.9236	3.5641	3.3205	3.2387	3.2033	3.4885	3.2531
3.8435	4.0318	3.4746	3.1767	3.2303	3.3977	3.8307	3.5278
3.0698	3.5612	4.0738	3.9299	3.1912	3.2552	3.3827	2.8408
3.771	2.9479	3.0417	3.9213	3.0649	3.4488	3.0814	2.8857
3.717	3.608	3.5485	3.0464	3.713	2.7524	3.111	3.1735
3.9374	2.8353	3.0591	3.5564	3.1227	3.9271	3.515	3.696
3.921	2.8348	3.9021	4.2393	3.3878	3.3662	3.9094	3.2696
3.4942	4.1495	3.4581	3.0241	3.9716	3.4017	3.044	2.8409
3.4014	3.2168	3.2386	2.8011	2.8234	3.4009	2.8663	2.8936
3.8799	3.3301	3.1065	2.7101	3.0625	3.4066	3.012	3.1853
3.5536	3.5948	3.0705	2.8423	3.5053	3.7622	3.5094	2.9419
3.0904	2.9524						
ARMC9	4.7054	3.3101	3.4409	3.2291	3.1584	4.0407	4.9652

5.8195	6.0443	4.0566	4.6415	4.986	6.0588	5.7757	4.4915	
4.686	2.7421	4.5591	4.8153	5.4989	4.6956	4.1549	4.4063	
5.4283	3.2354	4.473	4.4483	3.1961	4.9476	4.1561	2.797	
4.5798	6.245	5.9144	5.294	3.3069	4.3056	5.6644	4.2037	
4.9578	5.4188	4.2653	3.9867	4.3556	3.4828	3.5257	4.443	
4.2421	4.0423	3.0605	4.7811	2.7352	4.303	5.3816	2.9285	
3.5325	2.8797	3.1265	4.7641	3.1333	3.9955	3.4516	4.4652	
4.5414	5.941	5.856	6.7061	6.6137	3.739	3.8235	4.2954	
4.6871	4.4639	4.4469	4.6615	4.3953	5.0049	5.1202	4.8066	
3.541	4.4666	4.2363	6.7721	4.434	4.2501	4.157	6.7025	
5.6142	6.7624							
ARMCX1	4.5071	0.0904	0	1.689	0	2.9391	4.5962	0
	2.6679	0.5843	0	3.673	2.4412	3.5911	1.8267	
3.8318	4.0886	2.6076	0.5488	3.03	3.2399	2.9657	3.8179	
1.3176	2.8572	3.8349	4.5861	0.9226	3.1669	1.2362	0	
4.2077	3.9701	4.872	4.007	1.6134	4.1251	3.0025	1.6511	
3.9718	3.1213	3.4038	2.8682	3.1414	3.3648	3.2573	2.9395	
2.8384	3.8216	5.59	3.9605	4.1831	3.1859	0	0	0
	4.4431	4.1012	0.1422	2.853	2.3864	3.0267	3.1319	
4.2051	4.692	3.9035	1.1792	4.1468	3.8542	2.7572	2.4485	
2.6626	4.828	4.1398	2.1458	3.5062	4.1659	4.6483	3.5969	
0.1342	0.258	3.1211	4.2034	3.3284	2.8766	3.9425	5.116	
4.9973	5.2301							
ARMCX2	2.934	2.3311	0	0.7898	0	1.303	1.7927	0
	1.0138	1.9761	1.0588	1.085	0.3349	1.398	4.4882	
1.6645	4.4439	3.5193	2.1255	0.2443	1.9731	0.4973	3.4699	0
	4.2255	5.0376	2.9297	0	2.8413	3.5871	0	
2.1061	4.4913	4.344	1.5715	2.6119	3.432	0	1.6539	
2.8692	3.9573	3.86	4.3136	0.1182	2.1488	0.6581	0.6933	0
	0.1831	2.9522	4.0704	4.6127	1.4843	2.1421	0.1777	0
	4.5504	0.3393	3.827	0.1719	0.7949	0.8399	1.6208	
0.1811	0.5425	5.0209	0.1895	0	1.935	3.8787	4.5865	
1.534	1.6548	4.2126	0.168	1.1269	1.6595	1.2901	3.362	
0.2806	2.9489	3.2054	3.5162	3.6406	2.5023	1.6975	3.1051	
3.7302	3.0225							
ARMCX3	4.9093	2.8108	3.0744	2.8008	2.1369	3.8199	5.734	
4.8738	4.5215	3.0077	5.216	3.5617	3.8213	3.871	5.0665	
5.1332	3.8459	4.7038	2.5389	3.3364	3.9488	3.2397	4.1934	
4.3918	4.0874	5.1301	5.1003	4.3594	3.3915	3.8279	3.7334	
4.5472	3.8941	5.3984	5.6515	3.7934	4.121	2.1594	3.5617	
4.664	3.7044	4.031	3.5521	3.9675	4.2594	3.3589	4.3391	
4.4706	4.4224	4.9104	4.8416	3.5871	3.8827	4.1209	4.1655	
2.8327	4.6934	3.9453	3.9094	3.7902	3.9139	3.2077	4.64	
3.8326	4.4929	4.7611	5.3023	0.4798	4.6493	3.9917	3.9887	
3.8592	4.4568	4.1925	3.3451	4.28	4.0795	5.6435	4.2684	
3.343	3.6555	4.1231	4.9175	5.6775	3.2712	3.7324	5.3657	
4.7634	5.1782							
ARMCX4	1.8891	0.4353	0.093	0.0199	0.4174	0.0209	2.1359	
0.1082	1.046	0.7867	1.4418	1.0196	1.2477	1.5616	1.4887	
0.2829	0	1.8901	0.4694	0.0331	0.6033	0.4312	1.8073	0

	1.2685	2.2199	1.9179	0.3616	1.167	0.8405	0	
1.7035	1.7515	2.4002	1.0465	0.4147	1.5949	1.1503	0.451	
0.1339	0.8205	0.5966	1.9326	1.2046	2.7382	1.2483	0.9704	
0.5621	1.5386	2.999	1.6953	1.8324	1.3499	1.5412	0	
0.1287	0.8119	2.9206	1.1177	1.2116	0.3181	1.6979	0.5471	
0.1223	2.0276	1.4207	0.226	0.0945	2.2874	0.8878	0.7093	
0.7288	2.0597	1.2879	1.0226	1.9362	1.6921	0.7957	0.6905	
0.0637	0.0312	1.0612	1.3107	2.4143	1.6134	1.33	2.103	
2.5391	2.453							
ARMCX5	2.9925	1.795	2.5349	2.6245	2.0675	3.4443	3.4069	
2.6083	3.4124	2.6517	2.6229	3.1331	2.3613	2.5856	2.9564	
3.7813	3.1603	2.7147	2.4587	1.9989	2.3417	2.481	2.8168	
3.2663	2.0847	3.0085	3.3615	1.9882	2.7427	1.887	2.8833	
3.1265	3.4171	3.8145	2.8718	2.7554	2.9861	2.6697	2.0624	
3.9011	2.54	2.1054	2.2527	2.8438	2.5582	2.7427	2.6237	
3.5726	2.6315	3.7252	1.9968	3.4607	2.5752	3.1373	3.1751	
2.5739	2.4194	2.8406	1.8947	2.9567	2.0836	1.8802	3.288	
2.8511	2.6219	2.3322	2.921	2.1826	2.9251	3.0957	3.6189	
2.3469	2.9183	3.1781	2.5902	3.3843	2.6876	3.3645	3.8902	
2.2583	3.3161	1.8561	2.7401	2.0639	2.3784	2.5912	2.2673	
2.4763	2.3097							
ARMCX6	4.5734	3.9565	3.4608	3.8365	2.9926	4.3791	5.6826	
5.5131	5.075	3.8811	5.0842	3.4743	4.5119	4.4451	5.2117	
5.6878	4.4956	5.0873	3.5382	3.7252	4.8345	3.4835	4.8023	
4.9991	4.2446	5.0507	4.5721	4.1032	4.0219	3.6917	4.7339	
5.7578	5.3044	5.2254	5.3162	4.3303	3.6863	4.5734	3.1939	
5.7863	3.3882	4.5084	4.1694	4.7274	5.5296	4.5368	3.8597	
4.7223	4.0714	6.8152	4.6363	4.9618	4.5157	4.2153	4.6533	
1.4043	5.0859	4.7056	3.8931	4.9276	4.3563	4.3076	5.0259	
4.6353	4.3207	5.041	5.1539	0	4.6804	4.2336	4.5193	
4.1816	5.2629	4.3899	4.0684	4.9311	4.9311	5.2296	5.0051	
4.1601	4.3751	4.0691	5.0041	5.0758	4.5197	4.7214	5.2173	
4.8068	4.8736							
ARMS2	0	0	0.1092	0.3203	0.0255	0	0.0492	0
	0.0503	0	0.0364	0.0296	0	0	0	
0.0448	0	0	0	0	0	0.0345	0	
0.0634	0.0361	0	0	0	0.052	0	0.1999	0
	0.0691	0	0.0238	0.0476	0	0	0	0
	0	0	0.0851	0.0735	0	0	0	0
	0	0	0	0	0	0	0	0
	0	0	0	0	0	0	0.0778	
0.121	0	0	0	0	0	0.1437	0	0
	0.0912	0	0.1849	0	0	0	0	0
	0.0811	0	0	0	0	0	0	0
	0							
ARNT	3.2528	3.4541	2.926	3.4137	3.3422	3.3237	3.6476	
3.2143	3.9371	3.9643	4.3034	4.0865	3.2614	3.5405	2.9039	
3.3234	3.494	4.1281	3.2339	2.5871	3.4308	3.3644	2.8925	
3.3803	3.7278	3.4962	4.4753	3.5544	3.2617	4.2943	4.7327	
3.0733	2.9487	3.5133	3.3319	4.1171	3.7417	3.5334	3.2846	

3.6893	3.5981	3.265	3.8079	3.4507	3.6281	4.2716	3.5862
3.4808	3.5514	3.7209	4.163	3.8185	3.4137	3.1719	4.7061
3.2066	3.5386	3.367	3.9207	3.4293	3.4211	3.0817	4.003
3.3749	3.9105	4.0187	3.7	3.3084	3.8743	3.5739	3.1358
3.5693	3.619	3.321	3.8568	3.02	3.1638	4.1449	3.6772
3.3412	3.6906	3.5217	3.7313	4.2137	3.067	2.8168	4.0647
3.3904	4.1199						
ARNT2	4.2114	3.4322	3.6097	3.8661	3.856	4.9275	4.4111
5.076	4.4233	3.9081	3.3466	3.5719	1.8161	3.8776	2.9156
5.2398	0.17	5.3887	4.2521	3.2869	3.1257	4.0422	4.4187
4.9191	4.4074	2.6688	3.0045	3.4689	4.0242	3.0772	3.0907
4.2889	4.5071	3.2712	4.8125	3.1836	2.9551	3.3982	4.8025
5.0464	4.2227	0.0224	2.8525	3.9092	2.5682	1.75	3.5252
4.1481	4.6173	2.2784	3.9211	2.9473	4.1917	4.8902	2.2082
3.8873	0.5451	4.1391	3.3618	4.3441	3.6972	3.7945	5.9799
4.632	3.0505	2.7152	0.4102	3.4644	4.45	3.6636	3.3332
3.7924	4.5417	4.56	4.6979	4.0227	3.3658	5.0116	4.9215
4.2102	4.0585	2.0139	3.4496	4.7982	2.6797	3.4865	2.4654
3.3525	2.9006						
ARNTL	1.5881	3.1491	2.6385	2.7504	2.344	1.9093	3.2327
2.6764	2.6439	2.3548	2.8984	1.9679	3.3228	2.2376	2.9854
2.0581	2.8683	1.7091	2.8345	2.4246	2.254	2.5934	2.4639
2.2107	2.1802	2.5089	2.1012	2.628	1.905	2.7015	1.9006
1.7461	2.2571	2.2733	2.1346	2.7359	2.923	2.2625	2.3061
1.6535	2.0842	2.3389	2.9052	2.5075	2.1722	2.7329	2.7182
2.637	2.264	3.5381	2.8736	2.4437	2.3178	2.5205	2.2675
2.5152	2.4241	2.8452	2.5838	2.8778	1.4214	2.31	1.3755
2.4194	1.662	3.3791	2.242	2.348	2.1379	2.3531	2.074
2.6324	2.2084	2.6718	2.0387	2.6262	2.65	2.0089	2.042
2.5834	2.4113	2.5437	3.5491	2.0292	2.182	1.9422	2.8214
1.8848	2.4357						
ARNTL2	0.4736	2.1073	1.6255	2.2584	2.344	0.2876	0.7487
1.4992	1.0399	1.0717	0.3646	2.37	3.4211	1.7912	1.8029
0.1885	5.4215	1.5797	2.0606	0.5401	0.1993	1.234	1.3587
0.2148	0.6809	1.974	0.4136	1.2147	1.8999	1.6608	2.9759
0.8168	0.5011	0.8214	0.9655	1.7758	1.8919	0.3769	0.2482
0.4675	0.219	2.104	4.125	0.7521	4.2145	0.9736	1.0075
1.454	2.4719	2.7655	1.6134	0.9084	0.7082	0.269	2.6173
0.2772	5.1891	3.2376	1.8534	2.0905	1.1687	0.4412	0.9019
0.0968	0.0311	2.8823	0.0449	2.6793	1.5733	1.7631	1.1779
0.5498	0.4862	1.0608	1.516	1.4267	0.1264	0.1377	1.007
0.7795	1.6989	1.873	1.06	1.0139	2.5648	0.9944	0.4876
0.2716	0.2032						
ARPC1A	7.0614	7.994	6.6667	7.2857	6.7631	8.5469	6.0872
8.0921	7.4427	7.5578	7.7903	6.3045	6.9902	7.4167	6.9135
7.0882	6.8444	8.1165	8.1244	6.1417	6.9979	7.5409	6.9829
7.5756	7.5123	7.0829	6.0858	7.0722	7.5831	7.1279	6.399
7.0031	7.0004	6.3936	7.8381	7.4681	6.5854	6.5055	7.1014
6.7878	7.1686	6.5985	7.2613	7.5797	7.0665	7.0292	6.5389
7.2455	6.4788	5.921	7.0932	7.125	6.3374	6.5875	6.831

7.2553	6.6773	7.6122	7.4798	7.6561	7.2265	7.3025	7.2877
7.3085	7.2854	6.2491	7.8305	7.0321	6.7849	7.0893	7.6437
7.4267	7.8305	6.3789	6.9912	6.9149	6.9747	7.1229	7.0369
7.7169	7.4437	6.495	4.3382	7.0732	7.5552	6.8651	6.8401
7.0526	7.006						
ARPC1B	7.8609	7.8283	7.3264	7.4641	7.0904	8.7427	6.596
8.5508	8.2393	8.5079	8.5508	7.0361	6.4815	8.1438	7.193
8.083	6.7048	8.8953	8.1703	6.6674	7.8318	8.3903	7.5419
7.3159	7.8122	5.4656	7.2804	7.6438	8.3562	7.5838	5.6882
8.7943	7.1344	8.0368	8.6303	7.754	7.2963	8.1226	8.2453
6.6567	8.783	7.2092	7.915	8.6226	6.4724	7.0101	7.0519
8.0174	7.8482	4.4082	7.9286	6.8854	7.2201	7.229	6.8577
7.9624	6.4327	8.5488	8.5258	8.1819	7.8343	7.7148	7.998
8.2833	7.6792	5.8015	8.133	7.7898	7.6207	8.1016	8.4678
8.4809	8.1819	7.3097	7.3648	7.5715	7.7081	8.3676	8.1175
8.9744	7.9829	6.8114	4.4992	8.8412	7.6676	7.8897	7.8002
8.0085	7.9511						
ARPC2	7.6572	7.1939	8.2044	8.2044	7.2688	8.6676	7.684
8.1356	7.9411	7.8709	7.7352	7.9768	8.168	8.3758	7.8709
7.4453	8.1418	7.8596	8.0121	7.2688	7.7965	7.3636	7.7306
8.3466	8	7.6388	7.3988	7.7258	7.8373	7.9025	6.93
7.5822	8.3681	7.6697	7.5782	7.9025	7.9411	7.4305	7.0082
7.6697	7.5615	8.4419	8.7438	7.604	8.0947	8.0121	7.8968
7.9693	7.8655	7.6481	7.8218	7.5906	7.7011	7.393	7.2427
7.1464	8.223	7.6326	7.4214	7.4629	7.4838	7.4658	6.7606
7.9957	8.2097	7.6171	7.7152	8.0262	8.467	7.9489	7.7259
8.0262	8.2803	7.9142	7.6989	7.9142	7.8958	7.481	7.8355
7.6367	7.8133	7.9189	7.9404	7.1554	8.4904	7.7186	7.9768
7.8809	7.7722						
ARPC3	7.5443	7.7226	7.845	7.8299	7.9461	7.7124	8.2397
7.5699	7.7841	7.8682	7.594	7.3606	6.9531	7.9139	6.69
7.2397	8.2799	8.0965	8.3927	7.2577	7.845	7.5178	7.2976
7.8242	7.6043	7.4142	7.9885	7.6043	7.1827	7.6595	7.7951
7.7326	7.6127	7.5093	7.2152	8.2056	7.199	7.4372	6.8326
7.8352	7.4822	8.09	7.3567	7.779	7.8352	8.1237	7.4999
7.6312	7.378	8.3449	7.4503	7.7423	8.1753	7.2561	7.7591
8.0401	7.9828	7.6714	7.6418	7.4129	7.9704	8.0173	6.7739
7.5016	8.0126	7.3512	7.8883	7.9908	7.254	7.0878	7.521
7.2974	7.5611	7.4691	7.3083	6.768	7.4878	6.8158	8.2098
7.6232	7.7591	7.6451	7.8137	7.1905	7.4054	8.2433	7.9761
7.774	7.9696						
ARPC4	5.651	6.6513	6.3763	7.2312	6.7937	6.4568	6.4903
6.3918	5.7186	6.3936	6.3427	6.9907	6.7798	6.8234	6.9124
7.0853	6.9588	6.5309	6.5675	6.0913	6.2984	6.3644	6.2349
6.3363	6.1924	5.9724	6.2604	6.6688	6.8325	6.609	6.8934
7.1972	6.1496	5.7264	6.3119	6.8642	6.5573	5.9712	6.7147
5.9178	6.6668	6.9221	6.9007	6.4409	7.4905	5.6751	6.8714
6.1439	6.529	6.7277	5.9826	6.4814	6.328	6.3032	6.6358
5.9882	6.44	6.6611	6.4063	6.5298	6.881	6.7002	5.8882
6.2404	6.3854	6.066	6.5475	7.1926	6.796	6.5414	6.3722

7.7269	6.4292	6.4265	6.4671	6.2911	5.9909	6.6627	5.4938	
5.8946	5.9496	6.8528	6.1924	5.9953	7.1269	6.4425	6.7607	
6.7042	6.6844							
ARPC5	5.8957	6.1991	5.8368	7.3772	6.8616	6.7154	6.4873	
5.8194	6.5072	6.4661	5.7014	6.7934	6.3707	6.4519	6.7934	
6.1745	5.6945	7.1618	6.5351	6.156	6.4785	6.2352	6.8324	
6.3442	6.7718	6.5645	6.6031	6.2414	6.3671	6.2296	6.1106	
6.4449	6.0326	6.1621	6.7196	6.798	6.9941	5.5237	6.4854	
6.4891	6.798	6.8281	6.9863	6.4908	7.0018	6.5515	6.2609	
6.5312	6.2269	6.1174	6.3229	7.0579	6.4334	5.7899	6.4653	
6.1235	6.8552	6.3149	7.0585	6.6649	6.6685	6.3641	6.3829	
6.6649	6.5154	6.0231	6.7553	5.907	6.9281	6.757	5.9662	
6.6634	5.693	6.216	6.9117	5.8241	6.1094	6.7139	6.6102	
6.2034	5.7951	6.3775	5.6843	6.7123	7.1037	6.6265	6.8118	
6.4124	6.678							
ARPC5L	5.4057	5.8846	5.5393	6.3125	6.0386	5.7495	4.7744	
5.7758	4.6509	5.1504	5.4654	5.8592	5.957	5.4726	5.3598	
4.7609	6.2315	5.1364	5.2814	5.4908	5.6726	5.336	5.2192	
5.8858	5.6371	4.9618	6.0175	5.6657	5.2341	5.3144	5.1739	
5.9168	4.9051	4.7761	4.6766	5.6236	5.9374	5.2537	6.1966	
5.2843	5.6353	6.2427	5.3817	5.4992	4.7712	5.5657	5.5791	
5.3306	4.8907	6.1861	5.2949	6.2385	5.5491	5.2859	5.8965	
5.3151	6.6537	5.7752	4.9343	5.5054	5.9156	5.1092	5.1569	
5.4602	5.7094	5.1925	4.7491	5.23	5.6602	6.5228	5.072	
4.8837	5.2852	5.7731	5.0738	4.7408	5.6977	5.3024	4.8176	
5.4189	5.3277	5.8135	5.1016	5.6677	5.5079	6.0232	4.8717	
5.0576	4.9956							
ARPP19	5.5129	5.9338	5.4545	6.2908	5.8031	6.0638	6.1316	
5.916	5.5248	5.8391	5.5068	5.3862	5.2206	6.0246	4.8168	
6.2821	5.4265	6.6951	5.8278	5.7695	6.4358	5.8041	5.6548	
6.2502	5.5011	5.0155	5.9945	6.541	6.1276	5.8619	5.4015	
6.1678	5.1631	5.1438	5.9451	5.9304	5.5721	5.1094	5.625	
5.4571	5.7082	5.2065	5.4361	6.3948	6.8745	5.5669	6.2729	
6.279	5.9462	5.7333	5.8872	5.1492	5.8475	6.1471	5.2826	
6.6471	5.6628	5.4531	5.8955	5.6062	6.0395	5.7328	6.084	
6.3237	5.6991	5.2424	6.0224	6.1381	6.4217	5.3138	5.3215	
5.5201	5.5918	5.276	6.5355	6.1871	5.7284	5.9915	5.9484	
5.219	5.8145	5.7669	5.4854	6.202	5.4635	5.4335	5.873	
6.4294	6.3837							
ARPP21	0.0561	0.0275	0	0	2.8751	0	0	
0.0265	0.015	1.3733	0.0061	0	0	0	2.6885	
3.5394	0	1.2411	0	0	2.0671	0.0502	0.0925	
3.2096	0	0.0319	0	0.0495	0.0074	0	0	0
	0.1877	0.0058	0.1721	1.1837	0.0422	0.3893	0.0142	
2.806	0	0	0.0359	0.0252	0	0	0.037	
0.0307	2.5273	0	2.7182	1.836	0	0.2952	0	0
	0.1693	0.9193	0.0474	0.2783	1.5643	0	0	0
	0	0.0952	1.4054	0.0168	0	0	3.1603	
3.6501	3.0805	0.4881	0.0349	0	0.0244	0	0.0252	
2.5995	0	1.6389	1	0.4249	0	0	2.225	

2.2798	0							
ARR3	0.0742	0	0	0.0624	0.0126	0.1139	0.1145	0
	0	0.0322	0.082	0.0344	0.0494	0	0.0522	0
	0.0709	0	0	0	0	0	0.0316	
0.1415	0	0	0.0397	0	0	0	0.0523	
0.0058	0.0393	0	0.0074	0	0.0171	0	0	0
	0.0286	0.0707	0	0.0402	0.022	0.0357	0	0
	0.0887	0	0.0328	0	0	0.0541	0	0
	0	0.0456	0	0	0	0	0	
0.1086	0	0.0092	0.0661	0.0122	0	0	0	0
	0	0.0507	0	0.119	0.02	0	0.0949	0
	0.0745	0.0219	0	0	0	0.0826	0	
0.0544	0							
ARRB1	3.2232	3.7775	3.206	2.9293	1.6506	3.5127	1.9604	
2.2028	3.7428	3.4299	2.7812	1.777	2.4753	2.1054	0.4997	
4.727	1.3671	2.7677	2.116	2.2133	3.7625	2.8285	3.3165	
2.8074	4.2992	1.3987	1.0548	1.2564	2.927	1.4429	1.715	
1.9732	2.5269	3.3821	2.9285	3.2627	2.4395	3.1877	1.9963	
2.1806	1.9807	2.9726	1.4487	2.4299	1.4173	3.3592	2.5398	
3.2856	0.687	1.749	2.672	2.2233	3.0958	2.9525	2.3063	
3.3538	2.1372	1.184	4.5455	0.9056	1.3579	3.0125	1.7828	
3.8708	2.7735	1.0904	2.6716	2.5082	3.1113	1.7551	2.3248	
3.0529	2.7429	3.1697	2.7329	3.7307	2.5423	2.7358	3.8441	
3.2816	3.4324	1.8176	4.9513	1.7255	1.6176	2.5571	3.0635	
3.007	3.3158							
ARRB2	4.063	4.2582	3.4473	4.2842	3.6449	3.2245	3.3736	
3.456	4.056	4.1473	3.045	3.6241	3.661	3.3697	3.185	
4.0437	3.9793	3.5812	2.8375	3.8683	4.01	3.4576	3.1585	
3.2241	2.5737	2.7235	4.5993	3.9358	3.4806	3.8097	5.2758	
2.1468	3.241	3.0481	2.7033	4.2322	2.7641	3.0129	3.1647	
2.7141	3.606	2.0759	3.2931	4.29	3.3638	2.0305	3.4044	
3.4965	2.8751	4.8038	3.5711	3.3772	3.2387	2.782	5.1843	
3.6276	2.6571	3.4707	2.208	3.5839	3.2633	2.6755	3.3249	
4.4806	3.4177	2.8629	3.9688	2.917	2.9071	3.5326	3.7165	
4.3015	3.9436	3.6281	4.4946	3.5827	3.5782	3.4297	4.1192	
4.507	4.5893	3.7653	2.2006	3.2933	3.8059	3.181	2.9264	
3.231	3.4838							
ARRDC1	3.4731	2.6252	3.0297	2.0086	3.3494	2.2482	1.8809	
3.5288	2.1771	2.7407	3.2081	2.7105	3.4713	1.8809	2.7842	
2.1926	3.0074	2.4888	2.2887	3.3111	3.443	2.0164	2.4481	
2.5118	2.4135	1.6879	2.8827	2.9867	2.0379	2.5555	2.6033	
3.5617	2.0054	3.224	2.0695	2.131	3.3681	3.4548	3.3394	
1.4721	3.7548	3.4803	2.277	2.3002	2.234	2.1235	1.9593	
2.6446	2.9381	2.4108	2.1911	1.6422	2.1281	2.7728	3.088	
2.2527	4.3584	3.4206	2.9198	3.0846	3.0264	2.1755	2.0746	
2.3204	2.961	3.2089	2.197	3.2068	2.7425	3.5952	2.4222	
2.4025	1.9648	2.0295	1.3956	3.0974	2.6158	2.3244	2.6639	
3.1308	3.0374	2.8547	2.9947	2.879	2.4222	2.6635	2.7438	
2.8499	2.8553							
ARRDC2	2.353	3.0474	2.828	2.0081	3.0643	1.7265	2.2186	

1.1284	1.8973	2.5994	3.2827	2.2506	2.9575	2.5559	2.172	
1.3063	2.6431	2.5669	1.814	2.3155	2.191	2.6417	1.6614	
1.9315	2.4635	2.6224	2.8284	2.6471	1.9991	2.3305	2.0177	
2.1849	2.175	2.3276	1.4059	2.1877	2.8424	2.788	2.9542	
2.2462	2.1256	3.0017	2.2544	2.1871	1.3726	3.595	1.9099	
2.2792	2.7819	1.7272	2.47	1.9506	2.6617	3.8318	3.3536	
3.3454	2.7109	2.4323	2.9544	2.407	2.8182	2.0652	2.5286	
2.4122	2.5803	2.3568	2.4206	2.5902	2.1224	2.4997	1.9864	
2.6664	1.9981	1.5629	1.4394	1.6851	2.3155	1.0739	2.5599	
2.1273	2.4084	1.2464	2.5529	1.96	2.3717	2.284	2.9463	
2.7611	3.731							
ARRDC3	1.269	0.9851	1.2659	3.9014	4.4052	3.0678	3.3119	
2.6741	0.673	1.2641	0.9119	4.4057	4.9492	2.2039	2.5142	
2.8815	2.9006	1.0089	2.8235	3.7941	2.1229	4.075	3.4898	
2.9583	3.6544	4.8403	4.1377	1.4231	2.919	1.1175	4.9542	
2.2438	1.2519	2.578	3.4191	2.6444	2.0739	2.7117	3.3001	
4.6027	2.2914	3.9381	3.2585	3	3.192	4.4097	3.1397	
2.0398	4.9091	2.9862	2.5772	4.0253	3.6958	3.4436	3.0107	
0.7351	3.5826	1.6232	1.2263	1.9565	3.3028	1.7219	3.0931	
1.7885	1.5532	5.2065	3.1103	3.5614	1.3375	3.8639	3.6612	
2.1458	2.3882	3.3657	2.9549	2.1483	1.8142	2.8426	2.9793	
4.0306	2.9538	4.4711	5.155	2.4458	4.4299	1.5665	3.3239	
1.8813	1.9885							
ARRDC4	1.4417	2.3184	1.508	1.2397	1.0645	3.2697	4.3548	
1.4734	3.8734	3.1274	3.9023	2.0744	1.913	3.0573	2.4952	
4.9658	0	2.1406	2.4888	2.4878	2.3867	3.827	3.7668	
4.3207	0.7796	2.3939	4.7151	0.4503	3.7539	4.6379	0.0286	
1.4212	3.7585	3.9023	2.6998	2.8196	3.0923	0.4181	1.8163	
3.2281	5.0442	2.5171	0.3214	1.553	0.9613	3.0179	0.5598	
3.2673	0.9297	1.8844	1.962	3.1861	1.9636	4.3152	0.4582	
1.6392	0.0247	1.9181	3.0198	1.6392	3.2049	4.16	0.3121	
3.8085	0.4299	3.7709	1.0081	2.2503	2.4116	1.2874	1.9725	
3.666	2.7654	4.3685	4.4626	0.8486	3.0032	2.8746	3.6492	
2.9497	4.4229	3.4532	1.5152	0.7712	1.3394	2.0725	4.1201	
3.1326	4.1942							
ARRDC5	0	0	0	0	0	0	0	0
	0.0226	0	0	0	0.0173	0	0	0
	0	0	0	0	0.0457	0	0	0
	0	0	0	0	0.0246	0	0	0
	0	0	0.0045	0	0	0	0	0
	0	0	0	0	0	0.0586	0	0
	0	0	0	0	0.0656	0	0	0
	0.0384	0	0	0	0	0	0	0
	0	0	0	0	0.046	0	0	0
	0.0326	0	0	0	0	0	0	0
	0	0	0	0	0	0	0	0
	0							
ARSA	3.1907	2.7318	3.0677	1.9068	1.9835	2.3356	3.5917	
2.522	2.7932	2.5987	3.4421	2.1767	3.5943	1.9567	3.6207	
2.7314	2.6684	2.411	2.4397	2.8266	3.0964	2.2419	3.5302	

3.15	3.4313	1.9386	2.2011	2.9918	3.0789	2.411	2.542	
1.9031	3.4213	4.8518	2.9371	2.1124	3.2688	4.2485	3.2134	
2.1356	4.4504	3.4639	2.7668	3.0881	1.6693	1.9405	2.6071	
2.8694	4.088	1.2706	2.4175	1.9265	2.3318	2.9261	1.1098	
3.0447	2.9023	2.7287	3.6057	2.1249	3.0774	2.4899	2.9326	
2.997	1.5785	4.8693	4.3731	3.4325	2.5257	2.377	4.4333	
3.2319	2.55	2.1593	2.9603	2.6671	2.3795	3.0744	3.8153	
3.5014	2.9076	4.011	3.5469	3.5157	2.5797	3.3183	3.6155	
4.1505	3.4061							
ARSB	3.8673	3.0577	3.9487	3.1143	2.934	4.5113	4.4093	
3.8279	4.19	3.0983	2.984	3.5711	2.7999	4.0273	4.5535	
4.565	3.4363	3.105	3.9531	3.9419	4.0417	3.7682	4.1903	
3.9683	4.0023	2.9759	3.2878	2.6595	4.3089	0.3668	3.1079	
2.8105	4.2297	3.3042	4.816	3.432	3.3178	3.8472	2.7487	
3.921	3.3358	3.3757	4.1799	3.7016	3.6427	3.6578	4.3397	
3.5583	3.3892	3.0174	3.2957	4.5342	3.4195	2.6946	3.0065	
2.5885	3.1076	3.0896	3.1967	3.1416	3.6184	3.5223	4.3545	
4.1127	2.5989	3.9225	3.3742	3.8849	3.5514	3.7952	3.8494	
4.185	3.3507	3.4691	4.8148	5.8708	3.5923	4.5253	4.4669	
4.153	2.8884	3.8849	3.713	4.3215	3.4691	2.8429	4.4904	
3.9759	4.4719							
ARSD	3.4957	1.7257	2.5722	2.1334	1.8272	1.7865	3.4917	
2.0104	2.5567	1.6007	1.6281	1.4362	2.3108	1.4243	1.6984	
3.0124	0.5557	2.8502	1.5339	2.2078	0.2597	2.2417	3.5476	
2.903	2.8058	1.9588	1.4191	1.9831	3.0777	1.7517	1.6094	
2.6173	2.5361	2.7048	3.6226	1.7501	2.6239	2.6129	2.7965	
2.3981	3.0855	2.4901	2.1557	1.7567	2.8741	2.1193	2.3952	
2.016	1.8444	0.1863	2.5867	2.4	1.6961	1.625	2.7824	
1.9967	2.3755	2.0989	1.7746	2.0759	1.8339	2.0177	4.1434	
2.5548	3.4224	3.0798	0	1.8957	2.2674	2.257	2.5241	
1.0147	2.946	1.637	3.3818	2.3202	1.8781	3.3204	3.233	
2.4116	1.6076	2.2335	3.1144	2.7858	2.0548	1.664	3.8041	
2.2854	2.4186							
ARSE	2.6953	3.1706	0.9948	2.7337	1.8954	0.8051	2.2565	
1.2835	2.6084	0.8689	0.24	0.3123	0.2006	1.5795	1.3416	
1.2405	0.0575	1.9542	2.3778	1.5165	0.123	1.3373	2.9187	
2.6211	1.8705	2.7836	0.0939	1.2412	1.665	0.9967	0.0206	
2.0508	1.7478	1.4442	3.3133	0.3339	1.8047	2.1795	2.1644	
1.1455	2.0958	0.6892	1.8404	2.0825	0.1322	0.092	0.6772	
2.0294	0.6352	0	0.8543	0.8027	0.8326	1.9655	0.0359	
3.0264	0.1012	2.3785	0.6357	3.0146	0.8401	2.1911	3.9704	
1.2334	2.6006	0.021	0.0746	1.0205	1.305	1.1607	0.2602	
1.0558	1.1503	1.5459	2.1417	1.186	1.1438	2.0151	3.1423	
1.0321	1.8238	0.0962	1.9855	1.8321	1.4532	0.8097	2.5968	
2.1526	1.221							
ARSF	0	0	0	0	0	0	0	0
	0	0.0393	0	0	0	0.0921	0	0
	0.0813	0	0	0	0	0	0.0148	
0.0262	0.0477	0	0	0	0	0	0	0
	0	0	0.0277	0	0	0	0	0

	0	0	0	0	0	0	0	0
	0	0	0	0.0511	0	0	0	0
	0	0.2388	0	0.2605	0	0	0.0121	0
	0	0.0318	0	0	0	0	0	0
	0	0	0	0	0	0	0	0
	0	0	0	0	0	0	0	
0.0663	0							
ARSG	3.2669	4.6088	3.8215	1.9141	1.1541	2.3543	3.3827	
1.0502	1.1782	3.9786	3.555	1.854	1.6234	4.1349	0	
2.4821	0.7154	2.5377	2.5424	4.4746	3.3276	0.2566	2.2211	
0.9207	1.6503	0.3699	3.3269	4.0491	2.8783	4.1262	1.1182	
1.9251	2.7449	2.5079	0.0028	3.429	3.1066	2.6848	2.4864	
0.6745	4.3165	0.4325	1.4134	1.5828	0.7909	3.0819	4.6628	
2.2016	2.7594	2.0904	3.0227	2.9845	3.6174	4.4238	1.1334	
2.4297	2.0182	0.2921	3.0714	0.2751	2.3416	2.4102	1.7119	
5.0759	2.1787	1.482	3.9383	4.5689	3.6194	1.5604	2.1858	
3.2519	0.251	0.7108	1.5291	4.4104	2.5404	0.8815	1.8153	
2.6732	2.0353	1.3787	5.8481	2.2285	2.0509	2.766	3.1052	
2.4552	2.5362							
ARSH	0	0.0586	0.0442	0	0	0	0.0141	0
	0.0177	0.1611	0.0088	0	0	0	0	
0.0353	0	0.0387	0.236	0	0	0	0	
0.051	0	0	0	0	0	0	0	
0.014	0	0.0241	0	0.1012	0	0	0	0
	0	0	0	0.0267	0	0	0.0382	
0.0307	0	0	0	0	0	0	0	0
	0	0	0	0	0	0	0	0
	0	0	0	0	0.0542	0	0	0
	0	0	0	0	0	0	0	0
	0	0	0	0	0	0	0.033	0
	0.0775							
ARSI	1.5199	0.5305	0.188	0	0	0.1379	0	0
	0.3894	3.0989	0.1903	0	0	1.6423	0.0314	
0.321	0	2.9062	0.0168	0.5075	0.1563	0.0087	0	0
	2.8516	0	0	0.1278	0.1208	0.3687	0	
0.5305	0.648	3.8916	2.4261	2.4338	0	0	0.0957	
0.0525	3.1461	0	0.0116	0	0.3009	0	0	
0.4039	0	0	0.0658	0.5735	0.3045	0.1221	0	
0.1851	0.1369	0.117	0.5887	0	0.1504	1.2097	0	
0.1476	0.4698	1.3183	0.2127	0.1081	0.1186	0	0.127	
0.5372	0.1555	0.1996	0	0.2097	0.7681	5.3265	3.7635	
0.5778	0.4419	0	0	0.2013	0.1289	0.4056	1.0237	
1.7628	2.5821							
ARSJ	1.7407	0.1542	0.7942	3.3319	2.2669	2.8635	2.3185	
1.2998	1.9697	0.8814	0.211	2.8768	2.3989	1.3932	2.948	
1.171	0.0586	1.6281	1.2609	3.0528	2.4231	2.2542	2.773	
2.609	2.9471	4.7516	1.8813	0	2.0478	0.1515	1.5913	
0.5259	2.0842	2.0276	2.3789	0.8939	1.1109	1.3751	1.2431	
3.5615	1.2019	3.5904	4.1005	1.6614	3.24	3.5493	0.9597	
1.5249	3.0768	1.7322	2.0852	3.1048	1.7076	0.8342	1.8701	

1.2025	3.5668	0.9469	1.02	0.7541	2.9483	1.143	3.0076	
2.1833	0.354	3.6607	0.1491	0.6198	0.6193	3.8449	2.5015	
1.7878	0.7746	1.6117	3.4901	0.5593	1.5259	2.972	0.9403	
1.8939	1.249	3.4531	0.9988	3.5266	2.9761	1.7643	0.6438	
0.4451	0.4864							
ARSK	2.1963	1.4693	1.6993	2.6558	2.5768	2.6028	3.2499	
2.4555	2.0727	1.9029	2.2577	3.1043	2.3493	2.7595	2.7004	
2.2689	2.0836	2.1717	2.2401	2.8245	2.9556	3.4289	2.9828	
2.7541	2.5301	3.2072	3.9634	2.247	3.1654	1.9139	3.169	
3.1544	2.926	3.6702	4.2267	2.227	2.5379	1.8315	1.7188	
2.8466	2.9297	2.4093	3.3316	2.2422	2.6537	2.4499	2.3734	
2.1584	3.8177	3.5507	3.0043	2.6787	2.8486	1.7415	1.9048	
2.4896	3.5062	2.2974	2.003	2.0714	2.5913	2.86	2.4737	
2.929	1.9424	2.8158	2.6812	1.9235	1.8063	3.3087	2.5358	
2.7851	2.1011	3.3429	2.9576	2.1439	2.7013	2.6404	2.4767	
2.8798	2.2916	3.102	3.3836	3.6504	4.3141	2.9949	2.1868	
2.2406	2.1948							
ART1	0	0	0	0	0	0	0	0
	0	0	0	0	0	0	0	0
	0	0	0	0	0	0	0	
0.0435	0	0	0	0	0	0	0	0
	0	0	0	0	0	0	0	0
	0	0	0	0	0	0	0	0
	0	0	0	0	0	0	0	0
	0	0	0	0	0	0	0	0
	0	0	0.0572	0	0	0	0	0
	0	0	0	0	0.0407	0	0	0
	0	0	0	0	0	0	0	0
	0							
ART3	0.3224	0.4028	0.7421	5.0776	3.6506	4.2467	3.5192	
1.0421	0.1634	0.016	0.3524	2.2373	0	1.4766	2.1851	
0.925	0.2221	0.2484	0.2693	1.3604	1.0597	4.2619	1.6063	
2.0273	4.9262	0.1467	6.0705	0.8624	2.1413	0.1556	0	
0.7274	2.5633	0.0849	1.082	0.6217	2.3809	0.3849	2.3619	
5.404	4.5561	0.1176	1.4651	2.4577	0.1226	0.5593	1.0741	
1.6116	4.1673	0.1282	0.2677	5.7703	1.5783	0.7406	0	
2.2731	0	0.7023	0.5281	0.514	5.3227	0.6757	1.0345	
4.0718	0.6385	0	0.3067	0.1661	1.4813	3.3667	3.4065	
1.5176	0.8166	4.0526	3.5017	0.1797	1.6633	1.336	0.7524	
1.0224	0.3709	1.9585	0.4517	4.7985	3.9025	0.8156	0.1923	
0.4699	0.2846							
ART4	0	0	0	0	0	0.1118	0	0
	0.0614	0.1467	0	0	0.1186	0	0	0
	0	0	0	0	0	0	0.1034	0
	0	0.1213	0.1276	0	0	0.2527	0	
0.2042	0	0	0.2047	0	0.0426	0	0	
0.1186	0	0	0	0	0.0477	0.0729	0.2426	0
	0.2636	0.1792	0.0703	0	0	0	0	0
	0.0858	0	0.0539	0	0	0	0.1156	0
	0	0	0	0	0	0	0	0

	0	0	0.0364	0	0.0059	2.6476	0	0
	0	0	0	0	0.1138	0.1728	0	0
	0							
ART5	0.3391	0.2743	0	0	0	0	0	0
	0.0502	0	0.4072	0.0338	0	0	0	0
	0	0.2119	0	0	0	0.0739	0.025	0
	0.0849	0.0601	0	0.138	0.2662	0.0688	1.1331	
0.0313	0	0	0	0.0977	0	0.7066	0	0
	0.1816	0.0215	0.0964	0	0	0	0.1086	0
	0.0498	0	0.122	0.0728	0	0	0.7397	0
	0	0.0506	0	0	0.0285	0.0402	0.0344	
0.062	0.1434	0.2701	1.1907	0.6091	0	0	0.0355	
0.0448	0.1937	0	0	0.043	0	0	0	0
	0	0	0.0687	0	0	0	0.228	
0.1357	0.2979							
ARTN	0.1759	0.1172	0.1462	0.1199	0.175	0.2102	0.6549	
0.1856	0.5957	0.0857	0.0686	0.122	0.6433	0.1418	0.3896	
0.1367	0.9823	0	0	0.1131	0	0.0626	0.0135	
0.2776	0	0.272	0.3591	0.3749	0.1415	0.2547	0.1983	
0.2008	0.0916	0.1457	0.1023	0	0.1201	0.0972	0	
0.1641	0.3055	1.2093	0	0.0954	1.8608	2.0991	0	
0.2074	0.6236	1.1409	0.1647	0.6319	0.1878	0	0.1169	0
	0.2684	0.2051	0	0.3923	0.2423	0.0727	0.2145	
0.7588	0.0742	0.3046	0	0.0894	0.1077	0.6763	0.2084	
0.1411	0.0956	0.3273	0.1156	0.0747	0.065	0.2	0.1506	0
	0	0.3008	0.2414	0.096	2.3878	0.5896	0	0
	0.1227							
ARV1	4.2632	4.6196	3.8156	5.0587	4.4736	3.6382	4.3462	
4.8581	4.0761	3.7995	4.2389	4.404	3.3715	4.7796	4.5609	
4.2011	3.5999	5.1229	5.2421	3.9835	4.3839	3.9795	4.7328	
5.0661	3.4662	4.2856	4.2819	4.2118	4.566	5.7949	3.1185	
4.8413	4.1978	5.1778	4.5963	4.3374	4.6755	4.1278	4.894	
4.6781	5.0024	4.3232	3.8868	3.6241	4.9064	3.91	4.2336	
5.1617	4.1619	4.0467	4.9082	4.5394	4.7544	3.9403	3.8968	
4.9378	3.3793	3.7507	5.3088	3.8491	3.9552	4.7431	4.1933	
4.2591	4.2293	4.1933	4.6641	4.0205	3.763	3.3068	3.6817	
4.4037	4.4775	5.1004	3.853	4.1553	5.2774	5.17	4.7197	
3.5313	4.7241	4.3308	4.4432	4.4305	4.31	4.7872	4.258	
4.4361	4.4175							
ARVCF	1.5901	1.7038	2.5369	0.3792	0.5144	0.5981	1.0516	
1.7349	0.8991	1.0333	1.0623	0.2852	1.0778	0.72	0.9608	
1.6798	1.2755	1.2861	0.9884	1.9022	1.8231	0.8231	0.5407	
0.8926	0.6677	0.7467	0.3931	2.2726	0.3432	1.4567	2.133	
1.1777	1.0417	1.3567	0.4434	0.6015	0.8132	1.8118	1.1477	
0.36	1.117	0.4501	0.2784	0.5011	0.9447	0.4661	0.8182	
0.67	1.8502	0.3955	0.5645	0	0.7976	1.667	1.0986	
1.3425	1.3784	1.4286	1.4639	1.2862	0.3534	1.6243	1.3515	
1.5233	1.2625	2.0372	1.9614	2.7988	1.5819	0.7019	0.7628	
0.6085	1.3342	0	1.5781	1.7919	1.8604	1.139	0.7409	
1.569	1.4393	0.6187	0.4828	0.4634	1.6213	0.8487	0.94	

1.3998	1.3567							
ARX	0.0917	0.0585	0.0765	0.2723	0	0	0	0
	0.1156	0.0871	0.3307	0	0.1466	0	0.0698	
0.0968	0	0.0756	0	0.0844	0	0.1587	0.0421	
0.0285	0	0.0357	1.4433	0	0.112	0.4923	0.1266	
0.0094	0	0	0.0391	0.1284	0.2077	0.1034	0	0
	0.0362	0.0885	0	0.1817	0.258	0	2.097	
0.1997	0.036	0	0.0846	0	0.1003	0.2967	0.5831	
0.043	0	0	0.2484	0.2327	0	0.3398	0.0076	
0.241	2.231	0	0	0.0529	0.1924	0	0	
0.0451	0.0142	0	0.281	0.2822	0.2318	0	0.002	
0.0287	0.0088	0.1451	0.0074	0.0492	0	0.0863	0	0
	0.0718							
AS3MT	3.165	3.5353	0.1186	1.757	0.0793	0.2727	0.1718	
1.0045	0.703	2.4833	0.0877	1.0427	3.0184	0.8404	0.8095	
0.0698	0.1117	2.988	1.8089	0.1942	1.0729	0.5436	0	
0.1155	2.2306	0.7661	0.8605	0.4241	1.5756	1.7575	3.2541	
0.3264	0.7871	1.7447	0.0265	2.3836	1.729	0	1.0252	
0.0402	1.3172	0.3631	0.1038	0.8201	1.3741	0.0745	1.9116	
0.0132	0.3192	1.4502	1.9912	1.8797	0.6645	1.0585	0.2918	
0.2612	0.1245	3.2492	1.7469	2.6075	0.1855	2.699	1.3206	
0.2357	3.6081	2.5166	0.2184	3.2086	2.3339	0.1154	0.4909	
0.9959	0.2785	0.4069	0.831	2.4517	2.0446	0.1234	0.1089	
0.2185	0.2323	2.102	1.7457	0.1532	0.8305	0.8215	3.3492	
2.9028	3.7965							
ASAH1	8.7167	7.5925	7.8646	6.1775	4.3929	6.3364	8.3216	
7.4188	8.1169	8.5707	6.9763	7.068	4.8872	8.0993	6.111	
7.6247	3.414	9.3309	7.9964	6.2467	8.1658	5.0778	6.7143	
6.7049	5.5507	5.3228	6.0641	9.0631	6.5114	8.7978	6.2149	
8.1295	7.3992	9.3309	8.4111	8.4014	6.6893	9.2477	7.4321	
6.7085	7.4789	5.0108	4.5051	7.257	5.0178	5.0237	9.3015	
6.9543	5.7622	5.0318	8.5527	5.65	9.1533	7.3017	4.7715	
6.6414	2.9566	9.1468	7.8635	8.8036	5.9698	7.5816	7.3175	
7.2954	9.0127	6.5541	8.3846	8.1047	8.626	4.7049	6.9824	
7.0871	8.9413	5.8195	7.1662	8.27	8.4459	8.0863	7.8356	
6.5256	6.8829	6.3407	7.4444	7.8201	4.3423	8.4799	9.29	
8.8478	9.1267							
ASAH2	0	0.1933	0.3281	0.1325	0.1923	0.0777	0.1598	
0.026	0.1149	0	0.0271	0.0486	0.1949	0.1893	0.0071	
0.1447	0.3569	0.1634	0.2168	0.0926	0.1996	0.0488	0.0414	
0.1825	0.1114	0.1983	0.0357	0.149	0.0589	0	0.0245	
0.0934	0.1336	0.0714	0	0.0246	0.1905	0.1436	0.0833	
0.0313	0.0747	0.0621	0.1419	0.061	0.0806	0.079	0.0914	
0.0228	0.091	0	0.0317	0.2325	0.2538	0.0404	0.0953	
0.423	0.0271	0.0915	0.0648	0.0944	0.1379	0.0339	0.0591	
0.1205	0.2066	0.2149	0	0.2537	0.1171	0.0768	0.0126	
0.0161	0.2371	0.0337	0.1409	0.123	0.1348	0.0984	0.1183	
0.0723	0.0146	0.056	0.0696	0.064	0.0744	0.1498	0.0664	
0.0763	0.0854							
ASAH2B	0	1.0412	0.9261	0.7824	1.0802	1.0031	1.2186	

1.1198	0.428	0.376	0.3552	1.0399	1.3401	0.3809	0.6327
0.9657	1.4632	0.8372	1.1669	0.6973	0.6747	0.7461	0.4585
0.8587	0.3318	0.8907	0.7128	0.6035	0.6968	0.379	0.0843
0.698	0.3591	0	0.6618	0.1654	1.5917	0.5688	0.4189
0.1946	0.9346	1.1358	1.1219	1.0425	0.4661	0.8523	0.754
0.5141	0.4928	0	0.588	1.0248	0.5233	0.8323	0.7614
1.5212	1.1666	1.3588	1.0433	0.9352	0.3208	0.2214	1.2351
1.054	0.7167	1.0411	0	0.9592	1.0574	0.8461	0.0554
0.0785	0.8607	0.1199	0.6222	0.771	1.2324	0.7597	0.6747
1.0405	0.9604	0.4781	0.4811	0.6487	0.1525	0.5404	0.5825
0.4642	0.4487						
ASAP1	4.397	3.8814	4.9322	5.1605	3.5066	5.0031	3.9303
4.3545	4.6926	4.9911	4.6237	4.3478	5.5298	4.9744	5.1654
3.89	5.3047	4.3469	5.1104	4.2336	4.3064	4.2519	4.4313
4.7248	4.977	5.5902	3.9412	4.257	4.0734	4.8091	4.3257
3.9255	4.9936	4.8618	4.7906	4.7227	4.3719	4.8176	4.3041
5.2267	4.2211	4.4103	5.2336	4.8181	6.0024	4.7263	5.2254
4.7961	4.8306	5.2466	4.83	4.084	4.9305	5.2262	4.4824
4.504	5.6396	4.9865	4.365	4.8679	4.1223	3.8948	4.7103
4.9391	6.2379	4.2746	5.4955	4.6112	4.2241	4.8713	4.8933
4.2792	4.4984	4.0664	4.31	4.7551	4.325	4.3121	5.0655
5.0003	4.8089	5.225	4.0357	4.6032	4.1379	4.2068	4.4099
4.1206	4.3127						
ASAP2	4.3171	3.6801	3.7249	3.6877	3.3889	2.7358	3.5386
2.8767	5.2102	5.1404	4.2518	4.1554	5.0333	4.2135	4.6183
4.104	3.506	5.115	4.6944	3.9184	3.893	3.4604	4.9664
3.6918	3.7681	4.1824	3.6922	3.702	4.3512	3.8441	2.8108
4.0376	4.1449	4.4501	3.0168	4.1886	3.6843	3.9695	2.9045
4.1545	4.0695	3.9412	4.4515	3.9161	5.4667	4.1562	3.8584
4.8692	5.6038	3.3538	4.4892	3.8315	4.5349	3.9141	3.234
4.0482	5.5056	2.8988	4.0807	3.1998	3.8853	4.0482	4.3219
4.1758	5.1943	4.4159	5.2659	2.4856	4.4715	4.5648	3.677
4.5179	3.8881	3.2392	5.0523	3.1853	4.0569	4.581	4.2919
3.5323	3.7443	4.5016	4.7396	4.2536	4.2092	4.0362	4.4649
3.8264	4.5898						
ASAP3	1.9568	2.4605	2.0196	1.571	0.9437	1.9243	1.5705
1.5276	2.3947	2.5436	3.122	2.5945	2.533	2.7914	2.0796
3.4258	1.2109	2.4021	1.1089	2.6606	2.3738	1.93	2.1046
1.4147	2.4872	2.763	3.3445	1.7632	2.001	2.5293	3.5066
1.5686	2.7158	3.3199	1.6539	2.3409	1.2701	3.1477	1.6809
2.869	2.5963	2.4341	2.2195	1.6556	1.6539	3.6916	1.5575
1.2277	2.6177	1.976	1.4408	1.8921	1.9232	1.6841	3.3787
1.9045	1.6596	2.4773	3.8144	2.3831	2.2768	2.0114	0.8752
2.8115	2.5539	2.6875	2.5983	1.8921	1.2802	2.0669	2.1286
2.3222	2.0264	2.7469	3.0555	2.2733	2.1386	1.4578	2.014
1.5324	3.0292	1.3881	1.2486	1.5619	3.2904	1.3937	2.3533
1.6786	2.0217						
ASB1	3.0067	2.3185	2.6579	2.6116	2.6636	1.6671	1.3045
3.6854	2.4542	2.6313	2.1591	2.507	1.2786	2.6772	2.9251
2.2671	4.6258	2.4158	2.5767	2.5767	2.4538	3.1814	3.0548

2.5239	3.0754	2.9441	2.5528	2.0676	2.5332	2.2247	2.25	
2.4901	2.4967	2.3222	2.9348	2.32	2.4149	3.0908	2.6589	
2.6087	2.2931	2.6386	4.2454	2.8899	2.6251	2.6755	2.3539	
1.1948	2.787	2.3066	2.4002	2.0687	3.3433	2.3974	2.2897	
1.8075	4.3331	3.0521	2.6844	2.8351	2.7642	1.8177	3.3496	
2.0265	2.6835	2.0783	2.4102	2.4438	2.2658	3.0556	2.4208	
2.3003	3.0652	3.3087	3.0279	2.7058	2.0754	2.7071	2.8117	
2.0271	2.7131	2.7972	2.7593	2.44	2.7816	3.3175	2.4656	
2.5767	2.6275							
ASB10	0.0879	0	0.0701	0	0	0	0	0
	0	0.0403	0	0	0	0	0	
0.0249	0	0	0	0	0	0	0	0
	0	0	0	0	0	0	0	0
	0	0	0	0	0	0	0	0
	0	0	0	0	0	0	0	0
	0	0	0	0	0	0	0	0
	0	0	0	0	0	0	0	0
	0	0	0	0	0	0	0	0
	0	0	0	0	0	0	0	0
	0	0	0	0	0	0	0	0
	0							
ASB11	0.3356	0.1375	0	0.0221	0.0457	1.0642	2.6131	
0.0776	0.1406	0.563	0.0056	0.2195	0	0.1682	0	
0.1155	0	0.2283	0.7291	0.0523	0.0275	0.1994	0.0019	
0.6638	0	0.015	0.1202	0.1818	0	0.4557	0.0325	
0.0909	0.2187	2.1489	3.2052	0.0817	0.0094	3.5051	0.203	
0.23	3.3917	0	0.0403	0.0562	0	0.0556	0.186	
0.1279	0.0038	0.047	1.2902	0.4886	0.1427	0.0915	0.065	
0.8644	0.0751	0	0.1618	0.0406	0	0.1049	3.0651	
0.0501	4.453	0.0428	1.3423	0.1522	0	0	0.0341	
0.0832	0.1813	0.053	0.051	0.85	0.1063	0.1445	0.8879	
1.009	0.0353	0	0.6558	0.4589	0.0486	0.1533	0.9616	
0.2414	0.2442							
ASB12	0.0316	0	0	0	0	0	0	
0.0055	0	0.0089	0.0027	0	0	0	0.0039	
0.0036	0	0	0	0.0282	0.0266	0	0	0
	0	0	0.0137	0	0	0.0124	0.3233	
6.00E-04	0	0.0094	0	0	0	0.0211	0	0
	0	0.0011	0	0	0.0045	0	0	0
	0	0	0.0095	0	0	0	0.3508	0
	0	0.0091	0.0065	0	0	0	0.0455	0
	0	4.00E-04	0	5.00E-04	0.0026	0	0	0
	0.0065	0	0	0	0	0.0064	0	0
	0	0.002	0	0.0081	0	0	0.0216	0
	0							
ASB13	3.7159	2.4832	3.1743	3.5087	2.903	2.4076	2.4383	
2.2634	2.7609	4.1084	3.2354	2.6952	2.1423	3.7336	1.9705	
3.2945	2.4067	4.0077	2.3644	3.3401	2.6935	3.1953	2.5297	
3.251	2.841	1.9627	2.0283	4.3255	1.8735	3.7854	3.0574	
3.2699	2.5686	3.1481	1.8241	3.7379	3.2297	2.9221	2.9684	

2.7914	3.5336	2.6449	3.7423	4.0358	2.0841	2.1559	3.6157	
3.3926	2.6757	4.1919	4.1157	3.3404	3.8525	2.7423	2.7276	
3.8447	3.1746	1.4869	3.0564	1.6538	2.9282	1.3862	3.0362	
3.7444	3.9424	2.3825	3.2567	3.6028	4.4042	3.0176	3.0399	
3.034	2.9723	2.1123	3.253	4.1501	3.9861	2.9569	2.9188	
3.6043	3.2552	3.7752	2.4759	2.9142	2.0371	3.6097	3.6795	
4.2387	3.9188							
ASB14	0.3851	0.3884	0.3202	0.3374	0.1742	0.6036	0.2166	
0.5548	0.3754	0.7827	0.2449	0.2137	0.06	0.3488	0.4256	
0.3597	0.7506	0.5639	0.4102	0.6758	0.6492	0.224	0.172	
0.5639	0.3246	0.0898	1.3225	0.2926	0.2527	0.2375	0.3699	
0.0693	0.1952	0.1805	0.2756	0.4838	0.2457	0.3739	0.2115	
0.3384	0.3016	0.1414	0.2262	0.5366	0.4122	0.1315	0.4787	
0.4949	0.2736	0.4502	0.311	0.6583	0.7066	0.3454	0.3131	
0.4954	0.3215	0.2537	0.7563	0.1844	0.1144	0.2752	0.3247	
0.5949	0.7444	0.1476	0.4479	0.2777	0.3885	0.2905	0.282	
0.3802	0.1268	0.1085	0.2291	0.3007	0.3955	0.4005	0.3086	
0.3597	0.4413	0.551	0.149	0.238	0.2065	1.0148	0.2695	
0.334	0.3914							
ASB15	0	0	0	0	0	0.0239	0.0189	0
	0	0	0	0	0	0	0	
0.0208	0	0	0.0385	0	0	0	0	0
	0	0	0	0	0	0	0	0
	0	0.0118	0	0	0	0.0216	0	0
	0	0	0	0	0.0251	0	0	0
	0	0	0	0	0	0	0	0
	0	0	0	0	0	0	0	0
	0	0	0	0	0	0	0	0
	0	0	0	0	0	0	0	0
	0	0	0	0	0	0	0	0
	0							
ASB16	0.531	0.7966	0.6696	0.4068	0.5592	1.028	0.4336	
0.79	0.5672	0.4277	0.239	0.2051	0.5993	1.0838	0.5583	
0.9244	0.669	0.6435	0.6513	0.3996	0.8446	0.3302	0.3192	
0.351	0.4075	0.812	0.3578	1.3432	0.5206	0.2291	0.6399	
1.0002	0.4988	0.4512	0.8456	0.2087	0.4173	0.5871	0.4305	
0.5179	0.4588	0.376	0.3157	0.4421	0.5662	0.6817	0.0783	
0.9092	0.376	0.4282	0.577	0.5224	0.2403	0.4012	0.5068	
0.3938	0.2259	0.7171	0.8142	0.6926	0.1693	0.3377	0.403	
0.9311	0.5676	0.4695	0.5246	0.2736	0.3573	0.9325	0.6093	
0.7239	0.3949	0.1373	0.4201	0.4778	0.4226	0.4902	0.3222	
0.603	0.5728	0.2663	0.3576	0.1598	0.7457	0.4017	0.2162	
0.8076	0.6163							
ASB17	0	0	0	0	0	0	0	0
	0	0.1622	0	0	0	0	0	0
	0	0	0	0	0	0	0	0
	0	0	0	0	0	0	0	0
	0	0	0	0.1806	0	0.0484	0	0
	0	0	0	0	0	0	0	0
	0	0	0	0	0	0	0	0

	0	0	0	0	0	0	0	0
	0	0	0	0	0	0	0	0
	0	0	0	0	0	0	0	0
	0	0	0	0	0	0	0	0
	0							
ASB18	0	0	0	0	0	0	0	0
	0	0	0	0	0	0	0	0
	0	0	0	0	0	0	0	0
	0	0	0	0	0	0	0	
0.0645	0	0	0	0	0.0725	0	0.0884	0
	0	0	0	0	0	0	0	0
	0	0	0	0	0	0.0252	0	0
	0	0.0693	0.0447	0.0327	0	0.0598	0.0273	0
	0.0447	0	0	0	0	0.0644	0	
0.0277	0	0	0	0.0574	0	0	0	0
	0	0	0	0	0	0	0	0
	0							
ASB2	0.9125	2.034	0.7702	0.6306	0.513	3.5003	1.942	
4.1031	0.8637	0.8522	1.0515	2.8357	0.1215	1.6891	2.2375	
3.0082	0.2848	1.6089	2.0208	0.6306	0.1074	1.2579	2.8473	
2.4522	0.3249	0.3688	0.3956	1.803	2.7908	3.3418	0.9614	
2.3541	2.228	1.4186	2.4779	0.3559	1.7242	2.1778	2.3611	
1.5827	3.7479	0	0.5956	1.8825	0.2494	2.1256	2.9951	
4.3216	0.7833	0	1.8011	0.9862	2.212	0.8755	0	
0.6927	0	0	0.6307	0	2.1198	2.492	3.6601	
2.1404	3.7487	0	3.3649	1.4838	3.1392	0.5525	1.8015	
2.1292	1.8162	2.1184	2.4717	2.2841	2.0299	1.7845	2.8487	
2.2353	0.6984	0.1527	3.0159	2.9189	1.3802	2.3371	1.7206	
1.4776	2.0731							
ASB3	3.3548	3.3997	2.8076	3.4581	3.437	3.1106	2.007	
2.7952	3.9497	3.2077	3.337	3.6173	3.029	3.0584	3.0495	
3.0256	3.3821	3.6625	3.1586	3.1938	2.8516	3.4124	3.5999	
3.1782	3.4001	2.85	4.6313	3.2067	3.3707	3.8025	3.7616	
2.9525	3.4567	3.5585	3.3002	3.6643	3.5139	3.4035	2.8479	
3.6971	3.0464	3.7093	3.7981	2.8266	3.6059	3.9246	3.3068	
2.9374	2.9748	3.9391	3.1664	3.5536	3.4656	3.35	3.1655	
3.4101	3.5826	2.9919	3.5506	3.2884	3.3864	3.3692	3.0121	
3.0412	3.454	3.7374	3.8103	3.0016	3.2287	3.7149	2.8035	
3.5619	3.5354	4.2594	3.4208	2.683	2.5738	3.6163	2.995	
2.6775	3.0898	2.695	2.9832	3.0006	3.3813	3.5944	3.2132	
2.908	2.902							
ASB4	0.0209	4.1525	4.1161	1.7386	0	0.0979	5.5731	
0.4746	0.0016	1.2137	2.0839	1.6976	0	0	0	
0.2283	0	0.4026	0.909	0.3701	0.1256	0.0116	0.1256	
0.1554	0	0	0.5807	0.7934	0.0149	5.4404	0	0
	0.0863	0.1598	0	3.2676	0	1.3601	3.3128	
4.8246	0.9252	0	0.0094	0.1324	0.0099	1.2954	5.7057	
1.573	0.006	0.0379	0.1812	0.1321	0.0648	3.9395	0.3569	
1.4483	0	0.1238	0	0.1215	0.5832	0.207	1.285	0
	0.9862	0	0.4851	2.3946	5.7257	0	0.581	0

	0.4329	0.2708	0.189	0	0.553	5.1979	0.6885	
0.725	0.5555	0	0.0621	0.1869	0	0.1324	0	0
	0.0339							
ASB5	0	0.015	0	0	0	0.054	0.0049	
0.0296	0	0.0217	0.0105	0.0024	0.018	0	0	0
	0.2056	0	0.0357	0.0244	0	0	0	0
	0.0036	0.292	0	0.0294	0	0.0116	0.0425	
0.005	0	0	0.0045	0.1102	0	0.0554	0.0062	0
	0	0	0	0	0.0046	0.0208	0	
0.0173	0	0.2219	0	0	0	0	0	
0.0859	0.0467	0.0414	0.0626	0	0	0	0	0
	0.0859	0.0383	0	0.0046	0.0199	0	0	0
	0	0	0	0	0	0	0	0
	0	0.0894	0.023	0	0	0	0	0
	0							
ASB6	3.0143	3.4881	3.5276	3.1152	3.8617	3.2222	2.6355	
3.8229	1.9342	2.4884	3.3295	3.5899	3.6691	2.5792	3.3466	
2.6572	3.6756	2.5111	2.582	3.6962	3.6134	3.0597	2.6424	
3.278	3.6353	2.8202	3.1204	2.986	3.0092	3.0151	3.1306	
3.8361	2.9501	3.2177	2.4998	2.4812	3.4574	3.6853	3.7223	
2.4956	3.4516	3.3934	3.0944	2.9438	2.4425	3.0782	2.7757	
2.6719	3.217	2.8486	2.8034	2.9723	3.4671	2.9917	3.1663	
3.0299	3.5274	3.239	2.7323	3.1316	3.5412	2.9621	2.5487	
2.8603	3.5159	4.154	2.5909	3.4835	3.2038	3.756	2.6392	
2.7197	3.2244	3.3189	2.5536	2.2971	3.2822	2.6515	2.8268	
2.745	2.9426	3.4883	3.8397	3.6581	2.9621	3.6332	3.2463	
3.1215	3.072							
ASB7	2.4001	2.8612	2.7327	2.6687	2.6816	2.7648	3.7753	
3.3675	2.6447	2.8371	3.1725	2.4438	2.7179	2.8346	2.249	
3.0081	2.9766	3.3248	3.0053	2.6068	2.8375	2.7029	2.5899	
2.4019	2.7923	2.5872	2.2287	2.9402	2.5157	3.4138	2.4535	
2.4316	2.906	2.4071	2.3749	2.9395	2.7914	2.6178	2.9201	
2.6012	2.5406	2.5858	2.3416	2.6872	3.4789	3.0073	3.0053	
3.1333	2.7271	3.7299	2.8171	2.3485	3.2087	3.6475	2.5098	
2.9667	3.0535	2.7568	2.6285	2.5953	2.5724	2.6248	2.7175	
3.0352	2.4308	2.7501	2.6171	2.5468	3.4507	2.1512	2.3179	
2.3288	3.0979	2.4341	2.5672	2.8454	3.0833	2.4882	3.1905	
3.1613	2.9944	2.4708	2.629	2.4358	2.4077	2.9482	2.9329	
2.4758	2.8027							
ASB8	3.2208	3.7584	3.1504	3.621	3.3309	3.3234	2.5527	
2.8092	3.411	3.5435	2.8958	2.9951	2.854	3.5235	2.6293	
3.5418	3.6295	3.6601	3.6812	3.4258	3.35	3.0786	2.9583	
2.9851	2.9644	3.5945	3.7953	3.4137	3.0321	3.5221	3.0109	
3.1069	3.2469	2.9973	3.1705	3.5838	3.0235	3.8253	3.0644	
3.5605	3.3726	3.8516	1.6984	3.1794	3.2826	3.5451	3.3581	
2.7647	2.6843	4.0663	2.9398	3.7221	3.4729	2.9765	3.0706	
2.5353	3.1187	3.0395	3.1116	2.4353	3.4012	3.9965	3.6224	
3.3026	2.9593	3.304	3.5667	3.6316	2.9467	2.7404	3.2507	
2.8293	3.0802	3.3718	3.2792	3.7606	3.5202	3.5087	3.3118	
3.5575	2.9834	3.4771	3.3142	3.1013	2.5486	3.4886	3.6322	

3.117	3.617							
ASB9	1.3331	2.2847	1.02	4.0088	3.6468	4.004	4.663	
3.8424	4.9673	2.7184	1.8994	4.0658	1.3843	3.516	2.9098	
4.2173	0.1706	3.7484	3.3614	3.0959	1.1984	3.8141	4.4949	
4.5113	3.1692	1.3164	3.682	2.6443	4.1933	3.2065	0.0296	
4.6726	4.2251	4.7785	5.24	2.465	0.7767	3.383	4.2806	
4.5895	3.9626	1.2961	2.2495	3.2086	1.3056	2.5547	3.7063	
4.7411	3.504	2.2768	4.0224	2.9278	3.924	1.8282	0.7015	
2.0302	1.7459	2.8465	1.6418	2.0166	3.2456	3.0949	5.7133	
3.7665	2.6199	0.0706	1.085	0.8893	1.737	3.1016	4.1692	
3.0642	3.151	4.0474	4.7134	2.4367	2.1868	3.7086	4.6028	
4.0512	3.3756	3.4965	3.3426	4.9808	1.9514	4.1475	1.4412	
1.6268	1.6474							
ASCC1	3.1318	4.0914	3.3477	4.2027	3.3158	4.4298	3.2018	
3.0604	3.5551	4.284	2.9998	3.8247	3.8258	3.8372	2.8455	
3.5317	3.2252	3.642	3.4071	3.715	3.5186	3.0285	3.3218	
3.2236	3.5538	2.9383	2.884	3.5182	2.9188	3.083	3.6191	
3.3162	3.3959	3.2795	3.0834	4.2589	4.1379	2.7781	3.087	
3.042	3.4884	3.9374	3.9719	3.3439	2.7013	3.1595	3.5502	
3.7207	2.5217	4.4426	3.3615	3.3206	3.0887	4.1905	5.0526	
3.8102	3.778	3.2668	3.1991	3.1384	2.9149	2.9895	3.4906	
3.7743	3.6664	3.478	3.4718	3.2466	3.124	3.519	3.2697	
3.4219	3.2492	3.5521	3.4683	3.5906	3.6543	3.295	3.198	
4.2467	3.6927	3.5859	2.2909	2.6121	3.1929	2.9483	3.6233	
3.6604	3.3926							
ASCC2	4.9747	4.6416	4.4836	4.0767	4.1744	4.0552	3.9243	
5.0212	4.5325	4.588	4.3319	4.4755	4.7505	4.2496	4.7041	
4.5507	3.6652	3.6224	4.4682	4.2973	3.6392	4.7471	4.5822	
5.5079	3.8216	4.6102	4.2054	4.5418	4.7875	5.1691	4.0315	
3.6955	4.8786	4.703	4.8623	4.1855	4.4118	4.5988	5.001	
4.5498	4.3657	4.5076	3.8313	3.8688	4.0539	4.3837	4.2004	
4.4819	4.1797	3.6024	3.804	4.3097	4.2307	4.1594	3.7673	
4.3381	3.8986	3.9327	4.4641	3.8073	4.8069	5.055	4.6008	
4.0124	4.0326	4.125	5.2275	4.3543	4.1338	3.9105	4.8521	
4.3267	4.2217	5.2412	4.7587	4.3713	4.1161	4.6801	4.4876	
3.8439	5.1007	4.5707	4.5412	4.1059	4.4797	4.4381	3.9058	
4.1615	3.9745							
ASCC3	3.2122	2.8743	3.0828	3.4288	3.5513	3.1925	2.943	
3.6591	4.0677	3.357	3.1516	2.4755	2.9738	3.8228	3.0944	
3.5465	4.1877	3.4696	4.0234	3.357	3.3036	3.5553	4.0957	
3.3484	3.6078	3.8966	3.3848	2.288	4.0797	3.4601	4.2313	
3.1232	2.9817	2.3299	4.1842	3.7736	2.4276	2.7678	3.0871	
4.7447	2.7305	3.6046	3.6939	3.2821	4.3588	4.4454	3.7012	
2.8786	3.3808	3.6942	3.2572	4.1952	3.1734	2.6447	3.8315	
2.9827	4.8336	3.232	3.8569	3.573	3.9078	4.1619	3.167	
2.7306	2.5743	3.8632	3.1832	3.024	3.7648	3.7691	3.5936	
3.6126	2.9823	3.9364	4.0072	3.0268	2.8842	3.4187	3.0126	
3.1553	3.1773	3.4284	3.6803	3.8253	2.7385	3.0385	3.6144	
3.5604	2.9936							
ASCL1	0	0	0.096	0	0	0	0.1378	0

	0.0454	0	0.0493	0.0123	0	0.0799	0	
0.0179	0.6397	0.0424	0	0.0513	0	0.0611	0.0268	
0.1281	0	0.0207	0	0	0.026	0.0282	0	
0.749	0	0.1122	0.0066	0	0.0355	0.1014	0.0201	0
	0.2325	0.1557	0	0	0	0	0	
0.035	0.276	0.9884	0.0263	0	0	0	0	
0.1445	0	0	0	0.0258	0.5909	0.011	0.0169	
0.0835	0.6262	0	0	0.1315	0.0631	0	0	0
	0.0626	0.1785	0.0935	0	0	0.0894	0.0052	
0.1083	0.1194	0.0319	0	0.0872	0	0.0688	0	0
	0							
ASCL2	0	0	0	0	0	0	0	0
	0	0	0	0	0	0	0	0
	0	0	0	0	0	0	0	0
	0.071	0	0	0	0	0	0.5626	0
	0	0	0	0.1388	0	0	0.1849	0
	0	0	0	0	0	0.1436	0	0
	0	0	0	0	0	0	0.088	0
	0	0	0	0	0.066	0	0	0
	0	0.0219	0.1237	0.0261	0	0	0	0
	0.0859	0	0	0	0	0.0759	0	0
	0.0681	0	0	0	0	0	0	0
	0							
ASCL3	0	0.132	0	0	0.1413	0.3263	0	0
	0	0.0931	0	0	0	0.189	0.0745	
0.0725	0	0	0.2364	0	0	0	0	
0.102	0	0.0858	0	0	0	0	0	0
	0.1116	0	0.0482	0	0	0	0	0
	0	0	0	0	0.0682	0.0972	0	0
	0	0	0.0905	0	0.1761	0	0.1437	0
	0	0	0.1451	0	0	0	0	
0.1746	0	0	0	0	0.195	0.2049	0	0
	0	0	0	0	0	0	0	0
	0	0.0841	0	0.1274	0	0	0	0
	0							
ASCL4	0	0	0.4623	0	0	0	1.407	0
	0	0	0	0	0	0	0	
0.2111	0	0	0	0	0	0	0	0
	0	0	0	0.5602	0	0.5143	0	0
	0	0	0	0	0	0	0	0
	0	0	0	0	0	0	0.1575	0
	0	0	0	0	0	0	0	0
	0	0	0	0	0	0	0	
0.0251	0	0	0	0.1587	2.1032	0	0	0
	0	0	0	0.5251	0.0813	0	0	0
	0	0	0	0	0	0	0	0
	0							
ASCL5	0	0	0	0	0	0.1591	0	0
	0	0	0	0	0.1104	0	0	0
	0	0.1464	0	0	0.111	0	0	0

	0	0	0	0	0	0	0	
0.0575	0	0.0942	0	0	0.0531	0	0	0
	0.0644	0	0	0	0	0	0	
0.1003	0.2419	0.2685	0	0	0	0	0.1648	0
	0.047	0.0966	0	0	0.0148	0	0.0348	
0.0687	0.0646	0.1493	0	0.0122	0.0635	0.1716	0	
0.0947	0	0	0	0.0361	0	0	0.024	0
	0	0	0.033	0.1434	0	0	0	0
	0							
ASF1A	2.7427	3.3376	2.8907	3.8686	4.0967	3.2245	4.2018	
4.394	3.3297	2.9446	2.3822	2.8111	3.0543	3.8921	3.5914	
2.8396	3.9568	3.2245	3.8508	3.3658	3.4898	3.8198	3.975	
3.7667	4.8193	3.8482	2.3581	2.5127	3.6911	2.7668	4.1197	
3.4578	3.3164	2.828	3.3976	3.6026	4.341	3.1863	3.3863	
3.7579	2.4216	3.6045	4.1754	2.2176	3.8136	4.145	2.432	
3.4841	3.3673	4.2773	2.8469	3.2383	2.6932	3.4499	4.9814	
3.3682	4.1862	3.7306	3.0125	4.0473	3.0068	3.2671	3.4035	
2.7564	2.5911	3.6918	2.7333	2.661	3.5387	3.3609	3.0469	
3.5585	3.5378	3.5549	3.7502	2.6348	3.4458	2.7045	2.6996	
2.8808	3.1336	3.296	3.6642	2.8884	3.8166	2.7301	3.242	
3.1784	3.4926							
ASF1B	4.75	5.7892	5.1395	4.7786	5.6485	3.4542	3.5774	
4.4384	4.9028	4.4847	3.5887	4.1185	5.8921	5.4851	4.9938	
2.5465	3.0095	4.3379	5.1189	4.8055	5.3256	4.2117	4.0034	
5.2002	4.5693	2.965	1.5236	5.2365	4.9849	4.772	5.4789	
5.0889	5.0738	4.8636	4.8654	4.6154	5.0326	2.2937	4.6734	
4.0538	3.6307	3.1692	5.0532	5.1742	4.8697	5.6416	4.1158	
5.3349	3.4531	4.6504	4.5627	3.8915	4.0485	3.7463	5.0826	
5.2781	4.04	5.558	4.3246	5.63	4.3705	4.4216	4.3995	
5.3629	4.3475	2.0636	4.7012	2.2916	5.0637	5.1457	4.0628	
5.2425	4.7374	4.0084	3.3552	3.2812	5.159	3.3498	5.3696	
4.4971	5.1041	2.3722	2.5416	3.6811	5.6944	4.9647	3.4999	
4.9386	4.4546							
ASGR1	0.7072	0.1399	0.1007	0	0.3034	0.1108	0.1758	
0.1932	0.114	0	0.1409	0.0742	0.0888	0	0	
0.0565	1.0529	0.337	0	0	0	0.1496	0.1107	
0.1024	0.2195	0.0957	0.2765	0.1144	0.1779	0.6882	1.3151	0
	0.3068	0	0	0.1081	0.1106	0.1708	0.0956	
0.1463	0.19	0.267	0.6816	0.1346	0.1381	0.143	0	
0.2547	0.2176	0.6111	0.1614	0	0.2327	0.2587	0.7783	
0.3297	1.4085	0.1748	0.0098	0.2351	0.1494	0.1672	0.0284	
0.2162	0.1902	0.3383	0.0953	0.3765	0.1804	0.3178	0.282	
0.0256	0.0423	0.1582	0.1853	0.0021	0.2954	0.0302	0	
0.3934	0.2657	0.0762	0.0156	0.1603	0.0895	0	0.1369	
0.1365	0.0952							
ASGR2	0	0	0	0	0	0	0	0
	0	0	0	0.0296	0	0	0	0
	0	0	0	0	0	0	0	0
	0	0	0	0	0	0	0	0
	0	0	0	0	0	0	0	0

	0.0518	0	0	0	0	0	0	0
	0	0	0	0	0	0	0	0
	0	0	0	0	0	0	0	
0.0418	0	0	0	0	0	0	0	0
	0	0.0016	0	0	0	0	0	
0.055	0	0	0	0	0	0	0	0
	0							
ASH1L	2.7686	2.5813	2.7046	3.5888	3.2083	3.1243	3.1995	
2.7902	3.1356	2.9052	3.2689	2.6454	2.3817	2.8761	3.0946	
2.7895	3.1727	3.7581	3.0574	2.2712	2.568	2.8204	2.6612	
2.844	3.5334	3.3415	3.0164	2.3403	2.7741	3.2792	3.5474	
2.7544	2.286	2.3412	2.3795	2.8692	2.9807	3.0497	2.8811	
3.6225	2.7978	2.8403	2.9043	2.897	3.8967	3.9496	3.0534	
3.4027	2.3487	3.2458	2.5503	3.619	3.1023	3.3129	2.8375	
2.7654	3.0826	2.7199	3.0942	3.0606	2.7846	2.5386	3.3512	
2.6384	3.0867	3.3523	2.4634	2.7916	3.8199	3.0661	2.3532	
2.633	2.5216	2.585	3.1186	2.4271	2.7766	3.6795	3.1125	
2.4835	2.5948	2.7219	3.3825	3.492	2.2686	2.3959	2.8315	
2.3529	2.4975							
ASH2L	4.2882	3.8871	3.8821	4.5461	4.3794	4.9019	3.7433	
4.9739	4.5066	3.7928	3.5564	3.6556	4.2997	5.0659	3.9485	
4.1039	4.225	4.7384	4.7148	3.9248	4.3571	4.5393	4.0518	
4.0608	6.0252	3.494	4.0758	4.2666	4.5764	4.004	4.4068	
4.3828	4.971	4.6277	4.83	4.0506	3.9902	4.3454	4.4239	
5.143	3.8283	4.0405	4.1716	4.4064	4.5549	4.2236	4.7554	
3.525	4.2213	5.1201	4.3672	4.2226	4.8769	3.6986	4.37	
4.5005	3.1769	4.5886	4.2375	4.7869	4.0346	4.1757	4.8795	
4.6529	5.1045	3.1033	4.2798	4.353	3.8126	5.2768	4.7622	
4.2265	4.605	4.905	5.0239	4.3572	4.2857	4.5041	3.6725	
3.9645	4.2316	4.1009	3.6978	4.5796	4.7476	4.9592	4.5333	
4.5265	4.3721							
ASIC1	2.6312	2.6673	2.1308	2.2014	2.3881	1.0677	1.4005	
2.2858	1.8394	1.6945	3.7302	2.3575	1.0938	1.4941	1.3959	
2.2888	2.8772	1.2152	2.2408	2.2511	0.8661	1.7499	1.689	
2.0314	2.0319	1.7701	3.5818	2.5368	1.3558	3.0114	3.2387	
1.5821	2.1722	2.4375	1.0714	1.7671	1.9357	2.1088	1.4484	
3.3581	2.0013	2.657	1.8624	2.6592	2.732	1.174	3.0596	
0.8406	2.2362	3.2139	2.2215	1.716	2.4155	2.3763	2.2322	
3.0024	1.693	2.3124	0.1949	1.6809	2.3808	1.2553	2.4896	
1.6748	2.2677	1.7845	2.6278	1.5828	2.2234	2.2965	3.2288	
1.4981	2.8288	2.3598	3.0913	0.6419	1.512	2.4352	2.3903	
3.0854	2.2137	2.5556	1.5152	2.7443	0.7785	2.1017	1.3342	
1.6565	1.5383							
ASIC2	0	0	0	0	0.1102	0	0	0
	0.043	0	0	0	0	0	0	
0.8135	0	0	0	0	0	0	0	
0.0749	0.0939	0.0445	0	0	0	0	0	
0.0247	0	0	0	0	0	0	0	0
	0.1014	0.0258	0.3056	0	0.3087	0	0	0
	0.0733	0	0	0	0	0	0.0035	0

	0.23	1.2904	0	1.2381	0	0	0.0589	0
	0	1.00E-04	0	0	0.0156	0.17	0	0
	0	0.0576	0.0082	0.0012	0	6.00E-04	0.011	0
	0.0089	0	0.0147	0	0	0	0	0
	0							
ASIC3	0.7961	1.1067	0.4905	0.5293	0.9548	1.1596	1.5384	
0.379	0.7061	0.4086	1.2721	0.2381	0.5442	0.7989	0.6001	
0.9869	0.4181	1.106	0.2787	1.0691	0.9739	0.2128	0.571	
0.7234	0.6542	0.433	0.1478	2.0949	0.5137	1.4477	1.2876	
0.7863	0.2641	0.4562	0.5756	0.2321	0.7612	0.6438	0.3972	
0.3607	1.8395	0.2088	0.5489	0.4285	0.1278	0.6387	0.2832	
1.2133	0.5269	0.4476	0.9667	0.1305	0.4162	0.6201	1.4821	
1.6102	0.4657	0.2796	0.7195	0.3688	0.6777	0.7906	0.5092	
1.2135	1.3577	0.8078	1.5873	0.4292	0.9989	1.3774	0.0204	
0.8277	0.2146	0.1619	0.4052	0.1243	1.0492	0.4371	0.1868	
0.3338	0.7811	0.2295	0.7552	0.2357	1.0027	0.7431	0.3787	
0.2651	0.6271							
ASIC4	0.1974	0.074	0.2198	0.125	0.3792	0.6228	0.2019	
0.3632	0.0882	0	0.0942	0.0226	0.0255	0	0.9398	
0.2503	0	0.0578	0	0	0.0505	0.149	0.1059	
0.3583	0.099	0.0504	0.1636	0.2668	0	0.123	0.0459	
0.0528	0.0691	0.0938	0.1692	0	0.0746	1.5733	0	
0.1002	0.2198	0.0573	0.1226	0.0402	0.0559	0	0	
0.1521	0.0288	0	0.1129	0	0.1355	0.5393	0.2419	
0.0866	0.0076	0.0556	0.0731	0.0503	0.0686	0	0.9239	
0.0927	1.2505	0.0803	0.0726	0	0.2512	0.3654	0	0
	0.0038	0.0504	3.00E-04	0	0.1546	0.0141	0.3915	
0.2958	0.1149	0	0.1842	0.144	0.228	0.0494	0.046	0
	0							
ASIC5	0	0	0.0455	0	0	0	0.011	
0.0146	0	0.018	0	0.0178	0.0316	0	0	0
	0.1453	0	0	0	0	0.0204	0	
0.0113	0	0	0	0	0	0	0	
0.0147	0.1526	0.0194	0.0983	0	0	0	0	
0.0739	0	0.0256	0	0	0	0.0963	0	0
	0.0438	0.1107	0.0179	0.0605	0	0	0	0
	0	0	0.05	0	0	0	0	0
	0	0.1112	0	0	0	0	0	0
	0	0	0	0	0	0.0962	0.066	0
	0	0	0	0	0	0	0	0
	0							
ASIP	0.6357	0.2601	0	0.3226	0.3789	0	0	
0.1804	0.3423	0.1962	0.1403	0	0.153	0	0.1786	
0.1691	0	0	0.9768	0	0	0.1386	0	
0.7082	0	0	0	0.3113	0	0	0	
0.1819	0.2251	0.2243	0	0	0.1423	0.7312	0	
0.2457	0.1579	0.098	0.5299	0.2154	0	0.3863	0	0
	0.1656	0	0	0	0	0.1962	0.287	0
	0	0.0798	0.3557	0.3934	0	0.1834	0.0652	
0.2343	0.205	0	0	0.078	0.5333	0.2907	0	

0.2833	0.0786	0.0287	0.112	0.146	0.6024	0.2657	0.3629	
0.5959	0.6047	0.0253	0	0.2635	0	0	0.2235	0
	0.3973							
ASL	3.5276	2.9249	2.9854	2.5572	3.7203	2.5636	2.9882	
3.7494	3.2775	3.6397	4.116	1.6275	3.8969	3.1213	3.4313	
3.7706	3.1914	3.5441	3.6858	4.0315	3.8368	4.0203	4.1776	
3.6379	4.0133	4.8746	1.5553	3.1712	4.272	2.4624	4.2463	
4.0293	3.3362	3.4423	4.0108	2.8457	3.2515	3.7574	4.0065	
3.4862	4.0368	3.3137	3.9559	3.1577	2.233	4.1314	2.5131	
3.6485	4.2733	2.5693	3.2327	2.896	3.3807	2.9341	3.6448	
3.8893	4.0166	4.1128	3.6224	3.6416	4.0515	3.8411	2.4574	
3.957	3.0214	3.8421	3.0496	3.5767	2.5223	3.6831	3.0112	
3.899	3.718	2.4584	3.5034	3.1982	3.469	2.7697	3.502	
2.9776	3.8745	2.5877	3.5474	2.7386	4.7171	4.518	3.416	
3.3431	3.1225							
ASMT	0.138	0.4128	0.1302	0.0234	0.0757	0.0219	0.0081	
0.0371	0.0698	0.0622	0.0071	0.007	0.0083	0.0255	0.0079	
0.0078	0.023	0.0651	0	0.0242	0.0235	0.0066	0.0188	
0.0111	0.0077	0.0093	0.0508	0.0268	0.0129	0.0135	0.0085	
0.0489	0.0144	0.0879	0.0265	0.0366	0.0069	0.0192	0.0333	0
	0.0353	0.0061	0.0212	0.049	0.0323	0.0459	0.0673	0
	0.0374	0.1236	0.0113	0.0144	0.0298	0.0344	0.0411	
0.0975	0	0	0.064	0	0.2688	0.041	0.0689	
0.0486	0.0028	0.0449	0.0096	0.0546	0.0504	0.1022	0.0241	
0.046	0	0	0.0365	0.0472	0.149	0	0	
0.0074	0.0452	0	0.0886	0.0757	0.0066	0.0191	0.0756	
0.0224	0.0275							
ASMTL	3.2765	1.2778	2.0932	0.842	1.8791	2.9803	2.0413	
2.7329	1.9151	1.4	3.6762	3.0855	3.5201	1.6956	3.3645	
2.7828	2.5834	2.16	1.0961	1.8972	1.6443	2.325	3.7653	
1.7909	1.8203	1.7244	3.36	1.9098	2.206	2.308	3.3927	
2.3259	2.2005	4.7435	2.3017	1.0658	2.7953	3.5138	4.4582	
3.1055	4.3317	2.746	3.4575	3.0401	3.5189	2.299	2.0248	
1.4812	3.4234	3.0933	4.0847	1.3403	3.0737	1.6678	3.4954	
1.6077	2.6417	2.3672	2.0537	2.7439	2.5225	2.635	1.7443	
2.1487	3.1327	3.7195	4.9252	3.3839	1.0866	3.5556	3.7241	
2.0689	1.9823	3.6145	2.1364	1.4733	2.6145	3.4433	1.4392	
2.8673	2.4388	4.4052	1.5295	3.5173	2.308	3.4326	3.5958	
3.5936	3.7646							
ASNA1	4.8763	5.9596	6.1198	4.7904	5.7984	5.5842	5.4029	
5.6413	5.098	5.4941	5.4218	5.0321	6.1256	6.0574	5.8584	
5.6753	5.0992	4.7117	5.3442	6.0387	5.6824	5.2462	5.3558	
5.935	5.2351	5.4888	4.5893	5.5342	5.8555	5.4557	5.3545	
6.4433	6.0163	5.588	5.4534	5.1861	5.8986	6.0564	5.2718	
4.9835	4.5454	5.283	4.9454	4.9223	4.9599	4.743	4.8416	
5.2565	5.5389	4.9084	5.4269	4.4313	4.5813	5.1203	5.6937	
5.6556	4.9266	5.4909	7.254	5.6063	5.5881	5.6168	5.0792	
5.6207	5.5348	5.4228	6.2727	6.1525	5.5818	4.8308	5.6907	
5.7188	5.643	4.8325	5.1953	5.7918	5.989	5.2238	5.1578	
5.2654	5.0425	5.1418	5.1862	4.9189	5.8456	5.3272	5.8996	

6.1052	6.3018							
ASNS	7.1001	5.2896	5.2085	5.275	5.2085	4.414	4.762	
5.5044	4.3763	7.859	5.4267	4.2586	7.5163	5.2286	4.8731	
4.5552	7.0443	5.0463	4.7838	5.6445	3.4097	6.5358	3.4266	
5.1256	6.1343	5.5971	4.2223	4.1484	4.5048	4.5494	6.4068	
4.3712	4.5226	3.71	5.7342	5.4233	8.0384	6.7223	8.477	
4.4665	6.3425	3.0685	5.7237	4.807	3.6503	4.817	6.1617	
6.4655	5.1851	2.2416	6.0193	5.1335	3.9949	8.4965	6.823	
6.3529	7.1966	5.1707	5.4984	6.0576	5.1098	4.928	5.4847	
5.6173	4.9482	5.4112	5.2408	6.475	3.5185	3.9452	4.8115	
4.7222	4.9429	3.9374	4.0845	5.2354	3.737	4.1278	3.4815	
4.208	7.0435	2.6821	2.7797	4.5024	4.0276	4.7699	4.5813	
5.1335	2.8901							
ASNSD1	4.7164	4.3632	5.1035	4.6439	3.8658	4.7853	4.8181	
5.1509	4.307	4.3852	4.4056	4.4472	4.9407	4.9202	4.66	
4.8938	4.4311	4.9191	4.5152	4.4541	4.9344	3.9818	4.1531	
4.7447	4.3088	4.9259	4.1531	4.8361	4.2836	4.5387	4.2346	
4.3216	4.7301	4.1791	4.2793	4.622	4.506	4.2262	4.297	
4.9626	3.9462	3.7375	4.1272	4.1377	4.7814	4.6733	4.6858	
4.9489	4.497	4.8289	4.4909	5.0483	4.8452	5.1567	4.7166	
4.9983	4.1848	4.5571	4.6048	4.3638	4.3335	4.2385	3.9679	
4.1671	4.919	4.207	4.2045	4.4268	5.2082	4.7034	4.412	
4.545	4.3513	3.8255	4.5893	4.892	4.4989	4.4215	4.361	
4.2018	4.538	3.8922	4.5186	4.2288	4.6243	4.9369	4.3746	
4.5133	4.3507							
ASPA	2.1799	0	1.1269	0.8689	0	2.8803	0.5926	0
	3.1334	1.9684	0.1172	1.6567	0	1.9142	1.6284	
2.4836	0	2.9747	1.4584	2.111	2.5879	1.041	2.5525	
0.9649	0	0.2059	0.488	0.3225	2.2558	0.3359	0	
0.0878	1.0235	2.5745	2.8257	0.9244	0.1876	0.4824	0.1314	
2.4962	2.9274	0.6469	0	2.4623	0.0224	0.046	1.636	
0.9153	0.1565	0.0993	2.6412	2.0963	1.909	2.5803	0	
1.8148	0	0.3413	0.2266	0	1.3515	1.3786	0.5157	
1.3284	0.2932	1.1855	0	0.4851	1.7145	0	1.2529	
1.1372	2.3618	2.5594	0.5031	0.7284	0.6363	2.451	1.3162	
1.3082	0	3.1878	1.6057	4.8363	0	1.9792	3.6063	
3.4555	3.8189							
ASPDH	0.181	0	0	0	0.08	0	0	0
	0	0.0959	0.0493	0.0476	0.1044	0	0.0638	0
	0	0	0	0	0	0	0.034	0
	0	0	0	0	0	0.1845	0.0517	0
	0.0878	0	0.0882	0	0	0.0935	0.0647	0
	0.1683	0.0388	0	0	0.1247	0	0	0
	0	0	0	0	0	0	0	0
	0	0	0	0	0.07	0	0	
0.0811	0	0.1079	0	0	0.0491	0	0	
0.0531	0	0	0	0	0	0	0.0905	0
	0	0	0.0572	0	0	0	0.1122	
0.0977	0							
ASPG	0	0	0	0	0	0	0.7943	0

	0	0	0.1377	0	0.2029	0	0	
0.0783	0	0	0	0	0	0	0	0
	0.0273	0.0359	0.1584	0	0	0.0642	0.4617	0
	0	0	0	0	0	0.2197	0.2671	0
	0	0	0	0	0	0	0	0
	0	0	0	0	0	0	0	0
	0	1.0454	0	0	0	0	0.0213	0
	0	0.2711	0.036	0.0249	0	0	0	0
	0.0285	0	0	0	0.0745	0	0	0
	0	0	0	0	0	0	0	0
	0							
ASPH	5.2552	4.6601	5.2006	7.4668	6.0375	6.4117	5.5836	
5.9826	5.7484	5.5763	5.7015	6.0786	5.6559	5.7911	6.5395	
4.0744	8.1803	5.1681	5.8367	5.4522	6.0124	6.2298	7.7909	
5.8913	8.5842	5.5107	6.5655	5.129	5.5685	5.5203	7.3341	
5.6818	6.1648	6.8455	7.3447	6.7084	6.3389	6.0728	5.265	
7.0668	6.0666	8.6643	7.7241	5.8212	7.1801	8.0697	6.2269	
5.5852	5.8164	6.6516	7.1057	7.2687	6.3427	4.8977	8.3676	
5.9567	8.2818	5.4512	5.945	4.863	7.1856	5.6346	6.1703	
5.3568	6.4497	8.0485	6.0407	5.2705	4.3445	6.051	6.6913	
5.4049	5.5097	7.4837	5.9989	5.4197	5.7134	6.6406	7.1156	
6.6838	5.6796	6.7885	5.5028	6.9487	6.6974	6.0792	6.2112	
5.5908	5.4414							
ASPHD1	1.6975	0.9331	1.0864	1.164	2.8217	0.8889	2.291	
2.7482	0.6327	1.4492	2.5717	1.5044	2.7694	1.0471	4.7142	
0.2486	2.3128	1.7419	0.6267	1.3492	1.4699	3.1159	3.4916	
1.0846	1.7894	2.4171	2.894	1.5717	2.4122	0.7494	1.8089	
3.3873	3.3699	3.9558	1.4582	1.1392	3.0427	2.3872	1.3769	
1.6857	1.8911	3.1541	4.0578	2.0169	0.5251	1.546	1.4017	
0.9105	5.415	1.1052	3.7598	1.5684	2.257	1.4972	2.8091	
2.3504	0.9137	3.3637	2.1521	2.6083	2.374	0.2574	1.5534	
1.802	3.4935	3.1062	2.9972	2.6662	0.4337	2.9261	2.793	
2.0419	3.0805	3.4497	0.3806	0	0.7633	0.535	1.4281	
2.8598	1.697	2.5055	2.9298	3.8059	3.2861	2.634	2.3838	
2.8226	1.9463							
ASPHD2	2.1817	1.9801	2.1429	1.4378	0.9915	1.5885	2.029	
1.5489	2.574	2.0469	3.2164	1.6012	2.6303	2.0533	2.2313	
2.4817	1.457	1.9225	2.1817	2.4741	1.6857	1.8888	1.9699	
2.0612	1.387	2.3684	1.381	2.1641	1.4612	2.6024	1.42	
1.7034	2.3107	1.6176	1.5753	2.151	1.5843	1.646	1.6342	
1.3145	1.165	0.8804	2.5293	1.4668	1.6936	1.0745	1.5198	
1.9525	2.0779	0.961	1.5965	1.1541	2.3996	1.5997	1.3679	
1.3003	2.7031	1.6644	1.6349	1.7639	1.2571	1.6452	1.5686	
2.1512	1.1601	2.7937	2.0116	1.4957	1.5873	2.1402	1.473	
1.8584	1.4499	1.7481	2.2796	1.8969	1.8259	1.3112	2.2756	
2.2935	2.6691	2.4334	1.1346	2.2242	0.5437	2.3312	1.3894	
1.5022	1.3152							
ASPM	3.0729	3.44	3.5811	4.3854	4.1569	4.099	1.8749	
2.6091	4.4854	3.8545	1.8908	3.8528	3.7311	4.0901	4.3043	
3.1868	3.2283	3.8515	3.7022	3.1279	3.887	3.7445	3.5915	

3.4791	3.7324	2.1677	2.5493	3.0214	4.1205	4.0094	3.8991	
3.1061	4.1602	3.3438	4.0051	4.0011	3.1696	1.3246	3.4295	
4.1975	2.76	2.5877	4.5725	3.7782	4.2673	4.3825	2.7577	
4.3173	2.989	3.9473	3.5617	2.703	3.6143	1.9964	3.7993	
4.3978	3.8438	3.9491	3.958	3.9715	3.5429	3.2668	3.9959	
4.2605	4.2558	1.4599	3.5448	1.1709	3.7606	3.8934	3.5571	
3.9509	3.4514	3.505	3.4473	2.7453	3.7615	3.4199	3.8904	
3.1364	3.6679	2.376	1.5872	3.3659	4.1129	4.234	2.9623	
4.0312	3.9422							
ASPN	0.1544	0.4252	0.4667	0.0717	0.0757	0.2373	0.0293	
0.0568	0.0202	0.0252	0.0889	0.0545	0.0586	0.4445	0.0298	0
	0.2579	0.2968	0.0707	0.0952	0.1972	0.024	0.1052	
0.0446	0.3709	0.345	0.1132	0.1092	0.028	0.0633	0.1007	
0.0193	0.2547	0.1677	0.1389	0.0718	0.0674	0.1179	0.1101	
0.1073	0.054	0.1088	0.0443	0.2569	0.1024	0.1408	0.0611	0
	0	0.1936	0.1861	0.0768	0	0.0593	0.0977	
0.2882	0.2256	0.0358	0.1426	0.1534	0.0952	0.4085	0.0728	
0.0511	0.1587	0.0461	0.3214	1.0461	0.0432	0.0528	0.0605	
0.239	0.0477	0.0724	0.107	0.1264	0.4146	0.0153	0.1852	
0.3737	0.229	0.1269	0.0119	0.0611	0	0.0304	0.1126	
0.0558	0							
ASPRV1	0.2912	0.3457	0.1556	0	0.2294	0.6398	0.091	
0.1592	0.2164	0.0342	0.1257	0.0386	0.2278	0.2705	0.3708	
0.4072	0.1393	0.1671	0.54	0.0803	0.0681	0	0.2184	
0.1265	0.0727	0.0265	0.0731	0.2473	0.0118	0.1572	0.4562	
0.1185	0.4805	0.5465	0.2344	0.0609	0.0179	0.176	0.5264	
0.2235	0.2652	0.1852	0.246	0.1852	0.1817	0.162	0	
0.1792	0.6627	0.8175	0.0753	0.2347	0.1985	0.5745	0.2017	
0.3939	0.1619	0.0826	0.1565	0.1383	0.1701	0.0964	0.4556	
0.404	0.1859	0.124	0.7536	0	0.306	0	0.0433	
0.4328	0.0499	0.1704	0.3624	0	0.1569	0.0768	0.1949	
0.5318	0.0728	0	0.1116	0.1025	0.3043	0.1478	0.1748	
0.257	0.2405							
ASPSCR1	3.732	3.8306	4.0592	1.6098	3.4188	3.3041	3.166	
3.8158	3.1191	3.1574	3.8727	2.4579	3.4475	2.9704	3.3316	
3.6801	3.6907	2.5792	3.0295	3.3055	2.9627	3.4282	3.1623	
3.2708	3.0226	3.3581	2.6194	4.3201	3.37	3.5343	3.7259	
4.5811	3.2932	3.6856	3.5793	2.463	4.5002	4.2337	3.4077	
2.3455	3.5384	2.4158	2.7345	3.2386	3.2258	2.7716	2.8823	
3.7334	3.8996	2.7537	3.61	2.2262	2.5619	4.1277	4.1727	
4.4756	2.7976	2.9672	3.0906	2.9734	3.5552	3.2484	3.5019	
3.1402	4.5756	3.2767	4.1052	3.3893	3.5624	3.5313	3.3367	
3.2042	3.6424	2.8812	3.3031	3.8567	3.9963	3.0867	3.3149	
3.0315	4.2067	2.6611	3.8926	4.4256	4.4101	3.3545	3.0031	
3.4131	3.3833							
ASRGL1	2.113	4.664	3.4919	2.6371	1.6953	1.5233	2.0679	
2.9182	4.3606	1.7622	2.3002	2.3482	1.7782	2.271	1.7954	
3.965	1.4141	3.7028	5.2992	2.2425	1.4975	1.3634	1.6942	
3.096	1.8698	0.1396	1.0406	3.2385	1.1729	2.6511	4.331	
3.3206	3.0771	2.9579	2.1489	2.7735	1.7832	2.023	1.8965	

1.2205	1.4472	0.4491	0.7763	2.484	1.8916	0.2182	2.1008	
1.9113	0.9872	1.5393	2.785	1.3613	2.5227	3.1654	4.1331	
3.0844	0.6309	3.8305	1.5228	3.5169	1.2637	3.6187	2.5117	
1.9792	1.4731	1.0453	2.3505	0.6334	2.9112	1.4101	0.6068	
1.7019	3.0107	1.6459	1.595	1.4208	3.4538	2.4037	4.1508	
3.041	4.0526	0.6068	2.7073	1.0637	1.6438	2.8303	0.8408	
1.2593	1.4524							
ASS1	5.5896	0.2596	0.3255	1.9973	0.092	0.2358	0.0286	
1.6359	0.1381	5.1946	3.2421	0.585	0.7223	0.4778	0.6766	
0.559	0.1665	3.0095	0.6138	0.5407	0.5002	0.634	2.6446	
0.1627	1.8587	0.7072	0.1446	0.1232	0.4717	1.181	8.1142	
0.5568	0.2623	0.9915	0.3788	4.9	4.4323	0.634	6.4515	
0.0748	0.2609	6.3081	0.0472	0.2218	1.6843	3.2995	3.3002	
0.2518	0.2623	0.019	0.7873	2.8117	0.2311	6.3512	9.452	
3.1249	4.3247	1.457	0.7861	2.9081	0.3272	1.2198	0.6242	
0.5606	0.3927	0.881	0.4343	0.3653	1.2385	0.5021	0.4153	
0.3732	2.9592	1.8017	0.3352	1.3744	0.5617	0.3466	0.4355	
0.6906	0.4981	1.0819	0.7216	0.4596	0.7419	0.4492	1.5628	
0.0887	1.0279							
ASTE1	1.8093	2.3655	1.8778	2.1354	1.8023	1.4266	1.8413	
1.8496	2.0431	1.909	2.0142	1.4983	1.7346	2.4765	1.7721	
1.5392	1.8978	2.3206	2.213	1.3701	1.9567	1.9669	1.5208	
1.7916	1.2615	1.7187	1.8124	2.0777	2.2958	1.8691	1.4096	
2.1083	2.2461	1.9839	1.8093	2.213	0.9819	2.0051	1.9168	
2.6417	1.6443	1.9069	1.4893	1.9397	2.4765	2.3959	2.7801	
2.0482	1.4058	2.8747	1.814	2.3875	2.197	1.7599	1.7242	
2.2063	1.964	2.0846	1.6571	2.4433	2.4876	2.0374	1.301	
2.0657	2.1409	1.6969	1.7648	1.9441	2.0092	1.3295	1.5457	
1.9043	1.8701	1.7114	0.9389	1.6529	2.3069	1.8476	1.9018	
2.126	2.0846	1.0983	1.362	1.5767	2.2383	2.1506	1.6279	
1.6222	1.6345							
ASTL	0	0	0	0.1151	0	0.1007	0	0
	0	0	0	0	0	0	0	0
	0	0	0	0	0	0.0315	0	0
	0	0	0	0	0	0	0	0
	0	0	0	0	0	0	0.0473	0
	0.0853	0.0208	0	0.0661	0.045	0.2406	0	0
	0	0	0	0	0	0.0304	0.0996	0
	0	0.1665	0	0.0915	0	0	0	0
	0	0	0	0	0.0604	0	0	0
	0	0.0031	0	0	0.0233	0	0	
0.0774	0	0	0.0691	0	0	0	0	0
	0							
ASTN1	0	0	0	0.0591	1.1469	0	0	
0.0415	0	0	0.1108	0.0448	0.5038	0	1.7016	
0.09	0	0	0	0	0.1146	0.1461	0.205	
0.1228	0.3776	0.0622	0.0824	0	0.0811	0.0571	0	
0.0381	0	0.0448	0.2096	0.0422	0	0	0	
0.0548	0	0.0387	3.4437	0.0475	0.0777	0	0	0
	0.0711	0	0	0.1188	0.0821	0	0	

1.1285	0	0.7595	0	0	0.1479	0.0686	0.0393	0
	0	1.2429	0	0	0	0.9593	0	
0.1455	0.1942	0	1.3641	0	0	0.3768	0	0
	0	0	0	0	1.6023	0	0	
0.0564	0							
ASTN2	1.9447	2.0179	2.2875	1.4752	1.9594	1.2489	1.5687	
2.0904	2.8544	2.2904	2.8953	2.0776	2.3444	1.6143	1.4335	
2.0701	2.6934	2.079	2.2347	2.5247	1.4686	1.4877	2.1768	
2.29	2.4126	1.2774	2.0036	2.598	2.1795	2.8118	2.9309	
1.8982	1.7237	1.978	4.0396	2.2421	1.4735	1.872	3.9686	
3.8333	2.3614	1.9143	4.8624	1.9759	1.4535	2.0275	2.1837	
1.5583	1.8736	2.3782	1.4812	2.5349	1.7732	1.6881	2.8922	
1.3725	2.8587	1.7251	2.5428	1.7401	2.0699	2.5047	2.3481	
1.3993	2.9738	3.0699	2.2983	1.8084	2.1383	2.9059	2.2015	
1.4577	3.1139	1.3198	2.0346	1.3412	1.5838	2.5769	1.9227	
2.7667	2.1689	2.6813	1.4625	2.9787	1.6702	2.4326	2.4126	
2.5012	2.0875							
ASUN	3.8361	4.5946	3.9408	4.3539	4.4448	4.6672	3.2081	
3.955	4.5099	4.1259	4.4363	3.9104	3.9457	4.6592	4.58	
4.397	4.5703	4.4863	4.5577	4.3377	3.5543	4.2989	4.1288	
4.1681	3.4699	4.2448	4.346	4.0205	3.9794	4.4244	4.0052	
4.1723	4.8037	3.5298	4.756	4.7873	4.3827	3.7518	4.4011	
4.9981	3.7568	3.6832	4.1001	4.1984	4.6551	5.2721	5.5439	
4.1723	3.38	5.7165	4.9285	4.187	4.7788	3.9946	3.9012	
3.82	4.6029	5.1269	4.7664	3.9231	4.7204	5.1209	4.5061	
4.3784	4.6003	3.5682	4.8261	3.6381	3.9049	3.3969	4.6085	
4.2664	4.1571	4.8159	3.5593	4.2825	4.3075	3.7514	4.2865	
5.1847	4.6229	4.3888	4.5559	3.8294	3.4997	4.8015	3.951	
3.8835	3.4714							
ASXL1	4.2107	4.9672	5.4441	4.2769	4.6861	4.6856	5.4592	
4.616	4.5925	5.2606	4.6112	4.6485	4.943	4.6078	4.318	
4.9478	5.0934	4.6053	4.5587	4.0191	4.1487	4.4604	4.8512	
4.5929	4.6465	4.9374	4.2989	5.2135	4.7606	5.1903	5.0155	
4.7167	4.0292	4.5944	4.1032	5.0954	4.0233	3.9323	4.465	
4.5976	3.9874	4.0473	4.0967	5.2819	5.4871	3.9554	5.1553	
4.8434	5.2413	5.428	4.6623	4.4204	4.9852	4.5893	5.5203	
4.7176	4.0804	4.959	4.8418	4.7283	4.1372	4.3478	4.2724	
4.291	4.2752	3.8019	4.3348	3.88	5.2286	4.2715	4.8441	
5.2571	4.7978	4.7538	5.4054	5.1326	4.8746	4.4019	4.0664	
4.4912	5.0116	5.4738	4.4297	4.7754	4.595	4.6885	4.3344	
4.0096	4.5033							
ASXL2	3.2872	3.4198	3.5373	3.3834	3.1436	3.4279	2.7581	
3.1872	4.2753	4.015	3.135	3.5911	2.9461	3.5368	3.248	
2.8319	3.602	3.6047	3.7449	2.9956	3.0759	2.797	3.391	
3.6298	2.73	2.5526	2.5917	3.1795	3.3037	3.4117	3.081	
3.2385	3.1808	3.1414	2.9144	3.9979	3.4606	3.4597	2.9484	
3.519	3.0395	2.9286	3.2167	3.7288	3.5736	2.3666	3.7555	
3.7956	2.9866	3.3313	3.2843	3.6422	3.6527	3.3372	3.5105	
3.052	3.5968	3.2961	3.0656	3.2046	3.1787	3.574	2.4808	
3.3669	3.6517	2.8768	3.7205	2.8663	3.9948	3.3981	3.0869	

3.3329	3.6609	2.9704	3.0662	3.0361	3.2486	3.5045	3.7021	
3.0101	3.6921	3.241	3.4879	3.287	1.9123	2.7459	3.3601	
3.1174	3.2558							
ASXL3	0	0.0151	0	0	0	0	0.0036	
0.005	0	0	0.002	0	0	0	0.003	0
	0	0	0	0	0	0.0013	0	0
	0	0	0	0.0401	0	0.0173	0	0
	0.0122	0	0	0	0.0017	0.0185	0	0
	0	0	0.1042	0	0.0071	0.0935	0.0194	0
	0	0	0	0.0122	0	0	0	0
	0	0.017	0	0.1034	0	0	0	0
	0	0	0	0.0012	0	0	0.0043	
0.0052	0.0184	0	0	0.0846	0	0	0	0
	0	0	0	0	0.0163	0	0	
0.0257	0							
ASZ1	0	0	0	0.0637	0	0	0	
0.0319	0	0	0	0	0	0.0637	0	
0.1015	0	0	0	0	0	0	0	
0.0673	0	0	0	0	0	0	0	0
	0	0	0	0.0266	0	0	0	0
	0	0	0	0	0	0.0374	0	0
	0	0	0	0.1925	0	0	0	0
	0	0.1844	0	0.7193	0	0	0	0
	0	0	0	0	0	0	0	0
	0.0166	0	0	0	0	0	0	0
	0	0	0	0	0	0	0	0
	0							
ATAD1	5.5351	4.9083	4.8931	5.3562	5.1736	1.9212	5.0586	
5.0399	4.8067	5.3476	4.5431	5.184	0.6338	4.5869	0.6147	
4.4053	4.4387	5.1118	4.3291	5.3953	4.9946	5.5285	4.7162	
3.9402	4.7946	3.9435	6.1161	5.2213	4.2717	4.5729	0.9698	
4.7537	4.1405	4.3191	4.6372	4.8149	5.1601	0.5957	4.7816	
4.835	5.4693	4.126	4.4086	4.8689	4.635	4.7685	5.141	
5.0392	5.1051	4.97	0.5832	4.7892	4.5743	5.1999	0	
5.2159	5.2787	4.5573	4.7442	4.8519	4.9154	4.6365	5.8244	
4.6944	4.4828	5.0289	5.4637	4.4173	4.5822	4.4494	4.6033	
4.0212	4.8043	4.1473	4.8446	5.204	4.5921	5.3018	4.2163	
3.5393	4.3315	5.0706	4.3787	4.7406	3.586	4.2571	5.375	
5.3129	5.2884							
ATAD2	4.0354	4.4196	5.2133	5.4007	4.3986	4.7961	3.9207	
3.7807	4.0756	4.927	3.2775	4.1995	5.0497	5.2361	5.284	
3.3593	4.1491	5.1673	5.2106	4.0182	5.1371	4.2727	3.6947	
4.2256	4.8042	2.666	2.325	4.6344	4.4432	3.6573	4.9047	
4.3458	4.7633	3.9036	4.4418	5.596	4.3746	3.4749	3.6815	
4.2059	2.8818	3.3953	5.2258	5.5561	4.9348	5.39	5.3529	
4.4446	3.184	5.7414	3.9898	5.025	4.7764	3.6848	4.8733	
4.6327	3.9609	5.6961	4.5613	5.9283	4.2722	4.1798	4.857	
4.3658	5.6621	1.934	4.8802	2.8483	3.9657	5.0212	4.283	
4.7755	4.5237	4.062	4.1326	4.2966	4.5695	3.9166	5.1465	
4.6594	4.9892	3.5124	2.3468	3.4108	4.8452	5.3834	3.0074	

4.3746	4.0281						
ATAD2B	1.6972	1.5655	1.7036	1.6439	1.2203	1.4817	1.2638
1.0722	2.4441	2.1882	0.8605	0.8964	1.1965	1.2958	1.3218
1.0069	1.5319	2.3488	1.7727	1.5197	1.5535	1.1513	1.2489
1.4131	1.8541	0.855	1.7133	1.7609	1.515	1.3855	1.8394
1.0276	1.3419	1.2544	0.8993	2.0736	1.3617	1.4321	1.3352
1.9386	1.2085	1.0131	1.3522	1.4757	1.9237	1.6752	1.9022
1.9092	1.2133	1.7011	1.3272	1.3966	1.4137	1.489	2.2855
1.6575	1.2673	2.1057	1.4749	2.1209	1.3022	1.1297	2.1114
1.5016	2.1404	0.7225	1.4539	1.241	2.1005	1.5401	1.1055
1.309	1.6678	0.7534	1.4551	0.9254	1.4467	1.6497	1.2698
1.4239	1.624	1.2421	1.3715	1.376	0.8925	1.2082	1.2702
1.3542	1.4045						
ATAD3A	3.8735	4.4616	5.9866	3.0211	4.4126	3.4782	3.2912
4.5869	3.4238	3.1448	4.3149	2.8751	3.3889	3.0017	3.8517
3.3223	3.6797	2.514	3.6385	4.6836	5.9112	3.2248	3.5061
4.4405	3.2789	3.4493	1.6943	3.439	4.0236	3.904	4.0531
4.6902	4.9161	4.4552	4.2226	3.045	3.9218	4.298	3.7691
2.9954	3.8559	2.9339	3.4326	3.6437	2.4286	2.9915	3.2109
2.638	3.655	3.1323	4.057	2.4286	3.5423	3.9081	4.3266
4.3915	3.1645	5.0765	4.5635	4.9439	3.932	3.5357	4.1576
4.3949	3.4355	2.6821	4.1722	3.3836	3.7167	3.9035	3.5214
4.2412	4.6191	3.9852	2.5238	3.9661	4.8325	2.5275	3.8236
4.1599	3.9287	2.9614	3.1919	2.7747	4.7667	5.0311	3.2786
4.4563	3.9287						
ATAD3B	4.2279	5.159	6.4199	2.8466	4.6118	3.2871	3.3747
4.3102	3.0867	3.3996	3.113	3.6355	3.0905	2.756	4.0312
3.4705	2.7416	2.4522	3.3892	4.1527	5.5021	2.6935	3.892
5.2525	3.1145	2.8388	2.7535	3.5857	4.0247	3.6133	4.2024
4.1101	5.1449	3.5098	4.1542	3.2756	3.2391	3.3993	3.8448
2.198	3.5698	2.7063	2.9332	3.7225	2.1672	2.2621	3.1949
2.6088	3.3408	2.3052	3.8887	2.942	3.1577	3.6743	4.9384
4.352	1.8782	3.693	4.7495	4.1279	3.8991	4.165	4.4094
3.8755	3.6557	2.2868	3.6122	2.8806	4.3282	2.9349	3.0307
3.4942	4.3742	3.9498	2.732	3.1092	4.8567	2.7163	2.4362
3.5761	3.2467	4.2338	3.7354	1.6935	3.285	4.5781	2.6562
3.0839	3.4355						
ATAD3C	0.451	0.4163	0.5816	0.2642	0.0984	0.296	0.1221
0.1953	0.2245	0.1278	0.0808	0.1011	0.1276	0.2378	0.3939
0.2618	0.338	0.3093	0.2817	0.2181	0.9146	0.1665	0.1685
0.3223	0.1317	0.1189	0.2969	0.2283	0.1388	0.1783	0.2551
0.1449	0.1627	0.1309	0.1519	0.0945	0.1177	0.1517	0.1396
0.1591	0.1282	0.1281	0.1048	0.2969	0.1157	0.0659	0.1335
0.0933	0.1774	0.3053	0.1656	0.1647	0	0.5257	0.4064
0.3039	0.1945	0.0916	0.2059	0.239	0.4201	0.1023	0.2147
0.3017	0.2865	0.044	0.412	0.1167	0.3147	0.2238	0.011
0.443	0.2312	0.1096	0.3835	0.1992	0.3329	0.1151	0.0941
0.2196	0.2002	0.0771	0.0573	0.065	0.1381	0.0596	0.0754
0.2542	0.2243						
ATAD5	2.3968	2.2471	2.6175	2.2012	2.4424	2.3525	1.073

1.7514	2.2174	2.0089	1.6754	1.6039	1.9978	2.4336	1.7994
1.7739	2.2084	2.27	2.5211	1.8831	1.8249	1.611	0.9695
2.0189	2.1172	0.9579	1.4872	2.8904	1.882	1.9818	1.98
1.8027	2.0994	1.6716	1.6557	2.3598	1.8462	0.6271	1.4998
1.9656	1.2036	0.6106	1.8125	3.1519	1.6564	2.4614	2.4786
2.4548	1.1606	3.2102	1.9015	2.139	1.8359	1.577	2.0079
2.5063	0.8528	2.4475	2.3947	2.6594	2.3811	2.2354	1.7713
2.3499	2.024	0.4355	2.0628	0.7194	2.5307	2.283	1.5741
2.4378	2.4009	1.6714	2.0265	2.497	2.3947	1.6398	2.1292
1.7637	2.3019	1.0588	0.7882	1.0437	1.99	2.1874	1.0131
2.0239	1.645						
ATAT1	2.2108	3.1586	2.072	3.0314	2.5186	1.9604	2.165
2.7623	2.4335	3.207	1.7908	2.7925	1.789	2.9227	2.7543
2.1687	2.7954	3.1004	2.7925	2.6177	2.4067	3.0872	3.1064
2.5898	3.6605	2.753	3.6982	2.8225	2.6535	2.413	2.6177
2.7037	1.8963	1.6805	3.167	2.7162	3.0182	2.5674	2.1027
2.2896	3.0528	3.2913	1.6213	1.7475	2.3744	3.2082	1.6695
2.2304	2.7972	3.1341	2.7183	3.4885	2.759	2.24	2.6797
2.4923	1.6202	3.0831	2.1237	3.4825	2.6431	3.1778	2.3612
2.3635	3.2561	1.606	3.2324	2.9485	2.1426	2.0332	2.0812
2.8868	2.089	3.4362	3.2074	2.6809	2.9051	3.9118	1.9954
2.8353	2.3887	3.585	2.5447	1.8447	3.3958	2.5455	2.0331
2.1962	2.4274						
ATCAY	0.1269	0.0554	0.042	0	0.0554	0.0725	0.043
0.0563	0.053	0.0645	0.0673	0.0062	0.0469	0.0699	0.0292
0.0396	0.114	0.0525	0.064	0.044	0.1022	0.066	0.0289
0.0813	0.0909	0.0777	0.0389	0.0635	0.0425	0.0347	0.1617
0.0326	0.0256	0.0586	0.0462	0.015	0.032	0.0423	0.2266
0.1259	0.0361	0.0141	0.1506	0.099	0.0298	0	0.0367
0.0392	0.0571	0.069	0.0517	0.0489	0.1096	0.0349	0.0597
0.1766	0.0641	0.0365	0.044	0.0416	0.0261	0.0077	0.057
0.0536	0.0504	0.1161	0.1037	0.0374	0.0466	0	0.0097
0.0919	0.0414	0.015	0.0407	0.1309	0.1155	0.0141	0.0057
0.0284	0.0602	0.032	0.0818	0.0868	0.1196	0.0623	0.0315
0.0492	0.0563						
ATE1	3.4331	3.4497	3.158	3.5832	3.6561	3.0453	2.8451
3.9946	3.551	3.3636	3.0606	3.7862	3.1057	3.3913	2.3457
3.2142	2.8868	3.6859	3.6124	2.8361	3.0965	2.3289	3.0121
3.1133	3.1924	1.5047	3.7587	3.6087	2.8296	2.9195	3.8324
3.4601	3.053	2.988	2.5476	3.7403	3.3268	2.8574	3.1126
3.0518	3.7386	3.3485	2.8021	3.3724	3.1166	2.2738	3.3849
3.5324	4.4711	3.8813	3.1426	3.098	3.3924	3.6538	4.2804
2.9255	2.395	3.1166	3.1073	2.6437	2.8734	2.9539	4.3633
3.3493	3.7612	3.0193	4.04	3.1912	3.316	3.5262	2.9907
2.8124	2.9547	2.9049	3.5617	2.9534	3.3369	3.4724	3.2817
2.0053	2.9458	3.5291	3.3092	2.7641	2.9148	3.0207	3.6927
3.3278	3.7893						
ATF1	3.7222	3.9351	3.7092	3.8621	3.8801	3.7656	3.1001
2.9646	3.7422	3.6292	3.056	3.2138	3.6543	3.6463	3.7414
3.5064	4.1892	4.0812	4.3628	3.4644	3.8801	3.5268	3.1773

3.1683	3.6269	3.0617	3.315	3.5766	3.1693	3.5027	3.7104
3.6036	3.6167	2.6275	3.0582	3.7135	3.5722	3.28	2.8322
3.777	2.9851	3.9563	2.3142	3.5052	3.7825	3.8347	4.0521
3.5719	3.445	5.1805	3.6145	3.8215	4.0139	4.1447	3.6358
3.4642	4.1737	3.7116	3.8334	3.7287	3.9132	3.5208	4.0399
4.0832	3.1434	3.339	2.6771	2.8901	4.0284	3.4454	3.3224
3.5521	3.7556	2.7734	2.9494	3.236	3.7528	2.7078	3.6476
4.0267	4.076	3.2587	3.6289	3.3624	3.0654	4.2269	3.1043
3.199	3.0529						
ATF2	3.5569	3.2063	3.4999	4.185	3.0873	3.8364	4.5821
4.7953	4.1167	3.6858	3.8074	4.2847	4.1002	3.9697	3.8959
3.8289	4.1154	4.7495	3.5705	3.4637	3.722	3.7197	4.0562
3.6309	4.1545	3.7323	4.6595	4.0175	3.8387	4.1933	4.8488
3.5582	4.1056	4.175	3.8732	4.3129	3.565	3.6583	3.1983
4.0038	3.8483	4.0871	4.6005	3.9765	4.1314	4.1786	3.6321
4.6957	4.6656	3.9705	3.9897	4.2847	4.7845	4.1466	4.2399
4.1733	4.1366	4.5699	3.9643	4.7399	3.6597	3.3693	3.0517
3.7494	4.9296	3.8838	3.8995	3.8802	4.3749	3.6244	3.8272
4.1548	3.7223	4.0107	4.2208	3.8093	3.5297	3.8939	4.3005
3.9743	4.2348	3.563	3.7535	3.94	3.5521	4.5096	4.2207
3.9364	4.128						
ATF3	3.3078	2.0709	2.1543	1.069	4.4043	1.9604	4.8305
1.7765	1.8259	3.0252	1.6906	3.4982	4.1571	1.5586	3.2369
1.7167	3.6834	1.5213	2.185	3.9845	1.0131	3.3457	2.3692
2.4543	1.8426	5.3655	5.0878	1.6363	1.8442	2.1155	3.2153
1.3343	1.6877	1.3109	3.5787	2.9485	3.8612	3.512	4.9066
2.5205	4.5104	0.3885	3.792	3.529	3.1469	2.8591	3.3457
3.096	4.2064	2.2545	2.8991	3.0055	2.6376	4.0074	4.053
3.1726	2.693	1.6084	2.1889	3.1887	2.6902	2.6377	2.0378
1.9119	3.2307	4.985	2.9211	4.3349	2.4652	2.0007	1.9144
2.1039	2.8398	1.614	2.9225	2.0593	1.6907	1.8388	1.2532
1.627	1.4177	4.6578	5.7617	3.0778	2.4418	1.7348	5.0919
2.2349	1.786						
ATF4	8.1855	8.1413	7.8889	7.9612	7.5811	7.1535	7.9954
8.6019	7.4559	9.3725	8.4428	7.6283	8.6322	7.5883	7.5011
7.1535	8.6117	7.4288	7.47	7.6794	7.2741	8.3754	6.3363
7.7329	8.5486	8.3854	8.6751	8.0448	6.2192	7.2939	8.0147
7.3472	7.5964	7.3881	7.1132	7.7199	8.8574	8.6635	9.2696
7.5811	8.2616	6.9508	7.3603	8.2013	6.9043	7.4145	7.8829
8.7808	8.3956	7.4856	8.0382	7.205	8.4034	9.2247	7.6475
8.2672	8.4523	8.8115	8.2357	8.8862	7.6039	8.5969	8.1064
7.1652	7.0064	8.1004	7.4783	7.7719	7.8474	7.4527	7.6435
7.5683	7.2638	7.2473	7.3471	7.6219	7.1058	6.261	6.8685
6.9756	8.0638	7.5173	8.692	7.3774	7.1035	8	7.6101
7.4776	7.0697						
ATF5	2.6451	3.2782	4.4643	3.1367	3.3756	4.4357	4.4756
3.7602	3.2476	4.9638	2.0206	2.7802	2.8053	3.5844	4.6251
4.556	3.8459	3.1698	4.0267	4.1501	3.8035	2.5911	4.2707
2.7693	2.8662	4.684	1.6468	3.0071	3.8991	2.0039	4.1362
3.9174	4.2013	2.1037	3.1363	3.3052	4.5643	4.0611	4.9416

3.7024	3.3628	4.4024	2.5495	2.3939	4.9035	4.218	3.7753
3.4956	3.4967	2.9661	2.7162	3.3555	3.1905	3.7901	4.9191
3.4185	3.2115	4.6271	3.0685	4.7081	4.5312	2.9884	4.5008
3.6357	3.0245	4.5291	1.9427	5.2824	4.0122	2.6383	3.7444
3.2349	4.1602	2.0301	3.3347	4.1231	4.1466	2.9771	3.5425
2.5123	3.0276	2.9385	3.898	2.8908	4.1104	3.0146	2.9467
3.4729	2.6092						
ATF6	4.0057	3.3872	3.3222	4.5338	3.9462	5.1723	3.9263
3.4444	4.3401	4.1224	4.927	3.97	3.5241	3.779	3.9584
4.0424	3.9909	4.5205	3.9363	3.5899	3.6474	4.242	4.0608
3.9302	4.0278	3.9603	3.3315	3.3091	3.596	3.7217	4.4819
3.7558	3.4353	3.8824	3.2715	3.996	5.0089	4.2804	4.2852
3.7278	4.0424	4.0623	3.9572	3.9298	4.6335	4.7416	4.1039
3.5286	3.0329	3.5664	3.8701	4.4787	4.1048	4.7649	3.379
3.1323	4.2311	4.1516	3.7968	3.965	3.8409	3.5422	3.6856
3.6426	3.8122	4.0951	4.382	3.59	3.9044	3.8696	3.3203
4.0414	3.643	3.952	4.3226	3.3701	4.0134	5.1193	4.1759
3.6605	3.5079	4.1842	3.7564	4.6506	3.6499	3.7256	4.0867
3.7968	3.8055						
ATF6B	5.0016	5.0426	4.9151	5.0918	4.4473	4.3433	4.1916
5.1146	4.6501	5.5862	4.7134	5.3869	4.6299	4.5204	5.0371
4.9467	5.7722	5.0233	4.5977	4.7129	4.9541	5.5933	5.0888
3.2358	5.2963	4.9099	4.5209	4.8068	5.103	3.8023	4.8998
5.0467	4.5697	5.4839	5.6677	4.3252	5.8842	6.0272	5.6612
4.3025	5.1609	5.2088	4.9878	4.9965	4.4367	4.3895	3.8608
4.885	5.7106	4.7103	5.1428	4.6812	4.6403	5.7776	4.5792
4.5711	5.2172	5.0799	3.6418	4.6514	5.0762	4.4655	4.9304
4.7051	4.7253	4.5822	5.4096	4.9183	4.2388	4.8373	4.7037
5.3327	3.9018	5.877	5.3469	4.5367	5.3429	5.9995	4.8732
5.1528	4.4312	5.4533	4.9589	4.9409	5.0602	4.6094	5.2905
5.1172	5.3514						
ATF7	3.1532	3.7695	3.552	3.5579	3.2061	3.7945	2.702
3.1739	3.8744	3.7183	3.3742	3.0734	2.9122	4.0252	2.6813
3.4051	3.7084	3.9025	3.9874	3.1064	3.8056	3.2015	2.5396
3.2411	4.0202	2.9765	5.0586	3.6081	3.4051	3.2834	3.4098
3.1815	3.3581	2.7669	3.3418	3.8458	3.5034	3.5945	2.9461
3.6527	2.9412	3.2561	2.4019	3.4763	3.1465	3.7042	3.7036
3.4291	3.0047	3.7559	3.2169	3.6914	3.7208	3.2254	3.3613
4.291	3.0385	4.0157	3.5338	3.5581	3.419	3.567	4.0364
3.7725	3.86	3.0575	3.272	3.5869	3.2558	2.9396	3.4412
2.8664	3.4056	2.4511	3.2279	3.4365	4.0154	3.1583	3.5488
3.4082	3.1792	3.2171	4.0376	3.2558	3.0778	3.5375	3.8708
3.4696	3.6463						
ATF7IP	2.9812	3.2043	3.3671	3.1144	3.502	4.0127	3.3096
3.329	4.0222	2.9583	3.2402	3.6314	3.9779	3.3886	3.6222
3.4301	4.303	3.135	3.472	2.6379	2.7922	3.8103	2.9899
2.9804	3.1387	3.936	5.0765	2.8685	3.3562	2.6567	4.1698
3.1861	3.6458	3.6186	3.9086	2.98	3.6035	3.7648	3.4858
4.6434	3.0816	3.426	3.1061	3.5982	4.2363	3.6535	3.7049
3.3196	3.0667	3.8704	3.6604	3.821	2.8958	3.1855	3.7373

2.533	4.7508	3.1018	3.0656	3.5183	3.6218	3.531	3.6347
3.597	4.0895	3.7794	3.0386	3.4559	3.2575	2.6878	4.0161
3.0505	3.6895	3.9259	3.3869	3.4877	3.3846	3.949	3.9297
3.6338	3.453	3.3621	3.5266	3.9315	2.6832	2.4072	3.4544
3.5702	3.9407						
ATF7IP2	0.9929	0.0725	0.8842	0.0872	0.4218	0.2596	1.2513
0.5483	1.4722	0.6086	0.1487	0.3805	0.4177	1.3619	1.8128
0.2211	0	0.97	1.7432	1.3103	0.8647	1.488	0.7352
0.7546	0.2986	1.3228	1.3594	0.1119	1.9545	0.8192	1.9321
0.9854	0.8944	1.5632	0.0578	0.8603	0.448	0.6031	0.0427
1.4316	1.1724	1.1718	0.0637	0.5388	1.4464	0.0497	0.524
0.689	1.3966	0	1.3191	0.3968	0.9668	1.673	1.0486
0.7009	0.948	1.6027	0.0739	1.734	0.4447	0.6775	1.1038
1.3914	1.5402	0.4438	0.8632	0.8182	0.8344	0.006	0
1.664	1.5428	1.1561	0.7259	1.0943	0.3062	1.3622	1.444
1.3047	1.2569	0.8643	0.019	0	0.9805	0.6078	1.5059
1.1857	1.3424						
ATG10	1.6909	1.6139	2.0957	2.4305	1.5893	1.6327	1.8503
2.1968	1.1435	1.8751	2.4179	2.1539	2.3743	2.1567	1.4351
2.0381	2.7462	1.9459	2.8652	2.6089	1.8701	1.7555	1.5307
2.1045	2.0159	1.9508	1.3758	1.9734	1.8575	1.7791	3.4757
2.2245	2.6969	2.5144	2.8438	2.6902	2.0851	1.7189	1.1811
2.2028	1.6013	2.1525	2.716	2.0512	2.2198	2.9718	2.4188
1.297	1.2927	2.9298	2.0144	2.3888	1.7513	1.4701	3.0017
1.8539	1.6436	2.427	2.0972	2.224	1.6151	2.3479	1.9907
2.0814	1.8723	1.3644	2.4201	2.2532	2.4024	2.3489	1.5496
1.9548	1.6906	1.378	1.5936	2.1756	2.2876	2.0118	1.7122
2.8291	1.8691	1.1976	2.2553	1.783	3.2375	1.7505	2.0512
1.8684	2.13						
ATG101	5.5013	5.3692	5.0404	4.4479	5.9095	4.9067	4.6135
5.4934	4.7022	5.0604	5.4411	4.8028	4.7831	4.0314	9.0685
5.7896	5.3222	4.8799	4.63	5.4016	4.9695	6.0269	5.2522
5.3962	5.202	5.0995	5.0793	5.042	5.5349	5.1922	4.6414
5.83	5.2366	5.1238	4.9416	4.3734	5.9563	5.0578	4.9873
4.8913	5.3526	4.7751	4.2708	5.0967	3.6942	4.1378	4.5035
5.2757	4.844	4.4194	5.0241	4.5594	4.772	5.643	4.464
4.7469	4.6309	5.5031	5.4836	4.64	5.7935	5.8074	4.9987
5.2248	3.8103	5.0023	5.0433	5.1903	5.308	5.6223	4.9787
4.9558	4.4165	5.6246	4.9625	4.6271	4.9665	4.5417	4.2387
5.3629	5.6912	4.6466	6.7816	4.8896	4.5925	5.4792	4.7155
4.8134	4.5726						
ATG12	3.2659	3.4815	3.5884	3.4867	3.9335	3.7281	3.8106
4.1422	2.9695	2.5689	3.9023	3.2996	4.134	3.7215	3.855
3.6652	4.0211	2.7338	3.7937	3.7709	3.525	3.0897	3.4547
3.7051	4.1526	4.1479	4.0939	3.8514	3.4061	3.6001	3.9192
3.9491	3.6604	3.69	4.0401	3.2506	3.7701	3.2759	3.1845
3.2215	3.1601	3.6352	3.9356	3.1115	3.6149	4.1361	3.2181
3.3844	3.6156	3.968	3.7107	3.4271	3.6149	3.6729	3.4272
3.3083	3.7532	3.323	3.112	3.321	3.4525	3.588	3.7841
3.4046	3.1922	3.6019	3.6595	4.3853	3.2513	4.1953	3.3192

3.7188	3.6721	3.2038	3.1024	3.8438	3.463	3.4426	3.4308
3.2417	3.2209	2.8682	4.2666	3.5069	4.3947	3.5121	3.7645
3.7123	3.4435						
ATG13	3.0671	3.9899	3.7513	3.795	4.3415	4.0566	4.2644
4.3185	3.8603	3.5319	4.517	3.3914	3.0962	3.6905	4.0704
4.7173	5.0573	3.1601	3.5338	3.6509	3.5705	4.0634	4.0376
3.7619	4.2769	4.1327	3.6215	3.6657	4.4253	3.3427	4.1293
4.0289	4.1926	3.4681	3.7866	3.5794	4.0992	4.6874	4.0512
3.839	3.7997	4.1398	4.1515	3.6563	3.711	4.1904	3.7438
4.1154	4.3794	3.7357	4.2084	3.9337	3.9224	4.0971	4.2545
3.9527	4.1593	3.7865	3.7861	3.4617	3.5771	3.7743	3.379
3.9778	3.6473	5.1481	3.3589	4.6683	3.6523	4.0155	3.927
3.6855	3.9092	4.4179	3.8423	4.5894	3.5586	3.5325	4.0197
4.0123	3.4446	4.3254	4.4077	3.6066	3.7921	3.5031	4.4086
3.808	4.0823						
ATG14	2.6713	2.467	2.0678	2.3598	1.8786	2.8061	2.1333
2.5712	2.1441	2.1499	2.2559	2.8129	2.1094	3.1415	2.157
2.5764	2.7484	2.8787	2.6782	2.1111	2.7196	1.8123	2.8433
2.1395	2.0383	2.9772	3.6242	2.7204	2.5836	2.4807	2.9644
2.4947	2.3211	2.0399	1.9527	2.6935	2.7283	2.9113	2.099
3.0752	2.9776	2.902	1.6364	3.5928	2.427	2.2582	2.6894
1.9852	2.9125	3.5559	2.0039	3.0279	3.0547	2.4363	2.4162
1.9694	2.3156	1.8574	2.1943	2.0827	2.6967	2.4281	2.4408
2.0763	3.0333	3.0417	3.843	3.2409	2.1505	1.9264	2.4995
2.2429	2.9381	2.6517	3.2852	2.4287	2.4491	2.2161	2.964
2.5907	3.0995	2.718	3.1511	2.4866	1.8502	2.7305	2.9593
2.7254	3.2576						
ATG16L1	3.368	3.1975	3.2545	3.6335	3.5223	2.8539	3.6932
3.7532	3.4907	2.9454	3.0957	3.9856	3.7059	3.765	3.769
3.4374	2.7764	3.5776	3.355	3.5455	3.5379	3.7509	3.8606
4.0951	3.9585	3.7612	3.215	3.2226	4.1142	3.388	3.0853
3.5555	3.9165	3.2176	3.2815	3.2736	3.4854	3.1687	2.8249
3.7022	3.1503	3.7419	3.9301	3.2416	3.5562	3.7323	3.5421
3.5528	4.5103	3.2241	3.2759	5.2088	3.6307	3.1556	3.3445
3.1386	4.3794	3.2997	3.6286	3.3587	3.7504	3.3669	2.5938
3.267	3.5188	3.3203	3.1706	3.6778	3.6822	3.8252	3.0163
3.5162	3.8602	3.7268	3.8312	3.2437	3.3868	3.4443	2.9179
2.7593	3.1612	3.2701	4.5577	2.9369	4.0964	3.4723	3.3079
3.2435	3.3522						
ATG16L2	3.3197	2.7458	2.132	2.0763	1.7503	1.9427	2.2596
1.0032	2.3971	2.6432	2.5131	1.8961	2.309	1.9887	0.6442
1.7839	3.5861	2.2163	1.3233	2.105	3.1651	1.8794	1.9789
2.192	1.8493	1.2215	2.0719	2.3701	1.8209	2.4567	2.7892
1.322	1.6589	1.7282	2.8094	1.9209	2.4685	2.0756	1.564
2.1382	3.375	2.0949	2.6749	3.3136	2.4826	1.9708	0.9545
2.5219	3.561	1.3086	2.0266	1.7839	1.9479	1.4565	2.4075
2.0657	3.6569	1.3476	2.0672	1.4007	1.5402	1.3396	2.3701
2.2308	1.7716	3.3333	3.4328	1.3319	1.7871	1.9425	1.6624
1.6974	1.5493	1.9818	2.3991	1.7537	2.0511	1.7559	1.9565
1.8353	3.6349	1.7118	1.5556	2.631	1.2255	2.4518	2.0752

2.2838	2.8267						
ATG2A	2.5264	2.5795	3.3076	1.3509	2.9025	2.7585	2.6465
3.3049	2.249	1.9993	3.2618	1.6853	2.3319	2.0582	2.2455
2.8594	3.1113	1.6582	1.6663	2.7506	3.3654	2.5673	2.2907
2.0835	2.2799	2.2097	1.6963	2.3881	2.4372	1.8104	2.2704
2.4247	2.4831	2.4452	2.3221	1.4968	3.332	3.5787	2.3255
1.802	2.9411	2.6838	2.1263	2.0722	1.6923	2.0054	1.5735
2.3859	2.7897	1.9103	2.4141	1.654	2.2219	2.9288	2.5784
2.7943	2.9828	2.5405	2.3879	1.9586	2.1392	1.9258	2.2966
2.4474	2.2417	3.6415	3.064	3.4832	1.8281	2.2025	2.8843
2.0502	2.506	1.9857	2.2433	3.947	3.2937	1.8434	2.5916
3.0382	2.1435	2.6629	3.3206	2.2443	2.6987	2.4108	3.3466
3.0582	3.3776						
ATG2B	2.3035	1.781	1.7407	2.2616	1.7232	2.627	2.7472
2.3189	2.4759	2.8469	1.6662	2.5883	2.4558	2.8944	2.1478
2.666	2.183	3.2178	2.5951	1.6513	2.3357	2.0525	2.8286
2.3318	2.1193	2.2254	2.1115	2.2666	2.7319	2.1614	1.8593
2.1464	2.0165	2.1648	2.4511	2.5733	2.153	2.7126	2.2181
3.0188	2.3313	2.4045	1.7554	2.2029	3.0702	2.5449	2.1865
2.2543	2.3788	2.5659	2.0491	2.5777	2.586	2.2593	1.7046
1.8978	2.8128	1.915	2.4154	2.0352	2.2689	2.2468	2.7119
2.4144	2.0788	2.2533	2.3834	2.6391	2.2549	2.2921	2.476
2.178	2.2435	2.4764	3.195	1.5247	2.0054	2.4641	2.6617
2.2056	2.1825	2.4231	2.3176	2.7902	1.152	2.2161	2.8935
2.5449	2.9593						
ATG3	4.8326	5.5717	5.68	5.7056	5.2312	5.5462	5.3836
5.6507	6.0963	5.6726	5.4872	5.3254	5.5162	6.1507	5.6626
5.6891	5.8652	5.9173	6.1775	5.4187	5.7416	5.4094	5.4069
5.1117	4.6661	4.8347	4.3253	5.6837	5.5895	5.398	4.622
5.7036	5.4562	5.9563	5.2937	5.9405	4.9335	5.2175	5.8318
5.9595	4.9452	4.5684	4.8003	4.9209	5.4966	5.5454	6.0084
5.7582	5.0146	6.0507	5.8841	5.8095	5.5681	5.7199	5.0782
6.1397	5.1793	4.8303	5.2335	4.9592	5.5264	5.4618	4.4248
6.1565	5.1942	5.2216	5.7683	5.2067	5.8227	4.8807	5.1649
6.1864	5.6085	5.9144	5.1642	5.7663	6.0139	5.6328	5.4416
5.9997	5.8388	5.2314	4.6234	5.5775	5.7614	5.7813	5.2867
5.2055	5.2673						
ATG4A	2.5454	2.0618	2.9322	2.4731	2.5611	2.9744	3.2354
2.7006	3.0319	2.6067	3.6196	3.1691	2.2165	2.2365	2.6074
3.6967	2.2117	3.0487	2.4573	2.9443	2.683	2.3114	3.0681
2.9997	1.7883	2.9919	3.5371	2.3012	2.7363	3.1441	2.4786
2.5642	2.7974	3.4233	3.0197	3.0653	2.0738	2.4158	1.7186
2.6252	2.0399	3.0044	2.1416	1.9783	3.0574	3.3515	2.7767
2.535	2.523	3.34	2.509	3.4065	2.4105	2.1566	2.8604
2.6558	2.5522	2.509	2.4851	2.5433	1.9839	2.3628	2.1459
3.0232	1.8481	3.0021	3.0544	3.4188	2.7119	1.5405	2.3136
2.2821	2.9196	2.8257	2.7969	3.6209	2.8985	3.4848	2.4044
2.4222	1.9955	3.0603	3.3915	2.7456	2.0882	2.4768	3.3336
2.8542	2.9122						
ATG4B	4.0948	4.0459	5.1397	3.6732	3.8714	3.3418	4.0894

4.7473	3.7835	3.1771	3.9267	3.4313	2.5109	3.6247	4.3575
4.6421	3.4069	3.9973	3.8442	4.1645	3.9651	4.0197	4.0326
4.1194	4.3898	4.3038	3.3351	5.6118	3.7948	4.445	4.5739
4.7478	5.442	5.1986	4.0685	3.3832	4.4607	4.2733	4.349
3.7926	4.762	3.8795	4.2004	4.0105	4.058	3.7443	3.5033
4.1668	4.9042	3.5394	3.5528	3.2937	3.9133	3.8611	4.2529
4.1902	3.9338	4.5446	4.0857	4.5075	3.6687	3.6947	3.4995
4.4763	4.4398	4.1047	5.47	4.1876	6.0847	4.375	4.3599
4.3034	4.3657	4.2868	4.3294	4.1008	4.4485	3.7552	3.9155
3.4487	4.1391	4.2796	3.5665	4.1959	4.5031	4.1169	4.4442
4.6829	4.8291						
ATG4C	2.5239	3.0729	2.4339	2.7861	2.5689	2.637	2.92
2.3095	2.7276	2.5784	3.4214	3.1651	2.4999	3.3312	2.4227
3.0841	3.1603	3.1763	3.5192	2.8982	2.8492	2.9696	2.1826
2.5081	2.0187	2.7112	3.0505	3.4182	2.9733	3.7734	3.4707
2.917	3.716	3.4286	2.2579	3.0821	2.4549	2.6917	2.7356
3.5476	3.0983	2.653	2.6883	3.0489	1.8848	3.0861	2.9753
3.2878	2.5708	3.8859	3.0391	2.9454	3.2768	2.9846	3.0601
2.6253	2.8562	2.9783	3.2643	2.7655	2.5679	3.1875	2.8467
2.9637	3.9898	2.7305	3.5113	2.5495	2.45	2.3099	2.6752
3.4948	3.1505	3.5786	2.6312	3.0411	3.059	2.5069	3.1835
3.2198	3.4701	2.7924	2.2715	1.5593	2.2274	3.3897	3.2649
3.4037	3.5321						
ATG4D	3.4012	3.1002	4.3494	1.7323	3.5447	3.1772	3.0943
4.4034	3.044	2.887	3.8349	1.9537	3.2373	2.0483	3.4288
3.1905	2.2477	2.3084	2.971	4.8121	3.8504	3.1987	2.9776
3.7785	2.6695	2.8613	2.578	3.329	2.9387	3.9335	2.5099
3.1635	3.2012	3.7292	3.344	2.6778	3.4468	3.7345	4.1256
2.0813	3.5089	2.2805	2.4175	2.3239	2.5324	2.5306	2.5708
2.8708	3.5886	1.7578	2.7195	1.6684	2.1555	2.8189	3.3015
3.3167	2.2135	4.3393	4.9725	4.2733	3.198	3.9176	3.3332
3.0134	2.7939	2.2778	2.7684	2.79	3.4364	2.8189	3.4191
3.2502	3.6053	2.0189	2.2111	3.9017	3.1605	3.1543	2.5899
3.6126	2.8915	2.2297	3.8918	3.2436	3.6764	3.0822	2.8919
3.5243	3.3855						
ATG5	3.3901	3.4341	3.5621	4.4042	3.2314	4.1265	3.605
3.6735	3.8043	3.0996	3.1328	3.6482	3.6211	4.0824	3.944
3.3336	4.0727	3.6617	3.9825	3.5337	3.8675	4.0512	4.2356
3.6105	3.989	4.0846	3.3291	2.8148	3.5841	3.4138	3.9789
3.7342	3.5367	3.3709	4.0919	3.7393	2.8724	2.7488	3.1588
3.6307	3.3794	4.0425	3.7221	3.5278	3.9894	4.4722	3.6193
3.01	3.7506	4.3368	4.2348	4.038	3.687	3.2875	4.392
3.5235	4.1259	3.653	3.6178	3.9917	3.6022	3.7337	3.3563
3.217	2.5055	3.1665	3.034	3.2194	3.3907	3.6542	3.7795
4.0782	3.4059	4.0585	3.7341	3.3755	3.2921	3.2523	2.7782
3.4577	3.4309	3.9167	4.2033	3.6852	3.905	3.8291	3.602
3.2318	3.3196						
ATG7	2.0464	2.007	2.0775	2.3844	2.4132	1.9402	2.1855
1.8521	1.6958	2.1927	2.2984	3.4022	3.5434	2.3483	2.6807
2.8882	3.5843	2.1001	2.6977	2.2064	2.1708	2.4234	2.0174

2.3464	2.2745	2.4549	1.2788	1.7856	2.5588	2.1021	2.5061
2.4632	2.4024	1.6156	2.543	2.2521	2.3117	1.8001	2.5774
2.6357	1.9661	3.167	3.3113	2.131	3.5579	2.5335	2.7409
2.2268	2.8769	3.2196	2.0945	2.4636	2.1083	2.1153	2.0642
2.0864	3.4739	2.0093	2.0121	2.1193	2.5123	2.3113	1.7415
1.9815	1.9851	3.2938	2.7333	2.2316	1.82	2.6049	2.4607
2.9169	1.9829	2.4424	2.0899	1.7347	2.0289	2.2371	1.6514
1.6738	2.214	1.8723	2.1973	1.8181	2.9332	2.1842	1.9299
1.8521	1.9365						
ATG9A	3.9191	3.5953	4.3201	3.7361	4.0529	4.6486	3.4376
3.4213	4.1022	4.5748	4.1165	4.1458	4.5684	3.8567	4.0615
4.0704	3.9086	3.5848	3.6791	4.8704	4.3736	4.5358	4.3664
4.4997	4.0482	4.302	4.0924	4.037	4.2526	4.257	4.2036
3.633	4.4507	4.5188	4.244	3.9377	4.3727	4.9012	4.5358
4.1967	4.2257	4.1234	4.7387	3.9959	3.5035	3.8219	3.7892
4.3268	4.4762	3.7794	4.2329	3.8424	3.9884	3.8166	4.3311
4.6223	3.5666	4.624	3.8377	4.2835	4.2016	3.9591	3.6327
4.0873	4.948	4.3556	4.1526	3.8623	4.8817	4.227	4.2033
4.3148	4.2602	4.0486	4.2622	4.3761	4.2888	4.7005	3.8283
5.0722	4.1558	4.3936	4.3941	4.2115	4.303	3.8525	4.3837
4.2098	4.6624						
ATG9B	0.6423	0.039	0.0232	0.3701	0	0.3891	0.0775
0.3363	0	0.3267	0.4668	0.342	0	0.1281	0.0742
0.079	0.2942	0.2235	0.0511	0.3097	0.5134	0.3899	0.0573
0.2735	0.1	0.0123	0.0825	0.095	0.0808	0.4601	0.3879
0.0563	0.0069	0.0037	0.0133	0.2329	0.4541	0.1655	0.713
0.062	0.1038	0.0315	0.2472	1.123	0	0	0.4247
0.1188	0.0879	0.0528	0.0242	0.2307	0.3494	0.1944	0.1541
0.1672	0.0755	0.1848	0.0993	0.0574	0.029	0.4592	1.2611
0.1994	0.0838	0.0918	0.2916	0.5948	0.2594	0.1044	0.0628
0.3218	0.0681	0.6363	0.0574	0.1688	0.141	0.0555	0.0499
0.0868	0.0656	0.2318	0.5393	0.114	0	0.4373	0.0632
0.085	0.0501						
ATIC	6.4476	6.1376	5.8088	6.5549	5.9611	6.2275	6.005
6.6403	6.0521	6.0453	6.2453	6.497	6.1589	6.3725	6.0829
5.5574	6.6093	6.264	6.2344	5.5905	6.0431	5.9421	6.23
6.5224	5.9953	5.6097	6.332	5.7605	6.4755	7.0071	5.4961
6.4073	6.6995	5.9886	5.5064	6.2329	6.6834	6.0464	5.9018
7.0634	6.1249	6.3883	6.1949	6.5003	5.9648	6.09	7.0606
6.9714	6.218	6.56	6.0369	6.8227	6.5899	6.3225	6.0697
5.8097	5.9708	6.3507	5.9954	6.2997	6.7935	5.8774	5.5617
6.3199	6.8721	5.4032	6.1553	5.7298	6.5615	6.2144	5.972
6.0329	6.2313	6.4455	5.9071	6.2826	5.7724	5.8059	6.7459
6.4321	6.5359	6.1276	5.9091	5.2435	6.0494	6.4455	6.1326
5.9806	5.801						
ATL1	1.5618	0.404	1.4286	2.2652	0.4861	0.7194	0.4506
2.1225	1.993	1.4231	0.8325	1.4983	0.2318	1.4971	2.9187
1.326	0.3994	2.7307	1.0501	0.7571	0.7665	0.3635	2.2214
0.7022	1.1908	2.6943	0.3574	0.6904	2.4655	0.2266	1.2731
1.9877	2.2353	0.3004	0.2825	2.125	0.5631	0.2658	0.727

0.3921	0.5464	0.3317	2.3589	2.8803	1.2044	0.7221	0.2733
0.4637	0.678	0.9807	1.6054	1.3561	1.7299	1.1531	2.3299
1.7732	0.3545	1.3424	0.8247	1.4397	0.4429	0.9178	0.5285
1.3703	2.2397	0.8056	0.4624	1.3716	0.5759	0.8571	1.4676
2.1621	1.2761	1.6037	2.3745	0.2436	0.0706	1.179	2.1414
0.5415	0.4619	1.6808	2.8691	0.4603	3.1912	1.7179	2.4856
1.8948	1.6699						
ATL2	4.6653	4.3987	4.3548	5.2875	4.2085	4.5946	4.3781
4.4155	4.915	4.4534	3.8403	5.0192	3.9917	4.3604	4.7519
4.1273	4.3653	5.505	4.559	4.1923	4.276	3.8192	4.4883
4.81	4.3679	3.7957	4.2418	4.0979	4.4478	4.9559	4.5799
4.5699	4.7266	4.31	4.0789	4.9683	4.9605	4.263	3.7551
4.7223	3.8814	4.0999	5.4673	4.7282	4.8526	4.8167	4.9366
4.7822	4.3737	4.6402	4.446	4.4402	4.8779	4.2732	4.7649
4.6583	3.5865	4.6875	4.2423	4.8866	4.4475	4.6598	4.6562
4.3677	4.986	3.9333	5.0223	3.8685	4.9347	4.4023	3.7933
4.3859	4.8782	4.9203	3.8327	4.2138	4.2485	4.3077	4.6493
4.4109	4.8918	3.7124	3.8361	3.9222	4.3586	4.7414	3.937
4.3857	3.7848						
ATL3	4.5701	3.503	4.5088	4.5055	3.9591	5.3653	4.5059
4.1596	4.9497	4.2575	3.4907	3.864	4.6463	5.0668	2.6337
4.2713	5.2973	3.9956	3.6267	4.2567	4.633	4.6155	4.2481
4.8496	4.0351	4.2643	3.8656	3.3073	4.6753	3.1922	3.5573
3.3108	4.4999	3.9675	4.4885	4.5391	5.0325	3.3058	3.9159
4.9685	3.5204	4.6982	5.4312	5.2092	4.2422	5.3489	4.421
4.7613	3.7506	5.2208	4.5541	4.8948	4.1153	4.7997	4.2134
3.923	5.488	3.2037	4.5384	3.0037	4.1539	3.7753	4.2393
4.7677	4.5539	4.6853	5.0117	5.2917	3.9513	4.7	5.2823
4.3167	4.0541	3.687	4.3122	4.5548	4.8732	4.0019	2.7332
4.4013	3.7235	4.6553	4.8634	4.1805	4.1296	3.6375	4.9946
4.4022	4.3235						
ATM	4.47	3.643	3.7947	3.7831	2.6202	3.8702	4.5951
2.6687	5.6532	4.9766	4.5983	2.4799	2.5482	5.0125	2.8849
4.8228	4.2208	4.8601	4.5691	3.0276	2.7945	3.7803	3.6212
2.8832	2.7897	3.4572	4.0643	3.6907	3.9094	4.0903	3.2945
3.2573	4.3182	4.2613	2.976	3.7778	3.5519	3.3633	4.0544
4.7322	3.5752	3.438	3.2429	5.5187	3.8784	2.8391	4.375
4.9014	4.4333	3.7676	4.0944	3.5733	5.3212	4.9831	2.4952
4.5173	4.4143	2.338	4.4638	2.1872	2.1885	3.4129	3.5453
4.8628	3.5687	3.6186	4.0794	3.8461	4.704	3.0139	3.9968
4.0844	5.054	4.0471	4.3646	4.7673	3.4772	3.546	5.4677
3.701	4.0685	4.0471	3.2469	4.4866	2.6659	4.5511	5.0971
5.0009	4.8433						
ATMIN	3.8309	4.0968	3.6918	4.2063	3.8341	3.8894	3.6546
3.7722	3.8755	3.4518	3.3798	3.2834	3.0873	4.2213	3.9844
3.5219	3.8228	4.0938	3.633	3.688	3.7341	3.5509	3.8601
4.3252	3.9647	4.4861	4.0708	3.895	3.1558	3.3438	3.9979
3.9049	3.1613	3.3407	3.9782	3.5026	4.0334	4.2481	3.5532
3.8256	3.7609	3.8594	3.1226	2.8017	4.3815	4.0579	3.8723
3.7828	3.1347	3.3645	3.4677	4.1591	3.8345	3.8777	3.875

3.5574	4.1688	3.6462	3.6923	4.056	3.4025	3.5441	3.8774	
3.6218	3.7037	3.9119	2.9566	4.1397	3.9852	3.9217	3.9673	
3.5056	2.7609	3.2078	4.0991	3.6398	3.4425	4.3902	4.1933	
3.2324	3.6281	3.8665	4.0463	3.7786	3.5251	3.4048	4.0381	
3.7253	3.9794							
ATN1	4.8049	4.8122	5.4664	4.4742	5.6761	5.3199	4.506	
5.3951	4.734	4.3193	5.0185	4.301	5.8568	4.6044	4.9969	
4.8857	5.4782	4.6593	4.2086	5.0281	4.0718	5.9223	5.2249	
4.7704	5.6002	5.5874	5.8182	5.3343	4.6982	4.7944	5.4952	
4.787	4.3728	5.1105	6.0269	3.8148	5.1789	5.0345	5.1402	
4.5604	5.3079	5.6837	5.5068	5.0245	5.442	5.0245	4.5418	
5.0988	5.5883	4.8565	5.9725	4.2648	3.3369	4.3641	5.0151	
4.6649	5.5865	5.1986	4.5483	5.2146	5.1201	4.8431	5.824	
4.8204	4.8109	5.3562	3.9567	5.4325	4.9519	5.2348	5.2819	
4.9519	4.9335	4.3983	4.6701	5.2513	4.8198	4.6273	4.7399	
4.6766	5.4066	5.3056	5.4005	5.5312	5.1102	3.0275	5.0498	
4.8619	5.146							
ATOH1	0	0	0	0	0.0962	0	0	0
	0	0	0	0	0	0	0	0
	0	0	0	0	0.15	0	0	0
	0	0	0	0	0	0	0	0
	0	0	0	0	0	0	0	0
	0	0	0	0	0	0	0	0
	0	0	0	0	0	0	0	0
	0	0.1487	0	0.0425	0	0	0	0
	0	0.0147	0	0	0	0	0	0
	0	0	0	0	0	0	0	
0.0786	0	0	0	0	0	0	0	0
	0							
ATOH7	0.1981	0.233	0.104	0.0844	0.1103	0	0	
0.0456	0.1468	0.4488	0.2236	0.2512	0	0	0.0391	0
	0	0.0852	0	0.3249	0.2585	0.0651	0.0233	0
	0	0	0.5557	0.1857	0.0489	0.142	0.2301	0
	0.3179	0	0	0.2075	0.1838	0.1985	0.1247	
0.1061	0.0891	0.0855	0	0.0643	0.0366	0.1106	0.0772	
0.1364	0.1398	0.6445	0.2419	0.1546	0.3802	0.133	0.4486	0
	0	0.1061	0	0.2674	0	0.1414	0.0945	
0.0762	0.2268	0.0211	0.6576	0.2344	0.1451	0.1626	0.1293	0
	0.0585	0.0708	0.0807	0	0.3343	0.054	0.0362	0
	0.2933	0.1223	0	0.0528	0.1184	0.2074	0.0752	
0.0798	0.2248							
ATOH8	1.2153	1.1312	1.672	0.9778	1.0966	3.2563	1.856	
2.4365	1.7923	2.608	2.92	1.9764	1.1422	1.5409	0.6648	
3.6601	0.2593	0.7143	1.5528	0.1723	0.9227	2.9766	0.6753	
2.9513	3.6572	1.4951	1.9745	2.3048	1.342	1.4337	0	
2.4295	2.2019	0.1838	3.2913	1.6078	1.1618	2.8566	4.2359	
2.948	1.6423	1.0287	0.9454	2.9704	0.3627	2.356	1.0294	
0.901	5.5994	0.0141	1.6996	2.0213	1.407	1.4152	0.8277	
3.1014	0.1811	0.1643	1.2152	0.4383	2.8192	0.626	4.688	
0.811	2.6225	2.984	3.0899	1.3263	1.4142	1.7848	0.2187	

0.2239	3.3446	3.4451	2.6073	2.3698	1.621	2.4821	1.737
2.5282	1.1955	0.3581	3.5824	3.3705	4.0345	1.5107	0.3447
0.587	0.7394						
ATOX1	7.9264	8.61	9.9213	7.8184	8.4657	8.5526	10.3621
7.4229	7.7103	8.5213	9.0726	8.2493	7.2752	8.8774	7.8825
9.1676	7.378	7.5976	10.0245	10.7742	8.1037	8.141	8.0188
8.5325	7.8728	7.1325	6.3073	8.3287	8.6538	9.0868	6.6353
9.0179	8.7809	8.3737	8.2785	8.7272	6.7425	7.2306	7.6358
7.7183	7.9042	6.8043	7.3171	8.5213	6.293	6.612	8.4932
8.0432	7.3957	5.8866	7.736	6.6371	7.5826	7.2077	6.5503
9.2368	7.0422	7.8335	10.1085	7.4908	8.6889	10.1458	7.4976
9.0238	6.0757	6.5199	6.083	8.0378	8.363	7.7444	8.378
8.5048	9.4613	9.1043	7.7021	9.389	9.0781	7.3937	7.5695
7.851	8.7004	6.8892	7.5338	8.124	7.0375	8.3537	8.6738
9.2898	8.0432						
ATP10A	3.485	4.5699	1.9942	0.2733	1.7603	0.1708	2.7907
0.1581	3.0185	4.8528	5.2547	1.8319	0.1416	3.1821	0.2249
1.2319	0	4.2841	3.6949	1.6942	3.2214	0.2001	0.126
2.6532	2.7802	0.3838	0.1921	3.2614	1.0376	3.9424	0.3539
2.0188	2.676	4.309	1.9863	3.887	0.8402	3.3246	3.2595
1.149	0.9615	0	0.6125	0.6282	0.1888	0	0.8013
2.7252	0	0.1265	3.2729	0	2.911	4.1965	0
3.9158	0	0	4.9831	0	1.4426	3.8467	1.9769
3.3743	4.3307	1.6995	2.9539	2.5315	2.6504	1.5923	1.715
1.8776	2.6938	0.157	0.6434	1.4132	4.4234	1.133	3.3145
3.2085	2.9463	0	0	2.017	1.848	2.4643	5.7281
5.2366	5.8244						
ATP10B	1.2473	0.0154	0.1273	2.8113	1.4665	3.1654	2.8136
1.9279	2.6587	0.8038	0.3896	2.6598	0	2.7974	2.6158
2.8701	0.2873	0.6755	2.5768	1.9179	1.6431	1.5581	2.0594
3.171	3.1617	0.0104	1.9022	0.517	2.1814	1.8586	0
2.3926	3.2636	3.0366	3.4317	0.9567	2.013	1.9069	1.1931
3.0779	1.883	0.031	2.0572	1.2242	0.0286	0.0211	1.1565
2.3462	2.4154	0	1.661	2.0445	2.2021	1.5567	0.0989
1.2659	0.0753	1.4801	1.5714	1.6828	0.7462	2.4209	2.7148
1.4704	0.385	0.0481	1.8103	0.0524	0.4494	3.5787	2.854
1.1044	2.3004	2.9907	1.467	0.293	0.5577	4.3306	3.2558
1.9197	1.9827	1.6918	1.4878	2.7254	1.3064	1.8746	0.3835
0.3575	0.105						
ATP10D	1.7464	0.8992	2.5082	3.1781	1.8032	1.7527	2.7352
1.8718	2.6156	0.9153	1.3788	3.0577	0.7077	2.3141	1.6411
2.9087	2.2199	1.8032	1.4226	2.4351	2.1679	2.0591	2.481
1.8277	1.9686	4.3381	2.5932	4.1115	1.4187	3.0474	3.2969
2.1365	2.1365	1.762	3.2129	2.1834	2.6478	2.5001	0.8757
2.4458	1.9692	3.173	1.8147	1.6805	2.4243	3.8867	2.8267
2.2658	1.9648	2.6306	2.0574	2.3805	2.8969	1.099	2.5806
2.6598	2.5793	1.6378	1.5165	2.0022	1.8638	1.6498	1.7147
2.1755	2.2206	3.6059	2.2108	2.6753	2.7189	2.2039	2.1108
1.9451	1.6486	2.2227	2.1135	4.9645	1.9159	3.1601	1.5681
1.1214	1.7617	2.7573	2.736	2.8614	2.7109	2.1005	2.6567

2.0019	2.4517							
ATP11A	4.2909	3.8563	5.6962	4.0617	3.258	4.6175	3.0518	
3.8835	3.5839	5.5492	4.1436	3.5933	1.8011	3.7779	2.1612	
3.8266	0.0154	4.2716	4.4546	4.1933	4.1738	3.0184	3.5717	
3.7526	3.6615	1.6615	3.2413	4.7972	2.865	4.4426	5.1358	
3.0181	3.3914	3.0769	2.383	4.7832	2.6572	3.7488	4.6088	
3.1619	0.001	1.8209	3.2107	4.7759	3.2292	2.6513	3.8819	
3.2089	2.5836	1.567	3.7069	0.1215	3.3154	3.0257	4.0305	
4.9508	2.5998	3.8139	5.0741	4.0347	1.3633	3.5606	4.4323	
3.9391	3.6049	1.597	1.6712	2.2249	5.8666	4.978	2.3209	
3.1268	3.733	3.8574	3.3869	4.8708	3.6013	3.0968	4.4584	
3.7672	4.3694	2.7567	3.0066	3.2182	2.0381	2.5073	3.3976	
3.1918	3.0573							
ATP11B	4.0402	3.8413	5.0032	4.7274	4.0515	4.2067	5.1114	
3.8266	5.3621	4.5268	4.4735	5.6945	4.4735	5.0096	5.1243	
4.1211	4.5815	4.574	4.4605	3.9925	4.6954	4.3548	4.5824	
5.2985	3.6995	3.4829	5.2661	4.3715	4.6987	4.3283	4.6997	
4.2682	4.8371	5.4226	4.2332	4.5736	4.195	4.1267	4.28	
5.0025	5.2646	5.0383	4.9127	4.0729	5.1642	4.4027	4.9288	
5.3106	3.7976	4.5412	4.8493	5.151	4.8454	4.2606	4.885	
4.4544	5.8425	4.8528	4.2175	4.9976	4.5224	4.4352	4.0197	
4.9785	3.5829	4.1354	4.8181	3.5469	4.5944	4.1148	4.2211	
4.8997	4.0225	4.7581	3.9483	4.5421	4.8247	4.6958	4.8594	
4.6418	5.0844	4.7613	4.3181	5.4565	3.8438	4.4859	4.5563	
4.5534	4.7666							
ATP11C	3.0065	0.4981	2.8094	2.4377	2.3829	3.0239	3.2712	
2.8089	3.2026	2.5866	3.4354	2.7574	2.4908	2.3567	3.5938	
2.8601	3.8359	2.7031	2.0883	2.4989	2.55	2.7507	2.9497	
3.0788	2.7594	3.1335	2.8129	1.2917	2.732	0.0128	3.2738	
3.2177	2.827	3.6128	2.8781	2.9335	2.0581	2.6605	2.2261	
3.777	3.0701	2.5363	3.2296	2.516	4.1754	3.0335	2.9011	
2.9877	3.025	4.0168	2.8458	2.509	3.1116	2.8933	3.7929	
3.9191	3.5016	3.0681	2.189	3.5256	2.2016	1.3063	3.2332	
3.3614	3.3109	2.9423	3.0264	2.4148	0.2786	2.3875	2.6227	
2.4885	1.5652	1.9726	2.6884	2.9472	2.6624	3.0249	3.2552	
2.7313	2.4731	2.5397	1.9197	3.0993	2.0942	2.9157	2.7826	
2.7476	2.8555							
ATP12A	0.0471	0.6454	0.179	0.0962	0.0535	0.1188	0.2329	0
	0.0039	0.0062	0.0611	0.0603	0	0	0	
0.0302	0.0234	0.3003	0.1229	0.0432	0.0388	0	0.0969	
0.0097	0.1051	0	0	0.1997	0.0076	0.2914	0.0114	0
	0	0.0344	0.2446	0.0532	0.1194	0.1396	0.0017	
0.0191	0.3152	0.0181	0.1277	0.0688	0.0011	0	0	
0.2994	0	0.171	0.1421	0	0.0949	0.0229	0.0254	
0.2128	0.2711	0	0	0	0	0.0241	0.0167	0
	0.0416	0	0.1579	0.0956	0	0.0797	0.1028	0
	0.2534	0.0103	0	0.021	0.0281	0.081	0.3349	
0.5429	0.1496	0	0	0.023	0	0.0465	0.0162	
0.0652	0.0585							
ATP13A1	3.982	4.5706	4.4704	3.2963	5.2235	4.0683	3.6284	

4.3715	3.4976	4.0733	4.249	4.1246	3.4802	4.152	4.6693	
3.9388	4.1771	3.282	3.8216	4.3673	4.512	4.6362	4.9789	
4.2973	4.2368	4.0294	4.3028	4.3773	4.6923	4.2475	4.5405	
5.2806	4.4864	4.4213	4.3271	3.3999	4.357	5.0993	4.7152	
4.0463	5.1608	4.338	3.5738	4.1402	3.4922	3.3966	3.5168	
3.5201	4.6522	2.8877	3.7501	4.1451	3.4239	5.242	4.547	
4.8984	3.0933	4.6046	4.0048	4.5009	5.1185	4.705	5.3117	
4.086	4.6456	4.8336	5.1883	4.386	4.4276	4.016	4.6237	
4.2117	3.8453	4.1974	4.0343	4.1712	4.2472	4.6549	4.5091	
4.01	3.7556	4.9438	3.8434	4.5248	4.058	3.6059	4.1634	
4.2164	4.4381							
ATP13A2	4.2174	4.1507	5.0873	3.4733	4.4779	3.6962	3.9074	
3.1154	3.8488	4.7936	4.5956	3.4422	5.205	2.7945	4.3999	
4.4357	3.1099	3.6414	3.8023	4.5062	4.9491	4.393	4.8779	
4.7357	3.5542	3.4178	1.6449	4.558	4.4645	3.8589	4.2239	
4.2751	5.1456	5.3267	4.5302	3.7211	3.829	5.1379	4.9024	
3.7755	4.9358	4.0238	3.6996	3.7646	3.5172	2.6285	3.5176	
3.6511	3.8747	2.566	3.7851	3.1016	3.9914	4.2114	3.2391	
4.0401	3.8903	3.5987	4.9302	3.4846	4.1522	5.0267	3.6608	
4.4919	4.5013	3.0509	4.1567	4.4973	5.1821	3.7237	3.6623	
3.5061	4.0684	4.4232	3.3648	5.4341	6.0634	3.8633	4.1664	
4.3766	4.8011	3.9543	4.0049	3.5531	3.7328	4.8033	4.0929	
4.4113	4.4047							
ATP13A3	4.9406	5.0398	5.8949	5.1918	5.085	5.5489	5.4	
5.2947	5.4667	5.6985	5.1254	6.1372	5.535	5.6193	5.8896	
5.2802	6.4752	5.4866	5.8896	4.2407	5.1027	5.2277	5.0979	
6.3004	4.9291	5.0856	5.5254	4.4226	5.5472	4.1057	5.7163	
5.3683	5.5262	5.1889	5.1866	5.7578	5.2856	4.66	4.9884	
5.8439	5.2782	5.2006	5.5942	5.389	5.4228	5.1918	5.4381	
5.7097	5.1505	6.0255	5.4541	5.8537	5.6777	5.0607	5.2703	
5.567	6.0625	5.9174	4.95	6.7303	5.473	5.557	5.3636	
5.1894	4.885	6.6193	5.7755	4.5752	5.4303	4.8736	5.2316	
5.8878	5.0063	5.3081	4.7263	5.1447	4.8761	4.9667	5.0797	
5.3773	5.0507	5.242	5.5562	5.8956	4.984	5.0983	4.8321	
5.2184	5.1287							
ATP13A4	0.0859	0	0.1162	0	0	0	0.0046	0
	0.0866	0	0.0633	0.002	0.0236	0	0.0649	
0.0039	0.1157	0.0191	0	0	0	0.0145	0.0289	0
	0.0179	0.0222	0	0.0486	0.011	0.0448	0	
0.0022	0.0134	0.0952	0.0058	0.0309	0.0018	0	0.0053	0
	0.0081	5.00E-04	0.0208	0.04	0	0	0	0
	0	0.0653	0.0431	0	0.0308	0.0156	0	0
	0.0595	0	0	0	0	0	0	0
	0.0537	0.0034	0	0.0119	0	0	0	
0.1148	0	0.0027	0.0682	0.0504	0	0	0	
0.0379	0.0139	0.1036	0	0.046	0	0.11	0.0842	
0.0375	0.1707							
ATP13A5	0	0	0	0	0.0049	0	0.0092	0
	0.0614	0.3828	0.1751	0.0458	0.0097	0.0739	0	0
	0.0463	0	0.0999	0.0451	0.046	0	0.0048	

0.0231	0	0	0	0	0	0.0908	0	0
	0	0	0	0.1918	0	0	0	0
	0	0.0026	0.0382	0	0	0	0	0
	0	0	0	0	0	0.0031	0	
0.0416	0	0.0113	0.0354	0.0095	0	1.0339	0	0
	0.0128	0	0	0	0	0	0	0
	0	2.00E-04	0	0	0	0	0.0019	0
	0.0049	0	0	0	0	0	0	0
	0							
ATP1A1	9.6316	8.5901	8.9745	8.5597	8.2489	8.7204	10.2062	
8.8686	8.6764	9.8915	9.159	8.7329	7.6839	9.8071	8.4958	
9.7573	7.4306	8.9745	9.9637	9.274	8.9225	9.1195	8.2489	
8.5597	7.8636	7.3915	8.7582	9.2594	8.1514	9.5371	6.3174	
8.302	9.3762	10.0366	9.4885	8.8572	8.7329	8.6764	9.0143	
8.8207	8.8079	7.9766	7.8286	9.3625	7.2376	6.8838	8.6417	
9.2594	8.8079	6.9234	9.159	8.8816	8.9225	9.2921	6.5246	
9.9867	7.2142	8.2535	10.1026	7.518	7.7576	8.6558	8.2657	
9.5999	8.2477	7.3875	9.2921	7.5596	8.753	8.09	8.8832	
9.3482	9.0485	8.6856	9.0746	8.7048	9.9007	9.502	8.7248	
8.6068	9.2139	8.814	8.6468	8.9727	7.6822	8.46	9.4261	
8.8686	9.1451							
ATP1A2	0.0759	0.2462	0.0218	0.044	0	0.02	0	
0.0195	0	0.0494	0	0.0055	0.0524	0.1169	0	
0.0241	0.0426	0.0157	0.0679	0.0408	0.0209	0.0969	0.0022	
0.0179	0.0163	0.0459	0.0493	0.1118	0.0039	0.0062	0	
0.0157	0.0071	0.0325	0.0406	0	0.0341	0.0256	0.0708	
0.0282	0.1054	0.0175	0	0	0.0544	0.0384	0.0293	
0.0224	0	0.0747	0.0301	0	0.0528	0.035	0.0189	0
	0	0.0189	0.0249	0.0242	0.0147	0.0377	0	0
	0.0208	0.1124	0.0745	0	0	0.032	0.0178	
0.0314	0.0162	0.0152	0.0258	0.0161	0.0094	0.052	0.0447	
0.0356	0.0188	0.0265	0	0.0231	0.0321	0	0.0267	
0.0305	0.0567							
ATP1A3	0.6088	1.5464	2.2883	0.1829	0.0223	0.7297	0.4454	
0.1061	0.1193	1.1375	2.343	0.1582	2.6806	0.0971	0.1756	
0.281	1.0625	0.2622	0.1526	3.6796	1.2054	0.4069	0.2247	
0.0357	0.2075	0.1511	0.0408	0.0996	0.7701	0.777	2.1494	
0.0857	0	0.1327	0.0822	0.9609	0.2335	0.0699	0.8044	0
	0.0415	0.0609	1.0916	0.035	0.1928	2.3409	2.2575	
0.1565	0.0774	0	0.5583	0	0	1.2178	1.7168	
2.192	0	0.3282	0.9782	0.0432	0.9871	0.9669	0.0704	
0.602	0	0.4148	0.3457	0.1176	0.0195	0.1196	0.4756	
1.0353	1.9675	0.2106	1.00E-04	0.7545	0.1088	0.288	0.0021	
0.1155	0.2416	9.00E-04	1.4386	0	0.0581	0.0465	0.2538	
0.2245	0.1766							
ATP1A4	0.0468	0.4779	0.3787	0	0.0169	0	0.2965	
0.0153	0.2443	0.0407	0.3139	0.068	0.0209	0.1034	0.0097	
0.0497	0.0443	0.2168	0.0414	0	0.0232	0.0046	0.0051	
0.0229	0.0345	0.0143	0.0371	0.0286	0.0102	0.926	0.0372	
0.124	0.0507	0.0576	0.0188	0.0406	0	0.2758	0.0134	

0.0317	0.0416	0.0516	0	0.0368	0.0785	0.0107	0.3337
0.0152	0.0164	0.0448	0.1413	0.0131	0.4066	0.0168	0.0628
0.1565	0.02	0.048	0.0593	0.0301	0.0375	0.7769	0.0228
0.0222	0	0.0348	0.0304	0.0699	0.0173	0.0391	0
0.1387	0	0.0246	0.0738	0.0864	0.3004	0.0149	0.0336
0.0292	0.5804	0.0845	0.0313	0.0181	0.0683	0.0863	0.0184
0.0773	0.0428						
ATP1B1	3.5796	4.8643	3.5158	6.4457	5.1871	6.7661	8.03
4.9677	3.5239	3.7394	3.108	6.7285	5.4463	1.5916	4.8309
6.6315	5.4731	4.9422	3.4941	4.5564	3.4719	5.0374	5.228
5.7306	4.9538	3.6286	7.0957	4.0583	4.5623	5.9972	4.4481
5.7844	3.9313	5.6499	5.307	4.2155	3.173	5.4664	3.6648
5.4119	5.7058	5.7806	6.0957	2.9281	5.592	3.5938	3.8286
6.0766	3.5012	4.2761	3.8997	5.882	4.8784	1.0691	4.974
2.6127	5.444	5.2897	5.1	4.6886	5.0129	3.0772	5.6909
3.794	3.6829	5.9568	3.8709	1.7304	5.9717	5.8881	5.9695
2.8587	2.0314	5.8665	5.8581	5.5037	3.4808	6.9482	4.7334
4.2671	4.4533	5.9894	6.1728	6.0965	6.0671	4.2662	2.6985
1.6863	1.4576						
ATP1B2	0.2059	0.7885	0.2557	0.2651	0.2746	0	0.1591
0.5354	0.3776	0.3572	0.4973	0.5053	0.1665	0.2517	0.0954
0.049	0.077	0.259	0.0538	0.1385	0.0845	1.7604	0.8401
0.1663	0.3441	0.0865	5.6456	0.1553	0.9388	1.6727	3.6193
0.074	0.0165	0.1781	0.2677	1.2715	0.029	1.9972	1.6624
3.2701	1.245	0.1497	0.026	0.061	0.1579	0.0534	0.6303
0.187	0.3096	0.325	0.0114	0.729	0.0767	0.3599	1.3365
0.3789	0	0.933	0.1196	0.1033	0.3487	0.0869	2.0881
0.2184	0.1013	0.0829	4.0165	0.1209	0.1004	0.2627	0.2458
0.0438	0.1239	3.2701	0.1366	0.0978	0.1789	3.3673	0.1788
0.9238	0.7411	0.5346	0.1248	0.602	0.1899	0.1989	0.395
0.356	0.7608						
ATP1B3	6.4489	6.5964	7.5041	7.5562	6.9224	7.8038	8.3582
6.9665	7.3757	6.9639	7.6777	7.1334	6.4374	7.315	6.8305
7.6092	6.3541	6.8972	7.4252	7.0088	6.7999	8.1738	7.5072
8.5211	6.6726	6.3921	7.2427	7.9694	7.28	7.0552	7.8714
8.0627	8.0199	8.1191	7.5919	7.2736	6.6849	6.4489	7.8127
8.3327	7.4217	6.5869	7.2103	7.1865	7.0429	6.585	8.0988
7.4939	6.6351	6.3655	7.3969	8.472	7.5142	6.6715	6.871
7.263	7.174	8.5074	6.7625	8.48	7.4069	7	6.9095
7.8206	6.1207	5.8065	7.9419	6.9639	7.7633	5.8948	7.7596
7.6231	6.9385	7.7851	7.3299	7.5339	7.4429	7.6231	7.8684
7.8781	7.3649	6.9978	6.962	7.4723	6.4059	7.2883	7.3621
7.0777	7.1184						
ATP1B4	0.1606	0.0689	0.0628	0	0.0148	0.039	0.0086
0.0251	0.0387	0.0448	0.0103	0.0096	0.0436	0.04	0.0383
0.0509	0.0352	0.0665	0.0698	0.0572	0.1211	0.0029	0.0097
0.039	0.029	0.0107	0.0463	0.0764	0.0365	0.0242	0.006
0.0092	0.0201	0.0097	0.1214	0.0104	0.0178	0.0547	0.0094
0.0195	0.0224	0.0058	0.0197	0.0378	0.0486	0.0103	0
0.0604	0.0584	0.039	0	0.0269	0.0501	0.027	0.029

0.0903	0.0347	0.0448	0.0212	0.0118	0.0284	0.0264	0.0739	
0.0556	0.0176	0.011	0.0473	0.0232	0.0614	0.036	0.0526	
0.0352	0.0101	0	0.0069	0.1114	0.0034	0.0087	0.0057	
0.1122	0.0311	0.0609	0.0041	0.0767	0.0592	0.0515	0.0257	
0.097	0.106							
ATP2A1	0.721	0.6899	0.8362	0.2449	0.2158	0.6151	0.2359	
0.2878	0.4286	0.256	0.3555	0.1022	0.242	0.3162	0.3247	
0.3455	0.709	0.2907	0.4498	0.654	0.729	0.4053	0.18	
0.4482	0.2091	0.2641	0.2996	0.9711	0.4815	0.2387	0.299	
0.6648	0.2405	0.7862	0.2143	0.0782	0.2933	0.2589	0.1901	
0.3749	0.8796	0.3989	0.2115	0.5904	0.277	0.3088	0.1977	
0.6057	0.2021	0.5474	0.3673	0.0515	0.3424	0.4238	0.513	
0.329	0.4495	0.4	0.2616	0.3739	0.2292	0.5022	0.5503	
0.7562	0.1658	0.2168	1.1886	0.229	0.5479	0.5508	0.3183	
0.4073	0.15	0.2391	0.713	0.3791	0.7082	0.2182	0.2801	
0.3102	0.5913	0.1758	0.0928	0.1138	0.3055	0.2603	0.2279	
0.2852	0.2454							
ATP2A2	6.2348	5.8808	6.5942	6.5347	6.5729	6.8388	6.6665	
6.7258	6.8021	6.5347	6.0788	5.9985	5.0654	6.1206	6.4194	
6.6587	6.364	6.8603	7.2571	6.0115	6.2107	6.3334	6.0532	
6.7327	6.5083	6.027	6.1987	6.1263	6.4276	5.7997	6.8341	
5.9779	6.5271	5.8218	6.4173	6.7213	6.1549	6.6495	6.1106	
7.0479	6.0615	6.1446	6.5462	7.162	6.5211	6.8924	6.8718	
6.8669	6.1378	5.9867	6.5306	6.7732	6.3622	6.2066	6.5369	
6.8837	6.2213	6.7211	7.0269	6.608	6.5609	6.608	6.1293	
6.3668	7.281	6.014	6.9924	6.1865	6.4201	6.0214	6.5932	
6.3316	6.3341	6.2213	6.5383	5.5864	6.258	6.218	6.9577	
6.7396	5.9864	6.4483	5.7795	7.0312	5.5797	6.0298	6.4646	
6.6665	7.3076							
ATP2A3	1.4789	0.6603	0.581	1.0351	0.1143	0	0.223	
0.097	1.1629	3.4146	0.966	0.3422	0.8765	0.9934	0.0908	
0.0493	0.0715	3.7535	0	0.0349	0.7386	0.1152	1.3682	0
	0.4056	0.1088	0.1338	0.5843	0.5976	2.1301	2.752	
0.245	0.5246	0.3781	0.1633	2.7103	0.188	0.2323	0.1449	0
	0.2274	0.578	1.3506	0.2431	0.4801	0.3134	0.4499	
0.8447	0.1654	0	0.6858	0.1167	0.2755	0.9842	2.561	0
	0.2062	2.5872	3.8472	1.9706	0.2992	1.3666	0.0884	
0.5664	0.4838	0.22	1.3318	0.4186	0.4354	0.578	0.1464	
0.4749	2.0037	0.3133	0.227	0.178	0.1609	0.0791	0.6974	
0.1347	0.1788	0.146	0.0436	0.0979	0.345	0.4179	0.7649	
0.6972	0.7502							
ATP2B1	3.1843	2.652	3.3124	4.4066	3.7647	4.3614	5.6956	
3.7299	3.4606	3.5633	2.9849	4.7708	5.783	3.3368	3.3599	
4.3019	4.9181	4.0545	3.9121	3.8303	3.7584	4.1191	4.2434	
3.3031	4.3276	5.796	5.0038	3.2986	4.558	3.489	3.801	
3.7095	3.5647	3.3667	4.1601	3.9326	3.975	3.6915	3.8173	
5.2597	3.5518	4.3141	2.9849	2.9592	3.6894	5.3634	3.5351	
6.0502	3.9424	5.0326	4.1585	5.4417	4.0373	3.0965	3.6634	
4.1547	3.2396	3.6038	5.0085	3.5316	4.7062	3.5556	2.8663	
3.748	3.3973	6.5845	3.9153	3.4135	3.5716	3.1254	5.34	

4.0643	3.1453	4.0342	3.4216	3.6936	3.1621	3.8946	3.1221	
3.8875	3.6175	4.7709	5.1789	4.52	3.2948	3.7603	2.9749	
2.9424	2.7864							
ATP2B2	0.0511	0.142	0.0447	0.0185	0	0.0256	0.1361	0
	0.0634	0.0265	0.1334	0.0077	0.2103	0	0.0121	
1.4497	0	0.039	0.0302	0.2585	0.0174	0.0657	0.0176	
0.0372	0.0576	0	0	0.021	0.3303	0	0.1621	
0.0066	0.0171	0.0115	0	0.0075	0.002	0	0.0142	
0.0042	0	0	0.1079	0.0079	0.0167	0.0349	0	
0.0294	0.0281	0	0	0	0.0727	0.027	0.0381	0
	0.0834	0.0408	0	0	0.041	0.0473	0.0309	
0.0658	0	0.0377	0	0.0446	0.02	0.4012	0.0282	
0.0841	0	0.0215	0	0	0.0332	0.1192	0.0149	
0.0525	0.0239	0.0184	0.0299	0.0681	0.0494	0	0	0
	0							
ATP2B3	0.0351	0.0239	0.0388	0	0.003	0	0	0
	0	0.0721	0	0	0.0042	0	0	0
	0	0.0383	0	0	0.0372	0.0023	0	
0.0214	0	0.0117	0.0241	0.0815	0	0	0.0091	0
	0	0	0	0	0	0.089	0.0102	0
	0	0	0	0	0	0.021	0	0
	0	0	0	0	0.054	0.0097	0.0149	
0.0327	0	0.0143	0.0088	0.0126	0	0	0.0127	0
	0.0574	0.0104	0.1747	0	0	0	0	0
	0	0	0	0	0	0.0078	0	
0.0217	0.005	0	0.0074	0.0101	0	0	0.0226	0
	0.0442							
ATP2B4	4.5154	2.7027	3.0895	4.873	4.5045	6.8489	4.7721	
3.8627	5.4908	5.4436	5.1111	5.3892	3.9977	4.9447	5.3434	
5.8127	1.5719	5.4806	4.4948	4.8568	4.1468	3.9857	4.3124	
5.0222	4.4041	4.8539	5.1478	3.9325	4.7132	4.8165	3.9977	
4.3483	4.2699	5.134	5.7706	4.8925	3.5948	4.663	5.3101	
4.7555	5.1888	4.4675	5.5074	5.007	4.1814	3.1858	3.5154	
4.7155	2.3302	3.2552	3.7994	2.8625	4.8126	3.2006	1.9969	
3.1054	4.7737	3.5858	5.7905	2.5001	4.8194	4.0678	5.3439	
4.483	4.3021	5.4708	5.1339	5.7149	4.67	3.7169	5.4644	
4.5158	3.6533	4.6543	5.148	3.939	3.9451	5.9615	5.0323	
3.9109	3.7808	4.2963	6.9184	4.9374	4.5772	4.0285	5.1039	
3.835	4.4144							
ATP2C1	4.9666	4.7464	5.2509	5.4907	4.7792	5.2541	5.4907	
4.5349	5.6075	5.2633	5.4395	5.3797	5.0625	5.1604	5.56	
5.0586	5.5983	5.8825	5.9633	4.5968	5.0394	5.2569	5.3057	
4.7503	4.2542	5.5791	5.6065	5.2266	5.1611	5.0631	4.7665	
5.4372	5.3429	5.7801	5.2447	5.2221	3.5702	4.9415	4.8202	
5.7246	5.1712	5.4028	5.3869	4.4783	6.4774	6.3814	5.8256	
4.8103	5.2433	5.3057	5.6703	5.5372	6.1027	4.7884	4.5059	
5.5384	5.6372	5.1092	4.5007	5.552	5.2721	5.1351	3.7878	
5.1562	5.1067	5.9547	4.7501	5.7725	5.6314	4.1221	5.4292	
5.7402	5.0346	5.7997	4.5911	5.202	5.0989	5.1339	5.0006	
5.334	5.0229	5.1957	5.3002	5.7843	4.6921	5.8149	5.8035	

5.3565	5.4078							
ATP2C2	0.1403	0.1936	0.041	0.0759	0.331	0.1081	0	
0.0341	0.0752	0.1815	0.242	0.0115	0.6749	0.158	0.0567	
0.0544	1.6604	0.2733	0	0.0453	0.3694	0	0.0659	
0.261	0.1116	0.2301	0.2357	0.105	0.0408	1.3538	0.174	
0.1671	0.0508	0.0807	0.1438	0	0.191	0	0.0576	
0.1554	0.2395	0.174	0.3146	0.0271	0.1394	0.0977	0	
0.1328	0.1944	0.3965	0.0206	0.1541	0.0491	0.1025	0.2362	
0.1778	1.1167	0.0243	1.6411	0.0506	0.1281	0.1189	0.15	
0.2345	0.1426	0.1203	0.2153	0.2009	0.2263	0.3109	0.0249	
0.131	0	0.0223	0.1404	0.0808	0.187	0.0061	0.0835	
0.041	0	0.1584	0.0643	0.0711	0.1725	0.1737	0.034	0.2
	0.2043							
ATP4A	0	0	0	0	0	0	0.0292	0
	0.0981	0.0362	0.0455	0.0076	0	0	0.0244	0
	0	0	0	0	0	0	0.013	0
	0.02	0	0.084	0	0.065	0.0484	0.4103	
0.0208	0.0424	0.0414	0	0.0648	0	0.0283	0.0474	0
	0.0296	0	0	0	0.0283	0	0	0
	0	0	0.0375	0	0	0.0303	0.0786	0
	0	0	0.2561	0	0	0.012	0.0174	
0.0309	0.0878	0	0.1142	0.0034	0	0.048	0.0149	
0.0153	0	0.0278	0	0.2692	0.0068	0	0.007	0
	0	0	0	0.0118	0.0364	0	0	0
	0.1046							
ATP4B	0	0	0	0	0.0157	0.1469	0.0294	
0.0324	0.1639	0.0361	0	0	0	0	0.025	0
	0	0	0	0	0	0.0186	0.0157	0
	0	0	0	0	0.1198	0	0	0
	0.0437	0.0406	0.0859	0	0	0	0	
0.0576	0	0	0	0.5981	0	0.0404	0	
0.0631	0.4465	0	0.0495	0	0	0	0.0715	0
	0.1317	0	0	0.064	0.0217	0	0.0754	
0.1246	0	0	0	0	0.0214	0	0	
0.0336	0	0	0.0636	0.1309	0.0536	0	0	
0.1591	0	0.3684	0	0.284	0	0	0	
0.0665	0.0771							
ATP5A1	7.5846	7.8955	8.0221	8.4237	7.5682	8.1156	7.4524	
7.6504	9.4208	8.8809	8.6486	8.1039	7.1913	8.36	7.8243	
7.733	8.1096	8.0275	8.2313	8.3071	7.9827	8.0221	7.8142	
7.7772	7.5769	7.5073	8.1586	8.3949	6.9041	8.6183	7.5006	
7.8197	7.609	8.2708	7.1716	8.5864	6.9888	8.3342	8.6579	
8.0275	8.2014	8.2081	7.6886	8.1224	7.5927	7.9991	8.2391	
8.1284	7.5453	7.8197	7.3491	7.839	8.4704	7.8057	9.4044	
7.779	6.9076	7.824	8.5353	7.9033	7.7878	7.8532	8.222	
7.7386	8.1658	7.3452	8.5047	7.9827	7.939	7.5872	8.1026	
8.1245	7.7659	8.222	7.5957	8.3577	7.8617	7.8285	8.1421	
8.0519	8.3776	7.9187	7.3853	7.7838	6.9959	8.4297	7.922	
8.1096	8.3229							
ATP5B	9.4597	9.2883	9.7108	9.5068	8.7309	9.8643	10.0049	

8.3414	9.9715	10.1441	9.1076	8.8065	8.1517	9.2763	9.0805
9.3793	8.8951	9.6646	10.2158	9.6206	9.0944	8.3864	8.492
9.1736	8.5968	8.0241	8.6839	9.0805	8.7814	9.6956	8.5564
8.473	8.8951	8.436	8.7678	10.1022	8.832	9.1604	8.7436
9.9166	8.8951	8.8194	8.4824	8.639	8.5669	8.9744	9.9887
9.4597	7.8876	9.0419	8.7565	8.832	9.1868	8.8295	9.2239
8.6861	8.2375	9.2871	10.2283	8.9294	9.3421	9.6939	9.8516
9.5603	8.8871	8.146	8.8676	8.602	8.9211	8.077	9.0743
8.4604	9.4657	8.499	8.146	10.3526	9.3697	8.5604	8.9642
9.5068	9.0274	8.5604	8.6236	8.544	8.1566	9.0965	8.7678
8.9612	8.9612						
ATP5C1	7.1305	6.3522	6.466	7.4367	6.6077	6.5155	6.6159
5.9197	7.3176	7.5638	7.0153	6.9282	7.1281	7.7779	6.626
6.3317	6.9605	7.2829	7.3841	7.404	7.3876	6.3033	6.6174
7.139	6.75	5.812	7.2233	7.2266	6.4536	7.2613	6.8122
7.2142	7.0076	7.4367	6.635	7.9159	6.9064	6.445	6.94
7.3876	7.5361	7.1362	7.2701	6.8773	6.7443	6.2441	7.0939
6.8649	5.8259	8.0928	7.793	6.4739	7.064	6.9849	6.6002
8.306	6.6983	6.4818	7.5451	6.4924	7.0106	6.995	6.8674
7.0514	7.2631	6.2292	7.2551	6.4211	7.185	6.5926	6.7367
6.7201	6.6237	7.0106	6.152	7.4203	7.3847	6.4332	6.6596
7.2346	7.3547	6.8916	6.2811	6.1121	6.4886	7.2233	7.1442
7.1694	7.2482						
ATP5D	5.4525	5.3437	5.8959	3.2328	5.5887	4.347	4.2622
5.9137	4.4464	4.4828	5.323	3.5598	5.1303	3.464	5.2977
4.5967	5.6791	2.449	4.5741	6.2103	5.6709	5.3898	5.103
4.9302	4.0033	4.0656	4.6983	5.1911	5.0526	5.2359	5.9433
5.8466	5.3053	5.5629	4.3645	3.578	5.4118	6.0279	6.615
3.9373	6.5485	5.0075	4.9404	4.9479	3.1426	3.7467	3.6794
4.5321	5.8892	2.7309	4.5141	3.4378	3.6032	5.2214	5.7649
6.9937	4.5899	6.0414	6.5693	5.575	6.2843	5.3519	5.3353
4.957	5.1558	5.1972	4.5686	5.1871	5.2487	5.3895	6.3896
4.7767	5.6813	5.0965	3.4159	4.4241	6.5479	3.8789	5.1168
6.1589	6.0476	5.2233	4.7952	4.4654	5.5985	5.6375	5.3866
6.0649	5.9301						
ATP5E	9.1775	10.1008	10.0822	9.2636	9.6592	9.803	10.72
10.1674	10.2895	10.5925	9.5683	9.1304	9.4743	10.2895	9.5285
10.4311	8.9772	9.881	9.9993	9.3295	9.5683	10.2348	10.3704
9.5501	10.9434	9.5501	9.5834	9.8386	9.5683	9.7812	11.1638
9.4013	9.9013	9.2636	10.0232	10.5253	9.0087	9.5285	9.4013
10.4311	9.1608	9.512	9.803	9.1457	10.0624	9.1457	9.8605
9.7812	9.7454	9.1144	9.0847	9.5999	9.695	9.4276	10.2335
9.7512	8.7216	8.4212	10.3891	8.1098	10.5951	9.1921	9.5069
9.1803	9.0693	8.6891	9.6555	9.203	9.9159	9.3014	10.3891
9.9938	10.543	9.5813	10.4498	10.3891	9.5069	10.0824	9.3689
9.8714	9.6555	9.7512	9.2357	10.0506	10.1411	10.1557	9.3468
8.8707	9.1014						
ATP5F1	7.4587	7.5189	7.403	7.3001	7.0611	7.0466	6.8624
6.7055	6.8047	7.0433	6.9398	7.0055	6.3789	8.7254	6.5711
6.989	7.2141	6.5892	7.8898	7.4318	7.1838	6.6964	6.1983

7.0796	6.542	6.5125	6.8385	6.8219	6.1429	7.3453	6.4158
7.5549	7.6731	7.0083	7.1203	7.3001	6.4881	6.8336	6.9212
7.6572	6.6766	6.8266	6.49	6.6442	6.1939	7.0673	7.1603
7.5634	6.0092	7.1401	6.6832	6.8019	7.2039	6.9824	6.9721
7.1245	6.4592	6.9041	6.6701	6.7797	6.6275	6.8232	6.6973
7.3548	6.9172	5.9514	6.9705	6.4681	5.8157	6.3782	6.4999
6.9755	7.4334	6.731	6.2201	6.9635	7.5145	6.3608	7.0545
6.5339	7.5096	6.5325	6.3358	5.4153	6.478	7.2907	6.6442
6.8266	6.8701						
ATP5G1	7.9287	8.1912	8.1845	7.7562	7.5498	7.176	7.738
7.8649	6.8794	7.6977	8.0281	7.4698	7.7207	7.5907	6.7727
7.1004	7.9342	7.3014	8.0035	8.3623	7.0641	6.7685	7.0362
8.0978	7.1735	6.3008	6.3351	7.7991	7.4487	7.642	6.8426
7.1191	7.5114	7.3279	7.6102	7.5332	8.4751	6.9794	7.2399
7.9182	7.0282	7.1982	7.1272	7.1243	7.9287	6.8637	8.2677
7.8085	6.5702	7.7749	8.0721	6.8514	6.967	7.0719	7.6437
7.9361	6.8728	7.3131	7.956	7.2387	7.4126	7.5839	7.2713
7.3732	7.2547	6.0578	8.1376	6.4374	7.4344	7.4186	7.3183
7.2893	8.5293	7.0046	7.0992	6.488	7.8983	7.441	7.3519
7.8897	7.9457	6.8183	6.6399	6.7922	7.2601	7.166	6.4357
7.0138	6.8211						
ATP5G2	8.8181	8.6607	8.3305	7.3537	8.1194	7.9482	6.8583
7.8969	8.2515	8.3498	8.2778	7.6518	7.6202	7.4825	7.1923
7.9593	8.6268	8.1272	8.166	8.2354	8.0846	7.7717	7.4128
7.9184	7.9767	7.12	8.378	8.2444	7.8523	8.3498	8.5322
7.9246	7.9422	7.5101	7.6905	7.5301	8.2778	8.2778	8.3498
7.9422	8.2778	7.9246	6.6414	8.2778	7.6167	7.7717	7.8322
8.5726	8.1589	7.3472	7.7321	7.1781	8.4532	7.7784	8.2778
7.4431	7.5279	8.3168	7.5563	7.6917	8.1412	8.4566	8.9371
7.7288	8.5348	7.2259	8.5558	7.6129	7.2046	7.7829	7.8749
7.3804	8.0433	7.3967	7.1896	7.1394	7.999	7.4431	8.4105
8.2249	7.9862	7.8163	7.8198	7.7104	7.8163	8.1111	8.166
8.2606	8.3305						
ATP5G3	6.9857	6.4484	7.0878	6.1969	5.867	5.9941	6.4852
6.4872	7.0829	6.5231	6.5453	6.4703	5.9217	6.3429	6.2694
6.0997	5.9725	6.1969	6.7742	7.0245	6.6799	5.9189	5.9573
6.954	5.3294	5.036	6.1634	6.4703	5.9109	7.5975	5.2732
6.0465	6.9519	6.4392	6.2588	6.7682	5.9975	6.3186	6.2215
6.8977	6.5111	6.3058	6.4782	5.8044	5.9159	6.2215	6.8912
7.0689	5.9149	5.5344	6.3559	5.7967	6.5184	5.5939	6.5981
6.6698	6.0299	6.6716	6.9899	6.6797	6.7003	6.7348	6.2585
6.592	6.8902	5.3265	6.6191	5.7212	6.6556	6.3682	6.163
6.3162	7.088	6.0059	5.5125	7.5315	7.0137	6.6014	6.5307
7.0188	6.9793	5.8778	5.7056	5.8778	5.6947	6.3714	5.8051
6.4349	6.4319						
ATP5H	8.0429	7.8624	7.7318	7.8385	7.3568	7.4619	7.8437
7.5023	7.8756	7.3201	7.96	7.8756	8.1111	7.7236	7.3
7.4959	8.0593	7.7072	7.6006	9.041	8.7304	6.6736	7.1615
7.2914	7.3173	8.1691	7.9119	8.4299	7.3951	8.2396	7.8845
7.7363	7.2128	7.6006	7.4079	7.8715	8.2953	7.2735	7.2888

7.6328	7.6225	7.7873	7.2156	7.2078	7.8001	8.1111	8.3851
7.6566	7.0909	8.332	7.9702	7.4262	8.0926	7.8266	9.0547
7.7664	7.4485	7.4188	8.019	7.5782	7.6657	7.1146	7.8373
7.5359	8.4097	7.638	8.288	7.8913	7.2349	7.7466	7.5972
7.5835	8.3899	7.6657	7.6245	8.3527	8.3043	7.9141	7.6519
7.026	7.7415	7.2286	7.3401	8.1383	7.9456	8.2577	7.728
7.7406	7.9811						
ATP5I	8.2911	8.4408	8.6291	7.7938	8.4331	7.7987	7.1801
8.3523	7.8624	7.5398	7.6339	7.7617	6.9481	7.9538	7.2502
7.2502	7.1801	7.0396	7.462	7.9693	8.2104	8.3986	7.6892
7.5008	7.4874	7.4971	8.0946	8.1901	6.6659	8.1177	7.7019
8.5901	7.3861	7.7987	7.7756	7.6847	8.1115	8.2041	8.2911
8.1177	7.6776	8.2104	7.6699	7.8258	7.6184	7.7498	7.4941
8.4789	7.325	8.0658	7.1271	7.4805	7.7498	7.8953	7.6476
8.1757	7.4744	7.4823	7.9245	7.6871	7.8191	8.0158	8.3204
7.8803	8.2277	7.7629	7.8696	7.8538	7.7834	7.2961	7.7396
7.2961	8.0858	7.8191	7.7729	9.692	8.0984	8.0496	7.0582
8.3737	8.2507	7.8239	7.9116	8.7326	7.8084	8.2581	8.1231
8.6005	8.4789						
ATP5J	7.0561	7.8279	7.2817	8.1701	7.3269	7.1092	8.2886
7.4178	8.2809	7.0673	7.2386	7.4218	6.5455	8.1635	6.8318
7.3977	7.4218	7.0501	8.0571	7.4178	7.1417	7.6985	7.4886
8.0937	7.823	6.5614	7.4253	7.2457	7.4098	8.0571	7.0613
7.0116	6.9889	6.6139	6.961	7.4923	6.937	6.4686	6.9107
7.675	7.6217	7.7137	6.7887	7.1924	5.9833	7.3193	7.9551
7.1562	5.9607	7.4556	7.384	7.7393	7.4326	6.9394	7.6948
8.0385	6.1051	6.4919	8.2498	6.5006	6.9411	6.9961	6.7602
7.4616	7.1685	6.5947	7.5087	7.0141	7.3597	7.2835	6.8753
6.3617	7.4928	6.7634	7.0402	7.7195	7.3204	7.4107	7.1778
7.9232	7.6058	7.3182	6.7498	7.0954	6.7976	7.9039	7.0534
7.2051	7.3389						
ATP5J2	8.4273	9.7136	8.4577	9.0428	8.3571	10.4125	9.5087
9.3974	8.4998	8.0051	8.7699	7.845	8.4459	9.0428	8.4998
8.55	7.9714	9.884	9.8669	8.3389	8.2325	8.6973	9.1441
9.3502	9.3974	7.8332	7.5112	8.1674	9.2721	9.3974	8.3872
8.6753	8.0596	7.3515	9.2151	8.6856	8.1749	8.0524	8.7946
8.6632	8.7946	8.3872	8.5857	8.1674	8.4359	8.7699	8.1588
8.9996	7.8179	7.5461	8.5206	8.7822	7.5242	7.3207	9.09
8.9307	6.9896	8.9307	9.5951	9.0334	8.5528	8.7172	9.1765
8.8497	8.8585	7.6601	8.9307	7.8758	8.3296	8.6723	8.6633
8.5684	9.6815	7.8603	8.3093	8.463	8.512	8.9635	8.3161
8.8497	8.6993	8.242	6.0541	8.6447	8.7524	8.0532	7.9714
8.1018	8.0307						
ATP5L	6.8134	6.8643	7.0046	6.2873	6.7753	7.2493	6.4588
6.0224	6.881	5.5458	6.1613	6.012	6.4635	6.1625	5.8693
6.5935	6.9041	5.7391	6.2806	6.6446	6.0313	6.4906	6.5783
6.3818	5.7302	6.1714	6.4104	5.7725	6.4876	6.8024	6.6266
5.7464	6.3291	6.0608	6.3517	5.9718	5.5227	6.1963	6.3616
6.1526	6.5533	6.6004	6.1749	6.3979	6.1835	5.691	5.3805
6.8872	6.4332	6.4988	6.1453	6.1453	5.1597	6.4383	6.7381

7.0718	6.5987	6.3913	6.3057	6.2983	6.127	6.2438	6.6586
6.6314	5.4095	6.068	6.9931	6.5969	6.0254	6.1175	5.7061
6.2029	6.9829	6.1483	6.6676	7.4987	6.1323	5.9375	6.7485
6.5215	6.2553	6.5031	5.7964	6.073	6.1581	5.1972	6.6897
6.9018	6.8535						
ATP5L2	0.2162	0.1534	0.4794	0.1595	0.205	0	0.107
0.3179	0.7329	0.1938	0.1897	0.1539	0.1049	0.5231	0
0.2939	0.569	0.1718	0.7727	0.7382	0	0.201	0.0793
0.4486	0.1851	0.1134	0.2056	0.4248	0.2397	0.1212	0
0.0732	0.1409	0	0.3027	0.1511	0	0	0.1079
0.3063	0.1795	0.1701	0.2418	0.2237	0.2079	0.1224	0.2546
0.1497	0.2519	0	0.1181	0.393	0	0.5123	0.4195
0.6875	0.4535	0.1801	0.5921	0.2654	0.9465	0.023	0.4564
0.2585	0.1013	0.0245	0.4376	0.1521	0.0608	0.4503	0.0647
0.2675	0.4462	0.0106	0.1314	0.445	0.2164	0.0286	0.1129
0.2346	0.4594	0.0442	0.1935	0.1827	0.1898	0.4535	0
0.1692	0.1866						
ATP5O	7.9239	8.0714	7.7248	7.7392	6.9522	7.7014	8.3408
7.9593	9.1039	7.5577	7.7997	7.4356	7.1191	8.845	7.3265
7.525	8.3969	7.4588	8.2168	8.1739	7.6479	7.5998	7.1381
8.2086	8.2321	7.354	7.425	7.6295	7.4105	8.3408	8.0714
7.4037	7.7056	7.9353	7.4932	8.0408	7.5336	7.0551	7.7952
8.0657	7.8297	7.7103	7.5093	7.8965	6.7426	7.9593	8.1059
8.0086	6.5824	8.0223	8.034	7.4142	8.2086	7.5798	8.832
8.3993	7.5288	7.3014	8.2667	7.3301	7.386	7.1514	7.4797
7.9334	7.731	6.9059	7.6984	7.2853	7.848	7.5575	7.1187
7.367	8.0971	7.0661	7.1772	8.3012	7.7236	7.2573	7.82
7.7018	8.1107	7.5104	7.4582	7.7467	7.5604	8.2105	7.5294
7.7484	7.8412						
ATP5S	3.0154	2.3217	2.3171	2.6	2.2913	3.1475	2.4775
2.9294	2.338	2.6342	1.9384	2.9515	1.6594	2.9667	2.7183
2.9637	3.1077	2.7688	3.0996	2.3367	2.2126	1.9752	3.0675
2.3763	1.9449	2.1254	2.8047	2.0171	2.8077	1.8684	2.2518
3.3107	2.9267	2.3079	2.2126	2.8633	2.6577	2.9395	2.6976
3.0461	2.5628	2.0782	2.1047	3.3093	2.7248	2.7248	2.8247
2.545	2.6	3.6129	2.2698	2.8925	2.638	2.7598	2.4474
2.8551	2.1525	2.3361	2.3391	1.8726	2.4505	2.7815	2.2113
2.1999	2.5281	2.2754	2.6564	1.9495	1.4683	2.4558	2.4909
2.1173	2.4571	2.3843	2.6822	2.4283	2.4402	2.2844	3.0342
2.4765	3.406	2.7525	2.9323	2.2176	2.5943	3.0143	2.3834
2.4554	2.4982						
ATP5SL	4.2722	4.5575	4.412	3.6122	4.4244	3.8435	4.0243
4.5818	4.2891	3.8793	4.7696	4.2919	4.6675	3.9051	3.9692
3.2577	4.4462	3.2999	4.1024	4.3053	4.5735	3.9922	4.1465
3.9535	4.1676	4.4631	4.8379	4.2464	4.1334	4.479	5.472
4.2305	4.719	3.9122	3.6103	3.7241	4.1582	4.5054	4.5855
3.8061	4.2432	3.9017	4.2179	3.7048	4.7393	4.4033	3.8379
4.0422	4.2179	5.025	4.2294	3.8525	4.1482	4.0937	4.7587
4.5818	4.0351	4.868	4.1943	4.5164	4.6705	4.6918	4.6838
4.2833	4.2137	3.9013	3.1358	4.5266	4.8088	4.0937	4.3952

4.5851	4.2514	4.5812	3.4226	4.5126	3.8292	3.3635	4.3936
4.2215	4.0124	4.1053	4.2183	4.2894	5.1156	4.4485	4.5666
4.7792	4.8639						
ATP6AP1	6.596	6.5653	6.9519	5.6623	5.9672	6.5249	6.9753
6.0274	6.3616	7.2963	6.56	6.4659	6.2979	5.0288	6.7186
7.7124	5.1384	7.2126	6.0072	6.8236	6.5854	6.3906	7.1048
6.4705	6.2051	6.6107	6.5942	6.6034	6.5042	6.7552	6.8045
7.0599	6.558	7.6754	7.1144	6.4894	6.1506	7.7124	5.6871
5.9777	6.4213	5.9073	5.7798	6.1533	6.3662	5.3719	6.3349
6.4476	6.5284	5.784	6.9637	6.3586	5.9801	5.823	6.6259
7.3481	4.9606	6.4119	6.0832	6.3554	5.6352	5.6076	6.8563
6.198	5.9638	7.4727	6.7164	6.6058	6.7376	6.5892	6.8
5.7388	6.9359	6.2908	7.3269	6.8874	6.9293	7.4361	6.7449
6.1112	7.4255	6.6539	6.5756	7.01	5.7334	5.7823	7.4828
6.7576	7.1713						
ATP6AP1L	1.0173	0.4787	0.5529	0.5161	0.2643	0.535	0.9371
0.9486	0.4107	1.0625	1.5431	0.4082	1.6666	0.725	0.4415
0.5475	1.6042	0.9658	1.2063	1.4179	0.953	0.3832	0.2969
0.7749	0.258	1.2707	0.9353	0.5211	0.3854	0.4866	2.1183
0.8389	1.2245	1.9624	1.0504	0.5563	1.3856	0.6124	0.55
0.6261	0.7587	0.2803	0.946	0.5904	1.4823	0.4345	0.6319
0.3604	0.8904	1.5314	0.5001	0.3193	0.7356	1.2068	0.7818
0.6573	0.8405	0.5385	1.6122	0.499	0.5317	0.9647	0.3094
1.1316	0.7503	0.9539	1.2341	0.5948	1.0269	0.418	0.6726
1.0018	0.4737	0.6956	0.7572	0.8889	0.8671	0.3966	0.437
1.0426	0.6623	0.5596	0.7607	0.8365	1.1817	0.7484	0.7303
0.2391	1.0663						
ATP6AP2	6.1962	6.2107	5.8824	6.1109	6.2533	6.5818	7.564
5.8452	7.4018	6.7122	6.2383	7.0392	6.7762	6.2951	6.8985
7.3295	5.1482	6.8332	6.0915	6.1702	6.3819	6.5532	7.3976
6.6813	6.2471	7.1719	7.2314	5.9054	6.7446	6.2383	6.9283
6.5571	6.5072	7.7179	7.8104	6.8239	6.0611	6.4059	5.7166
6.8959	6.7465	6.7207	6.3316	6.7512	7.1488	6.6443	6.0762
6.343	6.0212	6.208	7.0008	7.555	6.2137	6.4548	6.9651
5.5461	6.4979	5.8281	5.6471	6.029	6.0472	7.3169	6.6594
6.8624	5.5213	7.6482	6.6865	6.6715	6.0054	6.0722	6.4521
6.222	6.5442	7.2263	7.492	6.543	6.1764	7.4241	6.9531
6.0083	6.2666	7.2841	6.8639	7.1763	5.9355	6.1229	6.9698
6.5421	6.5877						
ATP6V0A1	4.8884	5.3686	4.7114	3.8673	3.773	5.3665	4.6007
4.4176	5.1286	5.3003	4.9888	4.0421	4.8679	4.3187	4.4061
5.6798	3.9558	5.8807	5.0173	4.5049	4.4441	3.5428	4.6026
4.2438	3.9634	4.7453	3.3935	5.5015	4.4371	4.5799	4.8408
4.3268	4.7209	5.6139	5.1911	4.9497	4.4376	5.9906	3.6754
4.4584	4.5604	3.3769	3.8663	4.7436	4.1919	3.2079	4.8737
5.1155	4.5172	4.1983	5.1257	4.0111	4.8532	4.344	3.8117
4.6505	3.7362	3.8068	4.6679	3.8452	4.1493	4.3153	4.0282
4.9624	4.5689	5.2106	4.8354	5.4981	5.7918	3.7597	4.6904
4.9386	6.0969	4.533	4.5904	6.6009	5.0871	4.4742	5.7853
4.1737	4.8597	4.4667	5.9968	4.5897	4.2672	4.1387	6.6424

5.9567	6.5707							
ATP6V0A2	3.1404	2.8362	3.6545	3.3697	2.7038	3.0601	3.3937	
3.0529	2.8277	3.2947	2.5705	2.3095	3.0251	3.9032	2.9028	
2.5551	2.7651	3.8119	3.952	3.409	3.0171	2.8975	2.4486	
3.523	2.9163	2.3875	2.4575	2.6826	2.7554	2.4694	2.6394	
3.2468	2.3974	2.328	2.5632	3.2482	3.3575	2.6528	2.6217	
2.5898	2.7913	1.5951	1.56	2.9044	2.4041	2.705	3.6292	
2.7639	2.3183	3.1219	3.2449	2.8892	3.2929	2.9763	2.7863	
3.5796	1.9374	3.702	3.2401	3.3999	2.5493	3.2249	3.3622	
2.8415	3.2472	2.8057	3.0858	2.664	2.5628	2.7194	2.3978	
2.714	3.3453	1.9555	2.2174	3.6397	3.4593	2.6171	2.225	
3.0436	2.8807	2.4188	2.7609	2.9449	2.3436	2.9922	2.5701	
3.0054	2.6386							
ATP6V0A4	1.8333	1.8759	0.6756	3.1215	2.7617	3.1962	0.0366	
0.9695	1.8723	1.5448	1.7029	1.8742	0	0.8776	0.1483	
2.0492	0.7561	1.8138	2.1116	1.787	1.5472	0.1744	1.7084	
2.7482	0.0258	0.2378	0.0229	1.2525	0.6045	4.6645	0	
3.5457	0.2172	1.496	2.474	3.1843	1.5938	0.9212	1.5023	
1.9149	1.3688	0	0.0734	2.0616	0.02	0.0506	1.4107	
0.4964	0.1653	0	0.5692	0.2343	2.0286	0.7001	0.3551	
0.8686	0.4643	4.3292	1.5242	3.4171	0.9371	2.9466	3.0006	0
	0.4495	0.1044	1.8942	0.0275	0.2947	0.6395	1.3235	
0.4596	2.7496	0.5885	1.4273	0.1849	0.7211	3.5305	1.4278	
1.5612	1.5114	0.9419	0.5245	1.4677	0.0502	2.5952	1.3826	
0.596	0.8006							
ATP6V0B	6.5942	8.1662	7.9836	6.8613	7.5119	7.3259	7.6701	
7.598	6.9426	7.3415	7.7408	6.9196	6.8841	6.7088	7.1933	
7.3717	6.4549	6.4057	7.3358	7.7272	6.736	7.252	6.8197	
8.0649	6.839	6.7833	5.5498	7.5833	7.2291	7.6402	6.9909	
7.6443	7.7722	7.497	8.029	7.1295	7.1562	7.5051	6.8007	
6.1066	6.7917	6.5353	6.6198	6.4734	6.3265	6.2216	6.5297	
7.0661	7.0979	5.1293	7.4068	6.2033	6.8155	6.8736	6.8591	
7.6174	5.4657	7.6787	7.7948	7.8351	6.5701	8.2657	6.4622	
7.0737	6.6506	6.8943	7.683	7.0585	7.9443	6.3699	6.3295	
7.272	7.5949	6.984	6.0051	7.2609	7.9692	6.5984	6.8052	
7.2465	6.5918	6.2887	7.1878	6.2993	6.7129	7.1467	7.3068	
7.0612	7.4654							
ATP6V0C	8.142	8.3014	8.6261	6.879	7.284	7.3949	7.4902	
8.3439	7.0231	7.4747	7.4951	6.6624	7.4352	6.8926	7.5044	
7.8888	6.8015	7.832	7.5906	7.7946	8.5017	7.0546	7.3648	
7.7649	6.9333	6.5957	6.3321	7.9734	7.1031	6.9276	7.7479	
7.6717	7.6212	8.0547	7.6403	7.2395	7.8006	8.7192	7.2781	
6.3611	7.6924	7.1437	6.8246	7.0936	6.6754	6.3833	7.3365	
7.1505	7.355	5.989	7.3426	6.2715	7.0037	7.4247	6.8331	
7.8163	6.9975	7.5223	8.9199	7.8279	7.5582	7.998	7.487	
7.5731	7.5656	7.904	8.7629	8.5133	8.5579	7.0396	7.895	
7.5995	8.5579	7.1263	7.4382	9.1792	8.0847	7.6906	7.8475	
7.7631	7.9749	7.3097	8.9354	7.649	6.8825	7.7959	8.5262	
8.5318	8.3689							
ATP6V0D1	5.6422	6.4181	6.6877	5.0746	5.5894	5.8765	4.2466	

6.0549	5.5977	5.949	5.1235	4.9328	5.7899	5.3806	5.3024
6.7719	4.9968	5.3087	4.9518	7.0671	6.3674	5.1046	5.6977
5.9672	5.367	5.9945	3.7202	6.463	5.1556	5.6271	6.2169
6.2985	5.5612	5.5186	6.4407	5.531	5.7394	6.9731	5.2894
4.643	5.982	5.9015	5.3429	6.0871	5.3843	5.0193	5.5576
5.7089	4.7752	4.9233	5.7526	4.7327	5.3422	4.3439	5.8552
5.4521	5.3889	4.4658	6.1749	4.647	5.4814	5.9427	5.3098
5.9509	5.8578	5.6489	6.4047	7.06	5.8748	5.3037	6.0064
5.6383	6.6712	4.8047	5.8162	6.4248	6.1479	6.2563	5.8935
5.708	5.6177	5.2025	6.0559	5.3315	5.1275	5.8973	6.7758
6.7857	6.8254						
ATP6V0D2	0.2753	2.4592	0.7751	1.3162	0.0186	0.8662	3.5705
0.1816	1.0639	4.3143	3.4849	2.8163	0.2373	1.3023	0.2124
0.9471	0.045	4.8426	4.4753	1.7517	2.0199	0.0187	0.0616
0.4791	0.0088	0.5395	0	0.273	0.0537	2.4436	0.0148
4.1946	2.785	3.1682	0.1258	5.3335	0.6566	0.032	0.0468
0.034	0.1997	0.0919	0.0561	0.4867	0.3884	2.1961	1.7128
0.1662	0.1607	0.3189	1.8733	0	2.0902	0.3105	0
0.1994	0.2786	0.0332	5.5391	0	0.461	4.7606	0.0243
0.2154	0	0.801	0.0474	2.1846	1.0642	0	1.7421
1.7075	1.5744	0.0344	0.0763	3.4933	3.0158	0.0894	2.8554
0.5232	3.1501	0.0907	0.3726	0	0.1804	1.2151	0.5856
0.6892	0.0758						
ATP6V0E1	6.9101	7.4156	8.1101	7.6201	6.9705	8.0137	9.4117
7.8043	8.0137	7.4998	7.2631	7.2944	8.117	7.6717	8.5768
8.3048	7.2539	7.2386	8.1394	8.158	8.0915	6.7413	7.9279
8.1787	7.2721	7.9121	7.1768	7.9436	7.8969	6.7025	7.7212
8.6073	8.1101	8.1245	8.7927	8.0362	7.5339	7.9557	6.8694
7.9337	7.3099	7.316	7.2093	7.5733	7.469	7.5583	7.5076
7.5152	8.0362	6.8069	7.7212	7.5116	7.7254	6.8808	6.985
7.6503	7.4604	6.8266	7.4734	6.9565	7.3963	7.6204	7.6166
7.8779	7.3009	8.2487	7.8287	8.6263	7.5274	7.3183	7.5519
7.6277	8.7233	7.0636	7.5106	8.3887	7.8913	8.6931	7.4369
7.9531	7.4734	6.9695	8.9714	7.8022	7.3124	7.4842	7.9669
7.7481	7.8134						
ATP6V0E2	4.2747	6.5301	5.2154	4.4917	3.9206	5.7998	4.5342
5.4018	4.7821	3.872	4.1697	4.2269	3.9056	3.7309	4.5094
7.4772	2.5663	5.5976	5.0959	6.3875	5.276	4.3315	4.6814
5.1986	3.5035	2.305	3.5662	4.9906	4.4044	6.1562	6.2423
5.0829	3.5908	4.4332	5.0263	4.1334	3.974	5.4955	4.4395
2.4261	5.3456	3.2465	3.2217	4.6271	3.8969	1.0632	3.5761
5.5142	4.4174	1.3765	4.4143	3.5285	4.4889	2.8383	5.2161
4.4675	0.4708	5.1218	5.6886	4.828	2.4703	5.8648	5.7349
5.2341	3.6914	4.174	4.5326	5.131	5.5283	4.286	4.4675
4.4604	5.6792	4.1497	4.3132	6.5414	4.3535	5.8769	4.7632
5.7014	4.8239	3.6951	5.0967	4.8549	3.9687	5.4287	4.4985
4.5098	4.9643						
ATP6V1A	4.8669	5.1244	5.1628	5.1316	5.0946	5.5484	5.5305
4.8287	5.914	5.1988	5.18	5.1103	4.1285	5.4361	4.732
5.5988	4.0629	5.7256	5.4762	4.7159	5.6704	4.483	4.9257

4.5417	4.2583	4.4649	5.7965	5.0616	4.8266	5.6389	5.0839
5.1488	4.5828	5.9669	5.577	5.6314	4.3215	5.9219	4.4382
5.2083	4.9825	5.3045	4.564	4.666	5.6268	4.9361	5.2153
5.7528	4.2188	4.7392	5.3525	5.139	5.5033	4.3563	5.1874
5.4273	4.4793	4.1232	4.8973	3.992	5.1904	4.9983	4.3642
5.4021	5.2023	5.7718	6.0834	5.2658	5.3815	3.9552	4.9142
5.4136	5.5996	5.2606	4.6718	5.8674	5.3943	5.6068	5.5082
5.4576	5.3497	5.1636	5.434	5.7466	4.5498	5.247	6.2515
5.4434	6.0954						
ATP6V1B1	1.9649	0.6455	0.9545	0.3692	0.4142	0.5062	0.5272
1.4537	1.5451	0.5804	0.7514	0.3292	0.8617	0.6758	0.9316
0.9064	0.9627	1.0787	0.5528	0.8865	0.9392	0.7697	0.6927
1.3206	0.837	0.4138	0.5432	0.5833	0.9857	1.3537	0.8423
0.7044	0.951	0.8157	1.5023	0.3137	0.8522	0.7682	0.6348
1.0755	1.293	0.2861	1.0689	0.5869	0.7242	0.0646	0.5178
0.7135	1.1815	0.2878	0.7111	0.387	0.7472	0.6057	0.5314
0.9072	0.8117	0.673	0.1875	0.7381	0.781	0.461	0.3671
0.8179	0.9282	0.5763	1.7161	0.7694	0.5324	1.2037	0.4367
0.216	0.7307	0.5567	1.4298	0.6353	0.7588	0.9398	1.2851
0.8052	0.6524	0.178	0.8609	1.0967	0.4982	1.3721	0.7362
0.7671	0.7362						
ATP6V1B2	7.0551	6.3185	5.5352	6.5715	4.4987	6.5435	7.7112
6.4776	7.0774	7.2422	6.1468	6.4419	6.3264	8.0251	5.6302
6.5598	5.1374	7.5606	6.998	5.3894	7.0612	6.0887	6.1156
6.9262	6.0408	5.2108	5.9193	5.8761	7.0119	5.9576	5.0745
7.0831	7.0522	7.3479	7.2939	7.0309	6.5681	7.0774	6.3558
6.9473	6.251	4.8018	5.3298	6.4531	6.4941	5.6811	6.9473
5.9202	5.7795	6.7892	6.8197	6.3006	7.8286	6.4046	4.616
5.971	5.1522	5.0026	7.0033	5.4392	6.5323	6.7332	6.0643
6.8748	7.439	5.4613	7.1104	7.4832	5.6599	5.2445	7.2995
7.2501	7.2782	6.2267	6.3405	5.9927	7.0755	6.4903	6.1117
6.3697	5.9204	6.6776	6.5846	7.158	5.9667	7.5404	7.6785
7.0663	7.0831						
ATP6V1C1	4.816	5.1499	5.2928	5.0165	3.8745	5.3135	4.9695
4.7713	4.426	5.9403	4.8835	4.3335	4.0368	5.0377	5.2905
4.75	4.4571	6.1422	5.6133	4.1167	5.322	3.633	4.1976
4.5333	3.9923	4.3458	2.8997	4.8944	4.2116	4.5611	4.3445
5.0821	5.4674	4.4494	5.1122	6.3406	4.7312	6.5412	3.703
4.6681	4.3138	4.212	4.0623	4.5944	4.1214	4.5401	5.8614
4.7921	3.532	4.7895	5.2298	4.1568	5.9726	4.4905	4.2237
4.6856	4.2398	4.7931	6.0036	4.8969	4.1929	4.952	4.2475
3.9934	6.594	3.8342	5.4607	5.335	4.4306	4.1377	4.9803
4.4701	5.5819	3.9872	3.7896	5.8354	5.2316	4.5942	5.3941
5.2918	5.3055	4.0826	5.0068	4.3178	3.4006	5.4499	5.1437
5.1592	5.2776						
ATP6V1C2	0.8346	1.1721	0.7737	0.2185	0.1194	0.2672	0.3345
0.6356	0.6666	0.5712	0.6803	0.1485	0.206	0.1044	0.3422
0.7085	0.6131	2.6774	1.1288	0.4569	0.3612	0.1959	0.4529
0.5821	0.2928	0.1615	1.5953	0.676	0.735	1.2163	0.475
0.1636	0.4791	0.4633	0.068	0.557	1.2929	0.4791	0.4505

0.2434	0.7128	0.2047	0.3134	0.5972	0.3443	0.4746	0.8332
3.0191	0.2496	0.4055	0.8952	0.4209	0.865	0.2447	0.4953
0.7177	0.2743	0.3668	0.5723	0.4422	0.5032	0.3535	0.6168
0.5863	0.5543	0.2737	0.5785	0.2899	2.346	0.2976	0.325
0.6909	0.2589	0.2916	0.3228	2.4017	0.5073	0.2143	0.5683
0.7532	0.7844	0.4938	2.8445	0.3688	0.1227	0.4796	0.2804
0.2723	0.403						
ATP6V1D	5.8431	5.9379	5.478	5.637	4.9119	5.6345	5.9749
5.2984	5.5886	5.5955	5.3291	5.6245	4.952	5.4809	5.2883
5.7459	5.5284	6.1702	5.9863	5.5026	5.465	4.8926	5.7996
5.198	5.0672	5.6859	5.2711	5.5509	5.4412	5.8743	6.7836
4.7907	5.5071	6.378	5.0142	6.1982	5.4189	5.7633	4.6376
5.4724	5.3025	5.7093	4.8291	5.1148	5.5583	5.4943	5.5509
5.2735	4.4904	6.012	4.8583	5.8147	5.487	4.8583	5.5564
4.9807	5.4923	4.4719	5.2098	4.5747	5.6479	5.5176	5.3753
4.8807	5.1609	5.8836	5.9599	6.2068	4.8773	4.7988	5.7178
5.1991	5.5059	5.6032	5.9312	5.8413	5.4628	5.4711	6.4799
5.424	6.3913	5.1217	5.5009	5.099	5.2581	5.5564	6.3541
5.8578	6.2145						
ATP6V1E1	7.0364	7.5092	7.4419	6.76	6.1804	6.7358	7.6325
7.8317	7.2331	7.7152	7.0881	6.9696	6.6967	7.2836	7.3546
7.2042	5.2891	7.2476	7.5049	7.3777	6.8814	6.717	6.8534
7.5871	6.5575	6.6439	6.4695	7.5914	6.8237	7.3252	5.3133
6.2527	7.7969	7.5049	7.4453	7.567	7.1878	7.4352	7.1655
6.8661	6.612	6.86	6.2502	6.8128	6.4935	6.6423	7.08
7.0853	6.608	5.7371	6.3471	7.0029	7.848	7.1676	5.8509
6.2444	6.9216	6.298	6.7731	6.3579	6.4157	7.1564	6.5178
7.3859	6.4713	6.9533	8.0447	7.7016	7.3195	6.0997	6.5439
7.0055	7.2959	6.2238	7.361	7.7095	7.4302	6.785	6.8942
6.7331	7.559	7.1379	7.3105	6.703	6.0256	8.0028	7.6155
7.3252	7.4683						
ATP6V1E2	1.8737	1.7706	0.4487	0.8064	0.6646	1.0568	0.2559
0.5183	1.1795	1.1966	0.5541	0.4077	0.8496	0.6925	0.7025
0.6432	1.8187	1.159	1.0957	0.0874	0.4357	0.4047	0.6544
1.1152	0.2164	0.7037	0.3	1.0855	0.5932	2.4429	1.2386
1.2861	1.8771	0.9795	0.8307	1.0809	1.9462	1.1253	0.6871
1.0553	1.198	0.1106	1.6496	0.6907	0.693	0.9829	0.849
2.9433	0.3818	1.4013	0.7061	0.7756	1.13	1.0184	0.9585
1.051	1.1496	0.8449	0.7455	0.8504	0.6248	1.1627	1.057
1.105	1.9382	0.3375	2.8384	0.3595	0.7324	0.7034	0.4076
0.8647	0.3391	0.2641	0.5307	2.1474	0.9657	0.6272	2.2209
1.5474	1.9142	0.3875	1.3574	0.4386	0.7918	1.0634	0.8609
0.9678	0.744						
ATP6V1F	7.2533	8.0259	8.5812	6.8264	6.9289	8.054	7.5805
9.0158	7.1541	7.3714	7.8867	7.0119	6.847	7.3749	6.7806
9.082	5.957	8.675	7.7124	8.0653	8.2111	7.2276	7.3649
7.3515	7.0682	7.1425	7.1065	7.3714	7.7588	7.8965	8.1982
7.9754	7.3649	7.5561	7.3813	7.5947	7.4218	7.4883	7.4487
6.5434	7.5045	6.4959	6.3098	7.409	6.7722	6.5383	6.9244
7.4922	7.4522	5.9751	7.2219	6.3809	6.5553	7.3944	7.8189

7.3321	4.7182	7.0065	7.5957	6.946	6.8961	7.9329	7.8291	
7.8141	7.1616	7.0648	7.7085	7.8474	7.43	6.946	7.7819	
7.6789	8.65	7.1409	6.8312	8.1704	7.7316	7.5166	6.9779	
7.4681	7.2978	6.4965	8.4744	7.4789	6.9801	6.9721	7.5423	
7.611	7.4388							
ATP6V1G1	6.4661	6.7251	6.7952	6.2097	5.5038	6.6074	6.4744	
7.3532	5.5753	6.6445	7.1043	6.2726	6.7597	6.5484	6.3688	
6.7387	5.7774	6.9804	6.639	6.9916	7.0718	4.6596	5.1231	
6.0349	6.1757	5.5977	6.1659	7.1326	5.4803	6.5893	6.4615	
6.7765	6.0085	6.1783	5.9182	7.1555	6.7175	7.5696	6.8101	
5.7016	6.2111	5.6965	5.1814	6.4535	5.5298	6.0687	7.338	
5.5015	6.0234	5.5442	6.7406	5.9191	7.0441	6.289	6.216	
6.2104	5.7066	6.0893	7.5674	5.9504	5.9063	5.984	6.0204	
6.1408	7.3758	6.9542	6.7755	7.7057	7.1563	6.3683	5.7315	
6.0893	7.0432	5.5917	5.7872	5.9661	7.5638	5.9733	6.1624	
6.0405	6.083	5.5329	7.1271	7.0432	5.5382	6.7473	6.814	
6.8838	6.8729							
ATP6V1G2	0.5835	0.8611	0.4748	1.36	0.4689	0.4924	0.2542	
0.4606	0.2761	0.609	0.452	1.0704	0.3435	0.9288	0.5346	
0.395	0.8871	0.6863	0.3738	0.6339	0.6917	0.4524	1.068	
0.2177	0.364	0.3851	3.061	0.4116	0.2366	0.4241	0.2196	
0.205	0.3254	0.6652	0.1576	0.6626	0.4158	1.0876	0.4838	
0.2494	0.4124	0.7235	0.434	0.6152	0.1388	0.3751	0.1217	
0.5282	0.4878	2.4256	0.8195	0.5516	0.67	0.5584	0.607	
0.0695	0.7588	0.2735	0.0694	0.2694	0.3014	0.5977	0.7914	
0.3211	1.1255	1.0105	2.0395	1.3308	0	0.2841	0.7371	
0.4936	0.1662	1.2709	1.131	0.0597	0.6796	1.0166	0.236	
0.7528	0.7935	0.9902	1.0336	0.0994	0.5105	0.6435	0.8118	
1.6055	1.1594							
ATP6V1G3	0	0	0	0.071	0	0.7277	0	0
	0	0	0	0	0.156	0	0	0
	0	0	0	0	0	0	0	0
	0	0.2802	0	0	0	0	0	0
	0	0	0.0251	0	0	0	0	
0.2813	0	0	0	0	0	0	0	0
	0	0	0	0	0	0	0	0
	0.2462	0	0	0	0	0	0	0
	0	0	0	0	0	0	0	0
	0	0	0	0	0	0	0	0
	0	0.3829	0	0	0	0	0	0
	0							
ATP6V1H	5.3227	5.4662	5.589	5.4613	4.5042	5.1797	5.5412	
5.2898	4.7501	5.9536	5.2544	3.9187	4.7134	5.0515	4.9019	
5.1439	4.4139	6.261	5.3307	5.1261	4.9762	4.232	4.8998	
4.8186	4.6826	4.17	4.0919	5.5453	4.7824	5.274	4.0866	
5.4165	5.5436	5.5031	5.5772	5.9958	4.576	6.9098	4.6139	
4.8315	5.0074	4.743	5.4296	4.8484	4.4571	5.1486	6.1713	4.4
	4.5393	5.1155	5.1816	4.5951	5.4938	4.3924	4.3864	
5.4314	5.4063	4.5157	5.6692	4.2681	4.8294	4.9438	4.6601	
4.4571	6.6426	5.2253	6.5353	6.4268	4.8724	4.4384	4.7883	

4.5405	5.258	4.4164	5.0511	5.699	5.599	5.0745	5.823
5.7278	5.6578	4.4601	5.1523	4.609	4.2759	5.3413	6.3126
6.0065	6.1288						
ATP7A	2.7892	2.4411	3.0316	2.7123	2.1939	2.4716	3.4745
2.3409	3.3662	2.779	2.9154	3.3019	2.0435	3.2376	2.6057
3.0254	1.917	2.6291	3.2297	2.1308	2.9531	2.1296	2.7182
4.0153	1.7739	2.8935	3.5434	3.8509	2.2575	2.5755	2.5624
2.9026	2.926	4.1383	3.3158	3.2873	2.2887	2.4199	1.7889
3.271	2.8701	3.0937	1.2975	3.0126	2.4583	2.3236	3.0534
2.7146	2.5664	2.5884	2.4623	2.7429	2.5014	2.5155	2.2787
4.0753	1.9679	2.8388	2.4086	3.2508	1.7906	3.7158	2.9449
3.7977	3.2662	1.8456	4.4712	2.5858	3.3095	2.1949	2.526
2.3695	4.0051	2.9095	2.6823	2.6604	3.2468	3.4113	3.3696
2.5571	2.55	2.5367	2.7948	3.2255	1.7522	2.3455	3.2125
4.4243	3.8533						
ATP7B	2.0868	1.1911	1.9041	0.7544	1.1801	1.7597	1.4604
2.0085	2.871	2.935	2.0415	1.6474	0.7488	1.3993	1.4181
1.8663	1.6333	2.2748	3.3073	2.1438	1.6554	0.8258	1.1437
1.7633	1.432	0.6646	1.3592	2.2685	0.7055	1.6271	2.1984
1.0013	2.4943	2.3461	1.2923	2.5264	0.943	2.1182	0.9647
1.564	1.5129	0.3463	2.1988	1.4421	1.2715	0.4046	1.8518
1.7759	0.8575	1.7939	1.7615	1.0475	1.7528	1.3408	2.2451
1.9611	1.0975	1.9694	3.0471	2.2152	1.1389	1.4294	1.2121
1.6117	1.0473	0.7915	2.0471	1.4337	1.9332	1.0441	1.8396
1.7811	1.6699	1.252	1.1889	3.1339	1.9875	1.7468	2.8851
1.8486	1.5707	1.3479	1.2242	1.5631	0.3347	1.4611	1.7716
1.8247	2.0039						
ATP8A1	0.7487	0.2009	0.2157	0.0573	0.8328	0.9873	0.0099
1.8072	0.2391	1.4824	0.1107	2.0613	0	0.3559	0.153
1.1795	1.3818	0.3904	1.4684	1.8674	1.4754	0.3072	0.1089
0.8807	0.0019	0.078	1.4245	0.0228	0.0879	0.2602	2.0794
1.2904	0.0238	0.3078	0.1971	0.873	0.2637	2.9168	0.0887
0.1819	0.5138	0.8516	0.1143	1.6348	0.1115	0.1222	0.1118
1.8804	9.00E-04	0.1532	1.1557	0.1916	1.1196	0.1641	0.8735
0.7164	0.5621	0.5869	0.3899	0.4837	0.2471	1.3359	0.3062
0.2571	1.9777	0.1281	2.1684	0.2248	0.2493	0	0.0797
0.1617	0.5651	0.755	0.4066	3.1257	0.16	0.967	1.5081
1.3521	1.2642	0.5543	2.4423	0.338	0.1207	0.9496	0.5241
0.8328	2.23						
ATP8A2	0.1506	0.1226	0.8388	1.4101	1.2458	0.8204	0.3164
0.1377	0.3773	0.1419	0.1848	0.8142	0.054	0.3447	0.2713
0.5045	1.9988	0.712	0.2988	0.1627	0.3999	0.1171	0.3729
0.5093	0.6006	0.247	0.1337	0.1167	0.1475	0.3029	0.2722
0.0384	0.7483	0.0924	0.4574	0.2228	0.1566	0.2153	2.4402
0.0569	0.1853	0.1158	1.3813	0.222	0.0421	0.2713	1.039
0.0968	0.7158	0.5589	0.4309	0.911	0.3076	0.2116	0.0319
0.8253	1.1616	1.49	0.2076	1.3014	0	0.6662	0.2698
0.3612	0.0735	0.073	0	0.8417	1.0818	1.486	0.3486
0.5308	1.1058	0.5146	0	0.9499	0.1627	0.1833	0.5514
0.6656	0.2935	9.00E-04	0.0102	0.6571	0.0464	0.11	0.1805

0.4076	0.1788							
ATP8B1	0.0955	0.0786	0.0801	0.3267	0.0527	0.0778	0.0186	
0.2739	0.8415	0.4457	0.3492	1.3678	2.8324	0.2703	0.709	
0.1305	0.3495	0.3526	0.1476	0.1171	0.1456	0.3776	0.0815	
0.3099	0.0796	3.9049	0.1204	0.119	0.1649	0.0289	0.4548	
0.1823	0.3788	0.2893	1.8716	0.3649	0.0325	1.2847	0.1675	
0.2748	0.1506	3.0283	0.3879	0.2855	4.0252	0.2219	0.0743	
0.4889	0.7714	4.9863	0.1941	0.2221	0.0904	0.6182	0.7468	
0.1896	3.981	0.2362	0.3944	0.1126	0.0712	0.0278	0.1099	
0.1681	0.3155	3.9229	0.2984	0.8869	0.2074	0.1346	1.7159	
0.2421	0.1171	0.1656	0.6856	0.0757	0.2081	0.2571	0.218	
0.0933	0.1265	3.0002	0.1237	0.1096	0.9776	0.083	1.065	
0.5788	0.3751							
ATP8B2	4.4779	3.9585	4.0132	3.9528	4.7602	5.3268	5.1214	
5.1176	4.5046	4.5299	5.1689	4.4034	3.387	5.2161	4.0938	
4.7582	4.2225	4.998	3.5078	4.431	4.2582	4.3745	4.1752	
5.6244	4.306	4.4468	5.3413	4.3586	4.4389	4.0132	3.9925	
4.4754	3.852	4.8556	4.1055	4.2288	4.1873	4.858	4.1569	
5.4956	5.0463	4.4048	5.0777	5.4447	4.3242	4.5633	4.204	
4.66	3.3632	3.6282	4.5194	4.6594	5.3652	4.749	4.2869	
4.772	4.7145	3.1004	5.0549	3.5163	5.0219	4.0076	4.859	
4.6891	4.8347	4.6825	5.5505	4.8746	4.355	4.1657	4.7657	
5.0025	5.0182	4.0406	4.6736	3.8854	4.7429	4.4039	4.5802	
4.9121	4.4951	4.2989	4.9749	5.8054	3.2901	4.574	4.8275	
4.7356	5.0355							
ATP8B3	1.1881	0.1908	0.8368	0.8417	0.6085	1.0275	1.0219	
0.5772	1.4312	1.0834	0.9691	0.7404	1.5092	0.8536	1.7322	
0.5115	2.1325	1.3501	0.4852	1.1128	0.9009	0.572	0.7642	
0.8021	0.666	0.9643	0.3631	0.967	1.2513	1.0908	2.1785	
1.0213	0.5533	2.2407	0.7266	0.5137	0.9553	0.7244	1.2855	
0.9119	2.0296	1.4729	2.4869	1.074	1.8523	2.298	0.2249	
1.0301	1.6322	1.0773	0.2646	0.1752	0.9358	0.2035	1.6656	
0.7054	1.2158	2.5341	0.7803	1.9723	1.4832	0.3463	0.9531	
1.1439	1.4282	1.0141	1.9664	2.1554	0.4567	1.431	1.2424	
1.1385	0.5828	0.4969	0.9094	0.3881	0.5321	0.3493	0.8894	
1.4702	1.5632	1.5915	0.2181	0.8996	0.8122	0.8469	1.3789	
0.985	1.2449							
ATP8B4	0	0	0.042	0.1229	0.2698	0.0466	0.1537	
0.086	0.0149	0.033	0.037	0.0928	0.4884	0.0521	0.4849	
0.1508	0.0511	0.071	0	0.5484	0.0682	0.1097	0.0271	
0.0399	0.0175	1.9154	0.0286	0.0337	0	0.0569	0.1167	
0.0526	0	0	0.0251	0.1261	0.2447	0.0724	0.0137	
0.0171	0.0158	0.2423	0.0328	0.0201	0.0454	0.2528	0.0294	
0.0653	0.1664	0.2229	0.0392	0.1998	0.1328	0.0066	0.4707	
0.238	0.0081	0.4579	0.0387	0.2503	0.1404	0.0259	0.0191	0
	0.0018	0.1149	0.8226	0.0347	0.0439	0	0.1211	0
	0.0653	0.0418	0.1197	0.0969	0	0.1621	0	
0.0101	0	0.2665	0	0	0.0818	0.0666	0.2027	0
	0							
ATP9A	2.7755	3.4078	2.7109	3.4313	3.2761	4.0709	5.6017	

2.4633	3.8997	3.9177	3.0407	4.7724	4.2123	1.4201	3.738
4.7952	3.4526	3.7403	2.3425	0.655	3.3955	3.1315	3.1345
3.2005	5.1153	2.6	3.5639	2.6394	2.9607	1.8088	4.8206
1.0105	2.3581	3.7096	4.554	3.7318	2.2355	4.8223	2.7104
4.7527	2.7648	4.2755	5.1326	1.3823	4.8092	3.4495	2.6875
3.5461	5.3981	3.15	3.2702	3.8416	2.4522	3.659	3.4315
6.1171	3.1997	3.7883	2.2857	3.8034	3.4296	3.0414	3.7041
2.2011	4.2895	3.5127	2.9375	4.3732	2.802	2.0713	3.8212
1.9333	3.1077	2.68	3.7734	6.4225	2.7731	4.8494	3.7454
1.3802	3.9226	1.6433	3.9258	3.7505	3.3644	1.8099	3.149
2.6452	3.3062						
ATP9B	1.742	1.6797	1.844	1.8343	1.2703	2.0063	1.5815
1.8003	2.9693	2.5792	2.4061	1.327	1.7597	1.9215	2.1744
1.61	1.8365	2.3955	2.6177	2.2007	1.8489	1.2452	1.3117
1.8336	1.764	1.8048	2.192	1.8981	1.5735	1.6039	1.6902
1.6991	2.2087	2.3367	1.6685	2.2465	1.3937	2.943	3.2288
2.3251	2.2879	1.8745	2.1187	2.412	1.6839	2.2802	1.9794
1.9193	1.6998	1.8966	1.6537	1.9636	2.351	1.9191	1.5671
2.1855	1.7523	3.4812	2.0882	2.9078	1.9578	1.3122	2.2668
1.705	2.3956	2.461	2.665	2.561	2.0335	1.4957	1.8526
2.0158	2.4785	1.3227	1.8585	1.9694	1.6452	1.728	2.6006
1.4091	2.354	2.0329	1.873	2.1323	1.7695	2.4247	3.1206
2.6576	2.9529						
ATPAF1	5.0867	5.4786	5.1624	5.1484	4.877	4.2337	4.3634
4.2112	5.5911	5.0375	4.7763	4.8213	4.869	5.3151	4.034
4.2051	4.1227	5.1525	5.3789	4.8869	4.7533	4.2905	3.5987
4.3944	3.8451	4.2696	4.769	5.1519	4.5641	5.8283	4.7867
4.5308	4.6502	4.8627	4.8115	5.0725	4.9328	5.0449	4.8646
5.0003	4.9062	5.0111	4.0491	4.9503	5.395	4.6552	5.3508
5.0444	4.1594	4.6079	4.7809	4.9799	5.1983	5.0858	4.8791
4.532	4.1344	4.7856	4.5884	4.5188	4.5376	5.6985	4.2091
4.7777	4.4072	4.9831	3.8506	4.9311	4.8156	4.1961	4.241
4.9538	4.7283	4.7484	3.9655	4.6929	5.2883	4.4942	4.7149
5.4439	5.372	4.7256	4.376	4.6649	4.6254	5.4041	5.1453
4.9905	4.9747						
ATPAF2	2.8378	2.7599	2.4894	3.3188	3.4188	3.5142	3.7066
3.1236	2.8794	2.7442	2.1878	2.5944	3.0402	2.1551	2.6106
3.3173	3.1456	2.5062	2.3185	3.2824	2.7608	2.762	3.0145
3.4945	2.3994	3.2983	3.0853	2.7273	3.9952	2.8904	4.6047
2.1706	2.9299	2.0203	3.1974	2.7587	2.6518	4.0943	3.1677
3.0999	2.9723	3.4185	2.1449	2.8659	2.6775	3.0136	2.7539
2.6646	2.4071	2.7569	3.0459	2.5561	2.9217	2.8188	3.4771
1.7493	3.2554	3.0264	2.5307	2.8777	3.6762	4.0193	2.607
3.3702	2.7171	2.977	2.2305	3.3089	2.3363	3.933	3.3214
3.1047	3.0306	3.5642	3.6164	2.497	2.2999	3.5302	2.8985
3.1422	2.5459	2.9406	3.1606	2.3031	2.9394	3.0789	2.9414
3.0335	3.0617						
ATPIF1	5.9535	7.0089	6.0947	7.101	6.8055	6.2407	6.2295
5.5034	5.8877	6.4447	6.0903	6.7565	7.0592	6.4273	5.6045
5.8072	5.8012	6.1967	6.4534	6.1073	6.3923	5.5152	6.3656

6.3586	6.0104	6.0762	6.1012	6.782	5.9851	6.9294	7.0639
6.1631	6.7913	6.8413	6.1406	6.6917	6.8307	6.4506	5.7438
6.5424	6.3276	7.4787	6.5139	6.1845	6.9832	6.8689	6.1224
6.0379	5.6747	6.972	6.1176	6.0937	6.5394	5.8848	6.6358
6.4782	6.1758	6.0851	6.3681	6.1594	6.0769	6.7653	6.4483
6.8726	6.1803	7.3345	6.4324	6.9773	6.58	6.2729	6.5047
6.0409	6.3413	6.9036	5.5234	5.8263	7.2255	5.7743	6.4863
6.5105	6.8884	6.365	6.3041	5.3791	6.5493	6.8858	6.0401
6.17	5.8877						
ATR	2.6646	2.6054	2.7762	3.0574	3.0605	2.7715	3.3437
2.6251	3.5711	3.2155	3.4647	2.8963	2.9949	3.261	3.0797
2.8073	3.3852	3.2838	3.3242	2.6835	2.7076	2.7621	2.4314
3.3706	2.1355	2.6993	2.8283	2.2881	3.001	2.5552	2.4016
2.858	2.9486	2.9282	2.5739	3.2076	3.0187	2.639	2.8657
4.1414	2.4288	2.8025	3.3113	2.5971	3.4322	2.9688	3.3245
3.2331	2.6029	3.6678	3.1775	3.2277	2.9559	3.1356	2.8438
2.8362	3.6045	2.8514	2.7968	3.06	3.1653	2.6468	2.2287
3.4051	2.4567	2.829	2.9728	2.8677	2.8029	2.7341	2.9533
3.4299	2.7508	3.3852	2.1046	2.7823	3.1356	3.0752	2.9829
2.7365	2.706	2.1677	1.9344	3.0932	2.3414	2.7953	2.2049
2.7271	2.5042						
ATRAID	6.7514	5.9838	5.6801	6.4056	6.1684	6.157	5.8439
6.4263	7.0772	6.4569	6.7445	6.404	6.1401	6.1274	5.9922
5.717	6.6312	7.0746	6.2015	6.2585	5.7479	6.7244	7.6746
6.2647	6.9422	6.1783	7.1598	5.7654	7.0834	6.7754	6.8134
5.9026	6.4108	6.9633	6.5188	6.6695	6.5223	6.5679	6.3338
7.1965	6.6335	7.0121	7.1772	6.6359	6.6782	6.5316	6.8134
6.7138	6.9473	6.2291	6.8113	7.0235	6.2823	6.3752	6.4565
6.1528	5.6065	6.2977	5.7108	6.1738	6.6388	6.2327	6.4316
5.8503	6.4527	7.3904	7.0234	6.1301	6.2121	6.9939	6.8142
6.3629	6.8832	6.1877	6.7233	5.5426	5.4413	7.0293	6.8409
6.6829	6.2582	6.9155	6.5238	6.717	6.1998	6.1762	6.4494
6.247	6.2258						
ATRIP	1.893	2.3075	2.317	2.3409	2.7959	2.056	1.8998
2.2845	1.818	2.2927	2.0942	2.1756	2.402	2.4042	1.6188
1.7159	2.0946	2.3604	2.5749	2.3249	2.2348	1.9202	2.0167
2.6079	1.8023	1.8808	0.9628	2.0783	2.5326	2.0038	1.4744
2.2437	2.2654	1.5537	1.509	2.6204	2.0107	1.3962	1.8697
2.5536	1.4469	1.5991	2.3015	2.0448	2.8712	1.8355	2.7392
2.1845	1.4679	2.5267	1.4889	2.7355	1.945	2.0657	2.2723
1.83	1.6119	2.5332	2.6428	2.3745	2.1315	2.2319	2.2232
2.1266	2.3358	1.4485	2.3114	1.8023	2.0644	2.0036	1.9083
1.8037	2.6911	1.7616	1.8506	1.6419	2.3096	1.9792	1.4485
2.0633	1.9725	1.2111	1.4212	1.2181	2.7014	2.229	1.4889
2.0576	1.6301						
ATRN	4.4721	3.9223	3.5709	3.6862	4.3173	4.4782	4.5693
4.9479	5.242	3.9748	4.9983	4.3512	4.4493	3.9365	3.4703
4.5927	3.8129	4.9372	4.6223	3.8946	3.8	4.3615	5.0843
4.2104	4.6037	5.1735	4.3258	4.2086	4.4317	4.1999	4.0886
4.3772	4.8321	4.4165	4.3491	3.976	4.2339	4.8777	4.911

5.0225	4.2036	3.6152	3.8244	5.7887	5.0761	4.084	4.8944
3.8598	4.9219	4.3763	4.9038	5.4532	5.8227	4.7314	4.2167
4.3063	3.7366	3.676	4.8665	3.6721	4.1444	4.0367	4.573
4.0679	3.9209	4.6786	4.2135	4.1582	4.4501	4.7499	4.0687
4.7305	4.8792	5.0433	5.7912	3.1147	4.2302	5.2529	4.5361
3.9963	4.7677	4.6521	4.2968	5.4586	3.4679	4.9143	4.7935
4.2435	4.5339						
ATRNL1	2.7955	4.0947	2.5751	2.1082	1.0537	2.9141	2.055
2.0711	3.1767	2.6616	2.1163	0.9026	1.9762	3.1846	0.91
2.2317	0.8085	0.2829	2.5619	2.4118	2.4171	2.2063	1.6991
2.5941	1.5814	0.635	1.8451	2.497	1.8891	1.7557	2.8257
1.6929	2.0193	2.6565	2.6823	3.0204	1.5279	1.8366	1.4395
2.1008	2.63	0.0872	1.7032	3.1289	0.8538	0.404	3.045
2.0397	2.6494	0.9804	1.8638	2.3003	2.3146	0.7555	3.5529
1.7834	0.0836	1.8879	2.3844	1.3337	1.9398	2.4744	2.7993
2.2786	2.7531	2.7199	3.4663	0.6679	1.7714	1.2654	2.9334
2.2938	1.6217	0.9668	2.7725	2.8889	1.7734	2.574	1.2299
1.7539	2.172	1.5109	1.4637	2.9595	2.2572	2.504	1.543
2.6168	2.2604						
ATRX	2.878	3.1805	3.0307	3.0405	3.3857	2.9136	4.4213
2.7026	3.8551	2.9859	3.1933	3.2585	3.2316	2.8384	3.1261
3.5035	3.5851	3.3952	2.1827	2.6444	2.7263	3.0976	3.6004
3.2569	3.4168	3.3924	3.5674	3.1119	3.1373	3.0899	3.6762
3.4385	2.8708	3.5065	3.6938	3.0928	2.8057	3.2494	2.7026
3.7534	2.8788	3.2361	2.9535	3.0307	4.3707	3.3949	3.5588
4.1181	3.6141	4.6419	3.5395	3.2983	3.2035	3.6732	3.3133
3.458	3.4441	3.4354	2.8715	3.6415	2.7398	3.6568	3.7987
3.6357	3.4983	3.3672	3.2902	3.8224	3.6533	2.7768	3.7433
2.651	3.0804	3.3852	3.8788	3.5099	2.7226	4.287	3.4268
2.4282	2.7868	3.3078	3.474	4.0136	2.3179	2.7382	3.418
3.3606	3.467						
ATXN1	2.2643	3.296	3.177	3.6834	2.8957	3.7457	3.5941
3.2574	3.363	3.2624	2.8521	3.8489	3.7035	3.0729	3.0986
3.5586	3.4402	3.2353	3.601	2.0738	2.9121	3.8154	3.363
3.8599	4.4653	3.8445	3.8437	2.948	3.466	2.8323	2.1551
2.4742	2.9154	3.2965	3.5356	3.1802	3.0697	2.8092	3.2071
4.4485	3.1708	3.8361	2.5661	3.0282	3.1822	4.9928	3.5058
3.0636	3.0466	3.7043	3.0431	4.0073	3.3758	3.1389	2.3503
1.4827	3.0175	2.7125	3.318	2.682	3.4323	3.1583	3.8081
3.3272	2.4516	3.2357	3.8481	3.9716	2.7814	2.9407	3.714
3.0859	2.3513	3.4735	3.5395	3.6783	3.3055	3.7114	3.1119
3.6661	2.9144	3.9633	4.2222	3.1011	3.829	3.0152	3.4421
2.4767	3.0849						
ATXN10	5.8405	5.3298	5.2009	5.6181	4.4453	5.1275	4.829
5.8327	5.8967	5.335	5.2044	5.2658	5.1041	5.383	5.4741
4.7764	4.4162	5.6013	6.5154	5.0291	5.206	5.4781	5.2344
5.8493	5.9183	5.1105	4.6779	5.3972	5.2637	5.9206	5.3051
4.1327	5.1405	5.3269	5.8588	5.3291	5.5327	4.8053	5.8769
5.8294	5.3342	5.8273	5.6633	4.7781	5.7256	5.879	6.0729
5.345	4.7462	5.9242	5.0189	5.23	5.7584	6.374	5.007

5.1379	6.0538	5.2447	5.3768	5.2722	5.1041	5.4514	5.9627
4.7824	5.1937	4.8705	5.707	4.7592	4.9952	5.0573	5.6199
5.128	5.0907	3.9911	5.506	5.1848	4.7505	5.0501	5.5261
5.6857	5.6421	5.449	4.1419	5.1888	5.722	5.6521	4.8364
5.2336	5.0449						
ATXN1L	2.9342	2.4851	3.2097	3.1313	2.7113	2.9015	2.6648
2.958	2.9572	3.1051	3.2325	2.7208	3.5559	3.9795	2.9273
3.1652	3.0497	3.0067	2.6882	3.8302	3.3256	2.636	2.6418
3.0431	3.2858	3.2707	3.2155	3.2261	2.338	2.5382	2.9238
2.9626	2.429	2.5226	3.0666	3.017	3.4153	3.0889	3.0944
3.0361	2.5291	2.4302	2.3224	3.6571	3.4366	3.0243	3.1008
3.3496	2.4842	3.0647	3.1453	3.1445	2.9392	3.2024	3.2927
2.5441	3.3684	2.697	3.1952	3.1473	2.7992	2.7833	2.7468
2.8304	3.106	2.4517	3.0592	3.132	3.2193	3.3446	2.7772
3.2045	2.6159	2.359	3.1773	2.7764	3.0562	3.5686	2.8304
2.4497	2.486	3.0213	2.9776	3.2304	2.8286	2.5169	3.2832
3.2429	3.2433						
ATXN2	3.6656	3.2077	3.5749	3.741	3.8383	3.6656	3.924
3.5083	3.6916	3.5071	3.9079	3.6323	3.6097	3.9329	3.0244
3.2935	4.3284	3.8694	4.1181	3.3653	3.7564	3.2978	3.2875
3.4843	3.6783	3.8602	4.8047	3.7948	2.6969	4.2568	4.219
3.8579	3.4408	3.4676	3.5772	3.9181	3.8721	3.9397	3.0279
3.9546	3.9477	3.9283	3.5225	3.7033	3.6532	3.8341	3.7467
3.7134	3.4726	3.7495	3.2383	3.9421	3.8528	3.6389	4.1209
4.0198	4.0211	4.0627	3.6367	3.7547	3.6986	4.0434	2.8916
3.9153	4.0309	4.0572	3.8402	3.8227	3.9186	3.0968	3.5097
3.2898	3.7357	3.7089	3.4155	2.9345	3.7984	3.2763	4.2947
3.4746	3.6367	4.0337	4.1209	3.7187	3.2618	3.5408	4.0506
3.4799	3.5905						
ATXN2L	5.757	5.6973	6.0783	5.3885	5.9594	5.6215	5.3307
6.2568	5.0407	5.4437	5.292	5.0155	6.6081	5.1297	5.5022
5.2759	5.9267	4.5408	5.564	5.5475	5.7875	5.8411	5.0572
5.4616	5.7893	6.1027	5.7314	5.6948	5.35	4.8853	6.6302
5.6301	5.1823	5.2418	5.6668	5.3544	6.3006	5.6335	5.8037
5.1555	5.6	5.0555	5.4674	5.6245	5.6717	5.3071	5.0775
5.627	5.6939	5.1236	5.8439	5.0303	4.9682	6.4094	6.2931
5.2492	5.5873	6.097	5.4844	6.4348	5.5863	5.4165	5.7762
5.5651	5.1833	5.5746	5.6084	5.513	5.8941	5.9335	5.1308
5.4631	5.3968	4.9411	5.5101	5.5315	5.5061	5.0971	5.1436
5.1269	5.4918	5.3002	6.4148	5.4956	5.4784	4.771	5.1635
5.0878	5.0506						
ATXN3	2.6589	3.2566	2.4353	3.0815	2.9075	2.6008	2.9351
2.3639	3.2024	2.9648	2.3254	3.5901	3.1188	3.3176	2.3914
3.3557	3.2998	3.7748	3.2662	1.8462	2.8144	2.8227	3.1497
2.2503	2.7984	2.5205	3.1705	2.4198	2.8227	2.9119	2.8539
2.7694	3.2618	3.1088	1.7025	3.1343	2.9387	2.895	2.5463
3.319	3.1212	3.1476	2.8255	2.663	3.1303	3.9943	2.8433
2.9751	2.792	4.5906	2.4724	3.3245	3.3859	2.8297	2.5303
2.912	3.887	2.507	2.5122	2.8961	2.6801	3.1409	2.7027
2.8716	2.8456	3.7761	2.9262	3.5107	2.4605	2.364	3.2242

3.3095	3.0288	3.3628	3.8516	1.5746	2.9935	2.425	3.6313	
2.7397	3.39	3.3037	3.6689	2.7795	2.8581	3.0152	3.8118	
3.1743	3.6755							
ATXN3L	0	0	0	0	0.874	0	0.5137	0
	0	0	0	0	0	0	0	0
	0	0	0	0.1932	0	0	0	0
	0	0	0	0	0	0	0	0
	0	0	0.0068	0	0	0	0	
0.0087	0	0	0	0	0	0	0	0
	0	0	0	0.9843	0	0	0	0
	0	0	0.3786	0	0	0	0	0
	0	0	0	0	0	0	0	0
	0	0	0	0	0	0	0	0
	0	0	0	0	0	0	0	0
	0							
ATXN7	2.2243	2.403	3.2007	2.629	3.1347	2.6062	3.4492	
3.0077	2.3049	3.383	2.8652	3.0331	3.1264	3.2782	2.8639	
2.8822	3.288	3.0693	2.7189	2.4724	2.9552	2.5422	2.2784	
3.253	2.212	2.5616	2.2595	2.6832	2.5527	2.3092	2.4655	
3.1378	3.736	2.3072	2.4419	3.0858	3.0492	2.6842	2.7732	
3.0427	2.4383	2.5163	2.5817	3.0949	4.0146	2.2058	2.7858	
3.2987	2.4428	3.2469	4.4483	3.0396	4.0426	2.9052	3.0829	
2.1662	2.8624	2.1266	2.5095	2.482	2.7753	2.6608	2.6338	
3.6761	2.434	3.2501	2.8096	2.6087	2.9699	2.3042	2.3608	
2.4719	2.3771	2.1545	2.645	2.3425	2.8731	2.5659	3.3152	
3.6893	4.1723	2.7018	2.8366	2.3234	2.4452	3.67	2.633	
2.4743	2.6501							
ATXN7L1	1.8226	2.3838	2.004	1.553	1.5469	3.1227	2.6008	
2.2979	1.8188	1.6998	1.6206	1.5245	1.7629	2.5574	1.9909	
1.6637	1.6845	2.4959	2.6138	2.0916	1.6211	1.4713	1.4295	
2.0675	1.7447	1.9832	0.9862	1.8948	1.7842	1.2795	1.6544	
1.6662	1.9981	1.274	2.3801	1.7905	1.7623	1.9245	1.7101	
1.868	1.5287	1.2383	1.6986	2.3709	2.2162	2.5248	1.7486	
2.1442	1.4995	1.9513	1.751	2.1804	1.5968	1.9914	1.686	
1.4912	1.6395	1.8718	2.1202	1.9011	1.3719	1.7554	2.2214	
2.3633	2.271	1.9392	2.2146	2.337	1.2774	2.3833	1.9604	
1.6828	1.8328	1.2326	1.7155	1.7928	2.4441	1.9367	1.7211	
1.9632	2.1298	1.6244	0.7711	2.6387	1.812	1.1217	1.7677	
1.2838	1.6017							
ATXN7L2	1.2613	1.8198	2.1536	0.7644	2.1675	1.5637	1.3818	
1.4983	0.8976	1.1811	1.0389	1.1091	1.0422	1.6633	1.069	
1.4295	2.021	0.8283	1.2281	1.6298	1.5367	1.6227	0.335	
1.1369	1.2102	1.4178	0.9735	1.3695	0.9527	0.594	1.6489	
2.3469	1.3637	1.1046	1.0546	0.4374	1.5053	1.5014	1.1784	
1.0696	1.2967	0.9596	0.873	1.2824	0.654	1.1693	0.8033	
1.4153	1.2575	1.3797	1.2626	1.102	1.2607	2.0114	1.907	
2.0626	1.0475	1.5779	0.8873	1.8924	1.2943	1.3892	1.1919	
1.6406	1.4306	0.8635	1.4795	1.4023	1.0994	1.0875	0.7502	
1.5574	1.379	0.4145	1.027	1.2497	1.8782	0.5124	0.9143	
0.9713	1.4317	0.8891	1.4235	0.9792	1.7907	1.379	1.2017	

1.4625	1.4593						
ATXN7L3	4.2348	4.0654	4.8227	4.31	4.8169	3.9924	3.8742
4.6343	3.5119	4.0604	4.2745	4.2263	4.5112	3.2754	3.6798
4.2749	5.0119	3.8099	3.3962	3.8472	4.2118	4.0091	4.232
3.6237	4.4554	4.7598	3.4227	5.0284	3.9452	3.8274	4.5657
4.5729	3.7331	4.122	3.936	3.8717	4.7636	4.4682	4.2393
3.7145	3.9602	4.3536	4.3299	3.793	4.7035	3.9541	3.6594
4.8366	4.5757	4.4314	4.3227	3.9325	3.5368	4.653	4.0232
4.6106	3.8861	4.659	4.2079	4.3766	4.4868	3.9616	3.6147
2.9126	3.9905	4.7575	3.589	4.1763	4.5305	4.5656	4.1196
4.0388	3.7441	4.226	4.3619	4.6498	4.2138	4.2381	3.5237
4.0331	4.1576	4.1988	4.6038	3.7531	4.5822	3.5264	4.1958
4.0566	4.2569						
ATXN7L3B	4.9877	4.5788	5.2765	5.3389	5.3238	5.3856	6.083
4.8489	4.2675	4.5749	4.6257	4.3556	4.549	4.392	4.0884
4.8421	5.1811	4.7349	4.2843	4.7857	4.8805	4.8922	4.6016
3.4647	5.0769	5.0257	4.8816	5.3734	4.3114	4.9748	4.9668
5.4832	4.4569	4.4482	4.5628	4.6822	5.1811	5.5882	4.8578
5.238	4.8839	5.0873	3.9147	4.0211	4.9962	5.3161	4.7845
5.4708	4.9573	5.1045	4.3173	5.0853	5.4504	5.4285	4.9749
4.7049	5.0788	5.4121	4.4072	4.8491	5.219	4.9309	5.1707
4.5945	5.0611	4.9285	5.4072	4.6625	4.5577	4.8821	4.2894
3.7827	4.741	4.2783	4.6864	5.6984	4.9489	5.191	4.1855
4.4443	4.5283	4.8349	4.4873	5.0056	4.589	4.6639	4.7833
4.6252	4.6882						
AUH	3.2754	2.9145	2.2503	3.3575	2.3559	2.7106	2.7973
3.3508	3.0293	3.2767	4.0075	3.2212	3.8553	2.7382	2.1743
2.5784	3.2288	3.4859	2.9194	3.2655	2.6539	2.3316	2.1436
2.6745	2.2943	2.0874	5.2049	2.6736	2.2947	3.9428	2.9055
2.4111	2.4696	3.8892	2.05	3.2799	3.0543	4.2051	3.7546
2.8838	3.3839	3.6842	2.1473	3.2363	2.5985	3.3943	2.9376
2.4772	2.9682	3.3383	3.1556	2.2401	3.5833	3.711	2.9989
2.0038	4.1912	3.4145	4.5435	3.1027	2.8138	2.8297	2.9897
2.9917	3.6707	4.0889	2.6349	3.3014	3.3415	2.9384	2.6533
2.4413	2.8933	3.7599	2.4049	2.5792	2.721	2.9106	2.4595
3.293	2.5771	2.4959	3.3986	3.548	2.7296	3.7629	3.8739
3.5288	4.2626						
AUNIP	2.8188	3.7227	3.3241	3.2754	3.0948	2.5671	2.7874
2.2198	3.3035	3.3929	2.6889	3.0295	2.8859	3.3804	2.3965
2.7378	2.4317	2.6801	3.7855	3.3662	3.1834	2.6234	2.402
3.2104	2.0896	1.2044	2.5357	2.8621	2.7743	3.6387	2.7582
2.6086	3.593	2.5724	2.3068	3.7364	2.1288	2.2609	2.9186
3.5048	2.3625	2.3302	2.6813	3.021	2.4143	2.6907	3.4619
3.2215	1.5935	3.5076	2.8103	3.1775	3.0061	3.1745	3.317
2.89	2.0857	2.9842	2.8345	2.9439	2.791	3.1957	2.3829
3.2438	2.4258	1.0758	2.6853	1.6688	3.4413	2.7296	2.0387
3.0036	2.7902	2.9089	1.7672	2.4001	3.4968	1.8834	2.7822
3.1151	3.5167	2.3038	1.7748	1.8225	3.0163	3.0423	1.7605
2.405	1.9119						
AUP1	6.8582	6.9495	6.7496	6.5608	6.5113	6.4089	6.6235

6.8534	7.2384	6.6214	6.3481	6.3555	6.1468	6.8671	6.6196
6.2118	7.2802	6.2274	6.4089	7.2149	6.7714	6.8896	6.5307
7.4426	6.599	6.3527	6.2597	6.6748	7.271	6.6009	6.3705
6.6047	7.13	6.2931	6.9598	6.3942	7.3269	6.7672	6.4536
6.8337	6.199	5.9648	6.6638	6.4592	5.7287	6.1162	7.0735
6.9327	6.8856	5.3777	6.8716	6.0816	6.5645	6.6951	6.3645
6.7644	5.8266	6.7871	6.7936	6.8733	6.7306	6.979	6.6623
6.443	7.34	5.8794	7.735	6.463	7.1066	6.697	6.3997
6.4957	7.0438	6.3758	6.1792	6.6331	6.8366	6.7895	6.8388
6.81	6.5057	6.3533	6.7621	6.2051	5.6918	6.401	6.6658
6.5149	6.6579						
AURKA	5.6963	5.633	6.4637	5.892	5.6233	5.268	5.4168
5.8363	5.8385	6.5745	4.7324	5.8885	6.2596	5.7147	6.0084
5.2074	5.423	5.4344	5.892	5.5654	6.0678	5.6752	4.9403
5.4573	5.3298	5.0993	3.72	5.3323	5.7316	5.7213	6.4157
5.0693	7.0024	5.6263	5.0428	6.8511	5.2948	3.1389	4.7988
6.168	3.5141	4.3326	6.0471	5.8897	6.5264	6.436	6.0762
5.4653	4.5957	6.2057	5.0962	4.6513	5.7571	4.0805	6.4154
5.6814	5.1677	6.4658	5.8513	6.5225	6.4429	5.411	5.1618
6.2194	5.1268	3.1279	4.8631	3.2601	5.6262	5.4899	5.6782
6.3757	6.1535	5.7034	5.7396	5.7599	6.0568	4.7381	4.7848
5.6689	5.7674	4.4877	3.5716	4.7393	5.8286	5.8084	4.067
5.02	4.6156						
AURKAIP1	6.3072	6.6592	7.6508	5.2627	6.9977	5.5057	5.7627
6.2189	5.865	5.1378	6.2048	6.0816	6.3481	5.1669	6.2387
5.9015	5.3865	4.5384	5.4852	6.8075	7.542	5.7992	6.5385
6.6276	5.6103	5.7168	4.9272	5.9626	6.2134	6.3714	6.48
6.8879	6.9921	6.951	6.1044	4.7877	6.4474	6.3938	6.0465
5.1787	6.41	6.2387	5.6348	5.6519	4.9601	5.2112	4.615
4.9657	6.1728	4.7991	5.6505	4.6993	5.6289	6.0714	6.6734
6.4205	5.0014	6.8543	5.8861	6.2606	6.4544	5.6678	6.292
5.6662	5.3383	5.7023	6.0357	5.5844	6.103	5.7806	5.5478
5.7891	6.0576	6.5879	4.8145	6.0675	7.2283	5.3076	5.4599
6.2405	6.2648	5.825	5.7309	5.0918	6.8035	6.8666	5.7907
6.4179	6.1333						
AURKB	5.6558	6.8283	6.0493	4.6958	5.7026	3.8206	3.4624
4.9936	5.4909	6.321	4.1083	4.8429	5.3179	4.1028	4.6048
4.0293	5.0232	5.2908	5.4273	5.3312	6.7741	4.8799	4.4622
5.0318	4.401	3.6619	4.3259	5.8565	5.2199	5.3689	5.8565
4.1843	5.8619	4.8197	4.6718	5.8827	3.8695	2.8552	4.6468
4.4651	3.9377	3.454	5.5656	5.9468	4.5177	5.451	5.497
5.4224	3.1792	5.3593	4.9558	4.113	4.7913	3.681	6.0754
5.2678	4.5127	5.4532	5.0417	5.3989	5.2074	5.5877	5.7964
5.5963	5.1598	2.0191	5.1995	2.7225	4.9267	5.4655	4.7281
6.0766	5.7165	4.7566	4.438	3.9667	5.5089	4.3113	5.2177
5.6893	6.1899	3.3577	2.4939	3.9129	5.5442	5.8937	3.9435
5.7226	5.1107						
AURKC	0.3217	0.7183	0.2071	0.1797	0.1621	0	0.0904
0.0065	0.3666	0	0.3138	0.0395	0.7314	0.4503	0.2357
0.1876	0.9636	0.3932	0.4778	0.2563	0.5154	0.4325	0.2695

0.0217	0.2951	0.4403	0.8465	0	0.2044	0.5619	0
0.3222	0.4416	0.4286	0.2018	0	0.1487	0.2695	0.438
0.1895	0.4083	0.0087	0.3159	0.0796	0.4828	0.3615	0.4023
0.5349	0.4267	0.4361	0.4398	0.152	0.328	0.3735	0
0.8544	0.5185	0.1604	0.5599	0.238	0.1935	0.785	0
0.1556	0.2563	0.1159	0.4827	0.1627	0.9839	0.114	0.1428
0.079	0.1274	0.2359	0.1047	0.6792	0.217	0.0802	0.1909
0.7219	0.3874	0.1404	0.4552	0.0809	0.2152	0.2686	0.2415
0.7782	0.3599						
AUTS2	3.0431	1.2664	1.5562	1.0513	0.0741	3.087	1.8175
0.9338	2.9845	2.3797	2.636	0.1172	1.6614	0.2612	4.4554
3.2996	0.1057	3.5886	3.5679	3.1421	2.4363	1.7906	0.5458
3.5941	3.7453	0.4796	1.5723	1.7051	0.18	2.6805	1.5071
1.9642	0.9829	1.398	0.5803	1.6035	1.0166	2.2142	2.7296
1.6168	0	0.5434	0.1673	3.0164	2.661	2.9502	2.4995
0.2612	0.247	1.7457	0.337	0.679	0.4723	0.2207	1.126
3.4333	1.6535	2.4507	0.6814	2.9417	0.3691	0.8294	2.1571
3.2875	3.2656	2.7559	2.5721	1.7017	2.4681	3.2036	0.5709
1.7794	2.7327	0	4.3126	1.4684	0.1479	3.4923	1.9397
0.0081	1.9446	2.3641	1.372	1.7763	0.2031	0.2671	2.5815
2.9989	3.021						
AVEN	2.6261	3.6286	2.8271	3.8313	2.9575	3.4226	3.1879
3.0827	3.1966	2.1544	2.3103	2.6423	3.6696	3.1306	2.5875
2.8349	2.7651	4.0029	3.9395	2.8357	3.0931	3.1951	3.0039
2.592	3.0317	3.2735	2.8233	2.8583	3.8576	1.8806	1.6824
2.5804	2.6658	1.8425	3.2366	2.6059	2.8314	2.2588	2.6581
2.908	1.0689	3.3418	3.3477	2.7969	3.5012	4.0812	3.9697
2.7117	3.1788	4.09	3.4613	2.9423	2.4933	4.0501	2.9794
3.9339	3.5837	3.3035	2.6882	2.9617	2.8397	2.8757	2.9451
2.9998	3.4253	3.6613	1.6827	3.9906	2.9265	2.8762	2.7445
2.77	3.2924	1.6846	2.2771	3.2134	2.2657	2.8993	3.2092
3.0297	2.6204	2.8167	3.9988	2.054	3.7394	2.2313	3.3325
3.3411	3.1947						
AVIL	0.4621	0.1106	0.5101	0.4243	0.3344	0.3414	0.6614
0.1716	0.3217	0.3697	0.1379	0.1892	0.4686	0.376	2.6831
0.2549	0.5574	0.2369	0.7117	0.3788	0.2857	0.1892	0.1883
0.0567	0.2827	0.3351	0.1345	0.5277	0.1498	0.2385	0.1222
0.1575	0.5369	0.213	0.4196	0.2482	0.2848	0.467	0.3237
0.447	0.3411	0.2916	0.1528	0.3432	0.2458	0.3538	0.3337
0.2059	0.8633	0.7809	0.2052	0.212	0.6523	0.7555	0.0988
0.6795	0.2843	0.2202	0.2056	0.1483	0.1468	0.3379	0.3356
0.5661	0.1764	1.0791	0.1522	0.3188	0.143	0.2118	0.1314
0.1317	0.1252	0.5085	0.1745	0.7842	0.792	0.1701	0.4791
0.3074	0.4074	0.3783	1.6698	0.0103	0.2312	0.6442	0.3092
0.1269	0.2583						
AVL9	4.5765	3.6208	3.6256	4.3996	3.7376	3.9878	3.9368
4.4578	4.0883	4.1217	3.9313	3.318	4.2801	3.8752	3.2206
4.0363	4.1565	4.5677	4.2504	4.2455	3.5859	4.4929	4.5097
4.4448	4.915	4.5864	4.3005	3.7638	4.2504	4.0194	4.1927
3.841	3.4915	3.6944	4.248	4.2557	4.5405	3.9758	4.0664

4.5687	4.2646	3.3015	4.5489	4.6263	4.5968	4.5362	4.8483	
4.6403	4.32	3.8487	4.467	4.4157	5.2154	3.5869	4.6799	
4.2299	4.0775	4.3385	3.9588	4.3075	3.853	3.8366	4.229	
3.5878	4.203	3.9134	3.8488	3.9228	3.7551	4.4247	3.3777	
3.9196	4.9584	4.0072	3.9914	3.9725	3.2354	4.4952	3.8488	
3.6822	4.0751	3.4557	5.3214	3.9361	3.3992	4.331	3.5364	
3.6644	3.4584							
AVP	0	0	0	0	0	0	0	0
	0	0	0	0	0	0	0	0
	0	0	0	0	0	0	0	0
	0	0	0	0	0	0	0	0
	0	0	0	0	0	0	0	0
	0	0	0	0	0	0	0	0
	0	0	0	0	0	0	0	0
	0	0	0	0	0	0	0	0
	0	0	0	0	0	0	0	0
	0	0	0	0	0	0	0	0
	0	0	0	0	0	0	0	0
	0							
AVPI1	5.7624	5.8579	5.382	4.8775	5.1375	4.6525	4.8509	
3.7371	4.6478	5.0673	5.9146	5.0957	4.257	4.7059	4.0012	
4.9589	2.6571	5.7509	4.4359	5.8608	5.8495	3.5964	3.6062	
3.7192	3.3682	3.3982	4.7916	5.6464	3.232	4.3639	2.7309	
5.5587	3.5997	3.3689	3.1796	5.4364	6.3594	4.1875	4.2406	
2.8262	4.7825	2.8482	3.7936	3.6054	2.8395	2.678	5.0403	
4.2057	2.8575	3.3359	3.6918	3.0566	4.1383	6.2056	4.9222	
5.2365	2.3835	5.6845	4.4637	4.8071	3.9694	5.5575	4.1958	
4.8752	4.3804	4.4082	3.9956	5.0448	5.1678	3.6558	3.182	
5.2535	5.137	3.7239	2.1072	3.2183	6.5843	3.5796	3.5116	
4.5032	4.2081	3.241	5.3959	3.5972	3.344	4.0362	6.4201	
5.648	5.9075							
AVPR1A	0	0	0.5055	0.0589	0	0.0426	0.1804	0
	0.2526	0.0268	0	0	0.0028	0.0202	0	0
	0.0288	0	0	0.0517	0.042	0.0099	0.0558	0
	0	0	0.0206	0.506	0.0194	0	0	
1.0354	0.0037	0	0.0945	0	0.0107	0.0115	0	0
	0	0.0213	0	0.1959	0	0.2238	0.1125	
0.0069	0.3198	0.0395	0.0269	0.0077	0.0711	0.0116	0.0226	0
	0.0231	0	0.0168	0	0.0374	0.0139	0.0213	
0.0207	0.0242	0.0124	0	0.0163	0.0461	0.0866	0.0401	
0.1152	0.076	1.4643	0.157	0.1818	0	0.0187	0.059	
0.0611	0.049	0.0757	0	0.0405	0.0724	0.0317	0.0116	
0.1219	0.0324							
AVPR1B	0	0	0	0	0.0445	0	0	0
	0	0	0	0	0	0	0	0
	0	0	0	0	0	0	0	0
	0	0	0	0	0	0	0.024	0
	0	0	0	0	0	0	0	0
	0	0	0	0	0	0	0	0
	0	0	0	0	0	0	0	0

	0	0	0	0	0	0	0	0
	0	0	0	0	0	0	0	0
	0	0	0	0	0	0	0	0
	0	0	0	0	0	0	0	0
	0							
AVPR2	0.5304	0.056	0	0	1.1555	0.0781	0	
0.8343	0.1518	0.7448	0.1134	0.1052	0.1356	0	0.0326	
0.5144	0	0.2664	0	0	0.3032	0	0.1714	
0.2135	0.2164	0.0707	0	0.0792	0	0.0549	0.1243	
0.1644	0.0966	0.3403	0.0387	0.1053	0.1405	0.1481	0.0703	0
	0.1686	0.1284	0	0.0521	0.0374	0.05	0	
1.2049	0	0.1359	0	0.7804	0.1061	0.1132	0	0
	0	0	0.3814	0	0.0702	0.2362	0.14	0
	0	0	0.0616	0.061	0.0504	0.1339	0	
0.1171	0.0402	0.0136	0.1912	0.0654	0	0.9911	0	
0.8065	0.5367	0.216	0	0	0.5047	0.2928	0	0
	0.0701							
AWAT1	0	0	0	0	0	0	0.0242	0
	0.0273	0	0.0165	0.0142	0	0	0	0
	0	0	0.0916	0.1291	0	0.0145	0	0
	0	0	0.0391	0	0	0	0	
0.0061	0	0	0	0.0933	0	0	0	0
	0	0	0	0	0	0	0	0
	0	0.1001	0	0	0	0	0	0
	0	0	0	0	0.1253	0	0	0
	0	0	0	0.0195	0	0	0	0
	0	0	0	0	0.0305	0	0	0
	0.0504	0	0	0	0	0	0	0
	0							
AWAT2	0	0	0	0.1255	0	0	0	0
	0	0	0	0.0076	0	0	0	
0.0122	0	0	0	0	0	0	0	0
	0	0	0	0	0	0	0	0
	0	0	0	0	0.009	0	0	0
	0	0	0	0	0	0	0	0
	0	0	0	0	0	0	0	0
	0	0	0	0	0	0	0	0
	0	0	0	0.0085	0	0	0	0
	0	0	0	0	0.0179	0	0	0
	0	0.0262	0	0	0	0	0	0
	0							
AXDND1	0.124	0.033	0.0714	0.1296	0.0682	0.0255	0.1017	
0.1162	0.038	0.0354	0.0427	0	0.1496	0.0405	0.024	
0.0105	0.0601	0.0632	0.0738	0.0821	0	0.0489	0	
0.0515	0.0481	0.1637	0.0458	0.0379	0.0442	0.0266	0.0501	
0.8633	0.1127	0.0168	0.0368	0.0429	0.0512	0.0964	0.0373	
0.0391	0.1481	0.0212	0.0384	0.1162	0.0167	0.0293	0.0944	
0.0477	0.0156	0.0459	0.0287	0.0687	0.0678	0.0025	0.0852	
0.0592	0.0243	0.0143	0.2288	0.13	0.027	0	0	
0.0245	0.0097	0.1652	0.2245	0.105	0.1534	0.1132	0	

0.0037	0.1998	0.2574	0.0773	0.1289	0.0952	0	0	0
	0	0	0.1556	0.1108	0.0279	0.0419	0.0794	
0.0692	0.0512							
AXIN1	3.7015	3.9356	3.9409	2.9202	2.804	3.2602	3.4432	
3.9222	2.7619	3.3313	3.5877	2.3312	4.1635	2.7023	4.0859	
3.0333	3.5674	2.3845	3.8	3.6647	3.5643	3.912	2.7467	
3.6957	3.5073	3.1078	2.875	3.9397	3.2451	3.3005	2.7619	
4.0134	3.3858	3.2389	2.7445	2.6569	3.9615	3.736	3.6383	
2.8151	3.7324	1.8995	2.7258	2.7737	3.4049	3.0571	3.2705	
3.6394	3.9124	2.9628	3.4079	2.4362	2.8823	4.4966	2.9732	
3.8109	3.2449	4.1677	3.3121	4.2606	2.4733	3.5355	3.5422	
3.2899	3.8243	3.2145	3.2702	4.0286	4.1359	1.9463	3.0891	
3.5698	3.3606	2.5413	2.9687	3.3756	3.9067	3.0444	3.2687	
3.3552	3.4954	2.709	4.357	3.1316	3.5282	3.3877	2.9433	
3.4238	3.2852							
AXIN2	1.7801	2.8487	4.9092	0.1829	0.3866	0.9058	4.4878	
0.7573	0.4369	1.8516	0.9857	0.4099	0.4671	1.2053	1.5444	
1.9299	0.7712	2.2695	0.4844	1.8425	1.2711	0.9895	0.7206	
0.7279	1.2024	0.9667	1.2483	5.4906	0.9455	3.6212	0.8367	
0.7022	0.8391	0.4788	1.481	1.3567	0.622	1.8347	1.6161	
1.164	0.7793	0.3278	2.0007	0.1788	1.9981	1.0681	4.6378	
1.868	0.3849	1.6631	0.8845	0.2956	0.761	0.751	0.9449	
1.6175	0.6076	1.0868	1.7579	1.7708	0.7888	0.6441	0.6809	
1.5143	0.9731	0.9786	1.5314	6.2917	4.8411	0.8215	0.5899	
0.4501	1.1666	0.4017	1.52	3.5683	1.9349	0.8063	0.3814	
1.5405	1.9144	0.3439	0.7689	1.36	1.2702	0.7142	1.4452	
1.2371	1.9184							
AXL	2.4146	0.0403	0.1298	4.8614	5.7205	0.9138	0.0729	
3.397	0.095	0.0858	0.1269	5.4875	7.4181	1.1873	1.8243	
0.1087	7.136	1.0004	1.1062	1.4204	3.8729	6.3906	6.0389	
0.3675	5.5042	7.1755	5.291	0.2481	6.0988	0.0531	2.2511	
2.862	1.1048	0.3252	4.0455	1.2112	5.7932	0.0853	2.3518	
1.0295	0.1287	4.9827	6.1525	2.2818	7.0787	5.339	0.3151	
0.4252	6.7653	8.7917	5.0509	5.3832	0.7625	0.7118	3.311	
0.2296	7.4229	3.2964	0.3424	3.211	5.8769	0.9455	3.8446	
0.1602	0.199	5.7735	0.1782	3.3433	0.1963	7.128	2.7917	
4.3978	0.3073	5.4696	4.2928	0.1538	0.3647	4.1544	0.2538	
0.41	0.1485	6.2972	3.4653	6.455	7.6967	1.5065	2.8573	
1.2921	2.5179							
AZGP1	1.3197	2.0401	0.2918	0.6449	2.2858	1.9593	3.2272	
1.5897	1.1535	0.505	1.3818	1.1763	0.2513	2.6595	3.6139	
1.0194	0.8261	1.3431	1.2103	2.0081	0.855	4.7368	4.756	
1.4725	4.1824	0.2168	5.6002	0.156	5.4618	0.6112	0.3555	
4.2862	2.7705	3.3713	3.4271	0.6354	3.4523	1.7596	3.6808	
3.2722	4.1868	0.7643	2.4498	2.3499	0.3108	1.0548	0.8524	
0.7016	2.9537	2.461	2.5478	5.5693	1.525	4.5842	0	
1.1489	1.4614	2.7834	1.0833	2.5407	2.8164	2.4246	3.3289	
1.2676	1.5569	2.4383	0.8889	1.0891	0.8475	0.596	1.3646	
1.8212	1.8979	5.1161	0.6051	0.0639	1.4652	1.5105	2.4695	
3.7457	1.6992	4.1053	1.8069	3.356	3.8973	1.9881	1.7879	

1.1341	1.0904						
AZI2	4.0751	4.3928	4.3045	4.3815	4.2299	4.2923	5.071
4.4923	3.7523	3.9758	4.4902	5.3005	4.2169	4.2598	3.4921
5.0266	4.8823	4.2919	4.3274	3.9601	4.6223	4.0901	4.4151
3.7066	3.9471	4.2639	4.4937	4.4673	3.9849	4.1884	5.1858
4.243	4.1476	4.0535	4.2402	4.344	4.8334	4.6665	4.4009
4.0059	4.4229	4.3893	4.0984	3.8892	4.9455	3.7214	5.0071
4.2809	4.743	5.2533	4.453	4.6475	4.4772	4.9285	4.4581
3.6784	4.6907	3.7064	4.2639	4.0155	4.1811	4.0974	4.1742
4.2812	4.0934	4.9375	4.9685	4.5921	3.9829	3.9308	4.356
4.0953	4.5593	4.5757	4.7529	4.0542	4.168	4.3464	3.4828
4.063	4.3268	4.668	4.3141	3.8838	4.2761	4.5958	4.4755
4.3334	4.4133						
AZIN1	6.0907	4.839	5.9626	6.5954	6.1012	6.7494	5.9501
5.6301	5.7269	5.7983	5.3864	5.7124	6.2687	6.2189	7.0318
5.4817	5.9109	6.3285	5.8457	5.4768	5.6969	5.8318	5.8792
5.5362	6.2487	5.4125	5.1347	6.0589	5.5954	5.0208	5.8987
5.8185	5.9191	6.4072	6.1435	6.1328	5.9594	5.7944	5.7085
6.0162	5.7691	5.8196	7.2065	5.7742	6.3159	6.7391	6.0356
5.7233	5.8104	6.4005	5.943	5.7844	6.2259	6.1714	5.9755
5.3048	6.0942	6.5173	6.3221	6.1838	5.8682	5.4834	6.3546
5.3176	7.2166	5.1229	6.1606	6.1375	5.1255	6.2058	6.1052
5.6203	5.3444	5.5576	6.0849	6.0906	5.892	6.2377	6.1464
5.642	5.6873	5.7234	5.4073	5.3912	5.8014	5.8396	5.4869
5.6649	5.4262						
AZU1	0.1889	0	0	0.0927	0.1107	0.3064	0.0349
0.1076	0.1523	0.0822	0.0784	0	0.1103	0.2232	0.0291
0.0607	0	0	0.1338	0	0.1204	0.0483	0.0138
0.1117	0.0494	0.0657	0.0551	0.1432	0.0363	0.1041	0.2699
0.0054	0.1088	0.0457	0.0099	0.0687	0	0	0.0804
0.0505	0.0919	0.172	0	0.0469	0.0669	0.1241	0.1472
0.0536	0.0916	0.137	0.0783	0.0513	0	0.0296	0.2386
0.0971	0.2433	0.0448	0.0508	0	0.0923	0.1036	0.1362
0.1118	0	0.0113	0	0.0629	0	0	0.0704
0.1525	0	0.0757	0.0628	0.0382	0.0891	0.0404	0.0227
0.1409	0.1536	0	0.0637	0.0416	0	0	0.0544
0.0544	0.1133						
B2M	9.0494	8.6735	8.4795	9.1985	9.183	9.6496	12.1099
8.3923	10.5352	9.1163	9.2268	9.4292	11.1819	9.3792	10.1099
9.8507	8.7463	9.8853	9.183	9.1017	9.2437	10.0327	10.7881
9.4129	9.9587	10.6353	9.5897	9.5256	9.5097	8.6519	9.183
9.4129	8.4495	11.2844	11.4493	9.2706	9.9385	9.7817	8.1971
9.6053	10.1325	11.1819	10.6353	9.3792	11.1819	10.1325	9.4129
10.1099	10.3984	9.4763	11.6716	10.6353	9.0753	9.3431	8.7948
9.2015	9.9871	8.2975	8.3256	8.3912	9.6878	8.304	8.7492
10.2811	8.4679	12.1792	8.9037	10.4974	9.614	9.8547	9.227
9.3151	9.5204	10.6186	10.1005	9.6716	9.2658	10.7961	9.6558
8.5698	9.2398	10.2119	10.8882	10.6443	10.5555	9.4651	10.3645
9.9034	9.7122						
B3GALNT1	0	0.8842	0	3.1241	0.0086	0.0441	4.2313

0.0441	3.464	1.8981	1.2476	2.1968	1.7162	1.8851	1.141	
0.5993	0	0.0548	3.0101	0.0334	0.9761	3.3856	1.6143	0
	3.5305	1.9794	0.8693	0.0391	0.9322	1.427	0	
0.275	3.2845	2.6889	0.1303	1.3074	2.2314	0.9842	1.6075	
0.0234	0.9272	3.7611	0.0568	1.6751	2.2265	4.654	1.5296	
0.0244	1.32	0	1.8883	2.8744	0.5066	3.501	0.0239	
4.139	4.0256	0	3.3159	0.0225	0	0.0516	0.5083	
1.6409	0.6272	2.6021	0.0707	1.7961	2.1569	0.5769	0.304	
2.4649	0.0603	0.054	1.4462	4.6366	2.0439	0.0197	1.859	0
	2.2552	1.2866	1.8488	1.6454	2.8281	0.1737	3.2747	
3.9177	3.4202							
B3GALNT2	4.3983	3.3664	3.6499	4.2028	3.7834	3.3637	3.2199	
3.1577	2.6701	3.0387	2.6072	3.2937	2.8827	3.4301	3.3902	
2.8772	3.3713	3.709	3.2877	3.0826	3.2356	3.0711	3.731	
3.4874	4.3612	3.487	3.1758	3.4776	3.6973	4.1104	3.7434	
2.9421	2.6719	2.807	3.1472	3.2695	3.6663	3.0068	4.3112	
4.1436	3.5653	3.4036	3.6806	3.2308	3.8006	3.6319	3.5033	
4.6918	4.0982	3.6198	3.5725	3.9595	3.7927	3.521	4.0829	
3.9484	3.2375	4.049	4.1405	3.886	3.5668	3.4119	3.978	
3.227	3.7543	2.9013	3.8818	2.6883	3.1022	3.7518	2.9196	
3.3736	3.5904	3.4765	3.7808	2.9659	3.4469	3.7644	3.1827	
2.11	3.3654	3.0433	2.0814	4.0506	3.5377	3.5325	2.9302	
3.0103	2.9221							
B3GALT1	0.3844	0.0552	0.9207	0.3543	0.1984	1.2685	1.4815	
1.169	0.7355	0.0377	0.3572	0.2324	0.1916	0.7619	1.0171	
1.2377	1.6523	0.3434	0.1735	1.4516	0.6671	0.8541	0.4232	
1.1493	1.6484	0.1565	0.9776	1.3545	0.9336	0.3741	0.0149	
1.048	0.8103	1.8394	1.2082	0	0.5456	0.9303	1.7051	
1.9513	0.8242	0.055	1.3621	0.8617	0.4384	0.3455	0.4164	
1.8866	0.5662	0.6822	0.5083	0.6755	1.3794	0.1755	0	
0.8695	0.1627	0.2707	0.4543	0.5202	0.8081	0.1396	1.3329	
0.847	0.8773	0.3862	1.9536	0.4283	1.3248	0.5697	2.1905	
0.6131	0.4523	0.6362	1.6039	1.5817	0.7334	1.6698	1.5008	
1.3097	1.0368	0.87	1.0795	1.3707	0.5171	0.9843	0.3729	
0.7306	0.8078							
B3GALT2	0.5329	0	0.1332	0.9052	0.1748	1.2125	0	
1.0505	0.6727	0.1204	0	0.1061	0.0624	0.3348	0.6758	
0.1329	0.1872	0	0.2977	0.394	1.2197	0.4016	1.6179	
0.4831	0.5485	0.5317	0.1706	0.0711	0.6456	0.026	0	
0.6586	0.0137	0.0987	2.8382	0.0504	0.5372	0.5808	1.1812	
0.8023	2.44	0.0291	0.1061	0.2735	1.1951	0	0.2524	
0.2716	1.0857	0.1985	0.1473	0.773	0.7019	0.0992	0	0
	0	0.249	0.1137	0.1859	1.4914	0.0599	0.73	
0.1031	0.3205	0.1051	0.7176	0.0656	0.1101	2.6556	0.1502	
0.1852	0.523	0.2272	0.9334	0.0847	0.1364	2.764	0.0923	
0.1258	0.1093	1.0439	0.0615	0.9434	1.3406	1.0361	0.0552	
0.1269	0.0268							
B3GALT4	0.8051	0.5247	0.9895	0.4805	0.4013	0.2808	0	
0.808	1.3636	1.5643	0.7056	0.2566	0	0.3707	0.2963	
0.2777	1.2238	1.1992	0.6869	0	0.824	1.0023	0.947	0

	1.7353	0.5053	0.7718	1.8615	0.3515	0.1134	1.4319	
0.1226	0.0652	1.2564	0.2904	0.6891	0.8603	2.0711	1.0391	
0.0716	0.7215	1.0479	1.389	0.8937	0.6822	0.9762	0.177	
0.3328	0.6704	0.3038	1.3457	0.5888	0.4907	0.229	0.8188	
0.8064	1.1276	0	0.2557	0.0237	0.3257	0.6256	1.7339	
0.0998	0.7091	1.147	1.7899	1.2314	0.2904	0.6298	0.0554	
0.5203	0.801	0.8227	0.7966	0.3224	0.6134	1.0762	0.733	
0.7361	0.994	0.9726	2.241	0.7197	2.7634	1.1519	2.787	
1.8706	2.6555							
B3GALT5	0.0849	0.1094	0	0	0.6551	0.4346	1.221	0
	0.1942	0	0.029	0.405	1.3939	0	0.2664	
0.6686	2.0151	0	0	0.7961	0.065	0.0943	0.0912	
0.1914	0.1964	0.9002	0.3364	0	0.2645	0	0	
0.4719	0.1036	0	0.0777	0.02	2.4906	0	0	0
	0	0.1887	1.2965	0	0.2491	1.6575	0	
0.1925	0.1047	0.7378	0.0796	0.0282	0.0878	0	0	0
	0.8451	0	0.6235	0.0518	0.006	0.36	0	
0.0671	0.0087	0.8355	0.0187	0.7067	0	0.9653	0.1367	
0.2513	0.0269	0.0613	0.5276	0	0	0.9684	0.0515	0
	0.0349	0.1706	0.0331	0	0.1956	0.3157	0.0338	
0.1168	0							
B3GALT6	3.4594	3.5563	4.7406	4.0781	3.8219	3.0909	3.0154	
3.5205	2.9953	1.7193	2.7826	3.096	3.0442	3.0118	3.5694	
1.6142	2.7433	3.5345	3.8168	3.5265	3.9925	3.1922	4.0574	
3.6985	3.8765	4.3535	1.4967	3.027	3.9724	3.1531	4.7235	
3.3651	3.9786	3.1489	3.7272	2.653	4.0346	3.6945	4.192	
3.4363	3.5563	3.4575	4.0765	2.7472	4.2191	3.9562	2.8001	
3.2729	3.8773	3.4097	3.856	3.997	4.6126	4.3275	4.0034	
4.0775	2.8331	4.6581	2.8263	5.0072	3.2858	2.3393	3.8603	
3.6645	3.6062	3.8129	3.6766	3.6797	3.8069	3.6827	3.2778	
3.7033	3.7356	3.4868	2.3982	2.5701	2.9273	2.8271	3.686	
3.259	3.611	3.1652	3.2703	4.1132	4.6467	4.9719	3.2169	
3.3346	3.2513							
B3GAT1	1.8503	1.2272	3.5932	2.3757	3.0005	0.0675	1.1858	
0.5466	1.959	0.2318	3.8114	0.0581	2.6919	0.0812	1.3319	
4.7892	0	3.0561	2.2385	4.6737	3.0362	0.9355	3.0292	
2.1986	1.2974	0.044	2.4658	0	2.8441	2.7165	0.2884	0
	0.1523	0.715	1.5886	0.9944	0.0759	0.3016	2.5471	
1.9303	0.8515	0	4.2276	0	0.8374	0.0725	0	
0.9936	1.073	0	0.3586	0	0.2429	3.6526	0	
2.6145	0	5.4614	2.1123	5.3302	1.1376	1.7781	3.2531	
0.4551	0.8317	0.4457	0	1.8787	2.0805	2.8009	0	
0.1986	3.3464	1.4152	1.1517	0.0116	0	0.6018	1.6894	
2.2611	1.8192	0.0441	0.0679	1.9283	2.2386	0.0994	0.4551	
1.5797	0.4051							
B3GAT2	1.3538	2.0564	1.5649	2.2063	0.9593	1.8452	1.062	
1.7119	1.339	1.4839	1.6302	2.1671	1.1118	2.5504	2.1853	
0.8503	2.3394	1.726	3.0314	1.5439	1.7433	1.7583	2.2407	
2.2209	1.4796	1.1286	2.8435	1.3132	1.174	2.1557	1.9077	
1.8238	1.4018	1.6237	2.2701	2.1903	1.6646	1.485	1.3751	

1.6019	1.0226	1.9172	1.7391	1.5017	1.7089	1.4277	2.1033	
1.2976	0.9535	2.6621	1.9122	1.8132	1.8414	1.8664	2.1108	
1.7903	1.6826	1.2918	1.6964	2.1782	0.9	1.7694	1.235	
1.4317	2.0497	0.8956	1.3415	1.7645	1.7216	1.4205	1.2093	
1.305	1.241	0.9464	1.0485	1.4367	2.0432	1.7158	1.478	
2.3426	1.4509	1.4035	2.4178	1.0386	1.5308	2.2569	2.1274	
1.1968	1.5296							
B3GAT3	4.4407	5.4154	5.2038	3.7185	5.7212	4.7372	4.3621	
5.2461	3.5694	3.9421	4.7721	4.2246	4.1759	3.4351	4.539	
4.6261	4.5059	3.205	3.4063	4.9819	4.3186	5.9128	5.3012	
5.0427	4.5325	3.5345	4.6689	4.6922	5.0926	4.5272	4.4007	
5.253	4.9209	5.9107	5.4964	3.2122	5.3369	5.7786	4.9115	
3.5052	5.812	4.8358	4.7269	4.9279	3.9037	3.695	3.9446	
4.7403	4.9747	2.8026	5.0544	3.7962	3.5704	5.4923	4.6652	
4.9263	3.2026	5.7266	4.8084	4.9206	4.8453	4.274	5.0785	
4.0292	5.0716	5.6442	5.3325	5.613	3.4658	4.0082	4.6239	
4.683	4.2913	4.9939	4.0871	5.0478	5.455	5.7989	4.6709	
5.719	3.2953	4.2943	5.5324	4.7608	4.8421	3.9389	5.0087	
5.2077	5.2856							
B3GNT2	3.6934	3.6943	3.5816	4.1214	3.4867	4.4524	4.6908	
3.0509	4.998	4.4309	4.4745	4.1386	3.6623	4.811	3.7293	
4.8062	3.5995	5.3143	3.9283	3.8198	3.7281	3.7348	4.5093	
4.766	3.2912	2.8595	4.2059	4.1066	4.6031	4.2907	3.8854	
3.7837	4.74	5.0921	4.06	4.1836	3.2204	3.484	2.7477	
4.0816	4.9402	3.6003	3.8562	4.6982	4.1088	3.6541	4.7512	
4.7775	3.685	3.1268	4.1106	3.8698	4.8466	3.8113	3.6369	
3.024	3.1778	3.4303	4.7081	3.6242	3.9883	4.5558	2.9322	
4.4597	4.0588	3.9188	5.1593	4.7837	5.0399	3.3051	3.9004	
4.3402	3.4296	4.0005	3.5956	5.2459	4.1285	3.4448	4.167	
4.7686	4.516	3.8221	4.8047	3.6309	3.0824	4.4455	4.2494	
4.0613	3.9957							
B3GNT3	0.0441	0.0256	0.0385	0.4187	0	0	0.0126	
0.0156	0.0158	0.298	0.156	0.5819	0	0	0.0442	0
	1.4371	0.0345	0.0601	0	0	0.3623	0.0064	0
	0	0.0145	0.0476	0.0433	0.3175	0	0	
0.2226	0.1894	0.0428	0.0122	0.1099	0.3575	0.7294	0.0318	
0.0208	0.0656	1.8681	0	1.2894	0.0283	0.0828	0.0356	
0.0754	0.0157	0	0	1.2548	0.0494	0.1227	0	0
	1.1723	0	0.0261	0	0	0.034	0.0394	
0.0692	0	0.0085	0.0416	0	0.0078	0	0.5028	
0.5572	0	0.0881	0	0	0.0029	0.0191	0.4702	
0.0415	0.1478	0.133	0.271	0	0.093	0	0	
0.0798	0.0651							
B3GNT4	0.1246	0.5059	0.1919	0.1201	0.4509	0.1096	0.5391	
0.1471	0.0641	0.5482	0.1675	0.0463	0.7498	0.257	0.0581	
0.299	0.3315	0.4803	0.2963	0.3975	0.1001	0.1829	0.3355	
0.8924	0.2169	0.4889	0.4214	0.1934	0.066	0.1943	1.0434	
0.5059	0.2816	0.0768	0	0.2305	0.0844	0.3411	0	
0.079	0.859	0.5533	0.5923	0.1321	0.3312	0.6286	0.1066	
0.1371	0.4223	0	0.244	0	0	0.1237	1.0347	

0.8171	0.0681	0.4317	0.3931	0.3185	0.5976	0.143	0.1538	
0.3307	0.6448	0.1842	0.375	0.3207	0.0958	0.2752	0.2654	
0.2017	0.3554	0.2175	0.2295	0.1579	0.4172	0.397	0	
0.135	0.2501	0.4294	0.0305	0.0563	0.1305	0.0891	0.4169	
0.0893	0.0999							
B3GNT5	1.878	0.0652	2.8666	3.4578	2.7655	3.085	3.0191	
2.3771	2.1693	2.4515	1.8093	2.2914	2.1034	2.0927	2.7969	
2.6304	1.6393	2.1633	2.6384	2.1598	2.1392	3.2016	2.1917	
4.5598	3.296	1.3996	3.6296	2.4075	3.3981	0.347	1.4279	
0.9638	3.6642	2.5276	2.4939	2.0727	1.8217	0	1.7262	
4.2067	3.23	2.9059	4.1559	2.6223	3.0549	3.1392	1.9397	
2.8282	2.7225	0	2.799	3.2032	2.2909	0.7194	2.9419	
3.4137	4.4011	3.5866	2.1037	3.9942	2.9818	2.1338	2.9289	
3.4907	1.7336	1.1001	0.38	2.3385	2.922	2.846	3.2099	
2.7091	0.4412	1.5911	2.5946	1.8883	2.6467	1.9396	2.0763	
2.7755	1.6051	1.8315	3.1707	2.8688	3.4393	2.2616	2.053	
2.3101	1.9872							
B3GNT6	0.0714	0	0	0.072	0.0092	0.0589	0	
0.0268	0.0531	0.0295	0.0159	0	0.0186	0.0858	0	
0.0185	0	0.0551	0	0	0	0.013	0	0
	0.0381	0.0231	0.0419	0	0.0279	0	0	0
	0.0705	0.0347	0.0054	0	0.0139	0.0399	0.0259	
0.0805	0	0.0237	0.0522	0	0.0503	0.0372	0	0
	0.023	0	0.0612	0	0	0.0168	0.0321	
0.0659	0	0	0	0	0.038	0	0.018	0
	0	0.0042	0.0432	0	0	0	0.0871	
0.0183	0.0563	0	0	0.0211	0.0097	0	0.0103	
0.0473	0	0	0.017	0	0.0753	0.0765	0	0
	0							
B3GNT7	1.2358	0.1363	0.0044	0.1239	2.7014	3.3191	0.0194	
5.0281	0.2005	1.2358	0.4763	1.579	0.1097	1.8089	1.6093	
3.4832	0.1495	1.0419	0.0946	0.0464	0.0922	4.2335	0.0747	
5.8132	2.5525	0.1359	2.1561	0.0466	3.1364	0.2346	0.3028	
1.0026	0.062	0.664	1.6243	0.8277	0.3314	0.9228	2.7883	
1.4885	1.3357	0.8797	2.6484	5.4608	2.1481	0.1517	0.0742	
1.6251	3.4589	0.1607	0.4699	2.6618	0.1035	0.4587	0.2547	
0.1152	0.6003	5.7583	0.1003	5.3463	3.4054	0.0686	0.2934	
1.5645	0	0.2539	0.4699	0.0913	0.2458	4.3326	3.7288	
1.4562	0.0798	3.435	3.671	2.8321	0.2383	1.9266	0.1394	
0.1891	0.1276	3.0079	0.37	2.8213	3.4037	0.1207	0.091	0
	0.087							
B3GNT8	0.3057	0	0	0	0.0366	0	0	
0.0413	0.0769	0.2272	0	0	0.0301	0	0.074	
0.0303	0.3462	0.2692	0	0	0	0.0245	0	0
	0.0525	0	0	0.1448	0	0	0.0293	
0.009	0.0488	0	0	0.0827	0	0.0522	0	0
	0	0.2349	0	0	0.0364	0.0538	0	
0.0544	0.0335	0	0	0	0	0	0.0524	0
	0	0.0959	0	0.0925	0	0.0715	0.0273	
0.0609	0.1277	0.2057	0	0.1549	0.2704	0	0.0795	0

	0	0.0494	0	0.0308	0.0114	0.0378	0.0125	
0.3599	0	0.0496	0.0233	0.0413	0.1346	0.1458	0.3662	
0.5585	0.653							
B3GNT9	1.3968	1.4716	2.0238	1.3956	0.8121	1.7429	2.5528	
2.4918	1.0313	1.2468	1.2431	2.1701	3.9163	3.1078	2.6819	
1.9576	1.8967	1.7287	1.417	2.3522	2.0356	3.074	3.9543	
1.6843	2.5734	4.28	1.7999	1.4589	2.9631	1.7349	2.5739	
1.0946	1.2607	1.049	1.3838	1.5693	2.5611	1.4041	1.1188	
1.1867	2.702	4.9573	1.1704	2.1834	4.0513	3.9654	2.2146	
2.1701	3.2959	2.6281	3.1918	4.0251	2.6273	1.7077	2.2106	
1.2636	1.6846	1.9819	1.7962	1.7302	1.15	1.5499	1.3667	
1.3339	1.224	5.0355	1.2863	1.7999	2.3095	3.3703	1.9349	
3.0974	1.621	3.4775	1.3821	3.052	1.4491	1.736	2.1013	
1.1819	1.1161	3.2206	3.1294	3.3399	4.0336	3.2535	2.4611	
1.8738	2.057							
B3GNTL1	2.924	1.7701	2.0522	1.2874	2.8944	2.4605	1.4281	
2.2188	1.6475	1.4663	1.8798	1.3118	2.709	1.2421	1.3038	
1.9325	4.691	1.8904	1.9948	1.5215	1.2817	1.4526	1.8539	
2.1581	1.9545	1.6052	1.2987	2.1208	1.7587	1.4743	1.9058	
3.1662	1.6944	1.9213	2.2742	1.2977	2.6461	1.8363	1.5144	
1.6052	1.29	3.192	2.9498	1.8675	1.8687	2.5619	1.7797	
2.29	2.3904	2.3616	1.6287	1.5038	1.5782	2.3096	3.0485	
2.0539	3.4575	1.7946	1.8927	1.8152	2.0888	2.1067	2.2813	
1.1444	2.9017	0.5121	2.8678	1.7901	1.4549	3.0515	1.7385	
1.2671	1.8674	1.872	2.4448	2.2532	1.9877	2.1989	1.4503	
1.4287	2.0215	1.653	2.0625	2.0882	2.0011	1.3858	1.4465	
1.6838	1.3857							
B4GALNT1	1.9816	0.4075	1.1753	2.7866	4.4936	3.582	1.1848	
3.9292	1.0818	1.607	0.6875	1.9727	3.7187	3.0873	3.321	
1.7776	2.2296	0.7608	0.4317	0.5611	1.2838	0.1025	0.2603	
3.4604	5.3065	5.7956	1.0989	1.8148	3.1264	0.8014	5.5721	
1.1312	0.4418	3.104	2.3081	2.7261	4.0471	0.556	2.4854	
0.7183	0.9648	3.2322	5.6591	3.3248	4.3152	1.3496	0.886	
1.0616	3.7707	1.631	2.1332	1.0043	0.8848	0.8575	5.1787	
0.8608	1.1099	4.5497	0.5964	4.2351	3.9394	0.44	3.3715	
4.5176	3.1	2.6831	3.7638	3.0207	0.542	2.5731	2.7791	
0.6112	0.9488	2.2906	1.1475	4.3959	0.1844	3.5101	0.3762	
0.2877	1.6402	0.515	3.0937	3.8408	3.7504	1.368	2.3317	
1.7466	1.7018							
B4GALNT2	0	0	0	0	0	0	0.0116	0
	0	0	0	0.0304	0.0273	0.046	0	0
	0	0	0	0	0	0	0.0048	0
	0	0.1923	0	0	0	0	0.0692	0
	0	0	0	0	0	0	0	0
	0	0.0021	0	0	0.0112	0	0	0
	0	0.3171	0	0.0239	0	0	0.0759	0
	0	0	0	0	0	0	0	0
	0	0	0	0	0	0	0	0
	0	0.0079	0	0	0	0	0	0
	0	0	0	0	0	0	0	0

	0							
B4GALNT3	1.7182	0.9239	1.5763	1.8824	1.3495	1.6009	2.4524	
2.7492	2.0443	0.7354	1.5313	2.0877	0.8893	0.7244	1.3133	
3.0623	0.1283	2.6866	1.7402	0.333	0	2.6485	4.3909	
1.387	1.9287	0.1595	2.5085	1.0386	2.2657	1.9071	1.1158	
3.2684	0.9268	0.4634	5.0634	0.6897	1.5752	2.5389	0.8031	
2.7205	0.8326	0.154	3.1206	0.954	1.4584	2.311	1.4442	
2.536	1.2942	0.3898	3.2623	0.2637	3.0073	0.4316	0.5015	
1.4626	0.1097	2.9854	1.9108	2.634	1.5912	1.8452	3.3382	
1.2785	1.853	0.23	0	1.2111	1.7336	1.7728	1.0078	
1.2053	0.9164	2.7135	2.996	4.6344	0.1321	3.9035	0.8721	
2.5301	1.7956	1.2624	2.8544	3.284	1.1184	2.7629	0.1935	
0.6319	0.2571							
B4GALNT4	0.4968	0.2295	0.6519	3.3555	2.7928	3.2264	1.9763	
1.1646	0.7644	0.2499	0.9022	0.5736	2.3133	1.3212	3.7732	
0.7375	0	0	0.2966	0.6717	1.9867	2.3759	1.0108	
0.2129	4.5089	1.9067	3.211	0.9618	2.2893	0.3622	3.8104	
0.6857	0.6695	0.379	0.7894	0.5867	0.5031	3.2152	3.1967	
2.7078	0.959	1.0103	3.547	0.8541	2.5363	1.7968	1.0299	
2.0525	2.7693	0.3824	3.21	0.9812	2.7889	2.8717	1.2076	
3.5437	0	3.0334	0	3.0551	2.1938	0	1.9177	0
	2.8621	2.3455	1.8166	2.6066	0.1588	1.7725	2.3791	
0.2102	1.8566	2.1562	1.7502	0	0	2.53	2.7096	
3.4087	2.0507	3.7066	0.7681	3.0084	3.2067	2.6062	1.2944	
0.7473	1.0949							
B4GALT1	4.8408	3.3361	3.7273	5.7261	5.1286	4.5582	4.1268	
3.9463	4.6942	5.983	5.0025	5.6446	6.3153	5.8839	6.2066	
5.4378	5.7976	5.2648	4.3659	5.4142	3.7302	4.3834	4.1086	
4.0636	4.0022	4.9504	4.4333	3.7281	4.019	3.0961	5.3588	
3.8158	4.2048	5.16	3.9572	5.0976	3.5322	4.6933	4.5184	
4.006	5.0889	5.7349	6.604	5.329	4.8432	5.5122	5.0731	
3.0902	4.5929	4.9497	4.5148	4.8073	4.4702	5.2038	5.2444	
5.0838	5.6921	4.4177	5.5595	5.1696	4.1707	5.95	3.4257	
5.0479	3.7554	7.4954	4.4556	4.9151	4.0116	4.8405	4.6761	
5.3513	3.7472	5.1007	3.98	2.9267	3.8039	4.6246	4.5124	
4.257	3.9723	5.7704	3.6867	3.9831	4.5021	4.1275	5.3737	
4.1071	4.4269							
B4GALT2	4.5465	4.6488	4.5601	4.128	4.7914	4.1647	4.3001	
4.5374	4.9357	4.6996	5.4447	4.1988	4.7619	3.8071	4.5273	
4.3062	4.9451	3.4932	4.1494	5.0069	4.5624	5.1798	4.6387	
5.022	4.8713	5.1877	4.214	4.5942	4.8622	4.7183	4.6939	
4.3632	4.6742	5.0735	4.8115	4.0821	4.3238	4.5619	4.7628	
4.5642	4.7848	4.7478	5.2704	4.6646	4.7011	4.1697	3.6481	
4.4581	5.4115	4.1423	3.6518	4.1242	3.9806	4.5234	4.8224	
5.0703	4.2557	5.1642	4.672	5.3576	4.8147	4.83	4.7145	
4.8236	4.9606	4.4426	4.4026	4.4092	4.4163	4.5369	4.9384	
4.9329	4.5531	4.6035	4.6087	3.4834	4.3392	4.2464	4.6965	
5.3817	4.6665	4.5263	3.6386	3.9005	4.8409	4.5633	4.4435	
4.7681	4.431							
B4GALT3	4.316	4.8274	4.4106	4.9504	5.0568	5.8519	4.4645	

4.8955	4.3981	4.5723	5.3188	4.9457	3.2436	3.5158	4.4823
4.8517	4.1273	4.6748	3.9464	4.4614	4.1761	5.1492	4.4564
4.7193	4.4997	4.3611	4.8237	4.5665	4.6105	4.9131	5.5335
4.7235	4.0466	5.0607	3.5406	4.4773	4.7511	5.0204	4.6191
4.2085	5.1662	4.4327	4.9558	3.5316	4.4683	5.1601	4.7755
4.4207	3.9449	3.369	4.1602	4.8694	4.6859	4.4561	5.4149
4.5589	3.9209	4.9198	4.7158	5.2045	4.4684	4.506	5.1368
3.6533	5.0418	4.6462	4.9656	4.0134	4.4832	4.7148	3.9006
4.4045	4.2582	4.1511	4.6655	4.2228	4.6863	5.8119	4.2656
5.0955	4.1345	3.6497	4.4261	5.1428	4.8649	4.5152	4.289
4.0885	4.5418						
B4GALT4	3.485	3.2002	3.7168	3.6675	3.2623	3.8534	4.1268
4.2182	4.8633	3.752	4.8666	4.4198	3.348	3.9115	4.3538
4.6321	3.4031	3.8287	4.1389	3.6365	3.6847	4.3919	4.0186
3.7124	3.4818	4.0616	5.1941	3.9285	3.7207	3.6981	3.677
3.9206	4.5064	5.6021	4.1259	3.9321	3.366	3.4357	3.1014
4.9481	3.3922	3.6031	4.9611	3.8038	4.1886	3.6694	4.1989
3.8069	3.0181	4.0756	4.3316	4.2778	3.8677	3.7292	3.4638
4.1899	3.4418	3.409	3.3298	3.0733	3.9591	3.6361	2.8187
3.842	3.2214	4.5066	4.9922	3.9724	4.1683	3.5426	4.1158
4.2542	3.5844	4.1912	3.6203	4.11	3.9448	4.359	3.9694
5.0597	4.2778	3.3833	3.2099	4.8157	3.221	3.9974	3.451
3.55	3.2356						
B4GALT5	5.1969	6.025	6.2138	4.8749	5.3131	6.145	6.5773
5.7065	5.9431	6.4376	5.6599	6.3768	5.3075	5.74	4.8038
6.1795	4.9513	6.4569	5.6671	4.3926	5.1384	5.6724	5.317
5.428	6.1538	5.568	5.7254	6.1894	5.4211	5.9637	4.983
5.3664	5.6162	6.6095	5.0764	6.2288	4.2866	4.2274	5.7035
4.9285	4.8846	5.317	4.899	5.6569	7.3229	4.8301	6.7074
5.5208	6.22	6.1102	5.7114	5.5376	6.0927	5.3469	4.8848
5.2059	5.4193	5.3904	5.7542	5.9055	6.1659	6.2246	5.1587
5.5618	4.3018	4.7272	4.2251	5.7098	6.4668	4.8243	6.0422
6.4551	5.9915	5.9275	6.3317	7.8274	5.9387	5.573	5.1807
5.9266	5.7861	5.7091	4.335	5.7279	5.4447	5.3136	4.7451
5.4459	5.7654						
B4GALT6	2.5665	2.2708	3.3521	3.896	4.3868	5.4947	4.5676
4.7556	4.3735	3.5373	3.2143	3.4227	1.4778	3.8177	5.3678
4.7322	4.8633	3.6691	4.5642	4.2071	3.9759	5.3387	4.8176
5.0641	4.8793	2.7748	3.3085	3.0923	4.289	2.7739	3.2169
3.672	4.9668	3.9581	5.2601	3.8891	3.0984	1.7735	5.1159
4.2197	4.2826	3.1264	5.48	4.538	3.0071	1.4021	2.7517
4.7628	4.2341	0.3137	1.988	4.3315	4.3885	1.9419	3.2523
1.8831	4.2443	2.1511	3.2155	2.2084	4.6334	3.1606	4.9963
4.5576	3.5045	2.913	5.9725	3.3396	2.7706	3.9427	4.8227
5.3139	3.2409	4.9507	5.3146	3.8582	2.8859	3.229	4.5571
4.7488	4.1889	4.8032	5.0201	5.3132	3.3536	4.1649	2.7578
3.2929	3.2797						
B4GALT7	3.4729	2.735	3.9184	2.3186	3.785	2.2517	2.9957
3.6155	3.3748	2.8802	3.3903	3.2912	3.4875	1.9483	4.3988
2.95	3.2889	2.1449	2.7269	3.6396	3.1627	4.1056	4.1603

3.6429	2.9786	4.2202	2.405	3.1662	3.5554	2.5287	4.3732
4.513	3.7759	4.8543	4.235	2.1848	3.8809	4.2647	3.0536
2.6514	3.7876	3.4394	4.2705	3.1728	2.7385	3.3099	2.5498
3.1549	4.0261	2.1337	3.6225	2.7296	2.4642	3.341	3.3448
3.2245	3.7527	3.0586	2.1893	3.1002	3.2888	4.1282	3.837
2.3738	2.9339	4.2806	3.3616	3.5466	2.539	2.9568	3.8571
3.3884	4.6165	3.8085	2.3314	2.9915	3.48	4.8331	3.2474
4.1769	3.0804	3.7969	4.1718	3.7634	4.1481	3.2706	3.4011
3.4857	3.3099						
B9D1	3.8013	3.2442	2.7194	3.071	3.9832	2.6519	4.0017
4.6473	3.1302	2.8839	3.5929	3.1776	4.0963	1.8535	2.4553
3.3303	4.099	2.5737	2.0571	3.8712	2.8451	3.1023	3.8489
2.7816	3.0684	3.7484	3.6937	2.2453	3.505	2.2654	4.8124
5.09	4.0437	3.9245	3.9942	2.7532	0.4139	3.8563	3.4051
4.0318	4.3974	4.7444	3.3475	2.7056	3.3888	3.7651	2.8541
3.4498	3.2333	2.7532	3.503	3.0586	2.7583	2.698	4.3048
2.5779	3.8335	2.2522	2.7339	2.4053	3.7746	3.5128	2.8413
3.3996	2.8056	4.0187	3.2595	3.1534	1.9709	3.8935	4.4883
3.357	3.2165	4.5166	3.1077	2.211	3.3186	3.49	3.8275
3.8678	4.3039	3.6008	2.8923	0	3.3515	3.2856	2.6689
2.8579	2.939						
B9D2	1.2547	1.5412	1.5933	1.0809	1.8084	0.9571	0.7283
1.6129	0.5275	0.7185	0.2702	0.7658	1.0161	0.5827	1.8131
1.0709	1.7143	0.5382	1.0383	1.8996	1.5147	1.4723	0.8569
1.411	1.4161	1.592	0.9835	1.4553	1.4404	0.8037	1.8164
1.4676	1.5445	0.85	0.7642	0.4086	1.1067	1.8065	0.8086
0.8403	1.1654	0.8794	0.929	0.6332	1.1661	1.5615	0.44
0.9167	1.4304	2.0617	0.8494	0.9014	0.9403	1.5534	1.539
1.4099	1.1699	0.9423	1.1338	0.9541	1.9564	1.6211	1.767
1.4215	1.4882	1.69	0.9351	1.693	1.4365	1.527	1.3793
1.2226	0.8055	0.8102	0.9011	1.0316	1.217	0.8494	0.9564
0.9429	0.649	1.1299	1.3202	1.03	3.1336	1.6326	1.0777
1.2997	1.465						
BAALC	0.9763	0	0	0.0454	0.3215	3.6373	3.6182
1.865	3.3547	1.075	0.921	4.096	0.8973	3.0901	1.7344
3.3298	0	3.7526	1.8707	0	0	2.6234	0.6002
3.3158	5.696	0.6214	3.958	0	3.6381	0.1844	0.15
0.2279	0.1141	5.9051	5.2484	1.2067	0.3421	0	0
5.0357	2.0896	1.3655	4.0562	0.0015	0	0	0.2656
4.8164	0.1856	0	2.5466	5.8066	2.9085	0.8119	0.317
0.2742	0.2732	4.8742	0.7859	4.7082	2.5338	0	1.4525
0.2778	0	3.043	0	0.3628	0.2646	2.081	2.0666
3.9881	0	4.7261	2.7341	4.9197	0.2887	0.2419	0.6387
0.2867	0.2369	1.959	6.0996	5.6478	1.5985	2.6228	1.7176
1.7536	1.187						
BAAT	1.1313	2.5964	4.9775	1.5415	0.0259	0.0739	2.4304
0.1001	0.0208	0.036	0.0289	0.7354	0.0705	0.0566	0.3677
0.132	0.1652	0.7263	0.0434	1.2394	0.248	0.0049	0.0181
0.1105	0.0686	0.0579	0.0262	4.3126	0.1079	5.1668	0.0215
3.1195	0.597	0.014	0.0197	0.0991	0.1604	0.2268	4.6926

0.1379	0.462	0.0042	0.0256	0.0779	0.0898	0.037	0.2477	
0.1226	0.0135	0.2413	0.0142	0.1358	0.2977	0.1396	0	
0.2898	0.0221	0.1695	0.6046	0.0363	1.2374	2.04	0.0477	0
	0.6416	0.0749	0.0891	0.0276	2.0624	0.8604	0.0392	
0.0926	0.0154	0.0163	0	0.0182	0.186	4.6356	0.3631	
1.6252	5.7903	0.0175	0.1501	0.1027	0	0.129	0.0705	
0.0481	0.0731							
BABAM1	4.872	6.0946	5.4659	5.2974	6.2888	5.2395	5.4147	
5.7151	5.2782	5.116	5.8653	5.8261	5.6699	5.9958	5.4233	
5.324	5.5602	4.7207	5.2776	5.4422	5.5696	5.4605	5.7441	
6.0818	5.2236	5.8235	5.8115	5.5296	5.5249	5.5579	5.7751	
6.226	5.6633	5.758	5.4824	4.9868	5.7921	4.8285	5.5127	
5.1487	5.5557	5.71	5.4877	5.1645	5.3157	5.4118	4.3516	
4.7885	5.2029	4.6339	4.9397	5.3157	4.524	5.8056	5.4291	
5.4751	5.5005	4.7463	6.658	5.0686	5.7807	5.6134	5.5618	
5.8197	4.8496	5.2742	5.4327	5.7674	5.4152	4.8789	5.2125	
5.6739	5.3617	5.5761	5.3545	5.1626	5.6373	5.3271	5.1556	
5.1961	5.3959	5.698	5.4365	4.7818	6.1617	5.041	5.4659	
5.5696	5.419							
BACE1	3.0473	2.2928	2.4471	4.2375	4.3624	1.9616	2.6278	
1.4811	3.6007	1.4402	2.4553	2.2736	3.2084	2.6587	3.4164	
2.6095	2.4863	2.8716	2.0197	4.1825	2.6051	4.4592	4.7268	
2.9525	2.9646	3.8169	3.8991	1.938	4.7209	3.4066	2.7474	
1.657	1.6977	3.5005	4.3154	1.8493	2.1276	2.93	3.3313	
3.3626	2.9882	4.4272	4.6239	1.8256	3.4699	3.9772	2.0431	
3.2552	3.3219	2.6373	2.8356	4.8681	2.5324	2.456	2.2686	
3.0883	3.2849	3.2916	1.504	3.5018	2.8475	2.6885	3.6353	
2.8703	3.1665	5.0967	3.8558	3.4086	1.7556	3.9727	1.7786	
2.43	2.4253	3.7402	3.2112	2.204	2.0556	5.091	3.9776	
2.0219	2.3644	2.7411	2.5026	3.8596	3.4826	2.1542	4.1612	
3.1718	3.773							
BACE2	6.901	0.3712	6.0027	4.9955	4.2634	5.7417	5.538	
5.1059	6.1833	6.2169	6.2118	4.7973	3.5194	5.9577	5.4227	
5.965	4.4196	6.1718	5.6539	5.261	6.6408	5.5932	5.6599	
5.4509	5.5693	4.9182	4.907	5.4803	5.9982	6.5325	2.9835	
5.1966	5.5814	5.9928	6.0372	5.8278	5.2049	5.3932	5.3505	
5.965	5.7622	3.7807	4.9819	5.8511	3.4043	2.2047	5.6547	
5.8319	5.1458	2.4723	5.3622	5.6018	5.7984	4.9619	0.0657	
5.8413	3.6602	6.0961	6.4327	5.8289	5.1389	6.0613	4.9976	
5.9235	5.2378	4.0951	5.1809	5.2616	6.3838	5.2429	5.2808	
5.0376	5.3773	4.6669	5.5134	5.7401	5.4395	6.3024	6.2692	
5.4125	5.3565	5.5881	4.1072	6.8094	4.3317	5.4133	5.6771	
5.3843	5.2857							
BACH1	2.5812	2.7403	3.0235	3.438	3.1302	4.1533	5.0732	
3.9825	3.7676	2.4133	3.0525	4.1076	3.9205	4.1337	4.3387	
3.4968	5.6264	3.0361	3.294	2.6343	2.9596	4.581	3.5826	
3.5096	4.6651	5.1305	4.1672	3.1735	3.923	3.0512	3.7036	
3.4787	3.3761	2.863	3.9628	2.9811	3.911	2.5348	3.1259	
4.222	3.5775	3.1877	3.9367	3.4557	2.9108	3.6884	3.6592	
4.1768	3.9493	4.3587	3.7595	4.5192	3.9049	5.1607	3.7721	

2.9544	4.9698	2.7647	3.0265	3.0247	3.351	2.5868	2.9214
3.5573	2.8662	3.7746	3.0771	3.2326	3.4897	4.6293	3.4003
2.8286	3.1635	4.3685	3.4663	3.4606	3.1754	2.9284	3.4275
3.5944	3.2181	4.0937	5.1258	4.9703	3.4023	3.2279	3.0198
2.6958	2.4686						
BACH2	0.0668	0.9151	0.6801	0.4963	0.285	0.5366	0.4743
0.2684	0.701	0.4782	1.0389	0.1297	0.0377	0.1575	0.1243
0.966	0.5226	1.0124	1.3601	0.5313	0.9543	0.2536	0.473
0.5026	0.9601	0.3815	0.6705	0.6817	0.4221	0.4838	0.2693
0.2582	0.4949	0.6591	0.1908	0.4273	0.127	0.4126	0.4794
0.606	0.4472	0.3552	0.7257	0.7204	0.1274	0.6499	0.3591
0.9059	0.5037	1.3668	0.326	0.0668	1.3087	1.1546	0
1.0897	0.8583	0.7662	0.0068	0.795	0.2039	0.2312	0.2263
0.8941	0.7123	0.0923	0.7657	0.3301	0.9963	0.7881	0.7217
1.186	0.3207	0.7117	0.3159	0.8086	0.6722	0.2739	0.2897
0.8419	0.6955	0.2712	1.0576	0.2576	0.9377	0.6146	0.6789
0.8145	0.9736						
BAD	3.6164	4.4174	5.0356	2.9445	4.6542	3.955	3.3394
4.9044	3.3417	2.6858	3.8318	3.1443	4.4302	2.6281	4.5144
3.9227	4.4197	2.7705	2.6993	5.217	4.502	4.7763	4.4878
3.809	4.3496	3.6081	4.044	4.145	4.0757	3.8997	4.7062
3.7916	3.8358	4.2225	4.1172	2.6048	3.7984	5.1608	3.9
2.4278	4.8677	4.697	3.8279	2.554	3.8705	3.5669	2.5488
3.7305	4.9137	3.0471	4.1936	2.9042	2.6263	4.1029	5.9531
4.1516	3.7754	5.0476	3.7778	4.5429	3.8698	3.7461	4.1381
2.6479	3.4978	5.3391	4.5146	4.931	3.1175	4.5727	4.9358
3.5385	4.0841	3.0116	3.6024	5.1375	4.325	3.9369	3.4163
4.1855	2.8538	3.8029	4.5484	3.9374	4.8635	4.0197	4.4455
4.4553	4.473						
BAG1	3.3588	4.1371	4.0389	3.5466	3.437	2.6173	1.9346
4.0161	2.5483	3.2695	3.3045	2.5208	2.6152	3.5696	3.0529
3.1941	3.6207	3.1078	3.7699	3.9631	3.1354	2.502	2.9041
2.9319	3.0226	2.2353	4.1664	2.629	3.3196	3.276	1.9526
3.1488	2.0339	1.7382	1.7591	3.6113	2.5815	3.6472	4.5378
2.6303	2.8727	2.4262	1.9423	2.8144	2.5586	3.372	4.0418
2.3544	3.4979	3.4221	3.2721	3.3756	3.1789	3.5349	3.3305
3.1434	2.1401	3.5235	4.989	3.4243	3.3918	4.5296	3.9813
2.8131	3.4956	3.3347	2.6913	3.6032	2.808	1.9771	2.2973
2.3251	2.9061	3.5962	2.6872	2.6927	3.3136	3.3136	3.1079
2.7134	2.7061	3.3208	3.6959	2.3562	2.98	2.9566	3.1086
3.518	3.3986						
BAG2	3.2125	4.773	1.3996	5.3205	4.0171	2.187	4.9178
3.4899	3.7761	4.4743	3.113	4.8243	1.7409	4.2115	2.9146
0.6901	2.1789	3.0131	5.2727	2.9317	2.8432	3.0549	3.6484
4.7673	3.5964	4.5171	4.3065	4.4368	3.5201	3.9012	4.2594
2.576	3.7379	3.1289	1.1744	5.241	4.8744	3.1222	2.6862
3.9478	4.0241	4.9291	4.0857	3.4395	4.2665	4.6625	4.6151
4.1016	2.9381	4.9652	4.1931	4.361	2.913	2.6655	5.5068
3.8976	4.249	3.4385	4.4915	3.0971	4.0531	4.1373	3.2039
1.7313	5.5477	3.2802	4.5224	3.0907	3.4694	1.9145	3.8582

2.6591	3.317	3.2701	2.973	4.2832	4.5091	3.7157	3.4211
4.3341	4.166	4.6671	2.552	1.1589	4.2911	2.7448	1.9923
2.0684	1.7014						
BAG3	4.0006	3.9866	4.1051	5.1882	5.1795	4.8651	4.3818
5.534	4.508	4.215	3.6601	4.921	5.4374	4.4151	4.6071
4.0268	4.4932	3.7536	3.7674	5.2154	4.8326	3.609	4.8905
4.913	4.7382	3.9289	4.6223	4.5878	3.671	3.7615	3.7349
4.2769	4.7733	4.97	4.5629	4.6075	5.4929	5.1987	4.504
4.259	4.6277	5.9324	5.1539	4.6757	4.8731	4.4903	4.7983
4.5892	5.1526	4.7936	4.4347	3.2369	4.1403	3.9378	5.674
4.0935	4.6961	4.7007	4.5586	4.3684	4.7893	3.8268	5.0207
5.065	4.9376	5.0587	4.3495	4.763	4.1711	5.4186	4.4962
4.1124	3.4421	4.9545	3.8914	4.8819	5.0439	4.7951	3.9514
4.1386	4.2974	4.838	5.5309	3.9773	5.3	4.1568	4.7843
4.5382	4.5719						
BAG4	2.5902	2.6643	2.7017	3.3232	2.5724	2.684	2.8583
2.9544	2.309	1.9808	2.5911	2.8579	2.9399	3.0006	2.3762
2.77	2.903	3.2756	2.9209	2.6217	2.9144	3.1582	3.1164
2.84	3.3179	2.5478	4.2441	3.3256	3.4226	2.6701	3.5345
2.5312	2.8601	3.143	2.9901	2.881	2.5574	2.5979	2.4775
2.9743	2.3542	3.051	2.9317	2.5658	3.7423	3.166	3.5091
2.1493	2.8186	4.3156	2.8877	3.4257	3.1911	2.7101	3.2082
3.2064	2.2079	3.4924	3.1209	3.5633	2.8441	2.977	3.5393
2.5693	4.1986	2.0555	3.4331	3.0884	2.4001	3.3085	3.1599
2.5712	3.216	3.0289	3.2483	2.9265	2.9566	3.19	2.3639
2.6201	2.9822	3.3537	2.343	3.0439	3.1276	3.1404	2.9443
2.7941	2.863						
BAG5	3.8572	3.3705	3.0927	4.1217	3.3966	3.8891	4.3327
3.5335	3.26	3.7099	3.4457	3.5845	3.6702	4.0402	3.8766
3.633	3.4467	4.0358	4.0484	2.736	3.8528	3.4415	3.8474
3.1891	3.4337	3.6533	2.9504	3.8228	3.8247	3.1839	3.1289
3.919	3.6691	2.9275	2.7337	3.8286	3.5239	3.509	3.2757
3.8603	3.3198	3.431	2.9439	3.5561	4.3653	3.9818	3.5526
3.5711	3.397	4.3399	3.201	4.0122	3.6168	3.6337	3.024
3.429	3.8333	3.8142	3.1322	4.1747	3.6148	3.6542	3.7994
3.3042	3.6963	3.9304	3.6491	3.9473	3.0949	3.2685	3.4119
3.2992	3.7087	3.2178	3.9934	2.3643	3.601	3.0936	4.2226
2.9137	3.4419	3.648	3.0684	3.3688	3.3521	3.223	3.565
3.8411	3.6759						
BAG6	5.8883	6.2603	5.9878	6.1005	5.9521	6.0235	5.1356
6.2834	5.881	7.0889	6.1515	6.6308	5.4065	6.0635	5.8418
6.0635	6.6762	6.189	6.2804	6.0436	6.2793	6.5103	6.4697
5.8519	6.282	6.4271	6.465	6.504	6.4079	5.9585	6.396
6.4422	5.8468	6.1456	6.3413	6.3944	7.0454	7.0027	6.6013
5.4636	6.0518	6.2289	5.8764	6.197	5.587	6.3522	5.0039
5.7405	6.3681	5.9448	5.9953	6.197	6.1158	6.3335	6.2885
5.9672	6.2004	6.2583	5.7764	6.2928	6.2764	6.2826	6.0607
6.2725	6.8316	4.9387	6.9636	6.3928	6.3458	5.8821	5.8575
6.553	5.7813	6.6434	5.7772	6.6457	6.4599	6.7663	6.2131
6.1704	5.9474	6.54	6.951	5.8681	6.5276	6.1595	6.1393

6.1603	6.3238							
BAGE	0.0976	2.8029	0	0.0817	3.7161	0.4328	0.9541	
4.3393	0.6616	1.0394	1.8778	0.1256	0	0.0013	0.0203	0
	0	0.115	2.6757	0.5298	0	2.4551	2.3594	
3.1637	0	0	3.0041	0.0261	2.7936	0	0	
0.3379	0.0557	0	1.8819	0.9401	0	0.2595	0.1903	
0.0597	0.1828	1.9072	0.0393	0	1.9245	0.1194	0	
2.2681	3.2103	0	0	0.0203	0	1.7242	1.3793	
1.6694	0	0.1953	0.2721	0.2162	4.6884	4.1311	0	
0.1428	0	0	0.4453	0.0958	0.0986	0	0.1387	
0.8282	0.1445	1.1658	1.9255	0	0.0965	3.0382	0.1627	
0.1153	1.3119	0.2229	0.0996	0.2373	0	0	0.0127	0
	0							
BAGE3	0.2465	0.0328	0	0	0.4939	0.4841	0.145	
1.9052	0.1666	0.2622	0.0249	0.1491	0	0	0.459	0
	0	0	0.057	0.059	0.0436	1.2951	0.2908	
0.2474	0	0.2239	0.0339	0.5076	1.1793	0	0	
0.785	0.78	0.2878	0.0236	0.0276	0.0247	0.0392	0.0271	0
	0.0286	0.3388	0	0	0.4374	0	0	
0.0357	1.7156	0	0	0.0335	0	0	0.1872	0
	0	0	0.4959	0.2963	1.9433	1.005	0	
0.6867	0	0	0	0.0866	0	0	0	
0.1099	0	1.1652	0.3476	0	0.1028	0.5229	0.4388	
0.9684	0.185	0.3119	0.4207	0	0	0	0	0
	0							
BAHCC1	3.5281	2.5415	3.4572	1.4737	1.7871	2.5911	1.2837	
2.5609	3.0376	3.6978	3.3928	1.8423	1.7302	2.5732	1.4677	
3.1668	1.3963	2.8081	2.8844	2.3254	3.2794	2.001	1.1899	
2.5942	1.762	1.0754	1.5557	3.9966	1.9793	2.9175	1.4913	
2.3214	2.3366	3.6584	1.4192	2.3094	2.4102	3.9706	4.0337	
1.7129	4.2463	1.1272	1.3222	2.4865	1.7147	1.2298	1.9258	
3.58	2.0948	1.1407	1.8028	0.5948	2.0257	3.5677	1.7574	
2.9615	0.9609	2.2433	2.5053	2.3686	2.4444	2.3373	3.6338	
2.6121	3.0062	2.0026	3.8238	1.6351	3.6071	2.111	2.7804	
3.5306	3.0114	1.4551	2.464	3.8493	3.2953	2.3333	2.9535	
3.0058	3.1546	1.6127	0.7585	3.5422	1.8521	1.9949	2.4847	
3.1714	3.1256							
BAHD1	3.532	4.3298	3.6591	3.0631	3.2641	2.9965	3.0459	
3.2696	3.1628	2.8882	3.5783	2.3591	2.8995	3.0171	2.9499	
3.5509	3.1946	3.8399	3.2228	3.2284	3.277	3.3765	3.2599	
2.8468	3.2269	2.7999	3.1108	3.6412	3.2202	3.0081	3.2946	
3.2603	3.0088	4.6082	2.9339	3.0879	3.2621	3.0536	3.5917	
2.599	2.1715	3.5724	3.2144	3.0244	3.6308	2.6948	3.401	
3.3561	3.0132	3.0991	3.2569	2.1835	2.8908	3.6722	3.5209	
3.8778	2.9784	3.4935	3.9297	3.4276	2.959	2.8366	3.4434	
2.9513	3.2197	2.7081	3.692	3.6699	3.2641	2.618	3.0283	
3.2722	2.7556	3.0554	3.0376	3.6567	2.8106	2.9599	3.1198	
3.0974	3.3838	2.9068	3.8415	3.5045	3.4973	2.9394	3.6975	
3.5869	3.5446							
BAIAP2	4.3236	3.7843	3.7995	2.0631	3.0445	3.1071	2.9687	

4.5149	2.9769	2.7923	3.2987	2.6259	3.2198	3.082	3.3721
3.8197	3.3811	3.3666	3.8018	2.5092	5.4194	2.4391	3.0787
2.5811	3.1114	2.9287	3.3472	4.7854	3.8246	3.9314	2.9142
4.2523	3.6293	3.8958	2.6269	2.815	4.9222	4.3223	4.6729
2.8864	3.4485	3.4598	3.0114	3.12	4.4995	3.845	3.7718
3.4485	2.0822	2.6681	4.3995	1.4129	3.3831	3.1303	4.2578
4.1614	3.2935	1.6097	4.5016	1.308	3.5766	3.4126	3.8409
3.7196	4.7534	4.0217	4.494	3.7496	4.4688	3.5913	3.0894
3.5348	4.0057	2.8054	3.5751	4.6865	4.7359	3.1604	4.6116
2.4059	3.1021	3.0582	4.4773	4.4253	3.059	3.7697	4.8471
4.6375	4.9407						
BAIAP2L1	3.7472	2.9632	2.4834	3.4179	3.7959	5.9324	2.7683
3.7391	4.7408	4.4641	4.9746	2.6859	3.4015	4.3725	2.1811
4.2087	2.0269	5.3796	5.0751	2.2479	3.5171	3.7431	3.1034
4.1667	3.0069	4.0512	3.5452	2.349	2.6312	4.5201	4.3827
3.7551	4.468	3.8904	2.3625	4.3917	1.4287	2.6608	2.7299
3.324	3.8663	3.5069	4.3735	5.0071	3.9919	4.3807	3.1629
3.8853	3.3276	3.3285	3.338	3.6235	3.9335	1.4372	4.5715
3.4021	0.8544	3.8803	5.2543	4.0273	3.8261	2.7727	2.0522
4.7407	3.6037	3.5621	2.8729	3.2924	2.788	3.8114	4.7087
3.9411	3.5056	2.4302	3.5043	4.1052	3.6164	3.2055	4.3456
4.8921	4.516	3.4653	2.2924	2.7307	3.6197	3.6593	4.0575
2.4629	3.5576						
BAIAP2L2	2.4973	0.5859	2.8369	3.3868	4.4515	4.2615	2.8407
1.0681	2.4444	2.201	2.8938	3.5756	4.1268	3.0482	1.6103
3.4036	2.4133	0.7608	3.5068	1.2576	5.4827	4.6353	0.1348
0.8044	1.2237	1.9214	1.0185	2.082	4.4753	0.7619	0.212
1.2452	6.5922	2.7567	3.4252	2.0858	4.0211	2.091	2.9883
1.5598	2.4395	3.2949	3.8616	3.8463	1.3354	1.3085	2.905
2.0743	0.6245	0	2.478	3.1517	0.7231	4.3937	0.2381
5.6206	2.6443	0.1516	1.4995	0.2115	4.1259	2.4638	2.5275
4.1854	5.3981	0.1275	1.3497	3.2214	1.8615	3.7968	4.7797
4.6359	3.4447	2.1848	1.8041	4.735	4.5835	0.0537	2.3681
0.6412	2.6458	2.423	3.5668	1.5673	2.2362	1.3147	3.7337
5.1064	3.5326						
BAIAP3	0.2873	0.2502	0.1213	0.1511	0.1496	0.1848	0.0862
0.4992	0.1263	0	0.327	0.1088	0.2822	0.1421	0.0957
0.3522	0.1411	0.1556	0.1062	0.2303	0.2595	0.4059	0.0706
0.1742	0.1925	0.094	1.375	0.2585	0.1122	0.1863	1.5362
0.2415	0.0802	0.2459	0.052	0.1429	0.1271	0.4093	0.1787
0.1305	0.3654	0.3938	0.1186	0.2727	0.3723	0.0838	0.1568
0.1914	0.2246	0	0.0908	0	0.1832	0.1566	0.4704
0.2168	0.0085	0.7859	0.761	0.4671	0.0909	0.088	0.4111
0.183	0.4274	0.0339	0.7071	0.0946	0	0.3972	0
0.0053	0.6617	0.4429	0.049	0.2254	0.0586	0.2845	0.1037
0.5438	0.4429	0.0265	0.2685	0.0162	0.0865	0.3593	0.1044
0.1191	0.2013						
BAK1	3.7075	4.6081	4.5525	4.3131	3.9348	3.8729	4.3499
3.878	4.509	5.1107	4.1756	4.7807	3.6398	3.7581	3.9887
4.4528	3.6984	4.1267	3.574	4.5612	3.9717	4.1126	4.5206

4.052	3.9263	4.2319	4.5024	4.8481	4.2	3.7545	3.4541	
3.7352	3.8946	4.0949	4.3077	4.6643	4.0949	4.4519	4.7639	
4.3732	4.12	4.6494	3.9798	3.2998	3.8339	4.0696	3.6308	
3.6761	3.4947	3.8033	3.8465	4.204	3.7561	3.607	4.475	
3.6611	3.9294	4.5981	3.5062	4.1525	4.5008	3.966	4.2542	
4.0644	4.2434	3.409	4.5922	3.6286	4.3197	3.4171	4.1416	
3.7406	4.001	3.9746	4.0332	4.2129	4.5144	4.9329	3.2873	
4.0688	3.7367	4.5827	3.9517	3.5775	4.0412	3.6247	3.9729	
3.9437	3.7906							
BAMBI	6.7773	4.7236	6.4625	6.6792	5.5422	5.2635	7.1591	
4.9987	5.4995	7.4741	7.7868	6.2341	5.0553	7.1617	5.1617	
7.9208	2.4887	7.9269	6.8127	6.7908	6.6297	7.2099	6.8084	
6.8236	6.8259	3.8245	6.5123	7.4012	6.1839	5.3242	2.8336	
6.5846	7.4511	8.0112	6.6448	6.7425	5.1437	5.6539	7.3779	
6.6851	6.3099	3.8326	7.381	6.9653	2.7023	3.5046	6.6874	
6.6792	6.8084	2.8293	7.9743	6.2677	5.877	6.8701	0.3074	
5.7748	2.8933	3.8462	6.2182	4.5519	6.2821	6.6123	6.2014	
6.4903	4.4216	7.9538	5.9409	7.909	7.2519	6.5226	6.3217	
6.7341	4.8819	6.5776	6.8318	7.2471	6.9728	6.655	6.2489	
6.5254	6.0512	5.435	6.3624	7.1427	5.8945	6.1756	5.746	
5.5119	5.8131							
BANF1	6.8099	6.9783	7.2681	6.8806	6.9606	6.8075	5.9839	
7.4517	6.8689	6.9959	6.7122	6.1936	6.9189	6.192	7.1917	
6.1608	7.3118	5.9017	7.7563	6.6632	6.9142	7.593	6.7815	
6.9499	7.2293	6.1907	6.4036	6.6389	7.132	6.5714	7.4883	
5.814	6.821	6.1936	6.7284	6.7179	6.7179	6.9288	6.7857	
6.2563	6.3497	6.7372	6.8075	6.4304	6.8788	7.1887	6.6632	
6.432	6.0965	7.8836	6.2707	6.3947	6.3542	7.073	7.5118	
7.0708	7.0839	7.4838	6.8739	7.0459	6.4127	6.5609	7.247	
6.7712	6.6837	5.733	5.8732	6.1434	6.6565	6.4903	6.7602	
6.4254	6.7252	6.1138	6.5487	6.39	7.1063	5.9546	6.68	
6.9939	6.4142	6.1584	5.8116	5.8445	7.0304	6.6379	5.9839	
6.5159	6.2877							
BANF2	0	0	0	0	0	0	0	0
	0	0	0	0	0	0	0	0
	0	0	0	0	0	0	0	0
	0	0	0	0	0	0	0	0
	0	0	0	0	0	0	0	0
	0	0	0	0.2712	0.1283	0	0	0
	0	0	0	0	0	0	0	0
	0	0	0	0	0	0	0	0
	0	0	0	0	0	0	0	0
	0	0	0	0	0	0	0	0
	0	0	0	0	0	0.2963	0	0
	0							
BANK1	0.1318	0.0131	0.0564	0.4258	0.0961	0.0421	0.0105	0
	0.0044	0.0075	0	0.004	0.0887	0.0594	0.0084	0
	0	0.02	0	0.1316	0	0	0	
0.0049	0	0.103	0	0	0	0	0	
0.1684	0	0	0.0148	0	0.1272	0	0.0128	0

	0	0.0072	0	0	0.0422	0.0353	0.0191	0
	0	0.0476	0.0208	0.0563	0	0	0.3975	0
	0.0921	0	0.0271	0.0849	0	0	0.0644	0
	0	0.3899	0.0492	0.0064	0	0	0	
0.0137	0.0167	0.0072	0	0	0.0122	0.0158	0	
0.0444	0	0	0	0	0	0	0.0939	
0.1473	0.1806							
BANP	2.3585	2.9651	3.3312	1.9686	2.2188	2.1525	1.6207	
2.5806	1.6833	2.2727	2.1685	1.7775	2.2832	1.8072	2.5994	
1.9298	2.3768	2.3009	2.4211	2.2682	3.0057	2.5866	1.8607	
2.4945	2.6702	3.1141	1.6166	2.7613	2.233	2.9302	2.8115	
2.1928	2.4222	2.0926	2.5896	2.2956	2.302	2.1445	2.2365	
2.3895	2.1743	1.8222	2.2593	1.3028	2.0818	2.6569	2.3379	
2.2883	2.286	2.4825	2.2423	2.2145	2.4738	3.0318	2.8898	
2.9632	2.3244	3.0355	2.3019	3.2645	2.3844	2.9985	2.7431	
2.9198	2.5081	1.9073	2.6632	2.2856	2.8937	2.6612	2.0865	
2.5875	3.376	2.0456	2.1643	2.7019	2.7444	1.7502	2.4258	
2.4153	2.5245	1.5126	2.697	2.1146	3.224	2.4196	2.5263	
3.6162	3.1348							
BAP1	4.672	4.8431	5.394	3.8617	4.6758	4.5298	4.4778	
5.2161	3.9896	5.239	5.1052	4.9193	4.7773	4.1281	4.2733	
5.372	4.833	4.7256	4.0197	5.1845	5.2329	4.8527	4.4686	
4.8472	4.5506	4.4166	4.139	5.0049	5.0665	5.2408	4.9537	
5.5838	4.4995	4.644	5.0062	4.5986	5.2577	5.2645	5.147	
3.8335	4.5981	4.4379	4.3477	4.5038	3.9884	3.5955	4.4946	
5.1746	4.7963	4.1332	4.2446	4.3822	4.0053	4.6718	4.7812	
4.4277	4.4726	4.5909	4.8807	4.7134	4.661	4.8079	4.7924	
4.616	5.0155	4.3346	5.8008	5.8834	5.1765	4.2082	4.5217	
4.3606	4.9952	4.3821	4.9654	5.3427	4.915	5.4629	3.7691	
4.2931	4.6114	4.2759	5.1173	4.9634	4.9383	4.3011	5.3898	
5.3676	5.4656							
BARD1	2.1432	1.4671	2.5623	2.6087	1.6152	2.7047	2.0071	
2.1285	2.1104	2.4167	0.8531	1.6892	2.7375	2.8731	2.8535	
1.1729	2.2729	2.3173	2.6606	2.2006	2.5633	1.6614	1.696	
2.1222	1.9991	1.0459	1.1985	2.4968	2.2428	1.9761	2.6419	
2.0409	2.394	1.487	1.8345	2.9472	2.1646	1.1437	1.355	
2.0648	1.3799	1.3077	2.7354	3.1711	2.935	2.884	2.4802	
2.5153	2.0299	3.0361	1.8485	2.6703	2.4496	1.2105	2.934	
1.6786	1.929	2.9895	2.0783	3.1318	2.462	1.9928	1.6977	
2.4086	2.6886	0.9667	2.4608	0.8496	2.3513	2.5807	1.5557	
2.5639	2.6	1.6003	2.1179	1.9183	2.5306	1.9872	2.5414	
2.0202	2.2624	1.4082	1.0862	1.2312	3.1079	2.999	1.6485	
2.5797	2.3607							
BARHL1	0	0	0	0	0	0	0	0
	0	0	0	0	0	0	0	0
	0	0	0	0	0	0	0	0
	0	0	0	0	0	0	0	0
	0	0	0	0	0	0	0	0
	0	0	0	0	0	0	0	0
	0.0642	0	0	0	0	0	0.0292	0

	0	0	0	0	0	0.063	0.0187	0
	0	0.0046	0	0	0	0	0	0
	0	0	0	0	0	0.0235	0	0
	0	0	0	0	0	0	0	0
	0							
BARHL2	0	1.448	0.9408	0	0	0.1038	0.4015	
0.0553	0	0.0613	0.0425	0.1896	0.1678	0.2702	0.2217	
0.1236	0	0	0.1731	0.6347	0.1447	0	0.03	
0.4431	0.0695	0.1318	0.0812	0.823	0.0969	0.2072	0.2597	0
	0.3615	0.0671	0.0269	0.0936	0.1898	0.421	0	0
	0	0	0.5292	0.0718	0	0	0	
0.2363	0.0867	0	0.0621	0	0.1272	0.0617	0.4659	
0.971	0	0	0	0	0.0386	0.2828	0	
0.1283	0.2636	0	0.1053	0	0.322	0	0.0101	
0.4845	0.2581	0.0767	0.0082	0.2178	0.3068	0.1506	0.1568	
0.3946	1.1421	0	0.1599	0.0187	0	0	0	0
	0							
BARX1	0	0.1445	0.1711	0	2.8886	0	0.8884	0
	0.1983	0	0.5303	0.1288	0.6394	0.2874	1.2246	0
	0	0	0	0.2204	0.1505	0.5318	0.2436	0
	0	0	0.2828	0	0.119	0.1375	5.0934	
0.5597	0.4639	0.1337	0	1.1196	0	1.3951	0.2981	0
	0.3297	0	0	0	0.111	0	0	0
	0	0	0	0	0	0.1029	4.3866	0
	0	2.0105	0.0619	1.2909	0.1242	0.9907	0.6778	
0.1178	2.3291	0	0	0.292	0	0	0.0297	
0.891	0.6811	0	0	0	0.0176	0	0.2504	
0.1706	0	0.0518	0	0.2018	0.0666	0	0.813	0
	0							
BARX2	0	0.1475	0	0.1835	0.0987	0	0.118	0
	0.1705	0	0.1067	0.102	0	0.1885	0.1609	
0.1137	0.1917	0.1687	0	0	0	0.4633	0	
0.1104	0.947	0.1203	0.599	0	0.1296	0.196	0.6849	0
	0	0	0.0895	0.1211	0.105	0	0.2366	
0.2502	0.1741	0	0.3648	0.142	0.1135	0.138	0.5373	
0.1507	0	0	0	0	0	0	0.0143	
0.0163	0	4.985	0	4.2134	0.0595	0	0	
0.1222	0	0	0.0358	0	0	0	0.0197	
0.0736	0	0.0016	0.3915	0.0092	0.0114	0	0	
0.0043	0	0	0	0	0	0.0141	0	0
	0							
BASP1	0	0.2454	0.226	0.2004	0.1384	0.3537	3.2491	
0.1478	0.7633	0.1586	0.2806	4.3989	6.3266	0.6695	1.3404	
0.4622	6.9232	0.2576	0.3579	3.808	0.1963	1.6683	4.2088	
0.375	0.1741	5.3391	3.9132	0.6036	0.1869	0.1918	7.3561	
2.0874	0.3587	0.8307	1.9708	4.6389	0.1606	0.8267	0.1675	
0.1969	0.2338	5.6048	4.7209	0.6208	6.2362	6.3008	0.9566	
0.5368	3.4288	7.456	3.1239	0.5123	0.291	0.1131	7.9315	
1.7225	6.9695	4.805	0.0838	5.3855	0.1008	1.0305	0.1945	
0.9448	1.9419	7.4095	1.4024	5.3665	0.073	0.5724	0.6953	

0.5471	3.1696	3.3483	0.571	0.0854	0.4338	0.4832	0.3252	
0.4132	0	3.9917	0.4803	3.3357	2.1547	0.253	5.3235	
3.8526	1.7664							
BATF	2.313	3.6662	3.1318	0.1055	0	0.089	1.1751	0
	0.7477	1.4518	0	0.0591	0.2823	1.5567	0	0
	0	2.1683	3.0407	0.1084	1.3555	0.6662	1.1973	0
	0	0.3447	0	1.7901	0.961	2.2017	0.0041	
1.2035	0	0	1.215	0.1623	1.2636	0	0	0
	0.6811	0.5141	0.068	0	0.0154	0.0405	2.0139	
1.0844	0.0145	0.3525	1.1045	0.8171	0.323	1.2979	0	0
	0.1139	0	1.681	0.1603	0.0847	1.9928	0	
0.8397	0	0.0749	0	0	3.3151	0	0.1009	
0.1019	2.229	1.289	0	3.723	1.6563	0.1058	1.6963	
0.2303	1.5942	0.1445	2.1086	0.1063	0	0.5993	1.9351	
1.9698	1.7908							
BATF2	1.2072	0.5682	0.6592	0.9862	0.8839	0.8862	3.5425	
2.4553	1.4157	0.4196	0.151	1.3029	1.4616	0.4836	0.9371	
1.9472	1.0683	0.2779	0.3156	1.241	0.2646	1.3818	1.2971	
0.8239	0.2586	2.6566	0.4592	0.7909	1.6563	0.4074	0.8245	
0.2838	1.1507	0.7424	1.9707	0.1916	3.2201	2.9991	0.9402	
0.1576	2.272	1.5446	1.6432	0.6344	1.6198	0.6134	0.5018	
1.934	0.8038	0.9744	1.8884	0.5698	0.8832	1.6244	1.2578	
0.6856	2.095	0.1234	0.6155	0	0.4496	0.4804	1.8446	
0.6163	0.2944	3.5726	0.3476	1.1567	0.5573	1.1567	0.6111	
0.6335	1.5433	1.9253	0.8541	0.4299	2.2311	0.9327	2.6127	
1.0351	0.3649	0.6183	1.901	1.1099	0.9854	1.3588	1.1645	
1.2166	1.0695							
BATF3	1.1685	1.0631	1.2345	1.5832	2.2396	1.0188	2.1024	
2.3696	1.0618	0.4908	1.568	1.6802	2.4807	2.449	0.5809	
0.5559	2.4753	0.5613	1.2502	1.356	1.4409	1.9839	1.8062	
2.0192	2.1598	1.3116	2.9227	1.9547	2.135	1.4043	2.4782	
0.8718	0.9411	1.2137	1.4269	1.8432	2.7721	1.2675	1.2543	
2.8198	0.531	1.1706	2.865	1.5972	2.3323	1.5441	1.2754	
2.8258	1.8094	2.2626	0.7532	2.2661	0.6978	0.8742	3.0895	
2.3917	1.3924	2.4475	1.4164	2.4141	2.5062	0.5612	1.0703	
1.9324	2.1305	0.5381	2.2639	1.2117	1.2776	2.0451	2.5806	
1.9295	1.677	1.3628	1.1507	1.601	0.9186	1.0039	1.0527	
1.7809	1.5148	1.214	0.4327	0.853	3.705	1.0973	0.6729	
0.8082	0.5076							
BAX	6.8658	6.0714	6.5345	4.9378	7.3855	6.3019	5.4173	
7.112	6.4115	6.4584	8.1489	5.8784	6.3102	4.4151	6.2746	
6.0806	7.3613	6.1306	6.1763	6.7244	5.7755	5.5178	7.1457	
6.4715	6.6817	6.8696	6.5909	6.9273	6.898	6.5345	5.8717	
5.5652	6.1316	5.7737	6.5734	5.7933	6.2504	6.5066	6.3323	
6.2691	6.4851	6.5873	6.9119	6.8868	7.0667	6.3601	5.1756	
6.3323	7.4272	4.905	5.4875	5.7914	5.3052	5.294	5.9563	
5.7159	7.6564	5.4926	6.3153	5.7896	7.0748	6.6031	7.0849	
5.4774	6.735	7.3802	6.0201	6.9653	6.8343	6.7871	7.1644	
6.5659	6.2783	5.8621	6.6114	6.1796	5.0388	5.7658	6.295	
7.0748	5.5252	7.1508	6.1343	7.0597	6.7292	6.2205	7.1148	

7.2711	6.9527						
BAZ1A	3.5466	3.2723	3.4395	3.9873	3.367	3.8398	4.5977
2.8806	2.8666	3.1011	2.4265	3.4579	3.1093	3.6876	3.6533
2.7812	3.5754	3.9504	3.9719	3.2741	3.3741	3.3837	3.8958
3.5875	3.2815	3.5029	3.4852	3.8331	3.6837	2.5374	2.956
3.0324	3.3915	2.8571	2.5493	3.6887	3.3626	2.8137	2.9545
4.1715	3.0613	2.6499	3.3731	3.6093	3.9829	4.2593	4.0165
3.58	3.7265	3.532	2.7999	4.3401	3.6589	2.5214	3.5345
3.3049	3.6414	4.2516	3.5917	4.5061	3.7597	3.5551	3.9022
3.5263	4.17	3.0016	3.6297	3.7487	3.2343	3.4283	3.1294
3.4762	2.805	3.4578	3.7736	3.1242	3.5532	2.8111	3.3045
2.9303	2.5734	2.9999	2.5771	3.1414	3.6329	3.7054	2.9283
3.4077	3.1411						
BAZ1B	4.8134	4.2259	4.5339	5.509	4.7701	5.8649	4.9477
5.5695	4.8589	4.86	4.9051	3.8466	5.2731	5.3085	4.8723
4.9969	4.0588	6.283	5.8352	4.6915	4.8288	5.1472	4.8049
5.2922	5.9437	5.1715	3.5534	5.0929	5.4195	5.036	4.2799
4.8493	3.8405	3.7024	5.2766	5.3015	5.3758	4.1123	5.1641
5.3196	4.596	4.3464	5.2276	4.9956	5.0125	5.4436	5.1375
5.888	5.3065	4.2865	4.9998	5.5279	4.3583	4.9465	5.2705
5.2186	3.3729	5.7019	4.8697	6.052	5.0892	5.0464	5.4553
5.4448	5.5171	4.6081	4.922	4.9332	4.9839	5.5878	4.0901
5.5141	5.2044	3.9901	4.9085	4.9897	4.9372	5.068	4.7406
4.0688	4.9965	4.0725	4.7716	5.176	4.9029	4.2432	4.4428
4.687	4.6321						
BAZ2A	4.1716	4.2744	4.56	3.9851	4.5298	4.6594	5.3245
4.2568	4.5874	4.4297	4.959	4.4654	3.9392	4.0825	4.3273
4.2559	4.7298	4.6805	4.1254	4.0213	4.3594	4.1578	4.1625
3.6074	4.548	4.5815	4.8606	4.5316	3.8025	4.9549	5.0307
4.2966	4.0768	4.342	4.0078	4.1936	4.5471	4.5505	4.0698
4.367	4.3517	4.1191	3.7692	4.9462	4.5434	4.0727	4.761
5.0663	4.7304	3.9628	4.2788	4.2406	5.2267	4.0399	4.4678
3.6746	4.6071	4.5463	4.5058	4.364	4.6183	4.4355	4.521
4.1373	4.2943	4.5472	4.5359	4.2499	4.9208	3.8351	4.4365
4.0073	4.4949	4.7937	3.8057	4.5573	4.2233	3.495	4.1725
4.4148	4.5144	4.3619	4.704	4.9213	3.6561	4.4209	4.5172
4.1744	4.399						
BAZ2B	2.1865	2.0032	1.8747	2.5296	1.7362	2.4766	2.951
2.3494	2.697	2.2834	1.9309	2.1598	1.6827	2.8006	1.6899
2.5027	2.0718	3.2628	2.2361	1.2389	2.0561	0.9624	2.0414
2.0924	2.3155	2.1754	2.7244	3.1089	1.7351	2.849	2.4547
2.3096	1.9189	2.1038	1.7698	2.5103	2.2221	3.0064	1.6777
2.2988	2.2487	2.312	1.8205	2.2185	2.8158	2.3768	2.6772
3.4221	2.2093	2.5977	1.9083	2.2979	2.8755	2.2794	2.3786
2.1804	2.4973	2.499	2.7324	2.4526	1.5231	2.1153	1.9511
1.4632	2.6666	2.5116	2.3791	2.4563	3.9	2.1681	2.2016
1.8588	2.5389	1.5885	2.3187	2.6368	2.3483	2.5102	3.1422
2.1333	3.076	2.2663	2.028	2.0181	1.8372	2.2658	2.5851
2.5086	2.694						
BBC3	2.4746	2.0849	2.7616	1.0973	2.7718	1.5698	1.7256

1.8797	1.174	1.8864	4.0017	2.0837	2.2108	1.4362	1.1147	
1.9499	3.3777	2.2466	1.615	2.5689	2.1798	1.8915	2.1995	
0.9437	1.5041	1.7391	4.44	2.0554	1.8606	3.7372	1.1257	
1.6629	1.8145	2.5388	0.9962	1.1345	1.6566	4.3958	3.9709	
1.2619	3.9875	1.2131	2.1747	2.2948	1.8888	1.2439	1.6901	
2.0262	2.7008	1.3226	1.4965	1.8322	1.8781	3.9078	1.1179	
1.5221	3.7071	0.6971	1.5617	1.4245	3.148	2.7401	1.7414	
1.2968	3.2145	3.9515	3.0273	3.1972	2.7804	1.2706	2.1947	
2.839	2.1085	1.7549	2.1489	2.1598	1.6422	2.0081	2.7547	
3.9898	2.2277	2.8776	2.9547	2.6356	1.7202	2.2075	3.5027	
3.0997	3.6585							
BBIP1	3.1701	3.5908	2.9106	3.5665	3.6911	3.9994	3.2421	
3.8816	3.8001	3.8665	2.7882	3.5143	3.5816	3.3295	3.0303	
3.3856	3.2179	3.5143	2.8637	3.2775	3.3661	2.5868	3.1945	
3.4373	3.0791	2.7713	3.7413	3.6894	2.6414	3.1327	3.8396	
3.8825	3.2861	3.2971	3.2442	3.8185	3.3425	2.9763	3.1384	
3.0623	3.2668	2.4578	4.0146	3.193	3.0436	3.112	3.3748	
3.6626	4.2186	4.0496	3.5616	3.2468	3.6112	3.2726	4.0117	
3.1364	2.8855	3.1213	3.3822	2.5798	3.2982	3.625	3.6857	
3.519	4.3721	3.5785	4.0899	3.501	3.2048	3.4701	3.0605	
3.1299	2.5827	3.1918	3.3929	3.542	3.6351	3.4665	2.7065	
4.0165	3.2676	3.3326	3.3077	3.1825	2.7847	3.5278	3.4716	
3.4353	3.5234							
BBOX1	0	0	0	0.2095	0.0103	0	0.256	0
	0	0	0.0527	0.1037	0.5427	0.0873	0.7064	
0.5166	0	0	0	0.1072	0	0.0211	0	0
	0.0345	0	0	0	0.1497	0	0	0.2
	0.0557	0	0	0	0	0	0.1289	
0.2733	0	0.0054	0	0.029	0.0621	0	0	
0.2332	0.0519	0	0	0	0	0.022	0.0436	0
	0	0.0444	0.025	0	0.0959	0	0	0
	0.0448	0.216	0	0	0	0	0.1764	
0.0861	0	0.0066	0.0309	0.0275	0.0329	0.0363	0.1126	
0.3345	0.1506	0.9347	0	0.1079	0	0	0	0
	0							
BBS1	2.6362	2.2157	2.0356	1.3767	2.4026	1.8285	2.6286	
2.0837	1.9876	1.2327	2.0125	1.3015	2.1024	1.8506	2.4646	
2.0765	2.1833	1.2946	1.4533	2.3623	1.7164	1.6654	1.8451	
1.8016	2.2997	1.8927	2.9181	2.3233	2.1087	2.4469	2.2353	
1.3657	1.8307	2.0645	2.1331	0.8037	2.6447	4.0235	2.9324	
1.8171	2.932	2.7521	1.3359	1.5561	2.0105	2.1574	1.9704	
2.527	2.8658	2.479	1.9881	1.375	1.6457	3.2104	1.9593	
1.6426	2.0421	1.4093	2.2649	1.3055	1.8439	1.8844	1.9114	
2.0215	2.2304	3.6627	1.9985	2.8686	2.568	2.2514	2.2001	
1.4956	1.3383	2.16	2.1266	1.8382	2.9978	2.4862	2.5756	
1.7049	1.5704	2.7368	2.6966	1.495	1.8078	1.4074	2.7735	
2.1249	2.6273							
BBS10	2.4139	2.3611	2.5363	2.8915	2.8602	2.2731	3.364	
2.5003	2.1775	2.5635	2.6855	2.25	2.7619	2.4356	2.805	
2.0424	3.1846	2.7829	2.4061	2.1494	2.3564	2.9225	2.9295	

1.8876	2.8138	2.6864	2.5824	2.5008	2.5557	2.1034	2.3939
3.0475	2.0414	2.083	2.7747	2.8442	3.0933	2.6035	2.3811
3.3478	2.5388	3.4374	2.3267	1.5505	2.8133	3.6183	2.3162
2.5105	2.6256	3.8253	2.421	3.2894	2.8663	2.9172	2.8157
1.62	3.5663	2.6148	2.183	2.0792	2.7571	2.9328	2.1766
2.4171	2.9628	3.7796	2.6848	2.6162	2.0545	2.7365	2.6268
2.1873	2.6277	2.845	2.3068	2.5502	2.3917	2.5633	2.0341
2.423	2.3335	3.39	2.5907	2.7702	2.4738	2.7144	2.7838
2.4704	2.5319						
BBS12	1.301	1.0133	1.0603	1.8283	0.7781	1.3537	1.7832
0.7961	0.9415	0.7358	1.1447	1.7706	1.5685	1.4008	1.4422
1.078	1.0726	1.7357	0.617	1.5186	1.7561	1.4028	1.7979
0.9427	1.0734	2.6212	2.1103	1.7538	1.4372	1.063	0.8308
1.5567	1.3626	1.342	1.5308	1.2693	1.6643	1.7603	1.2444
1.3891	2.1135	1.4778	0.9858	1.2896	1.1203	1.1417	1.1271
1.2182	2.0666	2.1218	1.3659	2.321	1.6981	1.3351	0.959
0.8766	1.7825	0.6426	1.2094	0.5645	1.6497	1.3085	0.8037
1.3188	1.1728	2.0907	1.6685	1.3608	0.5785	1.7763	2.0044
1.5842	1.4749	1.378	1.3614	1.2942	1.0071	1.5078	1.5709
1.9464	1.2882	2.0404	1.6161	2.0844	1.7622	1.3863	1.8388
1.7368	1.633						
BBS2	3.8954	3.5801	2.8219	3.7514	3.6149	4.3315	3.6195
3.2509	3.9205	3.1169	3.5179	3.9307	4.0884	4.3982	2.9646
3.5144	3.9067	3.871	3.1149	3.8297	2.8338	3.7462	3.5362
2.9735	3.7792	4.1673	4.7129	3.4236	3.0929	3.7047	4.2887
3.7961	2.9995	4.1315	3.9961	3.1582	4.1571	3.5222	2.568
3.6819	3.8552	4.4152	3.6557	2.9267	4.7749	3.9448	3.3799
3.639	3.9621	3.3677	3.6237	3.9629	3.5547	3.556	3.2253
3.5171	4.6052	2.6787	3.8726	2.8247	3.6586	3.5139	3.0496
4.2627	2.8326	4.0767	2.8053	3.192	3.6077	3.9892	4.4942
3.6633	2.8183	3.2447	4.3282	4.0728	2.9182	3.9084	3.9237
3.3656	3.4815	4.459	3.6776	4.3395	4.1069	3.2617	4.5504
3.7217	4.0127						
BBS4	3.7366	3.9534	2.7947	3.119	2.773	3.1055	3.9408
2.7955	3.3064	2.9361	3.3691	2.8539	2.1602	3.2542	2.467
4.2074	2.9403	4.135	3.1063	2.7203	2.8077	2.777	2.8987
3.2169	2.973	3.2915	2.5542	4.275	2.8735	3.7531	3.3381
2.8514	3.0503	3.1758	3.3464	3.2574	3.1788	3.6925	3.054
3.332	3.9099	3.3691	2.8225	3.4505	3.1139	2.7357	2.7086
3.5747	2.9018	3.6211	3.011	2.9056	3.4126	3.456	3.2729
3.1134	2.8809	2.3383	2.3527	2.2821	3.1932	2.9893	3.222
3.5572	2.8238	3.5321	3.5174	3.558	3.7845	2.8147	2.8473
2.4682	3.8737	3.0302	4.079	3.288	3.1221	3.5686	3.4063
2.9488	3.0523	3.3128	2.7994	2.5295	2.5902	3.0873	3.5666
3.2764	3.3762						
BBS5	3.3428	2.9642	2.8962	2.6686	0.8384	3.8358	3.608
3.666	3.6905	3.2847	3.2873	2.0431	2.4447	3.1467	3.2302
3.7139	2.2797	3.1662	3.2511	2.1762	2.9757	2.2369	2.4415
2.9879	2.0722	2.5757	2.7386	3.0392	2.5689	3.4689	1.9641
2.3648	3.0355	3.1802	2.7407	3.052	2.8908	3.1534	3.0396

3.7678	3.1079	1.8114	1.6337	1.8415	2.3418	2.7042	3.6379
2.9706	2.5627	1.8989	2.4419	2.1824	2.7753	3.1926	2.1567
2.2553	1.235	2.1079	3.3813	1.8153	2.1271	2.9943	2.3099
2.2677	2.9284	2.5127	3.0123	3.7793	3.7246	2.3444	3.471
2.6442	3.8464	2.0685	3.0011	2.8158	3.0139	3.332	3.1769
3.4472	3.2913	2.726	2.6327	3.0109	2.389	3.0333	3.4966
2.9594	3.3874						
BBS7	3.2425	2.8215	2.658	2.7262	2.6183	2.456	3.1239
2.5786	2.4353	2.7059	2.4438	3.5914	3.205	3.1609	2.7001
2.7706	2.7068	2.8554	2.772	3.2707	2.7072	2.4689	2.3324
2.846	2.2402	3.3046	3.4244	3.2182	1.8762	2.8718	2.9202
3.3837	3.0951	3.3248	2.6655	2.9675	2.9581	2.7167	2.4303
2.498	2.485	3.2858	3.4528	2.6223	2.6303	2.8886	2.9244
2.6393	2.1063	3.2549	3.4119	2.8744	2.4783	2.4612	3.2448
2.6756	3.529	3.0859	3.172	2.8264	2.3955	3.1103	2.8374
2.4347	2.8478	3.5497	3.109	3.6071	2.5065	2.7917	3.0952
2.5897	3.3241	2.4706	2.7822	3.0607	3.0421	2.2126	2.4221
2.6445	3.027	2.9419	2.4271	2.2504	2.6245	2.6255	3.7388
3.2618	3.3968						
BBS9	2.8895	2.3403	1.5506	2.3321	1.8332	2.0127	3.0876
3.1656	3.4375	2.9318	3.4051	2.2851	2.3481	3.0007	2.2339
3.0696	2.4517	3.0627	3.0077	3.1525	2.3099	2.4482	2.9433
2.5435	3.3591	3.3672	3.2132	2.5731	2.2648	3.5645	3.5752
3.1855	2.4426	3.567	3.3535	3.1234	2.828	2.5367	2.8084
3.4245	3.3733	2.5345	3.0397	2.4267	1.8157	3.773	2.8174
2.9026	2.9937	3.2638	3.2782	2.9173	3.6266	2.9528	2.459
2.8946	3.0847	1.9263	2.472	2.4295	2.0824	2.5493	2.0209
2.9072	2.2736	2.9554	3.2227	2.8889	2.6421	3.2488	2.0938
2.8351	3.3262	3.4172	2.6759	3.2888	2.0051	3.0748	3.2826
2.7221	3.0368	1.8447	3.3658	2.4908	2.8146	3.5865	2.8205
2.7277	2.7258						
BBX	3.2693	3.3902	3.217	4.1614	4.1878	4.5681	4.5562
3.4577	4.2876	3.7347	4.552	4.2972	4.552	4.0748	4.059
4.5207	4.388	4.265	3.4009	3.7835	3.7245	4.2868	4.3555
3.5529	3.2138	4.208	3.8066	3.8104	4.164	3.7954	3.2637
4.1505	3.6333	3.7992	4.1466	4.024	3.8545	4.3685	3.9785
4.7811	3.3889	4.3704	3.5821	3.7101	4.3622	4.8363	4.0024
4.5574	4.187	4.5201	4.3689	4.2254	4.51	3.7038	4.2884
3.3742	4.3591	3.1507	3.5329	3.0164	4.0893	3.6858	3.2131
4.4678	3.9723	4.8418	4.2665	4.155	3.9699	3.7715	4.0393
4.2144	3.5773	4.4715	3.6962	4.0994	3.7232	4.5304	4.3904
4.191	4.2109	4.072	3.5329	4.6615	3.2613	3.8371	3.9754
3.4682	3.7307						
BCAM	2.2773	0.774	2.5141	2.0432	2.2017	1.8132	1.6194
1.8896	1.8607	0.4503	1.3824	2.5146	3.3526	1.3016	2.4723
2.5084	0.5623	1.824	0.4115	1.0878	0.9595	2.2637	3.3314
0.235	3.0884	2.4469	3.5166	1.7065	1.8883	2.2621	4.9189
0.465	1.2128	0.6068	1.3698	0.881	1.5457	5.349	2.9699
1.2539	2.7981	2.4243	2.9214	1.3711	4.3426	2.0345	0.5377
1.5283	3.9426	1.3642	1.263	1.2364	1.216	2.1719	3.1509

1.3739	2.6631	1.9586	0.6935	1.6842	2.6302	0.1324	2.4093
0.6234	3.1678	3.6869	1.9521	0.6058	2.0711	3.0817	2.73
1.2214	1.842	2.518	2.4503	0	0.4917	2.8682	1.874
1.8808	2.3797	4.6446	0.737	2.8128	4.9632	1.6309	0.5682
0.8653	0.6725						
BCAN	4.2085	4.5662	1.6326	0.5842	1.4993	1.0137	1.1899
1.2243	7.1285	5.8706	2.8359	0.8334	0.3834	1.6953	0.4914
1.7151	1.0321	4.6742	4.4825	0.513	1.9171	0.7079	0.4988
0.6265	1.081	0.2185	0.8579	5.4331	0.6328	3.2767	1.0306
1.9246	0.5407	5.1153	1.779	3.3779	1.8558	5.4331	2.5329
0.2448	0.6633	0.3521	0.5191	1.0557	0.4025	0.2829	4.7145
2.5855	0.5367	0.259	3.5599	0.2453	2.6849	6.4484	0
5.9912	2.3715	0	0.3158	0.043	0.4725	0.8092	4.1922
0.9056	4.2996	0	3.9528	1.1038	2.2288	2.5627	1.3245
2.3168	5.0799	0.7807	2.7809	1.4691	2.6304	2.7809	6.3461
5.209	4.9922	0	0.0439	3.9283	0.387	1.7316	5.1472
5.7776	6.4263						
BCAP29	3.6588	4.272	3.7935	4.5772	3.328	4.5694	4.4375
4.9645	4.2016	3.888	4.535	3.4481	3.7788	4.532	3.8552
4.0013	3.8223	5.3972	5.4023	2.8998	3.0003	3.8035	3.2794
4.4395	4.6734	3.8243	3.8804	4.7192	4.6067	4.5364	3.2235
3.6545	3.956	3.1544	5.0923	4.2784	3.4057	4.086	4.1649
4.2743	4.3792	3.5384	4.5051	4.4201	4.0017	3.7587	3.7831
4.2185	3.6537	2.9688	3.9517	4.4781	3.5702	4.611	3.8593
5.0449	2.9903	3.8007	5.0149	4.0311	3.8227	3.7685	3.6599
4.2965	3.241	3.3684	3.7148	3.4412	4.2765	4.0528	4.3721
3.9539	5.0781	2.9032	3.7689	3.9048	3.7928	4.4064	4.3142
4.8652	4.6959	3.9408	2.3713	3.837	3.9452	3.607	3.9572
3.6246	3.5718						
BCAP31	7.0677	6.6503	7.699	6.9875	7.6034	7.1754	7.6262
7.0357	7.1461	7.0246	7.0927	6.798	7.0899	6.3732	7.1696
7.8211	6.9315	7.3668	6.5994	7.4908	6.6614	7.5601	8.0153
7.6942	6.8547	6.5464	6.7552	7.3453	7.318	7.5734	6.4428
8.4639	7.1999	7.6124	7.4362	7.1083	6.8174	7.5563	6.3481
7.3967	7.0246	6.7488	7.0622	6.7212	7.2803	6.6334	6.8025
6.825	7.5135	7.6347	7.0017	6.937	6.3187	6.7481	6.7055
8.3423	6.7917	7.293	6.7549	6.9334	6.4056	6.6371	7.616
6.896	6.9093	7.1817	6.6184	7.0309	7.4117	6.5418	7.3155
6.316	7.0585	6.5845	7.293	7.4892	7.386	7.3628	7.3572
6.8778	7.6227	7.5558	7.0208	6.6866	6.2353	6.3489	7.4983
7.0648	6.8359						
BCAR1	3.1926	3.2542	4.6931	2.7566	3.9529	4.3151	3.0074
4.2645	2.5885	2.5374	3.2717	2.1071	3.984	3.3361	3.7876
4.0062	3.4951	2.6113	2.6571	5.3721	4.0854	4.0234	3.4278
3.3872	3.8088	4.5704	1.9849	3.7231	2.8383	2.7864	2.9619
3.7478	4.142	3.5073	4.142	2.1853	3.8742	3.7797	3.5374
1.9774	3.2964	3.6005	3.3267	4.1978	3.5756	3.456	2.5881
3.0561	3.811	3.6796	3.5462	2.1393	2.2887	3.0114	4.0209
2.9331	4.3103	3.9117	4.4866	4.1986	3.7508	3.8537	3.3778
3.8965	2.9463	5.0143	2.9439	5.9087	3.7834	4.313	4.313

4.3615	2.9383	2.8219	4.1014	4.0436	3.908	3.3166	3.3654
3.5112	2.7816	4.1296	5.4795	3.8683	4.7504	3.2937	3.9645
4.2194	3.5726						
BCAR3	2.7011	3.9001	3.1522	4.9587	4.9944	3.9861	3.6364
1.9261	2.5681	3.1538	2.8451	4.5109	5.4277	3.3894	3.1929
4.3861	5.4414	2.892	3.7805	3.7216	2.2857	3.5799	3.1916
3.6199	4.4839	5.4341	3.8553	2.9846	3.2544	2.3637	0.9863
3.2843	3.8778	3.3724	3.9021	2.8925	1.9503	2.0748	3.1522
2.2428	2.2286	5.6095	3.6889	3.6924	3.7161	4.4905	2.9136
1.6181	5.0672	6.4973	2.6795	3.791	2.6126	3.3542	3.5234
2.946	6.2149	2.919	2.813	3.3016	3.1156	3.4599	1.4801
3.6405	1.7174	4.1805	0.9139	3.3946	2.946	4.9796	5.0181
3.6515	2.6611	3.1176	4.4747	1.8443	3.643	3.7681	2.5336
1.9267	2.946	5.0058	5.1374	3.2998	4.7058	2.7891	3.4515
2.4919	2.6						
BCAS1	0.2335	1.6261	0.1857	0.045	0.0499	0.1275	0.0567
0.0427	0.1456	0	0.1891	0.0668	0.0989	0.1051	0.114
0.1213	0.0525	0.2028	0.0653	0.189	0.6857	0.2078	0.6658
0.1241	0.0254	0.1007	0.1091	0.1038	0.115	0.3286	0.0091
0.0801	0.1542	0.1135	0.5258	0.0549	0.3768	0	0.3171
0.1263	0.1622	0.0078	0	0.1247	0.0273	0.0502	0.1567
0.0906	0.0455	0.6658	0.1528	0.3237	0.0637	0.0373	0.062
0.2641	0.0969	3.4768	0.2517	1.1755	0.1153	0.9214	0.0351
0.091	0.0384	0.0173	0.1508	0.0196	0.0236	0	0.0404
0.3267	0.0584	0.3107	0.075	0.0341	0.4012	0.3285	0.0471
0.0467	0.0555	0.0501	0.0871	0.0297	0.0385	0.0708	0.0778
0.2745	0.3091						
BCAS2	5.2488	5.5197	5.002	4.9939	5.1547	4.6439	5.7352
4.66	4.3978	4.617	4.8412	5.131	5.1292	5.9642	4.8695
5.3212	5.1585	4.603	5.4657	4.6322	5.1811	4.7904	4.3395
4.7093	4.4376	5.5288	4.0401	4.782	3.999	4.9486	4.9809
5.9881	6.0269	4.8611	4.9688	4.8212	5.2068	4.9125	4.9
5.0831	4.2012	4.5314	4.3143	4.6495	4.3522	5.4703	4.5264
5.5412	4.4708	5.3206	4.865	5.1606	5.0265	5.9173	4.9649
4.9079	5.2147	5.1219	4.1415	4.931	4.4499	5.0182	4.1027
5.5821	4.6849	5.3554	5.0788	5.1352	4.4869	4.4287	4.3615
5.2272	5.1895	5.0382	4.7952	4.7767	5.6905	4.3914	5.0169
4.4783	5.3524	4.4888	4.9739	3.9536	4.88	5.3668	4.622
4.7032	4.4535						
BCAS3	6.216	4.9568	2.2659	3.267	2.6912	3.4489	2.6035
4.1907	6.0104	5.8779	3.6558	5.5789	2.4959	3.8323	2.2229
2.7411	3.0968	2.8912	4.8094	5.0387	4.1318	3.7073	3.3146
5.67	2.8666	2.5204	4.8202	4.7378	2.9308	4.1709	2.3438
6.8948	5.9578	6.6946	6.0274	4.6266	5.0044	6.5222	4.2258
3.9479	6.3218	2.5695	1.5841	2.3211	2.8513	2.9209	2.1237
6.7355	2.1953	3.0291	5.7656	3.711	5.9424	5.4621	1.861
3.0152	2.3491	3.0228	4.737	2.4396	4.1434	5.5655	4.8485
2.6549	2.9156	2.5768	7.5242	3.1068	3.461	2.1346	4.6108
2.5219	2.6647	5.3585	3.2841	4.8652	2.2378	4.957	5.4142
4.5861	5.728	2.3727	3.7122	7.401	2.6713	6.2582	4.5683

2.9205	4.4628							
BCAS4	3.0395	2.4378	0.8761	1.2864	1.8527	0	0.4798	
2.2713	1.5843	1.6796	2.4092	2.0091	3.2772	0.487	1.7147	
2.3376	3.0524	1.4145	0.6793	1.7437	1.8347	2.3521	2.4213	
0.5292	1.8633	1.9954	3.7449	0.5095	1.2656	1.7516	4.0177	
0.9583	1.2582	1.467	0.9334	2.0082	1.2243	2.3212	0.7767	
0.6736	2.5804	1.8791	3.3013	0.6723	2.2327	1.7431	0.2971	
0.4235	2.0069	2.5398	2.6707	1.5426	1.3321	1.013	4.458	
1.2375	2.4822	2.9177	0.6076	3.0139	1.3648	0.8284	0.659	
0.5887	2.4127	3.7614	1.5666	2.7909	0.9307	2.5829	2.4959	
1.2184	1.9187	1.2713	0.8933	0.3143	0.7915	1.9586	0.6126	
1.0112	0.997	2.0221	1.6922	1.7495	1.9013	2.2392	2.1974	
2.0585	2.5636							
BCAT1	2.1521	0	0	0	4.0219	0.0124	0	
4.4196	0	2.7814	0.443	0.4569	0.0183	0.6653	1.0824	0
	1.53	0	1.8893	0	0.3165	3.6892	0.0763	0
	1.5877	4.6705	0	0	0.9574	1.9627	4.4383	
0.4688	0.5579	0.6259	0.1393	2.3488	2.3212	0	2.9261	
1.9983	0.2637	2.4533	3.1604	0	1.1384	0.798	1.7697	
0.9798	0	0.0054	0	1.678	0.5836	5.2091	2.9471	0
	5.7109	3.3856	2.2013	1.4075	0.1822	2.027	1.1871	
0.1512	0.3918	3.7324	0.1394	0.3625	0.2648	0.1489	0.2561	
0.5381	0.3192	2.6749	0.1838	0.9757	0.1427	0.1896	0.2968	
0.1531	0	0.2705	0.5685	0.1606	2.9198	0.5698	0.8429	
1.045	0.7673							
BCAT2	4.3205	4.5257	3.959	3.1685	4.7066	2.715	2.7415	
3.6564	3.9648	3.4269	4.968	2.1755	4.3253	0.5799	3.629	
2.8656	3.8914	3.2515	2.6687	3.8787	4.0842	3.6764	4.0518	
3.4322	2.9763	3.9928	4.1163	3.878	3.5538	4.2024	4.816	
4.031	4.213	3.7603	3.0567	2.8254	4.044	4.282	4.2106	
3.8031	4.4888	4.6187	3.5965	3.4484	4.055	2.9918	2.2201	
3.4767	2.9658	3.4563	3.4503	2.8733	3.1645	3.6917	4.4004	
3.4099	3.9412	3.6225	3.6232	4.1431	4.566	4.8228	3.7093	
3.5799	3.5452	3.8806	3.3344	1.2506	3.6939	1.7211	4.1721	
3.6333	3.3495	4.2523	2.8274	3.1462	3.5379	3.5383	4.0022	
4.8897	4.3306	3.8664	2.7179	3.224	4.8072	4.4094	4.0796	
4.2925	4.296							
BCCIP	5.1185	5.6629	5.3562	5.6333	5.9693	5.3311	4.8833	
5.2254	5.2591	5.1668	4.5775	5.4512	5.2625	5.782	4.613	
4.677	5.9958	4.7004	5.1222	5.2672	4.7295	4.7066	4.5066	
5.5298	5.3282	4.1384	4.7663	4.918	4.2237	4.4768	5.6533	
5.1235	5.0473	4.4302	4.419	5.9616	5.6762	4.3041	4.7714	
5.1204	4.6838	5.3925	4.9343	4.7931	5.0799	4.615	5.2637	
5.3812	5.1724	6.5523	4.8767	4.8719	5.0969	5.3955	6.6814	
5.1816	4.7584	5.376	5.2301	5.1691	4.9872	4.741	5.5325	
5.2306	5.2661	4.9127	5.3284	4.5923	4.138	4.9733	4.8896	
4.8509	4.5008	5.1006	4.6148	5.0866	5.6843	4.6407	4.3128	
4.0432	5.1822	5.1321	4.6888	4.6172	4.8681	5.4122	4.5531	
4.7137	4.4926							
BCDIN3D	1.9349	2.1111	1.3475	1.7249	1.9682	0.9192	1.1633	

1.0849	1.0812	1.1562	1.5743	1.3065	1.0812	1.3387	1.4903
1.0176	1.3591	2.0198	1.413	1.5911	1.7169	1.3038	1.1658
1.7082	1.2719	1.5187	0.2518	1.8498	1.3591	1.7135	1.4086
1.6948	1.4758	1.1652	1.0377	1.4684	2.5583	1.4494	1.5397
1.3947	1.503	1.6954	0.5681	1.2996	1.3398	1.4593	1.3735
1.0551	0.8869	1.9511	1.1449	1.6003	1.3142	1.5014	1.629
1.4386	1.526	1.9819	1.8073	1.6665	1.4518	1.6206	2.2562
1.109	1.6309	1.5036	1.7856	1.3821	1.3747	1.2844	1.1494
1.0793	1.654	0.9187	0.8664	0.494	1.6047	1.0144	1.5662
1.7128	1.3607	0.7024	0.9033	0.9739	1.277	1.2796	1.416
1.2613	1.3333						
BCHE	4.6511	3.38	3.188	3.2816	1.8476	6.0492	0.6399
4.6838	4.0512	2.9457	1.5305	1.02	0.4548	4.6145	3.9242
4.045	4.6145	4.4294	1.3601	3.2154	3.1835	5.5105	4.5538
1.3601	5.0961	3.8884	5.9846	3.4293	4.4169	3.4544	5.9316
5.3351	6.2584	6.0527	6.0146	3.7021	4.7456	5.6729	4.6186
6.4024	7.1011	0	0	5.5661	0.6682	4.6361	5.1961
5.5782	4.7333	4.5583	3.6826	4.2397	6.1766	4.6271	2.7834
5.1234	6.0452	4.8444	3.2866	4.3222	4.7878	4.0911	5.595
5.8176	5.2087	2.5247	5.3863	0	4.0653	4.7244	4.8491
4.9287	4.7524	0.1553	5.003	4.243	2.9732	4.5135	5.3169
5.0535	1.6287	6.5979	2.8312	6.84	2.0802	5.7449	5.6019
5.3778	4.3795						
BCKDHA	3.9914	3.33	3.1924	2.6959	4.6931	3.0939	3.3135
3.2843	3.6321	2.6034	4.7799	3.3391	3.5237	2.8754	3.8133
3.6945	4.1332	2.8136	2.8612	4.6326	3.119	4.0649	3.916
3.344	3.4536	3.6035	4.1491	3.2805	3.8549	4.4518	4.8682
3.8213	4.1274	4.7757	3.2031	2.2037	4.028	4.5646	4.7331
2.9885	5.0775	4.0479	2.8772	2.7556	2.5954	3.8091	2.3597
3.6084	2.8225	3.6035	3.6145	2.4518	3.1731	5.2597	3.4092
4.12	4.0315	3.0729	3.5264	3.3396	3.1753	3.3272	2.7735
4.54	3.7296	5.0431	2.591	3.8982	2.9423	3.7503	3.3291
4.0937	3.4904	4.2789	3.02	3.0234	3.1863	3.1047	4.8221
4.1067	4.0265	4.2077	3.7958	4.0961	3.4925	3.8879	4.4441
4.3319	4.2796						
BCKDHB	3.7084	3.4037	2.696	3.7562	2.8184	3.3571	3.3023
3.6011	3.7643	3.4088	3.4758	2.6399	3.0386	3.9838	3.3329
3.156	3.3659	3.1593	4.4451	3.225	3.7499	2.5039	3.9941
3.0491	3.3691	3.1194	3.4666	2.6375	3.6945	3.0072	4.6958
3.4332	3.3624	3.5701	3.896	3.3825	4.0164	3.189	3.0397
3.5243	3.8602	4.3856	3.1912	3.7139	3.1452	3.6921	3.4065
3.668	3.4241	3.5582	3.8436	3.4146	4.2945	2.9946	4.0022
3.147	2.8763	3.3261	3.2106	3.4148	2.6167	3.491	3.046
3.3497	3.9777	3.6825	3.3079	3.515	3.0119	3.3354	3.631
3.6929	3.7124	3.3478	3.4552	3.0323	2.832	3.4012	4.706
3.6689	3.4292	4.8527	3.2318	3.5733	3.2468	3.9173	3.9211
3.9042	4.0132						
BCKDK	4.9619	5.3751	4.4245	4.5359	4.9946	3.2259	3.9452
4.7922	4.5711	5.05	4.7488	4.3195	4.8396	4.0286	3.9786
3.4162	4.795	4.2294	4.1809	3.9727	4.5378	4.722	4.7361

4.8183	4.1678	4.9163	4.7525	4.3298	4.8154	4.3835	5.145	
4.3432	4.6461	5.3314	4.5699	4.6253	4.6502	5.3435	4.2714	
4.2025	6.0088	5.1416	4.1999	4.4459	4.1893	4.2531	4.2037	
4.3735	4.1104	4.5528	4.7109	4.227	4.0568	4.1776	4.8355	
4.2515	4.5089	4.482	4.3722	4.3551	5.2224	5.0834	4.9054	
3.9437	4.8931	4.6686	5.5495	4.4239	3.7739	4.4832	5.4777	
4.6594	4.6271	4.7773	3.8529	4.2174	4.2816	4.6301	4.9947	
5.9618	5.257	5.31	4.6066	4.5025	4.4844	4.499	4.8548	
5.1313	5.1146							
BCL10	2.1613	2.4868	2.582	3.0977	2.5658	2.896	3.8935	
2.9971	2.2575	2.3022	2.5033	4.2894	4.3632	2.9901	2.6624	
2.6727	3.6187	2.5114	2.7348	2.8064	3.058	2.3399	2.5254	
2.4221	2.9553	4.2608	1.8671	2.7878	3.0617	1.3591	2.9545	
2.3227	3.2654	2.8161	2.0937	2.7585	3.5484	2.3431	2.3575	
2.2459	2.5611	3.1404	3.3978	2.7192	2.6405	3.6569	2.6179	
2.5703	2.9924	4.7643	2.9631	2.8456	3.2083	2.2299	2.8942	
2.486	4.8289	2.9136	2.2921	3.3101	2.6753	2.4196	2.0138	
2.9901	2.4299	3.7699	2.8573	3.2419	2.7437	3.2904	2.9479	
2.8698	2.8134	2.6758	2.6196	2.1074	3.1794	1.9524	2.63	
2.5174	2.8868	2.8842	3.2787	1.5671	3.9032	3.2007	3.4167	
3.0469	2.7844							
BCL11A	0	1.7481	0.1395	0	1.1364	0.3357	0.4842	
0.1566	0.6715	0.1916	1.1164	0.2505	0	0	0.1553	
0.1141	3.9275	0.8609	0.3476	1.2773	0.3944	0.1152	0.1606	
0.4138	0.5732	0.1563	1.9828	0.3585	0.0655	0.5884	0.2697	0
	1.0201	0.0679	0.1872	0.2468	0.1329	1.1177	0.7453	
0.5191	0.0629	0.1085	3.032	0	0.1351	0.3772	0.9504	
0.2193	0.2114	0.1177	0	0.5528	0.101	1.05	0.114	
1.6041	1.2101	1.3347	0	2.0796	0.4623	0.6265	0.0797	
0.0559	2.6181	0.101	2.5092	0.1498	1.0588	0	0.0292	0
	0.8834	1.7946	0.2601	1.8456	0	0.4827	0.5786	
1.0866	0.3831	0	0.1455	0	0	0	0.6528	
1.0248	1.1725							
BCL11B	0.0598	0.0246	0	0	0.0255	0	0.0171	0
	0.0429	0	0	0.0155	0.1404	0.0361	0.0287	
0.0262	1.9207	0	0	0.0346	0	0.0243	0.2511	0
	0	0.0336	0.0244	0	0	0	0.1	
0.0224	0.0248	0	0	0.0276	0.0288	0.0302	0	
0.0487	0	0.04	0.3634	0	0.1281	0.0222	0	
0.0904	0	0.0502	0	0.0247	0	0	0	
0.4127	0.6659	0.1868	0	1.0583	0	0.0145	0.3531	0
	0.0624	0	0	0	0	0.0087	0	0
	0.1391	0	0	0	0	0	0.2898	0
	0.0581	0	0.0223	0	0	0.0086	0.1629	
0.0398	0							
BCL2	2.3122	3.2613	2.8994	2.035	1.0669	2.5698	1.7844	
2.7056	2.8585	3.047	2.8676	1.569	1.0142	2.352	1.7386	
1.9223	2.3174	2.6892	3.5439	2.3569	3.6848	1.3319	1.5097	
2.3084	1.6993	1.1416	2.1646	2.6581	1.2114	3.1795	0.255	
2.3338	2.1991	2.7394	1.8678	3.0569	1.5563	3.1779	2.8737	

1.3577	2.2483	0.0637	0.8702	2.0605	1.1791	0.3413	3.0693	
1.6959	2.2541	1.2217	1.6773	1.7862	3.0902	2.7814	0.6529	
2.3019	1.0216	3.4031	2.5501	2.3758	2.0688	2.2183	3.1564	
1.9961	3.9934	0.399	4.1789	1.8748	2.1212	1.1548	1.8987	
2.3769	1.9335	1.2336	2.1269	2.3338	2.8162	1.679	2.3203	
1.9271	2.2605	1.4555	2.5611	2.3208	1.3409	2.6757	2.7559	
2.6231	3.0009							
BCL2A1	4.4426	5.9339	6.2689	4.318	3.1577	6.071	6.937	
0.3143	4.6975	6.7856	5.6171	5.9094	1.9232	6.4196	2.3206	
7.867	5.1397	7.3492	6.3183	3.6071	3.7542	4.433	2.9655	
4.4064	5.5822	2.011	2.3957	4.9479	4.9957	4.9595	0	
3.5387	6.6499	6.6434	4.2095	6.5669	4.253	2.8429	0.6363	
5.898	5.6329	0	4.5831	4.8401	0	0.0343	2.0425	
7.3336	3.205	3.7934	3.6222	3.0379	6.329	6.9254	0.5595	
4.46	4.1615	0.5589	3.3283	0	5.7332	3.4863	2.8611	
6.7575	0.5601	1.9106	0.9842	3.7157	7.6014	0	6.0864	
6.1862	6.1676	4.0872	2.5666	7.2218	6.5899	3.4878	5.957	
6.0144	6.0747	1.7027	6.9213	2.186	4.6925	5.6745	6.4882	
5.8142	5.8							
BCL2L1	4.8812	4.7915	5.3679	3.9573	4.9681	5.1046	5.5869	
5.2045	5.9542	5.402	5.425	4.8094	6.5617	4.4047	5.1329	
5.4532	6.2487	5.0099	4.9126	5.0286	4.0847	4.3842	5.0553	
4.423	5.1599	6.6406	3.5527	4.7291	4.9007	3.7369	5.0041	
4.5784	4.3736	6.2369	4.6084	4.7833	5.1263	6.0377	4.5547	
4.0293	4.8919	5.0203	4.0305	4.2159	7.2196	4.7251	4.2043	
4.7566	6.1633	4.8146	4.517	4.095	4.8991	4.9332	5.3474	
4.2679	6.9423	4.5352	3.9215	4.6695	4.131	4.5192	4.4034	
4.6204	3.8054	7.084	4.281	6.2319	4.994	5.2038	5.511	
5.3295	5.4659	3.8073	5.4817	5.7741	4.6194	5.0085	5.2231	
4.9814	5.4026	5.9768	5.6241	4.7245	5.8422	4.1388	5.8833	
4.9304	5.4075							
BCL2L10	0	0	0	0	0	0	0.4166	0
	0	0	0	0	0	0	0.1773	
0.0793	0	0	0	0	0	0.0648	0	0
	0	0	0	0	0	0.1012	0.1751	0
	0	0	0	0.079	0	0	0.2453	0
	0.078	0.3341	0	0.119	0.2518	0.2274	0	0
	0	0	0.4404	0	0	0.3854	0	0
	0	0	0	0	0	0	0	0
	0	0.1367	0	0	0.1864	0	0	0
	0	0	0	0	0.1016	0	0	0
	0	0	0	0	0	0	0	0
	0							
BCL2L11	1.2713	2.8473	2.624	2.7948	1.9187	3.0015	3.2574	
3.468	2.9559	2.5186	1.7176	2.5139	2.8014	2.3378	1.1008	
3.4574	1.3833	2.7814	3.0617	1.3499	1.8448	2.2654	1.6317	
3.0563	1.5186	1.1393	2.9766	2.8444	2.2518	3.4261	2.4966	
3.1717	2.193	1.6434	1.3473	2.1373	1.8163	2.0375	2.9288	
2.6783	2.1296	2.9458	1.6172	3.3698	3.2419	2.3559	2.0347	
3.4179	3.0506	1.8413	3.3101	1.4777	3.0394	2.3362	1.9073	

2.6192	1.0135	2.3557	2.3496	2.9685	2.0105	3.6944	1.648	
1.8049	1.3839	2.3179	1.8867	2.835	4.1059	1.95	2.2127	
2.2418	2.9185	2.454	2.6944	1.8125	2.0484	3.7928	3.3024	
2.8076	3.687	1.9855	2.9742	1.733	1.5911	2.6417	2.0105	
1.6969	1.8223							
BCL2L12	3.6796	4.0771	4.1861	2.8565	4.0413	3.2475	3.0203	
4.0579	3.1553	2.7317	4.0069	3.6458	4.3436	3.0042	3.9743	
3.2117	3.8189	2.5756	3.2117	4.1867	4.0466	3.6845	3.4994	
3.0977	3.5414	3.745	3.4913	3.8115	3.2875	3.5121	3.2509	
3.6926	4.4665	4.055	3.2674	2.8683	4.0296	2.9114	4.18	
2.8537	3.3064	3.0963	3.4086	2.9629	3.5854	3.6699	2.7497	
2.8622	3.4669	3.0247	3.0097	2.9404	3.0547	3.532	3.9572	
3.4635	3.1323	3.5623	3.2842	3.7596	3.766	3.4946	4.3705	
3.7449	3.5778	2.3223	3.2253	2.4045	3.5422	3.6751	3.2546	
3.9269	3.2812	3.9255	2.8866	3.2445	3.8299	2.4041	3.4181	
3.9675	4.0205	2.5977	3.4028	2.7837	4.7282	3.7932	2.676	
3.5938	3.0225							
BCL2L13	4.8976	4.8712	5.0948	4.235	3.7901	4.4265	5.271	
5.2682	4.9339	4.7782	5.25	4.4487	5.0381	4.9156	4.3488	
5.3019	3.7777	4.6474	4.9339	5.0508	4.5149	4.7996	4.2745	
4.9884	4.1108	5.0857	4.5876	6.8326	4.1406	5.5479	3.0087	
5.2419	4.6976	5.3208	4.9812	4.5529	4.9716	5.1275	5.3838	
4.3361	4.5985	4.6069	4.3293	4.4125	4.6065	4.5432	4.8976	
5.3741	4.783	3.3833	4.0271	4.2715	5.4752	6.2747	3.829	
4.1082	4.5159	4.1021	4.5154	3.3985	3.9654	4.6629	4.5125	
5.3199	4.3975	5.0407	5.1161	4.6349	5.7588	3.9954	4.7402	
4.3527	5.0138	3.7218	4.9462	5.0866	4.8763	4.6683	5.03	
4.4435	4.9223	4.7159	4.49	4.8288	3.8286	5.1696	4.6712	
4.1477	4.3655							
BCL2L14	0.0575	0.0884	0.0866	0.033	0.0143	0	0.008	
0.0276	0	0.045	0.0169	0	0	0	0.0065	
0.0252	0.0363	0.053	0.1478	0	0	0.0025	0.0353	
0.0572	0.0054	0	0.0917	0	0.0317	0	0.0108	0
	0	0.0569	0.0295	0	0.043	0.0478	0	
0.0791	0.0319	5.00E-04	0.0518	0	0.0219	0.0143	0.0274	
0.035	0.0346	0.1827	0	0.0558	0	0	0.0868	0
	0	0	0.0958	0	0.0485	0	0	0
	0.0504	0.0187	0	0	0	0	0.0638	0
	0	0	0	0.0475	0	0.0717	0.02	
0.0669	0	0	0.0593	0	0	0	0.0973	0
	0							
BCL2L15	0.3307	0.1312	0.1713	0.2436	0.6484	0.1682	0.1859	
0.0458	0.1034	0.1366	0.053	0.0264	0.0818	0.0935	0.0833	
0.1069	0.3375	0.0269	0.1311	0.157	0.1999	0.0298	0.0048	
0.1966	0.0541	0.097	0.0872	0.1911	0.0576	0.0663	0.1194	
0.0494	0.0381	0.0492	0.0549	0.0229	0.0959	0.0586	0.0573	
0.1214	0.1262	0.0062	0.0704	0.1318	0.0841	0.0828	0.0929	
0.0263	0.0744	0.2119	0.0701	0.2209	0.0804	0.0567	0.151	
0.2179	0.1908	0.0659	0.1117	0.1627	0.5857	0.0718	0.0441	
0.1248	0.1029	0.049	0.1374	0.0585	0.0486	0.0492	0.0417	

0.0271	0.0491	0.0413	0.0656	0.0695	0.1544	0.0673	0.0318	
0.0731	0.2282	0.0481	0.1396	0.0397	0.1822	0.183	0.0487	
0.0392	0.1161							
BCL2L2	3.8077	2.9915	3.2934	3.649	3.5605	3.5264	5.4822	
4.0013	3.5635	3.7542	2.6319	3.8464	3.8946	3.7054	4.0096	
4.4764	3.734	3.9276	3.5425	3.724	3.6329	3.1358	4.3102	
3.5242	3.4683	3.6302	4.3156	2.9806	3.9325	2.8888	3.5728	
3.354	3.9339	3.5917	3.6906	3.9073	3.4161	3.8592	3.7281	
4.0568	3.3484	3.2721	4.1128	3.5365	4.413	3.4839	3.0666	
4.3334	4.7025	3.1827	3.6632	4.3075	3.8173	3.0164	2.8604	
3.2733	3.6428	3.5995	3.2969	3.3345	3.8948	3.4227	3.496	
2.9825	3.2085	4.2467	3.8159	3.7169	3.164	3.8534	3.731	
3.1775	3.0892	4.0822	4.755	3.3615	3.1537	3.8696	3.5077	
3.2268	3.4756	4.0215	5.0303	3.791	2.9007	3.9759	4.3691	
4.0827	4.6683							
BCL3	1.8804	0.4435	1.7396	2.8672	3.5519	3.5007	2.5947	
2.1831	1.8814	1.7313	1.1656	4.4482	4.9615	2.9027	3.6837	
2.7699	3.4008	1.1858	0.7048	2.2295	1.8871	4.052	4.1025	
1.9541	3.7765	4.1597	3.827	1.4604	3.431	2.1999	2.1852	
1.1342	1.53	3.2407	2.7888	2.0177	2.7279	3.4666	3.8422	
2.5861	1.9639	4.3248	3.9845	4.039	3.8127	4.0821	1.1189	
1.6845	3.79	2.1178	3.0815	3.563	2.142	1.8062	3.4949	
1.4848	3.1479	1.7957	1.638	3.3071	3.6646	0.9778	1.7686	
2.6369	2.2587	3.6404	1.1658	2.3548	0.8415	3.6865	3.881	
2.7948	2.6602	4.2375	2.7634	0.74	0.8468	3.8937	2.5572	
4.0571	1.9849	4.4935	2.8598	2.745	5.1644	2.4674	1.9986	
1.4309	0.8862							
BCL6	1.731	1.4341	2.4648	2.0051	3.0018	3.3496	4.1067	
2.2724	2.1434	2.386	2.6594	4.7136	3.4913	2.8589	4.7184	
3.5508	3.8739	2.5077	1.8463	2.3901	2.4326	3.8121	3.3356	
3.2417	1.4023	3.8063	5.4831	1.8756	2.9791	1.9301	2.4113	
3.1008	3.4121	3.8625	3.1952	2.1788	3.2334	3.3627	3.3774	
3.0829	2.8659	3.9548	4.7284	3.4749	2.4551	2.3418	2.6188	
2.9902	3.2722	3.2026	3.1107	3.5238	3.138	1.9962	1.8489	
1.8564	3.4656	3.1746	1.7038	3.7076	2.7533	1.7015	1.4794	
2.8612	2.9489	4.2481	3.595	2.9735	2.1425	2.5485	3.3085	
3.4573	2.5164	3.8742	3.9867	3.5108	2.0889	2.5989	2.5074	
2.8183	2.6137	3.9139	5.2118	3.5454	2.8368	2.6378	2.8828	
1.8306	2.4484							
BCL6B	0.0387	0.0121	0.0307	0.0637	0.0121	0	0	0
	0	0.109	0.006	0.0401	0	0.2273	0.0102	0
	0.0346	0.0538	0.055	0	0.0283	0.0477	0.012	0
	0.0389	0	0.0344	0	0.0062	0	0.2628	
0.0266	0.0101	0.0052	0	0.0175	0.0249	0	0.8156	
0.0056	0.0803	0	0.2644	0.3462	0.0118	0.0071	0.0803	0
	0	0.0624	0.0462	0	0.2201	0	0.224	
0.0428	0.0383	0	0.0235	0	0	0.0157	1.0388	
0.458	0.0412	0.0145	0.0326	0.0119	0	0	0.0286	0
	0	0.0264	0	0.0343	0.021	0	0.0309	0
	0.0175	0	0	0	0	0	0.1193	

0.0527	0.1761						
BCL7A	3.417	4.0513	4.0598	2.9319	1.5584	2.8521	2.643
2.2164	2.5065	3.1108	2.2846	1.855	0.942	3.137	1.8938
2.1457	3.7494	3.3649	3.5716	2.6231	3.3001	1.7238	1.2767
0.9092	1.8166	1.2463	3.417	4.0149	1.2106	3.5621	3.1015
2.6067	2.3634	1.5855	0.801	3.0141	3.2654	3.8681	2.9319
2.4274	3.5241	0.6614	1.7932	3.3008	2.3552	1.0388	3.3587
2.5321	3.1837	2.9353	2.1719	2.6568	3.4248	3.3397	3.6185
4.115	2.3665	3.0577	0.6481	3.2012	2.7136	3.6986	3.337
2.6853	4.4861	1.929	3.6705	2.973	3.2489	1.1202	1.1446
1.9799	3.4542	2.0819	1.8925	3.3386	3.0848	1.621	2.249
3.088	3.0823	1.2596	1.7004	2.0036	1.7583	3.0058	2.7623
2.9903	3.0534						
BCL7B	4.5267	4.465	4.8319	5.3276	5.1021	5.5486	4.9123
5.3792	4.2891	4.1078	4.1182	4.314	5.0421	4.863	4.238
4.4846	4.8938	5.5016	5.3973	4.4378	4.5386	5.1009	4.9383
4.9146	5.1969	4.8215	3.4209	4.2999	5.6686	4.203	4.7828
5.1199	3.8692	3.7259	5.4002	4.3417	4.9543	5.0317	4.866
4.0699	4.5267	4.4471	5.1096	4.192	4.9595	4.8442	4.2292
4.8755	5.6314	3.6976	4.8901	5.1844	3.315	4.6979	5.4368
4.9829	3.5643	5.271	4.4063	5.4205	4.7948	4.4624	4.6198
4.9782	4.8185	4.4685	4.2091	4.4671	4.4635	5.699	4.1
4.7856	5.2834	3.3677	4.6525	4.319	4.9428	4.9391	4.1883
3.7331	4.2997	4.7029	5.7814	4.7979	5.5432	3.7958	4.4644
4.261	4.3502						
BCL7C	4.1023	4.3756	4.8158	3.0314	4.2649	3.6223	2.2802
4.3041	3.4441	3.2862	3.6526	2.4466	4.3843	2.5828	4.6404
3.9846	3.898	2.7128	3.3166	4.2093	4.5204	4.43	4.3254
4.3525	4.0026	3.907	3.3509	3.9241	4.176	3.3567	4.8812
3.6845	4.0405	3.9352	3.9324	2.4792	4.0722	4.3668	3.967
2.5936	4.1825	3.2713	3.752	3.0682	3.555	3.2052	2.687
3.3812	3.7516	2.5264	3.7569	2.9253	2.5482	3.5009	4.7986
3.8705	3.1309	4.8027	3.3902	4.4415	4.3477	4.2874	5.0357
3.1621	3.8343	3.6895	3.1972	3.8619	3.9087	4.1164	3.455
3.8725	4.0454	2.5097	3.2965	3.8946	3.8431	3.5482	3.6178
4.2207	3.571	2.9178	2.8722	3.4815	5.5088	3.7222	3.5827
4.4643	3.9861						
BCL9	2.2474	1.6859	2.7228	3.495	3.4549	4.267	3.7514
3.397	2.9202	2.9587	3.1088	3.4389	2.1994	3.5801	3.8386
3.9893	3.0292	3.1077	2.4143	3.1029	2.6713	3.7615	2.8048
3.541	3.899	2.8709	3.4781	2.1617	3.6595	2.6206	3.6317
3.1289	3.1074	3.0032	3.7833	2.9718	3.0554	3.0296	3.7182
3.8443	3.0882	3.0626	3.7043	3.5381	3.2537	3.4846	2.7731
3.0746	2.4084	2.7339	3.3799	3.7132	3.2351	2.7265	3.081
2.4375	2.6542	3.0309	3.4391	3.2288	3.2534	2.5107	3.5827
3.8919	3.1381	2.4463	2.497	2.9254	2.9352	3.4399	3.3235
3.2297	2.7479	3.1454	4.3578	3.2613	3.0483	4.4099	2.7729
3.1536	2.9156	3.2795	2.9412	4.2625	3.1368	2.7797	2.3639
2.4014	2.4192						
BCL9L	2.7342	1.5148	2.5594	2.6226	4.3503	4.7236	2.1414

2.9841	2.2402	1.9905	1.9358	3.1456	4.7388	2.5923	2.404
3.9349	5.0105	1.8597	1.8879	3.5797	2.6179	3.5654	3.8719
2.9412	2.3481	3.5662	2.792	1.9214	2.9754	1.2963	2.3927
2.3055	2.4302	3.4733	3.3773	1.678	2.4801	3.4606	3.1747
2.1618	3.3927	4.1462	3.3127	3.7216	3.4224	2.7767	1.6762
3.4379	3.1391	3.3839	3.5985	2.6568	1.3978	2.8101	3.7255
2.4626	4.4686	1.4168	2.7349	1.6379	2.7741	2.3424	3.6347
3.5124	2.1482	5.2402	3.5935	3.9812	1.9375	3.6525	2.9171
2.9524	2.6202	3.0138	3.156	3.7846	2.4711	2.3288	2.9529
2.6609	1.6769	3.6213	3.4497	3.0088	3.7533	1.4413	2.7227
2.4427	2.6409						
BCLAF1	4.4928	4.789	4.7596	5.1711	4.9261	5.2792	4.7489
4.7934	4.9795	4.4326	3.6204	4.2352	4.8566	5.3147	5.1639
4.4239	4.8657	4.9529	5.0898	4.4612	4.8329	4.9598	4.6695
4.891	4.9455	4.7696	3.6463	4.3035	5.1128	3.9635	5.2126
4.7644	4.9744	4.0029	4.72	4.5462	4.1561	4.2764	4.4973
5.2064	4.2593	4.3345	4.3442	4.4011	5.4098	5.5118	5.2161
5.1115	4.7648	5.4789	4.9915	5.1763	5.1289	4.7483	5.2986
4.9305	4.8924	5.5503	4.6638	5.8532	4.8095	4.4185	4.918
4.9418	4.6037	4.0993	3.8999	4.3056	5.1401	5.114	4.4773
5.0846	4.5702	4.3703	4.7472	4.2754	4.7364	4.2903	4.524
3.8626	4.8437	4.9174	4.8173	4.7562	4.562	4.8812	4.8502
4.9852	4.965						
BCO2	0.3319	0.1062	0.2651	0.935	0.0937	0.431	0.7586
0.2434	0.3538	0.1234	0.2323	0.3919	0.1419	0.353	0.1761
0.396	0.6359	0.1066	0.4929	1.1482	0.6716	0.1801	0.1397
0.2091	0.3542	0.2057	1.6228	0.3503	0.1599	0.4838	0.1406
0.1824	0.3155	0.7437	0.6195	0.1231	0.0977	0.704	0.118
0.258	0.5764	0.5727	0.3358	0.0531	0.0547	0.2757	0.1257
0.1575	0.1527	0.4648	0.4676	0.3076	0.1604	0.2352	0.1142
0.2297	0.5059	0.1615	0.4148	0.1138	0.163	0.148	0.1192
0.1129	0.1285	0.4258	0.4846	0.3296	0.3513	0.0479	0.548
0.1936	0.2183	0.4119	0.286	0.5395	0.2317	0.5486	0.257
0.3145	0.2613	0.3218	0.1994	0.3976	0.4098	0.2471	0.1555
0.1597	0.1503						
BCOR	3.0025	4.2735	3.4981	3.5023	4.187	3.8447	3.0952
3.5957	3.577	4.0833	3.3191	3.3135	4.1612	2.9728	3.4374
3.8161	2.4016	3.6595	3.2797	2.9677	2.6848	3.4528	3.6899
3.2471	3.5019	3.0126	2.8795	2.9899	3.5472	3.15	4.3516
2.4493	3.0515	2.6978	3.6595	4.0644	3.2561	2.999	3.3878
3.2787	2.4864	2.2322	3.058	3.6336	3.0812	4.2314	3.2867
3.5338	3.1403	3.1472	3.099	3.7831	2.9724	4.3553	4.0247
4.0142	2.9518	4.1774	3.3096	4.2202	3.2495	3.9643	4.0142
3.751	3.1422	3.5268	3.1135	2.5875	3.1189	3.0749	2.6866
3.1419	3.5226	3.5537	3.4065	2.7287	3.0936	2.5542	2.9547
2.6895	3.2051	2.6866	2.7987	2.937	3.8267	3.1487	2.9202
3.1566	3.0712						
BCORL1	2.418	1.4674	2.4972	1.9051	2.6159	2.2253	2.1374
2.4872	1.7341	2.1438	2.6635	3.2145	2.192	1.8759	2.4397
1.9934	1.9998	2.2294	0.9451	2.3746	2.1941	2.8323	3.48

1.6299	2.4529	2.1506	3.071	2.8121	2.3923	2.3157	4.1495
2.1851	1.9945	2.5293	1.3778	2.2299	2.7284	1.904	2.2906
1.2102	2.6229	2.6168	2.7073	2.1083	2.5666	2.0962	2.1221
2.0133	2.8373	2.9183	2.1032	2.6074	1.9813	1.9578	3.8256
2.1279	1.8511	2.7583	2.3929	2.6677	1.658	1.2175	2.2556
2.3471	2.3455	2.355	2.844	2.4622	3.3231	2.5493	2.3281
2.123	2.5806	3.0607	2.5439	2.7901	2.45	2.1062	1.8776
2.3332	1.9596	2.9175	2.51	2.7922	2.4722	1.6851	2.51
2.4809	2.6789						
BCR	2.3839	3.0073	3.4703	2.8732	3.4767	3.2095	2.9056
3.4911	2.3874	3.1132	3.3258	2.7921	3.8624	2.2351	2.7163
3.4353	2.5328	2.5061	2.4981	3.8639	2.4803	3.3118	3.0397
3.3885	3.1181	3.3979	2.116	3.915	2.9876	3.18	3.1016
1.3151	3.2674	3.0021	2.9919	2.9115	2.6282	3.6105	3.6437
2.7629	2.9258	3.5739	3.3797	2.1189	3.4138	2.1499	2.4597
2.5746	3.4403	1.1992	2.3208	2.5589	0.8553	2.981	2.8747
2.9269	3.3959	2.8612	3.3388	2.9252	2.6585	2.5331	2.6747
2.9152	2.9361	3.1955	3.1682	3.8776	3.6657	2.9799	3.3854
2.3344	2.4001	1.6058	3.9813	3.5977	3.2224	3.7654	2.3588
2.9181	3.6091	3.6167	2.8952	3.2607	3.5482	0.5797	2.9106
2.7387	2.9794						
BCS1L	4.8651	4.3254	4.4454	3.6011	3.6386	4.1835	3.1364
4.5486	3.9989	4.0539	4.3711	3.9146	4.1168	4.2437	4.4408
4.0074	4.3962	3.7558	4.1448	4.1661	4.062	3.6085	3.9046
4.6226	3.7374	4.0157	3.7591	4.2661	4.192	4.7046	3.7543
3.4467	4.9162	4.4607	4.0648	3.8042	4.3372	4.1653	3.6941
4.6023	3.8877	4.1792	4.1747	4.5139	3.7739	3.8399	4.0586
3.9259	3.9345	4.2467	3.9085	3.8846	3.8565	4.1752	4.2492
3.7502	3.8634	4.1621	4.5693	4.1461	4.4725	4.3632	4.0674
4.2764	4.4876	3.205	3.4523	3.9721	4.4108	3.57	4.0788
4.1875	4.557	4.1525	4.3047	4.1887	4.4073	4.2714	3.9407
4.6377	4.1835	4.1372	3.7479	3.24	4.299	4.4923	3.9306
4.4394	4.1399						
BDH1	1.7824	4.4751	2.2489	2.4587	1.7123	0.4002	2.7567
2.1098	2.0555	2.4381	2.0063	2.6325	1.6285	2.0125	2.1313
1.8203	0	2.9134	3.5168	3.2594	2.9774	1.9084	2.9947	0
	0.3001	1.3874	1.6742	2.8	2.6819	2.5808	1.7956
3.4278	3.2703	2.6662	1.1376	2.4933	1.8751	1.3454	3.6559
0.0018	3.1134	0.9528	1.6687	1.7164	0.1789	1.7206	2.8884
2.7375	1.3281	2.7057	3.0799	2.4222	2.2809	1.3069	1.6805
2.649	1.3729	1.9508	2.3654	0.9145	2.909	4.0567	0.4002
3.5034	0.6862	0.9491	0.2576	1.6649	2.3473	1.3907	0.3723
2.9402	3.4696	2.1357	1.4265	2.3301	3.4956	0.5205	3.0734
3.431	3.2171	2.539	0.8831	2.77	2.011	2.4892	2.9228
3.9679	3.882						
BDH2	2.9663	3.1058	2.7724	3.1126	2.3937	2.6328	3.2985
2.4277	2.7315	3.7245	3.4058	3.0889	1.6972	2.9299	2.3745
2.1115	1.8734	3.4712	2.7862	2.6196	3.1221	2.0297	2.3365
2.6644	2.8706	3.0165	3.9528	3.4632	2.0053	3.0712	2.9723
3.5051	3.0962	3.2728	2.6851	3.5898	2.7099	4.0853	2.7384

2.4426	3.3708	3.4168	2.5589	2.9002	2.0124	2.3525	3.0853	
2.5619	2.8031	3.2583	3.1986	2.8174	3.3313	2.565	2.4994	
2.6541	2.9236	3.0459	2.7341	2.8709	2.3347	3.3286	3.455	
2.6143	3.3747	4.3914	3.5285	4.3112	2.5494	2.6767	3.0708	
2.9903	3.7697	2.4132	3.0643	3.3641	2.7449	3.211	2.2601	
2.4871	3.0339	2.8558	1.7397	3.1628	1.9414	3.3166	4.226	
4.0278	4.632							
BDKRB1	0	0	0	0.0799	0	0.0749	0.2303	
0.0453	0	0	0	3.0378	0.3334	0	0.0394	0
	3.0357	0.1914	0	0	0.0971	0.8918	0.1078	0
	0.8245	5.012	0	0	0.1112	0	0	
0.6491	0	0	0	0	0	0.0712	0.0301	0
	0.209	1.9479	3.9211	0	0.1367	3.0353	0	
0.2845	0.7268	0.1835	0.1092	0.4534	0	0	0.8144	0
	0.2769	0	0	0.0829	0	0.1506	0.0899	
0.2169	0	2.4712	0.1419	0.0998	0	0	0.4644	
0.2371	0.1417	0.7041	0	0	0.1029	0	0	0
	0	1.1175	0.1929	0.0981	1.1079	0	0	0
	0							
BDKRB2	0	0.0094	0	0.0221	0.0064	0	0.2634	
0.039	0.0057	0	0.0325	1.8866	1.2806	0	0.1368	
0.0011	0.1624	0.0266	0	0	0.0291	0.4037	1.3403	0
	2.4378	2.5911	0.1746	0	0	0.0164	0.0585	0
	0	0	0.0522	0	0.0026	0.1686	0.0472	
0.007	0.0999	4.0317	3.5025	0.0368	1.8805	3.9118	0.0171	
0.0889	4.1437	0	0.2284	2.8515	0	0	2.4036	
0.0804	1.248	0.0381	0	0	0.0981	0.0097	0.0718	
0.0269	0	2.2768	0.0586	0.0164	0.0595	0	1.5718	
0.4793	0.1199	0.8906	0.0756	0.0354	0.0132	0.0474	0.0072	0
	0.026	1.2455	0	0.0791	3.818	0.0523	0.1967	
0.0488	0.0322							
BDNF	1.4069	0	0.279	3.3403	1.5648	0.8325	1.3402	
2.1594	0.0775	0.1042	0.3945	2.2239	3.7111	0.7678	2.3369	
1.5943	4.9415	0.067	0	1.962	1.2951	0.5635	0.6653	
0.1029	2.2542	5.1182	1.4002	0.3653	1.1715	0.0943	0.2455	
0.1611	0.1772	0.655	0.9606	0.277	3.4398	0.4544	0.4591	
0.1512	0.2321	3.0565	1.6118	0.1414	3.1232	1.989	0.0955	
1.2797	2.2081	2.9807	0.3557	0.8894	1.3203	2.2817	2.4612	
1.4708	1.8297	0	0.19	0.1809	0.5164	0.4758	1.1963	
0.1821	0	4.0904	0	1.965	0	1.5251	3.2044	
0.7752	0.1234	2.6792	0.9523	0.0857	0	0.5963	0.308	
0.0681	1.8669	3.0962	3.5707	0.143	2.1737	1.0041	1.0427	
0.1515	0.6642							
BDP1	2.6218	2.3141	3.1619	2.8702	2.823	3.2171	3.0115	
2.8974	2.3722	2.7936	2.5882	2.6408	3.2653	3.0908	2.9029	
2.8793	3.2728	2.6339	3.2054	2.9903	3.0035	2.1915	2.4887	
2.8517	3.1196	2.6774	2.4487	3.2994	2.81	2.3219	2.6802	
2.605	2.6934	2.0894	2.9189	3.0137	2.8119	2.7557	2.1771	
3.0712	2.0195	2.263	2.4959	3.1153	3.2672	3.2636	3.1369	
2.897	2.8754	3.216	2.9766	2.9743	2.8796	2.8224	2.2097	

2.7255	2.9098	2.4519	2.9267	2.464	2.7009	3.3761	3.3119	
2.7076	3.0603	2.6913	3.5953	2.8746	3.4181	2.8318	2.6074	
2.6291	2.9563	2.5068	2.4154	2.9511	2.7958	2.7577	2.5938	
2.8272	2.6105	2.6291	3.1676	2.8033	3.2112	2.8228	2.7464	
2.8517	2.8372							
BEAN1	0.1764	1.1198	0.383	0.1417	0	0.0739	0.2284	
0.0262	0.1949	0	0.4224	0.0164	1.2543	0.1949	0.0383	
0.1068	0	1.3928	0.0757	0.2389	0.0906	0.131	0.247	0
	0.74	0.1139	0.1373	0.5264	0.0748	0.5302	0.1187	
0.0182	0.0314	0	0.0644	0	0.0293	0.9593	0.2147	
0.1437	0.3506	0.1492	2.3996	0	0.4083	0.1896	0.1335	
0.1057	1.7209	0	0.3236	0.1993	0.1603	0	0.1124	
0.82	0.0833	0.0902	0.5185	0	0	0.0045	0.2771	
0.3752	1.3408	0.0045	0.2819	1.6693	0.8341	0.335	0.1352	
0.0306	0.0545	0	0.0291	0.5055	0.696	0.279	0.2588	
0.3046	0.1483	0.2686	0.2155	0.213	0.6696	0	1.7214	
0.577	1.3096							
BECN1	5.7382	5.436	4.2945	5.8614	5.104	5.9495	4.9976	
5.2677	5.8114	5.4704	5.5368	5.589	5.1523	5.8961	5.0538	
5.9463	5.4041	5.6947	5.562	4.7825	5.4603	5.2069	5.4791	
5.7529	5.4076	5.2698	4.8894	5.9859	5.9475	4.831	4.6871	
6.0662	5.4799	5.4569	5.7477	5.3013	5.681	5.111	4.782	
6.5059	5.5547	5.329	5.5064	6.0094	4.6322	4.8968	4.8715	
5.8114	5.4714	5.111	5.5376	5.9352	5.2609	5.2789	4.0988	
5.5117	5.2833	4.8559	5.2906	4.9	5.9233	5.45	4.8149	
5.3003	4.4994	5.1045	5.3098	5.4514	5.4365	6.2607	5.6558	
5.5854	5.6306	5.7171	6.0895	5.7035	5.0028	5.6993	5.7092	
5.3367	4.8782	5.5632	5.4722	5.2034	5.5475	5.1305	5.5555	
5.5306	5.5466							
BEGAIN	1.3753	0.0683	2.3885	0.6074	0.4721	0.0991	0.145	
0.1349	0.765	0.4973	1.5269	0	0.366	0.286	0.1241	
0.7396	0	0.3904	0.4536	0	1.1577	0.356	0.5971	
0.0616	0.9873	0	0.0819	0.1013	0.8266	0.6976	1.1438	
0.2923	0.594	0.1099	0.0455	0.4117	0.8844	0.3973	0.3258	
0.5143	0.2807	0.0685	1.2653	0.3003	0.2459	0.5236	0.5636	
0.3568	0	0	0.1013	0.0728	0.2295	1.8095	2.1682	
2.8683	0.0167	0.725	0.3779	0.9681	0.2922	0.5226	0.9739	
0.1679	1.2357	0.5119	1.0099	0.5639	0.1145	0.571	0.0572	
0.0454	0.4758	0.0247	0.15	0.0135	0.4991	0	0.9573	
0.6402	0.3878	0.041	0.8375	0.1201	0.09	0.3632	0.3437	
1.4364	0.8105							
BEND2	0	0.0253	0	0.0239	0.0055	0.0643	0.0044	0
	0.0069	0.0176	0	0	0.0281	0	0.0206	
0.0171	0.0583	0.0327	0	0.0509	0	0.0045	0	
0.0077	0.0031	0.0331	0.0266	0.0414	0.0181	0.0177	0	
0.0019	0	0	0.001	0.029	0	0.0192	0	
0.0093	0.0079	0	0	0	0.0099	0	0.0312	0
	0	0.0561	0.0182	0	0	0.0091	0.0141	
0.0347	0	0.083	0.0205	0.0451	0.0169	0.033	0.0159	0
	0	6.00E-04	0	0.0027	0.0202	0	0.0056	0

	0.0108	0	0	0.027	0	0	0	
0.0379	0.006	0.0039	0.006	0.0209	0.0199	0	0	0
	0.0562							
BEND3	1.9734	1.8325	1.7347	1.8867	1.2295	1.3135	0.7332	
1.363	1.2877	1.8782	0.9985	0.6451	0.3611	1.437	1.3893	
0.8748	1.908	1.6495	2.1033	1.3905	1.7762	1.2957	1.0598	
1.5767	1.3948	0.9185	0.2526	1.304	1.393	0.6153	1.7831	
1.3168	1.32	0.7815	0.6588	1.7719	0.815	1.1378	1.2464	
1.7226	1.0017	0.4242	1.1028	1.3001	1.4692	1.507	1.8734	
1.4101	0.8456	1.8468	1.2865	1.0478	1.5307	1.3985	2.292	
2.2248	1.4411	1.7757	1.7028	2.2279	1.3847	1.6019	1.7971	
1.4376	1.7294	0.2309	1.1449	0.9283	1.4424	1.2623	1.0105	
1.73	1.2468	1.051	1.1816	1.2898	1.8699	0.8697	0.932	
1.1911	1.4734	0.7399	0.6553	0.6492	1.28	1.3704	0.6968	
1.4512	1.1108							
BEND4	0	0	0	0	1.2895	0.0273	0	
0.0139	0	0	0	0.1278	0	0	0	0
	0.0668	0	0	0.3571	0	0.0087	0	0
	0	0	1.0977	0	0.0166	0	3.3044	0
	0	0.0475	0.0406	1.1061	0	0	0	
0.0464	0	0.0082	0.0649	0	0.0638	0	0.0249	
0.0534	0.0403	0	0.0444	0	0	0	1.8622	
0.4321	0.0392	0.6768	0	2.4477	0	0.0731	0	0
	0.0067	0	0	0	0	0	0	0
	0.1535	0	0	0	0	0	0	0
	0.0808	0	0	0	0	0	0	
0.11	0							
BEND5	0	0.4255	1.2467	0	0.491	0	1.1746	
0.0681	1.8084	0.4815	0.4716	0.0511	0.2944	0.2812	0	
0.7916	0	0	0.7294	0.2269	1.7477	2.1168	0.4996	
0.8917	0.9977	0.2991	0.496	1.1513	0.6712	1.5175	0.5559	
0.3311	1.8077	0.6942	0.1377	0.7069	0.2466	0.438	1.5214	
0.3546	0	0.194	0.1741	1.0422	0.673	0.0809	1.0646	
0.5909	0.7896	2.4619	0.2198	0.631	0.9612	1.1569	0	
0.9295	0	0.8157	0.282	1.5469	0.6253	1.3872	0.5506	
0.4775	0.6551	0.4668	0	1.0762	0.4112	0	0.7404	
1.579	0.8092	0.2447	0.6666	0.6442	0.4158	0.0737	0.7074	
3.017	2.0535	0.6302	2.174	0.0968	1.618	0.5185	0.7671	
1.2537	1.5655							
BEND6	0.3854	0.0409	0.4564	1.781	0.7524	1.2236	0.1167	
0.1591	0.3174	0.0306	0.1463	0.341	1.3041	0.8702	0.8874	
0.1134	1.2454	0.0674	0.8564	0.8516	0.1535	1.9823	2.2546	
0.8678	1.4067	1.8566	0.7056	0.2694	1.8379	0.2907	1.8273	
0.3829	0.7973	0.4834	2.1413	0.7251	1.0885	0.4486	0.6631	
1.3191	0.3998	1.6522	1.6426	0.4834	1.8566	1.6539	0.0452	
0.4532	1.157	1.8495	0.4136	1.641	1.0196	0.1265	1.1065	
0.506	0.9614	0.5054	0.269	1.832	0.5163	0.4177	0.4611	
1.014	0.6637	1.0264	1.1509	1.265	0.2063	1.3453	0.4389	
1.2664	0.5143	0.8457	0.5593	0.4273	0.1342	1.4821	0.3283	
0.4629	0.2808	1.8698	3.1314	0.3125	1.3566	1.0735	0.7663	

0.1244	0.4839							
BEND7	0.0564	1.3186	2.3234	1.0211	0.404	0.4743	1.8783	
0.1664	0.9729	1.9435	2.1314	2.0595	2.5751	2.006	1.4524	
1.4135	0.0231	0.072	1.6188	1.2225	0.0626	1.3063	0.7965	
1.0758	0.6711	1.537	2.8313	2.0363	0.5981	0.7936	2.6193	
0.1011	0.4474	0.1369	0.8	2.1715	2.5187	1.3445	0.8474	
0.9391	0.2083	0.8389	1.3049	1.617	2.1266	0.056	1.1818	
1.2658	1.6125	1.5513	2.145	0	0.0429	1.7857	2.8812	
1.4584	2.7567	1.2088	0.6421	1.3678	0.2683	0.3564	1.0028	
1.7228	1.6597	3.3911	0.3436	1.7971	1.6152	0.7032	0.7509	
0.5362	0.962	0.6126	0.6987	0.8138	0.2206	1.4245	0.573	
1.3594	0.4014	1.4749	1.7303	0.9671	0	0	1.2201	
0.9159	1.7908							
BEST1	6.4217	5.8671	4.1539	2.076	0.2295	4.0794	3.4602	
3.4206	3.4983	6.3001	3.8096	4.128	0.3933	3.4642	1.2788	
3.9149	1.0968	2.8757	4.0401	3.6732	4.9421	1.3921	2.2048	
3.8183	1.6792	0.7862	3.909	4.792	1.0795	4.2009	0.1676	
3.7782	3.7238	4.2298	4.5771	4.9146	5.9626	3.9647	4.9434	
3.2368	4.7133	0.0414	0.0779	5.6666	0.2376	0.103	5.0786	
5.3454	1.3718	0.5795	4.4954	1.7376	4.2446	5.7595	0.143	
5.0677	0.2759	1.5939	5.2053	0.8809	2.6175	4.9316	5.1329	
4.0137	3.4282	1.8549	5.147	0.5023	4.098	0.0943	3.8576	
2.7223	5.043	2.957	1.7337	2.9959	4.3326	4.8122	4.9311	
4.0588	4.3379	1.2753	4.7216	4.6688	0.1075	4.7058	4.7788	
5.1662	5.0726							
BEST2	0	0	0	0	0	0	0	0
	0	0.0412	0	0	0	0	0	0
	0.0884	0.0754	0	0	0.0639	0	0	0
	0	0	0	0.0789	0	0	0.0301	
0.0087	0	0	0	0	0	0	0	0
	0	0	0	0	0	0	0.0754	0
	0	0	0.0417	0	0	0.0273	0	0
	0	0.0454	0.0532	0	0	0	0	0
	0.0563	0.0309	0	0	0	0	0.0315	
0.0316	0	9.00E-04	0	0.0357	0.0175	0	0	0
	0	0	0.0278	0	0.0659	0	0	0
	0.0681							
BEST3	0.0414	0	0.0979	0.2972	0.0948	0.033	0.3144	
0.1394	0.0135	0.0096	0	0.0015	0.1163	0	0.0415	0
	1.5701	0	0.0495	0	0	0.0044	0.0122	
0.1189	0.0075	1.558	0	0.0435	0	0.0191	0.0373	
0.0756	0.0253	0	0.0026	0.0145	0.0845	0.0742	0.0119	
0.0277	0	0.1613	0.0528	0.038	1.0188	0.0611	0	
0.0257	0	1.5195	0	0.0209	0	0	0.3734	0
	0.797	0	0	0	0	0.0162	0.1758	
0.1527	0.0661	0.1612	0	0.0142	0.0358	0	0.2044	
0.083	0	0.0035	0.0677	0.4311	0.0249	0.108	0	0
	0.0202	0.1338	0.0706	0	0.1459	0	0	0
	0							
BEST4	0.1373	0.0897	0	0.0746	0.0295	0	0.0267	0

	0.0297	0.0337	0.0433	0	0	0	0.0218	
0.0221	0.0708	0.0606	0	0.0693	0.1083	0.04	0	0
	0	0.026	0.0455	0	0	0.0406	0	
0.0203	0.0403	0.1615	0.0286	0.0301	0.2353	0	0.056	
0.041	0.0273	0.0589	0.1813	0	0.0814	0.0403	0.0607	0
	0.0586	0.1144	0.0344	0.0439	0.084	0	0.0321	
0.0641	0.0906	0	0.0636	0.0795	0	0.0161	0	
0.0368	0.035	0.1185	0	0.0058	0.0349	0.09	0.0453	
0.0759	0	0	0	0.0716	0.1251	0.0248	0	
0.0461	0	0	0.04	0.05	0.0412	0	0	0
	0							
BET1	2.9437	3.1081	2.4174	3.3259	2.8955	3.8479	4.8074	
4.6374	3.5375	3.0905	4.5187	3.0068	4.2037	3.5331	3.6504	
3.93	3.327	5.1058	3.9404	2.4656	3.0361	3.9549	3.5375	
3.0459	3.2886	4.2284	3.454	3.3089	3.6514	3.5386	2.9666	
3.6406	3.3326	3.969	4.2337	3.2961	3.4212	3.372	2.9715	
2.9817	3.2374	3.1678	3.1112	3.2322	3.2824	4.5372	2.7925	
3.3366	4.6667	3.6094	4.1367	3.6698	2.9636	3.8311	3.702	
3.3191	3.4372	3.1007	3.6669	3.2261	3.4199	3.5126	3.0149	
3.7854	3.4598	4.0383	3.542	3.5996	2.7994	3.6557	3.6801	
3.7898	3.8839	3.2193	2.9198	3.5265	3.3325	3.576	3.3974	
3.8898	3.1717	2.7864	1.6752	3.5662	3.5951	2.8436	3.162	
2.8103	2.7772							
BET1L	4.2488	4.652	4.4558	4.194	4.2479	3.9991	4.4183	
5.5913	4.2683	3.8731	5.4468	4.2722	4.1432	3.6002	4.6648	
4.2713	4.5522	3.2633	3.6813	4.2765	3.9553	5.2389	4.6598	
4.1445	4.9568	5.5092	4.668	4.1742	4.7784	3.5169	4.3978	
4.0613	3.8692	4.5795	4.79	3.7769	3.749	5.4216	4.8071	
4.0363	4.4615	4.853	4.9434	4.2704	3.8058	4.3117	4.004	
4.5338	5.1254	4.0875	5.0334	3.7127	3.8161	4.7896	4.8061	
4.1336	4.2212	4.1173	4.2154	3.8308	4.4339	4.0935	4.4698	
2.5188	4.4165	5.0736	3.8503	4.6364	3.5953	4.354	4.2285	
3.7529	4.8005	5.336	4.154	4.3193	4.0904	4.745	4.4522	
4.6599	3.7157	4.9866	4.8646	4.2753	3.9927	3.9182	4.1131	
4.1328	4.0206							
BEX1	0.1776	1.2861	0.0849	0.1648	0.0085	0.491	0.3728	
0.6306	0.2028	0.3099	0.3319	4.0176	0.8148	0	4.2578	
2.2349	6.6423	0.1648	0.7428	0.1067	2.8119	1.1007	5.3261	
0.9016	0.706	7.2014	3.5131	0	0.7756	0	5.0507	
3.4079	1.299	0.4488	1.4709	1.1469	5.2864	0.3483	0.8552	
0.1961	0	2.2681	0	0	7.2353	4.8637	0.1595	
1.4642	3.683	0.3114	3.7873	0.8023	1.1928	2.061	0.2383	0
	0	5.1559	0.1853	3.3582	0.157	1.5109	0.9127	
0.8765	0.1877	5.7824	0	3.7657	1.2403	0	0.2558	
0.2447	0	4.0067	1.0484	0.4308	0.4748	1.7584	0.2746	0
	0.231	1.9477	2.6985	3.0662	3.7587	1.4865	1.6074	
1.6818	0.3373							
BEX2	0.195	0	0.8029	1.2999	1.5716	0	3.3756	
2.6723	2.1606	2.6016	3.9412	4.0175	2.8338	2.1694	3.0592	
2.6148	0.0482	0.3759	1.3945	1.5968	2.8125	1.9075	2.5905	

1.1725	0.6367	2.6723	4.1001	2.6706	1.4958	3.3173	1.9265	
2.2478	2.8015	2.3723	2.3318	1.7899	5.4463	4.1081	4.0383	
1.0913	0.3451	0.3299	0	1.4822	1.5308	0.5082	3.5135	0
	1.9977	0.1022	3.3586	2.8246	1.8372	0.6225	0	0
	0.2175	3.8991	1.309	2.994	1.8653	0.4055	2.2759	
2.0448	2.0861	4.6234	3.1237	4.0635	2.5776	0.8042	2.1555	
1.5202	1.8481	3.3281	0.7112	3.6985	1.5173	2.2231	2.0812	
0.2558	0	0.5448	3.3724	3.0503	3.044	1.6101	1.8316	
1.2372	0.9847							
BEX4	2.9096	0.2482	0	4.812	2.4173	0.8618	2.5775	
2.6662	3.1234	3.976	4.6728	5.4147	4.5757	3.4959	3.6429	
5.668	2.9312	5.2798	2.7239	0.4879	1.7574	0.0538	3.2977	
0.8085	2.3862	1.8058	6.0726	1.0139	2.8232	4.4268	0	
3.9379	5.8077	6.3221	2.8263	4.3605	4.7184	5.6556	3.8915	
1.6401	0.587	5.4059	4.9842	5.0365	2.0405	0.2215	3.1507	
0.4783	1.4246	1.6954	2.7001	5.6585	3.6762	3.7118	0.3199	
0.3781	6.505	6.4366	1.2174	6.0896	1.0664	0.683	2.4058	
3.1376	2.476	5.6292	5.014	3.5453	3.1812	1.3255	3.783	
3.3999	3.0608	3.1681	4.3902	4.6648	1.1227	5.976	2.9042	
0.4933	0.4105	2.0076	4.0318	4.6594	4.533	3.3316	4.9892	
5.6192	5.6027							
BEX5	0.3202	0.0515	0	0	0.1371	0	0.5445	
0.2026	0.1302	0.145	0.0827	0.5225	0.1132	0	1.2757	
0.1221	1.7563	0	0	0	0	0.3554	0.1063	
0.5254	0	0	0.2552	0	0.0343	0.0831	0	
2.0278	1.0319	0.2242	0.4365	0.0591	1.1551	0	0	
0.0862	0	0.0303	3.1722	0.1704	1.3707	0.0351	0	
0.2577	0.0897	0	0.6635	0.0403	0	0.6022	0	0
	3.0197	0.2611	0.3579	0.6619	0.1178	0.0939	0	0
	0	1.6989	0	0.1763	0.3329	0	0	0
	0	0.5137	0.1842	0.1095	0	0	0.2038	
0.244	0	0	0	0	0.1141	0	0.2387	
0.5936	0.2802							
BFAR	5.173	5.7542	5.5212	5.7504	5.3111	5.14	4.8261	
6.0188	5.5829	4.7482	5.5557	4.6351	5.2816	5.2706	4.786	
5.2582	4.404	5.472	5.3133	5.1655	5.4703	5.4793	5.0788	
5.9401	4.9898	5.3444	5.6561	6.228	5.2582	5.0965	4.307	
5.4137	5.4878	5.3244	5.4769	5.3395	4.785	5.288	5.3936	
5.4271	5.3209	4.7148	4.7228	5.9652	4.9898	4.5767	5.5891	
5.3768	4.9406	4.5818	4.6694	5.1195	5.2112	5.6858	4.0601	
5.03	4.5652	5.2646	5.4981	5.3936	5.7615	5.4777	5.2279	
5.6457	5.165	4.6321	5.6567	4.8027	5.7107	5.4808	5.8191	
5.6526	5.4255	4.9966	5.4024	5.1344	5.3345	5.5356	5.1599	
5.5857	5.0259	5.0924	4.9865	5.6067	4.7763	4.6472	4.9028	
4.7898	5.0586							
BFSP1	0.5357	0.4067	0.2159	2.2337	1.1248	4.1724	1.3394	
1.8452	1.7343	0.1564	0.6889	1.3318	1.8723	1.2791	0.8155	
2.1678	0.6561	1.0432	1.1283	1.6242	0.6217	1.5081	3.0174	
2.6344	2.6281	2.5157	4.385	1.3546	1.178	1.5413	0.5368	
2.9234	1.9703	3.0788	3.5346	0.6809	0.7846	0.6679	1.8931	

2.9198	3.2273	2.0907	1.3968	1.1445	2.2613	1.9808	1.7956	
1.1096	4.3689	1.4128	1.373	3.2298	2.8683	1.3306	0.8276	
0.7206	1.49	0.2896	0.5855	0.7466	1.6879	1.516	1.5018	
2.7629	0.5888	0.8604	1.1492	1.3266	1.9988	3.0781	2.7495	
1.4458	0.7835	0.5968	3.6018	0.7928	1.2346	3.458	3.1716	
2.2328	2.9472	1.3209	2.4777	5.551	1.8075	2.407	0.8107	
0.619	0.6867							
BFSP2	0	1.4714	0	0	0	0	0.0271	0
	0.3155	0.2869	0	0.0136	0	0	0.0216	
0.0633	0	0	0	0	0	0.0137	0	
0.1374	0	0	0	0	0	1.2402	0	
1.7745	0	0.2525	0	0	0.0152	0.1545	0.1932	
0.043	0.6122	0	0	0	0	0	0	
0.2114	0	0	0.0825	0	0	0	0	0
	0	0	0.0865	0	0	0.9733	0.2644	0
	0	0	0	0	0	0	0	
0.0514	0	0.0435	0	0	0.0164	0.1148	2.7993	
0.3325	0.2501	0	0	0.0297	0	0	0	0
	0							
BGLAP	0.0361	0.0229	0.0331	0.0328	0.8656	0.0312	0.0171	
0.018	0.0189	0.0203	0	0.0159	0.0172	0	0.0167	
0.0166	0.0319	0.0285	0.0449	0.0333	0.4732	0.7337	0.0158	
0.0198	0.0164	0.0181	0.0237	0.0363	0.0219	0.0226	0.0174	
0.4192	0.0236	0.0206	0.0148	0.0185	0.0157	0.0283	0.0178	0
	0.458	3.3394	0.0303	0.0234	0.0171	0	0.0294	
0.0255	0.0199	0.0474	1.1581	0.0235	0.5563	0	0.2303	
0.0019	0	0	0	0	0	0	0	0
	0	0	0	0	0	0.5669	0	
0.3095	0	0.0816	0	0	0.7763	0	0.1323	
0.5275	0	1.3336	0.2855	0	0.2597	0	0.0291	
0.0315	0.0371							
BGN	1.4197	0.5609	0.4431	1.2243	0.6353	0.4385	0.0575	
0.4621	0.06	0.5026	0.3674	0.1262	0.2139	0.9388	1.1906	
0.706	0.5118	0.8947	0	0.3002	0.2437	2.4811	1.8708	
0.0955	6.5699	7.6262	1.9104	0.1747	0.2943	0.1224	0.1777	
0.2214	0.6843	0.3717	0.4226	0.0611	2.3971	1.1138	2.0664	
0.7643	0.2698	1.144	4.1811	0.727	0.9256	3.9864	1.4162	
1.2575	0.135	1.1132	0.2919	0.6095	0.8382	0.6349	0.4776	
0.4412	1.1087	0.6088	0.9266	0.306	0.3971	0.5798	0.1166	
0.5551	4.0549	0.8632	0.7292	0.3086	0.3182	5.9007	0.6641	
1.0904	0.1447	0.6602	3.265	0.2417	0.8694	0.2949	0.3526	
0.8923	0.7413	1.0399	0.1058	3.2059	4.6002	0.9066	1.8793	
0.206	0.7392							
BHLHA15	0	0	0	0	0	0	0	
1.135	0.1879	0.1956	0.131	0.1073	0.1352	0	0	0
	0	0	0	0	0	0.1301	0	0
	0.2041	0	0.2638	0	0.1663	0.3576	0.6666	0
	0	0	0	0.1641	1.57	0	0.28	0
	0.1428	0.0905	0	0	0.167	0.2314	0	0
	0.3257	0	0	0	0	0.0414	1.3492	0

	0	0.6749	1.1479	0	0.1598	0.23	0.2757	
0.0925	0	0.1836	0	0.1157	0.0944	0.1193	0.1806	
0.1449	0.2478	0.2877	0	0.0507	0	0.0599	0.0333	0
	0.0604	0.0771	0	0	0.0969	0	0	0
	0							
BHLHA9	0	0	0	0	0	0	0	0
	0	0	0	0	0	0	0	0
	0	0	0	0	0	0	0	0
	0	0	0	0	0	0	0	0
	0	0	0	0	0	0	0	0
	0	0	0	0	0	0	0	0
	0	0	0	0	0	0	0.0878	0
	0	0	0	0	0	0	0	0
	0	0.0418	0	0	0	0	0	0
	0	0	0	0	0	0	0	0
	0	0	0	0	0	0	0	0
	0							
BHLHB9	0.6276	0.2148	0.6396	1.1471	0.5082	1.161	1.8046	
0.6944	0.545	0.321	0.8003	0.9597	0.4416	0.4245	0.8816	
0.7348	1.0549	1.3976	0.2904	0.799	1.8123	0.3548	0.926	
1.0797	0.8502	0.8835	0.6062	0.5476	0.6385	0.7997	1.9151	
0.4558	0.9502	1.5402	0.6307	0.8072	0.265	1.0054	0.3309	
0.843	1.0565	1.1436	0.8454	0.2044	1.6814	1.0908	0.6271	
1.0035	0.8799	1.797	0.8078	1.158	1.2007	0.9012	1.5795	
0.8248	0.6724	0.9712	1.5412	0.9537	0.3802	0.2012	1.3231	
0.9464	1.4248	1.9148	0.9185	0.4009	0.837	0.7889	0.7158	
0.8645	0.6396	1.2341	0.7859	0.5639	0.3251	1.6413	0.8968	
0.4246	0.4196	1.3697	0.5017	1.0173	0.5963	0.7577	1.5239	
0.5763	0.8876							
BHLHE22	0	0	0	0.0997	0	0.0365	0.1459	
0.0159	0	0	0	0.1372	0	0.2654	0	
0.0488	0	0	0.2592	0.0403	0	0.109	0.0434	
0.0246	0	0	0.0915	0	0.1231	0.0401	0	
0.0044	0.7754	0.0423	2.7808	0	0	0.4786	0.0853	
0.1396	0.6298	0.0052	0.4014	0	0	0	0	
0.1471	0	0	0.1732	0	0	0.0171	0	
0.1802	0	0.2144	0	0	0.0514	0.0643	0.0842	
0.0225	0.0917	0	0	0	0	0.0408	0.1622	
0.0077	0.284	0.2985	0.088	0.0348	0.0145	0.0129	2.2835	
0.1347	0.4078	0	0.0247	0.0721	0.0861	0	0	0
	0							
BHLHE23	0	0	0	0	0	0	0	0
	0	0	0	0	0	0	0	0
	0	0	0	0	0	0	0	0
	0	0	0	0	0	0	0	0
	0	0	0	0	0	0	0	0
	0	0	0	0	0	0	0	0
	0	0	0	0	0	0	0.1511	0
	0	0	0	0	0	0	0	0
	0	0	0	0.0263	0	0	0	0

	0	0	0	0	0	0	0	0
	0	0	0	0	0	0	0	0
	0							
BHLHE40	4.7118	4.1432	5.7066	5.1751	4.3794	6.281	6.4529	
3.9273	4.654	5.0116	5.3113	7.1746	6.1939	5.7988	6.0065	
7.0066	4.6653	6.9103	4.9882	6.4368	5.5669	4.7118	5.4946	
5.185	4.5218	4.6866	3.2046	5.0855	6.0615	5.6093	6.5177	
5.4285	5.4293	4.2681	5.7224	5.6463	4.9428	4.7923	6.281	
4.5884	4.4048	4.62	4.6455	5.8731	5.0702	3.6013	5.5799	
5.9354	2.4397	2.9549	5.1971	4.0901	6.1509	4.2016	5.4116	
4.2645	6.8942	3.1081	5.7409	5.0023	5.1633	5.0587	5.1302	
5.5095	3.9701	5.8357	4.5576	4.5654	5.9805	2.7694	5.1115	
6.7795	5.437	5.0116	6.4798	5.9657	4.7693	5.8266	5.4652	
6.6274	4.8532	5.1212	7.3559	4.7656	3.1081	5.2101	5.3113	
4.8498	3.9139							
BHLHE41	3.2506	5.9412	2.2693	0.9826	0.9993	2.1044	2.3609	
2.7262	6.7747	6.5069	6.2037	2.8177	2.6138	3.5127	0.2886	
3.9574	1.2805	6.0334	6.0726	2.3191	3.6067	1.823	4.02	
3.4321	1.914	2.4835	0.4267	4.0871	1.2711	6.0969	3.5301	
4.8931	4.9741	5.4673	2.4242	5.9067	0.8761	5.2733	3.1089	
3.306	3.3218	4.9349	5.3447	2.6919	3.0493	2.1864	2.9482	
3.4584	2.5547	2.3038	4.0357	1.3422	5.6519	3.7297	2.0272	
3.9444	3.632	0.5045	4.8505	0.4013	2.799	5.7752	3.7577	
2.2234	0.9383	5.4451	1.8012	4.114	5.682	0.7818	4.6606	
3.1752	3.5867	3.9569	1.2983	3.9963	4.6001	1.254	5.7041	
5.1335	6.9738	2.4056	4.3949	1.3389	1.132	4.8768	5.7196	
4.247	5.9591							
BHMT	0.0329	0	0	0	0	0	0	0
	0	0	0	0.0055	0.087	0.0305	0	0
	0.0311	0	0.0433	0	0	0	0	
0.0087	0.0253	0.0542	0	0	0	0	0.0216	0
	0	0.0464	0	0	0.1337	0	0	0
	0	0.0867	0	0	0	0.042	0	0
	0	0.0561	0	0.1924	0	0.0689	0	
0.0675	0	0	0	0	0	0	0	0
	0	0.1085	0	0.0035	0.0603	0	0	
0.0201	0	0	0	0	0	0	0.0694	0
	0	0	0.064	0	0	0	0.0279	
0.032	0							
BHMT2	0	0.0192	0	0	0.0628	0.0291	0.0191	
0.0398	0.0392	0.2469	0.0046	0.0223	0.0081	0.1044	0.0188	
0.0361	0.1061	0.0264	0.0778	0.0807	0.0584	0.057	0.0181	
0.0251	0.0052	0.0929	0.0339	0.0637	0.0432	0.0324	0.0507	0
	0.0385	4.7984	0.0071	0.0662	0.0433	0	0.0243	
0.075	3.4997	0.0085	0	0.0569	0.019	0.078	0.0456	
0.0426	0.0306	0	0.045	0.0344	0.0393	0.0099	0.0813	
0.0602	0.1609	0	0.0492	0.0246	0	0.1002	0.0641	
0.0672	0.0315	4.1937	0	0.0308	0.0324	0	0	
0.072	0.0228	0.0271	0.0383	0.1302	0.0627	0.0698	0	
0.0411	0.0154	0.0621	0.0577	0.0712	0.0609	0.0837	0.128	

0.1128	0.0595						
BICC1	2.3601	2.5982	2.4417	2.1871	1.5612	1.8066	2.8324
0.976	2.0632	0.88	1.4167	3.0272	3.8373	2.8073	2.1385
1.7279	1.2539	2.5179	1.4467	2.8883	3.064	2.5234	2.3435
0.8769	3.0745	4.1203	1.453	1.9276	1.9605	1.7843	0
0.4987	2.7381	1.8789	1.908	1.46	1.085	0.684	2.0743
2.4087	0.7675	2.3083	3.1247	2.0768	0.3416	2.5431	2.3346
1.3722	3.1717	1.4956	0.0223	3.6085	1.7113	1.524	0
2.0973	2.1161	0	1.5781	0.2758	2.2382	0.9655	1.6427
2.726	0.8092	4.1744	0.434	3.5479	2.1212	2.4815	2.7877
2.7652	1.4824	1.1417	3.2531	2.1524	2.2623	1.7286	1.7149
2.2885	2.1411	3.498	0.9294	1.4041	2.7164	1.5676	2.0941
1.6893	1.4631						
BICD1	3.2108	2.9535	1.8407	3.377	3.5862	2.4716	1.6767
3.2124	2.9721	2.7658	2.1456	2.632	3.3594	3.0877	3.5085
2.5564	2.7344	3.1784	3.4881	2.6143	2.3973	3.6657	3.345
2.9399	2.9588	2.8787	2.8829	1.633	3.2212	1.7856	2.0312
2.4085	2.1352	2.2716	4.6366	2.5712	2.8431	3.232	3.4492
3.7849	2.3952	3.2907	4.3692	1.873	3.334	3.3042	1.7703
2.9996	2.802	2.8261	3.3688	3.4575	2.8142	3.0331	1.7843
2.2146	3.224	4.3291	2.5224	3.1566	3.2102	2.9824	3.3967
2.9667	1.823	3.0364	2.4516	1.8341	1.8206	3.197	3.4295
2.738	1.962	2.7586	3.7764	1.0599	2.1295	3.5195	2.6903
2.6844	2.8485	4.2744	3.4061	3.7026	2.7246	2.831	2.1832
1.6973	1.7417						
BICD2	4.2605	4.1906	4.2629	4.0818	4.2049	3.8048	2.9526
4.7165	3.74	3.7841	5.2868	3.9777	4.3078	4.5405	4.1109
3.1053	4.1289	3.8223	4.383	4.3316	3.4386	3.2503	3.4974
4.2333	3.6955	3.7096	4.7826	4.183	3.8039	4.3216	0.8372
4.2105	3.7271	4.493	3.4705	4.021	3.6612	3.7966	4.5497
3.8248	4.149	3.7746	4.1307	4.2737	3.6836	4.1061	4.6733
3.2902	4.2131	3.7553	4.1166	3.6969	4.542	3.513	1.3448
4.2231	4.5112	3.8711	5.3758	3.9681	4.2064	4.3003	3.4884
4.044	4.3847	3.5522	3.9272	3.5474	4.4031	4.4107	4.8279
4.1611	4.393	3.8158	3.7274	3.7849	3.8288	3.8188	3.3761
4.0944	3.8012	4.1689	4.1439	5.1894	3.46	4.5576	4.471
4.6718	4.3952						
BID	3.6482	3.7404	4.1026	4.7292	3.7928	3.1638	3.293
3.053	3.5386	2.5277	3.872	3.4104	3.8795	3.5577	4.205
2.5211	3.2122	3.0816	3.9242	3.3753	4.0462	3.9314	4.1146
2.9592	3.3561	3.1922	3.6856	4.2614	3.4634	4.2332	2.5283
3.5142	3.2674	2.3178	2.651	2.866	3.925	2.2475	3.8019
2.6764	3.9235	4.9404	2.8247	2.4352	4.3972	4.4787	4.596
4.1763	3.1847	3.0588	2.6936	3.6563	4.217	4.8384	2.9071
3.6966	4.2319	4.2304	1.9574	4.4275	3.7327	3.4026	3.3054
3.9951	3.6277	3.6118	1.4261	3.9913	3.884	3.0984	3.7464
3.7006	3.7648	2.9228	3.6594	3.7675	3.5245	1.8921	3.7986
4.2309	3.8209	4.1505	2.6577	3.6907	3.2462	4.0718	2.5237
3.2367	2.8121						
BIK	0.2766	2.2055	5.156	1.3736	1.2091	1.1632	1.92

1.4378	0.5291	0.9032	1.0941	0.934	0.4287	2.0256	0.4735	
1.996	0.7731	1.7452	1.5769	1.6136	0	0.736	0.2184	
1.4951	0.3719	0.0734	1.3444	4.3493	0.3421	4.1994	1.9556	
0.3534	0.7039	0.4089	0	1.4167	0.3658	0.7632	0.4664	
0.4115	0.2757	0.2164	0.5823	1.6764	0.4967	0.8949	3.6967	
0.1142	0.4193	0.4341	1.2339	1.5851	0.8547	0.263	1.0877	
0.7866	2.4136	3.1266	2.0407	2.087	0.4257	2.0593	0.468	
0.8671	2.5481	0	2.8721	4.0077	3.8695	0.2774	0.2927	
0.4652	1.7904	0.3505	0.5665	2.1685	2.5452	0	0.1602	0
	0.7808	0.0741	0.0473	0.802	0.4117	0.7317	0.2696	
1.4301	1.131							
BIN1	3.4266	2.0292	3.5644	3.1674	1.4054	3.84	2.7704	
4.4428	2.6112	2.9893	4.1781	2.2707	4.9672	0.8505	1.3436	
4.0784	1.483	1.8038	1.7062	2.1273	3.4575	3.7651	4.5035	
2.9549	3.9664	4.2203	3.9901	0.7339	3.2054	4.1215	3.2009	
1.3016	2.363	4.4269	3.1582	2.2925	3.6757	4.7216	4.5868	
3.2358	4.3959	5.1887	4.1454	3.8772	3.9783	3.5059	0.4112	
4.1392	5.1989	3.7063	3.927	3.2866	4.2577	4.4339	3.6294	
3.875	3.8875	4.0059	2.628	4.3796	1.5474	2.0507	0.1495	
3.8914	2.7437	4.2239	4.2897	4.4748	4.5639	4.3503	4.2897	
1.8704	1.1758	4.2864	4.1735	4.1047	2.5519	3.5884	0.6115	
2.4183	1.8684	3.8947	3.8836	3.8289	4.9162	4.5355	3.2173	
3.7769	3.4961							
BIN2	0	0.2013	0	0	0	0	0.0101	0
	0.0145	0	0.0371	0	0	0.0477	0	0
	0	0.0369	0	0	0.0708	0	0	0
	0	0	0	0.0493	0	0	0.029	
0.1019	0.1032	0.0451	0	0.0796	0.0206	0	0.0105	0
	0	0.0014	0	0.0236	0	0.0189	0	0
	0	0	0	0	0.093	0	0.2739	0
	0.0455	0	0.0173	0	0	0	0	0
	0	0.0024	0	0.0118	0.0781	0.0373	0	0
	0.0171	0	0	0	0.0486	0.0154	0.0187	0
	0.0668	0.0205	0	0	0	0	0	0
	0							
BIN3	6.9935	5.0339	5.1607	4.423	5.1235	3.3384	4.5425	
5.2854	4.5437	5.4106	5.9999	4.6947	4.0181	4.6297	4.2372	
4.1828	3.0711	5.3161	4.1956	5.1208	5.6981	6.0427	5.0837	
5.1164	5.6236	3.6247	4.423	4.3832	5.4897	5.3189	3.6051	
5.4707	4.7234	5.2414	5.7203	4.7258	6.1327	5.3284	6.7743	
5.1568	5.7827	2.9753	4.8522	4.7011	3.3427	3.2551	4.8076	
5.3955	5.881	3.1971	5.032	4.2764	5.6777	3.9463	3.3093	
5.5655	2.6548	5.2917	5.5944	5.7292	4.9589	5.4529	5.906	
4.4678	5.8258	2.5513	5.8956	4.0161	3.8481	6.3442	4.859	
5.297	5.8754	5.7701	4.2576	4.3914	5.0655	6.1156	4.4001	
4.9203	4.727	4.2005	4.6133	6.0143	5.2183	5.8338	5.3962	
5.6436	5.6244							
BIRC2	3.7258	2.9129	3.161	4.4716	5.0031	5.7165	6.1257	
3.6439	4.1178	3.3498	3.5022	4.4361	5.3286	4.9938	5.0533	
4.8509	5.1554	3.6811	3.7345	4.4272	3.1758	5.6106	4.4252	

4.1024	4.127	5.5814	4.5851	3.5491	4.4616	3.9294	3.9274	
3.9232	4.7378	4.257	3.4514	3.6947	5.4388	3.2061	4.7974	
4.3254	4.4815	4.5017	5.6068	5.8932	4.6273	6.8163	3.4376	
4.6901	5.505	4.4444	4.696	5.3536	0.7788	4.5856	4.2927	
3.7499	4.7583	3.1545	3.8359	3.84	4.5436	3.5221	4.1007	
4.5875	3.7973	5.124	4.149	4.905	3.941	5.1427	5.8513	
4.5888	3.5872	4.5153	5.634	4.5649	3.4883	4.1933	3.8494	
3.9602	3.5037	4.4383	7.6951	5.0184	5.1296	1.1189	4.357	
3.7013	3.9064							
BIRC3	0.1796	0	0.3073	1.6756	3.8617	0.0379	4.2489	0
	0.9869	0.0477	0.3078	4.622	3.5085	1.4385	2.9892	
2.0397	3.3744	0.4632	0.7825	2.0757	0.12	2.908	0.8077	
0.2999	0	2.9719	1.6422	1.003	0.0308	0.993	0.2142	
2.0068	0.0329	1.131	0.3367	0.1823	2.4761	0.1416	0	0
	3.579	2.4131	3.4009	2.131	1.2481	3.4342	0.2458	
0.8628	4.0137	1.6252	3.5372	3.2805	0.0166	0.5905	0.8484	
0.2859	3.379	0.3772	0.513	0.3026	0.8029	0.6165	0.3594	
0.5138	0.2859	5.4385	0.2758	2.1523	0.3349	0.6303	1.0607	
1.2097	1.1257	3.5298	1.2301	1.1464	0.7874	1.1631	0.5937	
0.5894	0.2563	1.1263	4.9845	3.9295	1.0325	0.2718	0.0883	0
	0							
BIRC5	5.5731	5.361	5.9668	5.6386	4.6168	4.9726	2.5789	
5.3558	4.7803	4.6589	3.9807	4.734	5.5119	5.1037	5.6394	
4.2919	4.7623	4.3298	4.6903	6.9724	7.005	5.0091	4.2434	
4.9777	5.0318	3.3725	3.4428	5.5755	4.9249	4.5338	5.1767	
5.5973	5.6419	4.8414	4.8741	5.258	4.6168	2.8974	4.3701	
4.913	4.3765	3.5876	5.6075	4.1542	5.9328	5.5982	5.5393	
4.9396	3.9359	5.3278	4.8782	4.3957	4.15	3.9735	6.5173	
5.532	4.7367	6.0398	4.5803	5.6936	5.3202	5.1194	5.8698	
4.7023	6.0456	2.5162	5.2843	2.0116	4.9673	5.6008	4.6133	
5.1924	5.56	4.6081	4.8163	5.3453	5.5697	4.3261	4.8413	
4.3829	5.3703	3.4427	3.0615	5.2198	5.5893	4.8937	3.5917	
4.6422	4.503							
BIRC6	3.0586	2.7915	3.0078	3.5078	3.2146	3.3972	2.9511	
2.4304	4.3815	3.9768	3.6147	3.3288	2.7004	3.594	3.0819	
2.9682	3.6974	3.793	3.3785	2.5844	2.8931	2.7124	3.2193	
3.1276	2.8304	2.6613	2.9476	2.7614	3.1098	3.348	3.142	
3.1172	2.7978	3.1333	3.159	3.676	3.4257	3.2372	2.9051	
3.9163	2.891	3.2091	3.5095	3.6243	3.9534	3.1465	3.8017	
3.3491	2.9387	3.4334	3.17	3.3091	3.3429	3.3745	2.7834	
3.0272	3.4497	3.1224	3.0608	3.0216	2.9308	3.2536	3.2374	
3.2201	3.3292	3.3311	4.0509	3.1333	4.0528	3.1209	3.1616	
2.8043	3.3121	3.1326	3.0916	2.9452	3.1089	3.7291	3.2326	
3.0024	3.14	3.4133	2.9894	3.3027	1.744	2.4753	3.172	
3.0364	3.1257							
BIRC7	6.6348	6.4191	7.2868	0	0	2.1459	0	
6.2253	5.0474	5.6822	3.5216	2.6395	0	0.3008	0	
5.8193	0	4.8623	4.2383	0	5.9741	1.8154	0	
0.2418	0	0	0	0.7007	0	6.253	0	
4.3703	5.7187	4.6768	0.2069	4.5817	5.9712	7.9306	6.6348	0

	0.4738	0	0	0.1786	0.5144	3.5528	5.1821	
6.5816	0.198	0	5.0942	0	5.9111	5.6678	0.0012	
4.438	0	0	7.0901	0	0	4.6902	5.8633	0
	2.2638	0	5.7694	3.0749	5.2469	0	0	
0.6623	0.6409	5.0252	0	6.6484	5.8992	4.214	6.3367	
2.3835	5.2189	0	6.2528	0.7941	0	6.5654	4.3699	
5.314	4.1965							
BIRC8	0	0	0	0	0	0	0	0
	0	0	0	0	0	0	0	0
	0	0	0	0	0	0	0	0
	0	0	0	0	0	0	0	0
	0	0	0	0	0	0	0	0
	0	0	0	0	0	0	0	0
	0	0	0	0	0	0	0	0
	0	0	0	0	0	0	0	0
	0	0	0	0	0	0	0	0
	0	0	0	0	0	0	0	0
	0	0	0	0	0	0	0	0
	0							
BIVM	3.5565	3.2309	3.3694	4.0247	3.9828	3.3376	3.4636	
3.5968	3.4851	3.329	3.6465	3.2037	3.5763	1.3914	3.3084	
2.8314	4.3088	3.2599	3.6601	0.2096	3.6443	3.2312	3.2706	
3.395	3.6948	3.4614	3.5579	3.4043	3.1299	3.7317	4.8463	
0.5435	2.6252	3.3328	3.7741	3.7418	2.747	3.0724	3.638	
3.2759	3.7713	3.805	4.3467	3.6363	3.2226	2.3973	3.1923	
2.5159	4.5067	3.6163	3.6229	4.0394	3.5053	3.3669	4.2069	
3.8617	4.0128	2.6071	2.5263	1.8591	3.3761	3.5957	4.1881	
2.9366	2.8176	2.7979	3.6389	3.0526	3.8064	4.2296	4.0295	
3.8732	3.2439	2.9044	0.6154	3.1687	2.6793	3.4181	4.0101	
2.8141	3.5744	4.1361	2.8548	3.9441	3.0522	3.1638	3.4789	
3.088	3.5946							
BLCAP	5.1245	5.2122	5.0541	4.1137	4.6897	5.3272	6.6827	
5.6682	5.5452	5.5253	5.4842	4.8924	5.9378	5.2191	5.1227	
5.8229	5.2868	5.0423	4.9158	4.5728	4.6614	4.3741	5.6188	
4.9267	5.1129	6.3502	4.8937	4.7323	5.2636	5.1286	4.3917	
5.2291	6.3645	5.7948	5.1782	5.1864	4.7091	5.2386	4.8489	
5.6066	4.8294	5.3052	4.2405	4.8646	6.0433	5.593	4.7973	
5.2936	5.4869	5.5175	5.0889	4.8315	5.3486	5.7284	4.6511	
5.1374	5.3966	4.3772	4.9884	4.5573	4.673	4.9307	4.4629	
5.2753	4.5549	5.8613	4.7353	5.1956	4.403	4.7	5.8463	
4.8295	6.5363	5.1065	5.3345	5.4857	5.1521	5.5534	4.7334	
5.3352	4.7672	5.6428	5.2501	4.9129	4.7916	5.0186	4.7644	
4.565	4.6037							
BLID	0	0	0	0	0.4385	0	0.5665	
0.0761	0.0778	0	0	0.1868	0.137	0	0	
0.0725	1.2144	0	0	0.1892	0	0.0873	0.045	
0.0457	0.1065	0.0796	0.649	0	0	0	0	
0.0147	0	0	0.1437	0	0.1608	0	0	
0.9311	0.0414	0.3877	0.4798	0.0556	0.4891	0.0397	0	0
	0	0.2449	0	0.1218	0.08	0	0	0

	1.0709	0	0	0	0	0	0	
0.1701	0	0.2613	0	0	0	0.1874	0.1035	0
	0	0.1256	0	0	0	0	0	
0.1962	0	0	0.3389	0.2983	0.1302	0	0	0
	0							
BLK	0.2486	0.1408	0.3272	0	0.0539	0	0.2921	
0.0591	0.2086	0.4293	0.3681	0.2511	0.8109	0.1713	0.0665	
0.0628	1.3812	0.1432	0.2785	0.2173	0.1153	0.1507	0.1103	
0.0425	0.0477	0.1904	0	0.3424	0.1674	0.0445	0.174	
0.0831	0	0.1244	0.1635	0.1526	0.0376	0.1104	0.1401	
1.0432	0.051	0.0335	0.2925	0	0.018	0.3546	0.1161	
0.0528	0.0867	2.4551	0.2753	0.7113	0	0.1034	0	
0.2452	0.8833	0.0294	1.4481	0	0.0282	0.6374	0.0498	0
	0.6712	0	0.8143	0.0443	0	0	0.8966	
0.1649	0.1442	0.3425	0.0161	0.0224	0.0581	0.1242	0.0507	0
	0.0958	0.0176	0	0.2698	0.0605	0	0	0
	0.0622							
BLM	3.0088	4.1616	3.5105	2.9664	2.7254	2.5383	1.9138	
2.922	3.0707	4.2356	2.3923	2.1234	2.6817	3.2192	2.4154	
2.3225	2.3685	3.5404	4.1809	2.8024	3.1356	3.1468	1.9986	
3.0795	2.7478	0.9455	0.6694	3.2314	2.8016	3.3414	2.6667	
2.618	2.8858	1.6685	2.2418	4.6171	2.7768	0.9037	2.8433	
2.3159	1.5409	0.9881	2.2662	3.5552	2.9179	3.2044	4.1023	
3.0811	1.3623	4.0022	2.3882	2.7298	3.4914	2.9845	2.621	
3.4305	1.9013	3.2193	2.9355	3.4712	2.9879	3.196	3.1913	
3.9088	2.4709	0.3566	2.9171	0.9776	3.6291	2.7752	1.7335	
3.1348	3.4434	2.297	2.3004	2.434	3.4984	2.1481	3.0395	
2.9618	3.5145	1.1531	0.9985	1.5703	3.4052	3.6476	2.3274	
2.6363	2.9224							
BLMH	4.5814	4.2863	3.9252	5.2576	5.0482	4.7058	3.2731	
3.6477	5.0924	4.7204	4.9917	4.8697	4.1667	4.6317	4.2358	
4.0191	4.4334	4.8824	4.8059	4.3382	3.3163	4.5534	4.3153	
4.7354	4.8388	3.642	5.8881	4.7233	4.4856	5.1613	4.1357	
4.6327	4.5065	4.3361	4.9767	5.1355	3.7769	4.2569	4.3671	
5.4344	4.4958	4.2956	4.2041	4.9634	3.5754	4.6312	4.6378	
4.6682	3.8401	5.1662	4.4194	4.4782	3.836	3.7725	4.2577	
4.0895	3.2024	5.338	4.4396	5.0738	5.3116	5.0406	4.5069	
4.6122	4.464	3.2144	4.5911	3.2148	4.6032	4.8554	4.4856	
4.853	4.3416	4.3352	4.8692	4.5454	4.7759	4.8706	4.8487	
4.4428	4.2311	4.2063	3.3772	5.3007	4.6756	4.0583	3.9684	
3.8257	4.3526							
BLNK	0	0.0596	0	0	0.0157	0	0.3639	0
	0	0	0	0.0182	0	0	0	
0.0236	0	0.0723	0	0.0967	0	0.0491	0.1052	0
	0.0242	0	0.047	0.1041	0	0.0458	0	0
	0.055	0.1394	0.0751	0	0	0	0.0362	0
	0.0432	0.321	0	0.0558	0.0979	0	0	0
	0	0	0	0	0.108	0.0389	0	0
	0	0.0293	0.0701	0.0446	0.0322	0.0171	0	0
	0.0556	1.2534	0	0.0654	0	0	0.0175	0

	0	0.0178	0.0309	0.0295	0	0.0216	0.0133
0.0388	0	0.0511	0.0367	0	0	0.0445	0.3506
0.0853	0						
BLOC1S1	6.4454	5.8688	0.9412	5.194	5.836	4.9244	4.6491
5.7495	5.2436	5.831	6.3486	4.7747	5.2296	4.8134	5.5222
4.578	5.8	5.9809	5.8093	1.4571	5.5993	5.5631	5.8168
5.4494	5.77	5.0289	5.6834	6.1587	5.7344	6.1912	6.387
6.4382	5.275	5.5103	5.9326	5.4444	6.3003	5.7495	5.9621
5.1394	6.1725	5.9443	4.8646	5.9164	4.6078	5.4745	4.8892
5.91	5.2639	5.2189	5.2415	4.9695	5.1328	5.35	6.1246
5.0508	5.0785	6.009	5.7945	5.2739	5.8244	5.926	7.538
4.9968	4.8303	5.5147	7.4198	5.9313	5.0878	6.8261	5.093
4.7952	6.0527	5.6235	4.0298	4.6178	5.1384	4.9715	5.7106
6.0905	5.6465	5.2341	5.9812	5.5742	5.4042	5.3422	6.0267
5.9145	5.7929						
BLOC1S2	3.8995	4.7119	4.4573	3.8566	4.3672	4.4277	4.0943
4.5741	4.3195	3.9375	4.2656	3.8326	3.6988	4.5238	3.2354
4.1834	4.1548	4.645	4.1777	4.2274	4.9013	2.8999	3.6627
3.9096	3.7317	3.1487	3.8603	4.1768	3.128	3.0556	4.4474
3.6187	3.5039	4.5213	3.441	4.6559	4.3514	3.2286	3.0939
3.6826	3.6279	3.2498	4.2694	3.4705	3.9247	4.2616	3.9236
4.133	3.497	3.9955	3.5507	3.8405	3.9411	3.5315	4.5108
3.1218	4.4755	3.6648	4.0105	3.4528	3.7067	4.3491	3.5824
4.1112	3.4257	5.0856	4.1397	4.8054	3.7784	3.9663	3.759
3.709	3.395	3.3553	3.5729	4.187	4.4851	3.6002	3.154
3.7472	4.0045	3.6533	4.7752	3.1368	3.4931	4.1812	4.7871
4.3409	4.1089						
BLOC1S3	2.6012	2.712	2.6981	2.4109	2.1381	2.6499	2.6679
2.1826	2.4052	2.6366	2.6544	2.5517	3.3337	3.1951	3.0995
2.7403	2.4744	2.7691	2.4331	2.52	3.0929	2.1323	2.6862
2.1391	2.7333	3.0695	1.4441	2.8949	2.899	2.8307	3.3401
1.848	3.1792	3.4947	2.542	2.7905	3.258	2.5745	2.5731
2.7384	2.1335	2.1198	2.4488	2.487	3.1567	2.6836	3.2754
2.2996	2.2571	3.2235	2.6927	1.5863	2.5035	2.9958	2.6526
2.8562	2.8017	2.5696	2.1795	2.8503	2.7594	2.527	2.95
2.6224	2.6086	2.6808	2.0874	2.8631	3.4845	2.4539	2.7737
3.1233	2.6606	2.3436	1.6223	2.977	2.3129	1.9369	2.7153
2.7364	2.4939	1.9635	3.588	2.2834	2.8734	2.4961	3.1845
2.6389	2.7563						
BLOC1S4	2.8241	3.0055	3.4885	3.432	3.4064	2.1872	2.2683
3.6248	2.4129	3.1779	2.7669	3.6099	2.2103	2.7046	2.4009
1.6826	3.5531	2.4369	2.1347	2.8863	3.4198	2.9194	2.4645
3.3287	3.0079	3.4007	2.6389	3.0584	2.2882	3.2046	2.962
3.4863	2.9188	2.6862	3.0529	3.0799	3.9118	3.9181	2.9533
2.7184	2.2882	3.5072	3.2581	2.8552	2.4125	3.567	3.1373
3.1176	2.3332	3.6401	2.6319	2.9776	2.8984	3.7594	3.125
3.3672	3.7572	2.9472	2.2134	3.2941	3.0102	2.8802	3.1418
1.915	3.3934	3.5541	3.3185	3.5913	2.9623	2.9821	2.709
2.6457	3.3226	3.289	2.6312	3.937	3.1959	3.125	2.2762
3.543	3.0365	3.0052	3.2522	3.1851	3.5825	2.5549	2.929

3.0336	3.0854						
BLOC1S5	3.2927	2.9831	2.5057	3.1808	1.9442	2.7692	1.6909
2.8096	2.6736	3.2123	2.8234	2.5524	2.7522	2.4925	2.4942
2.6381	2.7434	3.6526	2.9762	1.7747	2.9906	3.452	2.1699
2.1649	2.6705	2.7305	2.7891	3.5286	2.4143	3.0587	2.5681
2.5359	2.513	2.5774	2.0996	3.2085	2.9623	2.7703	2.143
2.3477	2.646	2.4729	1.9067	3.4698	2.4378	3.4669	3.5555
2.672	2.4639	3.8832	2.6888	3.2853	3.0954	3.2757	2.7484
2.5348	3.2175	2.7037	2.8863	2.4846	2.4219	2.75	2.4974
2.4692	3.4618	1.1906	3.6234	2.333	2.6109	2.5024	2.5283
2.4918	2.6282	2.5753	2.8768	2.587	3.1299	2.7606	3.3522
3.3576	2.8111	2.9067	3.0619	2.1819	2.5595	3.1408	2.88
2.5559	2.7787						
BLOC1S6	4.1998	5.0261	4.3059	4.4411	4.2128	4.879	4.8748
5.1139	4.8843	4.6723	4.668	3.8662	3.3778	4.1481	4.0949
5.0591	4.3966	5.7517	5.0865	4.3763	5.0723	4.5428	4.6309
4.5816	4.8319	4.3639	4.175	4.6813	4.6422	4.1746	4.5225
4.3603	4.0802	4.2475	5.2432	4.7181	4.1443	4.1174	4.3753
4.3137	4.1279	4.2896	4.5081	4.4577	4.9015	4.9597	4.6093
4.912	5.0993	4.3833	4.5428	4.3938	5.0174	5.2978	3.9921
5.1001	4.8066	4.2421	4.5007	4.254	4.4348	4.3346	4.371
4.846	4.5461	4.2406	4.7758	4.9107	5.1346	3.9975	4.0351
4.3953	4.7435	4.2332	5.1225	4.8849	3.3088	4.5399	4.7479
4.0725	4.87	4.5824	4.6114	4.7384	4.5056	4.7975	4.8807
4.8136	4.8748						
BLVRA	5.8723	4.7811	5.7063	4.7978	4.7619	5.2737	5.6668
5.0511	5.9478	4.9063	6.2567	4.7503	5.5224	6.1535	5.2596
5.8051	4.0094	5.2533	5.1636	6.3897	5.8331	5.5514	5.3956
5.4564	6.4498	5.3202	5.6198	6.5228	5.3733	5.5997	5.391
4.5767	5.5745	5.8691	5.6032	5.3273	5.1154	4.3107	4.5787
5.664	5.7583	6.1927	6.4679	4.6658	4.8862	5.5719	5.6734
5.4267	5.8929	2.2862	5.5971	4.8721	5.4376	4.6855	5.8599
4.9851	5.1137	5.3038	5.439	5.1365	5.0402	5.1884	4.3498
4.7962	4.4804	5.1347	5.5529	6.9956	6.1313	4.9913	5.8287
5.701	5.7078	3.9794	5.2004	7.0039	4.8928	4.4979	5.3008
5.4441	5.7856	4.9851	5.1265	5.2479	5.4874	6.1429	6.0916
6.3297	5.7524						
BLVRB	5.5973	4.539	5.4058	2.9689	4.5478	4.4802	5.2575
5.2767	5.0038	4.0711	5.8662	4.1826	6.4319	3.6122	4.6376
5.5383	4.8985	3.2123	3.6768	5.8853	5.2034	4.4336	5.6308
5.046	4.7833	4.5478	4.7458	5.8115	4.3094	5.1708	6.6064
5.3825	4.9502	7.2339	5.6247	3.9078	5.8971	5.858	4.8855
4.017	5.9147	4.9252	5.5169	4.299	3.8535	4.7577	3.4033
4.9294	4.5593	4.0884	5.0818	2.7838	3.6022	4.0904	6.8674
5.101	5.4485	3.6544	4.9935	3.3298	4.1937	4.555	4.4121
5.7698	5.5566	5.7441	5.8571	4.761	4.8195	4.7747	5.4735
3.5786	5.7465	4.8512	4.142	5.786	4.7695	5.2698	5.4185
4.5739	5.5006	3.2336	6.36	5.0761	5.0559	5.2678	5.4982
5.6011	5.6378						
BLZF1	3.6338	3.7166	3.2398	4.4853	4.4626	3.902	5.2295

3.5822	3.8551	3.2662	4.0626	4.194	4.1627	4.1982	3.9573	
4.2815	4.132	3.9479	3.7024	3.4267	3.5781	3.5437	3.8908	
4.3378	3.7218	4.7005	4.6873	3.405	4.0269	3.1667	3.4728	
4.1656	3.6672	3.6839	3.4062	3.8716	4.8248	3.4938	3.4136	
4.2213	4.3666	4.7474	4.0964	3.5581	3.9584	4.1209	3.6156	
4.0156	2.2396	4.1434	3.694	4.7485	3.9837	3.3537	3.8703	
3.2768	4.6042	3.2359	4.1925	3.5906	4.4046	3.3453	3.1479	
3.7038	3.6608	4.6388	4.0109	4.2735	4.1891	4.2372	3.7145	
3.8127	3.4247	4.2574	4.4638	3.4783	4.3845	4.6323	3.587	
2.698	3.3469	4.2026	4.2815	3.6929	4.0156	3.9073	3.8878	
3.8003	3.7005							
BMF	0.763	1.4754	1.0862	0.5516	0.4584	1.5448	2.0037	
1.0987	1.3968	1.2132	1.2459	1.833	3.0834	1.1802	1.1955	
1.4565	1.2756	1.57	0.5898	0.8282	1.1802	2.4282	1.5937	
0.5855	0.6431	1.912	2.4915	2.1985	1.5109	2.259	1.0882	
1.0114	1.3458	1.0626	2.8504	0.8386	0.7318	3.7167	3.1492	
0.9012	1.7875	0.5346	0.6163	2.2745	0.8276	1.4023	1.0094	
1.998	1.988	1.5397	1.3678	0.3074	1.9717	2.1327	0.5815	
1.8909	0.7145	0.9123	0.8073	1.1286	1.0214	0.8651	2.5513	
1.2771	2.5373	2.0061	3.5326	2.1599	1.8011	0.3495	0.6282	
1.1646	1.1554	1.416	2.0849	2.3211	1.3629	1.7819	2.0153	
2.571	1.8323	0.7959	2.505	1.0743	1.7501	0.9372	2.0399	
1.3357	2.3854							
BMI1	4.8607	3.5949	4.9397	4.1253	4.3398	3.7085	4.9542	
4.1563	4.2905	5.3146	3.8387	3.8626	4.3668	4.5699	4.0112	
4.0316	4.9973	5.1945	5.0313	4.9448	5.3558	3.923	4.2758	
4.772	4.4964	3.7673	4.3645	5.6698	3.9046	4.6779	4.8816	
4.8783	4.2352	4.0703	4.1034	5.3019	4.449	3.8118	4.2864	
4.0875	4.4415	4.5867	4.2314	4.7625	4.0911	4.0484	4.5943	
4.1861	4.4465	4.933	4.6287	4.3917	4.6481	4.4792	5.607	
4.9992	4.4345	3.7641	5.2691	4.0187	4.142	4.3324	4.5176	
4.21	4.1732	4.5908	4.5961	5.1662	5.481	3.7632	4.1802	
4.5221	3.5484	4.1684	4.0371	4.7242	4.3431	4.296	3.8654	
4.5042	4.8558	4.3381	4.6032	3.718	4.2879	4.7142	3.7924	
4.1111	3.8917							
BMP1	3.59	2.4902	2.6793	3.3479	3.2462	2.3492	3.551	
3.0508	3.0169	3.0652	3.2141	2.7098	4.9217	2.414	3.2137	
3.2508	2.1623	3.2777	2.5559	2.9181	3.3211	4.2157	4.0079	
2.8526	4.1071	4.9021	3.6168	3.0834	4.3555	2.3055	3.2282	
3.2749	3.8816	4.6545	4.6559	2.1909	3.0313	4.2179	4.0969	
3.3352	4.0305	3.7701	3.8058	3.1837	3.2416	3.5199	2.7366	
2.1526	3.02	2.4755	3.8031	3.0643	2.8158	3.0759	2.0833	
3.3666	1.8066	3.6362	3.2439	4.1667	3.1645	2.8822	4.327	
3.2071	4.3265	4.2353	3.7472	4.3506	2.1902	4.123	3.9569	
3.4831	3.7505	3.2549	3.6406	2.4491	3.0926	4.2614	2.8309	
3.0299	2.4743	4.287	3.1471	4.265	4.2668	3.114	4.0798	
3.7723	4.0258							
BMP10	0	0	0	0	0	0	0.0142	0
	0.057	0	0	0.0081	0	0	0.0314	0
	0	0	0	0	0	0	0	0

	0	0	0	0	0	0	0	0
	0	0	0	0	0	0	0	0
	0	0	0	0	0	0	0	0
	0	0	0	0	0	0	0	0
	0	0	0	0	0	0	0	0
	0	0	0	0	0	0	0	0
	0	0	0	0	0.0486	0	0	0
	0	0	0	0	0	0	0	0
	0							
BMP15	0	0	0	0	0	0	0	0
	0	0	0	0	0	0	0	0
	0	0	0	0	0	0	0	0
	0	0	0	0	0	0	0	0
	0	0.0392	0	0	0	0	0	
0.0773	0	0	0	0	0	0	0	0
	0	0	0	0	0	0	0	0
	0	0	0	0	0	0	0	0
	0	0	0	0	0	0	0	0
	0	0	0	0	0	0	0	0
	0	0	0	0	0	0	0	0
	0							
BMP2	0	0.551	0.7263	0.0524	3.3577	0	3.8065	
1.045	0.2777	0.5263	1.4161	0.6722	1.2635	0	0.9096	
0.2825	5.2938	0.0471	0.213	0.7078	0.2398	1.0387	0.6209	
0.5865	2.6832	1.9384	2.8287	0.1117	0.1446	0.6288	0.6499	
0.0741	1.6814	0.0353	0.0486	0.4363	0.0254	0.0472	1.0771	
1.5437	0.5438	0.7504	1.752	0.1777	0.7903	2.9595	0	0
	3.2922	0	0.0351	2.5328	0.0855	0.1581	0.4561	0
	3.0444	1.1291	0.0347	0.6005	1.1663	0.1277	0.0796	
0.4798	0.8896	4.5216	0	2.632	0.6359	1.9237	2.632	
0.2467	0.8101	0.5562	0.6782	0	0	0.6669	0	
0.05	0.098	0.3094	0.7097	0.6487	3.1753	0	0.5636	
0.292	0.1537							
BMP2K	3.5392	2.5543	3.4759	3.32	2.0012	2.967	3.7056	
2.5195	3.2475	3.2867	2.4323	3.1475	3.0954	3.1731	2.1043	
2.5052	2.6444	3.7167	3.4352	2.6663	3.5273	2.4218	2.6	
2.8644	2.0181	3.402	2.3521	3.2602	2.5354	2.6975	2.1264	
3.1591	3.4138	3.1377	3.2234	3.2557	2.6092	2.8818	3.1925	
2.1949	3.0905	3.1423	3.2475	3.2489	2.6735	2.0445	3.421	
2.7824	1.752	3.2184	3.6058	2.7372	3.2369	2.7429	2.4545	
2.8134	3.1519	2.9686	3.1551	2.6632	2.5399	3.3108	2.8839	
3.307	2.7611	2.877	3.3696	2.8578	2.9279	2.2878	3.2824	
3.2367	2.5348	2.2141	2.7386	2.8397	3.3934	2.8374	2.3497	
1.7757	3.3873	3.8796	2.4502	2.2582	1.41	3.0931	3.5785	
3.3513	3.3178							
BMP3	0.028	0.0125	0	0	0	0	0.0079	0
	0.0064	0.0081	0	0.0023	0.01	0.0535	0.0179	
0.0033	0.2743	0.0169	0.0554	0	0.0387	0.0075	0.0022	0
	0	0.0107	0.0116	0.0387	0	0	0.0053	
0.005	0	0	0.0086	0.006	0	0.0185	0.01	

0.012	0	0	0.0192	0.019	0.0238	0.0149	0	0
	0.008	0.0519	0.0082	0	0	0.0035	0.0155	0
	0.0297	0	0.0308	0	0.0352	0.0114	0	0
	0.0415	0.2177	0	0	0	0	0.0063	0
	0.0206	0	0.0162	0.025	0.0052	0.0105	0	0
	0.0065	0.0181	0	0	0.0213	0.0335	0	
0.0335	0							
BMP4	0	0.3366	2.2377	0	2.0325	1.3995	3.8657	
0.4249	0.2911	1.7997	0.19	0.5883	1.6899	0.1698	1.1861	
0.1728	0.0836	0	0	1.7887	1.1638	0.3297	1.4644	
0.3779	0.2909	3.3974	0.3285	1.9234	1.8966	1.8794	0.1032	
0.0194	0.2064	0.171	1.0436	1.6465	0.3327	3.3451	0.4832	
2.2769	0.0432	3.5514	0.8105	1.4674	1.4983	2.8499	0.4006	
0.0995	0.6578	1.0976	0.1117	0	0.1568	0.0868	0.2307	
0.6159	1.6756	0.053	0.0586	0.081	1.6733	0.2098	0.6532	
0.6264	3.0447	3.057	4.2333	3.4445	0.5009	2.7058	0.7452	
1.4659	2.1858	0.0193	2.1353	1.5726	0.6765	0.6922	0.5044	
0.943	0.0188	1.9234	0.0588	1.8279	0.2202	0.1638	1.9221	
2.1813	2.8005							
BMP5	0.0639	0.1099	0	0	0	0.0871	0	0
	0	0	0	0	0.0876	0	0.0308	
0.0119	0	0	0	0	0.0593	0.0598	0.0382	0
	0	0.0148	0.0273	0	0	0	0.2068	
0.0665	0	0	0	0	0.0064	0	0	0
	0	0	0	0.0683	0	0.4616	0	0
	0	0.4996	0	0	0	0.0087	1.2941	0
	0	0.0208	0	0	0	0	0.0154	0
	0	0	0	0	0	0	0	0
	0.0355	0.0521	0	0	0	0	0	0
	0	0	0	0	0	0	0	0
	0							
BMP6	0.2301	0.0906	0.14	0.3023	1.9915	0.1286	0.9843	
0.6814	0.2116	0.0795	0.0763	0.2114	0.4708	0.0838	0.3578	
0.0865	0.7613	0.076	1.284	0.8943	0.1677	1.3937	0.1615	
0.6948	2.9093	0.1124	2.9145	0.0866	0.326	0.5302	0.3874	
0.0938	0.1093	0.1027	0.1948	0.2239	0.3703	0.2608	0.859	
2.22	0	0.058	3.216	0.3601	0.4643	0.1464	0.2031	
0.3911	3.3826	0.1443	0.0969	0.7623	0.2619	0.0063	0.0956	
1.8502	1.6083	0.454	0	0.3369	1.9319	0	1.1144	
0.5473	0	2.8829	0.1606	0.0708	0	0.2114	0.0071	
0.4609	0.1379	0.086	0.6494	0	0	0.4824	0.7481	
2.5394	0.5441	0.0223	1.122	0.5484	1.037	0.2025	0.1521	
0.0772	0							
BMP7	0.1428	0.669	1.1443	0	1.0688	0.4318	4.0233	
0.1009	3.9907	0.1529	2.6606	0.2635	0.8185	1.0097	0.1996	
5.4747	0.144	0.1472	2.4351	0.1973	0.0785	0.2913	3.6971	
1.1488	4.1416	0.1996	0	0.1852	0.2002	4.6412	4.0572	
0.1417	4.0299	0.2541	0.3455	1.6452	0.1283	0.4094	0.4271	
3.9769	0.0599	1.4069	3.4105	0.1082	0.4996	1.9888	3.6177	
1.8899	0.0603	0	0.4356	0.1147	0.6674	0.0209	3.8444	0

	0	0.8357	0.1371	0.0578	1.5805	2.3965	0.7093	
0.4902	1.7336	0	1.0478	1.5278	5.2691	0.4724	0.2306	
2.5038	2.1539	0.8625	0.3303	5.9434	0.0337	0.4946	2.6517	
0.5175	4.6774	0.0304	0.0072	0.9889	1.5817	0.5669	0.1617	
0.2683	0.1447							
BMP8A	0.3568	0.5311	0.163	0.2224	0.3586	0.2535	0.3086	
0.2605	0.5841	0.3568	0.1366	0.2745	0.2539	0.0936	0.4747	
0.1073	0.5369	0.5754	0.7153	0.2465	0.3564	0.1046	0.1304	
0.0984	0.1716	0.301	0.1574	0.3892	2.5277	0.3183	0.8916	
0.2717	0.3152	0.1145	0.2022	0.2717	0.665	0.0697	0.0411	
0.2714	0.2101	0.1031	0.1823	0.3697	0.6675	0.4403	0.1521	
0.0477	0.431	0.1749	0.4952	0.2708	0.3592	0.5968	0.9521	
0.7377	0.0927	0.2221	0.5458	0.0918	0.1793	0.9316	0.5341	
0.172	0.3423	0.189	0.2957	0.337	0.4813	0.1651	0.02	
0.5089	0.051	0.3728	0.0144	0.1327	0.5531	9.00E-04	0.0438	
0.4024	0.2516	0.1161	0.2793	0.0291	0.1246	0.7917	0.2955	
0.2866	0.3539							
BMP8B	2.0262	0.2015	0.5985	1.1925	2.233	1.0668	0.1095	
1.02	2.654	1.0411	1.5298	1.8286	0.8583	1.3749	2.5276	
1.6054	0.1564	2.6691	1.6809	3.0373	2.1457	3.0799	4.0309	
0.4605	0.7436	0.1585	0.5015	0.8098	2.6187	2.021	0.9503	
1.9087	2.0303	1.8017	1.2866	0.6454	3.454	1.4166	3.0436	
0.6203	1.9111	0.1786	2.4471	0.6607	1.3497	0.251	0.5382	
3.873	0.4632	0.3237	1.6253	0.6739	1.6303	2.5992	0.6181	
1.2995	0.1625	2.4148	3.2178	2.2083	1.9252	2.4077	1.162	
1.4528	0.6185	0.071	0.7191	0.0509	2.1511	3.5671	0.3917	
2.4348	0.005	4.2747	0.1949	1.667	0.9884	1.2427	2.4184	
1.8506	2.8447	1.2577	0.3025	1.2678	1.6513	1.7258	0.1259	
0.215	0.1101							
BMPER	0.0234	0	0	0.0512	0	0.043	0.0824	
0.0655	3.00E-04	0	0.011	0.5948	1.6993	0.0065	0.1104	
0.2147	1.5297	0.0361	0	0.0177	0	0.4854	0.1643	0
	0.7716	3.475	0.0078	0	0.4777	0.0451	0.097	0
	0.1458	0	0.5558	0	0	0.0351	0.0166	
0.1648	0.0151	0.0936	1.911	0.0647	1.8805	2.7761	0	
0.3356	1.4221	3.5918	0.1745	1.146	0	0.0336	0.0525	0
	0.3778	1.4714	0.0683	3.7427	0.1413	0.0401	0.0887	0
	0	3.3926	0	0.2243	0.1276	0.12	0.3308	
0.0653	0	0.2082	0.3881	0.2611	0.0321	0.0739	0.0583	
0.1246	0.3629	0.1363	0.1393	0.0577	1.1268	0	0.8272	
0.0025	0.0369							
BMPR1A	3.4105	3.302	4.0696	4.3307	3.8063	4.4968	3.4933	
3.9191	3.5736	3.9956	2.6597	3.8009	3.2815	4.8949	3.8659	
3.4125	3.4214	4.1574	3.588	3.6772	4.189	3.0213	3.0934	
3.9003	4.62	3.1762	2.8788	3.9338	3.2296	2.7809	3.5004	
3.3237	3.6707	2.8235	3.4455	3.8949	3.527	3.179	3.8232	
3.7717	4.0506	2.9876	3.5119	3.9628	4.0065	4.2592	4.124	
3.9604	3.5497	3.9616	3.4438	3.1794	3.712	4.4983	4.4158	
3.8627	3.7219	3.8171	3.4601	3.9162	3.8572	3.1408	4.3815	
4.1104	4.4463	3.4878	3.8389	4.0911	3.5302	3.4577	3.5493	

3.3179 3.3802 2.4482 3.3843 4.4672 4.0176 3.4909 3.4534
2.7432 3.6572 3.7742 3.3172 3.9973 3.3228 3.4446 3.5373
3.7437 3.3968
BMPR1B 1.9096 1.5094 1.8234 0.7318 0.3484 0.4689 1.6409
0.4115 0.4398 1.1073 0.9794 0.4861 0.1843 1.5967 0.7628
1.4803 0.5298 3.1902 1.2403 2.0858 2.0823 0.8652 0.406
1.8552 0.2682 0.2661 1.3626 0.1174 0.1959 0.4288 2.0198
1.6071 1.7012 0.1463 0.5142 1.6876 0.8166 0.5985 0.9162
1.2142 0.0607 0.2743 0.4566 1.8966 0.4011 0.9398 2.0533
0.9062 0.1989 0.7146 1.0089 0.6028 1.8444 0.2179 0.9608
1.3466 0.5765 2.898 2.1585 2.7237 1.4277 1.1377 1.7478
1.2938 1.0824 0.3372 2.3233 0.9863 0.9385 0.0811 0
1.2228 0.522 0.2627 0.8324 2.53 1.3984 1.3978 1.4036
1.0726 1.6716 1.0818 1.8301 1.0408 0.1078 1.4645 1.9794
1.84 1.3518
BMPR2 2.7246 4.6286 4.4005 2.9204 2.3212 3.408 4.5556
2.8331 4.1354 4.5869 4.3329 3.0907 3.2898 3.4713 4.1059
4.075 3.9982 5.7498 4.2505 3.4194 3.0333 2.1496 3.056
2.8789 3.3082 3.9212 3.1516 5.4566 2.7541 5.1179 3.6185
3.9276 3.1953 3.9706 3.2763 3.8647 2.7594 3.014 2.4038
3.0329 3.0205 3.6109 3.7429 2.6114 3.9092 3.6085 4.3048
3.7436 3.598 3.3937 3.1521 2.6047 3.2568 3.8556 3.554
2.8301 3.7117 3.1159 4.5124 3.5407 2.8459 3.7854 2.7082
3.2383 3.6809 3.6611 3.6031 5.3884 6.0018 3.0813 3.8523
3.4064 2.8198 3.137 3.4454 6.2276 4.7229 3.5251 3.6798
2.7305 3.9793 3.0176 3.8454 2.8672 2.9935 2.6892 4.9758
3.4105 4.6512
BMS1 3.5789 4.0991 3.7059 3.8209 4.0139 2.9969 3.3578
2.9869 3.7416 3.8172 3.0066 3.2055 3.6662 3.6049 3.1555
3.3877 3.5278 3.4558 3.5246 3.5236 3.8376 3.1089 3.1461
3.7661 3.3196 3.4516 2.6725 3.196 3.1216 2.88 3.4033
3.6141 2.9381 3.175 2.9061 3.8683 3.8157 2.9982 3.1956
3.3241 3.1539 3.3888 3.4347 3.136 3.4178 3.0549 3.7685
2.8469 2.7845 4.0612 3.0619 3.5015 3.3612 3.6197 3.8722
3.6988 3.8891 3.909 3.4492 3.7088 3.4106 3.5937 2.5481
3.648 3.6514 3.0412 3.1972 3.2306 3.413 3.3676 3.2102
3.2093 3.2635 3.022 3.1121 3.2624 3.7558 3.236 2.7242
3.7395 3.3282 3.6529 2.6147 3.0306 3.0453 3.1969 3.0232
3.0952 2.8631
BMX 0.0447 0.1521 0.0409 0.4782 0.0614 0.154 0.1051
0.0333 0.1102 0.0984 0.0164 2.0953 0 0.4641 0
0.6082 0 0.0894 0.4288 0.0638 0.0892 0.0225 0.0987
0.2335 0 0 0 0.2503 0.0262 0.0499 0
5.2612 0.2378 4.2345 0.1195 0.0975 0.2307 0.1022 0.7923
0.0572 0.1063 0 0 0.5466 0.0186 0.0253 0.1741
0.5778 0.068 0 0.1163 0.1158 0.0877 0.4739 0
1.0661 0 0 0.1242 0 0.097 0.0037 1.6808
0.1581 0.0208 0 0 0 0 0.3307 0.8514
0.0106 0.0608 0 0.0238 0.2 0.0456 1.8416 4.3335
0.482 0.2005 0 0.0432 0 0.0693 0.1506 0.0527 0

	0							
BNC1	0	0	0.3747	0	0.2846	0	0	0
	0.2365	0	0.1246	2.509	2.4096	0.2824	0.2798	0
	2.6691	0	0	0	0	0.2903	0	0
	0.096	0.8475	0.0147	1.1944	0.056	1.5432	0.2081	0
	0.1743	0	0	0.0357	0	0	0	
0.0334	0	0.6324	0	0	3.7931	0	0.0627	0
	0	0.9106	0	1.1265	0	0.1347	1.1048	0
	4.2042	0	0	0	0	0	0	
0.4651	0	0.0941	0	0.2806	0.7071	0.3332	0	0
	0.0721	0.0355	0.1635	0.4053	0	0	0.0761	0
	0	1.7773	0	0.1385	0.1822	0	0.6673	
0.048	0.1842							
BNC2	2.4319	3.4206	3.3284	2.9963	2.2815	3.0428	2.5888	
2.6321	3.9904	4.2326	3.0302	2.4832	2.4194	3.7681	1.7016	
2.4273	3.0712	3.6946	3.7943	2.4445	2.7923	1.3006	0.8379	
3.0707	1.1509	3.126	1.8648	3.1965	2.1879	2.3289	3.1039	
2.127	2.6596	3.9462	1.6697	3.5911	3.8215	3.682	2.4319	
3.0065	3.5011	2.1632	2.549	3.2458	2.1792	1.5364	3.2543	
2.8645	2.1782	2.8175	2.8162	0.7408	3.8133	3.3577	3.0417	
3.0209	4.059	0.7709	1.9195	0.7634	2.4501	2.8266	2.3908	
3.0332	3.8653	2.8289	3.0713	2.3177	3.1947	2.1995	3.6504	
3.126	2.7672	1.4944	2.2117	2.6689	3.563	2.6815	3.5445	
3.2578	4.3857	3.3896	1.595	3.004	1.8742	2.9132	3.88	
3.6521	3.9531							
BNIP1	3.1184	3.5785	3.4252	3.5768	3.7612	3.0964	3.0023	
3.5377	3.106	3.0454	3.1345	3.1801	3.7355	3.0737	3.3229	
2.8854	3.4663	2.604	3.4427	4.0012	3.3011	3.7914	2.4758	
3.4784	3.2779	3.5819	4.9577	2.5248	2.8983	3.0032	3.2427	
4.0947	3.3932	3.6491	3.2999	3.2622	3.2362	3.4716	2.7793	
3.0687	3.1129	2.8247	3.3148	3.2533	3.0227	3.478	2.9754	
2.8946	2.8527	3.6919	3.4133	3.0726	3.0096	3.1808	3.4873	
3.3901	3.6065	3.6539	2.7898	3.2609	3.3571	3.9552	3.2509	
3.3115	2.9506	2.5995	3.1049	3.0613	2.1838	3.5708	2.7062	
3.2744	4.0329	3.2012	2.7066	3.0697	3.5259	3.6448	2.6627	
3.4723	3.3	3.0315	3.6902	3.1234	3.7344	3.4012	3.1566	
2.8636	3.0263							
BNIP2	4.628	5.5604	5.5658	5.3561	5.227	4.9197	5.6236	
4.8857	5.2807	6.0677	5.0026	4.6999	4.8805	5.3923	4.08	
5.2429	4.9731	5.7306	5.3424	4.1164	5.7092	5.1166	5.0947	
5.5087	4.951	4.0752	3.8072	5.637	5.0137	5.2175	4.054	
4.8823	4.4894	4.5935	5.2326	5.3749	5.4638	5.3283	5.1682	
5.0611	5.0594	4.904	4.5054	5.3845	5.6065	5.1836	5.9089	
5.4645	5.0947	5.4513	5.2465	4.6956	5.1072	6.0587	3.8398	
5.5817	5.4868	4.7099	5.0715	4.7574	5.2397	5.4727	5.1656	
5.3891	4.6271	4.8223	5.141	4.8767	5.7385	4.3243	4.6764	
4.5915	5.9616	4.8249	5.0698	5.549	5.0496	4.8793	5.0128	
5.2843	4.9886	5.1338	4.6722	5.4375	4.7313	4.8401	5.323	
5.4481	5.3156							
BNIP3	5.1955	5.8871	5.3973	6.1676	6.5369	6.0984	5.8801	

4.989	5.7199	5.6737	4.7805	4.9635	5.9642	5.3222	4.4512
4.4357	5.1683	4.6199	3.8346	6.0719	5.7246	6.0575	6.3594
6.3782	4.7649	5.888	4.8927	4.7215	4.2216	6.4406	7.5
3.592	4.9707	5.5211	3.4171	6.612	5.4262	4.0227	3.4287
5.7612	6.0744	4.8609	5.4168	4.4669	4.5995	5.8478	5.5699
4.7415	5.7031	6.5848	5.5766	5.9442	4.4045	4.2772	6.5824
5.0671	5.5055	5.2112	4.5004	4.7849	5.567	6.5328	5.6688
5.2783	6.1258	6.6423	6.5023	6.2131	3.946	4.6701	5.5536
6.1366	4.1408	5.9803	4.8572	4.906	4.9621	4.0937	5.0962
6.4273	5.2275	6.5676	7.2197	3.9419	4.6402	4.1801	6.9076
5.6458	5.6467						
BNIP3L	4.4807	4.5271	3.8838	4.8895	4.2196	4.8737	5.8326
4.5437	5.4801	5.2449	5.3587	5.0541	5.5327	5.2493	3.9485
4.6365	3.9705	4.1517	4.5241	4.4723	5.2803	5.2408	5.5105
4.7035	4.0543	5.9468	5.4558	4.8963	4.7564	5.6536	6.6631
4.554	4.7507	5.8086	4.6662	5.4518	4.3265	5.1363	4.092
5.4007	5.181	4.3784	4.254	4.0142	5.1513	6.1776	4.8012
4.2165	4.4736	6.07	5.0061	4.7871	6.2717	4.6561	4.2478
4.292	4.5698	4.3804	3.9682	4.5158	4.8113	5.3531	4.5784
4.715	5.7953	5.3822	5.5779	5.7945	3.6062	3.6218	6.1246
5.3121	5.0728	5.1869	5.1749	4.2951	4.4075	4.386	4.9995
6.0267	4.9814	5.7614	6.0591	4.9689	4.0936	5.5779	5.984
5.0464	5.2995						
BNIPL	0.2428	0.0702	0.3171	0.2342	0.142	0.2694	0.152
0.1101	0.0941	0.3101	0.3387	0.0632	0.1279	0.1459	0.2285
0.0547	0	0.1111	0.1962	0.2743	0.2831	0.1731	0.0477
0.2148	0.1421	0.4356	0.1562	0.451	0.1951	0.3084	0.1992
0.032	0.2887	0.0862	0.1403	0.137	0.1134	0.1115	0.1524
0.2823	0.2142	0.1105	0.0657	0.2076	0.3351	0.3351	0.1023
0.286	0.1972	0.3635	0.1809	0.2808	0.2497	0.1367	0.2738
0.5774	0.2466	0.095	0.3366	0.1011	0.1121	0.1401	0.2112
0.3728	0.1514	0.0917	0.3304	0.1885	0.1113	0.0716	0.1272
0.3462	0.1564	0.1514	0.2164	0.0654	0.3874	0.1131	0.1163
0.322	0.1264	0.1003	0.2447	0.1806	0.1944	0.251	0.1812
0.2304	0.239						
BOC	0.1246	0.8083	0.1747	0.5259	0.6184	0.304	1.1377
0.6724	1.3495	1.9178	1.3793	0.5736	0.9449	0	0.1096
2.7161	0.5333	0.2005	1.8165	2.2725	0.3868	0.2231	0.5962
1.2703	1.4272	0.6271	0.4391	0.6829	0.2543	1.3539	1.2316
0.0452	2.891	2.732	1.0882	0.8267	0.5463	1.8196	0.3457
1.7016	0.4796	0.8988	0.6584	3.0655	1.0012	2.2391	0.2908
0.5387	1.4345	1.1571	1.2466	0.1828	0.5801	0	1.1435
1.1556	0.7501	0.0132	2.828	0	0.839	0.9984	0.2028
3.2493	0.7628	1.3367	0.283	0.3386	1.992	1.2507	1.1243
2.9332	0.1497	0	1.6083	2.5397	0.7868	0.1861	1.6518
1.7849	2.5066	1.0962	1.8121	0.3045	1.2648	0.4528	2.8469
2.4217	3.555						
BOD1	5.0295	4.1749	5.065	4.4079	4.1703	4.2166	4.3251
5.4618	4.5609	4.3018	4.4769	4.5788	6.2158	4.289	5.7861
3.4275	4.931	3.7566	4.9526	4.9315	4.4744	4.4554	4.3287

4.4352	4.6949	5.6699	4.8888	4.362	4.2858	4.5721	5.6643	
4.6751	4.7739	4.9103	4.803	4.5502	5.0656	4.7519	4.5033	
4.3707	4.2447	4.063	4.7039	4.2602	4.3315	5.1234	4.6477	
4.3517	4.9598	5.047	5.0642	4.4318	4.3915	5.3729	4.6032	
4.9538	4.2753	4.4929	3.2702	4.6373	4.6388	4.6004	4.9933	
4.3559	4.6689	4.7429	4.5973	5.0534	4.0726	4.8587	4.238	
4.7455	5.2912	4.5865	3.9322	3.9058	4.6388	5.1848	4.6149	
4.6473	4.4991	4.0828	5.5427	4.5608	5.7053	4.313	4.4118	
4.3882	4.2896							
BOD1L1	2.5086	2.7423	2.9643	3.7719	3.7034	3.2189	2.9998	
2.9421	2.584	3.002	2.5876	3.4096	2.6326	3.2486	2.4164	
2.8427	2.8607	3.3098	2.7276	2.9357	3.0592	3.2744	2.6193	
2.891	2.7007	3.7312	3.4724	2.9879	2.4844	3.2558	1.7078	
3.0658	2.6737	2.4347	2.6967	3.1119	3.4631	2.6902	2.8319	
2.9426	1.8856	3.058	2.2211	3.4084	2.9674	3.1217	2.9416	
3.0496	2.2599	3.5116	2.1852	3.4261	3.0957	3.5524	2.1149	
3.7399	3.9135	2.6094	3.0693	2.8324	2.6062	3.4979	2.6804	
2.6913	2.7939	2.9385	2.8026	3.1266	3.6168	2.5769	2.923	
2.8451	2.9604	3.1763	3.6031	3.9322	2.6225	3.2131	2.3701	
2.815	2.9078	3.0504	2.9412	3.2443	2.4169	2.6015	3.0937	
3.0238	3.2909							
BOD1L2	0	0.0149	0	0	0	0	0	0
	0	0	0	0	0	0	0	0
	0	0	0	0	0	0	0	0
	0	0	0	0	0	0	0	0
	0	0	0	0	0	0	0	0
	0	0	0	0	0	0	0	0
	0	0	0	0	0	0	0	0
	0	0	0	0	0	0	0	0
	0	0	0	0	0	0	0	0
	0	0	0	0	0	0	0	0
	0	0	0	0	0	0	0	0
	0							
BOK	0.2066	0.3589	3.5584	1.0457	1.5249	0.4126	1.3524	
3.185	1.1646	0.9604	1.6307	0.7281	0.3033	0.205	1.6514	
0.1814	3.0868	0.3618	0	2.131	1.3252	1.4406	0.2918	
0.186	0.4004	3.1755	1.4483	2.783	0.3445	0.6499	2.9703	
2.7414	2.1771	1.6181	0.1791	0.8406	0.2824	3.0776	1.3531	
0.1874	1.7003	1.442	0.7488	0.5332	1.3328	1.682	0.1982	
1.8032	3.0197	1.1049	1.3268	0.1907	1.1463	1.9027	2.4326	
0.6444	2.6507	3.8323	0.4791	3.4275	1.504	0.8227	0.1205	
0.8324	3.3124	2.358	0.1265	2.2989	1.063	2.0042	0.0187	
0.3593	1.7679	2.6041	1.4019	0	2.3725	0.1325	1.9697	
1.5873	2.767	2.3412	0.4114	0.3435	3.0339	1.7883	1.0541	
2.1468	2.1015							
BOLA1	3.2325	3.6899	0.076	3.6342	0.5245	2.596	3.7652	
3.3064	2.973	3.7469	4.477	4.028	2.8817	3.4002	2.877	
2.8372	2.3089	3.5867	2.8344	3.0913	2.4004	3.169	2.6723	
3.167	2.6908	2.5688	3.7774	3.1747	3.0408	4.4949	3.9111	
3.2176	2.6478	3.7803	2.6058	3.685	4.4235	3.2085	3.7786	

4.1538	4.0635	3.542	4.0319	2.8713	2.6924	4.3417	3.5244	
3.3403	1.882	3.4677	3.5202	4.0044	3.1698	3.2745	4.0743	
2.6502	1.9579	0.7692	3.479	1.464	3.1636	3.8806	2.9991	
3.4335	4.1917	3.7381	3.987	3.8443	3.3209	3.0016	3.2164	
3.5677	3.2255	3.6175	3.7702	2.9826	3.5025	4.4733	2.9361	
4.5318	3.389	2.9495	3.4501	4.0822	0.4566	3.5732	3.5985	
3.1946	3.5169							
BOLA2	1.9683	2.775	2.3774	3.1242	2.6384	2.9835	3.1359	
3.5128	2.609	2.2969	2.3941	2.1703	2.63	2.6532	2.8922	
2.3202	2.5312	3.2005	3.4029	2.4728	2.7703	3.1076	2.7505	
3.4731	2.7949	2.0819	3.9917	2.4478	2.915	3.1046	1.8257	
2.2803	2.6607	2.0651	1.9112	2.4093	1.4825	1.5701	2.21	
3.0165	2.7165	2.3434	2.346	2.5113	2.8068	3.3483	2.8579	
2.4018	2.3871	2.8247	3.754	3.2724	2.6808	2.9839	2.2025	
1.6703	1.5931	0.7497	2.8764	0.6703	2.8365	2.429	2.348	
2.6031	2.3852	2.8081	2.6307	2.3349	2.5364	2.9923	2.689	
2.3822	2.5119	2.5569	2.1414	1.8331	2.2922	2.8025	2.9698	
2.438	2.6406	2.8967	2.6712	2.4834	3.0653	2.4525	1.8461	
2.7542	2.1304							
BOLA2B	0	0	0	0	0	0	0	0
	0	0	0	0	0	0	0	0
	0	0	0	0	0	0	0	0
	0	0	0	0	0	0	0	0
	0	0	0	0	0	0	0	0
	0	0	0	0	0	0	0	0
	0	0	0	0	0	0	0	0
	0	0	0	0	0	0	0	0
	0	0	0	0	0	0	0	0
	0	0	0	0	0	0	0	0
	0	0	0	0	0	0	0	0
	0							
BOLA3	6.2392	6.8714	6.5567	5.9313	6.1531	5.3629	6.409	
5.9192	7.0127	5.9927	6.1126	6.1093	5.7834	6.1287	5.8888	
5.5729	6.0262	6.0851	7.0554	6.2232	6.1569	5.2958	5.9916	
6.4854	5.3199	5.3899	5.7547	5.9375	5.9098	6.7141	5.6043	
5.3655	6.2702	5.9631	5.4977	6.6562	6.1352	5.8238	5.5305	
6.638	5.7627	5.1761	5.9153	5.0988	6.0023	6.049	6.9428	
5.2964	5.6378	5.6766	5.7378	5.973	5.9843	5.8263	6.2593	
6.4187	5.2221	5.9718	7.0369	6.0054	6.4111	7.0903	6.4503	
5.8036	6.5709	4.8684	6.4151	6.2132	6.4521	5.2989	6.041	
5.7954	6.5253	5.9002	5.6265	6.3968	6.5274	6.1565	6.0728	
6.5397	6.1368	5.5318	6.369	5.4059	5.4485	6.5092	5.2603	
6.1604	5.4294							
BOLL	0	0	0	0.0359	0	0	0.0221	
0.0329	0	0	0.0908	0.0173	0	0	0.0076	0
	0.0493	0	0	0	0.0484	0	0	0
	0.0193	0	0	0.0442	0.0172	0.0191	0.0322	0
	0	0.0608	0.0112	0.0112	0.0068	0	0.0132	
0.0493	0	0	0	0	0.0069	0	0	0
	0	0	0	0.0199	0.0477	0.0076	0.0238	0

	0.0258	0	0.1317	0	0	0	0.0363	0
	0	0.0032	0.0349	0.0136	0	0	0	0
	0.0401	0	0	0.0223	0.0055	0.0177	0	
0.0289	0	0.0096	0	0	0	0	0	0
	0							
BOP1	4.9686	4.3484	5.6351	3.5591	5.4911	4.8594	2.568	
4.9248	3.1205	4.7278	4.2505	3.6456	4.723	3.5429	5.3748	
3.5904	5.9471	2.8522	3.9056	5.9949	5.2545	5.0826	4.2775	
4.9068	4.6846	3.842	3.0936	3.9907	4.9732	3.747	4.1124	
5.345	5.4654	4.3157	4.3421	3.7618	4.8994	5.9561	5.1711	
4.1975	4.5617	3.598	5.1409	4.1877	3.5176	4.3607	4.0025	
4.2724	4.5913	3.8037	3.9754	3.0602	3.5142	5.2578	4.8058	
5.485	4.2127	5.9054	4.9868	5.7265	5.0891	4.7673	5.1863	
4.6099	6.8036	2.2479	4.6261	4.5125	3.3557	4.93	5.3973	
4.2963	4.759	4.2848	3.814	4.1704	4.9556	3.8821	5.105	
5.6204	4.6681	4.7251	4.0823	4.3728	4.9933	4.9859	3.5547	
4.9981	4.0611							
BORA	3.0682	2.6319	3.0056	3.6038	2.4572	2.334	2.0166	
3.3914	2.6692	3.1288	1.9478	2.3798	3.1645	2.7781	2.8178	
1.6654	3.0207	2.6347	3.281	2.4591	2.7495	2.8142	1.9149	
2.3974	3.3408	1.6228	1.8931	2.4436	2.8219	2.4997	2.1576	
1.155	2.5881	2.2929	2.3329	3.3646	1.5894	1.7288	2.174	
2.0803	1.1326	1.8551	3.5011	2.7856	2.7576	3.2607	3.6146	
2.0114	1.6506	3.215	2.9817	2.2531	2.4216	2.1668	2.52	
3.2416	2.5363	2.6949	2.4625	2.7196	2.8541	2.8348	2.6455	
2.824	2.3036	0.9365	2.1882	1.2658	1.7994	3.9903	2.2292	
2.9943	2.8013	2.1639	2.2699	2.0912	2.7703	1.5682	2.5806	
2.4821	3.5537	1.3541	1.3858	1.971	1.9955	2.6704	1.8895	
2.1971	2.1167							
BPGM	4.2546	4.1365	2.9839	3.9707	3.2147	4.5462	4.358	
4.2177	3.7639	3.5233	4.1018	3.8675	3.4887	3.9488	5.4845	
5.1793	2.6624	4.356	3.9041	4.9241	3.3283	3.9335	4.0407	
3.8643	4.2835	4.5928	3.9573	4.0992	4.5167	4.1311	4.2683	
3.0276	3.805	4.0815	4.5891	4.0685	3.1965	3.3687	3.4572	
3.725	3.7235	3.3225	3.7119	3.1451	2.9636	3.9424	3.0522	
3.5351	4.6252	3.1365	3.8221	4.4031	4.1905	3.1607	3.7837	
3.4521	2.1728	3.7964	4.6087	3.9127	3.2612	4.5288	3.8586	
4.5145	3.9417	5.0799	3.5812	3.5011	3.6164	4.2086	3.8896	
4.1436	4.1423	3.9709	4.1768	4.2025	3.7833	4.2685	3.1281	
3.4727	3.0673	3.3619	3.2529	3.7084	4.6173	4.1517	3.6982	
3.4821	3.5968							
BPHL	4.2463	3.4974	3.2601	3.2399	1.8498	2.3958	2.7134	
3.0464	4.2154	4.1838	3.5581	3.302	3.3725	2.7571	2.9922	
3.1605	2.3944	4.0974	3.6466	2.6147	2.6657	3.4279	3.6236	
3.2558	3.4038	2.4347	3.3863	4.5568	3.2852	4.0471	4.2622	
2.6224	2.8784	4.0923	3.0937	3.8923	3.2415	3.785	2.6623	
2.9588	4.176	2.5229	3.4865	4.6234	2.3644	4.3259	4.445	
3.1307	3.2229	3.6018	3.6974	2.9391	3.8037	3.2252	3.4586	
3.1174	2.9796	3.1403	3.927	2.8236	2.7164	3.4979	3.4254	
2.6649	4.2969	2.026	4.5998	3.454	3.4845	2.2313	4.1036	

3.125	3.4862	2.8547	3.305	3.1542	3.9228	4.3251	4.0184	
4.634	3.162	3.5082	3.273	2.592	3.6136	3.6572	4.2538	
4.2503	4.3612							
BPI	0	0	0	0.159	0.014	0	0	0
	0	0	0	0.0395	0.0753	0	0.0271	0
	0.0914	0.962	0	0	0	0.1408	2.0281	0
	0	0.7712	0	0	0.3853	0	0.0279	0
	0	0	0.051	0	0.0535	0	0.0114	0
	0.1604	0.0822	0.0598	0	0.0089	0.4261	0	
0.1287	0.0552	0	0.7405	0.1254	0	0.1083	0	0
	0	0	0	0	0.1999	0.0414	0	0
	0	0	0	0	0	0	0.0459	
0.0997	0	0.7521	0.1507	0	0	0.1437	0	0
	0	0.0367	0.0435	0.0601	0.3087	0	0	0
	0							
BPIFA1	0	0	0	0	0	0	0	
0.0439	0	0	0	0	0	0	0	
0.0353	0	0	0	0	0	0	0	0
	0	0	0	0	0	0	0	0
	0	0	0	0	0	0	0	0
	0	0	0	0	0	0	0	0
	0	0	0	0.2311	0	0	0	0
	0	0.1743	0	0	0	0	0	0
	0	0	0	0	0	0	0	0
	0	0.0564	0	0	0	0	0	0
	0	0	0	0	0	0	0	0
	0							
BPIFA2	0	0	0	0	0	0	0	0
	0	0	0	0	0	0	0	0
	0	0	0	0	0	0.1194	0.0552	0
	0	0	1.885	0.0717	0.2117	0	0	0
	0	0	0	0	0	0	0	0
	0	0	0	0	0	0	0	0
	0.0336	0	0	0.3502	0	0	0	0
	0	0	0	0	0	0	0.0979	0
	0	0	0	0	0	0.4327	0	0
	0	0.0698	0	0	0	0	0	0
	0.1873	0	0	0	0	0	0	0
	0							
BPIFA3	0	0	0	0	0	0	0	0
	0	0	0	0	0	0	0	0
	0	0	0	0	0	0	0	0
	0	0	0	0	0	0	0	0
	0	0	0	0	0	0	0	0
	0	0	0	0	0	0	0	0
	0	0	0	0	0	0	0	0
	0	0	0	0	0	0	0	0
	0	0	0	0	0	0	0	0
	0	0	0	0	0	0	0	0
	0	0	0	0	0	0	0	0

	0							
BPIFB1	0.138	0	0.133	0	0.0661	0.5055	0.135	
0.2654	0.0664	0	0.0684	0.2469	0	0	0.0288	0
	0.0815	0.0579	0.2334	0	0	0.1503	0.6393	
0.0883	0.1414	0	4.5055	0	0.0982	0.0427	0	
0.1754	0	0.0704	0.2499	0	0.1033	0.3354	2.7765	
0.7976	0.0644	0.0399	0	0	0.0344	0	0.0608	
0.2122	0	0	0.0996	4.8023	0	0.0891	0.0936	0
	0.3088	0.4132	0.1284	1.902	0.0248	0	0	0
	0.0167	0	0	0	0	1.6326	0	0
	0.0817	1.4456	0.2261	0	0.0313	2.045	1.4769	
0.8136	0.0413	0.189	0	0	0.2844	0.1087	0.06	0
	0							
BPIFB2	0	0	0	0	0.017	0	0	0
	0.0379	0	0	0.021	0	0	0	
0.0963	0	0	0	0	0	0	0.0612	
0.0267	0.104	0	0.1638	0	0	0	0	0
	0	0	0	0	0.023	0	0.1052	0
	0	0.0133	0	0	0.0324	0.0513	0	0
	0	0	0	0	0	0.1055	0	0
	0	0.0456	0	0.0199	0.0326	0	0.0146	0
	0.0275	0	0	0	0.0095	0.0425	0	0
	0	0.0613	0	0	0	0	0.0407	
0.0794	0.0612	0.1898	0.0118	0	0	0.0575	0.0619	0
	0							
BPIFB3	0	0	0	0	0	0	0	0
	0	0	0	0	0	0	0	0
	0	0	0	0	0	0	0	0
	0	0	0	0	0	0	0	0
	0	0	0	0	0	0	0	0
	0	0	0	0	0	0	0	0
	0	0	0	0	0	0	0	0
	0	0	0	0	0	0	0	0
	0	0	0	0	0	0	0	0
	0	0	0	0	0	0	0	0
	0	0	0	0	0	0	0	0
	0							
BPIFB4	0	0	0	0	0	0.0403	0.0568	0
	0	0	0	0	0	0	0	
0.065	0	0	0	0	0	0	0.017	0
	0	0	0.2565	0	0.0203	0	0	
0.0101	0.0492	0	0	0	0	0	0	0
	0	0	0	0	0	0	0	0
	0	0	0	0	0	0	0	0
	0	0	0	0	0	0.0134	0	0
	0	9.00E-04	0	0	0	0	0	0
	0	0.1439	0.0407	0	0	0.0804	0	0
	0.0179	0	0	0	0	0	0	0
	0							
BPIFB6	0	0	0	0	0	0	0	0

	0	0	0	0	0	0	0	0
	0	0	0	0	0	0	0	
0.0314	0	0	0	0	0	0	0	0
	0.034	0	0	0	0	0	0.0226	0
	0	0	0	0	0	0	0.0485	0
	0.0261	0	0	0	0	0	0	0
	0	0	0	0	0	0	0	0
	0	0	0	0	0	0	0	0
	0	0	0	0	0	0	0	0
	0	0	0.0417	0	0	0	0	0
	0							
BPIFC	0	0	0	0	0	0	0.1792	0
	0	0	0	0	0	0	0	0
	0	0	0	0	0	0	0	0
	0.01	0	0	0	0	0	0	0
	0	0.0209	0	0	0.0294	0	0	0
	0	0	0	0.026	0	0	0	0
	0	0	0	0	0	0	0	0
	0.0966	0	0	0	0	0	0	
0.0474	0	0	0	0.0239	0	0	0	0
	0.0685	0	0	0	0	0	0	0
	0	0	0.0211	0	0	0	0	0
	0							
BPNT1	3.4271	4.0146	3.9232	4.6693	4.6777	4.8821	4.5776	
4.1183	2.9409	3.0164	3.2611	4.3722	3.5354	4.7811	3.7812	
4.1763	3.1375	4.2415	4.3309	4.284	3.7948	3.7653	4.2163	
4.651	4.3869	4.2037	3.1879	4.0142	3.8522	4.2015	4.0173	
4.2098	4.2611	4.3242	4.4101	3.9362	4.3631	3.6358	3.9408	
4.9113	4.4842	4.1052	4.3093	4.2586	3.7254	4.0584	4.0576	
4.6288	4.8203	4.2777	4.3047	4.8899	4.7165	3.3575	4.6012	
4.4293	3.649	4.2562	4.6469	4.1063	3.9674	3.5582	4.6061	
4.1093	4.7543	3.2127	4.2959	4.3116	3.2673	4.0686	3.5667	
3.8619	4.3864	4.4408	4.4792	3.6958	4.4825	3.8992	3.5704	
3.6414	3.5064	3.9998	3.5956	4.4508	4.7585	4.7421	3.9422	
3.9194	3.9074							
BPTF	3.8849	3.9206	4.1776	4.1564	3.8067	4.2232	3.5507	
3.5495	4.0161	3.741	4.3925	3.7626	3.376	4.3458	3.917	
4.242	4.2146	4.2826	3.8772	5.0113	4.7226	3.0043	3.3634	
3.4735	3.7003	4.0678	3.8369	4.8317	3.4485	3.9231	4.1547	
4.027	3.4675	3.6455	3.4683	4.0479	4.4842	4.1154	3.3764	
4.1559	3.842	3.902	3.2162	4.3031	4.7773	3.5073	4.7165	
4.5454	3.6069	4.7649	4.1591	3.6429	4.4806	4.3113	4.1041	
4.0711	4.222	3.7051	4.1033	3.7172	3.8593	2.9501	4.3844	
3.9134	5.2509	3.8727	5.0987	4.2677	4.7148	3.3764	3.7036	
3.5168	4.119	3.667	4.0246	4.4305	3.9232	3.9954	4.0937	
3.9807	4.2965	4.1464	3.7172	4.5407	3.2794	3.932	4.0049	
3.7553	4.205							
BPY2	0	0	0	0	0	0	0	0
	0	0	0	0	0	0	0	0
	0	0	0	0	0	0	0	0

	0	0	0	0	0	0	0	0
	0	0	0	0	0	0	0	0
	0	0	0	0	0	0	0	0
	0	0	0	0	0	0	0	0
	0	0	0	0	0	0	0	0
	0	0	0	0	0	0	0	0
	0	0	0	0	0	0	0	0
	0	0	0	0	0	0	0	0
	0							
BPY2B	0	0	0	0	0	0	0	0
	0	0	0	0	0	0	0	0
	0	0	0	0	0	0	0	0
	0	0	0	0	0	0	0	0
	0	0	0	0	0	0	0	0
	0	0	0	0	0	0	0	0
	0	0	0	0	0	0	0	0
	0	0	0	0	0	0	0	0
	0	0	0	0	0	0	0	0
	0	0	0	0	0	0	0	0
	0	0	0	0	0	0	0	0
	0							
BRAF	2.1462	2.3117	2.3212	2.5089	2.4757	3.3827	2.9575	
3.1366	2.7663	2.661	2.2946	2.9134	2.5262	2.717	1.9935	
3.3166	2.7147	3.3677	3.1614	2.1222	2.2154	2.1868	2.657	
2.5427	3.0191	2.4643	4.0829	1.8766	2.6981	2.5143	2.6601	
2.581	1.9082	2.8383	2.6919	2.7449	2.9949	2.414	2.8008	
2.8175	2.4585	2.4047	2.6967	2.7932	3.0046	2.8567	2.5988	
2.8221	3.0903	2.609	2.9544	2.9716	3.0782	2.6531	2.4993	
2.6258	2.3345	2.8028	2.7294	3.0965	2.5247	2.6014	2.9448	
2.5461	2.7456	2.317	2.3905	2.2565	2.2676	2.4594	2.4009	
2.5588	2.9793	3.4993	2.0926	3.1202	2.4534	2.8021	2.5466	
2.668	2.8572	2.3863	4.1935	2.56	2.005	2.5116	2.4249	
2.0738	2.1898							
BRAP	2.8868	3.1012	3.4647	3.1885	3.1178	3.0047	3.7966	
3.5933	3.1303	3.3108	2.9864	2.6201	2.6367	3.2639	2.7257	
2.9611	3.2956	3.6098	3.7533	2.951	2.9611	3.0838	2.7071	
3.2012	3.2368	3.13	3.4148	2.9194	2.5313	3.2237	2.7133	
3.1905	2.9972	2.3243	2.5739	3.3357	3.2651	3.2904	3.3608	
3.4013	2.8101	3.032	2.3845	2.8493	2.7759	3.2262	3.2666	
2.9897	3.0493	3.5955	3.0386	3.2722	3.4552	3.5225	2.9018	
3.4398	3.1166	3.2635	3.1455	2.9046	3.3741	3.4522	2.8361	
2.8352	3.2721	3.0738	2.9874	3.328	2.9892	2.7414	3.2349	
2.7057	3.0219	3.0698	2.6747	2.3174	3.4379	2.5643	3.71	
3.3209	2.9046	2.8243	3.8487	2.9778	2.6494	3.2844	2.948	
2.9318	2.7097							
BRAT1	3.9357	3.4678	4.9253	2.7468	3.8039	3.2356	2.4822	
4.396	3.0868	2.7738	4.4417	1.9038	3.0612	2.9978	3.8097	
3.3377	3.2559	2.9506	2.7759	4.3836	4.7083	4.3972	4.0155	
3.9066	4.4444	3.0456	2.6701	3.5473	3.8814	4.0136	4.1654	
4.4626	3.663	3.2094	4.0518	2.4901	4.4202	4.0697	4.2368	

2.6998	4.0263	2.7611	3.5866	3.4825	2.6836	3.1724	2.8492
3.4589	4.3067	1.7749	3.0612	2.4091	2.931	3.4559	4.3351
4.7055	2.6256	4.2953	3.4255	4.3734	3.9762	4.1202	4.0757
2.4364	4.8297	2.8916	3.4185	3.2191	3.0994	3.6717	3.2897
3.9584	4.7464	3.5184	2.8774	3.6912	2.9673	3.7722	3.053
3.2397	2.9813	2.7872	3.9979	3.6436	4.5608	4.4687	3.0316
3.7927	3.6917						
BRCA1	3.8179	3.8227	3.6494	4.1143	3.2772	3.3658	2.3345
2.9896	3.7008	4.3091	3.6914	2.9512	4.0517	3.9229	2.9178
2.8312	3.3692	4.1118	4.6216	2.8718	3.5598	3.3595	2.4313
3.1567	3.1578	2.4017	0.9896	5.3144	3.3151	3.6424	3.3417
4.1914	3.4561	3.2985	3.4109	4.5316	3.8708	1.8826	2.7683
3.9365	2.3704	2.0485	3.3997	4.2494	3.3618	4.1548	4.3633
3.9767	2.1238	3.9264	3.4749	4.0123	3.4403	2.9746	3.0497
3.9581	2.5551	3.2945	3.8683	3.5155	3.8064	3.824	3.007
3.9483	3.7805	1.3444	3.3824	2.3377	4.0291	4.0942	3.4733
4.1536	3.3751	3.1999	3.2862	4.0271	3.6052	2.7215	3.9987
2.2922	4.2068	1.8276	1.7327	2.4067	4.1962	3.7094	3.091
3.5269	3.3993						
BRCA2	1.9	1.996	2.2919	2.1496	1.4885	1.948	1.8407
1.8218	2.0128	1.7701	0.9963	0.9281	2.768	2.252	1.5223
0.9394	1.9291	1.5671	2.182	1.1975	1.8154	1.6866	0.9531
2.1743	1.9932	0.8504	0.0465	2.1539	1.0531	1.2786	1.0781
1.0994	1.5835	0.9531	1.2464	1.7918	1.3733	0.6324	1.2517
1.2737	0.8817	0.6353	1.8195	2.6128	1.7718	1.4849	3.3069
1.7614	0.8018	2.6427	1.9007	1.9498	1.631	1.3302	1.3467
2.4189	1.5354	1.1171	2.0868	1.2395	2.0187	1.6031	1.748
2.1064	1.6675	0.4084	1.6229	0.5562	2.1258	2.8809	1.3126
2.3144	2.5672	1.1505	1.1746	1.3576	1.7875	0.8292	1.9001
2.6156	2.33	0.9001	0.6775	0.7634	0.881	1.8615	1.4898
2.2361	1.6682						
BRCC3	2.4093	2.3696	2.7947	2.6429	2.9464	2.4661	2.3347
2.2265	2.931	2.406	1.9213	2.3036	2.3952	2.7076	2.703
2.1276	2.8992	2.44	2.7522	2.5657	2.571	2.6161	1.9976
3.2214	1.931	2.5219	1.4039	2.7084	2.5823	2.3099	2.9687
2.5657	3.2679	2.1111	2.2406	2.6115	2.2167	1.9627	1.7387
3.2268	1.9808	2.1674	1.9713	2.0447	2.6978	2.7174	2.8068
2.7258	2.2007	3.9548	1.8938	2.6282	2.0342	2.2895	2.4547
3.3831	2.3797	2.7008	1.8898	3.0325	2.021	2.1804	2.5686
3.049	2.3189	1.8774	2.5656	2.7471	2.8994	2.7859	2.3871
2.2715	2.3387	1.4668	2.6373	2.3541	2.7628	2.6906	2.7962
1.7839	3.1224	2.3731	2.3406	2.229	2.1565	1.559	2.074
2.3273	2.0548						
BRD1	3.623	3.2806	3.6173	3.0305	2.4132	3.3706	2.8832
4.0433	3.0052	3.5906	3.3116	2.1047	3.6007	3.0713	3.6971
2.6791	2.4175	2.991	3.584	2.9959	3.5021	3.5473	2.5492
3.2748	3.8718	2.5399	2.9325	3.8759	2.8988	3.1109	2.9097
1.8453	2.967	3.0939	2.7799	3.1364	3.4416	2.7325	4.3916
2.9544	2.8264	2.2366	2.6327	3.7633	2.9674	2.8824	3.8995
3.122	3.7664	2.8365	3.0565	2.4403	3.7539	4.2846	2.5961

3.5934	3.4079	3.4695	3.572	3.4259	3.4255	3.4506	3.8775
3.3117	3.3974	3.0094	3.5077	3.0066	3.8023	3.0441	3.5598
3.3755	3.1781	1.1297	3.7351	2.7099	3.2804	2.8231	3.0701
3.414	3.4399	3.247	2.6038	3.4837	3.7088	3.7501	3.4751
3.6967	3.6959						
BRD2	5.9433	6.0215	5.7867	6.2736	5.643	6.2664	5.1496
6.088	5.5688	6.1193	5.5762	6.3426	4.7134	5.8356	5.8871
5.1721	6.7871	6.0717	5.7658	5.6549	5.7627	6.073	5.5331
5.3869	6.1068	6.078	6.3307	6.166	5.9797	5.1786	6.3574
5.664	5.3731	5.1051	5.6515	5.896	6.5517	7.007	6.0458
5.7019	5.6451	5.646	5.8621	6.2048	5.4097	6.1828	5.7678
6.1478	6.0555	6.0168	5.9763	5.6487	5.6421	6.4528	6.4608
5.6283	5.7481	6.3997	5.2468	6.408	6.0554	5.6615	6.0146
5.8563	6.2944	5.1166	6.4769	5.9221	6.0488	5.784	5.5888
5.914	5.5417	5.8146	5.5943	5.6515	6.2239	6.0784	6.0453
5.4727	5.4342	6.0735	6.8486	5.3069	5.6274	5.0333	5.6292
5.4889	5.6308						
BRD3	2.4801	1.9836	2.9042	2.9426	3.0805	2.6426	2.4098
2.8085	2.1179	2.3793	3.0902	3.1377	3.5991	2.6129	2.8148
1.8506	3.5082	2.9371	2.2836	2.931	2.9467	2.4776	2.505
1.974	3.7139	2.7281	3.1789	3.7182	2.2105	2.9401	4.2118
2.5585	1.8861	2.6196	2.0956	2.5793	2.879	3.4807	3.9751
2.2816	2.9228	3.2061	3.1035	3.0363	3.0773	3.095	2.8466
2.7433	3.3106	4.3818	2.6001	3.4007	3.363	3.2532	3.2678
3.1856	3.9559	3.3196	1.8572	3.5777	3.1241	2.0185	3.0504
3.3112	3.8345	3.6073	2.1116	3.1047	3.5472	4.0247	2.606
2.7833	2.6143	2.5699	3.0533	2.7804	2.5826	2.8421	2.9131
2.8492	2.9406	2.8746	2.286	4.0665	2.9733	3.3293	3.0321
3.2516	3.2833						
BRD4	3.9984	4.4886	4.8297	4.0921	4.5918	3.8889	4.1443
3.9716	3.8698	4.7996	3.9283	4.2758	5.2839	4.6399	4.1427
3.8011	4.5604	3.5173	3.9388	3.8705	3.4358	4.3436	3.8896
2.8202	4.1663	4.7928	5.0643	4.8166	3.7232	4.0979	4.1942
4.2794	4.2223	3.8745	3.8233	3.9637	4.6441	4.4866	5.1387
4.1322	4.0504	3.71	4.3284	4.5485	4.4641	3.9217	4.0045
4.3509	4.8635	4.3567	4.0059	4.0031	3.9896	5.7509	4.2662
4.8705	4.6745	4.3869	3.9303	4.9101	4.5578	4.0963	3.5635
4.3813	4.3424	4.2405	4.2132	3.8723	5.4913	4.2771	4.4565
4.5033	3.8554	4.1837	4.0195	4.2459	4.3783	3.9515	4.1116
3.8841	4.4223	4.294	4.2593	4.4892	4.5303	3.7747	3.7638
3.7425	3.7147						
BRD7	4.4094	5.2349	4.8744	5.048	4.6463	4.668	3.2731
4.3708	4.644	4.5299	4.0823	4.0478	4.7094	4.9512	4.412
4.0904	4.5054	4.3025	4.5104	4.2155	4.7416	4.7191	4.1857
4.5633	4.6529	4.5851	4.2718	5.0949	5.2927	4.4777	4.9925
4.5787	3.9063	3.8362	4.672	4.7604	4.6738	4.5906	4.3164
4.4071	4.4094	4.1701	4.1994	4.3262	5.4018	4.8738	4.8242
4.3777	3.8898	4.8361	4.0536	5.024	4.4971	4.3615	5.3843
4.6205	4.9511	4.4087	4.295	4.9185	4.5765	4.6736	4.9712
4.4532	4.3705	4.0195	4.2621	4.8434	4.7094	4.5229	4.5241

4.7234	4.3445	3.8166	4.8958	4.796	4.3163	4.8175	4.9357	
4.2485	4.6159	4.3433	3.5748	4.5319	4.7099	4.5525	4.2869	
4.3289	4.4435							
BRD8	3.2393	4.0845	3.8852	4.2728	4.3558	4.3282	4.2812	
3.6918	4.1401	3.8612	4.1957	4.3783	4.4892	4.6546	4.8984	
4.062	4.8928	3.924	4.3011	4.0521	4.0092	3.9169	3.9522	
3.7978	4.1738	4.2652	3.9933	4.3233	4.087	3.7531	4.2754	
4.9429	4.3697	4.0348	4.449	4.2977	3.4623	4.0899	3.4264	
4.4242	3.6643	4.3267	3.5846	3.2263	4.4585	4.9746	3.6377	
3.9705	4.5423	4.6447	3.8475	4.4851	4.5741	4.3948	3.6707	
3.7502	4.083	3.9794	3.5226	4.1281	4.2467	4.262	4.5193	
4.2559	4.1014	4.0322	3.7853	3.3809	3.9374	4.3851	3.2751	
4.3784	3.9166	4.4642	3.7248	4.071	4.6257	4.1369	4.2473	
4.1346	4.855	3.6981	4.242	3.7016	4.5434	4.1583	4.1852	
3.9454	4.3187							
BRD9	4.8036	4.6499	4.1096	4.2051	3.8075	3.63	3.0295	
3.8741	4.1279	4.0141	3.8011	3.8223	4.0915	3.7836	4.2275	
3.6774	4.2347	3.4735	4.4308	3.1592	3.649	4.1258	3.367	
4.5291	3.7138	3.6617	3.7616	3.1627	4.1016	4.1522	5.9668	
2.2801	3.4158	3.1663	3.7558	4.2136	3.6312	3.9606	3.7297	
4.0475	3.9255	4.2725	4.5732	4.1408	3.7301	3.6849	3.6359	
3.2206	3.833	3.5083	3.7065	4.2694	3.5973	4.2733	5.2845	
4.2271	3.7783	3.7917	5.2	3.8363	4.4424	4.593	4.3086	
3.8939	3.4843	2.7708	3.4409	3.8908	2.75	4.0098	3.678	
3.9606	3.8879	4.0077	3.2763	4.1319	3.4706	4.1344	3.3495	
3.9215	3.419	4.5303	3.6092	3.3691	4.5318	4.0781	3.2137	
3.7188	3.6177							
BRDT	0.0873	0.0423	0.0258	0	0.0128	0	0	0
	0	0.0099	0	0	0	0	0	
0.011	0	0	0.0509	0	0	0	0	0
	0	0	0.0133	0.0294	0.0112	0	0.006	
0.0028	0	0	0	0	0	0	0	0
	0	0.0016	0	0	0	0.0107	0	0
	0.0095	0	0	0.0133	0	0.0037	0	0
	0	0	0.0659	0	0	0.0203	0	0
	0.0299	0.0018	0	4.00E-04	0	0	0.0058	0
	0.0378	0	0.009	0	0.0282	0	0	0
	0.0062	0	0.0063	0.0135	0.0359	0	0	0
	0							
BRE	4.9633	4.7378	4.1578	4.8701	4.7619	4.7237	3.9968	
4.0322	5.4229	5.5168	4.8487	5.2687	5.3222	4.9966	4.4072	
4.9031	5.1878	5.3153	4.822	3.9279	4.1232	3.9896	4.7871	
4.9753	4.279	3.8834	4.3718	4.4709	4.494	5.4003	4.8487	
5.1204	4.9578	5.1807	4.8017	5.2506	5.4627	5.5038	4.3251	
4.9465	4.7281	5.075	5.0966	5.4826	5.0301	4.4573	5.1864	
4.5661	4.9477	6.579	5.0078	4.885	4.7308	4.4365	4.7212	
4.5316	5.3437	4.1661	4.1972	3.8181	4.6011	4.6256	4.2737	
4.9124	5.1048	5.0777	5.8535	5.0743	4.9792	5.0222	5.4535	
4.594	4.585	4.5422	5.7506	4.4476	4.5765	5.2669	5.1507	
4.7136	4.8266	5.7275	4.8803	4.5187	4.3376	4.2265	5.3387	

5.0756	5.1688						
BRF1	3.1047	3.1976	2.7988	2.4703	2.9717	3.2304	2.6378
3.2056	2.3782	2.6588	2.6084	2.025	2.7513	2.2533	2.8782
3.1018	2.6446	2.5768	2.9601	2.2252	3.2078	2.8136	2.9075
2.5206	2.583	2.5959	2.1298	2.7795	2.7663	2.2366	2.6478
3.0915	2.8099	2.1349	2.0935	1.9821	2.8168	3.6731	3.1088
2.5622	2.9897	2.5385	2.4206	2.9931	3.2897	2.6696	1.9627
3.1285	2.99	2.6215	1.9609	2.7052	1.9337	2.8681	2.3759
3.1227	2.6813	3.2163	2.5577	3.0994	3.05	3.318	3.0934
2.3731	2.9262	3.0118	2.6589	2.9208	2.4866	3.0139	3.0878
2.4242	2.9021	2.3397	3.7033	1.6459	2.9344	1.9161	3.2686
2.9657	2.8446	2.8571	2.8192	2.8178	3.0925	2.69	2.5039
2.9726	3.1756						
BRF2	3.1392	2.7739	2.368	2.7399	2.4783	2.4455	2.6284
3.3637	2.2504	1.7624	2.6387	2.0647	2.7975	2.8268	2.5171
2.8669	2.7975	2.2922	2.5056	2.5004	2.6848	3.2141	2.2285
2.4921	2.9701	3.0864	2.9749	2.6224	2.3087	2.3852	3.2826
2.4298	2.6118	3.4965	3.4242	2.1353	3.4513	3.2386	3.304
2.8608	2.3607	2.4989	2.8451	1.7315	2.8595	3.2207	2.905
2.1071	2.4056	3.8292	2.6768	2.4223	2.9848	2.6677	3.1395
2.6635	2.2447	3.1221	2.6911	3.2472	2.603	2.7564	3.1651
2.3992	3.6005	2.3075	2.6844	3.2067	1.7543	3.1106	2.3835
2.2116	3.3631	2.4714	2.5195	2.7314	3.0016	3.1278	1.8345
2.1123	2.2144	2.0897	3.7869	2.3124	3.1537	2.8104	2.7208
2.458	2.2375						
BRI3	8.3486	7.9955	7.5642	6.4778	6.0396	8.7706	8.1014
8.305	8.0499	8.1889	8.741	6.6916	8.1219	7.6516	7.3609
8.305	6.6181	8.5903	8.3428	7.4166	7.6958	7.1511	7.9144
8.4792	7.555	6.963	7.1675	7.6677	7.9372	7.9048	6.6686
7.7756	7.5875	7.6743	8.3968	7.3519	8.1669	7.6648	8.0399
6.3406	8.1281	7.1463	7.5581	8.1944	6.918	7.0481	6.737
7.9187	8.4792	6.0598	7.9955	6.7137	6.6307	7.0659	7.0368
7.7103	6.6285	7.0427	9.3308	7.2603	7.3994	7.8859	7.6119
8.4514	7.8167	8.2451	9.0016	8.4273	7.9114	7.1105	7.8524
8.346	8.2836	6.2645	7.9614	7.5683	8.0028	7.8284	7.682
8.2175	7.94	6.7204	6.7594	7.5231	7.3184	7.3994	8.0196
7.7721	8.0095						
BRI3BP	2.8707	2.9172	4.1805	2.1195	3.6243	2.4597	1.4437
2.7771	1.6949	2.779	2.1099	1.0057	1.3135	1.9051	2.4618
2.6499	2.5413	1.7376	2.5898	3.3459	3.5058	2.6098	2.2882
3.6352	2.0886	0.6687	0.5589	2.5862	2.0116	1.6564	2.724
2.9536	3.2934	2.3272	2.0293	1.9922	2.3141	2.1123	1.8457
2.1608	1.9527	0.8655	2.2009	2.9044	1.7752	1.7602	2.1419
2.3487	2.4483	1.5753	1.4271	1.8314	1.6484	1.4809	3.0543
4.1451	1.6502	3.6726	2.6005	3.3717	2.9626	3.3122	2.1088
2.917	2.6075	0.5205	3.1435	2.1597	2.7575	2.0957	2.5035
2.4034	2.9558	1.3124	3.0119	4.1437	2.7626	2.202	1.6762
2.4722	1.9492	2.202	2.481	1.7765	2.1907	2.3494	1.8695
2.987	2.3433						
BRICD5	1.1541	1.8383	1.5435	0.511	1.1103	0.623	0.9762

1.3772	0.7704	0.8831	1.232	0.5169	1.0082	0	1.4465	
0.9388	1.5876	0.8548	0.7704	1.9765	1.951	1.4733	0.95	
1.4715	1.086	0.5248	0.806	1.4208	0.8694	1.2871	1.1521	
1.8483	1.1664	1.1187	1.1182	0.418	1.2107	1.2297	1.3254	
0.5802	1.201	0.7065	0.7305	1.2643	0.5886	0.9573	0.4878	
0.8354	1.3907	0.7561	1.0992	0.4432	0.7981	1.4712	1.1127	
1.5639	0.7561	1.2736	1.7125	1.5301	2.1086	1.4328	1.6691	
1.5419	1.2048	0.8691	1.2521	1.0664	2.1223	1.525	1.3321	
1.3869	1.0943	0.5065	1.3887	1.1508	1.4303	0.5949	0.9364	
0.9281	1.3469	0.8797	1.4513	1.3321	1.4076	1.7614	0.9034	
1.2666	1.4006							
BRINP1	0	0	0	0.1063	0	0	0	0
	0	0	0.0469	0.0459	0.1439	0	0.0501	0
	0	0	0	0	0	0	0.0557	
0.0976	0	0	0	0	0	0	0	
0.0722	0.0706	0	0.0483	0	0	0	0.0539	0
	0	0.15	0	0	0.053	0	0	0
	0	0	0	0	0	0	0.0794	
0.0828	0	0	0	0.0062	0.0098	0	0	
0.0134	0.3015	2.7571	0	0.0386	0	0	0	0
	0.005	0	0	0	0	0	0	0
	0	0	0	0	0	0	0.0823	0
	0							
BRINP2	0	0	0	0.036	0.4752	0.1398	0.0804	
0.045	0	0	0	0.0201	0.0159	0	0.4344	
0.0329	0.0345	0	0	0.1622	0	0.2468	0.0933	
0.7506	0.1281	0.1027	0	0	0.2049	0	0	0
	0.0564	0.0129	0.1847	0	0.0131	0	0.0224	
0.1064	0	0.0538	1.1927	0.0157	0.0649	0.0144	0	0
	0.0832	0	0.1057	0.2066	0	0	0	0
	0.0572	0.1265	0.0195	0	0.2269	0.0101	0	
0.0614	0.0237	0.4063	0	0	0.0105	0.5381	0.1397	
0.092	0	0	0.3057	0	0	0.1335	0	0
	0	0.0391	0	0	1.147	0	0	0
	0							
BRINP3	0	0.1984	0.0071	0.0434	0.0352	0	0	0
	0	0	0	0.057	0	0.1211	0	0
	0.0709	0	0	0.0097	0	0.5276	0.1278	0
	1.0221	0	0.1099	0.25	0	0.7175	3.0585	
0.4485	0	0	0	1.1906	0.1241	0	0	0
	1.3391	1.385	0.0039	0	0.0098	0.0492	0.5351	0
	0	0.0726	0	0.0124	0.0991	0.0639	3.1965	0
	0	0	0.5166	0	0.1017	0.0958	0	0
	0	0.065	0	0	0	0	0	0
	0.1681	0.273	0	0.3525	0	0	0.122	0
	0.0995	0.0531	0	0.0645	0	0.2113	0	0
	0							
BRIP1	2.5269	1.9691	2.7373	2.6847	2.1702	2.4468	1.8609	
1.6777	2.292	2.1094	1.3703	1.5818	2.8033	2.9787	2.1807	
2.6381	2.106	2.44	2.6656	2.2911	4.1809	0.9503	1.1808	

2.1614	1.572	1.9796	0.6293	3.4197	1.9199	1.9992	1.8322
2.0982	2.1791	1.6603	1.7312	2.5687	2.758	0.823	1.3153
2.1177	1.5702	0.9888	1.3238	2.8684	3.3836	2.5172	3.2004
2.4879	1.3742	3.6702	2.1289	2.3108	2.5943	1.3261	1.8803
2.2706	1.2097	2.5058	1.9089	2.7689	2.245	2.0897	2.4115
2.7497	2.9536	0.8523	2.8117	1.0652	3.154	2.0198	1.6309
2.6506	2.5431	0.8177	2.4058	2.9442	2.5789	1.4911	2.3245
2.45	2.9442	1.1707	0.5484	1.9151	2.6681	3.1263	1.4705
2.7325	2.1781						
BRIX1	6.4184	6.6746	5.8461	6.7231	6.5252	5.3381	4.8692
5.8106	5.3062	5.2082	5.4686	5.9241	5.8352	5.8451	5.5899
4.9932	5.7678	5.2003	6.5131	5.4547	5.8737	5.9251	5.066
6.3518	5.7101	5.1669	5.885	4.8468	5.6334	6.3655	5.7603
4.4331	5.1507	3.9633	4.8184	5.8175	5.6828	5.1725	5.5832
5.8579	4.9169	5.6665	5.911	5.6179	5.4195	5.5648	5.8819
5.3557	4.6605	5.9595	5.4233	6.5541	5.4378	5.9199	6.335
6.2913	5.1205	5.5909	7.2021	5.8926	5.9731	6.1648	5.5042
5.6941	5.2438	4.4278	5.4641	4.2904	4.7095	5.8115	4.8023
5.781	5.7223	5.5271	4.5741	5.322	5.8352	5.1218	5.2736
5.937	5.4136	5.8021	4.4322	4.7669	5.9979	5.6284	3.9022
4.9482	4.3026						
BRK1	6.3473	6.9343	6.4192	6.7952	6.5042	6.49	6.4343
6.1752	6.0274	6.39	7.0146	7.2571	7.0395	6.676	7.1534
7.9552	6.9848	6.8782	6.4145	6.5558	6.4428	6.153	6.7075
6.4294	6.4552	6.9948	7.1019	7.0259	7.0614	6.6868	7.9074
6.7453	6.49	6.2489	6.7453	6.8496	6.6678	5.7848	7.0285
6.3296	6.39	6.8807	6.7114	6.9639	7.6333	6.269	6.8312
6.1863	7.1357	7.6477	6.2967	6.6741	6.6157	6.5222	6.8251
6.1845	6.6274	6.4238	6.2635	6.4918	6.5051	6.2431	5.7679
6.4524	6.439	7.3051	7.161	7.2183	7.0207	6.4496	6.7838
7.874	6.7904	6.3136	7.2871	6.279	6.311	6.9222	6.0585
6.1522	6.2031	6.8614	6.8101	6.5293	7.2276	6.0651	6.939
6.8018	6.739						
BRMS1	4.4786	4.5991	5.4927	3.8753	4.8658	4.6548	4.5589
5.9277	4.1654	4.3733	4.5917	3.6953	4.7172	4.2149	5.6173
4.5642	5.0409	3.0409	4.7574	5.1379	5.36	4.8132	4.4656
5.1213	5.3236	4.7235	3.2599	4.8497	5.203	3.7351	4.8793
4.0287	5.071	4.0211	4.6289	3.9331	5.0933	5.2338	4.9126
3.7943	4.4946	4.2786	4.148	3.9599	3.8991	4.2853	4.0726
4.9041	4.5793	3.8771	4.0937	3.6265	3.5218	5.0389	5.1957
5.0709	3.9731	4.9504	5.1307	4.7252	4.2881	4.189	4.8056
4.2127	4.0975	4.1154	4.4044	4.9435	4.9639	4.7252	5.6174
4.3446	4.8879	3.5784	3.9063	5.0133	6.3199	4.0783	4.2844
4.6106	3.6395	4.2304	5.4516	3.9073	5.1611	4.2286	4.3783
4.893	4.6674						
BRMS1L	2.7171	2.7916	2.7479	3.0762	2.8241	3.0045	3.7999
2.6791	2.6249	2.4936	1.455	3.2522	2.3728	3.28	3.4672
2.8016	2.9625	3.2371	3.0695	2.7701	2.8908	2.697	3.5039
2.5728	2.3822	2.2383	2.2718	2.6581	3.1554	2.0188	4.2401
2.888	3.1411	2.0337	2.1949	2.8699	2.614	2.5925	2.2303

3.3858	2.602	1.8304	2.3912	2.8933	3.5976	3.4631	2.8704	
2.9089	2.4978	3.919	2.6948	3.8548	2.9001	2.8419	3.0984	
2.7934	2.4752	4.4575	2.7307	4.4673	3.2715	2.9485	2.9251	
2.822	3.1456	2.5901	3.33	2.5905	2.2397	2.1531	2.8261	
2.8672	2.9394	2.3549	3.6431	2.8237	3.0015	2.2729	2.9963	
2.0735	2.3342	2.8995	2.2344	2.6138	2.5478	3.182	2.7619	
3.3219	3.1266							
BROX	3.8208	4.4664	3.9471	4.8583	4.6747	4.5672	4.2321	
4.1987	3.6133	3.9843	3.3929	4.0269	4.1823	4.3451	4.6799	
4.3135	3.9522	4.5183	4.1	3.845	4.4697	3.6609	4.4323	
4.4477	4.6814	4.4721	5.4027	4.3821	4.3849	4.4387	3.8761	
4.1091	3.9127	3.9016	4.252	4.2233	4.3037	3.8765	4.0343	
4.7093	4.4426	4.2651	4.351	4.0169	4.3377	4.7493	3.6651	
4.8577	5.0275	4.2428	4.047	4.5628	4.2909	3.9052	4.0152	
4.7924	4.5353	4.0581	4.6981	4.4298	4.1773	4.2347	3.8109	
4.2405	4.6952	3.9753	4.2365	4.295	4.0467	4.3799	3.6489	
4.6696	4.2285	4.4089	4.7286	3.7113	4.2177	4.7555	4.3007	
3.3289	3.7292	3.5932	4.2492	3.8093	4.721	4.1638	4.4898	
4.0616	4.2449							
BRPF1	2.5282	2.8755	3.2495	2.3884	2.9578	2.5813	2.5012	
3.0877	1.8202	2.5539	2.664	2.5488	3.053	2.4143	3.0943	
3.4759	3.1263	2.3779	2.5841	2.8027	2.6794	2.566	2.2158	
2.5652	2.595	2.2582	2.0454	2.8563	2.6869	2.4414	3.0596	
3.3421	2.3168	1.9012	2.0956	2.2769	3.1694	2.0932	3.2186	
2.0666	2.0676	1.7963	2.2213	2.8921	3.0904	1.8407	2.6757	
2.3937	2.9128	2.497	2.5527	2.1593	2.1917	2.7719	2.6237	
2.6723	2.3123	2.9505	2.8211	3.1378	2.6691	2.5048	2.2567	
2.5703	2.4414	2.3083	2.7748	2.6672	3.3885	2.6101	2.3098	
3.8038	2.86	2.1656	2.956	1.9806	2.8188	2.5533	1.5525	
2.1762	2.2775	2.202	2.8298	2.2481	3.2131	2.3655	2.1479	
2.3775	2.4679							
BRPF3	3.2186	3.638	4.1511	3.766	3.1466	3.5156	3.4077	
4.317	3.0357	2.8691	1.8264	3.665	2.6442	3.0716	3.7985	
3.0155	3.95	3.875	3.7878	2.9452	3.1522	3.9543	3.6735	
2.5253	3.7601	4.4127	2.9897	4.0047	3.6271	1.845	3.5915	
3.3727	2.9919	2.1527	4.0514	3.0693	4.2768	4.2573	3.5252	
2.8487	2.5097	3.8442	2.8817	2.8639	3.866	4.4237	3.5287	
3.2151	3.8427	4.131	4.2972	4.0357	3.4278	3.8232	4.2697	
3.1488	3.2199	3.7266	1.9793	4.0006	3.0255	2.8782	3.7331	
3.2121	4.3744	2.5908	2.5685	3.9634	3.3395	3.1804	3.1454	
3.293	2.7578	3.166	2.9605	3.4342	3.9884	4.7253	3.188	
2.7927	3.0143	3.9544	4.6174	3.7883	3.887	3.2756	3.1974	
2.9498	2.9738							
BRS3	0	0	0	0	0	0	0	0
	0	0	0	0	0	0	0	
0.039	0	0	0	0	0	0	0	0
	0	0	0	0	0	0	0	0
	0	0	0	0	0	0	0	0
	0	0.0456	0	0	0	0	0	0
	0	0	0	0	0	0.0177	0	0

	0	0	0.0647	0	0	0	0	0
	0	0.0073	0	0	0	0	0	0
	0	0	0	0	0	0	0	0
	0	0	0	0	0	0	0	0
	0							
BRSK1	2.6955	2.241	2.4032	2.1281	3.4062	2.4974	2.5024	
4.366	1.1455	1.6828	2.6782	3.2738	2.6034	2.4075	2.5671	
1.8217	2.4051	1.6786	1.6615	4.6892	3.1783	4.009	3.9221	
3.1048	4.1614	3.0502	4.3131	2.0691	2.657	3.8094	2.5436	
2.9211	1.3259	2.8222	3.3306	1.7857	4.9915	1.6325	4.8801	
0.9128	3.2174	2.0171	3.8428	1.9223	3.0293	1.1615	1.901	
3.6756	3.1276	1.2103	3.5014	2.7618	1.7944	3.6725	2.0394	
1.1816	0.1262	2.2772	2.6824	2.3928	3.3892	3.5402	2.1784	
0.7713	2.2257	3.4025	3.4757	2.7838	4.1401	4.1283	2.133	
3.7621	1.843	3.9545	2.8054	3.2634	2.3635	2.6828	1.0972	
2.6497	2.4113	3.1437	2.7902	4.6994	3.9611	2.1252	1.4969	
1.4112	1.9128							
BRSK2	1.613	3.3362	4.3335	1.7556	0.6384	1.8054	1.204	
1.2032	1.1715	0.4545	1.9922	1.6142	1.2788	0.5542	1.4504	
2.1449	1.9361	0.66	3.2598	3.6046	2.6461	0.37	0.3707	
0.3007	0.9958	0.841	0.3006	1.9348	0.4149	2.9812	3.0387	
2.5312	2.254	0.8328	0.3673	1.1181	0.4002	1.4684	1.2573	
1.6029	0.4339	0.1943	2.0723	0.378	0.2992	0.1984	0.8771	
2.2071	1.2199	1.1049	2.9112	0	1.7477	2.3956	2.632	
3.666	0	3.6329	0.3321	3.6754	1.0607	1.5418	0.934	
1.0447	2.4223	0.5552	1.7621	1.7951	2.39	0.4799	0.8827	
1.1863	2.8536	1.1969	0.1613	0.6695	2.3108	0.5435	2.9043	
2.415	1.6259	1.4296	3.2497	1.3644	0	1.6053	0.454	
0.5815	0.9757							
BRWD1	2.9949	2.5632	2.7793	3.0764	2.7479	2.6385	4.0768	
2.4863	4.2941	3.3292	2.7271	2.5201	3.1619	3.8346	2.987	
3.4404	3.4797	3.1502	2.8373	3.0597	2.5962	4.0576	2.9082	
2.5937	3.9632	2.947	4.606	2.6249	3.0353	2.7293	4.0832	
2.6217	2.7228	3.4512	3.0593	2.9642	3.4014	2.3563	2.4178	
3.2264	2.9972	3.0672	2.4079	3.2903	2.6806	3.43	3.2903	
3.318	2.8532	3.5546	2.9851	3.3	3.6	3.7068	3.7692	
3.6965	4.2028	2.6174	3.1486	3.108	3.0316	2.6876	2.7344	
3.0542	2.6241	3.3742	3.0952	2.3562	3.7051	2.6626	2.4895	
2.7018	3.5752	2.4769	2.9296	3.3525	2.6831	3.2347	3.5646	
3.0876	3.4277	3.3348	2.7244	3.597	2.2654	2.7778	3.0895	
2.8393	3.0627							
BRWD3	1.7459	1.7527	1.8312	1.0031	2.3006	2.1606	1.804	
1.6994	2.5486	1.8879	2.8386	3.164	1.3823	1.9585	1.8739	
2.2523	2.3518	2.3181	1.8594	1.8354	1.8643	1.6872	2.1616	
2.4207	1.6717	1.435	3.1675	2.6328	1.8388	2.2763	3.1124	
2.3091	2.3078	2.4032	1.6517	2.376	2.1258	1.7349	1.2536	
2.5261	1.8029	1.5713	1.2696	2.4139	2.6084	2.1601	2.2565	
2.4466	2.6155	4.0596	1.8862	2.2583	2.4932	2.1255	2.849	
2.306	1.9286	2.0125	1.9689	2.2109	1.8926	1.5477	2.4012	
2.559	2.5665	1.2767	2.8186	1.0054	2.5132	1.8274	2.8978	

1.9261	2.3462	2.5857	2.1442	1.8535	1.6289	2.7543	2.0693
1.9004	2.0477	2.1158	1.9573	3.4448	1.426	1.9286	1.8471
1.7481	1.9815						
BSCL2	2.8884	5.7313	3.4691	3.0406	5.1891	3.1647	5.2999
5.6397	3.5784	2.5423	3.9274	3.0221	3.2328	2.539	2.5919
3.6928	2.019	2.2533	1.6456	4.1201	3.2906	5.2484	6.7848
3.5711	5.5239	3.9279	4.9991	4.63	5.3277	4.1951	4.6845
3.4035	3.7661	5.0867	4.9493	1.9071	4.2077	3.3686	4.4985
2.8194	5.944	5.6234	2.8892	3.3878	2.8797	2.4055	3.1902
3.9022	4.967	3.002	5.543	4.3297	2.6576	3.4125	4.2151
3.9684	0.8721	1.9036	4.9204	1.6869	3.9757	2.8471	4.8261
3.3409	3.9121	5.6848	1.8441	1.9071	3.1323	4.1116	5.7443
4.205	5.3483	4.8907	4.3342	4.4054	4.1836	5.7144	4.2181
5.0207	3.1294	5.1174	5.3179	4.5304	3.4191	3.4659	4.7688
4.8774	4.9327						
BSDC1	3.7034	3.7212	3.7171	3.3816	4.2412	4.4231	5.0531
4.0298	4.6344	4.1394	4.0729	4.1044	4.8738	4.1204	3.9995
4.7608	4.1862	3.7616	3.7217	4.4214	3.7963	4.1805	3.9295
3.8681	3.9042	5.3952	4.4803	3.9513	4.2281	3.9785	4.5135
3.9459	4.4278	4.6334	4.7143	3.8065	4.7672	4.5925	4.014
3.9142	3.8868	4.4585	4.1414	4.1441	4.7011	4.4209	3.0876
4.3432	4.5709	3.6932	4.1208	3.7129	4.4943	4.5909	3.6441
3.8084	4.5311	3.711	3.8035	3.7021	3.775	4.1855	3.1697
4.4729	3.9038	5.2934	3.5992	4.8212	4.2969	3.8614	3.7563
4.3327	4.1489	4.616	4.2216	4.4314	4.0356	4.3221	3.9932
4.0577	3.7466	4.1211	4.7351	4.1781	4.2075	4.3915	4.632
4.0876	4.5144						
BSG	8.3483	7.9385	8.0412	7.1948	8.0534	7.93	7.7289
8.1308	7.4811	7.6292	9.1344	6.8014	8.6187	6.7149	8.5949
7.659	8.0286	7.2026	7.224	8.296	8.555	8.1727	7.8627
7.6203	7.2779	8.2584	7.612	7.7994	7.8516	7.7324	8.4533
8.5149	8.107	8.2684	7.4994	6.8253	8.2906	8.9301	8.5949
7.0353	8.7653	8.2426	8.1582	7.6438	7.4367	7.2371	7.131
7.5791	8.1024	6.4778	7.6292	6.8355	7.0863	7.2511	8.0917
8.6804	7.0727	8.551	7.6034	8.154	7.9368	6.9377	7.9561
8.2735	8.2621	8.9657	7.6034	8.6471	8.7872	8.2089	8.3147
8.0169	7.4806	8.1024	7.5848	8.4309	8.2089	7.3193	8.8742
8.0532	7.872	8.6992	8.2089	7.6924	8.7166	7.8299	8.3994
8.7202	8.4675						
BSN	0.201	0.4083	0.4344	0.052	0.1048	0.084	0.0981
0.415	0.1115	0.2644	0.2248	0.0211	0.1461	0.1683	0.3588
0.2087	1.2337	0.1201	0.2459	0.1019	0.1354	0.067	0.0346
0.1695	0.0579	0.0519	0	0.1213	0.3666	0.1191	0.4099
0.1789	0.141	0.0351	0.0654	0.0802	0.1901	0.1907	0.0898
0.1418	0.0543	0.0224	0.0845	0.0983	0.0404	0.0125	0.0709
0.1695	0.0899	0.1597	0.0471	0.0633	0.0411	0.7736	0.4063
0.2156	0.0156	0.2232	0.1213	0.2953	0.0841	0.144	0.0514
0.0623	0.1287	0.0892	0.1663	0.2878	0.3083	0.0923	0.0477
0.2225	0.2959	0.0353	0.1907	0.0645	0.087	0.0748	0.0959
0.0828	0.0203	0.0705	0.9232	0.0987	0.0472	0.0268	0.0654

0.1309	0.0942							
BSND	0	0	0	0	0	0	0	0
	0	0	0.0463	0	0	0	0	0
	0	0	0	0	0	0	0	0
	0	0	0	0	0	0	0	0
	0	0	0	0	0	0	0	0
	0	0.0113	0	0	0	0	0	0
	0	0	0	0	0	0	0	0
	0	0	0	0	0	0	0	0
	0	0	0	0	0	0	0	0
	0	0	0	0	0	0	0	0
	0.037	0	0	0	0	0	0	0
	0							
BSPH1	0	0.159	0.1151	0	0	0	0	0
	0	0	0.0356	0	0	0	0	0
	0	0	0	0	0	0	0	0
	0	0	0	0	0	0.0606	0	0
	0	0	0	0	0	0	0	0
	0	0	0	0	0	0	0	0
	0	0	0	0	0	0	0	0
	0	0	0	0	0	0.1315	0	0
	0	0	0	0	0	0	0	0
	0	0	0	0	0	0	0	0
	0	0	0	0	0	0	0	0
	0							
BSPRY	0.055	0.1298	0.0486	0	0	0	0.0405	
0.0413	0.1564	0.9903	0.2835	0.009	0	0.1056	0.0538	
0.0566	0.0509	0.0798	0.1226	0.0495	0.0432	0	0.0064	
0.0928	0.012	0	0	0.0921	0	0.5847	0.3092	
0.5307	0.1547	0.1129	0	0.6511	0.0966	0.0352	0.0766	0
	0.0206	0.0044	0	0.1136	0	0.0495	0	0
	0.0204	0	0.285	0.0299	0.058	0.1201	0.229	
0.1897	0.1919	0.1247	1.0333	0.0553	0.0589	0	0.0198	0
	0.0684	0.061	0	0.0209	0.0144	0	0.0198	
0.0465	0.0272	0.1987	0.0203	0.0257	0.054	0	0.0528	
0.2234	0.0774	0.1718	0.2651	0.0536	0.0412	0.1716	0.0357	
0.164	0.0454							
BST1	0.1728	0	1.0299	3.9286	2.3767	0.2428	3.418	
0.3398	3.5182	1.0003	1.0495	2.1536	2.2368	3.4163	2.741	0
	0.1815	1.1811	0.4863	0.2951	0.7816	3.2728	3.3307	
1.5162	3.4054	3.8942	1.2234	1.1599	3.8638	0.4729	0.0471	
1.0399	3.2904	1.7956	3.8338	0.3598	1.4538	0.2241	0.2436	
0.7198	1.0269	2.8808	4.7088	3.7586	3.4363	0.5331	2.129	
1.3874	0.2273	2.2101	3.182	4.3198	2.098	0.5682	0	
0.6624	2.8074	0.942	1.3208	0.7832	0.6023	0.741	2.9399	
2.5656	0.6854	0.4534	0.3408	3.4282	2.4255	4.8758	0.3066	
1.8014	0.7746	3.301	4.5393	3.9303	2.0623	0.6385	2.2304	
0.4085	0.386	4.9171	2.2952	1.9472	3.6051	2.0761	3.5894	
3.464	3.0452							
BST2	5.3956	1.7839	0.5997	2.9537	0.4354	1.9571	8.6868	

0.0503	1.102	6.0809	0	7.1481	6.668	5.8639	1.25	
4.9426	0.9611	4.6038	4.8837	0	0	2.2021	5.6017	
5.7582	1.5627	5.8916	3.1633	2.5953	0.1897	1.1014	1.3125	
3.327	6.4625	8.2924	5.4888	5.1853	8.1758	7.6	0.6665	
5.6961	2.2444	8.2924	0.146	5.4018	4.0373	6.448	0.1235	
6.875	7.2985	2.1333	6.8584	6.3285	5.7938	3.4267	0.6082	
1.4373	1.1279	0.59	2.4446	1.1509	2.4714	3.742	1.9939	
5.3037	0.8887	8.0984	2.4489	7.8523	1.919	0.6138	1.5821	
5.4772	3.4311	7.9685	0.8087	0.8191	7.981	7.6686	7.9958	
2.9422	1.5353	7.8659	8.2796	0.5861	2.6965	6.9114	1.3795	1.4
	0.7164							
BSX	0	0	0	0	0	0	0	0
	0	0	0	0	0	0	0	0
	0	0	0	0.2482	0	0	0	0
	0	0	0	0	0	0	0	0
	0	0	0	0	0	0	0	0
	0	0	0	0	0	0	0	0
	0	0	0	0	0	0	0.1031	0
	0	0	0	0	0	0	0	0
	0	0	0	0	0	0	0	0
	0	0	0	0.1629	0	0	0	0
	0	0	0	0	0	0	0	0
	0							
BTAF1	3.4751	3.4446	3.5406	3.3272	3.4725	3.5025	3.2453	
3.4108	3.0775	3.8318	3.5326	3.8414	3.7407	3.9647	3.4446	
3.0467	3.481	3.5275	3.5937	2.9942	3.5798	2.7476	2.5914	
3.3634	3.2006	3.3287	3.5504	3.7844	2.6948	2.6897	3.0251	
3.6496	2.8866	3.0291	3.1024	3.8774	3.9813	2.7024	3.7179	
3.5794	3.5911	2.6368	3.0532	4.0505	3.3832	3.5895	3.8234	
3.6739	3.1389	3.8713	3.6248	3.3959	3.8257	3.7961	3.7803	
3.1606	3.4908	3.2349	3.6098	3.4409	3.0571	3.1964	3.9919	
3.7791	3.4061	3.6137	3.8235	3.2062	3.4095	2.8884	3.0908	
3.2944	2.7314	3.5437	3.0621	3.2739	3.8228	3.2012	3.0102	
2.8664	3.6852	3.5835	3.2604	3.359	2.029	3.2293	3.147	
3.4949	3.4073							
BTBD1	5.59	5.6769	5.8694	5.6601	5.1643	5.6236	6.0132	
5.1017	5.4054	5.7056	5.7453	5.1615	4.8855	5.5832	4.9529	
5.8475	5.0132	6.3144	6.3061	5.1023	5.828	4.7264	5.5348	
5.8864	4.9497	5.0775	4.5151	5.4973	5.5497	6.5505	5.5133	
5.4276	5.6264	4.9361	5.5271	6.1512	5.3715	5.5928	5.3463	
5.5382	5.5288	5.6057	5.0982	5.3659	5.8058	4.8712	5.0939	
5.6556	4.8783	6.1803	5.3174	4.8809	5.5737	5.9963	4.9357	
5.7184	5.0803	5.1761	5.86	5.2405	5.6347	5.7413	5.0395	
6.0777	4.9672	5.4077	5.4683	5.7652	6.2999	4.6843	4.7976	
5.1825	6.6267	5.4228	5.6899	5.5915	5.5419	5.3303	5.9124	
5.439	5.466	5.3889	5.6992	4.6038	5.1886	5.4011	5.5046	
5.6039	5.2433							
BTBD10	3.5574	5.6291	5.8219	3.7007	3.3118	3.5829	5.3421	
4.1827	4.1457	3.3818	3.7655	3.2766	3.3213	3.5936	3.8128	
3.7327	4.8469	3.3671	3.816	3.532	3.2848	3.7218	3.4582	

3.9094	3.9036	4.3038	3.5624	6.3599	3.607	5.4521	3.6149	
4.0245	3.916	3.4013	3.4479	3.8168	3.477	3.822	2.8259	
3.5111	3.7818	3.8608	4.346	3.3466	4.3662	4.1364	5.8775	
3.557	3.5395	4.8272	4.1078	3.6911	3.7015	3.3034	3.9385	
2.998	5.1746	3.3318	4.3118	3.5637	3.3131	4.9178	3.5426	
3.94	3.7135	4.6963	4.3717	4.2628	6.7458	3.1703	3.6564	
3.5119	3.6124	3.5573	4.101	4.3454	3.4709	3.815	2.0824	
4.0187	3.3549	4.0055	3.9686	3.2868	3.4302	4.1229	4.1038	
3.5432	3.4504							
BTBD11	0.0201	0.6141	0.3026	0.0396	1.8908	0.0328	2.1317	
0.0049	0.0658	0.031	0.0263	0.2218	0.8977	0.0845	0.2136	
0.1954	0.1721	0.0475	0.4751	0.0151	0.031	0.057	0.1368	
0.0923	0.2297	0.0436	0.0169	0.0644	0.0567	2.1472	0.0697	0
	0.0381	0	0.0635	0.0377	0.0046	0.044	0.0266	
0.013	0.0413	1.0732	1.9605	0.0956	3.5482	4.5692	0.1376	0
	0.3421	0.8172	0.0365	0.0011	0.0743	0.0542	0.0465	
0.0708	2.1006	0	0.0458	0.0345	0.0727	0.0626	0.0959	
0.0385	0.0492	1.8753	0	4.9052	1.7654	0.0645	0.0696	
0.0299	0.173	0.026	0.0311	0	0.0421	0.2303	0.1743	
0.0565	0.2436	0.5976	0.0421	0.0356	0.5584	0.0731	0.1345	
0.0588	0.0341							
BTBD16	0.1347	0	0	0	0.1426	0.7953	0.0411	
0.0646	0.1444	0.0487	0.151	0.0141	0	0.4952	0.9178	
0.1474	0.053	0.0433	0.0685	0.3874	0.2169	0.863	0.0865	
0.74	0.0727	0	0.4437	0	0.8522	0	0.8746	
0.1871	0.2569	0.1297	0.5075	0.0408	0	0	0.1027	
0.5431	0.0984	0.0446	0.3338	0.0872	0.0189	0	0	
0.0344	1.3271	0	0.0489	0.1545	0.4712	0	0	
0.137	0	0.1483	0.261	0.0166	0.2507	0.2866	0	
0.379	0	0.0532	0.0926	0.0073	0	0.53	0.9715	
0.3837	0.0854	0.382	0.7843	0	0.1964	0.297	0	
0.0965	0.1069	0.4843	0	0.4823	0.0645	0.908	0	0
	0							
BTBD17	0	0	0	0	0	0	0	0
	0	0	0	0	0	0	0	0
	0	0	0	0	0	0	0	0
	0	0	0	0	0	0	0	
0.0347	0	0	0	0	0	0	0	0
	0	0	0	0	0	0	0	0
	0	0	0	0	0	0	0.0618	0
	0	0	0	0	0	0	0	0
	0	0	0	0	0	0	0	0
	0	0	0	0	0.0152	0	0	0
	0	0	0	0	0	0	0	0
	0							
BTBD18	0	0.0138	0.0855	0.0754	0.0257	0.0746	0.0043	
0.1169	0.1085	0.0978	0.049	0.0195	0.0045	0	0.0208	
0.0123	0.2314	0.0708	0	0.0471	0.0405	0.015	0.0187	
0.0238	0.0185	0.0473	0.0387	0.0288	0.0714	0.0462	0.0149	
0.0064	0.0131	0.0227	0.0308	0.0151	0.0016	0.0188	0.0053	

0.0977	0.0295	0.0028	0	0.0692	0.0565	0	0.0499	
0.057	0.0425	0.0701	0.032	0.013	0.0521	0.0254	0.07	0
	0.0776	0.0255	0.0856	0.0231	0	0.0118	0.0155	
0.1371	0.0514	0	0	0.05	0.0434	0.1583	0.0139	
0.0161	0.0628	0.0063	0.0326	0.0223	0.0668	0.0389	0.051	
0.039	0.0145	0.0109	0.0295	0.0234	0	0	0	
0.0544	0.0612							
BTBD19	2.0133	1.9536	1.4845	1.3937	2.3529	2.6958	2.7172	
2.4647	2.6418	1.5682	1.8325	2.1412	3.0147	2.248	1.1573	
2.3218	3.8268	1.5912	1.5563	2.1734	3.0457	0.6773	1.7712	
1.2847	1.8445	3.5277	2.8944	0.0706	2.1648	0.1477	0	
2.6996	3.0188	1.5735	3.3506	0.795	2.0333	2.9467	1.8463	
2.3364	2.1553	3.6274	1.0663	2.0077	3.2778	1.6627	1.1499	
2.9603	3.6327	1.601	1.3909	1.4409	2.8891	1.6488	0	
0.6345	2.7835	0.6171	2.1784	1.0895	2.2367	2.708	1.2407	
0.5494	1.1287	3.4355	0.6795	2.3107	0.5174	2.3009	2.8049	
3.0512	1.1529	2.842	1.2663	1.9429	2.0276	1.9873	3.0645	
1.998	2.5697	3.1017	4.1723	2.4347	2.3586	2.8122	2.6542	
2.169	2.2067							
BTBD2	4.6246	4.0575	5.3123	2.4343	4.3228	3.0221	3.2271	
4.2611	3.579	3.7315	5.5236	3.0846	4.7452	2.9151	4.2311	
3.3577	4.3278	3.0602	3.1415	4.411	4.7711	5.0347	4.8037	
3.7774	3.5673	3.7149	4.6665	3.9662	4.4939	4.6575	5.2621	
4.3044	4.1317	5.0422	3.8137	2.6966	4.5107	5.5677	5.3965	
3.3605	5.3622	4.4697	4.653	3.5284	4.0029	3.6679	3.0972	
3.8952	5.4835	2.786	3.7332	3.1228	3.0194	4.0525	4.7101	
4.2406	4.9078	5.8445	3.5459	5.5531	4.702	4.2688	3.6208	
3.5682	5.3836	4.7083	4.6751	5.2284	3.3801	4.5923	5.204	
4.2675	4.1672	3.9007	3.5138	2.0875	3.9856	4.0237	4.2142	
4.7187	4.3826	4.7341	4.1935	4.954	4.8763	4.1549	4.672	
4.7813	4.9124							
BTBD3	3.5912	4.6324	4.2597	5.0824	4.6604	5.3769	4.5283	
5.097	4.8158	3.3517	4.3863	4.9547	4.3089	4.4038	5.2466	
4.5942	3.5416	3.7687	5.2385	4.3831	4.2397	5.504	5.3019	
4.4679	6.7013	4.9844	5.333	5.2044	4.554	5.3655	3.3453	
4.7362	5.4988	4.7032	5.2818	3.8047	3.7979	5.4022	5.3188	
5.6102	4.7641	4.0976	5.0109	6.0336	4.825	3.6462	5.0862	
5.0445	5.0055	4.2146	4.7955	5.481	5.3601	4.6009	3.4225	
5.4438	3.3853	4.8836	5.2383	5.3353	4.201	4.3297	4.886	
5.2636	5.0949	2.8895	4.4346	3.7297	4.7947	4.4851	4.2422	
4.3916	5.1678	5.5285	6.112	4.2547	4.788	5.4393	4.7525	
4.5887	4.7975	4.5646	4.6393	4.9918	4.9846	5.1618	4.316	
4.4124	4.8643							
BTBD6	4.6844	4.4845	4.2912	3.8748	4	3.7269	4.2465	
4.3573	3.7578	3.6936	4.2797	3.4975	4.3863	3.8133	3.8646	
4.5104	3.2991	3.6802	4.7928	3.1856	4.5818	4.3096	4.3058	
4.0552	3.657	4.5563	1.6772	4.1422	4.8808	4.1725	0	
1.202	4.7055	3.8923	3.741	3.4662	3.9742	4.3604	4.2179	
3.6705	3.7875	3.5553	4.6453	4.3258	4.9285	3.968	3.6404	
4.5849	4.8332	3.7196	3.8927	4.2969	3.903	3.6953	0	

4.7412	3.1651	4.1909	3.3937	4.0628	4.3916	4.7985	3.1218	
4.1937	3.8552	4.2434	2.9183	4.1762	4.1567	4.1579	4.3711	4.5
	4.22	3.6197	4.4354	2.6059	4.4472	2.0631	4.3776	
4.1345	4.5563	3.4953	5.1513	3.8927	4.7036	4.3547	4.9332	
4.5978	5.1738							
BTBD7	2.4252	2.1804	2.5775	2.9862	2.3772	3.0006	2.1697	
2.5103	2.3316	2.2428	1.7083	2.4766	1.9082	3.0694	2.4058	
2.5006	2.9007	2.9026	2.9384	1.7214	2.6842	2.2657	2.6159	
2.5334	2.5195	2.4943	1.9377	2.6602	2.6445	2.0452	2.8812	
2.2759	2.3426	2.0452	2.2135	2.5391	2.258	2.5815	2.0484	
2.8919	2.1412	2.5083	2.3013	2.6772	3.2527	3.3477	2.8942	
2.8647	2.5242	3.8628	2.1554	3.2253	2.6944	2.3326	2.0959	
2.5518	3.497	2.6955	2.4002	3.0588	2.2153	2.5666	2.922	
2.1887	2.6351	2.4669	3.1091	2.9139	2.8105	2.5176	2.4049	
2.3387	2.4722	1.7461	3.0118	1.2434	2.4416	2.4986	2.735	
2.1193	2.343	2.6391	2.7823	2.3629	2.3488	2.4049	2.7364	
2.6731	2.8045							
BTBD8	0.9233	0.535	0.7371	0.7744	0.177	0.6381	0.7361	
0.4551	0.2667	0.66	0.9175	0.4399	0.6956	0.6916	0.1358	
0.7003	1.1603	0.7906	0.7229	0.5273	0.1175	0.3723	0.3028	
0.3931	0.1311	0.752	0.2411	0.8224	0.1024	0.4641	0.9001	
0.4021	0.4798	1.2194	0.1873	0.7614	0.6257	0.3834	0.0869	
0.6309	1.0325	0.6501	0.6124	0.4999	0.8175	0.0292	0.3711	
0.3091	0.4655	1.5555	0.4664	0.3168	0.3236	0.1851	0.6514	
0.1297	1.0021	0.4793	0.8257	0.6554	0.736	0.1548	0.2996	
0.7679	0.5199	0.6106	0.8545	0.361	0.3254	0.4625	0.5209	
0.6881	0	0.43	0.2671	0.5712	0.7691	0.1505	0.896	
0.9132	1.0729	0.1391	0.2954	0.4388	0.3447	0.1792	0.6462	
0.5485	0.6723							
BTBD9	2.307	1.978	1.8412	1.8406	1.4289	1.6971	1.4701	
1.6324	1.6677	2.367	0.9306	1.9088	1.2506	2.3531	1.7953	
1.4677	1.8518	1.9108	1.9898	1.5532	1.6514	1.1206	1.1626	
1.4522	1.1041	2.3461	1.155	1.7306	1.4767	1.1491	1.744	
1.5299	1.4479	1.4438	1.8876	2.1443	2.4598	3.02	1.7569	
1.4266	1.4165	1.8593	1.5023	1.0925	1.7056	2.4133	2.0485	
1.3602	0.6932	1.7475	1.5234	2.1021	1.7435	2.3598	1.6675	
1.2658	1.735	1.5978	1.7612	1.5481	1.5084	1.5985	1.5168	
1.6097	2.2323	1.2025	1.6938	2.477	1.8945	1.0086	1.534	
1.2961	1.5041	1.3197	1.2134	2.0458	2.1095	2.6423	1.5155	
1.6597	1.1647	2.0025	2.7303	1.248	1.5127	1.4385	2.4693	
1.7623	1.9492							
BTC	0	0.1986	0.056	1.0806	0.7234	0.1103	0.7329	
0.0069	0	0	0.0742	0.4293	0.59	0	0.4581	
0.0048	0.157	0	0	0.834	0.3106	0.0487	0.3772	0
	0.1979	0.0053	0.1989	0	1.2759	0	0	0
	0.0251	0	1.1063	0.0032	0.4938	0.8473	0.1191	
0.3028	0	1.1908	0.169	0.1173	0.3131	0.8032	0.0296	
0.2212	0.0929	0	0.0136	0.018	0.1144	0	0	0
	0.7399	0.5694	0.2447	0.1611	0.1678	0.5407	0.4536	
0.0942	0.2469	2.2577	0.1726	0.1865	0	0.4433	0	

0.076	0	0.1075	0	0.1648	0.0928	0.1132	0.3322	0
	0	0.0614	0	0.2428	0.0851	0.1096	0.0334	0
	0							
BTD	3.5177	3.0889	2.8268	4.8134	3.9047	3.7123	3.9892	
1.9613	3.5537	3.4386	4.1659	5.1526	3.3726	4.3741	4.3455	
4.446	2.7718	4.6189	3.6856	4.6541	3.2784	3.3989	4.8636	
3.1696	2.1349	3.3303	3.5109	3.287	4.6909	3.5592	3.4918	
4.0724	3.9118	1.1833	3.4098	3.5987	3.4242	3.5987	3.776	
3.0241	4.9479	3.2938	3.4322	4.5693	4.9775	3.7575	3.9985	
2.8821	3.6309	4.4158	4.0568	3.1587	4.3331	4.4107	2.7425	
4.6613	2.9502	3.1475	3.9502	2.76	3.386	3.5494	2.1959	
3.3589	2.7405	4.296	3.8401	3.7873	3.4539	3.9191	4.0736	
3.8772	4.0731	4.3353	4.436	2.4539	3.1126	4.7249	3.1036	
2.7615	3.3567	4.7377	5.5415	3.7596	3.441	3.9129	5.0073	
4.1047	4.1904							
BTF3	8.3954	8.3676	8.6164	8.9027	9.2274	8.374	7.5565	
8.7071	7.8193	8.0812	8.6075	9.0115	9.1378	8.665	8.3954	
8.13	9.417	7.6045	8.8788	9.0875	8.5071	8.6471	8.1656	
8.9637	8.7762	7.8854	9.5834	8.1987	8.7664	8.6873	8.2999	
8.9267	8.6361	7.4029	8.2404	8.4032	8.8788	8.3533	8.3291	
8.8544	8.726	8.6873	8.1847	8.8665	8.4889	8.8423	8.6075	
8.7071	8.6973	9.5078	8.279	8.7458	8.5071	9.1219	8.4325	
8.1957	9.006	8.2164	8.1817	8.0498	8.8922	9.0908	8.9341	
8.4325	8.4132	8.145	9.0206	7.9023	8.4325	8.9053	8.1517	
8.594	8.636	8.9053	7.9979	8.3724	8.6847	7.9146	8.9617	
9.4098	8.6847	8.8026	8.5495	7.9146	9.3051	8.0498	8.2046	
8.2644	8.4296							
BTF3L4	3.3465	4.0114	3.6534	4.1051	4.2595	3.8735	3.7936	
3.0523	4.1531	3.9608	3.2835	4.2545	4.9982	4.4747	3.7229	
3.549	4.2883	3.7069	4.1804	3.7291	3.7202	3.4571	3.5678	
3.8772	3.4091	4.4354	3.7703	3.6763	3.9346	4.1681	4.319	
4.2095	4.2142	3.5509	2.6868	3.8701	4.4884	3.7895	4.205	
4.2478	3.8449	3.8823	3.3103	4.0155	4.8024	4.057	3.4388	
4.18	3.6248	4.7157	3.5635	3.9305	4.0043	5.0234	3.8707	
3.5432	4.1444	3.5423	3.8289	3.9397	3.739	4.0357	3.6047	
4.0199	3.7571	4.16	3.9945	3.7361	3.6283	3.6358	3.3342	
4.0099	3.7867	4.1498	3.7771	3.1186	4.1037	3.4791	4.0807	
3.8365	3.7116	3.696	3.3177	1.9385	4.1094	3.8091	3.3951	
3.5527	3.462							
BTG1	4.9347	4.0562	3.7377	4.4527	5.478	4.4239	5.7086	
5.0321	3.4661	4.7785	5.169	5.297	5.7096	3.7727	4.8073	
5.1211	5.7862	4.4546	4.3009	4.5121	4.5592	5.0446	3.9695	
3.8125	4.6935	4.5767	6.08	4.4532	3.7677	4.6101	1.8826	
4.2881	3.7536	3.9719	3.4624	3.8151	7.1773	3.1537	5.3342	
4.8372	4.1191	5.7903	4.3138	5.3051	3.1906	4.5415	3.9958	
5.3074	5.3245	4.5157	4.1294	5.2478	5.1697	6.6397	1.7377	
4.842	4.9831	3.9032	4.5218	3.7604	5.1108	4.7797	4.208	
4.6038	3.7523	5.5431	3.7421	3.4476	4.665	5.2819	5.4659	
4.109	4.3074	5.116	4.2862	3.441	3.7787	3.7465	4.4858	
4.8389	4.7834	6.006	5.4574	4.4159	4.3534	4.443	5.2774	

4.0913	4.1397							
BTG2	2.0694	1.1647	2.8926	1.0622	3.7863	4.2276	3.6322	
0.7609	1.7715	1.4017	2.903	2.4534	0.3902	0.9901	1.9282	
3.3243	1.7878	3.1085	1.3153	2.0167	0.5066	1.6276	3.7389	
1.4082	2.8217	2.0029	4.1522	1.887	3.6434	2.6401	1.7983	
0.6713	1.4835	1.3237	2.7773	1.5217	1.0622	2.1832	1.9802	
3.1701	3.521	3.4653	3.0523	2.8591	2.9789	2.2519	2.957	
2.4386	1.0037	0.0946	0.7233	2.9425	0.8825	0.8794	2.0083	
1.3801	3.6183	2.7987	1.7938	2.0691	3.2444	1.4455	1.9644	
0.9048	3.0998	5.0983	3.7433	3.02	2.8892	2.6531	1.8887	
1.2095	1.198	1.7	3.2002	1.4898	1.2004	3.736	2.4496	
1.9496	1.2903	2.8117	4.1523	1.4933	0.9292	0.944	2.5637	
2.9968	1.7835							
BTG3	6.0783	5.765	5.8208	5.2348	4.3746	3.9191	6.6216	
5.3664	5.7979	5.1433	5.5925	5.0786	4.5069	6.4264	5.7438	
5.3476	5.1747	5.8864	5.9724	5.766	5.6805	4.9235	4.9215	
5.5185	5.281	4.778	6.6699	5.9984	5.3785	5.5391	5.18	
5.478	5.3872	4.8827	5.4275	5.8504	5.5834	5.2363	4.0104	
5.4742	4.8206	4.9506	5.5477	5.8826	4.4757	5.1093	6.1374	
4.9209	4.3033	5.369	5.0881	5.2817	5.7505	4.0073	5.1889	
5.4818	4.7388	4.2061	5.9633	4.221	4.9081	4.6398	4.5819	
5.4916	5.4091	5.8676	5.6185	5.0217	6.2065	5.4756	4.9474	
5.3839	5.6045	4.0677	5.672	6.0158	5.6986	4.7839	5.4635	
6.0669	4.8353	4.9502	4.6979	5.736	5.1947	6.2346	5.3751	
5.9908	6.0951							
BTG4	0	0.0434	0.1712	0.0949	0.0117	0	0	0
	0	0	0.0811	0.1393	0.4863	0.3911	0	
0.0232	0.3149	0	0.0817	0	0	0.0512	0	0
	0.0238	0.09	0.0408	0	0.1947	0.0803	0.1025	
0.3621	0.1123	0.3246	0	0	0.0182	0	0.029	0
	0.0991	0.0791	0	0.1287	0	0.2115	0.1209	
0.0596	0	1.1181	0.2052	0.0862	0	0	0	0
	0	0	0	0	0.6017	0	0	0
	0	0.0562	0	0.33	0	0	0	0
	0	0.3481	0.2339	0.1631	0	0.2638	0.1036	0
	0	0.0838	0	0	0	0	0.1806	
0.0731	0							
BTK	0.4589	2.1456	0.8601	0.0527	0	0	0.6275	
0.1626	0.4707	0.4765	0.14	0.1005	0.1056	0.1592	0.0631	
0.3162	0	0.444	0.7961	0.1753	0	0.0126	0.0876	
0.9567	0	0	0.3268	1.3346	0.0582	1.4386	0.0698	
2.4963	0.5815	0.3543	0.6305	0.1681	0.1626	1.2733	0.3147	
0.2398	0.9228	0	0.0264	0.0689	0.0065	0.0677	1.3762	
0.5363	0.1345	0.358	0.7009	0.0647	0.1461	0.1529	0.2618	
0.7939	0.1955	0.1549	0.1915	0.199	0.8766	0.4129	0.6302	0
	0.658	0.0359	0.3829	0.2148	2.029	0	0.0477	
0.2876	0.2032	0.0462	0.0738	0.4274	0.8114	1.8449	0.4649	
0.3283	0.3614	0	0.5892	0.2787	0.0977	0	0.4943	
0.4692	0.5084							
BTLA	0	0.0293	0.0504	0.0447	0.0101	0.0235	0.2314	

0.0433	0.0175	0.0117	0.0233	0.0155	0.0559	0	0	
0.0156	0.2345	0	0	0.0413	0.0285	0	0	0
	0.0057	0.0158	0	0.3337	0.0218	0.0486	0.0088	
0.0064	0.0163	0	0.0314	0.015	0	0.0219	0.0246	
0.0584	0.0228	0.0032	0.0222	0.0152	0.0196	0	0.0753	
0.033	0	0.1289	0.0307	0.0239	0.0308	0	0	
0.0549	0.0283	0.0218	0	0	0.0565	0.03	0	
0.0283	0	0.016	0.1294	0.003	0.0516	0	0	
0.0114	0	0	0.075	0	0.0948	0.2071	0	
0.1042	0	0.0211	0.0273	0.0911	0	0.0689	0.0217	
0.0259	0							
BTN1A1	0	0	0	0.0648	0.0165	0	0	0
	0	0.0371	0.0029	0	0	0	0.025	0
	0	0	0.0427	0	0	0	0	0
	0	0	0.0153	0	0.0125	0.098	0.0191	
0.0185	0	0.0424	0.0116	0	0.0232	0.0713	0.0485	
0.0113	0	0.0051	0	0.1218	0.0054	0.0524	0.0227	0
	0	0	0	0.0678	0	0.013	0.0327	0
	0.0321	0	0	0	0	0	0	
0.071	0	0	0	0.0053	0	0	0	0
	0	0	0.0591	0.0783	0.0861	0	0	0
	0	0	0	0	0	0	0.0207	0
	0							
BTN2A1	2.8895	3.2732	3.1727	3.8041	2.5958	3.4411	3.8282	
3.6323	3.3395	3.5789	3.008	3.9948	3.3697	3.3616	3.7856	
3.8491	3.3204	3.6591	3.0996	3.4678	3.3678	4.793	3.4414	
3.8487	3.7987	4.8905	3.6865	3.5589	3.7866	2.218	2.7778	
2.4085	3.6693	3.1944	4.1036	3.4858	3.5227	3.592	3.1219	
3.1844	2.7684	3.4858	2.8312	2.9652	3.7759	4.8146	2.6353	
3.4624	3.6522	3.3402	3.4532	3.5503	3.2903	3.5578	3.5665	
3.1211	3.1233	3.7694	2.8216	3.6155	2.9866	3.8239	3.135	
3.5344	3.1911	2.577	3.6715	3.7886	3.5127	3.34	3.1701	
4.2205	3.4427	3.7338	3.4619	3.2019	3.8583	3.5563	3.0041	
2.9599	2.5863	3.6174	4.5439	2.7145	4.5666	3.2591	3.9363	
3.2449	3.2617							
BTN2A2	3.7209	3.3376	2.2516	3.204	2.4601	2.9806	2.9224	
2.5686	3.5698	4.0269	3.6939	4.3213	1.8216	3.8636	2.5748	
3.0799	2.5681	3.8879	2.7466	3.0369	3.0542	4.0559	3.1187	
3.6764	2.2631	3.1437	4.3995	3.354	2.8686	2.7276	2.0066	
2.5605	3.9838	4.3724	3.6496	3.8895	3.4154	3.8029	2.7494	
3.0336	3.4697	2.8883	2.582	4.4005	2.7665	3.3353	3.3717	
2.731	2.7163	3.0276	3.4834	3.1184	4.0925	3.0241	2.2301	
3.4316	3.795	2.979	2.7715	2.429	2.6345	3.3885	2.832	
3.6046	3.6784	2.0788	3.7064	3.0891	2.8091	3.354	3.8159	
3.843	3.0041	3.8916	2.8481	3.5239	3.838	3.7994	3.7356	
3.9269	3.589	4.0084	3.7897	2.6369	2.6857	3.756	4.1607	
3.8753	3.65							
BTN3A1	2.1396	2.4204	2.1737	2.7391	1.9101	2.3849	3.7043	
2.5143	3.318	2.418	3.743	3.6259	2.0198	2.8308	2.2874	
2.9281	2.5713	3.3676	2.3151	2.0022	1.8119	3.8738	2.5323	

2.8836	1.5838	4.3162	3.5585	2.7284	2.0959	2.0104	2.3024	
2.5538	2.9873	3.9624	3.3876	2.7474	2.6009	3.2512	2.0009	
2.5138	3.0889	3.3726	2.7076	2.6009	2.5583	4.1046	2.1062	
2.5567	2.7256	2.7421	3.5062	2.9979	3.2929	1.9769	2.3294	
1.8217	3.1275	2.2091	1.8773	2.0447	2.6063	3.1012	2.1552	
2.9352	2.99	3.3466	3.5912	2.9226	1.4792	2.0048	2.8286	
2.9433	2.4769	3.5205	2.8251	2.8694	2.9599	3.6084	2.6245	
2.9356	2.569	3.7304	2.8402	3.0501	1.9956	2.9362	2.8121	
2.5669	2.4992							
BTN3A2	2.9806	3.4741	2.3122	3.5584	2.4114	3.4052	2.0869	
3.0351	4.545	3.2252	4.3285	4.5249	2.9948	3.9771	2.0647	
3.6288	3.1246	4.6863	3.2564	1.0646	2.6191	4.7819	4.1698	
3.4329	2.8506	4.0802	4.179	3.4948	2.6967	2.5837	2.2619	
3.2955	3.6912	4.9443	4.2413	3.6909	2.6872	3.6507	2.4993	
3.5353	4.446	4.0103	3.4756	4.1963	2.6796	4.6719	2.7812	
3.8822	3.4226	3.3933	4.3632	3.8237	4.2113	2.6621	2.5345	
1.0338	4.2072	3.4321	2.9777	3.0111	3.106	2.6683	2.8707	
3.7195	3.8071	3.1408	4.155	3.5449	2.1139	3.3167	4.0883	
4.0561	3.343	4.4313	3.9147	3.6391	3.4847	4.8699	4.4592	
4.0435	3.6098	4.0862	2.7904	1.8218	3.714	3.8402	3.6496	
3.5584	3.8966							
BTN3A3	1.8844	1.8397	1.2838	2.4	1.6258	1.6864	3.7529	
1.8566	2.6849	0.829	2.316	3.2019	1.7192	2.327	1.8884	
1.9772	1.9836	2.6267	1.8386	1.7143	1.1566	3.4442	2.7079	
1.9143	1.2875	3.6813	2.8219	2.0001	2.1651	1.0896	0.4437	
1.9229	2.567	3.321	3.6432	1.4403	2.2761	3.0971	1.0629	
2.024	2.9891	3.1983	1.956	2.0296	2.0478	3.6659	1.5492	
1.9001	2.536	2.3133	3.2962	2.8028	2.2812	1.5398	1.118	
0.7781	2.257	1.6139	1.1451	1.2157	2.0294	1.7752	1.8728	
2.2615	2.9601	3.4465	3.0209	2.6282	1.3506	2.5047	2.7874	
1.9617	2.5164	2.9844	1.9886	1.0628	2.0251	3.708	2.4237	
1.9955	1.6929	3.5574	2.3928	2.8613	1.7556	2.1325	2.7733	
2.1939	2.4235							
BTNL2	0	0	0	0	0	0	0.2026	0
	0	0	0	0	0	0	0	0
	0	0	0.1504	0	0	0	0	0
	0	0	0.065	0	0	0	0.0475	0
	0	0	0	0	0.0354	0	0	0
	0	0	0	0.0714	0	0	0	0
	0	0.1794	0	0	0	0	0	0
	0	0	0	0	0	0	0	
0.1725	0	0	0	0	0	0	0	0
	0	0.0396	0	0	0	0	0	0
	0	0.0867	0	0	0	0	0	0
	0							
BTNL3	0	0	0	0	0	0	0	
0.0353	0	0	0	0	0	0	0	0
	0	0	0	0	0	0	0	0
	0	0	0	0	0	0	0	0
	0	0	0	0	0	0	0	0

	0	0.01	0	0	0	0	0	0
	0	0	0	0	0	0	0	0
	0.0543	0	0	0	0	0	0	0
	0	0	0	0.066	0	0	0	0
	0	4.00E-04	0	0	0	0	0	0
	0	0	0.1293	0	0	0	0	0
	0							
BTNL8	0	0.1784	0.0415	0	0.0188	0	0	0
	0	0.026	0.0129	0.0271	0	0	0.182	0
	0.052	0.0435	0.0722	0	0.1277	0.0109	0	
0.1093	0	0	0	0	0	0	0	
0.9663	0.0335	0	0	0	0.0127	0.0386	0	0
	0	0.0292	0	0	0	0	0	0
	0.0244	0	0	0.1056	0	0	0	0
	0	0	0.3565	0	0	0.2831	0	
0.0418	0	0	0	0.1374	0	0	0	
0.1374	0	0	0	0	0	0	0	0
	0.0229	0	0.656	0	0	0	0	0
	0							
BTNL9	0	0.1227	0.897	0	0	0	0.7338	0
	0.073	0	0	0.0672	0.2168	0	0	0
	0	0	0.0634	0.047	0	0	0.0196	
0.1523	0	0	0.0827	0	0	0	2.1982	0
	0.2215	0	0.0176	0.3817	0	1.2486	0.0683	
0.0295	0	0.0303	0.3055	0	0.0233	0.0611	0	0
	0	0	0	0	0	0.4415	1.1907	
0.228	0.0283	0	0	0	0	0	0.1029	0
	1.1337	0	0.1311	0.1495	0.0498	0.0087	0	0
	1.2282	0	0	0	0	0.7497	0	
0.0342	0	0	0	0.0033	0.0142	0	0	
0.0693	0							
BTRC	2.621	2.8564	2.6395	3.1602	2.5353	3.268	2.638	
3.3314	2.6992	2.4392	2.3194	2.8057	2.0045	2.8206	2.6114	
2.5039	2.8569	3.075	2.4691	2.9907	2.5454	1.6013	2.471	
2.2723	2.0273	2.6762	2.2335	2.9663	1.8679	2.4794	4.0811	
2.3843	2.1589	2.5902	1.8624	3.5016	3.1832	2.4476	2.3427	
2.6068	2.3649	1.7788	2.0962	1.8752	3.1238	3.4428	2.6746	
2.1442	2.2745	3.6394	2.38	2.5978	2.8992	2.8746	4.0368	
2.6687	2.442	2.5635	2.2177	2.2997	2.8951	3.4037	3.1934	
2.3727	2.9086	2.7685	2.6506	3.1698	2.0854	2.2055	2.1489	
2.0076	1.7855	2.8561	2.5965	2.5444	3.3687	3.0259	1.9118	
2.751	2.4191	1.8595	3.4863	3.2625	1.5737	2.2781	3.0397	
2.3517	2.6653							
BUB1	4.6433	5.0811	5.0339	5.0368	4.0664	4.4085	3.6179	
4.2203	5.404	5.3188	3.9206	5.0581	4.6404	5.0762	5.1501	
3.6879	4.3997	5.201	5.3535	4.327	5.0762	4.4231	4.2568	
4.8389	4.4975	3.0056	3.1657	4.5934	5.0234	5.7753	4.9213	
4.1766	5.5542	5.175	4.4733	5.6641	4.2943	2.6549	3.3063	
5.1508	2.9446	3.3157	5.7436	4.2494	4.8688	5.8586	5.1015	
4.7586	3.5695	4.5002	4.4474	4.1509	4.7497	3.3829	4.9663	

4.693	4.3697	5.0302	4.6542	5.0637	4.4848	5.1604	4.4207
4.8626	5.6331	2.0893	4.8573	2.2024	4.6032	4.9808	3.8334
4.878	5.0275	4.7741	4.2529	4.36	5.0094	3.9548	4.8587
5.0308	5.3741	2.7279	2.7987	3.5806	4.9486	4.9605	3.3998
4.6234	4.344						
BUB1B	3.8492	4.8188	4.1229	4.9403	4.5544	3.8948	2.4346
4.0548	5.1191	4.4206	3.6426	4.2673	4.4	4.9463	4.3155
3.6284	3.9042	4.6638	4.7542	3.9896	5.0637	4.5534	3.8308
4.3151	4.1159	2.7255	2.7383	4.0111	4.8092	4.6911	4.696
4.1769	4.3638	3.741	4.3146	4.9288	3.4982	1.6002	3.8172
4.7255	2.1372	3.3301	4.3977	4.3652	5.1612	4.87	5.2262
4.4079	2.875	4.999	3.2191	3.9469	4.0915	3.3677	4.7846
4.7713	3.8237	4.7249	4.2128	4.749	4.1305	4.3934	4.6586
4.7111	4.6147	1.3075	4.4929	1.8365	4.4824	3.9673	3.4759
4.458	4.1068	4.4661	4.5563	3.8445	3.7664	3.8685	4.3782
4.0403	4.8006	2.866	2.213	3.8055	4.6747	4.3748	2.9602
4.2297	3.6773						
BUB3	5.666	6.2205	6.3036	6.5456	6.482	6.2608	5.25
6.1049	6.084	5.8667	5.2261	6.5216	6.5529	6.3918	5.6942
5.0898	6.6529	5.5178	5.9561	6.278	6.2749	5.399	5.3266
6.1061	5.598	5.2847	5.7657	5.8502	5.7703	5.2557	6.7147
6.2866	6.1388	5.1753	5.0169	6.5635	5.984	4.5492	5.4879
5.6652	5.5754	5.9217	6.3387	5.6593	5.5136	5.7504	5.7959
6.3323	6.2067	6.998	5.6321	5.6253	5.9875	5.6102	6.8301
6.052	5.8776	6.7467	5.5726	6.1252	6.0431	5.3519	6.042
6.5474	6.0611	5.6282	5.9458	5.7638	5.4295	6.0486	5.4603
5.8333	5.4295	5.7411	5.5367	5.0543	6.4659	5.4564	5.3078
4.5988	5.9314	5.9478	5.136	5.1727	6.0385	6.2281	5.5267
5.9596	5.6848						
BUD13	3.172	3.2332	3.1749	3.5388	3.5251	2.6724	2.8791
2.3834	3.3231	2.5207	2.4438	2.7193	3.1326	3.3719	2.3837
2.7223	3.5626	2.8298	3.3249	3.0884	2.4261	3.4412	2.8162
2.5919	2.7543	2.567	3.6602	2.6912	3.1929	2.9752	2.9439
2.238	2.7086	2.4844	2.4349	2.9641	2.5944	2.4256	2.7162
2.877	1.7357	2.6211	3.1356	4.0297	3.5622	2.9099	2.9177
3.2301	2.94	3.651	2.8791	3.5469	2.139	2.7181	3.2094
3.8099	2.9348	3.3127	2.7426	3.2135	3.0217	2.5955	2.9532
3.185	2.3029	2.3754	3.0909	2.5965	2.766	3.417	2.2093
3.1302	3.2699	2.1558	3.2968	2.8686	2.7078	2.1428	3.0567
2.9324	2.8263	2.721	2.0587	2.7336	3.656	2.4495	2.786
3.0437	3.0584						
BUD31	5.5842	6.3413	4.5437	6.3855	5.6245	6.554	5.8625
6.2373	5.2602	5.4658	5.4037	4.9539	6.1754	6.2385	5.5937
5.2167	5.2721	6.2287	6.2467	5.1078	4.7961	6.3906	5.5719
5.9286	6.1074	5.7768	6.0372	4.8092	6.105	5.4666	4.9221
5.5953	4.8253	4.3208	5.8471	5.6298	6.2314	5.1122	6.556
5.7131	5.7241	5.2222	5.3681	5.1962	5.0063	6.3116	5.5314
5.5537	4.8664	5.1284	5.326	6.4691	5.0349	6.0065	5.954
5.7272	4.2053	5.6037	5.6487	5.8435	5.6852	5.9821	6.0053
5.7709	5.2933	5.4028	6.1845	5.4042	4.7445	6.1997	5.4765

5.6265	6.1033	4.8227	5.4356	5.0562	5.0549	6.0873	4.7463
5.534	5.5433	5.0401	3.7031	5.4563	6.4411	5.2761	4.917
5.0147	4.869						
BVES	3.0804	2.0856	2.3455	2.9429	1.4786	1.807	0.1392
2.655	2.9357	2.6219	2.8463	2.0797	2.3775	2.924	4.2137
2.8223	2.0339	3.005	3.1161	3.244	2.5242	2.2748	3.8552
2.3925	3.7273	2.6792	3.6584	1.8272	3.5543	2.3317	2.4434
2.3478	2.6333	2.9699	3.4667	2.8489	1.606	1.449	0.8241
2.7794	2.6542	2.1489	3.2131	2.4973	2.439	2.1498	2.5393
2.1304	4.0383	2.4278	3.3123	3.8509	2.4128	2.8357	3.2594
2.3951	1.9065	2.4059	2.6526	2.535	2.7467	2.7616	1.8915
2.3133	1.2495	2.3881	2.1612	1.8296	2.6138	2.7092	3.3871
3.8455	2.0504	1.8186	3.6786	1.504	2.7332	2.5404	1.957
2.1421	2.8205	4.2581	2.6985	3.4748	3.0446	2.3393	3.82
2.6854	3.4514						
BYSL	4.1466	4.5456	4.4871	4.2556	4.2463	4.3057	3.7606
4.6804	3.6439	4.7288	3.9337	4.7288	3.7258	4.3151	4.4909
3.5646	4.8482	4.1236	4.5482	5.0492	4.196	4.5847	3.992
4.4861	4.3475	4.5814	4.1487	4.5089	4.597	3.6081	3.1937
4.4027	4.1245	3.0635	3.761	4.4366	5.5494	4.5782	4.6179
4.1043	3.9251	3.4615	3.8656	4.0957	3.6815	4.2046	4.103
3.6659	3.4033	3.6625	3.9083	4.292	4.114	4.7773	4.5829
4.4817	3.8202	4.8694	3.4293	4.802	4.3567	4.2723	4.4719
3.7545	4.6071	2.3186	4.6042	3.9184	3.8233	3.7375	4.4754
4.0621	4.1882	4.1096	3.5777	4.071	4.6873	4.2433	3.9266
3.6715	3.8642	4.0071	4.6052	3.6719	4.6593	4.147	3.2666
3.9412	3.4367						
BZW1	6.7191	6.579	6.9843	7.6166	6.598	7.3209	6.558
7.2006	6.5108	6.865	6.5293	7.0143	6.634	7.259	6.8848
6.4323	7.5582	7.2364	7.0471	6.5395	6.8758	6.2118	6.9976
7.0445	7.5018	7.2852	5.6044	6.7659	6.7552	7.0143	6.565
6.7784	6.8249	5.6945	6.681	7.3276	6.9601	6.3392	6.3935
7.2462	6.1227	7.4815	7.4003	6.598	7.4696	7.9411	7.3629
7.0046	6.7638	7.3843	6.5224	7.1184	7.0116	6.992	6.7798
5.7689	7.7005	7.4783	7.2691	7.606	6.9132	7.0357	6.1178
6.2518	7.1809	6.5617	6.1469	6.3954	7.1662	7.3787	7.2382
6.5123	6.8758	6.4121	6.4884	6.5504	6.753	6.5568	6.7139
6.291	6.7157	6.6191	6.9175	6.0464	7.1251	6.746	6.4039
6.6212	6.252						
BZW2	6.3185	5.92	6.2379	6.9456	6.2586	6.2349	5.9168
6.7071	6.5355	5.0935	6.6026	6.2364	4.7075	6.7003	6.4625
6.0221	6.364	5.3589	7.0856	7.5665	6.8433	7.0647	6.6818
6.7091	7.3547	6.656	7.0507	6.1028	7.1262	6.8264	6.6284
6.6501	6.1725	5.9296	5.9671	6.1444	6.5392	5.0958	7.0856
7.2471	6.7853	5.2268	6.5392	7.068	5.2025	7.3886	6.8812
7.0173	6.7524	5.6796	6.3908	6.1682	7.5978	6.3417	6.5172
6.9615	5.564	6.0946	6.6265	6.3565	6.2608	6.4699	6.5187
6.6071	6.8972	4.1266	5.7074	4.3222	5.79	6.82	6.2538
7.1154	7.084	7.6254	5.7735	5.2956	5.6414	5.7976	6.448
6.7604	6.3526	6.2867	5.9166	6.3431	6.5558	7.5433	4.8158

5.8427	4.9801							
C10orf10	1.8513	0.5285	0.0752	0.2324	0.3411	0.3201	0.1125	
0.5259	1.6847	0.8738	0.294	4.0899	0.5928	0.5031	1.3304	
0.4775	1.4252	0.1829	0.3984	0.5729	0.4062	2.0031	1.9785	
0.9326	1.1034	4.7122	3.2456	0	0.3466	0.356	0.08	
1.3886	0.1614	1.584	2.3568	0.1794	2.1992	3.4744	3.3986	
1.0393	2.7231	3.0639	3.7159	1.8169	1.2568	0.6977	0.2184	
0.365	1.4686	2.5096	0.9438	2.1827	0.8483	0.8048	0.047	
0.3951	2.1953	0.2771	0.3286	0.5491	0.7808	0.5813	1.349	
0.5277	1.5736	3.5758	1.475	0.3009	0.0284	0.9821	1.9179	
0.6675	0.3445	3.5899	2.9025	0.1328	0.7412	2.2584	1.4024	
1.5427	0.5211	3.617	0.2983	0.3249	3.1011	1.2624	1.1906	0
	0.4344							
C10orf105		0	0	0.0242	0	0.0021	0	
0.0053	0	0.0159	0	0	0	0.0051	0	0
	0.0036	0	0.0205	0	0	0.0361	0	0
	0	0.0034	0.0244	0	0	0	0	
0.0773	0	0	0	0	0	0	0	0
	0.011	0.0079	0.0011	0	0	0.0123	0	0
	0	0	0	0	0	0	0	
0.02	0	0	0.0196	0	0	0.04	0	0
	0	0	0	0	0.0034	0	0.043	0
	0	0.0151	0	0	0	0	0	0
	0	0.0083	0	0.0081	0	0	0	
0.0184	0	0						
C10orf107		0.9363	0	0.0515	0.1502	0	0.0338	
0.1249	0.0358	0	0	0	0	0.1715	0	
0.1187	0	0	0.4234	0.0563	0.043	0	0	0
	0	0.0202	0.5118	1.2788	0	0.0755	0.6232	
0.0016	0.0314	0	0.0951	0.1362	0	0	0	0
	0	0.2668	0.4974	0	0	0	0	0
	0	0	0	0.2451	0	0	0.073	0
	0	0	0	0.0975	0	0.1145	0	
0.1001	0	0	1.9017	0	0	0	0	
0.2798	0.1497	0	0	0	0.0725	0	0.1548	
0.0781	0	0	0	0	0.2441	0	0	
0.3731	0.6325	0.5769						
C10orf11	4.8056	4.1907	2.8754	2.8362	0.7944	2.9707	3.679	
3.2971	3.5318	3.5478	3.7441	3.8154	2.7025	4.5642	2.9874	
4.2133	0	4.2213	3.6319	2.1678	3.6019	2.3733	2.5303	
1.8495	2.3319	1.2215	4.1078	1.7162	2.7003	4.0847	0	
3.9417	3.4052	4.701	3.7966	2.3764	3.1149	3.6952	2.3066	
1.534	5.4332	4.0501	1.645	2.9436	1.2622	1.6744	3.2015	
3.9381	2.5104	0.3508	3.3873	1.4588	3.248	4.3748	0	
1.8752	3.8629	0.3454	2.3575	0.1877	0.6503	1.9908	3.0356	
4.0696	4.3941	3.5767	2.0072	3.9051	3.4533	2.2671	3.7573	
3.2297	3.5232	2.5959	2.8294	4.3204	3.9854	0.5459	4.6007	
4.5436	4.6567	2.8855	3.2743	3.3653	2.2134	3.1234	4.9456	
5.0744	4.9895							
C10orf113		0	0	0	0	0	0	0

	0.0731	0	0	0	0	0	0	0
	0	0	0	0	0	0	0	0
	0	0	0	0.0843	0	0	0	0
	0	0	0	0	0	0	0	0
	0	0	0	0	0	0	0	0
	0	0	0.23	0	0	0	0	0
	0	0	0	0	0	0	0	0
	0	0	0	0	0	0.14	0	0
	0	0	0	0	0	0	0	0
	0	0	0	0	0	0	0	0
	0	0						
C10orf12	1.248	2.2184	2.3249	2.1087	1.8583	2.2307	1.6522	
1.9596	1.958	2.2152	1.3735	1.7986	1.4702	1.8891	0.6165	
1.5756	2.3572	2.0364	1.9446	1.6407	2.058	1.3785	1.3531	
1.8353	1.436	1.1922	1.611	1.7324	1.2749	1.03	1.0863	
1.3354	1.4217	1.3184	1.3987	2.1143	1.8815	1.184	1.2298	
1.6121	1.1055	0.6866	1.6069	1.6598	2.0441	2.5162	2.0597	
2.0221	0.7928	2.6733	1.4093	1.2578	1.6244	1.6764	2.4893	
2.1995	2.1659	1.6099	1.8175	1.7025	1.1197	1.8865	2.0397	
2.1966	1.9808	0.8689	2.0367	1.6206	2.0135	1.0863	1.385	
1.6368	1.0552	0.9998	1.1311	2.0286	2.2178	1.566	1.4007	
1.4797	1.7019	1.4879	1.9537	1.1445	0.7285	1.2834	1.2706	
1.3198	1.4336							
C10orf120		0	0	0	0	0	0	0
	0	0	0	0	0	0	0	0
	0	0	0	0	0	0	0	0
	0	0	0	0	0	0	0	0
	0	0	0	0	0	0	0	0
	0	0	0	0	0	0	0	0
	0	0	0	0	0	0	0	0
	0	0	0	0	0	0	0	0
	0	0	0	0	0	0	0	0
	0	0	0	0	0	0	0	0
	0	0	0	0	0	0	0	0
	0	0						
C10orf128		0	0.0281	0	0	0	0	
0.39	0.2207	0.2662	0.0585	0.097	0.201	0.0075	0.669	
0.1326	0.171	0	0	0	1.0442	0	0.9131	
1.953	0	0	0	0.2614	0	1.1992	0	0
	0	0.9849	0	1.5346	0.1429	1.9484	0	
0.6322	0.0202	0	0	0	0	0.0065	0.137	0
	0.4297	0	0	0.2683	0	0	0.0494	0
	0	0.0488	1.2834	3.3847	0.0634	0.0334	2.0303	
0.0372	0.1815	0	0.0464	0.1887	0.0542	0.0727	0	
0.0343	0.2525	0.0381	1.686	0	0	0.1193	0.1287	
0.047	0.1019	0	0	0	0	0	0.1549	0
	0.0475	0.0561						
C10orf131		0	0.2088	0.664	0.1204	0.0892	0.2865	
0.178	0.122	0.0296	0.3307	0.1694	0.0393	0.2315	0.1998	0
	0.1304	0.1713	0	0.2584	0.1412	0.1662	0	

0.0431	0.2188	0.0723	0.0292	0.0335	0.589	0.0721	0.2468	
0.2869	0.2166	0.472	0.0792	0.0103	0.1848	0.1952	0.3868	
0.0539	0	0.1104	0.0318	0.2103	0.2094	0.0473	0.1597	
0.2407	0.0996	0.0449	0.6658	0.0701	0.0378	0	0.3179	
0.3854	0.1711	0	0.1586	0.0694	0.0897	0.228	0.2182	
0.0833	0.1618	0.2256	0.0752	0.7624	0.0859	0.2619	0.0684	
0.0972	0.4112	0.4608	0.1316	0	0.3813	0.7416	0.0396	
0.0266	0.2902	0.2278	0.0556	0.033	0.0398	0.0442	0.1002	
0.2829	0.0737	0.4332						
C10orf2	3.116	4.4716	3.408	3.7834	3.997	3.2215	3.1264	
3.4117	2.9748	3.2414	3.0626	3.9778	2.7207	3.1201	2.3467	
2.753	3.7005	2.8605	2.868	3.3477	2.9737	3.6029	3.044	
3.5343	2.4378	2.1648	3.0951	3.22	3.039	3.6135	2.3795	
3.7565	2.7678	1.9873	1.8065	3.0834	3.6922	2.6988	3.6043	
3.0911	3.8573	2.0263	1.7858	2.9668	2.3147	2.9031	3.3161	
3.5621	2.2599	3.5181	2.7327	3.0386	3.1067	3.6935	3.5638	
3.6383	2.6703	3.5616	2.8782	3.2266	3.9216	4.5024	3.6095	
2.9176	3.327	2.2638	3.0442	2.1193	2.7	2.7434	2.6689	
2.4162	2.438	3.5419	2.8617	2.5893	3.3516	2.8377	2.3978	
3.0638	3.2855	3.5275	2.0161	2.4415	2.5763	2.8713	2.0775	
2.8719	2.2347							
C10orf35	1.6749	3.0884	2.7874	0.5708	1.0501	0.5066	2.6096	
1.5831	2.2324	2.2789	1.9479	1.6679	2.2279	1.7507	1.4763	
3.1464	1.9851	1.835	1.585	2.1737	3.1773	0.3688	0.6638	
2.128	0.5072	1.1664	1.9327	2.9048	0.2562	1.9643	3.5053	
1.2277	1.1216	0.271	0.426	2.3726	1.5736	2.1702	0.881	
0.7761	1.6366	2.4909	0.8144	0.3024	1.8362	0.2643	0.1092	
0.461	0.5409	0	0.615	1.1893	0.1334	1.7653	4.1566	
2.1552	1.7272	2.268	0.7478	0.1593	0.226	0.0457	2.1226	
1.0781	0.9637	2.4818	1.6785	2.871	1.8824	0.5311	0.6656	
0.4072	1.698	0.7612	0.3477	0.3477	2.7713	2.125	1.4834	
2.7334	2.2555	1.0295	2.5111	0.7524	0.6165	0	0.3178	
0.3994	0.232							
C10orf53	0	0	0	0	0	0	0	0
	0	0	0	0	0	0	0	0
	0	0	0	0	0	0	0	0
	0	0	0	0	0	0	0	0
	0	0	0	0	0	0	0	0
	0	0	0	0	0	0	0	0
	0	0	0	0	0	0	0	0
	0	0	0	0	0	0	0	0
	0	0.028	0.0739	0	0	0	0	0
	0	0	0	0	0	0	0	0
	0	0	0	0	0	0	0	0
	0							
C10orf54	0.443	0.0384	0.0443	0.4973	1.4727	0	0	
0.7187	0.126	0.0866	0	2.3613	2.2684	0.0689	0.2873	
0.2643	0	0.1945	0.0193	0	0.0287	1.3482	0.1654	0
	0.2236	2.0557	0.1057	0.1609	0.7614	0.0683	1.4966	
1.4863	0	0.2848	0.8614	0.1925	0.9831	1.0052	0.155	

0.0666	0.2141	2.7356	1.5808	0.0781	1.5322	0.392	0	0
	0.5602	0	0.7946	0.1428	0.2975	0.4667	0.0642	0
	1.2602	0.2241	0.0532	0.0698	0.0613	0.0456	0.8718	
0.0674	0.1008	1.5895	0.4711	0.5245	0.0573	2.8784	0.0544	
0.0547	0	1.9026	0	0.226	0.191	0.1548	0.2371	
0.1377	0.0546	2.4571	3.2921	0.5875	0.7836	0.372	0.2281	
0.075	0.1673							
C10orf62	0	0.2993	0.0692	0	0.104	0	0.0625	0
	0.097	0	0	0	0.0254	0	0	
0.0258	0	0.1232	0	0.2179	0	0	0.0884	0
	0	0	0	0	0	0	0	
0.2096	0	0	0	0.0313	0.0478	0.0487	0	0
	0.0323	0.0312	0	0	0	0	0	0
	0.0329	0.1132	0	0	0.0835	0.0257	0	0
	0.0487	0.0449	0	0.0879	0.0889	0.2124	0	0
	0	0.0532	0.2696	0.0396	0	0	0	0
	0.0891	0.003	0	0.0408	0	0.0795	0.0206	0
	0	0	0.0285	0	0	0	0	0
	0.0648							
C10orf67	0.1463	0.0605	0.0343	0.1447	0	0	0.0095	
0.0121	0.0125	0.1408	0.0385	0.0476	0.1931	0.0355	0.1057	
0.0473	0.1012	0.1682	0	0.0862	0.2452	0.0636	0.0128	
0.0118	0	0.0738	0.0341	0	0.0424	0.0329	0.025	
0.2741	0.2026	0.0335	0	0.0562	0.2685	0.0263	0.0109	0
	0.028	0.0646	0.1921	0.0742	0.0937	0.0168	0.1941	
0.0227	0	0	0.0439	0	0.1116	0	0.0416	0
	0.0452	0.1821	0.1819	0.0781	0.0336	0.043	0.0644	
0.2028	0	0.0251	0	0	0.1255	0.1476	0.076	
0.0085	0.0134	0.0314	0	0.078	0	0	0.0599	0
	0.1243	0.1252	0.0726	0.0507	0.1792	0.0423	0.1659	
0.1251	0.0583							
C10orf71	0	0	0	0	0	0	0.0039	0
	0	0	0	0	0	0	0	0
	0	0.0172	0	0	0	0	0	0
	0	0	0.0124	0	0.0091	0	0	0
	0	0	0	0.006	0	0	0	0
	0	0.001	0	0	0.004	0	0	0
	0	0	0	0	0	0.0039	0	0
	0	0	0.0132	0	0.0107	0	0	0
	0	0	0	0	0	0	0.0063	
0.0076	0	0	0	0	0	0	0.0025	0
	0	0	0.0063	0	0	0	0.0164	0
	0							
C10orf76	3.2058	3.4117	3.0807	3.1922	3.2655	3.4069	2.9291	
3.0223	3.6167	3.8993	2.7598	3.434	3.5327	3.7274	3.0315	
3.474	3.1088	3.507	3.0013	3.2399	2.7157	2.4946	2.632	
3.2819	3.0973	2.31	4.361	3.5834	2.6583	2.8447	4.0352	
3.2093	3.2574	3.354	2.9151	4.1811	3.4267	2.8318	3.1576	
2.9376	3.3305	2.3923	3.3562	3.0344	3.0197	3.431	3.443	
3.0833	2.4	3.6264	2.9918	2.8561	3.4406	2.8582	4.2497	

2.6173	3.1419	3.2469	2.958	3.1122	3.1757	3.7148	3.2557	
3.6694	3.0737	3.3562	3.1547	3.6849	2.5934	2.8762	3.1842	
3.078	2.9482	2.7763	2.9952	3.7719	3.7319	2.8772	2.4622	
3.0459	3.7974	3.4743	3.334	2.4497	2.9139	2.9647	3.7221	
3.4295	3.4838							
C10orf82	0.1169	0.1982	0.0551	0.1394	0.0198	0.0502	0.0485	0
	0.1295	0.6665	0	0	0	0.1223	0.143	
0.0662	0.0613	0.4537	0.1522	0.056	0.1428	0.0111	0.009	
0.194	0.0353	0.0205	0.1012	0.1067	0.052	0.2002	0.0198	
0.0616	0	0.0963	0.0769	0.1694	0.0944	0.038	0.0466	
0.2156	0.0233	0.0487	0.0453	0.0327	0.084	0.0626	0.1328	
0.0749	0.12	0.0884	0.0804	0.0335	0.2042	0	0.1381	
0.2802	0.0514	0.0488	0.0936	0.1314	0.0762	0.0719	0.0369	
0.1718	0.0559	0.0334	0.4298	0.0387	0.0609	0	0.0342	0
	0.1328	0.08	0.0666	0.1177	0.2612	0.042	0.0232	
0.2726	0.0363	0.0248	0.0316	0.0836	0	0	0.0974	
0.0794	0.0508							
C10orf88	2.208	2.647	2.3874	2.8407	2.2437	2.6388	2.4683	
2.2892	2.3408	2.333	2.1825	2.5018	2.3908	2.708	1.8366	
2.4363	2.3887	2.4805	1.9565	2.8684	2.5675	1.8149	1.6309	
2.3855	1.9175	2.3908	1.8372	2.5917	1.4966	1.8449	2.8811	
2.1396	2.3602	1.8324	1.778	3.0827	2.778	1.6158	1.6085	
1.7115	2.0332	2.3741	2.0845	2.1644	2.0517	1.9165	2.5254	
2.3088	2.6729	2.7918	2.1392	2.0381	2.6781	2.3263	3.2986	
2.0377	1.6656	2.1677	2.1105	1.6856	2.0752	2.3864	2.5031	
2.1928	2.2903	2.3651	2.2519	2.7371	1.9521	2.295	1.9633	
2.176	2.3697	1.7617	1.8139	2.6937	2.7174	2.1379	1.559	
1.4761	1.856	2.0085	2.597	1.7795	2.1666	2.422	2.1682	
2.3755	2.3375							
C10orf90	4.4133	4.1018	3.661	1.8499	3.4114	4.1431	3.2042	
4.6028	4.8276	3.8749	3.9139	3.8803	0.1712	4.7945	3.8788	
4.0217	0.0371	4.2409	3.8359	4.5008	4.2079	2.9318	2.6616	
3.6228	0.2861	0	3.6444	3.9784	3.5111	3.2698	0	
3.3275	3.7548	4.0321	3.2321	3.9421	3.5614	3.4053	4.9113	
3.8359	4.5956	0.1998	2.4387	5.0144	0.4563	0.0138	4.1818	
3.5468	5.8705	0.2027	3.7107	4.3657	3.8953	4.535	1.5599	
1.9418	0.1307	0	3.5648	0	3.9864	2.946	5.0029	
4.9212	2.8947	0.2554	3.2547	5.7283	3.5242	3.3917	3.8312	
4.1162	3.3457	3.03	4.6737	4.4319	4.1626	4.6732	3.9832	
3.0165	3.8582	4.686	3.7237	4.7162	0.1374	3.5663	4.9472	
5.057	5.0254							
C10orf99	0	0	0	0	0	0	0	0
	0	0	0	0	0	0	0	0
	0	0	0	0	0	0	0	0
	0	0	0	0	0	0	0	0
	0	0	0	0	0	0	0	0
	0	0	0	0	0	0	0	0
	0	0	0	0	0	0	0	0
	0	0	0.1007	0	0	0	0	0
	0	0	0	0	0.0302	0	0	0

	0	0	0.0319	0	0	0	0	0
	0	0	0	0	0	0	0	0
	0							
C11orf1	3.9403	3.2961	2.3952	4.0168	3.8793	3.2018	4.0005	
3.2633	3.5784	2.893	3.4904	2.5933	3.5677	3.6934	2.7455	
3.5191	3.24	2.7739	3.1215	3.9823	2.2061	3.3906	3.8696	
2.8201	2.3915	2.9933	4.6727	2.456	3.7791	3.1636	2.9439	
3.5807	3.6843	3.7128	1.9977	3.1648	2.5466	3.553	3.3547	
4.2367	2.2569	3.7999	3.298	3.8793	3.9453	3.1433	3.5095	
4.5971	3.9109	5.0757	3.4124	3.9485	2.8886	4.6422	2.0275	
2.8397	3.772	2.2142	3.8107	1.7458	3.2903	3.0374	2.7398	
3.5703	2.9777	3.7731	4.3451	4.1028	2.781	3.5151	3.2373	
2.7092	3.4647	3.428	3.6945	4.3174	2.8603	3.1137	4.3688	
3.4845	3.5936	3.9625	3.5333	3.3607	3.1214	2.5952	4.0362	
3.7737	3.4081							
C11orf16	0.0585	0.0695	0.0516	0	0.0378	0.0759	0.0449	
0.0472	0	0	0.0121	0	0.0389	0.0636	0.0864	0
	0.1172	0	0.0809	0.1091	0.1091	0.0294	0.0051	
0.0231	0.0324	0.0437	0	0.0477	0.0732	0	0	
0.015	0.1261	0	0.0282	0	0.0566	0.0559	0.0425	0
	0.0465	0.0346	0.0446	0.0304	0.0121	0.0462	0.0453	
0.0353	0.0218	0.2568	0.0522	0	0	0.0261	0.1249	0
	0	0.1156	0.0459	0.0412	0	0	0	
0.0546	0	0	0.0646	0.027	0.0247	0	0.095	0
	0.0408	0	0.0309	0.0745	0	0.0739	0.0215	
0.1673	0.0325	0.0489	0	0	0	0.0907	0	
0.041	0.0857							
C11orf21	0	0	0	0	0	0	0	
0.0131	0	0	0	0.0373	0	0	0	0
	0	0.6775	0	0	0	0.0321	0	0
	0.0057	0	0.0229	0	0	0	0.0263	0
	0	0.0175	0	0	0	0	0.03	0
	0	0.3206	0	0	0	1.076	0	0
	0	0	0	0	0	0.0415	0.1266	0
	0	0	0	0.062	0	0.1865	0	0
	0	0	0	0	0	0	0	0
	0	0	0	0.1521	0.0291	0.1081	0	0
	0	0	0	0	0	0	0	0
	0							
C11orf24	5.2847	4.45	5.4356	4.1461	5.1058	4.3019	5.0512	
6.128	5.3362	5.3651	5.9018	4.794	4.1435	4.3003	4.3988	
4.5736	3.9177	4.5535	5.0323	5.5425	5.1914	5.4621	5.2003	
5.7677	5.1819	4.5126	2.9377	4.8788	5.2326	4.2478	3.3822	
4.6001	5.3902	5.4682	4.8744	4.3844	6.3785	5.2769	5.353	
4.9094	4.9838	4.0307	5.5272	5.4412	4.1276	4.5465	5.0283	
4.459	4.6765	3.2844	5.0623	5.4906	4.2363	6.3273	4.1002	
5.1782	2.8509	4.6058	5.931	4.3167	4.655	5.2288	5.3588	
5.1253	5.1827	4.764	5.3069	4.8008	5.8046	5.1757	5.3018	
4.3891	5.2513	5.4495	4.0654	4.7724	6.3447	5.047	5.3351	
5.0969	4.3849	4.7447	4.4148	5.5282	4.5727	4.6429	5.0456	

5.6372	5.0628							
C11orf31	5.3519	5.83	5.6223	5.6628	5.9554	5.4255	4.7279	
5.5978	5.0041	4.7417	5.3817	6.3212	5.2902	5.1375	4.5334	
4.7576	5.0632	5.5646	5.2914	4.3004	4.3468	5.364	5.9698	
5.3376	5.8323	4.5638	5.9835	4.4828	5.5654	5.5359	4.0065	
5.2937	5.1443	4.8669	5.5404	5.1458	6.2121	6.3778	5.7613	
5.3273	6.1809	5.2262	4.8797	5.5862	5.4224	5.3933	5.195	
5.0383	5.6244	5.7341	4.7066	5.8315	6.0038	5.5749	4.5225	
5.1797	5.8619	4.9251	5.4494	5.1737	5.4541	5.0489	6.3357	
5.0686	5.9796	5.0173	6.2244	5.8401	4.9959	5.0303	6.061	
4.9202	5.6364	5.2172	5.6581	5.7054	5.7935	5.2532	5.9083	
6.5575	5.603	5.5736	5.3625	5.3821	5.6156	6.4113	5.358	
5.6048	5.4603							
C11orf40	0	0	0	0	0	0	0	0
	0	0	0	0	0	0	0	0
	0	0	0	0	0	0	0	0
	0	0	0	0	0	0	0	0
	0	0	0	0	0	0	0	0
	0	0	0	0	0	0	0	0
	0	0	0	0	0	0	0	0
	0	0	0	0	0	0	0	0
	0	0	0	0	0	0	0	0
	0	0	0	0	0	0	0	0
	0	0	0	0	0	0	0	0
	0							
C11orf42	0	0.1456	0	0	0.0182	0.0927	0.0907	
0.4011	0.0905	0.0981	0.1843	0	0	0	0.0844	0
	0.4197	0.0919	0.1576	0	0.3176	0	0	0
	0.0873	0.041	0	0	0.0435	0	0	
0.0595	0.0574	0	0	0	0	0.064	0	0
	0	0	0	0.0603	0.0391	0	0	0
	0.1474	0	0.1872	0	0.2729	0.1513	0	0
	0.082	0	0.4394	0	0	0	0.0645	0
	0.0936	0	0.1179	0	0.1023	0	0.1183	0
	0	0	0.0579	0.0703	0	0	0	0
	0	0.0469	0.1136	0	0	0.1781	0	
0.2566	0.0774							
C11orf45	0	0.0862	0.0362	0.4438	0.7082	0.0088	0	
0.0385	0.9695	0.7933	1.0431	0.3091	0.9798	0.574	0.1117	
1.1005	2.4501	0.6102	1.0211	0.7784	0.3954	0.3001	0.2544	0
	0.1662	0.5185	0.4551	0.0155	0.3233	0.0906	0.0714	
0.1099	0.1519	1.0248	0.0939	0.2917	0.1888	0.0846	0.0706	0
	0.9538	0.5911	0.8037	0.3491	0.3362	0.4236	0.8651	
0.0016	0.0646	1.5051	0.8732	0.11	0.3926	0.3822	0.1446	0
	1.8146	0.5477	0.0838	0.5078	0.079	0.0522	0.0555	
0.1214	0.5516	1.2582	0	1.195	0	0.8099	0.5023	
0.0559	0.785	0.2631	0.1141	1.3695	0.3866	0	0.1964	0
	0.0917	0.6867	0.6601	0.4778	0.488	0.5321	0.4095	
0.8008	0.7984							
C11orf49	3.4795	4.0303	3.4327	3.7179	3.9348	3.6489	3.8136	

3.9716	4.0538	3.1454	4.4957	3.5842	3.1327	3.066	3.9304	
3.6956	4.4868	2.7955	3.7452	3.8884	2.3493	4.1815	3.8525	
3.333	4.7849	4.1397	4.2739	3.8759	3.6489	4.3411	3.9188	
3.4681	3.4744	3.7672	3.7878	2.9451	3.925	4.5612	4.0557	
3.5941	5.1009	4.7375	3.6559	2.9383	3.77	4.1839	3.4887	
3.8204	4.3201	4.277	4.1982	3.2246	3.692	4.2042	4.7352	
3.3942	3.567	3.5265	3.1915	3.7514	3.1412	3.9681	4.1262	
3.7809	3.4179	4.6616	3.1338	4.7773	3.3893	3.8176	3.7996	
2.9129	3.1372	4.4779	4.0132	4.2966	2.9464	3.6946	3.876	
3.99	4.2847	3.4562	3.7491	3.6336	4.5827	3.5433	4.2695	
4.0341	4.3052							
C11orf52	0.1342	0	0	0	0.0839	0.3191	0.0229	0
	0.1219	0.0702	0	0.1277	0	0.0711	0.5916	
0.3282	0.0699	0.0541	0	0	0.0561	0.0376	0.0313	
0.2991	0.0976	0.0231	0.118	0	0.0909	0	0	
0.0258	0.04	0.1575	0.0691	0.0251	0	0.4261	0.025	
0.5658	0	0.0067	0	0.0835	0.0209	0.0354	0	0
	0.0886	0	0.0321	0.0751	0	0.0203	0	0
	0.3123	0.0463	0	0	0.2899	0.4005	0.4484	
0.0851	0.168	0.0089	0.3927	0.1156	0.0212	0.1468	0.0772	0
	0.0349	0.0061	0.0096	0	0.0169	0.0108	0.7753	0
	0	0.064	0.051	0.0909	0.0759	0.1188	0.3988	
0.2991	0.635							
C11orf53	0	0	0	0	0	0	0	0
	0	0	0	0	0	0.1282	0	0
	0	0	0	0	0	0	0	0
	0	0	0	0	0	0	0	0
	0	0	0	0	0	0	0	0
	0	0	0	0	0	0	0	0
	0	0	0	0	0	0	0	0
	0	0	0	0	0	0	0	0
	0	0	0	0	0	0	0	0
	0	0	0	0	0	0	0	0
	0	0	0	0	0	0	0	0
	0							
C11orf54	3.4665	3.3167	2.4673	2.6029	3.1326	1.9583	3.0629	
2.1048	3.8663	2.4596	3.1657	2.8261	3.0141	2.8103	2.1602	
2.4766	3.0671	3.2813	2.5523	2.1873	1.8135	3.2269	3.1571	
3.0517	3.4234	2.1418	3.2315	2.9602	2.9433	3.6943	4.095	
1.7394	3.0232	2.9068	3.3134	3.1529	3.0129	2.9126	2.6748	
2.7968	3.4262	3.7608	1.9603	3.2765	2.2577	3.4295	2.3862	
3.5379	2.8829	4.3502	3.1973	3.3525	3.0854	3.1502	4.0974	
3.1785	3.4457	1.9891	2.4139	1.8295	2.1044	2.9739	2.9387	
3.1257	2.679	3.5201	3.5821	2.1801	3.2962	2.7103	3.4596	
2.6715	2.9568	2.6112	3.4992	2.9641	2.5512	3.0212	2.9218	
2.0365	2.113	3.4393	4.3326	2.9433	2.4834	2.8627	3.203	
2.8573	3.4299							
C11orf57	3.0771	3.6764	3.2033	4.2994	3.3935	3.534	3.9272	
2.8191	3.3831	2.6485	3.1447	2.6547	3.4103	3.6993	2.8825	
3.3666	3.7942	3.0911	3.2464	3.3014	2.485	3.8251	3.4772	

2.6475	3.2013	3.5857	3.6702	2.7555	3.7498	3.6287	3.1408
2.725	3.2352	2.6161	1.7884	3.0559	2.8653	3.4274	3.1577
3.6317	2.7063	3.5968	2.9726	3.3153	3.2702	3.1052	2.9277
3.6953	3.498	4.3141	3.1725	4.1039	2.645	4.6239	3.0899
3.4848	3.3253	2.6904	3.1146	2.867	3.577	3.5132	3.2194
3.6222	2.7865	3.5598	3.6118	3.3813	2.6482	3.4352	2.827
3.3491	3.8523	3.5699	3.6881	3.7205	3.0868	3.1079	3.6449
3.0961	3.1322	3.9761	2.5187	3.0625	3.2574	2.1898	3.6537
3.3194	3.5344						
C11orf58	5.2698	5.5367	5.6908	5.6747	4.9261	5.3442	5.7035
6.1571	5.528	5.1158	5.6178	4.9962	5.6079	5.5874	6.1085
5.8334	5.5506	4.8423	5.7665	5.4717	5.7199	5.6538	5.3355
5.5359	6.0266	5.6261	4.2635	5.7831	5.6715	5.0896	5.6913
5.5777	6.2389	4.7767	5.5908	5.5208	5.851	5.684	5.3966
5.3697	4.8668	5.2999	6.0549	5.4453	5.1986	5.7035	5.5746
5.6597	5.8325	6.0952	6.1247	5.5185	5.5975	6.573	5.9419
5.5972	5.603	5.7703	5.6438	5.6719	5.0079	5.5768	5.5584
5.3424	5.6092	5.5089	5.288	5.7435	5.3192	5.2263	5.244
5.4657	5.69	5.936	5.5679	5.9467	5.4472	5.2006	5.7727
5.5603	4.9085	5.3581	5.9725	4.7801	5.3406	5.5808	5.1986
5.5589	5.3688						
C11orf63	1.8596	1.2758	1.6087	1.7075	1.5039	1.672	1.8524
0.5824	1.8774	1.441	1.4488	1.1109	2.3816	1.9577	1.3176
2.4093	1.5499	1.494	1.7319	1.6142	1.3619	1.6319	1.8493
1.7331	1.0273	2.5871	1.604	1.1379	2.1877	1.8486	1.565
0.6865	1.9129	1.661	2.0562	1.8625	1.1852	1.433	1.3816
1.6155	1.2911	2.267	1.8077	1.6223	1.0266	1.1704	1.4857
2.1511	1.9297	0.7802	1.929	1.8108	1.1393	1.8357	1.5834
2.1755	2.1201	1.8122	1.5669	1.8139	1.1553	1.3836	0.7617
2.2793	1.0226	2.2837	1.633	2.0492	1.4815	1.9084	1.3811
1.809	1.8068	0.866	2.0481	2.1321	1.637	1.54	1.7966
1.419	1.3365	0.5453	1.7917	1.5734	1.1456	0.8835	2.0216
1.6155	1.8218						
C11orf65	0.8035	0.2901	0.17	0.2181	0.2564	0.2659	0.3299
0.2413	0.5536	0.5814	0.179	0.0851	0.3888	0.7859	0.2264
0.3244	1.0703	0.448	0.6683	0.3543	0.0653	0.1861	0.1016
0.0552	0.0338	0.2731	0.2759	0.1788	0.1441	0.2875	0.3711
0.1572	0.3017	0.487	0.1854	0.1177	0.2604	0.151	0.3056
0.7791	0.2258	0.226	0.3821	0.903	0.2922	0.4811	0.446
0.5229	0.2674	1.0691	0.412	0.3356	0.3935	0.4506	0.3267
0.7241	1.0838	0.3273	0.2471	0.0898	0.0745	0.2478	0.0808
0.4091	0.6737	0.7605	0.6793	0.425	0.1289	0.416	0.1002
0.2948	0.5351	0.1717	0.2771	0.3568	0.4297	0.1933	0.5219
0.9513	0.5558	0.2302	0.1094	0.6249	0.2991	0.2522	0.2341
0.6661	0.5116						
C11orf68	4.3987	3.323	3.8547	3.0329	4.6076	4.9836	4.5038
4.8756	3.2804	3.4918	4.4538	4.4086	5.6267	3.9031	5.572
4.8388	5.8641	2.9795	2.8767	5.4911	4.7057	5.0757	4.9276
4.9569	4.9627	5.986	3.5868	3.2896	5.2104	3.4257	3.544
3.761	5.041	4.117	4.4715	2.868	5.6077	4.5125	4.469

3.6056	3.8859	5.0743	5.2001	4.6076	4.6313	5.0757	3.1908	
4.1757	5.764	5.2167	4.3145	3.5487	3.2446	4.477	3.6166	
3.4307	5.7568	3.7699	4.343	3.1714	4.6572	3.521	4.0313	
4.0455	3.4706	5.7394	3.018	5.5979	2.5635	5.2423	5.5325	
4.756	3.7394	3.772	5.0474	4.1782	4.3047	3.7745	3.9393	
4.0332	3.1288	5.3662	5.4853	4.7856	5.1006	4.2546	4.5183	
4.3776	4.7069							
C11orf70	0.0914	0	0.0138	0	0.7032	0	1.1904	
0.0246	0.0795	0.0881	0.3608	0.2056	0	6.00E-04	0	0
	0.1101	0	0.0871	0	0	0.991	0.2478	0
	0.1063	1.4513	0.0212	0.0133	1.924	0.1857	3.0276	0
	0.1004	1.5465	0	0.3869	0	0	0	
0.0986	1.3823	2.4807	0.8614	0.0465	0.7508	0.0044	0	0
	0.9551	0	0.8483	0	0.0118	0.4338	2.0674	0
	0.1948	0.2825	0.0951	0.3798	0.0726	0.067	0.1657	
0.2228	0.0813	2.2225	0	0.1242	0	0	0.091	
0.0732	0.1255	0.3251	0.1093	0.0813	0.0971	0	0.0623	0
	0.0939	0.1277	3.2097	0.0751	0.6554	0	0.6082	
0.4772	0.1672							
C11orf71	1.7336	1.9778	1.7985	2.5774	2.314	1.9418	2.5103	
1.1187	3.3019	1.7963	1.8672	1.5744	1.3446	1.4555	0.8332	
1.7393	2.271	1.842	1.6619	1.8302	1.8431	2.3999	2.8175	
1.4882	0.6658	1.5627	2.5894	1.6488	2.0733	2.5496	2.9632	
2.5778	2.1049	2.1223	0.7076	2.065	1.233	2.7583	2.1332	
2.5894	1.7429	2.5699	1.0116	1.9037	2.3519	1.458	1.6684	
3.5741	2.1239	2.7742	1.9093	2.215	1.5787	2.0378	2.497	
2.2661	2.8185	0.9616	1.0453	0.875	1.8861	1.8817	1.235	
1.9361	1.6072	3.004	2.2689	1.7348	1.5663	1.9089	1.7373	
1.5389	2.6519	2.3074	2.3049	2.4783	1.7668	2.6644	3.1946	
2.4087	2.4666	2.3858	1.854	3.3732	1.5657	1.5205	2.5999	
2.1934	2.5971							
C11orf74	3.4627	4.2496	3.9349	3.0558	3.0647	2.5366	4.0431	
3.74	3.6764	3.3835	4.3153	3.2681	3.3943	3.8813	3.7869	
3.887	3.9482	3.3654	3.3573	3.2754	3.2693	3.1376	2.3983	
3.2785	3.6195	4.4661	3.8648	4.8851	2.6224	4.1575	4.3197	
3.7677	4.2366	3.5286	2.93	3.9201	4.071	3.1178	3.0546	
3.3668	3.3401	3.5969	3.3891	3.6986	2.3542	2.6175	3.5385	
3.7254	1.9441	4.6376	4.0964	3.4843	3.5922	4.1811	4.2148	
3.4772	3.5044	3.4134	3.8041	3.5661	1.8474	3.437	3.2715	
3.1152	3.4275	4.1849	3.7802	4.3152	3.9743	2.9991	3.1118	
3.3762	3.2357	4.2822	3.7107	4.812	3.5105	3.6297	4.0725	
3.7524	3.6015	2.8761	4.3062	3.7731	3.1913	4.0748	4.1499	
3.6268	4.0964							
C11orf80	2.4588	1.8844	2.5836	2.5676	2.22	2.2161	2.1012	
2.8033	2.4462	3.1243	2.1806	1.4328	2.2224	2.6609	2.4802	
1.8844	2.2845	2.6953	3.5428	2.3169	2.0264	0.8028	1.3352	
2.3275	1.85	0.5452	2.5925	2.8652	2.4525	2.0518	3.0694	
1.3841	2.2123	1.8962	1.8909	3.277	1.6682	1.5629	2.3211	
1.7994	1.9212	1.4225	2.8117	1.9581	2.3229	1.8715	2.9842	
1.8838	1.9207	1.8303	2.1579	2.4917	2.1224	2.7838	2.6034	

2.7726	1.9311	2.5403	2.7968	1.6787	1.5015	2.0366	2.3821	
1.7854	2.1421	1.3656	2.2367	2.7531	2.9213	1.9224	2.5451	
1.7924	1.68	0.8821	1.7794	2.8018	3.4544	1.3049	2.1106	
2.4657	1.8917	1.3042	2.5509	1.6506	2.7057	1.8974	2.5704	
2.7122	2.4705							
C11orf84	4.2317	4.6421	5.4119	3.1946	4.5278	3.8018	3.2659	
4.8895	3.0109	2.7076	3.5506	2.3622	3.4392	2.7643	4.2265	
3.4853	3.513	2.6045	3.0873	4.2603	4.3622	4.2985	3.5652	
3.8315	4.6094	2.9209	3.1461	4.2981	3.6672	3.1765	4.0527	
3.1946	3.5666	3.2191	3.367	2.9475	4.2115	4.0757	3.8634	
1.5954	3.7521	2.9047	3.5941	3.4163	3.2572	3.4609	3.9841	
3.5208	3.9982	3.4554	3.1965	2.3892	3.0006	4.837	4.4219	
4.3882	3.2551	5.3319	3.7215	4.9462	3.3743	3.159	3.9474	
3.354	4.0444	3.1558	3.053	3.9196	3.4813	4.0798	3.9568	
3.75	4.1616	2.2912	3.4929	3.9115	4.8565	2.79	3.21	
3.8304	3.2127	3.2915	4.8572	3.2631	4.6333	3.4947	3.621	
4.262	3.7796							
C11orf86	0.2028	0	0	0	0.185	0	0	
0.0944	0	0	0	0.0595	0	0	0	0
	0.211	0	0	0.2038	0.1529	0	0	0
	0	0	0	0	0	0	0	
0.0327	0	0	0	0	0	0	0	0
	0	0	0	0	0.1596	0	0	0
	0	0	0	0	0.3762	0	0.6832	0
	0	0	0	0	0	0	0	0
	0	0.006	0.2748	0.0301	0	0	0	0
	0	0	0	0	0	0	0	0
	0	0.0157	1.0194	0	0	0.0794	0	0
	0							
C11orf87	0	0	0	0	0	0	0.0186	0
	0.0513	0.019	0.0131	0.0126	0	0.058	0	0
	0	0	0	0	0	0	0	0
	0	0.0231	0	0	0	0.0233	0	0
	0.0835	0	0	0	0	0	0	0
	0	0.063	0	0	0	0	0.0368	
0.0319	0.0186	0.0751	0	0	0	0.0035	0	
0.0221	0	0.0083	0	0	0.0091	0	0	0
	0.0098	0.5682	0.0242	0.0069	0.0018	0	0	0
	0	0	0.0148	0.0138	0	0.0051	0	0
	0	0	0	0	0	0	0	0
	0							
C11orf88	0.0355	0	0.0327	0.0681	0	0.0258	0	0
	0	0.0131	0.0292	0.005	0.0456	0.0557	0.0584	0
	0.1167	0.0394	0	0	0	0.0297	0.0196	
0.0103	0.0252	0	0.0159	0	0.0483	0.015	0.0703	
0.0502	0	0	0.0266	0.0183	0.0363	0	0	
0.0121	0.0126	0.0217	0.0242	0.0326	0.1737	0.1629	0.0228	0
	0.0137	0.2682	0.0516	0.0287	0.0538	0	0.091	0
	0.1313	0.0217	0.072	0	0	0.0049	0.0111	0
	0	0.043	0.0776	0	0.0263	0.0473	0.0714	

0.0116	0.0415	0.1101	0.0503	0	0.055	0.0144	0.0187	
0.0338	0.008	0.0208	0.0076	0	0.105	0	0.0246	
0.0288	0.035							
C11orf91	0.8892	0.9475	0.1675	1.8301	1.6822	2.4297	2.3486	
0.236	1.1855	0.8697	1.765	1.2104	1.2208	0.7951	2.4792	
1.5177	1.1618	0.6075	0.9458	1.3295	1.3798	1.7435	3.0687	
3.2294	1.9714	0.2743	1.5051	0.7441	2.471	0.5415	0.1207	
1.7856	2.3062	1.4406	2.1631	1.4273	0.551	1.3673	1.3941	
2.4394	1.4674	1.9899	1.3425	0.698	1.0407	0.51	0	
1.5492	2.0488	2.1396	0.9663	4.0229	0.7275	0.4296	0.5485	
0.5498	2.1592	0.9788	1.126	1.4295	1.686	2.073	0.477	
2.9553	0	0.8847	0.4112	0.3771	0.1601	0.9706	1.1036	
1.1363	0.4017	3.5744	1.4547	0.6214	1.9112	1.5386	1.2635	
1.9531	1.1931	1.8462	2.1778	0.8538	1.8313	0.9274	1.1063	
0.1891	0.341							
C11orf94	0	0	0.4767	0	0.3313	0	0.3349	
0.2807	0	0	0	0	0	0	0	
0.4758	0	0	0	0.6301	0.7009	0.468	0	
0.194	0	0	0	0.746	0	0	0.2101	
0.4795	0	0	0	0	0.2199	0	0	0
	0.2385	0.3696	0	0	0	0	0	0
	0	0	0	0	0	0.1412	0.2207	0
	0	0.208	0.662	0.1864	0	0	0	
0.7863	0	0.2063	0	0.3125	0.1365	0	0	0
	0	0.2121	0.462	0	0.1783	0	0.1286	0
	0	0.5747	0.1648	0	0.2549	0	0	0
	0							
C11orf95	2.1133	3.1423	3.9311	2.3472	2.8918	2.8358	2.7588	
3.1505	2.2546	1.9106	2.4442	1.346	1.8127	2.9739	0.2965	
2.2718	2.9941	2.0041	2.1744	2.5178	2.4913	2.1365	2.3251	
2.3975	4.0212	2.462	3.0742	3.1189	2.7027	1.7896	1.4555	
1.8935	2.1861	2.0754	2.6057	2.0822	2.4851	3.006	2.462	
2.3889	2.0816	2.2338	2.4842	2.7323	2.3557	3.1746	3.0517	
2.2556	2.999	2.7974	2.035	2.3021	2.7825	3.7722	2.5192	
2.8341	2.5243	3.4851	2.2674	2.9742	1.9666	1.685	2.6193	
2.5434	2.7216	2.1554	2.8382	3.3132	1.9815	2.7171	2.9466	
2.4334	2.787	1.3833	2.3265	2.2787	3.3215	2.1238	1.842	
2.3733	1.5974	2.8036	2.9025	2.0335	3.3947	1.8407	2.5456	
2.7233	3.1623							
C11orf96	0.5489	5.3793	2.5659	0	1.6796	1.5388	5.037	
2.8011	2.8041	3.9688	6.6056	3.2546	0.4157	2.9391	0.6228	
4.0473	0	4.1939	4.2769	0.6719	1.3751	2.2924	0.6292	
2.1582	0.6285	0.4778	2.6752	5.9655	0.5935	5.5648	0.4612	
2.8778	4.0891	5.6011	0.3232	4.1715	1.8414	5.4152	3.8933	
1.715	4.7847	0.3562	2.0789	3.3398	0.2821	0	3.942	
2.5043	0.6529	0	2.6004	0.8821	4.7281	3.6001	0	
3.6541	0.8148	3.8381	1.8222	2.6516	1.6327	3.9751	1.9037	
2.097	5.0702	4.0364	2.9151	1.0662	2.5488	0	2.1321	
2.8898	1.9123	2.4575	0.5857	4.8335	4.7369	0.4424	5.4623	
6.0535	2.6548	0.3405	8.4891	2.1157	0.2509	3.7129	5.2196	

4.6198	5.1069							
C12orf10	4.7563	5.2971	4.4934	4.0048	5.4576	4.0102	3.6614	
4.8667	4.4871	4.3117	4.7766	4.311	4.221	4.1595	3.834	
4.4256	5.3681	3.7772	4.5026	5.097	4.6378	4.9751	4.6849	
4.5931	4.858	4.5053	5.9103	4.5983	4.8237	4.9281	5.1031	
4.9885	4.8108	4.8023	4.6109	4.023	5.3916	4.9983	4.8518	
4.6227	5.3339	5.0835	3.6051	4.5884	3.9212	4.3293	4.1468	
4.4803	4.7881	4.9036	4.3468	4.1995	4.4173	4.6604	5.0745	
3.9208	4.6802	4.7238	4.489	4.1687	5.3598	5.2248	5.3926	
4.6202	4.2644	4.7783	5.2791	4.5182	4.0364	4.6926	4.8443	
4.341	5.2455	4.9692	4.2999	4.1857	4.7218	4.113	4.7541	
4.8705	5.1445	4.9352	4.9461	4.2593	4.762	4.6677	4.4196	
4.565	4.5522							
C12orf29	3.235	2.9068	2.7879	3.7851	3.8856	3.3026	3.9222	
3.4222	2.779	2.8647	3.109	3.2919	2.6769	3.5394	2.8706	
2.8521	3.4829	3.1252	3.5819	3.26	3.561	3.4489	3.0079	
3.3483	2.8831	2.8924	4.2142	2.7952	3.2273	2.7847	2.9577	
3.2766	2.9364	2.5787	2.8741	3.269	3.6946	2.4863	2.7356	
3.5455	2.922	3.1391	2.7121	2.2447	2.9304	3.357	2.9551	
3.0285	2.9026	3.7164	3.1535	3.7595	3.5034	3.0488	3.7012	
3.2794	3.1159	3.2916	3.6708	3.137	3.8345	3.9065	2.888	
3.4005	3.3346	2.7861	2.9322	2.8123	3.2255	2.976	3.0904	
3.1353	3.4975	3.3584	3.0426	2.4412	3.0552	2.5118	2.3025	
3.3333	2.8346	3.4691	3.2383	2.9196	2.3192	3.6284	2.6295	
3.035	2.4174							
C12orf4	2.3918	3.1977	2.8955	3.1993	3.1453	2.8679	2.4712	
3.115	3.48	3.1683	2.6884	2.4336	3.9142	3.7855	3.2189	
3.0798	3.0852	2.8809	3.6936	2.7885	2.2517	2.567	2.8337	
3.1474	2.5037	3.296	1.7784	2.7189	2.7455	2.717	3.1243	
2.8086	3.0713	2.3903	4.2732	3.3947	2.9238	2.9377	3.1097	
3.8157	2.5006	3.0371	3.1806	3.2012	3.8964	3.4074	2.9493	
2.6577	2.3088	3.8521	3.9058	3.2992	2.0098	2.6767	2.7128	
2.4926	2.8692	2.6814	3.1883	2.7094	2.9835	3.4678	3.2472	
2.9568	3.0845	2.9479	2.5084	3.0663	2.859	2.7098	2.9046	
3.1914	3.5643	3.2206	3.0106	5.3118	3.3198	2.7302	2.9755	
2.7003	3.393	2.3744	2.6508	2.5993	2.4624	2.1221	2.9397	
3.0316	2.9312							
C12orf40	0	0	0	0.023	0.0073	0.0218	0	
0.0069	0.0356	0.0098	0	0.0032	0	0.0243	0	
0.0047	0.0444	0	0.0362	0	0	0.0065	0	
0.0083	0.0095	0	0	0	0	0	0.0065	
0.2169	0	0	0.0018	0.0071	0	0	0	
0.0247	0.0399	0.0094	0	0.0135	0.0105	0	0.0196	0
	0.0336	0.0585	0.0103	0.0219	0.0293	0.0037	0.0515	
0.0581	0.0968	0.05	0.0374	0	0	0	0.0119	0
	0	0	0.0423	0	0.0261	0	0.0078	0
	0	0.0078	0.033	0.0222	0.0152	0.0162	0.0036	0
	0	0	0.0291	0.0206	0	0	0	0
	0.029							
C12orf42	0.1005	0	0.2481	0	0	0	0.0137	0

	0	0	0.0299	0	0	0.0871	0.0123	
0.0747	0.2858	0	0	0	0	0	0	0
	0	0	0	0	0	0	0	0
	0	0.0904	0	0	0	0	0	
0.0481	0	0	0	0	0	0	0	0
	0	0	0	0	0.097	0	0	
0.1827	0	0	0	0	0.0877	0.026	0	0
	0.046	0.0054	0	0.1059	0	0	0.063	0
	0.0378	0	0	0	0.0164	0.0336	0.1677	
0.2026	0.0305	0	0	0.0456	0	0	0	
0.0725	0							
C12orf43	2.5465	3.2163	3.0962	2.6279	2.7159	2.3248	2.8349	
2.8209	2.607	2.6522	2.3319	2.031	2.4618	3.0454	2.2391	
2.0328	3.3624	2.5178	2.9293	2.7726	2.8982	2.1642	1.9017	
3.0832	2.6298	2.7966	1.8924	2.888	2.4604	2.3813	2.5483	
2.7806	2.8273	2.1992	1.985	2.6317	2.9959	3.1828	2.5574	
2.837	2.5107	2.5727	2.3391	2.9332	2.2961	2.786	2.7442	
2.7762	1.8451	3.0671	2.4166	2.4792	2.8327	2.7399	2.8283	
3.1162	2.8012	3.4063	2.5362	3.0933	2.9149	3.0804	2.3099	
2.4693	2.7996	2.3387	2.3095	2.5193	2.5403	2.489	2.7684	
2.2307	2.9819	2.078	2.204	3.3263	2.8614	2.0177	3.1945	
2.9921	2.6239	2.1798	2.9751	2.2966	2.4127	2.8743	2.3285	
2.6623	2.3526							
C12orf45	4.5928	4.2606	4.4116	3.6427	4.3317	4.2705	4.656	
3.9415	4.2698	4.062	4.2726	3.8494	3.7949	4.728	3.4484	
3.8486	5.0437	4.2175	5.5658	4.4603	3.7051	3.3361	3.4884	
4.3883	3.9343	3.0582	5.2575	3.8151	3.9122	4.4611	3.1977	
4.159	4.4804	3.1858	3.1629	4.5966	4.6655	3.6302	3.4953	
5.1299	4.363	3.4326	3.6831	4.3182	3.1597	4.4887	4.8766	
4.1422	3.4238	4.0075	3.3519	3.6536	3.9438	4.1927	4.3722	
4.8846	4.2308	4.3346	4.6188	3.9353	4.4643	4.4291	3.2072	
4.264	4.6577	3.7931	4.4285	3.7234	4.1496	3.5483	3.9403	
3.6902	5.1503	3.5231	3.6692	3.1377	4.2183	2.8658	5.1158	
4.4138	4.7285	3.484	3.9184	3.1838	3.7002	4.1682	3.868	
3.6531	3.45							
C12orf49	4.4449	5.4642	5.4851	3.8782	3.8689	4.8758	4.7547	
4.3643	4.4148	5.1194	4.7309	3.5138	2.6883	5.087	3.7094	
4.9094	3.9823	5.2202	5.7176	4.2893	4.9257	3.6257	3.6606	
5.6601	3.9571	3.8321	3.7877	4.4694	3.8099	4.7133	3.987	
5.7626	4.5015	4.0681	3.9535	5.5576	4.2336	4.7414	4.1188	
4.0445	3.8666	3.4475	3.5555	4.1711	3.6824	3.737	5.1124	
4.3849	3.9093	4.0988	4.1743	3.9851	5.1232	4.5368	3.9456	
4.9869	4.374	4.5996	5.8724	4.0831	3.8758	5.7066	3.8188	
4.9785	4.7739	3.9669	5.627	4.9226	5.8152	3.1529	3.6888	
3.6245	4.749	3.5765	4.0748	4.5258	4.8659	3.6043	4.6216	
4.2918	4.7203	3.7513	4.7084	3.9464	3.2385	4.4872	5.2855	
5.2071	5.2863							
C12orf50	0.0997	0.0788	0.0397	0.0314	0.0085	0.0646	0.2794	
0.0311	0.0365	0.0519	0.013	0.0934	0.0354	0.1225	0.0057	
0.1072	0.0732	0.0188	0.1337	0.0935	0.0399	0.006	0	

0.0936	0	0.0568	0.0333	0.07	0.0255	0.0203	0.0359	
0.744	0.0147	0.3	0.0034	0.0183	0.0552	0	0	
0.1467	0.1686	0.0046	0	0.0752	0.0344	0.0246	0.0362	
0.0472	0	0.0396	0.0249	0.0723	0.0698	0.018	0.0548	
0.1953	0.017	0.0739	0.0221	0.0319	0.2209	0.0353	0.0608	
0.0176	0.0568	0.1416	0.1582	0.0404	0.0656	0	0.0325	
0.0252	0.0256	0	0.037	0	0	0.0603	0	0
	0.0576	0.0242	0.1506	0.0816	0.021	0.057	0	0
	0.0291							
C12orf54	0	0	0	0	0	0	0	0
	0	0	0	0	0	0	0	0
	0	0	0	0	0	0	0	0
	0	0	0	0	0	0	0.0424	
0.0152	0	0	0.0181	0	0	0	0	0
	0	0	0	0	0.0391	0	0	0
	0	0	0	0	0	0	0	0
	0	0	0	0	0	0	0	0
	0	0.031	0	0.017	0	0	0	0
	0	0.008	0	0	0	0	0	0
	0	0.069	0	0	0	0	0	0
	0							
C12orf56	0.2025	0.9329	0	0	0.2428	0.0906	1.2351	
0.535	0.0313	0.1609	0.2656	0.4704	0.1191	0.1744	0.1445	
0.0508	0.2362	0	0.2172	0	0.1125	0.2661	0.0159	
0.6074	1.2988	0.114	0.0593	0	0.1598	0.0581	0.1252	
0.1682	0.0363	0.2355	0.0296	0	0.0651	0.0836	0.0869	
0.0553	1.4146	1.5808	0.082	0.1339	0.0439	0.0586	0.2054	
0.2055	0.2802	0	0.0485	0.1475	0	0.1421	0.1942	0
	1.5836	0.1849	0.8656	0.1281	0.7232	0.0448	0.406	0
	0.065	0.3419	0	0.1642	1.228	0.1945	0.1347	0
	0.056	0.1587	0.0031	0.1399	0.0279	0	0.1132	0
	0.0498	0.0295	0.4601	0.0534	0.2885	0	0.0422	
0.0947	0							
C12orf57	4.6685	6.1006	6.3658	4.0503	7.1116	5.6754	5.1372	
5.7609	5.2277	2.8903	5.8663	5.4741	5.8798	3.3226	5.863	
6.7026	5.0427	3.4833	3.8732	6.6353	4.9638	6.2499	6.4915	
4.4545	6.3566	5.9853	5.9572	6.2324	5.4937	5.8304	6.4372	
7.3363	5.3185	6.0998	7.185	2.9034	6.1933	6.2402	7.062	
3.472	5.7806	5.9367	6.0239	5.0082	5.7504	6.4038	4.1259	
6.0151	7.4957	5.6125	6.8876	4.3227	4.2791	6.6362	5.6797	
5.7564	5.98	6.5119	5.0795	5.392	6.1672	5.4523	7.3252	
7.0699	5.6457	7.1141	4.755	7.038	6.9317	6.722	4.2776	
4.4567	5.3103	5.5294	6.2034	5.1912	4.1361	6.0145	6.5921	
6.0422	6.9971	5.901	7.0484	5.8192	6.3504	4.2837	5.7541	
6.4868	6.3638							
C12orf60	0.8995	1.5487	1.0629	1.573	1.1758	1.155	0.9826	
1.2769	1.0028	0.6229	2.1082	0.8528	1.6448	1.5618	1.5798	
1.5012	1.3037	1.4704	1.5589	1.3202	0.4636	1.4469	0.741	
1.3359	0.5162	1.3259	0.9814	1.87	0.4349	3.2859	1.1051	
0.8949	1.8348	1.3359	1.785	1.4611	1.1422	0.6167	0.9521	

1.5266	1.5266	1.4086	1.1878	1.5639	1.171	1.8038	3.021	
0.998	0.8079	2.5002	1.8898	1.0223	2.1424	1.3276	0.8774	
0.9619	2.4607	0.305	1.5269	0.3541	0.8729	1.7514	1.3335	
1.4591	0.8115	1.8016	0.8161	1.0563	1.7412	0.9698	1.8098	
1.1394	1.9195	1.4682	1.0448	1.783	1.6262	0.6444	1.0186	
1.6401	1.7857	1.6693	0.5342	1.9863	1.5725	2.119	1.5202	
1.4526	1.2578							
C12orf65	2.3067	2.8948	2.7114	2.6829	2.1073	2.6181	2.8357	
2.8043	2.1166	2.6572	2.7171	2.1171	3.6037	2.8974	2.0701	
2.043	3.008	2.9277	2.8349	2.9251	2.6905	2.5264	2.0329	
2.5246	3.0004	2.9682	1.4349	2.5083	2.2416	2.196	2.9116	
2.1715	2.9896	2.6493	2.5273	2.951	2.9865	2.5776	2.7929	
2.5314	2.3786	2.7225	2.9388	2.4088	2.6991	3.2914	2.4053	
2.6519	2.7727	3.5688	2.7921	2.7766	2.6846	3.1697	2.8785	
3.4235	2.9687	2.9492	2.6582	2.7325	2.5379	2.8499	2.3836	
2.4215	3.0396	2.4737	2.408	2.0756	1.7362	2.3778	2.4993	
2.4403	2.7662	2.3048	2.7861	2.8423	2.5046	1.7925	1.8513	
3.2448	2.5969	2.748	2.2824	2.8129	2.944	3.1791	2.3471	
2.0623	2.19							
C12orf66	2.562	2.1583	2.467	2.4271	1.8665	2.2954	2.3657	
2.2256	2.1004	2.3643	2.6618	1.8166	2.7057	2.1329	1.1043	
2.225	2.4002	2.5954	2.7432	2.2241	1.9374	2.2136	1.8073	
2.0542	1.6846	1.259	1.506	1.9342	2.2707	2.3671	2.1335	
2.1866	2.2116	2.0019	1.3819	2.4548	2.537	1.6604	1.5493	
2.7741	1.8432	1.6645	1.6585	2.5317	1.7268	1.8854	2.714	
2.5283	2.0858	2.3817	2.2336	1.7898	2.5288	1.9292	2.3617	
1.8399	2.3271	1.9603	2.3409	1.5035	2.2139	2.3876	1.9945	
2.05	1.8424	1.8071	2.3982	2.0527	1.6306	2.2917	2.4438	
1.757	2.7061	1.4954	1.6344	1.9156	1.889	1.6037	2.6883	
2.5403	2.3374	1.3246	2.2636	2.2721	0.9467	2.2452	2.0837	
1.8843	1.7031							
C12orf71	0.0813	0	0.2221	0.068	0.0838	0.0691	0.2674	
0.0329	0.4805	0.3021	0	0.0389	0.12	0	0.0244	
0.0645	0.3482	0	0.2855	0.0784	0	0.0166	0	
0.019	0.0481	0	0.3563	0.08	0	0.0412	0	
0.2222	0.1465	0.043	0.1245	0.029	0.0822	0	0.0277	
0.3862	0.1652	0	0.1543	0.1417	0.0591	0.0835	0.234	
0.0494	0.0373	0.1217	0.038	0.043	0.0832	0.1513	0.0981	0
	0.2097	0	0.2395	0	0	0	0.08	
0.2049	0	0.0767	0.4541	0.1698	0	0	0	0
	0	0.0289	0.0716	0	0.1195	0	0.0581	0
	0.0793	0.0604	0.1851	0.085	0	0	0.0571	0
	0							
C12orf73	2.9236	2.4083	2.678	2.8361	3.1564	1.7772	2.6979	
3.1241	2.6032	2.9369	2.7833	1.899	1.3692	2.5489	2.3995	
2.5284	2.4149	2.7558	3.4237	3.3008	2.7406	2.4149	2.3292	
2.5628	2.9521	2.0703	3.8856	2.8361	2.0381	3.073	2.6167	
2.813	2.3333	2.1857	2.673	2.9936	2.985	2.2669	2.2635	
2.985	2.6601	2.3693	1.8169	2.6251	2.0724	2.1765	2.6827	
3.1903	2.3613	2.9342	2.5321	2.638	2.7736	2.7412	2.9802	

3.4265	2.0536	2.6721	3.0428	2.2929	2.4484	3.2983	2.9117	
2.3971	2.5894	2.4164	2.8279	2.0627	2.3701	1.8825	2.2301	
2.1961	2.9667	2.3012	2.0919	2.3686	2.8397	2.461	3.6287	
3.2628	2.4421	2.6377	2.6964	2.6487	1.9216	2.8971	2.4217	
2.2788	2.5297							
C12orf74	0	0	0	0	0	0	0.0147	0
	0	0	0	0	0	0	0	0
	0	0	0	0	0	0	0	0
	0	0	0	0	0	0	0	
0.0029	0	0	0	0	0	0	0	0
	0	0	0	0	0	0	0	0
	0.0145	0	0	0	0	0	0	0
	0	0	0	0	0	0	0	0
	0	0	0	0	0	0	0	0
	0	0	0	0	0	0	0.0154	0
	0	0	0	0	0	0	0	0
	0							
C12orf75	3.9281	3.0102	5.0578	4.7621	5.7343	5.3709	6.4567	
3.633	5.1681	3.6127	3.9627	5.0655	7.0724	5.1751	6.0721	
4.6525	6.9875	0.5962	4.7679	4.7412	2.8647	4.5183	4.5267	
5.0387	4.6993	6.8774	5.4248	2.9346	5.6624	3.3549	5.5897	
3.6949	5.5926	4.7058	5.4375	3.956	3.7694	1.1454	1.9565	
4.7886	3.3164	5.4116	7.946	3.5567	6.7951	6.6276	3.374	
3.9598	5.8009	7.6382	4.6503	4.5389	4.214	2.0699	4.7419	
3.6897	6.6216	6.2153	1.4112	5.261	5.4253	4.3247	3.6	
5.0188	2.405	6.7761	2.6508	6.1389	3.5507	5.1293	5.9064	
5.0696	5.1983	4.4115	5.4703	0.481	4.0841	3.6978	4.4111	
4.3062	4.4533	6.0799	4.4957	4.2871	6.4256	4.8206	5.0519	
5.0242	4.3267							
C12orf76	1.6015	2.4875	1.9192	2.809	2.9333	2.7702	3.3982	
2.4995	3.7192	2.8633	3.011	3.3711	1.7177	2.6911	2.582	
1.9215	2.7428	2.75	2.4498	2.2626	2.5194	2.889	3.1624	
2.6319	2.0762	2.102	5.9003	3.1173	2.7269	3.5787	1.4468	
3.7723	3.2193	3.0984	3.2111	2.8183	2.5978	4.335	3.2719	
3.5703	3.8628	2.4338	1.6098	3.7205	2.7955	2.3858	2.9492	
3.5218	2.8556	3.4573	2.5824	2.4755	3.3226	3.0555	1.6774	
2.5728	2.7212	1.4403	2.5971	1.323	3.1726	2.8287	2.6558	
3.0393	4.4673	2.9654	3.5112	3.1027	2.7374	2.1634	2.5894	
2.7115	2.1984	3.5668	3.2257	2.0838	2.6307	3.6536	4.9495	
3.9788	3.2083	3.2179	3.186	4.0806	1.8536	2.711	2.9177	
2.3942	3.5527							
C14orf1	4.9137	4.8506	5.0712	4.2915	3.6398	5.6691	3.8944	
4.5105	4.2415	4.5484	4.5636	5.0676	4.0402	4.7322	5.1814	
5.1608	4.841	5.6092	5.1735	4.8639	5.6854	4.5627	5.0557	
4.0175	4.8079	4.6147	3.4381	4.7535	4.9645	4.3817	7.522	
4.0243	5.1434	4.3569	5.0076	5.2979	4.1416	4.5169	4.2918	
4.9422	5.284	5.1298	3.9149	5.0235	5.8002	4.5869	6.5498	
5.68	3.4947	5.9349	4.9832	4.7664	5.8462	3.6801	5.2894	
5.86	6.4221	4.9675	5.0825	5.3137	4.5523	4.9058	4.8044	
4.5841	4.8897	4.6096	4.7395	4.7509	5.9039	3.7554	4.876	

3.9102	5.3888	4.0489	4.9695	6.2156	5.7996	4.9583	5.4737	
4.8297	4.9569	4.9663	5.3168	5.1996	3.7618	5.4596	6.5304	
6.0334	5.7141							
C14orf105		0	0	0	0.0222	0.0026	0	
0.0102	0.0062	0.0142	0.0269	0.0144	0.012	0.022	0.0234	0
	0	1.4948	0.0539	0.0348	0.0903	0.024	0.0019	
0.0026	0.0076	0.0034	0.014	0.0368	0.0269	0	0.0112	0
	0	0.0132	0.0092	0.0131	0	0.0253	0.0186	
0.0055	0.0148	0	0.0013	0	0	0.0045	0.0172	
0.0393	0	0.0089	0	0.0175	0.0304	0	0.0041	0
	0	0.8782	0	0	0	0.0868	0.0515	0
	0.0276	0	0.0024	0	0	0	0.0511	
0.0082	0	0	0.0159	0.0139	0.0227	0.0239	0	0
	0.0999	0.0096	0	0.0094	0	0.032	0	0
	0.0376	0.0279						
C14orf119		4.1405	4.4349	4.3076	4.5608	3.8311	4.3155	
5.3557	4.3947	4.3076	4.2902	4.1064	4.7617	3.8906	4.5805	
4.3767	4.2868	4.4992	4.8968	4.3251	3.8592	4.1734	3.4735	
4.6015	3.811	3.9138	4.7877	3.105	4.5572	4.221	4.3301	
4.7444	4.3974	4.4527	4.3222	3.3131	4.8136	4.4032	4.5401	
3.9842	4.4357	4.2154	4.427	4.3937	4.272	4.8147	4.6674	
4.4744	4.2047	3.9462	4.9178	4.1578	4.5882	4.511	3.7376	
3.7406	3.9046	5.0062	4.1431	3.6632	4.2385	4.2948	4.2357	
3.8075	4.0422	4.0928	4.706	4.4856	4.981	3.5092	3.965	
4.5988	3.99	4.0856	4.4256	4.7053	3.8937	4.3667	3.863	
4.6813	4.0095	4.6449	4.3905	4.3153	3.9977	4.2607	4.4509	
4.6349	4.5647	4.3419						
C14orf159		2.4396	1.7671	1.4023	2.5057	2.4716	2.4802	
1.3982	3.3513	1.2394	1.4203	1.4978	2.7849	3.0864	2.1855	
1.2881	1.7667	2.8834	2.1103	2.0347	2.3094	2.0758	1.265	
2.425	2.9945	1.6187	2.3919	1.5111	1.1859	2.1289	2.6376	
3.1449	2.6791	1.8855	1.4575	1.7251	1.5725	2.6734	2.2995	
1.8997	2.583	2.5132	3.7646	2.6109	2.8235	2.8283	3.0804	
0.9592	2.8329	2.3455	2.4993	1.6716	2.2123	1.9899	2.1024	
1.7895	1.3323	3.1991	2.7279	2.3072	2.5517	2.1101	2.3872	
2.6338	1.9541	1.5752	4.0976	2.109	3.107	1.048	2.5872	
2.3329	1.475	1.7201	2.0734	1.6632	0.7481	1.928	1.8455	
2.1357	1.8887	1.7577	1.7384	2.3668	2.3329	1.5424	1.9634	
1.6985	1.271	1.0646						
C14orf166		6.9955	6.5436	6.6028	6.8391	6.2225	6.4555	
7.8247	6.7267	6.4211	6.7048	6.3987	7.0328	6.7208	6.9647	
6.9144	6.8234	7.4294	7.4481	7.1229	6.5287	6.8866	6.6358	
6.6945	6.1939	6.4508	6.8462	7.0191	6.7288	6.8682	6.4877	
6.6828	6.6214	6.6399	6.3488	6.2751	6.8592	6.9955	6.6561	
6.6012	7.1862	6.2975	6.5835	6.4193	6.7248	6.9907	7.0433	
6.498	6.5835	6.5737	8.1928	6.4326	7.0536	6.4023	6.9976	
6.8825	6.2687	6.7518	6.5719	6.3276	6.5531	6.4882	6.7249	
6.8483	6.1958	6.5956	7.068	6.9677	6.9525	6.0152	6.4563	
6.6832	6.2386	6.2106	6.7403	7.0116	6.3341	6.5751	6.2992	
6.8643	6.3411	7.7152	6.7828	6.4591	6.6873	6.6638	6.5388	

6.4587 6.6417 6.5049
C14orf169 4.0652 3.4259 3.107 3.8184 2.7774 2.4831
2.7808 3.4724 2.9213 3.5555 2.806 3.4763 2.3437 3.8042
3.1188 3.2229 3.4379 3.8694 3.6922 2.9128 3.7156 2.9769
3.4337 3.586 2.9069 2.5381 1.9716 3.7961 4.0035 2.7526
4.1138 3.7035 3.4883 2.3212 2.1404 3.7461 3.3259 2.7008
3.5562 4.1812 3.6103 2.7138 2.5779 3.3146 3.6658 3.424
2.933 2.995 2.4727 3.9325 2.8721 3.7973 2.8195 3.3803
3.5832 3.2727 2.3239 2.7648 3.269 2.1157 3.6195 3.7846
4.3803 2.8862 1.0647 2.821 3.3242 3.6012 3.1471 3.3739
3.5754 3.2994 3.3761 2.7043 3.0058 2.9402 3.6065 3.1034
3.9354 3.1772 3.1503 3.0165 3.0706 3.1096 3.2361 3.0226
3.2245 3.5069 2.9906
C14orf177 0 0 0 0 0 0 0
0 0 0 0 0 0 0 0
0 0 0 0 0 0 0 0
0 0 0 0 0 0 0 0
0 0 0 0 0 0 0 0
0 0 0 0 0 0 0 0
0 0 0 0 0 0 0 0
0 0 0 0 0 0 0 0
0 0 0 0 0 0 0 0
0 0 0 0 0 0 0 0
0 0 0 0 0 0 0 0
0 0
C14orf178 0 0 0 0.1454 0.1378 0.3204
0.1567 0.2232 0 0.1779 0 0.0462 0.0705 0
0.1547 0 0.3925 0.2739 0.2303 0.3523 0.4663 0
0.1921 0 0.0534 0.1703 0.1071 0 0.0776 0.1666
0.1685 0.8805 0.1067 0.1048 0.0474 0.0696 0.0589 0.1184
0.0725 0 0.142 0.1083 0 0 0.1328 0.0957 0
0 0.2148 0.4363 0 0.18 0.2953 0.0816
0.2431 0 0 0.1229 0.3976 0 0.0719 0.2349
0.3397 0 0 0.0528 0 0.1806 0 0
0.0974 0.124 0.2419 0.0372 0.6934 0.1189 0.0866 0.3147
0.2823 0 0 0.1458 0.1895 0 0.253 0 0
0 0.2471
C14orf180 0 0 0 0 0 0 0
0 0 0 0 0 0 0 0
0 0 0 0 0 0 0 0
0 0 0 0 0 0 0 0
0 0 0 0 0 0 0 0
0 0 0.0146 0 0 0 0 0
0 0 0 0 0 0 0 0
0 0 0 0 0.0204 0 0 0
0 0 0 0.034 0 0 0 0
0 0 0 0 0 0 0 0
0 0 0 0 0 0 0 0
0 0
C14orf2 7.4761 7.7486 6.6151 7.6132 6.7465 6.9356 7.652

7.66	6.8996	7.1009	7.7152	7.7486	6.8948	7.1501	6.9066	
7.3834	6.8433	7.8569	7.6433	6.4376	7.4802	6.8803	7.176	
7.3239	6.7989	6.3633	7.6294	7.4231	7.2004	8.0305	7.4521	
7.173	6.978	6.6517	6.0342	7.679	6.8594	7.3201	6.9961	
8.0245	7.3756	7.1527	6.7793	6.8013	7.4133	7.3834	7.7065	
7.1123	6.1554	7.826	6.4751	7.66	7.1702	6.611	7.4114	
7.0955	6.5029	7.3622	7.1928	7.0688	7.1734	7.6496	7.6994	
6.7527	7.4459	6.6596	8.2655	7.6425	6.7941	6.611	6.9911	
6.5251	7.1708	7.1881	7.5709	5.7102	7.277	7.1781	7.162	
6.9911	7.7768	6.7425	6.9357	6.9488	6.4535	7.075	6.9933	
7.0916	6.9685							
C14orf28	1.3406	0.8797	0.998	0.6796	0.4556	0.4456	2.0689	
0.9921	0.8296	0.8736	0.7422	0.6791	1.3118	1.1394	1.0022	
0.994	2.1499	1.3089	1.3247	1.0068	1.2485	0.7312	0.9405	
0.8693	0.3284	1.0472	0.7243	1.3485	0.8951	0.6171	0.59	
0.8399	0.8487	1.2976	0.631	0.7163	1.1451	0.8916	0.5179	
1.2123	0.8797	0.6453	0.7675	0.924	1.3727	1.2994	1.117	
0.988	1.095	1.8231	0.9112	1.1405	1.2165	0.8488	0.9673	
1.1431	1.517	0.7061	0.6416	0.6651	0.6108	0.7923	0.9455	
1.0826	1.4842	1.6844	1.4701	1.3138	0.1624	0.9064	1.1011	
0.9217	1.0833	0.627	1.2087	1.2039	0.7221	0.7167	1.1659	
1.2576	1.1071	0.7167	1.2188	0.7452	0.6521	1.2094	1.7653	
1.1039	1.6466							
C14orf37	0.8454	1.7032	0.727	0.1103	0.1422	0.4597	0.6684	
1.0704	2.1836	2.9152	2.7547	1.3262	0.2184	1.1336	2.6997	
2.065	1.1072	0.1499	1.5087	1.2786	0.56	0.8511	0.9021	
0.8198	1.5556	1.9203	1.1785	2.1524	0.5211	2.8322	0.7334	
0.8437	2.6669	3.8532	1.4328	1.9987	0.6804	2.8008	1.6517	
2.5478	0.1661	0.1796	1.3299	1.1504	0.2958	0.0956	2.3022	
0.9572	0.1041	0.7677	0.8684	0.751	0.8628	0.0547	0.5429	
0.6106	1.2525	1.395	2.5344	0.735	1.3384	1.6419	1.5167	
1.6343	1.8876	2.4624	3.282	2.9274	1.5408	0.2984	2.2113	
0.501	0.4811	0.1322	2.3113	2.3892	0.8871	1.2058	0.4663	
1.5717	1.002	0.4495	2.582	1.0555	0.2709	0.4082	0.9927	
1.7548	1.9822							
C14orf39	1.0668	0	0	0	0.16	0	0.0127	0
	0.3901	0.5062	0	0.0782	0	0.9801	0.1448	
0.0608	0.0282	0.0247	1.7242	0	0.0538	0	0.175	
0.0194	0.0602	0.3612	0	0.0305	0.0118	0.2311	1.938	
0.1596	0.0939	0.3361	0.0288	0.6702	0.0061	0	0.992	
0.0107	0.3999	0	0	0	0.0176	0	0.0782	0
	0.0115	0	0	0.3299	0.3472	0	1.8338	0
	0	0	0.0283	0	0	0	0.6792	
0.2872	0	0.024	0	0.0313	0.0621	0	0.4497	
0.8805	0.1472	0.2527	0.7285	0.2662	0.0078	0.0232	0.0988	0
	0.3295	0.0683	0	0	0	0.4213	0	
0.0264	0							
C14orf79	1.984	2.2156	1.224	1.5407	1.7844	1.123	1.5565	
2.3528	1.0866	1.0667	2.3059	1.7681	2.1989	1.4739	1.7268	
1.1287	2.2727	1.5722	0.8715	1.2018	2.1497	2.0471	2.0872	

1.4442	1.4024	2.6659	1.7128	2.1663	2.1112	1.8132	2.571
2.3747	1.3788	1.1586	1.2379	1.2688	2.7104	2.6241	2.0424
2.2742	2.6673	2.1825	1.28	1.1465	2.176	2.1919	1.4533
2.0277	1.8939	2.0321	1.4502	2.1775	1.4942	2.329	1.5425
1.2008	1.6986	0.7726	1.3043	0.7137	2.2077	2.1035	1.6647
1.3089	1.8183	2.5684	2.0291	2.1929	1.1674	1.8268	2.495
1.6979	1.8346	2.2809	1.4299	0.6916	1.7378	2.0404	1.4019
1.5886	1.4412	1.9206	1.4889	1.6099	2.3768	1.3406	1.6571
1.4847	1.663						
C14orf80	2.3966	2.131	2.4803	0.7929	2.5137	1.4693	1.2478
2.7029	1.1355	1.4652	1.7563	0.931	2.3555	0.9466	1.8402
1.2834	2.5563	1.3615	0.8861	1.8796	3.0734	2.4311	1.7008
1.9112	1.1479	0.7305	0.7305	2.3714	2.0398	1.1906	3.3666
3.6095	2.2272	2.1273	0.9639	0.8798	1.8718	2.2852	1.9338
0.9221	2.4403	0.8872	1.7696	1.8921	1.6761	1.8063	0.8771
1.6969	1.782	1.3691	1.1788	1.0903	1.331	1.7909	2.4953
2.109	1.6805	3.3482	1.866	3.0668	3.0675	2.5519	2.9262
1.7125	1.8686	0.7225	2.1929	0.949	1.8588	2.5719	2.2692
2.437	1.2047	2.0692	1.4098	0.935	2.3781	0.8351	2.1427
2.4097	2.3524	0.9862	0.8883	1.8181	3.4267	2.6996	1.4227
2.7264	1.9469						
C14orf93	2.1647	2.1155	1.9658	1.9803	1.6083	1.9187	1.8759
1.5279	2.1797	1.9663	2.4332	1.8846	1.4393	2.1656	1.4058
1.719	2.6591	1.9737	1.8981	2.2503	1.5358	1.2211	1.4996
1.69	1.8506	1.556	3.107	2.337	1.3107	1.9483	2.7299
1.5262	1.8421	2.0081	1.1428	2.2291	1.8239	1.9013	2.3167
1.8426	2.5786	1.3089	1.9489	2.8504	1.5762	2.375	2.0367
1.9275	1.9994	2.2527	1.7292	1.9868	2.2344	1.857	2.4279
1.5834	2.235	1.5211	1.8018	1.9227	2.0011	1.9061	2.316
1.9108	1.8558	1.4975	2.3295	1.866	1.2101	2.205	1.6231
2.1021	1.8467	1.6993	2.2476	2.0563	2.101	1.18	2.265
2.3665	2.6793	2.2633	0.8722	1.7953	1.9666	2.3278	1.9691
1.9774	2.1686						
C15orf39	3.7876	4.2125	3.6557	2.715	3.2691	2.8247	3.1129
3.7319	2.853	3.5	3.7392	2.5393	4.1146	2.3312	2.7787
4.5371	2.1626	3.3619	3.2618	3.3318	3.4663	2.9212	3.2535
3.3593	2.8885	1.4516	0.7333	3.1823	2.7636	3.4559	1.8336
3.7084	2.5301	2.6291	2.7339	2.321	3.1755	3.8314	3.9019
2.1754	2.9373	1.5685	2.6936	3.2117	2.6931	3.14	2.5765
3.4002	2.7529	1.528	3.248	1.6232	2.2682	4.4291	2.207
3.6637	2.1552	3.4823	3.6283	3.0857	3.0627	3.7608	3.6921
2.7494	2.9723	2.7032	3.2889	1.9122	4.0998	2.9755	1.8893
2.9957	3.5437	2.7852	2.1701	1.5074	3.6987	2.7064	3.5829
3.4106	3.48	1.2991	4.7687	2.6998	3.3447	2.9499	2.8534
3.3061	2.5412						
C15orf40	2.878	4.4553	3.5053	3.5924	3.8778	3.4829	3.8617
3.6288	2.9329	3.0641	3.3162	3.1835	3.6082	3.4721	2.7875
3.7277	3.3176	3.944	3.9521	3.0792	3.5345	3.1047	3.5811
4.0433	3.5586	2.9948	3.1091	4.078	3.369	4.5328	2.9768
3.9735	3.8476	3.0261	3.3218	3.7056	3.877	3.2091	3.6994

3.3331	3.388	3.2795	3.1257	3.4526	3.9118	3.2816	3.6319	
3.6068	3.355	3.8617	3.263	3.848	3.8522	3.8019	2.9425	
3.5354	2.896	3.1422	3.056	2.9761	4.1901	4.0064	3.1831	
3.1701	2.9512	3.2494	3.2696	3.242	3.7488	3.2424	2.69	
2.7849	4.1026	3.4833	3.75	3.3844	3.746	3.4503	3.5693	
3.8546	3.5729	3.1418	3.8108	3.1354	3.8657	3.6423	2.8916	
3.3284	3.097							
C15orf41	3.0666	3.3538	2.7784	3.2174	2.3999	2.2808	2.7839	
2.9162	2.8296	2.3003	2.4613	2.6335	3.4238	3.3992	2.1602	
3.7482	3.2154	3.0075	2.6249	2.3591	2.4967	2.8901	2.246	
2.7476	2.9856	3.2464	1.9166	2.0718	2.3341	2.3116	3.6163	
2.5757	2.5824	2.2952	2.6302	2.6069	3.3553	1.4316	2.6077	
3.5819	1.7316	3.3236	2.2533	3.4892	2.4361	3.725	3.1956	
3.248	2.1927	3.6551	2.8782	2.115	2.9048	3.8686	3.3948	
3.3486	2.6766	1.6288	3.2443	1.8328	1.7746	2.7809	2.3647	
2.6139	3.6155	2.7293	3.8133	3.2689	2.4305	2.0064	2.474	
2.9228	2.7392	2.7631	3.5672	3.0138	2.4399	2.7902	2.8447	
1.9905	2.5937	2.2771	3.0636	1.9165	2.7338	2.8531	2.213	
2.379	2.3513							
C15orf48	0.33	0.9496	0.8106	3.6236	4.4777	0	3.047	
2.3526	0.7592	0.3935	0.1496	6.6981	4.8317	0.1598	1.0586	
3.4493	0.6582	1.0869	1.4976	2.1527	0.6874	0.5505	0.2261	
0.4166	0.6644	4.0015	0.1954	0.4144	0	1.0388	1.0929	
4.8003	0.1511	0.2226	0.4249	2.0924	0.6538	0	0.1562	
0.117	0.9665	2.6597	1.2268	0.4787	2.0792	6.4331	0.3294	
0.2485	0.7091	0.2507	3.1719	0.5738	0.5262	0.0993	7.3825	
0.2132	0.5453	4.98	0.9666	3.2252	0	0.8998	0.3072	
0.4568	0.1497	5.3033	1.0086	2.8896	0.5139	0	2.6756	
0.1568	2.0078	2.9792	0.2934	0.0835	0.1593	0.3785	0.6775	
0.7032	0.125	2.8074	2.4353	1.0711	0.2691	0.2792	0.5655	0
	0							
C15orf52	4.0668	3.2331	2.0104	2.7218	4.4799	3.6971	2.1881	
2.7892	2.2962	2.5671	3.728	3.5466	4.6668	3.1641	4.7442	
3.7095	3.8758	2.8582	2.4852	3.8979	3.356	3.0809	4.5082	
2.0948	3.6945	4.6811	1.9322	0.7682	4.4621	2.3197	0	
2.4815	2.1835	2.3048	4.72	2.3418	4.7362	2.3384	3.4891	
2.7904	1.9942	4.4172	4.0685	3.9639	4.7126	2.7506	2.579	
3.3185	4.9424	2.3692	3.2648	3.5466	1.5294	2.8033	0.6463	
2.1675	4.4038	2.3503	2.2983	2.5617	4.1327	2.9392	3.0694	
0.8658	2.0703	5.3243	1.9574	3.545	2.7605	4.1184	3.2452	
3.7101	2.9205	3.5785	4.0487	1.0696	2.2258	3.6706	3.0095	
2.0382	1.4954	4.9751	4.4564	4.1514	4.4303	2.4067	3.8248	
3.2103	3.7154							
C15orf53	0	0	0	0	0	0	0	0
	0	0	0	0	0	0	0	0
	0	0	0	0	0	0	0	0
	0	0	0	0	0	0	0	0
	0	0	0	0	0	0	0	0
	0	0	0	0	0	0.0195	0	0
	0	0	0	0.0419	0	0	0	0

	0	0	0	0	0	0	0	0
	0	0	0	0	0	0	0	0
	0	0	0	0	0	0	0	0
	0	0	0	0	0	0	0	0
	0							
C15orf57	2.9789	3.561	2.7472	3.0048	3.1838	3.3023	3.0938	
3.3928	2.9403	2.3493	2.7031	2.7968	2.7162	3.2926	2.0592	
3.3344	3.1637	3.0946	2.5853	2.9945	2.9569	2.9545	2.8987	
2.5241	3.3001	3.2276	3.6649	3.0322	3.3405	3.1875	3.0324	
1.9035	2.2602	2.7316	3.3831	3.091	3.7775	2.3436	2.2729	
3.5552	1.5593	3.027	3.1313	2.9998	3.4751	3.6808	3.6188	
3.4521	2.214	3.1733	2.9813	2.5404	2.8938	3.1737	3.3729	
2.8515	3.4679	3.1019	2.3634	2.8841	2.5623	2.7779	2.5683	
2.849	3.4439	2.8342	3.6241	3.4701	2.5712	3.1393	2.4438	
2.5651	3.6036	3.0386	3.0682	2.4857	2.3211	3.4735	2.9652	
2.5386	2.7467	2.6086	3.888	3.2121	3.5261	2.4643	3.3045	
2.9762	3.2754							
C15orf59	0.5477	0	0.9493	0	0.1094	0	0.2524	
0.0694	0.9463	0.2424	0.0865	0	1.0593	0	0	
0.1312	0	0	0	0.1219	0	0.18	0	
0.0545	0.315	0.066	0	0.1138	0	0	1.2166	0
	0.0801	0.1322	0.1542	0.1066	0.3494	2.4117	0.1342	
0.6213	0	0	0	0.0821	0.4694	0	0.1162	0
	0	0	0.3848	0	0	0.4795	1.2384	
1.4966	0	0	0	0	0.3027	0	0.9158	0
	0.9244	1.2358	0.3309	2.0551	0.1611	0	0.1324	0
	1.4688	0	0.0466	0	0.0512	0	0.0058	
0.6127	0	0.4656	0.2788	0.0759	0.0139	0	2.2228	
1.7067	2.183							
C15orf61	2.7205	3.1687	3.3332	2.1328	2.8618	2.4467	2.7973	
2.6972	1.9091	2.5483	2.1676	2.3552	2.3851	1.9014	1.7874	
2.7271	2.3127	2.7641	2.2478	2.9133	3.0519	2.2272	2.3936	
2.8053	1.2602	1.7757	2.3804	3.0687	2.332	2.4159	1.8611	
2.9046	1.9908	2.29	1.3103	2.6516	3.0853	2.9727	2.5365	
1.8588	2.4597	1.7623	1.4207	2.1002	2.1141	2.0666	2.5895	
2.1496	2.6873	2.8798	2.2093	1.8025	2.2701	2.6982	2.7719	
3.2981	2.3233	2.051	2.0306	2	2.7723	3.3299	2.9037	
3.0147	2.555	2.6193	2.7073	2.5025	3.5954	1.7758	2.177	
2.1292	2.0823	1.8855	2.9969	3.2568	3.1795	2.9324	2.6424	
3.5399	2.6338	1.9192	3.3059	2.1562	2.278	2.1695	2.0144	
2.0484	2.0647							
C15orf62	0.6289	0.5288	0.5825	0.4016	0.3362	0.5463	0.1971	
0.4647	0.3427	0.2313	0.1536	0.1841	0.275	0.5131	0.182	
0.1763	0.3921	0.7417	0.3478	0.1958	0.7498	0.334	0.1892	
0.4274	0.3784	0.2094	0.1883	0.5408	0.4028	0.263	0.451	
0.2292	0.209	0.0204	0.3321	0.2946	0.2414	0.0766	0.0898	
0.1188	0.0949	1.0353	0.2649	0.4654	0.9631	0.3285	0.1803	
0.4465	0.5241	0.3606	0.2884	0.2964	0.1593	0.261	0.7024	
0.7131	0.2761	0.3201	0.3896	0.2322	0.8904	0.315	0.5624	
0.2804	0.2848	0.1817	0.7969	0.2313	0.119	0.2784	0.0701	

0.3962	0.2779	0.1143	0.4068	0.3059	0.1927	0.3839	0.3449
0.2463	0.2421	0.2949	0.1436	0.4793	0.8185	0.4088	0.2317
0.5658	0.5572						
C15orf65	1.4153	2.026	1.1995	2.4021	1.6835	0.7338	2.4201
0.983	1.0211	0.7985	1.0375	1.178	1.4744	1.4486	1.1105
1.2838	1.2348	1.3918	0.5326	1.2955	0.7413	1.1159	0.5442
1.6835	1.8617	1.9733	1.5438	1.7358	0.6545	1.3487	3.8492
1.4806	1.4472	1.7537	1.3135	1.4965	3.4667	1.178	0.9732
1.3077	1.4346	2.1949	0.9406	0.5741	0.8052	1.6736	2.1058
1.0653	0.7985	2.3223	2.3945	1.2327	1.4388	3.2268	2.3247
1.3838	1.6633	1.0418	1.4332	1.7434	0.5521	1.3141	0.5501
1.8504	1.0157	3.189	1.4437	2.3099	1.8787	1.1433	0.7214
0.9873	0.9963	0.7338	1.0302	1.1187	1.9003	1.645	1.0252
1.0045	0.7289	1.3448	2.5837	1.2887	2.7574	1.2105	2.0764
1.6083	1.7384						
C16orf45	0.6883	0.8034	1.5808	1.5877	3.4435	1.884	1.3009
1.3574	2.451	0.4075	3.0359	2.027	3.6233	1.9365	5.3786
1.1153	3.3194	1.7635	0.751	2.2656	0.4943	1.4859	3.3951
1.5751	4.745	4.061	3.5479	1.0613	1.1536	2.0474	2.6462
0.6708	2.5417	2.9686	1.4619	0.952	1.705	1.4078	2.8173
0.9266	0.9223	3.6543	4.9314	2.4277	3.629	4.2914	0.547
1.4854	3.4495	0.6386	1.6086	3.1211	0.7196	1.3559	2.045
0.2099	0.2469	1.2581	0.2012	2.0548	2.3963	0.7765	1.7073
1.7767	1.8927	4.5276	1.1125	4.9278	2.4534	2.5612	2.6932
2.4652	1.3052	1.7587	3.6854	2.4625	1.1821	2.4979	0.9729
0.9971	2.5513	2.9263	5.8412	2.9337	4.2903	0.7743	3.8793
2.566	3.0404						
C16orf46	1.2637	0.9706	0.8289	1.3304	0.9425	1.3988	1.1151
1.1555	0.7606	0.6983	0.5348	0.8676	0.8958	0.9401	1.1402
0.5914	1.3715	1.0282	0.8077	1.5053	1.1032	0.9956	0.8039
1.5082	0.7399	1.2432	2.4734	1.2317	0.561	0.7219	0.9034
1.4192	0.6677	0.723	1.3926	0.7488	1.1145	1.3234	0.4612
1.3668	1.7424	1.0334	0.7138	0.3332	1.25	1.5897	0.825
0.6861	0.8429	1.1334	0.8832	1.0934	1.2571	1.2287	1.0639
1.0066	1.7451	0.7756	0.7981	0.5744	0.5912	1.1416	1.2921
1.2812	0.9884	0.6111	0.8287	0.7906	0.5994	1.205	0.6762
0.883	0.4328	0.6438	1.3545	1.3757	0.8347	1.5289	1.0594
1.2914	0.7695	1.1219	0.7872	1.4121	1.0652	1.0145	0.7493
0.4718	0.6671						
C16orf52	2.4744	2.8874	2.9601	4.6382	5.8348	4.7025	4.4499
5.1505	3.2574	2.9916	3.1236	4.2141	3.6975	4.324	4.825
2.4377	3.7831	2.4086	3.1993	3.3834	3.7474	4.8881	4.0914
4.8412	5.498	3.1562	5.6983	3.0192	4.3285	3.0337	3.4922
3.7525	4.1451	4.0534	5.0573	3.35	5.1448	3.4026	4.3632
4.8272	4.0056	4.3034	4.8144	3.8763	4.2753	3.2699	2.8843
3.7189	5.478	3.4145	3.8289	4.2554	3.5622	4.138	2.1821
2.648	4.615	3.0516	3.2652	3.5696	4.7583	2.7592	4.7885
3.6363	2.3575	3.434	3.2018	2.8141	3.0449	5.195	4.9147
3.7997	3.2009	5.0038	4.4842	3.8606	3.9676	3.8275	3.8806
4.5275	3.6694	4.0469	5.1136	4.7731	4.5216	3.2812	3.4837

2.944	3.0192							
C16orf54	0	0.1628	0.0323	0.369	0.2862	0.0261	0	
0.0251	0.0931	0.1269	0.0617	0.0026	0.0146	0.1587	0.0139	
0.0144	0.0362	0.3813	0.1962	0.0331	0.1746	0.0229	0.0519	
0.1049	0.0422	0.0439	0.1911	0	0.0251	0.0085	0	
0.1028	0	0	0.0402	0.06	0.008	0	0	
0.0068	0.6057	0.0214	0.0228	0.1251	0.0946	2.1353	0.1054	
0.1062	0.0228	0	0.1237	0.0115	0.1879	0.078	0	
0.1369	0.038	0.0562	0.078	0.0714	0.0476	0.0443	0.0315	
0.0431	0.043	0.0469	0	0.0311	0.0666	0.0565	0.0751	
0.0317	0	0.0848	0	0.2088	0.064	0	0	0
	0.0667	0.0387	0.121	0.0363	0.0962	0.1287	0.0805	0
	0.0293							
C16orf58	5.0758	4.8593	4.7344	3.4696	4.7449	3.4726	4.3367	
5.3812	4.7367	4.0229	5.4429	3.9008	4.3913	3.9411	4.8701	
4.2282	4.4941	4.2523	3.9395	4.8208	4.2627	5.3216	4.7716	
4.8924	4.7812	4.5037	4.5297	4.8628	5.257	4.1959	4.7638	
5.1199	4.7322	5.841	5.4374	3.3387	5.2916	5.7796	4.7929	
3.9338	5.3516	4.7299	4.7381	4.5541	4.1518	4.1999	4.041	
5.0059	4.8811	3.065	5.7056	3.8758	3.7629	5.2662	4.6656	
3.7186	4.6481	4.0792	4.9069	4.0713	4.3052	5.0187	4.4716	
3.9945	4.5861	4.7854	5.3223	4.575	4.1981	5.3815	4.6605	
4.3052	4.9574	5.1815	3.554	4.3113	4.4269	5.4316	5.4054	
5.0951	4.3109	4.4323	5.041	5.3167	4.577	4.1115	4.8492	
4.8316	5.0958							
C16orf59	2.6755	4.1778	2.9467	2.4674	2.913	1.9118	1.6507	
3.6607	2.3083	2.2443	2	1.9862	2.7856	2.4808	2.8395	
1.8091	2.8373	2.7593	3.3526	2.795	2.2292	3.7752	2.8125	
3.3682	2.4596	1.3902	0.7548	2.7132	2.8736	2.8399	3.1135	
4.114	2.9549	2.1947	2.4262	2.1913	2.8533	2.2007	2.5076	
1.864	2.6886	2.0602	2.9773	2.3758	2.5836	2.5302	2.1007	
2.6669	1.8383	2.5031	1.9124	2.6245	1.7908	2.4473	2.7522	
2.8592	1.1152	3.3167	2.9267	3.4955	3.8537	3.3246	2.7843	
2.2343	2.7569	0.6951	3.0049	1.748	3.0437	3.2516	2.5619	
3.1392	2.9157	2.7294	2.3291	1.8786	3.2016	2.3611	2.4838	
2.4757	3.0263	2.0632	1.617	1.9345	3.757	3.0721	1.2415	
2.622	2.2143							
C16orf62	3.796	2.8529	3.1043	3.858	3.64	3.6992	3.6279	
3.2869	3.8118	3.5553	1.8233	3.6455	3.8872	3.6377	3.991	
3.5313	2.0037	4.9622	4.7171	3.0145	3.8263	2.257	4.1806	
2.8444	3.1585	1.6023	3.9396	3.9468	3.9633	2.6021	4.2225	
3.7873	3.469	3.9682	4.0006	3.5535	3.3937	4.121	3.25	
4.0561	3.6568	4.2974	3.6429	3.8541	4.6482	4.2555	3.1649	
2.408	3.5205	4.1382	2.2647	3.7449	3.4893	3.818	3.2023	
3.4528	2.7956	1.3497	3.2121	1.0258	2.0474	1.8276	4.0587	
3.3563	3.8885	4.1027	3.6544	3.979	3.8993	3.8509	3.9569	
3.0713	2.5062	3.6875	4.2648	1.8004	3.2883	4.2465	4.2529	
3.9565	4.1084	3.7281	3.9732	3.7913	3.7307	3.53	4.9942	
4.6609	4.6668							
C16orf70	2.9272	3.2209	3.7301	2.9485	2.9908	3.2533	2.6016	

3.2284	2.9059	3.0633	2.8122	2.8902	3.5607	3.6813	2.9951	
3.9118	2.8593	3.3392	2.7544	3.9576	3.3347	3.201	2.5869	
3.1587	3.0801	3.3392	2.5223	3.2742	2.3519	2.9289	3.3793	
3.1966	2.9202	2.7413	3.0538	3.2888	3.4796	2.9332	2.4189	
2.7221	2.636	3.212	3.0201	3.5473	3.4413	3.2303	3.2355	
3.0709	2.4378	3.3095	2.768	2.9935	2.887	2.7986	3.3677	
2.6872	3.0985	2.9106	3.541	3.1256	2.8839	3.2196	3.0073	
3.0471	2.7445	2.6014	2.661	4.1611	3.6694	2.9909	3.1498	
3.1595	3.3259	2.4052	3.2225	3.2948	3.0631	3.024	2.8475	
2.8907	2.7275	2.7241	2.8452	3.086	2.7462	2.8542	3.1192	
3.0284	3.1053							
C16orf71	0	0.173	0.0461	0.2036	0.137	0.2685	0.1387	
0.5159	0.0786	0.0699	0.113	0.0518	0.256	0	0.1973	
0.1443	0.0922	0	0	0.4411	0.2012	0.0681	0.0953	
0.4264	0.1436	0.4087	0	0.3857	0.2198	0.116	0.1081	
0.5034	0.1238	0.305	0.1638	0	0.1351	0.1832	0.0706	
0.1104	0.2624	0.1567	0.0457	0.0314	0.0579	0.142	0.0492	
0.1242	0.1283	0.0803	0.0844	0.0363	0.1026	0.1295	0.2185	
0.0367	0.0783	0.1871	0.1379	0.0466	0.135	0.1423	0.0744	
0.2717	0.2232	0.4598	0.1512	0.1431	0.1333	0.4393	0.3094	
0.0871	0.0502	0.0278	0.1053	0.0213	0.0773	0.3037	0.1236	
0.1718	0.0171	0.058	0.0795	0.1712	0.1158	0	0.1032	
0.0638	0.0963							
C16orf72	3.8403	3.7947	3.8866	4.5124	4.2662	3.9734	4.4176	
4.6248	3.9541	3.7984	3.9767	3.5397	4.3241	4.4746	3.8907	
4.0441	4.2229	4.0154	4.0225	3.5755	4.0449	4.2866	4.07	
4.1125	4.551	4.4777	4.7883	4.1256	4.2331	3.415	3.8121	
4.0107	4.0409	3.8739	4.2982	4.2116	4.4226	3.829	4.051	
4.6259	3.377	4.2289	3.4504	3.8238	4.2662	4.4934	4.2101	
4.379	3.828	4.5701	3.9003	4.0428	3.6529	4.2301	4.1335	
3.5387	4.2456	4.2316	4.3716	4.2434	4.0969	3.9424	3.7943	
3.7445	3.7769	4.2767	4.1675	4.5903	4.0509	4.2793	4.4163	
3.8946	4.0337	3.8299	4.0426	4.0402	3.7777	4.2879	3.7664	
3.5813	3.4823	3.9584	5.4384	3.983	3.9784	3.6793	4.2008	
3.7261	3.9647							
C16orf74	2.398	2.0686	2.3015	1.6729	0.9771	1.1715	0.5924	
1.0434	1.5947	1.1007	2.3819	0.537	1.5017	2.0032	0.7284	
0.5587	4.6372	0.8336	2.3804	0.8194	1.6051	0.8901	1.6254	
1.069	1.359	0.9052	1.093	2.6274	0.8131	2.472	2.041	
0.6109	0.8972	0.437	0.9139	0.5251	1.9672	3.2022	0.6886	
0.504	3.4988	0.2584	1	0.815	2.0272	1.6871	0.912	
0.9064	0.367	1.5116	1.1014	0	1.538	2.2826	1.7326	
2.4356	4.507	1.3087	0.5555	0.3166	1.3633	1.9684	0.4905	
1.1413	3.1048	0.6046	2.661	0.4726	1.2965	1.5524	1.502	
0.7039	1.5963	0.2108	0.6703	0	2.1724	1.8579	1.8096	
2.1911	0.5955	0.9948	0.6227	0.5561	2.1805	2.2148	1.7827	
2.8851	2.0089							
C16orf78	0	0	0	0	0	0	0	0
	0	0	0	0	0	0	0	0
	0	0	0	0	0	0	0	0

	0	0	0	0	0	0	0	0
	0	0	0	0	0	0	0	0
	0	0	0	0	0	0	0	0
	0	0	0	0	0	0	0	0
	0	0	0	0	0	0	0	0
	0	0	0	0	0	0	0	
0.0333	0	0	0	0	0	0	0	0
	0	0	0	0	0	0	0	0
	0							
C16orf86	1.0432	0.6123	0.3864	0.8546	1.5628	0.7612	0.2126	
1.105	0.4084	0.6733	0.9167	1.0901	1.2138	0.4287	0.797	
0.6205	1.3839	0.8773	0.5638	1.488	1.7173	1.4303	0.7226	
0.4305	0.3904	0.2306	2.2367	0.3404	0.8452	0.7705	1.1442	
1.2905	0.8314	2.3009	0.137	0.6439	1.4066	1.8489	0.9053	0
	1.9052	0.6248	0.443	1.1292	0.4252	0.2365	0.4545	
0.9505	0.6603	1.1181	0.7078	0.8295	0.6523	0.7906	1.4756	
1.0502	1.181	0.15	1.3458	0.2655	1.3375	1.6498	0.2385	
0.636	0.8485	1.437	1.5817	1.1145	0.7251	1.2678	1.1554	
1.4647	1.1475	1.5836	1.578	0.4402	0.0947	0.975	1.0325	
1.4351	0.1126	1.8651	0.8505	1.38	0.1298	0.898	1.3067	
1.7215	2.0504							
C16orf87	2.325	3.229	2.5609	3.3671	3.6713	2.839	3.4687	
2.6003	3.2369	2.6474	2.707	3.0452	3.6985	3.6839	3.206	
1.9546	2.2652	2.1356	2.9855	2.8371	2.8375	3.5784	2.9076	
3.4768	3.566	3.3797	5.36	3.4856	3.214	2.9184	3.5828	
2.8697	2.2877	2.9908	3.2713	3.2383	3.0647	3.2171	2.713	
3.3868	3.5838	3.199	3.187	3.0793	4.0191	3.4893	2.7095	
3.2703	2.5669	3.1183	3.0608	3.524	2.7289	2.586	4.6928	
2.9044	3.6621	2.8709	3.0463	3.1987	2.8406	2.8884	3.7537	
2.9419	3.6155	3.3524	3.7432	2.1805	2.5566	2.473	3.0555	
3.2231	3.2162	3.1511	3.3103	2.8613	2.6852	3.5193	3.2775	
2.985	2.682	3.8411	1.9968	3.5277	3.3536	3.1671	2.8037	
2.8421	2.7915							
C16orf89	0.2488	1.379	0.1914	0.0649	0.0555	0.0557	0.0524	
0.0654	0.3948	0.0728	0.2283	0	0	0.1351	0.0214	
0.1601	0.0751	0.2584	0.2773	0.1148	0	0.1241	0.3561	
0.0243	0	0.0715	0.2341	0.1891	0.1403	0.1086	0.0231	
0.0353	0.0686	0.2247	0.0861	0.0339	0.0386	1.5004	0.0579	
0.2566	0.8317	0.1472	0	0.1275	0	0.0788	0.3094	
0.1462	0.1514	0.249	0.2155	0.2379	0.1974	0.258	0.0616	
0.0659	0.0309	0	0.0345	0	0.1045	0.0063	0	0
	0.5808	0.0081	0.245	0	0.0966	0	0.0178	
0.1177	0.1227	0.0251	0.0363	0.5003	0.3423	0.5835	0.5094	
1.0513	0.6325	0.0348	0.74	0.1931	0.1337	0.2448	0.2221	0
	0.5908							
C16orf90	0	0	0	0	0	0	0	0
	0	0	0	0	0	0	0	0
	0	0	0	0	0	0	0	0
	0	0	0.194	0	0	0	0	
0.0826	0	0	0	0	0	0	0	0

0 0 0 0 0 0 0 0
0 0 0 0 0 0 0 0
0 0 0 0 0 0 0 0
0 0.0641 0 0 0 0 0 0
0 0 0 0 0 0 0 0
0 0 0 0 0 0 0 0
0
C16orf91 3.449 4.4166 3.4446 4.4254 4.6919 3.4558 3.2804
4.3491 2.7458 2.8022 3.7156 3.7896 3.6426 3.2951 3.4612
2.9507 3.6821 2.1875 3.4712 3.9898 3.641 4.5275 3.5895
4.2139 3.3817 3.4456 5.1075 3.2135 3.9279 3.7489 3.2427
4.3474 3.2983 3.4946 3.4742 3.0477 4.1261 3.7601 3.4183
3.243 4.0546 3.5184 3.1702 2.9537 3.2974 3.3782 3.477
3.5515 3.2919 3.218 2.9345 3.8215 3.2507 3.5566 3.0148
3.3173 3.2447 3.3924 3.5665 3.6597 4.2152 4.0014 3.6144
2.9587 3.6433 3.2127 4.0355 3.5342 3.2828 3.671 3.5987
2.8653 4.2038 3.883 3.5656 3.1542 3.6575 4.6949 2.7931
2.9797 2.8642 4.1284 3.3889 3.266 3.6682 3.6377 3.0207
3.4888 2.9599
C16orf92 0 0 0 0 0 0 0 0
0 0 0 0 0 0 0 0
0 0 0 0 0 0 0 0
0 0 0 0 0 0 0 0
0 0 0 0 0 0 0 0
0 0 0 0 0 0 0 0
0 0 0 0 0 0 0 0
0 0 0 0 0 0 0 0
0 0 0 0 0 0 0 0
0 0 0 0 0 0 0 0
0 0.0333 0 0 0 0 0 0
0
C16orf95 2.7728 2.9404 1.8889 2.3677 1.2071 2.7526 1.0021
1.6303 1.9188 1.5808 2.099 1.5495 1.3525 2.6574 1.6291
1.7256 2.1986 3.0402 2.1636 3.1368 2.9792 2.5258 1.7639
1.8817 1.0351 1.6093 1.5862 1.7571 1.4954 1.8901 2.6909
2.5367 1.7239 2.6098 2.3482 1.3622 1.8751 1.6762 1.9235
1.7979 2.1223 1.7388 2.2953 1.4597 2.0086 2.9432 2.0245
1.5096 1.5 2.1444 1.9767 1.0772 2.3695 2.0594 2.6424
2.6703 2.0929 1.147 2.4211 1.4907 1.8274 2.8884 1.8224
2.9843 1.7263 1.2953 2.2457 2.2636 2.0759 2.0077 2.0576
3.0796 1.5938 1.5519 2.3296 1.6838 2.5429 1.5108 2.2904
2.0657 1.7676 1.0741 2.0782 1.1203 2.0793 2.382 3.0107
2.7437 2.6124
C16orf96 0.1364 0.1812 0.0374 0 0 0 0.0101
0.0775 0.0462 0 0.0769 0 0 0 0
0.0223 0 0.1233 0 0 0 0 0 0
0 0 0 0.0781 0 0.0422 0.099
0.0573 0.0222 0.0387 0 0.0132 0.0046 0.1464 0.0102 0
0.0137 0 0 0 0 0 0 0
0 0 0.0174 0 0 0.0203 0.0463

0.1193	0	0	0	0.0103	0	0.012	0.0059	0
	0.1367	0.0302	0.1763	0.0037	0.0506	0	0	0
	0.0097	9.00E-04	0.0146	0	0.0595	0	0.2232	
0.0943	0.2692	0	0	0	0	0	0.0929	
0.1567	0.176							
C17orf105		0.3822	0.3177	0	0	0	0	
0.0368	0	0.414	0.2779	0.0644	0.0184	0.039	0.3945	0
	0.0913	0.4758	0	0.3503	0	0	0	0
	0	0.0274	0.1958	0	0.2109	0	0	
0.1535	0.0885	0.2818	0.0563	0.0562	0.035	0.0751	0.1514	
0.0339	0	0.0485	0.0126	0	0.0679	0.031	0.1052	
0.0727	0.0669	0	0	0.1071	0.0533	0.105	0.074	0
	0	0.0923	0.0714	0.0599	0	0	0.0592	0
	0.0841	0	0	0.6576	0.0358	0	0	0
	0.0691	0.3161	0.0487	0	0	0.0462	0.0623	
0.0796	0.5401	0.2193	0	0	0.0657	0	0	
0.1559	0	0						
C17orf107		1.1073	0.0558	0.0748	0.0552	0.0285	0	
0.0383	0.5421	0.1172	1.6747	0.0549	0.0471	0.1301	0.4592	
0.4472	0.0495	0.3759	0.6787	0	0.0474	0.3004	0.2275	
0.0359	0.1514	0.2442	0.6474	0.1611	0.3448	0	0.0276	
0.1309	0.065	0.5208	0.0234	0.0648	0.8309	0.0553	0.054	
0.0169	0.1782	0.1526	0.121	0.2068	0.7898	0.035	0.1	0
	0.0563	1.0095	0.2107	0.2161	0.4465	0	1.3119	
0.3067	0.168	0.5911	0.1323	0.0968	0.1631	0.1697	0.0271	
0.2055	0.5921	0.1301	0.0744	0.0667	0	0.0558	0.2602	
0.1576	1.0284	0.3057	0.0593	0.0622	0	0.1128	0.0674	
0.3395	0.0363	0.0347	0.4691	0.0094	0.1356	0.502	0.2108	
0.4222	0.5204	0.4292						
C17orf47	0	0.0412	0	0.0319	0.0375	0	0.0358	0
	0.083	0.0127	0.0151	0.0218	0.0815	0.0911	0.0051	
0.0058	0.0334	0	0	0.0321	0.028	0	0.0549	
0.0834	0.0272	0	0	0	0.0266	0.0158	0.0627	0
	0.0175	0.0134	0.038	0.0095	0	0	0.0083	0
	0.0116	0.0693	0	0.0168	0.0458	0.0313	0.0258	
0.0377	0.267	0	0.0299	0.0818	0	0.0239	0.0897	0
	0.051	0	0.0433	0	0	0	0	0
	0	0.0105	0	0	0	0	0.0288	
0.0344	0	0.009	0	0	0.0173	0	0.0739	0
	0	0.051	0	0.0422	0	0.1478	0	0
	0							
C17orf49	2.4176	2.0341	1.5894	2.1489	3.8417	2.116	1.5974	
1.2144	2.7227	2.1457	1.9411	2.5564	1.6757	1.1822	2.541	
1.974	2.5487	1.9831	1.7033	1.3421	1.059	2.7976	2.3737	
2.0022	2.3485	2.4031	2.9758	1.6691	2.9851	1.7002	4.3186	
1.2717	2.5097	2.517	2.4933	1.7746	1.088	2.4238	3.0074	
2.5239	1.4534	2.1478	3.1058	1.7918	2.1241	2.0438	1.1816	
1.9432	0.9865	2.7164	1.8286	1.9559	1.0293	2.5177	3.8134	
2.6334	2.6424	2.8905	0.8015	2.7843	2.2025	2.0124	2.9627	
3.0078	1.1335	1.5674	1.3154	1.5286	1.4105	2.3903	1.8386	

2.5004	1.8609	2.2923	2.4478	1.6525	1.7578	1.9213	2.1452	
2.3989	2.6415	1.0842	0.6689	1.9641	3.2207	1.7787	1.4593	
2.1032	1.6115							
C17orf50	0	0	0	0	0	0	0	0
	0	0	0	0	0	0	0	0
	0	0	0	0	0	0	0	0
	0	0	0	0.3078	0	0	0	
0.0338	0	0	0	0	0	0.1145	0	0
	0	0	0	0	0	0	0	0
	0	0	0	0	0	0	0	0
	0	0	0	0	0	0	0	0
	0.132	0.0415	0	0	0	0	0	0
	0	0	0	0	0.0661	0	0.1273	0
	0	0	0	0	0	0	0	0
	0							
C17orf51	2.1207	2.598	2.6241	1.5276	1.6538	1.98	3.0006	
2.516	2.4141	2.6268	1.3604	1.6102	2.6449	2.2244	2.3067	
2.6563	3.2158	2.5521	2.1787	2.2342	2.2992	1.8662	2.1337	
2.3391	1.2455	2.8373	0.443	2.1284	2.2183	1.6372	1.8611	
1.7156	2.7196	1.7575	2.789	2.2161	2.3462	2.1359	1.0881	
2.3375	1.2231	1.1702	1.8762	1.7832	1.1767	1.3957	2.5526	
1.8564	1.4712	1.3401	2.2296	1.7715	1.2718	2.9413	2.4296	
2.133	3.1358	2.3361	1.9842	2.1155	1.7199	1.4417	1.8833	
2.2811	1.8045	2.5801	1.2416	2.1355	1.5745	1.9159	1.7068	
1.6555	3.5241	2.2485	1.9352	2.2605	2.0563	2.2286	2.0709	
2.0972	2.918	0.7025	1.2948	1.5699	1.151	1.1678	2.4404	
2.4336	2.0035							
C17orf53	2.3248	3.0243	2.8477	2.6051	2.6395	1.7826	1.1778	
2.2389	2.307	2.1834	1.8824	1.7941	2.4493	1.9375	1.7374	
1.947	2.4813	1.9713	2.5531	2.2362	2.3126	2.2996	1.4491	
1.9082	1.5744	1.3978	0.3771	3.0641	2.2045	2.0786	2.3472	
3.0739	2.3302	1.8346	1.5639	2.1488	2.2949	0.9901	1.2988	
1.8364	1.2489	1.0918	1.9279	2.4038	2.5105	2.2011	2.3947	
2.1509	1.2988	2.6222	2.4632	1.6784	1.9099	1.7257	1.9023	
2.3118	1.7061	2.6533	2.2403	2.4463	2.4271	2.5902	1.6843	
2.5055	2.1618	0.7177	2.0025	1.0281	2.4343	2.388	1.5158	
2.509	2.1031	1.9425	1.6296	2.281	2.9632	1.3995	1.7786	
2.3576	2.6108	0.9589	1.4024	1.0946	2.4048	2.1788	1.8761	
2.3711	1.9854							
C17orf58	4.2267	4.0515	3.4853	4.028	3.401	2.8463	3.4568	
3.7189	3.422	3.7771	4.1228	4.1347	3.1352	4.0772	1.961	
4.0179	3.0992	3.6664	3.68	5.1171	4.4382	2.907	3.1133	
4.2994	3.3507	2.6288	4.1801	4.9484	3.2053	4.0768	4.0401	
3.8628	3.5304	4.3623	2.9879	4.2565	4.0999	3.6723	3.6152	
3.8848	4.0143	4.1996	2.4981	3.6388	3.4452	3.6482	4.4426	
3.9264	2.4824	3.8175	3.6156	3.4877	4.6874	3.7514	4.7341	
3.8082	3.3324	2.9463	3.6334	2.6064	3.9556	3.0555	3.6239	
3.4167	4.9506	2.4804	4.6489	3.7884	3.2185	2.8	3.5056	
3.4562	4.5784	3.4247	4.1662	4.1298	3.6083	3.7226	3.8687	
4.6722	3.9455	2.9776	2.4317	3.319	3.4389	4.3636	3.2993	

3.2566	3.3873							
C17orf62	4.8418	4.1479	4.5753	2.6682	4.0324	3.6992	3.885	
3.8606	3.8309	4.043	4.3391	3.4375	4.2782	3.2878	3.9471	
4.0249	4.2967	3.7789	3.259	3.5648	3.1188	4.3668	4.1838	
4.0333	4.2644	4.6083	3.7811	5.0855	4.0136	3.4471	4.6218	
4.7614	3.7261	4.342	4.0909	3.2311	4.5962	4.3587	3.7711	
3.0913	2.9012	4.2463	3.3344	3.6029	3.7917	3.8053	3.1795	
4.4788	4.574	3.5733	4.1491	3.0846	2.8212	3.8973	5.1143	
4.0503	3.1317	3.6246	3.445	3.9805	3.9501	3.7217	4.0729	
4.0439	4.5428	4.0177	5.0613	4.3122	3.5394	4.3063	4.4037	
4.1266	3.7681	3.685	4.1337	4.6234	3.7998	4.4144	3.7837	
4.0389	4.4363	3.6282	3.9195	4.4515	4.8051	3.3458	4.1109	
4.2429	4.2155							
C17orf64	0.0986	0	0	0	0.0219	0	0	0
	0	0	0	0	0	0	0	0
	0	0.0849	0	0	0	0	0	0
	0	0	0	0.0928	0	0	0.0664	
0.0146	0.058	0	0.0183	0	0.3052	0	0.1116	0
	0	0.0191	0	0	0	0	0	0
	0	0	0.0495	0	0	0	0.14	0
	0	0	0	0	0	0.1142	0	0
	0	0	0	0	0	0	0	0
	0.0639	0	0	0.0607	0	0	0.0344	0
	0	0	0.0445	0.124	0	0.127	0	0
	0							
C17orf67	0	0.9157	1.0306	0.6488	0.2625	0.2939	0.1919	
0.0651	0.6763	0.7302	0.4917	0.3207	0.4561	0.6348	0.2671	
0.2071	1.0485	0.701	1.1845	0.2916	0.8848	0.1224	0.1097	
0.6904	0.1736	0.6639	0.8603	1.4442	0.1353	1.1946	0.4823	
0.9721	0.1626	0.3803	0.063	0.3423	0.7831	0.1727	0.1916	
0.0926	0.5419	0.3163	0.7933	0.4323	0.3627	0.6811	0.4361	
0.1115	0.5112	1.1049	0.2908	0.2516	0.7672	0.521	0.8877	
0.9402	0.9288	0.2109	0.5484	0	0.5627	0.5296	0.0283	
0.7182	1.8387	0.6463	1.2094	0.7055	0.5981	0.4022	0.3224	
0.6299	0.3585	0.1215	0.2718	0.6299	0.8218	0.1248	0.6113	
0.1311	0.9426	0.2378	0.4799	0.3232	0.4905	0.6206	0.6031	
0.5949	0.4365							
C17orf74	0	0	0	0	0.0679	0	0.0946	0
	0.0462	0	0	0	0	0	0	0
	0	0	0	0	0	0	0.0456	0
	0.0297	0	0	0.1014	0	0	0.0342	
0.035	0	0	0	0.0461	0	0	0	0
	0	0.0435	0	0	0.0403	0	0	0
	0	0	0	0	0	0	0.1696	0
	0	0.0311	0	0.0284	0	0	0	0
	0	0.0234	0	0	0	0	0.022	0
	0	0	0	0	0.0139	0	0	0
	0	0	0	0	0.0429	0	0	0
	0							
C17orf75	2.7878	2.384	1.8506	2.4324	2.4939	2.2773	1.847	

2.7459	2.5015	2.4255	2.1932	2.1593	2.6495	2.6378	2.1075	
2.637	2.7295	2.7524	2.7439	0.4373	2.0537	1.877	1.9276	
2.066	1.7974	2.0655	2.1264	3.2101	1.8329	2.6102	1.8738	
2.3835	2.0537	2.4333	1.6467	2.6734	2.3483	1.9394	2.0491	
1.7289	1.951	1.9021	1.4225	2.5219	2.3232	2.7206	2.7808	
1.5979	1.6256	3.2256	2.4255	2.1873	2.4748	1.9734	2.2092	
2.3529	1.5324	2.6751	2.281	2.5844	2.0229	2.8325	1.56	
2.2881	1.3306	2.2779	2.1106	2.2563	2.4313	2.1129	2.0989	
2.4775	1.0577	1.6836	2.0017	3.051	2.4907	1.8529	1.9427	
2.3352	2.5342	1.4546	2.0271	1.449	2.5529	2.639	1.9697	
1.8786	1.9201							
C17orf78	0.0762	0	0.1353	0	0	0.2391	0	0
	0.0235	0	0	0.0093	0	0.1518	0.0137	0
	0	0.3293	0	0	0	0.0524	0.0275	0
	0	0	0	0	0	0.1243	0.021	
0.0677	0	0	0.0631	0.0205	0.0108	0	0	0
	0	0	0	0	0.0153	0	0.089	
0.0421	0.0682	0	0	0.0358	0	0	0.022	
0.0422	0	0	0.1338	0	0	0	0	0
	0	0.0215	0	0	0.0418	0	0.0121	
0.0364	0.0174	0	0	1.1398	0	0.0159	0.0212	0
	0	0	0	0	0.0411	0	0.2033	0
	0.0751							
C17orf80	3.5348	3.7705	3.6117	4.0535	3.4052	2.997	2.7341	
3.5943	3.0264	2.7325	3.2987	3.035	3.3783	3.6749	3.2181	
3.3201	3.5111	3.7438	3.7274	4.5679	3.7623	3.1461	3.1661	
3.4768	3.5913	3.7195	1.6688	4.1829	3.131	3.1188	3.1168	
3.9944	3.2993	3.1087	3.2081	3.6435	3.8773	3.3449	3.1588	
3.5055	2.6607	3.1375	2.6715	3.0081	3.6301	3.4232	4.1597	
3.5225	2.9992	4.1944	3.2768	3.2966	3.8903	4.0292	4.161	
3.3854	2.6487	3.6004	3.3659	3.5802	3.2291	2.6903	3.5594	
2.9217	3.9098	2.8153	4.2338	3.1912	3.1353	3.4519	2.837	
3.2157	3.8407	2.9266	3.6095	3.6815	3.7417	2.9292	2.9522	
2.4203	3.7382	3.2022	2.5243	3.8197	3.3345	3.7183	2.8877	
2.8937	2.9116							
C17orf96	1.5277	2.3662	1.5517	2.6624	2.6929	2.9222	1.3213	
1.6852	1.8978	2.1811	2.6186	2.601	1.7264	1.4791	1.7999	
1.9623	2.664	2.2053	1.8445	2.524	1.2884	3.0112	3.2162	
2.0747	3.1232	1.4678	1.7142	3.2816	3.3129	2.5743	2.9796	
1.9128	1.6876	0.797	1.4089	2.4065	3.395	2.2468	2.6125	
2.877	2.3566	1.9426	2.0242	2.4884	2.4282	2.2577	2.4314	
3.5311	1.7244	2.5188	1.6785	3.0626	2.0903	1.9854	2.6719	
2.2774	1.9421	4.1177	1.1087	3.5661	3.0281	2.136	1.8678	
1.7702	2.9965	1.2052	2.5162	1.7834	1.7566	2.5494	2.0446	
2.7477	1.8916	2.1785	2.3338	0.8523	1.3764	1.4379	2.3737	
1.724	1.5217	2.5348	1.3895	1.6	2.7965	1.832	0.7129	
0.8746	0.877							
C17orf97	0.7148	1.612	1.3241	2.9594	0.7504	0.7941	0.6286	
1.1207	1.9041	0.9793	0.8488	1.4702	2.3528	0.6996	0.6768	
0.5674	1.1031	1.0605	1.7836	1.8054	1.778	0.1408	1.0043	

0.4907	1.4956	1.6077	0.1273	1.1056	0.9799	0.7692	2.1396	
0.6673	0.6748	0.554	1.3509	1.8992	1.9588	1.6245	0.4384	
1.4738	1.1239	0.7388	0.6652	0.3672	0.6799	2.1299	1.4962	
0.5869	1.3066	2.1592	0.5949	2.2852	1.1892	1.5017	1.8772	
0.4664	0.0923	1.0508	0.7825	0.9146	0.5069	2.1518	1.6195	
1.5832	3.1184	2.214	1.1612	1.4556	0.4713	1.3082	1.7184	
0.4669	1.4945	1.7156	1.2558	1.1507	0.8031	1.4379	0.5698	
0.8655	0.9354	1.6904	0.4901	0.5769	1.4682	0.7362	1.9182	
2.0802	1.7991							
C17orf98	0.1973	0	0	0	0	0	0	0
	0	0	0	0	0	0.2494	0	0
	0	0	0	0	0	0	0	0
	0	0	0	0.2005	0	0	0.0806	0
	0	0	0	0	0	0	0.0837	0
	0.0946	0	0	0	0	0	0	0
	0.1858	0	0	0.3281	0	0	0	0
	0.3948	0.1221	0	0	0	0	0.1134	0
	0	0	0	0	0	0	0	0
	0	0	0	0	0	0	0	0
	0.1171	0.0763	0	0	0	0	0	
0.148	0.1679							
C17orf99	0.0429	0.0488	0	0.1486	0.091	0.2801	0.0109	
0.0708	0.0474	0.0576	0.0806	0.0304	0.1914	0.0511	0.0652	
0.0741	0.081	0.2606	0.0617	0.1671	0.2491	0.018	0.0252	
0.1244	0.059	0	0.109	0	0	0.0418	0.0263	
0.006	0.0427	0.0425	0.0093	0.0133	0.023	0.0258	0	
0.0438	0.0506	0.0417	0	0.1301	0.0271	0.1139	0	
0.0232	0.0906	0.2569	0.0788	0.0891	0.1304	0.0236	0	
0.0849	0.2022	0	0.1207	0	0.2052	0.1127	0.0272	0
	0.0857	0.0223	0.1048	0.0548	0.0704	0.0737	0.0264	
0.0683	0.0362	0	0.0283	0.1002	0.1454	0.0727	0.0408	0
	0	0.0194	0.0246	0.0668	0.1048	0	0	
0.0757	0.0619							
C18orf21	3.3058	4.14	3.6718	4.0845	3.2508	3.6288	3.2861	
3.9234	3.5446	3.7297	4.2358	3.8311	3.4173	2.8336	3.6237	
3.269	4.066	3.6094	3.9555	3.7091	3.8821	3.6552	3.3289	
3.7745	3.7895	4.0499	4.7921	3.498	2.1505	3.6579	3.1361	
3.9701	3.2789	2.6905	2.364	4.0594	4.1846	3.8435	4.0609	
3.8328	3.1778	3.6691	2.8901	3.8861	3.262	4.0499	3.9476	
3.5607	2.6698	4.0047	2.7741	3.9338	3.8452	3.8439	4.2116	
3.5068	2.5104	3.7942	3.9232	3.9026	3.1161	4.2526	3.8122	
2.6934	3.6405	3.6208	4.8713	3.93	3.3141	3.3457	3.0001	
2.9474	3.2967	4.0598	3.1968	3.4333	3.8216	3.7534	3.8118	
3.3826	3.9601	3.0706	3.9873	2.6134	3.2478	3.6267	3.2274	
3.2068	3.4161							
C18orf25	2.5924	3.2004	3.2776	3.8643	3.5337	3.5281	3.2468	
3.0111	3.8345	3.5982	3.7248	3.7296	2.9205	3.7966	3.4128	
3.0531	3.188	3.2692	3.3818	3.4233	3.3763	3.9069	3.5345	
3.5788	3.1526	3.9216	4.8176	3.6211	3.0338	3.5233	2.6201	
3.7466	3.2028	3.4911	2.9379	3.8751	3.2322	3.248	3.7189	

2.8066	3.3042	3.431	3.4203	3.6347	3.6068	3.7522	3.0972	
3.2199	3.0531	3.9669	2.6369	3.8298	3.5475	3.1743	4.8923	
2.934	2.7885	3.1701	3.7411	3.3604	3.564	3.4543	3.3487	
3.3838	3.1604	3.3811	3.6522	3.2748	3.4344	3.2785	3.7992	
3.8233	3.0848	4.0025	3.8508	3.187	3.1911	3.6078	3.251	
3.1739	3.0578	4.0364	3.22	3.392	2.7691	3.514	3.1421	
3.289	3.1898							
C18orf32	3.4129	3.9943	4.1091	4.5398	3.4562	3.8022	4.7974	
3.7635	5.4553	5.184	5.1042	4.8861	3.6339	3.5823	3.9208	
4.1303	3.6101	4.8041	4.9476	3.4766	4.226	4.5845	4.969	
3.7463	4.396	4.8318	5.0246	4.8875	3.3736	3.8772	4.3429	
4.2648	4.1108	4.9823	3.9247	5.3238	4.707	4.7929	4.7429	
3.6508	4.413	4.6095	4.2982	4.4174	3.942	5.206	4.3008	
3.6339	4.3243	3.1443	4.5296	5.2067	4.8135	3.5813	4.4703	
4.0085	3.6146	3.8576	4.5087	3.5315	3.6696	3.9748	4.8006	
2.0207	4.1233	5.2532	5.108	5.4256	4.1646	3.2074	4.3972	
4.1881	4.2501	4.5008	4.4772	4.7402	4.1833	4.911	4.7106	
4.8649	3.33	4.7076	5.0762	4.7085	4.6007	4.3333	5.3594	
5.156	4.7629							
C18orf54	1.2367	1.481	1.8788	1.535	1.5368	1.9804	1.1973	
1.6129	2.4304	2.0716	2.0378	2.4235	1.8919	2.0588	2.8837	
1.8658	2.8358	1.9946	2.3855	2.2353	2.6377	2.0883	1.229	
2.0838	2.3181	1.9033	1.7222	2.4688	1.4253	1.2895	2.1907	
1.7972	2.398	2.7457	1.6926	2.7286	1.3565	0.5911	1.253	
1.6783	0.8588	1.8489	2.4457	2.7495	2.1675	3.1013	2.1517	
1.8582	1.4129	2.4819	0.9517	2.2024	2.7861	1.5107	2.9902	
1.4579	2.1647	2.3784	2.1343	2.5124	1.8106	1.8592	2.0111	
2.4661	1.8535	0.7327	2.6583	1.8128	1.987	2.1839	2.1882	
2.8279	1.7258	2.1608	2.524	2.5249	1.7646	1.4692	1.8022	
1.6838	2.7006	0.7107	1.0293	1.5596	2.2508	3.1318	1.9352	
2.4112	2.1773							
C18orf63	0	0	0	0	0	0.0174	0.0031	0
	0	0	0	0	0	0	0	0
	0.0204	0	0	0	0	0	0	
0.0115	0	0.0137	0	0	0	0	0	
0.0011	0	0	5.00E-04	0	0	0	0	0
	0	0	0	0	0.0031	0	0	
0.0125	0	0	0	0	0	0	0	0
	0	0	0	0	0	0	0	0
	0	0.0024	0	0	0	0	0	0
	0	0	0	0	0	0	0	0
	0	0	0	0	0	0	0	0
	0							
C18orf8	3.1732	3.481	3.6807	3.7594	3.7007	4.0196	4.0352	
3.6282	4.464	4.3615	4.1906	3.9476	3.3443	3.9819	4.0313	
3.9511	3.0751	4.1744	4.132	4.1366	4.2951	3.8991	3.896	
3.6836	3.9484	3.8653	3.6175	3.674	3.2505	3.1639	3.5377	
3.8322	3.9143	4.0819	3.2847	4.5865	3.8725	3.4202	3.9396	
3.1237	3.39	3.549	3.3965	3.579	6.5639	4.0014	3.9793	
3.8706	3.271	3.2326	5.1777	3.9185	4.2796	2.988	4.4076	

3.4061	3.1402	4.0397	4.3532	4.4216	3.3497	4.0324	3.177	
4.3564	2.592	3.751	3.7914	4.0643	3.8819	3.4297	3.6524	
4.4586	3.4867	3.855	3.3488	4.3318	3.9372	3.2665	3.56	
4.2577	3.8588	3.155	4.2811	3.4154	3.6336	4.7826	4.0996	
3.8068	3.7193							
C19orf12	3.013	3.2169	3.5791	2.2194	2.5985	3.1098	3.1793	
2.8135	3.3661	2.5417	3.9205	3.4937	2.8545	3.8193	3.8758	
3.204	2.5104	3.3578	3.3979	2.8414	3.598	2.4343	3.1681	
2.7256	2.2083	3.1503	2.475	3.1773	2.5798	3.8114	2.8785	
2.9407	3.4571	5.0887	2.6547	3.0366	3.0498	3.8888	2.7161	
2.3537	2.9141	2.3742	2.3157	1.9929	3.0658	2.3584	2.6942	
2.7991	2.6152	2.632	3.7473	2.2198	3.7353	3.2212	2.6437	
3.5814	2.7838	1.7881	3.5597	1.7194	2.8201	3.363	2.7222	
2.8187	3.6731	2.6704	3.0873	4.2151	3.8336	2.1827	3.3033	
3.0023	3.545	3.0643	2.2234	3.7987	3.4592	2.6072	3.5289	
3.0922	2.8339	2.9207	3.2849	2.6957	2.9476	4.2874	4.4812	
4.2683	4.9115							
C19orf18	0.1681	0.2525	0	0.1262	0.0299	0	0	0
	0	0.0527	0	0	0.3779	0.0772	0.0382	0
	0	0.2544	0.2003	0	0	0.0313	0	0
	0.1139	1.7067	0.0564	0.2356	0	0.1264	0.1987	
0.0538	0	0.0571	0.0867	0	0.422	0.0729	0.1687	
0.0471	0	0.0989	0	0.1146	0.6123	0.1345	0	0
	0	0.3263	0.0931	0	0	0	0	
0.2846	0.0797	0.0571	0.041	0.0571	0.0366	0	0	0
	0.3712	0.9892	0.1038	0.3863	0	0.0981	0	
0.0521	0.1217	0	0.0358	0.0633	0.0226	0	0.1084	0
	0.1099	0.4947	0.3537	0.173	0.8015	0.1834	0.3706	
0.1431	0.2131							
C19orf24	4.8795	4.5681	5.4389	2.4592	4.8469	4.15	4.2805	
5.2689	4.2086	3.8122	4.8164	2.9373	4.5526	2.7354	4.5684	
5.1072	5.0906	3.2484	3.7376	5.2568	6.0153	4.9619	5.3698	
4.8488	3.6301	4.0928	2.9612	4.4912	4.5623	4.0819	5.1298	
5.5407	5.3866	4.6875	4.6388	2.9567	5.1532	5.9856	4.9266	
3.1621	4.9627	4.0738	4.1481	3.3935	3.1853	3.1786	3.1171	
4.307	5.3216	2.5007	3.348	2.7086	3.1322	3.7433	5.6077	
6.1545	3.2563	4.9304	4.4841	4.2558	4.9217	4.4701	4.5733	
4.5717	4.7961	4.6584	5.8982	5.2636	5.1257	4.0253	5.4845	
5.0588	5.3087	4.0816	3.4967	5.8576	5.5236	4.7909	5.1194	
5.0882	4.9689	4.4222	6.1811	4.0298	4.5502	4.6397	4.3445	
4.9959	4.7729							
C19orf25	2.5781	3.3427	3.0884	1.5571	2.9749	2.8998	2.4302	
3.4591	2.4681	2.3749	2.8586	1.9569	3.8928	1.843	3.1516	
2.817	3.1489	1.9801	2.5067	3.5721	3.0545	3.7195	2.7007	
2.9647	1.7553	3.4164	2.5772	3.1723	2.5699	3.1776	3.5652	
4.2765	3.3108	2.4672	2.9425	1.9214	3.0233	3.5198	4.1101	
1.6342	2.9958	2.9946	2.3161	2.2741	2.2044	2.7854	1.8677	
2.7617	3.5121	1.8677	1.6509	1.627	2.1448	2.8297	3.2265	
3.6282	2.7141	4.4111	2.5326	3.743	3.6522	2.7974	3.0024	
2.3591	2.2202	3.6095	3.6266	3.2131	2.4259	2.8387	2.8904	

2.6664	1.5963	2.6334	2.0873	2.7805	2.9465	3.94	3.5476	
2.44	3.554	2.7601	3.3127	2.6129	3.3104	3.0049	3.1306	
3.7945	3.526							
C19orf33	0	0	0	0	0.175	0	0	0
	0	0.3156	0	0.1804	0	0	0	0
	0	0	0	0	0	0	0.1802	0
	0	0	0	0	0	0	0.2106	0
	0	0	0	0	0	0.3395	0	0
	0	3.8384	0	0	0	0	0	0
	0	0	0	0	0	0	0	0
	4.89	0	0.1183	0	0.8433	0	0	0
	0.1089	0	0	0	0	0.1512	0.0705	0
	0	0.042	0	0	0	0	0.0501	0
	0	0.1709	0.0685	0	0	0.2157	0	0
	0							
C19orf35	0.1798	0.0649	0.2114	0	0.0224	0.1651	0.0876	
0.0854	0.1964	0.0879	0.0633	0	0	0.1398	0.1144	0
	0.4958	0.1546	0.2743	0.0886	0.1175	0.0843	0.0225	
0.0951	0.1012	0	0.5274	0.0842	0.0443	0.0579	0	
0.0309	0	0.0546	0.0891	0.0782	0	0.1836	0.1135	
0.1261	0.039	0.0877	0.2543	0	0.1064	0.107	0	
0.1594	0.0378	0	0.1228	0	0.1101	0.1691	0.0145	
0.4048	0.036	0.0888	0.1723	0.0915	0.0585	0	0	0
	0	0.0152	0.0661	0.0366	0.1107	0.1992	5.00E-04	
0.0859	0.102	0.0309	0.0798	0.2567	0.1668	0.1113	0.1801	
0.1779	0.203	0.1359	0.1543	0.1355	0.0926	0	0	
0.0614	0.0714							
C19orf38	0.1162	0.1493	0.1015	0	0.0318	0	0.9412	
0.0574	0.0542	0.1563	0.3283	0.0861	0.6449	0.2309	0.0906	
0.2331	0	0.1726	0	0.2124	0.0846	0.0383	0	
0.1452	0.0711	0.6853	0	0.7813	0.0563	0.067	0.5553	
0.5388	0.2261	1.0305	0.3251	0.0539	0.2047	0.0757	0.8176	0
	0.0901	0	0.4008	0.2559	0	0.3078	0	
0.0698	0.0918	0.395	0.274	0	0.1207	0.1248	0.1498	
0.11	0	0	0.1063	0.0891	0	0.0201	0.4781	
0.1849	0.4927	0.3295	1.1369	0.5547	0.2407	0.2954	0.109	
0.5664	0.0927	0	0.1896	0	0.5082	0.1198	0.1114	
0.5707	0.3917	0.0104	0	0.3547	0.051	0	0.5063	
0.1913	0.0959							
C19orf43	6.3327	6.9996	7.1874	6.948	7.5523	6.571	6.8161	
7.0865	6.2557	6.495	6.8	7.1574	6.8232	8.1075	6.7486	
6.9284	6.2697	6.3911	7.0841	6.9109	7.1117	6.1931	6.3539	
7.4847	6.9022	6.7982	7.3696	7.2479	6.7413	6.9869	7.2984	
7.0665	7.3826	6.7503	6.0489	7.23	6.6537	6.4724	7.141	
6.362	5.5934	6.6917	6.5431	6.8308	6.8878	6.8461	6.4937	
6.6072	6.6319	7.0777	6.6864	6.6289	6.6273	7.4608	7.39	
7.4563	6.3379	7.0162	7.8866	7.0538	6.8557	6.8801	6.5277	
7.0395	6.8557	6.7082	7.0625	7.1309	7.6788	6.918	7.0625	
7.1726	6.3783	6.3021	6.5581	7.0656	7.2172	6.3038	6.8301	
6.6952	6.9556	6.6888	7.0924	6.3985	7.7709	6.994	6.4339	

6.6537	6.4482							
C19orf44	1.5594	2.1262	1.6609	0.9565	2.0891	1.2237	1.3802	
1.4152	0.9975	1.0482	2.1054	1.4331	1.6886	1.8129	1.6054	
1.1911	1.9577	1.3256	1.3718	1.7117	1.5715	1.5037	1.1544	
1.533	1.3427	1.9767	2.6379	1.4526	1.5405	1.631	1.4123	
1.2497	1.8803	1.2771	1.1388	1.1314	1.9298	1.3809	1.5289	
1.4508	1.5801	1.1028	0.9584	1.043	1.5342	1.3821	1.243	
1.4342	1.6853	1.5866	0.837	1.4601	1.3045	2.2464	1.7725	
1.5967	1.5135	1.3236	1.4004	1.4889	1.8697	1.9071	1.8024	
1.4085	1.9315	1.7702	1.6664	1.3104	1.1166	1.1194	1.3583	
1.6232	1.282	1.3097	1.2451	1.2729	1.5033	1.1445	1.4716	
1.0372	1.306	1.3973	0.9946	0.9559	1.5901	1.1014	1.198	
1.1917	1.1039							
C19orf45	0.0869	0.1076	0	0.0887	0	0	0.0365	0
	0.0377	0.1621	0.4395	0.1717	0.1486	0.1111	0	0
	0.0854	0	0	0.2128	0	0	0	
0.0477	0	0	0	0.176	0	0.1634	2.12	
0.0844	0.1196	0.4301	0	0.0376	0.1288	0.1171	0.2218	
0.0508	0.0799	0.1523	0	0.0502	0.0331	0	0	0
	0.1085	0.4325	0	0	0	0.2255	1.4305	0
	0	0.1858	0.144	0	0	0	0.0947	
0.0603	0.116	0.0594	0	0.1712	0.2001	0	0.0861	0
	0	0.0837	0	0	0.099	0	0.0198	0
	0.037	0.3091	0.147	0	0	0.1207	0	0
	0.1178							
C19orf47	2.5673	3.6229	3.8103	2.7101	3.6517	2.3457	2.0273	
3.2701	2.3548	2.4352	2.8979	2.6299	3.2202	2.1214	2.7974	
2.8245	2.715	2.0675	2.8347	3.3964	3.7104	3.2711	3.0545	
3.1032	2.8855	3.0981	2.4888	3.3872	2.8643	2.4154	3.7422	
3.3326	3.5786	2.9504	2.5216	2.3379	3.0204	3.1505	3.3068	
1.9227	3.0007	2.4669	2.4541	2.1962	2.6659	2.6464	2.1658	
2.525	2.3986	3.1736	2.4366	2.6929	2.0417	3.1353	3.1771	
3.5064	2.056	3.2857	2.7265	3.2301	2.9406	3.2842	3.2567	
2.6124	2.7699	2.4927	2.5442	2.2763	3.2728	2.9485	2.876	
3.317	2.7283	2.7886	2.159	3.356	3.3005	2.4348	2.4881	
2.5359	2.7991	2.9537	2.7342	2.0227	3.8132	2.9363	2.5309	
2.9554	2.6404							
C19orf48	5.1226	5.5729	6.1119	4.1449	5.8744	3.5405	4.1477	
4.9563	4.2306	4.6303	5.0413	4.2598	5.5876	3.9782	5.0512	
4.2811	6.2508	3.5886	4.3238	5.2897	5.4466	4.8951	4.8091	
4.4055	4.5311	4.7473	4.2005	5.4938	3.9361	4.2236	4.5287	
5.8347	5.5098	3.9102	3.8369	4.0741	5.3022	5.1316	5.8187	
4.1697	4.8488	3.2033	4.1023	4.9913	4.8014	4.6105	3.784	
4.9836	4.5088	4.0938	4.1496	3.7486	3.5338	5.8726	5.1826	
5.085	4.2062	5.4223	5.3675	5.6463	5.5579	5.5527	6.1095	
4.5126	4.7529	4.2849	4.0332	3.4924	5.2503	5.0702	4.7426	
4.8587	4.3964	4.5421	2.8749	4.4002	5.0522	2.9643	5.0182	
4.6467	5.0046	3.8131	4.1445	3.9176	5.2143	4.9874	3.398	
5.2654	4.2126							
C19orf52	3.2199	3.321	3.3977	4.0356	3.8585	3.4106	3.454	

3.6586	3.2463	3.3477	3.4512	2.9985	3.3881	3.6456	3.6823
3.744	2.5563	3.4663	3.8383	3.6475	3.3084	3.3989	3.2074
3.4928	2.8169	3.6351	4.1348	3.4636	3.5638	3.8819	3.3843
3.3102	3.5167	3.7134	3.2801	3.43	3.4592	3.3087	3.5584
4.1078	3.3102	3.3249	3.1157	2.7615	3.4051	3.659	3.277
3.0567	3.2502	3.8955	2.8478	3.4883	3.1036	3.538	3.4522
3.6202	3.3299	3.9595	3.7375	3.5522	3.7296	3.5321	2.9789
2.9597	2.9659	3.6045	3.3756	3.4903	3.1389	3.3476	3.3656
3.404	3.1048	2.9476	2.9058	3.2688	3.5026	3.0374	3.4532
3.3683	3.1415	2.988	3.4387	3.0115	4.2936	3.4748	3.4143
3.3421	2.9801						
C19orf53	5.8482	5.8427	6.3367	5.3391	7.2764	5.1506	5.7272
6.427	5.3979	5.4759	6.405	6.3442	6.4452	5.5879	6.0362
6.0242	5.3178	5.1311	5.166	6.2193	6.1836	6.4153	6.3673
6.7149	5.8906	6.0351	6.5769	5.6165	6.2614	6.3257	6.3455
6.6629	6.2478	6.3367	5.7449	5.1382	6.3414	6.0206	6.2899
5.4736	6.338	6.4554	5.6659	5.1247	5.5553	5.2554	4.8291
5.6616	6.2651	5.525	5.1855	5.6403	5.1671	6.0377	6.8673
6.4271	5.3355	6.0548	5.4279	5.7889	6.7202	6.5217	5.645
5.2969	5.5942	5.8768	6.9048	6.1936	5.7147	5.4686	6.5832
5.9619	5.8787	6.2254	5.2195	6.0214	6.2598	6.0914	5.7053
5.6606	5.7986	6.4685	6.687	6.0012	6.3682	5.8698	5.6689
6.0041	5.7411						
C19orf54	2.5111	3.2032	1.5862	2.0885	2.5492	3.2414	2.2592
3.1408	2.9402	2.7478	3.7288	2.7928	2.8238	2.6657	2.3492
2.0117	2.7435	1.8531	2.4361	2.6075	2.2364	2.1204	2.5689
2.4708	2.4495	2.4109	3.4628	2.6221	2.1873	3.1794	3.4261
2.8704	2.8382	3.7153	2.1144	2.0011	2.8712	2.7746	2.8134
3.2729	3.2103	2.2234	2.7068	2.8985	2.9755	2.7356	1.9968
2.7705	2.6544	3.4376	2.2553	1.7824	2.5119	3.272	2.0497
3.0753	3.0576	2.4053	2.473	2.6332	2.8483	2.9386	1.8342
2.7121	3.0377	2.6815	2.4208	1.71	2.4739	2.9047	2.8094
3.069	2.4515	3.4173	2.1309	1.6431	2.5837	1.959	3.1518
3.3373	3.1613	1.8694	2.6087	2.1007	2.7937	2.3184	2.6344
2.3416	2.7738						
C19orf57	1.0469	1.1091	0.943	1.2228	0.9736	2.924	1.376
1.1434	0.6415	0.4672	1.1917	1.1923	1.2215	2.0269	0.6848
2.2765	0	1.2234	0.6361	1.5439	1.6542	0.3485	0.5502
2.0779	0.3136	0.3566	0.4324	1.0538	0.619	1.2915	0.8966
2.105	1.7401	1.744	0.5434	0.3342	0.7668	0.3794	0.5139
0.9133	0.7033	0.4531	0.712	1.5288	0.2444	0.455	0.3019
2.135	0.7607	1.0007	0.4697	0.8898	0.9792	2.0612	0.8671
1.1906	0	0.7877	0.8186	1.1419	0.4457	1.0931	0.3914
2.1215	0.938	0.1979	1.7574	0.4247	1.1088	1.3112	1.5003
1.9718	0.4015	1.4698	1.6329	2.3022	1.4498	0.3186	0.9472
1.1833	1.1492	0.2502	1.3404	0.4347	1.1348	1.1129	0.5118
0.8414	0.76						
C19orf60	3.9816	3.8054	4.3664	2.7396	4.3194	2.5199	3.9403
4.3563	3.2797	3.0154	4.2919	3.3341	4.1711	3.1351	3.8816
3.5001	3.4884	2.8217	3.3282	3.5033	4.1611	3.3723	4.0704

4.3823	3.5872	3.2774	3.5313	3.9139	3.5905	3.2053	4.4392	
4.2884	4.126	4.6436	3.4466	3.3512	4.3097	4.5422	4.0711	
2.8375	4.2417	3.7119	3.9542	3.2096	3.1624	3.5947	3.1729	
2.9973	4.0663	2.5353	3.0058	3.0493	3.0171	4.4487	3.8467	
4.5914	3.53	3.3308	5.5416	3.17	4.3601	3.8489	5.334	
3.8091	4.2043	4.2021	4.3589	4.2923	4.1141	3.6956	4.6595	
4.0895	3.4411	3.8962	2.8977	3.9965	3.9698	3.1174	4.0287	
3.9147	3.9297	3.1226	4.6442	4.2563	4.3857	4.1104	3.1389	
4.2861	3.7476							
C19orf66	3.386	2.9769	2.8678	2.1053	2.936	3.3667	4.9918	
2.9899	3.2701	3.5314	4.5507	3.3062	3.5246	3.8496	4.3533	
3.3451	2.9984	3.6643	3.1887	3.252	3.9601	3.108	3.1275	
3.1533	2.1282	4.8367	3.5989	2.7521	3.1679	3.3913	3.6316	
3.2088	3.632	4.6558	3.8191	3.4682	3.6098	3.676	3.3264	
3.6248	3.8278	3.5373	3.7122	3.4161	4.373	2.9538	2.2048	
3.6705	4.3226	3.1581	3.2337	3.1374	4.293	4.3754	3.1698	
4.1649	2.6038	2.3514	3.9525	2.4034	3.5204	3.2756	1.6416	
3.6583	3.0826	5.1672	3.2591	3.9388	3.1859	2.916	3.9734	
3.9999	3.4301	3.6826	3.1222	2.8308	4.3337	2.7611	3.6226	
3.1652	3.412	3.4611	4.7878	3.7265	3.4462	3.5146	3.3688	
3.2845	3.3264							
C19orf67	0	0	0.083	0	0	0.0896	0	0
	0	0	0.0308	0	0	0	0	0
	0	0	0	0	0	0	0.0182	0
	0	0	0	0	0.0437	0	0	
0.0329	0	0	0.0462	0	0.0609	0	0	0
	0.0755	0.0161	0	0.0573	0	0	0	
0.0607	0	0	0	0.0604	0	0	0	
0.1113	0	0	0	0	0	0	0	0
	0	0	0.0758	0	0.0293	0	0	
0.0415	0	0.0071	0	0	0	0	0	0
	0	0	0	0	0	0	0	0
	0							
C19orf68	1.1034	2.2287	1.6335	1.5694	1.535	1.2952	1.4087	
1.243	1.28	0.9873	1.8179	1.2131	1.5006	1.2139	1.7231	
0.9255	1.9439	1.333	1.9189	1.6329	1.4872	1.7068	1.3144	
1.2852	1.2265	1.6721	1.4844	1.8272	1.2477	1.8185	1.1677	
1.5963	1.6133	1.7492	1.1019	0.6182	1.333	1.9177	1.1543	
0.9296	1.4958	0.9679	1.5413	0.9177	1.9349	1.7218	0.5763	
1.3636	1.5061	1.535	1.5618	0.7764	1.0979	1.6753	1.203	
1.249	1.8455	1.2407	1.2054	1.4965	1.6795	1.6158	1.6034	
1.4106	1.1484	1.8332	0.9624	1.2599	1.3266	1.6687	1.3445	
1.8144	0.8575	1.654	0.5778	0.9211	1.6442	0.9733	1.6148	
1.2893	1.123	1.2339	1.6996	1.1443	2.4034	1.8063	1.3519	
1.2773	1.6525							
C19orf70	5.6124	4.9474	5.5474	3.5792	5.8109	3.5935	4.2596	
5.2877	4.4133	4.3599	4.8585	4.1283	5.1969	3.951	5.154	
4.2481	5.1348	3.4727	3.9673	5.1912	5.3719	5.2716	5.2646	
5.0608	4.1467	5.1673	4.4155	4.6991	5.1263	5.0821	5.344	
5.5412	5.452	5.2178	4.6041	3.6405	5.0536	5.2227	5.7313	

4.1422	5.5105	5.0948	4.3658	4.469	4.1223	3.8515	3.6983	
4.5515	4.892	4.3779	4.1934	3.8559	3.8334	4.9982	5.3298	
5.3221	4.04	4.853	4.186	3.9826	5.8537	5.321	4.4853	
4.4682	4.6658	4.91	5.1822	4.9252	4.1721	4.049	5.6874	
5.0756	4.7875	4.6607	3.7369	5.2169	5.1377	4.5941	4.7024	
5.1165	4.4923	5.2809	5.3363	4.853	5.5207	4.7841	4.9856	
5.3953	5.1546							
C19orf71	5.6374	2.2026	6.1411	0.6654	2.7062	3.4436	2.5903	
2.8215	1.1145	3.135	3.8782	1.0389	2.5102	2.9416	5.1731	
5.5183	1.6404	3.5496	2.1572	6.2133	5.1823	4.3701	4.0147	
3.7484	2.4904	1.4025	1.8508	2.3472	4.9662	0.7671	0.9375	
3.0457	3.6207	3.1774	4.7111	2.708	3.7978	3.9817	4.1058	
0.8309	3.3455	1.6769	2.431	4.6373	1.5322	1.3045	2.5967	
3.3387	5.2354	1.0048	3.7248	2.2515	2.9779	4.6231	1.5775	
4.2786	0.8182	2.204	4.7042	2.8445	5.5257	2.2309	2.4883	
4.0694	2.4855	1.1361	2.2224	3.1118	4.0965	4.0251	2.5441	
6.2525	2.5319	2.6688	5.2375	6.1646	2.1684	5.8939	1.865	
2.9442	1.2068	4.3518	4.4226	6.1231	4.0611	4.6902	6.5076	
6.3521	7.1155							
C19orf73	1.1313	1.0488	0.9533	1.3302	0.7611	1.0798	0.3521	
0.4318	0.3226	1.3376	1.414	0.7269	1.3039	0.5919	1.0602	
0.278	1.2125	0.2941	0.7142	1.4077	1.0969	0.4479	1.2427	
0.7642	0.6212	1.1965	1.293	0.7794	1.0211	1.31	0.9618	
1.3881	1.0762	0.9015	0.8995	0.7007	1.1867	1.0416	2.3462	
0.6265	0.7346	0.3602	1.7476	0.4291	0.851	0.4933	0	
0.883	0.6554	1.1808	0.9248	0.2126	0	1.6505	0.525	
0.7139	0.7911	0.1425	0.6771	0.3619	0.5753	1.6881	0.9955	
0.8399	1.7046	0.5716	1.4108	0.4553	1.2697	0.5386	0.521	
1.0325	1.1324	0.5617	0.6204	1.0641	1.0189	1.5972	0.9426	
0.994	0.5221	0.7079	1.2725	0.8731	0.703	1.0505	0.9195	
0.7285	1.071							
C19orf81	0	0	1.7108	0	0	0	0	
0.3727	0	0	0.3295	0.3032	0.3342	0	0	
0.4908	1.1349	0.5531	0	0	0	0	0	0
	0	0	0	0	0	0.4115	0.7077	
1.2054	0	0.4357	0	0	0.6607	0.868	0.5118	0
	0.3447	0	0	0	0	0	0	0
	0	0	0	0	0	0.739	1.2622	
1.0878	0.3915	0.9397	0	0.8091	0	0.6969	1.5651	
0.2534	0.9102	0	1.1038	0.4676	1.1627	0.4182	0.0088	
0.0251	0.6775	0.2739	0	0.4666	0.6857	0.8307	0.7893	
1.1157	0.704	0	0	0.6089	0.1307	0.18	0	
0.5705	0.6292							
C19orf84	0	0	0	0	0.0172	0.0745	0	0
	0	0	0.0285	0	0	0	0	0
	0	0	0	0	0.0612	0	0	
0.4936	0	0	0	0	0	0	0	
0.032	0	0	0	0	0	0	0	0
	0	0	0	0	0	0	0	0
	0	0	0	0	0	0	0.1279	0

	0	0	0	0	0	0	0.0451	0
	0	0	0	0.2073	0.032	0	0	0
	0	0.1272	0.1964	0	0	0	0	0
	0	0	0	0	0	0	0	0
	0							
C1D	4.0994	4.5748	4.3282	4.8516	4.6195	4.5608	4.9278	
4.7942	5.4542	4.705	4.5381	4.8086	4.195	4.6221	4.7731	
4.6498	4.7883	5.0797	4.8498	4.2067	4.4624	4.5673	4.8847	
4.7181	4.1444	3.5867	4.7538	4.451	4.653	4.6671	4.1198	
4.496	4.9558	4.4906	4.3015	4.8798	4.3776	4.0986	3.5614	
4.9345	4.3632	4.7831	4.8863	4.3383	4.7603	4.6498	4.7592	
4.7302	4.6648	4.9388	4.6956	4.7968	4.9393	4.9087	4.308	
4.6862	5.0676	4.4116	4.8573	4.833	4.6986	5.009	4.1945	
4.0854	4.3355	4.4678	5.3824	4.4072	4.5718	4.4417	4.1141	
4.612	4.6933	4.8008	4.6124	5.1762	4.6683	4.4007	4.5484	
4.2906	5.1321	4.3949	4.3037	4.1853	4.1395	4.8641	4.2763	
4.1448	4.1824							
C1GALT1	4.9709	3.5194	4.6357	4.6126	3.9107	4.2106	4.8008	
4.0197	4.8024	3.1299	3.5774	3.885	3.5336	4.3047	3.7089	
4.502	4.2393	4.3126	4.1763	4.6095	3.7667	3.8445	4.4608	
4.443	4.0578	5.0385	3.3933	4.7389	3.946	3.924	4.5248	
4.1474	4.1563	3.5641	4.6389	3.8763	4.9949	3.9279	4.292	
4.434	3.7265	3.2435	4.0306	5.7418	4.3551	4.7172	5.1799	
3.8398	4.4926	3.2981	3.9008	5.5033	5.1729	4.2583	3.9438	
4.3605	4.1256	3.1572	4.4147	3.5073	4.0437	4.1554	3.9085	
4.1872	2.1983	4.7482	4.1728	4.3417	4.8884	3.95	4.3586	
4.4703	3.9398	4.6769	3.894	3.9583	4.0291	5.1257	4.2695	
4.7043	4.2316	4.0913	5.1344	3.6199	4.6046	4.6821	3.629	
3.6678	3.0765							
C1GALT1C1		3.6396	3.495	3.8461	4.7124	4.2642	3.878	
5.3921	3.5832	4.3614	3.6701	3.6343	5.1231	3.9718	3.8209	
4.4744	3.8516	0	4.2155	3.4946	3.9611	3.7359	4.4098	
5.2412	4.2578	3.4832	4.0892	3.4505	3.7032	4.1768	3.1932	
4.2669	5.0069	4.4906	4.9664	4.3606	4.2534	3.6675	3.831	
2.9737	3.9157	3.4355	4.7347	4.1437	3.4505	5.016	3.9774	
4.004	3.6271	5.2231	5.3054	4.1437	4.8546	3.5115	3.1912	
4.8585	3.0512	4.9295	3.999	3.3858	3.685	3.4474	3.7149	
4.1685	3.8715	2.9778	5.7416	4.5083	3.852	4.3471	3.873	
4.1071	3.4181	3.5844	3.5664	4.5927	3.368	3.8422	5.2902	
3.6514	3.0518	3.3713	5.256	4.2719	4.3212	4.1917	3.4662	
4.1781	3.8106	3.8074						
C1orf100	0.3053	0	0	0	0	0	0	0
	0	0.1389	0	0.1688	0	0	0.0689	
0.3533	0	0	0	0	0.2876	0	0	
0.6811	0.1039	0.1772	0	0	0.1546	0	0	0
	0.209	0	0	0.1272	0	0.2164	0	
0.2099	0.1473	0	0	0	0	0	0	
0.2205	0	0	0	0	0.3003	0	0	0
	0	0	0	0	0	0	0	0
	0	0	0	0	0	0.3571	0	

0.2558	0	0	0	0	0.2134	0	0	0
	0	0	0.6856	0	0	0	0	0
	0							
C1orf101	0.0891	1.258	0.1467	0.1751	0.0893	0.2678	0.1062	
0.008	0.0152	0.0093	0.0239	0.0354	0.0727	0.4525	0.126	
0.1673	0.1481	0.0913	0.1473	0.0522	0.0596	0.0016	0.068	
0.2707	0.0442	0.4435	0.0118	0.3408	0.0276	0.0661	0.2933	
0.2219	0.1797	0.1197	0.0532	0.0129	0.1253	0.0426	0.3047	
0.3072	0.0724	0.1432	0.1174	0.1922	0.0709	0.0602	0.1339	
0.1443	0.388	0.2707	0.1077	0.1025	0.1547	0.0276	0.0609	
0.1751	0.1952	0.1812	0.0464	0.1651	0.0644	0.0161	0.3756	
0.1871	1.1621	0.3956	0	0.1496	0.1406	0.1293	0.2133	
0.1529	0.3559	0.0851	0.202	0	0.2656	0.0641	0.0958	0
	0.0607	0.0446	0.136	0.0795	0.1044	0.1475	1.3168	
0.3065	0.0582							
C1orf105	0	0	0	0	0.0582	0	0	0
	0	0	0	0	0	0	0	0
	0.1866	0	0	0	0	0	0	
0.0661	0	0	0	0	0	0	0	0
	0.1164	0	0	0	0	0	0	0
	0	0	0	0	0	0	0	
0.1138	0	0	0.0949	0	0	0	0.029	0
	0.0893	1.1583	0	1.1603	0	0	0	0
	0	0	0	0	0	0	0	0
	0	0	0	0	0	0	0	0
	0	0	0	0	0	0	0	0
	0							
C1orf106	0.4126	0.4074	0.0135	2.985	3.3261	1.9631	0.0345	
1.1043	0.0202	1.6174	0.1121	0.0778	0.0993	0.0895	1.6968	
1.3686	0.1567	0.1079	1.0234	0	0.0541	0	2.1181	
1.0696	2.0555	0.1444	1.6211	0.0181	0.9299	1.3508	1.889	
0.4409	0.0521	0.0846	0.406	1.2649	1.1804	0.005	0.2373	
0.0528	2.456	3.6489	1.1251	0	0.5471	0.6458	0.0108	
1.9268	3.5895	0	0	3.1481	0.0254	0.7735	0.9215	
2.2181	0.2671	3.183	1.5778	3.3758	0.068	1.5968	1.0559	
0.1556	0.7923	0.0953	1.2541	0.0398	0.0409	1.2814	0.0886	
1.0944	0.0478	0.0737	2.1386	0.0576	0.0442	1.9612	0.368	
0.0795	0	0.1019	0.2564	0.9511	3.132	0	0.0942	
0.3831	0.2666							
C1orf109	3.4141	3.8527	3.5773	3.7235	3.5332	3.4598	4.124	
3.3158	3.7508	3.5926	3.1531	3.4316	3.5396	3.6167	3.5676	
2.9736	3.5408	3.167	3.6391	3.6527	3.8527	3.6949	2.7847	
3.5629	3.2963	3.7264	3.0103	3.5345	3.3163	3.6928	3.138	
2.8208	3.975	3.098	3.3196	3.8817	4.2497	3.2165	3.655	
3.7431	3.2325	2.5821	2.8033	2.9287	3.4137	3.4438	3.0741	
4.4782	3.1541	3.819	2.9536	3.3653	3.1577	4.1208	3.6003	
3.5187	2.9609	4.1434	3.7539	4.1729	3.3476	3.6373	3.1649	
3.8077	3.3714	3.3018	3.1396	2.9798	3.7397	3.2679	2.8265	
3.4095	4.0471	3.4048	3.0029	2.838	3.8429	2.8249	3.5933	
3.6848	3.3074	2.666	2.6239	2.6088	2.9746	3.6106	2.7597	

2.9748	2.7108							
C1orf111	0	0.1901	0.0795	0	0	0.0775	0	0
	0.0373	0	0.0271	0	0	0	0	0
	0	0	0	0	0	0	0	0
	0	0	0	0	0	0.1657	0.0596	
0.0101	0	0.0492	0	0	0	0	0	0
	0	0.0718	0.1228	0.147	0	0	0.0734	0
	0	0	0	0.0957	0	0	0	
0.1481	0	0	0	0	0	0.0497	0	0
	0.0783	0	0	0	0	0	0	0
	0	0.0113	0	0	0	0	0	0
	0	0	0	0.0625	0	0	0	0
	0							
C1orf112	4.552	5.733	5.1296	4.545	5.171	4.6035	4.4411	
4.0303	5.1624	4.2338	5.3622	3.8606	4.6456	4.1058	5.1868	
3.8019	3.4755	4.8484	5.0856	3.6618	4.8873	5.0338	4.4877	
5.2828	4.2597	6.2544	1.9235	5.706	5.0216	4.4214	4.5649	
5.1941	4.9474	4.2733	5.2814	5.3148	4.9918	3.2238	2.8569	
3.9441	4.6891	3.5639	5.2566	4.8873	4.1569	4.4939	4.354	
4.5752	2.1728	4.7662	3.7254	5.3545	4.4407	3.078	5.6654	
5.1224	3.0907	4.9582	4.3884	5.4215	4.9582	4.7936	4.6441	
5.3144	5.0145	3.7122	4.8927	1.7735	5.0475	5.3174	3.7067	
5.6616	5.0696	4.7566	3.9321	4.4457	5.0629	4.3219	5.2035	
3.7897	4.6715	3.1957	3.5407	4.4103	5.2377	4.5367	4.1447	
5.3755	4.1465							
C1orf115	3.1373	3.1894	3.3207	1.5757	0.1121	0.7137	0	
1.5121	1.9827	2.2716	2.7256	1.8628	1.2228	1.9869	0.1019	
1.6463	0	2.1915	2.7055	0.0754	2.8715	0.1619	0.8878	
3.0099	0.2849	1.1349	1.7911	3.2541	0.4365	3.3214	3.7828	0
	0.8104	3.5012	0.254	2.4042	1.1951	3.387	1.3874	
2.377	4.3168	1.5845	0.3124	2.1451	1.5295	0.2439	2.3459	
3.851	0.6488	0.0902	2.8702	1.656	3.4574	2.5951	2.7099	
3.22	2.8741	1.4027	3.2232	0.5229	1.6935	0.1101	3.2127	
1.6724	2.3537	0.4497	3.4543	1.8448	2.2359	0.2427	0.5025	
0.7549	0.2586	0.1298	0.5071	2.8293	3.6681	0.266	3.3582	0
	3.1238	0.2739	2.3064	2.2329	0.2427	3.0343	0.9273	
0.8833	1.4128							
C1orf116	0.3544	0.1853	0.1734	0.078	0.0537	0	0.0669	
0.0063	0.1638	0.436	0.0614	0.0016	0.011	0.0867	0.0083	
0.0098	0.0266	0.2566	0.0684	0.0409	0.0924	0.252	0.1671	
0.1874	0.0148	0.0568	0.0093	0.0463	0.1914	0.4324	0	
0.2076	0.2126	0.1912	1.9486	0.0368	0.1017	0.5528	0.0035	
0.216	0.7189	0	0.0334	0.214	0	0.0048	0.7534	
0.1102	9.00E-04	0.5819	0.0476	0.1285	0.0753	0.0275	0	
0.0809	0.6491	0.0368	0.1244	0.0404	0.0199	0.0326	0.1409	
0.0426	1.3342	0.0121	2.9496	0.0379	0.0108	0.099	0.0437	
0.0443	0.0717	0.0873	0	0.0148	0.1264	0.3332	0.2123	
0.381	0.0153	0.0642	1.0681	0.0261	0.0343	0.1188	0.1676	
0.0666	0.2811							
C1orf122	4.9624	5.2601	4.9676	5.1716	5.5844	4.9907	5.0813	

4.8792	4.8481	4.6981	4.8016	5.1193	5.0478	4.4734	5.1729	
4.692	4.2984	5.1098	4.7552	4.8544	4.0379	4.7975	5.47	
5.3581	4.2777	6.1343	5.5058	5.5429	5.3855	6.0687	5.1572	
5.516	5.3979	5.2666	5.7279	4.886	4.9073	5.3528	4.8005	
5.0425	5.2156	5.5735	5.2389	4.4647	5.3994	4.5609	4.4379	
4.4437	4.9657	4.9644	5.0459	5.2169	5.4254	5.1108	5.3487	
5.0914	4.1319	5.7395	4.3808	5.2189	5.2767	5.7403	4.601	
4.6494	4.6406	5.7693	5.4038	5.5317	4.9129	4.5092	4.8854	
4.456	5.2582	4.4382	4.3534	4.6785	5.3353	5.1638	4.9515	
5.0093	4.7832	5.0952	5.4606	4.5741	5.6212	5.1303	5.2467	
5.0617	5.1146							
C1orf123	4.3638	4.7043	4.309	3.8638	4.1866	3.6437	3.5337	
3.7235	4.7921	4.4741	3.8667	4.2423	4.0106	4.5891	3.6437	
3.8259	4.1576	4.1513	4.8632	4.2353	3.7204	3.8296	3.6902	
4.052	3.5344	4.415	4.0995	3.8404	4.3972	4.3953	4.8926	
4.1802	4.4692	4.5284	3.7602	4.1667	4.51	4.1768	4.1614	
4.1942	4.2353	4.3059	3.4111	5.0921	4.2458	4.3051	4.2218	
3.8388	4.3544	4.6764	4.4609	4.2651	4.4665	4.4995	4.7651	
4.5629	4.795	4.4227	4.0646	4.2265	4.1993	4.6473	3.9943	
4.5096	3.8904	4.0472	4.4244	4.2235	4.1638	4.2164	3.6514	
4.298	4.3157	3.6432	4.2704	3.9002	4.2252	3.8642	4.0067	
4.6187	4.5858	3.9474	3.7706	2.9428	4.8084	4.6328	4.7089	
4.6137	4.6245							
C1orf127	0.2914	0.0326	0.1831	0	0	0	0.0669	0
	0.042	0.0476	0	0	0	0	0	
0.0113	0.0544	0.2181	0	0	0	0.006	0.0049	0
	0	0	0	0.3174	0	0.1543	0.1738	0
	0	0	0.1578	0	0.0073	1.1758	0.0629	0
	0.4918	0	0	0	0	0	0	
0.0316	0	0	0	0	0	0.0313	0.0585	
0.0527	0.0453	0	0	0	0.0286	0	0.0519	0
	1.1486	0.0042	0.2689	0.3692	0.0982	0.0405	0	
0.0164	0	0.0155	0	0.1925	0.0086	0.0199	0.039	
0.267	0	0	0	0.0216	0	0	0.1246	
0.0989	0.3915							
C1orf131	3.339	3.3789	2.7772	3.8301	3.7086	2.6477	2.6057	
2.6199	2.3507	2.7198	1.7357	2.9215	3.0153	2.811	3.4108	
2.5102	2.3775	3.4962	2.6369	2.7521	2.5963	3.0574	3.3738	
3.3138	3.9857	2.9375	4.1566	3.1535	3.236	3.3457	3.1744	
2.5842	2.9334	2.3865	3.0709	3.2153	3.1275	3.2551	4.1886	
3.7795	3.2196	3.2122	3.0801	2.9343	3.0517	3.1905	3.3364	
3.7291	2.7434	2.931	3.0884	4.1059	3.001	3.153	3.6916	
3.948	2.8936	3.4642	3.3357	3.6351	3.5148	3.027	3.6017	
3.0586	3.9454	1.7647	2.9443	2.1271	2.4352	3.5228	2.8676	
3.271	2.6941	3.3515	3.6098	2.5921	3.2527	4.0835	3.0113	
2.1312	3.0406	2.9118	2.0579	3.5444	3.7224	3.091	2.1686	
2.5851	2.5455							
C1orf141	0	0	0	0.0285	0	0	0	
0.0105	0	0.015	0	0	0	0	0.018	0
	0	0.0249	0	0	0	0	0	

0.0183	0.0062	0.0114	0.0164	0.0416	0	0.0152	0.0107	
0.0014	0	0	0.0014	0	0.0061	0.0291	0	0
	0	0.0013	0	0	0	0	0	0
	0	0.0511	0	0	0.039	0	0	
0.05	0	0.0259	0	0.0476	0.0212	0	0	0
	0	0	0	0	0.013	0	0	0
	0	0.0019	0	0	0	0	0	0
	0	0	0	0	0	0	0	0
	0							
C1orf146	0	0	0	0	0	0	0	0
	0	0	0	0	0	0.081	0	0
	0	0	0	0	0	0	0	0
	0	0	0	0	0	0	0	
0.0662	0	0	0	0	0	0	0	0
	0	0	0	0	0	0	0	0
	0	0.2765	0	0	0	0	0	0
	0	0	0	0.201	0	0	0	0
	0	0	0	0	0	0	0	0
	0	0	0	0	0	0	0	0
	0	0	0	0	0.0992	0	0	0
	0							
C1orf158	0	0	0	0	0	0	0	0
	0	0	0	0	0	0	0	0
	0	0	0	0	0	0	0	0
	0	0	0	0	0	0	0	0
	0	0	0	0	0.0783	0	0	0
	0	0	0	0	0	0	0	0
	0	0	0.0518	0	0	0	0	0
	0	0	0	0	0.0248	0	0	0
	0	0.0377	0	0	0	0	0	0
	0	0	0	0	0	0.0351	0	0
	0.0415	0	0	0	0	0	0	0
	0							
C1orf159	2.0797	2.2054	3.8774	1.1592	2.3785	1.9244	1.4798	
2.3826	1.7653	1.7638	2.2017	0.805	2.3556	1.5875	2.4385	
2.0071	2.0205	0.9548	1.4504	2.8904	3.487	2.0399	1.4651	
2.0846	2.1224	1.7355	0.799	2.022	1.8158	1.86	2.3966	
2.9556	2.7906	2.2281	1.8814	1.0604	1.9841	2.5906	1.8017	
1.5868	2.3091	1.4613	1.7843	1.3864	1.4371	1.6536	1.2953	
1.5759	2.1649	1.4594	1.7457	1.0678	1.8294	2.1858	2.4915	
3.0011	1.4455	3.0317	2.7606	2.8537	1.9929	1.92	1.8678	
2.0308	2.0023	1.6859	2.035	1.839	2.3009	2.1444	1.9759	
2.3084	1.714	2.4039	0.909	2.4689	2.6814	1.484	1.583	
2.4793	1.5421	1.7844	1.7252	1.5495	2.8728	3.4111	1.6668	
2.5389	2.388							
C1orf162	0.7706	0.2269	0.2953	0.4657	0.0117	0.4206	0.1814	
0.0868	0.1362	0.0399	0.1206	0.2112	0.0716	0.5127	0.3476	
0.1652	0.6148	0.151	0.4313	0.5755	0.2525	0.0905	0.0759	
0.0169	0.0545	0.6881	0.0457	0	0.0371	0.4907	0.1313	
0.1369	0.36	0.4851	0.4949	0.1956	0.1437	0.3165	0.3516	

0.6203	0.1237	0.0748	0.1795	0.1731	0.1428	0.0923	0.2091	
0.4313	0.3214	0.6275	0.278	0.1926	0.378	0.2154	0.0971	
0.2885	0.1906	0.3059	0.2854	0.0909	0	0.0776	0.2126	
0.8452	0.1643	0.1063	0.1294	0.1806	0	0	0	
0.4488	0.2118	0.2341	0.2554	0.2567	0.5357	0.0829	0.2785	
0.3121	0.2663	0.2733	0.4674	0	0.0945	0.5632	0.1988	
0.1497	0.236							
C1orf167	0	0.1781	0	0	0	0	0	0
	0	0	0	0	0.026	0	0	0
	0	0	0	0	0	0	0	
0.0408	0	0	0	0	0	0.1658	0.1712	0
	0	0	0	0	0.0376	0	0	0
	0	0	0	0	0	0	0	0
	0	0	0	0	0	0.0339	0	0
	0.0138	0.0142	0	0	0	0	0	0
	0	0.0037	0.5265	0	0.0056	0	0	0
	0	0	0	0	0.0104	0	0	0
	0	0	0	0	0	0	0	0
	0							
C1orf168	0	0	0	0.0632	0	0.1929	0.0251	
0.0329	0	0	0.0381	0.046	0.0085	0	0.0027	
0.0093	0.0506	0.0655	0.097	0.0433	0	0	0.0284	
0.0829	0.0022	0	0.062	0.0541	0.0396	0.0401	0.0108	0
	0	0.0159	0	0.007	0	0	0	
0.0674	0.1857	0.0611	0	0.3233	0	0	0.0309	
0.0252	0	0	0.0072	0	0.1962	0	0	
0.0428	0	0	0.0171	0.0215	0.0826	0	0	0
	0.0414	0.0054	0.0297	0	0.0147	0	0	
0.0392	0.0377	0.667	0.0592	0	0.051	0	0.0085	0
	0.0163	0	0	0	0.0249	0.0391	0	0
	0							
C1orf174	3.3315	4.3495	4.0927	3.9407	3.5948	3.5065	3.802	
3.8154	4.2731	3.2146	4.1789	3.7078	3.7925	4.0701	3.4872	
3.6886	3.7268	3.457	3.9532	3.6125	4.2354	3.0515	3.0798	
4.2873	3.3773	3.345	3.8041	3.8061	3.6657	4.6164	3.8582	
3.7681	4.7615	3.7713	3.2608	3.7165	3.0726	2.8076	3.2637	
3.7434	2.8114	3.1799	3.7331	3.5061	4.0369	3.7541	4.1301	
2.9935	3.1765	4.6048	4.3219	3.3921	4.3296	3.911	4.0429	
4.2199	3.9312	4.0847	4.0172	4.1066	3.5077	3.5107	3.3196	
4.2697	3.911	3.2453	3.7511	3.2647	4.0798	3.1351	3.2429	
3.9961	3.8811	4.0866	3.2237	3.9018	4.3077	2.9855	3.7387	
4.1901	3.8774	2.9265	3.3746	2.6915	3.8118	4.7384	3.2561	
3.776	3.6325							
C1orf185	0	0	0	0	0.0231	0.2734	0	0
	0	0	0	0	0	0	0	0
	0	0	0	0	0	0	0	0
	0	0	0	0	0	0	0	0
	0	0	0	0.0312	0	0	0	
0.0337	0.0443	0	0	0	0	0	0	0
	0	0	0	0	0	0	0	0

	0	0	0	0	0	0	0	0
	0	0	0	0	0	0	0	0
	0	0	0	0	0	0	0	0
	0.1764	0	0.0767	0	0	0	0	0
	0							
C1orf186	0	0	0	0	0	0	0	0
	0	0	0	0	0	0	0	0
	0	0	0	0	0	0	0.0137	0
	0.0192	0.0296	0	0	0	0	0	0
	0	0	0	0	0	0	0	0
	0	0	0	0	0	0.0371	0	0
	0	0	0	0	0	0	0	0
	0.1	0	0	0	0	0	0	0
	0	0	0	0	0	0	0	0
	0	0	0	0	0	0	0	0
	0	0	0	0	0	0	0.1015	0
	0							
C1orf189	0	0	0	0.2088	0	0.1334	0.0649	0
	0	0.0818	0.0979	0.1788	0	0	0.0606	
0.062	0	0	0	0	0	0	0	0
	0.0482	0.064	0	0.4617	0	0.0793	0.0615	0
	0	0.1844	0	0.1551	0.1049	0.1044	0.2412	
0.0796	0.0756	0	0	0.1014	0	0.08	0.2191	
0.108	0	0.2209	0.0779	0.4511	0.4443	0.0518	0	0
	0	0	0.0834	0.0855	0.3769	0	0.076	
0.1084	0	0	0.1381	0.0401	0.1767	0	0	0
	0	0.0796	0	0.0893	0.5814	0	0	0
	0.1523	0	0.064	0.0802	0.0871	0	0	0
	0							
C1orf194	0	0	0	0	0.2466	0.2152	0	
0.0439	0	0	0	0	0	0.2534	0	0
	0	0	0.1628	0.218	0.2456	0	0.0822	
0.0837	0.0747	0	0	0	0	0	0.1186	0
	0.1748	0.1731	0.2699	0.05	0.2159	0	0	
0.1451	0.0578	0.0631	0	0	0	0	0	
0.1702	0.1412	0	0	0	0.5259	0	0.2992	0
	0.1597	0	0	0.261	0	0	0	0
	0	0	0	0.1481	0	0	0.2153	0
	0	0	0	0	0.2354	0	0.3437	0
	0	0.0868	0	0	0	0	0	0
	0							
C1orf198	6.3096	5.7306	3.0582	3.8907	4.0039	3.2304	4.9012	
4.0721	3.3763	3.209	4.0021	3.931	2.21	2.8591	5.6823	
3.703	2.5951	5.173	4.0727	3.2251	4.4618	5.1617	5.6124	
4.6191	4.4717	4.5248	5.6495	1.9843	3.4193	4.8963	4.5585	
5.133	3.0022	3.9149	5.1823	3.8102	4.6771	3.6196	5.4052	
4.4712	3.8046	2.5047	6.0226	3.5548	3.1144	2.8471	2.9685	
2.9732	3.8083	2.7736	4.5813	4.3379	3.7397	3.3104	4.1049	
5.0464	3.3363	6.8659	5.3355	6.8982	3.8635	4.9862	5.3075	
2.9928	5.1225	1.6778	5.6363	4.0728	2.7648	4.7036	1.7376	

4.079	4.2281	5.1208	4.9752	3.672	3.474	5.6697	3.3138	
2.4687	3.4118	3.1984	4.3387	4.8277	4.7707	3.8807	5.814	
5.3556	5.2233							
C1orf204	0.0728	0.694	0.2659	0.4613	0.1947	0.4756	0.4804	
0.0454	0.3677	0.0518	0.5871	0.0924	0.4181	0.3797	0.1954	
0.458	1.0243	0.4965	0	0.0381	0.1504	0.6135	0.3279	
0.0577	0.1853	0.9532	0.2921	0.0979	0.5137	0.5855	0.7549	
0.2826	0.3309	0.4315	0.0108	0.3696	0.1464	0.0896	0.2895	0
	0.4652	0.5321	0.316	0.6856	0.4666	0.4146	0.6243	
0.3099	0.1819	1.6038	0.7258	0.8139	0.841	0.1608	0.527	
0.1212	0.6945	0.0553	0.1489	0.0521	0.4675	0.3248	0.0159	
0.5749	0.0637	0.6698	0	0.7081	0.3491	0.5678	0.3788	
0.3324	0.3504	0.1847	1.1976	0.2018	0.2439	0.623	0.2535	
0.0428	0.6312	0.2117	0.4441	0.7453	0.3003	0.6476	0.8562	
0.6847	0.5238							
C1orf21	1.9408	3.1877	2.1768	2.5082	1.4989	3.6415	3.1622	
2.7047	3.6495	3.0673	3.7384	3.1589	1.3229	2.8148	2.5851	
3.0582	2.5501	3.2509	3.7564	2.0706	1.3448	2.7106	2.599	
3.1232	2.065	3.0834	3.568	3.6065	3.1703	3.9661	1.1837	
2.9954	2.7899	4.0012	3.4342	2.5181	1.2862	4.8268	3.1917	
3.2896	3.4478	1.9535	2.76	2.9097	1.7667	0.1368	3.2369	
3.0534	2.1034	2.9317	3.1314	2.7793	3.6852	2.7774	1.579	
2.8914	2.092	2.2224	3.5564	2.153	2.6784	1.4377	3.2212	
3.427	4.9937	1.7139	3.0881	1.1314	4.1771	2.2826	3.2883	
3.1595	2.1923	1.8117	3.4274	3.0676	2.7949	3.4959	4.0772	
3.9326	3.5709	3.0917	3.3036	3.3202	2.023	2.7323	2.6321	
2.8362	2.5935							
C1orf210	0.0929	0	0	0	0.0203	0.2815	0.0368	
0.1739	0.073	0.0423	0.028	0	0.0937	0.0908	0	0
	0.3534	0	0.2977	0.1645	0.138	0	0.0201	
0.1306	0.0542	0.1394	0.1859	0.146	0.0402	0.0475	0.0645	
0.096	0	0	0.016	0.0362	0.0575	0.1499	0.0681	
0.0938	0.0733	0.0392	0.1189	0.0514	0.0327	0.0903	0	0
	0.0731	0.1254	0.0426	0.0504	0	0.2548	0	
0.1168	0.1221	0	0.1816	0.1511	0.0193	0.0828	0.0893	
0.1453	0	0.0873	0	0.0719	0.1073	0.0863	0	
0.0384	0.1618	0.041	0.0715	0	0.0632	0.0405	0.0624	
0.1842	0.0951	0.0588	0	0.0955	0.0599	0	0.0601	
0.1096	0.2106							
C1orf216	3.2239	4.0219	3.9952	3.8598	3.1877	4.8408	4.8142	
4.0451	3.9107	3.5924	3.0329	4.4053	3.0036	4.5482	3.502	
5.3326	3.6083	4.4813	3.8002	3.9795	4.0327	3.1672	3.7748	
3.7179	3.5335	4.3903	3.3472	4.4891	4.2515	3.706	4.5008	
4.5585	4.2274	4.1373	4.9274	4.0692	3.8262	3.7288	3.426	
3.8898	3.6556	4.1279	3.6221	4.3017	4.323	3.9944	3.6295	
5.0735	4.3319	3.7637	4.0322	3.8731	4.4846	3.7744	3.5255	
3.6513	3.0205	3.3486	3.9014	3.5141	3.4584	3.4671	2.491	
4.5026	3.2558	3.8311	3.3706	4.7854	4.3689	3.4561	3.8284	
4.3592	4.1818	3.9236	4.7194	4.7734	4.3556	3.9046	4.2208	
3.8524	4.3335	4.0387	4.6519	3.9695	4.2677	4.3812	4.4078	

4.046	4.2986							
C1orf220	0.6178	0.1763	0.4567	0.2635	0.2594	0.3057	0.1566	
0.6422	0.3063	0.2237	0.0809	0.0909	0.3584	0.4008	0.317	
0.2521	0.3153	0.521	0.3573	0.2633	0.7765	0.1713	0.31	
0.4192	0.1759	0.2322	0.1402	0.1133	0.3291	0.0585	0.2129	
0.7974	0.2082	0.146	0.1385	0.1098	0.1288	0.2958	0.091	
0.3555	0.3281	0.1334	0.206	0.3212	0.1148	0.1031	0.2265	
0.3462	0.4106	0.5191	0.3417	0.5152	0.284	0.2498	0.4044	
0.496	0.2324	0.145	0.1895	0.1494	0.5048	0.1601	0.2158	
0.502	0.2999	0.1381	0.4057	0.0903	0.2811	0.213	0.1921	
0.4057	0.1116	0.1246	0.2531	0.1994	0.4125	0.1329	0.1718	
0.045	0.1983	0.4199	0.613	0.7374	0.1808	0.4105	0.1184	
0.2187	0.1442							
C1orf226	1.7979	1.9364	1.5238	0.9505	1.8134	2.8975	1.0093	
1.198	0.7077	1.2083	2.5558	0.7826	0.767	0.442	1.3677	
2.9528	0.1362	2.3883	0.97	2.1793	1.1533	0.7393	0.7735	
1.5682	1.0829	1.1096	2.6741	0.6761	0.6767	0.8087	0.8749	
1.3938	0.8385	0.3069	0.2527	1.3368	0.2264	0.6063	0.9471	
1.0682	0.8149	3.5754	2.0208	2.0751	1.1341	1.0455	1.0669	
0.1356	1.7337	0.9723	0.558	2.1668	0.6634	0.0602	0.9177	
1.6163	1.2126	0.7359	1.4679	0.7659	0.9938	0.1218	2.3243	
1.0737	0.5609	0.3881	0.2992	0.311	0.3096	2.0549	0.3897	
0.8584	0.8281	2.2864	1.834	1	2.2671	1.2961	1.2992	
1.5728	1.0296	1.8028	0.8136	0.9881	2.5559	0.6676	0.0465	
0.0498	0.1179							
C1orf228	0.106	0.1041	0.0465	0.0463	0.0326	0.0422	0.3866	
0.0914	0.0143	0.0783	0.0442	0.0624	0.3172	0.0514	0.1086	
0.2381	0.1138	0.0398	0.0722	0.1086	0.04	0	0.0639	
0.1148	0.0601	0.2915	0.0589	0.8851	0.0437	0.3243	0.7428	
0.0714	0.0598	0.5221	0.3561	0.0375	0.6843	0.0303	0.1413	
0.0205	0.3866	0.2271	0.1127	0.154	0.1414	0.0825	0.1993	0
	0	0.3088	0.252	0.0843	0.1083	0.2192	0.1297	0
	0.1049	0	0.206	0.0552	0	0.1435	0	
0.2357	0.3807	0.5475	1.0062	0.1628	0.3998	0	0.1293	
0.0861	0.0545	0.0351	0.0714	0.0547	0.1502	0.1138	0.0619	
0.2475	0.2466	0.1014	0.1511	0.0539	0.0688	0.1573	0.1977	
0.33	0.2527							
C1orf234	0	0.1569	0	0	0	0.1907	0	
0.0967	0	0	0.2512	0.0552	0.0877	0.3385	0.0872	
0.0916	0	0.3108	0.2504	0	0	0	0.1197	
0.2296	0	0	0	0.1991	0	0.2809	0.1528	0
	0.5484	0.2478	0	0	0	0	0.1625	
0.1116	0	0	0	0.2419	0	0	0	0
	0.1066	0.6577	0	0.2092	0	0	0.1795	0
	0	0	0	0	0	0.1582	0	0
	0.1794	0	0.4691	0.0864	0.2369	0	0	0
	0	0	0	0	0.1276	0	0.2038	
0.4911	0.1581	0.1949	0	0	0.4621	0	0.2794	0
	0							
C1orf27	3.0576	2.6601	2.2817	3.0764	2.7131	3.3542	2.9292	

2.6563	2.5261	2.5092	2.1909	2.249	2.4139	2.4859	2.836
2.8704	2.4081	3.3897	2.5412	2.5843	2.3975	3.1402	2.6849
2.7631	3.236	2.7875	3.2923	2.4641	2.3187	2.3512	2.2207
2.3275	2.1052	2.1718	2.7842	2.7671	3.2829	2.2044	2.827
2.8103	2.499	2.097	2.4135	2.1776	2.7615	3.0461	3.0646
2.2947	2.5232	2.8111	2.9711	3.2829	2.9135	2.6971	2.5201
2.5235	2.6801	2.3132	3.3801	2.6941	2.754	2.5291	2.798
2.1325	3.0005	2.301	2.7142	2.459	2.6194	2.942	1.8788
2.4494	2.5196	2.1216	3.3285	2.3939	2.9679	3.2259	2.7592
2.1485	2.3481	2.6184	2.8336	3.0417	2.4334	2.7088	2.5187
2.3027	1.9549						
C1orf35	3.0779	3.45	3.233	2.8954	3.8168	2.8487	1.9343
3.3794	1.834	2.563	2.6672	2.0153	2.5901	1.8399	3.2084
2.7711	2.7981	3.0649	2.8054	3.1706	3.3769	3.1254	3.208
3.3358	3.241	3.0574	2.6987	3.475	2.7178	3.442	3.1695
3.5822	2.9523	2.7059	3.2162	2.4864	4.2077	3.4921	3.3387
1.7733	3.4618	2.6086	3.2234	3.0273	2.5668	2.8462	1.9586
3.565	3.1199	2.7071	3.0372	2.2689	2.4835	2.5233	3.9557
3.8994	2.8636	3.7383	3.4049	4.0284	3.2697	3.8156	3.8888
2.5716	3.6771	3.0318	3.6048	3.2241	2.5617	3.3067	2.6077
3.3248	3.119	3.0609	3.0728	2.5399	3.5411	3.5277	2.8729
2.3183	2.941	2.6425	2.922	3.1767	4.1338	3.8309	2.7737
3.3208	2.9868						
C1orf43	7.736	7.1861	7.3965	7.9561	7.4774	6.5422	7.4972
7.2941	6.5969	7.9561	7.7271	7.2561	6.5591	6.8663	6.6968
6.7459	6.7858	7.7219	7.1392	7.1471	6.9727	7.0619	6.5257
7.6099	7.5326	7.0007	7.8731	7.3965	6.8128	7.5974	7.0086
7.2437	6.5606	6.8621	6.4588	7.9268	7.736	7.3323	7.4452
7.0063	7.8614	6.7919	7.046	6.9632	6.9632	7.7811	7.2561
8.2799	6.8146	6.8266	6.9286	7.5926	7.4565	7.561	7.4338
7.113	6.3918	7.3207	7.3781	7.2575	6.9988	7.2213	7.6784
6.7562	7.8587	7.1006	7.4837	7.1107	7.7533	7.016	6.3788
7.0474	7.0085	6.5233	6.8258	6.6356	7.504	7.3845	7.3814
7.0925	6.9302	6.8122	7.7694	7.3695	6.4082	7.0303	7.3845
7.1124	7.1969						
C1orf50	2.5186	2.84	2.5352	2.5317	2.2858	2.3742	2.9169
2.3955	2.9156	2.448	2.4576	2.3	2.5976	2.6735	2.1513
2.6335	2.6167	2.5818	2.7559	2.7333	2.1568	2.0386	2.0047
2.732	2.4856	2.9516	3.1339	2.7759	1.7337	3.1752	3.0171
2.5535	3.1358	2.9173	2.8342	3.0461	2.5801	2.8567	2.047
2.6778	2.4572	2.6932	2.6572	2.1002	2.6983	3.3209	2.7359
2.3857	2.2067	3.2259	2.5796	2.6525	2.9169	2.7929	2.7485
2.7225	2.8096	2.9497	2.6838	2.7327	2.1609	2.5198	2.0374
2.6132	2.5977	2.8526	2.4833	3.1106	2.6132	2.3269	2.1451
2.7181	2.3878	2.2109	1.8532	2.3622	3.2767	2.2658	2.7562
2.4735	2.8202	2.2118	2.7721	1.8464	2.8302	2.5646	2.6069
2.2618	2.6421						
C1orf52	3.5242	4.3795	4.1844	3.794	3.7222	3.3322	4.1238
3.6493	2.4029	3.2228	3.6608	4.7526	3.9328	3.414	3.356
3.5364	3.7736	3.0756	3.6429	3.5514	3.6929	3.9987	3.7191

3.277	3.2504	4.1668	4.5564	3.9339	3.5939	3.1975	3.2135
4.4237	4.2996	3.7583	3.0597	3.209	4.0257	4.2645	4.1546
2.7691	4.2522	3.6063	3.2047	3.4463	2.7784	3.6342	3.0115
4.0235	4.1235	4.5782	3.4459	3.7037	3.7747	3.2793	3.8511
4.068	3.9505	4.3237	2.6996	4.2887	3.8568	3.847	3.4536
4.0398	3.5908	3.8178	4.0061	3.3065	4.2553	3.8154	2.941
3.859	4.0686	4.1584	3.9298	3.1369	4.2753	3.6263	3.9081
3.9599	4.5635	4.3917	3.2001	2.961	4.3713	4.1042	3.0567
3.4341	3.6491						
C1orf53	1.6035	1.6176	1.8604	3.0228	2.7465	2.3808	1.6817
2.2361	0.7242	2.0369	1.429	1.8772	1.4073	2.9593	1.3135
1.6919	1.8563	1.8449	1.9965	1.4924	1.8592	0.7148	2.0925
1.9829	1.1797	2.3377	2.0152	1.2628	2.0396	2.113	3.0894
2.3321	1.6528	0.6691	1.29	1.5772	1.6619	2.4698	1.8557
2.8924	0.9454	1.8026	0.8433	1.7499	1.5266	2.6849	2.1799
2.0785	0.9299	3.1004	0.5174	2.3907	1.6701	2.0002	3.1115
2.0723	1.5084	2.6062	2.1452	2.3249	2.3529	1.596	1.7614
0.9021	2.0579	2.9761	2.3161	1.9395	1.9149	1.4155	1.4299
1.0174	2.0589	0.083	2.0696	0.6926	1.904	1.8026	1.8556
0.9651	1.4578	1.3926	2.1416	1.4637	3.2544	1.9172	1.016
1.0853	0.4968						
C1orf54	2.3164	2.351	1.6186	1.2317	0.7988	1.2143	1.1928
0.7048	1.3607	1.8424	2.7924	1.0513	0.2107	1.3558	0.2736
1.5716	0.2509	2.1904	1.9502	0.1451	1.1805	0.8408	1.1315
0.4466	1.6946	1.5093	3.3803	1.3248	0.3092	2.2534	0.7166
0.582	0.7015	2.4636	0.7424	1.8784	0.3457	2.0047	1.0977
0.9152	3.8151	3.8544	0	0.8012	0.6042	0.1387	1.3092
0.4133	0.4914	0.7027	1.2591	2.3159	2.1031	2.8504	0.4694
1.4817	1.0975	0.7312	1.5082	0	0.5716	1.346	3.3505
1.2961	0.8648	0.2909	1.4655	3.4389	1.0648	0.4084	1.0531
0.7992	1.9353	0.921	0.9177	1.6722	2.1485	3.2089	1.2184
1.037	1.2974	0.1919	1.1732	3.0735	0.5987	1.3842	2.1794
1.5752	2.6216						
C1orf56	1.8672	1.9891	1.2893	1.6636	2.1202	1.1958	1.7266
1.6693	1.0327	1.837	2.1533	1.7151	1.3037	0.7255	1.0354
1.3346	1.3306	2.1595	1.2279	1.6319	1.0536	0.8338	2.3314
0.9056	1.539	1.4112	2.1127	1.7609	1.9418	2.1577	0.3704
2.0283	1.6061	1.0858	1.137	1.9293	2.7152	2.6817	1.4847
0.6154	2.7482	2.6427	1.5297	1.2439	1.9801	2.4252	2.5314
2.0592	2.2115	1.482	2.4594	2.1612	1.0891	1.5051	0.314
1.5377	1.5282	1.787	1.5797	1.7913	2.3564	1.507	2.6275
1.236	1.9378	2.1326	1.7393	1.3501	1.453	2.098	1.7347
1.6961	1.8039	1.6261	2.1456	1.2876	1.583	2.0543	2.6517
0.3022	0.8019	2.3781	2.0693	2.5388	2.0918	1.3376	1.2924
1.3972	1.5871						
C1orf61	0.0426	0.4231	0.2187	0	0.0557	0.0141	1.1944
0.0899	0.272	0	0.5236	0.0725	0.0944	0.152	0.054
0.077	0.0027	0.0542	0.2832	0.9278	0	0.4944	0.2421
0.1276	1.2378	0	0.1674	0	0.1467	0.5393	3.2261
0.0906	0.0463	0.1939	0.1578	0.8851	0.3346	0.0273	0.1568

1.2222	0	0	0.8678	0.1097	0	0.013	0.32	
0.287	0.2393	0.0296	0.1179	0.0271	0.2752	0.1924	0.3822	
0.2395	0.1049	1.6919	0	3.4685	0.1826	0.2045	0.0936	
0.1747	0.2561	0.0911	0.7309	0.0531	0.2777	0.1639	0.0952	
0.0552	0.2249	0.1224	0.0829	0	0.1014	0.1611	0.1745	
0.1214	0	0.0502	0.1158	0.4986	0.0694	0.1781	0	
0.2604	0.0472							
C1orf64	0.2472	0.1636	0	0	0.0599	0	0	0
	0	0	0	0	0.0953	0	0	0
	0	0	0	0	0	0	0.0618	
0.0746	0	0	0	0	0	0	0.1624	0
	0	0.1407	0	0	0.1611	0	0	
0.1498	0	0	0	0	0	0.1409	0	0
	0	0	0	0	0	0	0	0
	0.0486	0	0	0.0857	0	0	0	0
	0.1001	0	0.0682	0	0.0929	0	0	0
	0	0	0	1.3028	0.0638	0.04	0	
0.0775	0	0	0	0	0	0	0	0
	0							
C1orf68	0	0	0	0	0	0	0	0
	0	0	0	0	0	0	0	0
	0	0	0	0	0	0	0	
0.1072	0	0	0	0	0	0	0	0
	0.0594	0	0	0	0	0	0	0
	0	0	0	0	0	0	0	0
	0	0	0	0	0	0	0	0
	0	0	0	0	0	0	0	0
	0	0.0321	0	0.0376	0	0	0	0
	0	0	0	0	0	0	0	0
	0	0	0	0	0	0	0	0
	0							
C1orf74	2.1144	2.4283	1.8518	2.6439	2.6152	1.6483	2.2425	
1.8261	1.4791	1.9781	1.1146	2.2318	2.0333	2.584	2.0634	
1.7276	1.4499	2.6066	2.2194	1.8066	2.1163	2.2157	2.4887	
2.461	2.3599	2.1383	0.8846	2.5235	1.9752	2.6943	1.8358	
2.2455	1.78	1.9875	1.7299	2.1664	2.2329	2.0499	2.2349	
2.0169	2.6697	1.9229	2.5629	2.572	2.1502	1.8512	2.4405	
2.2114	1.5611	1.8139	2.4391	3.4705	2.614	1.5902	1.6042	
2.188	1.9385	1.023	2.0107	1.0704	2.2102	2.2578	2.2515	
1.7446	2.5148	1.3179	3.0027	1.5793	2.1996	2.4772	2.0058	
2.155	2.4659	1.9753	2.0987	1.6326	2.0864	1.9795	2.4254	
2.1786	2.1711	1.4657	1.416	2.2574	2.7309	2.5797	1.5873	
1.7437	1.6312							
C1orf87	0	0	0	0	0	0	0	0
	0	0	0	0	0	0	0	0
	0	0	0	0	0	0	0.0049	0
	0	0.0127	0	0	0	0	0	
0.0021	0	0	0	0	0	0	0	0
	0	0	0	0	0	0	0	0
	0	0	0	0	0	0	0	0

	0.0218	0	0	0.0093	0	0	0	0
	0	0	0	0	0	0	0	0
	0.0327	0	0	0	0	0	0	0
	0	0	0	0	0	0	0	0
	0							
C1orf94	0	0	0.0757	0	0	0	0.1006	0
	0	0	0	0.06	0.0221	0	0	0
	0	0	0	0	0	0.0116	0.0992	0
	0	0.1895	0.0522	0	0	0	0	0
	0	0	0.064	0	0	0	0	0
	0	0.0825	0.2175	0	0	0	0	0
	0	0	0	0	0	0	0	0
	0	0	0	0	0	0	0	0
	0	0	0	0.0557	0	0	0.0245	0
	0	0	0	0	0	0	0	0
	0	0.0062	0.0217	0.0144	0	0	0	0
	0							
C1QA	0.2923	0.3046	0.1122	0	0.0464	0.2206	0	
0.0722	0	0.081	0.093	0	0	0	0.2061	0
	0.1344	0.3559	0.2001	0.5869	0	0.2011	0.1247	0
	0.1704	0.0686	0	0.1271	0.074	0	0	0
	0.0861	0.2068	0.0442	0	0.2492	0.0892	0.0682	0
	0.0684	0	0.1091	0.1472	0	0.0891	0	
0.0924	0.0686	0.1982	0.0792	1.262	0	0.1959	0.0927	0
	0.4209	0.0687	0.4543	0.2659	0.0374	0.0627	0.1967	
0.0323	0	0.0359	0.2866	0	0.2391	0.0607	0.0568	
0.2267	0.0904	0.5126	0	0	0.1872	0.1341	0.0644	
0.3807	0.2224	0.2595	0	0.0063	0	0	0.0921	0
	0							
C1QB	0.4756	0.7484	0.3794	0.1489	0.2467	0.1338	0.0775	0
	0.0783	0	0	0.1161	0.0676	0.3255	0.1203	
0.0696	0	0.3928	0	0.3842	0	0.1377	0.1402	0
	0.1272	0.122	0.1895	0.1411	0.081	0	0	
0.0404	0.095	0.2879	0.0461	0	0.1751	0	0.156	0
	0.163	0.183	0.189	0.1644	0	0.1598	0	0
	0	0.2151	0.0863	0	0.1639	0.0729	0.2139	
0.2919	0.2344	0.1087	0.4082	0.1954	0.1592	0	0.2002	
0.2105	0.1864	0.0146	0.2068	0.0218	0.3387	0.0581	0.2823	
0.0735	0	0.5602	0.0092	0.1062	0.0934	0.0183	0.0335	
0.2749	0.4138	0.2891	0.0095	0	0.0394	0.2687	0.1028	0
	0							
C1QBP	7.9978	8.7693	7.9288	9.3895	8.4474	7.6957	7.5675	
7.8707	7.3881	7.9017	6.7107	7.2554	6.5693	7.0389	7.2703	
7.4116	7.6529	7.7713	7.6361	7.8081	8.1645	7.944	7.3439	
8.1645	6.803	7.2095	7.8506	7.1855	7.8707	7.6234	7.3574	
6.6212	7.8755	5.9675	6.8582	8.2278	6.5656	7.1495	7.3741	
8.9159	7.4324	7.1552	6.8007	7.1665	7.0688	7.594	7.9547	
7.6446	6.2416	7.7488	7.4254	8.0339	7.5513	7.4107	8.6404	
7.4655	6.6495	7.8021	7.3343	7.7099	7.479	8.7253	8.5064	
8.2991	7.8358	5.9497	7.0214	6.7745	6.5615	7.5735	7.702	

7.4966	7.6162	7.7588	7.5626	6.9535	7.7672	7.2663	7.7898	
8.2446	8.1704	7.4418	5.7941	6.8236	6.6007	7.3752	6.2469	
7.0417	6.5518							
C1QC	0.3082	0.2317	0	0.1902	0.1894	0	0	0
	0	0.1041	0.0757	0.0659	0.0795	0.5915	0.3462	0
	0	0.2894	0	0.3355	0	0.0709	0.3091	
0.1451	0.1159	0.272	0.1341	0.1672	0.1539	0	0.0763	
0.0799	0.413	0.2028	0	0	0	0.1168	0.0905	0
	0.1416	0.0907	0.1434	0.1175	0	0.1968	0.1635	0
	0	0.2696	0.1021	0	0	0.0908	0.2368	
0.1658	0.2532	0	0.3877	0.2116	0.1826	0.3259	0.0841	
0.0845	0.1933	0.0685	0.1673	0.0186	0.2168	0	0.2125	
0.3051	0.1066	0.5929	0.0086	0.0139	0.1482	0	0.117	
0.2728	0.5001	0.3326	0	0.0181	0.0357	0.167	0.1222	0
	0							
C1QL1	0.5161	1.6893	2.9043	0.7301	1.2567	1.2193	2.1045	
1.8785	1.183	0.2697	0.3462	1.4092	4.6937	0	2.0439	
1.4891	0.6994	0.6339	0.5071	1.8186	0.6256	2.2243	2.3413	
1.1687	3.1463	0.6801	1.7511	1.25	1.6464	0.5449	4.7565	
2.4099	1.3857	0.939	1.696	0.8913	0.4403	1.6188	0.9428	
0.4995	0.9823	0.9371	2.6212	0.4564	2.278	1.2487	0.3597	
1.1109	3.879	0.4823	0.5263	0.4923	0.394	0.7913	3.2459	
3.205	0	1.9814	0	0.9411	2.1553	0.3472	2.6163	
1.1353	0.8635	3.7743	2.5103	0.242	1.6829	2.0484	1.3984	
2.5091	2.0102	1.0361	2.8202	0.155	0.1813	2.4263	2.1283	
1.4758	1.5357	1.3264	0.1049	1.7767	4.0343	0.1832	0.4636	
0.7749	0.9623							
C1QL2	0	0	0	0	0	0	0	0
	0	0	0	0	0.5557	0	0	0
	0	0	0	0	0	0	0	0
	0	0	0	0	0	0	0	0
	0	0	0	0	0	0	0	0
	0	0	0	0	0	0	0	0
	0	0	0	0	0	0	0	0
	0	0	0	0	0	0	0.0322	0
	0	0.3809	0	0.0113	0	0	0	
0.0334	0	0	0	0	0	0	0	0
	0	0	0	0	0	0	0	0
	0							
C1QL3	0.1572	0.0393	0.7046	0.0518	0.1534	0.2287	0.7177	
0.2755	0.05	1.6409	0.128	0	0.0866	2.127	1.4362	
0.4117	0.8086	0.4488	1.7036	0.9227	1.1519	0.0153	0.3077	
0.0763	0.0549	0.0248	0	0.1057	1.2155	0.0325	0.5895	
0.2017	0.0382	0	0	1.3355	0.4059	0.6607	0.2564	
0.5775	0.8034	0.0388	0.9544	0.3254	0.1175	0.4025	0.3947	
0.6643	0.5401	1.1689	1.189	0.2449	0.2557	0.0506	0.8723	
0.2548	0.9952	0.0214	1.7426	0	0.289	0.5962	0.1981	
0.8619	0.7447	0.7344	0.0407	0.1449	0.554	1.834	0.6991	
0.5181	0.7946	0	0.0358	0.7063	1.1937	0.8976	0.0023	0
	0.5573	0.389	0.3388	0.6184	0	0.2697	0.518	

1.0372	1.0668							
C1QL4	0.914	2.3787	1.8028	0.7729	0.6422	0.2184	0.2307	
0.6256	0.5731	0.5526	2.4449	0.7747	0.9717	1.3028	1.0387	
1.393	1.4328	1.7663	0.6527	1.6085	0.4279	0.5239	0.1272	
0.404	0.6639	0.519	1.2554	2.0415	0.3435	2.1793	3.7968	
0.5771	1.2528	0.9568	0.1368	1.5995	0.6161	0.3778	0.7029	
0.6487	0.7051	0.2582	1.7132	2.0136	0.9116	0.3099	1.1233	
0.5882	0.9842	1.1653	1.1305	0.1022	1.0672	0.7823	2.8071	
0.1768	0.0059	1.9208	0.6325	1.6363	0.8827	1.3219	0.4988	
1.2023	1.9334	0.016	2.496	1.2074	2.283	0.3121	1.0744	
1.4048	1.6029	0.4181	1.0621	0.3621	1.2689	0.1793	0.6686	
1.1517	1.0738	0.0148	0.1674	0.4737	1.3193	1.2987	0.5619	
0.2215	0.3306							
C1QTNF1	3.8063	0.7032	0.6604	2.1319	2.9967	2.0885	1.4538	
0.9799	3.5796	2.4691	2.9957	5.0728	4.6773	2.7215	0.8612	
2.7507	1.8153	1.8387	1.1043	1.9455	1.8298	1.4815	3.6227	
1.6341	1.8165	2.7802	1.2723	0.8181	1.1747	1.862	0.1934	
3.6297	0.8955	2.5399	3.4392	1.621	2.3066	3.6875	3.2366	
2.2907	4.2009	5.403	4.6829	4.3506	1.5735	2.9743	2.393	
2.3941	1.0768	1.8222	1.7594	3.3134	1.198	2.4375	0.328	
0.8905	3.4334	1.2642	1.5979	0.6475	2.2478	2.0325	2.733	
1.0086	0.3565	5.0291	0.3036	2.9348	3.31	2.4258	3.865	
1.343	3.9075	3.7176	2.2293	3.5033	2.2613	4.5121	2.9452	
3.001	2.5013	3.4643	4.5221	3.6666	4.1798	1.3966	3.5808	
1.5735	3.0028							
C1QTNF2	0.4987	0.0789	0.0258	0.3706	0.1928	0.1145	0.4886	
0.0431	0.1666	0.0248	0.0379	0.0799	1.3624	0.5017	0.6649	
0.1005	0.6649	0.1058	0.2457	0.0278	0.193	0.0172	0.3215	
0.4308	0.6766	0.5972	0.0054	0.5242	0.4685	0.0023	0	
0.1302	0.9027	0.573	0.8719	0.1076	0.3437	0.258	0.1392	0
	0.3157	0.1593	0.5398	0.35	0.1715	0.3222	0.0951	
0.1355	0.2169	0	0.1008	0.239	0.2249	0.1319	0	
0.3075	0.411	0.6378	0.1162	0.097	0.6312	0.0336	0.2348	
0.3713	0.3981	0.094	0.2175	0.1607	0.1607	0.7449	0.4507	
0.7306	0.3507	0.0697	0.2356	0.6884	0.1188	0.2211	0.2743	0
	0.1919	0.2339	0.1194	0.1481	2.2317	0.2035	0.1742	
0.6954	0.4818							
C1QTNF3	0.4663	1.6816	0.9823	3.5102	0.2876	0.7562	0.0377	
0.4691	0.0196	0.6824	0.19	0.3027	0.1028	0.1624	2.923	
0.2573	0.7149	0.908	0.2839	0.1011	0.4715	3.4363	0.4846	
1.0421	3.8723	0.1947	1.7174	4.578	1.6767	4.1676	0.5496	
0.2535	0.0702	0.0797	0.72	0.3299	2.0245	6.2223	5.5458	
5.7177	0.4538	0.0266	0.2984	0.1628	0.1857	0.2717	0.0237	
3.485	0.3718	0.5845	0.1578	0.544	0.3847	0.6106	0.1982	
0.5883	0.4647	0.1384	0.6161	0.2308	0.9178	0.4272	5.179	
0.2985	0.5556	0.2878	0.5082	0.3344	1.9175	6.7984	0.231	
0.2725	0.2452	2.3353	0.6049	0.2557	0.5556	5.1529	0.4928	
0.9502	0.895	0.2775	0.5792	4.7711	1.737	0.3276	0.167	
0.2185	0.2499							
C1QTNF4	0	0.2766	0.2984	0	0	0	0	0

	0	0	0.2679	0	0	0	0	
0.1661	0	0	0	0	0	0	0	0
	0	0	0.3427	0	0	0	0	0
	0	0	0	0	0	0.2153	0	0
	0	0	0	0	0	0	0.4221	0
	0	0	0	0	0	1.0883	0	
0.1558	0	0.1746	0.1911	0.2054	0	0.0812	0.1929	0
	0.1824	0.0726	0.1914	0	0	0	0.0512	
0.1426	0	0.0542	0.1213	0.0427	0	0	0.0754	0
	0	0.0307	0.1009	0.0555	0	0	0	0
	0.2526							
C1QTNF5	0	0	0	0	0	0	0	0
	0	0	0	0	0	0	0.0022	0
	0	0	0	0	0	0	0	0
	0	0	0	0	0	0	0	0
	0	0	0	0	0	0	0	0
	0	0	0	0	0	0	0	0
	0	0	0	0	0	0	0	0
	0	0	0	0	0	0	0	0
	0	8.00E-04	0	0	0	0	0	0
	0	2.00E-04	0	0	0	0	0	0
	0	0	0	0	0	0	0	0
	0							
C1QTNF6	1.6299	1.2032	0.1893	2.7001	0.5461	3.0515	0.7558	
0.74	2.8967	1.8994	1.3128	1.9795	1.8948	0.7796	1.8012	
1.6777	2.4075	1.4617	0.952	0.518	0.5868	2.5656	1.8581	
0.8313	2.0855	1.946	2.2242	2.8462	2.1902	1.3875	3.0053	
0.4906	0.3764	2.1382	3.1066	1.5345	0.9082	5.3355	1.1565	
3.2454	3.2142	3.2362	1.1538	0.2658	0.2993	1.8599	1.4432	
0.9745	0.7966	2.3205	2.255	1.9448	2.6559	2.377	1.9443	
2.2178	1.2768	0.9402	1.0554	0.2949	1.2663	1.4463	2.1891	
0.4948	4.5865	1.7367	1.0841	1.3339	2.7251	2.8465	2.4479	
0.3539	2.0361	2.0617	1.5963	0.8564	1.4425	4.3465	2.8801	
2.8581	3.1978	3.1163	0.1973	3.7536	0.9971	2.0421	1.7163	
2.1693	3.1226							
C1QTNF7	0	0	0	0	0	0	0	
0.0044	0	0	0	0	0	0.0208	0	0
	0	0	0	0	0	0	0	0
	0.0021	0	0	0	0	0.0091	0.0039	0
	0	0	0.0031	0	0	0	0.0041	
0.0072	0	0	0	0	0.011	0.3195	0	0
	0.0064	0	0.007	0	0	0	0	0
	0.0329	0	0	0	0	0	0	0
	0	0	0.0851	0	0	0	0	
0.0159	0	0	0	0.0263	0	0	0	0
	0	0	0	0.0446	0	0	0	
0.0188	0							
C1QTNF8	0	0	0	0	0.0043	0	0	0
	0	0	0	0	0	0	0	0
	0	0	0	0	0	0	0	0

	0	0	0	0	0	0	0	0
	0	0	0	0	0	0	0	0
	0	0	0	0	0	0	0	0
	0	0	0	0	0	0	0	0
	0	0	0	0	0	0	0	0
	0	0	0	0	0	0	0	0
	0	0	0	0	0	0	0	0
	0	0	0	0	0	0	0	0
	0							
C1QTNF9	0.0457	0	0.228	0.0973	0	0.0726	0.0031	
0.0564	0.0547	0.0434	0.0241	0.0357	0	0	0.0292	0
	0.0458	0.1009	0.0609	0	0.1076	0	0.002	
0.0618	0	0	0	0.0865	0.1212	0	0	
0.013	0	0	0.0029	0.0341	0	0.0334	0.0143	
0.0863	0.0195	0.0038	0.0657	0.0108	0	0	0.0636	0
	0.0651	0	0.0067	0.0921	0.1563	0	0	0
	0	0	0.1032	0.0731	0	0	0.1242	0
	0.0409	0.0052	0.0586	0.0073	0.0203	0.207	0.0824	
0.0689	0	0.031	0.006	0.0741	0.001	0	0.0165	
0.0534	0.0622	0.0417	0.0521	0	0	0	0.0155	
0.0676	0							
C1QTNF9B	0.1764	1.3277	0.6137	0.3643	0.2294	0.6567	0.3529	
0.804	0.0413	0.387	0.0722	0.1528	0.285	0.6214	0.4283	
0.2161	0.076	0.2126	0.895	0.5539	1.1392	0.0953	0.565	
0.2307	0.1868	0.1937	0.1625	0	0.5481	0	0.0632	
0.3814	0.8753	0.4851	0.4626	0.415	0.0767	0.5611	0.2143	
0.3388	0.3181	0.0957	0.2865	0.463	0.0362	0.0878	1.1197	
0.0565	0.2955	0.9429	0.3008	0.1449	0.3963	0.093	0.1225	
1.0372	0	0.1071	0.3723	0.1556	0.2894	0.5375	0.4064	
0.6336	0.7583	0.0186	0.5187	0.3429	0.3573	0.751	0.4977	
0.7983	0.7811	0.1373	0.0158	0.0754	0.83	0.4253	0.2683	
0.7635	0.4444	0.1349	0.2469	0.206	0.2833	0.3683	0.323	
0.568	0.5514							
C1R	1.0651	1.9934	1.3426	1.1573	2.5502	1.3631	5.7917	
2.8197	4.5569	1.1456	2.2003	5.7398	5.4696	3.1828	3.4545	
4.6476	0.8251	1.1348	1.7494	2.5132	1.4863	4.8893	4.3414	
1.0384	4.4228	5.2281	6.5233	0.8639	1.4496	1.0271	2.9781	
1.8343	2.9914	6.7324	3.2195	1.4887	1.8261	4.3549	3.1699	
2.0858	4.4203	6.3505	5.4841	3.2664	4.2792	4.4816	0.9195	
2.0784	6.3791	1.0547	6.3172	6.4202	4.0399	1.2655	2.3451	
0.9124	1.2641	1.6478	0.6759	2.9976	3.2882	0.8352	2.6165	
2.4902	1.6371	9.5271	2.0123	4.5751	1.961	1.0265	3.5693	
2.2753	3.2804	6.0704	3.3903	0.8798	1.9697	4.1821	4.5656	
5.2801	3.1544	3.8932	2.7589	4.3406	3.1521	4.4932	3.1968	
2.3191	2.3307							
C1RL	1.1867	0.3087	0.2721	2.0651	2.018	0.9989	2.285	
1.1824	2.0193	1.3432	0.5088	3.5128	1.1018	0.5277	1.7451	
2.3732	1.9324	0.5432	0.0913	0.1205	1.3687	2.0595	0.9165	
0.5779	3.1098	0.7625	3.2175	0.0714	1.6451	0.6145	3.8192	
0.4562	1.7138	3.5432	0.2542	1.407	2.2947	2.5722	1.9538	

1.9538	3.2209	4.2839	3.6488	2.7315	2.2785	3.2068	0.1234	
3.1789	3.5386	0.134	3.621	3.9655	1.3728	0.2487	2.1796	
0.1792	3.1811	1.9901	0	2.3177	2.1383	0.0928	0.2692	
1.9344	1.3024	3.2133	0.4703	0.5414	1.2489	1.6261	2.1972	
0.0557	0.1468	3.7635	2.1412	1.2307	1.7211	3.3692	3.3281	
2.8283	2.9791	3.7875	1.5326	1.764	2.3827	1.3024	1.9573	
1.4478	1.9193							
C1S	0.6084	1.1098	0.5432	0.4281	0.4425	0.4158	5.7621	
2.5014	5.2279	0.2231	0.2818	5.3105	3.6384	4.2408	2.9722	
4.1669	0.5494	0.6204	1.5661	0.7333	1.1905	4.5643	4.7576	
1.0045	4.0049	4.5319	5.523	0.4609	0.1541	0.3557	2.0981	
2.0467	0.5019	6.6199	3.204	1.4202	0.3347	5.7621	2.6164	
2.0605	2.2805	5.5835	5.3543	1.0628	2.9984	4.0011	1.0485	
0.0637	6.0842	1.4246	6.898	7.1336	4.2169	0.3736	3.1901	
0.4727	1.6632	0.1979	0.692	1.0152	2.5677	0.5253	1.639	
0.6585	0.5994	8.7652	0.7415	4.1684	0.437	0.2799	3.2206	
1.071	3.4814	5.8927	2.2222	1.0338	1.2	5.7186	4.9037	
5.997	2.6921	1.8685	2.9582	3.9157	1.4379	4.2541	2.657	
0.5697	0.3892							
C2	0.6617	1.1716	0.2601	0.163	0.1653	0.379	0.162	
0.2542	2.0659	2.2048	0.8865	3.2479	0.1574	0.52	4.0015	
0.3806	0.3639	0.7652	0.5535	0.3597	0.4427	1.0916	0.6794	
0.5366	0.1727	0.3824	1.1578	0.3006	0.565	0.201	0.17	
0.336	0.5549	2.5349	0.4128	1.45	0.1917	5.8619	0.4949	
0.682	0.5577	0.1766	0.125	0.42	0.2496	0.3536	0.4511	
0.2866	0.1295	0.4088	0.3573	2.5998	0.3475	1.2366	0.1993	
1.7112	0.494	0.2778	0.8597	0.0722	0.7495	0.1644	0.3278	
0.3679	0.3223	0.3196	1.0671	0.7538	0.1203	0.003	0.4107	
0.3479	0.1624	1.8964	0.1359	0.0655	1.0564	0.8772	3.9445	
1.2366	1.5888	0.2051	3.947	0.1567	0.0123	0.2541	0.674	
0.673	0.6388							
C20orf141		0	0	0	0	0.064	0.2444	
0.1234	0	0	0	0	0.0692	0	0	0
	0	0.318	0	0	0	0	0	
0.134	0	0	0	0	0	0	0	0
	0	0	0.17	0	0.0925	0	0	0
	0	0	0.135	0.4365	0	0	0	0
	0	0	0	0	0	0	0	
0.0455	0	0	0	0	0	0	0	0
	0	0	0	0	0.0479	0	0	
0.0784	0	0	0.029	0	0	0	0	0
	0	0	0.0941	0	0	0.2786	0	0
	0	0						
C20orf144		0.3939	0	0	0	0.4154	0	0
	0.8515	0	0.4255	0.5325	0.3403	0	0	
0.2423	0.1976	0	0	0	0	0.5062	0	
0.2411	0	0.1585	0	0	0.5834	0.3863	0	
0.4316	0.1086	0	0.6384	0	0.2061	0.2822	0.4176	0
	0	0.578	0	0.3184	0	0.2244	0	
0.4176	0	0.3899	0	0	0	0	0.1503	

0.3123	0.2624	0.234	0.5855	0.1296	0.1991	0.0898	0	0
	0.2896	0.246	0.3739	0.3522	0.1009	0.0412	0	
0.4284	0.6264	0.5915	0.081	0	0.4497	0.1935	0.0805	
0.1138	0.3802	0.2182	0.2433	0.3518	0	0	0	0
	0.2317	0.2513						
C20orf173		0	0.0697	0	0	0.0414	0	0
	0	0	0.0534	0.0081	0	0	0	0
	0	0	0	0	0	0	0	0
	0	0	0	0	0	0	0	0
	0.0156	0	0	0	0	0	0	0
	0	0	0	0	0	0.0302	0.0228	
0.0866	0	0	0	0	0	0	0	0
	0	0	0	0	0	0	0	0
	0	0	0.0157	0	0.003	0.0918	0	0
	0	0	0	0	0	0.0067	0	
0.0331	0	0	0	0	0	0.0566	0	0
	0	0						
C20orf194		2.3557	1.9982	1.8547	1.8065	1.184	2.5095	
2.5231	2.7689	3.3145	2.1789	2.6883	1.6319	2.2639	2.1492	
2.4427	2.6088	2.5241	3.2882	2.933	1.8765	1.7434	2.0589	
3.1609	1.7763	2.9524	3.2694	1.5653	1.7879	2.1172	1.9919	
1.7074	2.1336	2.9652	3.1091	2.9164	2.1204	2.08	2.4331	
2.1471	2.5354	1.9417	1.8855	1.8382	3.0559	2.8161	2.0422	
2.4127	1.9693	2.8944	2.8513	2.9112	1.9988	3.5065	2.6754	
1.931	2.0264	2.5497	1.957	2.9534	1.5553	1.6725	2.0098	
2.08	2.0061	1.9731	2.2443	1.7585	2.513	2.6244	2.329	
1.5422	2.9004	3.1807	2.9687	3.574	1.3551	2.3796	2.7599	
2.4118	1.6163	2.6312	1.7894	3.1903	2.0109	2.2321	2.7809	
2.9116	2.1361	2.4215						
C20orf196		1.4945	1.6171	0.9466	1.5587	1.1135	1.2772	
0.9741	1.3919	2.0009	0.6595	0.9396	0.8432	2.0793	0.8672	
2.0988	1.1679	0.9295	1.1466	1.9813	0.978	1.7237	1.6353	
1.1418	1.4511	1.1561	1.0605	1.0204	1.1699	1.1277	0.8388	
0.8469	1.1096	1.5389	1.807	1.5728	0.8538	0.7887	2.5524	
1.5947	1.0738	1.4194	1.596	0.5918	0.9182	1.1365	1.8865	
1.2888	0.3457	0.6622	1.6041	1.2068	2.0842	1.5686	2.1825	
1.5718	2.2811	1.5078	0.7375	0.8102	0.3087	1.174	1.6108	
1.243	1.117	1.4727	0.7779	0.8031	0.8152	1.809	1.1349	
0.6537	0.9422	0.9385	0.6358	1.1734	0.9594	1.7885	1.279	
1.1499	1.4751	1.277	0.7671	1.8844	0.9663	1.5164	1.9043	
2.1497	1.4176	1.9619						
C20orf202		0	0.1731	0	0	0	0	
0.0609	0	0	0	0.0765	0.0372	0.0147	0	
0.012	0	0.0515	0.0389	0	0	0	0.008	
0.0216	0	0	0.018	0.0261	0	0	0.05	
0.0386	0.0019	0	0.0254	0.0027	0.0168	0	0.1271	
0.0385	0	0.1392	0.0174	0	0	0.0701	0	
0.1051	0	0	0	0.0215	0	0	0	0
	0	0.1216	0	0	0	0	0	0
	0	0.06	0.0129	0.1492	0.0703	0	0	0

	0	0	0.0452	0	0	0	0	0
	0	0	0	0	0	0	0	
0.0679	0	0.0482						
C20orf203		0	0	0.0417	0.0279	0	0.0532	
0.0206	0.0053	0.0059	0.017	0.0014	0.0179	0.0281	0.0484	
0.0025	0.0022	0.0442	0.0344	0.1372	0.0244	0.0223	2.00E-04	
7.00E-04	0.0113	0	0	0.0246	0.0773	0.0096	0.0116	
0.021	0.0459	0.036	0.0184	0.0045	0.0316	0.0055	0	
0.0046	0.0428	0.0065	0.0107	0.0206	0.0116	0	0	
0.0491	0.0144	0.0063	0.0439	0.0173	0.0353	0	0.0076	
0.0193	0	0	0	0.017	0.0164	0.0129	0.0273	
0.0107	0.0212	0	0.0053	0	0.0043	0.0144	0.053	0
	0.0335	0.0155	0.0058	0.0298	0.0384	0.0393	0.014	
0.0058	0	0	0.0128	0.1303	0	0.0264	0	
0.0431	0	0.0272						
C20orf24	6.6511	7.2367	7.5306	7.2545	6.7866	6.6758	8.8906	
7.8006	7.0086	7.1046	6.4382	7.0086	7.148	7.3195	6.6202	
7.5348	6.541	7.0086	7.6064	6.7746	6.6414	6.7648	7.2937	
7.4497	6.932	6.7707	7.0199	6.6685	6.7905	7.0549	5.9832	
7.2053	7.3676	6.8583	6.8189	7.5424	6.3194	6.0424	6.3731	
6.9276	5.9164	6.3409	6.0223	6.3908	7.11	6.5815	7.5464	
6.6396	7.0271	7.4461	6.8623	7.4604	6.8813	6.5254	7.1248	
7.3281	5.9929	6.8592	7.8136	7.0717	6.1732	6.8029	6.2901	
7.1936	5.7423	6.1719	6.61	6.5982	7.4384	6.1861	7.6004	
6.8957	7.6698	6.7193	7.0438	7.4421	7.0169	7.6076	5.6938	
6.7514	6.535	6.6468	7.1706	6.7029	6.7926	7.0743	5.9977	
6.0424	5.8305							
C20orf27	4.7722	5.5481	5.5804	3.7237	5.2619	4.6384	4.1219	
5.8439	4.9922	4.3023	5.8706	4.0879	5.0529	4.1047	5.8823	
5.1094	5.7086	3.8344	4.5953	5.5062	5.575	5.0363	5.0864	
4.7744	4.8586	4.8938	2.8439	5.0555	4.3688	4.7691	5.1838	
5.5832	5.9229	5.4385	3.9129	4.1968	4.6529	5.0216	4.9073	
4.1335	4.8311	4.1063	4.548	4.9306	4.5221	3.9587	4.6784	
4.2074	5.0803	5.1272	4.8053	3.6241	5.0976	5.2629	6.251	
5.899	3.8216	5.1638	5.2339	5.3763	4.377	5.754	4.99	
4.4175	5.3477	3.4953	4.3334	5.3697	4.933	4.6889	5.0331	
5.1717	5.8837	4.3157	5.0959	3.9944	5.6339	4.5041	4.8126	
4.6335	5.3213	4.0697	4.3905	4.6788	5.002	6.0792	4.2129	
5.185	4.624							
C20orf85	0	0	0	0	0	0	0	0
	0	0	0	0	0	0	0	0
	0	0	0	0	0	0	0.0617	0
	0	0.1131	0	0	0	0	0	0
	0	0	0	0	0	0	0	0
	0	0	0	0	0	0	0	0
	0	0	0	0	0	0.1438	0	0
	0	0	0	0	0	0	0	0
	0	0	0	0	0	0	0	0
	0	0	0.0762	0	0	0	0	0
	0	0	0	0.0506	0.1223	0	0	0

0
C20orf96 2.226 2.6676 1.7564 2.1607 1.6144 1.6369 3.1381
3.3719 3.2228 2.4256 3.2389 2.7649 3.5411 2.6886 2.7356
2.1397 2.9668 2.8409 2.6566 2.6676 2.644 1.8976 3.0135
2.2094 3.5232 3.6244 3.2819 2.6952 1.4812 3.0724 3.483
1.9519 2.4399 2.7286 2.5786 2.6587 1.5146 2.1554 1.6415
2.8941 2.968 2.5696 1.3712 3.1041 1.8641 2.0479 2.9672
2.0156 2.8373 2.9539 3.1602 2.21 2.9711 3.0882 3.254
2.5019 1.729 1.4469 2.3227 1.8447 1.2326 2.2629 2.7804
2.5034 2.6047 1.9978 2.8519 3.1718 2.8495 2.5357 2.2853
3.0029 3.612 3.2199 3.629 2.0875 2.392 2.8233 2.6565
2.6589 3.0289 2.6976 2.2869 2.46 2.8631 3.0746 2.1264
2.3572 2.7822
C21orf140 0 0 0 0 0 0 0
0 0 0 0 0 0 0 0
0 0 0 0 0 0 0 0
0 0 0 0 0 0 0 0
0 0 0 0 0 0 0 0
0 0 0 0 0 0 0 0
0 0 0 0 0 0 0 0
0 0 0 0 0 0 0 0
0 0 0 0 0 0 0 0
0 0 0 0 0 0 0 0
0 0 0 0 0 0 0 0
0 0
C21orf2 2.2062 2.376 2.1004 2.0457 2.5039 3.0805 3.0556
2.7597 2.6792 1.1165 1.6197 0.8698 1.1888 2.2415 2.4739
2.4665 1.7255 2.1999 2.371 3.0218 2.7419 2.7094 2.6012
2.7166 3.2949 3.2279 1.559 2.96 2.3094 2.2041 3.3537
3.4538 1.8305 1.8517 3.0196 1.3103 2.0828 2.4717 2.6162
1.8043 3.0801 2.1188 2.3228 2.1747 1.6128 3.0013 1.7719
2.5159 2.6896 1.7664 2.4753 2.0564 1.9918 2.3862 2.9998
3.4645 1.3821 2.1436 2.0925 2.1967 2.3919 1.6067 2.8119
2.782 2.001 2.6133 1.9763 2.7685 2.8696 3.3763 1.794
2.0473 2.0639 1.13 2.1887 2.7052 2.3444 2.7412 2.8954
2.1186 2.2984 2.2745 3.0767 3.3938 3.1042 2.8125 1.9893
2.664 2.6632
C21orf33 2.3465 2.6669 1.8948 2.0145 1.7525 1.7724 2.0367
2.2004 2.4143 1.4144 2.0557 1.5637 1.2426 2.1272 1.4855
2.011 2.2848 1.6714 2.261 2.2314 1.3702 1.6831 1.8559
2.1177 1.8553 1.6606 1.5325 1.9845 1.9166 2.2342 2.1487
2.0191 1.8881 1.6891 1.6124 1.763 1.9173 2.2153 2.0925
1.7037 2.0999 2.0162 1.3517 1.9987 1.209 1.8342 1.6789
1.5993 1.3156 2.3263 1.9923 1.6213 2.0774 2.2778 2.5436
2.4815 1.4129 1.7925 2.194 1.9466 1.9577 1.873 2.1291
2.2693 2.1006 2.2364 2.0757 1.5409 1.1177 2.3668 1.639
1.6552 2.3558 1.9806 1.5717 2.0807 1.9244 2.2088 2.1152
2.2003 2.5542 2.1819 1.6059 1.7267 1.8323 2.4114 1.6442
1.8261 1.4607
C21orf58 1.8076 1.5621 1.85 2.3122 2.0521 1.5198 1.2432

2.0247	1.6951	0.7171	1.3263	0.7831	2.5239	1.9274	2.057	
0.7098	1.534	1.818	1.5981	2.2716	1.4945	2.4487	1.805	
2.5526	2.9169	1.0264	0.3757	2.3563	1.9895	1.3568	1.474	
2.8074	1.8076	2.2323	2.7232	0.6043	1.7468	0.4896	0.4904	
0.8978	2.0669	1.2327	2.4688	1.6377	1.2168	1.9987	0.7177	
2.0701	1.6874	2.0166	1.3461	1.1753	1.2004	1.1481	2.2004	
2.9152	1.0886	1.5134	1.6041	2.0443	1.8873	1.1916	2.1328	
2.7169	2.0327	0.4516	1.9943	0.6105	1.9759	1.6023	0.9327	
2.426	1.5251	1.5499	1.6103	0.6559	2.1699	1.5943	2.0072	
1.5736	2.2408	0.6741	0.8459	1.7609	2.7661	2.0486	1.2878	
2.0714	2.1558							
C21orf59	4.6918	5.3264	4.6697	5.5915	5.0777	4.7053	5.6975	
4.8185	4.9377	4.8471	4.2601	5.1212	4.3671	5.5012	4.8073	
5.5285	5.068	4.7012	4.9129	4.9365	5.0448	5.3659	4.7537	
5.6616	5.1331	4.6978	4.512	5.2315	5.1886	5.0208	5.3271	
5.4631	4.7981	4.0496	4.6163	5.0675	5.8472	4.3052	4.6228	
4.6542	4.8727	5.0669	4.0416	4.5518	4.1616	4.6866	5.1643	
4.4471	3.8214	5.3919	4.7992	5.4435	5.0066	5.5264	6.1412	
5.4987	4.9717	4.4174	5.1566	4.2181	4.8862	4.4238	4.2532	
5.0422	4.5254	4.8661	4.5146	5.3899	5.2161	5.1435	4.4303	
4.6178	4.5381	4.6215	5.3192	5.2982	5.5115	5.1404	4.8315	
4.2794	4.9092	5.0135	4.3264	4.5123	5.1501	5.2197	4.0595	
3.9924	4.0753							
C21orf62	0.1234	0.1278	0.0639	0.1014	0.0566	0.1485	0.0435	
0.0762	0.1525	0.0781	0.0258	0.0374	0.0373	0.045	0.0673	
0.0991	0.2237	0.0565	0.1895	0.2156	0.1219	0.0643	0.0272	
0.0971	0.1024	0.0265	0.1261	0.1998	0.0442	0.0354	0.0767	
0.0805	0.0496	0.004	0.0509	0.0156	0.031	0.043	0.0673	
0.129	0.0353	0.0017	0.0956	0.0986	0.0937	0.0134	0.1137	
0.0934	0.0795	0.2151	0.073	0.1112	0.1088	0.0666	0.1003	
0.2402	0.0593	0.0971	0.0647	0.0749	0.1131	0.0366	0.0779	
0.0275	0.0929	0.0618	0.0728	0.0352	0.074	0.0576	0.0373	
0.0724	0.0953	0.054	0.0479	0.0787	0.0944	0.0662	0.057	
0.1625	0.1214	0.0397	0.068	0.0685	0.0308	0.1516	0.0847	
0.1127	0.0939							
C21orf91	2.9557	4.1477	3.5055	1.5011	1.467	2.2118	4.5561	
2.2715	5.422	4.3955	3.6028	2.5107	2.3862	4.1378	2.0562	
4.8416	2.2796	4.5616	4.384	2.7359	3.2882	2.0109	2.6274	
2.8663	2.2777	1.1914	0.476	5.7776	2.3944	3.926	2.3514	
4.0835	4.348	3.7897	3.1564	4.1386	2.1115	2.5843	1.9094	
2.4058	3.5957	4.2278	1.5172	2.7258	1.9164	3.373	3.5997	
3.2458	1.7504	3.1037	3.2549	2.0658	3.8096	2.2727	2.7358	
3.3285	1.7993	2.6261	3.9549	2.4658	2.1823	3.4643	2.051	
3.8009	1.6016	2.8614	4.0776	3.2595	5.3687	2.5288	3.0116	
2.8562	4.3604	1.1557	3.4054	4.7474	4.4387	3.0286	4.7312	
4.2782	5.2876	1.6749	3.1559	2.9702	1.8856	3.3633	4.4607	
3.6061	4.7027							
C22orf15	0.1585	0.3429	0.0749	0	0.046	0.147	0.1097	
0.261	0.6135	0.6155	0.1021	0.0304	0.1011	0.0537	0.0086	
0.089	0.1885	0.2929	0.181	0.2559	0.1274	0.0339	0.0238	0

	0	0.2105	0.0937	0.1487	0.0352	0.1902	0.01	
0.1433	0.023	0.0451	0.0611	0.0786	0.0047	0.0277	0.0944	
0.4426	0.1495	0.0341	0	0.0736	0.1255	0	0.068	
0.4012	0.0507	0.128	0.0763	0.1079	0.1307	0.0533	0.1493	0
	0.1093	0.0428	0.1257	0.0406	0.0775	0.0913	0.6886	
0.2588	0.0879	0.0831	0.1659	0.0135	0.025	0.074	0	0
	0.0755	0.0292	0.2647	0.2438	0.222	0.0687	0.069	
0.0645	0.198	0.0619	0	0.0922	0	0.1529	0.0994	
0.5675	0.1608							
C22orf23	2.8916	2.0209	2.3191	2.5158	3.566	3.2072	3.058	
2.7255	1.8672	2.2998	2.9873	2.2431	2.5049	2.2882	3.1958	
3.0271	2.3835	2.3438	2.4666	2.4153	1.5227	2.4383	3.2441	
2.3176	3.5133	3.5257	3.1706	2.3849	2.7721	2.6144	1.507	
1.3767	1.7101	2.5594	3.1801	1.9234	2.3907	2.5249	3.3045	
3.125	1.9484	3.346	2.9091	2.532	3.4209	2.4481	2.3095	
2.1989	3.844	2.7406	2.1002	2.2088	3.0156	3.708	1.5356	
2.5962	2.3479	2.9201	2.3538	3.1493	2.4805	2.5643	2.0336	
1.7947	1.932	3.0493	2.2247	3.1308	2.4713	2.7384	3.0365	
2.6098	2.3686	2.2071	3.5023	2.4896	1.5936	2.7789	1.9918	
2.3012	2.1788	3.7828	2.8281	2.6149	3.5626	2.572	2.147	
1.9337	1.9119							
C22orf29	1.9249	2.3165	2.3399	1.4022	1.6916	2.3248	2.2196	
2.4314	2.1362	1.9314	2.3684	1.1819	2.3322	1.9879	2.6435	
1.8382	1.4856	2.328	2.0829	2.2097	1.9095	1.9201	1.2942	
2.1678	1.4209	2.2535	1.2777	2.7573	1.9337	2.1489	1.743	
2.3808	2.2017	1.7645	1.9284	1.6992	1.973	2.3512	2.614	
1.8411	1.5224	1.5523	1.2997	2.0575	1.8516	1.9736	1.906	
1.8334	1.9095	2.4812	1.9465	1.2264	1.9261	2.5507	1.9968	
1.4217	2.1161	1.9347	1.7603	2.0412	2.007	2.1633	2.9145	
2.1398	2.1843	1.5593	2.2225	1.9115	2.3695	1.9367	1.772	
1.9388	1.6403	0.6528	2.3228	1.8989	2.3423	2.001	1.9884	
1.7599	2.3031	2.6637	1.4031	1.7368	2.1371	2.1202	1.8462	
2.1438	2.0076							
C22orf31	0	0	0.0665	0	0	0	0	0
	0.0288	0.0335	0	0.0157	0	0.0622	0	
0.405	0	0.054	0	0	0	0.0402	0	0
	0	0	0	0	0.4847	0.0364	0	0
	0	0.0379	0.0764	0	0	0	0	0
	0	0.0113	0	0	0.0227	0.1307	0	0
	0	0	0	0.0384	0	0	0	0
	0	0	0	0	0	0	0.0607	0
	0	0.0565	0	0	0	0	0	0
	0	0.0673	0.2102	0.1564	0	0.2401	0	
0.1172	0	0	0	0	0.0881	0	0	0
	0							
C22orf39	3.0702	3.4865	3.0833	2.7173	1.7536	3.023	2.6127	
3.4839	3.6583	3.3709	3.2593	2.871	3.4445	2.901	3.4815	
3.0635	2.8249	3.2383	3.6643	3.2464	2.7524	3.1627	2.2159	
3.2921	2.0308	2.5265	3.3735	3.8823	3.0524	3.5718	2.7366	
3.5792	2.9379	3.4488	3.2409	2.9829	2.9334	3.0155	3.0776	

2.7117	2.289	2.2661	2.1937	1.8235	3.4495	3.2346	3.2142	
3.4827	2.7685	3.2522	2.9165	2.3811	3.1112	3.4919	2.5656	
2.191	2.9723	2.4797	2.9146	2.3871	3.269	3.48	3.2627	
2.3441	2.8965	3.0339	3.3288	3.0569	3.0887	2.7416	2.6411	
2.8261	2.5864	1.6218	3.6327	2.8862	2.9828	3.2989	3.6412	
3.052	3.8691	3.4672	2.9019	3.5438	2.7751	3.2647	2.9597	
2.4619	1.9691							
C22orf42	0	0.4486	0	0	0	0	0	0
	0	0	0	0	0	0	0	0
	0	0	0.1217	0	0	0	0	0
	0	0	0	0	0	0.7184	0	0
	0	0	0.0205	0.0713	0	0	0	
0.0509	0.0439	0	0	0	0	0	0	
0.1776	0	0	0	0	0	0	0	0
	0	0	0	0	0	0	0	0
	0	0	0	0	0	0	0	0
	0	0	0	0	0.1735	0	0.1516	0
	0	0	0	0	0	0	0	0
	0							
C22orf46	1.919	2.5588	1.8009	1.939	1.9807	1.9727	2.2293	
1.8032	1.8819	1.2484	2.3648	2.5234	2.0291	2.02	2.1636	
2.8315	2.0747	2.3656	2.05	1.7709	1.8569	2.1201	2.123	
2.2726	2.3038	2.7805	0.7372	2.5657	2.0636	1.7643	2.4918	
4.6278	2.3009	1.8462	2.482	1.5181	2.3718	1.2739	2.4598	
2.2434	2.2499	2.3384	1.9035	2.4435	2.165	2.9143	1.8793	
2.6835	1.7498	2.4494	1.5836	2.8679	2.2061	2.6855	2.1418	
1.5124	1.2568	1.3984	1.4224	1.352	1.6305	1.6732	1.7159	
2.9147	2.3437	2.3549	2.8775	2.0081	1.8671	1.6612	2.4931	
2.4276	2.2645	1.9078	3.2979	2.0258	1.9195	2.5924	2.4055	
1.7446	2.9424	2.5164	1.56	2.2096	2.111	2.117	1.7264	
1.8298	2.108							
C2CD2	3.0173	2.5383	0	2.4432	0.0159	0.7003	3.8581	
2.3108	3.3665	3.2823	0.6209	2.5016	2.5025	2.7974	0.1312	
1.6672	4.0864	3.6725	3.5745	0.059	0.8771	2.3938	0.6306	
2.4964	2.5362	2.5924	0.8607	0.0301	1.9926	3.7043	3.0753	
2.532	4.0529	2.3347	2.6148	2.6908	1.7204	0.0917	0.3733	
2.2411	2.5915	4.1634	2.8406	0.3948	1.8203	2.1224	0.1938	
1.158	0.3079	1.4704	2.3895	0.4078	3.5481	1.6666	2.9227	0
	4.1133	2.404	3.2097	2.018	0.3644	0	1.723	
3.5097	2.7495	2.2209	0.087	1.8435	4.7451	1.5483	0.9834	
0.0915	2.154	1.8706	0.6411	2.4007	2.493	0.4156	2.5666	
0.0876	3.5151	3.1709	0.8854	2.0275	1.4539	3.0701	2.0865	
1.5627	2.2595							
C2CD2L	2.8571	2.693	2.8877	2.4254	3.7624	2.0612	2.6598	
3.3919	2.0028	1.8553	2.0597	2.7904	2.0627	2.3161	2.1602	
2.8384	2.3227	1.9962	2.4843	3.7059	2.2397	3.4232	2.8193	
3.1707	1.9012	2.4287	2.558	2.3112	2.8047	2.29	2.0493	
2.5534	2.7547	2.8058	2.8827	1.5366	2.4717	2.6913	2.8774	
1.3944	3.2129	2.2628	2.3305	2.0592	1.8705	1.3275	1.6009	
3.2126	2.9607	1.7967	3.7609	2.2411	1.7288	2.9695	2.2671	

3.4368	2.5456	2.7329	2.4128	2.7555	2.3857	2.3192	3.0882	
2.7943	0.696	2.8401	2.2888	2.8029	2.6496	2.4128	1.7247	
2.5208	2.9565	2.5477	2.4305	3.3524	2.1415	1.8085	3.0422	
2.5505	2.0503	2.2968	2.6532	2.7037	2.4516	1.6701	2.7149	
2.802	2.6318							
C2CD3	2.0154	3.2978	2.9736	2.779	2.662	2.7982	3.3211	
1.9563	2.5356	2.5654	2.1779	2.0369	2.0271	2.4178	1.739	
2.4427	2.808	1.9557	2.3115	2.6054	3.8671	2.4769	2.1108	
2.4314	2.7188	2.5842	2.2484	2.675	2.5981	2.247	2.3507	
1.1656	2.4972	1.4625	2.4458	3.024	2.5148	1.7017	2.2702	
2.366	1.6457	1.9943	1.4243	1.8949	2.6982	3.1437	3.1285	
2.7153	1.8758	2.9111	2.0002	2.6291	2.0991	2.4037	2.6092	
2.6213	1.7151	2.4085	2.4165	2.0748	2.2335	2.1337	2.5481	
2.318	1.836	2.5633	1.7562	2.1456	1.6276	2.04	2.5016	
2.0778	2.1781	1.4348	2.2228	2.6294	3.5857	1.8348	2.6471	
2.2202	3.6749	1.8878	3.2216	2.0738	2.2614	1.7968	1.8086	
1.8284	1.7625							
C2CD4A	0	0	0	0	0	0	0	0
	0.0771	0	0	0	0	0	0.0152	0
	0.0698	0	0.2017	0	0	0	0	
0.0198	0.0138	0	0	0	0.0572	0.0325	0.0227	
0.0043	0	0	0.0186	0.0685	0	0	0	0
	0	0.0055	0	0.0753	0	0.0306	0	0
	0	0	0.0297	0	0	0.0141	0.0796	
0.0317	0.0445	0.0273	0	0.0383	0.0073	0	0	0
	0	0.4175	0	0.0062	0	0	0	0
	0	0	0.0188	0.0356	0.002	0	0.0028	0
	0	0.0041	0	0	0	0	0	0
	0.0687							
C2CD4B	0	0	0	0	0	0	0	0
	0	0	0	0	0	0	0.3497	0
	0	0	0	0	0	0	0	0
	0	0	0	0	0.0875	0	0.063	
0.0311	0	0	0	0	0	0	0	0
	0	0	0	0	0	0	0	0
	0	0	0	0	0	0	0.0905	0
	0	0	0	0	0.0641	0	0	0
	0	0	0	0	0	0	0	0
	0	0	0	0.123	0	0.0782	0	0
	0.0718	0	0	0	0	0	0	0
	0							
C2CD4C	0.571	0	0.1609	0	0.0677	0.3402	0.0703	
0.2693	0	0	0	0	0.8986	0.1646	0.1014	
0.0606	0.6803	0	0	0	0	0.0539	0.2898	0
	0.2479	0	0.2546	0	0.0742	0	2.3973	
0.041	0.082	0.0799	0.0654	0.1382	0	0.7219	0.3745	0
	0	0.0466	0.4051	0.1658	0.6369	0	0	0
	0	0	0	0	0	0.0205	1.3241	
0.5115	0	1.4026	0	1.5219	0.2628	0	0.226	
0.0383	0.7856	0.0131	0.4119	0.0202	2.00E-04	0.2395	0.0539	

0.2328	0.3882	0	0.0617	0.0082	0	0.2593	0.012	0
	0	0	0	0.1475	1.2988	0.0218	0	
0.1272	0							
C2CD4D	0.2653	0.2044	0.8419	0	0.358	0.2608	0.2647	
0.3195	0.3372	0.9173	0.4487	0.2206	0.1521	0	0.2426	
0.447	0	0.7849	0.3615	0	0.6354	0.2023	0.2511	
0.5732	0.191	0	0	0.7211	0.3081	0	0	
0.6992	0.3691	0.1902	0.2323	0.1633	0.6872	1.0421	0.2362	
0.4783	0.7357	0.1607	0.8158	0.2729	0	0	0	
0.298	0.3175	0	0.4721	0.1935	0.2818	0.4241	0	
0.7264	0	0.6291	0.4459	0.4706	0.4126	0.4955	0.9618	
0.5927	0.466	0.0337	0.6277	0.0827	0.466	0.4	0.2722	
0.639	0.24	0.0126	0.1595	0.311	0.9029	0.4764	0.7176	
0.7059	0.5418	0	0.5065	0.4436	0.7801	0.6022	0.1994	
0.2792	0.3065							
C2CD5	3.0871	3.0617	2.6903	3.2326	3.0038	3.1519	2.4082	
3.7885	3.437	2.786	3.0103	2.9413	3.8807	3.4821	2.8088	
3.1244	4.0694	4.2145	3.3052	2.9309	2.8341	3.1799	2.7927	
2.6174	2.8822	3.1921	3.4612	3.1381	3.2814	3.5551	4.6882	
2.7164	3.6473	3.1708	3.9318	3.3372	3.1445	3.2513	3.3578	
3.4382	3.1955	2.9935	3.4278	3.5052	4.0562	3.7186	3.7405	
3.7732	3.2938	5.0959	4.0042	3.1362	3.5811	2.9946	3.7239	
2.5713	3.8178	3.8838	2.8756	2.7028	3.0892	3.6097	3.9302	
3.3135	3.5145	3.1672	4.8033	3.0784	2.8472	2.3697	3.1409	
3.1942	2.8368	2.747	3.4729	3.067	3.0801	2.5828	3.7686	
3.3951	4.0442	2.7664	3.17	3.2699	2.7566	3.6603	3.1948	
3.0581	3.5709							
C2orf15	0.4268	1.1093	0.7943	0.7353	0.2934	0.5106	0.3213	
0.7955	0.2318	0.4629	0.1749	0.307	0.1184	0.8023	0.0923	
0.3795	0.0606	1.2487	0.4003	0.4589	1.0246	0.0361	0.1103	
0.4888	0.091	0.0979	0.0557	0.5739	0.1668	0.7631	0.5569	
0.7304	0.4007	0.0607	0.1595	0.7135	0.1534	0.311	0.018	
0.2104	0.4756	0.2041	0.0795	0.3474	0.4598	0.0237	0.419	
0.6915	0.2886	0.7862	0.1137	0.1632	0.2334	0.6453	0.8786	
0.6576	0.1247	0.2988	1.023	0.1854	0.0445	0.528	0.2819	
0.706	0.1884	0.0247	0.348	0.5689	1.4775	0.2465	0.2515	
0.2684	0.4578	0.02	0.4325	0.507	0.5322	0.1825	0.5275	
0.5051	0.4953	0.2364	0.5455	0.0977	0.059	0.098	0.2493	
0.1739	0.4093							
C2orf16	0.2693	0.3596	0.6882	0.3232	0.0962	0.1679	0.1774	
0.2873	0.5307	0.228	0.1353	0.1126	0.1393	0.1363	0.1288	
0.2188	0.9508	0.165	0.4469	0.1087	0.3807	0.1517	0.2927	
0.2497	0.2823	0.2567	0.1404	0.1083	0.3995	0.0681	0.4535	
0.1888	0.2345	0.0725	0.26	0.1596	0.2738	0.6218	0.2071	
0.1958	0.2188	0.2915	0.2689	0.2904	0.0992	0.3313	0.1239	
0.4397	0.4924	0.6262	0.2553	0.5918	0.3564	0.5778	0.2445	
0.1598	0.233	0.3524	0.1284	0.2571	0.1228	0.2671	0.1408	
0.3289	0.3043	0.3342	0.4132	0.2002	0.116	0.0825	0.457	
0.2288	0.6801	0.0708	0.227	0.1815	0.2461	0.3142	0.3064	
0.3678	0.3756	0.2427	0.2384	0.178	0.1136	0.1781	0.2266	

0.1743	0.2306							
C2orf40	0	0	0	0	0	0	0	
0.162	0	0	0	0	0	0	0	0
	0.2102	0	0	0	0	0.1337	0.0575	0
	0	0	0	0	0.2451	0	0	
0.0494	0	0	0.0567	0	0.0698	0	0	0
	0	0.0502	0.1437	0	0.1536	0	0	0
	0	0	0	0	0.3248	0.0766	0.0627	
0.1404	0	0	0.0663	0.0563	0	0.1114	0	
0.0803	0.1349	0.414	0	0.021	0.1316	0.099	0.1485	0
	0	0.0069	0	0.113	0.089	0	0.0738	0
	0	0.07	0.0447	0.055	0	0	0	
0.1382	0.2619							
C2orf42	2.2577	2.2941	2.1127	2.3693	2.1707	1.6952	3.2091	
1.9367	2.6442	2.3191	2.0375	2.2422	2.662	2.4059	2.2327	
1.9862	2.2209	3.0056	2.2082	1.9108	1.9811	2.1542	2.3906	
2.1519	1.7468	2.3071	1.328	2.0675	2.7735	2.2859	1.5462	
2.3612	2.8229	2.2086	2.278	2.4406	2.5632	2.5121	2.4013	
2.3952	2.3478	1.8305	2.4426	2.1494	2.3786	2.3938	2.3938	
2.4273	2.7364	2.3225	2.3563	2.7136	2.3728	2.7231	2.0616	
2.2985	2.0601	1.715	2.2118	1.9484	2.2019	2.812	2.2919	
2.0882	2.3869	2.3214	2.9282	2.8235	2.4041	2.4327	1.7572	
2.4882	2.2049	2.0992	2.1004	1.7684	2.2012	2.0813	2.474	
2.3933	2.188	1.9189	2.4757	2.1069	1.7455	2.0911	2.4373	
2.035	2.2395							
C2orf47	3.8587	4.7609	4.1441	4.426	3.2786	4.2274	4.0738	
4.8343	3.8991	3.805	3.7457	3.724	3.2466	4.0938	3.89	
3.519	4.0844	3.874	4.536	3.921	3.5799	3.413	3.805	
4.0404	3.7669	3.1955	3.6238	3.5163	3.6158	4.1666	2.926	
3.9069	4.3989	3.3993	3.2811	4.2169	3.5987	3.8771	3.4359	
4.4953	3.6766	3.8054	3.6535	3.5721	3.7672	4.0724	4.4497	
4.3366	3.2913	4.3371	3.9715	3.5832	4.1645	3.73	3.8093	
4.3252	3.6084	4.2058	4.2284	4.2021	3.4922	4.083	3.3606	
3.2412	3.7428	3.4595	3.2801	3.4926	4.1905	3.7501	4.4869	
3.3438	4.27	3.6777	3.2426	3.8068	4.3806	2.9559	3.9468	4.2
	4.1523	3.3715	3.5749	2.9306	3.4306	4.3111	2.7922	
3.2189	3.0798							
C2orf49	2.8415	3.1663	2.7287	3.0619	2.6393	3.1066	3.2901	
3.4897	2.8187	2.8385	3.3472	3.3238	2.8504	3.5451	3.2068	
2.931	2.9702	3.4163	3.0144	2.5454	2.9329	3.014	3.0716	
2.9917	3.0503	3.2313	4.0069	3.0559	2.7892	3.5343	2.5899	
2.9482	3.0791	3.0679	2.8404	3.1968	3.242	2.6179	2.2854	
3.047	2.736	3.1882	3.2442	3.1349	3.0389	3.2331	3.1591	
3.0205	3.46	3.24	3.275	3.2057	3.5169	2.853	2.7712	
3.1134	3.0871	2.9385	2.8641	2.9337	2.8051	3.2677	2.5114	
2.6363	3.6106	3.2127	3.5861	3.0314	3.0621	2.9273	2.6903	
3.1499	3.5004	3.6809	3.0804	2.6097	2.92	3.0387	3.0601	
3.0481	3.1779	3.0642	3.4637	2.926	2.684	3.0319	3.0619	
2.7955	3.1637							
C2orf50	0.1947	0.071	0.3367	0.0381	0.0015	0.093	0.0248	

0.011	0.1995	0.7555	0.2308	0	0.0775	0.1142	0.1311	
0.079	0.1128	0.1702	0.1601	0.0362	0.0312	0.0627	0	
0.109	0.0603	0.0264	0	0.0708	0	0.057	0.2531	
0.0135	0.0583	0	0.0173	0.0795	0.0292	0.0469	0.0416	
0.0791	0.1222	0	0.0805	0.0653	0.0826	0.226	0	
0.0697	0.0473	0.1095	0.0997	0.0196	0.118	0.3217	0.0781	0
	0	0.0659	0.0767	0	0.0441	0	0.0415	
0.1235	0.2017	0.0161	0.5641	0.009	0.0849	0.0525	0	
0.0584	0.0937	0.0076	0.0211	0.0591	0.1922	0.0459	0	
0.116	0.1165	0	0.1027	0.0475	0	0.3466	0.0506	
0.0286	0.0803							
C2orf54	0.0736	0	0	0	0	0	0	0
	0	0.0318	0	0.0149	0	0	0.0209	0
	0.069	0	0	0.0546	0	0.047	0.0362	0
	0	0	0	0	0	0	0	0
	0	0.0729	0	0	0	0	0	0
	0.027	0.009	0	0	0	0	0	0
	0	0	0.0319	0	0	0.0143	0	0
	0	0	0.0314	0.0252	0.0652	0	0	0
	0	0	0	0.0204	0.0117	0.0528	0	
0.0186	0	0	0	0.0466	0	0.0233	0.0212	
0.0459	0	0.0478	0.0146	0.024	0	0	0	0
	0							
C2orf57	0	0	0	0	0	0	0	0
	0	0	0	0.0214	0	0	0	0
	0	0	0	0	0	0	0	0
	0	0	0	0	0.0438	0	0	0
	0	0	0	0	0.0247	0	0	0
	0	0	0	0	0	0	0	0
	0	0	0	0	0	0	0	0
	0.0279	0	0	0	0	0	0	0
	0	0	0	0.0508	0	0	0	0
	0	0	0	0	0	0	0	0
	0	0	0.0166	0	0	0	0	0
	0							
C2orf61	0.0167	0.1104	0.0772	0.0138	0.2109	0.142	0	
0.0981	0.001	0.0398	0.0024	0.0779	0.0922	0.0756	0.0313	
0.0045	1.014	0.1377	0.0246	0.2446	0.1627	0	0.0874	
0.3784	0.0226	4.00E-04	0.0478	0.1476	0.0037	0.0403	0	
0.0366	0.0989	0.0446	0.1505	0.0327	0.0952	0.0097	0	
0.1424	0.1052	0.0756	0.0115	0.0052	0.0945	0.003	0.0584	
0.0566	0.286	0.1153	0.1184	0.0054	0.0882	0.0164	0.2053	
0.1092	0.0326	0.0558	0.1017	0.029	0.2131	0.0167	0.0939	
0.036	0.0369	0.0126	0.1695	0.0131	0.0176	0.2921	0.0193	
0.2088	0.0275	0.0121	0.023	0.0257	0.0632	0.1771	0.0148	
0.0445	0.0194	0.0166	0.1825	0.0311	0.0428	0.0621	0.0103	
0.0126	0.0174							
C2orf66	0.0557	0.0363	0.0568	0.2082	0	0.7368	0.2223	
0.0595	0.0763	0	0.0305	0.0506	0.1605	0.1056	0.222	
0.0904	0.1029	0	0.0903	0	0.2404	0	0.2747	

0.4887	0.0481	0.2552	0.1758	0.1552	0.0465	0.0632	0.2347	0
	0.2557	0.1588	0.32	0.0739	0.0545	0.1762	0.214	
0.4438	0.1507	0.3606	0.0641	0.1676	0.1887	0.027	0.0378	
0.0371	0.3507	0.0679	0.0654	0.1139	0.0528	0	0.0823	
0.2395	0.0727	0.1098	0.0056	0.0451	0	0.0611	0.1703	
0.2985	0.2826	0.4512	0.0864	0.2497	0	0.1944	0.234	
0.0426	0.0736	0.141	0.2788	0.0541	0.1992	0.5761	0.1179	
0.0784	0.0681	0.0581	0.0552	0.1697	0.1705	0.1876	0.0699	0
	0.1233							
C2orf68	2.2214	3.3135	2.7762	3.1472	3.4668	2.1123	2.6589	
2.1691	3.5818	2.7211	2.7707	3.3824	2.7245	2.7977	2.6498	
1.8567	3.4252	2.517	2.6672	2.503	2.4196	2.7004	3.2585	
2.8687	2.9126	2.5056	3.848	3.1479	2.7484	3.895	2.9576	
3.194	2.7409	2.6204	2.8146	2.9187	3.0776	2.8094	2.6076	
2.9392	3.3787	3.5207	2.4795	2.5964	2.7301	2.8633	2.8532	
3.2723	3.2596	3.3331	3.0127	3.1001	2.9388	2.6068	2.9647	
3.5334	2.7628	2.4696	2.5397	2.4741	2.9197	3.3566	3.1296	
2.6221	2.8581	2.933	3.1258	2.0448	3.1824	3.1344	2.1513	
2.8538	2.7815	3.7236	2.6636	2.6081	3.038	3.3536	3.0003	
3.4439	3.6353	3.3386	2.6099	3.027	2.0401	3.0353	2.5997	
2.1184	2.4784							
C2orf69	2.7509	3.4252	2.9531	3.3341	2.6126	3.8677	4.2317	
3.1071	3.9367	3.8501	3.3197	3.6443	2.5667	3.9665	3.2863	
3.8337	2.6121	3.8277	3.6911	2.9039	3.4525	2.9976	3.5223	
3.2877	3.0469	2.9305	3.7682	3.4733	3.511	4.1059	3.1918	
3.1623	4.2137	3.8373	2.7746	4.0668	3.11	3.0685	1.79	
3.4483	2.7309	2.8997	3.6698	3.3651	3.7224	3.0993	3.7088	
3.5517	3.3104	3.1932	3.6512	3.1921	3.6735	2.5222	2.8747	
3.1327	3.1071	2.7607	3.9934	2.8025	3.0425	3.3135	2.8313	
3.3126	3.8542	3.2686	3.1286	3.9136	3.6909	2.7712	3.7756	
3.6963	3.6963	3.7005	3.2847	4.0337	4.0415	2.7005	3.4173	
3.7686	3.5214	3.26	3.11	2.3984	3.0986	3.9964	3.5836	
3.5267	3.4553							
C2orf70	2.2111	0.1572	0	0.2036	0.6041	0.205	0.1077	
0.1949	0.1094	1.0396	0	0	0.6979	0.2623	0	
0.0919	0	1.9427	0.5565	0.2332	1.9791	0.1496	0.5139	0
	0.071	0	0.1536	0	0.2636	0.1212	0	
0.505	0	0	0	1.185	1.9796	0	0.7847	0
	0.5308	0.1675	0.1552	1.1692	0.1802	0.3377	0.9676	
1.2519	0	0	0	0	0.781	0.3685	0	
0.5612	0.2601	0.1564	1.7985	0.325	0	0.1468	2.5225	
0.1043	0.9054	0	0	0	0.1093	1.3748	0	0
	0.2535	0.2453	0.1099	0.2996	0	0.2797	0.3979	0
	0.8925	0.0448	0.3108	0.3698	1.7827	0.981	0	
0.9187	0.2926							
C2orf71	0	0	0	0	0.0029	0	0	0
	0.0203	0.1085	0	0	0	0	0	
0.0039	0	0.2957	0	0	0	0	0.0082	
0.0252	0	0	0	0	0	0.0162	0	0
	0	0	0.0021	0.0724	0	0.022	0	0

	0	0	0	0	0	0	0.0196	0
	0	0	0	0	0.0299	0.0121	0	0
	0	0.2336	0.0878	0.0174	0	0.0463	0	0
	0.024	0	0	0	0.0595	0	0	0
	0	0	0	0.0103	0	0.0342	0	0
	0	0	0	0	0	0	0	0
	0							
C2orf72	2.4186	1.3909	2.5577	0.4025	0.241	0.0403	2.1868	
0.7372	1.0835	2.7131	0.8969	0.6984	0.9327	0.6756	0.8305	
0.2451	0	2.2135	1.5952	0.9219	0.1017	0.7586	0.6273	
1.0955	2.1362	0.293	0.4557	1.9108	1.7838	1.8682	2.9689	
3.0453	0.856	3.3859	0.4478	1.9674	2.8497	0.3374	0.3114	
1.1555	0.0959	0.0091	0.4032	1.708	0.3027	0.4465	2.7439	
0.4353	0.3185	0.113	0.9761	0.1736	1.265	1.2169	1.208	
0.5921	0.4036	4.44	0.7593	3.8629	0.8515	1.8874	0.6749	
0.3992	2.3143	2.1108	3.0736	1.2912	1.5698	0	0.8673	
1.3022	1.1241	1.5047	0.6174	1.383	1.001	0.1013	0.617	
0.6317	1.3004	0.4374	1.2121	0.3314	0.2929	0.885	1.1447	
0.79	1.7701							
C2orf73	0.0631	0.0339	0.0958	0.0389	0	0.0826	0.0101	
0.0169	0.0422	0.0207	0	0.059	0.0926	0	0.2995	
0.0106	0.0606	0.0608	0.1114	0.1012	0	0.0024	0	0
	0	0.0403	0.0219	0.151	0.021	0.0384	0.1195	
0.0599	0.0555	0.086	0.1334	0	0.0691	0	0	
0.0401	0.0222	0.0269	0.1092	0	0	0.0182	0.0343	
0.0345	0	0	0.0496	0.0523	0	0	0.055	0
	0.1175	0	0.0293	0	0	0	0	
0.0818	0.0665	0.0401	0.3706	0.0539	0.1142	0	0	
0.0314	0	0.0084	0.0688	0.0731	0	0.1223	0.0622	0
	0.0996	0	0.0253	0.033	0	0	0	0
	0.0607							
C2orf74	3.6693	4.3532	2.1443	2.7115	3.0063	2.4932	3.5396	
3.7026	4.5193	3.0125	3.9949	4.0662	3.9599	4.4035	2.7955	
3.1384	4.6133	3.2273	2.9503	2.3586	3.8467	3.5683	3.8147	
4.3152	3.7026	3.466	3.1535	3.9368	3.9104	3.2006	4.1008	
3.4476	3.5813	4.3836	3.9633	3.5335	3.223	3.809	3.425	
3.4231	3.4619	3.9345	4.1822	3.7528	4.125	3.3922	3.5171	
4.2082	3.3516	4.6861	4.335	4.228	3.8224	4.2987	4.0889	
2.5527	3.5354	2.5285	3.8317	2.3132	3.322	3.6469	4.0224	
2.7887	4.4896	4.0107	4.4571	4.3142	3.9691	3.71	3.3134	
4.0927	3.7699	2.7342	3.832	3.8593	2.7679	4.2337	3.8771	
3.8328	4.0553	3.3293	3.8455	4.6376	2.765	4.049	4.404	
3.4708	3.8554							
C2orf76	4.3104	4.2961	3.5748	3.5408	2.9444	3.0548	3.025	
3.8297	3.7766	4.0986	3.7327	2.9799	2.8084	4.3428	3.7023	
3.4389	2.7423	4.6583	4.0115	3.0365	3.2269	3.3641	3.2467	
3.9045	3.7445	3.3199	3.7713	3.4567	3.66	4.066	3.9583	
3.1167	3.7924	3.6792	3.9269	4.3365	3.2895	3.8538	3.7593	
4.1631	2.6704	3.427	4.1286	2.9192	2.5004	3.7673	3.7344	
3.2377	3.2303	3.6225	3.4545	3.8735	3.7359	3.4534	4.1816	

4.6626	2.6996	3.9776	3.7828	3.5348	3.205	3.7894	3.6402	
2.9258	3.7989	2.9122	4.2488	3.7004	3.831	4.0623	3.1903	
3.588	4.3628	3.6753	3.5487	4.2956	3.981	4.0931	3.3191	
4.0476	4.0563	3.5711	3.2283	3.6293	3.9522	3.8258	4.4382	
3.6622	3.9352							
C2orf78	0	0	0	0.0435	0	0	0.0056	
0.0076	0	0	0	0	0.0716	0	0	0
	0.0284	0	0	0.0476	0.0256	0.002	0.0078	
0.0137	0	0.0169	0	0	0	0	0	
0.0242	0	0	9.00E-04	0	0.011	0	0	0
	0	0.0044	0	0	0.0327	0.0119	0	0
	0.0093	0	0.0213	0	0.0336	0	0.0234	0
	0.0232	0	0	0	0	0	0	0
	0	0	0	0	0	0	0	
0.0332	0	8.00E-04	0	0	0.0298	0	0	0
	0.0494	0	0	0	0.0309	0	0	0
	0							
C2orf80	0.0704	0.0866	0.3264	0	0.0167	0.0555	0	
0.0565	0	0	0	0	0	0	0	0
	0	0	0.0752	0	0	0	0	0
	0.0211	0	0	0.9384	0	0.0639	0	
0.0113	0.0816	0	0	0.1035	0	0	0.0268	
0.0298	0	0	0	0	0	0	0	0
	0	0	0	0	0	0	0	0
	0	0	0.1139	0	0.1313	0.0401	0	0
	0	0	0	0	0.1389	0	0	0
	0	0	0	0.0643	0.3605	0.1655	0.0257	
0.2077	0	0	0	0	0.3337	0	0	
0.0581	0							
C2orf81	0.9157	0.7358	0.7514	0.4834	1.4615	0.4914	0.9407	
0.9255	0.5243	0.4155	0.6595	0.7453	0.8221	0.4122	0.6756	
0.4067	0.9672	0.6643	0	0.8186	0.193	0.6331	0.9765	
0.4487	0.3734	1.0625	0.3318	1.1372	0.6479	0.6088	2.0363	
1.2779	0.7125	0.8721	0.3475	0.3406	1.2049	0.8029	0.517	
0.2561	1.0106	0.7249	0.5066	0.2641	0.3023	0.3566	0.3466	
0.8147	0.9986	0.9538	0.47	0.6175	0.5685	0.5759	1.2623	
0.479	0.4178	0.4265	0.265	0.3736	0.5743	0.3086	0.4114	
0.8138	0.8015	1.5329	1.5297	1.04	0.6658	1.342	0.7971	
0.9988	0.6681	1.0812	0.5844	0.2844	0.566	0.4693	0.6784	
0.6731	0.9565	0.6349	0.4331	0.8689	1.4318	0.6726	0.9476	
1.0808	1.0714							
C2orf82	0	0	1.0267	0	0	0	0.583	0
	0	0	0.3828	0	0.8187	0	0	0
	0	0	0	0	0	0	0	0
	0	0.4542	0	0	0	0.564	0.3371	0
	0.5756	0	0	0	0	1.0936	0.4619	0
	0	0	0	0	0	0	0	0
	0.7653	0	0	0	0	0.3719	0.2303	
0.639	0.997	0	0	0.2802	0.8787	0.6189	0.4223	0
	0.2792	0.4152	0	0.2206	0.0869	0.4883	0.1245	

0.0505	0.1812	0.1639	0	1.8436	0.5841	0.155	0.4246	
0.2695	0.2547	0.3934	0.7447	0	0.3221	0	0	
0.5194	0							
C2orf83	0	0	0	0	0.0061	0	0.0115	
0.0409	0.0388	0.0201	0	0.0062	0.0371	0.0423	0.0099	
0.0357	0.1135	0	0.1518	0	0.0489	0	0	
0.0131	0.028	0.0805	0.0237	0.1375	0.0427	0.0458	0.0583	
0.0319	0.0307	0.0208	0	0.0141	0	0	0	
0.0176	0.0452	0.002	0.0677	0.0271	0.0104	0.0209	0	
0.0306	0.0221	0	0.0204	0	0.0532	0.0574	0.078	
0.1525	0	0.0533	0.0233	0	0	0.0163	0.0217	
0.0342	0	0	0.1583	0.0473	0.0158	0.0466	0.0172	0
	0	0.0034	0.0385	0	0	0.0221	0.0115	
0.0785	0	0.0468	0	0.0461	0	0	0	
0.0463	0.0536							
C2orf88	1.7884	0.0273	0.2615	0.1849	0	0.2233	0	
1.0989	2.287	1.1939	1.114	0.3975	0.239	2.302	0.3179	
1.7238	0.4899	1.9507	2.8258	0.0858	0.435	0.0577	0.0051	
0.1415	0.0662	0.1483	0.3462	1.6307	0.136	3.0411	0.6717	
1.6457	1.2613	0.8797	0.0656	1.1439	0.8066	1.555	0.4045	
0.4746	1.1917	0.0724	0.039	0.1923	0.2104	0.1633	2.7518	
1.4253	0.056	0.3779	1.0965	0.2793	1.6123	1.1052	1.6088	
0.1086	0.1937	0.0868	2.0486	0.1453	0.0828	1.1321	0.1834	
0.2593	1.3411	0.1434	0.2967	0.2889	2.9617	0.1231	1.6956	
0.2262	0.8652	0.2505	0.1601	2.8047	2.6209	1.1543	1.9172	
0.2043	1.0956	0.2119	0.9086	0.232	0.1751	1.65	0.8435	
1.8839	1.8482							
C2orf91	0	0.0234	0.0233	0	0.0058	0.021	0	
0.0133	0.0226	0.0363	0.0024	0.0063	0	0.0258	0.0088	0
	0.0589	0	0.0523	0.0545	0.0227	0.0182	0.0843	
0.0161	0.0079	0.0061	0.0591	0.0425	0	0.0291	0.0052	
0.0021	0.0236	0	0.211	0	0	0	0	
0.0264	0.0078	0.0072	0.0436	0.012	0.0046	0.0101	0.03	0
	0.008	0	0.0089	0.041	0.0291	0.0212	0.0249	0
	0.025	0	0	0.0204	0	0	0.0529	0
	0	0.0011	0.0391	0.0076	0.0235	0	0	0
	0.0191	0.1093	0.0298	0	0	0.0565	0	
0.0671	0	0.05	0.0104	0	0.0519	0.0519	0	0
	0.0394							
C3	0.035	0.6105	0.1353	0.3163	5.4503	0.0121	4.4059	
4.5038	4.3164	0.0304	1.2239	5.028	6.1724	2.9763	0.0294	
0.9537	4.1337	0.245	0.7902	0.3191	0	4.6892	3.163	
0.2635	0.0883	3.2857	5.9522	0.0435	0.1792	1.616	0.3312	
2.7328	0.1344	1.2896	0	0.318	0.1555	0.0233	0.2393	
0.1063	2.5415	5.8876	2.7515	3.3035	1.9286	6.682	0.1153	
0.0233	7.1301	0.6258	1.3558	7.5189	0.3263	0.6034	0.683	
0.4244	2.663	0.4645	0.638	0.3898	1.1972	0.6182	0.3169	
0.4613	0.585	9.0768	0.8308	2.9413	0.5523	0.5107	2.6576	
0.8417	0.4001	7.1054	2.3318	0.3882	0.9854	1.4699	0.434	
0.6901	1.2576	3.4638	0.9828	3.4371	2.2779	0.3895	1.6863	0

	0							
C3AR1	0.5038	0.6285	1.479	2.262	1.5038	0.7702	3.7059	
0.5278	0.8707	0.0954	0.3189	2.3316	0.9544	0.7529	1.0993	
1.3222	1.4915	0.918	1.0112	0.995	0.3763	1.641	1.1806	
1.3873	1.9046	0.8328	0.0257	0.2778	0.7989	0.1094	0	
1.1253	1.7697	0.1387	2.0819	0.1129	0.733	0.1279	0.2763	
1.3358	0.3577	1.1412	0.6625	0.9107	0.9317	1.0788	1.043	
0.982	0.211	0.9514	1.2327	0.2024	0.4419	0.3383	0	
0.0804	1.9816	0.1058	0.938	0.3204	0.4098	1.6015	0.2639	
1.0596	0	0.9457	0.1615	2.7709	1.2065	1.3501	2.2498	
0.4754	0.8433	0.718	0.2878	1.9659	1.1304	0.479	1.0727	
0.4067	0.595	0.5943	1.3796	0.6845	0.6229	0.1222	0.5004	
0.2234	0.2706							
C3orf14	4.7501	4.2687	5.1043	4.114	4.1846	5.17	4.8597	
4.378	4.3473	5.24	4.7102	3.7422	5.6835	5.6357	5.6398	
5.8376	5.5729	4.5762	5.1529	3.257	4.2687	4.1331	4.3238	
5.7867	3.6439	4.2056	1.9327	4.833	4.9095	3.4537	4.5621	
4.8256	7.078	3.8596	3.9955	5.4415	4.5417	2.5128	4.0152	
4.0972	3.2814	2.8133	4.2537	4.8684	4.7048	4.1723	5.3136	
4.2406	3.3197	5.5517	4.8489	5.1226	3.7237	4.8498	4.8821	
4.845	1.8524	3.6398	4.7802	4.3373	4.6759	4.406	3.9419	
6.3918	4.0975	3.3729	3.954	5.0406	4.8466	3.983	4.4016	
4.3326	4.5471	3.1018	5.0143	4.7579	4.6213	3.2896	5.7916	
5.6015	5.9325	3.1286	4.8793	3.1263	4.7441	4.7046	5.1938	
5.2877	4.7832							
C3orf18	2.1444	2.6073	0.2235	0	1.1323	0.2429	1.2411	
0.1517	1.2981	3.1708	3.0396	0.8214	2.5363	2.0546	0.5283	
3.018	2.1421	2.6359	0.8104	0.0196	1.4113	0.0015	0.2196	
1.3968	1.6663	0.6689	0.4373	0.4078	2.2208	3.4955	2.9134	
0.1747	1.3613	2.0871	0.8767	2.7707	1.8627	0.2595	1.4789	
0.1754	1.8153	2.489	0.105	0.2486	0.582	0.0327	1.0267	
0.3234	0.8358	2.4065	2.1274	1.3963	1.618	1.6559	2.8664	
0.2228	1.7961	1.8494	3.2025	1.8236	0.1311	1.45	1.1418	
0.6757	0.4852	3.1902	3.0671	0.2394	3.3283	2.7535	0.4041	
0.3029	0.9364	1.6984	2.719	1.5697	0.2242	0.2508	0.9195	
0.2067	1.0052	0.2197	2.5169	0.935	0.3255	1.8132	3.1137	
2.7202	3.1119							
C3orf20	0.0744	0.0428	0	0.0466	0	0.1098	0.1717	0
	0.0071	0.0494	0	0.0684	0.0212	0	0.0136	
0.2415	0.2206	0	0	0	0	0	0.0105	
0.0062	0	0.034	0	0	0.025	0.0346	0.0444	
0.0352	0.0381	0	0.0293	0.0062	0.1686	0	0.0255	
0.1036	0.0271	0	0.0492	0	0.0486	0	0.051	
0.036	0.0574	0.0911	0	0	0	0	0.0852	0
	0.0874	0.082	0	0	0	0	0	0
	0	0.0546	0	0.0247	0	0	0	0
	0	0.082	0.1064	0.0654	0.0866	0	0	0
	0.0876	0	0.0964	0	0	0	0.047	
0.0398	0							
C3orf22	0	0	0	0	0	0	0	0

	0	0	0	0	0	0	0	0
	0	0	0	0	0	0	0	0
	0	0	0	0	0	0	0	0
	0	0	0	0	0	0	0	0
	0	0	0	0	0	0	0	0
	0	0	0	0	0	0	0	0
	0	0	0	0	0	0	0	0
	0	0	0	0	0	0	0	0
	0	0	0	0	0	0	0	0
	0	0	0	0	0	0	0	0
	0							
C3orf30	0	0	0	0	0	0	0	
0.0204	0	0.0253	0	0	0	0	0	
0.0133	0	0	0	0	0	0.0062	0.0068	0
	0	0	0	0	0	0	0	0
	0	0	0.0018	0.0188	0	0	0.0174	
0.0241	0	0	0	0	0	0	0.0456	0
	0	0	0.0557	0.0623	0	0.1218	0	0
	0	0	0	0	0	0	0	0
	0	0	0	0	0	0	0	0
	0	0.0016	0	0.0421	0	0	0	0
	0	0	0	0	0	0	0	0
	0							
C3orf33	2.0492	2.3112	2.0606	1.8503	1.6299	1.6707	2.2296	
2.4565	2.4796	1.9888	2.1618	1.9859	1.3641	1.2951	1.9343	
2.1957	1.9262	2.3898	2.38	1.7889	1.8377	1.4967	1.1134	
2.8195	1.0054	1.0873	3.6448	1.8933	1.4092	1.7748	2.1077	
1.8594	1.7831	2.0276	0.8883	2.2995	2.1043	2.4124	1.763	
2.4616	2.1994	2.0176	1.4466	1.4992	1.8286	2.2592	2.2176	
1.8727	1.3173	2.1467	1.8246	2.2325	2.3893	1.8634	2.3414	
2.2445	2.3832	1.9977	1.4619	2.2042	1.8338	2.3867	1.2842	
0.9792	1.5461	2.0938	2.027	1.8008	2.55	0.9361	2.1458	
2.3615	2.0287	1.3953	1.29	2.033	1.8008	2.113	1.7883	
2.3762	2.5412	1.8623	1.259	2.3179	0.7684	2.3463	1.7207	
1.6563	1.1019							
C3orf36	0	0	0	0	0	0	0	0
	0	0	0	0	0	0	0	0
	0	0	0	0	0	0	0	0
	0	0	0	0	0	0	0	0
	0	0	0	0	0	0	0	0
	0	0.0059	0	0	0.0405	0	0	0
	0	0	0	0	0	0	0	0
	0	0	0	0	0	0	0	0
	0	0	0	0	0.0211	0	0	0
	0	0	0	0	0	0	0	0
	0	0	0	0	0	0	0	0
	0							
C3orf38	2.4393	3.2602	3.1133	3.6584	3.5858	3.4885	3.6408	
3.2905	2.367	3.082	3.2909	3.6972	3.367	3.6438	3.436	
3.7251	3.5638	3.8528	3.5535	2.784	3.2802	3.155	3.311	

3.4377	2.81	3.3763	3.1384	3.2173	3.3775	3.039	2.3962
3.5858	3.0516	3.5221	2.7423	3.3949	3.6294	2.7553	3.2881
3.2779	2.6991	3.5663	2.6759	2.8053	4.151	2.9025	3.4792
3.2338	2.9771	3.9803	4.4547	3.4651	4.4242	3.3753	3.0616
2.4737	3.404	2.9946	3.2116	3.1406	3.5926	3.1585	2.9404
3.6866	2.7089	4.295	3.395	3.6152	3.8693	3.2573	2.769
3.754	3.1423	2.9034	3.6949	3.0441	3.3107	3.2177	3.3519
3.5212	3.1597	2.9198	3.63	2.4259	3.6599	4.5052	3.6157
3.6013	3.1906						
C3orf52	1.1357	0.6653	3.0738	2.0451	2.458	2.765	3.1075
2.369	2.6852	1.9493	2.1057	2.9498	1.9075	2.4666	2.0234
1.5567	3.6327	0.9149	2.2259	1.9019	2.132	2.2504	2.0463
2.2664	1.3958	2.6118	1.3741	2.1573	2.2466	3.0434	1.3016
2.5611	1.7909	1.5052	1.6178	2.5367	2.0779	0.2402	1.7787
2.5443	1.4068	1.4267	2.6772	1.7434	2.3278	2.049	3.6688
3.8083	1.8331	2.1943	2.7727	2.32	1.8915	1.424	1.3437
3.1131	2.1737	5.0894	1.9352	3.2241	1.6723	2.0809	0.736
2.6087	1.215	2.4606	2.238	2.4945	3.013	1.4292	1.4682
2.1363	2.5645	2.0926	1.3427	2.5164	2.7188	2.0892	1.7916
2.3381	0.9412	1.6637	2.8872	1.1955	2.3752	1.8818	0.9298
1.9369	1.2968						
C3orf58	2.0933	2.2347	2.7433	2.9953	2.9537	2.5432	3.7231
2.4485	3.0165	3.0642	2.6077	2.9493	2.1355	1.9593	3.4313
1.5711	2.5041	2.4968	2.6839	3.1861	2.9629	3.104	2.2137
3.4575	1.7659	1.7001	3.3595	3.3531	2.1165	2.835	3.7508
2.9871	3.0711	3.178	1.9148	3.1649	2.3326	1.8721	1.2746
3.6503	1.5315	2.3067	2.9385	3.2966	3.1332	3.2134	3.3956
3.1158	4.2277	3.7492	1.0116	3.3636	2.8792	1.9834	2.9519
3.175	3.6093	3.2775	1.6401	4.4812	3.0697	2.7303	2.6764
3.5096	2.6772	3.7635	2.4412	2.3027	3.0742	1.8323	2.8087
3.5864	2.5089	0.419	2.5475	2.7917	2.5382	2.1869	2.5859
3.7203	2.4584	2.4673	2.9815	2.3789	2.6648	2.4994	2.8998
2.694	3.4392						
C3orf62	0.8581	1.4726	1.1309	0.9239	0.857	1.2329	0.7093
0.8951	0.763	0.9323	0.6882	0.5961	0.4734	0.8497	0.734
1.3228	1.1232	1.2736	1.1558	1.0644	1.2006	0.4726	0.4957
1.3417	0.4702	0.9359	0.2776	1.4951	0.7711	1.0317	0.7198
1.1723	0.8213	0.5856	0.7385	0.7764	0.7303	1.3205	0.6378
0.7641	0.882	0.8507	0.476	0.7608	1.3323	0.516	0.937
1.2171	0.65	1.6073	0.9227	0.9148	0.7906	1.247	0.9701
0.9002	0.998	0.566	1.2084	0.8312	0.6993	1.1769	1.0209
1.4894	1.0104	0.8593	1.0913	0.8686	1.4957	0.6595	0.6906
0.7196	0.8223	0.4324	1.0488	0.5497	1.3211	1.2159	0.3634
0.9438	0.7743	0.9464	0.5205	0.732	0.7874	0.7048	0.7848
0.8086	0.8598						
C3orf67	1.964	1.0565	2.4964	2.5102	1.8776	3.0894	3.8562
3.7989	3.1403	3.2715	3.581	3.0868	2.5587	4.2813	2.7364
3.9824	2.8326	2.4875	2.5854	2.4653	2.5828	3.2304	2.9584
4.1209	2.4084	1.4842	1.6082	1.8456	4.4823	2.451	2.3286
3.4953	3.6644	3.9636	3.9201	3.2459	2.0894	0.623	1.6441

4.2724	1.8979	1.4284	3.0346	2.2573	1.7601	1.5158	3.2205	
4.5831	2.5765	2.4006	2.9936	2.8569	5.0288	1.9413	2.4093	
2.2736	0.6647	2.8395	2.8904	2.407	3.3496	2.4446	1.9543	
2.7193	0.843	2.2412	3.6888	2.4586	2.8465	2.4	4.3466	
3.5074	2.4194	3.1903	3.0792	3.4355	2.0415	2.0123	3.0153	
3.5839	3.6002	1.2393	2.9771	4.0289	2.1097	4.9861	1.9392	
2.097	1.7505							
C3orf70	0.7938	0.8271	2.0854	1.8254	1.0317	0.9225	1.5175	
1.6662	2.4219	2.6517	2.0416	1.4239	0.8004	2.4057	0.2655	
0.5548	0.061	0.9421	2.0166	0.1125	1.2779	1.6899	0.9909	
2.4998	1.5405	0.7708	1.9418	1.6323	2.4912	1.2821	0.2026	
1.63	0.2282	2.7709	0.8124	2.3396	0.7431	1.4599	1.9897	
2.321	1.1006	0.05	1.3193	1.6169	1.3034	0.0697	2.3249	
1.3589	2.6757	0.078	0.2539	3.5427	2.2245	2.4832	0.695	
1.4125	0.2314	1.8909	1.2487	1.9052	2.8581	0.5847	0.7388	
0.6049	0.1942	0	2.248	0.729	2.6848	2.1787	1.7662	
2.3013	1.695	2.7454	1.5449	0.8068	1.1408	2.2508	1.4339	
1.8652	2.3151	1.9623	0.063	3.2005	2.0568	2.1027	2.5795	
2.7042	2.5869							
C3orf80	0	0.1995	0.6053	1.2742	0.6619	0.102	0.3324	
0.2957	0	0.4213	0.4174	0.0838	0.624	0.1255	0.1885	
0.2496	0.3085	0	0.1277	0.0692	0.1685	0.0833	0.1457	
0.2259	1.6135	0.2412	0.3805	0.1631	0.4243	0.4027	0.2102	0
	0.5968	0.2405	0.0906	0.1054	0.2667	0.3465	0.3176	
0.7178	0	0.3069	0.2806	0.3627	0.3341	1.5518	0.3389	
0.4127	0.1432	0.1573	0.2498	0.1195	0.189	0.2731	0.2917	
1.4079	0.0644	0.7078	0.1137	0.7646	0.6767	0.1845	0.0352	
0.2183	0.2251	0.5649	0.011	0.1929	0.7061	0.0964	0.1412	
0.9978	0.1077	0.1363	0	0.5803	0.1166	0.0897	0.0516	
0.1026	0.5776	0.0769	0.3549	0	0.5535	0.2983	0	
0.1327	0							
C3orf84	0.1705	0	0	0	0	0	0	0
	0	0	0	0	0	0	0	0
	0	0	0	0	0	0	0	0
	0	0	0	0	0	0	0	0
	0	0	0	0	0	0	0	0
	0	0	0	0	0	0	0	0
	0	0	0	0	0	0	0	0
	0	0	0	0	0	0	0	0
	0	0	0	0	0	0	0	0
	0	0	0	0	0	0	0	0
	0	0	0	0	0	0	0	0
	0							
C4A	0.0825	0.0302	0.042	0.036	0	0.0415	0.0189	
0.0537	0.0379	0.0248	0.0155	0.1762	0.0247	0.0553	0.0857	
0.0168	0.08	0.0275	0.0486	0.0614	0.0395	0.031	0.0213	
0.0424	0.097	0.1199	0.0649	0.0634	0.0253	0	0.0886	
0.0101	0.0395	0.0964	0.0393	0.0225	0.075	1.153	0.0573	
0.0279	0.0851	0.7991	0.0391	0.0487	0.0567	0.1027	0.0296	0
	0.2052	0	0.0509	0.0506	0	0.1687	0.0417	

0.0181	0.126	0.0268	0.0166	0.0337	0.0567	0.0434	0	
0.0388	0.0903	0.1502	0.0869	0.0335	0	0.1119	0	
0.1042	0	0.0414	0	0.0199	0.0776	0.897	0.1086	
0.1226	0.052	0.4119	0.0261	0	0.1044	0.0641	0.0666	
0.059	0.035							
C4B	0	0	0	0	0	0	0	0
	0	0	0	0	0	0	0	0
	0	0	0	0	0	0	0	0
	0	0	0	0	0	0	0	0
	0	0	0	0	0	0	0	0
	0	0	0	0	0	0	0	0
	0	0	0	0	0	0	0	0
	0	0	0	0	0	0	0	0
	0	0	0	0	0	0	0	0
	0	0	0	0	0	0	0	0
	0	0	0	0	0	0	0	0
	0							
C4BPA	0	0.1002	0	0	0	0	0	0
	0	0	0	0	0	0	0	0
	0	0	0	0	0	0.0044	0	
0.0147	0.0476	0	0	0	0	0	0	0
	0	0	0	0	0.0073	0	0	0
	0	0.144	0	0	0	0	0	0
	0	0	0	0.0283	0	0	0	0
	0	0	0	0	0	0	0	0
	0	0	0	0	0	0.036	0	0
	0	0.0017	0	0	0	0	0	0
	0	0	0.2442	0	0	0	0	
0.1208	0							
C4BPB	0.107	0.0552	0.0949	0.154	0	0.0735	0.0737	
0.0788	0.081	0.2615	0.0064	0	0	0.0862	0.1792	0
	0.1161	0.388	0.1253	0.3482	0.2899	0.0042	0.0329	
0.1413	0	0.1876	0.1028	0.1946	0.0271	0.088	0.0216	
0.059	0.1938	0	0.0762	0.0606	0	0.2751	0.225	
0.2567	0.0263	0.0497	0	0.1152	0	0.0338	0	
0.133	0.14	0.3259	0.0855	0.0382	0.3557	0	0	0
	0.0832	0	0.2017	0.1175	0.0813	0.0923	0.2383	
0.0899	0.0816	0	0	0.0931	0.057	0	0.0634	
0.0686	0	0.0536	0.149	0	0.059	0.1836	0.1094	
0.1011	0.1105	0.1312	0.8701	0.1469	0	0.1122	0.1349	
0.2201	0.0833							
C4orf17	0	0	0	0	0	0	0.0567	0
	0	0	0	0	0.0108	0	0	0
	0	0	0	0	0.0356	0	0	0
	0	0	0	0	0	0	0	0
	0	0.0166	0	0.0116	0.1021	0	0.0114	
0.0145	0	0	0	0	0	0	0.0486	0
	0.0169	0	0	0	0	0.0546	0	0
	0	0	0	0	0	0	0	0
	0	0	0	0	0	0	0	0

	0.0462	0	0	0	0	0	0	0
	0	0	0	0	0	0	0	0
	0							
C4orf19	0.3401	0.0606	0.4761	2.4733	1.089	2.5982	1.1231	
1.5146	0.86	0.3002	0.104	1.6558	0.0893	1.2969	0.2317	
1.2805	0.1164	0.0803	0.7093	1.9979	0.2947	1.5916	1.1363	
1.6752	0.6763	0.1068	0.7463	0.3858	0.6411	0.3822	0.2041	
0.9125	0.858	0.1288	0.8966	0.2122	0.3474	1.0182	1.2014	
2.0051	0.291	0.0666	0.4126	1.4393	0.1728	0.2792	1.3475	
1.4977	1.548	0.1672	0.358	2.8493	1.0194	0	0.0736	
1.3144	0.3159	1.5488	0	1.8044	1.7396	0.0163	1.0205	
0.8696	0.1455	0	0	0	0.7075	2.2553	1.3432	
0.7596	0.3327	1.4377	1.4115	2.173	0.3512	1.7904	0.5741	
1.7735	1.3473	0.658	0.2696	1.6878	0.9364	0.5659	0.0953	
0.5362	0.1872							
C4orf22	0	0	0	0	0	0	0	0
	0	0	0	0.0458	0.2158	0.0675	0.3696	0
	1.3777	0	0	0.5234	0	0	0	0
	0	0.2398	0	0	0	0	0	0
	0.0551	0.0469	0.1392	0	0	0	0	0
	0.041	0	0.2317	0	0.2532	0.2739	0	0
	0.0858	0.3962	0	0	0	0	0	0
	0.5994	0	0	0.1675	0	0	0	0
	0	0.9143	0	0.1701	0	0	0.1288	0
	0	0	0	0	0	0	0	0
	0.0747	0	0	0	0.2211	0	0	0
	0							
C4orf26	0.2065	0	0	0.3007	0.0983	0.0465	0.0095	
0.1936	0	0.1541	0.0037	0.0191	0.0434	0	0	
0.0398	0.154	0.1746	0	0.2355	0.1358	0	0	
0.0658	0.0932	0.5017	0.0232	0.1861	0.0207	0.0207	0.0494	
0.1459	0	0.0646	0.0585	0	0.0348	0.0969	0.0652	0
	0.0534	0.076	0	0.1211	0.1409	0.1989	0.0373	0
	0	0.3396	0	0.0592	0	0	0.0373	0
	0.189	0.0365	0.0308	0	0.0275	0.1136	0	0
	0	0.1394	0.0901	0.051	0.0639	0	0.7933	
0.0279	0.0321	0.0108	0.0638	0.037	0.0678	0.053	0.0206	
0.0776	0.0277	0.3829	0.063	0	0.1663	0	0	0
	0.255							
C4orf3	4.0232	3.5917	4.4353	4.1651	3.6195	4.7628	0.3455	
4.0232	4.5564	4.2918	4.3305	4.2058	3.6769	4.4364	4.2544	
3.9205	3.8578	3.9879	3.4664	4.0208	5.0153	4.672	5.0728	
4.0194	4.1914	6.242	4.0917	4.144	3.4711	0.3544	6.3607	
4.0917	4.6258	4.7338	4.6573	4.3151	4.8048	5.1261	4.3206	
4.7738	4.8908	5.3301	4.4609	4.1452	4.2111	4.3474	4.2862	
4.9568	4.1348	4.6171	4.5864	4.95	4.3763	4.1169	4.1164	
3.8558	5.0972	3.5687	3.3488	2.8467	3.5165	4.6316	4.5601	
3.4215	4.4966	6.4328	5.1911	3.035	3.8054	3.1358	5.4726	
3.5374	4.7473	3.6226	4.5855	4.7413	3.5628	5.5197	4.6658	
6.21	4.7649	5.9772	3.9167	4.754	4.5386	4.0363	5.9927	

5.1783	5.2859							
C4orf32	0	1.365	1.8994	3.0404	3.4173	2.2208	3.2261	
1.2984	1.6338	1.9009	1.8208	2.7519	2.9081	2.3136	1.2428	
1.2046	2.8729	0.7142	1.8781	2.9519	1.8636	2.5888	0.9282	
3.0249	1.1232	2.0971	3.7114	1.903	1.9239	2.1664	2.476	
0.2003	2.2694	2.9295	1.2523	2.5527	1.2684	1.6321	1.9019	
3.5493	3.0528	4.637	2.5334	1.8311	1.9533	2.8914	1.1246	
1.656	1.4356	2.1799	1.6881	3.773	1.7569	1.782	3.1161	
1.5397	4.2097	0.9696	3.0799	1.2047	2.3104	0.8561	0.7174	
1.8651	3.2174	2.1239	2.4286	2.251	0.8577	1.0697	2.2919	
1.4757	1.2136	2.3959	1.8677	2.0934	2.4612	1.5221	1.1393	
2.3918	2.4105	3.5141	1.5542	0.9273	4.2697	1.6262	1.4886	
1.3346	1.2176							
C4orf33	2.034	1.6048	1.7548	2.3123	1.6622	2.3182	2.1064	
1.9893	2.3697	1.9903	2.1393	1.8996	0.6675	2.3051	1.5319	
1.9777	1.6911	2.4117	1.7884	2.3523	2.6579	1.3447	1.5295	
1.5277	1.9348	2.4895	1.8433	2.5275	0.7404	1.5195	2.8282	
1.8657	1.8118	2.397	2.5503	2.5872	2.208	1.949	1.3268	
1.6378	1.9598	2.9319	2.0738	2.4456	1.0413	2.8305	2.0038	
2.2656	1.1935	2.9673	2.2728	1.9214	1.9626	1.2697	3.1495	
1.804	1.6677	1.5466	2.198	1.3387	1.3011	1.7425	2.0264	
1.8997	2.3001	2.8367	2.5816	2.3602	1.4895	2.3958	1.9588	
2.1737	2.0388	1.3739	2.4462	3.08	2.1188	1.7029	1.3103	
1.9965	2.7725	1.3551	1.5745	1.7647	2.0118	2.1858	3.1339	
3.0454	3.3455							
C4orf36	0.8238	0.7771	0.6031	0.2253	0.2152	0.1006	0.3753	
0.2365	0.0462	0.3163	0.1352	0.1613	0.7422	0.6102	0.0944	
0.1455	0.8091	0.0927	0.6314	0	0.1078	0.114	0.092	
0.2114	0.2244	0.5909	0.3004	0.2057	0.3396	0.5566	0.5234	
0.3563	0.1419	0.4084	0.0214	0.2327	0.3383	0.2114	0.1765	
0.3349	0.3627	0.3763	0.4392	0.3369	0.0823	0.8025	0.5516	
0.2202	0.0549	1.1482	0.5234	0.1112	0.446	0.5383	0.6472	
0.8403	0.2868	0.3939	0.183	0.1691	0.2406	0.241	0.1567	
0.3085	0.1895	0.2341	0	0.0507	0.1265	0.3432	0.7314	
0.0924	0.3286	0.0925	0.3077	0.6904	0.6739	0	0.2499	
0.715	0.0883	0.1109	0.1426	0.161	0.1088	0.5723	0.4213	
0.1545	0.0961							
C4orf45	0	0	0	0.1428	0	0.1854	0	0
	0	0	0	0	0	0	0	0
	0	0	0.2313	0	0	0	0	0
	0	0.1471	0	0.0822	0	0.0978	0.0325	0
	0	0	0	0	0.059	0	0.03	0
	0	0	0	0	0	0	0.2788	0
	0	0	0	0	0.1804	0	0	0
	0	0	0	0.2376	0	0	0	0
	0	0	0	0	0	0	0	0
	0	0	0	0	0	0	0	0
	0	0	0	0	0	0	0	0
	0							
C4orf46	2.643	2.6135	3.7597	3.4744	2.8745	3.1621	2.7147	

3.0613	3.0627	3.0921	2.6466	2.9728	2.66	3.5222	2.9384	
2.1296	2.5667	2.8632	3.2037	3.765	4.3863	2.9461	2.0907	
2.7634	2.927	2.9113	2.8693	3.9016	2.2752	2.0555	3.3152	
3.1314	3.1343	2.8325	2.5833	3.5017	2.4671	1.6784	2.4888	
2.5477	2.3254	1.9453	3.2834	3.415	3.3306	3.7874	3.3221	
2.6021	2.055	3.7464	1.9621	3.2033	3.3395	2.4661	2.968	
2.232	2.7093	3.554	2.5829	3.6753	2.8134	2.883	3.0472	
3.17	3.7273	3.3132	3.2808	2.3646	2.5593	3.0715	2.9183	
3.1542	3.6884	2.426	2.7449	2.7034	3.4399	2.3467	2.0673	
3.3433	3.1207	2.2662	1.1777	2.5117	3.2497	3.7741	2.7304	
3.6415	3.2707							
C4orf47	0.467	0.5736	0.7915	0.4076	0.2095	0.32	0.884	
0.0361	0.057	0	0.5916	0.656	0.408	0.5947	0.4264	
0.0592	0.7696	0.7477	0.1735	0.4834	0.1319	0.0966	0.3926	0
	0.0833	1.7777	0.0826	0.7395	0.1454	1.3196	2.1822	
0.2578	0.6416	0.5605	0.2313	0.2112	0.2276	0.4398	0.145	
0.9449	0.6169	0.1242	0.1419	0.3778	0.1872	0.7642	0.8767	
0.3858	0.5195	1.1427	0.618	0.8706	1.0895	0.7361	0.3246	0
	0.1779	0.1081	0.051	0	0.3533	1.4102	0.3635	
0.3733	0.7054	0.5785	1.037	1.2362	0.5379	0.0998	0.6981	
0.2336	0.4307	0.9264	0.1606	0.203	0.7912	0.0956	0.4055	
1.1513	0.4673	0.3347	0.0502	0.2329	0.1061	0.6935	0.3914	
0.3462	0.295							
C4orf48	2.7373	3.0885	4.1459	2.3519	3.3818	2.7202	2.7462	
2.1846	2.2262	2.7025	2.4243	2.1869	3.2109	0	2.4228	
2.9009	3.3712	2.133	0	3.6478	3.6077	3.2786	2.5549	
2.5516	2.1839	0	0	3.3178	2.4361	2.8703	2.5485	
4.0338	3.3259	3.2851	2.3761	0	3.0269	4.969	3.0726	0
	3.5076	2.4337	2.313	2.1432	2.1685	0	2.6538	
2.6758	3.2689	0	2.7689	0	0	3.6841	3.1759	
3.9602	2.3025	4.4129	3.3161	4.8083	4.0118	3.579	4.6541	
2.6757	4.0578	3.956	4.3771	5.003	3.5347	3.0276	4.6019	
2.8811	3.7687	3.1508	3.0966	6.204	4.0322	3.7206	3.3393	
4.4876	3.6949	3.5512	4.456	4.4912	3.7114	3.9783	3.6832	
5.1457	4.7252							
C4orf51	0	0	0	0	0	0.069	0	0
	0	0	0	0.1203	0	0	0	0
	0	0	0	0	0	0	0	0
	0	0	0.1298	0	0	0	0	
0.0141	0	0	0	0	0	0	0	0
	0	0	0	0	0	0	0	0
	0	0	0	0.8482	0	0	0	0
	0	0	0	0	0	0	0	0
	0	0	0	0	0	0	0	0
	0	0	0	0	0	0	0	0
	0	0	0	0	0	0	0	0
	0							
C5	1.2974	0	0.4421	1.3562	0.0684	0.7257	0.1687	
0.6973	0.306	0.3436	0.2277	0.2685	1.7719	0.6889	1.1665	
0.3933	0.876	1.5523	0.757	1.6128	0.7805	1.5449	0.4234	

0.4695	1.2857	0.4368	1.4437	0.7932	1.333	0.2531	0.6269	
1.0847	0.864	1.2713	1.3189	0.2371	1.1565	0.1579	1.8018	
0.497	0.7034	0.3946	1.6642	0.7484	1.1003	1.9102	0.9847	
1.1477	0.888	2.159	0.6252	1.1735	1.6729	0.526	1.0876	
0.5368	0.7127	0.8186	0.3353	1.1155	0.4837	0.2799	0.3741	
1.4799	1.6193	0.7061	0.8458	0.6899	0.8073	3.0871	0.5451	
1.3128	0.8118	0.4771	0.6006	0.4709	1.2334	1.0107	0.8963	
0.5137	0.7045	0.2123	0.65	1.6241	0.886	1.7571	0.8961	
1.3373	1.184							
C5AR1	0	0.0966	0	0.2187	0.3555	0.066	0.0244	
0.0642	0.0931	0.0345	0.0429	0.1764	0.2299	0.0788	0.2372	
0.0546	0.1596	0	0	0.2148	0	0.1102	0	
0.2014	0.0175	0.4696	0.126	0.1415	0.0675	0.0407	0.0857	
0.1982	0.09	0.0367	0.0502	0.028	0.73	0.0985	0.0881	
0.1495	0.1001	0.9037	0	0.0443	0.2102	0.4233	0	
0.0969	0	0	0.0724	0.0456	0	0.4197	2.0342	
0.0741	0.8163	0.0361	0.0544	0.0166	0.0105	0.0252	0.0432	
0.1098	0.1854	0.3395	0.0287	0	0.0702	0.1627	0.1917	0
	0.0684	0.0318	0.0365	0.0327	0.0708	0.0145	0	
0.1095	0.0626	0	0.2046	0.0161	0.0689	0	0	
0.0638	0							
C5AR2	0	0	0	0	0	0	0	0
	0.0585	0.068	0	0.0662	0	0	0.0547	
0.0483	0	0	0	0.2533	0	0.04	0.0284	
0.0376	0	0.0565	0	0	0	0	0.0918	
0.0494	0.0731	0.0741	0.1057	0	0	0	0.0555	
0.0841	0	0	0	0.0772	0	0	0.115	0
	0.0981	0	0	0.0789	0	0.0403	0.1799	0
	0.1179	0.0567	0.1488	0	0.0195	0.0388	0	0
	0	0.0471	0	0.0174	0	0	0.0463	
0.0391	0	0	0	0	0.1298	0.0527	0	
0.0999	0	0.0647	0	0	0.0784	0	0.2437	0
	0							
C5orf15	4.4401	4.8334	4.9749	6.1363	6.0988	5.1952	6.6042	
5.4604	6.4653	5.2061	5.9425	6.1619	6.236	5.5151	6.5532	
5.6054	5.1315	5.0709	5.6243	6.0265	5.1861	5.9414	6.274	
6.4175	6.0177	5.6147	6.0274	5.8591	5.5574	5.0876	5.9714	
6.6874	6.3366	6.1848	6.7222	5.636	4.336	5.5368	4.4317	
7.1212	5.7228	6.4724	6.3235	5.3264	6.2165	5.945	4.7563	
5.7365	6.0727	5.7201	5.9143	6.7059	6.1022	5.539	5.0123	
5.0441	5.5253	5.6631	5.3528	5.0216	6.4557	5.9258	6.2943	
4.7891	5.0565	6.8672	5.7797	4.6998	5.3213	6.718	5.5516	
5.66	5.546	6.849	5.9624	4.9586	5.5724	6.4493	5.937	
5.7581	6.1034	6.7625	4.7599	6.5249	5.4497	5.7424	5.1924	
4.6698	5.0655							
C5orf22	5.4182	5.2025	4.2856	4.7504	3.8144	4.3624	3.9077	
4.1522	4.9809	4.4336	5.7122	4.2601	3.8468	4.6346	4.2736	
4.8357	3.4295	4.9222	5.2472	3.7823	5.0594	4.2811	3.9061	
4.3565	4.1212	3.8015	4.1561	4.1981	4.1069	5.4737	4.3394	
3.3363	4.0034	3.4671	4.4167	4.9088	4.2649	3.7438	4.5225	

4.4025	4.4002	4.0718	4.2972	4.8508	3.7966	4.0156	4.2227	
3.8442	3.888	3.7043	4.4236	4.6466	4.444	4.6888	4.5486	
4.2563	3.4413	3.665	4.9366	3.7742	4.5057	5.1552	4.254	
4.5647	4.8386	3.2736	5.1476	3.3775	3.5288	3.9614	3.6433	
4.7577	4.5087	4.3245	4.4015	4.4244	4.7913	4.8671	4.0927	
4.8961	4.3309	4.6473	3.6494	4.4344	4.0393	4.4235	4.203	
4.2882	4.1335							
C5orf24	4.0544	3.6101	4.8279	4.6868	4.7754	5.2672	5.2003	
4.5565	4.353	4.3151	4.623	4.7074	4.641	4.8666	5.1857	4.8
	4.7093	4.3312	4.4232	4.7843	4.7277	4.2707	4.4379	
4.4514	4.874	4.8424	4.8113	4.9928	4.8459	3.6486	4.7716	
4.9352	4.6321	4.457	5.0362	4.5303	3.2475	4.6167	4.0698	
4.8043	4.2827	4.8811	3.9864	3.8005	4.9359	4.7827	3.6017	
4.5505	4.9346	4.4925	4.6615	5.0197	4.6576	5.044	4.0138	
3.8358	4.632	4.0336	3.966	4.2332	5.024	4.5069	4.6118	
5.1736	4.6305	4.5903	4.919	5.4631	4.1309	4.5903	4.5417	
4.3725	4.9462	4.0621	4.4636	4.8397	4.9166	4.8799	4.9367	
3.9704	4.2525	5.1448	4.8786	4.8845	4.1208	4.3131	4.8666	
4.7749	4.8471							
C5orf30	3.1554	3.2854	4.4648	4.0517	3.6938	3.5575	4.1513	
3.5532	3.1078	3.9439	3.8428	3.8948	2.9083	4.2329	4.4794	
3.5314	3.2956	3.5724	4.3819	4.2104	3.58	3.9319	3.9908	
3.9074	3.1474	4.4696	4.3644	5.0068	3.6495	4.0822	2.5084	
4.6057	4.201	5.0497	3.9625	3.6456	3.0098	3.0958	2.5053	
4.0416	3.9706	3.486	2.9709	3.9789	3.3673	3.1443	4.3156	
3.5204	3.4883	4.1972	3.9115	3.1133	3.5025	3.1519	2.5027	
3.1198	5.2533	2.8262	3.4007	3.134	3.7751	4.1167	3.8573	
3.9715	3.5406	3.6657	3.9517	3.2811	3.6239	3.6153	4.0377	
3.8397	3.037	3.6711	3.1019	4.0865	4.3196	3.0512	4.4366	
4.4874	4.2453	4.1102	3.5414	2.867	4.1583	3.5166	3.5092	
3.5761	3.3978							
C5orf34	2.549	3.1908	2.7433	3.2471	2.8157	2.0582	1.8458	
2.3546	2.5645	2.2587	1.8303	2.2693	3.0434	2.8199	2.6717	
2.2123	2.1666	2.6312	3.2447	2.0304	2.0766	2.5721	2.5824	
2.6364	2.6674	1.9661	1.4908	2.0103	2.8844	2.8964	1.6961	
1.6452	3.0496	1.912	2.1656	2.8216	2.0275	1.1829	2.1392	
2.3565	1.5028	1.5755	3.3239	3.0331	2.3263	3.1013	2.6324	
2.232	1.8276	2.5264	1.6378	3.1339	2.4551	2.1052	2.1161	
3.0511	2.0845	2.1106	3.1234	2.466	2.0244	2.8928	2.1318	
2.8357	2.2471	1.1049	2.603	1.2399	1.8832	2.8965	1.8102	
3.026	2.2827	2.3868	1.5556	2.1152	2.6175	1.8195	2.5918	
2.742	2.8021	1.3819	1.6716	1.419	1.7691	2.9943	1.5085	
2.7181	1.7008							
C5orf38	0	0	0	0	0.1577	0	0	0
	0.6492	0	0.1454	0.1859	3.1322	1.7052	0	0
	0	0	0	0	2.8261	0	1.5047	0
	1.0012	0.4804	0	0	0	0	0	0
	0.2428	0	0	0.2665	2.2326	0	0.1637	0
	0	0	0	0	0.3144	0.8418	0	0
	0	2.1429	2.0849	0	0	0.104	1.4099	

2.2499	0.0569	0.047	0	0	0.8566	2.1692	0.0401	
2.2595	0.0498	0	0	0.1303	0	2.2441	0	
0.1414	2.7264	0	0	0	0.1271	0.052	0	
0.1244	0.0542	0.2578	0.0376	1.3416	0	0	1.7142	0
	0							
C5orf42	2.9285	2.2195	1.9906	2.2171	1.6378	2.1837	1.7003	
1.685	2.7938	2.3325	1.1527	1.5827	1.9249	1.4827	1.9978	
2.1517	2.0198	2.5186	2.4793	1.9249	1.9002	1.4186	1.4447	
0.9416	2.2274	2.2399	1.6921	1.8498	1.8003	2.839	3.1041	
0.5273	1.7807	1.8098	2.3805	2.55	1.9302	1.5381	1.0135	
2.7144	1.5562	2.1577	2.4949	1.8607	2.306	2.1289	2.3183	
1.9061	1.7773	1.807	2.1298	2.2656	1.7334	1.8238	2.3442	
2.1397	2.026	1.4372	2.9778	1.705	1.7101	2.2433	1.317	
1.5816	2.0023	1.8454	0.2701	2.6322	1.9687	1.6719	1.6611	
2.0248	1.9043	1.4431	1.7364	2.7302	2.0728	2.2867	2.1797	
1.9492	2.3549	2.4511	1.6357	2.0502	1.1695	1.4829	1.8162	
1.904	1.8836							
C5orf45	6.7854	5.47	6.5473	6.2409	7.0718	6.3867	7.5579	
7.6689	6.2304	5.5378	4.5688	7.3095	6.9534	7.1227	7.1119	
5.7087	6.9394	5.5295	8.1486	7.2844	5.8275	6.8157	7.3944	
7.6854	6.5127	6.8077	6.646	6.3186	6.0589	5.2678	7.6016	
8.8274	6.4377	7.0289	8.1244	6.1692	7.3517	7.8447	5.832	
5.8843	7.2195	7.442	6.7307	7.4484	5.1785	7.0171	5.3712	
7.046	7.8742	5.1961	8.8872	6.6405	6.4328	6.0638	7.6275	
6.2186	7.699	5.2138	6.5357	5.9677	7.098	6.1873	7.4753	
6.3269	5.7233	8.7031	6.152	7.7328	5.3943	6.1464	5.6301	
6.7188	7.6399	6.5661	5.6014	6.301	6.2893	7.5885	6.595	
6.1545	5.9233	5.7314	8.9427	7.6749	6.1425	6.0736	6.0046	
5.8727	5.7964							
C5orf46	0	0.6308	0	0	1.2954	0	0	0
	0	0	0.1746	0.9733	0.8696	0	4.5374	0
	0	0	0	0	1.2427	0.0889	1.9528	0
	0	6.3075	0.29	0	0.5687	0	0	0
	0	0.2995	0.768	0	0.5067	0.1639	0.4016	0
	0	0.5244	0	0.286	0.8306	0	0	0
	3.5393	0	0	1.0251	0	0	0	0
	0.2753	0	0	1.1446	0.4281	0.4206	0	0
	0	6.8126	0	0.4944	0.5314	0.2906	0	0
	0	0.1991	1.7399	0	0	1.8495	0	0
	0	1.1759	0.1505	0.18	2.6135	0	0.3548	0
	0							
C5orf47	0.0895	0	0.224	0.0334	0.0095	0.0355	0.1851	
0.0821	0.0153	0.0396	0.0218	0.0087	0.0543	0.0518	0.071	
0.1233	0	0	0.2924	0	0	0	0	
0.3361	0	0	0.02	0.0462	0	0.0825	0	0
	0.3938	0.0207	0.0048	0.0135	0.0116	0	0.0154	
0.0551	0.0204	0	0	0	0	0	0.0657	0
	0	0	0.0392	0.0222	0	0	0	0
	0	0	0	0	0	0.1282	0.0274	
0.047	0.0746	0.1046	0.0592	0.1802	0	0	0	

0.0522	0.0527	0	0	0.4106	0.089	0	0.1365	
0.0466	0	0	0	0.0304	0.0301	0	0	0
	0							
C5orf49	0	0	0	0	0	0	0.0196	0
	0.1104	0.1255	0.1959	0	0	0	0.1733	
0.1753	0	0.2308	0	0	0.1137	0.0319	0	0
	0.0788	0	0	0	0.1684	0.9811	0.324	
0.0039	0.0406	0.031	0	0.0227	0.0122	0	0.0225	0
	0	0.0551	0	0	0.0927	0	0.0918	
0.0859	0.0276	0	0.0619	0	0.1779	0.2751	0.1679	0
	0	0.1395	0.4907	0.1045	0	0.0452	0.0635	
0.1184	0.0821	0.0215	0	0.3011	0.0384	0	0.0573	
0.0729	0	0.0696	0.0276	1.339	0	0	0.0558	0
	0	0.0079	0.0115	0.0513	0	0.038	0.2308	
0.3104	0.3001							
C5orf51	4.7977	4.6359	4.135	4.604	3.6356	4.0658	3.9945	
4.0455	4.5045	4.6504	4.6608	3.9892	4.6317	3.973	3.8262	
4.4175	4.017	4.4025	4.389	3.9265	4.3269	3.4085	3.8274	
3.9726	3.8467	4.5529	3.1395	4.7421	3.9607	5.1161	3.5697	
3.0077	3.79	3.1678	4.4526	4.9703	4.1815	4.4175	4.021	
4.1339	3.7809	3.7073	4.0738	4.0856	3.7117	3.9844	3.9373	
4.0036	3.611	3.5846	4.4254	4.4535	4.0706	4.3435	3.6475	
4.1879	4.1183	3.8981	5.2571	3.9842	3.954	4.6476	3.5376	
4.2376	4.38	4.2327	4.3098	4.2853	3.8965	4.0177	3.6155	
4.2557	4.4375	3.8799	4.0156	4.5555	4.2542	4.8141	4.2521	
4.4581	4.2151	4.8559	3.556	3.9438	3.2662	3.5792	4.6625	
4.3518	4.5821							
C5orf52	0	0	0	0	0	0	0	0
	0	0	0	0	0	0	0	0
	0	0	0	0	0	0	0	0
	0	0	0	0	0	0	0	0
	0	0	0	0	0	0	0	0
	0	0	0	0	0	0	0	0
	0	0	0	0	0	0	0	0
	0	0	0	0	0	0	0	0
	0	0	0	0	0	0	0.047	0
	0	0	0	0	0	0	0	0
	0	0	0.0466	0	0	0	0	0
	0							
C5orf63	0	0	0.0678	1.7392	0.4692	0.007	0.3694	
1.3993	0.4904	0.7243	0.6541	0	1.1463	0.7305	0.8121	
1.2521	0	0.7518	1.2816	0	1.1863	1.5091	0.7857	
0.8291	0.7453	1.2325	1.2836	1.5631	0.2609	1.3905	1.0085	0
	0	0.9611	1.1187	0.7785	0.8043	0	0.2977	
0.8043	0.985	1.5385	0.1604	0.5593	0.4965	1.0343	1.4815	
1.3681	0.9371	0.038	1.3575	1.957	1.2289	1.6514	0.7395	
0.0921	0.781	0.1074	1.6549	0.0965	1.0557	0.0632	1.7433	
1.1501	1.0144	1.5013	0.4242	0.0685	0.0681	0.378	0.798	
1.0112	0.1283	1.0837	1.7444	0.7623	0.0962	0.87	0.3985	0
	0.372	1.1151	1.6465	1.3229	0.8834	1.2886	0.4562	

1.161	1.1672							
C6	0	0.0212	0	0	0.0205	0	0	
0.0055	0	0	0	0	0	0.0244	0	0
	0	0	0	0	0	0	0	0
	0	0.0401	0	0	0	0.0107	0	0
	0	0	7.00E-04	0	0.0021	0	0	0
	0	0.0057	0	0	0	0	0	0
	0	0	0	0.0119	0	0	0	0
	0	0	0	0	0	0	0	0
	0.0354	0.0085	0	0	0	0	0	0
	0	0	0	0	0.0029	0.0125	0	
0.0281	0	0	0	0	0	0	0.0278	0
	0							
C6orf1	4.6	4.9266	4.9112	3.6338	4.5517	4.0816	4.2204	
4.4996	4.526	5.0179	4.8816	4.9112	4.7898	4.3532	4.848	
4.186	4.2283	4.4878	4.2918	5.0057	4.9245	4.7451	5.2897	
3.8324	4.4148	4.8541	5.1267	5.3558	5.2644	4.0138	3.2884	
5.3749	4.3713	5.6844	5.1445	4.0804	5.3937	5.933	5.1679	
3.2816	5.1272	4.5285	5.2695	4.3565	4.6306	4.5827	3.5523	
3.7165	5.3186	3.7274	4.6202	4.0171	3.8676	4.7948	2.7635	
4.5167	4.643	4.0804	3.9622	4.1535	4.9984	4.5823	4.8221	
4.8268	4.9956	4.143	5.6354	5.8625	4.3466	3.9404	5.1827	
5.0343	4.6257	4.9449	4.6082	4.8717	4.9809	5.6139	4.664	
5.2369	4.4297	4.9463	6.2325	4.7983	5.2905	4.8321	5.8697	
5.5412	5.4397							
C6orf10	0	0	0	0	0.12	0	0.0698	0
	0	0	0.0019	0.0351	0	0	0	0
	0	0.0259	0	0	0	0	0	0
	0	0	0	0	0	0	0	0
	0	0	1.00E-04	0	0	0.0202	0	
0.0088	0	0	0	0	0	0.0101	0.0205	0
	0	0	0.1059	0.0165	0	0	0	0
	0	0	0	0	0.0249	0	0	0
	0	0	0	0	0	0	0	
0.0394	0.0257	0	0	0	0.2417	0	0	0
	0.0136	0	0	0	0	0	0.0889	
0.0307	0.2316							
C6orf106	4.557	5.2516	5.8351	5.2829	3.939	4.8718	4.507	
4.9345	5.393	6.0625	5.1503	5.8288	4.0675	4.8837	4.8964	
4.475	4.4244	5.8471	5.6486	5.0106	4.9549	4.9687	5.1371	
4.2625	5.1061	5.4553	5.3563	6.0587	4.9491	4.8974	4.3789	
5.1458	4.7289	5.0774	5.1321	5.8066	4.8128	5.9575	5.2177	
4.2433	5.2465	5.1463	4.7528	4.4788	5.4545	5.3973	5.0794	
5.1032	4.9303	4.9726	5.2593	5.187	4.9649	5.0167	4.9919	
5.263	5.1861	4.787	4.3753	4.6741	4.698	4.6024	5.329	
4.8625	5.8191	4.1844	5.9282	4.4598	5.2127	4.2049	4.9362	
5.1683	4.6634	4.9945	4.5628	5.6176	5.694	5.8336	5.0521	
5.1239	4.7179	5.1549	6.1082	5.0604	4.8308	4.3372	5.1581	
4.7653	5.3897							
C6orf118	0	0	0.101	0.2397	0.0596	0.1408	0.129	0

	0	0	0	0	0	0	0.0171	
0.0201	0	0	0	0.059	0	0	0	0
	0.0724	0	0	0	0	0	0.0458	
0.0063	0.0403	0	0	0	0.0369	0	0	
0.0272	0	0	0	0	0.0185	0.0301	0	0
	0.0593	0	0	0	0.0753	0.0089	0.0996	
0.0543	0	0.0233	0	0.0738	0	0	0	0
	0	0	0	0	0	0	0.0378	0
	0.0466	0.0056	0.0142	0.0738	0	0	0.0075	
0.0368	0.1125	0	0	0.021	0.2054	0.0943	0	0
	0							
C6orf120	3.0411	3.1322	3.0926	4.0963	3.4056	2.8328	3.5247	
3.5797	4.1132	3.4348	3.1266	3.89	2.6507	3.9513	3.4832	
2.2675	4.2755	3.9284	3.9383	2.3519	3.9068	4.2239	4.4306	
3.8363	2.6342	3.8607	3.0759	2.3326	4.2087	3.5725	3.8863	
4.1129	3.6725	3.7345	3.941	3.5755	3.0574	3.6762	3.4659	
4.3831	4.6506	4.5537	3.3302	2.0829	4.1121	4.104	3.9302	
4.068	3.5686	4.3653	3.9265	4.4733	3.3483	4.9602	4.3379	
3.4047	4.2296	3.0709	2.4491	3.1315	4.0208	3.7984	4.1894	
2.6351	2.5336	4.0067	3.6189	3.4098	3.2135	3.3285	3.5724	
3.8523	3.8659	4.3674	3.5456	2.8303	3.1336	4.2862	3.4387	
3.8854	3.671	3.9867	4.1579	4.366	3.6571	2.7264	3.8	
3.8844	3.4663							
C6orf132	0.0664	0.0912	0	0	0	0	0.0062	
0.0026	0.0292	0.1554	0	0	0.0129	0.0523	0.0112	0
	0.1734	0.0667	0	0.0402	0.0337	0.0039	0.0039	0
	0.0018	1.4726	0.0488	0.0315	0	0.0193	0.005	0
	0	0	0	0.0567	0.0394	0.019	0.0275	
0.017	0.0094	0.2043	0	0	0.1056	0.0085	0.0226	
0.0222	0.0291	0.0737	0	0.0124	0.0369	0.0134	0.0183	
0.047	1.6856	0.0352	0.0238	0.0155	0.0166	0.0132	0.011	
0.0273	0.0273	0.0051	0.0336	0.0064	0.0073	0.0328	0.0324	
0.0351	0.0117	0.0045	0.0165	0.0142	0.0237	0.011	0.0192	
0.0254	0.023	0.016	0.008	0.0209	0.0504	0.0599	0.0799	
0.0714	0.0318							
C6orf136	3.2958	3.9945	3.5864	3.7584	2.6934	2.9608	2.3255	
3.5693	3.2477	4.1884	3.5088	3.8352	2.0864	3.4101	2.7855	
3.2496	3.2539	3.4108	3.6053	3.5504	3.7369	3.3131	3.1754	
2.9831	2.5575	2.3928	3.9855	3.418	2.8092	3.5735	2.5123	
3.2644	3.0045	3.1543	3.0478	3.8957	3.7738	3.3984	3.0993	
2.5872	2.8345	3.3878	2.3887	3.0885	2.9024	3.5685	3.0271	
2.8739	2.5003	3.5852	3.0029	3.0924	3.1818	2.4533	3.1354	
3.3746	2.7062	3.4539	2.9864	3.3624	2.6925	3.8537	3.3036	
3.0858	3.1426	2.4012	3.7475	3.2082	2.7385	2.328	2.8711	
3.0661	2.6964	2.7844	2.4208	3.5368	3.8754	3.6553	2.8336	
3.7706	3.5839	3.137	4.2446	2.8771	3.4509	3.476	3.3295	
3.1854	3.4033							
C6orf141	0.0568	0	0	2.0634	2.1132	0.1246	0.8842	
2.3325	0	0.021	0	1.1283	0.9994	0	0.3432	
0.1067	2.1773	0	0.0763	0	0	0.2678	1.1617	

0.5639	4.652	0.7663	1.9832	0	0.1149	0	0	
0.1153	0	0.0243	0.3129	0.023	0.0777	0.0385	1.5731	
1.071	0	2.4211	0.7731	0.4601	2.9033	1.9403	0	
0.2664	2.7284	0	0.0767	1.7993	0.0612	0	0	
0.0958	1.7914	0.4114	0	0.5825	0.8978	0.152	0.524	
0.0746	0	1.9345	0	0.9056	0	0.1845	1.5674	0
	0.0519	1.9879	0.0434	0.2371	0	0.0498	0.0412	
0.155	0.4573	1.5604	0.1279	0.0809	4.19	0.0948	0.0811	
0.0383	0							
C6orf15	0	0	0	0	0	0	0	
0.041	0	0	0	0	0	0	0	0
	0	0	0	0	0	0	0	0
	0	0	0	0	0	0	0	0
	0	0	0.0182	0	0.0961	0	0	0
	0	0	0	0	0.0374	0	0	0
	0	0	0	0	0	0	0	0
	0	0	0	0	0	0	0	0
	0	0	0	0	0	0	0	0
	0	0	0	0	0	0	0	0
	0	0	0	0	0	0	0	0
	0							
C6orf163	0.2206	0.0907	0.3157	0.1383	0.0918	0.4179	0.0937	
0.1656	0.4107	0.1963	0.2184	0.156	0.0193	0.4661	0.1831	
0.081	0.8709	0.2203	0.5188	0.2649	0.3276	0.0556	0.067	
0.3168	0.0725	0.2895	0.0495	0.2335	0	0.1691	0	
0.1232	0.1455	0.2809	0.0985	0.0791	0.1144	0.1542	0.1258	
0.488	0.2224	0.0447	0.0672	0.53	0.2213	0.1275	0.1703	
0.0867	0.2714	0.4453	0.0888	0.1656	0.378	0.3924	0	
0.5577	0.1313	0.3028	0.3401	0.1798	0.1899	0.0279	0.0805	
0.0521	0.2857	0.0915	0.4811	0.0851	0.0243	0.2599	0.1605	
0.0901	0.0365	0.3117	0.2722	0	0.2124	0.2624	0.0503	
0.0653	0.4094	0.1606	0.3	0.2588	0.0903	0.4326	0.1776	
0.2227	0.2086							
C6orf201	0.2895	0.0666	0.1794	0.2299	0.014	0.2188	0.074	
0.0713	0.1361	0.3	0.0951	0.1034	0.1464	0.1299	0.1204	
0.1542	0.6723	0.1498	0.1436	0.1043	0.3559	0.1212	0.0713	
0.0892	0.074	0.1188	0.4051	0.3315	0.1273	0.0848	0.0846	
0.0566	0.0832	0.1155	0.3618	0.1324	0.2618	0.1194	0.0386	
0.2133	0.0719	0.099	0.1942	0.3966	0.1511	0.294	0.2195	
0.0483	0.1578	0.3416	0.1197	0.242	0.1891	0.0634	0.1258	
0.1317	0.0483	0.0462	0.1441	0.1762	0.0857	0.2076	0.0437	
0.2813	0.1872	0.0573	0.3638	0.0801	0.2315	0.1767	0.0976	
0.0725	0.0684	0.1627	0.2667	0.2883	0.1434	0.1678	0.0941	
0.0813	0.06	0.3231	0.0759	0.1628	0	0.3483	0.0565	
0.1667	0.3946							
C6orf203	3.1821	3.2711	2.8026	3.7754	2.6034	2.7935	3.0707	
2.4216	3.5259	2.5899	2.6572	2.7405	2.2224	3.726	3.5114	
2.346	2.7165	3.4621	3.3989	3.1802	2.9097	3.225	3.3845	
2.9044	2.9923	3.6438	2.0158	2.5354	3.5244	3.1755	2.8235	
2.9452	3.1411	2.706	3.3515	3.1282	2.3877	2.7363	2.8661	

3.4922	3.4739	3.5244	3.1552	3.0652	2.867	3.4525	3.4196	
2.5979	2.8416	3.3494	3.3011	3.6477	3.0818	2.6948	2.9687	
3.0986	3.2546	3.0109	2.8222	2.9989	2.4785	3.9308	2.4564	
2.9204	2.7724	3.1655	2.5499	2.9126	2.89	3.0766	3.2011	
3.2108	2.5648	4.0148	2.7411	2.2141	3.2648	3.0468	2.6163	
3.0861	3.1694	3.6861	3.6156	3.2349	3.2401	3.4183	3.1399	
3.5424	3.4538							
C6orf222	0	0	0	0	0	0	0	0
	0	0	0	0	0	0	0	0
	0	0	0	0	0	0	0	0
	0	0.0292	0	0	0	0	0	0
	0	0	8.00E-04	0.0063	0	0	0	0
	0	0	0	0	0	0	0	0
	0	0	0	0	0	0	0	0
	0	0	0	0	0	0	0	0
	0	0	0	0	0	0	0	0
	0	0.0081	0	0	0	0	0	0
	0	0	0	0	0	0	0	0
	0							
C6orf223	1.0178	2.6391	2.3048	0.0652	0	0	0.0064	
0.0089	0	1.2866	0.0265	0.6664	0	0	0.0313	0
	0.6804	0.6878	0.1383	0.0931	0.0258	0.1173	0.0162	0
	0	0.0227	0	0.1131	0	0.2093	0	0
	0	0	0.1392	0.5249	0.3037	1.848	0.0477	
0.0144	0.154	1.6163	0.4659	0	0.0057	0.0331	2.2422	
0.3224	0	0	0.1522	0	0.1289	0.1439	0	
0.7649	0.5046	1.3087	0	1.9741	0	0.0147	0.3235	0
	0.0993	0.0708	0.04	0	2.2461	0	0	0
	0	0.2173	0	1.7279	0.1316	0.0414	0.0253	
0.0707	0.0175	0.0634	1.4228	0.0257	0.0986	0	0.0931	
0.0434	0.0504							
C6orf226	2.54	2.6373	1.4633	1.9313	1.3472	2.6129	1.1491	
2.6296	2.6056	3.838	2.54	2.6267	1.4728	0.922	2.1293	
1.9908	2.3778	2.7685	2.2688	2.734	3.8424	2.7703	3.3646	
0.5254	1.4964	2.4017	4.8001	3.1452	2.6095	1.5767	2.5086	
3.5546	2.9104	2.6916	2.307	2.807	4.0855	3.831	3.67	
2.0262	2.5246	3.2936	2.0054	2.1558	2.252	2.7437	1.9247	
1.8685	2.2381	2.4148	2.0089	2.4352	1.9027	2.6208	2.7126	
2.0036	3.0106	0.637	1.8902	0.0815	2.2924	2.9163	2.1757	
2.3065	3.24	3.4907	3.688	3.2123	2.3715	1.6328	2.2865	
2.2593	2.7522	2.7643	3.0753	3.0816	3.5271	4.1361	3.4928	
2.4307	1.7757	3.1722	3.7557	2.7782	3.934	2.4968	3.5185	
2.7929	3.1375							
C6orf229	0	0	0	0	0	0	0	0
	0	0	0	0	0	0	0.0562	0
	0	0	0.1533	0	0	0	0	0
	0.048	0	0	0	0	0	0	0
	0	0.1639	0	0	0	0	0	
0.0651	0	0	0	0	0	0.0719	0	0
	0	0	0	0	0	0	0	0

	0	0	0	0.0515	0	0	0	
0.067	0.0543	0	0.0814	0	0	0	0	0
	0	0	0	0.193	0.0284	0	0.029	0
	0.0412	0	0	0	0.053	0.0755	0	0
	0							
C6orf25	0	0.0728	0.0554	0	0.0878	0.3776	0.0667	
0.1513	0.1854	0.1187	0.0997	0.0276	0.0395	0.1052	0.149	
0.1362	0.6442	0.282	0	0.1024	0.199	0.0961	0.0386	
0.1511	0.0898	0.0444	0.3073	0.097	0.1092	0.0548	0.0184	
0.0165	0.0636	0.0277	0.0447	0.0207	0.0351	0.1305	0.0451	
0.2186	0.0674	0.0069	0.1059	0.2244	0.0817	0.1568	0.0441	
0.0954	0.0234	0.0794	0.0961	0.0582	0.0624	0.0906	0.1132	0
	0.0337	0.1377	0.3683	0.1018	0.1922	0.0146	0.0224	
0.2724	0	0.0594	0.3171	0.0565	0.0693	0.1075	0.0518	
0.1393	0.0675	0.0226	0.0195	0.0962	0.2454	0.1195	0.0089	
0.099	0.0589	0.0599	0.0471	0	0.2265	0.0802	0.1301	0
	0.1201							
C6orf47	2.9819	3.4632	3.7302	3.7524	2.7959	2.5259	3.2733	
3.5502	3.0136	3.7302	2.8849	3.9352	2.9097	3.3218	3.2021	
2.5745	3.4031	3.5339	3.3873	2.9455	3.3331	3.3744	4.078	
2.4489	3.5148	3.9684	2.7794	3.8137	3.6853	2.7038	3.2004	
2.9385	2.8473	3.1134	3.3339	3.7743	4.2844	3.9937	4.0372	
2.697	3.1215	3.7372	3.2445	2.7205	3.0408	3.8074	2.9582	
3.0221	3.5653	3.1328	3.3727	4.0163	3.0736	3.2003	3.6733	
2.9068	2.4209	3.4237	2.5246	3.3379	3.6714	3.2974	3.4296	
2.5228	3.4527	2.4324	3.7732	3.8974	2.9169	3.1367	3.4021	
3.3179	2.8719	3.5254	2.7013	3.367	3.6876	4.1523	3.286	
3.2519	2.6442	4.0053	3.7855	3.2476	4.1136	3.2234	3.3252	
3.6067	3.3339							
C6orf48	7.6254	6.7952	6.4468	7.5517	5.943	7.2682	5.8645	
7.0889	6.759	8.0409	7.4852	7.4193	6.9317	7.3343	6.7821	
6.1585	7.6301	7.8203	7.0333	6.2421	6.8515	7.1932	6.6009	
6.1462	7.6301	7.9741	9.1386	6.5018	6.7207	6.8062	6.6257	
5.8858	6.3733	6.3391	6.5187	7.4815	7.0357	7.8106	7.8872	
6.8062	7.4193	6.1478	6.1264	7.9405	6.7228	8.6885	6.3655	
6.7461	8.5502	9.0054	7.4577	8.1973	8.7612	8.8416	6.4427	
6.4427	6.9445	6.6249	5.6154	6.6426	7.7126	7.7682	7.7827	
6.9466	6.8811	7.1024	8.7678	7.6012	6.2307	7.2817	6.6014	
7.3177	6.9269	6.4162	6.996	5.7821	6.8714	6.8161	7.1428	
7.3463	6.6456	8.353	8.072	6.4557	7.1885	7.8801	7.5892	
7.5441	7.8044							
C6orf52	1.0946	0.6571	1.1872	1.3886	0.5421	1.6584	1.1834	
1.3591	0.9506	3.0575	0.5179	0.2683	1.4875	1.2146	0.7901	
1.1082	2.5934	1.6759	1.509	1.9191	0.7312	1.6945	0.1253	
2.4299	1.5948	0.2449	2.2698	1.5929	0.4243	0.3546	0.4036	
1.6619	0.6561	1.4627	1.7298	2.7172	1.0209	0.5798	3.0026	
0.548	0.7895	0.3215	0.3381	2.0776	0.1088	2.2948	1.4891	
0.448	0.3754	2.1433	1.3781	1.6572	1.1492	1.7308	0.9397	
2.118	1.9575	1.3872	1.8596	0.6656	0	1.8228	0.3544	
0.8002	1.6044	0.1487	1.9095	0.9666	0.9469	1.8427	0.7327	

2.1444	1.1611	0.1035	1.4256	1.5405	2.5605	0.7942	0.7345	
2.0179	0.8299	1.0963	0.1248	1.5482	3.0865	1.4339	1.6864	
0.8343	1.7381							
C6orf58	0.0463	0.0625	0	0.6051	0.1848	0	1.5908	
0.4011	0.1265	0	0.1225	0.1151	0.3995	2.2373	0.0794	0
	1.1222	0.1622	0.3286	0	0.0492	0.7548	0.1466	0
	0	0.723	0.0085	0.2044	0	0.1803	0.0074	
0.8591	0	1.1173	0.7699	2.00E-04	0.0254	0	0	
0.0334	0.4509	0.006	0.2665	0.8216	0.0232	1.1252	0.0221	0
	1.8956	0.5841	0.4683	0.46	0.7944	0.1043	0	
0.2185	0.7834	0.1421	0	0	0	0.1923	0.1268	0
	0	1.4215	0	0	0	0.2705	0	
0.1342	0.5836	0.1587	0.2233	0	0.1037	0	0.1076	0
	0.1247	0	0.1181	1.0901	0.4502	1.3473	0.0695	
0.0371	0.046							
C6orf62	5.2656	5.5555	5.5864	6.4535	5.4116	5.5805	5.8033	
5.8296	5.8434	6.227	5.2918	6.4292	5.0188	6.013	5.8238	
6.1611	6.0105	6.6814	6.0281	5.0889	5.6804	6.378	6.4434	
5.72	6.1857	6.7399	4.0682	6.0323	5.7592	3.3671	5.0917	
5.711	5.0062	5.5075	6.1028	6.4434	6.8812	5.9426	5.4486	
5.6602	5.5667	5.9915	5.4144	5.8473	6.026	6.961	5.791	
5.3078	6.1214	6.768	6.1689	6.7912	6.1291	6.1313	5.1782	
4.6665	5.3763	5.2862	5.2384	5.7109	6.5963	6.0562	5.8176	
5.7763	6.0329	5.7573	6.1378	6.2063	5.1495	5.5359	5.8066	
5.9187	5.214	6.5594	5.492	5.8164	5.7335	7.2107	5.3763	
5.2441	4.9792	6.4948	7.0464	5.3172	5.8318	5.944	6.0312	
5.702	5.6562							
C6orf89	3.5816	4.1207	3.3339	4.468	3.3351	3.6967	3.3058	
3.6527	4.0034	4.6847	4.0043	5.1493	3.2579	4.1083	3.644	
3.2226	4.0538	4.8296	4.1536	3.2939	3.423	4.7327	4.436	
3.7705	3.6056	4.7824	4.8857	4.2465	4.3193	3.7305	3.6126	
3.8159	3.471	4.1478	4.2422	4.3254	4.0673	4.9645	4.5	
3.4718	4.4827	4.7432	3.901	4.1964	4.1207	4.5361	3.6677	
3.6389	4.0133	4.1654	4.3231	5.3487	4.4871	4.2169	3.393	
3.472	3.8846	3.8498	3.2732	3.8556	4.3563	3.7665	4.0861	
3.6139	4.194	3.927	4.7098	3.7958	3.8621	3.5262	4.0049	
3.7563	3.5152	4.6694	4.1698	3.735	3.9306	5.7888	4.0462	
4.3107	3.8	5.2366	4.498	4.5028	3.775	3.913	4.421	
3.9816	4.4766							
C7	0.0678	0.0278	0	0.4578	0.0925	0.0249	0	
0.0774	0.0089	0	0.0494	0	0.0121	0.0424	0.3601	0
	0.0423	0.0349	0	0.0992	0.045	0.0701	0.0079	
0.0319	0.0336	0.113	0.0839	0.0481	0.0364	0.0791	0.0343	
0.1486	0.0157	0.0798	0.0478	0.0789	0.0089	0.2137	0.0121	
0.0682	0.018	0.0319	0.0355	0.0381	0.0366	0.0327	0	
0.0184	0	0.1091	0.0186	0.1519	0.0337	0.0412	0.0139	
0.0565	0.1707	0.0138	0.0104	0	0.0763	0.0522	0.0174	
0.0308	0.2552	2.00E-04	0.2823	0.0247	0.0118	0.136	0.0793	
0.0431	0.0195	0	0	0.0104	0.0955	0.263	0.0494	
0.0365	0.0417	0.0265	0.2686	0.0216	0.0433	0.0318	0.0846	

0.0547	0							
C7orf25	3.3908	2.7408	3.1011	4.1124	3.1723	2.3591	3.2978	
3.2896	3.2247	3.5035	3.2679	3.1251	3.5438	3.3337	3.0235	
2.9789	3.1174	3.6046	3.2956	2.9236	2.8637	3.6781	4.0284	
3.7072	3.32	3.9293	2.0135	2.7827	3.9356	3.1015	2.9044	
3.4325	3.1673	2.7535	3.8901	3.8086	3.7826	3.0882	3.2724	
3.9213	3.5318	3.7406	3.9647	2.8295	3.1475	3.8622	3.3647	
3.8344	3.411	3.3359	3.6461	4.6475	3.7878	2.7643	3.2832	
3.1011	3.0907	2.6313	2.8777	3.065	3.1851	3.1268	3.0069	
2.9786	3.1262	4.0701	3.1688	3.8985	3.0741	3.7498	2.7255	
3.7238	4.0039	3.8812	3.0545	3.1485	2.8509	4.1096	3.0669	
2.866	3.025	3.1432	3.7628	3.2525	3.5831	4.1817	3.5646	
3.3054	3.1396							
C7orf26	4.4052	3.7115	4.653	4.3173	4.0696	3.9547	3.8682	
4.4992	3.9583	3.9724	4.3198	2.8233	3.6705	3.7051	4.0142	
4.1302	4.0055	3.8804	4.0311	5.0085	3.9535	4.5287	4.4143	
4.5595	5.161	4.3378	3.7725	4.1131	4.8443	3.9317	3.6898	
4.1878	3.6665	3.7906	4.1847	3.6513	4.1894	3.8522	4.6598	
3.4515	4.2423	3.3557	3.9606	4.1113	3.743	4.2188	4.3964	
3.8256	5.1977	2.274	3.6968	4.1696	4.0796	4.4868	4.0618	
4.8281	3.0204	4.5808	3.5487	4.5732	4.0024	3.7509	4.4936	
3.5572	4.1803	3.3979	3.2395	4.2107	3.9176	4.477	3.5164	
4.0856	4.5422	3.588	4.2424	4.0448	3.6543	4.5547	3.6614	
3.7677	3.8349	3.6479	5.1301	4.2399	4.6339	4.2098	3.6924	
3.7234	3.8171							
C7orf31	2.3527	1.4505	1.4243	1.4376	0.8248	1.3208	1.4395	
2.3806	2.2855	1.5858	2.1418	0.7798	1.4904	1.8338	1.9243	
1.8435	1.8608	2.219	1.8786	1.6585	1.81	2.0186	2.2536	
1.5044	2.1763	2.1718	2.2764	1.8453	2.0432	1.1946	2.0458	
1.9791	1.5997	1.5493	2.1124	2.1221	1.8153	1.589	1.5604	
2.1165	1.3603	0.9579	1.8216	2.2925	1.2352	2.1477	2.1551	
2.1639	2.3981	1.176	1.7749	1.7049	2.5798	2.3164	2.1418	
1.8227	1.3047	1.7411	1.4052	2.0252	1.3294	1.2919	1.5117	
1.5295	1.3821	0.6969	2.3446	1.848	1.5934	2.3774	2.0868	
2.3725	2.0787	1.7	1.413	2.0701	1.6406	1.8986	1.8651	
1.6579	1.9212	2.1618	1.8815	1.9173	1.5523	2.4337	1.9033	
1.4314	1.6789							
C7orf33	0	0	0	0	0	0	0.0462	0
	0	0	0	0	0	0	0	0
	0	0	0	0	0	0	0	0
	0	0	0	0	0	0	0	
0.0226	0	0	0	0	0	0	0	0
	0	0	0	0	0	0	0	0
	0	0	0	0	0	0	0	0
	0	0	0	0	0	0	0	0
	0	0	0	0.0126	0	0	0	0
	0	0.0251	0	0	0.0224	0	0	0
	0	0	0	0	0	0	0	0
	0							
C7orf34	0	0.2659	0	0	0.0301	0	0	0

	0	0	0	0	0	0	0	0
	0	0	0	0	0	0	0	0
	0	0	0	0	0	0.0698	0	
1.2228	0	0	0	0	0	0	0	
0.0807	0	0	0	0	0	0	0	0
	0	0	0	0	0	0	0	0
	0	0.1458	0	0	0	0.1163	0	0
	0	0	0	0	0	0	0	0
	0	0	0	0	0.0911	0.4963	0	0
	0	0	0	0.1345	0	0	0	0
	0							
C7orf43	2.5537	3.7933	3.2006	3.2783	3.7207	3.7632	3.49	
3.7062	2.9127	2.9118	2.35	2.8926	3.157	3.2103	3.1156	
3.4022	2.6178	3.4783	3.3976	2.9677	3.0483	3.5245	3.5621	
3.8494	3.7567	3.1435	2.7726	2.7646	3.0556	2.5621	3.7663	
4.6946	3.1726	2.0902	3.8362	2.666	3.1244	3.5279	3.0417	
2.119	3.9747	3.7419	3.1017	3.286	2.587	2.6975	2.2941	
3.1071	3.403	1.7039	2.7168	3.6028	2.4783	2.4337	3.4565	
3.3489	1.5407	3.6155	3.1655	3.5593	3.3836	3.4417	3.67	
3.2911	2.7187	2.8092	3.8168	3.429	2.8974	3.54	3.106	
3.1345	3.7476	2.9736	2.9572	2.897	3.1295	3.8246	2.2303	
3.0512	2.8299	3.5727	2.0905	3.4511	3.281	2.6979	2.6791	
3.3276	3.1555							
C7orf49	4.6961	5.5307	4.7779	5.3107	5.0431	5.9236	5.464	
5.8038	4.5743	4.939	4.2918	4.4799	4.7663	5.0246	4.6371	
5.7781	4.1992	5.7781	5.2158	5.1769	4.5747	5.3214	5.4027	
5.0485	5.8028	4.91	2.4605	4.748	5.5177	4.8636	4.3684	
5.4427	4.5935	4.7172	5.7635	5.0412	5.35	4.8579	5.1782	
4.455	5.244	4.7551	4.8127	5.2089	4.6302	5.1629	4.2804	
5.1763	5.6961	3.9619	4.8426	5.7961	5.2407	5.3607	4.8098	
4.9991	2.9501	5.1546	5.3792	5.3542	5.1046	5.1478	5.8791	
5.3294	5.145	5.0091	4.449	3.9271	4.0713	5.6438	4.9346	
4.9924	5.418	4.8879	4.5971	4.7741	4.3396	5.4108	4.5844	
4.9463	4.6091	3.9674	4.5829	5.0238	5.1306	5.6484	4.5436	
4.4463	4.4088							
C7orf50	6.2019	4.8383	6.6882	4.2404	5.2272	3.954	3.5629	
6.648	4.3631	3.7193	5.3872	3.7196	5.7041	4.4053	5.7953	
5.0932	4.5428	3.7839	5.057	6.4322	5.6655	6.4119	5.77	
5.1467	6.5223	4.5144	3.9196	4.6702	5.4642	4.3292	6.544	
5.7664	4.8578	5.809	5.7449	3.7219	5.7718	6.1524	6.5166	
3.5925	6.1921	4.5341	5.2821	4.9556	4.4011	5.0807	4.3947	
4.6669	5.9358	3.5749	5.2201	4.1418	4.0871	4.4364	6.6	
4.9966	4.8912	5.8325	4.9657	5.8461	6.0383	5.0244	6.2287	
4.3811	5.1894	6.1263	3.2485	6.178	4.1236	6.1064	5.7944	
5.4413	6.5126	5.6732	5.1929	5.4226	5.2547	6.4604	5.3756	
5.9104	4.9696	4.5819	6.3242	6.6258	6.8515	6.0598	4.6639	
5.4115	4.9285							
C7orf57	0.481	0.1528	0.1169	0.2853	0.1235	0.0919	0.1665	
1.0882	0.7644	0.13	0.4164	0.2302	1.336	0.4036	0.5266	
0.2425	0	0.2139	0.2054	1.0194	0.3398	0.1873	0.7328	

0.3741	0.3391	0.175	0.2485	0.0695	1.8795	0.1006	0.2564	
0.2186	0.1085	0.4172	1.5339	0.1504	0.152	0.0815	0.118	
0.1212	0.116	0.0209	0.1826	0.4917	0.0287	0.8982	0	
0.0503	0.6514	0	0.8711	0.1436	0.5412	0.279	0	
0.2807	0.0084	0.0593	0.2057	0.3299	0.1321	0.433	0.2243	
0.3685	0	0.0079	0.5399	0	0	0.8193	0.3505	
0.7964	0.5189	0.1804	0.2725	0.3182	0.1809	0.1291	0.1794	
0.4333	0.0495	0.3083	0.7831	0.5353	1.5759	0.9684	0.3594	
0.6032	0.326							
C7orf61	0.3797	0.7526	0.828	0.2916	0.0961	0.6122	0.1128	
0.2081	0.4656	0.2344	0.4487	0.0366	0.5936	0.2595	0.2101	
0.0511	0.8836	0.425	0.3167	0.4943	0.4066	1.1514	0.0316	
0.2902	0.5748	0.2598	0.493	0.3781	0.2112	0.328	0.1277	
0.0834	0.4916	0.1457	0.3985	0.056	0.2759	0.2133	0.3064	
0.1547	0.4943	0.2083	0.282	0.501	0.194	1.002	0.1052	
0.2749	0.3348	0.885	0.7323	0	0	0.409	0.5644	
0.3986	0.6081	0.3274	0.3004	0.4611	0.0781	0.0396	0.1404	
0.879	0.7839	0.2856	0.7971	0.0189	0.033	0.1566	0.2315	
0.6689	0.594	0.0475	0.1404	0.0977	0.8056	0.0471	0.4694	
0.6874	0.5557	0.0603	0.191	0.0469	1.0081	0.2103	0.2156	
0.2197	0.1035							
C7orf62	0	0	0	0	0	0	0	0
	0	0	0	0	0	0	0	0
	0	0	0	0	0	0	0	0
	0	0	0	0	0	0	0	0
	0	0	0	0	0	0	0	0
	0	0	0	0	0	0	0	0
	0	0	0	0	0	0	0	0
	0	0	0	0	0	0	0	0
	0	0	0	0	0	0	0	0
	0	0	0	0	0	0	0	0
	0	0	0	0	0	0	0	0
	0							
C7orf72	0	0	0	0	0	0	0	0
	0	0	0	0	0	0	0	0
	0	0	0	0	0	0	0	0
	0	0	0	0.051	0	0	0	0
	0	0	0	0	0	0	0	0
	0	0	0	0	0	0	0	0
	0	0	0	0	0	0	0	0
	0	0	0	0	0	0	0	0
	0	0	0	0	0	0	0	0
	0	0	0	0	0	0	0	0
	0	0	0	0	0	0	0	0
	0							
C7orf73	4.2556	5.254	4.5004	5.4991	5.1836	6.0504	4.874	
5.7806	5.1451	5.1707	4.9672	4.8932	4.2933	5.4112	5.0138	
5.1859	3.6717	6.0315	5.6543	4.3138	3.886	5.571	5.6139	
5.1445	5.8697	4.5075	5.3334	4.8969	5.4931	5.439	6.0018	
5.4649	4.8921	4.9832	5.4433	5.3222	4.7808	4.2795	5.2047	

4.5859	5.5109	4.8384	5.3087	5.3987	5.1126	5.6147	4.6856	
5.5493	5.6673	4.5962	5.202	5.8635	5.5657	4.2581	5.175	
5.4824	3.911	5.3685	5.34	5.5468	4.8681	5.3373	5.5071	
5.7025	5.4654	4.7776	4.8937	4.0841	4.5168	5.7609	4.6055	
5.3586	5.486	5.2111	5.2607	5.1334	4.222	5.8818	4.9966	
4.7715	5.0516	5.1574	4.1585	5.7025	5.608	5.1595	4.4525	
3.9687	4.4623							
C7orf76	0	0	0	0.0636	0	0	0	0
	0	0	0	0	0	0	0	0
	0	0	0	0	0	0	0	0
	0	0	0	0	0	0	0	0
	0	0	0.0024	0	0	0	0	0
	0.0194	0	0	0	0	0	0	0
	0	0	0	0	0	0	0	
0.0696	0	0	0	0	0	0	0	0
	0	0.0079	0	0.0015	0	0	0	0
	0.0322	0	0	0	0	0	0	0
	0	0	0	0	0	0	0	0
	0							
C8A	0	0	0	0	0	0	0	0
	0	0	0	0	0	0	0	0
	0	0	0	0	0	0	0	0
	0	0	0	0	0	0	0	0
	0	0	0	0	0	0	0	0
	0	0	0	0	0	0	0	0
	0	0	0	0	0	0	0	0
	0	0	0	0	0	0	0	0
	0	7.00E-04	0	0	0	0	0	0
	0	0	0	0	0.0032	0	0	0
	0	0	0	0	0	0	0	0
	0							
C8B	0	0	0	0	0	0	0	0
	0	0	0	0	0	0	0	0
	0	0	0	0	0	0	0	0
	0	0	0	0	0	0	0	0
	0	0	0	0	0	0	0	0
	0	0.0045	0	0	0	0	0	0
	0	0	0	0	0	0	0	0
	0	0	0	0	0	0	0	0
	0	0.0184	0	0	0	0	0	0
	0	0	0	0	0	0	0	0
	0	0	0	0	0	0	0	0
	0							
C8G	0.657	0	0.5814	0	0.0761	0	0	
0.125	0	0.4243	0.1633	0	0.1042	0	0.1204	
0.1984	0	0	0	0.4974	0.7016	0	0.0769	
0.2649	0.0937	0.4689	0	0.2207	0	0	0.4911	
0.3623	0.1472	0.2798	0	0.1197	0.1655	0.1533	0.1161	0
	0.6666	0.1525	0	0	0	0	0	
0.5011	0.4181	0	0.135	0.1535	0.2567	0.1059	0.1056	

0.248	0.283	0.1837	0.1331	0	0.2189	0.023	0.4456	
0.2671	0.1164	0.0874	1.0758	0.5491	0.1336	0.0777	0.0265	
0.1476	0.1048	0.0488	0	0.4358	0.5442	0.2471	0.1563	
0.1826	0.0899	0.2312	0.4819	0.0338	0	0.3513	0	0
	0.2961							
C8orf22	0	0	0	0	0.0893	0	0	0
	0	0	0	0	0	0.0865	0	0
	0	0	0	0	0	0.1435	0.0423	0
	0	0	0	0	0.0343	0	0	0
	0	0	0.0634	0	0.0237	0	0	0
	0	0.2397	0	0	0	0	0	0
	0	0	0	0	0	0	0	0
	0	0	0.0424	0	0	0	0	0
	0	0	0	0	0	0	0	0
	0	0.183	0	0	0	0	0	0
	0	0	0.1851	0	0	0	0	0
	0							
C8orf33	4.6112	4.2395	4.3469	4.6584	4.1092	3.7287	3.2379	
4.2986	3.4308	5.4364	4.4817	4.0362	3.689	4.5474	5.0115	
3.2658	5.2471	4.7032	4.168	4.349	3.8241	4.8103	3.7049	
3.7843	4.9137	4.0105	5.4101	4.3934	4.4213	3.8655	4.6296	
4.5701	4.4033	4.3056	4.6374	5.1691	4.0802	5.6122	4.9969	
5.0391	3.5494	3.7202	4.6102	4.1262	3.8957	4.1567	5.1789	
4.296	3.821	4.9688	4.3242	4.2303	4.6704	4.5593	5.2124	
4.9519	4.3342	5.5041	4.329	5.4319	4.4063	4.4218	4.7847	
4.0812	5.7035	3.1887	3.9788	4.8066	4.5289	4.901	4.1827	
3.8367	4.4063	3.8386	3.6172	4.3899	4.408	4.2562	4.7701	
4.7103	3.9456	4.1548	3.3328	4.3464	3.7755	4.1327	4.1936	
4.5828	4.8944							
C8orf34	0	0	0	0	0	0.1243	0.0039	
0.0064	0	0	0	0	0.0639	0	0.0026	0
	0.4025	0	0	0	0	0	0.001	0
	0.002	1.2416	0	0	0.0228	0.0832	0.0031	0
	0.0328	0	0.3885	0	0	0	0.0043	
0.0299	0	0	0	0.0301	0.0133	0.43	0	0
	0.0404	0	0	0	0	0	0	0
	0	0	0	0	0	0.0365	0	
0.0927	0	0.0973	0	0.133	0	0.2617	0.0397	0
	0	0	0.1055	0	0	0.085	0	0
	0	0.0862	0	0.0489	0.0674	0	0.0196	0
	0							
C8orf37	1.0496	0.6875	1.3154	1.5469	1.0938	1.9017	1.0798	
1.2649	0.8161	1.127	1.3085	1.5794	1.0279	1.4431	1.1809	
0.6975	2.0912	1.1032	1.318	1.1441	1.2371	0.6648	0.9501	
0.9596	0.9924	0.9208	0.8122	1.6838	0.8224	1.0522	2.8623	
1.4369	1.0827	1.2789	0.7486	1.4964	1.2319	1.7585	0.5569	
1.4792	1.0361	1.5856	1.3826	1.517	1.4907	2.0813	1.5453	
1.427	0.9649	2.4528	1.1356	1.1893	1.4861	1.3432	2.1721	
1.3441	1.8099	0.8915	1.1781	0.7428	0.8303	0.9132	0.9253	
1.2256	2.053	0.9405	1.5443	0.5437	0.6445	2.0023	1.0612	

1.0015	1.1434	1.0494	1.2052	0.6248	1.1288	0.831	1.4773	
1.4277	1.6124	0.7986	0.5501	0.7725	0.9324	1.1998	0.8286	
0.7901	0.9299							
C8orf4	0.0899	0.0408	0.9693	0.0485	0.0612	0	0	
0.1091	0	0.0539	0	4.4136	2.0263	0	4.2945	0
	0.1392	0.0702	0	0.0545	0	2.6201	0	
0.0492	0.106	0.5592	0.0849	0	0	0	0.0664	
0.0305	0.0429	0.1177	0.3336	0.0291	0.1528	0.0727	0.0438	
0.2892	0.0541	3.3186	0.4083	0.0397	0.0546	0.1357	0.2699	
0.0415	0.0596	0.2913	0.0534	3.1208	0.0583	0	1.8409	
0.2032	1.1055	2.356	0	3.8854	0.0492	0.291	2.2793	
0.1169	0.1948	0.2891	0.0859	0	0.0087	0	0.0742	
0.0335	0.068	0.1185	0	0	0.0927	0	0.0279	0
	0.0202	0	0	1.4741	0.886	0	0	
0.0547	0.091							
C8orf44	0.9006	0.7101	0.918	1.1481	0.6705	1.1205	0.7196	
0.8117	0.6422	0.5223	0.6872	0.884	0.7539	0.9516	0.9319	
0.5582	1.5866	0.9906	0.9663	1.133	1.0722	0.3682	0.6751	
0.7633	0.5478	0.7976	1.109	0.9669	0.6663	0.5618	0.8533	
1.3332	0.7638	0.7302	1.0413	0.5373	0.8721	1.1216	0.7587	
1.0379	0.5357	0.8721	0.7807	0.7874	0.7892	1.029	1.0384	
0.4971	0.765	1.8376	0.6852	0.9127	0.8989	0.6701	0.7685	
1.08	1.4341	0.864	0.5444	0.5133	0.7152	0.9134	0.786	
0.536	1.1735	1.0341	0.7212	0.9805	0.3647	0.8063	0.4751	
0.9061	0.7235	0.8017	0.7121	1.0912	1.1826	1.1011	1.1408	
1.0666	0.9742	0.7114	0.5704	1.0595	0.7559	0.794	0.8409	
0.4935	0.8754							
C8orf46	1.4194	1.3556	1.3163	1.0693	0.2601	4.0631	1.0369	
1.1774	1.8309	1.4036	1.5253	2.3449	0.9028	2.1932	2.13	
2.4309	0.3857	1.3516	2.5646	1.0313	0.2902	1.1408	0.9982	
1.8641	0.5261	1.02	0.0849	0.2851	1.1206	0.5879	0	
1.9598	3.2397	3.4133	3.1296	1.6836	0.6071	2.1227	1.1396	
1.8833	0.3915	0.0824	0.8425	2.8884	0.0128	0.3834	1.1353	
1.0572	0.6209	0.9713	2.0735	0.3824	2.1324	0.4245	0.3227	
2.0043	0.3367	0	2.2394	0	1.5479	2.0315	1.0798	
2.8534	3.8281	0.357	1.1097	0.2834	0.1371	0.3156	2.7718	
1.85	1.9306	1.0819	1.9234	1.3445	1.6868	0.9469	1.9408	
1.806	0.3579	1.2794	0.746	1.3596	0.2268	1.9745	0.9713	
1.6088	1.5981							
C8orf48	2.2583	1.0643	1.4264	1.5045	0.7661	1.0992	2.8836	0
	1.4736	0.4024	0	0.3207	2.3081	1.9378	1.9518	
1.2544	0.0746	1.5263	0.9088	0	1.0674	1.8897	2.4627	
0.8133	2.871	2.085	0.4435	0	1.8655	0.2922	0.1588	
2.4739	1.8018	2.7224	2.0068	0.5135	1.2949	1.1031	0.6381	
3.221	1.5045	0.3921	0.062	0.6607	0.6952	1.3979	1.908	
1.3936	2.1176	1.5721	1.9144	1.7562	2.037	1.4	0.0442	
1.8309	0	0.9614	1.696	1.5213	1.5666	1.4022	3.204	
1.5085	2.2448	0.9428	2.1337	0	0.0468	2.3425	2.0412	
1.5768	1.1305	1.0744	0.9527	2.2981	1.6581	1.9662	0.1363	
0.665	0	1.0964	1.958	1.4	0.9421	2.0607	1.7484	

1.6385	1.4592							
C8orf58	3.6701	2.761	2.3297	3.0311	3.675	3.0274	2.5211	
3.1263	2.4992	2.7133	2.5441	2.4176	3.1645	2.9849	2.1038	
4.1787	2.6273	3.4076	2.1043	2.8713	3.8654	2.988	3.5609	
2.5677	2.4368	3.5865	2.4704	2.0239	3.1039	2.96	2.7766	
3.1751	2.5266	2.6421	2.3169	2.5054	3.2287	2.2739	3.4094	
2.1939	2.6191	1.9195	2.7443	2.7457	3.2571	2.7023	2.0563	
2.9175	2.979	3.7019	2.8693	2.7697	3.1211	1.6227	2.8668	
2.7207	1.4196	2.3356	3.2772	2.9402	3.3597	3.3435	2.6109	
2.0838	3.0235	2.508	2.7745	3.4206	1.6852	3.4957	2.9685	
2.9457	2.886	3.387	3.0857	2.1892	3.0493	3.0879	2.3874	
3.2758	2.78	3.2291	4.0586	2.6839	3.3755	2.9645	3.0494	
2.9567	2.8764							
C8orf59	5.4672	4.9571	5.1278	5.5995	5.4287	4.6618	5.1976	
4.8245	4.489	4.8893	5.718	5.6259	5.2547	5.1516	6.0066	
4.1169	5.9033	5.2347	5.0421	4.6544	5.432	5.0848	5.0643	
4.6592	5.2872	4.5639	6.2561	4.9737	4.8736	5.296	5.8142	
5.1584	5.0961	4.9459	4.8848	5.6825	5.347	5.8599	5.2684	
5.3949	4.6881	5.8988	5.3463	4.7252	5.3925	6.1729	5.8692	
4.9044	4.5716	5.7635	4.7571	5.4132	5.2741	5.163	5.8145	
5.1081	5.6282	5.64	5.6479	5.6009	4.6206	4.9243	5.1726	
4.7321	6.3151	4.2317	5.4741	5.0075	4.3257	5.2079	4.7362	
4.6135	5.2283	5.3895	4.2475	5.0786	5.2928	5.119	5.5864	
5.363	5.6981	5.0059	4.4259	4.8161	5.2785	5.4791	4.7157	
4.9002	4.7753							
C8orf74	0	0	0	0	0	0	0	0
	0	0	0	0	0	0	0	0
	0	0	0	0	0	0	0	0
	0	0	0	0	0	0	0	0
	0	0	0	0	0	0	0	0
	0	0	0	0.0622	0	0	0	0
	0	0	0	0	0	0	0	0
	0	0	0	0	0	0	0	0
	0	0	0	0	0	0	0	0
	0	0	0	0	0	0	0	0
	0	0	0	0	0	0	0	0
	0							
C8orf76	3.6364	3.6064	3.8612	5.0536	4.1195	3.9603	3.9223	
3.4362	2.9458	3.41	3.6122	3.9877	4.2978	3.4168	4.3255	
2.9872	3.9873	3.9119	3.6345	3.9069	3.4955	4.0156	3.5389	
3.9142	4.0786	3.8624	3.1313	3.6923	3.8149	3.9223	3.813	
3.6958	3.8455	3.6591	3.5952	4.0914	3.6971	4.4108	3.6771	
3.4912	3.2227	4.3521	3.8844	3.0248	3.9082	4.1578	4.5503	
3.1401	2.7835	3.8956	3.8126	4.2085	3.412	3.7386	3.8098	
3.7079	3.8921	4.5586	3.847	4.3972	3.9428	3.6373	4.0805	
2.84	4.3556	2.9256	3.467	3.7575	2.6865	4.134	3.8641	
2.814	3.2384	3.4991	3.4844	3.7426	3.545	3.7146	3.5924	
3.959	3.4569	4.2468	3.2292	3.4958	3.6948	3.4914	3.3573	
3.6613	3.4638							
C8orf82	3.4023	3.4364	2.8202	3.8705	3.2504	4.1679	2.5093	

3.6985	2.4409	3.8236	3.3728	3.302	3.3406	2.9613	4.1972	
2.449	4.5513	2.606	3.4372	3.6919	3.6601	3.4659	3.9579	
2.9215	4.0222	3.4639	3.9734	3.2321	3.591	3.0016	4.1764	
2.3944	3.6578	2.4666	3.3259	3.8513	3.4674	5.7722	3.9876	
3.2756	3.8105	3.9832	3.899	3.11	3.2073	3.5635	2.9141	
2.6636	3.7034	3.5211	3.1698	3.7166	3.8759	3.4802	4.31	
3.7213	3.0622	4.5535	3.6773	4.0383	3.3921	3.5675	3.4599	
2.8934	5.0554	2.9931	3.227	3.5977	2.1843	3.1161	4.2758	
3.0576	3.7866	2.7286	2.6604	3.5761	3.1037	3.3506	4.31	
4.3451	3.6505	4.2836	3.4294	3.2023	4.5466	4.1587	3.9163	
3.8347	3.7372							
C8orf86	0	0	0.0353	0	0	0.0304	0.0083	0
	0.0267	0.0308	0.0171	0	0.0091	0	0.0356	0
	0.151	0.0498	0	0.0338	0.0895	0.0141	0	
0.0361	0	0.0114	0.0186	0.0374	0	0	0	
0.0076	0	0.0149	0.0409	0.01	0	0.071	0	
0.0136	0.045	0	0.0501	0	0.0077	0	0.0264	
0.0426	0.0137	0.0536	0	0	0	0.084	0.0617	
0.0659	0	0	0	0	0	0	0.0203	0
	0	0	0.0488	0.0042	0.0143	0	0.0431	
0.0209	0.0846	0.0024	0	0.0297	0.029	0.0241	0	
0.0455	0	0	0	0	0.0389	0	0	
0.0561	0.0628							
C8orf88	3.1454	1.9708	0.6532	3.9767	3.3291	1.1666	3.2072	
2.6767	2.7025	3.5926	3.1337	3.723	3.6667	3.5622	3.622	
0.4046	4.3495	2.4571	3.0831	3.4233	3.0817	1.5888	3.2028	
0.4126	3.3758	2.4104	4.6319	3.3666	2.9004	3.1648	4.1683	
3.3152	3.9258	3.73	1.3953	4.1053	4.3297	2.1971	2.1172	
0.9516	3.4966	4.4419	4.3166	3.1186	3.4191	4.5643	4.4043	
3.0026	2.6432	4.5153	3.2228	3.4496	3.2537	2.812	4.6819	
0.7399	4.083	4.3641	3.624	4.4179	3.3905	2.8793	1.5331	
1.9934	3.0013	2.1802	3.7027	2.5014	2.088	2.1162	3.5327	
3.2369	1.3043	2.9921	3.1105	3.3987	3.6028	1.6609	3.7592	
3.8021	4.1695	3.9321	1.5677	2.2501	4.0803	3.5658	2.8432	
3.4276	3.1108							
C9	0	0.0212	0	0.0522	0	0	0.0211	0
	0	0	0.0157	0	0.0417	0.0354	0	0
	0.072	0	0	0	0.0706	0.0034	0.005	0
	0	0.0661	0	0	0.018	0.0182	0.0111	0
	0	0	0	0	0	0.0296	0.0093	0
	0.0507	0.0021	0	0.0203	0	0	0	0
	0	0	0	0	0.0437	0.0142	0	
0.065	0	0	0	0	0	0	0	
0.0417	0.0185	0.136	0	0.0076	0	0	0.0356	0
	0.0447	0	0	0.036	0.0614	0.0125	0.0384	
0.0243	0	0	0.0083	0	0	0	0.0965	0
	0.0707							
C9orf116	1.9408	2.7518	1.2641	2.4284	2.0389	1.5643	1.9457	
2.8187	0.6083	1.4816	2.635	1.4044	2.2107	1.4664	1.0132	
1.004	1.5602	1.4223	1.0526	1.3824	2.3351	0.6504	1.9283	

0.791	1.9758	1.1359	2.7207	2.2924	1.3602	1.6391	2.6636	
2.4501	1.0021	1.0996	0.7648	2.1456	2.3424	2.3696	1.1698	
2.1095	3.163	2.6662	1.3744	1.3295	2.341	1.7669	1.6672	
1.3696	0.9583	2.2933	1.2017	1.2093	1.9478	1.2343	2.7299	
1.3469	1.5247	2.6468	1.3327	2.9369	1.066	1.9494	1.3969	
1.7129	1.9688	2.3319	2.1497	2.7364	1.1922	2.5152	2.3957	
1.0154	2.4751	1.9068	1.0948	1.0989	1.21	1.892	1.0683	
1.4262	1.5441	0.9132	1.6578	2.2523	0.5951	2.1138	2.076	
2.1337	1.7504							
C9orf129	0.1398	0.4428	0.3925	0.4509	0.0907	0	0.2067	
0.1258	0.1275	0.4709	0.5447	0	0.217	0.182	0.7622	
0.538	0.1431	0.2345	0	0.1412	0.3206	0	0.0285	
0.0745	0.1157	0	0	0.3209	0	0	0.3336	
0.1126	0.0778	0	0	0.5185	0.1322	0.6381	0.0564	0
	0.2946	0.0561	0	0.2223	0.0558	0	0.1223	0
	0.0544	0	0.0689	0	0	0.1658	0.5253	
0.5157	0.2381	0.0939	0.5218	0.049	0.0266	0.0379	0	
0.2332	0.7266	0.0898	0.7795	0.1007	0.0661	0.2189	0.0791	
0.2309	0.1518	0.0288	0.0369	0.125	0	0.0895	0	
0.0847	0.092	0.0593	0.1131	0.0951	0	0.3073	0	
0.3748	0.1036							
C9orf131	0.0304	0.03	0.0256	0.0658	2.00E-04	0.0409	0.1008	
0.0848	0.1224	0.0582	0	0.014	0	0.0523	0.0917	
0.0327	0.2869	0	0	0	0	0	0	
0.0247	0.0264	0	0.0437	0	0.0102	0	0	
0.011	0.042	0	0.0521	0	6.00E-04	0.0338	0.0361	
0.0987	0.0186	0.002	0.0374	0.0392	0.1	0.0474	0.0217	
0.0552	0.0181	0.046	0.009	0.05	0.0753	0.036	0	
0.0489	0.0832	0.0236	0.0218	0.0208	0.0158	0.0248	0.0318	
0.0265	0.0268	0.0424	0	0.0611	0	0.043	0.0257	
0.0147	0.0201	0.0051	0.0157	0	0.0176	0.0184	0.0084	0
	0.0307	0.0108	0.149	0.036	0	0	0.0348	0
	0.0287							
C9orf135	0	0	0	0	0.0444	0	0	
0.1431	0	0	0	0	0	0	0.1384	
0.0628	0	0	0	0	0	0	0.4553	
0.376	0.0485	0	0	0	0	0	0.1795	
0.2673	0.0933	0.0987	0.2114	0	0	0	0	0
	0	0.0377	0	0.1116	0.0659	0	0	0
	0.0885	0	0	0	0	0	0	0
	0	0	0.1204	0	0.3907	0.0524	0	0
	0	0	0	0	0	0.3226	0	0
	0	0.0397	0.1278	0	0	0.4427	0	0
	0	0.1072	0	0	0.3262	0	0	0
	0							
C9orf142	4.147	3.9216	4.3449	2.8162	4.7304	3.0233	2.3036	
5.1507	2.8054	3.1373	4.3047	2.8429	5.0223	2.4559	3.8109	
2.7208	4.1445	2.54	3.7514	4.4912	4.5294	3.5183	3.2058	
3.7525	3.8202	3.1025	3.3922	4.0667	3.7399	3.3599	3.5977	
4.4807	3.4691	3.4419	3.3243	2.4652	4.1943	4.2363	4.5757	

2.271	4.3944	3.558	3.7847	3.5117	2.8141	3.2201	2.8571	
3.1227	4.5303	3.119	3.1661	2.9671	3.1036	2.9575	4.1592	
3.9699	3.9001	5.114	3.8491	4.6499	4.4036	3.945	4.0782	
3.4103	4.1781	3.42	3.8296	3.3822	3.8171	4.7957	3.1522	
3.4353	3.3906	3.2017	2.2978	3.9327	3.8171	2.0799	3.577	
3.8196	3.9262	2.6469	3.0819	4.297	3.8562	4.5366	2.8157	
4.2284	3.6482							
C9orf152	0.0784	0.095	0.0716	0	0.006	0.058	0.0107	
0.0758	0.043	0.0496	0.0658	0.0751	0.0543	0.0384	0.009	
0.053	0.5454	0.0537	0.0518	0.1668	0.034	0.0163	0.0444	0
	0.0214	0.0573	0.3569	0	0.0173	0.0197	0.0121	
0.003	0	0.0552	0	0.0273	0.0716	0	0.0782	
0.1028	0.0161	0.0121	0.0299	0.0418	0.1421	0.1149	0.0742	
0.0455	0.0339	0.1874	0.0947	0.0709	0.1768	0.0557	0	0
	0.2248	0.0286	0.1336	0	0.1	0.0091	0.016	
0.1265	0.0344	0.1009	0.0469	0.0998	0.0849	0.0986	0.0124	
0.1915	0	0	0.086	0	0.025	0.0199	0.0926	
0.0454	0.0154	0.1082	0.2755	0.0529	0	0.1748	0.0702	
0.0994	0.0628							
C9orf153	0	0.0224	0	0	0.0296	0.0334	0.0568	0
	0.0997	0	0	0.0039	0	0.0517	0.006	0
	0	0	0.0798	0.1474	0.0442	0.0147	0.0046	
0.021	0.0354	0.0271	0.0183	0.116	0	0.018	0.0121	
0.0417	0.052	0.0163	4.00E-04	0	0	0.0897	0.0413	
0.0431	0	0.01	0	0.0223	0.0071	0.0335	0	
0.0259	0.0458	0.0619	0	0.0396	0.0446	0.0106	0.1428	0
	0.0381	0.0754	0.0984	0.0557	0	0	0	
0.1282	0.0289	0.0051	0	0	0.0599	0.0783	0	0
	0	0	0.0602	0	0	0.0668	0	0
	0	0	0	0.0481	0	0.0378	0.0321	
0.0399	0.0479							
C9orf16	4.3424	3.7749	4.1099	2.6967	5.5217	2.8508	2.3275	
4.2225	2.7832	2.7472	4.8373	3.5882	4.3077	2.2886	4.3713	
3.2793	3.6175	3.2184	2.6159	4.1363	4.2304	4.3324	4.7608	
3.1872	3.875	3.1239	5.5408	4.2918	3.6002	3.798	4.7548	
5.1559	3.034	4.3763	3.4891	2.4651	4.9137	6.0222	5.6254	
2.1342	5.1823	4.507	4.0994	3.5426	2.8345	2.8034	2.6059	
3.5457	6.3423	3.3598	3.3842	5.1082	2.9024	4.1164	4.4102	
2.9977	4.4672	3.9714	2.9752	3.6896	4.3791	3.2472	4.3492	
2.9574	4.6173	4.7378	3.7213	5.4551	3.5919	5.2696	3.5905	
3.5459	3.6412	5.8028	2.7563	3.928	3.9958	3.9903	4.2658	
3.5746	3.8541	4.2274	4.7501	6.1993	4.5075	3.7357	4.3387	
5.0146	4.5923							
C9orf172	0.8578	0.4619	1.2767	0	0.8357	0.4187	0	
0.5966	0.3136	0.3218	0.448	0.3234	0.9123	0	0	
0.6683	0.6985	0	0	1.2697	0.9657	0.2832	0.3789	
0.5944	0	0.3646	0.5352	1.1838	0	0	0.3922	
1.3322	0.4101	1.3969	0.3136	0	0.5137	1.2119	0.6929	0
	0.9353	0.8709	0.8517	0.7103	0.3134	0.7562	0	
0.509	0.5923	0	0.5542	0	0	0.2212	0.7305	

1.4346	0.5713	0.8631	0.1834	0.9656	1.0032	0.7593	1.5271	
0.8166	1.3872	0.7134	0.8447	1.6251	0.3581	1.0594	0.6546	
0.4348	0.3344	0.726	0.0345	1.2477	1.2173	0.8043	0.4873	
0.321	0.548	1.0748	0.5574	1.0261	0.9977	0.3817	0.3339	
0.3875	0.484							
C9orf24	0.3224	0.059	0.0474	0	0.0409	0.0885	0.7179	
0.0822	0.1446	0.2364	0	0.0176	0.0887	0.1335	0.135	
0.0749	0.2185	0.0439	0.1544	0.1019	0	0.099	0.1531	
0.0506	0.0229	0	0	0.0977	0.0398	0.5527	0.155	
0.0357	0	0	0.1711	0.0985	0.2013	0	0.0936	
0.0597	0.1404	0.0295	0.225	0.0613	0.1438	0.0524	0	0
	0.1382	0	0.2024	0	0	0.0478	0.5299	
0.1337	0	0	0.071	0.4166	0	0.102	0	
0.1678	0.075	0.4916	0.092	0.0908	0.0468	0.0642	0	0
	0.0377	0.4455	0.0518	0	0	0.0602	0.0748	0
	0.0577	0	0.0921	0	0	0.1278	0.0346	0
	0.2057							
C9orf3	1.4495	1.7353	2.4719	3.6552	3.0373	2.3242	1.9709	
2.1691	1.1798	1.9951	2.0866	2.6734	5.5837	1.6267	3.8237	
2.0425	1.7765	2.7275	1.913	1.4146	0.8913	1.666	1.7146	
3.2497	2.913	2.3654	1.8444	1.6648	3.2685	2.1574	1.5566	
1.4854	2.0022	1.4802	2.5193	2.1379	1.5925	1.53	3.2242	
3.5096	1.7428	3.8893	2.9418	3.1186	2.6251	3.6221	2.7653	
1.5672	1.7653	2.0414	1.3979	3.2189	1.4121	1.3507	3.2047	
2.8771	2.0685	1.4343	4.1536	1.8154	3.5786	1.964	1.5683	
1.5465	1.398	4.836	1.0427	0.8669	3.4341	2.7847	1.5334	
1.1478	1.293	2.0607	1.6959	2.1454	1.7195	2.2079	1.2571	
2.2484	1.9071	1.8786	1.9752	2.8232	2.7343	1.5659	1.5466	
1.7287	1.1811							
C9orf40	2.6801	2.8051	2.8284	3.7436	3.099	2.4535	2.3335	
2.1828	2.1847	2.5999	2.9345	3.0721	3.5694	2.5777	2.4859	
2.3419	3.2695	3.029	3.7371	3.6343	2.6822	2.276	1.925	
3.2003	1.6888	2.1792	2.2514	3.2244	2.5526	2.7268	3.7018	
3.6104	2.8362	3.0915	2.4412	3.486	3.198	2.3687	2.6958	
3.3962	2.3955	2.943	2.9947	3.196	3.3316	4.0154	4.0107	
2.1172	1.69	3.3998	2.3809	2.6316	3.1153	2.1174	4.0347	
3.4687	3.4035	3.623	3.1417	3.3791	3.2008	2.4882	2.6988	
3.1246	4.133	2.0929	2.2523	2.6234	2.195	3.1891	2.4733	
2.2045	2.7501	2.5284	2.489	2.9824	2.5307	2.3248	2.3091	
2.1853	3.0693	1.7398	2.3974	2.9545	2.4217	3.4588	2.6974	
3.1831	3.2354							
C9orf43	0.4208	0.338	0.4273	0.4228	0.1328	0.4701	0.3566	
0.5274	0.3707	0.3911	0.5812	0.1276	0.8291	0.5672	0.2111	
0.1477	0.6604	0.6184	1.0535	0.5963	0.4289	0.1551	0.1204	
0.6483	0.0422	0.0878	0.5182	0.631	0.3707	0.2002	0.7195	
0.3396	0.9885	0.5403	0.0699	0.4289	0.2588	0.1913	0.4008	
0.5696	0.3258	0.0747	0.39	0.4559	0.2032	0.1783	1.1273	
0.4134	0.1349	0.985	0.0997	0.1858	0.1781	0.6515	0.7223	
0.9182	0.5258	0.3001	0.6746	0.2219	0.1932	0.2946	0.1683	
0.2696	0.6027	0.504	0.5897	0.4667	0.8698	0.565	0.2286	

0.5557	0.894	0.0187	0.2539	0.2513	0.7252	0.1573	0.0514	
0.6585	0.1921	0.1343	0.7312	0.4828	0.1526	0.1531	1.0349	
1.0485	0.9495							
C9orf47	0	0.0723	0.0234	0	0.002	0	0.1005	0
	0.079	0	0.0668	0.0284	0.227	0	0.0232	
0.0104	0.0405	0	0	0	0.0708	0.0043	0.0275	0
	0.0824	0.2995	0.0195	0	0.0436	0	0.0462	0
	0.0345	0.0394	0.0354	0.0339	0	0.0658	0.0121	
0.0327	0.0247	0.0629	0.0891	0	0	0.1933	0.0509	
0.026	0.1613	0	0	0.1432	0	0	0.094	0
	0.2164	0.0216	0	0.0335	0.0139	0	0.0259	
0.0473	0.1004	0.1114	0	0.0043	0.0077	0.067	0.0089	
0.1039	0.0313	0.0018	0	0.0349	0	0	0	0
	0.0588	0.0394	0.0369	0.1098	0.0295	0	0.0413	
0.0587	0.0388							
C9orf50	0.1199	0	0	0	0.0718	0.1158	0	
0.0488	0	0	0.0312	0	0.035	0	0.1581	
0.0362	0	0.3115	0	0	0.0746	0.1141	0.0782	0
	0.4655	0.043	0.4267	0.1085	0.1025	0	0.1021	
0.0557	0	0	0	0	0.3706	0.2513	0.0905	0
	0.3261	0.0131	0.0932	0.3007	0	0.0697	0	0
	0.0385	0	0.055	0.209	0.1424	0.0868	0	0
	0.0461	0.0472	0	0	0.0715	0	0.1868	0
	0	0.0408	0.1159	0	0	0.5581	0	0
	0.048	0	0.0848	0.0678	0	0.1317	0.3293	0
	0	0.1056	0.0365	0.5512	0	0	0.1424	
0.1329	0							
C9orf57	0	0.103	0	0	0	0	0	
0.0205	0	0	0	0	0	0	0	0
	0	0	0	0	0	0	0	0
	0	0	0	0	0	0	0	0
	0	0	0	0	0	0	0	0
	0	0.0057	0	0	0	0.024	0	0
	0.0258	0.0699	0	0	0	0	0	0
	0	0	0	0	0	0	0	0
	0	0	0	0	0	0	0.0517	0
	0	0	0	0	0	0	0	0
	0	0	0	0	0	0	0	0
	0							
C9orf64	1.9854	3.1733	2.2073	2.0771	1.8404	2.1786	2.8756	
3.6647	2.0402	3.0497	2.9784	3.2582	4.0336	3.5104	1.5297	
2.5139	3.2314	0.868	3.5888	4.2235	2.6286	2.7838	2.3473	
2.5254	2.6067	2.2785	0.3148	2.8654	2.0998	2.4997	4.0011	
3.468	2.33	3.2678	2.3137	3.2314	4.3471	1.1315	3.1943	
0.609	3.1748	3.8479	2.1693	3.64	2.8806	3.4913	3.814	
2.9803	2.5645	3.1315	3.4424	2.9927	3.7135	3.647	3.8042	0
	3.5065	3.6318	3.9494	3.5774	3.174	3.2089	1.5265	
1.2242	3.1245	2.7144	2.9049	2.9714	3.52	4.2529	1.8842	
2.6727	3.5069	3.1223	1.488	2.5232	2.7339	2.0314	0.8473	
2.1248	3.3253	2.8189	2.2951	3.0561	2.6367	3.6844	2.5948	

2.7626	2.4929							
C9orf66	0	0.0749	0.829	0	0.0817	0	0.0703	
0.0269	0.0701	0.0299	0.1259	0	0.0235	0	0.0424	
0.0447	0.2901	0.0833	0.0728	0.1541	0.1383	0.1342	0.0449	
0.0492	0.0378	0.0464	0.0597	0.0616	0.0307	0.0332	0.3234	
0.0369	0	0.0878	0.0573	0.0446	0.0772	0.1378	0	0
	0.0285	0.0115	0.0474	0.1506	0.021	0	0	
0.0743	0.0958	0.0821	0.0781	0	0.6225	0	0.3498	0
	0.2498	0.0106	0	0	0.049	0	0.1169	
0.0756	0.1079	0.044	0.1559	0.2146	0.1511	0.0616	0	
0.6687	0.2596	0	0.1455	0.2302	0.3858	0.0069	0.0685	
0.0232	0	0.11	0.0016	0.1361	0	0.4481	0.1363	
0.3365	0.4557							
C9orf69	3.6963	3.9854	4.0851	3.2164	4.6296	2.9682	2.8733	
4.5081	2.4917	3.3054	3.863	3.3357	3.3082	2.9533	4.0406	
2.5655	4.2578	3.1563	3.2981	4.1449	3.9578	3.2129	3.0361	
3.4623	3.7607	3.2574	2.7386	3.9696	3.9557	3.223	3.307	
4.6832	3.3739	3.2531	3.3101	2.8342	4.6872	4.7221	4.6717	
3.0911	3.7345	3.9984	3.9365	3.0377	3.4896	3.6061	3.4486	
3.7013	4.1437	3.953	3.6203	3.4773	3.4938	3.774	3.9055	
4.0078	4.7724	4.2839	3.4789	3.8772	4.1572	3.5228	3.6353	
3.6472	4.0578	3.8534	2.6137	3.7883	3.9986	4.9513	3.1247	
3.3443	3.3013	3.0581	2.1381	4.1994	3.0908	3.1854	3.2159	
3.4487	3.0593	3.4411	4.345	4.3518	3.9282	4.1874	4.0125	
3.949	3.9971							
C9orf72	2.1994	2.0873	1.6667	2.0521	3.0178	1.9168	3.1871	
2.5617	0	2.3243	2.1573	2.288	1.9286	3.1143	1.3402	
2.6373	3.3727	2.425	2.0357	1.6951	1.5599	2.1383	1.8558	
2.3499	2.0552	0.9664	3.3109	2.8586	1.7606	2.0571	2.339	
2.2994	1.508	2.5313	2.2871	2.2418	2.2452	2.2369	0.7566	
2.5134	2.1691	1.3425	2.2999	2.3276	2.1049	1.6594	1.7913	
2.3935	2.9069	2.3921	2.4241	2.5134	2.4947	1.3895	2.5551	
2.1039	2.5491	4.2078	2.7086	3.5719	1.4264	3.3604	2.1896	
1.9428	2.1167	2.3472	2.377	1.4514	1.8706	2.0666	1.802	
2.1519	2.3227	2.0157	1.8901	2.0108	1.693	2.0573	2.6647	
2.4337	2.2047	1.4558	1.487	1.9587	1.7549	2.2719	2.311	
2.2312	2.3057							
C9orf78	4.2924	4.8536	4.5444	4.9085	5.1701	5.0963	4.6336	
4.6817	4.2077	4.7063	5.3464	4.8699	5.4226	4.9833	4.8728	
4.5949	4.8075	4.8647	4.5588	4.5975	4.9434	4.4845	4.3029	
4.7644	4.8687	4.3579	5.4281	4.5554	4.8959	4.6515	4.9801	
4.5752	4.4282	4.7718	4.0663	4.7679	5.0169	4.5191	5.2899	
4.5474	4.5134	4.7812	4.3246	5.0094	4.2389	5.014	4.7131	
4.4919	4.5993	5.4594	4.4594	5.2634	4.7444	4.9135	4.9531	
4.7343	5.0452	4.809	4.2466	4.7575	4.9486	4.3718	4.33	
4.8084	5.0485	4.8286	3.9463	5.3325	5.4965	5.1951	4.461	
4.441	5.0112	4.8632	4.7852	3.944	4.5425	4.5514	4.6766	
4.5157	4.6033	5.0041	4.6716	5.0752	4.5069	4.8203	4.8991	
5.092	4.6736							
C9orf84	0.0249	0	0.2256	0.0892	0	0	0.4639	

0.0102	0.0041	0.0143	0.4549	0.0514	0.0705	0.1655	0.0035	
0.0107	0.0805	0.0692	0.0689	0	0.0872	0.2356	0	
0.1923	0	0.1915	0.0319	0.6219	0	0.0466	0.0118	
0.0061	0.0473	0.0357	0.0026	0.0162	0.3899	0	0.0086	
0.0238	0.0114	0.0219	0.0365	0.0652	0.0761	0.0909	0.024	
0.0234	0.0414	0.1333	0.0142	0.0509	0.0663	0.0497	0.0297	
0.0648	0	0.0193	0.0499	0.1495	0	0.0515	0.0288	
0.043	0	0.0309	0.0996	0.0243	0.1213	0	0.282	
0.0284	0.0528	0	0	0.6866	0.4056	0	0.0067	
0.0726	0.0192	0.0456	0.0474	0.0921	0.0238	0.0908	0.0344	
0.0201	0.0554							
C9orf85	2.987	3.3545	2.3151	3.1913	3.327	2.9567	2.4178	
3.5804	2.7856	2.4049	3.7666	3.0578	3.6463	2.9738	2.3276	
2.3179	3.1382	2.7876	3.3537	2.9428	2.3673	2.339	2.3699	
2.8454	2.9601	1.9577	4.3366	2.5509	2.2119	3.0106	2.5282	
3.2462	2.5286	3.2961	2.0947	3.0396	3.2034	3.146	3.6222	
3.2215	3.48	3.0036	2.5126	3.5885	2.5803	3.0666	3.4416	
2.2645	2.761	3.1126	2.6841	2.6746	3.6913	3.4067	2.816	
2.704	3.2801	2.9514	3.8917	2.8867	3.2933	2.7691	2.8969	
2.5292	3.8057	2.6676	2.7573	2.7499	2.3566	3.7453	3.4163	
2.1194	3.1638	3.1896	2.687	2.0671	2.2165	2.9785	2.7159	
3.4015	3.0706	3.3676	2.6947	3.2798	2.2958	3.9808	3.2238	
2.6975	2.9818							
C9orf92	0	0	0	0	0	0.2103	0.1031	0
	0	0	0.0742	0	0.0938	0	0	0
	0	0	0	0	0	0	0	0
	0	0.1366	0.1212	0	0	0	0	0
	0	0	0	0	3.9886	0.1601	0	0
	0	0.0499	0	0	0.086	0	0	0
	0	0	0	0	0	0	0	0
	1.1805	0	0	0	0	0	0	0
	0	0	0	0	0	0	0	0
	0	0	0.3891	0	0	0	0	0
	0	0	0	0	0	0	0	0
	0							
CA1	0	0	0	0	0	0.051	0.0211	0
	0.0252	0	0	0	0	0	0	0
	0.1507	0	0	0	0	0	0	0
	0	0	0	0	0	0	0	
0.0082	0	0	0	0	0.0183	0	0	0
	0	0.0263	0	0	0	0	0.0426	0
	0	0	0	0	0	0	0	0
	0	0	0	0	0	0	0	0
	0	0	0	0	0	0	0	0
	0	0	0	0	0	0	0	0
	0	0	0	0	0	0	0	0
	0							
CA10	0	0.0137	0	0	0	0.0813	0	0
	0	0	0.0013	0	0	0	0	
1.8656	0.0271	0.0411	0	0	0	0.0169	0	

0.0129	0.099	0	0	0	0	0	0	
0.0026	0.0128	0	0	0	0.0216	0	0	
0.0336	0.0077	0.0618	0.0205	0	0.1131	0.0259	0.0365	0
	0.0474	0	0	0.029	0	0	0	
0.1756	0.1011	0.0453	0	0.1179	0	0	0	0
	0	0.0105	0	0	0	0.3373	0	
0.0329	0	0.0093	0	0.0431	0	0	0	
0.19	0	0	0.0275	0	0.0611	0	0	0
	0.0259							
CA11	1.1755	1.3691	1.5996	2.1138	2.1555	2.1235	2.3721	
2.0928	1.9824	1.72	2.7174	2.9133	2.8529	1.4304	0.6819	
2.6075	2.0351	2.4339	0.9616	1.5451	0.8982	0.5436	3.1492	
1.0969	1.8651	2.3056	3.5519	2.7356	1.3397	1.7312	3.3974	
2.261	2.133	2.9447	1.0913	1.6646	1.3404	4.8598	3.2436	
1.8377	2.3874	3.5251	1.9991	2.6032	2.1313	0.0675	0.7837	
2.1586	1.8027	2.3954	2.1801	2.0521	2.3628	1.9189	2.9775	
1.5014	0.4023	1.2764	1.5628	1.1645	1.4918	0.7464	3.7541	
1.7286	2.7924	3.7661	2.9088	3.0281	2.2302	2.3081	3.6887	
1.3705	1.2559	2.9046	1.4506	1.2716	0.7768	2.026	2.6227	
2.5486	2.3403	2.3174	2.0341	2.9667	1.3145	1.9929	2.2488	
1.784	1.9677							
CA12	0.3069	0.3305	0.0406	0.0841	0.1908	0.144	0.0095	
2.9122	0	3.3835	0.0349	0.0267	0.8017	3.0353	0.2186	
0.0419	0.1431	2.6744	0.036	0.1776	0.4392	0.1689	0.4076	
5.4326	3.4873	0.5726	2.1251	0.0594	3.3824	0.1288	0.694	
0.0979	0.5876	0.0773	0.1303	2.4486	0.3027	0.3075	0.2175	
3.9912	2.637	3.9916	3.7666	0.0877	0.4027	4.8372	0.2421	
0.081	0.1404	1.1795	1	0.0029	0.1127	0.2002	0.1756	
0.3264	2.9748	0.2478	4.5083	0.2936	0.2124	0.8533	0.486	
0.3965	2.1844	4.9137	0.3153	0.1976	0.2196	0.1221	4.021	
0.3283	0.2365	3.1773	0.2286	1.0128	0.5738	0.1712	0.2009	
0.6137	0.3937	0.5132	2.4511	0.9821	0.5775	0.5242	3.1029	
1.0142	1.7749							
CA13	1.5321	0.0295	1.8498	0.902	1.1767	2.2858	2.2392	
1.6881	0.9303	0.5617	0.8612	0.9495	0.6756	1.6514	1.3471	
0.6756	2.3398	0.8764	1.1399	1.9315	2.1944	1.087	1.1946	
0.6494	0.8344	0.8872	1.5685	1.442	0.9171	0.7405	1.7848	
1.1604	0.6063	0.95	1.0727	1.5967	2.1914	1.0802	0.8204	
1.3228	0.2304	1.6562	2.0833	1.8688	0.7329	2.0674	1.9871	
1.5296	1.5646	0.8492	0.1933	1.1573	1.8733	0.6947	2.2728	
0.4311	2.3339	1.6226	1.7651	1.4423	1.04	0.1051	0.3111	
1.7064	1.2743	0.6597	0.7513	1.2347	1.3931	2.1632	0.8366	
1.4314	1.2314	0.7954	0.9522	1.9841	0.9572	0.5885	1.1774	
1.3416	1.8178	1.0144	0.8417	0.9867	1.8477	1.6363	0.7684	
0.6628	0.7806							
CA14	6.3009	4.8114	3.0306	3.6343	0.2875	1.7188	0.6334	
1.2071	2.9514	6.4921	3.086	1.9719	0.1499	4.091	0.4586	
3.1282	0.7693	3.3989	4.4337	0.5811	4.6075	0.3646	0.0172	
2.5382	0.2029	0.0832	0.9989	5.5214	0.1175	4.5359	0.0825	
3.5173	0.3749	2.8532	0.7599	5.1692	3.6063	5.7768	3.892	

0.9168	5.9678	0.0903	0.3626	0.3581	0.3223	0.1316	4.9949	
0.5689	0.0858	1.1396	4.9363	0.2925	3.9504	6.112	0.3857	
5.1824	0.5699	1.1087	5.1298	0.5096	0.4597	3.6959	5.7782	
2.0056	6.976	0.3791	3.0789	1.5928	3.5157	0.3515	1.6792	
0.6826	5.673	1.0713	1.5718	0.3292	5.5782	5.0298	4.9238	
1.1259	0.9779	0.209	0.439	2.9256	0.1933	2.9914	5.5177	
6.2101	6.0472							
CA2	0.1868	0.1293	1.5387	0	0	0	0.1372	
0.4205	0	0.1544	0.3757	0.0265	0	0.0974	0	
0.0274	1.774	0.0199	0.8346	0.0311	1.7413	0.1233	0.0041	0
	0	0	0.4326	0.4726	0	0.5353	0.0873	
0.8678	0	0.1349	0.0188	0.9226	0.1874	0.2448	0.1007	
0.2289	0	0.5548	0.1943	0.0463	0.4794	0.1317	1.2082	
0.3668	0	0.1995	0.106	0	0.5236	0.1152	0.0681	
0.2739	0.8758	0.9131	0	0.7153	0	0.1895	0	
0.0742	0.5587	0.0962	0	0.1056	0.0757	0.3504	0.4036	
0.0589	0.6037	0	0.0554	2.5753	0.0456	0.0604	0.0901	
0.0515	0.0569	0.0889	0.0539	0.4671	0	0.4332	0.0894	
0.1045	0.1135							
CA3	0	0	0	0.0496	0.2051	0.2244	0.2002	0
	0.0161	0	0.0287	0.1272	0	0	0	
0.0108	0.5745	0	0.0813	0	0	0.0571	0	0
	0.005	0	0.164	0	0	0.0247	0.4426	
0.1615	0	0.1586	0	0.011	0.0408	0	0.0121	0
	0	0.2894	0.0987	0.0334	0.0046	0.2629	0	
0.1943	0.027	0.1052	0	0.103	0	0.0216	0.0607	0
	0.2721	0	0	0.0822	0.0261	0.0258	0.0295	0
	0	0.0137	0	0	0	0	0	
0.0303	0	0.04	0	0.0581	0	0	0	0
	0.0287	0	0	0	0.0369	0	0.0507	0
	0							
CA4	0.0936	0	0	0	0	0	0	0
	0	0	0.0192	0	0.0531	0	0	
0.0364	0	0	0	0	0	0	0	0
	0	0	0	0	0	0.0781	0.1511	
0.0125	0	0	0	0.0397	0	0	0.0357	0
	0.0303	0.0108	0	0.0592	0	0.0411	0	
0.0559	0	0	0	0	0	0	0.1363	0
	0	0	0	0	0	0.0496	0	0
	0	0	0	0	0	0	0	0
	0	0.0173	0	0	0	0	0	0
	0	0	0	0	0	0	0	0
	0							
CA5A	0	0.1429	0	0	0	0.0967	0.0442	0
	0.0985	0	0	0.0537	0.1833	0	0	0
	0.115	0.0936	0	0.1084	0.0881	0	0.1333	
0.1164	0.0651	0	0	0.2713	0.0481	0	0	0
	0.125	0.2452	0.1247	0.0448	0	0	0.0426	0
	0.0893	0.0876	0	0	0.0411	0	0	
0.0704	0	0	0.1606	0.1168	0	0	0	

0.1169	0	0.0989	0	0.0521	0.0803	0.0419	0	
0.0698	0	0	0	0	0.0354	0	0.1553	0
	0.0537	0	0.1382	0.3715	0.0321	0.0491	0	
0.0867	0	0	0	0.0978	0	0	0	
0.078	0							
CA5B	1.2729	0.5537	0.8205	0.4204	1.085	3.1237	0.8286	
0.526	0.8736	1.3786	0.8454	0.4151	0.6123	1.2876	0.6893	
1.7726	1.0272	0.8777	0.7323	1.0201	0.9507	0.5914	0.661	
1.0212	0.3689	0.7309	2.4426	0.8397	0.603	1.3838	0.3773	
0.1331	0.8996	1.0351	2.5262	0.8386	0.3516	0.7788	0.3998	
1.0832	0.8074	1.3092	0.9784	0.6277	0.8205	2.6323	0.5797	
0.3224	0.6721	1.1198	0.7198	0.9175	0.8834	0.6432	0.9865	
1.6189	0.6743	0.5973	0.6468	1.4817	1.2293	1.5183	0.6426	
2.2584	0.3215	0.5574	2.0688	0.4739	0.4345	0.4749	2.0559	
1.6219	0.8063	0.4325	0.8711	0.9344	0.8105	1.2072	0.7113	
1.1635	0.3445	1.4012	1.3395	0.259	1.5315	0.7705	1.9153	
1.3544	1.3441							
CA6	0	0	0	0	0	0.2065	0.166	0
	0.065	0.3684	0.0498	0.034	0.0589	0	0	
0.2698	0	0.1134	0	0	0	0	0	0
	0	0	0.162	0.1321	0.1645	0.2535	0.0611	
0.0604	0.2909	0	0.0271	0.1759	0	0	0	
0.3449	0.0698	0.0285	0	2.0739	0	0	0.1504	
0.0774	0	0.1976	0.0617	0.1999	0	0	0.0335	0
	0.0375	0.0328	0.5095	0.029	0	0.0962	0.0513	
0.0448	0.0815	0.0454	0	0	0.9016	0	0	
0.0264	0.0706	0.4815	0	1.9345	0	0	0.2673	
0.3283	0.063	0.06	0	0	0.1721	0	0.0975	0
	0							
CA7	0.0741	0.1366	0	0	0.0945	0	0.0266	
0.1446	0	0	0.0416	0.0516	0.1308	0	0.0532	
0.1767	0.2203	0	0	0	0.049	0.0398	0.03	
0.0928	0	0.0538	0	0	0.0602	0.1342	0.3891	
0.0383	0	0.0375	0.0078	0.0818	0.135	0.0821	0.0928	0
	0	0.027	0	0.0814	0.0247	0	0.0614	
0.0424	0.0786	0	0.0657	0.0427	0	0.0716	0.2417	0
	0	0	0	0	0.0511	0.0339	0	
0.0607	0.0644	0.0624	0	0.0152	0.0287	0	0.1545	
0.0371	0.1489	0.0061	0	0.0869	0.0249	0	0.0254	0
	0.0415	0.0263	0.0346	0.1366	0	0	0	0
	0.0559							
CA8	4.8954	3.7509	3.9977	0	0	4.4559	2.3886	
3.1032	5.1674	4.5637	4.0244	4.2866	1.0439	5.4747	3.3664	
3.2402	0	5.1627	4.6897	4.9327	5.1919	2.8235	3.7716	
4.7306	1.2797	3.1001	0	3.537	4.0303	4.6182	0	
4.9402	4.896	5.8747	4.5549	4.7689	3.8832	2.4908	4.2515	
1.8581	3.241	0	6.5787	3.9666	0.4355	4.5294	4.0291	
0.6197	3.9172	0	3.6159	1.1089	5.1935	2.7518	0.4764	0
	0.7309	0	5.5764	0.6674	4.6217	4.701	1.8922	
2.8289	0.5002	0.4468	5.5283	3.8387	3.9558	0.9013	4.7856	

4.5816	3.8899	3.4139	6.4412	5.1216	0	5.686	3.9939	
3.4105	3.4467	4.5556	0.5156	4.147	2.9409	6.1521	5.2339	
4.1044	4.4874							
CA9	0.0888	1.1644	0.0769	1.924	3.4241	0.2956	0.0379	
0.1417	0	0.0447	0.108	0.5293	0.0908	0	0.0496	0
	0.4645	0	0	2.6761	0.3126	3.6766	1.8599	
0.2762	0.077	1.2726	2.1446	0	0.1413	0.5494	0.3521	0
	0.0521	0.2306	0	0.1734	0.082	0	0	
0.4978	2.1674	1.684	0	0	0.1267	2.2531	1.0783	
0.2695	0	0.1352	0.0454	2.8684	0	0.4703	0.036	0
	0.0729	0	0.0372	0	1.5965	2.1988	0	
0.1321	0	3.4539	0	0.0473	0	0	1.6461	
0.0596	0	2.7456	0.0196	0	0.0397	0.0276	0.4305	
0.6226	0.8742	2.0901	0.0752	0	0	0	0	0
	0.1273							
CAAP1	2.9861	3.2629	2.7643	4.1818	4.4772	2.7033	3.1146	
3.0456	0	2.8126	2.7047	3.7175	3.1232	3.7972	2.4418	
4.6587	3.5206	3.6131	3.3453	3.244	2.736	3.0576	2.8805	
3.4038	2.9998	1.6432	2.5079	3.7858	3.3	3.2327	4.0676	
3.5446	2.693	2.7155	2.5927	3.1396	2.9059	3.3512	3.2482	
3.0382	3.9041	4.0489	2.7788	2.8075	3.9053	2.784	3.3172	
3.1767	3.2625	4.0669	3.1265	3.7838	3.6932	2.981	3.5971	
2.6772	4.1164	3.7401	4.2779	3.7249	2.8886	6.1821	4.284	
2.6002	2.9184	3.0682	2.6389	3.1254	2.5679	3.147	2.7405	
2.7511	2.6286	3.6372	3.136	2.7435	2.7448	3.3212	3.7635	
2.5821	3.417	3.2704	2.3576	2.9738	3.0258	3.3607	2.6134	
2.7566	2.6571							
CAB39	4.7472	4.0703	4.738	4.6858	4.4525	4.1471	4.7985	
5.316	4.7519	4.548	4.572	5.0135	5.3721	5.0305	5.2022	
4.633	4.5588	5.2287	4.9436	4.4148	4.9979	4.6003	4.4513	
5.2912	5.0471	5.2476	4.5653	4.8044	4.7409	4.932	4.3266	
4.7613	5.0685	5.0293	4.7985	4.743	4.765	4.6116	4.2344	
5.2042	4.4449	5.4853	5.532	4.6604	5.4214	5.5966	4.843	
4.8498	5.3088	4.4412	4.6116	5.0914	5.2457	5.0286	4.2593	
4.3093	5.2899	4.5532	4.4843	4.4033	4.6484	4.2994	4.2744	
4.7795	4.9266	5.1589	4.8583	5.1029	5.5574	5.1848	5.2796	
4.7402	5.0268	4.3573	4.8695	5.1319	4.7359	4.7396	4.81	
4.2744	4.7725	4.4874	4.935	4.6537	5.1359	5.0156	4.9138	
4.7624	4.8624							
CAB39L	2.825	2.3	2.7766	2.7448	0.8948	2.1326	2.7405	
2.4745	1.8504	3.0406	2.5619	2.0307	1.0225	2.512	1.5575	
1.8755	2.4146	2.4907	2.8876	1.954	2.4459	1.672	1.2049	
2.1521	1.8008	1.5661	0.7918	2.344	1.349	2.7691	1.621	
1.2308	2.0059	2.3617	2.1315	3.1616	1.4933	2.7285	2.1798	
1.5494	2.428	1.9687	3.2245	1.4259	1.0189	1.9815	2.5367	
2.2874	1.8089	1.7189	3.3232	1.2584	2.8757	2.0865	1.6215	
3.6325	1.7949	1.1538	2.6018	1.028	1.858	2.3627	2.6038	
2.1596	2.8801	1.1104	1.8464	3.562	2.5255	2.9861	2.483	
2.3289	2.8077	1.6566	1.4348	2.9689	2.3303	1.7431	1.7519	
2.6339	1.5448	1.703	2.9056	2.0778	1.3848	2.6183	3.25	

2.8749	2.9761							
CABIN1	3.9136	4.1809	3.7922	3.311	3.1215	3.8925	3.5894	
4.046	4.3502	3.8698	4.3705	3.6869	3.5868	3.7417	4.0162	
3.8737	3.1643	3.2407	3.9763	4.1834	3.5846	3.9932	3.0732	
4.3305	3.3034	3.6808	3.0322	4.1534	3.647	4.2976	5.1285	
3.8961	3.7884	4.4164	4.4093	3.2613	3.3025	4.3834	4.3763	
4.5113	3.6804	3.2387	3.3169	3.8163	3.351	3.466	3.7988	
3.9363	2.8196	2.7674	3.219	2.8801	3.5407	4.5396	3.5232	
3.5305	3.1916	3.1787	3.5674	3.0051	3.534	4.3264	4.3679	
3.6682	3.5065	3.4002	4.455	3.9198	4.3089	2.9352	3.6442	
4.1508	3.612	4.3123	3.6169	3.7881	4.5932	4.0826	4.4425	
4.628	4.3163	3.6497	3.9627	3.7708	4.3363	4.0161	4.0239	
3.6854	3.9779							
CABLES1	5.3287	5.0574	5.3614	4.3912	2.7107	3.9507	4.4877	
3.5072	6.0131	6.7957	6.0297	5.246	2.0572	5.4516	4.1916	
4.388	1.6881	5.3207	5.4177	4.934	5.7049	3.3318	2.3989	
5.0805	2.3871	2.6812	1.3777	5.851	3.0309	5.8456	1.2984	
5.2861	5.0899	5.5837	3.0013	5.7385	3.1407	5.7839	5.2048	
2.9585	6.0945	1.8032	2.8681	6.1059	1.4378	2.397	4.6953	
3.8762	4.9658	2.5583	6.732	2.832	5.897	5.8475	2.8198	
5.4885	1.9514	0.8151	5.6076	1.2408	4.0149	5.4842	5.1736	
5.7304	5.9097	1.1271	6.783	5.1603	6.0479	2.6159	4.256	
5.1431	5.9828	3.0934	3.7019	5.5822	5.4026	4.6515	5.7987	
4.8093	5.2032	4.2855	3.8847	4.0817	3.1902	5.3153	6.5749	
6.7238	7.008							
CABLES2	3.179	2.0873	4.1613	1.7964	1.8614	2.4742	2.7744	
2.8752	3.0034	1.8804	2.2732	0.8524	2.8641	1.5152	2.8522	
2.9756	1.9405	2.4456	2.3406	3.2521	3.5932	2.3621	2.1072	
2.0176	3.1254	2.911	1.1763	2.3824	2.3406	2.5759	4.6059	
1.9919	2.2324	1.6685	2.5548	2.379	2.533	1.6886	1.8054	
0.9914	1.9058	1.4908	2.8243	1.0366	2.9165	2.4475	2.5618	
2.9963	1.274	1.8184	2.9114	1.3657	2.3171	2.179	3.8904	
2.996	1.7249	3.0694	1.4237	3.2459	1.924	1.3837	2.3638	
2.8658	1.8125	2.0291	2.4312	2.2601	2.3805	2.5488	2.4338	
3.5344	3.6222	0.7246	2.6532	3.507	2.1598	2.7478	2.1332	
1.6126	2.1189	0.6127	2.1589	2.673	3.0045	2.6153	1.5307	1.8
	1.998							
CABP1	0.0965	0.4883	1.0634	0	0	0.3313	0.4132	
0.7515	0.156	0.3492	0.2617	0.01	0.0261	0.1464	0.2139	
0.25	0.6656	0	0	1.2542	0	0.2108	0.1036	
0.4634	0.1107	0.2673	0	0.3687	0	0.3317	0.3466	
0.045	0.0543	0.2911	0.1236	0.267	0	0.1134	0.0605	
0.785	0.0794	0.1778	0.9004	0.1644	0	0	0	
0.3909	0.0332	0.5737	0.0454	0	0	0.2409	0.352	
1.8637	0.4917	0.1077	0	0.1212	0	0.1505	0.0464	
0.1139	0.0628	0.164	0.4173	0.0933	0.0842	0.2276	0.3364	
0.2509	0.4701	0.383	0	0.1084	0.0871	0.2037	0.2157	
1.0435	0.1234	0	0.1525	0.1452	0.1086	0	0	0
	0.382							
CABP2	0	0	0	0	0	0	0	0

	0	0	0	0	0	0	0	0
	0	0	0	0	0	0.0496	0	0
	0	0	0	0	0	0	0	0
	0	0	0	0	0	0	0	0
	0	0	0	0	0	0	0	0
	0	0	0	0	0	0	0	0
	0	0	0	0	0	0	0	0
	0	0	0	0	0	0	0	0
	0	0	0	0	0	0	0.0406	0
	0	0	0	0	0	0	0	0
	0							
CABP4	0.1483	0.0429	0	0.0305	0.0134	0.0265	0.0436	
0.0482	0.0868	0.2351	0.0688	0.0281	0.0439	0.0634	0.1032	
0.0126	0.1193	0.0881	0.1115	0.1305	0.0765	0.0452	0.0519	
0.0377	0.0349	0.0256	0.0505	0.0331	0.0134	0.0473	0.0338	
0.0248	0.0552	0.0492	0.3831	0.0877	0.0361	0.0473	0.0434	
0.0639	0.0715	0.6304	0.067	0.0827	0.0065	0.0349	0.0525	
0.0294	0.0433	0.0986	0.0326	0.6011	0.0741	0.038	0.0679	
0.0708	0.4038	0.0334	0.5279	0.0661	0.0393	0.0108	0.4538	
0.041	0.0604	0.0174	0.1057	0.0209	0.0912	0.0715	0.0231	
0.0144	0.0097	0.1909	0.0475	0.0659	0.0829	0.3621	0.0313	
0.0495	0.0668	0.0459	0.0402	0.0393	0.0409	0.0426	0.0504	
0.0845	0.1386							
CABP5	0	0	0	0	0	0.0413	0	0
	0	0.0446	0	0.058	0	0.1279	0	0
	0	0.0381	0	0.0458	0.0417	0	0.0073	
0.0134	0	0.0162	0	0	0.0212	0	0	0
	0.0289	0	0.0032	0.0167	0	0	0	
0.0214	0	0	0.0381	0.0269	0.0135	0	0	0
	0	0	0	0	0.0533	0	0.0482	0
	0	0	0	0	0	0	0	0
	0	0.0284	0	0	0	0	0.0304	
0.0345	0	0	0.0302	0	0.0174	0	0	0
	0	0	0	0	0	0	0	0
	0							
CABP7	0	0.1659	0.221	0.1435	0.0011	0	0	
0.1973	0.0195	0.0245	0.0564	0.0245	0.0375	0.0927	0.0377	
0.0087	0.1313	0.0573	0.209	0.1248	0.1403	0.0015	0.0016	
0.2399	0.0307	0.1032	0.0858	0.2313	0	0.0729	0.0841	
0.0039	0.0709	0.0678	0.0095	0.0528	0.0279	0.0774	0.0758	
0.0753	0.1265	0.0482	0.1013	0.03	0.1511	0.0326	0	
0.037	0	0	0.0255	0	0.0865	0	0.046	
0.0936	1.844	0.0301	0.0491	0.0281	0	0.0203	0.0888	0
	0.0508	0	0.1194	0.0273	0.0374	0.0853	0.0355	
0.0239	0.0426	0.0095	0.0495	0.0367	0.0062	0.0143	0.0158	
0.0253	0.0242	0.016	0.0097	0.0518	0.1406	0.0392	0	
0.1238	0							
CABS1	0	0	0	0	0	0	0	0
	0	0	0	0	0	0	0	0
	0	0	0	0	0	0	0	0

	0	0.0291	0.0198	0	0	0	0	0
	0	0	0	0	0	0	0	0
	0	0	0	0	0	0	0	0
	0	0.0998	0	0	0	0	0	0
	0	0	0	0	0	0	0	0
	0	0.0406	0	0	0	0	0	0
	0	0	0	0	0	0	0	0
	0	0	0	0	0	0	0	0
	0							
CABYR	1.1715	0.2314	0.4199	0.1353	1.7525	1.7808	1.3454	
0.7033	1.259	1.7954	1.6827	1.4606	1.6599	1.413	2.5153	
0.2684	1.0113	0.9423	0.8996	1.8839	0.6214	0.8704	1.3216	
0.3077	2.2987	2.5537	2.0421	0.6869	0.3437	0.3208	3.4177	
1.3077	0.4199	0.6096	0.273	2.1694	1.1268	0.1925	0.1844	
0.3444	1.388	3.1599	2.8453	1.1414	5.366	2.1522	2.8295	
0.414	0.8833	1.6886	1.8666	2.6592	1.5539	0.2112	4.4425	
0.8543	1.6179	0	1.865	0.3691	2.6616	0.518	1.6277	
0.9686	1.1116	2.8348	0.5877	2.2097	1.6922	1.8044	2.5675	
1.8663	0.7599	0.2716	2.5209	2.3336	0.415	0.3588	0.4677	
0.5123	0.4905	2.4358	0.4831	1.605	2.4139	1.4121	1.3177	
1.7514	1.1606							
CACFD1	1.1771	1.5185	1.542	0.6257	1.3617	1.7625	1.2735	
2.1055	1.0058	0.9675	1.3091	0.5741	1.4649	0.8461	1.7292	
2.0513	1.5445	0.9727	0.234	1.4599	1.5123	0.7864	0.8246	
1.0028	1.6841	1.604	1.5244	1.7206	0.9681	1.3688	2.7874	
2.0983	1.3664	1.7827	1.4368	0.6811	1.1989	4.1252	2.3665	
0.3913	2.0627	1.4456	0.8461	1.2249	1.2363	0.737	0.3943	
1.4899	2.0476	1.2551	0.7975	0.6853	0.9076	1.4858	2.4775	
1.0357	1.2014	2.1582	0.8237	1.6321	0.8316	0.2683	1.7801	
1.6529	2.2999	2.6774	2.1984	1.4675	1.7276	2.0918	0.9802	
0.3507	1.2614	0.4595	1.0909	1.4159	0.8442	2.3349	0.7432	
1.068	1.4706	0.7043	2.2968	2.1963	2.1541	1.4522	1.6017	
1.5685	2.4744							
CACHD1	2.1402	3.1659	3.2291	2.2	1.6812	2.4395	3.6622	
1.8341	2.5795	2.7115	3.0159	2.7262	0.6858	2.7345	1.2748	
2.9743	2.9862	3.5236	3.2503	2.1773	2.2703	2.0239	1.5012	
2.688	1.9893	2.1613	2.1865	3.105	1.4686	4.9604	1.7967	
2.1268	2.423	2.596	1.7044	2.6072	1.0334	1.2004	1.4857	
2.9854	2.1705	2.7123	2.1787	2.4525	1.5562	1.6069	3.8925	
2.531	1.3318	1.9784	1.4062	2.1966	2.6397	2.4085	2.3245	
2.8119	1.8167	2.3492	3.1703	2.4604	2.2084	2.3202	1.2581	
3.2984	1.3165	3.5415	1.2726	1.521	4.9253	2.3341	2.1649	
2.1582	1.5627	2.3948	2.2485	3.3628	2.6106	1.5217	2.5225	
2.2485	2.8964	1.6215	1.9422	0.6929	2.1926	2.5368	1.6319	
1.8972	1.7222							
CACNA1A	0.1355	1.0593	0.0659	0.2552	0.3721	0.2187	0.5593	
0.0939	0.1309	0.5427	0.0471	0.2614	0.2392	0.2918	0.2121	
0.4278	1.2065	1.3658	0.247	0.3868	0	0.1245	0.0807	
0.1166	0.0487	0.6102	0.1371	0.0751	0.1939	0.0958	0.1192	
0.0498	0.0478	0.2015	0.163	0.2408	0.0592	0.0478	0.3307	

0.2006	0.3998	0.2059	0.5393	0.2832	0.2232	0.7588	0.1488	
0.0258	0.0771	1.527	0.1995	0.4738	0.1201	0.0441	0.1687	
0.1785	2.1266	0.0264	0.1658	0.0781	0.5041	0.1308	0.0676	
2.3897	0.422	0.8063	0.1496	2.5128	0.0227	0.127	0.1803	
0.1228	0.1622	0.0389	0.0464	0.1296	0.7185	0.0469	0.6237	
0.1329	0.0604	0.0542	0.3422	0.087	0.885	0.1706	0.5233	
1.6775	3.8146							
CACNA1B	0	0.0384	0.939	0	0.0203	0.0717	0.0976	
0.0259	0	0.0065	0.0703	0.0039	1.0072	0.2166	0.0062	
0.0083	0.0216	0.0158	0	0	0	0.0166	0	
0.0458	0.0046	0.0694	0	0	0.0295	0	1.3815	
0.0034	0.0161	0	0	0.2041	0.0029	0.023	0.0913	
0.0141	0	0	0	0.0171	0.0083	0.0087	0	0
	0	0.3211	0	0.0115	0	0.0612	1.4248	0
	0.0202	0	0.0126	0	0.0866	0.0055	0	0
	0.714	0.004	0	0.2151	0.0457	0.0317	0.0269	0
	0.0655	0.0192	0.0624	0.011	0.0568	0.0161	0.0202	
0.1955	0.0545	0.0054	0.0109	0	0.0212	0.0341	0.0444	
0.237	0.0257							
CACNA1C	0	0	0.0938	0	0.03	0.06	0.1683	
0.0756	0.2013	0.0357	1.271	0.0959	1.7628	0.0584	0.0395	
0.0456	0.0548	0.0614	0.077	0.0716	0	0.1776	0.2704	
0.0713	1.9477	2.3951	0.1437	0.0678	0.2232	0.1664	0.1241	
0.0291	0.0449	0	1.1492	0.0356	0	0.2251	0.7191	
0.4619	0.1524	0.1664	2.2781	0	0.0602	1.3821	0.1773	
0.0418	0.0367	1.6455	0.0504	0.0403	0.0929	0	0	0
	0	0.5036	1.8812	0.6166	0.15	0.2274	0.7453	0
	0.0763	0.2357	0	0.0429	0.2532	0.0989	0	
1.0844	0	0.2417	1.554	0	0	0.9632	0.4437	
0.007	0.0431	0	0	0.3109	0.8197	0	0.2215	
0.0759	0.0765							
CACNA1D	0.0368	1.0954	0.6007	0.4076	0.8628	0.0834	1.3513	
0.0345	0.2359	1.8747	1.3458	0.1369	0.1087	0.0369	0.0163	
1.7879	0.0373	0.6464	0.4528	0	0.8518	0.2075	0.0697	0
	0.3608	0	0.0223	0.4139	1.4021	0.5936	0.5532	
0.1193	0.6585	0	0.3935	1.6088	0.0837	0.4536	1.1368	
0.0847	0.0282	0.0045	0.6767	0.1813	0.0513	0.0354	0.481	
0.721	0	0	0.1045	0	0.2418	1.4772	0.4066	
1.0158	0.1069	0.6204	0.301	0.8032	0.213	0.1475	0.0142	
1.6885	0.121	0.0462	0	0.1695	0.8699	0.352	0.0021	
0.1507	1.5198	0.3712	0.2553	0.0842	0.4143	0.9817	0.3274	
0.0306	0.4235	0.0937	0.0397	0.5431	0	0.1488	0.5254	
0.562	0.7982							
CACNA1E	0.0221	0.0931	0	0	0	0	0.0736	0
	0.0041	0.4628	0.2002	0.0319	0	0.0346	0	
0.0794	0	0.5346	0.0335	0.4862	2.7246	0.1556	0.051	
0.0039	0	0	0.0141	0	0.0277	0	0.0262	
0.0181	0.0402	0.1278	0.0892	0.6571	0.1617	0	0	
0.0704	0	0.2611	0	0	0	0.2249	0	
0.0473	0.0016	0	0	0	1.3423	0.0291	0	0

	0.0222	0.0221	2.3062	0.0166	0.0148	1.7804	0	
0.093	0	0.0185	0	0.0477	0.0602	0	0	
0.1923	0.0317	0.0123	0.0226	0.09	0.0562	0.0426	0.0248	0
	0.015	0	0	0.0177	0	0.6775	0.0187	0
	0							
CACNA1F	0.1158	0	0	0	0	0	0	0
	0.0055	0.0072	0	0.0014	0	0	0.0026	
0.0022	0	0.0283	0	0	0	0.0232	0.0173	0
	0.0019	0.0046	0.0117	0.0248	0.0085	0	0	0
	0	0.0078	0	0.0255	0.0079	0	0	0
	0.0181	0.0315	0	0	0.0036	0	0.0413	0
	0.0107	0	0	0	0.0282	0	0	0
	0	0	0.0396	0	0.0125	0.006	0.0096	0
	0	0.0026	0	0	0	0	0	0
	0	0.0084	0	0	0	0	0	0
	0	0	0.0755	0	0	0.0602	0.0229	
0.0189	0.0246							
CACNA1G	0.0367	0.0401	0.074	0.0962	0.0686	0.031	0.0926	
0.0797	0.0214	0	0.0594	0.0268	1.3425	0.2513	0.0572	
0.0492	0.5905	0.0557	0	0.0681	0.0795	0.0452	0.7645	
0.019	0.4137	0.0398	0.0151	0.108	0.0767	0.047	0.9171	
0.0044	0.0337	0	0.07	0.1262	0.1281	0	0.0058	
0.0528	0.0411	0.0618	0.2566	0.0487	0.1028	0.6249	0.1331	
0.0854	0.2493	0.1144	0.1667	0.0511	0	0.0198	1.0162	
0.2772	0.1271	0.0244	0.0169	0.0581	0.0339	0.0605	0	
0.0589	0.0287	0.1374	0.6596	0.0121	0.0196	0.3992	0.1039	
0.0639	0.1407	0.0296	0.0287	0.0704	0.0749	0.0184	0.0168	
0.173	0.1138	0.0256	0.0774	0.0218	0.0779	0	0.0803	
0.0487	0.0279							
CACNA1H	2.8523	1.4009	2.082	0.0968	0	0.6479	0.1733	
0.0921	0.0992	0.0979	3.3654	0	0.3649	0.225	0.1055	
0.1416	0.2013	1.5765	0.5716	0.8102	0.0674	0.0543	0.0488	0
	1.2978	0.074	0.1575	1.2953	0.046	1.0388	2.197	
0.1956	0.0575	0.5298	0	0.4675	0.5585	0.5736	2.0688	
0.6468	0.1371	0	0.833	0	0.9008	0.0747	2.1005	
0.456	0	0	0.0759	0	0.7203	2.081	1.4997	
0.5105	0	0	1.2527	0	0.1845	0.4213	1.3021	0
	3.4578	0.1207	1.5117	0.802	0.0073	0.451	0	0
	1.8595	0	0	0.63	0.2507	0.8598	0.5057	
0.4903	1.7071	0	0.1782	1.6303	0	0.6365	0.7736	
0.3231	0.0728							
CACNA1I	0	0	0	0.0522	0.0016	0	0.0043	
0.0654	0.0049	0.01	0.002	0.0105	0.3886	0	0	
0.0072	0	0	0	0	0.0282	0.0035	0	0
	0.1156	0	0.0206	0	0.009	0.009	0.5005	
1.00E-04	0.0095	0.0162	0.0039	0.0671	0.0013	0.0155	0.0113	0
	0.0121	0	0.0221	0.0109	0	0.0105	0	
0.0359	0	0	0.0159	0	0	0	0	0
	0.0461	0.0792	0	0	0	0	0.0117	0
	0	0.0432	0.0752	0.0261	0	0.0704	0	0

	0	0.0284	0	0	0.0027	0.0264	0.0286	0
	0.0084	0	0.0082	0.0207	0.0408	0	0	0
	0							
CACNA1S	0	0	0	0	0.0426	0	0	
0.0041	0	0	0.0012	0.1514	0	0	0	0
	0	0	0.036	0	0	4.00E-04	0	0
	0.0015	0	0	0	0	0	0.0114	
0.1262	0	0	0	0.0049	0	0	0.0037	0
	0.1978	0	0	0	0.003	0.0505	0	0
	0.0869	0	0.0116	0	0	0	0.0172	0
	0	0	0	0	0	0	0	0
	0	0	0	0.0031	0	0	0	0
	0.0136	0.2195	0	0	0	0	0	0
	0	0	0	0	0.025	0.0393	0	0
	0							
CACNA2D1	0	1.9764	1.7551	0	1.9973	2.5369	0	
2.0687	0	2.4268	0.7764	1.3122	1.5328	3.01	2.5102	0
	3.6275	0	1.5002	1.971	2.9798	3.084	3.9489	0
	2.1106	2.4559	2.3771	0.5406	2.0417	1.5723	3.5769	
1.0204	0	0	2.1673	2.0956	2.1398	1.9428	0.9972	
3.6408	0	1.6393	2.7464	2.368	2.2746	3.4952	2.1302	
2.7296	2.1338	2.23	0	2.4002	0	1.7968	1.8271	
3.202	1.957	2.471	0	2.8706	2.4986	2.5189	0.3233	
0.2722	2.0149	5.9462	2.9841	1.6184	0	1.9028	0.3366	
0.3258	3.266	1.7974	0.8522	1.976	0.2621	1.8283	0.264	
2.7277	1.6866	2.0149	0.2813	1.5291	0.4074	0.2864	1.9327	
1.2574	1.0819							
CACNA2D2	0.0863	0.2616	0.1943	0.1384	0.0887	0.1046	0.2807	
0.0654	0.0345	0.1207	0.2685	0.0529	0.3281	0.043	0.3015	
0.7825	0.0494	0.6346	0.0529	0.0786	0.2021	0.0525	0.13	
0.0864	0.0837	0.0659	0.1238	0.6015	0.1008	0.0787	2.2333	
0.11	0.0623	0.2176	0.0427	0.274	0.0366	1.1905	0.065	
0.0959	0.1147	0.0832	0.0779	0.0433	0.131	0.0321	0.152	
0.0276	0.0574	0.074	0.0218	0.0355	0.0767	0.0782	1.0744	
0.6174	6.00E-04	0.0247	0.1736	0	0.0061	0.0643	0.1772	
0.2677	0.3278	0.1809	1.2333	0.0779	0.407	0.0831	0	
0.0262	0.1335	0.0288	0.3324	0	0.0153	0.3111	0.0025	
0.0861	0.041	0.0038	0.1437	0	0	0.0266	0.078	
0.1348	0.1125							
CACNA2D3	0	0.1058	0	0.0902	0.9857	0	0.5028	
0.0175	0	0	0.0221	2.9528	1.1917	0	0.304	0
	0.2289	0	0	0.0287	0	0.1892	0.0401	0
	2.8849	4.3933	0.2793	0	0.088	0.0439	0.033	
0.0064	0.1157	0	0.1302	0	0	0	0.0466	0
	0.0126	2.556	2.1865	0.0259	0.8157	0.0233	0.025	
0.0426	0.3314	1.0112	0.581	1.7405	0	0	0.3363	
0.5644	2.2851	0.1513	0	0.0882	0.7704	0.0353	4.00E-04	0
	0	0.4116	0.021	0.3409	0.1343	0.4146	0.0868	
0.016	0.1266	0.101	0.1463	0	0	2.9413	0.0028	0
	0	0.0029	0.1198	0.0097	0.6275	0	0	

0.0381	0							
CACNA2D4	0.0211	0	0	1.977	0.0295	0.8041	0	
0.0014	2.00E-04	0.7377	0	0	0	0.1117	0.0176	0
	5.3602	1.2351	0	9.00E-04	1.4062	2.4642	1.773	
1.81	0	0	0	0.0047	2.6805	0	0.0019	
3.7863	0	0	0.6694	0.1227	4.0945	0	0.5293	
0.0299	3.1021	4.1506	2.1409	0	0.1253	0.0187	0	
4.2735	3.5032	0.5158	0	2.4677	0	0.0794	0.077	0
	5.5933	2.5996	1.2706	2.649	2.2909	4.5228	0	0
	0	0.065	0	0	0	0.0932	0.069	
0.5788	0	4.3553	0	0.7625	0	0.1678	0	0
	0.1882	0.0673	0.1009	0.7395	1.7498	0.1244	0.096	
0.0575	0.1194							
CACNB1	1.8317	1.328	1.8219	1.0176	1.2792	1.5076	0.81	
1.245	1.3457	1.257	1.6809	0.9528	1.9414	1.5808	1.188	
1.1613	2.7137	1.0401	1.0384	2.2892	1.9854	1.7224	0.9408	
1.0811	2.1082	1.7851	1.5019	1.9075	1.1261	0.8721	1.2643	
0.7994	1.2247	1.6304	1.5059	0.9006	1.7041	1.1643	1.1711	
1.3487	1.7983	1.5188	1.6483	1.6948	1.0956	1.0449	1.3911	
1.5831	1.6871	1.9766	2.0148	1.0153	1.5318	1.8414	1.1917	
1.7318	2.2553	1.3607	1.29	1.4963	1.9743	1.7704	1.216	
1.4886	1.4548	1.7933	1.483	1.6422	2.1385	1.6055	1.4433	
1.4934	1.4474	0.7609	1.4576	2.154	1.8663	0.6846	1.013	
1.0614	1.3816	1.5076	1.7343	0.6624	1.3531	1.5266	1.6958	
1.5318	1.4567							
CACNB2	0.3118	0.1163	0.2732	0.6076	0.1122	0.4101	0.1084	
0.1565	0.2496	0.4895	0.3416	0.1075	0.2092	0.2077	0.1574	
0.1186	0.455	0.2233	0.4683	0.1768	0.6041	0.1469	0.0923	
0.5203	0.0803	0.1431	0.1041	0.2664	0.1466	0.1463	3.0202	
0.1798	0.0965	0.2688	0.146	0.1228	0.1729	0.1347	0.0842	
0.6244	0.0888	0.127	0.1185	0.4283	0.1556	0.103	0.1638	
0.1909	0.1493	0.2761	0.322	0.1433	0.2789	0.1303	0.9829	
2.2468	0.1319	0.3269	0.1967	0.5643	0.3062	0.2004	0.4365	
0.2562	0.1547	0.4235	0.2062	0.0331	0.1262	0.0525	0.1646	
0.1754	0.4334	0.1572	0.0838	0.1756	0.3346	0.1497	0.0423	
0.1355	0.2981	0.1489	0.0025	0.0659	0.1265	0.1358	0.073	
0.0803	0.1715							
CACNB3	2.3696	2.5946	3.0611	2.4837	3.1669	2.402	1.593	
1.664	2.4155	2.4076	3.2428	2.0077	2.5029	2.1744	2.7968	
2.652	3.2984	2.9581	1.6108	2.8299	2.5555	2.5166	2.6466	
1.894	3.8135	3.6067	4.1199	3.6636	2.0887	2.4476	3.5272	
1.7027	1.9908	2.4987	1.9998	2.4246	2.2913	2.7561	2.3762	
1.2799	3.6052	4.2248	2.8534	2.7815	1.8045	2.395	1.7715	
1.7074	3.5473	3.7806	2.2663	2.4121	2.0158	1.604	3.2684	
1.8141	2.362	3.3015	1.8859	3.4647	2.4447	1.4666	2.8468	
2.8173	3.6935	2.8885	3.6055	4.8254	2.0883	2.3126	3.5278	
2.5627	2.224	2.1462	2.3844	2.0293	2.523	1.7157	2.2772	
2.3501	2.7775	2.7211	2.7151	1.8298	3.359	2.0813	2.6483	
2.7976	3.4449							
CACNB4	0.2557	0.5103	0.849	0.2351	0.2334	0.2834	0.3716	

0.2088	1.0511	0.1525	1.1389	0.1589	0.1147	0.1972	0.0281	
0.5635	0.436	0.0509	0.2621	0.0911	0.1638	0.2991	0.0899	
0.2051	0.0931	0.2189	0.4919	1.4677	0.1758	1.4987	0.1584	
0.0609	0.2627	0.6954	0.1418	0.099	0.0724	0.1469	0.2768	
0.1675	0.0538	0.0864	0.2015	0.5033	0.3175	0.0616	0.3411	
0.6876	0.0624	0.1451	0.1096	0.0908	0.5859	0.1797	0.4604	
0.315	0.2885	0.7822	1.1008	0.5559	0.03	0.2202	0.0252	
0.1261	0.1589	0.266	0.014	0.3165	1.0208	0.0784	0.5696	
0.186	0.3358	0	0.4174	1.1437	0.2408	0.002	0.2942	
0.2316	1.1515	0.0248	0.2633	0.3302	0.0122	0.454	0.0475	
0.5039	0.3136							
CACNG1	0.1029	0	0	0	0	0	0.0451	0
	0.0948	0	0	0.0264	0.036	0	0	0
	0	0	0	0	0	0	0	0
	0	0	0	0	0	0	0	0
	0	0	0	0	0.097	0.1662	0	0
	0	0	0	0	0.1624	0	0	0
	0	0	0	0	0.1134	0	0	0
	0	0	0	0	0	0	0	0
	0.0785	0.0521	0	0	0.0369	0	0	0
	0	0	0.0887	0.1089	0.0331	0	0	0
	0	0	0	0	0	0	0	0
	0							
CACNG2	0	0.0139	0.0249	0	0	0	0.0192	0
	0	0	0.0031	0	0	0.0392	0	
0.0042	0	0	0	0.0252	0	0	0.0028	
0.017	0	0.007	0.0141	0	0	0	0	0
	0	0	0.0024	0	0	0	0	
0.0102	0	0	0.0211	0	0	0	0	0
	0	0	0	0	0	0	0	0
	0.0139	0.0137	0.0105	0	0.0085	0.003	0	0
	0	0	0	0.0013	0.0041	0	0	
0.0145	0	0	0.0073	0.0103	0	0	0	0
	0	0.0078	0.0052	0.0125	0.0206	0.0326	0	0
	0							
CACNG3	0	0	0	0	0	0	0	0
	0	0	0	0	0.0168	0	0	0
	0	0	0	0	0	0	0	0
	0	0	0	0	0	0	0	0
	0	0	0	0	0	0	0	0
	0	0	0	0	0	0	0	0
	0	0	0	0	0	0	0.018	0
	0	0	0	0	0	0	0	0
	0	0.0011	0	0	0	0	0	0
	0	6.00E-04	0	0	0	0	0	0
	0	0	0	0	0	0	0	0
	0							
CACNG4	0.1179	0.4022	0	0.1141	0.0356	0	0.1366	
0.0462	0	0.0493	0.0946	0.0491	1.4429	0.0846	0.0755	
0.0396	0	0	0	0	0	0.0485	0.0975	0

	2.0114	0.0445	0.0835	0	0.0504	0	1.1588	
0.0456	0.0542	0.0743	0.033	0.0818	0.1981	0.0606	0.0627	0
	0	0	0.0674	0	3.4072	0.1602	0	0
	0.0825	0	0.0899	0	0.125	0	0.5474	
0.055	0.0698	0.7676	0	2.505	0.0278	0.187	0.0106	
0.0542	0.5969	0.1825	0.0106	0	0.0288	0.3425	0.0403	0
	0.0341	0	0.1225	0.0145	0.009	0.0683	0	
0.114	0.0144	0.0356	0.0377	0	3.011	0.1312	0.0617	
0.0643	0							
CACNG5	0	0	0	0	0	0	0	0
	0	0	0	0	0	0	0	0
	0	0	0	0	0	0	0	0
	0	0	0	0	0.1966	0	0.3465	0
	0	0.0688	0.142	0.0521	0	0.2476	0.0491	0
	0	0	0	0	0	0	0	0
	0	0	0	0	0	0	0.0912	0
	0	0.0862	0	0	0	0	0	0
	0.1689	0.0279	0	0	0	0	0	0
	0	0	0	0.2654	0	0	0.0481	0
	0	0	0	0	0	0	0	0
	0							
CACNG6	0	0	0	0	0.5442	0	0	0
	0	0	0	0.9182	0.1181	0	0	0
	0.5079	0	0	0	0	0.0553	0.3845	0
	0.0836	0.0801	0	0	0	0	0.0651	
0.123	0	0	0.0185	0	0	0	0	0
	0	0.2596	0	0	2.8302	0.7814	0	0
	0.9324	0	0	0	0	0	0	0
	0.8661	0	0	1.5566	0.1145	0	0	0
	0.0542	0.1353	0	0	0	0.3291	0	0
	0	0.347	0	0	0	0	0	0
	0	0	0	0	0.6416	0	0.1143	0
	0							
CACNG7	0	0	0.1764	0	0.546	0.0612	0	0
	0	0	0.0127	0.4384	2.9965	0	0	
0.1025	1.3266	0	0	0	0	0.0084	0.3911	0
	1.2765	0.3726	0	0	1.2422	0	1.6416	
0.0801	0	0	4.00E-04	0.0252	0.0107	0	0.3263	0
	0	0.0028	0.5153	0	0.434	0	0	
0.0404	1.4939	0	0.1356	0	0	0.3518	0.334	0
	0	0.116	0	0.0765	0.0747	0.0343	0	0
	0	1.4347	0.1728	0.3521	0.0328	0.2981	0.0395	0
	0.1762	0.8269	0	0	0	0.045	0	0
	0	0.0759	0	0.0465	3.046	0	0.999	0
	0							
CACNG8	0.0288	0.0154	0.0252	0.0335	0.8158	0.0543	0	
0.0197	0.045	0.0533	0.0099	0.1974	0.1342	0.0544	0.0115	
0.0198	1.6869	0.0817	0.081	0.0686	0.0227	0.0219	0.295	
0.0707	0.0557	0.0383	0.0508	0.0621	0.1006	0.0226	0.1821	
0.0541	0.0137	0.0113	0.092	0.04	0.0118	0.0778	0.0211	

0.0374	0.0109	0.3304	0.13	0.0631	0.609	0.2349	0.0328
0.0176	0.7045	0.0599	0.0316	0.0293	0.0384	0.1654	0.3731
0.5023	0.7751	0.0403	0.0063	0.0481	0.199	0.0198	0.059
0.0061	0.0601	0.1451	0.0609	0.2235	0	0.0593	0
0.0181	0.0375	0.4075	0.0063	0.056	0	0.0037	0.0158
0.0161	0	0.0752	0	1.00E-04	0.4483	0.0358	0.0981
0.0378	0.0374						
CACTIN	2.8178	2.608	3.4426	2.0631	2.7424	2.3672	2.6227
2.9587	2.155	2.2628	2.7173	1.2938	2.8337	2.2745	3.0943
2.3064	3.1147	1.6774	2.2881	3.086	3.135	2.9824	2.5413
2.6684	2.5065	2.9753	2.4467	2.5396	2.6232	2.3236	3.1047
2.9127	3.1002	2.3763	2.2982	1.6826	2.9322	2.8253	3.2324
1.8867	2.0255	2.0642	1.8638	2.1116	2.2904	2.038	1.9756
2.3349	3.2245	2.111	1.9166	2.1311	1.8075	2.45	3.0563
3.5442	2.9775	3.4282	2.4174	3.3226	3.0608	2.3961	2.2032
2.375	2.4866	2.953	2.7545	2.8044	2.9403	2.9697	3.1266
3.1637	2.7939	1.9567	2.0874	2.4562	2.8047	2.1025	2.4268
2.6173	2.4604	2.7121	2.7705	2.5172	3.7037	2.7342	2.0891
2.6687	2.2791						
CACUL1	3.1646	4.0638	4.0731	3.8393	3.5824	3.6851	3.385
3.372	3.759	3.8792	3.1741	4.0852	3.4372	3.9291	3.134
3.3666	3.848	3.6756	3.5325	3.4078	3.556	2.5817	2.977
3.1483	2.8083	2.2485	3.3074	4.4505	2.6877	3.2579	3.8668
4.0921	3.0555	3.4631	2.9511	4.2784	3.597	3.0521	2.9796
2.6942	3.4166	3.6765	3.6099	3.7349	3.655	2.8589	3.8113
3.7776	3.4975	4.4029	3.896	3.0631	4.1686	3.1622	4.4946
3.0406	2.9529	3.7387	3.5543	3.2648	2.823	3.2298	3.4733
3.4608	3.9644	3.799	3.8232	3.8039	3.7367	3.1008	3.3908
3.4986	3.3892	3.5832	2.9546	3.8468	4.4938	3.718	2.949
2.5323	3.2713	4.0791	3.4749	3.1451	2.5518	3.8439	3.642
3.456	3.7375						
CACYBP	5.222	5.9959	5.6537	6.9748	6.4717	5.326	5.2559
4.986	5.0769	5.5385	4.6968	5.6106	4.3305	5.5012	5.922
4.9208	5.1027	5.8821	5.8977	5.4148	5.7928	5.7112	4.9004
6.3504	5.9756	4.701	5.7449	5.7112	5.4154	5.3499	5.5399
5.8955	5.5043	4.0641	4.6993	6.022	5.6529	4.6948	4.7721
5.437	5.6448	5.1479	6.002	4.9897	5.6224	5.7711	6.3712
5.5225	3.769	6.5024	5.4588	6.2253	5.7527	4.0674	6.2835
5.7876	5.8486	6.1122	6.1447	6.1743	6.0202	5.7684	5.6092
5.9076	6.673	3.855	5.6234	4.4966	6.0303	5.6651	5.1699
5.7731	5.4353	5.0756	5.7784	5.4723	5.9343	5.7146	5.3486
5.1624	5.0463	4.6589	4.7538	5.2581	5.7395	5.9254	4.8903
5.2479	5.1009						
CAD	4.9707	4.6396	3.975	3.9386	4.6183	4.0132	2.9532
3.9861	4.7785	4.3734	4.4592	4.3819	3.7555	4.2031	4.4194
3.9267	4.9755	4.3722	4.0761	3.4591	4.3369	3.6841	4.0816
4.5106	3.9302	3.8333	2.859	4.2148	4.8582	4.0094	4.6449
4.1387	4.8001	4.0514	3.8878	4.1383	4.3287	3.7114	3.9055
5.0691	3.4991	3.7389	4.7403	4.9339	4.4317	3.9569	4.5018
4.5301	4.4164	4.2486	4.0846	4.1089	3.8087	4.6411	4.2993

4.619	3.968	4.6415	3.6879	4.808	4.4369	4.3728	4.1877	
4.3525	5.1316	3.1833	4.6663	4.199	4.8535	4.3728	4.068	
4.2163	4.2487	3.837	3.6112	2.9682	4.3408	3.3534	4.8436	
4.1686	4.5881	3.6174	3.6319	3.924	3.9513	3.9753	3.3703	
3.995	3.6047							
CADM1	2.8418	4.6237	5.3579	5.0834	4.7334	5.4235	7.2608	
3.454	4.4136	1.7694	3.5614	4.1035	4.1141	5.0734	6.1209	
5.3187	0.1572	3.7939	4.147	5.1246	3.9461	5.7344	6.3448	
3.9353	5.4286	6.3535	5.4607	0.6833	6.4026	5.8183	0.2317	
4.908	6.0947	0.5076	4.0323	2.5472	3.8739	3.1368	4.5995	
4.4222	4.9687	2.9468	6.1517	5.4584	4.7253	3.4045	5.4474	
7.0652	5.7813	3.5217	5.9058	5.3326	2.8935	5.0114	0	
6.126	2.6981	4.8679	3.961	4.8792	5.0604	3.9266	4.4021	
5.5294	0.3977	5.8108	0.2615	6.8522	5.3677	4.6837	4.8325	
5.1886	5.432	5.853	7.1098	5.0044	2.9315	4.5875	6.0599	
3.435	4.1305	5.8576	5.3614	5.667	4.5457	2.4174	4.7165	
3.4037	3.9497							
CADM2	0	0.0133	0.0231	0	0.007	0	0.0067	
0.177	0.0087	0.0118	0.0634	0	0	0.0255	0.0069	0
	0.0831	0	0.0343	0.0348	0.0231	0.0326	0.0318	
0.031	0	0.0079	0.0162	0	0.0131	0.0123	3.2865	
0.0086	0.0204	0	0.0044	0.5857	0.0101	0	0.0143	
0.0107	0.0159	0.0044	0.0297	0.0923	0.0248	0.0761	0	
0.0158	0.0097	0	0	0.0172	0.0285	0	2.124	0
	0.0051	0.0929	0.0089	0.0911	0.2092	0.0303	0	0
	0.0288	0.0132	0	0	0.0109	0.0331	0	0
	0.121	0	0.5645	0	0	0	0	0
	0	0	0	0.0061	0.0147	0	0.1061	
0.0219	0.0272							
CADM3	0.7499	0.1065	0	0	0.1613	3.4201	0	
0.2141	0.7812	0.4387	0.5378	2.9326	0.2312	3.2816	4.0257	
5.5769	0.1231	0	1.588	0.2296	0.2007	6.8662	6.9088	
0.9994	1.5803	1.0977	3.2243	0	6.4988	0	0	
0.1077	0.4383	2.3631	0.3538	0.5988	0.1281	0	0.2103	
0.1035	0.1443	0.36	5.5006	4.0253	0.3357	0	0.8639	
1.5873	1.8011	0.2884	0.6185	6.8259	0.4734	0	0	0
	0	0.0158	0	0.0091	4.8129	0	0	
2.7464	0	2.1586	0	0	0	2.4257	5.4529	
3.8723	1.4616	4.3381	6.3397	0	0	2.2524	0	
0.0414	0	5.6951	4.9564	4.9413	2.5972	0.2264	1.0523	
0.8733	0.6485							
CADM4	5.5272	4.0768	3.8877	3.6153	6.5418	1.9605	4.2416	
4.7876	5.7384	4.0006	3.5868	4.9874	2.4358	4.0636	7.9753	
5.54	0.2591	3.6799	2.8901	6.5216	5.0002	7.7471	7.4876	
5.1056	7.3598	0.998	6.9637	2.6108	7.7643	4.4753	3.6668	
4.49	1.8399	6.3546	7.1002	2.8779	5.1695	4.4553	6.5806	
5.094	6.0083	3.1242	5.2668	2.4414	4.0493	0.7569	3.7423	
4.4553	5.8092	0.7548	5.0788	6.6246	5.0444	5.111	2.701	
4.0352	1.332	5.854	5.4243	6.7473	5.5629	4.814	6.1404	
1.5262	2.9729	2.6981	4.9636	3.8608	2.9069	7.222	2.9429	

6.4007	1.3231	7.5591	6.8253	5.0617	1.7533	6.5618	2.9371	
4.937	3.4334	6.4335	5.0124	8.0415	6.3116	5.7216	3.5585	
3.5437	3.5517							
CADPS	0.5403	0.1967	2.1944	0.1187	0.0267	2.1456	3.6701	
2.5706	0	0.8254	0.0474	0.1115	1.133	0.2537	0.9555	
3.9882	2.8854	2.5213	0.2029	0.0844	0.9768	0.47	1.6007	
3.399	0	0.4952	0	1.749	1.6032	0.0761	0	
1.0429	3.2693	0.0644	2.8167	0.2925	1.4993	0.7884	1.5242	
2.2498	0.0799	2.1003	1.3096	0.1726	2.3521	0.1198	2.0839	
0.4072	0.0416	0.2254	2.299	0.0652	0	0.8523	0	
0.0428	0.0172	0	0	0	1.1365	0.2118	0.5731	
1.0117	0	0.0393	8.00E-04	4.4892	2.7786	2.1948	2.5219	
1.0001	0.2993	0.2868	2.0251	0.6866	1.9951	3.2699	1.9081	
0.8628	1.8587	0.3167	3.1408	0.7866	0.5926	0	0.5752	
0.2026	0.1632							
CADPS2	0	1.2352	1.9302	2.5949	1.7788	2.3597	1.0265	
1.6928	2.5759	1.708	2.0422	2.285	0	2.6756	0.0111	
3.5548	0	2.9531	2.527	3.0649	1.9306	0.4574	2.0695	
2.0667	0	0.9323	3.2045	2.1888	0	1.1583	0	
3.1536	2.2336	2.7409	2.7267	1.9318	1.8056	2.9967	1.4656	
1.9707	0.8657	3.5067	1.4483	3.817	0.4557	1.467	2.7895	
2.9255	1.0367	1.6281	0.0427	2.4396	2.2098	0.3563	0.402	
1.9021	1.5192	4.2418	0.2854	4.029	1.7394	0.2673	2.3576	
3.294	1.6123	1.8057	0.322	0.9175	0.3098	1.7713	2.387	
1.2577	2.859	0.2316	2.7144	2.4106	0.5828	3.2423	3.0354	
3.1113	2.1035	2.5815	0.1937	2.3486	0.2278	1.7551	1.9752	
1.8002	1.1479							
CAGE1	0	0.0173	0.0279	0.0496	0.018	0	0.0098	
0.0174	0.0335	0.04	0.0104	1.0082	0.0164	0	0	0
	0.0605	0.0322	0.0641	0.0706	0.0734	0.0325	0.0208	
0.0415	0.0185	0.0108	0	0.0607	0.0302	0.0151	0.0103	
0.0381	0.0289	0.021	0.0071	0.0286	0.0326	0.0229	0	
0.0316	0.0224	0.0167	0.0235	0	0.0196	0.1568	0	
0.0186	0.0132	0.0659	0.0135	0.0724	0.0968	0.0544	0.0048	
0.0465	0	0.167	0.1093	0.0014	0.0836	0.0106	0.0213	0
	0.0081	0	0.1138	0	0.0282	0.0664	0	
0.0164	0	0	0	0.0043	0.0955	0.0821	0.0298	
0.1308	0.15	0.0877	0.0104	0.0012	0.0144	0.034	0.022	
0.0293	0.0322							
CALB1	0	0	0.5939	0	0.5825	0.0904	0.0386	0
	0	1.0002	0.2258	0.1837	0	0	0	0
	0.1326	0.0822	0	0	0	0.2989	0.0124	
0.832	0	0	0.03	0	0	0	0.2245	
0.9768	0.7075	0	0.2618	0	0.0135	0	0	
0.4799	0	1.3903	0.4154	0	0.0554	0	0.0736	0
	0.0332	0.1472	0.0571	0.6114	0.1056	0.0172	0	
0.3843	0	0	0	1.2066	0.1176	0	0.2267	0
	0.045	0	0	0.0152	0.0134	0.038	0.0104	0
	0	0.1429	0.0165	0.0262	0.007	0	3.1216	
0.3106	1.2877	0.0077	0.3183	0.0793	0	0.1418	0.9216	0

	0							
CALB2	0.1881	0	0.1706	0	2.0779	0	0.4022	
0.4533	0.0806	0	0.0676	2.6885	3.0699	0.3816	2.7587	
3.6336	1.354	0.1781	0	0.8643	0.2662	0.1839	1.162	
1.6243	0.0683	1.2453	0.3964	0	0.1765	0	0.4472	
2.6332	0.1776	0.133	0.2316	0.1299	0.3131	0	0.3439	
0.2881	0	1.7094	2.2616	0.1414	4.6819	0	0.1058	
0.1433	0.3312	0.2596	0.1962	0.131	0	0	0.1662	0
	1.7746	0	0.0121	0	0.3845	1.319	0.0281	
0.5726	0.0242	3.8926	0	0.8693	0	2.9541	2.219	
0.1806	0	0.3339	0.042	0	0	2.4303	0.0831	0
	0.0331	0.5232	2.6145	0.1432	0.7901	0	0	0
	0.1115							
CALCA	0	0	0	0	0	0	0	0
	0	0	0	0	0	0	0	0
	0	0	0	0	0	0	0	0
	0	0.0169	0.0271	0	0	0	0	0
	0	0.0524	0.0178	0	1.1152	0	0.1419	0
	0	0	0	0	0	0	0.0704	0
	0	0	0	0	0	0	0	0
	0	0.128	0	0.1199	0	0	0	0
	0	0	0	0	0	0	0	0
	0	0	0	0	0	0	0	0
	0	0	0	0	0	0	0	0
	0							
CALCB	0	0	0	0	0	0	0.4108	
0.3665	0	0	0	0.0718	0	0	0.0529	
0.1633	0	0	0	0	0	0	0	0
	0.0062	0.0112	1.1721	0.0468	0	0.5219	0	
0.0421	0	0	0	0	1.7241	0.2329	0.3973	0
	0	0	0.1863	0.0589	0	0	0	
0.2518	0	0	0	0	0	0.0481	0	0
	0.2845	1.7547	0	0	0	0.1138	0.1153	0
	0.1424	0	0	0.2203	0.2542	0	0	0
	0	0.045	0.0969	0	0	0	0.1468	0
	0	0	0.0534	0	0	0	0.0661	
0.1344	0							
CALCOCO1	2.9892	3.0362	2.6356	2.6798	3.6034	2.8679	3.9034	
2.5596	2.9186	3.4431	3.9636	3.3442	3.2566	3.3622	2.7664	
3.5451	4.236	3.4639	2.56	3.2373	2.8502	2.4825	3.8711	
2.1459	3.1875	4.0712	5.1139	3.4531	3.0576	3.8847	4.2626	
3.024	3.2051	3.4635	3.7862	2.947	4.4984	4.5315	3.663	
3.6985	4.4029	5.0637	1.539	3.4815	3.115	3.477	2.6802	
3.5761	4.7684	3.6998	3.5442	3.2336	4.1119	3.8089	2.7831	
2.4288	4.5921	1.8467	2.5317	1.8944	3.4218	3.2387	2.6855	
3.8208	3.136	5.1805	3.575	4.8073	3.1461	3.3677	3.7605	
3.3968	2.74	4.2562	3.8585	2.6976	3.2567	3.9887	3.8797	
3.5297	3.5815	4.0976	4.4601	3.5207	2.7831	3.3319	4.5572	
3.4286	4.6352							
CALCOCO2	4.7123	3.8428	3.7862	4.6076	4.482	4.3843	4.5505	

3.9895	4.2787	4.3656	4.5137	4.7739	6.0718	4.837	4.1427	
4.3924	5.0077	3.8109	3.5808	4.8352	3.7862	3.9973	4.0807	
4.3458	3.7622	5.1645	5.7089	4.819	4.3212	4.2449	4.39	
4.0907	4.1539	4.7465	4.5019	4.3385	5.9194	4.5515	4.3358	
4.9897	4.1523	4.8268	4.7806	3.7069	4.6595	4.82	4.4198	
4.5627	4.2971	5.0597	4.9585	4.1742	4.1623	5.1545	4.6393	
4.2095	4.9026	3.4919	3.9235	3.4042	4.6462	3.945	2.7172	
4.051	4.0877	5.2887	4.999	4.5997	4.149	4.3745	4.5082	
4.372	5.1152	4.4471	4.6846	4.8241	4.1795	4.3006	4.1932	
4.312	4.7332	4.1043	4.7034	4.123	3.9512	4.0255	4.1511	
3.6207	4.221							
CALCR	0.059	0.4976	0.1383	0.0275	0.0035	0	0.0525	
0.0665	0.0874	0	0.0718	0.0036	0	0	0.0711	
0.0061	0.1209	0	0.4353	0	0.0858	0.0444	0	
0.0451	0.068	0.0188	0	0.518	0.014	0.2891	0.2183	
0.006	0.0173	0	0.0053	0.0358	0.1192	0.1838	0.0318	0
	0.1713	0	0.0243	0.0158	0	0.013	0.1722	
0.0356	0	0.0476	0	0.1137	0.0347	0.0176	0.2397	0
	0.0463	0	0.3473	0.0182	0.1022	0.0255	0	
0.0778	0	0.0012	0.064	0	0.1116	0	0	
0.1408	0.2465	0.0029	0	0	0.4626	0	0.0043	
0.2027	0.0103	0	0	0.0182	0	0.0396	0.023	
0.0279	0							
CALCRL	0.2593	0.1755	0.02	0.1551	0.1757	0.2928	0.0945	
0.0276	0.0118	0.0674	0.0441	0.2877	0.125	0.2219	0.1154	
0.0528	0.8134	0.0254	0.1558	0.024	0.1288	0.24	0.4543	
0.0737	0.0496	0.3175	0.3288	0.7612	0.1598	0.02	1.0744	
0.3602	0.2122	0.0302	0.1602	0.3207	0.4021	1.3066	0.1732	
0.3017	0.0933	0.0949	0.0995	0.1813	0.0836	1.4851	0.2603	
0.249	0.3833	0.5387	0.1181	0.3921	0.7439	0.1946	0.5848	
0.195	0.91	0.0249	0.0701	0.0948	0.2729	0.1108	0.0261	
0.0274	0.053	0.3712	0.9234	0.0685	0.147	0.046	0.6396	
0.0601	0.8709	0.1298	0.0791	0.2716	0.3956	0.1379	0.2697	
0.066	0.1564	0.3106	0.1119	0.0586	0.0574	0.5519	0.0562	
0.2259	0.2668							
CALD1	6.1007	6.5851	5.1051	5.812	5.1128	7.4308	7.1557	
5.6064	6.2279	6.161	6.7778	5.5899	6.754	6.7259	6.8169	
7.5687	5.5321	7.7877	6.5319	5.9952	5.3475	6.5204	7.05	
6.2435	7.2454	8.0429	5.476	6.0902	7.7612	6.7924	4.9658	
5.3858	6.5093	6.3938	7.9012	6.1539	5.2108	5.3475	4.7313	
6.5554	6.3752	6.2423	6.7197	6.6561	6.6286	6.5442	6.353	
6.0624	7.4934	5.2942	6.4969	7.4536	6.9905	6.3613	4.9064	
5.7026	5.207	4.5731	7.2413	4.2327	6.0091	7.07	6.0102	
7.1749	6.7768	6.9515	5.9128	7.5624	6.1982	6.6984	7.0611	
6.8375	6.8359	6.1082	7.2069	5.9525	5.419	7.1704	5.7797	
5.9988	5.6583	6.9167	6.0358	6.2598	7.083	6.7293	6.7474	
6.1217	6.0717							
CALHM1	0	0	0	0.0612	0.0596	0.0554	0	
0.009	0.0524	0.0454	0	0.0025	0.0796	0.1036	0.0048	
0.0048	0.0651	0.0275	0	0	0	0.0121	0.0273	

0.0435	0.0148	0.0083	0	0	0.0137	0	0	0
	0.0165	0	0.0046	0.0095	0.0134	0	0	
0.0144	0.0251	0.0996	0	0.016	0.0344	0.151	0	
0.081	0.0413	0.1006	0	0	0	0.0137	0.0289	0
	0.0501	0	0.0715	0.0238	0	0.0135	0.0365	
0.0318	0	0.0062	0	0	0.0133	0	0	0
	0	0	0.0182	0.1943	0.0093	0	0.0097	0
	0	0.0122	0.015	0	0.0601	0	0	
0.0461	0.0536							
CALHM2	2.324	1.6416	0.5828	0.995	4.1937	3.16	1.6369	
0.6205	1.2521	2.6569	0.7874	0.5877	4.5294	1.8979	1.4432	
2.2336	0.9629	2.7567	0.4424	1.4586	1.5533	0.5356	1.9466	
1.2818	3.5723	1.2127	2.7586	0.6652	2.2126	2.2764	0.199	
1.5824	2.4373	3.3529	2.7457	1.8072	2.8463	0.709	2.1074	
1.7434	2.4625	3.9183	0.3628	2.4981	3.0485	4.1235	1.976	
2.4857	1.0046	2.378	0.8178	2.0172	0.6444	1.2579	1.1805	
0.7251	3.5198	2.9075	2.9576	2.9676	3.5668	2.1351	2.7091	
2.412	1.2384	0.8213	0.7391	0.6309	2.7055	1.3466	0.1978	
1.0703	0.3369	1.5874	2.452	3.9555	1.2952	0.7934	2.1654	
0.4491	0.8076	3.1719	1.1882	2.6133	3.4918	1.0417	2.9485	
3.006	3.026							
CALHM3	0	0	0	0.106	0.5713	0	0	
0.0416	0	0	0	0.0964	0.1654	0	0	0
	0	0	0.1626	0.5829	0	0.0606	0	0
	0	0	0	0	0.041	0	0	
0.0303	0	0.055	0	0	0	0	0	0
	0	0.5387	0	0	0	0.2896	0	0
	0	0	0	0	0	0	0	0
	0	0	0	0	0.0398	0.0604	0	0
	0	0	0	0	0	0	0	
0.034	0	0.0781	0	0	0	0.0395	0	0
	0	0	0.1069	0	0	0	0	0
	0							
CALM1	6.2205	5.9924	5.9176	6.1429	5.5026	6.2863	5.913	
6.0512	5.5551	6.3114	5.3659	6.1813	5.571	6.3965	7.1016	
6.6206	5.4802	7.2444	6.7583	5.354	6.6694	5.6571	6.579	
5.3633	5.9493	5.6683	3.7141	6.4814	6.6814	5.5813	6.0111	
6.3713	6.2772	5.6563	5.8558	6.7717	6.1884	5.5134	5.7298	
6.072	5.7282	5.5019	6.5073	6.1803	6.6055	6.1109	6.0404	
5.8412	5.7128	6.7775	5.5217	5.8819	6.3323	5.5767	5.4436	
5.0778	6.439	5.8593	7.7103	5.9484	5.6292	5.3209	6.4448	
6.7353	6.2778	5.8759	6.3761	6.354	5.7945	6.0266	6.0007	
6.6258	5.91	6.1198	7.2066	5.8153	6.1876	5.6685	6.6968	
5.2983	7.0897	6.0509	6.3009	6.1064	5.8153	6.4888	6.9997	
7.4557	7.0415							
CALM2	8.6245	8.3419	8.4454	8.9674	8.702	9.1732	7.891	
8.5549	9.5815	8.9336	7.8655	8.9674	9.6081	9.015	9.2099	
9.1127	8.3791	9.5082	8.9779	8.6159	9.1377	7.8374	9.4472	
9.1377	8.5365	9.1377	7.6548	8.2528	9.1859	8.2352	8.0164	
8.6636	8.9779	8.3564	9.2706	9.1	8.4696	8.276	8.2935	

9.039	7.8607	8.8988	9.7996	9.3127	9.2464	9.4191	8.5549	
8.8002	9.3916	9.0517	8.6636	8.7883	8.8988	8.2325	8.3015	
8.3528	9.3195	9.0358	9.0855	9.3035	8.9859	8.8929	8.7275	
8.7534	8.4128	8.6067	8.6311	8.6067	9.1498	9.4732	8.265	
8.9385	8.0471	8.8334	9.5081	7.9276	8.9224	9.2002	8.5337	
8.5071	8.5961	8.7275	7.9504	7.9354	9.5251	9.1314	8.1266	
8.3211	8.0697							
CALM3	6.1454	6.9567	6.8038	6.2612	6.5645	6.4638	6.3878	
6.6855	7.0151	6.8805	6.0131	6.6614	6.6783	5.8478	7.1647	
6.7681	7.3845	6.2926	6.108	6.3907	6.9088	6.382	6.5937	
6.2283	6.9975	6.6225	6.3977	7.6359	6.4726	7.2719	8.1878	
5.867	7.3395	7.5689	7.1623	6.5211	5.8016	7.1961	7.0676	
6.6783	7.3661	7.8175	7.354	5.9779	7.7435	7.0428	6.0504	
6.59	6.0461	6.6067	6.6294	6.2708	6.4853	7.1629	7.3696	
7.1053	7.1629	6.8583	5.8196	6.8655	6.5293	5.8844	7.3667	
7.108	7.1022	6.8448	5.7487	7.3636	7.0434	6.3717	7.4316	
7.0249	6.3794	6.8264	6.7952	7.4456	6.4321	6.1192	7.2958	
7.8559	7.5015	6.2152	5.374	6.6518	7.6874	6.7507	6.8913	
7.0865	7.5911							
CALML3	0	0	0	0	0	0	0	0
	0	0	0	0	0	0	0	0
	0	0	0	0	0	0	0	0
	0.0424	0	0	0	0	0	0	0
	0	0	0	0	0	0	0	0
	0.0537	0	0.1124	0	0	0	0	0
	0	0	0	0	0	0	0	0
	0	0	0.0431	0	0.074	0	0	0
	0	0.0083	0	0	0	0	0	
0.0278	0.0358	0	0	0	0	0	0.044	
0.0608	0	0	0	0	0.0526	0	0	0
	0							
CALML4	1.7007	2.4215	2.1961	1.6832	1.0973	1.4744	0.9138	
1.4182	1.6591	1.29	1.6136	0.6828	0.9592	1.8632	1.2427	
0.9288	2.012	1.8385	1.7002	1.5163	2.1424	1.0973	0.7568	
1.7588	1.2004	0.8116	1.5112	1.8847	1.0399	1.4836	0.8414	
1.069	1.0586	0.915	1.1454	1.3969	0.9887	1.0009	1.1677	
1.1576	0.9972	0.6701	1.2705	1.6329	2.0222	1.4749	1.9995	
1.5087	1.1053	2.141	1.5018	1.1653	1.7233	1.9114	1.2474	
1.7555	1.5001	1.0408	1.4995	1.29	1.2073	1.246	1.5156	
1.8729	1.3485	0.8038	1.5387	0.951	2.4762	1.1848	0.7133	
1.6299	1.2565	0.6574	1.0442	1.1525	2.2603	1.1551	1.4893	
2.0516	1.4931	0.8148	1.0358	0.7518	1.7517	1.6776	1.1415	
1.4698	1.4673							
CALML5	0	0	0	0	0.2923	0	0	0
	0	0	0	0.1439	0	0	0	0
	0	0	0	0	0	0	0.3026	0
	0	0.1906	0	0	0	0	0	
0.1017	0	0.265	0	0	0	0	0	0
	0.2906	0.1226	0	0	0	0	0	0
	0.1745	0	0	0	0	0.0577	0.0396	

0.2211	0.0383	0	0	0	0.657	0.1921	0	
0.1737	0	0.403	0.0666	0	0.2528	0.2148	0	
0.1784	0.0394	0.1893	0	0	0.0683	0	0.1671	
0.2933	0.1843	0.226	0	0.1662	0.0581	0.1205	0	0
	0							
CALML6	0.4141	0.0543	0	0	0.0614	0.2271	0.036	
0.2047	0.0772	0.1686	0.149	0	0.3036	0	0.0295	
0.0994	0	0.3607	0	0	0.2853	0	0.0186	
0.1146	0.053	0	0.0563	0	0.1417	0.0913	0.5702	
0.055	0.0977	0.2291	0.1481	0.0383	0.1838	0	0.0344	
0.1011	0.0727	0.0862	0.3873	0	0.0696	0	0	
0.1465	0.0745	0.1371	0	0	0	0.1019	0.2075	
0.2348	0	0.0508	0	0	0	0.0882	0	
0.1939	0	0.3024	0.4874	0.2128	0.3595	0.1617	0	
0.1013	0.0496	0	0.2008	0	0	0.0441	0	
0.1725	0.0933	0	0.0318	0	0.0641	0.2086	0	
0.1552	0.1697							
CALN1	0.0862	0	0	0	0.0199	0.0484	0.0446	
0.0226	0.0263	0.0272	0	0	0.0263	0.0709	0.0195	
0.0372	0.0509	0.079	0.0748	0.0521	0.0516	0.0172	0.3985	
0.2156	0.0199	0.0251	0.0434	0	0.0302	0.0316	0.0212	
0.1687	0	0.0363	0.0309	0.024	0.0177	0.0431	0.034	
0.0311	0.2911	0	0.0458	0	0.0224	0	0	
0.0375	0.026	0.079	0	0	0	6.00E-04	0.0024	
0.7123	0.0027	0.8092	0.0021	0.0767	1.0192	0	0.0518	0
	0.0088	0	0	0	0	0	0	0
	0	0	0	2.00E-04	0.0035	0	0	0
	0	0	0	0.0116	0	0.0168	0.0436	
0.0833	0.0589							
CALR	7.5873	9.0247	8.9439	8.9913	8.9206	9.3778	9.7841	
8.5141	9.4751	9.6814	8.3755	9.0371	8.1881	7.8643	8.6377	
9.4186	8.0763	8.2063	8.7619	8.3134	8.3413	10.2667	9.8469	
8.7222	9.6814	9.1739	8.6294	8.4331	9.3778	7.9984	10.2434	
8.183	9.1247	9.6679	9.4073	9.2129	7.873	8.6729	7.8408	
9.447	8.3978	9.461	10.0896	8.3679	9.5791	8.5827	9.0247	
8.3543	8.8635	8.4134	10.2871	9.1122	8.1404	8.6633	9.7628	
9.2162	7.6855	9.2162	9.0065	9.3937	9.3137	8.6768	8.3006	
9.201	8.525	10.0889	8.7408	8.0891	8.7143	8.5366	9.9198	
9.3937	8.265	9.7059	10.0515	8.211	9.4123	9.4462	8.7565	
8.8698	8.172	9.2334	7.6079	9.071	9.3442	8.3427	8.4553	
8.8526	9.1878							
CALR3	0	0.5751	0.0236	0.0577	0.1274	0.0582	0.2205	
0.008	0.2314	0.0698	0.2379	0.0521	0.078	0.072	0.0217	
0.1615	0	0	0.2142	0	0.0614	0.0598	0.0057	
0.2026	0.0208	0	0	0	0.0333	0.1575	0.2649	
0.0106	0.1836	0.1475	0.0047	0.0732	0.1102	0	0	
0.0628	0.0697	0.0295	0.0536	0.0419	0.0237	0	0.3239	
0.059	0	0.1816	0.0658	0.2169	0.122	0.1267	0.3121	
0.1793	0	0.0389	0.3214	0.0374	0	0.2555	0	0
	0.2089	0.0282	0.1378	0.0538	0.1535	0.0691	0.0633	0

	0.035	0	0.2201	0	0.2402	0.0311	0	
0.1375	0.028	0.0338	0.0602	0.074	0	0	0	0
	0							
CALU	7.3486	6.9266	7.7814	7.6284	6.6959	9.4876	8.6763	
9.0098	8.7319	8.0746	8.4975	7.832	7.2982	8.2734	8.5358	
9.8442	6.023	9.2817	8.4625	7.856	8.0151	8.6671	8.4885	
8.2526	8.0684	8.2016	7.6768	6.5681	8.9715	7.2929	7.0755	
7.4874	8.2393	8.8852	10.0368	7.9296	7.193	7.0076	7.6318	
8.1524	7.5037	7.2681	8.8598	8.4542	8.0046	7.8076	8.0684	
7.9676	8.1601	6.1465	8.7319	8.2016	7.6524	7.3205	6.6261	
7.307	6.6499	7.4883	8.2982	7.6733	7.9699	8.091	7.8285	
9.485	6.7651	7.4969	8.2908	7.4169	7.3421	8.2564	8.4609	
9.126	8.5515	8.3499	8.1482	7.9964	7.1204	8.7089	7.6182	
8.2081	7.0661	7.5542	8.4759	10.0442	7.7202	7.2098	7.7176	
7.43	7.5001							
CALY	0	0	0	0	0	0	0	0
	0	0	0	0	0.1035	0	0	
0.1125	0	0	0	0	0	0	0	0
	0	0	0.2139	0	0	0	0.4381	0
	0	0	0	0	0	0	0	0
	0	0.0663	0	0	0	0	0	0
	0.1096	0	0	0	0	0	0.5499	0
	0	0.1197	0	0	0	0.0782	0.119	0
	0	0	0	0.0714	0.121	0	0	
0.0365	0	0	0	0	0.0675	0	0	0
	0.0398	0	0.0319	0	0	0	0	0
	0							
CAMK1	3.0541	2.8	1.8774	2.9681	1.2256	0.5595	2.7044	
1.3792	0.1839	2.7391	2.5886	3.7892	2.0063	2.3249	2.5328	
0.838	1.1937	2.5551	0.6286	0.2888	2.2333	1.3903	3.0918	
2.4807	2.2003	1.0323	3.0909	0.4091	2.8381	3.5267	1.6674	
1.3961	0.3734	2.0491	0.4991	2.3087	3.1785	0.5367	2.6562	
1.9756	3.4343	3.6961	3.0603	1.9699	4.4411	1.1715	2.2825	
1.775	2.4923	1.9717	1.4202	2.7151	1.8814	1.0316	0.8852	0
	3.2423	3.1123	2.4496	2.242	2.0384	0.5924	3.1782	
1.1096	0.8624	3.8324	1.0188	2.7016	3.1598	0.7384	2.0389	
2.2508	0.8546	2.4336	2.4711	1.4959	1.2867	2.9882	2.0628	
0.7183	0.7174	3.2767	2.6318	2.4302	3.4108	2.8805	2.2033	
1.7539	1.9743							
CAMK1D	0.0621	0	1.2953	0	0.0753	0.174	0.7918	
0.0495	1.2155	0.0359	0.0688	0.0183	2.7231	0.0591	0.2221	
0.0951	0.3557	0.0489	0	1.8318	0.3362	0.0539	0.1453	
0.2012	1.2104	1.6606	0.1004	0	0.1517	0.0405	2.4795	
0.0389	0.0707	0.0633	0.3914	0.6678	0.0153	0.2093	0.0703	
0.2776	0	0	2.5128	0.0686	0.3168	1.8901	0.1211	
0.1084	0.1311	2.5614	0.2224	0.0437	0	0.0275	1.1712	
0.0899	1.4298	2.9709	0.0195	3.0777	0.0382	0.0627	0.2893	
0.1884	0.445	2.8493	0.1324	0.5348	0.1634	0.6098	0	
0.0358	0.9718	0.0403	0.0497	0.4584	0.0949	0.141	0.0685	
0.1036	0.034	0.0362	0.114	0.4837	0.0348	0	0.2481	

0.5378	0.66							
CAMK1G	0.1309	1.0494	0.0539	0.1711	0.0707	0.0621	0.3808	
0.0307	0.0995	0.1965	0.0875	0.4244	0.0425	0	0	
0.1173	0.1605	0.7878	0.5485	0.1172	0.2503	0.0432	0	
0.0585	0	1.56	0.0852	0	0	1.9108	0	
0.3333	0.1664	0.0585	0.02	0.1479	0.0259	0.428	0.4032	0
	0.321	2.4512	0	0	0.0424	0	0	
0.178	0	0.0817	0.1599	0.0746	0	0.0134	0.0072	
0.11	0.2749	0	0.4749	0	0	1.182	0	0
	0	0.0323	1.1782	0	0.0294	0	0.6769	0
	0.1144	0.0502	0	0.0131	0.8566	1.202	0.1456	0
	0.4591	1.0656	0	0	0.0033	0.0221	0.392	
0.1215	0.2719							
CAMK2A	0.2583	0.0104	0.0688	0	0.1026	0.1775	0.1324	
0.0029	0.2026	0.2671	0.1653	0.1026	0.1072	0.4574	0.0726	
0.0604	0	0.8325	0.0891	0.0758	0	0.045	0.243	
0.0193	0.3683	0.0521	0.0605	0.0578	0.097	0.0424	0.0161	
0.0632	0.2637	0.719	1.0526	0.04	0.1047	0.27	0	
0.0182	0.8378	0.0705	0.0328	0	0.0014	0.0763	0.1312	
0.2537	0.004	0	0.1261	0.0228	0.0878	0.0124	0.0336	
0.1634	0	0	0.1539	0	0.0506	0.1708	0.0435	0
	0.3705	0.0627	0.3106	0.016	0.1018	0.1659	0	
0.0459	0.0534	0.0391	0.0814	0.5206	0.1265	0.7704	1.0976	
0.0629	0.0669	0.0124	0.3207	0.0282	0.2964	0.0826	0.0411	
0.0694	0.558							
CAMK2B	0.1495	0.0897	0.3285	0.2669	0.1556	0.3516	0.0895	
0.1308	0.0902	0.1572	0.0861	0.0911	0.3258	0.392	0.2999	
0.1538	0.3105	0.5756	0.5714	0.3826	0.1954	0.0758	0.1982	
0.3319	0.3282	0.1261	0.1867	0.2515	0.2866	0.2013	0.5356	
0.1359	0.2765	0.2754	0.0695	0.1351	0.1472	0.4357	0.2124	
0.2022	0.3034	0.1177	2.2734	0.192	1.1392	0.0954	0.1384	
0.1158	0.1438	0.1117	0.1523	0.0581	0.4034	0.2187	0.5515	
0.7962	0.4764	0.1209	0.1821	0.1936	0.5371	0.4038	0.2604	
0.061	0.8707	0.0648	0.2745	0.242	0.0914	0.4992	0.1774	
0.2354	0.3528	0	0.0805	0.0969	0.1601	0.1829	0.0367	
0.032	0.2819	0.0717	0.3395	0.1549	0.6638	0.4291	0.277	
0.1787	0.2835							
CAMK2D	4.0575	2.5129	3.0991	3.9716	2.8787	3.6171	4.7489	
4.1872	2.8314	2.9899	2.9839	3.3934	2.8303	3.5431	4.0734	
2.8539	3.738	4.1442	3.297	3.4818	3.9218	3.3384	3.1989	
3.4205	4.1043	4.7959	4.4645	3.0126	3.0943	3.051	3.7828	
2.2137	3.6142	3.9618	3.8585	3.2548	3.7573	3.1168	2.0485	
4.4679	3.7965	4.1253	3.9058	3.6213	4.2925	3.7481	3.3663	
3.3433	3.2396	4.042	3.2063	4.2107	3.8042	3.2093	3.4018	
2.4114	3.6665	1.9575	3.1519	2.361	3.3907	3.5751	4.1042	
3.3703	3.6629	4.7975	3.6829	4.687	2.8994	3.2336	4.5354	
3.3328	3.618	3.6612	4.0903	3.8923	3.1306	3.645	2.8904	
3.6296	3.4158	4.1644	3.3738	3.8986	3.8628	3.4134	3.9457	
3.4267	3.1802							
CAMK2G	2.3797	3.1048	3.0532	3.3885	3.0532	3.3803	2.2008	

3.2554	2.9798	2.9568	2.5706	3.0438	3.4828	2.821	2.3434
2.3248	3.6721	2.7221	2.22	2.7008	3.1276	2.7085	2.7122
2.7517	2.8464	3.5231	3.4631	3.3615	1.5786	2.2501	3.0312
3.1982	2.0632	2.9405	2.7671	2.8813	2.7238	2.5914	2.1466
2.1027	3.1608	3.7701	3.5156	2.5853	3.799	3.9416	2.5423
2.953	2.7352	3.7777	2.9284	3.0658	3.2508	2.4122	3.8541
3.1775	3.9046	2.9759	1.9764	3.0785	3.0123	2.6801	2.7019
3.127	2.8853	3.6469	2.4614	2.6445	2.0083	2.9054	2.7163
2.2574	2.5158	2.9229	2.8738	2.5674	2.8212	3.2368	1.7725
3.5989	2.7968	3.2931	2.4117	2.5957	3.0932	3.4753	2.4233
2.2431	2.5543						
CAMK2N1	2.8952	0.8232	2.8721	0.2132	3.5918	3.8814	4.3351
2.0031	3.7547	1.0425	3.5567	3.7207	4.5421	3.316	4.5643
3.8377	3.8624	2.4253	2.5526	3.951	1.186	3.5799	4.6807
2.5831	3.364	5.1683	5.4506	3.0672	4.323	3.6991	0.62
1.7376	4.0603	1.9812	4.0864	1.549	4.603	3.3814	2.6197
3.8232	0.4734	4.4436	5.5055	2.9884	3.9675	3.305	2.1589
3.264	3.3818	4.3168	3.6956	3.6352	3.3491	4.1961	0.3826
2.6144	5.2734	2.3351	4.5364	2.2287	4.1969	1.843	3.0351
3.9547	3.3157	3.8795	2.0262	4.645	2.4173	4.673	1.6394
4.808	3.7217	3.9672	3.2989	0.4909	2.092	4.6358	2.8493
2.7883	3.0767	5.1927	2.6542	3.0491	4.4465	3.0522	4.0868
2.7642	3.6279						
CAMK2N2	1.9721	2.8605	2.3872	2.1496	1.7676	1.8887	1.72
1.6121	0.904	1.5417	0.7809	0.7809	3.4767	1.2016	3.1385
0.9635	3.524	2.1072	2.2408	3.1024	1.3671	1.485	3.6501
1.9885	1.7063	1.8714	0.8103	1.2792	2.3374	1.402	2.7816
1.6628	2.2344	0.635	1.5186	1.7189	1.2522	1.7894	0.9265
1.3797	0.4381	0.9677	4.1427	1.3326	2.1791	1.0149	1.1932
2.1655	1.8847	4.1884	1.2417	0.4909	0.8404	1.933	1.82
3.5458	1.5857	2.1823	0.3488	2.4446	2.4633	0.7879	1.0039
1.8794	0.8798	3.4263	2.5537	2.557	1.6089	1.5917	2.1288
1.4885	3.0948	1.2589	0.8972	1.1265	0.7568	1.5445	2.2683
3.1284	1.7771	2.0201	3.1677	0.8798	2.8164	0.3786	1.8398
1.9401	1.3461						
CAMK4	3.3827	3.1944	3.5807	2.8551	4.3833	2.1941	2.4146
1.6005	3.3197	3.4244	3.0711	1.9562	4.2752	1.2568	1.3522
3.3905	2.4402	3.2814	3.8228	4.5814	3.096	2.2231	2.6471
3.0193	2.9363	2.635	2.8782	1.9288	2.863	2.3521	0.0943
2.9948	3.4011	2.0042	2.4245	3.8339	0.7774	1.7458	1.1747
2.1453	0.7391	2.4562	3.9245	3.3337	0.5419	1.9628	1.946
3.9904	2.6078	3.5228	0.0977	3.4695	2.7618	2.9503	0
2.3062	1.3784	3.2137	3.9138	2.8431	2.5904	4.5617	3.2538
2.6357	2.1616	0.7702	1.0292	0.6854	2.3153	3.0616	0.2114
3.5319	3.5279	2.927	2.4606	3.4653	2.818	3.365	3.9254
2.1237	3.7697	1.7821	0	2.1737	3.2163	2.3319	0.1892
0.5832	0.2204						
CAMKK1	2.3624	1.8951	1.8104	0.7633	0.8325	0.8261	0.7561
1.7343	1.3768	1.8062	1.0755	0.3015	1.0029	1.309	0.8761
1.4311	2.3533	2.136	0.9343	1.2273	2.4283	0.8992	1.2701

0.9049	0.3848	0.5246	0.3408	1.272	1.3136	0.9742	1.634	
0.5312	1.102	0.9156	1.067	1.4892	0.6814	1.5849	0.5205	
0.7616	1.3742	0.8884	1.6181	0.7765	0.8568	1.4879	0.9708	
0.9562	0.5779	1.3478	1.8042	0.3088	1.3292	1.4742	1.9451	
1.6891	1.9256	0.9564	1.8135	1.3408	0.9964	2.0051	0.8815	
1.5704	1.5798	0.714	1.269	0.315	0.8392	1.3777	0.9038	
1.4246	2.2019	0.591	0.5247	0.8039	1.5804	0.6046	0.9544	
1.7953	1.6111	0.3919	0.8752	0.4617	0.5184	1.5462	1.4853	
1.7117	1.5341							
CAMKK2	3.5485	2.4565	3.5793	2.7379	2.8942	2.9352	3.3319	
3.2452	3.3758	3.2292	3.7669	2.6729	3.0763	4.1124	2.9014	
2.5573	3.7812	3.2908	3.0355	3.3357	2.981	3.1726	2.1181	
3.1499	2.8904	2.6643	3.3658	3.3961	2.5543	3.4888	3.4013	
2.779	2.8507	3.7348	3.2238	2.7301	3.566	3.1301	3.1676	
3.798	3.4261	2.8701	3.3425	3.2991	3.3579	3.5362	3.5769	
3.56	3.3998	3.387	3.6209	2.6433	3.8523	3.7293	3.2859	
3.7445	3.6555	3.4018	3.26	3.3539	3.0238	3.3432	3.1056	
3.2322	4.2005	3.1459	3.2559	4.0304	2.8941	2.7049	3.1691	
2.8075	3.3038	2.0982	2.3758	3.4937	3.253	3.1464	2.4167	
3.4411	3.1505	2.7117	2.849	3.0873	2.7328	3.4898	3.7801	
3.5525	3.7396							
CAMKMT	2.0402	2.4819	2.0506	1.7168	1.8329	1.7515	1.6464	
1.9255	3.0952	2.6955	2.5191	2.548	1.564	2.5701	1.5285	
2.1249	2.4731	1.9597	2.448	2.0371	1.9927	1.7018	2.0886	
2.8168	1.3285	0.9373	2.6221	1.8756	1.9525	2.8933	2.3148	
1.607	2.4137	2.6102	1.5229	2.6253	1.7231	2.0005	1.9431	
2.2489	1.8329	2.1782	1.9525	1.6083	1.8333	1.9239	2.5159	
2.266	1.3218	2.7669	2.3222	1.7168	2.4078	2.2547	2.4152	
2.3998	2.2174	1.5959	2.2387	1.697	1.7276	1.5805	1.8044	
2.5557	2.1652	1.6734	2.6373	2.2031	2.3246	2.1658	1.771	
1.6118	1.794	2.6887	2.2802	1.5618	2.7141	2.1585	2.6212	
2.1652	2.3525	1.8357	1.8749	1.5028	1.2608	2.5493	2.2208	
2.0804	2.1741							
CAMKV	0	0.0675	0.3287	0.1685	0	0.0739	0.0785	
0.7753	0.0227	0.1149	1.6947	0.0653	0.1536	0	0.0324	
0.0458	0	0	0.0704	0	0.0671	0.1674	0	0
	0.0586	0	0.2622	0.2635	0.0907	0.1217	0.9858	
0.0134	0.217	0	0.0176	0.1723	0.1121	0.1233	0.1082	
0.1611	0	0	0.0423	0	0.0313	0	0.487	
0.0722	0.023	0	0.0265	0	0	0.5115	0.4201	
0.109	0	0.6035	0.1661	0.0382	0	0.0088	0.5785	
0.0943	1.4659	0.0042	0.4124	0	0.0696	0.1367	0.0291	
0.0845	0.2747	0.0403	0	0	0	0	0	0
	0.0347	0.0128	0.1913	0.0372	0.0133	0	0	0
	0							
CAMLG	3.9223	3.8696	3.7299	3.8092	4.4771	3.1626	4.0979	
3.8918	3.5111	3.4462	4.8936	4.2425	4.7324	3.7646	4.1457	
3.8133	4.3442	3.2444	4.1933	4.5726	3.8394	3.8712	3.6162	
3.7511	3.9877	4.0163	6.1235	4.1496	3.6933	3.9074	4.2206	
4.1701	4.0485	4.8294	3.7016	3.9661	3.0459	3.584	3.1937	

3.5881	4.0345	3.786	3.9579	3.0699	3.6341	4.0753	3.8567	
3.8159	4.3746	4.9351	4.0737	3.4502	3.7227	4.487	3.5564	
3.423	4.4967	3.2596	3.6668	3.2405	3.5997	4.1275	4.2899	
3.8562	3.972	4.7301	3.9847	4.207	3.2666	4.0659	3.6377	
4.0257	4.0107	3.7234	3.3344	4.077	4.3674	3.5948	4.1084	
4.2534	4.4653	4.2026	5.2681	3.5079	3.588	3.2075	4.4261	
3.9298	4.3394							
CAMP	0.2791	0	0.2718	0	0.131	0.2935	0.0908	0
	0	0.2429	0	0	0.079	0	0	
0.9152	0.1817	0.456	0	0	0	0.2247	0.0949	
0.1429	0.2923	0	0.1155	0	0	0	0	
0.1649	0	0	0.0574	0	0.0708	0.1165	0.0827	0
	0	0.2761	0.1374	0	0.261	0	0	0
	0	0	0	1.8848	0	0.3941	0	0
	0	0	0.1489	0	0.5475	0	0	
0.0738	0	0	0	0.1363	0	1.1078	0.1526	
0.127	0	0.0173	0.6058	0.2711	0	1.1066	0.0171	
0.2226	0.0405	0.3876	0.1979	0	0.1458	0	0	0
	0							
CAMSAP1	3.3232	2.8415	3.5183	2.8451	3.0574	3.2968	2.0999	
3.9101	2.304	2.8352	3.2161	2.4456	2.0506	2.6328	2.8671	
2.573	3.7408	2.9282	3.4757	3.0345	3.5201	2.4097	2.4292	
2.9815	3.5419	2.5443	2.9033	3.1829	2.5685	2.6836	2.7274	
3.1628	2.061	2.8219	2.2732	2.9412	2.9983	2.9408	3.7389	
2.5757	3.0349	2.5937	2.9353	2.8619	2.8757	2.9934	3.2363	
2.827	3.1327	3.5412	2.8149	3.0357	3.1524	2.298	3.2279	
3.0026	3.476	3.3067	2.7262	3.4911	2.8186	2.9486	3.1724	
2.8072	3.9216	2.7527	2.4241	3.6264	2.9281	3.4544	2.8117	
2.5547	2.6418	2.3062	2.9805	3.6236	3.1216	2.741	2.9555	
2.6488	2.8013	2.5335	3.254	3.7594	2.4446	2.8844	3.106	
3.358	3.1749							
CAMSAP2	3.7051	3.4714	4.0265	4.3232	4.4321	5.658	4.4412	
3.6627	3.8615	4.1096	3.175	3.4512	3.4205	3.9356	4.0082	
4.2821	3.8408	4.6719	3.9377	3.742	3.9588	4.0772	4.4912	
4.3273	3.5153	4.3031	3.7924	3.8192	3.9872	3.5997	4.1406	
3.8897	3.0975	3.3004	4.3078	4.0714	4.0387	3.3795	3.8615	
4.236	3.7831	3.3467	3.7192	3.9492	4.0395	3.5453	3.7781	
4.1276	4.071	3.8146	4.0542	4.7587	3.7482	3.7697	4.2227	
4.4783	3.7489	4.153	4.2874	4.4025	4.049	3.5052	4.1919	
3.9394	3.9872	3.6704	4.1489	4.2758	4.569	3.9163	4.2012	
3.9935	3.9928	3.1543	4.4375	3.5855	3.8258	4.4375	3.4917	
2.6699	3.2359	3.8972	3.7501	4.3926	3.8477	3.319	3.8905	
3.6103	3.5357							
CAMSAP3	0.5639	0.1159	0.4729	0	0	0.1142	0	0
	0	0.0419	0.1201	0.1314	0.5913	0	0	
0.1961	0	0.1803	0	0	0	0.0759	0	0
	0.0471	0.0554	0.0828	0	0.1023	0.1793	2.7092	
0.0767	0	0	0	0.3636	0.194	0.1787	0.2323	0
	0	0.0769	0	0.0724	0.5697	0	0.1838	
0.0765	0	0	0	0	0	0.2658	2.061	

0.2731	0	0.2682	0.0132	0	0	0.0276	0.05	
0.015	0.556	0.0548	1.5133	0.0654	0.4273	0.0116	0.0012	
0.0286	0.255	0.022	0	0	0	0.0017	0.1072	0
	0.0567	0	0.1774	0	0.0232	0	0.0546	0
	0							
CAMTA1	5.1137	6.5609	6.0071	5.8877	5.5938	5.8587	6.2939	
4.9075	6.799	5.8939	6.3936	5.7733	5.7299	6.0948	5.6946	
5.9314	4.7792	6.1672	6.172	5.4984	6.0795	5.9634	5.899	
5.6582	5.8505	4.9961	6.7198	6.0306	5.7815	7.062	6.4964	
5.3349	6.8635	6.261	5.7061	6.1421	5.4298	4.7618	5.6929	
6.4388	5.9995	5.2744	6.0643	5.5938	6.0398	6.3829	5.9881	
4.8642	5.9041	7.3336	6.2356	5.1462	7.0822	6.0202	6.0177	
6.7297	5.4057	5.6204	6.3951	5.9349	5.0492	5.2263	5.2875	
6.6703	6.1659	5.6806	6.3074	5.2109	5.9578	5.1026	5.8718	
5.9223	5.9087	6.0401	5.8215	6.1552	6.5948	5.3011	5.892	
7.0306	6.6407	5.5529	5.1794	6.1442	5.9395	7.2319	6.0759	
6.0158	6.09							
CAMTA2	3.7035	3.1685	3.1489	2.6882	3.6498	2.8001	3.2554	
3.1281	3.2348	3.9383	3.0616	3.0624	4.0602	2.3805	2.9619	
4.2904	3.8711	3.2017	2.2633	3.3043	4.3549	3.1274	3.0132	
3.0261	2.3222	3.7368	3.2694	3.4026	3.2747	2.3473	3.1781	
2.11	3.4467	3.6854	3.4666	2.8785	3.6065	3.961	3.7313	
2.9233	3.0877	3.7064	3.8422	3.9561	3.1372	2.5715	2.1319	
3.0291	3.8319	2.311	3.3786	2.7858	2.5059	2.9665	3.4419	
3.0987	3.9346	2.659	2.8925	2.4669	3.0457	3.7687	3.5914	
4.4672	2.7742	4.2622	3.2516	4.2698	2.3777	3.2297	3.6156	
3.546	2.6823	3.4809	3.8688	3.7474	2.6798	3.4496	2.9427	
3.5007	3.3347	3.7929	3.3892	3.0812	2.7866	2.678	4.3269	
3.6601	4.0115							
CAND1	4.9631	5.1431	6.149	5.5042	5.4252	5.4086	5.9273	
4.4502	5.5122	5.4691	4.6931	4.7126	5.2776	4.9404	4.4263	
5.3935	5.1896	5.7252	5.5356	4.5446	5.1328	5.1961	4.8332	
4.6971	5.0858	4.578	5.1231	5.3257	4.813	5.4589	5.7501	
4.8818	5.0919	4.6952	4.9815	5.7619	4.09	5.0392	4.6911	
5.4384	4.7364	5.111	5.0734	5.2506	5.8412	5.2985	5.2099	
5.6269	4.5531	5.6326	4.9182	5.4331	5.4125	4.8857	5.4338	
4.527	4.9261	5.6526	5.2859	5.4386	5.3373	5.4268	5.8068	
5.3942	5.4509	4.7929	5.5819	4.6385	5.5932	4.811	5.3595	
4.8098	4.7395	4.6646	4.7564	4.6075	5.1467	4.5912	5.4525	
5.2496	5.2582	4.8133	4.6795	5.0354	4.2973	5.4464	4.9676	
5.1579	5.1013							
CAND2	0.6443	0.3268	0.2583	1.8846	0.3374	0.9769	0.1849	
0.5958	0.083	0.6245	0.4522	1.5815	0.0256	0.3536	0.7416	
0.2246	0.5145	1.6325	0.1113	0.1639	0.105	1.1919	0.2068	
0.3626	0.758	0.6512	1.4425	0.3825	0.6267	1.4215	3.6308	
0.7896	0.1651	0	0	1.1183	0.2337	0	1.2572	
0.0829	1.6948	1.2728	0.8087	0.3914	1.9493	0.1516	1.2808	
0.0937	0.0477	0.5628	0.2651	1.3679	0.4854	0.9421	1.258	
0.3289	0.2427	2.1968	0.1902	1.5099	0.1315	0.2012	0.683	
0.3091	3.4013	1.0599	0.2205	0.1692	0.1568	0.2267	0.2252	

0.2026	0.2463	1.5033	0.857	1.237	0.5193	1.3208	0.1979
0.2762	0.2319	0.4923	0.5275	0.6283	1.494	0.3055	0.3602
0.8181	1.3801						
CANT1	4.8842	4.7997	5.2176	4.9344	4.604	4.9106	4.4772
4.7263	4.4269	4.414	4.6283	5.0496	4.9678	4.453	4.4091
5.1953	5.7473	4.2775	4.3074	6.2025	5.7086	4.5305	4.5217
4.5619	4.5695	5.5404	4.0133	5.3561	4.5351	4.3307	4.9283
5.1316	4.5203	4.7851	4.6613	4.684	5.4321	4.9122	4.084
4.1703	4.3413	4.7918	4.7548	4.0859	4.8202	4.9266	4.8097
4.5627	4.6999	4.8768	4.6207	4.5994	4.075	4.7423	5.4002
4.4984	5.4144	4.7541	4.4608	4.6626	4.6288	4.7489	4.7515
4.1523	5.0281	5.0503	5.5325	4.8185	4.2706	4.9467	4.6202
4.5256	4.7899	4.8983	5.1351	5.305	5.0794	5.0201	4.1718
4.4024	4.6907	3.8449	4.6184	5.2319	5.4772	4.192	4.1183
4.1913	4.1497						
CANX	8.3386	7.3154	8.5333	8.5333	7.5499	8.0646	8.2959
6.9854	8.2663	8.4529	6.9522	7.7223	8.4446	7.7139	8.7468
6.4226	7.8608	7.1942	9.019	8.6744	8.2466	8.5805	7.9566
8.3863	8.3863	7.9819	7.9819	8.1479	8.2894	7.377	8.8072
8.3461	8.401	8.048	9.2769	8.6744	7.9979	8.0031	7.2273
8.4806	8.1951	7.631	8.2406	7.8704	7.6565	9.4144	8.3026
8.048	8.0329	7.5009	8.7968	8.4075	7.5071	8.3188	7.8865
8.5762	7.0452	8.4404	8.351	8.3106	8.351	8.4491	8.6788
8.2617	8.1326	7.8464	8.0202	7.3954	7.8464	8.2537	7.72
8.2701	9.2366	7.8125	7.5007	8.6347	8.7489	9.7605	8.4925
7.9367	7.5007	7.9989	8.3017	7.9491	8.1048	6.8464	7.8787
7.9924	8.1252						
CAP1	7.0681	7.428	7.2112	7.9173	7.5626	7.4703	7.5995
6.2974	8.8964	8.0197	7.2492	8.3476	8.039	8.4113	7.2378
6.966	7.7611	7.1966	8.4295	6.8544	7.2677	6.9375	6.8846
7.7611	7.1827	7.6669	6.7237	7.5113	7.739	8.2265	7.0759
7.5373	8.3847	7.6588	7.5157	8.1344	7.5826	7.0532	7.1011
7.5583	7.4404	7.8393	7.8609	7.4954	7.5233	7.5076	7.4028
7.7703	6.7006	7.5271	7.3818	7.6549	7.9743	7.6523	6.9996
7.1006	8.1002	7.1793	7.5518	7.3695	7.4592	8.0854	6.1612
7.8214	7.2023	7.0069	7.5586	8.2345	8.1568	7.2432	7.39
8.0626	7.3545	8.7476	7.2682	7.1924	7.9426	6.9483	8.0047
7.4277	7.7612	7.8679	7.2595	6.494	7.548	7.5024	8.1221
7.8808	7.9285						
CAP2	4.0736	3.8818	3.5391	3.5772	2.1965	4.3432	3.4966
2.7925	4.2827	3.782	3.4893	4.0286	3.2073	4.1035	3.7906
4.2206	4.1572	0.6271	4.775	3.2575	3.777	3.3282	4.0125
4.9301	3.0191	4.4589	2.9567	3.5589	3.228	4.0954	4.0443
0.1159	4.5394	3.7762	3.7246	3.6127	3.5501	4.3566	2.0582
3.7847	3.1219	5.194	2.9483	4.132	3.991	4.6218	4.2762
2.5476	3.3492	4.6922	3.8654	3.6454	3.363	0.7718	4.4473
2.0285	3.5906	3.2821	5.2387	4.3882	3.2994	4.6289	2.9951
4.1028	4.825	3.1659	0.8453	3.7605	2.7185	2.5433	4.8693
3.8307	2.15	3.2101	2.9845	2.8961	3.2296	4.7251	3.7074
4.2643	3.6412	4.1519	4.4833	2.3065	4.7122	3.8861	0.9237

1.6693	2.2813							
CAPG	7.7787	8.3109	5.6276	1.3424	6.1579	2.3854	0.8948	
1.3706	8.9389	8.9259	6.7506	3.2502	5.7172	1.7384	0.5099	
7.2063	1.5643	9.9799	7.6007	8.6624	7.5372	1.617	3.1878	
1.3483	0.6831	0.1817	2.3368	1.1525	6.7083	8.3208	0.7268	
6.8136	9.9465	7.5372	0.7826	7.1729	1.4311	0.9245	0.9346	
2.1205	3.3534	5.5651	0.6827	0.9448	0.6332	6.0706	2.6801	
4.3751	0.4039	3.8241	4.445	1.3619	6.7855	1.2187	0.7351	
1.4511	7.6857	3.2001	8.1566	3.3342	6.2772	8.8674	0.5607	
5.508	0.9055	0.0384	1.7851	0.805	9.1025	0.8287	1.1388	
7.8358	1.5702	5.2904	1.3365	4.8484	7.9734	5.9145	8.2057	
4.8648	7.8202	0.5354	1.5895	2.1513	0.3198	7.4658	9.789	
6.1419	7.4531							
CAPN1	5.346	4.1272	5.855	4.0965	4.9833	5.3814	4.4449	
5.8125	4.898	4.6588	5.2626	4.2364	4.9996	4.0244	5.232	
5.3377	5.5466	3.3717	3.5817	5.8311	5.6183	5.1589	5.2799	
4.9019	5.3923	4.92	3.7583	5.5751	5.2341	4.4481	5.4107	
3.7872	4.5356	5.4413	5.2792	3.9129	4.8636	5.9195	5.0389	
3.3235	5.6307	5.8766	5.6148	4.683	4.6868	4.5703	4.5424	
4.8316	5.1682	3.7188	4.7253	3.3887	3.7784	5.3028	5.2959	
4.9865	5.5486	5.5466	5.2453	5.2646	4.659	4.1083	5.3884	
5.0693	5.1774	4.9589	4.7491	6.7576	4.4797	4.9607	6.1416	
1.3425	5.1228	4.1416	5.0398	5.7857	5.5274	4.6001	5.1656	
5.4989	4.5096	4.9589	5.7847	4.5943	5.2325	4.6308	6.1061	
6.2775	6.3075							
CAPN10	3.1668	2.5439	3.5671	2.5768	2.5778	1.641	2.2647	
3.3944	2.1822	2.2472	2.9929	2.0797	1.5057	2.1673	3.0037	
2.7224	2.3198	2.6855	2.8369	3.3817	3.1278	2.3788	2.4081	
3.102	2.5088	2.2051	2.2772	2.6966	2.4959	2.8279	2.947	
2.9214	3.1154	2.7316	2.2553	2.2762	2.592	3.1923	2.8353	
1.8157	2.8763	2.1102	2.3627	2.4132	1.9284	1.9143	1.9625	
1.0432	3.5076	1.6398	2.5882	1.8824	2.382	2.8334	2.6115	
2.1836	2.0489	3.1527	3.6381	2.8957	2.3026	2.9862	3.3007	
2.2537	2.433	2.5912	2.3182	2.628	3.115	2.3594	2.8329	
2.6524	2.7812	2.8567	2.4907	3.1926	3.0227	2.2339	1.9533	
2.4431	2.6324	2.5951	2.5497	2.629	3.5103	3.2765	2.4137	
2.8635	2.818							
CAPN11	0.0416	0.1099	0	0.1443	0.0156	0	0	0
	0.0455	0.0352	0.0321	0.1398	0	0	0	
0.0682	0	0.3484	0	0	0	0	0.1894	0
	0	0	0	0	0.0425	0	0	0
	0.0221	0.1145	0.1041	0	0	0.0872	0.066	0
	0.1994	0.0905	0	0.0562	0.0118	0.0498	0	
0.0259	0	0	0.0478	0.0235	0.0961	0.033	0	0
	0.0607	0.0161	0.1186	0	0	0.0899	0	0
	0.1697	0	0	0.1626	0.0706	0.0387	0.1373	
0.0451	0	0	0.0086	0.0468	0.0447	0.1146	0.264	
0.0601	0.0068	0.1281	0.0887	0.0139	0	0	0.0501	
0.0314	0							
CAPN12	3.9469	3.8727	3.9343	4.6306	4.96	4.4809	3.5104	

4.3474	3.6691	3.2829	4.5102	4.2789	5.3487	4.424	4.8758	
4.4457	4.4272	3.7437	4.9141	4.8425	3.9625	4.7982	5.1272	
3.8021	4.9614	4.3362	5.0607	3.519	4.6918	3.7509	3.495	
4.2104	4.7714	4.0466	4.8315	3.2537	4.5446	3.0151	5.0274	
4.5144	3.623	4.7567	5.4349	4.4809	5.0612	4.5922	3.2588	
3.381	4.4331	4.4444	3.8075	3.2446	3.9052	5.1255	2.7413	
4.5765	6.0609	4.2118	4.0913	4.6328	4.4278	4.4314	5.0522	
4.0888	3.644	4.7511	3.8208	4.5528	5.1577	5.1314	4.5857	
4.8021	3.7046	5.3908	4.0564	5.3191	4.1981	4.1516	4.1216	
4.7367	4.3654	4.6756	5.3931	4.3547	6.0201	4.5508	3.4801	
3.0546	3.0202							
CAPN13	0	0	0	0.063	0.0452	0	0	0
	0	0.0571	0.01	0	0.0383	0	0	
0.18	0.044	0	0	0	0.0386	0.0207	0.008	0
	0.0312	0.0256	0	0.0646	0	0	0	0
	0.0724	0.0695	0.0216	0.0322	0	0.0428	0	0
	0.0185	0.0053	0.0511	0.0375	0.0227	0.0219	0	0
	0	0.2289	0	0.0379	0	0.0565	0	0
	0.0782	0.0218	0.0197	0	0.0096	0.0218	0	
0.0215	0.0455	0	0	0.0082	0.0885	0	0.0086	
0.0298	0.0343	0.1062	0.0245	0	0.0121	0	0	
0.0825	0.0266	0.1123	0.0648	0	0	0.1042	0	0
	0							
CAPN14	0.0152	0.5504	0.1644	0.0369	0.0636	1.0688	0.3491	0
	1.0152	0.1695	0.5248	0.3971	0.2209	0.1636	0.307	
0.3696	0	0.0849	0.0502	0.1161	0.1399	0.1707	0.4774	
0.3825	0.1828	0.2081	0.3248	0	0.7097	0.0927	0.1723	
0.0277	0.3971	0.4523	0.7724	0.1319	0	0.1442	0.1316	
0.0509	0.3164	0.1086	0.1184	0.565	0.0372	0.0426	0.0287	
0.4096	0.3929	0	0.2838	0.7153	0	0.2932	0.0642	
0.105	0.2178	0	0.1607	0	0.2322	0.3003	0.0674	0
	0.2024	0.0768	1.2796	0.0749	0.1829	0.2978	0.0552	
0.4895	0.8363	0.2293	0.9251	0	0	0.4414	0.4606	0
	0	0.4067	0.1039	0.3585	0.2745	0.0823	0.4303	
0.4531	0.4414							
CAPN15	3.119	3.7721	3.936	2.837	4.0516	3.2619	2.5701	
4.1973	2.6802	2.783	2.5087	2.088	4.0646	3.0697	3.3228	
2.7184	3.7253	1.9806	3.5381	3.3751	3.8738	3.7777	2.5305	
3.6676	3.4466	3.0685	3.1018	2.7184	3.1978	2.9074	2.3389	
4.0111	2.9844	2.7762	2.9542	2.4764	3.3387	3.4277	3.1874	
2.6924	3.1723	2.6379	2.7846	2.6571	3.0997	3.0155	3.1319	
2.8113	3.5723	2.4716	2.6409	2.4716	2.5059	4.1744	2.4559	
3.5493	3.1681	3.909	3.6709	3.6936	3.1056	3.7806	4.5873	
2.8154	2.9101	2.8652	3.149	3.0867	3.2979	3.6661	3.5212	
3.0641	3.586	2.7485	2.7621	3.0862	3.5201	3.1804	2.5543	
2.702	2.8326	3.269	3.9699	3.3362	3.9312	2.9775	2.3333	
3.3711	2.7503							
CAPN2	6.1687	5.3211	5.9011	6.227	6.6219	6.3557	6.8536	
5.8047	5.3968	5.2235	5.0872	5.999	6.6078	6.5359	6.4993	
7.1425	7.4567	6.6629	6.1091	6.5698	6.6059	5.264	6.2686	

6.5838	6.4683	7.8899	6.0721	5.1496	6.3266	6.0067	5.0309	
5.1961	6.041	6.4957	6.4531	5.4808	6.1291	5.1232	6.4433	
5.9937	5.8227	7.6427	7.8396	6.3726	7.9835	7.5307	5.9335	
6.0067	7.2435	7.1236	6.1756	6.3436	5.6706	5.2412	5.7913	
5.4656	8.0346	5.4707	6.8549	5.6368	5.9277	6.1753	6.0085	
6.5438	5.6946	6.6781	5.4105	7.9109	5.385	6.9768	7.0016	
6.3728	4.8511	5.47	7.2951	5.3762	6.0205	6.9571	5.8861	
5.2755	5.4058	6.4216	6.5868	6.1922	7.3648	5.8729	6.3883	
6.0886	6.1276							
CAPN3	8.2194	8.8201	5.1374	4.1681	0.4462	6.3428	6.5055	
7.5102	8.1682	8.951	5.9777	6.5268	0.5732	7.0083	2.1844	
5.0511	0.9218	7.1717	8.2278	6.1664	6.4656	3.0931	4.2862	
7.7656	0.6133	0.9237	5.5349	8.3654	3.5664	8.2824	0.5607	
8.1286	5.1674	7.2983	6.8492	8.573	6.7276	6.8973	7.5735	
5.9836	8.0275	0.711	0.3297	7.9397	1.2797	0.5971	7.7196	
7.7712	1.8659	0.4486	7.6109	2.0756	7.6961	6.8978	0	
7.6788	0.1426	3.3109	7.8653	1.3863	5.4414	9.0877	7.9608	
5.9117	6.3747	0.5604	8.2323	3.7771	8.0851	0	6.0283	
4.6609	8.38	5.2947	5.4193	7.0929	5.9033	7.2093	7.7185	
7.2865	8.0796	3.8809	6.7564	8.0902	0.0032	8.0902	8.5624	
8.491	8.421							
CAPN5	2.5737	2.9173	2.0704	2.8943	4.1949	2.6913	1.9715	
1.8638	3.8419	2.3575	2.4305	2.5548	2.7578	1.7695	1.7588	
3.6596	2.3866	2.4963	0.6236	1.145	2.8472	4.3808	4.2142	
1.4417	3.6178	2.8703	3.3583	2.2725	4.5756	1.4774	2.5543	
1.7141	2.4315	3.0697	2.9189	1.8044	2.3409	4.7175	3.8094	
3.449	3.3571	3.3091	4.766	1.4954	2.2019	1.7002	0.7721	
2.5941	1.9205	2.3249	2.8553	2.1646	3.7005	1.5595	2.5161	
1.8431	2.8195	1.6111	2.8041	1.7715	2.1648	0.9777	2.7359	
1.4267	1.7287	3.646	2.2465	2.487	2.0156	4.1915	3.9463	
2.6215	1.9453	3.4339	2.9468	4.3558	1.5441	3.2568	4.2448	
4.1034	3.8859	3.0108	3.9227	3.9155	2.8617	3.5771	3.4247	
3.3777	3.6091							
CAPN6	0	0	0	0.0272	0.0027	0	0	0
	0.0175	0.0319	0.4717	0.0737	0	0.0645	0.031	
0.0135	0.0468	0	0	0	0	0.0076	0.0226	
0.0192	0	0.0071	0.0378	0.0292	0	0	0	0
	0.0128	0	0.0036	0.0076	0.0188	0	0	
0.0186	0	0.0015	0	0	0.0056	0.0224	0	
0.1529	0	0.111	0	0	0	0	0.0299	
0.0656	0.1506	0	0	0	0	0	0	
0.0337	0.0636	0.0491	0.0444	0	0	0	0.0844	0
	0	0.0446	0	0	0.0624	0	0.0333	0
	0	0.0068	0	0	0	0	0.0195	
0.0223	0							
CAPN7	2.8187	3.5599	3.8434	3.5889	3.6493	3.5435	3.6019	
3.2648	3.1462	3.2669	3.9371	3.4829	3.108	3.7336	3.4472	
4.6832	3.2449	4.1493	4.0862	2.7129	3.1872	2.7909	3.17	
2.9936	2.9021	3.2529	3.7041	3.1774	3.1062	3.8438	4.0329	
3.5246	3.3735	3.0297	3.2812	3.6476	3.1555	2.8798	3.3252	

3.5675	3.0527	3.1221	2.4902	3.3578	4.2351	3.4787	3.8671	
3.0681	3.6497	3.6586	3.5791	3.0537	3.2832	3.5876	3.5386	
3.075	2.5496	3.7367	3.7489	3.3533	3.3502	3.7113	3.0312	
3.2477	2.7511	3.4426	3.2812	2.9435	4.5495	3.2492	3.3469	
3.179	3.6483	3.2022	3.4576	3.1763	3.5349	3.3779	3.0789	
3.0393	3.6797	2.6276	3.4378	3.2366	3.2077	3.3256	3.3357	
3.1025	3.1717							
CAPN8	0	0.1416	0.0349	0	0.0047	0	0	0
	0.014	0	0	0	0.0542	0	0	
0.0423	0	0.033	0	0	0	0	0.0049	0
	0	0	0.0238	0	0.0337	0	0.0284	
0.0072	0.0215	0.0181	0	0	0.0707	0	0.0115	0
	0.0149	0	0.0824	0.0209	0	0.0201	0	0
	0	0.0654	0.0179	0	0	0	0.0268	
0.0561	0.1123	0.0242	0.024	0.0221	0.079	0	0.0148	
0.0312	0.0555	0.0014	0	0.0022	0.0102	0	0.0362	0
	0	0	0.0349	0.0203	0	0	0	0
	0	0	0	0	0	0	0.0509	
0.0306	0							
CAPN9	0	0.0596	0	0	0.0182	0.0443	0.0352	
0.0174	0	0	0.0253	0.0219	0	0	0.0519	
0.0536	0	0.171	0	0	0	0	0.0324	
0.098	0.0092	0	0.0821	0.0929	0.0228	0	0	
0.0484	0.0281	0	0.0267	0.0163	0	0	0.0351	
0.0478	0	0.0033	0	0	0	0.0244	0	0
	0	0	0.0457	0	0	0	0	
0.0718	0	0.131	0.0333	0.1228	0.0103	0.0391	0.0386	
0.0209	0	0	0.0269	0.0529	0	0	0	0
	0.0139	0.0336	0	0	0	0.1607	0.0039	
0.0268	0	0.0054	0	0.0291	0.0567	0	0	0
	0.0489							
CAPNS1	6.9919	7.1094	6.9149	6.9383	7.6321	8.227	6.8415	
7.4726	7.4396	7.0791	7.4396	7.5931	7.813	8.4832	7.8697	
7.645	7.9827	7.0981	7.4396	7.5686	7.4434	6.9872	8.3427	
6.7963	7.6582	8.0516	7.4396	7.3413	7.6802	6.792	9.0207	
7.3482	7.8923	7.7435	7.4925	7.1663	7.703	7.5643	7.2812	
7.4231	7.5353	8.2025	7.7482	7.1266	8.6925	7.5598	6.3477	
6.557	7.5727	7.7205	7.5353	6.8959	7.5353	6.6433	8.3752	
6.7702	7.452	7.1322	7.0462	6.8409	7.8908	6.9602	7.9046	
8.183	7.431	7.5822	6.5414	8.1508	7.2274	7.5822	8.4162	
7.5861	7.1129	7.5822	7.431	7.2988	6.993	7.2274	7.5648	
6.8251	7.2966	7.9679	7.2855	6.936	8.7406	7.6799	8.3692	
8.0251	7.9484							
CAPNS2	0.2619	0.2229	0.2247	0.286	0.122	0.7479	0	
0.0444	0.295	0.1321	0.0328	0	0.1553	0.8496	0.2626	
0.1793	0	0.1316	0.6921	1.1887	0.9976	0.2511	0.0542	
0.8304	0.1486	0.2415	0	0.1596	0.0874	0.0878	0.0473	0
	0.4277	0	0.4027	0.1421	0.0365	0	0.0419	
0.2225	0.1167	0.0473	0.0718	0.2329	0.0704	0.1296	0.358	
0.1642	0	0	0.0885	0.263	0.1528	0.3303	0	

0.51	0.0762	0	0.3424	0.122	0.2192	0.2768	0.0367
1.0099	0	0.0703	0.643	0.0032	0.2368	0.5259	0.3791
0.535	0.1916	0	0	0.3026	0	0	0.0673
0.3193	0	0.1032	0	0.2255	0.2435	0.0982	0.118
0.1303	0						
CAPRIN1	5.9368	6.2754	6.3776	6.5364	6.3794	6.7766	6.0311
5.9465	6.3794	5.7645	6.4828	5.7702	5.9637	6.5827	6.5535
6.3666	6.5095	5.5907	6.5518	5.6018	6.2727	6.3887	6.1116
6.5518	6.8471	6.2658	6.0955	6.2917	6.5535	5.6428	6.0588
6.2613	6.1292	5.3464	6.3666	6.1985	6.1985	6.3067	5.9261
6.6936	5.8025	6.3712	6.6176	6.7499	5.788	6.6334	6.4485
6.5806	6.3373	6.7247	6.8041	6.4662	6.4979	6.2745	6.4823
6.3705	6.4351	6.407	6.4176	6.5649	6.1738	6.1037	6.1198
6.6691	6.1878	5.9187	5.7488	6.4809	6.4521	6.2537	6.3432
6.1724	6.3602	6.76	6.6129	6.6639	6.2666	5.9833	6.7037
5.961	6.0205	6.2998	6.3319	5.6612	5.4383	6.1452	6.0697
6.1276	6.1859						
CAPRIN2	2.63	2.8541	2.7079	2.5844	2.3737	3.614	3.1338
3.0197	3.817	2.8587	2.9531	2.2442	3.6477	3.7787	3.4288
3.0963	3.2013	3.0445	3.5419	2.5017	2.3507	3.1487	3.1236
3.02	3.7128	3.328	2.7741	2.3585	3.0261	2.4422	2.2134
2.4906	4.0015	3.3296	4.2918	2.9373	2.3146	2.1635	2.2171
4.4803	2.3285	2.505	4.5968	3.4883	3.9125	2.7776	2.7426
2.4007	3.4875	3.4308	4.3555	2.8326	3.2909	1.9589	1.9756
2.1079	3.9179	3.6643	3.2258	2.2977	3.0131	3.0449	2.7515
3.418	2.0244	3.4189	3.0299	2.3802	2.7447	2.4208	3.6263
2.9886	2.8376	3.1725	2.8629	2.4495	2.9785	2.1	3.21
3.485	3.6486	3.3835	3.168	3.0402	1.6546	3.1132	2.7634
2.4198	2.5971						
CAPS	1.5128	0.5907	1.7863	1.166	6.2411	1.2747	0.6244
0.9492	0.5142	2.1363	1.7893	0.4232	0.8241	0.8345	5.5888
1.0374	0.8054	2.1613	0.4538	1.3677	3.5621	1.4435	2.9532
1.3219	7.089	0.8087	2.162	1.1946	2.692	1.2693	0.6731
3.2326	2.7695	0.9544	2.9929	1.6982	1.2767	1.7152	1.4179
1.3564	1.187	1.0801	2.9077	0.6422	0.4489	0.9034	0.4541
0.6067	1.5135	0.4484	0.6738	0.5047	0.5569	1.2284	0.3641
1.594	0.9005	4.0894	1.6893	3.2381	3.8776	1.1442	1.2295
1.009	1.3894	1.7446	0.5562	0.4681	2.5261	4.956	0.3786
2.0985	0.1711	2.6994	1.1737	6.6574	1.7475	2.6643	0.6679
1.3466	0.6386	1.9141	1.5679	3.8447	4.3092	0.6266	0.9604
0.9598	0.9218						
CAPS2	0.9395	0.2535	0.7665	0.5375	0.1701	0.7481	0.4971
0.4866	0.4815	0.4192	0.5097	0.3946	0.3547	0.5136	0.2044
0.738	1.5094	0.9125	0.5787	0.7088	0.493	0.0908	0.3008
0.0748	0.2876	0.2301	1.3261	1.0614	0.1307	0.5605	1.7818
0.3209	0.2717	0.6796	0.3723	0.5508	0.2748	0.3113	0.1116
0.5565	0.5406	0.3707	0.1005	0.4886	0.2892	0.4088	0.4435
0.5306	0.5306	2.0917	0.5764	0.6501	0.7107	0.0821	1.5485
0.7103	0.6891	0.5969	0.7595	0.0646	0.2096	0.474	0.1183
0.4225	0.6482	1.1553	0.7809	0.5871	0.4646	0.3682	0.6364

0.1677	0.3231	0.2207	0.7955	1.3616	0.6081	0.4033	0.1946	
0.8665	0.3664	0.3826	0.1323	0.6141	0.1703	0.8311	0.4398	
0.3713	0.5415							
CAPSL	0	0	0	0	0.0406	0	0.0268	0
	0	0	0	0	0.3753	0	0.0521	0
	0.1411	0	0	0.0678	0	0	0	0
	0	0.0282	0	0	0.0355	0	0	0
	0	0	0.0144	0	0	0	0	0
	0	0.0145	0	0	0	0.1898	0	0
	0	1.1173	0	0.0702	0	0	0	0
	0	0	0.0657	0	0	0	0	0
	0	0.0127	0	0.1246	0	0	0.4684	0
	0.0755	0	0	0	0	0	0	0
	0	0.3323	0.1963	0	0.1577	0	0	0
	0							
CAPZA1	6.4977	6.5145	6.4732	6.7081	6.6608	6.6679	6.5078	
5.8313	5.9998	6.0464	5.9242	6.6513	6.3328	7.7348	5.851	
6.0382	6.8258	6.1625	7.0233	5.6563	6.5805	6.0327	5.8862	
6.0771	6.1249	6.8466	4.8589	6.2529	5.6254	5.2124	6.5879	
7.0233	6.9035	6.3355	6.5515	6.6608	6.6735	6.0978	5.8457	
6.771	5.7317	6.8087	5.7534	6.6661	6.4281	7.2462	6.2931	
7.0657	6.0562	7.1062	6.3128	6.681	6.7385	6.7031	6.9563	
6.2034	7.2645	6.5618	5.9198	6.8104	6.1437	6.034	5.3894	
6.7805	7.0279	6.2153	6.7109	6.8187	5.7588	6.3185	6.0018	
6.8505	6.7152	6.1336	6.0174	6.0315	6.8591	5.557	6.5633	
5.7528	7.1112	6.3626	5.6097	5.4182	6.6232	6.7613	6.5496	
6.4632	6.4732							
CAPZA2	5.7538	6.2764	6.2585	6.6242	5.7593	7.3321	6.4001	
6.4794	6.316	5.7037	6.0223	5.9092	6.1716	6.3838	5.9092	
7.3321	5.4889	7.6683	6.5725	6.032	5.9037	6.3692	6.1743	
6.3405	6.5482	6.1308	5.6309	6.0497	6.6505	6.1308	6.1603	
5.9906	5.9858	5.3577	6.1145	6.4285	5.6067	5.612	5.5445	
5.8585	6.0038	6.2178	6.1743	6.0567	6.2032	6.4534	5.5452	
6.4463	6.2662	5.8316	6.1798	6.4056	5.4822	6.0966	6.3782	
5.8351	5.1435	6.2782	6.7222	6.4894	6.0137	6.0357	6.4089	
6.1785	6.4074	6.0076	5.9114	6.2306	5.8536	6.1949	6.3065	
6.2772	6.6881	5.917	6.1493	6.2281	5.7296	6.1132	6.0493	
6.2829	6.2218	5.8982	4.0923	6.2617	6.0146	5.3322	5.8018	
5.8101	5.6955							
CAPZA3	0	0	0	0	0	0	0	0
	0	0	0	0.0148	0	0	0	0
	0	0	0	0	0	0	0	0
	0	0	0	0	0	0	0	0
	0	0	0	0	0.02	0	0	0
	0	0	0	0	0.0207	0.0318	0	0
	0	0	0	0	0	0	0	0
	0	0	0	0	0	0	0	0
	0	0	0	0	0	0	0	0
	0	0	0	0	0	0	0	0
	0	0	0	0	0	0	0	0

	0								
CAPZB	6.1778	7.1489	6.7639	7.018	6.768	6.595	6.8002		
6.0891	7.7534	6.6968	7.0592	7.5982	7.4522	7.0158	6.5695		
5.6438	6.0081	6.0395	7.1087	6.7024	7.2006	6.6159	6.8208		
6.9406	6.9929	7.1649	6.4208	6.7575	6.7902	7.9726	6.5647		
6.6545	7.8854	7.198	7.0963	6.7639	6.1963	6.5792	6.3964		
7.0018	7.1806	8.1897	7.0638	6.8635	7.4664	7.0246	6.8957		
6.6795	6.5061	6.9539	7.0567	6.4999	7.1806	6.9056	6.635		
6.6091	6.8692	6.6209	6.8717	6.9247	6.8815	7.7423	5.8075		
7.0714	6.5088	6.4501	6.734	7.1096	7.4183	6.4677	6.757		
7.0345	6.4549	7.6129	6.5825	7.1811	7.2776	6.5143	6.8815		
7.2208	7.0576	7.4076	6.013	6.134	7.4923	7.341	7.2065		
6.8449	7.245								
CARD10	2.0812	0.4074	1.2605	1.349	2.9232	0.1833	0.0374		
1.1678	0.5641	0.9926	0.1806	0.6454	3.9135	0.2243	3.1634		
0.4707	1.3672	0.7759	0.1026	2.3653	0.4979	1.8732	2.0708		
0.2645	2.2355	2.7357	0.3212	1.3915	1.2283	0.2286	1.3921		
0.143	0.5167	0.0715	0.7917	0.4288	2.3558	2.8657	2.6314		
0.2235	0.474	2.4906	1.7272	1.395	2.9501	0.981	0.6791		
0.4092	3.4025	2.491	0.6143	1.5435	0.4814	1.3177	1.8522		
1.1818	2.8028	4.7026	1.4291	3.6125	1.8703	1.8854	1.0841		
0.5255	1.1812	0.3223	0.386	0.2915	0.2589	3.4613	0.9493		
0.9859	0.5349	0.8106	1.4586	0.0095	0.3459	1.416	0.455		
0.9504	0.2626	3.4337	1.094	0.5875	3.461	0.6549	0.3149		
0.5965	0.6896								
CARD11	0.0829	0	0	0	1.3237	0	0	0	
	0.4303	0	0	0.3888	0.0443	0.0935	0.1738		
0.1167	0	0.0731	0	0	0	0.1278	0.6775		
0.0949	0	0.0597	0	0	0.0691	0	0		
0.0373	0.058	0.0711	0.7145	0	0.0591	0.1907	0.1355		
0.1194	0	2.8957	0.0735	0	1.7064	0.0568	0		
0.248	0.2208	0.1147	0.3316	0	0	0	0		
0.0224	2.884	2.9405	0	2.7685	0.0044	0.0068	0.015	0	
	0	0	1.00E-04	0.0863	0	0.1297	0	0	
	0	0.6261	0	0	0	0.0044	0.0447	0	
	0	0	0	0.0196	0	0.0096	0		
1.1413	0.1437								
CARD14	2.1449	3.0419	1.7547	0.7424	0.5545	0.8794	0.7955		
0.7412	2.2729	1.4828	1.5753	0.8218	0.4861	0.8089	0.3947		
2.3723	0.6149	1.3952	2.0514	0.652	1.5947	0.3078	0.4472		
0.8188	0.4261	0.4331	0.5883	2.8253	0.4987	1.5845	0.1906		
1.6751	1.132	0.9067	0.7468	1.1118	0.9241	1.031	1.3265		
0.8358	0.7369	0.2809	0.2163	0.8492	0.5156	0.2202	0.9858		
1.0142	0.5815	0.2759	0.533	0.4432	0.7441	1.158	0.4175		
1.4005	0.2247	0.6187	1.3473	0.4877	1.0935	1.3229	1.2114		
0.8604	0.8872	0.171	1.1267	0.4132	2.5454	0.4721	0.5868		
0.5655	1.3346	0.5972	0.838	0.9651	1.1898	1.7358	2.2808		
1.0364	2.9433	0.2487	0.3152	1.5186	0.3264	0.9456	0.8682		
0.4107	0.5522								
CARD16	1.2048	0.9525	0.1574	0.3003	0.1368	1.8418	4.6203		

0.0886	4.4097	0.799	4.9365	2.4923	4.1699	4.6642	0.1917	
5.2012	0.5123	5.74	1.6055	0.1658	0	2.5142	3.9418	
0.7509	0.2321	2.7583	2.9955	0.6787	2.1916	0	0.0838	
2.5908	4.4027	4.3487	2.0783	0.611	3.865	3.2429	0.0819	
3.282	5.5548	5.2611	0	1.719	0	5.1058	1.8082	
4.0481	3.2999	1.4394	2.8101	3.6315	3.7034	3.8513	0.1295	
2.009	4.264	0.7155	2.7494	1.9769	3.4194	3.0893	4.8806	
3.9541	0.0531	0.7897	2.3644	0	0	0.291	3.988	
0.1401	4.7279	3.1512	0	0.1373	3.6257	1.2283	4.4351	
2.1161	0.1122	1.3552	4.995	0.1104	1.5332	3.838	1.9743	
0.5046	0.925							
CARD17	0	0	0	0	0	0	0	0
	0	0	0	0	0	0.1638	0	0
	0	0.1528	0	0	0	0.0662	0	0
	0	0	0	0	0	0	0.1386	0
	0	0	0	0	0	0	0	0
	0	0	0	0	0	0	0	0
	0	0	0	0	0	0	0	0
	0	0	0	0	0	0	0	0
	0	0	0	0	0	0	0	0
	0	0	0	0	0	0	0	0
	0	0	0	0	0	0	0	0
	0							
CARD18	0	0	0	0	0	0	0	0
	0	0	0	0.0441	0	0	0	0
	0	0	0	0.1664	0	0	0	0
	0	0	0	0	0	0	0.4202	0
	0	0	0	0.2049	0	0	0	0
	0	0	0	0	0	0	0	0
	0.0948	0	0	0.0848	0	0	0.5364	0
	0	0	0	0	0	0	0	0
	0	0	0	0	0	0	0	0
	0.0733	0	0	0.0917	0	0	0	0
	0	0	0	0	0	0	0	0
	0							
CARD6	1.2803	1.3677	0.0377	2.9468	1.9322	1.9443	2.3796	
0.7697	1.4779	0.8454	0.5393	1.5562	1.5945	1.9179	0.789	
0.7931	1.7952	0.7714	2.0187	0.3004	1.2057	0.8834	1.652	
2.066	1.5118	3.0866	0.6069	0.0525	1.7865	0.4855	0.0412	
0.8943	1.3834	0.8834	1.7432	0.8736	1.3738	2.3769	0.3458	
1.8569	1.4587	2.4315	2.3083	0.3132	0.4728	2.1725	0.1297	
1.5445	2.0524	0.9819	1.2239	3.1485	1.2448	1.152	0.5272	
2.0519	2.5114	0.0651	1.6933	0.1498	1.2808	1.2775	1.2768	
2.0936	0.2544	2.8383	1.3667	0	0.02	1.7903	1.6182	
1.5222	1.7827	0.8715	0.4066	0.1156	1.636	1.7017	1.6533	
1.4565	1.4573	2.0181	1.7219	1.6051	2.2015	1.0025	2.3389	
1.8367	1.6902							
CARD8	2.6461	2.8736	2.4246	2.8158	2.4261	2.0154	4.5717	
2.3732	3.0016	1.8461	2.2254	4.274	2.9193	2.5221	3.0289	
2.5447	2.9453	3.0447	3.4768	2.5663	1.9738	1.8107	3.2979	

2.2903	2.5509	2.933	3.2157	2.9432	2.9299	2.6474	3.7483
2.5036	3.9576	3.7396	2.4246	2.357	2.0862	2.5843	3.0093
3.0652	2.9435	3.0502	2.3713	1.9632	3.2666	2.9132	2.3673
2.2038	3.5815	2.5226	2.7553	3.128	3.1424	4.8655	2.5556
2.233	3.8838	1.7813	1.961	2.2503	2.0045	2.2983	3.2243
3.6771	2.8569	2.596	1.9044	3.1498	3.0813	1.9884	2.9301
2.607	2.5527	3.4592	2.7139	3.3073	2.9899	2.8462	2.7816
2.6187	2.5125	3.2974	2.5822	2.2247	3.17	3.0087	2.9102
2.6351	2.5804						
CARD9	1.4339	0.5703	0.2326	0.1429	0.405	0.2398	0.0539
0.4344	0.0561	0.2217	0.1772	0.0527	0.1918	0.1919	0.1841
0.0534	0.0715	0.3403	0.1724	0.6896	0.2172	0.3988	0.123
0.1386	0.161	0.3774	0.0616	0.2172	0.0926	0.1113	0.178
0.4383	0.1424	0.0582	0.1369	0	0.5483	0.1674	0.4589
0.1935	0.3933	0.2114	0.1776	0.0614	0.1277	0.2048	0.0683
0.3583	0.4739	0.0889	0.0577	0.6052	0.0793	0.3545	0.3067
0.2613	1.3646	0.2794	0.9094	0.2632	0.3021	0.3292	0.3028
0.2208	0.2916	0.3751	0.434	0.0799	0.2501	0.6123	0.1911
0.2492	0.1602	0.0677	0	0.1399	0.2652	0.134	0.0493
0.1314	0.1853	0.2859	0.0844	0.2667	0.312	0.2859	0.0933
0.2501	0.1217						
CARF	1.5432	0.7019	1.1801	1.0032	0.8914	1.0546	0.888
1.9157	1.2033	1.3311	1.2653	0.6385	1.005	1.027	0.8337
0.5674	1.5867	1.9146	1.3139	1.077	1.1206	0.6044	1.0645
0.7464	0.9844	1.2591	0.9443	1.1744	0.9929	0.9797	1.0382
1.2371	0.8867	1.3911	1.1442	0.8848	1.1683	1.1966	1.0565
0.6799	0.7403	1.1579	1.1966	1.0868	1.1061	1.2014	0.9904
1.1161	1.4054	1.7803	0.9642	0.8888	1.3539	1.2997	0.7478
1.0731	1.2585	0.7027	1.2328	0.5784	0.8142	0.9966	0.4476
0.9312	1.3933	1.9369	1.3761	1.2189	1.5193	1.5509	1.3393
1.3375	1.4188	0.6634	1.0178	1.246	0.863	1.2157	0.9218
0.8392	1.2659	1.0509	1.2711	1.2385	0.672	1.1623	1.6829
1.4255	1.7099						
CARHSP1	4.6704	4.0683	4.1149	4.262	4.4814	5.1017	3.8401
4.1741	3.5204	3.6305	3.5758	3.1804	4.2524	4.2015	5.528
5.4478	3.8028	2.6341	3.6501	4.3329	4.9117	4.6409	3.7663
5.1235	5.6225	3.6655	4.6777	3.9556	3.8151	2.8759	3.9321
4.6445	5.2263	3.8891	3.9115	3.3518	4.0771	3.0894	3.4215
4.2608	3.7262	5.171	4.8157	5.4046	4.5231	3.7617	2.7157
3.8374	4.3408	3.9632	3.3271	3.9956	3.2383	2.4536	3.6609
3.1958	4.2614	5.1467	4.1798	4.9803	4.616	3.8745	4.129
4.3818	3.384	3.2608	4.8548	3.4506	3.4652	4.7791	5.2499
5.2328	4.1342	3.7305	5.245	3.7147	3.6664	3.5694	3.8616
3.8738	3.6748	4.7322	3.8123	3.9918	5.5046	3.9176	4.3758
4.5602	4.9329						
CARM1	5.2843	5.0359	5.6845	4.2066	5.8653	4.7116	4.2987
5.3576	4.9417	5.3142	5.5795	4.4886	5.2062	4.3969	5.4593
4.9644	4.7289	4.5082	4.6151	5.6939	5.2813	5.1915	4.9571
5.3155	5.0743	5.395	5.0956	5.4931	5.3072	4.5099	5.2612
5.0652	5.2647	5.8216	4.6214	4.6327	5.4034	5.0956	5.7849

4.737	5.3934	4.9891	5.425	4.8598	4.9964	4.8204	4.5233	
4.6196	5.3498	4.9891	4.5267	4.0323	4.4269	5.478	5.3256	
5.4358	5.0797	5.4292	6.2382	5.4722	5.0443	5.4722	4.812	
5.1008	5.6572	4.4132	5.1951	5.6538	5.4028	5.1753	5.7166	
5.4048	4.7366	4.5125	4.5524	4.4685	5.0496	4.4758	4.8952	
4.6847	5.3503	5.0803	4.9536	5.3761	5.0924	4.6985	5.212	
5.2914	5.3687							
CARNS1	0.2504	0.0059	0.3645	0.0412	0.4866	0.1501	0.0581	
0.2568	0.0195	0.317	0.1366	0	0.1257	0.0334	0	
0.1254	0.127	0.3465	0.263	0.3152	0.1749	0.1439	0.1244	
0.1541	0.0588	0.0418	0.3925	0.1598	0.3982	0	0.1358	
0.0517	0	0	0.05	0.0424	0.1704	0.2366	0.079	
0.3892	0.1088	0.3674	0.0591	0.2276	0.1588	0.1479	0.0178	
0.0529	0.1472	0.4261	0	0.1045	0.0435	0.1031	0.1335	0
	0	1.0056	0.24	0.1981	0.1276	0.0963	0.1613	
0.0612	0.1307	0.627	0.0688	0.0812	0.0476	0.0766	0.15	
0.0666	0.0948	0.1472	0.0766	0.1005	0.2509	0.0703	0.1019	
0.1336	0.0532	0.0685	0.0686	0.1219	0	0.1252	0.1215	0
	0.0622							
CARS	5.1983	5.0939	4.8032	5.098	4.8945	4.1513	4.9421	
5.3003	3.8942	5.8651	5.0279	4.5311	6.2618	4.0289	4.5891	
4.1504	5.9922	3.5958	4.6415	4.8343	3.7505	5.5588	4.2901	
4.9777	5.3381	5.7839	4.4523	4.2446	4.3832	4.3109	5.0856	
4.7694	5.1551	4.1443	4.1988	4.3814	5.4295	5.8343	6.3928	
4.3888	5.6817	6.0695	5.646	4.6822	3.8946	4.6535	4.6086	
5.2142	5.3213	4.7561	3.6327	4.6441	4.4799	6.2416	4.9329	
6.0766	6.2428	4.9575	4.6064	5.1978	4.7621	5.4021	4.276	
4.5833	3.9007	5.2068	3.6912	5.1248	5.8149	4.4522	4.4455	
4.0956	4.2149	5.6192	4.4488	5.0456	3.8036	4.0654	3.7466	
4.0526	5.0128	4.7764	5.3928	3.6958	4.3009	4.8633	4.4621	
4.2985	3.7796							
CARS2	5.0822	4.6106	5.7327	5.3094	5.175	4.4201	5.1226	
4.493	4.368	4.1856	3.1394	3.9915	5.0885	5.4927	4.7685	
5.0315	4.0212	3.958	5.137	4.8267	5.2856	4.2639	3.8571	
4.5019	4.1482	4.1527	3.2926	4.6386	4.4557	4.7065	5.1877	
3.2855	4.8374	4.4475	4.3354	4.5345	4.1754	4.444	5.1864	
3.8983	4.7239	4.9209	5.1775	4.335	4.5234	3.4453	4.9061	
3.8403	4.7808	5.0251	4.7971	4.119	4.5206	4.6122	4.9666	
5.8099	4.4898	4.1664	4.2105	4.0287	4.2207	4.8375	5.1857	
5.2208	4.3025	3.8009	3.1351	5.3879	5.5803	4.8556	5.1928	
5.3826	4.6514	3.6339	4.5594	3.9846	4.9946	4.1306	4.852	
4.2075	4.1476	5.3771	4.7442	3.8034	4.6349	4.872	5.5352	
5.6338	4.8729							
CARTPT	0	0	0	0.2456	0	0	0.6614	0
	0	0	0	0.1863	0.1051	0.168	0	0
	0	0	0.2176	0	0	0	0	0
	0	0	0	0.1818	0	0	0	0
	0	0	0	0	0	2.4916	1.4944	0
	0	0	0	0	0	0.1229	0	
0.4611	0	0	0	0.1243	0	1.2124	0	

0.0793	0.3884	0.1124	0	0.6422	0.3338	0	0.2899	0
	0	0	0	0	0	0	0.3829	0
	0.0058	0.3503	0	0	0	0	0.2968	
1.3972	1.2791	0	0.954	0	0.6357	0.0624	0	0
	0							
CASC1	0.2207	0.1509	0.0453	0.1119	0.1868	0.0653	0.0418	
0.1647	0.0991	0.0804	0.1123	0.1402	0.2076	0.0915	0.0317	
0.047	0.2598	0.0916	0	0	0	0.0456	0.1588	
0.0866	0.0283	0.1597	0.366	0.1903	0.0222	0.1364	0.7311	
0.0806	0.1206	0.1002	0.0606	0.1436	0.0675	0.0868	0	
0.1886	0.3234	0.1119	0.0377	0.1159	0.0322	0.2174	0.0915	
0.2157	0.0849	0.8368	0.123	0.1094	0.2001	0.1896	0.254	0
	0.0152	0.0766	0.1458	0	0.0617	0.0767	0	
0.1355	0.0798	0.247	0.4716	0.031	0.2294	0.3945	0.1467	
0.0777	0.1237	0.0307	0.1808	0.2657	0.0959	0.0433	0.0651	
0.2443	0.035	0.2347	0.0699	0.2675	0.0713	0.2302	0.1041	
0.0787	0.4436							
CASC10	1.3272	0.7796	1.2239	2.8382	3.3361	0.8879	1.1536	
1.6473	0.8022	0.9715	1.2336	0.5584	2.1849	0.5441	2.3828	
1.0902	2.1156	1.5812	1.4194	1.9034	1.5689	0.7603	2.1756	
1.2322	2.4295	0.966	0.0459	0.9421	0.8079	0.7849	2.1569	
1.2122	1.2994	1.3591	2.5845	1.8813	0.4984	0.7418	2.2358	
1.4133	0.8377	1.3657	1.9147	0.8952	1.6	0.387	0.4049	
0.9213	1.787	2.2171	1.4015	2.1863	1.1488	0.7203	3.366	
2.2875	1.731	0.5568	0.5847	1.0054	2.0221	0.6647	1.7882	
0.9139	0.4956	0.9139	0.2124	2.4869	1.1003	2.6228	1.2465	
1.3937	0.7065	1.1889	1.6176	0.6381	1.0155	1.7079	1.0064	
1.1548	1.0552	2.4468	2.9226	1.042	2.7932	1.587	0.5928	
0.3375	0.4378							
CASC3	5.3537	4.7973	4.0847	4.8251	4.9365	4.6319	4.3085	
4.3161	4.7587	4.9714	4.6112	4.5856	4.8629	4.7671	4.2677	
4.9714	5.7021	4.854	4.6051	4.2464	4.4107	4.5071	3.9266	
4.3938	4.8005	4.9823	5.0204	5.5273	4.5621	4.3406	5.1055	
4.8714	4.4185	4.1458	4.6137	4.6884	5.3803	4.7714	4.3977	
4.7268	3.7859	4.0658	4.5547	4.7936	4.2277	4.7361	4.6985	
4.9029	4.5512	5.3147	4.8087	4.5111	4.277	4.8255	4.8757	
4.9941	4.743	4.7627	4.6892	4.9452	4.871	4.4007	4.0365	
4.9821	4.9594	4.6177	3.893	4.4608	5.2799	4.8814	4.3674	
5.3139	4.7482	3.9492	5.1634	4.8976	4.6741	4.6908	4.5915	
3.816	4.5679	4.3631	5.3414	4.452	4.6935	3.92	4.4148	
4.1655	4.4566							
CASC4	4.2855	3.9629	3.8778	4.7606	4.9971	5.1398	5.4989	
5.2314	4.9054	4.6857	4.9809	4.616	5.0303	3.5607	4.9215	
5.3625	4.1432	5.6398	4.0567	4.9079	4.9324	4.7477	5.3299	
5.084	5.1504	5.1289	4.9638	4.6594	4.9387	4.6819	5.2614	
5.1959	3.7116	5.2453	5.5899	4.6793	4.5142	4.3869	4.1901	
4.726	4.9263	5.4155	5.556	3.889	6.4119	5.4863	4.1586	
4.9185	4.9721	4.4722	5.0123	4.7096	4.1416	5.1963	4.8467	
4.6205	5.0329	4.9398	4.8312	5.0062	4.1936	4.3559	4.8398	
4.5211	4.1515	6.1528	4.3503	5.4162	4.878	4.9029	4.5859	

4.4179	3.8446	4.522	5.4569	4.9785	3.6825	5.9166	4.8686
3.6017	4.2404	5.2776	5.3558	5.8437	4.8461	4.1177	4.3767
4.1498	4.1485						
CASD1	2.8511	1.9525	2.52	3.6857	2.3412	4.9962	3.7764
3.3256	3.5373	2.4938	2.4818	2.1925	2.4924	3.0264	3.1328
4.1169	2.8069	4.9387	3.4749	2.3802	2.555	2.9903	3.7281
2.9032	3.7452	2.7669	2.4594	1.7626	4.2376	2.0103	2.2756
2.9104	2.7812	3.2373	3.6705	2.7552	3.3284	2.7867	1.6662
3.0657	3.3484	2.6187	3.0284	2.9354	3.3614	3.5979	2.9499
4.0257	4.2904	2.9079	3.1718	4.0473	2.6473	2.2599	3.0183
3.0449	2.147	3.8845	3.9264	4.3164	3.2049	3.4948	2.8672
2.9666	3.1636	3.3969	1.7019	2.8369	2.8094	3.3036	3.1476
3.2334	3.4956	1.4906	3.1426	3.6371	2.3191	2.4918	3.2623
3.1701	2.714	3.0949	1.3461	2.9241	3.0495	2.1889	2.8952
2.8997	2.8229						
CASK	2.6352	2.3691	2.3296	2.8357	2.1879	2.4348	4.2654
2.7056	3.236	2.4709	2.3139	3.079	2.5705	2.0543	2.6118
3.1515	2.2982	2.9014	2.2866	2.5387	2.1525	2.5894	3.4665
3.0285	2.2857	3.7305	3.3577	2.5587	2.4002	2.9745	3.3483
2.759	2.4864	3.061	3.304	2.872	1.9168	2.2249	2.6122
3.2283	2.0186	2.5678	2.5304	2.1638	3.7013	3.1475	2.7324
3.082	1.6799	2.8843	2.5283	2.8325	2.3787	2.4921	3.3352
2.0997	2.7701	2.6723	2.419	3.3631	1.9819	2.9737	2.9987
2.4377	2.3492	3.488	2.8025	3.4496	3.647	2.294	2.7441
1.9579	2.501	2.8008	3.6239	2.8125	1.9875	3.7339	3.0344
2.4181	2.6032	3.2858	2.2073	2.7615	2.1954	2.1379	2.4792
2.4372	2.2843						
CASKIN1	1.7921	2.7632	2.3244	2.2295	2.3283	2.04	1.4202
2.5715	1.6035	1.2758	2.2761	1.5697	1.7758	2.226	1.578
1.4507	2.3885	2.3494	2.3372	2.1842	1.9957	2.3666	1.5978
2.2678	1.8523	1.4795	2.9823	2.2139	1.8932	2.278	2.3608
2.8213	1.6173	1.9387	1.4901	1.615	1.7707	2.6018	1.9972
1.7052	2.3288	1.8767	1.7063	1.5313	1.9819	1.8309	1.9231
2.0153	2.0131	2.4968	1.9124	1.8414	1.9687	2.5343	2.1535
2.6277	1.6958	2.4181	2.1654	2.5111	2.7663	2.4158	2.2284
1.812	2.7393	1.812	2.0565	2.5597	2.4421	2.4871	2.4205
2.3313	2.1529	2.4289	1.99	1.3156	2.0834	2.2334	1.5113
2.5965	2.2319	2.7746	1.7501	2.2194	2.5982	2.3408	1.9008
2.2054	1.9253						
CASKIN2	2.3609	2.364	2.9763	1.8742	2.4367	2.4962	1.752
2.4109	1.8731	2.053	1.7496	1.3828	2.7323	1.5045	2.2575
2.6173	2.7852	1.961	1.5929	3.8161	3.5724	2.1166	1.9521
2.1856	2.2999	2.4802	1.2422	3.145	2.1249	1.3207	2.031
2.2448	1.8199	1.5917	2.25	1.4497	3.1128	2.7462	2.2197
1.708	2.0947	1.7121	1.8367	1.6978	2.2483	1.6567	2.1519
2.1976	2.4316	2.0218	2.2365	1.2104	1.7269	2.7663	2.994
2.5082	1.9846	2.9215	2.2549	2.8316	2.1059	2.049	2.0794
2.2454	2.8373	2.302	2.4768	2.3434	2.1929	2.785	2.0131
2.0043	2.6006	0.9239	2.4036	3.1714	2.7095	1.7614	1.8306
1.4164	2.195	2.1298	2.6037	2.745	2.648	1.9349	1.9447

2.1017	2.0392							
CASP1	0.8281	0.2878	0.1303	0.7676	0.2658	2.1904	5.8864	0
	1.728	2.5528	2.1295	2.6796	3.9252	5.9655	0.1576	
5.7892	2.0309	4.2323	2.4829	0.0534	0.219	3.1671	3.3724	
1.4712	1.015	3.556	0.4614	3.6975	1.0206	0.1162	0.1847	
3.7545	5.1227	3.6105	1.32	2.2374	1.9657	1.1203	0	
0.5885	2.5417	4.6036	0.4502	3.41	0.9992	4.8433	0.3672	
1.191	3.7114	2.9692	3.3587	4.5835	3.0651	2.2942	0.1464	
2.0428	3.9707	0	1.1776	0.2422	2.5729	2.1475	0.5358	
5.8064	0.2443	4.8315	1.2892	1.0183	3.2416	0.8302	3.749	
1.37	4.9968	2.5725	0.117	1.4148	3.5405	0.4088	4.3745	
1.9737	0.2187	2.212	3.5686	0.1167	3.0346	3.324	4.1858	
3.5057	3.7264							
CASP10	1.3515	1.303	0.0121	0.8409	0.6595	0.1565	1.5098	
0.0816	1.0159	1.3024	0.991	2.0026	1.6581	1.2031	0	
0.8059	0.0692	1.69	1.7908	0.2847	0.0043	0.4	0.9056	
1.7079	0	0.63	0.6605	0.1781	0.6387	1.8781	0	
0.8487	0.776	1.5326	1.0929	0.6712	1.0097	1.1093	0.4311	
1.5722	1.491	2.4934	0.0267	0.0084	0.5679	0.9705	1.0935	
0.6246	1.0911	0.0537	1.3915	1.4059	1.7045	1.0178	0.5838	
0.6244	1.8527	0.1913	0.7295	0.3307	0.3904	1.2341	1.185	
0.1373	0.1231	0.2065	0.8945	0.1563	1.0652	0.1867	1.4774	
0.1913	0.3	1.3988	0.1341	2.4987	1.7423	1.5727	1.3053	
0.3995	0.3792	0.3843	0.8438	0.2294	0.183	1.4224	0.5633	
0.1914	0.0048							
CASP12	0	0	0	0	0.029	0	0	0
	0.0479	0	0	0	0	0	0	0
	0	0	0	0	0	0.0229	0.0288	
0.0305	0.0901	0.0981	0.1622	0	0	0.0546	0	0
	0	0	0.0809	0.0299	0	0.0668	0	
0.4379	0.2795	0.0482	0	0	0	0	0	0
	0	0	0	0	0	0	0	0
	0	0	0	0	0	0	0.0652	0
	0	0	0	0	0.0722	0	0.1161	0
	0	0.0455	0	0.2037	0	0	0	
0.1815	0	0	0	0.0902	0	0	0	0
	0							
CASP14	0.0375	0.0191	0	0	0.0104	0.0291	0.0189	
0.0375	0.0111	0.0138	0.0245	0	0	0	0.0067	
0.0068	0	0.0267	0	0	0	0.0104	0.0182	
0.0311	0	0	0.0496	0	0	0	0	
0.005	0	0	0	0	0	0.0246	0.021	0
	0	0.0026	0	0.0174	0.0077	0	0	0
	0	0.0545	0	0.0181	0	0.0251	0	0
	0	0.0312	0.029	0.0129	0	0.0173	0	0
	0.0344	0	0	0	0	0	0	
0.0078	0.0121	0	0.0198	0.0127	0.01	0	0.0016	
0.0247	0.0375	0.0138	0.0182	0.0395	0.0362	0.0354	0	
0.0287	0.0356							
CASP2	3.9768	3.8704	3.8643	3.6249	3.3787	5.2402	3.4636	

4.766	3.5776	3.578	3.1026	3.1023	3.6013	3.9576	4.3139	
4.7693	2.7108	4.7412	4.4321	3.5078	3.902	3.5038	3.1791	
4.0794	3.7875	3.755	3.1175	3.8221	4.0348	3.3329	4.1715	
3.7243	3.2845	3.2	4.0427	3.7406	3.7219	3.2168	3.5082	
3.7209	2.8089	2.5294	3.5654	4.5469	3.6514	3.9552	3.4705	
3.9987	3.2379	2.6636	3.3517	3.7586	4.3286	2.9113	3.7668	
3.2661	2.5275	4.0015	3.5451	4.2917	3.6776	3.9913	4.4744	
4.3641	4.241	2.4296	3.5741	3.4099	3.5615	4.5975	3.4247	
3.7939	4.3229	3.9205	3.4758	3.9985	3.4741	3.9852	3.8651	
3.4121	3.5516	2.4232	3.3622	3.3221	4.2727	3.9868	3.2363	
3.8938	3.5922							
CASP3	3.9415	3.6887	4.695	4.8454	3.9791	4.5111	4.4689	
3.3461	3.7966	3.9066	2.8695	4.1194	3.5605	4.2893	3.5247	
3.2285	4.1789	4.0225	4.0977	3.5427	3.3856	3.8023	3.8204	
3.308	3.6532	5.2779	3.0657	4.5589	3.3663	3.1902	2.9314	
4.0031	4.107	3.4045	4.0459	4.1108	3.8486	4.5674	4.2026	
3.372	4.1112	4.142	4.2697	4.2951	3.7608	4.4542	4.4227	
3.7002	3.4144	5.8262	3.3913	4.16	3.9423	3.6697	3.6587	
2.953	5.6548	4.0858	3.7871	4.0846	4.0478	3.6723	4.0874	
4.2251	4.2909	4.0061	3.0973	3.7187	4.2264	4.3475	4.0564	
4.4442	4.0591	3.0162	4.312	3.639	4.2011	2.928	3.3595	
3.9766	3.9586	4.7302	2.8201	4.6506	3.4479	3.8276	3.9375	
4.449	4.6529							
CASP4	3.8437	3.0424	3.4393	5.2323	4.4524	4.5413	6.3911	
2.3237	4.7961	2.5286	2.6172	3.8326	5.7493	5.3986	3.8179	
4.2457	6.6196	4.3327	3.8827	4.3494	4.2176	4.4232	4.626	
3.2054	3.9418	5.4868	4.2405	3.1323	4.477	2.8403	0.999	
4.0263	4.6333	4.6832	3.2551	2.9971	6.0648	4.4695	2.5644	
4.5459	6.0548	5.4033	3.7954	4.7213	4.0869	6.3645	3.759	
5.07	4.6991	5.101	4.8135	5.5458	4.8268	5.1709	2.9504	
3.7985	6.7105	3.2628	3.5615	3.7888	4.6043	3.3496	5.6031	
5.2156	0.8818	4.4364	2.7285	3.2765	3.1231	4.4842	4.2051	
4.0855	5.1566	4.0438	2.4706	4.9621	3.9574	2.5726	5.0775	
4.3986	4.4078	4.5736	6.0637	4.6613	5.5123	4.7053	4.9763	
4.9476	4.4051							
CASP5	0.0532	0	0	0	0	0	0.0687	0
	0	0	0	0	0	0	0	
0.0976	0	0	0	0	0	0	0	0
	0.0115	0.0691	0	0.0782	0	0	0	0
	0	0	0.0135	0	0.663	0.0547	0	0
	0	0	0	0	0	0	0.2772	0
	0.033	0.1196	0.0396	0	0	0.0836	0	0
	0	0	0	0	0	0	0	0
	0	0.0576	0	0.058	0	0	0	0
	0	0	0	0	0.0816	0	0	0
	0	0	0.0979	0	0	0	0.1074	0
	0							
CASP6	2.4695	2.7549	2.5894	3.4969	2.5205	3.3899	2.6509	
2.3785	2.4552	2.6564	2.1395	3.0456	2.0806	2.7195	2.2861	
2.3994	2.3511	2.6202	2.458	2.4018	3.189	2.5611	2.0746	

2.483	3.0535	2.7081	2.0411	3.3549	1.4156	2.4468	4.1266
2.4347	2.5421	2.3535	2.2562	3.2431	2.7821	2.5981	1.9989
2.4321	3.1689	4.2539	2.7321	2.4305	2.6606	2.9964	2.2202
2.8395	1.8686	2.6682	3.0862	2.8722	2.4412	1.3256	3.5322
2.2202	2.8131	3.3801	2.4384	3.4799	2.6251	2.3926	2.1893
2.3671	2.9115	2.2262	1.9379	3.8988	2.4667	2.4676	2.864
2.3305	2.4239	2.2297	2.8629	2.6074	2.8054	3.1514	2.3008
3.0524	2.736	2.7831	1.8767	1.6522	3.5248	2.8972	3.0574
3.2605	3.3514						
CASP7	1.8611	2.8207	2.9388	2.8489	3.3733	3.564	3.898
2.2809	3.2571	1.7806	2.4938	3.3517	3.4153	3.0767	3.3771
2.194	2.3938	2.7	2.3227	3.4002	2.738	2.1861	3.0233
2.9534	2.7496	2.9437	2.9064	3.189	2.3321	1.6217	2.9937
2.5892	3.2107	3.0167	3.1469	2.9652	3.0182	2.5409	1.7555
2.8147	2.5795	3.8109	3.5263	2.4537	3.0976	2.738	3.2364
3.2027	3.7797	3.0481	2.8887	2.769	2.3606	1.9464	3.9397
2.3505	2.2596	3.1137	2.0588	2.9112	2.8277	2.5772	2.8831
3.5783	3.3095	3.1404	2.0276	3.3427	2.3048	2.9964	2.9236
3.0626	2.5092	2.5485	2.7154	2.8083	3.2712	2.5174	2.393
1.7156	2.5179	3.3634	2.1787	3.0767	3.2798	2.3241	2.3444
2.817	2.8884						
CASP8	3.0471	2.3959	2.5561	3.8097	3.1428	4.3631	4.1293
3.067	3.1869	2.442	3.8468	3.8403	4.0862	3.1071	0.2382
3.291	4.1217	4.0853	3.9762	2.8723	2.5382	2.6805	3.8727
3.5125	3.4166	3.3469	2.5328	2.7756	3.0405	2.761	2.6868
3.5849	4.0772	3.3437	3.4658	3.3537	3.3188	3.029	2.6746
3.9766	3.5687	4.7825	3.7735	3.1456	3.2784	3.0609	3.1451
3.9665	4.2261	3.8123	3.369	4.0924	3.6038	3.0822	2.998
2.0587	4.2322	1.1466	4.0696	1.6929	3.9064	2.7499	3.3964
3.7157	3.192	2.7672	2.6884	2.6335	3.8956	3.4832	4.5224
2.967	3.041	3.4594	3.5275	3.1269	3.2811	3.5263	4.0237
2.8958	3.5286	3.6458	2.8471	3.8801	3.4313	3.4108	2.5213
2.6964	2.7589						
CASP8AP2	2.2515	2.3789	2.5391	2.8306	2.3711	2.2994	2.3084
2.0606	2.5723	2.5268	1.7477	2.1265	2.3136	3.2157	2.6073
1.4637	2.5062	2.7789	3.5713	2.3775	2.5255	3.0476	2.6418
2.2827	2.951	2.5468	2.4924	2.0573	2.6046	2.1795	3.0795
2.1479	2.152	2.0827	2.3534	2.6729	2.029	1.4514	2.3477
2.1678	2.4294	1.8589	2.713	3.5539	2.8188	3.1202	2.9518
3.0452	2.3599	3.1935	2.6971	3.1331	2.9384	2.5682	2.8719
2.8637	2.9317	2.7863	2.6431	2.9218	2.0615	2.5567	2.7411
2.402	1.9243	1.7237	2.3033	1.1951	2.9144	2.3358	2.2463
3.1983	2.3145	3.1542	2.1437	1.5607	2.685	2.0311	2.1603
2.6111	2.5424	2.1866	2.1872	2.4429	2.3958	3.106	2.0584
2.6051	2.5016						
CASP9	1.8712	2.8864	2.0266	1.8219	2.8134	1.5709	1.9768
2.1263	2.8809	2.4277	2.2578	2.5695	1.1594	1.9439	1.8248
2.1206	1.7877	2.2916	1.4712	2.8706	2.047	2.1828	2.4057
2.1801	1.3886	1.758	2.0625	2.7857	2.2689	3.0723	4.2201
2.7514	2.8125	2.3913	2.3748	2.5337	2.5998	2.4782	2.0266

1.9004	2.5657	2.3007	1.7172	1.6408	1.9902	1.8647	2.0481	
2.4186	2.1785	1.8213	2.1289	1.8166	2.1635	2.1394	3.1393	
1.288	2.2884	2.3778	2.5317	2.3538	1.6638	3.5822	1.6275	
2.1128	1.4275	1.8647	1.9528	1.4795	2.7255	2.0515	1.519	
2.0576	2.195	2.0916	1.6575	2.4305	2.515	2.0156	3.1543	
2.5338	2.6007	1.7802	1.5764	1.5144	2.4992	2.3411	2.1835	
1.7573	2.0949							
CASQ1	0.0747	0.1712	0.1631	0.0428	0.0911	0.0373	0.1154	
0.2743	0.1181	0	0.1892	0.0429	0.0453	0	0	
0.0125	0	0.0353	0.1032	0	0	0.0072	0.0186	
0.0563	0.0488	0.0467	0.0266	0.2253	0.0211	0.7154	0.0858	
0.0548	0	0	0.0139	0.017	0.0231	0.0751	0.0135	
0.1033	0.0836	0.0153	0.0599	0	0.0134	0.0221	0.2148	0
	0	0	0.0905	0.0467	0.1759	0.0573	0.1234	0
	0	0.1161	0	0.0249	0	0.1365	0.2249	
0.0358	0.0651	0.3435	0.1407	0.0547	0	0	0.1846	
0.0185	0	0	0	0	0.053	0	0.006	
0.099	0.3497	0	0.3414	0	0	0.062	0	0
	0							
CASQ2	0.4971	0.147	0.0514	0.0419	0	0.1058	0.0647	
0.0642	0.4998	0.2135	0.2722	0.1209	0	0.6418	0.2613	
0.2546	0	0.5372	1.1437	0.0237	0.1611	0.2242	0	
0.2027	0	0.0131	0.0091	0.0288	0.2722	0.0702	0	
0.0708	1.0051	0.8017	0.1178	0.2656	0.0428	0.053	0.587	
0.1669	0.6136	0	0.0384	0.5196	0.0099	0	0.2373	
0.3895	0.005	0	0.0057	0.1573	0.7361	0.1676	0	
0.1871	0	0.038	0.0334	0	0	0.105	0.0513	
0.197	0.1053	0	0.1792	0.04	0.075	0.0861	1.0062	
0.5334	0.9549	0.1021	0.0617	0.106	0.1155	0.2021	0.7036	
0.8971	0.5292	0.2225	0.0269	0.4528	0	0.5518	0.5787	
0.4507	0.4083							
CASR	0	0	0	0	0	0	0	0
	0	0	0.0021	0.0045	0	0	0	
0.0027	0	0	0	0	0	0	0	0
	0	0	0	0	0	0	0.0226	0
	0	0	0	0	0	0	0	0
	0	0	0	0	0	0	0	0
	0	0	0	0	0	0	0	0
	0	0	0	0	0	0	0	0
	0	0	0	0	0	0	0	0
	0	0	0	0	0	0	0	0
	0	0	0	0	0	0	0	0
	0							
CASS4	0	0.0568	0.1846	0.1341	0.0794	0.0694	0.4959	
0.1075	0.046	0.0412	0.071	0.0302	0.0707	0.0599	0.1095	
0.0543	0.0595	0.4032	0.0774	0	0	0.033	0.6346	
0.0481	1.2445	0.393	0.0339	0.0802	0.3269	0.0842	0.0419	
0.0232	0.1768	0.0155	0.073	0.0237	0.062	0.0394	0.1904	
0.0306	0.0999	0.1495	0.204	0.0181	0.1417	0.7227	0.0448	
0.682	0.0261	0.2281	0.1096	0.0629	0.1149	0.0305	0.0871	

0.1961	0.2959	0	0.245	0.0973	0.0014	0.0571	0.1211	
0.168	0.0454	0.0409	0.1249	0.0425	0.0095	0.0468	0	
0.1621	0.0289	0.2437	0.0555	0.2034	0.0707	0.0291	0.1045	0
	0.2875	0.0973	0.282	0.0299	0.3268	0.0912	0	
0.0765	0							
CAST	5.6067	4.9336	5.4044	5.9595	5.6759	5.6739	5.4993	
4.8635	5.3558	6.0048	5.6516	6.0219	7.1608	6.0743	5.3288	
5.4093	5.5466	5.6342	6.3569	5.9401	6.0182	4.1451	5.4859	
6.0157	4.9376	5.8613	6.0206	5.5884	5.0609	5.7087	5.4642	
6.0888	5.0992	5.9109	5.4633	6.277	5.5644	5.5979	4.6433	
5.4449	5.3769	6.8915	4.8	5.9343	6.2084	6.2482	5.5949	
5.1281	5.5215	5.3479	5.8052	5.5599	5.5755	5.1746	4.8765	
4.5371	6.2841	5.0916	5.926	5.1391	5.6676	5.7196	5.4745	
5.6132	4.8084	5.6999	5.1256	6.2896	5.1347	5.8368	6.2802	
5.5818	5.2812	5.1256	5.4118	6.2028	5.7416	5.7683	6.2659	
6.5527	5.9446	6.0576	7.1999	4.825	6.1123	5.3862	6.2084	
5.3964	5.77							
CASZ1	1.07	1.3929	2.2381	0.0714	0.4684	0.2123	0.5165	
0.0258	1.2409	0.6256	0.0398	0.2208	0.63	0.0667	0.3714	
1.0502	0.8036	0.6131	0.5406	1.3779	0.6685	0.2358	0.1332	
0.66	0.2407	0.0702	0.0885	1.7579	0.368	0.7585	1.8498	
0.2772	0.1764	0.0846	0.129	0.7223	0.3903	0.976	0.2429	
0.2129	0.1998	0.0786	0.1766	0.0954	0.8601	0.8022	0.7492	
0.2055	0.0966	0.294	0.0869	0.086	0.0973	0.3779	1.8772	
1.1739	0.3302	0.266	0.0924	0.1459	0.1415	0.1676	0.2241	
0.7604	0.8453	0.3672	2.2506	0.8658	1.9032	0.3699	0.0871	
0.1134	1.9095	0.0727	0.2209	0.6143	0.8185	0.2103	0.4741	
0.545	0.9789	0.0888	0.396	0.2374	0.0649	0.101	0.2003	
0.3695	0.1675							
CAT	4.7849	5.8224	4.5883	5.3545	5.2229	5.1413	4.9192	
3.6684	5.3327	5.2024	5.7097	4.4379	5.3788	4.87	5.4541	
5.5778	4.212	5.9081	5.3976	4.9628	5.3458	4.3749	5.5088	
5.1759	5.8455	3.9031	5.2665	5.1799	4.9849	4.5049	3.8111	
4.9558	4.2805	4.3419	5.2004	5.15	4.6552	4.0438	5.0735	
5.1065	5.0809	6.2656	5.3488	5.7812	4.1069	5.3004	4.6333	
5.34	3.7856	4.6479	5.7971	5.0622	4.5533	4.8862	4.3505	
5.163	4.9613	4.3778	5.5131	3.9713	5.0333	5.696	4.2862	
4.6861	4.3159	5.4393	3.6902	5.1925	5.3567	4.3531	5.2078	
4.8671	4.716	6.0815	5.5933	5.498	4.5343	4.9829	5.4988	
5.3302	5.2503	5.7155	5.3509	5.1426	4.1878	4.2619	5.3704	
4.66	4.6191							
CATIP	0.1488	0.0533	0	0	0.0184	0	0.0753	
0.0372	0.0375	0.0424	0.1708	0.0219	0	0	0.0304	
0.0317	0	0.0722	0	0	0	0	0.0409	0
	0	0.031	0	0.1504	0.1123	0	0.6146	
0.1103	0.0497	0	0	0.2402	0.0532	0	0	
0.2774	0.0988	0.2011	0	0.0503	0	0	0	
0.0542	0.0703	0	0.0424	0.0523	0	0.1012	0.0521	0
	0	0	0	0	0.0741	0.0323	0.0363	0
	0	0.217	0.2932	0.012	0	0	0.0357	0

	0	0.0412	0.1372	0	0	0.1695	0	
0.3321	0	0.0832	0.1078	0	0	0	0	0
	0							
CATSPER1	0.8619	0.1368	0.395	0.4384	1.5615	0.0356	0.2389	
0.5784	0.0142	1.262	0.1321	0.1251	0.5133	0.1956	0.1656	
0.0263	1.4299	0.1415	0.1219	0.2727	0.3173	0.3123	0.5561	
0.2695	0.4924	0.0485	0.2327	0	0.7019	0	0.0238	
0.0646	0.281	0.1787	0.4255	1.1715	2.2941	0.1145	0.0755	
0.098	0.0485	1.8567	0.7512	0.5932	0.1908	1.088	0.0376	
0.1363	0.0651	1.4831	0.4731	0.4996	0.0926	0.3748	0.0629	
0.0679	1.8183	0.0333	1.256	0.1515	1.0003	1.3919	0.5756	
0.3796	0	0.058	0.0496	0.0921	0.1442	1.7213	0.0845	
0.6908	0.0303	0.1366	0.3541	0.2185	0.072	0.1149	0.1996	
0.3935	0.0496	0.2592	0.155	0.2016	0.2848	0.1751	0.1226	
0.0804	0.0934							
CATSPER2	0.9525	1.34	0.8004	0.2214	0.3173	1.2516	0.735	
1.0431	1.4498	1.7908	0.7278	0.3862	0.852	1.2361	0.363	
1.3924	1.8057	1.1466	1.2885	0.4618	1.0513	0.2395	0.4112	
1.0437	0.5953	0.55	0.734	0.5047	0.4039	0.7412	0.8045	
0.4818	0.5468	0.8767	1.014	1.1267	0.8798	0.6436	0.3746	
0.9757	0.4199	1.0324	0.4746	1.6273	1.553	0.86	1.3323	
1.0881	0.5323	1.0362	0.6707	0.5283	1.3099	1.2995	0.6003	
1.8773	1.0665	0.365	1.341	0.1638	0.7867	1.2802	0.3313	
1.1581	0.3702	0.2642	0.6948	0.8434	1.1179	0.2119	0.3829	
1.1689	1.0694	0.5642	0.779	1.0902	0.8583	0.3183	1.1798	
0.9126	1.1605	0.7362	1.0184	0.5941	0.3003	1.2822	0.4813	
0.6021	0.5843							
CATSPER3	0.2774	0.2788	0.361	0.2215	0.0926	0.0645	0.0302	
0.0615	0.0357	0.0749	0.0645	0.0209	0.2997	0.0719	0.1695	
0.0085	0.6195	0	0.2959	0.1355	0.0739	0	0.0191	
0.2392	0.0479	0.1623	0.0591	0.1299	0	0.0422	0.3438	
0.1115	0	0.0787	0.0783	0.1286	0.0717	0.0506	0.0788	
0.0739	0.0991	0.1937	0.2161	0	0.3528	0.7079	0.1514	0
	0	0.8851	0.0396	0.1674	0.133	0.1873	0.4352	0
	0.2562	0.2479	0.1393	0.2183	0	0.1285	0.2611	
0.1233	0.4862	0.0995	0.0671	0.1624	0.1668	0	0.0241	0
	0.0858	0.0386	0	0.0888	0.5354	0.1187	0.0498	
0.0704	0	0.1541	0.2349	0.0763	0.0065	0	0.13	
0.1041	0.0293							
CATSPER4	0	0	0	0	0	0	0	0
	0	0	0	0	0	0	0	0
	0	0	0	0	0	0	0	0
	0	0	0	0	0	0	0	0
	0	0	0	0.0566	0	0	0	0
	0	0	0	0	0	0	0	0
	0	0	0	0	0	0	0	0
	0	0	0	0	0	0.1083	0	0
	0	0	0	0	0	0	0	0
	0	0	0	0	0	0	0	0
	0	0	0	0.0792	0	0	0	0

	0							
CATSPERB	0.072	0.0205	0.277	0.0475	0	0	0.2326	0
	0.0119	0.082	0.0156	0	0	0	0	
0.124	0	0.0264	0.0727	0	0	0	0	0
	0	0	0	0	0	0.4874	0	
0.0744	0.0215	0	0	0.0472	0	0.0187	0	0
	0	0.0104	0	0	0	0	0	
0.047	0	0	0	0.1981	0	0	0	0
	0.0481	0	0.134	0	0.1963	0	0	
0.0481	0	0	0	0	0	0	0	
0.1532	0.2979	0.0018	0	0	0.0038	0	0	0
	0	0.1081	0	0	0	0	0.0505	
0.0589	0							
CATSPERD	0.1471	0.033	0.0527	0.1433	0.0236	0	0.0817	
0.0203	0.086	0.0754	0.0453	0.04	0.0382	0	0.0935	
0.0748	0.1419	0.0772	0	0.2341	0.05	0.1088	0.0516	
0.119	0.0762	0.0313	0.0552	0.0577	0.1045	0.095	0.0193	
0.066	0.0331	0.0263	0.0178	0.0564	0.0312	0	0.0195	
0.0746	0.1425	0.0188	0.18	0.0317	0.0738	0.0277	0.0679	
0.1545	0.0934	0.1397	0.0257	0.0315	0.1008	0.0362	0.0391	
0.128	0.0211	0	0.0602	0.0147	0.1887	0.0853	0.1071	
0.0516	0	0.0367	0.0631	0.0103	0	0.1199	0.1039	
0.0573	0	0.164	0.0697	0.099	0.1851	0.1151	0.0809	
0.1538	0.09	0.0203	0.1317	0.139	0.1132	0.069	0.1258	
0.0784	0							
CATSPERG	0.0646	0.1762	0.1255	0	0.0619	0.0803	0.159	0
	0.0638	0.0439	0.1012	0.0754	0.1675	0.1449	0.133	
0.2256	0.1877	0.029	0.0921	0.1233	0	0	0.0369	
0.1757	0.0365	0.1257	0.0396	0.2293	0.042	0.1058	0.3658	
0.158	0.0346	0.2583	0.0497	0.0523	0.1497	0.0391	0.1072	
0.0341	0.3843	0.0834	0.1094	0.0555	0.0927	0.0494	0.0294	
0.0571	0.0651	0.1851	0.0298	0.0852	0.044	0.0524	0.0947	
0.1034	0.0862	0	0.2213	0.0171	0.0723	0.07	0.01	0.1
	0.1917	0.2684	0.2688	0.0257	0.1367	0.0586	0.0491	
0.0892	0.0165	0.0288	0.1677	0.0156	0.2024	0.0686	0.0886	
0.1512	0.0555	0.0354	0.2775	0.0167	0.077	0.0771	0.0419	
0.0449	0							
CAV1	3.7868	3.7594	2.8298	5.5327	5.2965	8.1093	6.6937	
3.207	5.4796	4.0714	5.2002	5.4513	8.8087	6.6423	6.6082	
7.6169	6.9924	6.9322	4.4656	5.4424	4.4859	6.2766	6.4052	
5.7034	5.4767	8.7978	4.9474	5.1913	7.9774	4.6424	0.9597	
4.4262	6.2766	5.1874	6.7957	3.2172	3.2182	3.6546	5.3325	
6.0169	5.4434	8.4827	7.4741	6.1861	7.5166	7.6665	4.0639	
4.2523	7.6257	7.9189	4.7759	6.3532	4.2282	4.3083	5.5097	
1.0014	7.2824	1.8291	4.6052	1.66	6.5952	5.1046	5.1732	
7.3004	6.0276	7.3209	1.2112	7.0458	5.1233	7.5878	7.8196	
6.503	5.4456	5.2375	7.227	5.2232	4.5136	6.277	5.5919	
5.5404	4.764	6.9774	3.573	6.6038	7.6682	4.5191	6.3737	
5.0565	5.5273							
CAV2	3.2183	2.9463	2.8512	3.5346	2.8332	4.2416	4.7556	

2.8166	4.1893	3.5956	4.0771	3.7504	5.3272	4.1034	4.3363	
5.0951	3.5365	5.3553	3.6185	3.9654	3.2315	4.3042	4.6308	
3.3194	4.0044	5.394	4.4238	4.3834	4.8637	3.862	1.5722	
3.0052	3.5132	3.9042	5.0233	3.19	2.5627	3.4991	3.0778	
3.8119	4.3006	5.2998	4.858	4.2932	4.1025	5.3413	2.8265	
3.7508	5.7443	5.047	4.1501	3.8005	2.9207	3.2971	3.7464	
3.1511	5.141	2.2976	3.9738	2.6791	3.769	3.223	2.5122	
4.0042	4.1074	4.4902	4.0947	5.0931	3.8146	5.4609	5.3439	
4.1851	3.7823	3.4145	4.5294	3.749	3.3373	4.9063	3.8214	
4.1122	3.1153	5.0436	2.652	4.7206	5.0919	2.9795	4.265	
3.3663	3.8354							
CAV3	0	0	0	0	0	0.0635	0	0
	0	0	0	0	0.0545	0	0	
0.0263	0	0	0	0	0	0	0	0
	0	0	0	0	0.0329	0	0	0
	0	0	0	0	0	0	0	0
	0	0	0	0	0	0	0	0
	0	0.1132	0	0	0	0.0682	0	0
	0	0	0	0.0505	0	0	0	0
	0	0	0	0	0	0	0	0
	0	0	0	0	0	0	0	0
	0	0	0	0	0	0	0	0
	0							
CBFA2T2	2.8727	2.6538	3.2366	2.0142	1.8986	2.5776	3.508	
3.2281	3.0884	3.2077	3.299	2.5015	3.0016	2.5109	2.9069	
3.0032	2.3963	3.0592	2.9178	2.3878	2.4829	2.6097	3.1005	
2.6617	3.482	3.2495	3.705	2.5776	2.5373	3.1929	2.8144	
2.3936	2.4382	3.3878	2.8428	2.9579	1.9673	2.5534	2.3652	
2.9455	2.6682	1.8672	2.0659	2.3386	3.2607	2.7869	2.8976	
2.6815	3.5893	2.7717	2.8106	2.6991	2.9751	2.9228	2.9874	
3.1719	2.0974	2.3312	3.0407	2.7917	2.2947	3.6162	2.2645	
2.6252	2.2198	2.2731	2.5882	2.4849	3.1372	2.2462	3.1023	
2.5852	3.1268	2.334	3.3539	3.2628	3.1395	2.6439	2.4669	
3.0375	2.8918	3.0645	3.0222	2.9962	1.9639	2.4956	2.5155	
1.9319	2.4095							
CBFA2T3	0	2.554	3.0996	0	0	0	0	
0.0487	0	0	0.0743	0	0.0852	0	0	0
	0	0.6011	0.4351	0	0.109	0	0	
0.0439	0	0	0.0597	0.081	0.052	1.7444	2.1677	0
	0	0.0532	0.0454	0.5706	0.0388	0	0.0685	0
	0	0	0	0	0.0819	0	0.1116	0
	0	0	0	0	0.6422	0.7229	1.6899	
1.7615	0	0	0	0	0.0788	0	0	0
	1.7355	0	0	0.0942	0.8298	0	0	
0.0797	0.5975	0	0	0.0153	0	0	0.3594	0
	0	0	0.0317	0	0	0.3848	0.0947	
0.067	0							
CBFB	5.0814	4.8635	6.0385	5.8149	5.3296	5.9114	4.8976	
5.5908	5.5127	5.6577	4.0804	4.7075	5.139	6.6374	5.3935	
5.7867	4.4634	5.8885	5.7023	6.0106	5.9878	5.8885	4.9286	

5.7479	6.361	4.1772	5.6033	5.7513	5.3783	5.055	5.5479	
5.426	4.9621	4.6553	5.8	5.8398	5.2874	4.3647	4.2884	
5.3703	5.0952	4.6893	5.6114	6.2825	5.5819	5.6782	5.5573	
5.8316	4.7217	5.1084	5.3789	5.3756	5.6439	4.9352	5.7347	
5.7244	5.6889	5.5508	5.6858	5.9515	5.7853	5.4856	6.073	
6.0168	5.5438	3.6028	5.1145	5.5866	5.567	6.2784	5.4102	
6.2649	5.8466	4.4218	5.4588	6.2132	5.3545	5.0326	5.5276	
5.2917	5.143	4.7335	5.1774	5.6552	5.3463	5.3232	5.2358	
5.5752	5.5367							
CBL	3.2509	2.4133	3.214	3.6644	3.776	2.6972	3.3245	
3.0704	3.7882	3.0517	3.6192	3.173	3.6356	3.5671	2.96	
3.4827	4.0747	3.1685	3.08	3.2106	2.6605	3.9038	3.2289	
2.9226	2.1948	3.1549	3.5865	2.1128	3.2536	2.6859	3.6439	
2.3288	3.1197	3.6921	3.272	3.2402	2.663	2.6239	3.051	
3.3967	3.2688	3.1021	3.638	3.63	4.0176	2.8009	3.0871	
3.9046	3.238	4.1073	3.2925	3.2662	2.8162	3.5055	3.4308	
3.2191	3.9792	3.3756	3.3064	3.4081	2.3367	2.7238	2.8941	
3.3549	2.8312	2.8654	3.3994	3.3718	3.1272	3.4259	2.5783	
3.3722	3.4299	3.2661	3.0446	3.8427	2.7676	2.2701	3.0446	
2.6786	3.0322	3.3531	3.7105	3.6596	2.5931	1.9279	3.2376	
3.0893	3.2219							
CBLB	2.4535	3.6133	2.7907	3.1133	1.9177	3.6498	4.3253	
2.3068	2.2591	1.3668	2.4185	2.1507	3.371	2.842	1.8593	
2.6001	3.028	3.3158	2.019	2.482	2.9791	1.7962	2.818	
2.1851	1.4879	3.0713	2.4191	2.8102	1.993	4.3344	1.7818	
2.5177	2.1826	1.9748	3.0327	2.1046	3.4438	2.6745	2.4825	
4.0149	2.4222	2.7504	0.8943	2.0276	2.0093	3.4913	4.7912	
3.0854	1.8643	3.9492	4.0667	1.7303	2.9982	3.817	2.5724	
2.765	2.2521	1.8415	2.0208	2.5097	3.1502	2.2531	1.4449	
2.6787	1.5141	3.6282	3.2267	2.7042	3.7005	2.1948	1.2937	
2.5147	2.3532	2.7816	1.7976	1.9984	3.1578	2.0777	2.6783	
3.1904	2.9577	2.4659	5.7907	3.607	2.4163	2.3281	2.9824	
2.1142	2.6254							
CBLC	0	0	0	0	0	0	0	0
	0	0	0.0227	0	0	0	0	0
	0	0	0	0	0	0	0	0
	0	0	0	0	0	0.0488	0	0
	0	0	0	0	0.0231	0.0495	0	0
	0	0	0	0	1.1527	0	0	0
	0	0.1307	0	0	0.0881	0	0	0
	0.1395	0.0665	0.0694	0	0.024	0	0	0
	0.0744	0.0056	0.2015	0	0	0	0	
0.1882	0.0562	0.0222	0	0	0.0253	0	0	
0.1132	0	0.1487	0	0	0	0	0.0505	0
	0							
CBLL1	3.2665	3.6668	3.6323	4.3097	3.4799	4.5572	4.1033	
4.3417	3.8144	3.7324	4.0419	3.1901	4.1484	4.2328	3.3415	
3.3719	3.245	4.4551	4.3024	3.1503	3.3411	3.8712	3.688	
3.6884	4.2145	3.4777	3.3411	3.6391	4.2194	3.9826	2.7642	
4.1116	3.3197	2.554	3.6413	3.8699	3.5581	3.5559	3.8423	

3.6245	3.7845	3.0341	3.5707	4.2432	3.4803	3.6402	3.5971	
3.5382	3.417	3.696	3.731	4.0802	3.2423	3.686	3.5682	
3.8174	2.4434	4.0464	4.038	3.9966	3.7832	3.9248	3.8897	
3.9601	4.0124	3.3154	4.0524	3.2193	3.6287	4.0565	3.5376	
3.961	4.3206	3.5592	3.4556	3.4347	3.7523	3.8865	3.4863	
3.6544	3.6663	3.3739	1.9292	3.4703	3.5595	3.239	3.0839	
3.1624	3.1745							
CBLN1	0	0.2801	0.247	0.1075	0.2007	0	0.2034	
0.1708	0.6007	0.2601	0.1225	0.1133	0.2479	0.1555	1.0659	
1.9261	0	0.0998	0.4171	0	0	0	0.0983	
1.3153	0.2382	0	0.0872	0.1607	0.1337	0.2025	4.1116	0
	0.246	0.0838	0.0764	1.2136	0	0	0.2015	
0.0815	0	0	0.4386	0	0.0721	0	0	0
	0	0	0.0816	0.2195	0	0.1404	1.1876	
1.6611	0	0	0	0	0.5102	0	0	
0.2289	0.0261	0	0.8538	0	1.2604	0.1627	0	
0.0654	0.4158	0	0.5513	0	0	2.3681	0.1999	
0.0366	1.7113	0.1979	0	0	1.3872	0	0.128	0
	0							
CBLN2	0	0	0.041	0.3784	0.0398	0.0332	0	
0.1758	0	0	0.0444	0.0074	0	0.4254	0	
0.011	0.6088	0	0	0	0	0	0.0491	0
	0.0692	0	0	0	0.0185	0	0.0953	0
	0	0.0412	0	0.0532	0.0521	0	0	0
	0	1.906	0	0	0	0.462	0	0
	0.0566	0	0	0	0.0428	0	0.0345	0
	0	1.0694	0.1022	0.3255	0	0.0112	0	0
	0.122	0.0528	0	0.0607	0.0057	0.0586	0.1666	
0.0462	0.3522	0	0.0375	0.0826	0.0017	0	0	0
	0.0424	0	0.0449	0	0.1961	0	0	0
	0							
CBLN3	0.2818	0.5009	0.5031	0.4193	0.2886	0.5093	0.7306	
0.684	0.7543	0.5243	0.3247	0.4583	0.2271	0.5417	0.4593	
0.6122	1.5131	0.7148	0.3408	0.6947	1.1785	0.4257	0.5813	
0.5477	0.1731	0.2625	0.4374	0.4247	0.6621	0.1696	1.1304	
0.5749	0.4145	0.4739	0.6126	0.5162	0.551	0.2419	0.1597	
0.6511	0.3774	0.2632	1.2518	0.2635	0.7219	0.9075	0.1127	
1.2518	0.5952	0.7418	0.3237	0.8966	0.3021	0.549	0.5607	
0.1706	1.2725	0.3233	0.1464	0.4448	0.5408	0.3423	0.1263	
0.4801	0.2438	0.5627	1.1074	0.8025	0.3461	0.523	0.2147	
0.44	0.5356	0.0407	0.618	0.9168	0.6906	0.7084	0.8385	
1.0418	0.8186	0.6444	0.4777	0.2489	0.4621	0.3413	0.5237	
0.4177	0.6065							
CBLN4	0	0	0	0	0	0	0.0302	0
	0	0	0.1041	0	0	0	0	
0.0294	0	0	0	0	0	0	0	
0.0327	0	0	0	0	0.2867	0	0	0
	0.6369	0	0.0327	0.0339	0	0	0	
0.0388	0	0	0	0	0	0	0	
0.0583	0	0	0	0	0	0	0	0

	0	0	0.0084	0	0.3276	0	0.0037	
0.1808	0.0131	0	0	0	0	0.0249	0	
0.1419	1.0567	0	0	0	0	0	0.0598	0
	0.0037	0	0	0.108	0	0	0	0
	0							
CBR1	5.2873	4.9879	4.6961	5.5651	5.1096	6.0406	6.4896	
5.4052	6.6357	4.9528	6.5476	6.091	4.8116	6.735	5.5589	
6.0719	4.8592	4.9116	5.4194	5.8304	5.6234	6.1313	6.1487	
6.7374	6.2008	5.0456	5.2541	5.6686	6.1917	5.1582	5.6905	
6.1602	6.0761	6.4333	6.2968	5.1473	4.9332	5.6807	5.3358	
5.9919	5.6625	5.9579	5.8014	5.7787	3.8191	5.0832	5.3111	
5.3676	4.632	4.3689	6.0431	5.3416	5.4787	4.5727	6.1596	
5.9584	5.4271	6.1513	5.5699	5.5976	5.4265	4.3631	5.247	
6.0238	5.1979	6.1654	5.8002	5.7026	4.1496	6.0228	6.2566	
5.3524	6.1034	5.3536	4.0802	5.2887	5.7723	6.3917	5.5466	
5.735	4.2657	5.1841	5.8122	5.1018	5.3416	6.1663	6.2022	
5.0199	5.4011							
CBR3	3.7374	4.1545	2.8424	3.9979	3.0102	3.9157	4.3655	
3.5654	5.586	3.3755	3.7848	3.2861	3.1335	6.2466	3.609	
4.6318	2.4677	4.4733	4.7447	3.8802	4.3421	3.9452	4.3193	
4.6785	3.8113	2.9374	2.9032	3.9713	4.2081	3.1026	0.9107	
3.6514	4.9424	4.0573	4.2759	3.9276	3.5327	3.0771	3.919	
3.6253	4.1308	3.7505	3.7333	5.26	2.463	3.4293	4.0051	
4.2952	2.7371	4.1077	4.9788	3.6582	4.4684	2.6564	2.9183	
4.5523	2.2418	3.5593	4.193	3.0464	4.2688	3.6696	4.0949	
5.3944	3.9912	4.6723	1.9809	2.4541	4.3842	4.9665	4.2762	
4.145	4.2972	3.0111	3.4853	4.332	4.8851	3.8823	4.5452	
4.4607	4.7972	2.7057	2.9689	3.8951	5.1332	4.9228	4.2407	
5.3058	4.9871							
CBR4	3.1929	2.4703	2.7546	3.0661	2.9841	3.9591	3.0708	
3.189	3.4118	3.8047	3.7068	2.9753	2.8346	3.1609	2.4271	
3.4917	2.4925	3.1021	2.9232	2.9902	4.0825	3.054	2.6528	
2.9312	2.4723	3.5649	3.6099	3.8894	2.2709	2.1503	2.1847	
3.0665	3.5763	3.4189	2.917	3.3713	2.0962	2.3851	2.877	
3.0435	3.1994	3.4786	2.8801	3.1477	2.9912	2.4348	3.2963	
2.5502	2.5049	3.3991	2.3708	2.7468	3.4385	3.3089	2.7056	
2.5461	3.706	2.9017	3.141	2.0707	3.1976	2.9791	2.3127	
2.7166	3.1365	3.6611	3.2499	3.0483	3.4248	2.9247	3.3911	
3.177	3.7346	3.0185	3.4758	4.046	3.0493	2.5759	2.6495	
4.3303	3.2914	3.0388	2.1731	3.7999	1.6185	3.2853	3.8449	
3.8882	3.6843							
CBS	1.9398	1.2349	1.1706	0.549	0.5019	0.2778	0.4781	
0.8031	0.1466	1.6144	1.4843	0.3208	2.0231	0.5851	0.2758	
0.5131	1.1675	0.33	0.2214	1.4489	0	0.5356	0.4503	
0.2619	1.2427	1.1207	0.2718	1.2272	0.2409	0.3136	2.4762	
0.4494	0.6896	0.902	0.536	0.4378	2.2351	2.4842	2.9919	
0.2962	1.147	0.1641	1.2585	1.167	0.2785	0.6457	0.5763	
1.3642	0.9779	1.1217	1.2057	0.486	0.7275	2.1607	2.6993	
2.0281	1.7158	1.6627	1.2366	1.4808	0.6355	0.6542	0.4714	
0.7629	1.9402	0.645	1.123	0.7077	0.2493	0.8295	0.7696	

0.342	0.8504	0.5948	0.6063	0.4485	0.0854	0.3698	0.1057	
0.104	1.6414	0.6141	0.1932	0.5124	1.1919	0.9783	1.2095	
1.4442	0.8273							
CBWD1	3.7727	4.1228	3.5592	2.6796	3.6965	2.7256	3.1076	
3.2039	3.0248	2.7367	3.0164	2.9738	2.8506	3.4284	3.1625	
2.4346	3.4883	3.6217	3.3139	3.3542	2.9685	3.2322	2.8595	
3.377	3.1127	3.3659	2.4118	3.0044	2.8028	2.6605	3.9747	
3.3889	3.7026	3.0488	2.4062	2.6237	2.9213	3.9345	2.82	
3.6928	1.9174	2.9258	2.3659	2.1937	3.4702	2.6447	2.5617	
3.4473	3.6252	3.6579	2.7623	3.2603	2.9322	2.9044	3.696	
2.841	2.6454	3.3299	2.8804	3.0857	2.309	4.4381	2.7108	
3.3715	3.4797	3.9347	2.9704	2.9906	3.823	2.6326	3.6465	
3.9079	3.0199	3.3489	3.5598	3.3772	2.8796	3.1029	3.0697	
3.2008	2.9906	2.4105	3.5549	2.9828	3.8747	2.5337	3.2738	
3.5133	3.3664							
CBWD2	1.4127	1.3838	1.5903	1.9428	1.6147	1.8069	1.1757	
2.5832	1.676	1.5698	1.6498	1.4205	2.3509	1.7927	2.1449	
1.3641	1.6995	2.3757	2.2168	1.2315	1.7895	1.0781	1.9249	
1.6428	1.5123	1.8951	1.8092	1.8458	2.1024	2.0216	1.4839	
1.0347	1.7281	0.8109	0.839	2.0482	1.3416	2.1898	0.74	
1.5662	0.9575	1.4559	1.3193	1.0305	2.8287	3.0115	1.8791	
1.3485	1.6639	2.828	1.9942	1.8405	1.9275	2.3445	1.4464	
1.3432	1.088	1.0839	2.4295	1.7158	2.2205	2.5533	1.4375	
1.4162	1.9871	2.1854	2.0385	2.0187	1.9176	1.2148	1.3034	
1.7144	1.4063	0.9731	0.9004	1.8792	1.8977	1.1004	1.3665	
1.7789	1.5564	1.4573	1.3733	0.4806	1.8582	2.2816	1.2948	
1.2103	1.1824							
CBWD3	0.3538	0.2694	0.1793	0.0546	0.0336	0.0522	0.0352	
0.2086	0.1627	0.0392	0.0338	0.1417	0.4175	0.3028	0.0347	
0.1226	1.0881	0.2771	0.3778	0.5364	0.1926	0.111	0.0337	
0.039	0.2819	0.0794	0.1039	0.058	0.0412	0.0597	0.3464	
0.0322	0.0596	0.0398	0.0325	0.3272	0.1112	0.1422	0.0363	
0.0402	0.0381	0.0327	0.0511	0.0431	0.0353	0.0405	0.2152	
0.0457	0.1068	0.687	0.6092	0.4605	0.3972	0.2335	0.6302	0
	0.5517	0.076	0.6594	0.0348	0.15	0.7843	0	
0.0795	0.3017	0	0.219	0.1046	0.4444	0.1773	0.0569	
0.1308	0.1352	0.1232	0.2413	0.0657	5.00E-04	0.0411	0.2124	
0.5069	0.4476	0.0127	0.0959	0.2062	0.1621	0.0678	0.1956	
0.6917	0.2796							
CBWD5	3.0209	2.921	3.3391	2.6498	3.6864	3.6647	2.1526	
3.5266	3.035	2.8282	2.9172	2.8742	3.5042	3.1239	4.0786	
3.4324	3.6102	3.0893	2.5362	1.9165	2.9525	3.7032	3.4733	
2.7462	4.3452	2.8227	2.3848	2.5473	2.9608	1.8267	3.5684	
3.1084	3.6156	1.556	2.2419	2.3178	3.3318	3.0451	2.7644	
2.893	2.3167	3.7115	2.4435	2.7682	2.3904	1.946	2.2885	
3.9756	4.4426	2.3963	2.8648	2.8809	3.2671	2.3683	4.0394	
2.0187	4.3925	2.9849	3.5802	2.9796	3.7564	2.3661	3.1754	
3.7679	3.1349	2.4034	2.0907	2.7863	3.3015	2.1553	2.8771	
3.1989	2.9964	3.5869	3.3492	3.035	2.2232	3.4144	3.256	
3.0952	3.7988	3.123	2.4535	1.9348	3.331	3.8749	2.4508	

2.3434	2.3686						
CBWD6	1.3937	1.1514	1.0996	0.7965	0.9264	1.1335	1.081
1.4579	0.9475	1.1052	1.0123	0.453	0.5803	1.8301	1.1047
1.1526	1.1841	1.6397	1.5646	1.3328	0.4922	0.4913	0.5518
1.0945	1.1593	0.6594	0.7042	1.1533	0.626	1.0578	1.1009
1.026	1.1212	0.5231	0.3682	1.2608	0.8041	1.2843	0.9377
1.6936	0.7159	0.4965	0.7717	1.3836	0.7407	0.8879	1.3495
1.0848	0.5912	1.3513	0.7456	0.9007	1.8723	1.1838	1.3972
1.0041	1.0233	0.4593	1.5407	0.6682	0.5226	0.7793	1.3185
0.6972	0.7731	0.7285	0.3916	0.8623	0.4407	1.0909	1.0691
1.1308	0.9244	0.7035	1.1432	1.2333	0.7939	0.5452	0.7768
1.0363	1.2107	0.7945	0.7644	1.2222	0.6919	2.0726	0.9032
0.8422	0.7091						
CBX1	5.1828	5.9615	5.9823	6.7744	5.2805	5.7187	5.1283
5.2014	5.5193	5.2546	4.8836	5.4703	4.7602	5.4001	5.3813
5.5509	5.6482	5.8074	5.5795	5.6014	4.9193	5.5309	4.9096
5.4045	6.1196	5.4165	5.7664	6.9397	5.2256	5.7461	6.1438
5.9189	5.3478	5.2419	5.5251	5.6395	5.4533	5.0121	4.7472
5.4102	5.1699	5.2152	6.1083	5.1297	6.2431	5.9742	6.1677
5.958	5.1265	6.3178	6.0821	5.5638	5.3343	4.5519	6.0817
5.4359	5.2845	6.0542	4.9396	6.0574	5.4579	5.4525	5.3336
5.27	5.9649	5.1688	6.1943	5.1929	5.7515	6.06	5.3145
5.3108	5.2114	4.8478	6.2827	6.0715	5.2866	5.8318	5.4625
5.5942	6.0749	4.6369	4.585	5.3833	5.948	5.5043	4.9504
5.3583	5.3336						
CBX2	2.8253	3.4109	4.0572	2.9444	3.2816	2.4777	2.2802
2.1148	2.3308	1.4317	2.9006	1.5735	2.4655	2.5873	2.9444
2.5601	1.8513	1.4181	2.0767	3.9334	4.0287	2.574	2.8197
2.5629	3.5303	2.1199	1.5535	4.5222	2.3253	2.0585	3.0382
2.7714	2.5669	1.6918	2.0716	1.8513	3.1849	3.2569	3.2167
1.832	2.2465	0.9774	2.229	3.3714	1.7731	2.1407	2.9283
2.9475	2.0511	2.1229	2.8442	2.1824	1.973	3.2873	3.7186
3.498	0.9454	3.9148	2.1086	4.0378	2.9671	2.1532	2.8682
2.8982	4.25	0.7329	3.7457	2.0867	2.9414	2.6239	1.9633
2.9397	3.3222	2.008	2.6999	1.4303	2.5628	2.4834	2.2985
2.35	2.9758	1.503	1.1124	2.7067	3.471	2.2057	1.2498
2.7937	2.1894						
CBX3	7.3624	7.16	7.1388	8.1355	7.4285	7.3223	7.1112
7.9335	7.6694	7.6562	7.2739	6.7254	7.0383	7.4654	7.3355
7.4502	6.8072	7.9541	7.6608	7.4778	7.4654	7.836	7.9487
8.0583	8.8657	6.8849	7.0498	7.4046	8.6068	7.169	7.2889
7.7918	7.5438	6.9856	7.6477	7.8812	7.7918	6.8444	7.6733
7.9168	7.4502	6.7236	7.8667	8.3593	6.949	8.1157	7.7688
7.9541	7.8914	6.5191	7.5127	7.9433	8.2769	7.4465	7.2503
7.9976	6.912	7.9174	7.7399	8.0915	7.8921	7.2221	7.5118
7.6934	7.6934	6.2275	7.3212	6.3799	7.4434	8.0915	7.1253
8.2124	7.9075	7.4266	7.9869	7.274	6.4578	7.3968	7.4465
7.6846	7.6846	7.1766	6.9382	7.4199	7.3677	8.2647	6.7131
7.4427	7.0571						
CBX4	5.1946	4.0056	4.9368	4.911	3.848	4.5856	4.5046

4.4628	4.3841	4.0461	4.8699	3.9609	4.4344	5.4884	4.2315
5.4405	3.4915	4.8125	4.7053	5.1539	5.1707	4.1181	3.8212
4.0355	4.2257	4.0317	5.1464	5.201	4.254	4.2303	4.501
4.3339	4.7221	4.5105	4.495	4.0586	5.3242	5.7567	5.5013
4.6482	4.0952	3.8925	3.2911	4.4833	4.3607	4.0257	5.2903
4.2227	4.2214	3.3989	4.9705	4.0266	3.9807	6.5747	5.3119
4.6719	3.4027	4.6339	4.5583	4.5363	4.5529	4.2624	4.9154
4.2235	5.0627	3.7436	5.8671	4.8556	4.3732	4.5384	4.3423
4.4838	5.5459	4.1735	4.7693	4.8918	5.0574	4.7994	4.7146
4.3192	4.5398	4.1124	5.1001	5.3128	4.7206	4.2604	4.0839
3.9495	3.6569						
CBX5	4.246	2.9453	3.4335	4.9552	3.8323	4.5116	2.1057
3.8616	3.175	3.0874	3.4085	3.1335	3.4155	3.7859	3.55
3.7094	4.0658	4.3016	3.6207	3.6513	4.0459	4.2942	3.0207
3.6333	4.413	3.8417	4.2398	2.3504	2.4769	2.8212	6.0464
3.3454	1.6631	3.0993	4.2255	3.5061	3.283	3.5863	3.2526
4.2741	3.1544	3.9278	3.8034	4.3645	4.6817	5.0828	4.3856
3.6552	3.4043	4.9635	3.2142	3.9814	3.6865	3.0787	4.5326
2.7928	4.6974	4.9821	3.5633	4.6858	3.857	3.0941	4.893
4.371	4.57	3.5926	4.1141	3.1895	2.957	5.3493	4.1537
3.9482	3.2283	2.9015	4.0229	3.6946	2.69	4.2873	4.2742
3.5183	3.867	3.3312	3.2524	4.38	3.6256	3.22	3.7671
4.0715	4.0108						
CBX6	4.8948	3.6544	4.5297	4.0927	4.4194	4.994	4.1206
4.6232	3.8747	3.8084	3.9412	4.6293	4.8347	4.4182	4.7521
4.6677	4.1892	3.4242	4.0518	4.4366	4.19	3.7349	4.1369
4.8176	4.566	4.0483	3.0797	4.008	4.4583	3.3266	4.2441
4.8792	4.6714	4.3571	4.5332	3.78	5.0014	5.3012	5.0611
4.7037	3.7865	5.0009	4.7897	5.2538	4.6554	4.2154	3.6733
4.1472	4.5528	4.1373	3.5258	3.8095	4.2139	4.7847	4.2278
3.7369	4.3356	5.1701	3.2715	4.6942	5.0246	4.0594	5.3432
4.1585	4.0162	4.9529	4.8855	4.0036	4.0289	4.8615	5.0789
4.1455	4.2151	4.3047	4.363	3.8905	3.792	4.9967	4.6732
4.0084	4.7042	5.2058	3.8095	5.0505	4.6779	4.2748	4.5112
4.56	4.9264						
CBX7	1.6284	1.5621	1.7152	1.4342	1.8144	1.8031	1.9571
1.3381	1.9198	2.2281	2.6922	2.6594	2.4736	2.2168	2.3041
3.4219	1.551	2.2362	1.755	1.9077	1.4623	1.6885	2.0889
2.1204	1.9716	1.5942	2.4048	2.0786	2.3041	1.8369	2.2029
2.5484	2.341	3.1021	2.4984	1.7219	1.8369	3.1703	2.6392
0.8698	1.6181	2.3247	2.4272	2.5714	1.8551	1.9855	0.1759
2.0797	3.0836	1.2176	2.0969	1.6748	2.1373	1.535	1.5135
1.2481	2.5882	1.3637	1.3439	1.7973	2.16	1.9129	1.4752
1.9962	1.3318	2.8181	2.5936	1.6895	1.3356	1.7336	1.3297
2.3818	1.3445	2.7195	2.2702	1.8724	1.3514	1.9872	1.777
3.2602	2.8863	2.3077	2.2413	1.3452	1.9419	2.1447	2.2829
1.2617	2.3475						
CBX8	2.7923	2.891	3.1153	2.6055	2.7844	2.2474	1.9302
3.0866	1.841	2.4312	2.701	2.3187	2.4293	2.2021	1.8098
2.4799	2.022	2.1868	2.2653	3.5631	3.7636	2.0718	2.015

2.4165	2.6707	2.1937	1.6879	3.4812	2.516	2.8941	3.0672	
3.0714	2.6231	2.5069	2.2522	2.5657	3.4848	4.1325	2.9283	
1.909	2.3648	2.3278	2.0163	2.037	2.1904	2.1507	3.2441	
2.1077	2.499	1.9211	2.8303	1.7144	1.9536	2.7065	3.5907	
2.9409	1.7392	2.622	2.6925	2.4349	2.4737	3.018	3.299	
1.7555	3.5566	1.413	4.0324	2.3447	2.5967	2.7741	2.5964	
2.2681	3.662	1.8674	1.9148	2.1909	2.5767	2.7061	2.2738	
2.6191	2.2756	1.9801	2.7871	2.5708	3.2475	2.1861	2.3266	
2.2593	2.2437							
CBY1	3.6618	2.9407	2.313	2.9889	2.7125	3.2333	3.9017	
3.2874	3.7086	3.6722	3.6705	3.2614	4.4804	3.5962	4.1715	
2.3137	2.7854	2.9654	3.6513	3.3326	2.93	3.0421	2.9177	
2.9837	3.7086	3.9293	3.535	4.2986	3.207	4.1918	4.1549	
2.8687	3.5385	4.2454	4.1076	3.331	3.6477	3.9539	4.3022	
3.4243	3.2821	4.1504	2.9629	3.8951	4.0371	4.1833	3.514	
3.1884	3.4579	4.1019	3.3787	3.2402	3.8171	4.3251	3.4443	
2.8872	3.4879	4.0063	2.9836	4.6181	2.5975	3.5745	2.9969	
3.4845	3.0516	3.783	3.8735	3.74	3.0762	3.3737	4.073	
3.1763	2.565	3.9771	3.4277	2.8651	3.1627	3.8166	3.2028	
4.2144	3.4828	4.2357	2.5438	3.8594	4.2616	3.6012	3.5568	
3.1694	3.3243							
CBY3	0	0	0	0.3293	0	0	0	0
	0	0	0	0.1788	0.226	0	0.1628	0
	0	0	0	0.3616	0	0.2378	0	
0.4399	0	0	0	0.482	0.166	0	0.2713	
0.1482	0	0	0	0	0.214	0	0.4897	0
	0.2378	0.1467	0.3875	0.2001	0	0.514	0	0
	0	0	0.1832	0	0	0.1666	0	
0.3651	0.045	0	0	0	0	0.0957	0.1505	
0.0644	0	0.1014	0.3994	0.2143	0.0597	0.1812	0.1388	
0.3259	0.5881	0.0961	0.1071	0.028	0	0.3743	0.2807	
0.2968	0.3374	0.1891	0.4162	0.151	0	0	0	0
	0.2243							
CC2D1A	3.4881	4.14	3.8252	2.7785	3.542	3.5439	2.7105	
3.2493	3.4451	3.6381	3.6911	3.1125	4.0209	3.844	3.228	
3.9512	2.6487	3.0792	2.9194	4.2426	3.6307	3.5094	3.389	
3.9826	2.7326	3.5238	3.3636	3.3178	3.1768	3.5299	3.8983	
4.2377	3.3285	3.8214	3.3715	2.585	3.961	3.9387	3.8773	
3.7554	3.832	3.0654	2.3996	2.758	2.9054	2.3721	2.2645	
3.4837	3.2425	2.6483	2.9549	2.4435	2.5877	3.7284	3.5344	
3.3406	3.0052	3.0399	3.2841	3.3239	3.7804	3.6131	2.7513	
3.421	2.7752	3.3559	3.4925	3.9305	3.0625	3.1817	3.557	
3.8528	2.691	3.6725	2.7845	4.8475	3.8637	3.2611	4.2729	
3.3756	3.3803	3.0581	4.133	3.6933	3.7655	3.3461	3.203	
3.0527	3.3615							
CC2D1B	4.4548	4.7574	4.1892	3.6935	4.3644	3.7574	4.6238	
3.8013	4.4498	4.6969	3.6483	3.7355	4.0474	4.0051	3.3717	
4.426	3.5111	4.3591	4.1955	3.4833	4.5844	3.2684	3.7321	
4.4657	3.5373	4.213	2.9345	4.3616	4.1531	4.0769	4.4727	
4.1922	4.3512	4.3864	3.7784	4.4324	4.3385	4.4027	3.9316	

4.1896	3.9767	4.0047	3.7112	4.6033	4.3207	3.1972	3.5115	
4.6691	4.3187	3.5595	4.1023	3.9688	4.1282	3.84	4.0149	
4.4402	3.4068	4.2396	4.2542	4.0541	3.8165	3.426	4.1549	
4.3992	3.4883	4.1503	4.2672	4.6537	4.6731	3.927	3.9033	
4.0835	4.3791	3.9023	3.9028	4.2355	4.3604	3.282	4.4297	
4.2041	4.4637	3.6065	4.0368	2.8181	4.2182	4.3961	4.5626	
4.3531	4.6539							
CC2D2A	2.4753	2.5755	2.8397	2.4611	2.6676	3.0912	2.8006	
2.8753	2.8791	2.798	3.0333	3.0809	2.5955	3.3896	2.1057	
3.4615	2.4243	2.9942	2.5214	2.2994	2.7807	2.191	2.5031	
2.7829	2.6368	3.0329	3.7285	3.2407	2.6105	3.11	1.8632	
2.6172	2.449	2.8536	2.9488	2.4895	3.6184	3.1768	2.513	
3.0006	2.0145	3.054	2.9599	3.6663	2.1792	3.3415	3.7693	
2.7763	2.6995	4.1794	2.2394	3.0121	3.1826	2.9052	1.762	
2.8937	3.2406	3.035	2.3223	2.9963	2.5567	2.3098	2.8418	
2.7088	3.2527	3.5545	2.4669	3.7048	2.9067	1.6666	3.7402	
2.5383	3.7112	2.8666	3.4023	4.3117	2.3455	2.6114	2.7359	
3.1084	2.5971	3.3021	3.0026	3.0684	2.9584	2.9771	4.1007	
3.9331	4.0481							
CC2D2B	0	0.0307	0.1969	0.0708	0.0012	0.1754	0.0709	
0.0303	0.0455	0.022	0.1052	0.0118	0.0109	0.0816	0.0114	
0.0583	0.0414	0	0.1486	0	0.0663	0	0.0434	
0.0433	0	0.0118	0.0169	0.2139	0	0.3407	0.0664	
0.0395	0.0402	0.0162	0.0252	0.0054	0.0014	0.4178	0.0109	
0.0549	0.0723	0.0465	0	0.0464	0.0213	0.0153	0	
0.1014	0.0182	0	0	0.0708	0	0.0814	0.0722	0
	0	0	0	0	0	0.0961	0.1037	0
	0.1773	0.0272	0.2564	0.0847	0.2376	0	0.0234	
0.0465	0.0399	0.0149	0	0	0.0464	0.0381	0	0
	0	0.025	0	0	0	0	0.0199	
0.0667	0							
CCAR1	4.2463	4.5032	4.5813	5.1803	4.3345	4.3793	4.0688	
4.1075	3.9501	4.4617	3.6177	4.1173	4.4703	4.7399	4.2519	
4.2121	4.2239	4.4861	4.4274	4.4533	4.8778	3.5393	3.8876	
4.1852	4.5446	4.3178	3.7622	4.6366	3.9173	3.8682	4.6307	
4.2768	4.3534	3.294	3.8819	4.8662	4.5023	4.0795	3.8764	
4.3822	3.8488	4.4175	3.9818	4.1191	4.4828	4.371	4.6622	
4.531	3.4657	4.9667	3.8107	4.5628	4.0454	4.3603	4.9878	
4.8218	3.9575	4.5861	4.458	4.6478	4.0735	4.1586	4.7321	
4.5749	4.5001	3.9537	3.6092	4.6957	4.4935	4.3453	4.2555	
4.1921	4.0164	3.9318	4.2725	4.2053	4.3323	4.0309	4.0123	
3.9756	4.5963	4.3562	3.2502	3.5422	4.2218	4.0822	3.8592	
4.3447	4.1701							
CCAR2	5.2838	4.9238	4.4269	4.8819	4.9917	3.8951	4.9471	
5.4618	3.917	4.7773	4.2915	4.6939	5.3903	4.5131	4.1876	
3.8716	4.4683	4.8099	3.9532	3.3883	5.2908	5.5391	5.3088	
4.9842	5.5015	4.9225	4.648	4.9315	5.4923	4.8418	5.6221	
5.6518	4.6525	4.783	5.1546	4.3321	5.2418	5.2307	5.485	
4.6151	5.2191	4.756	4.3291	4.1139	5.3775	5.0558	4.7825	
4.5153	5.1736	5.4397	4.3431	5.3229	4.6401	3.0194	5.367	

4.7881	4.1523	5.3424	4.5311	5.9067	5.0367	4.8457	5.1639	
4.1673	5.9306	3.8439	4.8625	5.0893	4.1644	5.1105	4.8226	
4.4741	4.7706	4.9612	5.1993	5.028	4.6655	5.0999	3.97	
4.5016	4.6606	5.2279	4.4879	4.9383	5.5422	4.8805	4.4845	
4.6975	4.6559							
CCBE1	0.0703	0.2135	0.0811	0.0532	0.2592	0.1229	0.0824	
0.0131	0.1238	0.0477	2.2223	2.1839	2.7202	0.7313	0.8419	
0.1621	1.4446	0.032	0.2561	0.8914	0.5008	0.6204	0.2813	
0.0976	0.0134	4.1176	0.5342	0.0512	0.1635	0.0235	0.0097	
0.0533	0.3307	1.3296	0.1458	0.0386	0	0.0107	0.0078	
0.0668	2.5926	2.1797	2.1957	0.0326	2.9597	0.2946	0.0682	
0.4318	0.5214	4.388	0.1155	0.1262	0.1224	0.0884	0.0655	
0.1566	3.559	0.0743	0.0841	0.0798	0.3715	0.509	0.1466	
0.3759	0.0779	3.7712	0.1287	2.7057	0.0888	0.1118	0.1703	
0.5463	0.0696	1.3573	0.2378	0.095	0.3232	0.2367	0.1284	
0.154	0.0817	2.7071	0.0511	0.0855	0.5222	0.1091	0.5478	0
	0.1778							
CCDC102A	1.2083	1.6401	1.6319	1.6374	2.4499	2.6071	1.2552	
1.8121	1.2867	0.5079	0.8198	1.3583	1.908	1.8894	2.0966	
1.5115	2.4664	0.7089	0.4405	2.5461	1.5149	2.7294	1.9432	
1.6155	2.0826	1.9853	1.8091	2.1132	1.168	1.0108	2.7699	
2.2267	1.0779	1.6374	1.811	0.6746	2.1479	2.7984	1.9737	
0.9294	2.1084	2.2186	2.4228	1.9961	1.4988	1.5067	1.112	
1.314	2.3976	2.3528	1.6519	1.6292	1.713	1.7969	3.2918	
1.6828	2.7146	2.1292	1.5004	2.2368	2.113	0.9275	2.3553	
1.6544	2.3222	1.4377	1.4314	1.7362	1.0987	3.3345	2.3185	
1.968	1.5103	0.823	2.3581	1.7391	0.9579	1.4937	1.5623	
1.911	1.4739	1.8555	2.1446	2.8292	3.4628	2.1333	1.0152	
0.965	0.9005							
CCDC102B	0.158	0.2785	0.0798	1.3184	2.8561	1.3825	1.0088	
0.0274	0.2436	0.0926	0.4583	0.7159	1.7037	0.324	0.0079	
0.1276	1.3656	0.8979	0.27	0.4657	0.2131	0.7671	2.8002	
0.1137	2.2486	1.0254	0.6362	0.8126	1.8145	0.259	0.8657	
0.4228	1.3596	2.9401	1.1328	0.3782	0.1224	0.4099	0.0942	
1.2446	0.5624	0.592	0.0429	0.4221	0.8255	2.5739	0.1977	
1.4147	2.0451	0.9427	0.8601	0.7721	0.9229	0.534	1.3376	
0.7175	0.5549	0.4079	0.1502	0.3699	0.835	0.1169	1.0186	
0.6011	0.5117	0.4872	0.7835	0.2078	0.7342	1.7692	0.1022	
0.9851	0.1276	0.6806	0.4778	0.4778	0.4156	0.2759	0.6813	
0.835	0.3386	0.3493	0.8646	1.9651	1.3769	1.1019	0.1774	
0.2686	0.2661							
CCDC103	1.1366	1.984	1.3302	1.5825	1.5316	1.0996	1.7049	
1.5943	0.8861	0.2493	0.9536	1.2921	1.9578	1.1165	1.0615	
1.2972	1.6976	0.7425	0.8628	1.5967	1.0102	0.999	1.4462	
0.9498	1.348	1.5142	0.0999	1.7879	1.622	0.7996	2.6521	
1.6861	1.3194	1.1087	1.2885	1.1299	2.8257	0.9377	0.4065	
1.3648	1.6195	2.3421	1.5668	0.5895	1.6195	1.8119	1.4376	
1.0102	1.3322	1.5443	1.4798	1.9056	1.0216	1.0604	1.5221	
0.934	1.2997	0.8095	1.2588	0.6251	1.3802	1.8819	0.9975	
1.0049	0.7574	2.2674	0.9265	1.3229	1.2114	2.2384	2.5977	

1.2917	1.4441	1.5474	1.4214	1.4141	1.1389	0.8733	0.6763	
1.7185	0.9347	0.6502	2.6874	1.4285	1.4189	1.0712	1.5278	
1.304	1.1465							
CCDC105	0	0	0	0	0	0	0	0
	0	0	0	0	0	0	0	0
	0	0	0	0	0	0	0	0
	0	0	0	0	0	0	0	0
	0	0	0	0	0	0	0	0
	0	0	0	0	0	0	0	0
	0	0	0	0	0	0	0	0
	0	0	0	0	0	0	0	0
	0	0	0	0	0	0	0	0
	0	0	0	0	0	0	0	0
	0	0	0	0	0	0	0	0
	0							
CCDC106	2.7617	3.7153	3.3974	2.6045	3.6625	2.2308	1.6276	
3.5242	1.7468	2.155	2.4156	2.9813	2.484	2.8061	2.7354	
2.7944	2.7589	2.0069	2.0159	2.3187	3.8462	2.2855	2.6184	
0.5016	2.6243	2.0169	2.9648	3.0831	2.0726	2.9039	3.7367	
2.8129	2.8209	2.8339	1.0939	1.656	3.8923	4.1355	3.6722	
1.2818	3.2173	4.1532	1.3863	2.3691	2.4089	0.9483	1.6748	
0.5029	3.4432	2.5032	2.6932	1.9051	1.987	2.9834	2.8284	
3.1033	0.3735	2.8554	3.1617	1.9336	2.5165	1.5921	3.0637	
2.1224	2.5732	3.7676	3.4868	3.6887	3.4472	1.7391	3.1622	
2.3597	0.6451	3.5851	2.6328	2.9089	2.4829	1.9839	2.6814	
3.7389	2.9817	3.85	1.9661	2.5976	3.9712	3.125	2.8632	
3.1813	3.0086							
CCDC107	1.327	1.3666	1.4791	0.553	0.7089	0.9706	0.4349	
1.3573	0.7498	0.968	0.8689	0.8018	1.3495	1.1902	1.4592	
1.6652	1.533	1.1994	1.1405	1.945	0.8722	0.7586	0.8334	
1.9132	0.6487	0.6447	0.8549	1.4653	1.0819	0.9444	0.9622	
1.7968	1.92	1.0883	1.2018	0.892	0.781	1.9014	1.7795	
0.8646	1.3231	0.7009	1.2192	0.9985	0.9319	0.6605	1.2211	
0.928	1.6822	1.2862	0.9622	0.6909	0.7429	0.8973	1.372	
1.033	1.5619	1.3331	1.914	1.125	0.9569	0.9926	1.6681	
1.364	1.2083	1.9137	1.1746	1.6316	0.9235	0.9391	1.2626	
1.3992	0.8806	0.5653	0.8072	0.6254	1.5837	1.2138	1.054	
1.5309	0.8717	1.5238	1.566	0.6772	2.0601	1.427	1.6151	
1.6229	1.6066							
CCDC110	0.6715	0.5612	0.5774	0.3453	0.0602	0.0858	0.3257	
0.1445	0.4818	0.2932	1.3497	0.817	0.2989	0.4809	0.337	
0.069	0.1506	1.1228	0.3464	0.1017	0.4131	0.033	0.1375	
0.0351	0.0392	0.1587	0.141	0.5509	0.0363	0.7949	0.2008	
0.1649	0.3036	0.774	0.1608	0.3313	0.0836	0.4334	0.0362	
0.3675	0.6972	0.0151	0.5116	0.1709	0.0769	0.1106	0.3197	
0.1927	0.0574	0.5914	0.3113	0.1563	0.802	0.5729	0.5466	
0.2853	0.9576	0.2915	1.1679	0.2046	0.1888	0.3942	0.0219	
0.1712	0.7963	0.3893	0.8964	0.2974	0.3131	0	0.2771	
0.1114	0.1934	0.2077	0.0775	0.3112	0.5385	0.0532	0.2778	
0.1945	0.4109	0.1584	0.0906	0.4658	0.0586	0.6397	0.922	

0.4236	0.5868						
CCDC112	2.2017	1.9478	2.4574	1.9857	2.0582	2.2244	2.6557
2.8623	2.2689	2.329	2.8085	2.8034	3.1834	3.1717	2.7758
2.7299	2.8213	2.0431	2.8502	2.5453	2.4444	2.3699	1.8429
2.8862	2.7936	2.4816	4.1708	2.0463	2.3694	2.017	2.7423
2.717	3.1121	3.2417	2.1444	2.7194	2.1861	2.1884	2.4236
3.0087	2.1454	1.7323	1.8471	2.5509	1.8294	2.0685	2.5949
2.0381	2.0136	3.0358	3.0849	2.4085	2.5529	2.5816	2.5704
1.8588	2.1555	2.3583	2.719	2.1831	2.2978	2.4266	2.4108
2.4586	2.4547	2.2506	3.0263	3.7235	1.8864	1.6123	2.806
2.934	2.3375	3.0592	1.9499	2.0447	3.3895	2.1505	2.9281
2.5609	2.6391	2.5653	3.0402	1.742	2.7273	2.6992	2.0009
2.293	2.06						
CCDC113	1.3927	1.4901	0.7924	0.7477	0.651	0.3807	0.8942
0.6805	0.6178	0.4816	1.0297	0.1812	1.3356	0.3329	1.101
0.1891	0.8226	0.4407	0.3814	1.279	0.1813	0.1467	0.1702
0.5506	0.5763	1.6725	0.5902	1.1716	0.3386	1.9447	1.2834
0.2132	0.166	0.3255	0.939	0.4842	2.1824	1.0184	0.4429
1.5083	0.7573	0.8111	1.1482	0.8654	0.1796	0.9653	0.7494
0.1636	0.2761	0.7732	0.6425	0.643	0.1596	0.8306	1.2292
0.2274	1.9417	0.1097	0.9857	0.0278	0.2414	0.4202	0.3642
0.4388	0.7239	1.6308	0.7576	2.0338	0.4331	0.9105	0.6756
0.3524	1.3866	0.1597	0.3532	0.4436	0.525	0.9811	0.7723
1.024	0.4003	0.5012	0.8306	0.5008	0.4714	0.2248	0.2373
0.5154	0.1889						
CCDC114	0.5059	0.3241	0.4944	0.1686	0.0962	0.3358	0.1213
0.3232	0.1717	0.4877	1.1867	0.2566	0.1561	0.5602	0.3038
0.6699	0.4231	1.1468	0.2263	0.9438	0.553	0.1566	0.1902
0.4285	0.1198	0.1224	0.2578	0.595	0.4767	0.2858	2.4271
0.617	0.4307	0.9421	0.3204	0.4804	0.1949	0.2303	0.1478
0.4334	0.5606	0.2616	0.5217	0.6397	0.6031	0.3269	0.1935
0.174	0.3068	0.1821	0.2582	0.2928	0.3941	0.8372	0.7733
0.3117	0.3535	0.05	0.368	0.0936	0.0055	0.6323	0.3064
1.4899	0.3438	0.2884	0.7113	0.0385	0.5139	0.5769	0.5237
0.58	0.2925	0.2536	0.8255	0.1986	0.1738	0.144	0.58
1.2292	0.7408	0.1506	0.5703	0.1957	0.0152	0.8276	0.8582
0.5831	0.8381						
CCDC115	3.4448	3.5803	3.2626	2.8132	2.7406	3.2523	4.1232
4.1784	3.9732	3.3916	3.8828	3.2827	2.7201	4.1638	3.7954
3.8137	2.8399	4.4858	4.1556	3.9649	4.3571	3.0383	3.5853
3.1525	2.8801	3.5005	3.3211	2.9986	3.6248	4.455	3.6705
3.2649	4.1301	3.7895	3.3659	3.5587	3.1564	3.7233	2.7922
3.5261	3.4175	3.2781	3.2964	3.1153	3.3554	3.9617	2.8342
4.6451	3.5692	3.4078	3.5698	2.8543	4.3842	3.3481	3.4329
3.0437	3.3058	3.3633	3.6229	3.3234	3.2596	4.4062	2.5723
3.8899	2.8193	3.3386	3.4304	4.4748	3.8198	3.7108	3.0716
4.2576	3.9469	3.5574	3.1995	3.991	3.6778	3.4789	4.0022
3.5964	3.3251	2.6895	3.801	2.5444	3.2975	4.2716	4.2808
3.9515	3.8186						
CCDC116	0.1929	0.2971	0.3828	0.1144	0.0307	0.1925	0.1105

0.1659	0.0736	0.0559	0.1309	0.014	0.3103	0.2311	0.1863	
0.0892	0.4311	0	0.434	0.259	0.0907	0	0.0283	
0.2765	0.0344	0.0226	0.0417	0	0.1981	0.0353	0.0857	
0.0906	0.4592	0.2472	0.1225	0	0.0333	0.1719	0.0996	0
	0.1417	0.09	0.0485	0.043	0.1057	0.1079	0.0988	0
	0.3463	0.094	0.0547	0.0384	0.1949	0.0479	0.1407	
0.1431	0.0245	0.0266	0.2108	0.0418	0.1199	0.1185	0.0643	
0.2992	0	0.0463	0	0.0504	0.523	0.1881	0.2424	
0.3029	0.045	0.0732	0.0838	0.1569	0.2482	0.0143	0.0386	
0.3289	0.0631	0.0854	0.2144	0.0477	0.0733	0.2265	0.052	
0.1136	0.0603							
CCDC117	3.1276	3.6404	3.4461	4.3086	3.1243	3.3492	3.9015	
3.7696	3.0868	3.7453	3.9079	4.0327	3.9287	3.6708	3.8804	
3.2619	2.4886	3.7515	3.5429	3.1451	3.3167	3.7107	3.5095	
3.9997	3.6022	3.6887	3.5132	5.1505	3.6217	4.4936	4.3823	
2.5395	3.5826	3.6741	3.7551	4.6525	3.4077	3.1784	3.4938	
3.3263	3.301	3.5697	3.6814	3.1269	3.9364	4.0539	4.374	
3.6244	3.4074	4.0201	3.4033	3.9618	4.5698	2.8832	4.4019	
3.3553	3.4249	4.3803	3.832	4.2843	3.7683	3.3545	3.826	
2.8537	4.2257	3.2074	4.004	2.6674	3.7492	3.1542	3.3853	
3.7196	3.4811	3.8048	4.0868	3.4182	3.2382	3.4918	3.6654	
3.8366	4.5335	3.6522	2.6092	4.2235	3.541	4.4668	3.0606	
2.9773	3.4298							
CCDC12	3.5515	4.3591	4.1639	3.3557	4.2251	3.4196	3.1668	
4.4375	2.9626	4.1651	3.8525	3.8814	4.0555	2.7941	3.215	
3.8336	4.0307	3.604	3.519	3.9902	4.3849	4.3081	3.6234	
4.0021	3.5121	3.4211	3.5742	3.5842	3.7853	4.0964	4.4701	
4.4018	3.9047	3.4149	3.544	3.5607	4.2993	4.6888	4.2358	
2.3871	3.5261	3.5409	3.8867	3.6466	4.0113	2.6782	3.4854	
3.9314	3.7124	3.8825	3.5532	3.2791	3.256	3.8488	4.2477	
3.985	3.6846	4.6051	3.7311	4.3156	3.9884	3.7431	4.4859	
3.8546	3.6994	3.8524	4.2357	3.7431	3.971	3.9695	3.1828	
3.6074	4.0138	3.7296	4.2516	3.2067	3.6023	3.9927	2.8092	
3.986	3.6131	3.5602	3.1968	3.8304	5.2792	3.7832	3.6791	
4.0005	3.9908							
CCDC120	0.5735	2.2654	0.3027	1.1305	0.9665	0.6086	1.3	
1.9722	1.2684	0.6634	1.7607	1.7423	1.4825	1.156	1.558	
0.8879	0.3414	0.558	0.3482	0.4646	0.9947	0.9747	1.0777	
0.8847	1.5341	1.2209	2.078	2.094	0.9565	0.9824	2.7438	
1.5341	1.5299	2.0811	1.0055	0.9694	1.6898	1.828	1.5182	
0.7388	1.1865	1.138	1.1336	1.378	0.7867	0.188	0.5084	
1.4195	0.4491	0.5229	1.7412	0.8557	0.6773	1.644	2.8694	
2.4302	0.4918	0.6241	0.99	1.3766	0.1396	0.8884	1.3995	
1.3726	1.8421	1.2864	1.5503	1.2266	1.6811	1.1917	0.613	
1.0148	1.4956	0.9949	1.8522	1.3841	1.3726	0.9839	1.0026	
1.2087	1.4609	1.2039	1.5612	1.1161	1.2299	0.1712	1.1085	
1.1566	1.2546							
CCDC121	1.9411	1.2421	1.0234	0.8938	0.5482	0.8043	0.5259	
1.2675	1.9798	1.7699	1.567	0.8327	0.7343	1.1286	0.6685	
0.7354	0.9587	1.5157	1.1754	0.9307	0.9628	0.7718	0.8334	

1.5737	0.7902	0.6629	0.5155	2.0598	1.1085	2.1686	1.3378	
0.6877	1.1737	1.1796	0.9574	2.0175	0.754	1.427	0.911	
1.5137	1.4662	0.7921	0.6988	1.4609	0.816	0.6988	1.7683	
1.7483	1.1079	0.9879	1.2249	1.0397	1.152	0.8373	1.7807	
1.7714	0.7983	0.8846	0.7447	0.8917	1.13	1.1423	1.5245	
1.4843	1.6228	0.709	1.9878	0.479	1.5186	1.4152	1.0535	
1.3661	1.7035	0.5728	1.0028	0.9752	1.1334	1.5571	1.4803	
1.9246	1.3561	0.6095	0.831	0.792	0.5849	1.175	1.3753	
1.1229	1.3251							
CCDC122	1.9425	0.1016	0.583	1.1243	1.2658	2.0808	1.184	
1.6794	0.6711	1.056	0.2706	1.4084	1.4813	1.7523	1.3762	
1.2191	2.3726	1.5468	0.6038	0.5568	0.792	1.1555	1.9051	
1.0227	1.8851	1.337	0.8958	0.9981	1.3845	1.1662	0.2756	
1.1884	1.7579	0.9315	1.8168	1.1774	1.6094	0.9956	1.116	
1.0567	1.6107	1.5847	1.9304	1.6377	1.0783	1.7955	2.3038	
0.8864	1.862	2.0954	1.4991	2.0176	1.1965	0.8985	0.6947	
1.1024	2.6727	1.2234	1.4275	1.0722	2.0599	0.9743	1.2686	
0.2803	1.1069	1.7097	1.4707	1.681	1.1516	2.1594	1.3766	
1.5145	0.7704	0.6096	1.425	1.934	1.3838	0.5007	2.1875	
1.2724	1.5915	1.6165	1.5063	1.4416	1.5494	1.5112	1.1675	
0.5688	0.9884							
CCDC124	5.0286	5.108	5.7118	3.2825	5.8505	4.6663	3.6708	
5.1684	4.0805	4.4495	5.446	4.2132	5.6133	4.1305	5.5148	
5.04	5.3821	3.1957	3.8165	5.5825	5.4948	5.1998	4.8445	
4.9644	4.5349	4.6914	3.9736	5.2464	4.689	4.653	5.5028	
5.5434	5.6629	4.8484	4.7369	3.5609	5.2914	5.4288	5.5241	
3.5299	5.1805	4.3691	4.5165	4.2608	3.6537	3.872	3.1795	
4.4831	5.4765	3.4943	4.1249	3.1475	3.3425	6.2153	5.893	
5.9186	4.4049	4.9929	6.944	5.0949	5.2194	5.5559	6.0202	
4.8785	4.9529	4.4856	5.5985	4.9697	4.9057	4.8306	5.702	
5.1586	4.648	4.4205	4.0728	4.7756	5.0258	3.925	4.8077	
5.038	4.6177	4.6942	5.4427	4.8569	5.9053	4.9995	4.8783	
5.4329	5.0595							
CCDC125	1.8998	0.4324	1.3527	1.4603	1.7493	0.945	1.0368	
2.0395	1.7838	1.4387	2.0252	2.2743	2.4852	2.1804	1.1308	
1.3405	1.5565	0.3078	2.7917	1.4081	1.5985	1.0201	0.9269	
2.3582	1.7817	1.4471	1.7582	2.5174	1.201	1.5656	2.4581	
1.6498	1.6492	1.6927	0.407	1.7377	1.9367	2.0621	1.7247	
1.1633	1.997	2.0894	1.1464	2.6622	1.0298	1.5328	1.7146	
0.6371	1.6391	2.2581	1.6922	2.1794	1.7854	0.6594	2.0964	0
	2.1886	0.7823	2.5099	0.8065	1.6727	0.7384	2.0677	
2.1044	1.8771	1.7974	2.0284	1.2333	1.8055	2.11	1.4234	
1.6435	0.7808	2.0158	1.4135	2.0833	1.6053	1.1587	1.36	
1.8253	1.9649	1.9541	1.8265	2.0234	0.5148	1.6068	0.5426	
0.406	0.4901							
CCDC126	1.8781	0.98	1.8793	2.7055	2.25	2.5592	2.1464	
2.4223	2.6959	1.8949	2.0171	1.8855	1.754	2.2399	2.5357	
3.0061	1.6531	1.8844	1.8687	3.189	2.63	2.8913	3.2787	
2.8268	3.2392	2.2436	3.1608	2.2078	3.2856	1.7271	4.3116	
2.2589	2.0851	2.5131	3.8807	2.5768	2.3501	1.692	2.2487	

2.5262	2.5705	2.1988	2.3014	2.7007	2.3084	3.5798	1.4655	
2.3822	3.0034	1.1582	2.262	3.287	2.7923	1.9811	2.8064	
2.0514	1.9742	1.9684	1.5799	2.6633	2.927	2.5234	2.1709	
2.2158	1.7759	2.0153	3.9684	2.1794	2.2614	2.975	1.6604	
2.6279	2.7411	2.3531	3.0837	2.7048	1.2986	3.496	1.9041	
2.0495	1.5403	2.497	3.0675	3.062	2.6886	3.2491	2.2684	
1.9508	2.0946							
CCDC127	4.1468	4.1536	3.4582	4.0397	3.0598	3.7869	3.1734	
3.4437	3.5157	3.3444	3.9576	3.1277	3.9387	3.3881	4.228	
3.7309	3.0868	3.6689	3.5967	3.8143	3.9859	3.1719	3.4908	
3.5467	3.3201	3.6207	2.9756	4.2986	3.4966	4.3386	4.5535	
2.3256	3.8236	3.3617	3.909	4.0631	3.6074	4.2739	3.1802	
3.2853	3.5112	3.9398	4.0815	3.4293	3.6933	4.0736	3.6582	
3.3419	3.3881	4.003	3.7121	3.792	3.572	3.1537	3.6496	
4.2865	3.4814	3.4145	3.8245	3.3786	3.4722	3.9994	3.8807	
3.3643	4.2458	4.3586	3.7532	4.0531	3.0351	3.9437	3.8634	
3.5735	4.0065	2.4359	3.697	4.4428	3.3653	3.7831	4.0009	
3.8615	3.692	3.8468	3.9986	3.4912	4.0648	3.4521	4.2552	
3.9942	3.9767							
CCDC129	0	0	0	0	0	0.0515	0	
0.0204	0	0	0	0	0	0	0	
0.0171	0	0	0	0	0	0.0189	0.0289	
0.1886	0	0.0734	0	0	0.0936	0	0	0
	0	0	0.0269	0	0	0	0.1655	
0.0618	0	0	0	0.1828	0.0434	0	0	0
	0.13	0	0	0.0342	0	0	0	0
	0	0.0057	0.0042	0	0	0	0	
0.008	0	0	0	0	0	0	0	0
	0	0	0	0	0	1.6113	0	0
	0	0.0025	0	0	0	0	0	0
	0							
CCDC13	0.2125	0.4134	0.0737	0.0775	0.1088	0.1818	0.0063	
0.173	0.0371	0.1736	0.1948	0.1989	0.2993	0.159	0.0283	
0.317	0.6962	0.4173	0	0.366	0.2957	0.0929	0.1781	
0.4066	0.0964	0	0.5723	0.4267	0.0389	0.1037	0.2397	
0.39	0.0328	0.1162	0.3346	0.0947	0.2645	0.1483	0.1337	
0.2176	0.3696	0.4961	0.169	0.1473	0.1298	0.1333	0.254	
0.141	0.2085	0.2569	0.1315	0.2866	0.29	0.1387	0.3219	
0.4508	0.3091	0.065	0.1398	0.1244	0.0374	0.1192	0.2157	
0.2771	0.1883	0.1657	0.4277	0.4147	0.0726	0.2692	0.1881	
0.0575	0.0639	0.1184	0.2389	0	0.2494	0.4257	0.1581	
0.4007	0.5416	0.1987	0.2278	0.114	0.7761	0.1248	0.7747	
0.4927	0.5611							
CCDC130	3.3338	3.1081	3.5624	2.2222	3.4885	3.0866	3.2523	
3.2369	2.8893	3.2507	3.9017	3.1919	3.7918	3.4205	3.5261	
3.1108	2.7317	2.4452	2.6147	3.548	3.5745	2.9866	2.9887	
3.5287	3.4724	3.9017	3.7786	3.593	3.1119	3.2371	3.6166	
3.4613	3.4317	3.5952	3.303	2.7197	3.9745	3.3772	3.788	
2.7632	3.5923	3.1357	3.3889	2.9085	2.641	2.9769	2.5752	
2.7246	3.6102	2.8852	3.0412	2.6161	2.4351	3.9609	3.6914	

3.4242	2.7335	2.8013	2.9703	2.9034	3.2587	3.4905	2.8578
3.3794	3.041	4.0125	3.2341	3.6101	3.1497	3.1431	3.0716
3.6629	2.7889	3.2776	3.1769	3.248	3.6622	2.5849	3.072
2.9261	3.0385	3.3562	4.3569	3.2166	3.8353	3.06	3.3599
3.6537	3.479						
CCDC134	1.8928	2.7569	3.1076	2.1415	1.8317	3.2357	2.2426
2.8778	2.5588	2.3897	2.4765	2.4294	2.6893	2.0008	2.7854
2.7191	1.8836	1.8147	2.4404	2.5701	2.4803	3.0143	2.2728
2.9192	2.0156	2.227	1.8465	2.0789	2.6141	1.6309	2.3782
4.5752	3.66	3.2702	2.6591	2.671	2.3782	1.5975	2.3046
2.3041	2.1731	1.8153	2.3338	3.1516	2.0644	2.8	3.2307
2.6656	2.4258	2.3611	2.0422	2.318	2.3112	1.3886	2.3925
2.4049	2.2894	3.013	2.6962	2.6396	2.7913	2.9597	2.973
2.4864	1.9715	1.97	3.1076	1.797	2.2257	1.9266	3.2838
2.73	2.9101	1.3203	3.0734	3.0019	2.647	2.4528	2.3491
3.5742	2.8858	2.3619	1.5935	2.4939	2.1535	2.6082	2.0422
2.5412	2.2911						
CCDC136	1.9557	3.4099	1.2918	2.0337	1.966	1.874	3.8772
2.6048	2.9014	3.9004	2.5915	1.4092	2.6324	1.2006	2.0947
2.6351	0.8171	3.3188	3.8207	0.9407	1.8878	1.2427	2.3996
3.0476	1.7513	1.6471	2.118	2.9772	1.738	4.0603	1.2447
2.0973	0.8622	3.5871	2.0619	2.8535	1.8141	1.5978	1.07
1.7502	3.4858	2.3236	2.0646	2.2811	1.6852	0.9994	2.7509
3.2174	2.6809	1.42	2.0362	1.6105	0.4889	1.6489	1.1461
3.4066	0.7479	1.2974	2.489	1.7662	1.4545	0.9812	1.6207
2.3895	1.4401	3.0295	2.14	2.7489	3.3557	2.1421	1.9672
1.7498	1.0053	2.4406	2.1647	2.7797	2.2332	2.7745	2.4495
3.3217	4.2584	1.1663	1.5214	1.4297	0.8462	0.8372	1.0205
0.886	1.5517						
CCDC137	3.982	4.8977	4.1726	2.9196	4.4468	3.8579	3.3681
3.6616	3.1762	3.0976	3.6139	3.6976	3.9282	3.4981	3.6961
3.6009	3.3333	2.8875	3.2351	4	3.6539	4.1889	3.4395
4.1712	3.616	4.0492	3.5331	4.231	3.8015	3.5	3.6197
4.6213	3.9439	3.0045	3.4907	3.3652	4.6393	3.747	3.6104
3.2867	4.0564	3.9049	3.0964	3.5424	3.5176	3.3433	3.7791
3.9683	3.2014	3.8296	2.8231	3.3401	2.9231	3.1821	4.7862
3.9627	3.2711	4.2584	3.8538	4.0713	4.4578	4.2857	4.2584
3.6547	4.0917	3.1877	4.1145	3.8285	3.8131	4.0457	3.9513
3.9219	4.6053	3.3771	4.1407	3.6222	3.8788	3.4276	3.6012
3.3982	4.2979	3.5403	2.9197	3.735	4.2467	3.4498	3.1568
3.9172	3.4331						
CCDC138	2.0807	3.318	2.568	2.9758	2.4858	1.8349	2.1508
2.2828	2.1426	2.1354	2.1039	1.9463	2.2698	2.6945	2.3461
1.2896	2.0268	2.2389	3.1273	1.8522	2.3013	1.908	1.3219
2.7297	2.2536	0.9364	2.0197	2.8341	2.0161	3.3899	2.5361
2.2291	2.7489	1.5149	1.3033	3.079	1.815	1.2212	1.9609
2.6645	1.5603	1.6017	2.437	2.1837	1.9689	2.6669	3.5163
2.294	1.1512	3.1781	2.56	2.352	2.6418	2.135	3.1118
2.7426	1.5077	2.6002	2.7677	2.6216	2.1083	2.5636	1.4982
2.7144	2.7835	0.6794	1.6402	0.8773	3.3791	2.6286	1.7485

2.0973	2.5438	2.1074	1.8436	1.5812	2.6986	1.5574	2.5502
2.2224	2.6232	1.9825	0.8023	0.839	2.2908	2.6743	0.8073
1.8216	1.5784						
CCDC14	3.7163	3.8709	3.6146	4.0468	3.5138	3.9615	3.5348
3.4315	4.9564	4.9449	3.8482	3.9239	4.259	4.4463	3.9268
3.3574	3.4555	4.5848	4.7621	2.9417	3.798	3.5302	3.5669
3.8528	3.4797	3.2691	5.2218	4.2385	3.7887	3.9264	4.1475
4.7205	4.4378	4.2821	3.9749	4.4338	3.5005	3.2044	3.8811
4.9118	4.0056	3.3785	4.1393	4.9287	4.5505	4.6822	4.0485
4.0005	4.6768	4.0726	4.4601	4.7054	4.5249	4.0056	4.0432
4.2368	3.5607	2.8629	3.4748	3.3343	4.0271	3.5628	3.4018
4.9495	3.5941	3.3026	4.4267	2.8264	4.0271	3.4668	3.69
4.8766	3.5719	4.7431	3.7974	3.9681	4.2857	4.0001	4.1187
4.2088	4.8911	3.6951	2.9897	4.2535	3.8066	4.6532	3.0075
3.8153	3.7627						
CCDC140	0	0.4484	0.6764	0.2883	0.1218	0.5471	0.5096
0.1217	0.3218	0.3248	0.3724	0.2697	0.2294	0.2878	0.386
1.2092	0.5476	0.0927	0.6015	0.3702	0.4764	0.2419	0.2209
0.3921	0.2759	0.143	0.512	0.7522	0.1048	0.3981	0
0.3932	0.6961	0.2781	0.4376	0.1196	0.5179	0.7798	0.2477
0.3078	0.7631	0.507	0.4358	0.4993	0.1507	0.4796	0.5523
0.8251	0.3856	0.7739	0.4772	0.3527	0.366	0.568	0
0.4147	0	0.2397	0.5186	0.3459	0.1161	0.3431	0.3123
0.6606	0.4436	0	1.0476	0.4093	0.6928	0.6344	0.4536
0.542	0.6017	0.1538	0.4686	2.0373	0.6386	0.1457	0.4128
0.8599	0.5684	0.2617	0.3971	0.4135	0.4419	0.4892	1.0002
1.4671	1.8425						
CCDC141	0.0309	0.0142	0.0302	0.0276	0.0066	0.0304	0.0116
0.0094	0.1037	0.0144	0.0164	0.0185	0.0095	0.0642	0.0084
0.2574	0.0419	0.0225	0.1368	0.0403	0.0445	0.0279	0.019
0.0419	0.0336	0.0076	0.0225	0.0539	0.1073	0.0296	0.0132
0.0084	1.032	0.013	0.6821	0.0076	0.0084	0.3443	0.0144
0.0322	0.0269	0.017	0.0355	0.0178	0.0056	0.0233	0.0441
0.1958	0.0168	0.0371	0.1914	0.047	0.0535	0.1043	0.0468
0.1317	0.0893	0.0163	0.0117	0.1037	0.0307	0.0282	0.0623
0.0214	0.0189	4.00E-04	0.0229	0.0108	0.001	0.0336	0.0278
0.0653	0.1253	0.0234	0.0176	0.0245	0.051	0.1096	0.9629
0.0306	0.0252	0.0153	0.0494	0.0278	0.0267	0.0283	0.0195
0.0491	0.0296						
CCDC142	1.8986	2.2747	1.7417	1.6476	2.1278	1.1066	1.6665
1.8431	1.3247	1.3754	1.0271	1.459	1.3279	1.5083	1.5794
1.0085	1.8365	1.6665	1.5309	1.6665	1.8015	1.5033	1.6596
1.9667	1.6232	2.0484	1.1924	1.8467	1.4975	1.5902	1.7712
1.3897	1.7387	1.3684	1.6943	1.6244	2.0184	1.5592	1.2215
1.9548	1.743	1.5336	1.4674	1.2487	1.1477	1.3773	1.3722
2.3015	1.7849	1.5556	1.4903	1.6665	1.6671	1.6865	1.8052
2.1454	1.2181	1.7133	1.8856	1.8809	1.8058	1.9068	1.9699
1.7103	1.4654	1.8499	1.8199	0.9048	2.0591	1.5876	1.2941
1.6833	1.8087	1.9525	0.7712	0.9909	1.7792	1.1598	1.2935
1.621	1.307	1.4228	1.5549	0.9498	1.41	1.6614	0.7814

0.9512	0.9908							
CCDC144A	0.0201	0.0244	0.0751	0.0529	0.0302	0.4858	0.1148	
0.0513	0.1602	0.0462	0.0422	1.00E-04	0.0016	0.0343	0.0375	
0.0176	0.1059	0.193	0.0282	0.4506	0.015	0	0.0133	
0.0892	0.0589	0.1144	0.07	0.0264	0.0903	0.0068	0.014	
0.0292	0.105	0.0215	0.2192	0.0119	0	0.0672	0.0149	
0.2474	0.0847	0.0324	0	0.2933	0.2023	0.0965	0.1291	
0.0667	0.1548	0.2192	0.0428	0.2117	0.0672	0.0329	0.0284	0
	0.119	0	0.0711	0.0451	0.0699	0.1387	0.0923	
0.0321	0.0319	0.0416	0.0441	0.0923	0.0359	0.2393	0.0941	
0.2375	0.0205	0.1023	0.0199	0.1407	0.1217	0.1317	0.1951	0
	0.0334	0.0184	0.0229	0.0486	0	0.085	0.0609	
0.0175	0.0267							
CCDC144NL		0	0.5447	0	0	1.5152	0.0232	
0.3676	0.0554	0.7246	0.0377	0.1741	0.0088	0	0	
0.0025	0	0.0599	0	0.2985	0.0265	0	0.0859	
0.0365	0.3695	0.0125	0.0058	0.3229	0.2327	0	0	
0.0222	0.0237	0.3034	0.0098	0.0324	0	0	0.0972	
0.0599	0.2395	0.1125	0.0204	0	0	0	0.051	
0.0197	0.0373	0.0279	0	0.024	0.0274	0	0	0
	0	0	0	0	0	0.3644	0.0214	0
	0.0445	0	0.0103	0	0.139	0.0434	0	
0.0485	0.0885	0	1.1821	0.1073	0	0	0	0
	0	0.4846	0	0	0	0.0405	0.0582	0
	0	0.0311						
CCDC146	0.8589	0.6305	0.7953	0.2004	0.1398	0.9298	0.8563	
0.1636	1.1287	0.0926	0.5478	0.1862	0.4007	1.068	1.0884	
0.4991	1.0224	1.327	0.5513	0.4011	0.273	0.3025	0.3988	
0.3581	0.2964	0.7405	0.1642	1.1722	0.8995	1.2177	0.4901	
0.4943	0.3808	0.5096	2.8443	0.1355	0.4488	0.9409	0.4332	
0.932	0.5647	0.3944	0.5253	0.2741	0.6744	0.9429	0.0756	
0.2707	1.5651	0.8583	0.5662	0.4381	1.0197	0.8709	0.2873	
0.4304	0.8103	0.201	0.3992	0.3942	1.0244	0.351	0.5016	
0.9586	1.2287	0.503	0.8656	0.4301	0.4421	0.2586	0.2449	
0.6786	0.5065	0.3815	0.6927	0.3545	0.366	0.4356	0.4674	
0.3972	0.4543	0.4149	1.4853	3.0301	0.3782	0.8918	0.5668	
0.3495	0.5597							
CCDC148	0.7126	0.5148	0.9586	0.3713	0.2102	0.6217	1.444	
0.8715	0.455	0.3956	0.432	0.1735	0.6929	0.4887	0.6717	
0.6619	1.7192	0.1271	0.4012	0.537	0.3177	0.1549	0.4132	
0.7238	0.1837	1.4723	0.5553	0.9559	0.1454	1.0616	1.0467	
0.5159	0.7112	0.8035	0.446	0.1535	0.3661	0.2585	0.0585	
0.6845	0.1653	0.4141	0.363	0.3389	0.1486	0.9181	0.4204	
0.6858	0.4519	1.9598	0.5689	0.3364	0.824	0.2244	0.7453	
0.2141	0.8501	0.1924	0.1662	0.2945	0.4945	0.5815	0.3697	
0.4368	0.5206	2.2091	0.5552	1.9185	0.7552	0.3245	0.4077	
0.7481	0.5924	0.4753	0.5159	1.2956	0.5569	0.351	0.4723	
0.1448	0.702	0.2274	0.4563	0.2448	0.7371	0.8411	0.8167	
0.4529	0.8108							
CCDC149	0.9534	0.0065	3.0209	0	0.3014	1.8337	2.7232	

0.9609	0.9789	0.5762	0.2962	0.0049	1.2549	1.3488	1.0518	
2.2165	0.2299	1.6124	0.8191	2.37	1.2705	2.3021	1.2126	
1.3501	2.4515	2.9975	1.2194	1.2897	0.3895	0.8289	1.3151	
1.6412	1.4175	1.5949	2.176	0.2982	2.1288	0.7342	0	
1.8897	0.6863	1.6215	2.53	1.5543	1.4476	2.3961	1.7802	
0.6777	1.304	1.0353	1.5043	1.5845	1.1468	0.2571	0.6506	0
	0.8524	2.2189	0.4418	1.7886	0.8757	0.8001	2.2508	
1.4658	1.7646	2.7508	1.9278	2.8445	1.2605	1.2676	1.6558	
1.8486	0.835	0.5471	1.5541	4.0029	2.4178	1.0553	0.9654	
0.1844	1.0647	0.7353	3.1567	2.0106	1.1879	1.048	2.5704	
2.7565	2.6084							
CCDC15	1.3982	1.501	1.5399	1.3899	1.5449	1.931	1.2142	
0.8145	1.9097	0.963	0.6577	0.7572	1.8278	1.898	1.0639	
1.3477	1.4662	1.4231	2.0949	1.8333	1.5684	1.3805	1.393	
1.7772	0.4354	1.0851	0.3087	0.7187	2.2536	1.1231	1.1042	
0.7959	2.1061	0.9825	1.2108	1.4472	1.1884	0.5547	0.5336	
1.2174	0.8898	0.4672	1.7216	1.7754	1.5365	1.4142	1.6686	
1.3516	0.891	2.471	0.8263	2.5339	1.076	0.9232	1.1306	
1.6129	1.6326	1.0183	0.9532	1.0113	1.3967	1.315	0.9666	
1.914	1.0014	0.5095	1.8255	0.7796	1.0056	2.0075	0.8854	
2.23	1.954	0.8078	1.0734	1.879	1.5007	0.8114	1.5403	
1.2332	1.2637	0.7986	0.5689	0.4141	1.8005	1.0921	1.0919	
1.4186	1.2335							
CCDC150	1.6387	1.4847	1.6278	1.4234	0.7733	1.4906	0.8468	
1.3314	2.5302	1.5958	0.48	1.015	1.5822	2.1502	1.8426	
1.069	1.6192	1.3886	1.9493	1.4003	2.3208	1.1166	0.3859	
1.9615	1.3833	0.8592	0.5905	1.6253	1.1582	1.2121	1.5937	
1.3132	1.7482	0.8383	1.1867	1.6832	1.2568	0.1577	0.5268	
1.7225	0.6425	0.7346	1.4296	1.6766	1.562	1.783	1.4132	
1.3258	1.142	1.9686	1.0229	2.0574	1.2329	0.9406	1.5222	
1.8492	0.9135	0.9259	1.3604	1.456	0.9057	1.2517	1.0213	
1.9003	1.3752	0.4124	1.4151	0.5521	2.3963	1.1836	0.8457	
1.8291	1.081	1.2767	1.2943	1.3793	1.7568	0.7138	0.9961	
1.5273	2.1158	0.5765	0.6771	0.6001	1.7001	1.5317	0.8389	
1.7678	1.4345							
CCDC151	0.7366	0.2836	0.426	0.0875	0.4309	0.1161	0.0361	
0.2677	0.1911	0.3527	0.2484	0.1283	1.0853	0.3186	0.194	
0.1907	0.1746	0.2329	0	0.5684	0.0949	0.1122	0.1401	
0.5226	0.1538	0.231	0.4173	0.5338	0.1072	0.32	1.5693	
0.3149	0.2343	0.8229	0.1946	0.3878	0.1065	0.22	0.0746	
0.3197	0.0955	0.2638	0.4109	0.2185	0.1906	0.3146	0.0675	
0.1668	0.2569	0.5003	0.1146	0.2237	0.0865	0.1772	1.5267	
0.3508	0.204	0.3442	0.2175	0.3581	0.4108	0.4279	0.0345	
0.2564	0.4919	0.4368	0.3349	0.2957	0.6493	0.1568	0.5081	
0.3309	0.2372	0.1041	0.1823	0	0.1167	0.1212	0.0186	
0.0133	0.2497	0.1211	0.1017	0.3791	0.4322	0.2638	0.1836	
0.1463	0.292							
CCDC152	0.1692	0.0162	0	0.1651	0.2161	0.0528	0.0594	
0.0299	0	0.0217	0.0158	1.1883	0.0271	0.7239	0.0743	
0.0845	1.9822	0.0203	0.1596	0	0	0.0054	0.2202	

0.0204	0.0039	0.0335	0.032	0	0	0	0	
2.4666	0.0165	0.059	0.06	0.0293	0.0763	0.0235	0.0065	
0.0834	0.0746	2.5049	0	0.0265	1.3267	0.2193	0.5962	0
	0.0394	0	0.0111	0.0906	0.0995	0	0	
1.1068	2.2934	0	0.2702	0.0183	0.105	0.1123	0	0
	0.0219	1.4597	0	0	0	0.0382	0.0096	
0.0464	0	2.1947	0	0	0	0.0306	0.0603	0
	0	0.1439	0.8759	0	0.2876	0.0777	0.1397	
0.2589	0.2538							
CCDC153	0.7539	0.2961	0.2701	0.1252	0.6011	0.1202	0.2251	
0.2384	0.1179	0.0758	0.2402	0.5152	0.1359	0.4411	0.1064	
0.2298	0.4216	0.2661	0	0.4572	0	0.0505	0.1288	
0.1098	0.1411	0.068	0.2203	0.2067	0.208	0.2206	1.8036	
0.1911	0.4864	0.3539	0.0416	0	0.1879	0.5033	0.1735	0
	1.0737	1.083	0	0.193	0.105	0.1386	0.1935	
0.2491	0.361	1.5331	0.8565	0.0856	0.1358	0.8866	0.6775	
0.4552	1.2209	0.4115	0.0273	0.145	0	0	0.198	
0.1977	0.2253	0.4894	1.5355	0.0271	0.1822	0.2491	0.5786	
0.2771	0.0279	0.1287	0.2718	0.0875	0.8203	0.4567	0.1547	
0.1796	0.2587	0.076	0.1569	0.0201	0.3188	0.097	0.2598	
0.3009	0.3858							
CCDC154	1.6523	1.1689	0.7583	0	0.0511	0.3072	0.4079	
0.193	0.8982	0.4342	0.3248	0.0779	0.2935	1.0622	0.5897	
1.1318	0.2595	0.5881	0	0.2577	0.8528	0.1559	0	
0.7919	0.2131	0.6395	0.1878	1.5814	0	0.5398	0.2376	
1.4607	0.4963	0.3578	0.9218	0	0.5036	0.5573	0.3887	0
	1.7078	0.1493	0.2618	0.3264	0	0	0.3063	
1.0018	0.584	0.4902	0.5136	0	0.8652	0.8689	0.278	
0.7156	0.1243	0.1399	0.3229	0.0178	0.379	0.3094	0.6705	
1.2971	0.8113	0.8804	1.1365	1.1188	0.8947	0.098	0.1834	
0.2046	1.003	0	0.0532	0.9427	1.3151	0.1678	0.7559	
0.1887	1.2266	0.0646	1.7662	1.275	0	0.7826	1.2075	
1.1137	0.9931							
CCDC155	0	0.0944	0	0	0	0.1798	0.0646	
0.0574	0	0.018	0.0405	0.0825	0.089	0	0	0
	0.0379	0	0	0	0	0.0193	0.0706	
0.0864	0	0	0.0304	0	0.0968	0.1993	0	
0.0337	0	0.4305	0	0	0.0584	0	0	
0.1298	0.3576	0.0794	0.038	0.1123	0	0	0	
0.0788	0	0	0	0	0	0.1778	0	0
	0.2312	0	0.0359	0	0.1598	0	0	
0.0874	0	0	0.0598	0.0263	0	0	0.0287	
0.0279	0.0345	0.012	0.1208	0	0	0.0684	0.0397	0
	0	0.0443	0	0.0622	0	0	0	0
	0							
CCDC157	0.3618	0.4995	0.762	0.3352	0.3439	0.7125	0.3764	
0.7867	0.2873	0.2989	0.2573	0.2757	0.5114	0.4355	0.5444	
0.4839	0.4174	0	0.3863	0.9908	0.5955	0.5555	0.5295	
0.676	0.4584	0.5176	0.2955	0.8453	0.3597	0.3715	0.5132	
0.3497	0.5448	0.3595	0.3592	0.2896	0.3227	0.7184	0.386	

0.2598	0.7727	0.3628	0	0.4594	0.2657	0.3609	0.3158	
0.5249	0.5847	0.5635	0.416	0.2879	0.3387	0.6706	0.4595	
0.7735	0.4178	0.5782	0.7407	0.5935	0.6883	0.8071	0.9215	
0.809	0.6185	0.2284	0.9148	0.4292	0.7834	0.4386	0.5815	
0.8324	0.7143	0.8638	0.6948	0.6992	0.9779	0.6467	0.6457	
0.8056	0.829	0.5525	0.6395	0.3386	0.4782	0.6074	0.4243	
0.5438	0.5872							
CCDC158	0.0676	0.0306	0	0.2187	0	0.0557	0.0362	
0.0116	0.0054	0	0	0	0	0	8.00E-04	
0.0418	0	0.0217	0	0.0447	0.0301	0	0	
0.1038	1.00E-04	0	0.0138	0	0	0.0122	0	
0.0072	0.0424	0.0176	0.0629	0	0.0311	0.0232	0	
0.0176	0.0229	0.1092	0	0	0.0141	0.0288	0	
0.0835	0	0.0556	0	0.0809	0	0	0.0264	0
	0	0	0	0	0	0.0795	0	0
	0	0.2705	0.2751	0	0	0.0258	0.0235	
0.0079	0.0427	0.004	0.0346	0	0	0.0681	0.0043	0
	0	0.0578	0	0.0174	0	0	0.0349	0
	0.0658							
CCDC159	2.3726	1.2774	1.385	0.7587	1.3993	1.2881	1.8989	
2.0842	1.7704	2.3419	1.8068	1.6015	2.5802	1.7963	2.2992	
2.1256	1.6044	2.0838	1.6201	0.7094	1.2838	1.1605	2.0716	
2.1915	1.5245	2.6524	2.3108	1.9557	1.6696	1.5943	1.7213	
0.972	1.9025	2.9729	2.9103	2.062	2.8489	3.23	2.408	
1.9886	2.5993	1.6975	2.0093	2.128	1.6918	1.9691	1.6818	
1.6414	2.7535	1.5536	1.7218	1.9142	2.2281	2.1902	1.2502	
2.1184	1.6532	1.3629	2.1989	1.0115	1.9097	1.9808	1.3766	
2.0308	2.2643	3.2452	2.5961	2.3514	1.7077	1.0794	2.1712	
2.2744	1.9648	1.6855	1.3936	1.5946	2.0421	2.3037	2.1707	
1.9794	1.2566	1.958	2.6012	2.9214	1.9489	2.312	2.7248	
2.4176	3.0456							
CCDC160	0.54	0.283	0.2095	0.1418	0.1768	0.7558	0.4895	
0.2492	0.3494	0.087	0.0888	0.0947	0.0312	0.1471	0.2617	
1.1991	0	0.3799	0.1055	1.0816	0.4467	0.1852	0.1175	
0.4049	0.2207	0.052	0.2984	1.5585	0.1258	1.4837	0.9112	
0.0845	0.0453	0.1745	0.3295	0.4038	0.2807	0.4213	0.1797	
0.2652	0.4799	0.158	0.0498	0	0	0	0.1269	0
	0.1654	0.3782	0.215	0.2688	0.1665	0.1292	0.9378	
0.3248	0	0.1404	0.4251	0.1899	0	0.006	0.1033	
0.5376	0	0.5741	0.955	0.7724	0.2055	0.1094	0.4028	
0.3449	0.0164	0.2055	0.2402	0.9941	0.4195	0.9362	0.1563	0
	0.0093	0	0.006	0.1308	0.2624	0.0872	0.1495	
0.1018	0.1807							
CCDC166	0	0	0	0	0	0	0	0
	0	0	0	0	0	0	0	
0.0878	0	0	0	0	0	0	0	0
	0	0	0	0	0	0	0	0
	0	0	0	0	0	0	0.085	0
	0	0	0	0	0	0	0	0
	0	0	0	0	0	0	0	0

	0	0	0	0	0	0	0	0
	0	0	0	0.0313	0	0	0	0
	0	0	0	0	0	0	0	0
	0	0	0.0548	0	0	0	0	0
	0							
CCDC167	4.8161	5.2379	5.2113	4.1599	3.5011	3.857	3.3815	
4.7254	5.1588	5.7403	4.9518	4.5004	3.1867	3.7826	4.9885	
4.0845	4.9833	4.6215	4.4732	4.2424	4.1445	5.2439	4.4865	
4.6866	5.044	4.7913	3.8237	6.1602	4.1318	4.8119	5.1409	
4.1186	4.9313	5.1195	5.3369	5.4271	4.821	5.4539	5.3137	
3.7225	4.7315	3.6087	4.8038	4.3853	3.6048	4.2594	3.9291	
4.7823	3.879	4.0677	4.8532	3.4014	4.4527	3.9533	5.7735	
4.5392	3.5563	5.4615	5.0838	5.0877	4.5493	4.7579	5.1297	
4.4519	6.3595	2.7933	6.1899	4.2795	4.7197	4.3326	4.8007	
4.8058	4.6822	4.6099	4.5397	4.943	5.1112	5.7101	4.4257	
5.1042	4.3642	4.1751	4.6918	4.848	4.9279	5.1467	4.6714	
5.0341	5.6358							
CCDC168	0.0255	0.0161	0.0419	0.0218	0.0024	0.0252	0.0047	
0.0043	0.0113	0.0069	0.0017	0.0019	0.0033	0.0253	0.0043	
0.0034	0.0223	0.0266	0.071	0.0226	0.0266	0.0011	0.0015	
0.0169	0.0041	0.0113	0	0.0502	0.0088	0.0095	0.0103	
1.00E-04	0.0125	0.0074	3.00E-04	0.0087	0.0014	0.0164	0	
0.0103	0.0063	6.00E-04	0.0183	0.0109	0.0071	0.008	0.0223	
0.0135	0.0063	0	0.0073	0.0174	0.0377	0.0032	0.0264	
0.0714	0.0219	0.0235	0.0193	0.0132	0	0.0039	0.0062	
0.0144	0.0161	0.0012	0.0236	0.0013	0.032	0.032	0.0229	
0.0059	0.0099	7.00E-04	0.018	0	0.0172	0	0.0028	
0.0191	0.0124	0.0035	0.0052	0.013	0	0.0306	0.0167	
0.0196	0.0255							
CCDC169	0.6418	0	0.3683	2.0657	0.3297	1.1386	0	
2.3937	0.1074	0.1613	0.1932	1.6181	1.5138	1.3045	0.0797	
0.0353	2.1955	0.1408	2.0731	0.2261	0.5286	0	0.1867	
0.0572	0.2721	1.0254	0.2207	0	0.18	0.1381	0.0873	
0.8815	0.2632	0.9153	0.0912	0.2556	2.0662	0.2357	0.9164	0
	1.1697	1.055	1.6864	0.5791	0.3177	1.1229	1.5264	0
	0.0094	1.256	1.5658	0.8789	0.3874	0.4648	0	
1.3162	2.1706	0.1215	0.2795	0.1497	0.9324	0	0.5534	0
	2.0334	0.746	2.7199	0.1557	0.2413	1.8509	0.2969	
0.6739	1.1263	0.461	0	0.2673	0	0.1619	0.4698	
0.6375	0	1.9023	0.8141	0.6821	0.35	0.4712	0.8145	
1.508	1.4866							
CCDC17	0.474	0.3794	0.4512	0.4873	0.1283	0.6181	0.3014	
0.243	0.8089	0.2991	0.2685	0.1858	0.1535	1.0465	0.317	
0.2308	0.9434	0.3283	0.5	0.578	0.722	0.151	0.0576	
0.6242	0.4313	0.344	0.3321	0.374	0.2402	0.2477	0.2536	
0.4153	0.358	0.2054	0.317	0.317	0.264	0.1596	0.1209	
0.9644	0.4592	0.1896	0.1806	0.2337	0.2875	0.3675	0.2445	
0.3349	0.4056	0.4084	0.2025	0.6192	0.5694	0.3587	0.2752	
0.9025	0.3933	0.1246	0.5594	0.2628	0.282	0.3033	0.1983	
0.4486	0.1519	0.2023	0.359	0.0737	0.1523	0.1716	0.3181	

0.4414	0.1666	0.344	0.2427	0.114	0.7469	0.0522	0.2128	
0.8664	0.4607	0.2146	0.3276	0.2568	0.344	0.7505	0.1298	
0.1913	0.3837							
CCDC170	0.0364	0.0116	0.0622	0.0541	7.00E-04	0.0216	0.035	0
	0.028	0.0072	0.0284	0	0.101	0.0457	0.0083	0
	0	0.0175	0.0562	0	0.0237	0	0.0022	
0.0066	0.014	0.0385	0.0486	0.028	0	0.028	0.2118	
0.0326	0.0177	0.0447	0.0012	0.0075	0.0628	0	0.0064	
0.0108	0.0543	0.0647	0.0622	0.0116	0.0092	0.013	0.0318	
0.019	0.0465	0.0672	0.0861	0	0.072	0.0285	0.0696	
0.0301	0.3672	0.0625	0.0258	0.0234	0.014	0	0	
0.0301	0.0486	0.126	0.0285	0.0611	0.0325	0.0435	0.0153	
0.0186	0.0289	0.0042	0.0453	0.0606	0.0248	0.0195	0.0212	
0.0257	0.0065	0.0073	0.0402	0.0468	0.0425	0.0329	0.2571	
0.2399	0.0499							
CCDC171	1.6976	2.9061	2.1976	0.6901	0.795	0.8772	0.6164	
0.5246	0.8766	1.3132	0.6424	0.6263	0.7144	0.9115	0.5351	
1.08	1.6598	1.3181	1.2682	1.4816	2.2621	0.2936	0.3079	
1.0719	0.423	0.3928	0.2725	2.7079	0.6876	0.7985	0.6828	
1.0484	1.174	0.8987	0.6501	1.5377	0.8306	0.8505	1.1447	
1.0982	2.431	0.3675	0.4766	0.7686	0.5476	0.5422	2.1863	
1.0812	0.4742	1.5574	0.6645	0.5272	1.0685	2.1089	0.7895	
2.2742	0.895	0.7875	1.7498	0.7868	0.7346	2.1469	0.1743	
1.2421	1.3672	0.6773	2.2737	0.9503	1.1745	0.9271	0.9877	
1.1924	1.7047	0.2979	0.4453	0.9435	2.0377	0.7374	0.8964	
1.3119	1.2261	0.3126	1.1108	0.2795	0.3061	1.0629	1.4888	
1.3979	2.8724							
CCDC172	0	0	0	0	0	0	0	0
	0	0	0	0	0	0	0	0
	0	0	0	0	0	0.0443	0	0
	0	0	0	0	0.0264	0	0	
0.0045	0	0	0	0	0.0125	0	0	0
	0	0	0	0	0	0	0	0
	0	0	0	0	0	0	0	0
	0	0	0	0	0	0	0	0
	0	0	0	0	0	0	0	0
	0	0	0	0	0	0	0	0
	0	0	0	0	0	0	0	0
	0							
CCDC173	0.2667	0.4823	0.2893	0.3821	0.2329	0.2783	0.1708	
0.2114	0.1815	0.1862	0.097	0.2935	0.3577	0.5107	0.4466	
0.1658	0.4728	0.139	0.2154	0.6097	0.2592	0.2304	0.0822	
0.3541	0.1302	0.7258	0.2147	0.5221	0.2094	0.1875	0.7352	
0.6584	0.4253	0.4056	0.4475	0.2237	0.2601	0.1331	0.1355	
0.4206	0.2365	0.2152	0.1146	0.1676	0.1786	0.5379	0.271	
0.5253	0.7703	0.8201	0.1634	0.5913	1.406	0.1857	0.7608	
0.2157	0.2393	0.2031	0.2203	0.185	0.2713	0.1634	0.0847	
0.3894	0.2527	0.2577	0.4599	0.1715	0.2268	0.3155	0.1474	
0.438	0.4362	0.3193	0.2471	0.2676	0.419	0.4887	0.1855	
0.1097	0.3746	0.1711	0.2279	0.1908	0.959	0.3599	0.218	

0.1407	0.1178							
CCDC174	2.6617	2.8117	2.5471	2.3803	2.4034	2.5937	2.8767	
2.1903	1.9958	2.4654	2.8554	3.2422	3.0082	2.8098	2.8189	
3.9828	2.8908	2.9266	2.7645	2.1217	2.571	2.6374	2.1824	
2.3362	2.4796	2.7067	2.7348	2.6349	2.6096	2.8185	3.1873	
2.5921	2.3414	2.2593	2.3737	2.6432	3.6776	2.6673	3.3663	
2.3309	2.5451	2.5608	2.3869	2.5451	3.1689	2.8373	3.2332	
2.5455	2.601	3.0811	2.5997	2.1611	2.4324	3.1754	2.6852	
2.3232	3.0964	2.1532	2.4748	2.1397	2.9852	2.5904	2.3524	
2.2506	2.61	2.7782	3.2458	3.0895	3.1913	2.5049	2.793	
2.2686	2.8357	3.1267	3.4045	2.4299	2.6619	2.9369	2.2534	
2.1887	2.9919	2.7146	3.145	2.3871	2.8301	2.3841	2.8378	
2.6782	2.7743							
CCDC175	0.0909	0.0765	0.1379	0.2551	0.0397	0.2246	0.0665	
0.2962	0.1193	0.1126	0.0781	0.0965	0.0682	0.2421	0.2747	
0.189	0.1987	0.2686	0.5115	0.3577	0.2062	0.0742	0.0638	
0.4853	0.0777	0.0217	0.1288	0.357	0.128	0.0544	0.3096	
0.1886	0.1932	0.1924	0.0545	0.1195	0.0268	0.0868	0.0196	
0.105	0.0612	0.064	0.1479	0.1564	0.3229	0.2491	0.1728	
0.0859	0.1607	0.5163	0.1359	0.2664	0.275	0.0808	0.3993	
0.1379	0.2003	0.0933	0.168	0.1194	0	0.0781	0.1515	
0.1148	0.0904	0.2021	0.5321	0.1786	0.1209	0.1643	0.1321	
0.0952	0.0418	0.0611	0.2341	0.2913	0.0886	0.0191	0.1459	
0.2267	0.1366	0.163	0.1908	0.1217	0.1698	0.3039	0.0839	
0.2441	0.1755							
CCDC177	0	0.0278	0	0	0	0	0	0
	0	0	0.0105	0	0	0	0	0
	0	0	0	0	0	0	0	0
	0.0106	0	0	0	0	0	0.239	0
	0	0	0	0.0176	0.0085	0	0	0
	0	0	0	0	0	0	0.0397	0
	0	0	0	0.028	0	0.0023	0.0796	0
	0	0.0332	0	0.0328	0	0	0.0396	0
	0.0454	0.0829	0.0862	0	0.0589	0	0	0
	0.0341	0	0	0	0	0.0115	0	0
	0	0	0	0	0.0299	0	0.0559	0
	0							
CCDC178	0	0	0.2377	0	0.0241	0.0664	0.0666	0
	0	0	0	0	0	0.1795	0.027	
0.0322	0	0	0	0	0	0	0	0
	0	0	0	0.0808	0.037	0.0382	0	
0.0422	0.1994	0	0.0733	0.0056	0.059	0.0604	0	0
	0	0	0	0	0	1.1381	0.0546	
0.0659	0	0	0	0	0	0.0768	0	0
	0	0.0421	0	0.0383	0	0	0	
0.3113	0	0.0293	0	0.2446	0	0	0	0
	0	0	0.0461	0	0.0777	0	0.0797	0
	0	0	0	0.137	0	0	0	
0.074	0							
CCDC179	0	0	0	0	0	0	0	

0.1038	0.0517	0	0	0	0.1043	0	0.092	0
	0	0	0	0	0	0	0	0
	0	0.0506	0	0	0	0.0562	0	
0.0323	0	0	0.1169	0.042	0	0	0	0
	0	0	0	0	0	0	0	
0.0879	0	0	0	0	0.1048	0	0	0
	0	0	0	0	0	0	0	0
	0	0	0	0	0.0676	0	0	0
	0.0853	0	0	0	0.1955	0	0	0
	0	0.0571	0	0	0	0	0.0935	0
	0							
CCDC18	1.7143	2.2466	1.9182	2.683	1.479	1.8742	1.3427	
1.3616	1.4534	1.6158	0.9549	1.8375	2.3908	2.6246	2.2609	
0.8967	2.1757	2.3159	3.2379	1.4906	2.3012	1.0088	1.0747	
2.2121	0.9895	1.6811	0.7263	1.9417	1.2854	1.2343	2.2955	
1.8966	2.4981	1.6748	3.9423	2.4563	1.5188	0.787	1.7571	
1.8359	1.3807	1.4052	2.7492	2.2108	1.9005	3.0383	2.1698	
1.385	1.6725	4.4634	1.9693	2.0855	2.3722	1.6461	1.9291	
2.7482	2.4205	2.1564	2.0517	2.0325	1.3878	1.9716	1.488	
2.7105	2.0228	0.7779	2.7741	0.9326	1.596	2.0276	1.361	
2.507	1.9022	2.5043	1.3075	0.8204	2.822	0.5295	2.6806	
1.971	2.7487	1.3728	0.9194	0.9853	3.0169	3.2286	1.7535	
2.4116	2.0703							
CCDC180	0.6753	0.2701	0.5953	0.5378	0.2459	0.2517	0.7557	0
	0.7189	0.0407	0.7207	0.307	0.5186	0.5827	0.6181	
0.0158	0.72	0.0798	0.0549	0.2173	0.5098	0.1307	0.0785	0
	0.4254	0.307	0.2262	0.8642	0.1257	0.2855	0.8895	
0.3551	0.2848	1.2698	0.2093	0.1962	0.5648	0.5856	0.1765	
0.4054	0.7313	0.2949	0.7096	1.6119	0.0189	0.5453	0.3018	
0.3448	0.9222	1.4939	0.3676	0.3691	0.619	0.6047	0.7694	
0.0323	0	0.4079	1.002	0.0586	0.1797	0.022	0.2409	
0.4785	0.065	0.6023	0	0.4588	0.4034	0.5536	0.2194	
0.2812	0.1529	0.1457	0.4151	0.4428	0.4334	0.718	0.9686	
0.9564	0.478	0.9298	0.3793	0.2088	0.3485	0.5765	0.5245	
0.4966	0.4154							
CCDC181	0.0365	2.2186	0.8819	1.2435	1.8117	1.2916	0.7748	
1.9506	0.5191	0.7716	2.4629	1.2094	0.9746	1.1555	1.3117	
1.4545	0.0952	0.4207	2.1471	0	0.0113	0.917	0.8302	
2.0106	1.9247	1.3963	2.5458	0	0.6577	1.6217	1.6847	
1.2863	0	1.1349	0.2433	1.2161	1.5695	0.145	1.3963	
1.522	0.2636	1.2748	0.0182	1.2115	0.2034	1.1872	0.2958	
1.8485	1.5584	0.8142	0.2895	2.1842	0.1992	1.6021	1.3901	
0.6154	0.1805	2.0764	1.4497	2.3709	0.3966	0.5984	1.197	
0.1372	0	1.357	0.1519	0.1623	2.1174	1.9108	0.7074	
1.9704	0.1675	1.5726	2.3593	0.9243	0.4736	1.2248	0.2891	
0.8611	0.4789	0.7645	1.4812	2.3479	1.8837	0.4986	0.0797	
0.6255	0.5559							
CCDC182	0	0	0	0	0	0	0	0
	0	0	0	0	0	0	0	0
	0	0	0	0	0	0	0	0

	0	0	0	0	0	0	0	0
	0	0	0	0	0	0	0	0
	0	0	0	0	0	0	0	0
	0	0	0	0	0	0	0	0
	0	0	0	0	0	0	0	0
	0	0	0	0	0	0	0	0
	0	0	0	0	0	0	0	0
	0	0	0	0	0	0	0	0
	0							
CCDC183	0.4895	0.2419	0.3589	0.1981	0.1348	0.2894	0.1584	
0.4885	0.2685	0.1295	0.4558	0.385	0.2669	0.0904	0.1643	
0.2324	0.3017	0.1153	0.1705	0.478	0.2854	0.0875	0.0876	
0.2706	0.1607	0.0676	0.4773	0.5405	0.054	0.4439	0.2654	
0.2515	0.2578	0.2651	0.1086	0.0737	0.2135	0.4348	0.1872	
0.0588	1.0241	0.4881	0.1895	0.0599	0.2399	0.1091	0	
0.187	0.1928	0.1142	0.3098	0.2058	0.1364	0.2796	0.5492	
0.4194	0.2548	0.2083	0.2063	0.0854	0.1374	0.2147	0.2066	
0.3228	0.4807	0.3871	0.4734	0.0808	0.1872	0.4613	0.1754	
0.0193	0.0258	0.14	0.0845	0.6844	0.2205	0.4426	0.3526	
0.3205	0.2796	0.3657	0.1329	0.2753	0.053	0.2325	0.0653	
0.1714	0.1626							
CCDC184	0.2678	0.2113	0.5332	0.3489	1.0101	0.1129	0.4029	
0.2552	0.3613	0.2325	0.4367	0.6316	0.9478	0.9288	0.236	
0.2399	1.1995	0	0	0.9261	0.6602	0.1158	0.8419	
0.1908	0.9975	1.645	0.6095	0.4227	0.1923	0.1497	2.5485	
0.5094	0.2478	0.0275	0.3346	0.5469	0.8739	0.2993	0.1129	
0.0292	0.1777	0.4187	0.9261	0.6322	0.0705	0.1376	0.2027	
0.187	0.6761	1.3379	0.2325	0.2881	0.9535	0.3548	0.4369	
0.131	0.2224	0.2436	0.3622	0.1743	0.411	0.0749	0.3973	
0.3346	0.0757	2.2798	0.2974	1.8919	0.3949	0.5958	1.1271	
0.7611	0.3064	0.9977	0.7648	0.1894	0.1047	0.1033	0.2268	
0.5777	0.133	0.3897	0.8455	0.8592	1.0606	0.2725	0.3592	
0.6499	0.4037							
CCDC22	3.2427	3.2245	2.9901	1.7132	3.0034	2.4798	2.5528	
2.7273	3.0486	2.705	2.5258	2.7038	2.9677	2.4124	3.1627	
2.9342	2.3743	2.0905	1.9387	2.9383	2.5575	3.007	2.7192	
3.5328	1.8332	2.6224	2.6678	2.6907	2.5784	2.0744	3.435	
2.9625	3.2825	3.8804	3.0733	2.4817	3.5009	3.7348	2.7668	
2.4202	2.4777	2.669	2.475	2.3238	2.2826	2.2955	2.3258	
2.3597	1.7318	2.339	2.561	1.9398	2.3675	2.8764	4.1058	
3.3008	3.1471	2.7602	2.0889	2.623	2.3417	2.9348	3.2547	
3.1989	2.6509	2.5294	2.8888	2.6089	2.7574	2.3452	2.2588	
2.5181	3.0049	2.8246	2.5258	2.8396	2.9342	3.0506	2.6161	
2.5849	2.8556	1.6121	3.4911	2.3528	2.7277	2.7148	2.891	
3.0536	2.9807							
CCDC24	1.6381	1.9242	1.9398	0.8677	1.7729	1.0422	2.0097	
1.6375	1.5032	1.8993	2.748	1.5838	1.9766	1.0861	1.2542	
1.436	1.9559	1.0607	1.2255	1.942	1.9128	1.5933	2.1167	
1.0931	1.3864	1.4643	1.0994	1.7815	1.8132	2.3513	2.7635	
2.2007	1.395	1.3209	1.6381	1.2275	1.7284	1.9286	0.8953	

1.1921	2.9042	2.9595	1.7231	1.135	1.55	1.4431	0.8387	
1.8354	1.9979	1.687	1.774	1.3318	0.9592	1.5231	2.1036	
1.2445	1.6383	1.2126	1.9356	0.9716	1.4726	1.8751	0.5782	
1.0552	0.661	2.8885	1.7332	2.0687	2.2397	1.4201	2.2319	
1.5138	1.3289	1.6274	0.8682	1.609	1.754	1.5225	1.5904	
2.0853	1.9946	1.8383	1.4865	0.8696	1.9644	1.5461	1.3234	
1.1025	1.2977							
CCDC25	4.0436	4.1459	3.0927	4.1268	3.6976	2.3757	3.7973	
3.7658	3.4169	3.5097	3.5426	4.5275	3.926	4.1729	1.9506	
2.8822	3.3363	4.0314	4.0787	4.0086	3.5397	4.0052	3.8984	
3.8702	3.9745	3.3471	4.2081	4.3313	4.1523	4.2769	4.2131	
4.3591	3.0654	4.237	3.6369	3.9533	4.1594	3.9253	3.688	
3.62	4.7586	3.7709	3.1275	3.9422	3.805	3.9397	3.9846	
2.9188	3.2551	4.6944	4.0197	4.4011	4.2787	3.8335	3.8834	
3.6628	2.7672	3.4218	4.2078	2.7739	3.8347	4.5533	3.9003	
3.2706	4.9858	2.9688	4.0069	3.7499	2.8926	4.1857	3.8663	
3.4011	3.9484	4.4965	3.7492	3.3319	4.0702	4.0052	3.2792	
3.8053	3.5093	4.6305	3.4153	3.5016	3.718	4.12	3.9437	
3.6175	4.0086							
CCDC27	0	0	0	0	0.0157	0	0	
0.0148	0	0	0.0075	0	0	0	0	0
	0	0.0379	0	0	0	0	0	0
	0.0222	0	0	0	0	0.0244	0.0553	0
	0	0	0	0	0.005	0	0	0
	0.033	0	0	0	0	0.0223	0	0
	0	0	0	0	0	0	0.0475	0
	0	0	0	0.0215	0	0	0	0
	0.0508	0	0	0	0	0	0	0
	0	0	0	0	0	0	0	0
	0	0.0089	0	0	0	0	0	0
	0							
CCDC28A	2.8901	3.4722	2.5071	3.6098	3.2043	2.9049	2.3799	
2.6253	3.1196	2.9251	3.2927	3.646	3.2135	3.492	2.3455	
2.6597	3.7555	3.849	3.1925	2.8558	3.0656	4.0433	3.9459	
2.4396	2.0835	2.9505	5.8424	2.4834	3.4193	4.2341	3.2992	
3.5319	3.1431	3.4597	2.9441	3.2205	2.9013	3.2697	3.3387	
3.1584	3.2813	3.7836	2.8353	2.6188	3.0226	3.9727	2.7212	
2.7111	2.704	3.7296	3.606	3.2927	3.4159	3.6839	3.793	
3.4608	4.3768	3.0142	2.6987	3.4587	3.2027	3.6158	2.0894	
2.7036	1.9717	2.5582	2.1321	2.0761	2.9447	3.2591	2.2202	
3.7513	2.9761	4.1492	3.3825	2.531	2.7215	3.1413	2.6223	
3.2977	3.3671	3.3297	3.3841	3.3417	3.1949	3.501	3.8359	
2.9663	3.4786							
CCDC28B	2.2697	3.157	1.2317	2.0667	4.4166	1.3955	2.6148	
2.0652	3.4063	2.5778	1.8291	3.0111	2.3724	2.2208	0.9409	
2.9352	2.3473	1.9231	1.8291	3.2898	2.8305	3.6519	3.3985	
2.891	3.0805	2.3916	2.9296	2.702	2.446	3.5248	3.3541	
3.0688	2.8194	2.6437	3.7886	2.6493	2.24	3.3274	2.9446	
1.7042	3.8327	4.4925	2.6477	2.6469	2.5548	1.7326	1.2513	
3.1158	2.7823	2.9605	2.7029	2.2673	2.6066	1.9167	2.2791	

3.5889	2.2025	2.9308	2.1367	3.0525	3.2625	3.4096	3.5641	
2.2204	2.6465	2.0358	3.4355	2.4463	2.6617	1.5786	2.9647	
2.8055	3.643	4.182	3.4426	2.7206	3.2464	3.8139	2.7936	
3.6816	3.3778	3.4088	1.3224	3.0616	3.672	3.6928	2.7497	
3.1226	3.1286							
CCDC3	0.1109	0	0.3055	0.0967	0.0453	0.13	0.3493	
0.0538	0.8098	0.0644	0.1047	0.3967	0.3153	0.1081	1.0003	
0.6128	0.2874	0.1362	0	1.47	0.6513	2.1781	2.3968	
0.8523	0.6055	0.6513	0.2064	0.1762	0.1592	0.3885	3.7028	
0.1539	0.1053	0.0819	0.0975	1.7257	0.0644	0.1629	0.3438	0.2
	0.0939	0.0963	0.1304	0.1129	0.0753	0.1667	2.3574	
0.0804	0.1523	0.2673	0.0686	0.2002	0	0	1.9417	
0.0204	0.0309	1.3809	0.0161	3.9365	0	0.5628	0.1048	
0.5619	1.4044	2.2641	4.2804	2.4529	0.2112	0.1238	0.0519	
0.3867	0.0833	0.0185	0.0409	1.0027	0.0544	0.0941	0.0525	
0.0302	0.0502	0.0279	0.0443	0.1415	0.0367	0.015	0.8613	
0.9763	1.2443							
CCDC30	0.3847	0.1102	0.5105	0.2047	0.0264	0.3059	1.6466	
0.137	0.3216	0.2708	0.4059	0.3029	0.2956	0.5999	0.1808	
0.1931	0.4889	0.3876	0.4673	0.687	0.576	0.0129	0	
0.3898	0.0577	0.4635	0.4165	0.4553	0.0378	0.2631	0.3662	
0.2897	0.2115	0.6039	0.2949	0.2479	0.5162	0.262	0.1107	
0.1151	0.9207	0.2483	0.0676	0.0978	0.2075	0.1145	0.8265	
0.3183	0.2123	0.6474	0.2771	0.1466	0.1652	0.3473	0.2104	
0.3918	0.3393	0.12	0.1457	0.1714	0.2345	0.1979	0.1486	
0.2999	0.1683	0.5981	0.5374	1.0168	0.3476	0.1628	0.1391	
0.6427	0.2407	0.1096	0.2117	0.2028	0.3808	0.3543	0.4781	
0.2691	0.6658	0.1048	0.5007	0.1671	0.1667	0.2089	0.3589	
0.3117	0.5947							
CCDC33	0	0	0	0	0.0095	0	0.0199	0
	0	0	0	0	0.036	0	0	0
	0	0	0	0	0	0	0	0
	0	0.0166	0.0197	0	0	0	0	
0.0027	0	0	0	0	0	0	0.017	0
	0	0.1679	0.7152	0	0	0	0	0
	0	0	0	0	0	0	0	0
	0	0	0.0346	0	0	0	0.0204	0
	0	6.00E-04	0	0	0.0173	0.1334	0	0
	0	0.0071	0.0209	0.0287	0	0	0.0372	0
	0.0572	0	0	0	0.3689	0	0	0
	0							
CCDC34	2.9391	3.8247	3.7386	3.866	2.9776	3.7482	3.4178	
3.5089	3.7364	2.9365	3.7762	2.9621	3.4982	3.6739	3.9272	
3.736	3.0284	2.6127	4.6038	4.1468	3.7281	3.946	3.3451	
3.8165	4.0015	2.9513	3.5339	4.038	4.1141	3.1854	3.7452	
3.749	4.4622	3.2539	3.5279	3.8786	2.7237	2.3401	2.2842	
3.7482	3.0868	2.852	3.6531	3.3539	2.6581	4.5127	3.9573	
3.7905	3.0752	4.1933	3.8441	3.5122	3.5398	2.9723	4.806	
3.7161	2.9044	3.2286	3.3762	3.7173	3.4401	4.0229	3.6054	
3.7315	3.555	3.3055	3.5528	2.5127	3.7683	3.4674	3.8903	

3.931	3.9055	4.2473	3.8938	4.732	3.5537	3.0545	3.7648	
4.242	3.3436	2.951	3.3702	2.3649	4.179	3.9642	2.7246	
3.5702	2.799							
CCDC36	1.5782	1.0459	0.0418	0	0.0389	0.553	0.5925	
0.022	1.595	2.6066	1.3028	1.8983	0.3653	0.0378	0.7476	
0.1242	1.403	1.3888	1.9104	0.0977	0.0964	0.2151	0	
0.019	0.0019	0.0754	0.209	0.0207	0.0139	0.7606	0.0855	
1.547	1.8801	1.8806	0.1135	2.163	1.3021	0	0.6095	
0.8316	0.0524	0.0581	0.1008	1.0893	2.902	2.2077	0	
0.291	0.8857	1.8261	0.0664	0.0961	1.6828	1.6467	0.0795	
0.3917	0.0845	0.1341	0.1359	0.1531	0.8596	1.6728	0.1769	
1.7023	0.0821	1.5664	0.3096	0.5751	1.0164	0.1053	0.1125	
1.5346	1.0452	2.0712	2.1142	0.5702	0.1807	0.0877	1.6067	
1.3221	0.1412	0.1275	0.5166	0	0.1181	2.1817	1.1751	
1.2185	0.7681							
CCDC38	0.0596	0.0179	0.0319	0.028	0.0037	0	0	
0.01	0.01	0.0131	0	0	0.0233	0	0.0057	
0.0071	0	0.0239	0.0657	0	0	0	0	0
	0	0.0098	0.0156	0	0	0	0.0237	0
	0	0	0	0.0082	0.0043	0	0	0
	0	0.1124	0	0	0	0.0282	0.0228	
0.0193	0	0.0482	0	0	0	0.0126	0	0
	0	0	0	0.0356	0	0	0	0
	0	0.0042	0	0	0	0	0.0207	
0.027	0	0	0	0	0.0101	0	0	0
	0.0224	0	0.0219	0	0	0	0	0
	0							
CCDC39	0.1492	0.1206	0.1917	0.4844	0.2357	0.8468	0.3521	
0.5002	0.3511	0.4147	0.228	0.2084	0.2258	0.523	0.8042	
0.3668	0.7352	0.6649	0.6038	0.3319	0.716	0.1695	0.1906	
0.6793	0.1846	0.2021	0.1875	0.6126	0.2927	0.2032	0.2358	
0.4188	0.4677	0.2116	0.404	0.1876	0.1474	0.132	0.0504	
0.4345	0.1263	0.2512	0.1036	0.401	0.7417	0.1965	0.1708	
0.4374	0.1755	0.6152	0.4177	0.2921	0.5137	0.4361	0.0822	
0.6334	0.5736	0.3414	0.1639	0.3608	0.0987	0.2648	0.0175	
0.5178	0.1326	0.2475	0.7694	0.1645	0.3657	0.4959	0.1144	
0.3374	0.5516	0.2585	0.1448	0.4846	0.2132	0.4343	0.2121	
0.3755	0.4146	0.5872	0.1489	0.3968	0.1998	0.6155	0.153	
0.3319	0.2708							
CCDC40	1.1926	0.9975	1.1156	0.5825	0.6669	1.0645	0.8395	
0.6397	0.9546	0.4817	1.0219	0.4744	0.7368	0.4872	0.9949	
0.9198	1.106	0.9418	0.5517	1.5055	1.7365	0.3891	0.7461	
0.8278	0.7625	1.1568	1.1308	1.3601	0.6759	0.5934	1.1195	
1.5826	0.8093	0.7672	0.7762	0.5258	1.4017	0.9546	0.4506	
1.1046	1.1188	0.4721	0.3023	0.6226	0.5482	0.5522	1.3426	
0.761	1.1388	0.8901	0.9676	0.6565	0.9141	1.3661	0.983	
0.7128	0.19	0.5086	0.7654	1.0292	0.5052	0.4299	0.9601	
1.0089	1.6183	1.1928	1.1921	0.9492	0.6792	0.7654	0.693	
0.9552	0.875	0.6705	0.808	1.074	0.9249	1.0272	0.3828	
0.4552	0.9037	0.8038	0.6282	1.7351	0.677	0.707	0.5039	

0.9577	0.9975							
CCDC42	0.0729	0	0	0	0	0	0	0
	0.1107	0	0	0	0.0256	0.086	0	0
	0	0.138	0	0	0	0.0154	0	0
	0.0701	0.0285	0	0	0.0722	0.085	0.0804	0
	0.0929	0	0.0081	0	0	0	0	
0.089	0	0	0.0628	0	0	0	0	0
	0.0343	0	0	0	0	0	0.0445	0
	0	0	0	0	0	0	0.0343	0
	0	0	0	0	0	0	0	0
	0	0.0231	0.0309	0.04	0	0	0.172	0
	0.0331	0	0	0	0	0.203	0.1011	0
	0							
CCDC43	5.0054	4.9045	5.2383	4.7869	3.9415	4.4687	3.8763	
3.9579	4.1488	4.0728	4.3556	4.2706	3.8471	4.8241	3.8724	
4.5162	4.1796	4.2865	5.0149	4.2371	4.7879	3.5668	3.4624	
3.806	4.0155	4.147	4.0983	5.94	3.8235	4.176	4.0752	
4.428	4.4389	3.7733	4.1172	4.5546	4.7549	4.3103	3.5709	
4.4775	4.1496	3.772	3.6789	4.7279	4.3022	3.8442	4.8241	
4.6459	3.3972	4.7187	4.6827	4.0151	3.9814	4.3125	3.7381	
4.7285	4.0239	4.1769	4.2634	4.1997	4.6247	4.6581	4.1865	
4.7536	5.0282	3.7271	4.3492	4.2939	4.6814	4.2933	4.3119	
4.2871	5.5683	4.224	4.7995	5.2241	4.8917	4.2481	4.3929	
4.2163	4.0682	4.1923	3.4984	3.8175	4.0165	3.8552	4.5923	
4.9465	4.7496							
CCDC47	5.7781	5.7003	5.9377	6.5141	5.6761	5.992	5.9548	
5.4961	5.447	5.6011	5.3391	5.5715	5.9089	6.5095	5.8114	
6.0392	5.7082	5.7179	5.946	6.212	6.6826	5.6174	5.384	
6.3688	5.1663	5.7801	4.6909	5.8667	5.8713	5.5156	4.9946	
6.2074	5.8114	5.3194	5.9449	5.9257	5.9257	5.3424	5.0689	
6.0413	5.8507	5.1485	4.7311	5.3708	6.1939	5.5915	6.6572	
6.15	5.5005	5.6282	6.3659	5.7837	6.1999	6.1288	5.8498	
6.2299	4.5671	5.1996	5.7561	5.3758	5.8412	5.9162	5.8009	
5.6628	5.7109	5.4975	6.0731	5.1839	6.139	5.029	5.6777	
5.5479	6.1149	5.0724	6.0539	6.5415	5.7551	5.4621	5.4904	
6.133	5.8924	5.5394	5.9614	6.2461	5.12	6.1513	4.9264	
5.0159	4.7683							
CCDC50	3.7161	4.3076	4.1334	3.8528	3.1279	5.4907	4.466	
4.1699	4.6231	4.4779	4.4957	5.0753	4.5269	4.9118	5.3715	
4.6407	5.37	4.2569	4.5023	3.4778	4.1886	4.2197	4.0403	
6.0324	3.1792	4.3512	5.1978	2.7072	3.232	4.1735	2.2144	
4.7727	4.8301	5.002	4.3929	4.0076	3.4832	4.2229	5.0272	
5.333	5.2792	4.2401	4.0684	5.9244	3.9562	3.9758	3.2443	
4.6328	3.6014	3.9673	4.6992	4.6885	4.7683	5.2544	3.3687	
4.699	5.2145	3.188	3.8478	3.3187	4.2699	4.1954	5.3457	
3.8975	3.4044	3.8963	4.6615	3.384	2.9761	4.5434	6.081	
4.5609	4.3926	4.0651	4.0428	3.8568	3.6201	4.2618	5.3157	
6.0417	5.4629	5.2616	5.4559	4.818	4.2622	4.2799	4.4274	
4.8603	4.252							
CCDC51	4.205	4.6535	4.4136	3.9163	4.5295	4.093	3.7679	

4.213	3.2182	4.5045	4.756	4.2762	3.6182	3.2641	2.7646	
4.6607	3.7558	3.797	3.6383	3.8271	3.9252	3.7723	3.6855	
4.47	3.3331	3.0749	2.8758	4.3483	3.8941	4.2794	3.6836	
4.3072	3.9617	3.507	2.9035	4.2738	4.105	4.0189	4.0842	
3.3095	4.3487	3.5517	3.384	4.3188	3.7453	2.3365	4.359	
4.4079	3.0582	4.0817	3.5303	3.6494	3.4518	4.1344	4.1781	
3.5532	3.3453	3.6113	4.4033	3.3576	4.0096	4.7265	3.9122	
4.1143	4.2606	4.7952	4.6725	3.7076	3.8116	3.3804	4.0922	
3.1661	4.7146	4.2328	4.4028	3.6086	4.1819	3.7981	2.8271	
4.1594	3.7391	3.5952	3.1709	3.0424	3.7386	3.9886	3.7175	
4.0984	3.8054							
CCDC53	4.6707	3.9828	3.2796	3.845	3.8446	3.8226	4.463	
3.8132	4.9997	4.6796	5.0352	4.1453	4.5791	4.429	3.6698	
4.0844	4.2592	5.2839	4.9496	4.1133	4.808	3.5311	4.4258	
3.7876	4.112	3.9037	4.2257	4.5395	3.9679	4.5584	4.2288	
4.4097	4.6525	4.599	3.9701	4.9311	3.9993	3.7245	3.2914	
4.7619	4.3017	4.9166	4.7181	4.3357	4.4142	5.0314	4.0207	
4.1509	3.7641	5.1786	4.7162	4.2754	4.2397	4.207	4.1461	
4.2157	4.3865	3.6909	4.4183	3.4448	4.6545	4.5123	3.1374	
4.4107	4.1416	5.1897	3.859	4.9138	4.6275	4.0577	4.5393	
4.4193	4.3096	4.2448	4.1448	2.9206	4.4144	3.3609	4.8903	
4.5017	4.7516	4.1853	4.4591	4.2711	4.2814	4.5339	5.5335	
4.9927	5.241							
CCDC54	0.0645	0	0	0	0	0	0	
0.0778	0.1429	0	0.1197	0	0.2509	0.1347	0.0351	
0.1663	0	0	0	0.0579	0.2619	0	4.00E-04	0
	0.0046	0.074	0	0.1274	0	0.0228	0	0
	0.1248	0.066	0.0852	0.0108	0.0042	0.0947	0.0403	
0.0154	0.0577	0	0	0	0	0.1791	0.0387	
0.0785	0	0	0	0.0261	0.0632	0	0.5877	0
	0	0	0	0	0	0	0.0615	0
	0	0	0.358	0.0326	0	0	0	0
	0	0.0113	0.0506	0.0421	0.1106	0	0	0
	0	0	0.0274	0	0	0.1778	0.0919	0
	0.2243							
CCDC57	2.4021	1.9767	2.0023	0.9302	2.1752	1.388	1.6764	
2.6678	1.9525	2.141	1.9441	2.3744	2.472	1.4906	2.1585	
2.229	2.6566	1.754	1.8974	2.6825	1.6213	1.9189	2.0496	
2.2245	2.9755	1.9904	1.9564	2.663	2.0139	1.725	2.0994	
2.3178	1.2324	1.515	2.5115	1.6414	3.1631	2.2182	2.0281	
1.6693	2.3167	1.949	1.4574	1.7629	2.4004	1.654	1.4436	
1.9159	2.4163	2.0766	2.612	1.5131	1.775	2.6195	2.0182	
1.7577	1.5694	0.7012	2.4647	0.9025	1.7751	1.8518	2.0303	
2.1069	2.1578	2.5883	2.3273	1.525	2.4724	2.4931	1.9606	
2.7518	1.4681	2.3484	1.9811	2.2473	2.0886	2.0498	2.5718	
1.7551	2.1256	2.1489	1.9704	2.9978	1.4081	2.1179	0.9873	
2.0581	1.7099							
CCDC58	4.7865	6.1525	5.3842	5.4556	5.4831	4.3667	4.7082	
5.0822	5.3287	5.0643	5.0994	5.0201	4.9729	5.5641	5.086	
4.168	5.7148	5.0212	6.0437	5.0185	5.3046	4.9452	4.4671	

5.3415	4.4961	4.2241	4.8892	4.8786	4.9285	5.3627	5.3911	
5.1236	5.5366	4.412	3.6594	5.6562	5.4199	4.3026	4.9468	
5.641	4.0795	4.231	4.9277	4.634	4.9593	5.3139	6.0701	
5.1966	3.9065	5.9336	5.4056	5.3267	5.056	5.6271	5.7454	
5.7064	4.6636	5.2306	5.0968	5.6037	5.5089	5.5159	4.857	
5.4451	5.0246	4.1315	4.929	4.4321	5.4579	4.3294	4.4401	
5.7549	5.0962	5.3122	4.238	4.5676	5.8647	4.6517	5.0384	
6.1223	5.5854	4.4922	4.5547	4.941	4.9307	5.1926	4.1792	
4.6912	4.3134							
CCDC59	4.7468	4.0518	3.3268	4.091	4.2644	3.3888	3.9981	
4.3357	3.5583	4.0277	3.9692	3.6025	3.8804	3.9345	3.4053	
3.5056	4.7955	3.8486	4.5328	4.1767	4.2479	4.242	2.978	
3.529	4.2763	3.5454	4.62	3.4491	3.2586	3.2113	3.6114	
3.7422	3.6275	3.2375	3.4174	4.0483	4.8598	3.7084	4.2869	
4.2282	3.1651	3.3626	3.5369	3.1025	3.7353	4.3115	4.4082	
4.1272	3.5523	4.918	4.2074	3.7059	4.5478	4.9709	4.2668	
3.8471	4.552	4.3654	4.4175	3.8727	4.2023	4.4985	3.3363	
4.09	4.5148	3.4477	3.8577	3.5327	3.708	4.0416	3.6417	
3.7871	4.2776	3.6481	3.2722	4.0294	4.3695	2.9956	3.0393	
4.2561	4.0136	3.5249	4.3075	3.6999	3.4605	4.754	3.6188	
3.6896	3.3219							
CCDC6	3.5436	3.8747	3.9691	4.0536	3.5468	2.9711	3.1915	
2.6115	3.2075	3.9901	3.3212	3.4598	4.0852	4.094	2.9277	
3.1155	4.4974	3.6039	3.6082	3.697	4.1002	2.9319	2.9749	
3.595	3.3116	3.6298	4.0703	3.3843	2.656	2.8851	3.3516	
3.6115	3.1603	3.2105	2.6836	4.1871	3.8581	2.7574	2.7122	
3.4573	3.4976	3.9971	3.4592	3.4365	3.6381	3.3913	3.7842	
3.2219	2.5387	4.787	3.257	3.1723	3.3006	4.2058	4.5049	
3.7103	4.2053	3.4084	3.7511	3.7326	3.3399	3.3707	3.481	
3.9216	4.2543	3.9431	3.2372	3.9939	2.9321	3.1875	3.7431	
3.454	2.7456	2.6995	3.2979	3.2958	3.8619	3.3784	2.954	
3.4661	3.726	3.6878	3.2184	2.551	2.9096	2.6964	3.7125	
3.5005	3.4451							
CCDC60	0	0	0	0	0	0	0	0
	0	0	0	0	0	0.0293	0.014	0
	0.0525	0	0.0686	0	0	0	0	0
	0	0	0	0	0	0	0	0
	0	0	0	0	0	0	0	0
	0	0	0	0	0	0	0	0
	0	0	0	0	0	0	0	0
	0	0	0	0	0	0	0	0
	0	0.0166	0	0	0	0	0	0
	0	0	0	0	0	0	0	0
	0	0.0164	0	0	0	0	0.0208	
0.024	0							
CCDC61	2.3599	2.3028	2.1048	0.9544	1.0633	0.898	0.9765	
2.1586	2.0577	2.0936	1.2855	1.9355	1.9994	1.9955	1.9164	
1.1379	3.0089	1.5492	1.8636	2.1169	2.6671	2.4005	1.8396	
1.3917	1.5103	1.335	1.7867	1.6103	2.0846	1.426	2.349	
2.0633	2.5503	2.998	1.5928	1.3797	2.5707	2.6513	2.1703	

0.7572	1.7974	0.9117	2.0246	1.5039	1.3644	1.1802	1.2583	
1.0041	2.5958	2.1672	1.4475	1.1287	1.8117	2.6712	1.4689	
2.6511	1.9333	1.5786	2.2365	1.042	1.9814	2.1728	2.726	
1.8179	1.7169	1.1932	1.5668	0.9929	1.2146	1.3094	1.917	
2.4209	1.5116	2.2688	1.1851	2.1459	1.9023	1.2794	2.3112	
3.004	1.5693	1.3046	1.8609	1.3529	2.6037	2.7548	2.5817	
2.4468	2.5958							
CCDC62	0.1731	0.169	0.3514	0.0855	0.0968	0.2721	0.4545	
0.0751	0.1006	0.1198	0.1558	0.1258	0.0231	0.2013	0.0296	
0.1022	0.168	0.2052	0.1916	0.1013	0.2529	0.0143	0.0808	
0.2698	0.0466	0.0794	0.0955	0.3972	0.022	0.1278	0.133	
0.0122	0.3127	0.2926	0.2976	0.1248	0.2159	0.1365	0.0492	
0.3086	0.1455	0.0566	0.1013	0.247	0.0836	0.0828	0.1929	
0.4399	0.3032	0.795	0.0998	0.0244	0.1933	0.1427	0.1362	
0.427	0.1599	0.1756	0.067	0.1528	0.0626	0.2878	0.0134	
0.2816	0.2203	0.0597	0.5318	0.3118	0.2705	0.032	0.0712	
0.0278	0.1035	0.0341	0.0987	0.412	0.4697	0.056	0.0108	
0.3014	0.3135	0.0581	0.3909	0.0237	0.0421	0.1175	0.0863	
0.1244	0.1723							
CCDC63	0	0.024	0	0	0	0	0.0128	0
	0	0	0.0087	0	0	0	0	
0.0118	0	0	0	0	0	0.0056	0.0291	0
	0	0	0.026	0	0	0	0.3894	
0.0223	0	0.0436	0.0031	0.0282	0	0	0	
0.0167	0.0171	0	0	0	0	0	0	0
	0	0	0	0	0	0.0301	0.2633	0
	0	0	0.0299	0	0	0	0	0
	0	0	0	0	0	0	0	0
	0	0	0	0	0.0356	0	0	0
	0.0541	0	0	0	0	0	0	0
	0							
CCDC65	0.1269	0.5353	0.0986	0.2177	0.1383	0.2055	0.0875	
0.1034	0.0774	0.0358	0.4754	0.1197	0.0843	0.2551	0.3073	
0.0444	0.4397	0.2405	0.2272	0.3187	0.1609	0.0883	0.0489	
0.1357	0.141	0.443	0.3477	0.4762	0.083	0.4844	0.1338	
0.1053	0.11	0.2236	0.0704	0.0548	0.3555	0.2631	0.0261	
0.1421	0.3595	0.1933	0.0769	0.1968	0.088	0.2016	0.0758	
0.0674	0.1756	0.9369	0.2101	0.0914	0.3428	0.3429	0.1298	
0.2091	0.219	0.1247	0.4669	0.4234	0.1332	0.0827	0.2433	
0.2312	0.5216	0.3748	0.2623	0.2543	0.1686	0.0611	0.2617	
0.0975	0.1247	0.326	0.0719	0.0342	0.1118	0.0548	0.059	
0.0497	0.0523	0.3041	0.1218	0.0866	0.2914	0.5907	0.291	
0.301	0.2596							
CCDC66	2.0267	2.1621	1.9558	1.9168	1.8328	1.8528	1.9994	
2.1151	1.7113	2.5261	1.7113	2.0513	2.4282	2.3372	2.4845	
2.1483	2.2426	2.5955	1.9414	2.2315	2.2576	1.7936	1.4602	
2.1951	1.6817	2.0277	2.3634	2.0573	1.9528	1.7995	1.5856	
2.2436	1.8911	1.2334	1.8694	2.5197	2.3094	2.3122	2.0941	
1.9692	1.828	1.4412	1.7091	1.8141	2.2342	1.7163	2.6672	
2.0148	2.0628	2.8379	1.6249	2.4073	2.1033	2.6327	1.7846	

1.8802	2.3164	1.6893	2.1348	1.8502	2.303	2.094	2.3226	
2.5063	2.0751	2.1812	2.017	1.824	2.4416	1.8073	1.544	
1.8838	2.0584	1.5487	2.1429	1.4506	2.0844	2.2033	1.4699	
1.7056	1.8683	1.8769	1.736	1.8387	1.6745	2.1392	1.7308	
2.1083	1.9094							
CCDC68	0	0	0	0	0.0676	0	0.1746	
0.0585	0.0602	0.0633	0.0534	0.9218	0.0956	0	0.4652	
0.0551	0.1385	0	0	0.0871	0	0.0823	0	
0.0808	0.6297	0.2549	0	0	0.0659	0	0.0582	
0.1002	1.1477	0.0746	0.0511	0.0749	0	0	0	
0.0692	0.0761	2.2816	0.1583	0	0	0.459	0	
0.0741	0.115	0	0.0632	0.2437	0.1116	0	0.5787	
0.0338	0.7676	2.4601	0	2.3036	0	0	0	0
	0	1.9129	0	0	0	0	0	0
	0	0.0707	0	0	0.0096	0	0	
0.0037	0	0	0	0.0026	0.025	0	0.0792	
0.1087	0.1421							
CCDC69	0.7955	1.1786	2.5633	0.8794	1.8038	0.1447	0.3344	
0.1891	1.5054	1.6008	0.1939	1.9642	0.7517	0.3563	0.3511	
0.622	1.0367	1.061	0.8379	0.7481	1.281	0.0359	0.548	
0.242	0.2397	0.3155	0.3963	2.1568	0.539	1.2476	1.826	
0.0965	0.175	0.1527	0.0529	1.1399	0.3829	1.2991	0.2705	
0.1174	0.4688	3.2626	0.8096	0.4239	0.7099	0.2986	1.3979	
0.8927	0.1119	0.4444	0.5981	0.3297	0.279	0.5206	1.1652	
0.6325	3.3866	1.3054	0.4872	0.3657	0.2905	0.2703	0.3153	
0.4287	0.2689	1.5285	0.1003	2.335	1.54	0.2416	0.6866	
0.3031	0.7026	0.3144	0.5385	2.2132	1.0437	0.2231	0.5013	
0.4164	1.3602	0.5518	1.5196	0.3774	0.1354	0.3062	0.569	
0.6932	0.841							
CCDC7	1.0907	0.4332	1.1723	1.0008	0.3311	0.7477	1.1533	
0.2966	0.9436	1.3336	0.5553	0.6654	1.1705	0.8494	0.5028	
0.6546	1.064	1.2579	1.1597	1.2116	0.7295	0.5522	0.1331	
0.9799	0.5888	0.5913	1.3087	0.4882	0.3955	0.2309	0.7176	
0.9407	1.1095	0.9375	0.6208	1.3555	0.5978	0.2666	0.6047	
1.0292	0.3472	0.4373	0.5563	0.8418	0.4475	0.6026	1.0735	
0.9943	0.7458	1.4414	1.0826	0.6788	0.8734	0.8181	1.212	
1.3426	0.8255	0.2421	0.7495	0.19	0.3633	0.8661	0.3587	
0.9949	0.6272	1.1024	0.6887	0.9372	0.5501	0.8352	0.4182	
0.9383	0.299	0.5037	0.4409	1.2004	1.1656	0.4785	0.6136	
1.1343	0.6986	0.9086	1.4737	0.248	0.4538	0.6677	0.8249	
0.674	0.6343							
CCDC70	0	0	0	0.1871	0	0	0	0
	0	0	0	0	0	0.3896	0.2086	0
	0	0	0	0	0	0	0	
0.0722	0	0.1205	0	0	0	0	0	
0.0563	1.2133	0	0.0127	0	0	0	0	0
	0	0	0	0	0	0	0	0
	0	0	0	0	0	0	0	0
	0.1196	0	0	0	0	0.1872	0	0
	0	0	0	0	0	0	0.0782	

0.0409	0	0.0633	0.1333	0.0948	0.0655	0	1.2038	0
	0	0	0	0	0	0	0	0
	0.5752							
CCDC71	3.1977	3.9453	2.9565	2.9096	3.8365	2.1147	2.219	
3.2341	2.4897	3.4965	3.5744	3.3219	3.2807	2.2745	2.1127	
1.9428	3.5661	2.783	2.451	3.1951	2.905	3.3064	2.1397	
3.1037	2.738	2.579	1.1478	2.7667	3.1969	3.0936	3.1499	
3.7649	2.8197	2.8098	2.467	2.7573	3.5567	3.8233	3.9833	
2.0974	3.4393	3.6579	2.9951	3.0975	3.6294	2.3498	2.783	
3.0368	3.1108	2.5234	2.9278	2.6769	2.5287	3.4508	3.1415	
1.9975	3.2277	2.5345	2.946	2.6231	3.4015	3.4444	3.0065	
2.8334	3.2648	3.1338	3.8567	3.0708	2.0624	3.2369	2.6908	
2.5724	3.2815	3.3834	2.7091	2.1262	3.0448	3.473	2.063	
3.0263	2.9535	3.3471	1.9832	3.0326	4.292	2.9169	3.1373	
3.4263	3.3919							
CCDC71L	2.6851	2.5507	2.8819	3.392	3.0642	3.9478	4.8881	
3.0472	2.6028	3.8266	3.1301	3.3041	5.2886	3.3486	3.5945	
3.9588	3.265	4.5604	3.2534	2.9278	2.0362	4.0337	3.6632	
2.7019	4.5041	4.9897	2.8375	2.2986	3.5539	2.3053	2.3489	
4.0564	2.4082	2.4467	3.7158	2.8415	3.6464	2.7276	4.6348	
2.7878	4.2135	3.9401	3.4597	2.6813	3.1294	3.3854	2.5983	
2.6829	5.4112	2.3305	3.3618	4.3496	3.0219	4.0951	2.5176	
3.5317	2.2859	2.597	2.8086	2.6602	3.73	3.5429	3.3146	
3.2145	2.0962	5.6058	3.0801	4.8967	2.1662	3.8002	4.3185	
3.1435	3.936	2.8492	2.5794	2.5845	3.2962	3.3448	2.6541	
3.0387	2.337	2.822	2.3596	4.0067	4.7556	2.804	3.1826	
2.6817	2.2055							
CCDC73	1.405	2.1448	1.5971	1.5693	1.7255	1.3415	1.0793	
1.2679	0.9265	0.8787	1.6125	1.0548	1.2517	1.615	1.686	
1.1705	2.1066	0.7072	1.8694	1.5801	1.5328	1.4455	0.9623	
1.7921	1.0132	1.0649	2.6189	1.2484	1.5245	1.0944	0.6716	
1.1979	1.4885	0.7346	0.6639	1.1592	1.7079	1.2593	1.1191	
1.4277	1.1446	0.7999	0.9754	0.89	0.9773	1.3176	1.4993	
1.1237	0.9232	2.305	1.382	1.5087	1.363	2.6226	1.258	
1.9377	1.6498	0.9177	2.0444	1.1769	1.5974	2.0303	1.5822	
1.4854	1.2466	0.6256	1.3242	1.3247	1.2208	1.012	1.1425	
1.1357	1.2913	2.0057	1.255	1.8537	1.4842	0.8117	1.1805	
1.4077	1.5656	1.4536	1.2249	0.4882	0.7311	1.8352	0.8657	
1.026	0.867							
CCDC74A	1.7191	0	1.3443	1.0477	1.3477	1.9367	0	
3.1432	0	1.2821	1.0757	0.8882	1.3646	0.1275	2.5763	
0.3625	2.3021	1.6247	0.0197	1.0151	0.2253	2.8285	2.1222	
0.0274	3.1763	2.5982	2.7961	0.2663	2.8218	1.4508	4.1812	
0.7508	0.3813	0.8381	3.3385	1.6096	1.9993	1.8865	0.5407	
0.3118	1.362	1.5097	2.2648	0.5763	1.0933	2.15	1.0974	
1.851	1.3764	2.4121	2.6075	1.4043	0.4012	0.8181	3.0154	
1.7537	1.0049	0.9735	0.562	0.8851	0.5368	0.3646	2.0138	
0.9433	4.3461	3.0943	3.9337	3.0063	0.5341	2.6344	0.9958	
1.0143	0.594	1.7236	1.595	1.8437	0.7075	2.1628	0.3154	
0.2526	0.2229	0.5289	1.6816	2.384	2.9061	1.3334	2.7154	

2.4312	2.6495							
CCDC74B	1.2911	0.0375	0.197	0.6728	2.14	0.7735	0.2586	
1.7801	0.0581	0.285	0.3163	0.3402	0.2395	0.2671	1.7147	
0.639	1.0674	1.078	0.2292	0.0686	0.0434	1.8908	0.9731	
0.0819	2.3827	1.3358	2.9385	0.5354	1.6403	1.6466	3.4869	
0.8621	0.4639	0.6341	1.2601	0.8982	0.8183	0.5318	0.9593	
0.2215	0.5579	1.6148	2.5891	0.1655	1.0466	1.1469	0.4172	
0.5232	0.4356	0.9277	1.2115	0.9182	0.2856	0.3859	3.077	
1.0025	1.0385	0.5135	0.4704	1.0148	0.2373	0.2913	1.5188	
0.5634	2.315	2.3642	1.465	2.0737	0.4027	2.2171	0.6006	
0.5996	0.6845	0.7524	0.9245	1.0763	0.3317	0.594	0.1758	
0.2014	0	0.2739	0.5503	1.5428	1.5045	0.9631	1.7278	
1.3614	1.6864							
CCDC77	2.1552	2.9032	2.5274	3.282	3.647	2.3008	2.4908	
3.1757	2.775	2.3919	2.8465	3.3784	3.5464	3.6998	3.4211	
2.0105	3.0735	3.3061	3.0125	3.0429	2.3842	3.4965	3.2769	
2.8955	3.4187	2.1704	3.1146	2.7111	2.9403	2.8433	3.324	
3.2898	3.3728	2.7701	3.7794	3.3459	3.9017	1.6224	2.8134	
3.502	2.0873	2.6589	4.207	3.1765	2.7223	4.201	3.2572	
3.1452	2.1805	4.2821	3.5043	2.8991	3.718	1.7095	2.8277	
2.6823	3.0755	2.7723	2.9297	2.8574	3.5056	3.9921	3.3977	
3.4679	3.1687	2.3586	2.1603	2.3781	2.8895	3.0918	2.4823	
3.3997	3.0637	3.7666	2.6599	3.6799	3.324	2.3607	2.6659	
3.1853	3.3586	1.5368	2.7502	2.4342	3.6276	4.125	2.1135	
3.027	2.6932							
CCDC78	2.8289	1.8931	1.4672	1.0282	1.5314	1.5132	0.4892	
1.9903	1.2754	1.3641	2.7042	0.7262	1.1382	1.401	0.9245	
1.4015	1.646	1.2305	1.8187	2.028	2.245	1.566	1.8972	
2.002	1.9782	1.0428	1.2853	2.0499	1.3328	1.4015	1.0488	
2.3926	1.1094	1.4041	1.2796	1.0329	1.7601	1.5196	1.7288	
2.1042	3.1172	1.4231	1.3377	2.2844	0.5731	1.021	1.1559	
1.9848	1.3696	0.606	1.616	0.7474	0.7857	1.899	0.6747	
1.8132	1.4422	1.0717	2.0734	1.1125	2.2184	2.0114	3.2252	
1.6264	1.5777	1.2444	1.5819	1.4123	1.3569	1.4094	2.4947	
2.1553	1.7121	1.4263	1.3407	2.2104	1.0538	1.231	2.2484	
1.9108	2.0772	0.7973	1.0481	1.8681	0.3033	1.4105	0.7387	
1.5236	1.326							
CCDC8	2.7307	0.082	0	0.0374	0.0241	0	0.0048	
0.0547	0.0071	0.1088	0	1.895	0	0.9422	0.031	
0.031	0.032	0.2081	0	0.0308	0	0.2221	0	0
	0.0466	0.9544	0.0178	0	0.0263	2.7763	4.2984	
0.0064	0	0.1266	0.3873	0.9705	0.8238	3.4268	0.3857	
0.0124	0.0069	0.0127	0	0.053	0.0334	0.0133	0	
0.0171	0.0647	0.1034	0.0106	1.0344	0.0418	2.4783	3.0862	
0.1622	0.0667	0	0.0643	0.0591	0.0211	0.1795	0.079	0
	0.1435	0.0167	3.0009	0.0294	0.0292	0.0737	2.9122	0
	0.1936	0.0255	0.0598	0.042	0.0145	0.0254	1.0788	
0.04	0.0373	2.3493	0	0.0861	0.4741	0.0548	0.4723	
1.6873	1.4108							
CCDC80	0.3337	0.2628	0.2159	1.4155	0.6236	1.8361	2.2542	

0.3542	0.79	0.8436	0.4513	1.7602	3.1436	0.7033	1.6303	
0.137	5.5941	0.5783	0.584	0.3856	0.4659	1.764	3.5568	
0.6206	1.8809	4.825	2.3709	0.4831	2.61	0.1279	0.6675	
0.2748	1.6949	2.1462	3.6585	0.2203	1.5333	1.2119	0.8791	
2.5053	1.7283	3.4087	1.9328	0.069	1.4187	3.8267	1.1655	
1.0423	4.8726	7.6176	2.584	1.4986	0.8779	0.5065	1.8405	
1.0137	6.3842	0.7029	0.4158	0.7145	1.5893	1.4157	0.4117	
1.5627	0.4843	7.9073	1.0006	1.0669	0.4906	1.1589	3.031	
2.0738	0.361	2.6338	0.8322	0.6396	1.9929	3.3108	1.2932	
1.2733	0.3051	3.8767	0.6227	1.8394	3.5802	0.7924	3.5263	
2.7005	3.2537							
CCDC81	0.3165	0.0391	0	0.0203	0.0782	0.1263	0.0453	
0.1273	0	0	0	0	0.1746	0.2427	0.0279	
0.6496	0.4755	0.33	0.1772	0.1246	0.2455	0.1525	0.0266	
0.1891	0.0942	1.0904	0.682	0	0.2544	0.0998	0.0323	0
	0.0591	0.0555	0.0572	0.0071	0	0.0241	0.0036	
0.0992	0.1097	0.0502	0.1432	0	0.201	0.1861	0	
0.0695	0.2137	0.8389	0.1184	0.3959	0.0654	0.1136	0.0874	
0.0703	0.2058	0.0464	0.544	0	0	1.6776	0.1685	
0.0507	0.0682	0.1845	0	0.0307	0.053	0.0645	0.0764	
0.0405	0.5497	0.2688	0.0394	0.0477	0.0795	0.0949	0.0475	
0.2087	0	0.1505	0.1245	0.0885	0.1977	0.116	0.0668	
0.0041	0.088							
CCDC82	3.131	3.2641	3.5318	3.7367	3.2527	2.6034	4.7384	
2.696	4.0159	2.5381	2.4503	3.6262	4.0828	3.9969	3.2233	
3.5233	3.8184	3.0976	4.4964	3.5168	2.5526	3.098	3.2321	
3.2356	4.0634	3.2938	3.397	3.1424	3.6227	3.3851	3.4403	
2.3848	4.0073	3.5027	3.7455	3.4014	3.7049	2.9096	2.4719	
2.9136	3.1402	3.1699	3.8193	3.7115	3.5607	4.3419	4.2276	
4.0846	3.5336	3.8363	3.7424	3.4026	3.8516	3.9196	3.469	
3.6177	3.6571	2.5388	3.6567	2.7236	2.8087	3.1843	3.1103	
3.8538	3.3464	3.9532	3.6558	3.8301	3.5084	3.2791	4.5303	
3.6193	3.632	3.2357	3.6289	3.9839	3.1124	3.0234	3.6163	
3.3719	3.2283	3.1913	6.0651	2.7551	3.7307	4.0164	3.7059	
3.8568	3.9182							
CCDC83	0	0	0	0	0	0	0	0
	0	0	0	0	0	0.0587	0	
0.0218	0	0	0	0	0	0.009	0	0
	0	0	0	0	0	0	0.0271	
0.0054	0	0	0	0	0	0	0	0
	0	0.0247	0	0	0	0	0	
0.0473	0	0	0	0	0	0	0	0
	0	0	0	0	0	0	0	0
	0	0	0	0	0	0	0	0
	0.0138	0	0	0	0	0	0	
0.1474	0	0	0	0	0	0.028	0	0
	0							
CCDC84	1.9414	1.9829	1.532	2.0649	2.3354	1.2094	1.7144	
1.1706	1.9909	1.4773	1.1451	0.8627	2.2398	1.781	1.5424	
1.7821	2.5207	1.6159	1.7672	1.1129	1.9077	1.0883	1.7018	

1.7446	1.2536	1.4107	1.9741	1.3245	1.7294	1.6986	2.4502	
1.0667	2.0412	1.318	1.6766	1.3399	1.3263	1.1489	1.1476	
1.7034	1.345	1.6713	0.9691	2.1522	2.054	1.4985	1.2853	
2.1973	1.5532	2.2433	2.2239	1.6203	1.0743	1.8354	2.1817	
2.3832	1.5686	1.5766	1.6607	1.6042	1.7523	1.7438	1.515	
1.7358	0.7568	2.017	1.7398	1.7019	1.6316	1.8067	1.0226	
1.6163	2.0565	1.0859	2.0039	2.185	1.1972	0.7647	1.9611	
1.725	1.6896	1.819	1.5231	1.4737	2.0128	1.2103	1.3499	
1.5368	1.476							
CCDC85A	0	0.0462	0	0.0289	0.0648	0.0416	0.0492	
0.057	0.0146	0	0.0397	0.0692	1.6319	0.0431	0.0462	
0.0238	1.2856	0.0566	0.0634	0.9208	0.0962	0.0615	0.0088	0
	0.0995	0.0125	0.0397	0.0948	0.0675	0.0242	0	
0.0035	0.0903	0	1.8766	0	0.0287	0.1377	0.0148	0
	0.3333	0.1474	0.0796	0	0.1185	0.5238	0.0828	
0.0347	0.2448	0	0	0.0259	0.0877	0.0311	0.0219	0
	0.0252	0.0231	0.016	0.2788	0.1692	0.0304	0.0402	0
	0.5005	2.5752	1.2089	0.017	0	0.0277	0.1249	
0.1709	0.0212	9.00E-04	0.1606	0	0.0858	0.0472	0.0225	
0.0352	0.0524	0.0182	0.4556	0.0211	0.0398	0	0.5471	
0.351	0.4648							
CCDC85B	4.0887	3.5442	4.1521	2.3056	5.2376	4.447	2.7552	
4.4733	2.2559	2.1601	3.1384	3.1477	4.3728	2.9059	4.9468	
3.2362	5.1696	1.8308	2.0087	4.9357	4.629	4.5925	4.6363	
4.4456	4.5089	3.4547	3.5335	2.6438	4.4847	1.9969	2.9979	
3.6909	4.0616	3.4624	3.8896	1.9309	4.2746	4.5616	4.4161	
2.0709	4.6568	4.652	4.1347	3.4734	2.7917	3.752	2.5567	
3.2098	5.7197	3.0743	3.419	2.519	2.0573	4.2283	4.421	
4.5056	5.0865	2.932	3.6861	2.5468	4.8546	3.7576	4.4439	
2.9124	4.2368	5.6661	4.4075	5.8113	1.8666	5.5193	5.468	
4.4266	3.7414	3.853	4.3174	3.2544	4.1574	3.3434	3.8325	
4.6413	2.8307	5.0971	5.002	4.4354	5.2573	4.4295	3.4213	
4.2541	3.4563							
CCDC85C	2.5474	1.6373	2.3813	2.2358	2.1257	2.0296	2.0933	
2.817	2.2795	2.5889	3.2651	2.6213	2.1688	2.7775	2.2127	
3.2713	2.4586	3.0497	2.3393	2.0426	2.5831	2.5218	2.7836	
1.7336	2.3925	2.7827	1.7782	2.5621	2.2471	2.5723	2.9013	
2.6783	2.948	2.7434	1.1042	2.5938	2.4571	3.3727	2.6712	
2.1492	2.6599	2.4606	2.5847	1.9076	3.0648	2.8137	2.3595	
3.3222	2.0217	2.8854	2.7306	2.0055	3.3839	1.7891	2.6681	
2.5549	3.5302	2.6	2.2408	2.7492	2.2851	2.0188	2.758	
2.4196	3.6851	2.9879	2.8548	2.3165	1.5932	2.5168	1.9876	
2.9612	2.0816	2.5754	2.6967	1.9678	2.3328	1.9301	2.8632	
3.0094	2.7901	2.111	1.498	2.8328	2.2968	3.2639	2.6312	
2.1096	2.4159							
CCDC86	4.6401	5.2045	5.2609	4.048	4.9986	4.6786	3.9837	
5.0508	3.8437	3.4977	4.5431	3.489	5.1545	3.6719	4.4928	
4.527	4.9472	2.5221	3.8198	5.6375	5.3051	5.2859	4.0374	
5.149	5.0345	4.7861	3.7244	4.0939	4.8361	3.7234	4.1057	
3.9583	4.871	3.5743	4.5218	3.4544	5.1485	4.8916	4.7608	

4.4286	4.289	3.4871	3.7527	3.8388	3.4595	3.5979	4.311	
3.9812	4.213	4.0779	3.5273	3.6815	3.6698	6.2137	5.3779	
5.1146	4.5254	4.7388	4.4589	4.5884	4.4498	4.2297	4.7669	
4.3142	4.4211	3.2027	4.0648	4.0788	4.0384	4.2696	5.3441	
4.0695	4.8156	3.4161	3.827	4.6608	5.1218	3.4455	4.5755	
4.553	3.4083	3.6891	5.0193	4.0197	4.516	4.1596	3.6043	
4.4251	3.7668							
CCDC87	0.1405	0.0289	0.5415	0.2729	0.2022	0.1255	0.0944	
0.1387	0.1569	0.04	0.083	0.0587	0.1956	0.08	0.1296	
0.0301	0.4167	0.0638	0.1845	0.2389	0.1204	0.0221	0.107	
0.1908	0.1819	0.1162	0.0297	0.4867	0.0529	0.0456	0.6089	
0.0822	0.0484	0.0441	0.0768	0.1362	0.1285	0.3539	0.0718	
0.1636	0.2605	0.1187	0.2231	0.0268	0.0592	0.2597	0.0384	
0.3244	0.1339	0.2095	0.0864	0.2062	0.084	0.1189	0.5497	
0.1935	0.0509	0.1418	0.1567	0.0976	0.0549	0.1798	0.1544	
0.0887	0.3024	0.4774	0.3737	0.223	0.146	0.1349	0.3169	
0.0779	0.2451	0.0507	0.0808	0.1115	0.2327	0.0199	0.1815	
0.0508	0.0739	0.0546	0.0724	0.1222	0.1489	0.0359	0.151	
0.138	0.0899							
CCDC88A	3.6208	3.6653	3.4939	4.0977	3.2807	4.1429	3.8855	
3.7319	5.747	4.457	4.0159	5.0157	3.7771	4.8543	3.6927	
4.4938	4.2267	4.4087	4.4863	2.8476	3.3711	2.6843	3.6124	
4.1777	3.7603	3.5294	3.2506	4.0925	3.9492	4.5261	4.3667	
3.5793	4.4699	4.3601	3.3699	5.0536	3.3846	3.8642	3.2604	
4.4729	3.5505	3.7853	4.2894	3.8824	3.6053	4.426	4.6442	
3.9613	4.3583	3.7738	3.7262	3.5721	4.4915	4.0731	4.3457	
3.9945	3.9066	3.8433	4.1723	3.931	3.6124	4.2258	3.8781	
3.4485	3.9589	3.0116	5.0147	2.84	4.1533	3.4438	4.3978	
4.382	5.0026	4.3014	4.388	3.3364	4.0533	4.1133	3.9717	
2.708	4.7805	3.2816	3.3581	2.7968	3.4043	4.2656	2.9173	
3.1141	3.0645							
CCDC88B	1.0269	2.0203	1.2226	0.2495	1.6616	1.4786	0.4168	
1.7255	0.7913	0.5075	1.0523	0.3897	0.9512	0.5985	0.6174	
1.0452	1.4648	0.4818	0.7897	1.5049	1.5921	1.1055	0.9531	
1.1886	1.1919	0.2563	0.3287	1.1626	0.4152	0.9976	2.7152	
0.2565	0.3127	1.1993	0.7249	0.3034	1.3482	2.1102	0.7874	
0.3062	1.4953	0.7856	0.7237	1.3959	0.679	0.1876	0.6349	
1.1999	0.1496	0.8794	0.9606	0.3842	0.5234	1.5921	1.6765	
2.0367	0.6987	0.7638	0.7705	0.6691	0.1703	0.0542	1.7434	
0.4117	1.9598	1.4661	1.6936	2.0313	0.5429	0.732	1.7782	0
	1.0485	0.2566	1.0025	1.4731	1.5351	1.2363	1.4345	
1.4259	0.6353	0.719	1.3341	1.2293	0.3322	1.3207	1.6247	
1.6278	1.1186							
CCDC88C	2.2372	0.6085	1.3636	0.4892	0.3838	0.0727	0.2904	
0.4332	0.1332	0.9146	1.3913	0.9762	0.1091	0.6787	0.8387	
0.7013	0.1273	2.3118	0.983	0.2149	1.1027	0.1281	0.1234	
0.1933	0.11	0.1393	0.2965	0.8711	0.1529	0.3403	1.4842	
0.8631	0.242	0.1009	0.0327	1.6325	1.4054	2.2026	0.3631	
0.4277	0	2.3478	0.2562	0.2518	1.9501	2.6227	0.737	0
	0.0464	0.1036	0.1184	0.1237	1.2278	1.198	1.5168	

1.3585	1.967	1.8616	1.858	1.7732	0.1963	0.1914	0.5689	
0.1003	2.1853	0.2683	2.1965	1.7862	0.3214	0.0644	0.5538	
0.0274	0.4343	0	0	0	1.8106	0.2025	0.7878	
0.2615	0.5169	0.4234	0.0449	0.1751	0.206	0.6767	0.142	
0.0632	0.0641							
CCDC89	0.833	0.0111	0.0919	0.0225	0.0149	0.0482	0.8158	0
	0.4125	0.04	0	0.0158	0	0.0228	0.6832	
0.0319	0	0	0.103	0	0	0.1936	0	0
	0.6144	0.1668	0	0.0902	0.7933	0.0249	0	
0.0091	0.0966	0	0	0.0809	0	0.0857	0	0
	1.0097	0.1281	0	0.1122	0.0501	0.0054	0	0
	0	0	0.7455	0.0438	0.4287	0.0315	0	0
	0.3304	0	0.043	0	0.0571	0	1.2151	
0.0861	0	0	0	0.3393	0.1275	0.0711	0.2042	0
	0.0688	0.0237	0.1448	0	0	0.1478	0.03	
0.0611	0.0612	0.4776	0.0365	0.0439	0.1168	0.5394	0.6741	
0.644	0.5112							
CCDC9	2.3365	2.8447	2.694	1.5944	2.2636	2.2378	2.7414	
3.1494	2.4681	2.2568	1.7544	1.8039	3.3137	2.0431	2.6426	
2.3031	3.1482	1.7375	2.2123	2.7761	2.6804	2.6297	2.1972	
2.2594	2.5375	2.76	2.873	2.2887	2.4801	2.2844	3.7398	
2.1711	2.9862	3.2774	2.3234	2.0361	3.453	3.3778	2.8516	
1.5968	2.3895	1.9309	2.9857	2.1304	2.581	2.0482	1.7684	
1.8868	2.6105	2.1333	2.2967	1.8247	2.1074	2.7382	3.7573	
2.4887	2.423	2.7967	2.5441	2.6478	2.4695	2.2786	2.6793	
2.2535	2.6918	3.1147	1.9497	2.5428	2.2617	2.5837	2.7016	
3.0019	2.8251	2.4157	2.0584	2.5858	2.5031	2.0179	2.4695	
2.9131	2.2157	1.7422	3.5085	1.9568	3.612	2.6382	2.1654	
2.2626	2.3303							
CCDC90B	3.1997	2.9716	3.6484	3.4887	3.712	2.7206	4.1007	
2.7546	3.2158	3.2587	3.3135	2.9299	3.2366	3.2091	3.1329	
2.9093	4.4044	2.7855	3.2082	3.0144	2.3708	3.3381	3.6995	
2.5261	3.9484	4.0885	2.9294	2.6993	3.5981	3.1942	3.6148	
2.7266	3.3356	2.7975	3.1469	3.3385	3.8226	2.7054	2.523	
3.193	4.6372	4.0681	3.5556	3.0191	3.0894	4.0469	3.8665	
3.2626	3.3485	3.6683	3.0938	3.9501	3.173	3.4295	3.344	
3.1884	4.0361	2.3622	2.8479	2.477	2.8209	3.0849	3.0596	
2.915	3.4827	4.1867	3.5467	4.0302	2.7612	3.1951	4.4359	
2.9094	3.8839	2.8224	3.2243	3.4827	2.4983	3.0552	3.2795	
3.3722	2.8228	3.6005	4.4851	2.7013	3.2503	3.5467	4.0653	
3.5084	3.6291							
CCDC91	3.4461	3.5026	3.3657	3.7981	3.6972	3.3646	3.5708	
3.6931	3.9087	3.5664	4.5614	3.8512	4.6032	3.9328	3.5325	
3.7506	4.5165	4.3066	3.7498	3.5248	2.3961	3.758	4.0236	
2.9312	3.6538	3.738	4.0947	3.6044	3.244	4.0863	4.0357	
3.9709	3.6665	3.4089	4.162	3.6731	3.6782	3.5391	3.841	
3.7209	3.9154	3.619	3.3554	3.8563	3.3739	4.4287	4.3311	
3.8936	4.0688	4.6912	4.9272	3.8323	4.5609	4.0711	3.2977	
3.4504	4.6075	4.7002	4.0469	3.0725	3.8505	4.202	3.6066	
3.981	3.2025	4.413	3.8442	4.26	3.6624	3.2208	4.1468	

3.6602	3.6694	3.9049	3.486	3.0307	4.0403	3.2585	4.2632
4.1024	4.1218	4.0993	4.1205	3.399	2.969	4.4429	4.093
3.5731	3.9937						
CCDC92	3.4464	1.3162	4.0821	2.9483	1.9759	2.8269	3.8373
2.3259	2.9663	2.2763	3.4088	1.9818	4.48	2.4628	3.1829
3.0717	3.0239	3.9707	2.7163	3.8496	3.2881	2.8566	3.0179
2.6507	3.4472	3.5824	3.1709	3.0456	3.1923	4.2094	4.5164
2.6585	1.5862	3.7229	3.577	2.162	3.7801	3.6399	3.1954
2.306	3.8696	3.5917	3.8376	1.3409	4.086	3.2636	2.7665
3.3695	4.2119	2.8411	3.6119	2.7	3.2928	3.1078	3.4528
3.5714	3.966	2.9358	1.433	2.3581	2.1258	2.9132	2.5097
3.0061	3.6011	5.5508	3.7474	5.0087	3.97	3.474	2.93
3.2124	3.0288	2.6051	3.1992	3.6318	3.6352	3.7495	2.5791
2.8225	3.044	2.6913	4.4316	3.6242	3.9487	3.767	4.1988
3.8894	4.0013						
CCDC93	3.2864	4.007	3.3473	3.0985	1.987	3.3957	3.2877
3.0447	3.2627	3.2809	3.2051	2.9607	3.2977	3.551	3.5306
3.0205	3.4894	3.4854	3.1571	2.8273	3.2736	2.6976	2.625
3.5915	2.6411	3.6908	2.9665	3.1567	2.6338	3.3269	2.5502
2.5493	3.1226	3.2412	2.9382	3.2952	2.8488	2.9502	2.4378
2.7568	2.483	2.7366	2.6085	2.8912	2.8159	2.5442	3.3222
2.7415	2.9522	3.754	2.9661	2.7194	3.3229	2.9778	2.7446
2.9778	3.7766	2.599	3.3999	2.8773	2.6752	3.3393	2.2255
3.1563	3.1068	3.4039	2.7935	3.2941	3.4591	2.5735	2.46
2.7814	3.3892	2.9492	2.9405	3.6238	3.3286	2.963	3.0691
2.9073	3.5287	2.2281	3.7025	2.4391	2.4853	3.1023	2.7613
2.529	2.6819						
CCDC94	3.1074	3.3479	3.3998	2.9178	3.6704	3.0776	3.6161
4.2046	3.183	2.9742	3.0311	2.7959	3.3668	3.4985	3.6059
2.8426	3.2644	2.5753	3.3289	3.6745	3.3122	3.0945	2.4438
3.6861	2.4235	2.9358	2.1653	2.4324	3.347	2.8818	3.1614
3.2911	3.7085	2.3369	2.636	2.5852	3.034	3.5739	3.5418
3.2428	2.7809	2.5992	3.0558	3.0929	3.1746	3.1318	3.1687
3.1291	3.2048	2.6063	2.7367	2.7171	2.9535	3.01	3.961
4.4827	3.153	3.6258	2.4575	3.4787	3.4129	3.2096	3.0817
3.3868	3.2257	2.9366	3.2947	2.9973	3.8905	2.9152	3.2416
3.4886	3.0038	2.6593	2.6174	2.8819	3.4989	2.5494	3.7663
3.379	3.3272	2.9247	3.3363	3.0507	3.8932	3.3631	2.9719
3.0855	3.0618						
CCDC96	0.7419	0.6374	0.7437	0.6177	0.3368	0.3117	0.199
0.2938	0.177	0.3929	0.186	0.1287	0.508	0.5098	0.5115
0.1206	0.6742	0.2017	0	0.2482	0.7652	0.2773	0.5607
0.2405	0.2598	0.8788	0.1639	0.6596	0.5325	0.1227	0.3704
0.3311	0.3827	0.1508	0.3827	0.3202	0.4098	1.0204	0.0786
0.2584	0.3787	0.3211	0.6441	0.3607	0.3504	1.091	0.271
0.4203	0.545	0.9296	0.4273	0.3101	0.8069	0.4572	0.7122
0.5926	0.4252	0.3611	0.0964	0.3147	0.2289	0.2724	0.137
0.2591	0.5513	0.4027	0.7984	1.0327	0.3115	0.7204	0.6133
0.5566	0.755	0.0723	0.262	0.4313	0.6177	0.2789	0.1139
0.5546	0.307	0.4923	0.469	0.4133	1.2529	0.5219	0.6293

0.9536	0.9549							
CCDC97	2.8704	3.4872	3.4461	2.8059	3.35	2.7816	3.3278	
2.8412	2.8735	2.5743	3.3281	2.9058	3.7092	3.0164	3.6257	
2.8473	2.994	2.4091	3.1751	3.1584	2.5562	2.3598	3.2319	
3.1422	3.5209	3.8457	2.7372	3.5188	3.3854	2.6244	3.755	
3.3311	3.8369	3.0009	2.7076	2.6191	3.8593	3.535	3.6302	
3.3763	2.8068	3.0701	2.4773	2.8	3.6994	3.1825	2.7744	
2.9825	3.2043	3.9497	2.8126	2.5813	2.8342	3.9632	3.3682	
3.6914	2.8054	3.5777	2.8143	3.5273	3.4104	3.174	3.7289	
3.1678	3.4891	3.3061	2.2888	3.2574	4.125	2.8895	2.8455	
2.5176	2.9324	3.1776	2.543	3.2577	3.0908	2.39	3.2358	
2.2095	3.0006	2.9388	3.5827	2.9819	3.9185	2.9801	3.0587	
2.7141	2.7569							
CCER1	0	0	0	0	0	0	0	0
	0	0	0	0	0	0	0	0
	0	0	0	0	0	0	0	0
	0	0.0081	0	0	0	0	0	
0.0206	0	0	0	0	0	0	0	0
	0	0	0	0	0	0	0	0
	0	0	0	0	0	0	0	0
	0	0	0	0	0	0	0	0
	0	0.0031	0	0	0	0	0	0
	0	0	0	0	0	0	0	0
	0	0	0	0	0	0	0	0
	0							
CCER2	0.1021	0.0693	0	0.1232	0.1586	0.2047	0.1515	
0.1425	0.1009	0	0.0345	0.0288	0.0397	0.1407	0.1241	
0.0882	0	0	0	0.1166	0	0.1386	0.0527	
0.0939	0.0661	0.0447	0	0	0	0.0679	0.1543	
0.1122	0	0.062	0.1206	0.0533	0.0686	0	0	0
	0.2876	0	0	0	0	0	0	
0.1722	0.1653	0	0.1607	0	0	0.126	0.0502	
0.295	0.0507	0.045	0.0516	0.0455	0.2283	0.1828	0.0766	
0.1669	0.1042	0.0382	0.072	0.0188	0.0288	0.1465	0	0
	0.047	0.0261	0.0337	0.0403	0.0285	0.042	0	0
	0.2937	0.1052	0.032	0	0	0	0	0
	0.086							
CCHCR1	2.9972	3.0885	2.95	2.5997	2.1468	2.7709	2.1403	
2.3806	3.0149	3.1701	2.7795	3.1701	2.5947	2.9374	3.1508	
2.5947	3.2993	2.6409	3.1535	2.8595	2.5802	3.1053	1.9786	
2.9903	2.5625	3.1978	2.5041	3.6093	3.0404	2.2579	4.2252	
2.6874	2.8556	3.1806	3.2582	2.7997	3.3386	2.5392	2.3226	
2.2073	2.7175	2.4908	3.0558	3.4693	2.3943	3.8675	1.5808	
2.2494	2.9132	3.4265	3.0337	2.8266	2.5724	2.4067	3.4606	
2.5211	2.9637	3.0863	2.0926	3.2399	3.0111	2.8019	2.6582	
2.9839	3.1599	1.5951	3.4677	1.8204	2.2617	3.2312	3.0036	
3.1606	2.7733	3.1	2.2403	2.7783	2.9935	2.6754	2.7767	
3.7148	3.2822	2.599	2.6453	2.3013	4.1726	3.5992	2.2802	
2.9718	2.4495							
CCIN	0	0	0	0	0	0.1071	0	

0.0233	0	0	0.0124	0	0.0164	0	0	0
	0.4394	0	0	0	0	0	0	0
	0.0133	0.1477	0.0374	0.0673	0	0	0.51	
0.0017	0	0	0	0.0502	0.0105	0	0	
0.0324	0	0.0041	0	0	0	0	0	0
	0	0	0	0	0	0	0.1178	0
	0.6636	0	0.0263	0	0	0	0	0
	0.0542	0.0152	0.2372	0.0057	0	0	0.0373	0
	0.0225	0	0	0	0	0	0	0
	0	0.0119	0	0	0	0	0.1524	
0.128	0							
CCK	0	0.1712	0	0	0.3014	0	0.9341	
0.1188	0	0	0.9459	0.848	0	0.2738	0.1113	
1.796	0	1.1704	0	0	0.3416	0	0	
0.39	0	1.6911	0	0	0	0	0	0
	0.1516	0	0	0	0.0893	0	0	0
	0	0.9186	0	0	3.7265	0	0	0
	0.2027	0	0	0.2633	0	0	0.0708	0
	0	0.1215	0	0	0	0.1287	0	0
	0	1.47	0	3.2782	0.0447	0.3643	1.8292	
0.1358	0	0	0.2845	0	0	0.1211	0	0
	0.2004	0.0387	0	0	0.4892	0	0	0
	0							
CCKAR	0	0	0	0	0	0	0	0
	0	0	0	0	0	0	0	0
	0	0	0	0	0	0	0	0
	0.1455	0	0	0	0	0	0	0
	0	0	0	0	0	0	0	0
	0	0	0	0	0	0	0	0
	0	0	0	0	0	0	0	0
	0.0605	0	0	0	0.0289	0	0.0417	0
	0	0	0	0	0	0	0	0
	0	0	0	0	0.0227	0	0	0
	0	0	0.035	0	0	0	0	0
	0							
CCKBR	0	0	0	0	0	0	0	
0.1017	0	0	0.0338	0.0625	0	0	0	
0.0413	0	0	0	0	0	0.2935	0	0
	0	0	0	0	0.1022	0.0695	0.0419	
0.0134	0	0	0	0.0538	0	0.074	0.049	
0.0715	0	0	0	0.0706	0	0	0	0
	0.05	0	0	0	0	0	0	0
	0	0	0.0788	0.2809	0	0.0266	0.4086	0
	0.2938	0	0	0.0242	0	0	0.0105	0
	0.0159	0.0031	0	0	0	0.0143	0.0043	0
	0.107	0	0	0	0	0	0	0
	0							
CCL1	0	0	0	0	0	0	0	0
	0	0	0	0	0	0	0	
1.905	0	0	0	0	0	0	0	0

	0	0	0	0	0	0	0	0
	0	0	0	0	0.0513	0	0	0
	0	0.0341	0	0	0	0	0	0
	0	0	0	0	0	0	0	0
	0	0	0	0	0	0	0	0
	0	0.6643	0	0	0	0	0	
0.216	0	0	0	0	0	0	0	0
	0	0	0	0	0	0	0	0
	0							
CCL11	0	0.0737	0	0	0	0.0907	0	0
	0	0	0	0	1.7441	0	0.0409	0
	0	0	0	0	0	0	0	0
	0	0	0.0562	0	0	0	0	0
	0	0	0	0	0	0	0	
0.0498	0.0513	0.0229	0	0	0	0	0	0
	0	0	0	0	0	0.1442	0	0
	0	0	0.2503	0	0.228	0.0908	0.1036	0
	0	0.3352	0	0	0	0	0	0
	0	0	0.0823	0	0.0628	0	0	
0.1947	0	0	0	0	0	0	0	0
	0							
CCL13	0	0	0	0	0	0	0.1444	0
	0	0	0	0	0	0	0.0428	0
	0	0.0849	0	0	0	0	0	0
	0	0.0464	0	0.1941	0	0.0558	0	0
	0	0	0	0	0	0	0	0
	0	0	0	0	0	0.0554	0	0
	0	0	0	0.0583	0.1819	0	0	0
	0	0	0	0	0	0	0	
0.1345	0	0	0	0	0.0695	0	0	0
	0	0	0	0	0.059	0	0	0
	0	0	0	0	0	0	0	0
	0							
CCL14	0.6184	0	0.2595	0	0	0	0	0
	0	0	0.1015	0	0	0	0	0
	0	0	0	0	0	0	0	0
	0.0937	0	0.1705	0	0.1361	0.1477	0.1039	0
	0.3137	0	0	0	0	0	0.1187	
0.1852	0	0	0.3091	0	0.1052	0.1566	0	0
	0	0.3907	0	0	0	0	0.1473	
0.3041	0.4421	0	0	0	0	0.1273	0	0
	0.2872	0	0.2291	0	0.3569	0	0.1011	0
	0	0.0357	0.0931	0.1305	0.3516	0	0.1615	0
	0.2704	0	0	0	0.1547	0	0.3712	0
	0.2334							
CCL15	0	0.0924	0	0	0	0	0	0
	0	0	0	0	0	0	0	0
	0	0	0	0	0	0	0	0
	0	0	0	0	0	0	0	
0.0208	0.0814	0	0	0	0	0	0	0

	0	0.024	0	0	0	0	0	0
	0	0	0	0	0	0	0	0
	0	0	0	0	0.1384	0	0	0
	0	0	0	0	0.0023	0	0	0
	0	0	0	0	0	0	0	
0.0648	0	0	0	0	0	0	0	0
	0							
CCL16	0.1073	0.0366	0	0.1746	0.0379	0.1713	0	
0.019	0.1293	0.0757	0.0121	0.0424	0.0898	0.1719	0.0576	
0.0437	0.1165	0.0895	0.0727	0.0544	0.2521	0.0599	0.0081	
0.1909	0.0297	0.0474	0.0542	0.1817	0.0692	0.0276	0.0192	
0.0915	0.0349	0.0608	0.0178	0.0183	0	0.0408	0.0441	
0.0742	0.0456	0.0598	0.0797	0.0322	0.0604	0.0267	0	
0.1058	0.0723	0.1437	0.1076	0.0597	0.0615	0.0641	0.0915	0
	0.1029	0	0.0527	0.0997	0.0577	0.1254	0.1334	0
	0.1564	0.0831	0.0821	0	0.0728	0.1971	0.0352	
0.0833	0	0.0693	0.0742	0.1254	0.0283	0	0	
0.1891	0.0451	0.0635	0.0398	0.0507	0	0	0.0428	
0.1125	0.0562							
CCL17	0.3193	0.4746	0	0.1724	0	0.4772	0.1758	
0.2414	1.181	0.4134	0.3225	0.0629	0.0823	0.516	0	
1.2725	0	0.3932	0.6198	0.2055	0	0	0	
0.6411	0	0	0	1.248	0	0.4001	0	
1.6013	0.4021	0.5993	0.1788	0.2029	0.1365	0.2234	0	
0.3659	0.2699	0	0	0.2145	0	0	0.4083	
0.7409	0	0	0.4079	0	0.5216	1.0792	0	
0.789	0	0	0.2912	0.1544	0.037	0.5939	1.1387	0
	0	0	0.259	0	0.1129	0	0.4872	
0.2554	0.1676	0	0.4554	0.3168	0.3553	0.4361	1.3427	
0.952	0.8898	0.0402	0.0547	0.56	0	0.299	0.1312	
0.3209	0.157							
CCL18	0	0.5195	1.3198	0	0	0	3.5429	0
	0.0887	0.5027	0.1199	0.0965	0	0.5262	0	
1.0719	0	0.4729	1.0637	0	0	0.169	0.1017	
0.7191	0	0.0914	0	2.8969	0.871	0.4737	0	
0.138	3.6415	0.7003	0.3694	0.2574	0.3292	1.9135	0.146	
0.0969	0.0909	0.0559	0	0.1133	0	0	2.9202	
0.1173	0	0.3717	2.4872	0.1628	0.5889	0.62	0	
1.2339	0	0.0413	0.0465	0	0.8284	1.7135	0	
0.339	1.797	0.5702	0.4092	2.792	1.2919	0	0.4508	
0.1524	3.5134	0	0.1483	0.2148	2.8305	0.1219	0.4806	
0.4253	0.4421	0	1.9682	0	0	0.3127	4.5931	
5.6616	2.9282							
CCL19	0	0.2309	0	0	0	0	0	0
	0	0	0	0	0	0	0	0
	0	0.2266	0	0	0.2544	0	0.0677	0
	0	0	0	0	0.1464	0	0	0
	0.1883	0.1514	0.1001	0	0	0	0.1133	0
	0	0	0	0	0	0	0	0
	0	0	0	0	0	0.0941	0.079	

0.1655	0.375	0	0	0.1077	0.1682	0.0428	0	
0.182	0	0.1015	0.1142	0	0	0	0.0554	
0.0519	0	0.0083	0	0.0659	0.1907	0	0	0
	0.477	0.0304	0	0	0	0.1525	0.3202	
0.1886	0							
CCL2	0	0.8774	0.136	1.9855	6.2478	3.6225	5.0106	
5.9336	6.1794	0.748	0.2108	8.824	7.8988	0.3087	5.379	
5.5948	1.7728	1.6811	1.9066	0	1.019	7.6064	8.5639	
1.7431	3.7109	8.6421	1.1137	0.3783	5.1894	0.3142	0.0486	
1.7785	0.5543	3.5118	5.305	0.6736	0	0.2041	0.1371	
0.0091	7.3265	6.3808	9.7205	3.6173	6.0749	7.2793	1.8978	
4.6385	8.824	0	3.9909	10.3765	4.2967	0.421	0.7914	
1.1238	0.945	0	0.3613	0	2.873	2.6988	0.9679	
1.6596	0	9.6902	0.5376	6.0969	1.2207	5.4843	1.0729	
4.2903	0.3592	5.5714	7.2338	0	1.3303	3.4131	1.4294	
1.23	0.6549	6.5521	3.1679	8.0102	7.7355	8.201	1.9117	
0.0699	0.537							
CCL20	0.477	0.6982	1.4337	1.8504	2.2718	0.294	5.5325	
3.203	1.066	0.816	1.1869	7.0477	3.337	1.85	0.2189	
1.7808	7.4846	2.0693	1.7584	0.2504	1.1041	2.2872	1.8876	
1.1854	0.7293	1.199	0.4142	1.939	1.6774	3.0562	0.3593	
3.6704	1.8642	0.7284	0.7018	1.1113	2.225	0	0.4586	
0.3482	2.9987	0.3464	4.2528	0.8334	0.2537	0.9645	2.2617	
1.7371	5.7805	2.0188	1.5443	8.3328	2.5316	2.8604	0.1188	
2.4209	0.6488	0.1828	2.6092	0	0.6765	3.0862	0.0162	
1.3986	0	5.7093	0	0.9425	2.3759	2.085	1.5785	
2.7923	0	5.8137	0.8944	0.5085	0.2906	0.0939	1.3412	
0.9673	2.2968	0.8216	3.6798	0.6409	1.0451	2.9403	0.6427	
0.2811	0.3065							
CCL21	0	0	0	0	0	0	0	0
	0	0	0	0	0	0	0	0
	0	0.1129	0	0.1316	0	0	0	0
	0.0397	0	0	0	0.0563	0	0	0
	0	0.1465	0.0629	0	0.1235	0	0.0542	0
	0	0	0	0	0	0	0	0
	0	0	0	0	0	0	0	0
	0	0	0.0731	0	0	0	0	
0.0847	0.1539	0	0	0	0.0411	0	0.0523	0
	0	0.0364	0	0.1267	0.0375	0.1729	0.0356	0
	0	0	0	0	0	0	0.2845	0
	0							
CCL22	0.0837	0.049	0.0389	0.0373	0.0336	0.0609	0.0085	
0.0795	0.1448	0.1025	0.0326	0.0039	0.0252	0.1095	0.0565	
0.0233	0.0407	0.0854	0.142	0.1074	0.1295	0	0.0125	
0.0501	0	0.0271	0.1215	0.0395	0.0488	0.0554	0.0798	0
	0.0657	0.1008	0.0641	0	0.0617	0.0261	0	
0.0157	0.0309	0.0192	0	0.1247	0.0076	0.056	0.1332	
0.0739	0.0124	0	0.0506	0	0.077	0.0343	0.033	
0.0607	0.0872	0.0309	0.0306	0.1395	0.0578	0.0157	0.0863	
0.0374	0.063	0.0154	0	0.0186	0.0337	0.1137	0	0

	0.0997	0.0291	0.0208	0.0499	0.0108	0	0.0123	
0.1636	0.0203	0.0466	0.0377	0.0701	0.061	0.1051	0.0932	
0.0789	0.0897							
CCL23	0	0	0	0	0	0	0	0
	0	0	0	0	0	0	0	0
	0	0	0	0	0	0	0	0
	0	0	0	0	0	0	0	0
	0	0	0	0	0	0	0	0
	0	0	0	0	0	0	0	0
	0	0	0	0	0	0	0	0
	0	0	0	0	0	0	0	0
	0	0	0	0	0	0	0	0
	0.0699	0	0	0	0	0	0	0
	0	0	0	0	0	0	0	0
	0							
CCL24	0	0	0	0	0	0	0.219	0
	0	0	0	0.2094	0.5619	0	0	0
	0.4882	0.3709	0	0	0	0	0.1123	0
	0	0.2453	0	0	0	0	0	
0.1078	0	0	0	0	0.1626	0	0	0
	0	0	0.2765	0.4579	0.1758	0.6438	0	0
	0	0	0	0	0	0	0	0
	0	0	0	0	0	0	0	
0.1916	0	0.0795	0	0	0	0	0	0
	0	0	0	0	0.1303	0	0	0
	0	0	0	0	0.3104	0	0	0
	0							
CCL25	0	0	0	0	0	0	0	0
	0	0.0584	0	0	0	0	0.0449	0
	0	0	0	0	0	0	0	0
	0	0.0479	0	0	0	0	0	0
	0	0	0	0	0	0	0	0
	0	0	0	0	0	0	0	0
	0	0	0	0	0	0	0	0
	0	0	0	0	0	0	0	0
	0	0	0	0	0	0	0	0
	0	0	0	0	0	0	0	0
	0	0	0	0	0	0	0	0
	0							
CCL26	0	0	0	0	0.1999	0.4948	0.6371	0
	0	0	0	0.268	2.6637	0	0.1647	0
	0.7023	0	0	0.1997	0.1629	0.1277	1.5939	0
	0	3.2906	0	0	0.078	0	0	
0.1515	0	0	1.0768	0.0629	0.1302	0	0	0
	0	2.0491	1.1634	0	1.3806	2.9449	0	0
	0	1.5871	0.0879	0.0928	0	0	0	0
	0.1848	0	0	0	0	0	0	
0.1865	0	0.4459	0	0.0893	0	0	0.8297	
0.1557	0.1583	0.1087	0.1214	0	0	0.7046	0	0
	0	1.5775	0.477	0	1.0808	0	0.1248	0

	0							
CCL27	0	0.1959	0.4158	0	0	0.6954	0	
0.3633	0.1322	0	0.1669	0	0.0999	0	0.2469	
0.4035	0.7143	0.3996	0	0	0	0.2007	0	
0.0762	0.0832	0	0	0.7475	0	0	0.4214	
0.1386	0	0	0.075	0.2984	0.09	0	0.5213	
0.1932	0	0	0.3176	0	0	0	0	
0.5955	0.217	0	0.142	0.1472	0	0.4164	0.7247	0
	0	0.1769	0.5306	0	0	0	0.1644	
0.3915	0	0	0	0	0	0.4561	0	
0.3309	0	0.4458	0.3842	0	0	0	0	
0.5039	0	0.1075	0	0	0.3543	0	0.3173	0
	0.1951							
CCL28	4.0962	2.8192	3.0444	3.68	2.2276	4.0139	2.8761	
0.4099	3.7115	3.6166	3.3564	2.322	3.3128	4.0593	2.8201	
3.1409	2.6039	3.2693	3.7312	3.4842	4.2114	1.6572	2.7184	
2.8038	2.1247	3.4926	2.3492	3.2841	2.7601	4.465	1.791	
1.2982	2.9718	2.7772	3.1045	3.5255	2.0525	3.3066	1.3435	
3.7465	3.5354	2.5346	2.6271	3.6505	1.0279	3.349	3.0892	
3.3341	2.8052	2.0168	3.0928	2.9608	3.299	3.1006	0	
3.3989	0	1.4028	3.264	1.3594	3.4932	3.9037	1.4952	
3.7628	3.0133	1.1027	3.6423	3.5607	2.8784	2.8106	3.2246	
3.6248	3.4884	2.9983	2.5238	2.3718	3.6401	2.846	4.0961	
4.6914	3.2567	3.2985	4.0479	2.3538	2.7169	2.921	4.8568	
4.2546	4.2694							
CCL3	0.1411	0.2239	0.2813	0	0.1805	0	1.6718	
1.4323	0.2773	0.4147	0.6906	2.0216	2.9466	0.1307	0.1851	
2.9998	0.9561	0.2165	1.2088	0.3172	0	0.2627	0.0876	
0.2367	0	2.5882	0	0	1.0201	0.533	0	
0.7495	3.5462	0.2434	0.0545	0.1322	0.7909	0.0716	0	0
	0	0	0	0.0581	0	0	1.8664	
0.7074	0.1031	0	1.8859	0.2105	0	0.1325	0	0
	0	0.1615	0.2451	0	0.2354	0.4284	0.1449	
0.7576	0.1693	4.889	1.3047	1.3596	0.6946	0	0.1412	
0.8058	0.5345	0.4714	0.1822	1.9977	0.1272	0	1.0118	
0.5004	0	0.3115	4.8163	0.1516	0	0.2294	4.1213	
3.1794	2.5249							
CCL3L3	0.0212	0	0	0	0	0	0.0021	
0.0868	0.004	0.0054	0	0.1502	0.0022	0.0199	0.0016	
0.2049	0.0173	0	0.03	0	0.0178	4.00E-04	7.00E-04	0
	0	0.0032	0.0092	0	0	0	0.0023	0
	0.0087	0.0059	0	0.0036	6.00E-04	0.0136	0	
0.0064	0	0	0	0.0087	0.0435	0	0.0147	
0.0109	0.0049	0	0.0054	0	0	0.0055	0	0
	0	0	0	0	0	0.0057	0.009	0
	0	0.254	0.0277	0.0031	0.0066	0.0363	0	
0.0086	0.0845	0.0023	0.0102	0.0124	0.004	0	0.0042	
0.0256	0	0.0057	0.0076	0	0	0	0.0142	
0.0169	0.266							
CCL4	0	0	0	0	0	0	0.432	

7.00E-04	0	0	0	0	0	0	0	
1.9771	0	0	0	0	0	0	0	
0.0029	0	0.436	0	0	0	0	0	0
	0	0	0	0	0	0.0125	0.148	0
	0	0	0	0.0069	0	0	0.2706	0
	0	0	0.0031	0	0	0	0	0
	0	0.0184	0	0	0.0946	0	0	0
	0	1.7215	0	0.0073	0.01	0	0.0108	0
	0.0155	0	0	0	0.0078	0	0	0
	0.0109	0.0094	0.0109	0	0	0	0	
0.131	0							
CCL4L2	0.0187	0	0	0	0	0.014	0.0725	
0.1455	0	0	0	0.1222	0	0	0	
0.5848	0	0.0125	0	0	0	0	0	0
	0	0.002	0	0	0	0	0	0
	0.007	0	0.16	0	0	0	0	0
	0	0	0	0	0	0	0	0
	0	0	0	0	0	0.0124	0	0
	0	0	0.2206	0	0.0223	0	0	0
	0	0.0084	0	0	0	0.0586	0	
0.0173	0	0	0	0	0.0099	0	0	0
	0	0	0	0	0.0407	0	0	
0.1166	0							
CCL5	0.8025	0.0927	0.2644	0.6462	0.3935	0.3367	2.8737	
0.1706	0.4248	0.1736	0.1775	4.3178	6.103	0.2228	1.516	
0.1717	1.2772	0.576	0.4375	0.3241	0.2696	0.5482	1.8672	
0.24	0.1623	6.2164	0.4375	0.1886	0.2832	0.0987	0.1299	
2.6482	0.373	3.5979	2.3489	0.2053	3.75	0.2133	0.0457	
0.2365	0.7201	0.4417	0.8995	0.4498	2.6567	3.3791	0.3217	
0.3931	1.9891	1.4231	4.7474	2.0269	0.4992	0.2357	0.1633	
0.2725	0.3438	0.2018	0.3165	0.1564	2.4369	0.8438	0.1451	
0.3538	0.4403	5.765	0.2907	1.3088	0.3634	0.596	0.544	
0.7342	0.779	5.8468	0.2291	0.7417	2.4819	0.972	0.3384	
0.2934	0.3319	1.0798	1.6098	0.1948	2.1146	0.2445	0.2228	
0.3538	0.2921							
CCL7	0	0	0	0.1426	0	0	0.9319	
0.1125	0	0	0.0821	2.4372	1.1658	0	0	
0.1074	0	0	0.2181	0	0	0	0	
0.1707	0	0.1791	0	0	0.1021	0	0	0
	0.1369	0	0	0	0	0	0	0
	0	0.5568	0.8336	0	0	0.2289	0	
0.1431	0.2097	0	0.4431	0.1269	0	0	0	0
	0	0	0	0	0.0339	0	0	0
	0	6.2714	0	0.2773	0	0	0	0
	0	0.011	0	0	0	0.045	0	0
	0	0.0897	0	0.0546	0.0495	0	0.3843	0
	0							
CCL8	0	0.1087	0	0	0.085	0	0.1819	
0.074	0	0	0	0.0449	0.125	0	0.057	
0.0648	0	0.1175	0	0	0	0.1393	0	

0.0535	0	0	0	0	0.0759	0.1435	0	0
	0	0	0	0	0	0	0.1214	0
	0	0.1315	0	0	0	0	0	0
	0	0	0.0808	0	0	0.0119	0	
0.0622	0	0	0	0	0	0.0491	0.0543	
0.0369	0	1.88	0	0	0.0157	0.0459	0.0182	
0.0241	0	0.0356	0	0	0	0	0	0
	0	0	0	0	0	0.0481	0	0
	0							
CCM2	4.3225	2.075	4.0495	3.6716	3.7228	2.4938	3.741	
4.5806	3.9731	3.7547	4.5227	3.8091	4.8834	2.8418	4.8153	
3.076	4.0572	3.272	3.5785	5.3604	4.4758	4.8116	5.0981	
4.7827	4.3244	4.9473	2.2277	3.8498	4.8267	3.5941	4.7583	
4.7002	3.2792	4.9748	4.7645	3.5167	4.4491	4.2243	4.3528	
3.7518	4.9743	3.7926	5.4549	3.1512	3.9733	4.3438	3.4518	
3.7478	5.2349	2.6996	4.2351	3.6942	4.7119	4.1846	4.2788	
4.1416	3.9866	4.8811	3.5447	4.8932	3.7423	3.126	3.4813	
3.3727	4.4633	4.4288	3.4864	5.1226	3.1053	5.2285	3.5557	
4.367	4.2176	4.2541	2.5854	3.7092	3.6318	4.4134	3.9335	
3.7238	3.8297	3.2557	5.4285	4.3619	5.5685	5.8418	4.6642	
4.5128	4.4884							
CCM2L	0.1158	0.1591	0.2639	0	0	0.0588	0.0556	0
	0.081	0.0634	0.0764	0.1263	0.1228	0	0	
0.0239	0	0.0559	0	0	0	0	0.0317	0
	0	0	0	0.3413	0	0.3336	1.8193	
0.0638	0	0.0365	0	0.3482	0.2711	0.1076	0	0
	0.0288	0	0	0	0	0.1021	0	
0.1096	0	0.188	0.1396	0	0.0753	0.0048	0.517	
0.0866	0.0122	0	0.031	0.0122	0	0	0.0994	0
	0.3385	0.0351	0.77	0	0	0.1315	0	0
	0.3014	0.0417	0	0	0.1114	0.1456	0	0
	0.6682	0	0	0.0638	0.0457	0	0	0
	0							
CCNA1	0	0.0251	0.4619	0	1.0934	0.2734	1.0018	
1.0893	0.0017	0.0604	0	3.7502	0.969	1.593	1.598	
1.529	0	0.0374	1.3318	1.8784	0.0416	0.173	0.3029	0
	0.5705	0.111	0.5985	0.039	0.3119	0	0.0568	
0.7281	2.2325	3.3827	0.228	0.0223	0.8956	0.1027	0.2764	
0.2177	0.0128	1.4977	2.5676	0.0684	2.1867	0.1102	0.4584	
0.4498	0.0057	0	0.4796	0.0384	0.1995	0.0679	0.2128	
0.1116	2.7259	1.1274	0	1.8776	0.2557	0.0731	0.1981	
0.3546	0.0764	0.8298	0	0.3941	0.0923	0.8346	0	
0.149	0.0676	0.314	0.3394	0	0.1094	0	1.3713	
0.0908	1.7672	0.1573	2.6951	0.1114	0.1741	0.232	0.1381	
0.2719	0.1645							
CCNA2	4.0093	4.6861	5.2412	5.6807	5.0184	4.2538	2.5314	
4.3934	5.0812	5.0825	2.9747	5.1552	4.4363	5.2376	4.9465	
3.0622	4.0891	4.4476	5.5897	5.1506	5.5546	5.0547	3.9778	
4.9991	4.1151	3.4113	4.796	5.3353	4.3182	4.2495	5.0677	
5.2404	5.1974	3.841	4.8039	5.5855	3.86	2.4899	4.1074	

4.7633	3.5536	4.07	5.4009	5.4236	5.094	5.0644	5.2109
4.1328	3.3071	6.2409	4.4873	4.9624	4.2658	3.3478	4.9902
4.8186	4.8382	5.3394	4.5351	5.8263	4.8899	5.2341	5.3957
4.7627	5.3022	2.7123	4.8991	2.4746	4.2674	5.0172	4.3289
5.3138	4.7797	4.5485	4.57	4.0135	5.1365	4.3007	4.2018
4.9768	5.1466	3.4141	1.8327	4.1634	5.2042	4.9155	4.1165
4.8554	4.4057						
CCNB1	5.5418	5.6535	6.4109	6.8589	6.5582	5.6236	3.8152
5.7513	5.7749	6.0356	4.6024	6.345	7.3033	6.0799	6.9116
5.1502	6.3005	5.0013	6.575	6.8031	6.064	6.0827	5.5332
6.5618	5.8977	4.6274	4.8958	5.7414	6.5732	5.6656	6.3679
5.6555	7.1592	5.5485	6.4312	6.5018	4.9987	4.0658	5.0321
6.4785	4.6949	5.5024	6.382	5.6391	6.7274	7.4703	6.1979
5.4367	4.8481	7.1965	5.5979	5.2207	5.4497	4.5042	6.2741
5.8276	6.2162	6.3352	5.8965	6.4124	6.4169	6.5963	6.3676
6.6669	6.1866	3.3434	5.2654	3.8761	5.677	6.5943	5.4524
6.3786	5.9387	5.9996	5.3695	6.0623	6.5647	4.7244	5.7747
6.5647	6.6619	4.5057	4.1939	5.069	6.6462	5.713	4.9954
6.0773	5.6198						
CCNB1IP1	5.8669	4.6487	4.2482	5.2535	4.1519	4.7369	4.4053
4.83	5.0081	5.4294	5.5976	5.4638	4.8245	4.5013	4.3932
3.5853	5.1086	4.6176	4.4729	4.6491	5.2372	4.381	4.6325
4.049	5.1355	4.5691	7.5992	4.5158	4.2531	5.4451	5.1475
4.1332	4.3239	4.75	3.4999	5.1399	4.9432	4.9328	5.841
5.0546	5.8378	3.6051	4.7713	5.8483	4.9506	4.8738	5.6931
4.6876	6.2184	5.6433	4.605	5.1447	5.608	5.5873	4.3605
4.908	5.4577	4.8916	4.0142	5.125	5.4034	5.7853	5.106
4.4691	6.0662	3.9885	6.3904	3.9699	4.761	4.1384	4.7809
4.7075	4.7107	5.1243	5.0407	4.2547	4.5075	3.5882	5.2788
4.9679	4.4958	5.5108	4.5695	4.2928	4.3851	5.0343	5.2506
5.0202	5.5605						
CCNB2	4.7875	6.0471	0.1272	5.5626	5.3175	5.058	3.2038
4.4611	5.7271	5.7709	4.4471	4.7839	5.5577	5.1454	5.1197
4.7018	4.7463	5.9186	5.5937	5.3937	5.502	5.4888	4.7468
5.3293	5.0445	3.133	4.647	5.3398	5.422	5.4702	4.69
4.902	5.2525	4.9264	5.2084	5.8724	4.7304	3.0569	4.6158
5.3674	4.1437	4.3905	5.7069	5.3293	6.2324	6.638	5.8458
5.1062	4.0097	6.4118	5.0861	4.1649	4.8546	4.2846	4.9356
5.7826	5.1376	5.3237	5.0333	5.1502	5.7449	5.9156	5.8511
6.4426	5.5399	2.4076	4.2415	2.3266	5.0666	5.1319	4.2584
5.3109	4.7927	5.0961	5.3663	5.0404	5.6125	3.9388	5.2558
5.9698	6.251	3.66	2.5477	4.51	5.9469	5.0095	4.6062
5.4273	5.1448						
CCNB3	0.6911	0.0275	0.3761	0.1475	0.3866	0.4006	0.8364
0.2501	0.1394	0.2614	0.1034	0.2875	0.7226	0.4099	0
0.8863	0.3089	0.5137	0.1456	0.1015	0.4369	0.0468	0.1137
0.4335	0.0941	0.1922	0.1369	0.3195	0.175	0.1218	1.7256
0.1702	0.51	0.5827	0.1997	0.4182	0.6882	0.1163	0.1295
0.2667	0.1006	0.0879	0.2499	0.6499	0.4133	0.0515	0.3887
0.3312	0.0746	0.9022	0.2465	0.559	0.3836	0.3996	0.9256

0.5861	0.8306	0.1235	0.095	0.0428	1.3441	0.0417	0.0751	
0.3895	0.1944	0.306	1.1832	0.3233	0.7316	0.2042	0.2351	
0.2086	0.2463	0.1223	0.572	0.6351	0.2074	0.3861	0.1604	0
	0.2277	0.2413	0.223	0.3632	0.1649	0.0856	0.7179	
0.3911	0.6525							
CCNC	4.0204	4.7998	4.7857	5.0476	4.8665	5.0616	4.4077	
4.8143	5.3974	4.4926	4.6464	4.6395	4.0137	5.8958	4.991	
4.4311	4.8835	4.4643	5.664	4.9474	5.2101	5.797	5.7702	
4.7775	5.1942	4.618	5.5709	3.7393	4.7526	4.9072	5.4348	
5.2295	4.9713	4.4242	5.1197	5.0891	3.7605	3.6351	4.552	
5.4672	4.9618	5.0009	4.7308	5.1487	5.1928	5.1142	5.1628	
4.5714	4.8115	5.2141	5.579	4.948	5.0143	4.6395	5.3788	
5.2105	4.6583	5.2254	5.1575	5.3357	4.9115	5.1564	4.7783	
4.1785	3.7116	3.5476	3.6799	3.4726	4.99	4.6102	4.9489	
5.3137	5.1722	5.8708	4.2929	4.9129	4.9626	4.4635	4.5396	
5.0591	5.1316	5.0764	5.0029	5.0447	4.2002	5.2307	4.0195	
4.373	4.2254							
CCND1	7.0019	6.9176	9.0348	5.755	7.728	7.3785	7.2244	
8.146	7.2275	6.7012	5.0487	5.4242	6.4486	5.7175	6.1653	
9.1544	7.1875	8.986	8.7332	7.3068	5.1655	6.5702	7.3947	
5.589	8.4718	8.4979	3.9814	8.7026	8.201	6.7052	1.2544	
7.7493	7.1584	5.7897	8.8379	6.1611	6.4949	8.4892	7.1027	
5.5154	6.0178	5.7569	7.3474	8.5816	7.1613	7.4572	7.5474	
7.6661	8.1047	6.9243	7.3036	5.9685	4.6207	8.4346	5.3984	
1.0867	7.7033	6.5283	7.1177	7.3718	6.8175	5.898	7.1154	
5.8434	6.7634	7.4403	6.6291	9.3307	9.0979	8.5855	6.8907	
7.5358	5.271	6.3509	8.4812	9.742	7.3633	6.3899	7.7327	
6.7755	8.1179	7.3689	7.7757	6.7833	7.4894	4.0912	7.0545	
8.1689	8.0057							
CCND2	0.0574	0.0167	0.0291	0.0393	0.0058	0.0272	0.04	
0.0139	0.0221	0	0.0867	0.008	0.4453	0.0852	0.11	
0.0885	0	0	0.0557	0.0291	0.0288	0.0559	0.0685	
0.0225	0.0092	0.4869	0.5509	0.0436	0	0.0154	0.0088	
0.0185	0.0451	0.0186	0.1083	0.0529	0.1521	0.0305	0.1428	
0.0242	0.0114	0	0.0251	0	3.3848	0.1244	0.5297	
0.0199	0.1271	0.0483	0.0807	0.0408	0.0362	0.0713	0.0147	
0.0259	1.0187	0	0.0061	0.0067	0.0333	0.1257	0.0066	
0.0286	0.0418	2.6762	0.0354	1.5402	0.0156	0.0228	0.0198	
0.0146	0	0.0625	0.0196	0.021	0.0456	0	0.1434	
0.0149	0.0362	1.00E-04	0.0241	0	0	0.0238	0.8384	
0.1421	0.1026							
CCND3	5.2201	5.5537	5.5266	5.2241	5.3262	4.9877	3.5728	
5.3162	4.6545	5.3277	4.6291	5.1832	4.6371	5.767	5.8844	
4.044	3.9768	4.6225	5.3858	6.2253	5.6975	5.3409	4.9762	
5.1243	4.7965	5.0455	5.0506	5.2562	5.5282	4.5139	3.66	
4.9721	5.4752	5.5887	5.6089	5.587	4.7045	5.0473	4.4567	
4.9121	4.4489	5.1445	5.602	5.2484	5.1783	6.4302	4.4185	
4.1081	4.0764	4.1425	4.8998	5.2076	5.1819	4.2482	5.0991	
5.4543	4.0747	5.5462	4.5219	5.1255	5.1588	4.8815	4.8796	
5.4597	5.7999	3.9767	6.9609	5.163	4.5653	4.8662	6.0115	

5.7581	5.6936	5.1638	4.2585	5.3442	5.7709	5.5007	4.7085
4.7073	4.164	5.3051	5.3904	5.1255	5.5796	5.5796	5.5245
5.6959	5.6922						
CCNDBP1	3.4362	4.2632	2.9822	3.8845	3.9619	3.4575	4.291
3.647	4.0868	4.0448	4.5995	3.4827	3.3264	3.6541	3.0828
4.7994	3.702	4.5029	3.6084	4.1298	4.0333	3.4448	3.7628
3.9103	3.0859	3.4103	4.2867	4.1368	4.1978	3.8464	3.6158
3.8542	3.2834	4.0993	3.9723	3.7541	4.064	4.3914	4.1508
3.7751	3.5202	4.4864	3.6865	3.6243	4.1539	4.6335	3.8395
3.7852	3.4269	3.353	4.1258	3.2214	3.4069	4.6457	3.6077
3.9983	4.8062	3.6994	3.9128	3.4498	3.7255	3.7402	3.1999
3.8294	3.2359	3.861	3.7656	4.7708	4.2793	3.3975	3.0945
3.1585	3.3466	3.592	3.4075	4.2575	3.2868	4.0881	4.1535
3.4747	3.5843	3.8614	4.2294	3.4111	4.2476	3.2366	4.7335
3.8647	4.2667						
CCNE1	2.8498	2.6839	3.6367	4.464	3.6976	3.7075	3.6416
2.9005	2.6607	2.8162	4.0509	2.9857	3.4543	3.8097	3.993
3.3778	2.0177	2.337	2.9506	3.4137	3.6567	2.7635	3.8288
3.1246	2.9394	3.134	3.2128	2.5323	3.7039	3.3948	4.0734
2.8887	3.4962	3.6521	3.2761	3.5545	3.4574	2.1928	2.9429
4.2408	2.5865	2.3597	3.1433	3.2765	3.3618	3.4064	3.3145
3.046	1.6426	1.7367	2.3587	4.1112	2.822	2.3088	4.7256
4.71	1.3783	3.8004	2.9176	3.3101	3.4464	3.5549	3.5149
3.1601	3.0353	2.1294	3.1353	2.717	3.063	2.6928	3.6068
3.0847	3.8365	3.2164	2.999	3.2205	2.7927	3.0615	3.186
3.0908	3.0788	2.4908	2.2867	2.5797	3.6678	3.0933	2.3592
2.9394	2.5012						
CCNE2	3.3905	2.8386	4.2687	4.7883	3.6005	4.5617	2.7177
2.5084	2.6282	2.6564	0.9829	1.8963	3.5398	4.7255	4.231
2.3486	2.3279	3.1945	4.5506	3.1275	3.8182	2.5434	3.3543
3.3157	3.6105	1.6302	0.7895	3.1631	3.4288	1.4199	2.9543
2.9035	4.1072	1.6393	3.5535	3.7948	3.7348	2.069	1.7607
2.8382	1.3436	1.9528	3.7503	4.8691	3.6844	4.6589	3.9336
2.9167	1.8783	4.2583	2.8386	4.503	3.3669	2.0306	3.6757
4.0065	2.5736	4.9971	2.7669	4.9872	3.3724	2.4434	2.9848
3.4101	4.2905	0.2593	2.5405	1.7235	2.4599	3.6048	2.067
3.8597	4.1318	2.0781	3.4584	2.9418	3.2031	2.4513	4.1986
3.4519	3.0394	2.1698	0.8605	2.4006	4.2534	4.0612	1.8464
3.9812	2.6973						
CCNF	2.657	3.1547	4.0405	3.3747	3.5851	3.2197	1.859
3.7018	3.3514	3.3274	1.9137	2.4438	3.3194	3.1368	3.6271
2.7737	2.6701	3.1547	4.0712	3.571	3.4583	3.4264	2.5849
3.7252	3.2865	1.8933	1.6261	3.5065	3.6136	2.642	2.8302
3.0191	3.9797	2.9696	3.3386	3.2873	2.2281	1.8795	2.4412
2.8902	2.09	2.2214	3.7014	3.6836	3.1946	3.3966	2.933
3.1075	2.1206	3.4398	2.2681	2.5173	2.1171	2.0174	3.2284
3.7139	2.4642	4.0779	2.9243	3.684	3.6524	3.1527	3.746
3.7083	3.5394	1.1559	2.8923	1.2628	3.3606	3.7514	2.8657
3.6023	3.5602	2.9853	3.3328	3.1485	3.4426	2.7555	3.2188
3.3372	3.6234	2.1524	1.4868	2.308	3.6773	2.981	1.9581

3.4944	3.0201						
CCNG1	6.1074	5.3682	6.5909	5.9484	5.7812	6.0602	5.8429
6.3318	6.3218	5.3923	6.0504	6.288	4.4155	4.7549	6.8196
5.9878	7.1333	5.4449	6.7967	6.3074	5.6597	6.0372	6.4956
5.706	6.3022	5.3213	7.9392	6.1172	6.0951	6.656	5.5182
5.8697	6.1676	6.1822	6.4171	5.696	3.582	6.4634	5.5182
6.3563	6.8962	6.5753	6.3947	6.34	6.0722	6.6814	6.2361
6.581	6.2752	5.4835	6.1145	5.7985	5.5917	5.4079	5.1379
5.9512	6.5082	4.7515	5.9444	4.7764	5.8303	5.705	6.8452
5.9984	6.6606	6.5278	6.2578	6.5055	6.2264	5.5395	6.6014
5.3171	6.2729	6.1773	5.5963	6.288	5.8021	6.7558	7.6417
5.7067	6.5941	7.2045	7.0871	4.6697	5.3649	5.5571	6.9934
6.8126	6.9749						
CCNG2	1.946	2.2114	2.6462	2.9253	2.5085	3.317	3.4832
2.2393	2.6851	2.6819	3.2733	3.6906	3.2429	2.6211	2.8032
2.3755	2.8582	2.8737	1.9072	2.6315	3.0572	2.7345	3.1473
1.7388	3.345	4.041	4.052	2.9819	2.1153	2.5024	3.5177
1.9951	2.7084	2.7084	2.3373	2.8402	2.0656	3.3339	3.2671
2.1313	3.2611	3.6207	2.5141	2.6394	2.9329	3.8591	2.7892
3.498	2.9483	3.7021	2.9535	3.7003	3.6594	2.2128	2.0423
2.1903	4.1097	2.544	1.7327	2.8711	2.736	3.0112	2.8427
2.9659	3.5827	3.7318	3.5802	3.5215	2.2962	2.2209	3.3609
3.1632	2.7074	2.6736	3.4047	2.607	2.7127	3.5104	2.3841
2.6021	3.3307	4.2107	2.1908	3.198	2.5587	3.3994	4.2815
3.1826	3.9367						
CCNH	4.6995	4.7288	5.1822	4.9934	5.5102	5.2536	5.3767
5.5134	4.63	4.693	5.3995	5.7585	6.5625	5.5905	4.8594
4.8622	5.3401	3.7056	5.8532	5.7126	5.1649	4.2683	4.6713
5.3737	4.7141	5.2696	6.1099	4.8495	4.7006	5.0487	4.1502
5.5619	5.7028	5.7553	4.4133	5.3536	5.9662	5.1872	4.5934
5.1543	4.9307	5.3131	4.4875	4.9934	4.9272	4.9675	5.4827
5.0235	4.4963	6.399	4.747	4.9656	4.9164	4.9966	4.1829
4.8846	5.6487	4.7954	4.819	4.5512	5.0713	5.0635	4.2494
5.4939	4.382	5.2377	5.0805	4.4552	5.1874	5.4744	5.1588
5.3894	5.7541	5.4781	4.5509	4.7191	5.0127	3.9103	5.8836
5.9419	5.7747	4.741	5.8818	4.5926	5.3917	5.2148	5.0663
5.1374	4.8746						
CCNI	6.8606	6.2973	6.8425	7.1138	6.0966	6.8475	6.7417
6.0938	6.7239	6.8313	7.1004	6.9233	6.3611	6.4933	5.7493
6.0277	6.6486	6.9924	6.959	6.7317	6.6464	6.3076	6.0135
6.1098	6.8446	7.0177	8.2754	7.0861	5.3273	6.7988	7.0752
6.6772	5.9666	6.675	6.5528	6.8766	6.6113	6.8122	6.7298
6.3769	6.8906	6.624	6.8696	6.816	6.2833	6.6464	7.0893
6.6486	6.9284	7.0662	6.9972	6.6837	7.162	7.5078	6.699
6.401	6.822	6.673	6.5132	6.7986	6.3348	6.861	7.1353
6.3852	7.3768	6.7065	6.5966	6.7232	6.3138	6.8528	6.6343
6.6939	6.1486	6.0554	6.7572	6.9264	6.0076	6.7156	6.4706
5.5896	7.1282	6.7255	6.7121	6.7739	5.4801	6.6208	6.9875
6.6733	6.9995						
CCNI2	0.0896	0	0.0458	0.0406	0.0527	0.0696	0

0.0361	0.0913	0.1564	0.0512	0.2364	0.032	0.0438	0.1019	
0.0312	0.144	0.1823	0.2685	0	0	0.0224	0.0068	
0.0683	0.0409	0.0154	0	0.1156	0.0203	0.043	0.0164	
0.0483	0.0512	0.0692	0.0409	0.0612	0.0271	0	0.0347	
0.0409	0.038	0.085	0.0354	0.0768	0.0751	0	0	0
	0.0604	0.3974	0.1164	0.0669	0.4502	0.1962	0	
0.1262	0.2038	0.0289	0.029	0.0857	0.092	0.0128	0	
0.0384	0.0359	0.0977	0.3423	0.0171	0	0	0.0452	
0.0805	0.0569	0.08	0.0184	0.0531	0.0516	0	0.0732	
0.0474	0.0464	0.0306	0.0156	0.0526	0	0.2638	0	0
	0.0727							
CCNJ	2.267	2.3905	3.3048	2.7379	2.6449	2.366	2.7686	
2.5118	2.0697	2.7151	1.1311	2.5355	1.8609	2.3848	1.9931	
1.9104	2.3314	2.1141	2.1935	2.3091	2.6397	1.7102	1.176	
2.4126	2.0835	1.3538	2.1855	2.8571	1.4318	1.4727	2.3665	
2.0466	1.9081	1.0067	1.2154	2.8082	2.6598	1.7138	1.9958	
1.681	1.793	0.8089	1.3182	1.8726	2.0758	2.4905	2.8714	
2.4595	1.1199	2.0854	1.9758	2.0429	1.9562	2.2702	3.5473	
2.4602	1.6414	3.0057	1.9151	3.2888	1.4798	2.3128	2.7017	
2.2837	2.601	1.8042	2.2353	2.6759	2.5538	1.6439	1.5574	
1.9566	1.6638	1.3667	1.4823	2.1272	2.5393	1.9191	1.1546	
1.9434	2.3158	2.0696	1.8366	1.1639	1.9003	1.8998	1.9296	
2.5681	2.0928							
CCNJL	1.7922	2.9404	2.8379	3.5434	3.3611	3.1346	2.8167	
3.6118	2.6856	1.2275	1.0228	2.6767	2.9166	2.8141	3.166	
3.585	2.1164	2.3243	3.4404	4.1969	2.6928	2.2745	2.9109	
3.7289	3.1745	1.8623	1.9264	3.045	2.6816	2.3733	2.261	
3.4834	2.7029	2.2892	2.9565	1.7254	2.7033	2.4661	2.8674	
1.3826	2.9435	2.4736	1.8518	3.0954	2.7125	2.6247	2.3253	
3.3218	3.7006	2.3074	2.8785	3.1103	2.1117	2.8655	2.25	
2.5276	2.9356	2.509	2.7382	2.6301	3.2424	3.1083	3.7339	
2.7176	0.9361	1.0691	1.4336	2.8012	2.8969	4.0328	2.4492	
2.9618	3.5609	2.5408	3.1225	3.0328	2.8803	2.9237	2.6258	
2.3465	2.8033	2.6489	4.3109	2.687	3.7722	1.9432	1.7742	
2.3859	2.2631							
CCNK	4.7188	4.0648	3.7188	4.6001	3.8752	4.4495	4.4823	
4.8016	4.1902	4.5438	5.2755	4.5844	4.3376	4.6405	4.2428	
4.4542	4.8271	4.6789	4.8655	3.1191	4.3293	4.1998	4.4598	
4.1668	4.1211	4.6575	3.7333	4.2664	4.3829	4.1043	4.4036	
4.3838	4.0041	4.1633	3.6661	4.596	4.4124	4.4173	3.9892	
4.7983	4.082	4.2066	4.6464	4.4563	5.1244	4.8117	4.5581	
4.0998	4.1668	5.4115	4.1315	4.5339	4.3184	4.3636	4.4276	
4.3908	5.0467	4.7933	4.1603	5.0261	4.2784	4.319	4.2029	
3.8744	4.2844	4.9757	4.9587	4.8921	4.0649	4.3291	4.3532	
4.1348	4.3441	4.7035	4.7681	2.8616	4.346	3.8389	4.4717	
3.9236	4.6693	4.2759	4.6791	4.2839	3.9874	4.1075	4.8503	
4.5289	4.4902							
CCNL1	4.2264	4.5039	4.1648	3.8815	3.8362	4.3641	4.834	
4.5358	4.8839	4.7508	4.3672	5.4676	4.2819	4.1394	4.5965	
3.8436	4.6787	4.5575	4.8734	3.7548	4.4381	4.2129	3.6525	

4.2761	3.7475	4.2761	6.6283	4.2845	3.9573	4.1223	3.6824
4.2957	4.1139	4.2089	3.8607	4.1309	4.5679	4.0885	4.3842
4.6798	5.1601	3.7059	4.4423	4.746	4.7014	4.3087	4.4994
4.5446	4.6426	4.127	4.4105	4.5679	4.9684	4.626	3.3875
4.717	4.8763	3.971	3.5507	4.5078	4.2223	3.62	3.4929
4.8879	3.6785	4.6337	4.915	3.9101	4.7868	3.5393	3.9982
4.9992	4.0998	4.9455	4.162	4.1272	4.3129	3.583	4.2756
4.0998	4.7706	4.5267	4.6879	4.2046	4.1147	4.4473	3.7627
3.9393	3.9433						
CCNL2	5.5101	5.345	6.1835	4.9997	5.2569	5.0784	5.5241
5.2768	5.7672	5.2561	5.6406	5.1262	5.1964	5.5007	5.0293
5.2952	4.6018	4.949	4.3018	4.7076	6.9788	4.6062	5.4767
5.1984	6.0733	5.6815	5.3763	5.4829	5.4418	5.3985	6.2868
4.7624	5.4652	5.4047	5.819	4.6292	5.76	5.0825	5.3029
5.6287	5.4001	5.7425	4.3603	5.9816	5.7353	5.1543	4.2496
4.6239	6.4875	3.9568	5.1042	5.1992	5.9406	6.2262	4.8458
5.1727	5.4188	4.1338	5.1917	4.9102	5.153	3.7932	4.667
5.7452	5.1889	5.6897	6.0213	5.5728	5.7721	4.193	4.8256
6.0284	4.7098	6.2387	5.4582	5.6721	4.9273	4.717	5.4524
5.1506	5.6036	5.9927	5.1501	4.7434	4.7659	5.9916	4.2707
5.2577	5.1868						
CCNO	1.8519	0.8602	1.8704	2.485	2.7934	1.1283	1.4395
1.5229	1.2465	2.1705	2.1333	1.6406	2.0014	2.3034	1.6465
1.4305	1.6158	1.9977	2.7812	1.6026	2.5565	1.4662	1.2102
0.2152	1.6566	0.6508	1.8912	2.5752	1.5349	1.8166	1.7959
2.4507	2.668	2.0883	0.3189	2.0065	2.9888	2.2288	1.4264
1.4095	2.9952	2.1712	2.0666	1.8982	0.9664	1.6988	1.5114
1.349	3.0221	0.9505	1.1941	0.4094	1.9431	1.4068	2.495
2.9614	2.3449	1.5506	1.8404	1.5997	1.6951	1.2443	1.7219
2.8876	1.7426	1.9979	0.2457	2.4429	2.3968	2.9114	2.1457
1.9511	1.8929	1.4651	0.2633	2.1762	2.1423	0	2.2272
2.2154	1.9109	1.4046	2.4843	0.8081	2.6332	1.8621	1.5746
0.4331	0.5288						
CCNT1	3.6503	3.2228	3.4655	4.0413	4.04	3.6019	3.6057
3.1013	3.1339	3.1747	3.3291	3.5445	2.9252	3.4553	3.6032
3.3979	3.7003	4.1137	3.6212	3.5242	3.3747	3.622	3.3801
3.1313	3.654	3.5853	4.2422	3.1451	3.3185	3.5232	3.6691
3.3052	3.1433	2.958	3.0391	3.6104	4.1504	3.4679	3.5116
3.8837	3.6197	3.6994	2.4538	3.7381	3.7125	3.6585	4.0658
3.1372	3.4643	4.0622	3.2386	4.1325	4.1039	3.7639	3.4595
3.3551	3.8767	4.0479	3.3649	3.8286	3.8727	3.7186	3.9822
3.368	3.6259	3.0815	3.8093	3.1932	3.5807	3.3874	3.3446
3.1146	3.6729	3.5299	3.2368	2.6566	3.4147	2.7328	3.9337
3.4428	3.3065	3.6537	3.5232	3.6174	2.6657	3.6183	3.1707
3.3729	3.3074						
CCNT2	2.7402	2.3347	2.6641	2.8664	2.0197	2.879	3.5944
3.2253	2.9192	2.8542	2.5479	2.7767	2.721	3.1461	3.102
2.7063	2.5539	3.1196	3.2888	2.4488	2.836	2.5349	2.7181
2.7987	2.9777	2.3024	3.0378	2.8428	2.6636	2.9456	2.6201
1.9823	2.8841	2.5922	2.6442	2.7934	2.5246	2.952	2.2926

3.1299	2.6941	2.4941	2.7675	2.9833	2.7686	3.1009	3.0361
3.779	3.4016	2.7859	2.8691	2.8802	3.3738	3.0147	2.508
2.7009	2.5103	2.8423	2.7686	2.8762	2.4371	2.6372	2.463
2.5324	3.2842	2.3475	3.5309	2.3396	3.445	2.9719	2.4701
2.7537	3.1346	2.544	2.6108	2.7799	2.798	2.6	3.0451
2.4049	3.1894	2.3594	2.9738	2.7104	2.533	3.0878	2.5532
2.6227	2.5532						
CCNY	4.2708	2.9774	4.6167	4.2614	3.9038	3.1315	3.2443
3.1311	3.4488	4.315	3.4158	3.7091	3.7	3.679	3.4857
3.4788	4.7844	3.9647	3.6503	3.4102	3.7058	3.5337	3.9521
3.596	3.817	3.2175	3.6687	4.0306	3.3608	3.2697	5.214
4.3533	3.2735	3.9268	3.4175	4.1655	3.8247	3.2126	3.9877
3.2169	4.1146	3.9379	3.9921	3.9594	4.1867	3.2703	3.5312
3.4261	3.82	5.0453	4.4847	2.9858	3.6281	4.2326	4.7118
4.6791	4.5132	3.285	3.6706	3.2367	3.6425	3.4712	4.0309
4.012	3.8957	4.7541	3.6824	3.9134	4.2184	3.7913	3.9193
3.6287	2.8721	3.6292	3.9826	3.6766	3.9581	4.0309	3.22
3.6556	3.7409	4.5939	4.3334	3.1831	3.391	3.065	4.0536
3.9295	3.9257						
CCNYL1	3.2701	3.4054	3.3931	3.2435	2.9443	3.5286	3.8257
3.6303	3.5305	3.5163	3.2423	3.1094	2.4632	3.3068	3.5094
3.3258	3.2693	3.1525	3.3622	2.7681	2.7685	2.3274	3.678
3.6942	2.9848	2.5767	1.5677	3.4282	2.8711	3.0071	3.8174
2.9888	3.348	3.572	3.1568	4.3185	2.2736	2.6611	2.4737
3.2435	2.7583	3.4603	3.9026	2.9087	3.3659	3.7482	3.3468
3.458	3.643	2.7245	4.0526	3.4336	3.7563	2.8613	4.8118
2.3779	3.1701	3.9823	4.002	3.5668	2.8745	3.0249	2.2185
2.8873	2.5634	2.6285	3.1028	2.4204	3.7027	3.4645	2.9157
3.0178	2.9106	3.0858	3.0486	2.9344	3.7769	3.1024	2.8038
2.7651	3.5202	2.9423	3.5618	2.1612	3.0261	3.4933	2.572
2.0376	2.3999						
CCP110	2.6489	2.7925	3.4633	3.23	3.6977	3.1359	3.2834
3.2805	2.9459	2.314	2.4033	2.6722	3.7645	3.2241	2.9492
2.6126	2.7318	3.0499	3.2598	3.2094	3.2159	3.1951	2.8232
3.1617	3.3037	2.6812	3.5477	2.8067	3.3496	2.7936	3.3477
2.8301	2.9569	2.8354	2.5621	2.8984	2.5689	1.6839	2.4016
3.1235	2.391	2.6399	2.697	2.8059	3.8302	3.39	3.1639
2.9179	3.4015	3.082	2.405	3.578	2.9836	2.7832	2.8592
2.8894	2.9048	2.952	3.0013	3.4134	2.5938	2.6805	2.4807
3.3172	2.7627	2.2971	2.7851	1.7874	3.5323	3.6448	3.0009
3.6731	2.7938	2.8733	3.5423	2.6785	2.6856	3.0941	2.4502
2.6757	2.5967	2.5963	2.2665	2.7578	3.2135	3.2123	2.2516
2.8909	2.4845						
CCPG1	5.5958	4.9704	4.0522	5.3454	4.5595	4.7766	6.6261
3.4392	4.9173	6.1682	4.1496	4.9192	4.7414	3.6589	4.5202
6.0872	3.7889	5.8162	4.6114	4.786	3.8169	4.8282	5.9627
4.8419	5.7286	5.1782	4.83	4.2053	4.3647	4.0676	4.0027
4.7801	4.5918	5.7337	5.7808	5.0246	7.4049	5.6619	4.9122
4.3937	5.1587	5.3201	3.889	4.2375	5.118	5.9075	5.355
5.4507	5.4248	4.0865	6.1133	4.6835	4.4191	7.0049	4.1728

4.2129	4.426	3.888	5.4173	4.1012	4.3561	5.191	4.48	
5.0845	4.1119	7.0715	5.106	5.8333	4.9638	3.8042	3.7892	
4.3191	4.5294	5.2437	5.1132	5.6741	4.5204	5.8962	4.5369	
3.4904	4.2169	4.5726	6.3611	5.0376	4.6559	4.0772	5.0098	
4.2833	4.5494							
CCR1	0.2192	0.09	0.669	0	0.0972	0.2983	1.5134	
0.3345	0.4555	0.4878	0.1344	0.3323	0.3223	0.7646	0.1689	
1.637	0.1166	2.7324	0.6223	0.5108	0.9641	0.0632	0.5063	
0.5689	0.147	0.1458	0	0.1629	1.0721	0.2673	0.074	
0.3584	1.1826	0.0993	0.2434	0.3078	0.2923	0	0.1511	
0.6115	0.3152	0.5	0.2505	0.175	2.0159	0.112	0.8206	
2.2447	0.2526	5.0919	0.9875	0.4594	1.3434	0.2111	0	
0.459	0	0	0.5002	0	0.0634	0.9128	0.1596	
0.7004	0	0.198	0	1.1343	1.5778	1.1109	0.6894	
2.1137	0.6951	0.1062	0	0	1.5299	0.5456	1.7427	
0.0207	1.2112	0.0905	0.459	0	0.3213	1.0827	0.2751	
0.4442	0.1714							
CCR10	0.82	0.2514	0.4269	0.5768	0.3284	1.0442	0	
0.4099	0.1919	0.3017	0.2366	0.1406	0.2299	0.4761	0	0
	1.2057	1.1098	0	0	0	0.3278	0.3002	
0.3964	0.2772	0	0.4146	0.2754	0.1831	0	1.4783	
0.2072	0	0.8586	0.135	0.3683	0.2951	3.0772	0.1847	0
	0.6616	1.1219	0.7548	0.7364	0.1807	0	0	
1.7445	0.8745	0.8342	0.4031	0.7401	0	0.6055	0.7334	
1.0684	0.1368	0.6602	0	0.306	0.2028	0	0.0485	
0.0084	1.1613	0.7385	1.4114	0.9413	1.8656	0.2727	1.2094	
0.5386	0.4997	0.3025	0.6186	0	0.1101	0	0.5338	
0.7993	0.3971	0.1934	0.1634	0.7222	1.5127	0.6023	0.6216	
0.6749	0.4294							
CCR2	0	0	0.0401	0	0	0	0.0079	0
	0	0.0144	0	0	0	0.0625	0.0053	
0.2617	0	0.1612	0.0581	0	0	0	0	0
	0	0	0	0	0.0175	0	0	0
	0.0199	0	0	0	0.0026	0	0	0
	0	0	0	0.0207	0.0063	0.016	0.0315	0
	0	0.153	0.0161	0	0	0	0.0206	
0.045	0	0	0	0	0	0	0	0
	0	0.0032	0	0.03	0.0116	0	0.0105	0
	0	0.0276	0	0	0.0069	0	0	0
	0.0304	0	0	0	0	0	0.03	0
	0							
CCR3	0	0	0	0	0.087	0	0	0
	0	0	0	0	0	0	0	0
	0	0.0376	0	0	0	0	0	0
	0	0	0	0	0	0	0	0
	0	0	0	0	0	0	0	0
	0	0.1064	0	0	0.0397	0	0	0
	0	0.3824	0	0	0	0	0	0
	0	0	0	0	0	0.0315	0.058	0
	0	0.0134	0	0	0	0	0	0

	0	0	0	0	0.0305	0	0	0
	0	0	0	0	0	0	0	0
	0							
CCR4	0	0	0	0	0	0	0.0545	0
	0	0	0.0191	0	0.0501	0	0	0
	0.07	0.0527	0	0	0	0	0.0508	
0.15	0	0.0777	0	0	0	0	0	
0.198	0	0	0	0	0.0445	0.0867	0.0277	0
	0	0	0	0	0	0	0.2116	0
	0	0	0	0	0	0.0412	0.0365	0
	0	0.0361	0.0719	0	0	0.0549	0.0236	
0.0443	0.0391	0.0193	0.1144	0	0.0461	0	0.0829	
0.0242	0	0	0	0	0.1357	0	0	
0.0554	0	0	0.0501	0	0	0	0	0
	0							
CCR5	0	0.0148	0	0.0384	0	0	0	
0.0161	0	0	0	0	0	0	0	
0.0052	0	0.0782	0	0	0	0	0	0
	0	0.0077	0	0.0299	0	0	0	0
	0.0281	0	0	0	0.0089	0	0.0067	0
	0	0.0099	0	0.0256	0	0	0	0
	0.0102	0.3123	0.011	0	0	0	0	
0.0488	0.0225	0	0	0	0	0	0	0
	0.0228	0.0032	0.034	0	0.0061	0	0.0778	
0.0102	0.0152	0.0069	0	0	8.00E-04	0	0.0017	
0.0627	0	0	0	0	0	0	0.0199	0
	0							
CCR6	0.1273	0.1873	0.435	0.2536	0.0704	0.3348	0.0998	
0.1163	0.1891	0.085	0.086	0.0655	0.0847	0.239	0.1391	
0.2378	0.1917	0.1122	0.3631	0.1682	0.2238	0.1073	0.1933	
0.4561	0.0573	0.0692	0.2302	0.1475	0.1435	0.0732	0.1619	
0.0566	0.0786	0.1317	0.116	0.0642	0.018	0.0866	0.0389	
0.2104	0.0183	0.0472	0.1407	0.1711	0.1947	0.1306	0.137	
0.1962	0.1154	0.1623	0.1171	0.164	0.1458	0.3219	0.3656	
0.264	0.2096	0.146	0.167	0.1568	0.1238	0.1282	0.1821	
0.1954	0.0446	0.0235	0.0627	0.0471	0.3193	0.191	0.0634	
0.096	0.0705	0.0576	0.2027	0.1045	0.2112	0.0462	0.0492	
0.1527	0.1537	0.0529	0.0619	0.1403	0.112	0.1516	0.056	
0.1386	0.1142							
CCR7	0.095	0.4606	0.1256	0	0.0052	0	0.1019	0
	0.0514	0.5701	0.7297	0	0	0	0.0471	
0.4409	0.0393	0.8319	0.2702	0	0	0.0115	0.242	0
	0	0.2456	0.0194	0	0	1.092	0.0011	
0.1022	0.0466	0	0.0012	1.3569	0.0516	0.0212	0.2251	
0.0132	0.0051	0.0168	0	0	0	1.4091	0.0318	
0.0183	0.0045	0.0708	0.0598	0.2219	0.2672	0.1124	0.0499	
0.3483	0	0.0471	1.1044	0.0664	0	1.8725	0.0386	
0.0533	0	0.0255	0	0	0.44	0	0	
0.0628	0.0686	0.0481	0.0553	0.0867	0.1429	0	0.0791	0
	0.1064	0	0.0367	0.0439	0	0	0.0231	

0.0262	0							
CCR8	0	0	0	0	0	0	0	0
	0	0	0	0	0	0	0	0
	0	0	0	0	0	0	0	0
	0	0	0	0	0	0	0	0
	0	0	0	0	0	0	0	0
	0	0	0	0	0	0	0	0
	0	0	0.0265	0	0	0	0	0
	0	0	0	0	0	0	0	0
	0	0	0	0	0	0	0	0
	0	0	0	0	0	0	0	0
	0	0	0	0	0	0	0	0
	0							
CCR9	0	0	0	0	0.0413	0	0.0574	0
	0.0584	0.0509	0.0296	0.0264	0	0	0	
0.0245	0	0	0	0	0	0	0	
0.0105	0	0	0.02	0	0	0	0	0
	0	0	0	0.0117	0	0	0	0
	0.0146	0	0	0	0.0087	0	0	0
	0	0.056	0.0159	0	0	0	0	
0.0601	0	0	0	0	0	0.0125	0	
0.0346	0	0	0	0	0	0	0	0
	0	0	0	0	0	0.0216	0.0076	0
	0.016	0.0101	0.0144	0	0	0	0	
0.0565	0							
CCRL2	0.057	0.0362	0	0.1918	0.0424	0	0.0169	
0.0905	0	0.0594	0	0.3807	0.0812	0	0	0
[truncated: 489,625 more chars]
